# *De-novo* transcriptome analysis unveils differentially expressed genes regulating drought and salt stress response in *Panicum sumatrense*

**DOI:** 10.1038/s41598-020-78118-3

**Published:** 2020-12-04

**Authors:** Rasmita Rani Das, Seema Pradhan, Ajay Parida

**Affiliations:** Institute of Life Sciences, NALCO Square, Chandrasekharpur, Bhubaneswar, 751023 India

**Keywords:** Molecular biology, Plant sciences, Climate sciences

## Abstract

Screening the transcriptome of drought tolerant variety of little millet (*Panicum sumatrense*), a marginally cultivated, nutritionally rich, susbsistent crop, can identify genes responsible for its hardiness and enable identification of new sources of genetic variation which can be used for crop improvement. RNA-Seq generated ~ 230 million reads from control and treated tissues, which were assembled into 86,614 unigenes. In silico differential gene expression analysis created an overview of patterns of gene expression during exposure to drought and salt stress. Separate gene expression profiles for leaf and root tissue revealed the differences in regulatory mechanisms operating in these tissues during exposure to abiotic stress. Several transcription factors were identified and studied for differential expression. 61 differentially expressed genes were found to be common to both tissues under drought and salinity stress and were further validated using qRT-PCR. Transcriptome of *P. sumatrense* was also used to mine for genic SSR markers relevant to abiotic stress tolerance. This study is first report on a detailed analysis of molecular mechanisms of drought and salinity stress tolerance in a little millet variety. Resources generated in this study can be used as potential candidates for further characterization and to improve abiotic stress tolerance in food crops.

## Introduction

Sustaining and improving crop productivity in response to emerging challenges of global climate change is a global concern. Various abiotic stress factors like drought, salt and high temperature affect the plant growth and yield and account for major crop losses in productivity around the world, reducing average yields of major crop plants by > 50%^1^. Hence, there is a pressing need to understand the plant responses to these stresses so as to mitigate the detrimental effects that lead to heavy yield loss. Improving stress tolerance in plants is critical for agricultural productivity and also for environmental sustainability because crops with poor stress resistance consume larger amounts of water and fertilizers and thus greatly burden the environment^2^. In this context, detailed study on locally adapted and underutilized species, being highly nutritious as compared to the staple food crops, holds significant promise and have potential for being used as future food security crops.

Millets are one of those underutilized group of cereal grains which, in spite of having high nutritional and nutraceuticals components, are still cultivated and consumed marginally^3^. Small millets are nutritionally rich, hardy and subsistence crops and are gaining importance because of their potential role in nutritional food security and health benefits^3^. These crops characteristically adapt to adverse ecological conditions with adaptation to abiotic and biotic stresses, and require minimal inputs^4^. In spite of their potential for future food security and abetting effects of abiotic stresses, with exception to finger millet and foxtail millet , reports on genomic resources for species like little millet are scanty and meagre. Therefore, the present investigation was undertaken to generate genomic resources in one of the important small millet species, *Panicum sumatrense*.

*Panicum sumatrense* (Little millet) is generally regarded as an orphan crop because of its restricted cultivation to some specific regions and limited consumption. It is a small seeded cereal crop mostly grown in the semi-arid tropical and sub-tropical regions of Asia and Africa. It is native to India and popularly called as “Indian Millet” which has a short crop cycle and is grown as a staple food next to rice in states like Rajasthan, parts of Odisha etc. which are prone to drought like conditions. Little millet was domesticated 5,000 years ago in India^5^ and is grown mainly in India, Myanmar, Nepal, and Sri Lanka^6^. It is a tetraploid (2n = 4x = 36)^4^ crop species and is characteristically adapted to unfavorable ecological conditions like various abiotic stresses. It can grow under low availability of water during dry seasons. Moreover It grows well under adverse environments such as soil with high salinity and high temperatures^7^. Although it is highly nutritious and rich in proteins, fibers, minerals and good lipids. The *Panicum sumatrense* ( var. OLM 20), studied here is a reported variety of little millet found in Odisha that is characteristically resistant to drought and one of the faster growing varieties of little millet (www.millets.res.in/technologies/little_millet.pdf).

Little millet is perhaps the least studied of the small millets species and there is so much that requires investigation, including the establishment of a genetic map and sequenced genome. It is necessary to dissect the transcriptome of this important plant under abiotic stress conditions for the identification and characterization of the key genes responsible for abiotic stress tolerance. The current study was aimed at exploring the gene expression profile of a drought tolerant variety of *Panicum sumatrense* (var. OLM 20) under conditions of abiotic stresses like salinity and drought to present its responsive mechanism.

## Results

### Sequencing, assembly and quality assessment

The leaves and roots samples of *P sumatrense* were sequenced in replicates and the raw reads have been submitted to SRA database at NCBI under the accession PRJNA554415. A total of 265.26 million clean reads from 12 samples (Table 1) were used to generate the assembled transcriptome of *P. sumatrense* using three assemblers i.e. Trinity, BinPacker and rnaSPAdes^8–10^. Using multiple assemblers ensured the best and longest transcripts. The assemblies were catenated and redundancy was removed using CD-HIT-EST and CAP3. The final assembly consisted of 86,614 unigenes with a N50 value of 1756 bp with the largest unigene was more than 16 Kb in length. Majority of the unigenes were 501–1000 bp in length (Supplementary figure 1a).

**Table 1:** Ilumina sequencing data pre-processing statistics.

Tissue	Treatment	Sample ID	Raw reads	Q20(%)	Q30(%)	Clean reads
Leaf	Control1	LMCNL1	19,544,326	97.85	94.27	18,699,166.0
	Control2	LMCNL2	19,645,375	97.88	92.27	18,892,743.0
	Salt stress1	LMSTL1	24,287,613	97.73	93.92	23,345,002.0
	Salt stress2	LMSTL2	22,396,357	97.88	94.25	21,505,248.0
	Drought stress1	LMDTL1	24,784,686	97.74	94.00	23,881,546.0
	Drought stress2	LMDTL2	24,357,781	97.85	94.19	23,401,437.0
Root	Control1	LMSTR2	16,676,009	98.74	96.32	16,410,630.0
	Control2	LMSTR2	23,584,517	98.72	96.31	23,213,392.0
	Salt stress1	LMDTR1	27,853,073	98.76	96.42	27,427,411.0
	Salt stress2	LMDTR2	27,513,002	98.73	96.35	27,106,949.0
	Drought stress1	LMCNR1	18,618,444	98.76	96.39	18,332,556.0
	Drought stress2	LMCNR2	23,472,683	98.75	96.37	23,098,126.0

The quality of the final assembly was assessed using three different parameters. All the clean reads were mapped onto the assembled transcriptome of *P. sumatrense* using bowtie2. On an average, 92.33% of the reads could be mapped back completely. After mapping the reads, TransDecoder tool was used to identify the coding regions within transcript sequences and the analysis revealed that 79% of the Little millet transcriptome codes for complete ORFs (Supplementary figure 1b). Finally BUSCO (Benchmarking Unique Single Copy Orthologs), was used to explore completeness of transcriptome according to conserved ortholog content. The software was used to compare little millet transcripts with the database for Liliopsida (for Plants). From the analysis 92.8% of complete BUSCOs were found with 62.7% of single copy BUSCOs (Supplementary figure 1c).

### Functional annotation

**Figure 1:**
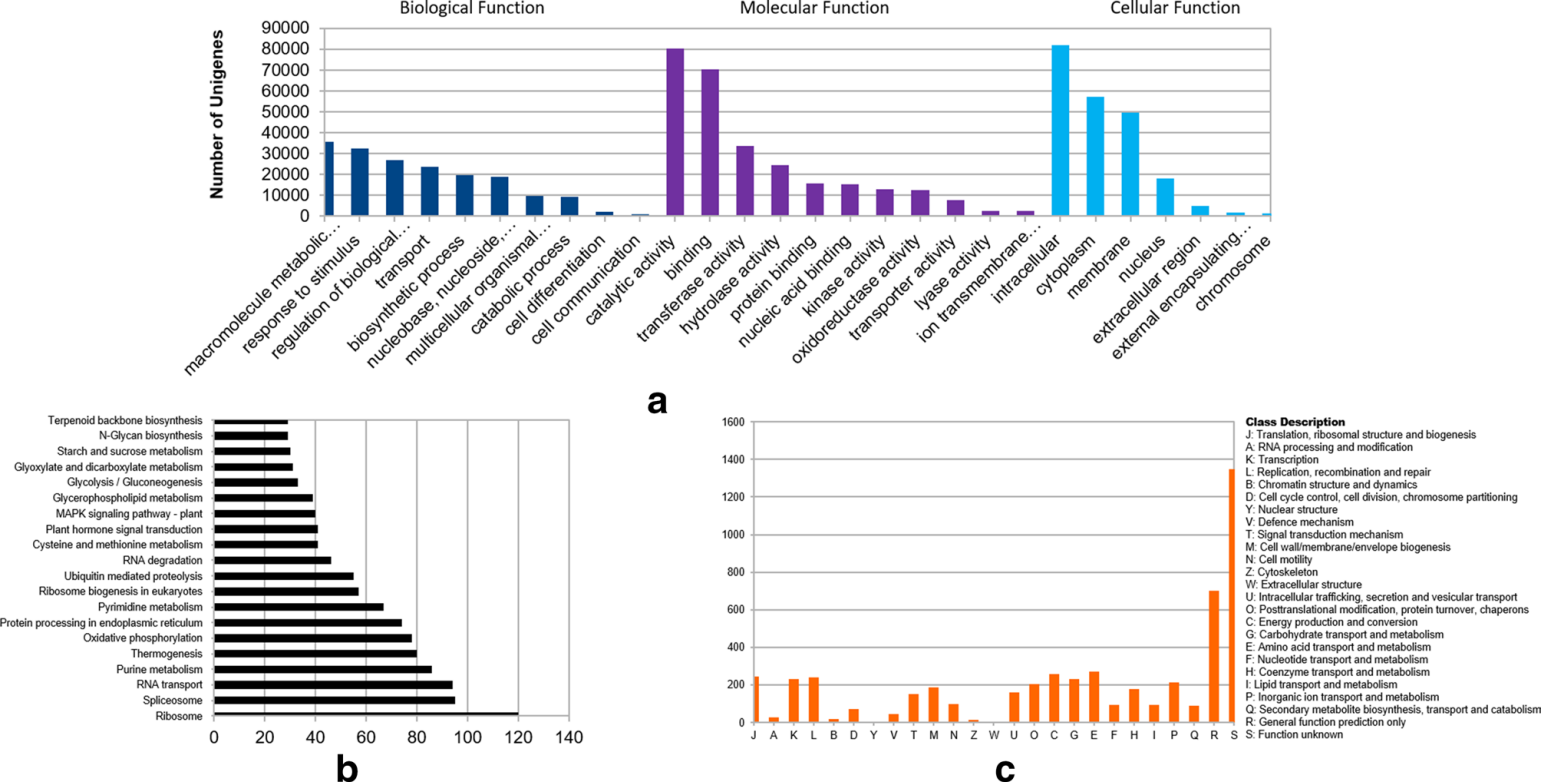
Functional annotation of unigenes. (**a**) GO terms classified into three major classes namely biological process, molecular function and cellular components. GO terms were assigned to the Unigenes after conducting Blastx search against the Uniprot Swissprot database (with a evalue cutoff of 10^−5^) using the standalone version of BLAST. GO annotations, GO terms and GO Slim terms were downloaded from various databases; and assigned to unigenes using linux shell commands. (**b**) Distribution of transcripts into biological pathways in KEGG database: The KAAS (KEGG automatic annotation server); web-server was used to assign biological pathways to the transcripts and (**c**) annotation based on comparison with COG database was done using the web-server on WebMGA.

After the quality assessments, the unigenes were annotated to various functions. Annotation based on GO terms (Gene Ontology) revealed that under biological function, majority of unigenes were categorised under “metabolic processes”, “response to stimulus” and “regulation of biological processes”. In case of molecular function, most of the unigenes were found to have “catalytic” and “binding” activity (Fig. 1a). The unigenes were also assigned to various biological pathways using the KAAS analysis tool (https://www.genome.jp/kegg/kaas/). Most of the unigenes were found to be involved in “Ribosome” and “Spliceosome” pathways. Along with these, some important pathways like “MAPK signalling pathway”, “Ubiquitin mediated proteolysis pathways”, “Terpenoid backbone biosynthesis” etc. were also detected (Fig. 1b). Finally, the transcripts were grouped into various orthologous groups on the basis of COG database (http://weizhong-lab.ucsd.edu/webMGA/server/cog/). Vast majority of the unigenes were categorised into “Function unknown” followed by “General function prediction only” (Fig. 1c).

### Differential gene expression

**Figure 2:**
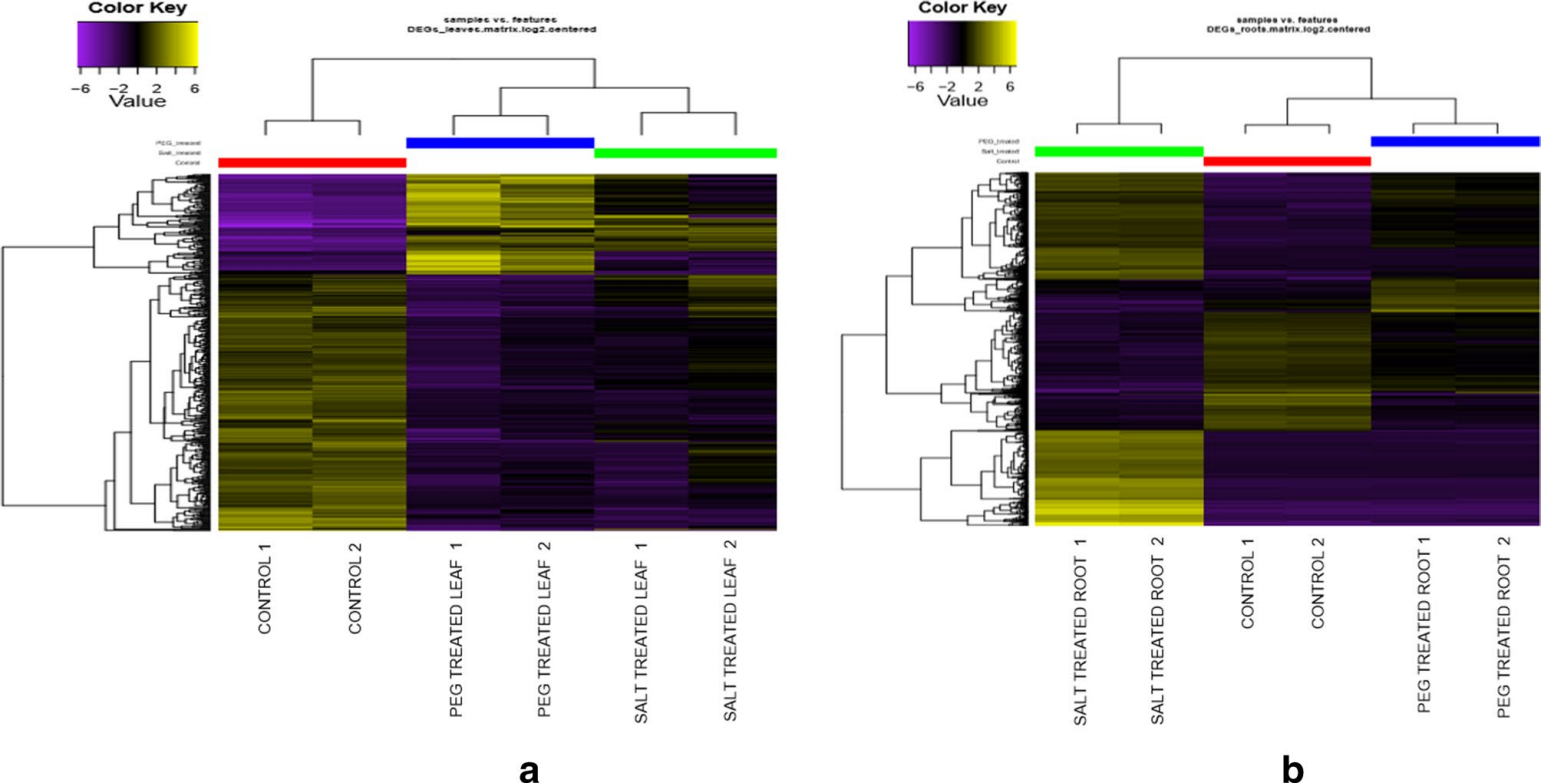
In silico expression analysis of Unigenes. (**a**) DEGs in leaves, (**b**) in roots under control and stress conditions. The short reads from individual sample libraries (including replicates) were mapped onto the assembled transcriptome using Bowtie2 and abundance was calculated using RSEM. DEGs were identified using the Empirical Analysis of Digital Gene Expression (edgeR) statistical package. The heat maps were generated using MeV v 4.8.1, and indicate the relative transcript level of genes. Detailed information about the DEGs is provided in Supplementary table 1.

The largest difference in gene expression pattern was observed in the case of salt treated roots compared to control where a total of 1187 unigenes were found to have significant differential expression with 738 unigenes being upregulated and 449 unigenes being downregulated. On the other hand, drought stress induced by PEG led to upregulation of 241 DEGs and downregulation of 134 DEGs in roots showing that salinity stress had a more pronounced impact on the plant roots as compared to drought (Supplementary table 1). Among all the DEGs, the 60 s and 40 s ribosomal proteins, NADH dehydrogenase, sugar transporters, some histone encoding genes and a number of genes coding for antioxidants and related proteins were found to be upregulated in response to drought and salinity stress in roots. Genes coding for plant cysteine oxidase, Zn finger containing CCCH proteins, ERF TFs and early nodulin were found to be downregulated in root tissues. In addition to these, genes coding for Ricin B-like lectin and various heat shock proteins were downregulated implying that these regulations may play crucial role in combating stress (Fig. 2a, Supplementary table 1). Drought stress had a slightly more pronounced effect on leaves as compared to roots. A total of 478 unigenes were differentially expressed in leaves as compared to 375 unigenes in roots under drought stress (Supplementary table 1). Unigenes coding for WRKY and Zinc finger containing transcription factors, cytochrome P450, some UDP-glycosyltransferase, LEA, and Aquaporins were found to be upregulated while those encoding LRR receptor like serine/threonine protein kinase, various lectins, F-box proteins, Ankyrin repeat containing proteins, some peroxidases and wall associated receptor kinases were downregulated (Fig. 2b, Supplementary table 1). On the other hand, only 286 unigenes were seen to be differentially expressed in leaf tissue subjected to high salinity. Most of these were downregulated with respect to control (Fig. 2a). It was interesting to note that a large number of kinases such as LRR receptor like serine/threonine protein kinase, Cysteine-rich receptor-like protein kinase, Wall-associated receptor kinase, Serine/threonine protein kinase were downregulated in leaves during salinity stress (Supplementary table 1).

### Validation by qRT-PCR analysis

**Figure 3:**
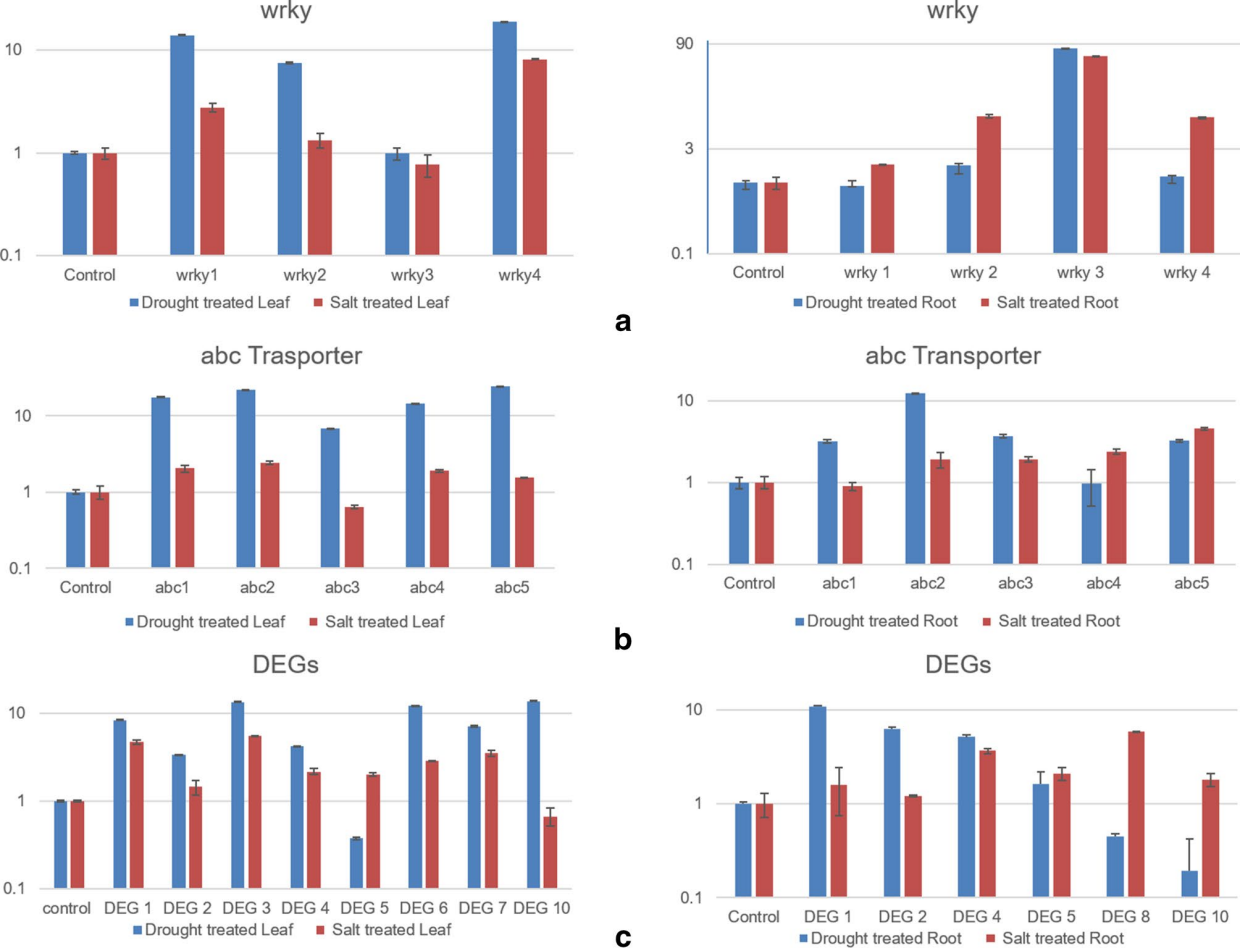
Validation of RNA seq data by qRT-PCR in Leaves and Roots. Comparative expression levels of genes encoding (**a**) WRKY transcription factors (**b**) ABC transporters and (**c**) various DEGs chosen randomly. Primers were designed based on the sequence of the corresponding Unigene and used to perform qRT PCR using SYBR green chemistry. The resulting graphs were drawn in MS Excel after taking the average of three technical and two biological replicates for each sample.

The digital expression of the transcriptome was validated by qRT-PCR taking 15 stress responsive DEGs chosen randomly based on in silico analysis. The comparative study between the digitally obtained data and qRT-PCR expression analysis under both control and stress conditions were found to be co-related for most of the genes (Fig. 3, Supplementary figure 2). The analysis also showed that WRKY transcription factors designated as WRKY1 and WRKY2 had very high expressions in leaves with fold change more than 13 times and 7 times respectively in response to drought stress while WRKY3 was seen to be upregulated almost exclusively with more than 50 times fold change in root tissue in response to both salinity and drought. WRKY4 was found to be upregulated in both leaf and root tissues with more than 8 times fold change under both stresses except for PEG treated root where no significant difference was seen (Fig. 3a). Similarly, members of another important gene family, ABC transporters were also analysed for their expression and it was observed that the transporter denoted as ABC1, ABC2 and ABC3 had higher expression in response to drought stress with more than 6 times fold change in leaf tissues and more than 3 times fold change in root tissues as compared to salinity while ABC4 and ABC5 had more pronounced upregulation with more than 14 times fold change in leaves in response to drought stress (Fig. 3b). Amongst the other DEGs, there were genes coding for Terpene synthase (DEG10) and cysteine desulfurase (DEG4) (Fig. 3c).

### Unigenes that coincide in leaf and root under drought and salinity stress

**Figure 4:**
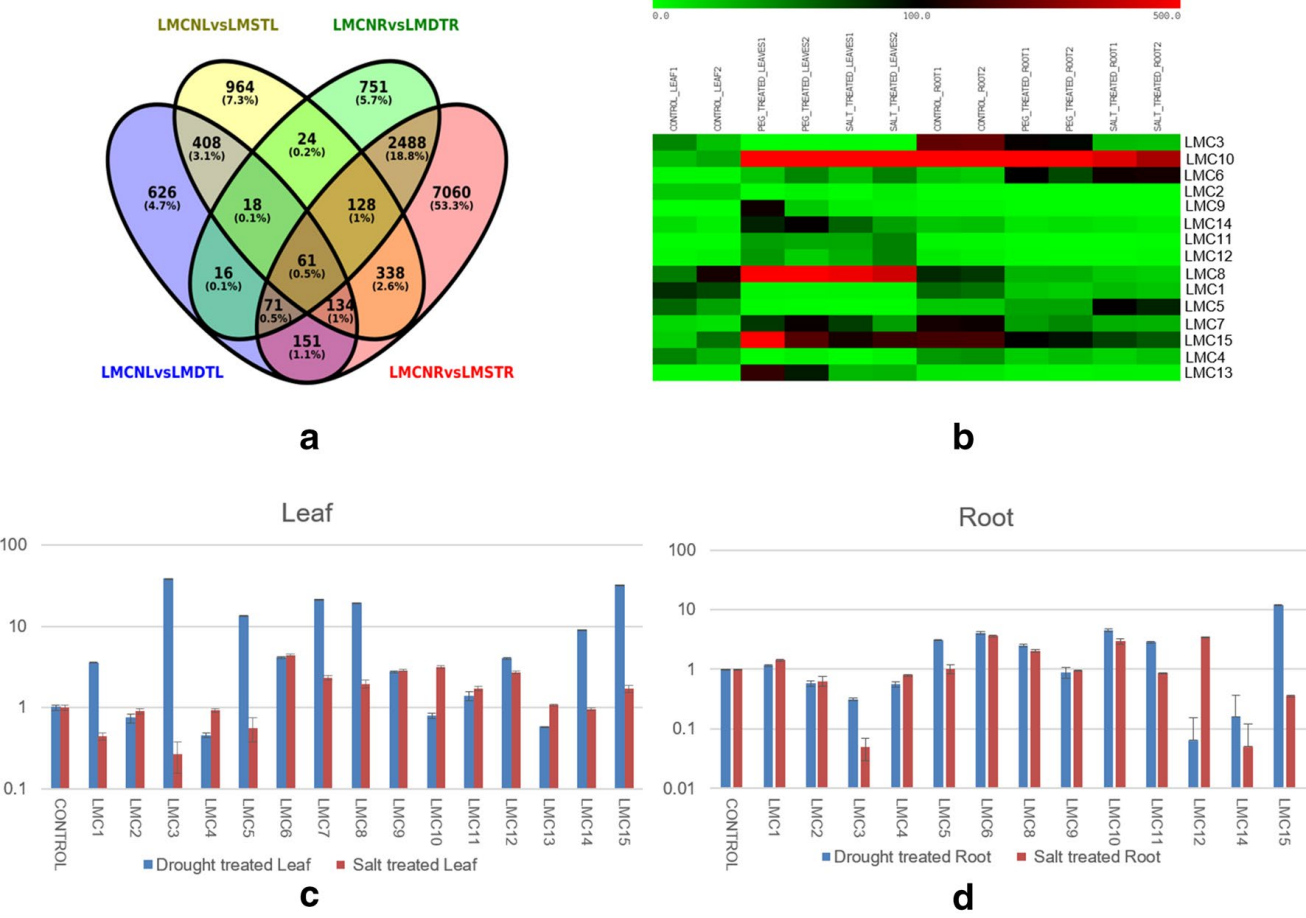
Expression analysis of the Unigenes that coincide in the leaf and root tissues during drought and salinity stress. (**a**) Venn diagram depicting the overlapping and unique Unigenes in leaf and root tissue of Little millet (LMCNL = control leaf; LMDTL = drought treated leaf; LMSTL = salt treated leaf; LMCNR = control root; LMDTR = drought treated root; LMSTR = salt treated root). (**b**) Heat map (prepared using MeV v. 4.8.1) depicting in silico differential expression of unigenes common to salinity and drought stress in leaf and root tissue (labelled as LMC). The scale signifies the range of expression levels of the Unigenes in the samples (in duplicate) and denotes the values in the matrix generated by edgeR. qRT PCR of selected unigenes in (**c**) Leaf and (**d**) roots tissue was performed using unigene specific primers and represent the relative expression of unigenes as compared to the control.

We further extended our search to identify those unigenes that were differentially expressed in both the tissues irrespective of stresses. A total of 61 unigenes were found to be common to both tissues and stresses (Fig. 4a; Supplementary table 2). These included some well characterised genes involved in abiotic stress tolerance such as peroxidases and glutamine synthetase (Supplementary table 2). Interestingly, a member of the family Ricin-B like lectin (TRINITY_Contig10696) was also found to be expressed almost exclusively in leaf tissue in response to salt and drought stress with the expression being highest in case of drought stress (Fig. 4b,c). Similarly, a unigene coding for copper transport protein (TRINITY_DN12117_c0_g1_i4) was also observed to have high expression in leaf tissue subjected to drought stress. Gene coding for Alcohol dehydrogenase (TRINITY_Contig6665) was found to be upregulated in both tissues under salt and drought stress while that for Cellulose synthase was downregulated (TRINITY_6483_length_2616_cov_33.980762_g4098_i0) (Fig. 4b–d).

### Transcription factors in abiotic stress response

**Figure 5:**
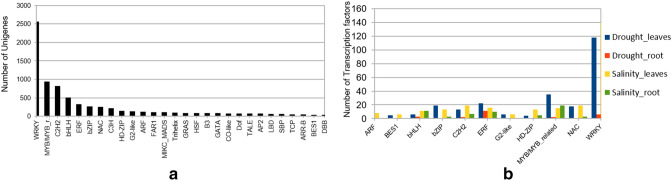
Transcription Factors in *P. sumatrense* transcriptome (**a**) TFs identified in *P. sumatrense* after comparison with TFs from *P. hallii* (**b**) Comparative levels of expression of differentially expressed TFs in leaves and roots under conditions of drought and salinity stress. The graphs were constructed on MS Excel.

**Figure 6:**
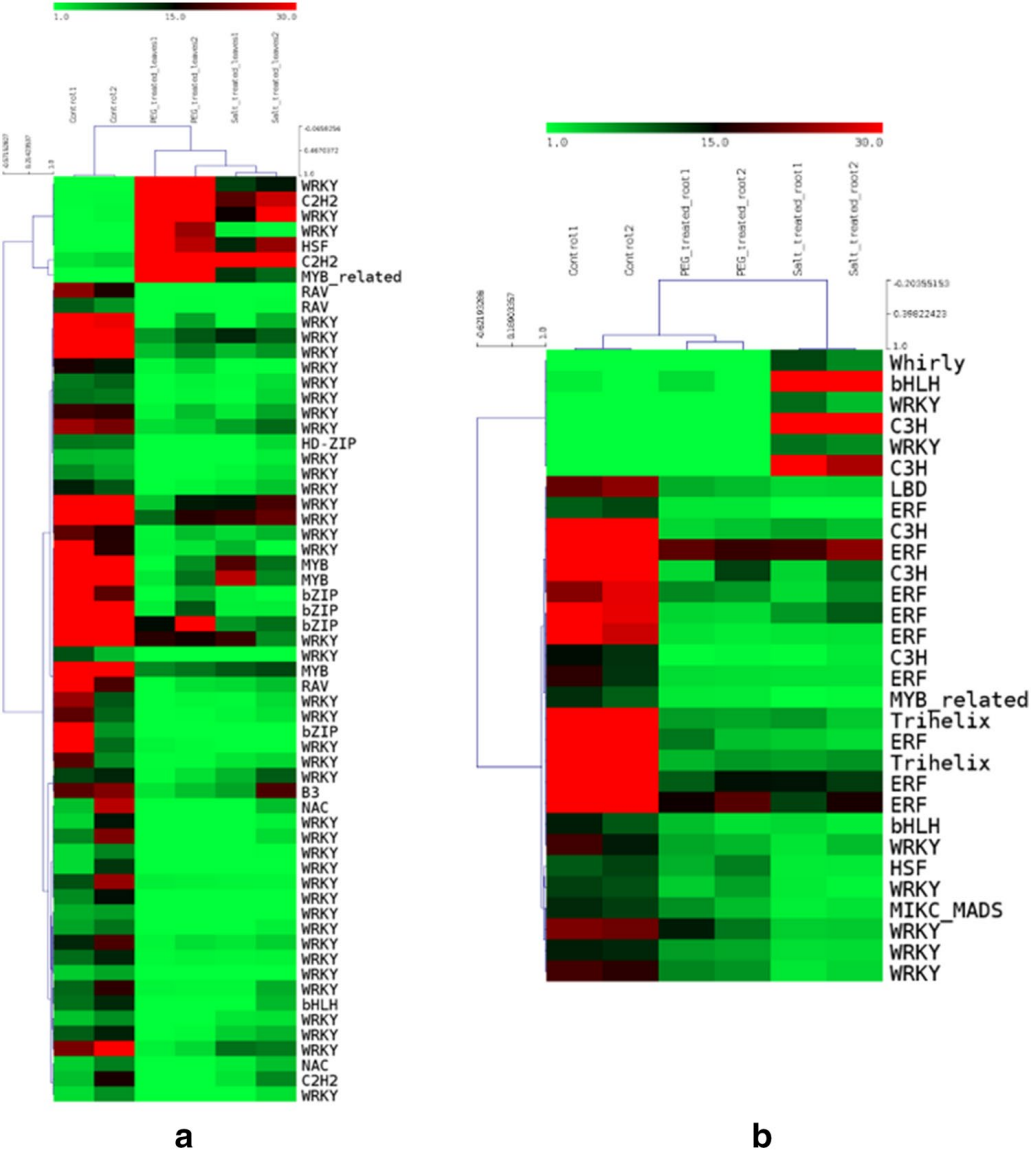
Digital expression analysis of unigenes encoding Transcription factors. Heat map illustrates the differential expression of TFs in (**a**) leaves (**b**) in roots. Heat maps were generated on MeV v 4.8.1. The scale represents range of expression values in the expression matrix generated by edgeR.

A total of 7698 transcription factors were identified in the transcriptome of *P. sumatrense* after comparison with the TFs identified in *Panicum hallii*. Out of these, a vast majority were represented by WRKY family of transcription factors (2565) followed by MYB/MYB-related (939), C2H2 (823) and bHLH (510) (Fig. 5a). A comparison between the significantly differentially expressed TFs in different samples showed that a higher number of TFs were detected in leaves in response to salinity and drought as compared to roots, MYB/MYB-related TFs being an exception with slightly higher numbers in root as compared to leaves in response to salinity stress (Fig. 5b). In silico gene expression analysis showed that TFs belonging to the HSF, MYB-related and C2H2 family are upregulated during drought and salinity stress. Majority of the WRKY TFs were found to be downregulated in leaves while only few of them were upregulated (Fig. 6a). Similar results were obtained in root tissue where TFs like Whirly, bHLH, C3H and WRKY were upregulated and a number of members of C3H, WRKY, Trihelix and ERF were downregulated in response to drought and salinity stress (Fig. 6b).

### SSRs in *P. sumatrense* transcriptome

**Figure 7:**
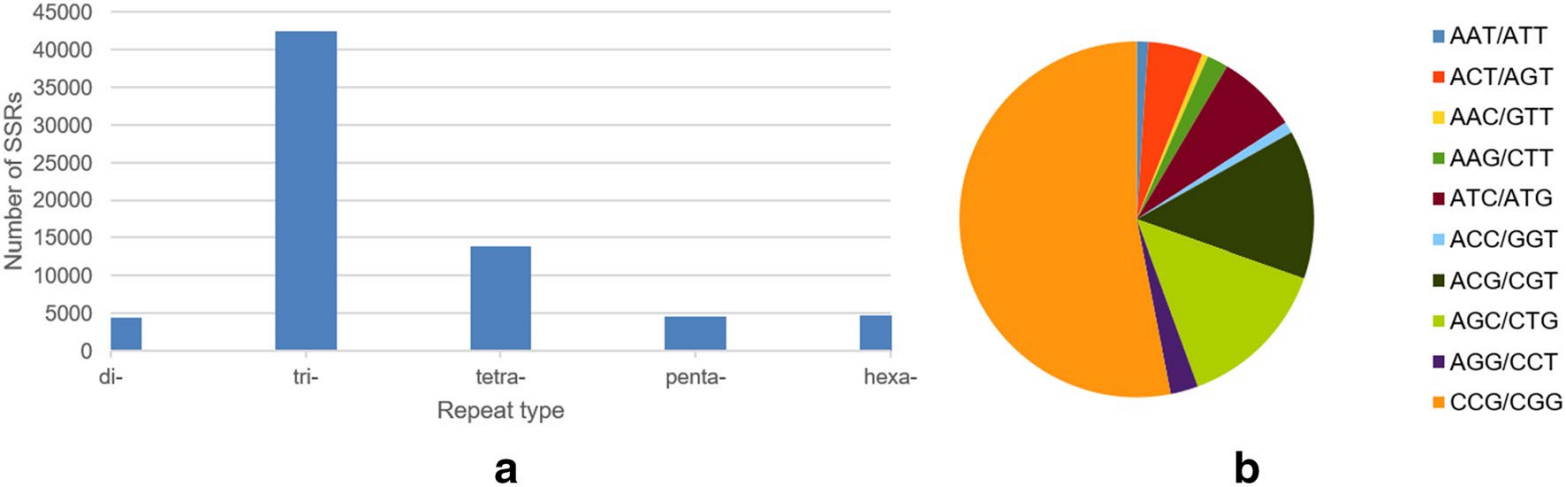
SSR sequences identified in *P. sumatrense* transcriptome. (**a**) Distribution of SSRs in the Little millet transcriptome, (**b**) pie-chart representing the abundance of tri-nucleotide repeats throughout the transcriptome.

A total of 69,900 SSR sequences, ranging from di- to hexa nucleotide repeats, were identified in the transcriptome of *P. sumatrense*. Out of the 86,614 unigenes, 37,100 were found to be SSR containing sequences (Table 2). Tri-nucleotide repeats (60.7%) were found to be the most abundant type of SSRs followed by tetra-nucleotide repeats (19%) (Fig. 7a, Table 2). Out of the tri-nucleotide repeats, CCG/CGG repeats were found to be the most frequently occurring SSRs (Fig. 7b).

**Table 2:** SSR sequences identified in *P. sumatrense* transcriptome.

Total number of sequences examined	86,614
Total size of examined sequences (bp)	114,926,266
Total number of identified SSRs	69,900
Number of SSR containing sequences	37,100
Number of sequences containing more than 1 SSR	17,217
Number of SSRs present in compound formation	2826

Distribution to different repeat type classes	
Unit size	Number of SSRs
2	4410
3	42,477
4	13,786
5	4598
6	4629

#### Digital expression data for the SSR containing unigenes

**Figure 8:**
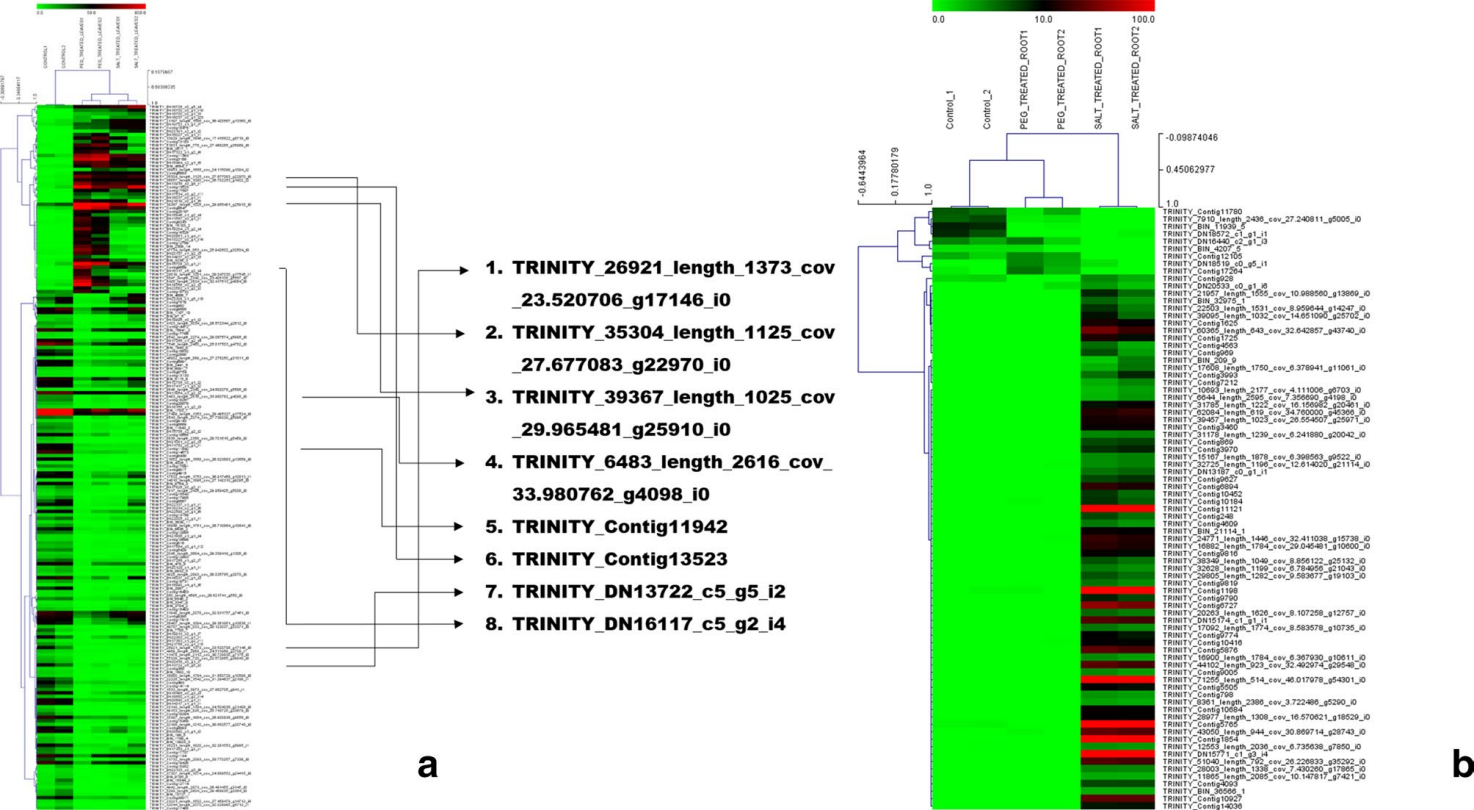
Differential expression analysis of SSR containing unigenes. Heat map represents the differential expression of SSR containing unigenes in (**a**) leaves (**b**) in roots. Heat maps were generated on MeV v 4.9.0. The scale represents range of expression values in the expression matrix generated by edgeR.

Revealed that higher number of SSR containing unigenes were differentially expressed in leaves as compared to roots with significant differential expressions to drought and salinity stress. In leaves, 202 such unigenes were found to be differentially expressed (Fig. 8a) whereas roots had a total of 81 differentially expressed SSR containing unigenes (Fig. 8b). Eight of the unigenes in leaves were common to both drought and salinity stress, coding for important proteins like chitinase (TRINITY_26921_length_1373_cov_23.520706_g17146_i0), cellulose synthase (TRINITY_26921_length_2616_cov_33.980762_g4098_i0) and heat stress transcription factor (TRINITY_DN16117_c5_g2_i4). The pattern of expression of unigenes was found to be interesting where most of the unigenes have shown upregulation in response to salt stress induced in roots with no significant change in drought stress as comparison to control but at the same time higher number of unigenes were downregulated in response to both stresses in leaves. The differential expression matrix of SSR containing unigenes are provided in Supplementary table 4. Since SSR containing unigenes showed differential expression patterns under abiotic stress, it was evident that these could be used as important molecular markers for crop improvement. Therefore, we designed forward and reverse primer sequences for the SSRs identified in this study. The software used for designing the primers was able to parse the data from the SSR identification software (MISA) to produce a comprehensive list of the types of SSRs, their position in the transcriptome and their respective primer sequences (Supplementary table 5).

## Discussion

The impact of salinity and drought stress is widespread in the world and requires identification of novel regulators that will enable genetic enhancement of major crop plants. Little millet is a nutritionally rich and hardy crop which can endure adverse climatic conditions. However, genomic resources for this important plant is lacking. This is the first ever report on generating transcriptome profiles in little millet root and leaf tissues after being subjected to drought and salinity stress. This study was undertaken with an objective to identify genes associated with the abiotic stress tolerance to facilitate the molecular engineering of plants with increased tolerance to severe environmental stresses.

The GO term analysis of DEGs suggested the involvement of various transcripts in a number of biological processes like ‘response to stimulus’, ‘transport’ and ‘metabolic pathways’. Further the transcripts were assigned to various biological pathways which revealed over-representation of categories like ‘plant hormone signaling pathway’ and ‘MAPK signaling pathway’. A number of DEGs identified in this study were found to be involved in signal transduction pathways thus fortifying the idea that response to stimuli and signal transduction are necessary processes in abiotic stress response^11^. Receptor kinases are an integral part of the signaling machinery and various receptor like kinases (RLKs) have been implicated in the abiotic stress tolerance^12^. However, differential gene expression analysis in our study showed that a number of kinases including LRR receptor like serine/threonine protein kinase, cysteine-rich receptor-like protein kinase, serine/threonine protein kinase etc. were downregulated in leaves during salinity stress in little millet. Similar results have been reported in rice^13^ and Arabidopsis^14^ where an *LRR*-*RLK* gene, *Leaf Panicle 2* (*LP2*), was downregulated by drought and ABA and was seen to regulate stomatal closure and density.

Abiotic stresses also leads to the active production of reactive oxygen species (ROS) which then produce detrimental effects on mitochondrial DNA, which is especially susceptible to oxidative damage^15^, as well as affects the cellular integrity by attacking the polyunsaturated fatty acids (PUFAs), which are the major fatty acids in the plant membrane^16^. ROS signaling has been reported to be a key factor in regulation of plants’ response to abiotic stress and is often coupled with calcium based signaling^17^. Ca^+2^ dependant signaling leads to activation of LEA proteins, a phenomenon which was also observed in this study where drought and salinity stress led to upregulation of LEA proteins in leaf tissue of little millet. This validates the role of ROS signaling in abiotic stress response of plants. Redox signaling is also known to regulate the expression of transcription factors^18^. These findings also hold true for little millet where TFs like WRKY, Zn finger proteins, MYB and ERF were found to be differentially regulated in response to drought and salinity stress.

ROS scavenging by a variety of anti-oxidant molecules is one of the most widely studied events for alleviating the effects of abiotic stress in plants. Some of the important DEGs were found to be late embryogenesis abundant (LEA) protein, cytochrome P450 and aquaporins. Many authors have reported the role of late embryogenesis abundant proteins in plants like Barley^19^ and Arabidopsis^20^ in response to abiotic stresses. The role of cytochrome P450 and aquaporins are established under both drought and salt stress in many plant species like rice, spinach cytochrome p450 in tobacco, banana aquaporins in *Arabidopsis* etc.^21,22^. The qRT-PCR analysis also shown the involvement of Terpene synthase gene in response to abiotic stress, which is responsible for producing terpenes. The main function of terpenes and their derivatives are widely recognized in plant defense mechanism^23^ and are produced by the plant in response to oxidative stress induced by various abiotic factors^24^. In addition to these, a number of sugar transporters were upregulated in roots in response to drought and salt stress, reiterating similar findings in a number of previous studies^25,26^. Genes coding for ABC transporters were also found to be showing differential expression in response to both the stresses that plays a vital role under stress induced due to various abiotic factors and few reports are there in support of its involvement in stress response^27,28^.

From the transcriptome analysis, a number of genes were identified which have very few or no previous reports of being directly involved in plants’ response to abiotic stress. For example, there are studies showing the involvement of Ricin B like lectin in biotic stress response against phytophagous insects^29^ but there are no reports of it having a role in abiotic stress response mechanism in plants. However, this gene was found to be differentially expressed in both leaf and root tissues of *P. sumatrense* during drought and salinity stress. It would be interesting to further characterize this unigene that might bring in a new insight to biotic and abiotic stress crosstalk. Similarly, genes encoding alcohol dehydrogenase and cellulose synthase have shown differential expression in response to abiotic stress. Alcohol dehydrogenases are one of the most abundant classes of enzymes found in most of the organisms. More importantly, the ADH genes are involved in stress responses, elicitors and ABA regulation^30,31^. Plant’s adaptation to stress is a tightly regulated process that depends on meticulously effected changes in cell division and expansion. The flexibility of primary cell walls is a key factor regulating such changes by expanding rapidly while limiting the internal turgor pressure^32^. The cellulose synthase gene is responsible for the synthesis of cellulose, the main load-bearing polymer of the cell wall^33^ and plays a crucial role in stress response. A previous study on stress tolerance in *Arabidopsis* reported that plants may adapt to various abiotic stress conditions by modulating cell wall cellulose synthesis^34^. Studies also indicate that exposure to drought stress might inhibit certain enzymatic activities involved in cellulose synthesis, thereby leading to elevation in sugar content^35^ as the cell wall cellulose is the main sink of soluble sugars produced by photosynthesis in plants^36^. Another important DEG found was the Copper transport protein (CCH). Being a component of Cu–Zn SOD, Cu plays an important role under oxidative stress response due to the ROS scavenging activity of SOD^37^. Therefore, copper transport proteins could have an important role in regulating stress response in plants. The cysteine desulfurase gene, reported in this study to be a DEG, is a sulphur donating gene to the Fe-S complex formed inside various plant cells and the cluster has a role in response to induced abiotic stress^38^. The genes involved in the Fe-S cluster formation have been reported previously to be differentially expressed in response to both drought and salinity stress and^39^ cysteine desulfurase isone of them. The expression analysis also revealed that several 60 s and 40 s ribosomal proteins were found to show differential expression under both the stresses in roots. Similar studies have been previously reported explaining the roles of different ribosomal proteins in response to drought stress in rice^40^ and salt stress in Arabidopsis^41^ as well as abiotic stresses in roots of Soybean^42^.

Under the Molecular function category of Gene Ontology, transcripts related to “binding” and “catalytic activity” were over-represented in keeping with various previous studies^43^. Interaction between genes is often an important phenomenon that regulates certain biological processes. For example, genes regulating the process of metal ion binding or DNA binding may enhance the plants’ response to drought stress by modifying the expression of downstream target genes or accumulation of microRNAs^43^. Therefore, binding activity of genes helps in regulating stress responses of plants. TFs play a pivotal role in simultaneous regulation of large groups of stress-responsive genes by binding to specific cis-elements in their promoters. Therefore, TFs are considered to be promising candidates for enhancing abiotic stress tolerance in transgenic plants by concurrent regulation of a large number of downstream genes^44^. We found many differently expressed transcription factors in our study, which play vital roles in drought and salt stress resistance. In the present study a number of TFs were identified belonging to the WRKY, bHLH, MYB, MYB-like, C2H2 , and bZIP families, which is consistent with reports on pearl millet (*Pennisetum* glaucum)^45^, tobacco (*Nicotina benthamiana*)^46^, kabuli chickpea (*Cicer arietinum* L.)^47^, different varieties of maize(*Zea mays*)^48^ and tea oil camellia (*Camellia oleifera*)^11^. In roots ERF like TFs are found to have pronounced expression in response to abiotic stress and the involvement of ERF subfamily members, which bind to the ethylene-responsive element (ERE), in abiotic stress responses, has been reported earlier in carrots^49^, rice^50^, tomato^51^ etc. Along with this, some other TFs were also identified in the current study like BES1, TALE, CO-like TFs which have very few reports indicating their roles specific to abiotic stress. BES1 TF is found to regulate Brassinosteroid (BR) hormone pathway that has role in plant growth and development. The importance of application of BRs in agriculture has been recently enumerated by Rao et al. (2017) to improve plant growth and yield under various stress conditions^52^. The CO-like TF is found to be regulating abiotic stress response through an Abscisic acid-dependent manner which is previously being reported in *Arabidopsis*^53^. The current study reveals that the expression of CO-like TF is more evident in roots that might have some role in response to abiotic stress in the tissue which is showing similar pattern of expression as reported earlier in roots of *Medicago truncatula* against salinity stress^54^. Following this, several key TFs will be selected as candidates for further functional validation.

Simple sequence repeats are one of the most versatile molecular markers used widely in genetic diversity, genetic structure and genetic mapping studies^55^. In the present study, a number of SSRs were also identified. SSRs are classic genetic markers which are a great help for marker assisted genetic breeding. Given their wide distribution throughout the genome, codominant inheritance and high polymorphism^56^, SSRs have become desirable molecular markers for the construction of genetic linkage maps^57^, genetic relationship identification^58^, fingerprinting^59^ and genetic diversity analyses^60–62^. Several previous studies have also reported about the role of SSRs under various abiotic stresses^63,64^. In our study, we found that the proportion of tri-repeats were most abundant among all the SSRs examined under both salt and drought stresses which is consistent with other reports on wax gourd and peanut^64,65^. In the current study CCG/CGG tri-repeats are more abundant among all other tri-repeat SSRs. These CCG/CGG repeats have been found to be dominant in some monocotyledonous plants, such as rice and maize^66^ which is congruent with our findings. Further GO enrichment study of various DEGs revealed a number of important activities that have active involvement in response to abiotic stress and this can be used for marker assisted breeding in future.

## Conclusion

In this study, we have provided the first insight into the transcriptome of little millet in response to drought and salt stress. The study has provided valuable genomic resources and information regarding putative novel regulators of abiotic stress response in plants. These genes can be used for molecular characterization for improvement of important crop species. It has also unraveled genes involved in the regulation of metabolic network for adaptation to extreme climatic conditions. In addition, a large number of genic SSRs have also been identified, some of which may be associated with important genes that regulate abiotic stress response in plants. Based on the plan of work presented in this study, similar assets can be generated for a number of varieties of millets and an exhaustive database containing detailed information about all the resources will certainly facilitate further studies on molecular mechanisms of stress tolerance in millets, and will be made available in near future.

## Methods

### Plant Material, growth conditions and stress treatment

*P. sumatrense* (var. OLM20) was obtained from CPR (Centre for Pulse Research) Berhampur, Odisha and grown under aseptic conditions in green house (16 hr/8 hr light/dark; 65% RH) after being germinated on moist filter paper for 24 hrs. Three week old seedlings were subjected to drought stress by keeping in autoclaved distilled water containing 15% PEG-6000^67^. Similarly, salinity stress was induced by keeping the seedlings in autoclaved distilled water containing 150 mM NaCl^68^ .After 48 h of exposure, leaf and root tissues were harvested and frozen and used further for RNA isolation. Tissues from a set of seedlings that were maintained in autoclaved distilled water were used as control.

### RNA extraction and QC, preparation of libraries for sequencing and data quality control

The method described by Nayak et al., 2020^69^ was used to extract RNA and prepare libraries. Briefly, total RNA of each sample was extracted using TRIzol Reagent (Invitrogen)/RNeasy Mini Kit (Qiagen) and quantified and qualified by Agilent 2100 Bioanalyzer (Agilent Technologies, Palo Alto, CA, USA), NanoDrop (Thermo Fisher Scientific Inc.) and 1% agarose gel. 1 μg of total RNA with RIN value > 7 was used for library preparation according to the manufacturer’s protocol using the NEBNext RNA Library Prep Kit for Illumina. The quantified libraries were sequenced on Illumina HiSeq2500 after pooling, generating 2 × 150 Paired End data, which was subsequently curated using Trimmomatic v0.36.

### Quality assessment of reads and transcriptome assembly

Reads were filtered for removing low quality reads (> 70% sequences with phred score of Q30), reads shorter that 100 bp and adaptor sequences. Filtered reads from all the samples were pooled together for normalization using BBNORM v35(9). A kmer value of 31 was used and the dataset was normalized to a kmer-depth of 40. The software was also tasked to correct errors, ignore duplicate kmers and fix spikes. Trinity and BinPacker^8,9^ at k = 25 were used for de novo transcriptome assembly. For rnaSPAdes^10^ the assembly was done using auto-mode, which computed at k = 69. In all the cases, the assembly’s minimum length for transcript reporting were taken as 200 bp. The assemblies from BinPacker, rnaSPAdes and Trinity were subsequently concatenated and taken for filtering and identifying the true transcripts. Filtering the false transcripts from the true transcripts was done using the Evidential gene^70^ packages: tr2aacds.pl, and retained if the minimal CDS was 90 bp in length. The okay and alternative sets were merged from Evidential Gene prediction. CAP3 and CD-HIT-EST^71,72^ were used for removing redundant reads. Quality of the final assembled transcriptome was assessed using these parameters: (i)mapping back the clean reads onto assembled transcriptome, (ii) identifying long ORFs within the transcript sequences using Perl script ORFPredictor and Transdecoder (iii) comparing with Benchmarking Universal Single-Copy Orthologs (BUSCO)^73^ database. In addition to these, indicators like N50 and contig length distribution were also used to determine assembly quality.

### Functional annotation and distribution into biological pathways

Functional annotation using gene ontology (GO) terms was done as described by Nayak et al., 2020^69^. Briefly, BLASTx search (with a evalue cutoff of 10^−5^) was conducted against the Uniprot-Swissprot database (https://www.uniprot.org/uniprot/?query=reviewed:yes) using the standalone version of BLAST (ftp://ftp.ncbi.nlm.nih.gov/blast/executables/blast+/2.9.0/). GO annotations of the proteins were downloaded from GO database under Uniprot (ftp://ftp.ebi.ac.uk/pub/databases/GO/goa/UNIPROT/gene_association.goa_uniprot.gz), GO terms and their corresponding GO Slim terms were downloaded from Uniprot GOA database (ftp://ftp.ebi.ac.uk/pub/databases/GO/goa/goslim/goaslim.map), plant GOSlim terms were extracted from EBI's QuickGO-Beta server (http://www.ebi.ac.uk/QuickGO-Beta/). The plant GOSlim terms were assigned to our corresponding unigenes using linux shell commands. The KAAS (KEGG Automatic Annotation Server; https://www.genome.jp/kegg/kaas/) web-server was used to assign biological pathways to the transcripts. Protein function annotation was done by comparison against COG database using the web-server on WebMGA^74^; http://weizhong-lab.ucsd.edu/webMGA/server/).

### Differential gene expression analysis

The short reads from individual sample libraries (including replicates) were mapped onto the assembled transcriptome using Bowtie2 (https://sourceforge.net/projects/bowtie-bio/) and abundance was calculated using RSEM (RNA-Seq by Expectation–Maximization-http://deweylab.github.io/RSEM/package). Differentially expressed genes (DEGs) among the treated and control libraries were calculated by using the Empirical Analysis of Digital Gene Expression (edgeR) (http://biocon-ductor.org/packages/ release/ bioc/ html/edgeR.html) statistical package. The trimmed mean of M-values (TMM) method was used to calculate the normalization factors. The threshold FDR < 0.05 was adjusted to identify the differentially expressed genes by fold change (≥ 2)^69^.

### Identification of transcription factors

The peptide sequences for transcription factors of *Panicum halii* were downloaded from Plant TFDB (http://planttfdb.cbi.pku.edu.cn/download.php). BLASTX program of NCBI (ftp://ftp.ncbi.nlm.nih.gov/blast/executables/blast+/2.9.0/) was used to search the unigenes against the *Panicum halii* transcription factors using an e-value cutoff of 10^−5^^69^.

### Quantitative real-time PCR (qRT-PCR) analysis

To validate the RNA-seq results, few genes with differential expressions were randomly picked up for qRT-PCR analysis. Gene specific primers were designed using the PrimerQuest tool by IDT along with some housekeeping genes (primer sequences provided in Supplementary table 3). Total RNA was isolated from both the treated plants as well as from the control plants in two biological replicates and 1 µg total RNA was used for cDNA synthesis using the 1^st^ strand cDNA synthesis kit according to manufacturer’s protocol (Thermoscientific First strand cDNA synthesis kit, USA). qRT-PCR was performed on QuantStudio-3 real time PCR system (ThermoFisher Scientific, USA) with SYBR green chemistry (Applied Biosystems, USA) in three technical and two biological replicates. The expression was normalized by housekeeping genes like Elongation Factor (EF) and Ubiquitin (UB). The datagenerated from different PCR runs were analyzed by normalizing the CT values of stress specific genes with the CT values of housekeeping genes. The expression values were calculated using the comparative 2^_ΔΔCt^ method^75^. The qRT-PCR analysis is presented graphically by taking the RQ values.

### SSR identification

SSRs were identified in the unigenes of *P. sumatrense* using MISA software (https://github.com/cfljam/SSR_marker_design/blob/master/misa.pl) with the following parameters in the misa.ini file: a minimum of 6 repeats for dinucleotide, 4 repeats for trinucleotide and 3 repeats for tetra, penta and hexanucleotide with a maximum interruption of 100 bases between two SSRs. Differentially expressed SSR containing unigenes and their expression data was extracted from the list of DEGs obtained after differential expression analysis. The primers for amplification of SSR sequences identified with MISA were designed using the “primer3_core” program of the Primer3 v. 2.4.0 software (https://sourceforge.net/projects/primer3/) and the supporting programs available at MISA-web with slight modifications to the perl program “p3_out.pl” (https://webblast.ipk-gatersleben.de/misa/index.php?action=3&help=3;)^76^.

### Ethics approval and consent to participate

The authors declare that the study has been conducted without violating any ethical codes of conduct.

## Supplementary information

Supplementary Legends.

Supplementary Figure 1.

Supplementary Figure 2.

Supplementary Information.

## Data Availability

The raw reads from individual libraries have been deposited in the SRA database of NCBI under BioProject PRJNA554415, Accessions SRR9678143-SRR9678154.

## Abbreviations

Hrs: Hours
RNA-seq: RNA sequencing
TF: Transcription factor
qRT-PCR: Quantitative real-time polymerase chain reaction
SSR: Single sequence repeat
mM: Millimolar
PEG: Polyethylene glycol
SRA: Sequence read archive
NCBI: National Center for Biotechnology Information
Bp: Base pair
Kb: Killobases
ORF: Open reading frame
BUSCO: Benchmarking unique single copy orthologs
GO: Gene ontology
KEGG: Kyoto encyclopedia of genes and genomes
MAPK: MAP kinase
COG: Clusters of orthologous groups
DEG: Differentially expressed gene
NADH: Nicotinamide adenine dinucleotide
BLAST: Basic local alignment search tool
