# Supplementary material for: De-novo transcriptome analysis unveils differentially expressed genes regulating drought and salt stress response in Panicum sumatrense
Source: Sci Rep. 2020 Dec 4;10:21251. doi: 10.1038/s41598-020-78118-3 (PMC7718891; doi:10.1038/s41598-020-78118-3)
Supplement: Supplementary file 1 — Supplementary Legends. [file 41598_2020_78118_MOESM1_ESM.docx]

**Supplementary Information**

**Supplementary figure 1.** (a) Distribution of contigs according to their length. Quality assessment of transcriptome assembly through (b) percentage of reads mapped onto assembled transcriptome and (c) percentage of Unigenes coding for complete ORFs.

**Supplementary figure 2.** Co-relation between gene expression values obtained through qRT PCR and *in silico* expression analysis.

**Supplementary table 1.** *In silico* expression matrix for DEGs in Root (control vs salt, control vs drought) and Leaves (control vs drought, control vs salt) and the UniProt Ids for the Unigenes.

**Supplementary table 2.** *In silico* expression matrix and UniProt annotations for DEGs that coincide in both root and leaf tissue under drought and salinity stress.

**Supplementary table 3.** Primer sequences used for qRT PCR.

**Supplementary table 4.** *In silico* expression matrix for DEGs containing SSR markers in Leaves (control vs drought, control vs salt) and Roots (control vs drought, control vs salt)

**Supplementary table 5.** Primer sequences for SSRs identified in the transcriptome of Little millet.
