# Supplementary figures and images for: De-novo transcriptome analysis unveils differentially expressed genes regulating drought and salt stress response in Panicum sumatrense
Source: Sci Rep. 2020 Dec 4;10:21251. doi: 10.1038/s41598-020-78118-3 (PMC7718891; doi:10.1038/s41598-020-78118-3)

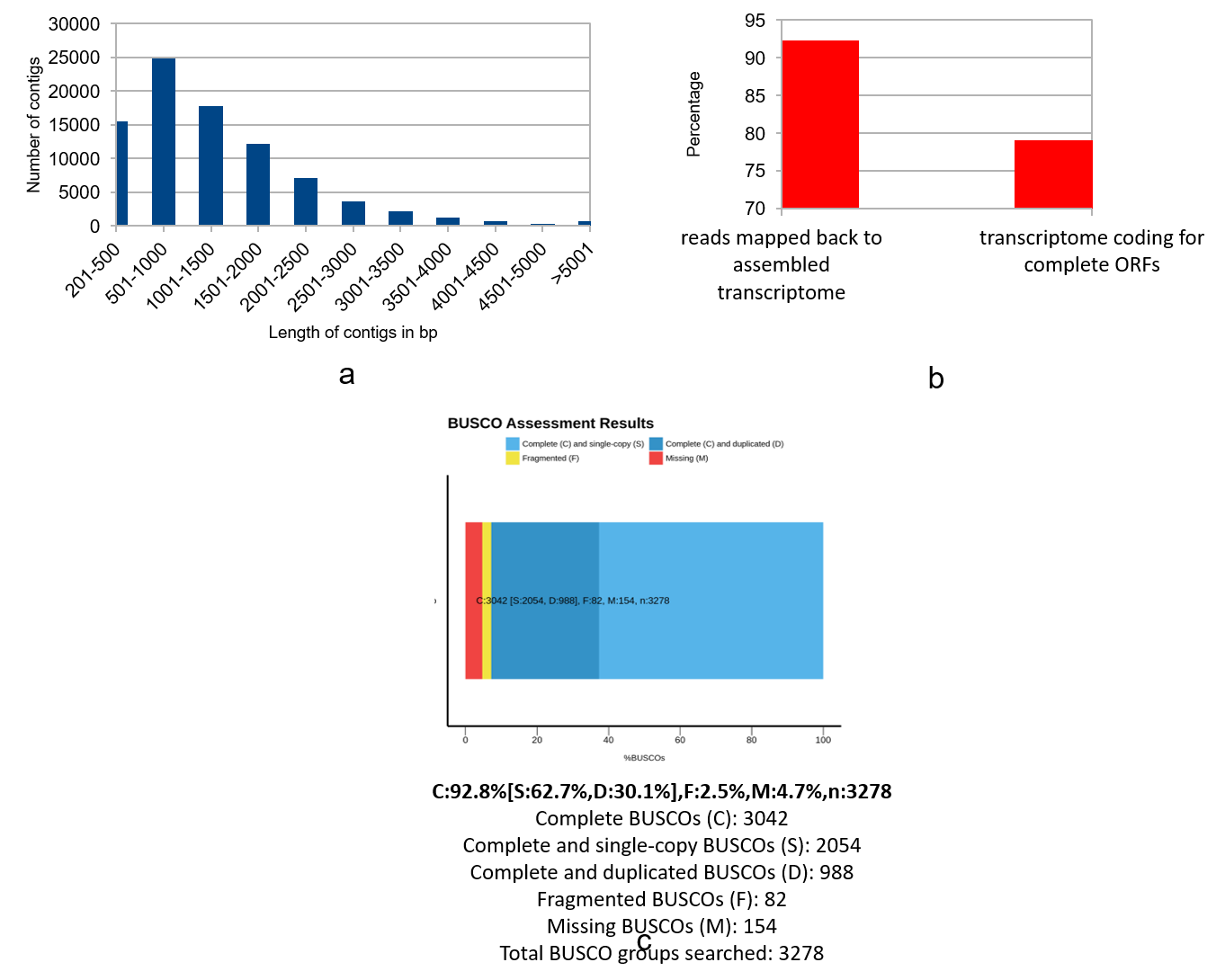

Supplement: Supplementary file 2 — Supplementary Figure 1. [file 41598_2020_78118_MOESM2_ESM.tiff]

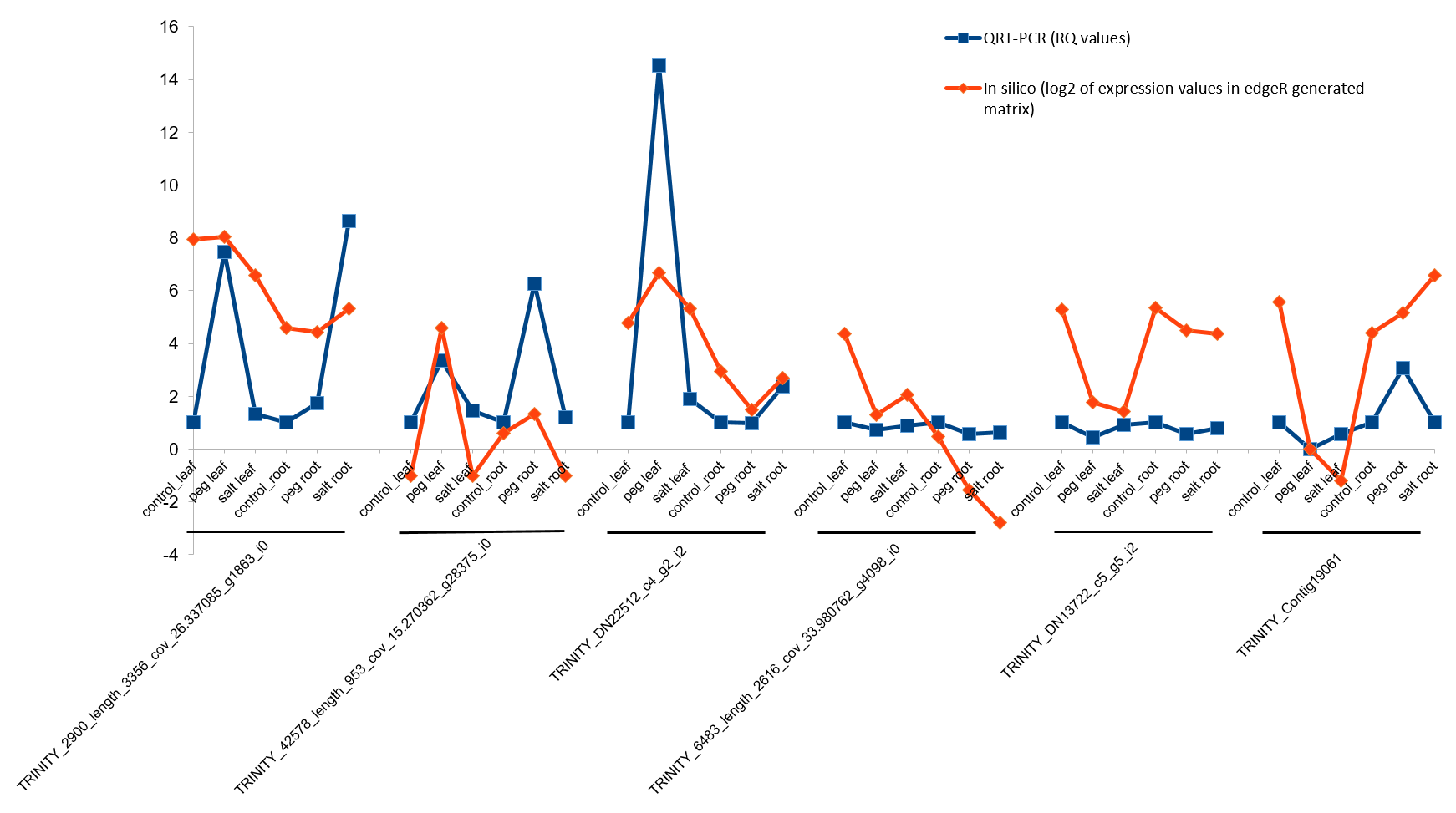

Supplement: Supplementary file 3 — Supplementary Figure 2. [file 41598_2020_78118_MOESM3_ESM.tiff]
