# Supplementary material for: De-novo transcriptome analysis unveils differentially expressed genes regulating drought and salt stress response in Panicum sumatrense
Source: Sci Rep. 2020 Dec 4;10:21251. doi: 10.1038/s41598-020-78118-3 (PMC7718891; doi:10.1038/s41598-020-78118-3)
Supplement: Supplementary file 4 — Supplementary Information. [file 41598_2020_78118_MOESM4_ESM.pdf]

Supplementary table 1. *In silico* expression matrix for DEGs in Root (control vs salt, control vs drought) and Leaves (control vs drought, control vs salt) and the UniProt Ids for the Unigenes.

Unigene ID	CONTROL1	CONTROL2	PEG_TREATED _ROOT1	PEG_TREATED _ROOT2	SALT_TREATED _ROOT1	SALT_TREATED _ROOT2
TRINITY_65049_length_582_cov_14.241715_g48223_i0	0.48	0	6.501	3.2	14.927	18.58
TRINITY_BIN_260_7	5.198	4.14	11.581	13.162	22.987	25.074
TRINITY_BIN_14087_1	16.074	14.417	13.394	9.589	4.368	2.584
TRINITY_5571_length_2759_cov_27.794796_g3523_i0	0	0	0	0	18.091	16.051
TRINITY_19576_length_1657_cov_44.516373_g12312_i0	23.88	19.318	12.408	11.267	3.163	4.946
TRINITY_DN20548_c0_g1_i7	12.017	11.708	2.662	2.641	0	0
TRINITY_Contig7688	17.858	16.372	9.046	7.767	2.792	2.206
TRINITY_DN14225_c4_g1_i6	17.138	6.866	100.665	91.502	210.917	257.892
TRINITY_56313_length_703_cov_26.293375_g39935_i0	1.338	0.779	3.892	6.389	102.138	82.184
TRINITY_DN18007_c2_g1_i9	15.611	8.567	107.516	59.949	246.479	184.084
TRINITY_67776_length_550_cov_21.802495_g50885_i0	9.993	11.589	7.88	12.365	0	2.272
TRINITY_7865_length_2443_cov_8.386689_g1146_i3	26.367	23.526	12.26	12.168	2.769	3.409
TRINITY_Contig6231	4.881	1.702	22.823	21.892	38.545	25.263
TRINITY_Contig3260	0	0	0	0	150.342	109.196
TRINITY_Contig7879	1.209	3.318	33.884	17.511	110.468	115.122
TRINITY_Contig11236	0	0	0	0	9.614	6.628
TRINITY_Contig16004	11.417	13.604	33.555	32.144	77.518	83.12
TRINITY_BIN_8418_1	9.984	8.449	0	0.394	1.914	1.303
TRINITY_67661_length_551_cov_31.452282_g50772_i0	0	0	0	0	31.701	31.925
TRINITY_30573_length_1257_cov_24.303030_g19609_i0	12.986	8.821	2.768	2.278	1.914	0.947
TRINITY_41707_length_971_cov_17.417960_g27694_i0	4.323	6.417	24.095	17.667	21.445	27.358
TRINITY_Contig9510	18.236	14.552	64.926	53.911	114.937	114.978
TRINITY_DN16475_c0_g3_i3	13.939	9.668	13.437	11.95	2.308	1.448
TRINITY_2655_length_3447_cov_11.604204_g1694_i0	0.369	0.356	0	0	6.777	7.263
TRINITY_68925_length_538_cov_16.682303_g52016_i0	3.474	2.26	5.653	6.493	27.738	20.284
TRINITY_Contig394	19.471	19.158	7.201	3.801	5.156	4.021
TRINITY_BIN_4741_2	0.575	0	3.086	4.443	15.479	11.484
TRINITY_22285_length_1540_cov_10.903467_g14094_i0	0	0	0	0	8.038	6.539
TRINITY_Contig12088	10.104	9.448	0.605	0.487	0	0.223
TRINITY_Contig3876	0	0	0	0	10.042	8.722
TRINITY_Contig10678	0	0	0	0	6.721	7.552
TRINITY_Contig852	0.112	0	0	0	29.325	27.625
TRINITY_DN16219_c5_g2_i11	38.402	26.235	10.764	13.7	6.214	4.734
TRINITY_Contig502	0	0.068	0	0	74.737	56.263
TRINITY_27101_length_1366_cov_84.599846_g17263_i0	41.627	45.164	105.194	99.952	314.023	304.709
TRINITY_Contig18354	11.048	8.051	24.551	26.862	48.553	49.569
TRINITY_38209_length_1052_cov_25.051882_g25026_i0	3.688	3.97	9.608	17.439	67.24	72.426
TRINITY_Contig12651	3.568	3.132	28.412	23.455	30.958	31.802
TRINITY_Contig2518	9.684	10.624	3.574	4.95	1.52	1.972
TRINITY_66951_length_559_cov_21.744898_g50075_i0	12.703	14.485	0	0	0	0
TRINITY_48039_length_847_cov_27.322622_g32756_i0	77.807	69.502	81.682	62.341	15.254	7.909
TRINITY_BIN_528_1	7.806	4.402	35.888	40.252	39.693	47.53
TRINITY_Contig9816	0	0	0	0	12.642	9.747
TRINITY_Contig5870	35.588	29.866	7.466	7.425	7.801	5.982
TRINITY_16773_length_1789_cov_243.990698_g10536_i0	531.233	531.789	1944.032	1739.243	4121.994	3827.498
TRINITY_19122_length_1678_cov_28.188937_g12037_i0	11.923	14.51	7.456	8.875	3.062	2.562
TRINITY_DN22097_c1_g1_i4	10.902	8.305	4.168	2.641	2.049	1.782
TRINITY_Contig4251	0	0	0	0	9.963	5.369
TRINITY_Contig11791	0	0	0	0	9.287	7.252
TRINITY_Contig2095	0	0	0	0	32.421	40.413
TRINITY_92840_length_339_cov_18.951852_g75769_i0	21.718	16.762	24.085	59.13	127.467	89.636
TRINITY_DN14358_c1_g1_i16	7.428	7.23	21.603	18.951	41.9	36.391
TRINITY_Contig3989	0	0	0	0	6.766	7.764
TRINITY_60583_length_640_cov_29.705779_g43944_i0	87.182	85.163	6.915	7.57	9.175	8.287
TRINITY_44102_length_923_cov_32.492974_g29548_i0	0	0	0	0	42.26	34.431
TRINITY_DN21554_c1_g3_i3	2.101	2.269	1.188	8.202	10.436	22.612

TRINITY_Contig6759	14.402	12.317	7.339	5.012	1.508	3.275
TRINITY_Contig11628	68.26	55.729	5.133	6.783	4.987	4.99
TRINITY_Contig1454	0.446	0	5.462	4.991	15.4	13.4
TRINITY_Contig16907	0	0	0	0	47.022	38.263
TRINITY_DN21277_c4_g1_i4	10.053	9.304	4.571	4.981	1.272	2.462
TRINITY_Contig9981	15.705	13.68	5.26	4.515	3.276	2.996
TRINITY_Contig2740	1.227	3.183	7.074	8.916	278.259	234.299
TRINITY_Contig892	3.843	3.911	2.153	0.466	54.418	55.45
TRINITY_BIN_6960_2	6.528	14.341	2.354	3.883	1.25	1.27
TRINITY_BIN_3983_4	10.199	11.276	3.882	2.817	1.508	2.038
TRINITY_Contig3277	0	0	0	0	53.36	30.443
TRINITY_Contig12675	1.613	2.506	7.699	7.156	38.241	45.158
TRINITY_DN19859_c0_g3_i1	28.46	21.672	13.002	12.416	5.156	5.759
TRINITY_55972_length_709_cov_21.023438_g39626_i0	17.738	16.914	8.59	8.813	4.03	2.061
TRINITY_5840_length_2712_cov_37.498297_g3680_i0	8.44	8.973	21.624	23.02	50.703	39.354
TRINITY_51087_length_791_cov_30.051247_g35333_i0	15.774	12.724	25.909	24.108	1.666	2.562
TRINITY_53973_length_742_cov_16.849926_g37829_i0	0.3	0	0	0	13.464	12.041
TRINITY_55261_length_721_cov_34.769939_g38988_i0	0	0	0	0	268.431	220.632
TRINITY_Contig18236	5.876	5.545	2.46	2.692	0	0.134
TRINITY_56538_length_699_cov_30.077778_g40144_i0	31.36	30.493	24.657	15.585	7.542	6.851
TRINITY_37696_length_1064_cov_33.411055_g23900_i1	33.735	26.971	2.535	2.019	2.375	2.551
TRINITY_68995_length_537_cov_34.965812_g52085_i0	0.575	0.457	1.103	0	31.836	20.061
TRINITY_Contig6747	0	0	0	0	8.916	6.06
TRINITY_DN14203_c1_g2_i2	26.771	19.928	12.217	2.32	4.165	2.128
TRINITY_67782_length_550_cov_19.070686_g50891_i0	0	0	0	0	14.432	13.077
TRINITY_63246_length_604_cov_37.749533_g46488_i0	23.245	20.631	8.516	4.235	3.445	3.72
TRINITY_Contig14655	69.118	64.304	21.105	22.244	12.349	12.932
TRINITY_DN22340_c0_g1_i6	33.358	31.695	16.491	11.236	7.081	7.641
TRINITY_17490_length_1755_cov_25.852906_g10980_i0	10.936	8.753	1.867	1.781	0.563	0.958
TRINITY_Contig458	11.142	7.213	3.807	2.765	1.835	1.314
TRINITY_Contig17213	0	0	0	0	16.267	9.245
TRINITY_Contig10753	0	0	0	0	14.409	14.347
TRINITY_DN11924_c0_g4_i2	8.38	17.778	49.018	30.725	87.706	119.578
TRINITY_41593_length_973_cov_27.050885_g27607_i0	16.932	17.625	43.439	41.07	91.578	90.75
TRINITY_DN16219_c5_g2_i3	14.599	9.862	2.238	5.209	0.63	2.985
TRINITY_53381_length_752_cov_13.234261_g37309_i0	0	0	0	0	7.711	13.322
TRINITY_DN12626_c1_g1_i16	8.852	0	21.317	21.788	56.804	54.325
TRINITY_Contig2180	3.122	4.317	14.338	14.229	34.357	29.786
TRINITY_16882_length_1784_cov_29.045481_g10600_i0	0	0	0	0	24.71	19.014
TRINITY_Contig11785	3.431	4.986	9.396	6.845	18.181	23.715
TRINITY_Contig15699	11.502	10.768	5.398	3.417	1.722	2.573
TRINITY_48460_length_839_cov_22.741558_g33092_i0	0	0	0	0	26.815	16.319
TRINITY_20204_length_1628_cov_35.821681_g12723_i0	26.316	23.966	7.689	8.668	6.158	5.603
TRINITY_21957_length_1555_cov_10.988560_g13869_i0	0	0	0	0	8.533	5.748
TRINITY_Contig16745	1.424	2.794	14.19	13.131	39.907	37.438
TRINITY_DN29075_c0_g1_i1	0	0.923	3.224	1.833	10.492	8.688
TRINITY_DN13194_c0_g2_i1	0.206	0	0	0	36.204	31.345
TRINITY_64300_length_591_cov_28.559387_g47498_i0	0	0	0	0	37.386	15.985
TRINITY_37441_length_1071_cov_20.983034_g24503_i0	0	0	0	0	14.623	17.11
TRINITY_27021_length_1369_cov_34.769231_g17209_i0	0.12	0	0	0	291.43	242.442
TRINITY_84968_length_398_cov_8.121581_g67905_i0	5.678	2.243	19.8	15.15	36.136	39.154
TRINITY_BIN_3370_3	97.141	102.628	11.676	10.697	14.015	16.909
TRINITY_41193_length_983_cov_15.234136_g27305_i0	0	0	0	0	13.599	10.593
TRINITY_BIN_32460_1	0	0	0	0	8.657	6.193
TRINITY_67928_length_548_cov_31.467641_g51035_i0	0	0	0	0	730.308	592.632
TRINITY_Contig3132	4.315	3.936	18.135	16.207	1.711	0
TRINITY_70538_length_521_cov_28.570796_g53591_i0	0	0	0	0	64.91	53.568
TRINITY_28199_length_1332_cov_25.331750_g18001_i0	24.841	27.75	15.494	13.7	2.33	3.353
TRINITY_Contig2892	5.893	5.782	18.262	25.029	40.042	44.033
TRINITY_Contig11874	0	0	0	0	9.749	7.976
TRINITY_Contig4303	0	0	0	0	9.073	4.667
TRINITY_53589_length_748_cov_31.410898_g37489_i0	28.409	23.314	3.775	0.86	0.462	1.248
TRINITY_Contig13250	18.742	15.848	9.842	11.702	1.396	0.468
TRINITY_Contig11474	0	0	0	0	12.192	12.097
TRINITY_41475_length_976_cov_20.943771_g27513_i0	0.978	0.152	6.119	2.661	9.231	8.644

TRINITY_57032_length_691_cov_47.041801_g40609_i0	0.343	0	1.336	2.962	207.36	154.154
TRINITY_BIN_17565_1	0	0	0	0	17.044	17.388
TRINITY_31785_length_1222_cov_16.156982_g20461_i0	0	0	0	0	9.355	12.175
TRINITY_46015_length_885_cov_35.610294_g31093_i0	1.261	1.456	7.646	4.681	11.471	12.22
TRINITY_Contig8835	0	0	0.053	0	9.355	9.067
TRINITY_Contig2198	3.525	5.545	4.984	11.277	544.855	466.638
TRINITY_66381_length_565_cov_32.981855_g49518_i0	0	0	0	0.487	645.912	531.044
TRINITY_62611_length_612_cov_30.747698_g45876_i0	0.437	0	1.273	1.243	234.186	196.672
TRINITY_DN15512_c3_g3_i2	6.039	2.743	9.545	5.105	26.849	45.035
TRINITY_Contig19343	43.583	38.721	163.417	149.121	471.806	427.919
TRINITY_50839_length_796_cov_8.536451_g35117_i0	0	0	0	0	8.646	5.915
TRINITY_BIN_4110_9	50.11	44.918	20.383	19.044	10.109	9.557
TRINITY_45575_length_894_cov_14.859394_g30748_i0	0	0	0.435	0	9.22	11.729
TRINITY_BIN_4432_1	17.37	22.544	38.752	41.578	112.776	113.518
TRINITY_48690_length_835_cov_13.203655_g33293_i0	0	0	0	0	9.512	9.39
TRINITY_DN14942_c2_g1_i20	4.932	6.806	18.379	16.548	54.564	52.844
TRINITY_DN19190_c2_g1_i6	163.539	159.093	48.784	56.345	7.723	10.448
TRINITY_Contig11940	0	0	0	0	19.847	11.729
TRINITY_Contig5192	7.008	4.698	5.25	5.199	127.399	134.404
TRINITY_DN16874_c0_g2_i5	19.239	19.843	12.122	10.376	5.561	3.386
TRINITY_DN19882_c0_g1_i7	13.827	11.454	3.998	4.018	0.822	2.685
TRINITY_Contig9627	0	0	0	0	7.193	8.031
TRINITY_30409_length_1262_cov_28.735960_g19501_i0	14.642	12.986	7.912	8.554	2.702	1.871
TRINITY_DN12349_c2_g1_i19	2.813	1.109	15.696	13.763	49.206	41.66
TRINITY_BIN_14439_1	0	0	0	0	27.524	23.626
TRINITY_BIN_5888_4	10.405	8.652	1.951	1.678	1.396	2.417
TRINITY_97110_length_303_cov_118.200855_g80038_i0	33.847	35.293	68.309	108.641	562.607	372.736
TRINITY_41670_length_971_cov_30.574279_g27665_i0	6.914	7.382	30.66	31.429	0.608	0.624
TRINITY_BIN_5_1	413.206	410.292	1338.927	1083.744	2359.164	2257.278
TRINITY_14077_length_1938_cov_22.678438_g8815_i0	8.861	7.517	3.277	3.593	1.936	0.446
TRINITY_DN18997_c2_g1_i1	11.082	9.744	5.324	3.055	2.139	1.437
TRINITY_68514_length_542_cov_30.860465_g51615_i0	0	0	0	0	37.881	44.768
TRINITY_Contig17910	1.561	1.659	0.18	0.186	19.25	17.555
TRINITY_54844_length_728_cov_11.807284_g38608_i0	0	0	0	0	10.087	7.53
TRINITY_Contig1860	0	0	0	0.394	112.472	112.661
TRINITY_BIN_5761_2	16.254	16.466	97.399	71.982	225.507	233.13
TRINITY_53732_length_746_cov_20.404727_g37612_i0	1.801	2.574	1.453	1.729	12.878	12.565
TRINITY_DN14370_c0_g2_i2	1.681	2.193	6.703	12.209	25.689	17.188
TRINITY_Contig869	0	0	0	0	7.047	7.441
TRINITY_Contig11412	0.858	0.584	7.827	8.574	11.415	12.376
TRINITY_Contig12789	7.565	8.931	8.484	4.981	0.698	2.161
TRINITY_BIN_268_1	48.034	57.236	11.486	13.079	0	5.703
TRINITY_Contig3328	5.215	3.708	10.054	13.545	1.407	0.457
TRINITY_DN12182_c0_g1_i1	12.377	9.964	3.892	2.724	2.195	1.448
TRINITY_Contig3003	0	0	0	0	27.806	27.681
TRINITY_66168_length_568_cov_20.166333_g49310_i0	29.104	40.457	1.464	4.329	0	5.291
TRINITY_38131_length_1054_cov_23.567513_g24973_i0	20.08	25.744	12.737	17.004	5.178	5.224
TRINITY_Contig19892	0.292	0.356	4.475	1.667	12.755	9.357
TRINITY_32360_length_1206_cov_23.992084_g20854_i0	21.229	22.4	3.139	1.843	1.767	1.069
TRINITY_Contig3626	0	0	0	0	45.221	45.704
TRINITY_DN14174_c3_g1_i22	4.435	4.241	13.702	11.981	23.494	24.751
TRINITY_BIN_29296_3	0	0	0	0	10.436	7.731
TRINITY_46179_length_882_cov_38.496925_g31223_i0	3.954	3.361	22.504	26.51	48.136	50.315
TRINITY_Contig12112	10.568	9.177	14.996	22.565	67.668	63.771
TRINITY_DN16359_c2_g1_i3	116.963	106.192	9.99	14.643	14.297	11.919
TRINITY_Contig11266	0	0	0	0	22.346	19.906
TRINITY_Contig16227	17.575	12.021	63.197	49.479	97.545	56.275
TRINITY_67666_length_551_cov_29.454357_g50777_i0	6.013	3.843	21.465	16.414	42.249	28.538
TRINITY_Contig14548	7.214	7.991	26.047	17.563	42.947	35.433
TRINITY_7372_length_2502_cov_32.012741_g4658_i0	13.021	12.512	4.507	4.774	1.531	1.158
TRINITY_27003_length_1370_cov_23.229055_g17194_i0	21.718	13.689	7.392	5.727	4.03	4.133
TRINITY_Contig482	0	0	0	0	6.495	8.132
TRINITY_57372_length_686_cov_35.113452_g40926_i0	0	0	0	0	108.599	85.504
TRINITY_DN14379_c0_g4_i3	20.981	21.951	5.366	6.907	4.221	4.333
TRINITY_57520_length_684_cov_28.788618_g41058_i0	43.145	49.261	4.072	2.009	6.462	9.88

TRINITY_DN15837_c0_g1_i4	20.886	23.771	15.049	11.36	4.267	5.725
TRINITY_Contig6289	0	0.626	3.797	1.481	35.078	23.102
TRINITY_Contig4162	0	0	0	0	6.124	7.875
TRINITY_20316_length_1624_cov_24.274598_g12454_i1	40.623	47.001	3.16	4.204	5.696	4.4
TRINITY_DN13377_c1_g1_i3	11.657	10.768	3.701	6.058	2.116	1.504
TRINITY_10786_length_2168_cov_17.008576_g6767_i0	0.292	0.398	0.255	0.29	9.287	6.572
TRINITY_81865_length_422_cov_35.141643_g64814_i0	1.149	0	0	0	1312.109	972.118
TRINITY_57894_length_678_cov_19.193760_g41409_i0	9.984	12.521	0	0.342	2.184	1.493
TRINITY_Contig3023	110.71	113.295	71.161	65.044	13.824	11.217
TRINITY_DN18481_c2_g1_i3	0	0	0	0	98.569	87.442
TRINITY_DN14058_c0_g1_i6	17.172	13.57	9.301	6.98	2.522	1.749
TRINITY_Contig4066	0	0	0	0	8.488	7.151
TRINITY_63265_length_604_cov_23.091589_g46506_i0	0	0	0	0	30.991	17.667
TRINITY_Contig19341	6.768	4.419	35.994	26.158	92.727	88.355
TRINITY_25493_length_1420_cov_37.376018_g14764_i1	14.839	15.619	7.286	6.555	1.767	3.732
TRINITY_Contig18163	1.235	2.252	11.581	9.931	25.982	27.625
TRINITY_Contig11483	0	0	0	0	27.907	21.042
TRINITY_Contig10480	14.222	12.292	20.33	14.581	2.353	2.829
TRINITY_37837_length_1061_cov_23.567540_g24777_i0	0.875	0.135	4.635	2.703	12.496	11.317
TRINITY_DN13829_c0_g1_i3	0.738	1.151	7.805	4.556	10.289	13.411
TRINITY_Contig2187	0	0	0	0	26.871	19.404
TRINITY_45521_length_895_cov_23.578692_g30705_i0	3.8	6.087	11.104	9.506	0	0.234
TRINITY_Contig3543	0	0	0	0	18.924	15.951
TRINITY_Contig7696	0	0	0	0	40.515	23.737
TRINITY_31407_length_1231_cov_39.333046_g20200_i0	37.347	33.456	9.428	6.762	2.364	2.685
TRINITY_Contig3397	14.436	15.23	11.136	10.2	1.407	0.869
TRINITY_57945_length_677_cov_37.174342_g41460_i0	0.36	0.559	6.692	6.151	25.115	23.904
TRINITY_Contig11854	40.323	36.156	205.605	141.312	338.215	326.108
TRINITY_25507_length_1420_cov_28.694301_g5628_i2	9.538	7.5	2.566	2.133	0.574	0.646
TRINITY_DN11686_c0_g1_i1	0	0	0	0	26.792	19.048
TRINITY_43006_length_945_cov_17.203196_g28709_i0	0	0	0	0	15.366	10.359
TRINITY_DN9975_c0_g2_i1	0	0	0	0	48.418	67.714
TRINITY_64669_length_586_cov_61.348162_g47857_i0	52.46	50.7	115.81	116.811	365.03	296.734
TRINITY_58163_length_674_cov_40.960331_g41660_i0	0.36	1.126	5.228	3.096	361.799	312.629
TRINITY_DN19001_c0_g1_i18	14.213	12.825	10.542	8.523	3.625	2.473
TRINITY_Contig13338	63.525	65.803	10.287	12.603	5.719	5.469
TRINITY_Contig5876	0	0	0	0	43.116	35.289
TRINITY_DN19026_c3_g2_i5	23.056	28.495	7.434	6.514	2.916	7.218
TRINITY_50521_length_801_cov_33.516393_g34846_i0	3.997	2.904	23.576	16.942	28.515	32.56
TRINITY_DN12269_c0_g1_i1	0	0	0	0	35.753	37.851
TRINITY_Contig12059	44.475	44.385	146.173	134.954	412.198	384.721
TRINITY_Contig772	2.908	3.166	9.364	5.064	22.762	21.532
TRINITY_DN20940_c3_g1_i13	0	0	10.754	4.712	12.079	12.465
TRINITY_BIN_26502_1	0	0	0	0	12.406	13.278
TRINITY_14533_length_1913_cov_28.687636_g9105_i0	27.645	21.02	9.555	8.595	3.996	4.378
TRINITY_Contig16276	0.309	0	0	0	417.973	329.672
TRINITY_Contig16753	80.878	72.118	269.045	238.272	827.886	731.759
TRINITY_34076_length_1159_cov_24.367890_g5130_i3	51.928	57.887	26.439	34.867	14.826	9.814
TRINITY_Contig15157	14.659	10.777	5.812	2.454	0.664	0.891
TRINITY_DN13939_c3_g1_i11	1.861	3.327	8.845	6.928	23.19	34.52
TRINITY_43790_length_929_cov_40.166279_g29307_i0	203.03	198.551	767.175	540.614	1301.853	1296.655
TRINITY_Contig12441	36.669	41.083	298.263	215.003	739.235	759.774
TRINITY_Contig13538	8.423	16.592	39.24	29.389	3.163	1.626
TRINITY_DN14423_c0_g1_i5	13.004	12.284	3.988	4.142	1.632	2.651
TRINITY_Contig19496	7.299	6.569	22.727	19.375	35.292	38.252
TRINITY_BIN_3224_4	0	0	0.392	0.228	29.224	29.864
TRINITY_53642_length_747_cov_28.740413_g37537_i0	0	0	0	0	180.331	153.53
TRINITY_BIN_11269_1	18.407	27.2	0.583	1.419	0.462	0
TRINITY_Contig3167	0	0	0	0	14.252	17.622
TRINITY_62084_length_619_cov_34.760000_g45366_i0	0	0	0	0	23.044	29.496
TRINITY_Contig14251	10.49	10.531	6.915	4.463	2.083	2.729
TRINITY_Contig8405	10.525	7.043	3.457	2.413	0.259	0.847
TRINITY_Contig9557	18.682	17.812	10.945	12.396	3.794	3.698
TRINITY_DN11924_c0_g1_i6	2.762	5.477	34.032	29.586	96.048	95.751
TRINITY_Contig11151	8.749	10.633	0.361	0.176	1.114	0.769

TRINITY_Contig3355	0	0	0	0	25.475	28.416
TRINITY_Contig7649	11.374	14.62	100.761	67.663	140.491	150.767
TRINITY_Contig19164	1.75	1.913	11.846	18.06	36.485	29.73
TRINITY_DN19161_c0_g4_i5	10.645	10.286	4.762	9.382	1.16	1.96
TRINITY_Contig2061	0	0	0	0	17.111	17.733
TRINITY_Contig5034	3.354	0.66	6.321	4.608	22.256	23.994
TRINITY_Contig6932	0	0	0	0	29.99	25.709
TRINITY_BIN_8228_2	16.04	16.101	11.189	9.216	1.554	3.119
TRINITY_Contig8164	13.046	13.469	7.805	7.601	1.497	2.373
TRINITY_Contig13935	0	0	0	0	34.898	31.758
TRINITY_Contig4093	0	0	0	0	7.126	7.062
TRINITY_Contig2476	12.66	13.003	7.562	7.446	2.882	2.896
TRINITY_79784_length_438_cov_28.607046_g62748_i0	22.43	21.672	17.202	2.775	0	3.13
TRINITY_64526_length_588_cov_48.736031_g47720_i0	2.393	0	10.945	10.045	25.577	31.312
TRINITY_BIN_12695_3	43.934	41.744	8.516	4.432	3.76	2.852
TRINITY_Contig16972	0.214	0	0	0	11.325	11.551
TRINITY_Contig10537	0	0	0	0	8.938	6.995
TRINITY_Contig11732	1.741	0.533	6.809	5.209	12.169	14.158
TRINITY_Contig11919	39.131	48.076	15.293	12.872	4.616	2.818
TRINITY_Contig3915	0	0	0	0	262.026	209.481
TRINITY_DN15899_c0_g3_i5	19.848	20.656	9.046	8.026	4.053	3.954
TRINITY_14577_length_1911_cov_22.877307_g9127_i0	9.71	8.44	4.157	3.883	0.124	0.256
TRINITY_65719_length_573_cov_36.797619_g48876_i0	0	0	0	0	324.166	266.413
TRINITY_21697_length_1566_cov_32.713427_g13361_i1	98.153	98.945	19.705	14.746	5.178	7.909
TRINITY_Contig18448	102.939	120.143	73.304	74.219	20.837	24.328
TRINITY_38445_length_1046_cov_43.795292_g25200_i0	0.18	0.135	0.17	0.342	211.3	169.993
TRINITY_40589_length_998_cov_16.700753_g26843_i0	181.981	196.426	38.996	28.581	28.841	28.739
TRINITY_90218_length_358_cov_22.591696_g73147_i0	4.349	6.933	36.206	22.554	91.522	53.557
TRINITY_Contig11674	0	0	0	0	46.594	43.108
TRINITY_91186_length_351_cov_16.436170_g74115_i0	7.111	5.672	28.528	12.375	131.069	85.492
TRINITY_39112_length_1031_cov_29.926195_g25714_i0	18.819	21.409	49.336	43.442	2.477	3.041
TRINITY_DN21756_c2_g1_i7	8.543	6.62	1.739	3.065	0	0
TRINITY_54832_length_728_cov_18.057663_g38598_i0	0	0	0	0	14.927	15.283
TRINITY_DN15047_c0_g1_i5	28.975	28.444	7.328	9.848	2.826	3.442
TRINITY_Contig17327	42.956	41.075	23.236	26.138	0	2.027
TRINITY_61087_length_633_cov_21.398936_g44418_i0	2.033	2.226	7.445	2.34	12.507	13.968
TRINITY_38821_length_1038_cov_22.473684_g25495_i0	103.557	134.027	11.74	10.273	19.115	19.393
TRINITY_43050_length_944_cov_30.869714_g28743_i0	0	0	0.202	0	45.243	39.343
TRINITY_73310_length_494_cov_25.134118_g56326_i0	0.721	4.495	13.596	5.302	41.236	52.387
TRINITY_36886_length_1084_cov_11.959606_g24103_i0	0	0	0	0	8.466	9.39
TRINITY_16908_length_1783_cov_27.085764_g10616_i0	3.157	2.59	9.99	10.117	0	0.278
TRINITY_Contig6548	0	0	0	0	23.032	20.941
TRINITY_Contig19163	6.785	5.782	15.452	10.853	96.937	62.524
TRINITY_Contig4097	0	0	0	0	8.139	7.831
TRINITY_61717_length_624_cov_29.054054_g45012_i0	59.442	49.041	44.351	45.71	10.942	5.224
TRINITY_Contig2733	10.027	10.599	4.21	4.774	2.51	2.361
TRINITY_Contig6727	0	0	0	0	61.015	51.763
TRINITY_61523_length_627_cov_38.087814_g44828_i0	4.143	1.905	3.192	1.968	24.879	20.563
TRINITY_Contig1853	2.342	2.184	12.005	8.015	18.372	21.008
TRINITY_Contig13657	32.209	33.007	28.984	61.616	6.068	6.349
TRINITY_36254_length_1100_cov_23.384093_g16284_i1	1.321	0.897	13.766	11.567	30.631	23.437
TRINITY_Contig11110	1.947	2.201	9.163	7.218	11.584	15.416
TRINITY_Contig672	69.204	63.424	29.334	23.217	7.621	7.775
TRINITY_Contig4006	0	0	0	0	7.317	7.006
TRINITY_BIN_19720_1	0	0	0	0	32.635	28.16
TRINITY_Contig3663	0	0	0	0	124.866	109.441
TRINITY_BIN_24068_1	0	0	0	0	17.381	18.079
TRINITY_28625_length_1318_cov_31.466773_g11111_i2	24.995	24.711	54.002	47.294	141.876	124.68
TRINITY_BIN_3188_1	0	0	0	0	150.342	129.514
TRINITY_Contig1849	291.053	289.285	45.772	44.498	70.595	74.543
TRINITY_Contig18835	0	0	0	0	9.287	13.144
TRINITY_BIN_22453_1	35.983	34.37	3.595	5.789	5.055	4.378
TRINITY_Contig1428	8.946	5.604	70.589	46.455	283.662	259.385
TRINITY_Contig11736	130.885	102.797	10.796	11.433	5.91	6.071
TRINITY_BIN_8556_1	0	0	0	0.238	15.591	12.866

TRINITY_DN16971_c0_g3_i2	10.019	46.366	127.688	92.341	376.434	345.066
TRINITY_Contig2221	0	0	0	0	38.973	31.334
TRINITY_92339_length_343_cov_13.273723_g75268_i0	0	16.838	39.006	25.112	107.519	92.543
TRINITY_43501_length_935_cov_35.773672_g29083_i0	0	0.161	0	0	21.445	16.943
TRINITY_50011_length_811_cov_22.212938_g23964_i1	16.435	12.817	8.23	6.545	3.996	1.37
TRINITY_47110_length_865_cov_11.727387_g31982_i0	0	0.186	0	0	10.436	8.644
TRINITY_DN12991_c2_g2_i4	8.955	8.271	23.491	16.165	72.036	69.296
TRINITY_88074_length_374_cov_32.947541_g71004_i0	74.161	74.675	0	0	2.904	0
TRINITY_Contig9566	12.669	10.286	3.372	4.008	2.477	2.762
TRINITY_Contig12393	0.755	2.362	21.391	14.591	41.055	31.769
TRINITY_55657_length_714_cov_25.865116_g39340_i0	0	0	0	0	26.353	21.342
TRINITY_Contig6398	35.888	52.707	74.248	72.23	370.726	286.998
TRINITY_Contig12692	10.602	8.973	29.992	36.814	61.33	62.49
TRINITY_Contig1685	0	0	0	0	13.216	8.377
TRINITY_Contig12572	11.623	13.189	104.738	74.519	98.457	98.859
TRINITY_DN21450_c0_g9_i1	1.956	3.826	17.838	20.773	12.079	12.186
TRINITY_65730_length_573_cov_26.823413_g48887_i0	0	0	0	0	33.22	32.192
TRINITY_20429_length_1619_cov_24.490323_g12855_i0	3.989	3.97	8.208	8.481	0	0.1
TRINITY_Contig3184	10.19	8.973	2.651	2.848	1.734	1.649
TRINITY_DN14950_c2_g4_i1	0	0	0	0	12.698	13.902
TRINITY_DN13949_c1_g3_i2	9.401	7.721	3.023	0.746	2.128	0.724
TRINITY_DN20665_c1_g2_i18	13.81	10.269	4.497	2.62	3.096	1.938
TRINITY_Contig19495	30.33	21.714	137.264	89.949	208.429	206.173
TRINITY_BIN_11503_2	31.728	24.592	8.611	8.057	5.021	5.492
TRINITY_BIN_3370_10	27.568	25.304	5.631	4.857	4.998	7.129
TRINITY_Contig3289	0	0	0	0	42.26	29.541
TRINITY_Contig3882	7.823	8.601	0.689	1.015	0	0.746
TRINITY_68244_length_545_cov_14.882353_g51349_i0	0	0	0	0	14.747	12.765
TRINITY_49762_length_815_cov_30.804290_g34196_i0	0	0	0	0	75.492	57.143
TRINITY_Contig3801	0	0	0	0	14.398	6.082
TRINITY_DN13853_c0_g1_i5	10.67	11.818	4.624	4.122	1.711	1.303
TRINITY_Contig1198	0	0.254	0.318	0	334.849	280.07
TRINITY_Contig16691	4.254	3.776	12.631	8.989	0.867	0.635
TRINITY_71652_length_510_cov_28.621315_g54693_i0	0	0	0	0	45.041	34.687
TRINITY_Contig10200	0	0	0	0	16.447	13.356
TRINITY_38231_length_1051_cov_39.189409_g13247_i1	8.337	8.499	3.479	3.272	1.193	1.114
TRINITY_11321_length_2125_cov_20.094358_g7084_i0	25.106	21.24	3.521	2.102	2.488	2.618
TRINITY_Contig20084	10.971	10.226	2.683	1.729	0.191	0
TRINITY_Contig12689	39.826	47.61	126.118	117.049	469.442	436.863
TRINITY_Contig19481	0	0	0	0	224.404	196.103
TRINITY_35020_length_1133_cov_26.391917_g22781_i0	12.034	14.789	8.527	8.637	2.409	1.504
TRINITY_BIN_5760_7	7.428	4.834	12.514	12.872	34.166	26.522
TRINITY_68916_length_538_cov_23.624733_g52008_i0	18.527	15.382	4.613	3.873	69.66	87.052
TRINITY_40668_length_996_cov_18.574973_g2381_i2	4.28	1.778	10.404	13.856	18.271	18.179
TRINITY_Contig3486	0	0	0	0	10.852	12.041
TRINITY_Contig3150	11.185	14.51	25.845	28.064	123.155	110.778
TRINITY_BIN_3907_5	4.495	7.238	15.409	12.427	35.888	36.202
TRINITY_DN20778_c0_g1_i2	23.074	21.782	9.874	11.008	4.976	5.302
TRINITY_Contig4770	31.154	34.853	207.291	183.128	580.619	541.649
TRINITY_Contig3165	0	0	0	0	78.272	67.091
TRINITY_Contig13563	5.069	5.782	27.69	29.244	35.483	35.445
TRINITY_53056_length_757_cov_24.606105_g37031_i0	14.942	10.506	7.965	4.505	2.691	2.451
TRINITY_23380_length_1496_cov_10.729502_g14820_i0	0	0	0	0	6.935	6.594
TRINITY_DN19858_c2_g2_i15	0	0.99	7.137	8.564	36.834	40.145
TRINITY_Contig11886	12.892	12.605	6.565	5.23	3.175	1.849
TRINITY_Contig7535	0	0	0	0	476.309	352.195
TRINITY_85878_length_391_cov_5.164596_g68813_i0	3.036	3.606	12.684	4.039	45.986	32.56
TRINITY_DN13988_c1_g1_i3	10.25	9.16	6.225	4.691	1.644	2.038
TRINITY_28977_length_1308_cov_16.570621_g18529_i0	0	0	0	0	10.672	9.858
TRINITY_18219_length_1719_cov_27.636970_g11446_i0	7.746	8.686	9.502	8.833	1.351	1.559
TRINITY_74576_length_482_cov_31.380145_g57578_i0	0	0	0	0	68.051	48.845
TRINITY_19266_length_1672_cov_20.601372_g9683_i1	13.081	13.435	6.66	5.903	2.387	1.236
TRINITY_Contig5505	0	0	0	0	11.708	9.39
TRINITY_Contig3614	0	0	0	0	10.706	12.832
TRINITY_Contig199	9.727	10.396	31.476	25.961	76.235	64.985

TRINITY_Contig16054	0	0	0	0	250.824	205.093
TRINITY_Contig10734	15.217	17.812	6.247	6.804	1.734	2.796
TRINITY_Contig7944	147.311	115.098	70.589	67.581	22.571	10.716
TRINITY_Contig19342	8.26	10.286	67.248	40.77	146.525	128.957
TRINITY_Contig3460	0	0	0	0	14.162	14.815
TRINITY_Contig3865	0	0	0	0	11.831	10.727
TRINITY_Contig1280	44.243	34.988	11.422	10.148	8.634	6.884
TRINITY_DN16926_c2_g1_i2	18.176	19.378	5.822	4.567	3.039	2.607
TRINITY_DN18016_c0_g1_i5	5.695	5.164	21.921	20.67	30.091	32.426
TRINITY_33486_length_1175_cov_25.656420_g21644_i0	13.407	17.93	28.772	33.78	75.919	92.577
TRINITY_54953_length_726_cov_16.487062_g33650_i1	0	0	0	0	15.445	8.232
TRINITY_42166_length_961_cov_30.558296_g28056_i0	0	0	0	0	116.592	101.633
TRINITY_42139_length_962_cov_15.804031_g28035_i0	0	0	0	0	11.606	10.716
TRINITY_Contig12647	3.997	5.435	15.844	12.251	43.161	41.783
TRINITY_Contig11545	0	0	0	0	172.451	162.497
TRINITY_95149_length_321_cov_30.690476_g78077_i0	0	2.904	0	0	197.746	174.349
TRINITY_DN12328_c0_g2_i3	0	0	0	0	38.714	28.739
TRINITY_Contig11784	0	0	0	0	10.132	10.237
TRINITY_60722_length_638_cov_31.063269_g44074_i0	1.201	0.313	0.774	1.139	196.362	192.216
TRINITY_Contig3523	2.796	3.987	11.39	8.771	0.709	0
TRINITY_Contig2993	10.396	6.679	4.847	4.339	1.002	1.214
TRINITY_BIN_26423_2	0	0	0	0	9.501	5.302
TRINITY_Contig14402	17.361	14.696	10.054	9.372	3.051	4.133
TRINITY_BIN_15986_1	39.054	39.89	19.196	19.344	4.188	3.932
TRINITY_86997_length_382_cov_30.827476_g69930_i0	0	0	0	0	872.038	677.635
TRINITY_34846_length_1138_cov_26.140318_g22654_i0	0	0	0	0	17.021	20.184
TRINITY_33056_length_1186_cov_38.690242_g2617_i5	12.497	11.572	4.666	4.349	3.85	2.038
TRINITY_Contig2916	0.386	0	0	0	38.568	31.379
TRINITY_Contig9790	0	0	0	0	8.769	7.318
TRINITY_BIN_17586_1	0	0	0	0	16.075	17.11
TRINITY_Contig17547	12.532	6.265	0.626	0.311	0	0
TRINITY_48405_length_840_cov_25.081712_g33047_i0	13.57	13.452	15.844	10.242	2.634	1.805
TRINITY_BIN_4522_2	2.462	3.471	12.292	6.048	21.299	11.25
TRINITY_92701_length_340_cov_43.162362_g75630_i0	8.226	15.357	71.087	86.935	157.107	147.637
TRINITY_Contig12499	86.908	87.187	695.409	454.642	1636.984	1548.655
TRINITY_36311_length_1098_cov_33.822157_g20413_i1	12.489	14.053	6.628	6.048	3.602	2.852
TRINITY_Contig5964	0	0	0	0	1033.411	886.18
TRINITY_Contig16502	0	0	0	0.145	82.167	75.991
TRINITY_DN19240_c1_g3_i4	34.533	34.328	88.533	88.603	249.586	262.47
TRINITY_DN16605_c1_g1_i2	13.312	5.757	5.43	3.749	1.148	2.239
TRINITY_Contig13705	32.423	27.132	26.693	22.554	6.642	4.545
TRINITY_Contig1625	0	0	0	0	18.518	11.484
TRINITY_DN12685_c0_g1_i3	19.025	15.56	7.88	5.975	2.634	3.631
TRINITY_Contig11560	2.256	2.362	10.128	11.909	15.355	17.978
TRINITY_57649_length_682_cov_23.876020_g25291_i1	78.905	86.603	14.9	16.269	14.477	17.956
TRINITY_Contig5843	38.976	35.784	6.045	9.993	4.053	10.927
TRINITY_DN14618_c0_g1_i1	8.921	7.56	2.089	3.117	1.126	2.284
TRINITY_20602_length_1612_cov_21.245625_g12967_i0	2.247	2.76	5.769	5.509	0.281	0.245
TRINITY_30372_length_1263_cov_52.868509_g19474_i0	8.2	7.467	40.682	30.197	116.851	109.13
TRINITY_10436_length_2197_cov_35.778665_g6436_i1	10.439	8.601	5.716	4.215	2.094	1.114
TRINITY_1782_length_3817_cov_28.259338_g1144_i0	0	0	0	0	19.7	19.961
TRINITY_DN20051_c0_g1_i1	33.221	28.233	11.92	15.326	7.272	6.405
TRINITY_38067_length_1055_cov_41.061866_g24926_i0	0	0	0	0	42.17	34.186
TRINITY_BIN_24640_1	9.838	35.919	97.356	55.268	448.346	237.296
TRINITY_Contig3308	0	0	0	0	544.765	488.047
TRINITY_Contig11403	262.782	309.873	97.303	352.815	28.323	75.868
TRINITY_74342_length_484_cov_40.106024_g57346_i0	0	0	0	0	746.845	581.972
TRINITY_Contig3993	0	0	0	0	5.471	8.51
TRINITY_Contig10452	0	0	0	0	7.824	5.803
TRINITY_Contig12433	31.969	25.71	184.299	144.253	246.693	214.773
TRINITY_30609_length_1256_cov_27.197978_g19641_i0	20.886	23.331	5.398	4.577	3.141	3.331
TRINITY_39915_length_1012_cov_33.579003_g26322_i0	7.059	8.677	17.923	17.428	56.332	48.366
TRINITY_DN15010_c0_g2_i2	6.836	5.68	0.392	2.755	0	0.434
TRINITY_DN9558_c0_g1_i2	10.602	16.948	2.789	19.872	0	1.147
TRINITY_56315_length_703_cov_22.380126_g39937_i0	0	0	0	0	16.875	13.824

TRINITY_55667_length_714_cov_23.524031_g39350_i0	0	0	0	0.311	27.839	26.088
TRINITY_DN14632_c0_g1_i11	69.169	66.387	9.651	7.798	13.711	11.685
TRINITY_Contig7275	0	0	0	0	7.61	5.803
TRINITY_20620_length_1611_cov_24.781453_g11960_i1	32.68	30.73	14.476	15.388	5.539	6.94
TRINITY_Contig7936	0	0	0	0	6.98	9.568
TRINITY_Contig18242	0.283	0.22	6.957	3.531	8.691	11.239
TRINITY_47723_length_853_cov_40.008929_g32493_i0	0	0	0	0	59.202	64.841
TRINITY_Contig11763	1.261	1.405	5.239	3.914	17.483	13.556
TRINITY_Contig1365	8.852	7.831	2.556	4.101	1.227	1.905
TRINITY_BIN_1201_2	0	0	0	0.58	116.344	92.766
TRINITY_DN16457_c1_g1_i16	63.045	66.7	8.113	15.409	2.803	1.793
TRINITY_45320_length_899_cov_14.833735_g30534_i0	0	0	0	0	12.192	11.395
TRINITY_BIN_4432_10	3.062	4.918	11.263	5.126	24.293	21.955
TRINITY_DN15299_c0_g2_i4	13.175	17.642	7.328	4.287	1.205	3.186
TRINITY_37796_length_1062_cov_24.088620_g23008_i1	14.247	13.088	1.188	1.201	0.259	0.267
TRINITY_Contig3483	0	0	0	0	6.833	7.898
TRINITY_BIN_11288_13	13.698	13.172	6.278	5.747	2.713	2.918
TRINITY_Contig18445	34.302	35.03	20.129	29.866	6.315	9.212
TRINITY_49783_length_815_cov_21.584450_g34212_i0	0.515	0.406	2.28	1.605	15.58	13.289
TRINITY_Contig14131	14.771	12.504	1.177	1.481	1.193	2.183
TRINITY_Contig3457	0	0	0	0	20.027	12.409
TRINITY_DN14113_c0_g1_i1	17.232	13.409	8.198	7.725	3.94	2.93
TRINITY_BIN_4529_4	33.512	28.207	11.337	8.699	4.751	5.937
TRINITY_Contig11773	0	0	0	0	20.95	22.679
TRINITY_Contig3506	0	0	0	0	9.524	12.108
TRINITY_65408_length_577_cov_32.879921_g48572_i0	6.905	9.642	62.423	43.245	87.469	70.588
TRINITY_Contig6512	112.323	100.215	49.049	43.649	12.574	12.52
TRINITY_DN19360_c1_g1_i2	45.247	38.019	17.509	14.819	11.437	8.343
TRINITY_DN12754_c0_g1_i3	0	0	0	0	60.598	60.842
TRINITY_DN18526_c2_g1_i3	19.102	10.159	8.866	7.176	2.263	2.462
TRINITY_BIN_10250_3	2.702	7.433	13.363	14.115	30.721	25.887
TRINITY_BIN_9607_1	9.727	7.323	10.383	5.903	1.024	2.161
TRINITY_Contig7214	6.459	4.665	39.07	37.001	42.159	35.556
TRINITY_BIN_19291_1	0	0	0	0	25.329	23.704
TRINITY_48406_length_840_cov_24.446174_g33048_i0	9.624	8.652	1.92	5.24	0.754	1.036
TRINITY_Contig11879	0	0	0	0	14.207	8.009
TRINITY_55144_length_723_cov_15.974006_g38880_i0	0	0	0	0	10.661	8.856
TRINITY_46079_length_884_cov_27.233129_g31144_i0	16.615	17.024	12.196	10.791	1.734	0.234
TRINITY_55064_length_724_cov_27.656489_g38808_i0	30.39	57.024	1.845	10.014	0.968	7.931
TRINITY_DN14018_c0_g6_i1	1.75	1.82	5.547	4.349	48.283	34.175
TRINITY_Contig17980	11.125	10.785	0.212	0.3	0.585	0.501
TRINITY_Contig1725	0	0	0	0	20.263	9.546
TRINITY_DN15262_c1_g1_i13	8.277	13.215	4.359	6.099	2.893	1.181
TRINITY_Contig13924	0	0	0	0	24.372	12.465
TRINITY_24771_length_1446_cov_32.411038_g15738_i0	0	0	0	0.114	29.213	22.523
TRINITY_DN22469_c3_g1_i1	4.194	3.539	12.111	9.682	21.986	18.814
TRINITY_DN10023_c0_g1_i1	0	0	0	0	7.61	6.728
TRINITY_Contig3608	0	0	0	0	14.139	15.394
TRINITY_52383_length_769_cov_26.514286_g36450_i0	23.957	16.897	4.433	3.013	1.745	2.685
TRINITY_82812_length_415_cov_25.352601_g65758_i0	0	0	0	0	50.23	59.895
TRINITY_BIN_30079_1	0	0	0	0	7.554	5.893
TRINITY_Contig15239	0.909	1.651	8.198	2.61	209.792	181.723
TRINITY_DN10531_c0_g1_i2	8.029	9.668	1.612	11.826	0	0.88
TRINITY_Contig6898	0	0	0	0	13.982	11.952
TRINITY_53374_length_752_cov_21.819912_g37303_i0	25.184	18.252	2.015	2.848	4.987	4.333
TRINITY_Contig3647	0	0	0	0	27.513	24.25
TRINITY_Contig10092	0	0	0	0	5.809	7.519
TRINITY_51638_length_782_cov_14.040673_g35804_i0	0	0	0	0	9.332	9.869
TRINITY_13996_length_1942_cov_29.030432_g2771_i3	34.405	31.39	8.251	8.191	5.798	6.026
TRINITY_13609_length_1966_cov_29.056405_g7710_i1	14.041	13.587	3.86	3.024	2.083	1.359
TRINITY_Contig13592	0	0	0	0	20.702	13.634
TRINITY_DN13209_c0_g3_i4	82.885	76.452	10.277	4.919	0	2.818
TRINITY_Contig11237	46.567	43.166	15.42	15.575	8.105	9.435
TRINITY_Contig4143	0	0	0	0	6.822	9.836
TRINITY_44798_length_909_cov_12.097619_g30102_i0	0	0	0	0	8.657	8.922

TRINITY_BIN_288_4	0.12	0	0	0.228	9.85	7.597
TRINITY_Contig849	16.031	15.492	62.009	63.252	113.361	120.67
TRINITY_DN16926_c2_g1_i6	21.247	18.116	9.736	2.413	4.199	3.932
TRINITY_Contig9287	12.72	9.837	2.227	2.154	1.069	1.359
TRINITY_Contig10432	8.715	10.032	4.019	6.358	1.272	2.551
TRINITY_Contig1057	58.971	53.629	1.633	2.299	4.008	1.604
TRINITY_Contig10571	0	0	0	0	11.055	8.466
TRINITY_67030_length_558_cov_24.044990_g50152_i0	38.805	31.644	8.983	7.611	5.921	4.534
TRINITY_1142_length_4255_cov_35.603679_g738_i0	20.689	19.488	12.249	9.786	3.535	3.13
TRINITY_DN20465_c1_g1_i4	68.046	66.573	7.965	4.536	4.942	1.704
TRINITY_80665_length_431_cov_32.560773_g63622_i0	5.37	8.466	32.176	19.406	82.876	59.327
TRINITY_BIN_5994_1	67.651	54.569	21.794	17.377	8.477	7.619
TRINITY_58030_length_676_cov_24.397035_g41538_i0	52.34	44.207	15.25	10.956	8.252	7.853
TRINITY_Contig20013	94.842	94.806	299.737	245.065	789.96	642.513
TRINITY_BIN_5117_3	17.987	25.185	1.464	1.16	1.79	0.847
TRINITY_Contig12007	2.985	1.456	5.398	6.783	19.464	24.094
TRINITY_Contig908	0.36	0.466	0	0.114	208.013	186.557
TRINITY_51964_length_776_cov_26.790665_g36081_i0	18.536	15.111	6.819	4.329	3.006	3.23
TRINITY_51040_length_792_cov_26.226833_g35292_i0	0	0	0	0	38.613	34.386
TRINITY_Contig1358	0	0	0	0	24.89	21.732
TRINITY_Contig2280	0	0	0	0	36.575	30.031
TRINITY_25536_length_1419_cov_25.470370_g16246_i0	30.63	26.929	14.169	10.335	5.358	4.946
TRINITY_DN18183_c1_g7_i2	14.427	16.245	10.446	10.552	2.803	2.74
TRINITY_5504_length_2770_cov_36.719733_g3491_i0	3.86	5.02	19.005	17.729	19.002	18.58
TRINITY_Contig16170	7.437	6.265	52.051	31.491	105.763	78.308
TRINITY_Contig10019	15.843	14.679	10.542	9.817	2.24	2.016
TRINITY_Contig15333	0	0	0	0.445	147.212	114.254
TRINITY_BIN_2061_1	30.536	29.392	9.439	10.956	2.938	3.888
TRINITY_Contig10257	0	0	0	0	25.723	25.464
TRINITY_Contig689	0	0	0	0	23.607	22.991
TRINITY_Contig16406	24.18	26.099	10.987	9.299	3.479	3.865
TRINITY_DN18929_c2_g3_i4	10.542	9.371	1.336	0.88	1.385	0
TRINITY_49361_length_822_cov_34.589641_g33852_i0	0	0	0	0	76.133	66.623
TRINITY_Contig14231	10.276	11.031	2.927	9.237	0.563	2.573
TRINITY_Contig11238	1.149	0.279	0	0	188.268	180.598
TRINITY_Contig12664	431.288	439.659	1611.79	1334.733	3970.797	3480.137
TRINITY_Contig913	8.114	7.907	2.354	5.261	0.518	0.847
TRINITY_BIN_5058_1	22.053	19.327	6.087	4.629	2.555	2.673
TRINITY_BIN_2562_1	0	0	0	0	137.79	159.422
TRINITY_46620_length_874_cov_9.279503_g31572_i0	2.084	1.084	4.741	3.366	66.598	66.188
TRINITY_Contig4707	0	0.389	1.432	0.466	392.43	260.231
TRINITY_54202_length_738_cov_12.753363_g38037_i0	0	0	0	0	11.719	9.914
TRINITY_DN14268_c0_g1_i2	11.279	15.661	2.938	1.823	0.574	1.582
TRINITY_77395_length_457_cov_42.881443_g60374_i0	5.361	7.026	25.612	20.39	82.516	79.7
TRINITY_DN14117_c0_g10_i1	46.019	53.612	48.159	63.625	10.312	10.649
TRINITY_30223_length_1268_cov_27.085071_g19373_i0	0	0	0	0	27.997	18.324
TRINITY_DN16405_c0_g2_i1	34.902	27.462	14.561	4.401	0	2.974
TRINITY_Contig12690	3.68	6.73	21.497	21.115	76.358	54.604
TRINITY_DN14973_c0_g1_i1	11.777	14.442	7.816	5.83	3.231	2.607
TRINITY_70362_length_523_cov_11.724670_g53418_i0	4.958	5.824	27.128	21.933	26.871	31.969
TRINITY_72664_length_500_cov_30.515081_g55692_i0	38.187	47.898	108.057	113.86	4.312	4.289
TRINITY_Contig8360	0.952	1.862	6.215	2.247	13.25	13.857
TRINITY_Contig11305	34.113	28.14	19.927	15.192	7.486	4.923
TRINITY_Contig11750	0.283	0	0	0	270.165	210.105
TRINITY_DN14971_c0_g1_i2	50.359	40.762	24.488	17.201	9.051	7.742
TRINITY_DN20731_c0_g1_i1	25.964	23.814	12.748	15.316	6.36	3.754
TRINITY_DN10571_c0_g1_i1	9.598	13.045	3.203	3.438	0.417	2.584
TRINITY_43818_length_929_cov_20.308140_g29324_i0	0	0	0	0	14.162	15.717
TRINITY_BIN_21114_1	0	0	0	0	14.511	11.273
TRINITY_46918_length_869_cov_21.746250_g31825_i0	1.638	1.727	19.429	15.347	32.781	31.156
TRINITY_Contig12657	0.197	0.152	3.51	2.32	10.965	11.106
TRINITY_BIN_10732_1	3.045	3.522	15.685	13.732	20.421	11.707
TRINITY_DN11532_c0_g1_i1	0	0	0	0	9.58	7.341
TRINITY_Contig8202	0.326	2.76	82.933	42.251	150.758	146.936
TRINITY_35958_length_1108_cov_20.987488_g23445_i0	0	0	0	0	12.349	13.723

TRINITY_55672_length_714_cov_18.810853_g39354_i0	0	0	0	0	16.413	12.531
TRINITY_Contig19131	9.238	11.361	43.142	26.8	52.583	48.7
TRINITY_98759_length_284_cov_36.069767_g81687_i0	566.538	691.797	424.911	444.949	70.302	105.086
TRINITY_Contig12329	3.105	4.444	19.397	9.185	15.952	25.152
TRINITY_71748_length_509_cov_94.388636_g54789_i0	4.203	9.041	21.847	18.474	67.893	60.775
TRINITY_DN14546_c0_g4_i3	21.675	16.923	4.359	7.808	3.929	3.286
TRINITY_32389_length_1205_cov_22.623239_g20878_i0	1.604	1.134	29.748	21.384	55.645	51.73
TRINITY_33167_length_1183_cov_40.830341_g21423_i0	0	0	0	0	36.586	26.388
TRINITY_Contig951	17.267	16.059	5.515	7.539	2.736	2.885
TRINITY_Contig1736	0	0	0	0	81.222	66.11
TRINITY_Contig10335	2.342	3.259	12.535	11.464	26.241	28.059
TRINITY_BIN_16716_1	0	0	0	0	31.093	22.991
TRINITY_BIN_2103_8	9.298	11.733	0.901	3.521	0	2.551
TRINITY_Contig15635	3.285	3.843	1.008	1.999	16.436	14.247
TRINITY_71460_length_512_cov_25.898420_g54503_i0	0	0	0	0	74.974	61.967
TRINITY_Contig10811	0	0	0	0	21.31	23.648
TRINITY_Contig14036	0	0	0	0	9.096	9.234
TRINITY_23048_length_1509_cov_11.136806_g14059_i1	0	0	0	0	6.597	7.664
TRINITY_DN12034_c0_g1_i31	2448.812	2619.268	3589.982	2919.026	189.81	351.46
TRINITY_34463_length_1148_cov_30.198332_g22359_i0	23.099	21.435	9.216	6.255	1.891	2.295
TRINITY_Contig5150	0	0	0	0	325.517	285.238
TRINITY_Contig1453	0	0	0	0	10.233	9.39
TRINITY_32679_length_1197_cov_27.954787_g21080_i0	120.352	111.542	64.395	60.673	9.76	11.44
TRINITY_Contig11592	0	0.178	3.807	1.999	9.118	8.666
TRINITY_43907_length_927_cov_29.083916_g29398_i0	0	0	0	0	40.47	29.674
TRINITY_Contig12340	24.909	29.409	133.086	112.058	418.108	381.758
TRINITY_BIN_18031_1	0	0	0	0	294.041	223.762
TRINITY_BIN_0_103	11.211	10.235	47.066	51.612	149.779	140.453
TRINITY_DN14639_c5_g1_i5	37.784	36.156	140.096	152.082	130.799	171.029
TRINITY_Contig1572	2.076	1.261	7.127	6.048	15.952	14.648
TRINITY_BIN_13116_7	2.659	2.37	8.961	9.009	0	0
TRINITY_DN9994_c0_g1_i3	0	0	0	0	767.04	660.191
TRINITY_63377_length_603_cov_11.930712_g46613_i0	0	0	0	0	11.055	10.727
TRINITY_BIN_2964_5	13.518	11.742	7.349	6.141	3.67	2.417
TRINITY_DN22133_c0_g2_i9	18.785	17.287	7.636	6.773	2.319	3.23
TRINITY_81365_length_426_cov_25.333333_g64318_i0	3.354	8.804	3.129	0	424.334	417.849
TRINITY_88192_length_373_cov_51.273026_g71122_i0	80.234	69.697	81.183	70.035	523.004	542.807
TRINITY_Contig2802	0	0	0	0	23.156	25.408
TRINITY_45254_length_900_cov_18.270758_g30482_i0	0	0	0	0	20.274	10.448
TRINITY_Contig16398	1.012	1.422	6.193	7.86	14.511	15.082
TRINITY_Contig19503	4.023	8.703	13.193	18.215	44.523	42.161
TRINITY_66384_length_565_cov_28.739919_g49521_i0	0	0.406	0.498	0.487	978.442	840.989
TRINITY_Contig14629	91.154	85.748	179.653	133.08	473.224	442.678
TRINITY_Contig17176	0	0	0	0	210.613	198.91
TRINITY_Contig758	25.681	26.311	9.852	7.249	4.12	3.732
TRINITY_59791_length_651_cov_30.013746_g43197_i0	10.01	6.315	14.497	12.106	39.626	42.117
TRINITY_48717_length_834_cov_30.366013_g33315_i0	27.783	28.647	85.288	69.351	1.52	2.094
TRINITY_18526_length_1705_cov_25.967604_g11646_i0	66.905	64.753	12.631	9.434	15.22	13.345
TRINITY_88096_length_374_cov_21.773770_g71026_i0	43.411	45.951	1.676	44.488	8.713	11.106
TRINITY_Contig11385	0	0	0	0	8.995	9.279
TRINITY_DN10665_c1_g1_i1	0	0	0	0	213.45	174.081
TRINITY_50268_length_806_cov_24.518318_g34625_i0	8.44	5.757	29.748	27.463	35.483	28.438
TRINITY_Contig1591	52.349	55.136	36.811	29.244	0	1.125
TRINITY_Contig17502	0	0	0	0	47.877	43.309
TRINITY_BIN_6018_5	0	0.085	0	0.114	40.74	44.267
TRINITY_47699_length_854_cov_15.402548_g2667_i5	0.48	2.057	6.787	5.343	16.514	17.199
TRINITY_Contig12510	0	0.254	1.4	2.413	7.835	8.466
TRINITY_68627_length_541_cov_26.548729_g51725_i0	2.282	0.897	1.527	2.402	19.419	17.355
TRINITY_Contig4020	0	0	0	0	10.177	6.316
TRINITY_29544_length_1289_cov_52.859016_g18918_i0	3.019	3.996	21.751	14.198	52.651	47.898
TRINITY_Contig18138	47.468	40.44	157.849	134.809	227.207	211.319
TRINITY_17018_length_1777_cov_15.492974_g10686_i0	0.532	0.279	4.677	2.113	7.993	9.802
TRINITY_BIN_11939_5	8.792	8.339	0	0.342	0	0
TRINITY_44607_length_913_cov_17.214455_g29951_i0	0.652	3.175	3.171	6.079	12.552	15.06
TRINITY_Contig10061	54.202	49.972	234.461	169.376	469.318	447.735

TRINITY_Contig3665	1.964	0.77	2.768	0.891	311.76	186.223
TRINITY_52786_length_762_cov_14.047619_g36801_i0	6.665	5.198	0	0.28	0	0
TRINITY_Contig17951	2.05	1.998	11.454	10.366	16.526	13.668
TRINITY_DN15798_c1_g1_i3	12.841	11.175	5.271	3.914	2.296	1.537
TRINITY_Contig13804	52.966	56.702	17.265	24.242	10.244	8.332
TRINITY_BIN_1443_3	10.473	11.894	0.615	2.672	0.63	0.88
TRINITY_Contig10213	0	0	0	0	14.938	11.963
TRINITY_49097_length_827_cov_34.362797_g33630_i0	3.38	4.334	16.884	18.267	29.393	20.05
TRINITY_Contig2003	0	0	0	0	28.796	25.419
TRINITY_Contig17571	17.335	12.394	2.832	2.651	2.589	2.506
TRINITY_87290_length_380_cov_28.405145_g70222_i0	74.65	68.613	11.008	10.459	2.713	1.738
TRINITY_Contig8037	35.417	34.319	25.941	22.596	6.315	6.984
TRINITY_Contig8136	0.515	1.126	4.581	4.774	12	11.819
TRINITY_Contig16118	4.538	4.436	13.532	17.905	30.733	25.341
TRINITY_Contig7277	105.212	96.135	19.185	18.34	18.293	18.079
TRINITY_Contig18675	10.087	11.437	5.271	7.984	2.026	2.206
TRINITY_Contig19933	1.355	1.405	4.178	4.267	15.107	16.853
TRINITY_Contig10266	2.402	1.371	6.543	6.938	16.841	13.1
TRINITY_65584_length_575_cov_22.092885_g48743_i0	24.257	19.555	6.787	6.472	1.565	3.119
TRINITY_90468_length_356_cov_57.822300_g73397_i0	11.134	15.856	111.334	89.39	166.395	162.597
TRINITY_Contig3852	0	0	0	0	21.85	15.194
TRINITY_BIN_5211_2	9.684	8.288	2.132	4.929	0.371	0.512
TRINITY_Contig14076	0	0	0	0	9.636	10.526
TRINITY_Contig12434	0	0	0	0	51.378	35.5
TRINITY_BIN_6782_2	9.761	8.279	3.479	3.842	2.116	0.802
TRINITY_Contig11504	0	0	0	0	20.747	14.013
TRINITY_Contig12628	7.617	3.674	12.567	16.445	32.612	41.181
TRINITY_DN21347_c0_g3_i11	7.711	7.602	3.203	0.932	0.484	0.512
TRINITY_Contig2388	9.89	11.014	42.22	48.992	51.029	89.235
TRINITY_Contig11279	0	0	0	0	14.353	12.821
TRINITY_DN14702_c3_g2_i4	9.452	4.665	29.78	29.71	48.53	68.806
TRINITY_Contig11088	0	0	0	0	16.256	16.174
TRINITY_DN21285_c0_g1_i16	31.016	39.322	3.309	0.331	1.576	2.762
TRINITY_Contig10086	5.189	7.348	35.263	32.113	0	0.111
TRINITY_1555_length_3959_cov_33.626221_g1005_i0	10.653	9.134	6.862	5.685	2.071	1.883
TRINITY_DN14015_c3_g1_i5	4.417	9.803	19.641	22.472	73.893	79.177
TRINITY_Contig1106	0	0	0	0	83.935	69.207
TRINITY_53238_length_754_cov_25.849635_g37188_i0	36.575	35.64	7.445	8.502	4.064	5.247
TRINITY_72660_length_500_cov_37.150812_g55688_i0	0	0	0	0	155.329	110.845
TRINITY_Contig5765	0.197	0.313	0	0	229.908	201.907
TRINITY_Contig10184	0	0	0.18	0	8.105	6.405
TRINITY_Contig13050	91.66	98.344	20.468	17.998	18.676	23.336
TRINITY_56438_length_701_cov_16.892405_g40047_i0	0	0	0	0	19.025	8.054
TRINITY_65405_length_577_cov_33.877953_g48570_i0	0	0	0	0	87.469	84.39
TRINITY_DN19528_c4_g3_i1	8.595	2.701	29.971	55.061	47.022	31.746
TRINITY_Contig11635	0	0	0	0	42.057	37.951
TRINITY_Contig11023	0	0	0	0	7.441	8.499
TRINITY_DN11789_c0_g1_i5	14.848	12.292	6.671	7.777	1.745	2.005
TRINITY_DN16287_c0_g1_i2	13.827	8.449	0.901	2.226	0	0.769
TRINITY_Contig1533	98.77	86.789	71.108	54.398	17.82	21.331
TRINITY_Contig13980	3.826	4.631	7.063	8.989	97.387	81.416
TRINITY_BIN_3678_3	0	0	0	0	28.413	36.169
TRINITY_Contig3069	39.457	42.514	17.987	13.524	6.439	6.561
TRINITY_Contig896	0	0	0	0	10.481	9.012
TRINITY_5374_length_2792_cov_42.929857_g3410_i0	2.805	3.175	11.199	12.375	21.186	20.44
TRINITY_48509_length_838_cov_29.072822_g33139_i0	69.83	66.751	38.084	27.018	16.65	14.793
TRINITY_DN17176_c0_g1_i5	21.89	17.337	10.414	9.682	306.965	273.13
TRINITY_Contig15838	268.125	278.254	1572.72	1181.812	2146.142	2079.264
TRINITY_Contig3552	0	0	0	0	21.411	23.325
TRINITY_68668_length_541_cov_9.033898_g51763_i0	6.073	4.648	13.628	24.532	37.892	39.8
TRINITY_DN15968_c0_g1_i8	13.338	8.626	3.15	2.931	1.666	1.314
TRINITY_Contig2556	24.858	22.468	10.075	8.719	4.604	5.169
TRINITY_DN22344_c2_g2_i18	12.12	10.768	2.132	2.765	2.465	1.281
TRINITY_62221_length_617_cov_36.240876_g45500_i0	0	0	0	0	39.536	32.949
TRINITY_Contig11541	0	0	0	0	14.229	8.789

TRINITY_17167_length_1770_cov_25.322163_g10782_i0	0	0	0	0	17.539	17.043
TRINITY_Contig1691	0	0	0.308	0	13.407	15.027
TRINITY_Contig2470	19.068	21.977	55.243	67.374	115.714	107.314
TRINITY_Contig12316	0	1.473	3.585	0.59	22.391	14.915
TRINITY_30349_length_1264_cov_32.415900_g19459_i0	31.102	29.689	22.812	11.961	1.396	0.947
TRINITY_DN16290_c0_g1_i1	13.355	11.335	6.936	4.888	2.961	2.685
TRINITY_Contig7549	48.952	41.464	32.685	25.454	7.621	9.669
TRINITY_Contig16540	476.14	477.618	131.166	143.187	17.044	14.492
TRINITY_BIN_1005_1	25.158	21.376	14.667	9.206	3.546	4.779
TRINITY_DN15880_c0_g5_i2	178.696	162.09	96.497	88.592	37.464	32.348
TRINITY_DN20626_c0_g2_i1	326.453	290.436	162.208	134.24	50.771	41.226
TRINITY_Contig1270	1.33	2.201	15.802	10.501	27.873	23.425
TRINITY_DN13067_c2_g4_i2	55.179	57.507	234.324	245.376	464.736	386.871
TRINITY_Contig1788	9.675	10.912	7.625	4.111	1.103	0.379
TRINITY_Contig17960	69.066	62.569	4.613	7.011	3.141	3.765
TRINITY_54511_length_733_cov_28.352410_g38316_i0	26.625	35.014	4.2	3.272	4.267	1.938
TRINITY_Contig19955	0.918	0.356	5.547	3.593	9.805	8.711
TRINITY_Contig1431	0	0	0	0	28.672	25.664
TRINITY_30534_length_1258_cov_27.903280_g19581_i0	5.361	4.063	11.178	9.662	0.214	0.724
TRINITY_90607_length_355_cov_71.762238_g73536_i0	58.628	95.39	338.923	194.478	581.283	554.403
TRINITY_Contig2279	24.283	25.684	97.441	99.714	286.78	268.296
TRINITY_48941_length_830_cov_29.049934_g33499_i0	5.026	9.211	15.664	12.137	1.925	0.791
TRINITY_53276_length_753_cov_68.157895_g37223_i0	13.124	5.951	69.825	52.172	201.461	195.658
TRINITY_Contig18086	0	0	0	0	34.211	25.341
TRINITY_DN22249_c0_g1_i3	3.594	3.606	11.252	7.187	22.785	19.371
TRINITY_Contig7272	2.419	2.54	8.802	10.19	15.648	14.559
TRINITY_70345_length_523_cov_19.814978_g53401_i0	9.907	11.158	2.354	2.309	0.957	0.635
TRINITY_Contig11121	0	0	0	0	134.638	104.841
TRINITY_71551_length_511_cov_31.798643_g54592_i0	0.66	0	2.927	1.833	28.515	25.041
TRINITY_DN16475_c0_g3_i4	7.634	8.635	7.583	8.833	0.743	1.838
TRINITY_Contig11536	0	0	0	0	17.01	16.553
TRINITY_DN11093_c0_g1_i1	24.806	30.239	13.501	29.441	5.111	6.138
TRINITY_BIN_11361_2	12.197	8.931	4.751	5.416	2.983	0.512
TRINITY_48977_length_830_cov_13.180026_g33530_i0	7.291	7.644	0	0	0.383	0.267
TRINITY_Contig3948	0	0	0	0	6.462	7.641
TRINITY_67474_length_553_cov_62.969008_g50588_i0	6.51	5.943	33.195	30.549	105.582	124.29
TRINITY_Contig3774	6.33	4.36	5.345	4.142	30.991	23.203
TRINITY_Contig12192	0	0	0	0	18.248	21.487
TRINITY_DN13783_c2_g1_i15	7.025	7.001	30.151	23.994	41.63	41.125
TRINITY_Contig379	4.769	5.943	20.214	16.714	36.766	36.447
TRINITY_65041_length_582_cov_18.855750_g48215_i0	0	0	0	0	15.434	17.444
TRINITY_Contig9863	0	0	0	0	37.025	39.288
TRINITY_DN14279_c3_g2_i6	5.421	3.699	13.776	20.432	30.654	22.991
TRINITY_31408_length_1231_cov_37.751291_g20201_i0	9.53	8.787	29.727	25.527	0.214	0.301
TRINITY_DN21758_c2_g1_i6	1.716	2.794	1.591	18.868	0	0.858
TRINITY_DN15771_c1_g3_i4	0	0	0	0	319.1	286.252
TRINITY_Contig3430	0	0	0	0	13.723	8.276
TRINITY_Contig17806	237.735	235.046	89.615	70.573	40.571	35.4
TRINITY_49435_length_821_cov_24.875000_g33912_i0	7.934	9.371	2.736	3.945	0.394	0.267
TRINITY_DN10930_c0_g2_i1	0	0	0	0	132.048	92.866
TRINITY_BIN_5217_3	7.96	8.525	2.938	2.195	0.225	1.103
TRINITY_25907_length_1406_cov_32.055348_g9321_i1	10.336	10.531	4.666	4.07	2.713	2.005
TRINITY_Contig17694	0	0	0	0	7.666	6.951
TRINITY_BIN_10626_1	31.994	31.28	6.321	5.789	6.563	3.932
TRINITY_DN21575_c1_g5_i1	112.194	94.137	26.937	27.929	21.727	22.913
TRINITY_Contig4016	0	0	0	0	9.94	4.812
TRINITY_Contig1901	10.061	10.243	2.8	2.796	1.024	1.158
TRINITY_54629_length_731_cov_29.590634_g38422_i0	98.625	89.396	20.818	25.382	17.64	13.98
TRINITY_Contig1409	0	0	0	0	33.254	24.305
TRINITY_Contig11475	0	0	0	0	485.979	400.984
TRINITY_Contig1416	0	0	0	0	7.531	6.973
TRINITY_36905_length_1083_cov_27.933925_g24117_i0	6.579	8.533	2.641	6.731	1.283	1.415
TRINITY_Contig18512	37.793	82.776	6.268	82.006	4.199	14.392
TRINITY_57259_length_688_cov_24.528271_g40822_i0	16.332	16.245	4.359	3.977	1.599	0.724
TRINITY_Contig12165	0	0	0	0	18.834	21.465

TRINITY_Contig12581	0	0	0	0	35.765	36.514
TRINITY_66567_length_563_cov_32.196356_g49701_i0	0	0	0	0	428.555	390.035
TRINITY_56425_length_701_cov_24.118671_g40036_i0	9.058	10.463	3.245	4.173	2.06	0.345
TRINITY_8118_length_2412_cov_37.173282_g5128_i0	9.538	8.889	3.595	2.278	2.06	2.016
TRINITY_Contig1283	5.001	3.217	21.062	12.965	58.234	51.574
TRINITY_DN20811_c1_g1_i15	18.596	13.951	5.78	4.846	2.42	4.612
TRINITY_5825_length_2717_cov_37.339124_g3671_i0	3.877	3.674	17.615	20.152	16.92	22.601
TRINITY_Contig5459	132.652	138.192	89.487	76.134	27.603	25.297
TRINITY_Contig3887	0	0	0	0	9.929	8.031
TRINITY_BIN_27713_1	0	0	0	0	13.88	17.99
TRINITY_Contig1855	0	0	0	0	11.291	12.743
TRINITY_50188_length_808_cov_9.849797_g34554_i0	0	0	0	0	8.837	6.327
TRINITY_28084_length_1335_cov_11.505529_g17924_i0	0	0	0	0	10.244	7.497
TRINITY_Contig10348	1.947	1.431	10.054	9.413	20.027	19.883
TRINITY_82023_length_421_cov_17.267045_g64972_i0	0	0	0	0	27.018	34.442
TRINITY_Contig15094	2.222	1.871	9.799	6.307	14.409	14.871
TRINITY_73080_length_496_cov_25.309133_g56099_i0	0	0	0	0	62.838	67.904
TRINITY_44173_length_922_cov_13.471278_g29605_i0	0	0	0	0	9.771	10.304
TRINITY_53321_length_753_cov_7.992690_g37257_i0	0	0	0	0	7.689	7.731
TRINITY_DN15636_c2_g3_i2	7.746	4.14	18.071	9.714	35.697	40.112
TRINITY_Contig11862	0	0	0	0	15.445	11.518
TRINITY_DN12804_c0_g2_i3	12.875	10.658	2.004	1.47	1.261	0.869
TRINITY_Contig14073	13.201	8.77	5.812	4.173	2.229	2.863
TRINITY_BIN_18001_1	38.144	42.641	4.815	3.883	7.666	5.603
TRINITY_DN20553_c2_g1_i5	19.325	25.659	178.879	106.818	142.506	95.841
TRINITY_46036_length_885_cov_19.969363_g31105_i0	0.678	0.711	7.307	5.934	9.873	14.303
TRINITY_Contig17073	28.563	16.609	91.333	96.732	139.951	118.108
TRINITY_Contig16296	44.397	39.907	15.59	12.706	6.586	3.943
TRINITY_38273_length_1050_cov_34.260958_g25070_i0	0	0	0	0	32.77	26.678
TRINITY_Contig1529	0.497	0.584	0.732	0.238	277.065	225.967
TRINITY_DN16840_c0_g1_i8	23.665	23.128	21.508	18.081	5.449	4.567
TRINITY_Contig1854	0	0	0	0	519.436	452.703
TRINITY_DN18882_c2_g4_i3	66.236	62.01	15.441	20.038	11.516	16.152
TRINITY_BIN_858_4	367.625	322.089	37.606	24.097	11.482	13.4
TRINITY_DN18572_c1_g1_i1	8.363	6.849	0	0	0	0.423
TRINITY_Contig11777	10.885	12.284	46.844	42.075	63.131	58.736
TRINITY_49533_length_819_cov_28.770667_g33999_i0	12.583	20.063	31.54	52.441	0	3.709
TRINITY_Contig19489	7.48	7.678	23.682	27.111	39.615	38.029
TRINITY_Contig15784	5.189	4.427	16.629	11.588	137.767	103.092
TRINITY_49538_length_819_cov_24.449333_g34003_i0	3.028	2.954	15.696	10.169	11.573	19.215
TRINITY_71555_length_511_cov_28.631222_g54596_i0	0	0	0	0	35.247	32.493
TRINITY_33006_length_1188_cov_25.395889_g21116_i2	6.596	5.833	2.577	3.034	0.293	0
TRINITY_56140_length_706_cov_29.390895_g39779_i0	36.42	44.935	4.168	3.49	4.064	4.144
TRINITY_DN17253_c1_g4_i1	1.364	3.64	10.383	11.402	31.847	40.145
TRINITY_34320_length_1152_cov_32.090489_g22257_i0	0	0	0	0	30.879	34.03
TRINITY_Contig6593	2.41	3.124	13.31	14.788	22.323	17.266
TRINITY_Contig12001	20.646	26.76	77.641	57.339	503.135	441.575
TRINITY_BIN_6485_4	0	0	0	0	7.835	7.14
TRINITY_Contig9774	0	0	0	0	10.571	9.056
TRINITY_DN14493_c1_g1_i17	14.076	14.612	8.824	20.287	3.141	2.863
TRINITY_Contig11056	0	0	0	0	7.61	7.062
TRINITY_Contig17820	1.33	1.558	6.045	4.95	15.929	19.571
TRINITY_DN16874_c0_g2_i2	28.958	24.372	9.704	8.782	2.747	4.199
TRINITY_74245_length_485_cov_25.615385_g57252_i0	0.755	2.98	14.911	12.313	30.913	32.192
TRINITY_DN14818_c1_g1_i2	2.264	4.385	8.983	9.04	32.286	24.528
TRINITY_Contig3017	15.663	15.839	8.792	8.057	3.681	2.852
TRINITY_DN19481_c1_g1_i8	22.019	21.249	13.819	9.755	5.066	4.266
TRINITY_DN15636_c2_g3_i5	2.024	5.816	23.639	23.87	43.69	41.315
TRINITY_Contig6935	0	0	0	0	13.464	10.515
TRINITY_BIN_4819_4	9.195	7.856	4.147	2.982	0.754	2.361
TRINITY_Contig12973	36.686	41.71	1.474	1.408	1065.101	883.34
TRINITY_BIN_10205_2	15.422	14.459	3.797	3.966	4.559	1.816
TRINITY_Contig2954	15.371	10.743	7.71	6.783	2.758	2.607
TRINITY_DN18720_c2_g2_i6	13.064	14.696	6.49	6.265	1.7	3.063
TRINITY_Contig1459	0	0	0	0	17.19	14.648

TRINITY_23604_length_1488_cov_23.832276_g14647_i1	6.382	7.289	2.248	1.812	0.473	0.434
TRINITY_37641_length_1066_cov_8.959880_g24640_i0	0	0	0	0	8.409	6.171
TRINITY_17846_length_1738_cov_29.339125_g11206_i0	14.204	11.649	0.732	0.487	0.946	0.769
TRINITY_BIN_2591_2	3.74	4.14	26.248	13.959	243.293	220.309
TRINITY_50588_length_800_cov_27.119015_g34903_i0	7.205	7.695	8.824	5.406	0	0
TRINITY_BIN_5680_1	9.804	9.93	25.018	28.954	0.642	0
TRINITY_Contig19112	0	0	0	0	14.713	9.257
TRINITY_Contig12363	5.824	8.152	49.315	33.304	103.714	102.513
TRINITY_Contig882	18.459	12.368	6.607	8.067	3.535	2.818
TRINITY_60365_length_643_cov_32.642857_g43740_i0	0	0	0	0	50.309	30.8
TRINITY_Contig9280	5.035	4.741	26.895	28.623	39.13	39.744
TRINITY_Contig17308	10.636	13.054	30.883	30.766	74.659	59.148
TRINITY_43965_length_926_cov_21.295216_g29443_i0	27.98	21.96	2.27	3.086	3.884	3.342
TRINITY_Contig12632	1.561	2.514	20.299	11.764	74.231	64.529
TRINITY_Contig11585	0	0	0	0	23.584	17.945
TRINITY_Contig1654	0	0.694	0.286	0	113.215	90.015
TRINITY_Contig3541	0	0	0	0	25.655	16.965
TRINITY_Contig11296	9.976	8.085	2.927	4.07	1.216	0.835
TRINITY_22890_length_1515_cov_27.502075_g14498_i0	17.49	18.81	0.53	0.735	0	0
TRINITY_56088_length_707_cov_11.719436_g39727_i0	0	0	0	0	10.627	8.967
TRINITY_Contig11045	0	0	0	0	249.507	204.781
TRINITY_17043_length_1776_cov_18.461629_g10702_i0	8.252	8.135	0	0	0	0.089
TRINITY_Contig1670	0	0	0	0	271.651	226.847
TRINITY_DN11735_c0_g1_i4	11.434	7.839	2.842	4.318	1.036	2.517
TRINITY_45183_length_901_cov_28.179087_g23111_i1	4.64	4.207	12.493	11.557	62.456	54.013
TRINITY_22892_length_1515_cov_24.430844_g14500_i0	74.076	81.447	266.691	213.708	484.268	451.834
TRINITY_Contig12237	0	0	0	0	51.12	53.902
TRINITY_59057_length_661_cov_26.596284_g42507_i0	12.343	17.812	0	0.352	1.148	0.78
TRINITY_Contig10793	4.632	3.378	9.598	9.393	21.31	25.453
TRINITY_48328_length_842_cov_8.978008_g32983_i0	0	0	0	0	7.137	6.695
TRINITY_17744_length_1744_cov_20.575522_g11073_i1	10.971	11.479	0	0	0.417	0.1
TRINITY_60032_length_648_cov_15.336788_g43424_i0	9.324	6.366	0	0	1.193	0.401
TRINITY_Contig707	8.252	8.034	36.768	32.807	77.934	72.304
TRINITY_BIN_5972_3	36.592	26.904	10.117	8.802	4.784	5.826
TRINITY_DN18844_c0_g1_i1	31.943	23.644	5.281	4.639	1.61	2.952
TRINITY_41032_length_987_cov_19.518519_g27176_i0	0	0	0	0	13.227	14.581
TRINITY_16010_length_1831_cov_24.440976_g10042_i0	8.535	8.093	6.681	5.602	0.653	0.813
TRINITY_Contig3750	0	0	0	0	8.556	12.966
TRINITY_Contig859	9.341	8.787	0	0.321	0.326	0
TRINITY_63030_length_607_cov_31.377323_g46280_i0	0	0	0	0	45.671	41.415
TRINITY_Contig19168	2.342	1.82	5.239	8.574	21.839	24.573
TRINITY_BIN_3016_28	24.832	23.179	13.352	12.996	4.582	5.514
TRINITY_21936_length_1555_cov_54.466353_g3031_i1	9.427	12.004	27.754	29.12	50.816	59.16
TRINITY_DN21802_c2_g3_i1	12.926	9.769	0	2.63	0	1.192
TRINITY_DN13833_c1_g1_i11	2.813	4.013	16.014	13.038	32.5	22.222
TRINITY_8109_length_2413_cov_37.381399_g3974_i1	40.718	40.482	4.974	6.493	5.899	6.851
TRINITY_30822_length_1250_cov_17.805250_g19790_i0	0	0	0	0	15.603	11.228
TRINITY_BIN_11487_1	33.375	32.372	8.208	13.442	4.503	9.68
TRINITY_BIN_22493_1	0	0	0.35	1.036	13.272	26.689
TRINITY_Contig2475	2.342	2.76	12.185	13.7	32.162	26.567
TRINITY_13077_length_2001_cov_25.057453_g8182_i0	3.766	5.223	8.346	8.585	0.462	0.078
TRINITY_32293_length_1208_cov_27.000878_g20802_i0	0	0	0	0	14.072	13.456
TRINITY_50808_length_796_cov_38.947730_g35087_i0	0	0.212	0.52	0.518	150.218	109.686
TRINITY_Contig13864	29.292	33.134	18.633	11.878	7.396	6.906
TRINITY_Contig3792	0	0	0	0	7.171	8.276
TRINITY_BIN_4975_1	0	0	0	0	32.961	19.972
TRINITY_43407_length_937_cov_31.114055_g29007_i0	0	0	0	0	26.061	20.763
TRINITY_Contig14797	18.562	17.075	12.122	9.838	2.375	2.84
TRINITY_Contig18609	4.152	3.903	18.124	13.794	37.87	32.17
TRINITY_Contig7711	12.369	11.581	11.443	8.295	1.531	1.404
TRINITY_DN11265_c0_g1_i2	23.314	17.888	3.118	7.415	5.719	1.248
TRINITY_44220_length_921_cov_10.535211_g29643_i0	0	0	0	0	8.803	8.967
TRINITY_Contig12299	0	0	0	0	35.314	43.676
TRINITY_BIN_9616_2	10.516	12.216	3.595	4.391	2.15	2.361
TRINITY_63561_length_600_cov_29.361582_g46790_i0	6.57	13.731	4.794	1.429	44.016	38.641

TRINITY_55155_length_723_cov_7.845566_g38889_i0	0.952	1.481	5.228	3.045	19.937	20.93
TRINITY_DN21819_c0_g1_i2	11.237	8.762	24.032	13.576	61.24	54.581
TRINITY_DN28970_c0_g1_i1	16.666	15.026	12.525	8.978	0.36	0
TRINITY_BIN_29282_1	0	0	0	0	8.724	7.04
TRINITY_Contig12385	2.273	0	7.89	7.684	23.595	17.199
TRINITY_DN16356_c0_g1_i1	12.549	12.766	8.283	8.274	3.591	2.517
TRINITY_DN12956_c0_g3_i1	0	0	0	0	34.279	25.085
TRINITY_DN20437_c0_g1_i1	52.134	32.694	12.451	12.499	0	5.336
TRINITY_BIN_7_6	396.257	385.344	4001.445	2291.238	2753.137	2834.639
TRINITY_Contig19486	91.883	88.423	4.359	3.997	344.711	313.676
TRINITY_DN17248_c0_g1_i14	73.415	79.475	40.141	24.936	11.212	11.473
TRINITY_19469_length_1662_cov_39.137476_g12241_i0	1.347	1.642	12.97	8.502	33.772	33.206
TRINITY_DN10991_c2_g1_i2	0	0	0	0	195.968	182.213
TRINITY_BIN_15300_1	15.328	10.802	4.804	4.546	1.407	1.96
TRINITY_DN14903_c3_g1_i2	1892.206	1680.048	541.262	455.864	248.269	203.322
TRINITY_44624_length_912_cov_35.240807_g29046_i1	11.357	6.451	4.412	7.87	1.328	2.361
TRINITY_71284_length_514_cov_20.253933_g54330_i0	0	0	0	0	23.066	28.015
TRINITY_50285_length_806_cov_7.518318_g34638_i0	0	0	0	0	9.276	8.287
TRINITY_Contig1543	0	0	0	0	9.738	7.742
TRINITY_BIN_18968_1	0	0	0	0	515.394	437.944
TRINITY_Contig11454	0	0	0	0	7.025	9.201
TRINITY_37393_length_1072_cov_25.673978_g24475_i0	13.115	17.126	3.86	13.203	1.306	4.121
TRINITY_DN12626_c1_g1_i12	1.707	3.031	5.79	8.098	39.344	39.922
TRINITY_2433_length_3521_cov_24.759560_g1555_i0	9.298	9.143	0	0	1.902	1.626
TRINITY_Contig12674	27.946	29.782	66.442	64.764	168.477	153.318
TRINITY_DN16992_c4_g1_i4	2.222	2.946	19.28	17.905	18.496	19.181
TRINITY_Contig5140	46.919	38.4	17.149	15.678	4.807	6.193
TRINITY_Contig8306	8.029	8.449	0.106	0.093	0.45	0.802
TRINITY_78191_length_451_cov_21.507853_g61163_i0	0	0.728	5.25	6.773	39.344	26.678
TRINITY_Contig2273	39.388	41.109	13.893	14.529	9.377	5.536
TRINITY_Contig14100	0	0	0	0	22.166	20.964
TRINITY_7910_length_2436_cov_27.240811_g5005_i0	6.15	7.094	0.583	0.849	0	0
TRINITY_Contig11908	0	0	0	0	11.64	8.967
TRINITY_Contig2220	0	0	0	0	92.288	97.244
TRINITY_Contig10927	0	0	0	0	44.275	43.71
TRINITY_Contig15773	60.832	58.006	25.124	20.867	11.618	13.133
TRINITY_DN19196_c0_g3_i5	8.749	7.788	3.563	1.916	0.946	0.78
TRINITY_Contig5388	0	0	0	0	76.325	51.563
TRINITY_Contig12769	35.314	64.16	6.416	187.726	2.251	8.198
TRINITY_43076_length_944_cov_13.602286_g28760_i0	6.588	5.613	0	1.999	0.315	0.212
TRINITY_36456_length_1095_cov_17.918129_g23786_i0	1.33	1.033	4.221	4.691	13.407	13.623
TRINITY_Contig8531	0	0	0	0	15.209	20.418
TRINITY_47440_length_858_cov_35.711027_g32255_i0	0	0	0	0	36.114	28.561
TRINITY_BIN_14504_2	100.263	99.081	78.108	74.602	19.081	18.112
TRINITY_DN21725_c0_g1_i15	1.021	1.024	26.407	11.153	23.393	8.421
TRINITY_DN11491_c0_g2_i1	5.919	5.283	10.425	8.844	29.956	32.047
TRINITY_DN19858_c2_g2_i5	9.324	8.313	42.729	32.486	53.844	52.008
TRINITY_29224_length_1300_cov_17.233144_g18694_i0	6.579	9.515	54.554	46.776	54.834	52.198
TRINITY_58533_length_669_cov_16.348333_g42002_i0	0	0	0	0	16.267	12.955
TRINITY_DN11514_c0_g1_i1	11.648	10.328	5.345	3.303	3.141	2.061
TRINITY_Contig6250	16.177	13.477	6.978	5.53	2.781	2.128
TRINITY_28808_length_1313_cov_20.019293_g18414_i0	1.878	0	5.886	2.661	13.835	11.184
TRINITY_51836_length_778_cov_26.496474_g24322_i1	25.415	25.143	3.298	1.274	5.133	4.277
TRINITY_Contig19757	2.119	4.224	6.257	6.741	12.428	20.396
TRINITY_21218_length_1585_cov_36.085092_g13373_i0	11.271	9.033	7.212	4.194	2.195	1.994
TRINITY_22139_length_1546_cov_38.231550_g13999_i0	2.282	1.862	6.469	7.135	41.438	39.833
TRINITY_60871_length_636_cov_25.777778_g44210_i0	16.923	20.14	6.607	4.215	1.857	0.835
TRINITY_Contig3756	0	0	0	0	7.993	8.209
TRINITY_Contig10009	0	0	0	0	15.175	10.794
TRINITY_BIN_5696_1	12.163	14.544	7.71	19.427	1.869	3.241
TRINITY_BIN_4977_1	14.805	14.518	6.766	8.699	3.298	3.587
TRINITY_45075_length_903_cov_25.934053_g30337_i0	32.123	32.702	20.892	17.066	1.013	0.234
TRINITY_DN21719_c0_g6_i1	17.85	17.354	0.997	0.642	0.754	1.158
TRINITY_Contig7075	19.128	11.437	3.086	8.253	1.959	1.337
TRINITY_39095_length_1032_cov_14.651090_g25702_i0	0	0	0	0	9.265	5.792

TRINITY_DN16512_c0_g2_i1	3.448	4.063	27.69	23.942	19.52	24.428
TRINITY_34510_length_1147_cov_16.309833_g22399_i0	0	0	0	0	12.563	11.796
TRINITY_DN18383_c0_g1_i16	22.387	21.503	13.872	8.461	3.67	3.899
TRINITY_Contig3963	0	0	0	0	10.458	6.338
TRINITY_31800_length_1221_cov_31.754340_g20474_i0	1.321	1.219	6.851	10.314	13.498	13.668
TRINITY_13148_length_1996_cov_25.654904_g8229_i0	3.929	4.978	7.509	9.165	0.349	0.891
TRINITY_Contig12360	2.865	3.217	13.564	12.675	37.768	43.476
TRINITY_Contig8378	7.6	12.453	46.525	49.282	71.889	72.571
TRINITY_49677_length_817_cov_7.887701_g34126_i0	0	0	0	0	8.285	6.483
TRINITY_63903_length_596_cov_31.576850_g47117_i0	0	0	0	0	133.928	110.845
TRINITY_DN12250_c0_g1_i1	0	0	0	0	12.586	13.423
TRINITY_Contig14549	7.214	8.83	32.792	25.63	59.169	55.539
TRINITY_DN14783_c1_g1_i5	6.09	5.299	8.993	6.275	39.918	39.722
TRINITY_BIN_3254_3	10.748	12.707	8.452	8.295	0.338	0.345
TRINITY_24064_length_1472_cov_12.982181_g15270_i0	0.223	0.432	3.691	3.49	9.163	10.805
TRINITY_Contig12126	18.107	11.005	1.241	1.636	2.657	0.446
TRINITY_Contig7926	2.35	1.837	13.013	10.604	42.271	30.878
TRINITY_63374_length_603_cov_13.058052_g46610_i0	0	0	0	0	12.439	13.523
TRINITY_49663_length_817_cov_19.518717_g34113_i0	22.945	21.697	5.016	13.152	1.182	2.428
TRINITY_41461_length_976_cov_30.585447_g27500_i0	13.913	14.01	5.631	4.753	49.915	60.496
TRINITY_DN13326_c0_g3_i6	49.012	43.267	25.06	21.716	11.584	8.51
TRINITY_Contig10944	9.727	11.564	1.559	1.077	0.484	1.437
TRINITY_70530_length_521_cov_35.092920_g53583_i0	0	0	0	0	116.266	89.703
TRINITY_26973_length_1371_cov_29.294931_g17176_i0	20.08	16.33	13.776	12.323	1.497	0.29
TRINITY_BIN_11479_1	29.798	27.369	6.84	8.119	3.276	4.89
TRINITY_DN21959_c1_g2_i1	0.978	3.056	9.163	15.616	25.487	21.454
TRINITY_DN14658_c0_g1_i2	19.677	21.333	2.418	1.512	0.507	0
TRINITY_BIN_28957_1	0	0	0	0	10.514	7.976
TRINITY_55595_length_715_cov_25.557276_g39282_i0	40.126	44.664	26.025	23.248	10.908	5.068
TRINITY_69006_length_537_cov_26.692308_g52096_i0	0	0	0	0	59.315	43.843
TRINITY_Contig8447	2.779	2.455	8.049	6.514	23.595	23.793
TRINITY_DN17253_c1_g1_i11	8.114	17.304	47.596	67.985	205.593	184.953
TRINITY_BIN_20507_2	36.06	33.507	12.917	11.08	4.357	6.995
TRINITY_78586_length_448_cov_23.781003_g61557_i0	0.952	2.988	9.81	13.856	27.817	31.156
TRINITY_Contig6427	14.119	8.212	2.577	4.577	0.574	2.172
TRINITY_Contig1936	34.087	30.713	0	0	2.184	3.932
TRINITY_Contig1597	0	0	0	0	56.343	41.404
TRINITY_DN14942_c2_g1_i22	0	4.14	8.855	19.23	50.872	41.716
TRINITY_63059_length_607_cov_12.252788_g46308_i0	0	1.041	4.263	3.78	11.584	11.963
TRINITY_19212_length_1674_cov_27.246729_g12093_i0	0	0	0	0	24.361	17.422
TRINITY_52186_length_772_cov_35.772404_g36280_i0	17.85	13.917	38.518	37.622	88.438	84.479
TRINITY_21347_length_1580_cov_33.256784_g13457_i0	5.936	7.035	8.527	7.549	0.934	1.292
TRINITY_Contig19085	0.815	0.322	7.636	3.179	14.972	14.614
TRINITY_55742_length_713_cov_11.625776_g39416_i0	0	0	0	0	9.467	10.025
TRINITY_34942_length_1135_cov_27.757036_g22725_i0	0	0	0	0	25.25	24.138
TRINITY_DN20626_c0_g3_i6	18.322	19.454	7.318	11.184	2.916	4.266
TRINITY_Contig10605	0	0	0	0	115.444	99.918
TRINITY_Contig2463	3.577	0	49.718	36.172	129.245	153.786
TRINITY_DN14127_c1_g2_i2	10.079	10.006	5.557	3.78	1.689	2.384
TRINITY_Contig10545	43.317	43.166	11.92	7.694	7.835	6.416
TRINITY_Contig8268	16.949	17.515	8.484	6.348	5.021	2.874
TRINITY_Contig11502	1.355	1.236	14.784	10.739	54.621	44.3
TRINITY_DN19725_c1_g1_i14	5.695	6.654	18.018	11.919	35.145	34.565
TRINITY_44830_length_908_cov_27.209774_g30129_i0	32.063	30.518	14.444	11.112	7.002	4.522
TRINITY_Contig16192	0	0	0	0	1001.576	827.644
TRINITY_Contig8054	25.089	24.762	104.154	99.093	327.521	318.533
TRINITY_73485_length_492_cov_37.903073_g56500_i0	33.332	27.284	15.113	17.418	5.64	6.717
TRINITY_Contig19504	8.157	4.021	7.869	10.117	31.352	35.478
TRINITY_Contig1536	0	0	0	0	9.186	9.446
TRINITY_47022_length_867_cov_21.624060_g31913_i0	51.851	44.046	7.084	2.496	2.51	2.952
TRINITY_Contig11715	0	0	0	0	123.313	116.782
TRINITY_DN12795_c0_g2_i3	2.548	3.86	5.451	6.42	42.237	55.116
TRINITY_Contig5841	11.288	9.329	2.927	3.303	1.621	1.404
TRINITY_DN19905_c0_g3_i2	12.463	10.624	6.957	6.524	1.711	2.918
TRINITY_Contig633	5.773	5.393	21.02	25.019	31.588	33.161

TRINITY_Contig960	66.536	56.135	33.364	24.025	8.252	5.402
TRINITY_Contig14514	0.232	0	0	0	455.134	388.163
TRINITY_60938_length_635_cov_29.932862_g44277_i0	0	0	0	0	32.939	28.505
TRINITY_Contig12547	1.492	1.397	9.863	4.308	19.216	21.833
TRINITY_51777_length_779_cov_29.663380_g35923_i0	4.743	4.783	18.973	7.787	2.139	0.29
TRINITY_DN16711_c1_g1_i11	8.114	7.848	27.086	22.793	41.934	39.577
TRINITY_Contig6503	0.343	0.737	6.098	5.323	20.218	19.805
TRINITY_Contig7651	4.949	7.153	36.111	28.188	89.158	71.379
TRINITY_DN15174_c1_g1_i1	0	0	0	0	40.74	34.798
TRINITY_Contig325	42.116	40.66	11.252	11.971	8.646	6.26
TRINITY_65261_length_579_cov_20.052941_g48427_i0	16.64	21.816	7.509	2.309	0.754	2.027
TRINITY_Contig19759	2.728	3.31	23.523	17.76	26.466	25.353
TRINITY_Contig2183	1.707	2.015	24.148	13.297	20.016	32.426
TRINITY_Contig2510	0	0	0	0	38.793	27.781
TRINITY_Contig3467	4.117	5.02	12.26	12.189	0.27	1.125
TRINITY_72987_length_497_cov_18.705607_g56008_i0	0	0	0	0	19.734	18.157
TRINITY_Contig3952	0	0	0	0	6.968	8.9
TRINITY_26921_length_1373_cov_23.520706_g17146_i0	252.257	260.773	113.18	97.301	26.117	24.372
TRINITY_Contig3551	4.958	2.794	9.364	9.144	0.777	0.356
TRINITY_Contig17572	20.269	22.019	128.409	106.932	322.556	327.645
TRINITY_23883_length_1478_cov_26.500355_g15145_i0	10.027	9.532	3.394	1.968	1.013	0.59
TRINITY_97643_length_297_cov_35.899123_g80571_i0	0	0	0	0	373.203	133.223
TRINITY_Contig19567	1.141	12.631	29.917	16.403	81.21	86.818
TRINITY_Contig1754	0.815	0.957	5.547	4.857	15.209	14.169
TRINITY_DN17699_c1_g2_i2	10.653	5.037	3.627	4.37	1.137	1.359
TRINITY_Contig15185	3.585	4.698	24.445	21.032	35.652	45.381
TRINITY_Contig11062	0	0	0	0	367.36	302.905
TRINITY_Contig7165	6.107	5.765	20.468	18.785	43.802	50.326
TRINITY_DN10099_c0_g1_i3	0	0	0	0	257.804	210.25
TRINITY_46552_length_875_cov_14.988834_g31517_i0	1.081	1.642	9.937	10.915	10.604	12.799
TRINITY_71889_length_508_cov_19.735763_g54926_i0	0	0	0	0	21.715	23.359
TRINITY_DN15401_c0_g3_i5	0	1.456	0	1.16	22.064	17.266
TRINITY_Contig3104	24.849	18.463	5.801	13.897	3.659	3.208
TRINITY_43352_length_938_cov_34.561565_g28965_i0	0	0	0	0	14.184	10.025
TRINITY_21897_length_1557_cov_37.351478_g13826_i0	13.029	11.767	7.074	4.598	1.902	2.529
TRINITY_DN17520_c1_g1_i5	35.708	26.108	10.966	10.293	4.582	4.4
TRINITY_35099_length_1131_cov_25.828625_g18363_i2	18.562	13.401	13.575	6.876	2.938	3.865
TRINITY_44022_length_925_cov_15.968458_g29486_i0	1.913	2.811	11.136	9.455	23.19	20.964
TRINITY_21825_length_1561_cov_14.347855_g13774_i0	5.198	6.933	0	0.104	0	0.111
TRINITY_28282_length_1330_cov_8.290246_g18060_i0	0	0	0	0	6.991	6.583
TRINITY_Contig6407	14.016	9.075	6.65	2.869	3.152	0.958
TRINITY_BIN_18342_1	10.559	9.896	1.326	1.854	1.238	1.426
TRINITY_DN13209_c0_g3_i1	46.01	42.328	2.323	2.63	3.681	3.219
TRINITY_Contig1948	0	0	0	0	11.28	15.684
TRINITY_Contig11865	11.502	10.641	6.183	3.956	1.61	2.685
TRINITY_21779_length_1563_cov_10.491968_g13740_i0	0	0	0	0.104	6.631	7.006
TRINITY_DN21347_c0_g3_i4	14.522	15.094	5.345	4.981	1.689	2.373
TRINITY_DN11765_c4_g9_i4	2.033	0	2.407	2.33	9.141	10.593
TRINITY_DN17828_c1_g2_i3	28.117	28.106	11.867	12.085	5.786	6.372
TRINITY_Contig444	0	0	0	0	9.839	10.671
TRINITY_DN18943_c1_g2_i3	8.68	10.074	6.13	6.037	1.508	2.094
TRINITY_19072_length_1680_cov_24.467412_g12002_i0	0	0	0	0	19.622	14.425
TRINITY_24941_length_1440_cov_27.471189_g15849_i0	0	0	0	0	15.175	10.226
TRINITY_75245_length_476_cov_27.039312_g58246_i0	4.761	1.752	16.502	8.761	24.755	22.835
TRINITY_34763_length_1140_cov_25.963585_g22597_i0	11.563	13.291	7.678	10.221	2.15	3.208
TRINITY_65268_length_579_cov_15.229412_g48434_i0	3.422	1.524	13.267	8.771	28.053	21.075
TRINITY_DN14093_c6_g1_i1	44.432	50.345	108.439	157.333	456.158	265.377
TRINITY_58645_length_667_cov_26.615385_g42113_i0	3.68	2.294	14.922	10.873	12.417	13.033
TRINITY_10300_length_2207_cov_78.101497_g6166_i4	174.407	173.425	25.081	28.892	14.049	16.486
TRINITY_55314_length_720_cov_26.265745_g39034_i0	4.152	10.472	29.737	22.368	26.939	30.053
TRINITY_Contig14050	0	0	0	0	9.974	7.318
TRINITY_Contig16956	0.935	0.728	6.936	6.275	16.683	15.929
TRINITY_Contig11776	0	0	0	0	11.347	7.463
TRINITY_Contig12026	388.546	378.546	89.127	97.684	88.167	81.371
TRINITY_DN19862_c0_g1_i3	9.435	8.813	6.013	3.914	0.991	0.991

TRINITY_65059_length_582_cov_9.343080_g48232_i0	0.48	0.753	8.357	6.855	12.687	13.556
TRINITY_Contig5370	0	0	0	0	10.334	10.081
TRINITY_Contig1250	14.599	16.762	12.504	10.532	1.047	0.178
TRINITY_Contig16117	3.903	3.513	15.431	18.309	27.558	23.682
TRINITY_Contig12488	1.209	1.575	14.667	10.376	33.693	26.032
TRINITY_Contig6292	0	0	0	0	14.409	9.847
TRINITY_Contig1350	3.5	2.421	4.741	5.654	408.123	347.027
TRINITY_Contig17214	0	0	0	0	9.355	11.106
TRINITY_13668_length_1962_cov_12.956683_g8555_i0	0	0	0	0.072	12.068	6.227
TRINITY_Contig4042	10.773	9.803	2.238	1.885	2.51	1.392
TRINITY_Contig12458	2.573	3.065	17.35	11.319	35.922	34.954
TRINITY_Contig17304	0	0	0	0	51.3	40.669
TRINITY_54238_length_737_cov_40.712575_g38072_i0	56.577	33.744	7.063	4.474	4.57	2.941
TRINITY_59961_length_649_cov_10.224138_g43356_i0	4.263	3.327	13.129	19.189	30.946	35.545
TRINITY_Contig1200	0	0	0	0	9.355	4.589
TRINITY_Contig19652	1.381	5.096	16.979	8.75	21.67	17.956
TRINITY_BIN_3181_1	3.723	3.005	9.534	7.663	36.44	28.939
TRINITY_65816_length_572_cov_27.196819_g48968_i0	1.004	3.928	7.71	10.418	145.017	119.868
TRINITY_Contig4220	0	0	0	0	9.051	7.964
TRINITY_35939_length_1108_cov_41.467757_g23432_i0	215.314	195.021	102.309	98.316	27.625	36.425
TRINITY_DN12369_c0_g1_i1	16.177	15.627	7.148	7.373	4.312	2.696
TRINITY_Contig7457	3.139	6.154	5.928	6.493	25.678	37.071
TRINITY_Contig556	3.157	3.903	24.763	23.6	50.084	44.59
TRINITY_DN13966_c2_g2_i3	12.214	13.994	5.79	2.993	0	0
TRINITY_19610_length_1656_cov_26.684310_g12334_i0	4.675	3.403	8.548	7.808	0.732	0.813
TRINITY_39457_length_1023_cov_26.554507_g25971_i0	0	0	0	0	16.74	19.805
TRINITY_Contig10684	0	0	0	0	13.903	12.821
TRINITY_DN14345_c5_g1_i2	65.49	80.981	3.107	11.598	3.287	9.134
TRINITY_Contig10704	3.079	4.14	16.47	14.767	15.141	14.893
TRINITY_66387_length_565_cov_28.356855_g49524_i0	58.379	61.418	21.274	20.411	7.182	7.497
TRINITY_Contig7580	1.107	1.371	1.845	2.382	19.318	16.074
TRINITY_Contig12423	0.875	2.751	6.512	10.407	30.609	18.892
TRINITY_BIN_7739_1	11.014	10.277	6.862	4.173	1.385	2.462
TRINITY_BIN_10690_3	2.796	3.141	7.943	8.864	15.242	16.82
TRINITY_DN19320_c1_g1_i1	9.187	8.051	5.737	4.184	1.88	1.392
TRINITY_Contig18816	8.835	8.161	4.56	6.576	1.734	1.337
TRINITY_DN16975_c0_g2_i6	17.773	17.202	1.495	1.098	1.069	0.457
TRINITY_Contig1914	4.477	3.556	26.566	15.958	31.791	34.899
TRINITY_36799_length_1086_cov_26.382498_g24034_i0	43.394	38.671	3.553	6.949	5.077	4.6
TRINITY_Contig10968	0	0	0	0	12.676	7.552
TRINITY_85417_length_394_cov_26.606154_g68353_i0	0	0	0	0	65.765	43.676
TRINITY_DN18813_c1_g1_i3	1.818	2.37	8.665	9.776	13.554	14.024
TRINITY_Contig2443	0	0.305	3.797	3.003	15.76	9.88
TRINITY_DN13113_c0_g1_i1	0	0	0	0	5.696	7.808
TRINITY_DN12707_c0_g4_i2	10.576	8.931	7.159	7.435	1.801	2.451
TRINITY_DN21484_c2_g2_i8	46.842	52.3	27.637	16.755	10.177	10.036
TRINITY_BIN_26046_1	2.745	0.88	10.181	20.68	28.841	22.991
TRINITY_Contig1568	2.856	5.266	41.223	38.875	76.741	73.952
TRINITY_Contig11922	39.654	33.278	25.166	25.558	6.687	8.02
TRINITY_Contig2133	2.153	2.667	6.109	8.005	26.77	16.998
TRINITY_Contig19744	0.24	0.186	0	0	478.2	390.67
TRINITY_29826_length_1281_cov_21.981023_g19120_i0	30.716	31.822	5.653	3.407	9.186	5.792
TRINITY_Contig18352	105.289	107.563	252.119	258.02	910.481	781.462
TRINITY_Contig8255	2.668	2.785	21.645	12.199	49.285	44.267
TRINITY_Contig16942	6.296	6.645	39.165	26.448	180.815	154.867
TRINITY_Contig10416	0	0	0	0.114	11.584	9.802
TRINITY_Contig11948	32.457	31.052	3.351	2.982	2.657	2.161
TRINITY_Contig18227	29.198	26.988	11.602	8.005	3.253	3.676
TRINITY_Contig7290	26.608	25.981	10.807	10.977	5.1	4.968
TRINITY_Contig12207	2.307	3.92	23.12	18.464	46.02	42.039
TRINITY_70529_length_521_cov_35.712389_g53582_i0	10.628	12.241	57.746	48.35	0.968	0.646
TRINITY_23350_length_1497_cov_25.478291_g14798_i0	9.941	7.898	6.999	5.903	1.655	1.158
TRINITY_Contig5773	0.497	0.516	0.817	1.626	71.09	70.02
TRINITY_DN14058_c0_g1_i2	28.049	28.647	10.881	9.227	2.994	2.952
TRINITY_Contig17688	0	0	0	0	351.69	285.027

TRINITY_Contig3266	0	0	0	0	28.785	23.136
TRINITY_Contig11837	0	0	0	0	12.541	7.92
TRINITY_92471_length_342_cov_12.992674_g75400_i0	29.232	4.411	28.602	35.468	96.948	79.277
TRINITY_Contig16783	16.572	15.831	9.301	7.539	1.542	2.573
TRINITY_Contig1168	0	0	0	0	14.995	10.025
TRINITY_50726_length_798_cov_23.219479_g35018_i0	200.946	184.896	134.22	88.53	22.548	30.599
TRINITY_DN16567_c0_g3_i5	16.263	14.62	6.904	5.965	3.107	1.526
TRINITY_DN14998_c1_g5_i1	0	0	0	0	17.663	15.261
TRINITY_Contig18228	20.037	14.476	4.454	4.008	2.6	0.579
TRINITY_Contig3970	0	0	0	0	8.072	8.655
TRINITY_4308_length_2994_cov_35.929231_g2740_i0	7.6	6.933	23.013	22.202	56.714	46.628
TRINITY_Contig2379	5.412	4.563	22.08	15.047	69.401	65.587
TRINITY_Contig11814	225.169	221.569	696.777	598.854	1186.882	1164.334
TRINITY_DN15195_c4_g1_i12	3.731	2.946	45.168	26.697	104.513	72.326
TRINITY_53593_length_748_cov_27.405007_g37493_i0	70.868	104.448	6.384	8.326	13.272	9.067
TRINITY_53585_length_748_cov_32.690722_g37487_i0	23.134	19.598	101.705	71.288	126.307	134.159
TRINITY_39895_length_1013_cov_22.093220_g26304_i0	0	0	0	0	39.896	36.302
TRINITY_40180_length_1007_cov_15.253731_g26533_i0	2.445	2.15	9.683	10.977	13.88	12.665
TRINITY_75892_length_470_cov_29.094763_g58887_i0	4.117	0.643	3.595	5.312	396.866	303.373
TRINITY_71255_length_514_cov_46.017978_g54301_i0	0	0	0	0	528.565	425.591
TRINITY_Contig7876	0	0	0	0	11.212	11.262
TRINITY_Contig6894	0	0	0	0	27.434	20.184
TRINITY_BIN_9436_1	11.185	7.924	25.156	28.281	59.011	53.713
TRINITY_DN22355_c0_g5_i2	1083.507	1120.281	366.985	412.557	136.191	130.305
TRINITY_DN18932_c2_g1_i5	0.703	0.55	11.984	8.378	73.094	62.836
TRINITY_BIN_6542_1	23.254	20.8	8.537	7.694	4.075	4.879

Supplementary table 1. *In silico* expression matrix for DEGs in Root (control vs salt, control vs drought) and Leaves (control vs drought, control vs salt) and the UniProt Ids for the Unigenes.

Unigene ID	CONTROL1	CONTROL2	PEG_TREATED _ROOT1	PEG_TREATED _ROOT2	SALT_TREATED _ROOT1	SALT_TREATED _ROOT2
TRINITY_DN20548_c0_g1_i7	12.017	11.708	2.662	2.641	0	0
TRINITY_DN14225_c4_g1_i6	17.138	6.866	100.665	91.502	210.917	257.892
TRINITY_Contig6231	4.881	1.702	22.823	21.892	38.545	25.263
TRINITY_Contig7879	1.209	3.318	33.884	17.511	110.468	115.122
TRINITY_BIN_8418_1	9.984	8.449	0	0.394	1.914	1.303
TRINITY_30573_length_1257_cov_24.303030_g19609_i0	12.986	8.821	2.768	2.278	1.914	0.947
TRINITY_41707_length_971_cov_17.417960_g27694_i0	4.323	6.417	24.095	17.667	21.445	27.358
TRINITY_Contig12088	10.104	9.448	0.605	0.487	0	0.223
TRINITY_Contig12651	3.568	3.132	28.412	23.455	30.958	31.802
TRINITY_66951_length_559_cov_21.744898_g50075_i0	12.703	14.485	0	0	0	0
TRINITY_BIN_528_1	7.806	4.402	35.888	40.252	39.693	47.53
TRINITY_Contig5870	35.588	29.866	7.466	7.425	7.801	5.982
TRINITY_60583_length_640_cov_29.705779_g43944_i0	87.182	85.163	6.915	7.57	9.175	8.287
TRINITY_66665_length_562_cov_28.018256_g49797_i0	28.752	38.848	195.71	146.501	64.178	46.918
TRINITY_Contig11628	68.26	55.729	5.133	6.783	4.987	4.99
TRINITY_BIN_3601_2	18.699	18.768	4.56	2.713	6.788	4.901
TRINITY_35927_length_1109_cov_12.350000_g23421_i0	0	0	6.225	6.835	0	0
TRINITY_37696_length_1064_cov_33.411055_g23900_i1	33.735	26.971	2.535	2.019	2.375	2.551
TRINITY_74790_length_480_cov_31.072993_g57792_i0	20.157	27.987	107.76	101.35	15.704	12.754
TRINITY_BIN_23317_1	2.625	1.837	11.729	8.844	2.803	1.103
TRINITY_17490_length_1755_cov_25.852906_g10980_i0	10.936	8.753	1.867	1.781	0.563	0.958
TRINITY_BIN_3370_3	97.141	102.628	11.676	10.697	14.015	16.909
TRINITY_Contig3132	4.315	3.936	18.135	16.207	1.711	0
TRINITY_Contig3748	1.75	1.253	7.127	8.937	0	0
TRINITY_53589_length_748_cov_31.410898_g37489_i0	28.409	23.314	3.775	0.86	0.462	1.248
TRINITY_DN20225_c5_g1_i6	10.67	8.567	53.175	31.025	17.46	9.134
TRINITY_Contig19343	43.583	38.721	163.417	149.121	471.806	427.919
TRINITY_BIN_5888_4	10.405	8.652	1.951	1.678	1.396	2.417
TRINITY_41670_length_971_cov_30.574279_g27665_i0	6.914	7.382	30.66	31.429	0.608	0.624
TRINITY_BIN_5761_2	16.254	16.466	97.399	71.982	225.507	233.13
TRINITY_5956_length_2693_cov_37.538110_g3396_i1	29.893	25.955	3.51	4.246	9.816	8.688
TRINITY_Contig11412	0.858	0.584	7.827	8.574	11.415	12.376
TRINITY_63737_length_598_cov_28.483932_g46956_i0	6.853	6.781	33.364	27.225	7.036	7.597
TRINITY_BIN_268_1	48.034	57.236	11.486	13.079	0	5.703
TRINITY_66168_length_568_cov_20.166333_g49310_i0	29.104	40.457	1.464	4.329	0	5.291
TRINITY_32360_length_1206_cov_23.992084_g20854_i0	21.229	22.4	3.139	1.843	1.767	1.069
TRINITY_46179_length_882_cov_38.496925_g31223_i0	3.954	3.361	22.504	26.51	48.136	50.315
TRINITY_DN16359_c2_g1_i3	116.963	106.192	9.99	14.643	14.297	11.919
TRINITY_Contig16227	17.575	12.021	63.197	49.479	97.545	56.275
TRINITY_67666_length_551_cov_29.454357_g50777_i0	6.013	3.843	21.465	16.414	42.249	28.538
TRINITY_57520_length_684_cov_28.788618_g41058_i0	43.145	49.261	4.072	2.009	6.462	9.88
TRINITY_20316_length_1624_cov_24.274598_g12454_i1	40.623	47.001	3.16	4.204	5.696	4.4
TRINITY_57894_length_678_cov_19.193760_g41409_i0	9.984	12.521	0	0.342	2.184	1.493
TRINITY_Contig19341	6.768	4.419	35.994	26.158	92.727	88.355
TRINITY_Contig18163	1.235	2.252	11.581	9.931	25.982	27.625
TRINITY_31407_length_1231_cov_39.333046_g20200_i0	37.347	33.456	9.428	6.762	2.364	2.685
TRINITY_Contig11854	40.323	36.156	205.605	141.312	338.215	326.108
TRINITY_Contig17064	0.935	2.277	16.788	15.078	6.439	5.636
TRINITY_BIN_234_2	0	0	25.601	22.41	0	0
TRINITY_Contig13338	63.525	65.803	10.287	12.603	5.719	5.469
TRINITY_47954_length_849_cov_12.915385_g32688_i0	0.489	0	6.851	7.031	0	0
TRINITY_50521_length_801_cov_33.516393_g34846_i0	3.997	2.904	23.576	16.942	28.515	32.56
TRINITY_41027_length_987_cov_22.171024_g27171_i0	8.2	10.006	34.605	37.643	13.036	18.591
TRINITY_Contig12441	36.669	41.083	298.263	215.003	739.235	759.774
TRINITY_BIN_11269_1	18.407	27.2	0.583	1.419	0.462	0
TRINITY_DN11924_c0_g1_i6	2.762	5.477	34.032	29.586	96.048	95.751

TRINITY_Contig11151	8.749	10.633	0.361	0.176	1.114	0.769
TRINITY_Contig7649	11.374	14.62	100.761	67.663	140.491	150.767
TRINITY_Contig19164	1.75	1.913	11.846	18.06	36.485	29.73
TRINITY_67483_length_553_cov_29.580579_g50597_i0	1.081	4.665	15.558	14.249	1.677	1.682
TRINITY_17639_length_1748_cov_28.599762_g11079_i0	2.805	2.675	11.645	11.888	0.687	0.958
TRINITY_BIN_12695_3	43.934	41.744	8.516	4.432	3.76	2.852
TRINITY_Contig11732	1.741	0.533	6.809	5.209	12.169	14.158
TRINITY_21697_length_1566_cov_32.713427_g13361_i1	98.153	98.945	19.705	14.746	5.178	7.909
TRINITY_64539_length_588_cov_26.105973_g47730_i0	35.511	32.567	193.175	190.512	63.48	56.509
TRINITY_40589_length_998_cov_16.700753_g26843_i0	181.981	196.426	38.996	28.581	28.841	28.739
TRINITY_28673_length_1317_cov_23.326923_g18323_i0	22.019	25.067	5.536	5.364	5.471	6.661
TRINITY_38821_length_1038_cov_22.473684_g25495_i0	103.557	134.027	11.74	10.273	19.115	19.393
TRINITY_Contig1853	2.342	2.184	12.005	8.015	18.372	21.008
TRINITY_36254_length_1100_cov_23.384093_g16284_i1	1.321	0.897	13.766	11.567	30.631	23.437
TRINITY_Contig11110	1.947	2.201	9.163	7.218	11.584	15.416
TRINITY_Contig1849	291.053	289.285	45.772	44.498	70.595	74.543
TRINITY_BIN_22453_1	35.983	34.37	3.595	5.789	5.055	4.378
TRINITY_Contig1428	8.946	5.604	70.589	46.455	283.662	259.385
TRINITY_Contig11736	130.885	102.797	10.796	11.433	5.91	6.071
TRINITY_56012_length_708_cov_25.733959_g39660_i0	3.826	2.861	14.328	11.868	3.974	3.442
TRINITY_Contig14256	14.668	15.28	75.138	74.83	43.937	32.504
TRINITY_88074_length_374_cov_32.947541_g71004_i0	74.161	74.675	0	0	2.904	0
TRINITY_BIN_7560_1	15.259	19.547	87.992	101.091	40.087	28.048
TRINITY_BIN_18101_1	27.345	24.956	2.089	5.499	8.454	5.659
TRINITY_Contig12572	11.623	13.189	104.738	74.519	98.457	98.859
TRINITY_DN21450_c0_g9_i1	1.956	3.826	17.838	20.773	12.079	12.186
TRINITY_DN13949_c1_g3_i2	9.401	7.721	3.023	0.746	2.128	0.724
TRINITY_DN15239_c3_g3_i3	143.013	135.627	1.506	2.869	132.071	183.995
TRINITY_Contig19495	30.33	21.714	137.264	89.949	208.429	206.173
TRINITY_DN12995_c2_g1_i5	132.48	96.406	1.145	1.491	261.271	300.744
TRINITY_BIN_3370_10	27.568	25.304	5.631	4.857	4.998	7.129
TRINITY_Contig3882	7.823	8.601	0.689	1.015	0	0.746
TRINITY_DN6989_c0_g1_i2	14.239	18.683	4.072	3.407	8.432	6.761
TRINITY_11321_length_2125_cov_20.094358_g7084_i0	25.106	21.24	3.521	2.102	2.488	2.618
TRINITY_Contig20084	10.971	10.226	2.683	1.729	0.191	0
TRINITY_BIN_23690_1	6.219	2.125	15.367	19.241	1.193	2.027
TRINITY_40668_length_996_cov_18.574973_g2381_i2	4.28	1.778	10.404	13.856	18.271	18.179
TRINITY_Contig4770	31.154	34.853	207.291	183.128	580.619	541.649
TRINITY_Contig13563	5.069	5.782	27.69	29.244	35.483	35.445
TRINITY_DN19858_c2_g2_i15	0	0.99	7.137	8.564	36.834	40.145
TRINITY_DN16575_c2_g3_i1	6.63	2.083	71.193	73.276	2.06	3.42
TRINITY_Contig11289	40.572	33.287	145.42	162.78	126.926	130.695
TRINITY_Contig19342	8.26	10.286	67.248	40.77	146.525	128.957
TRINITY_DN18016_c0_g1_i5	5.695	5.164	21.921	20.67	30.091	32.426
TRINITY_Contig17547	12.532	6.265	0.626	0.311	0	0
TRINITY_92701_length_340_cov_43.162362_g75630_i0	8.226	15.357	71.087	86.935	157.107	147.637
TRINITY_Contig12499	86.908	87.187	695.409	454.642	1636.984	1548.655
TRINITY_Contig11560	2.256	2.362	10.128	11.909	15.355	17.978
TRINITY_88083_length_374_cov_25.714754_g71013_i0	14.47	8.618	77.058	55.61	11.618	22.211
TRINITY_Contig10552	0.129	0.703	10.266	9.31	0	0
TRINITY_57649_length_682_cov_23.876020_g25291_i1	78.905	86.603	14.9	16.269	14.477	17.956
TRINITY_Contig5843	38.976	35.784	6.045	9.993	4.053	10.927
TRINITY_Contig7587	22.636	29.486	110.878	106.062	40.121	26.544
TRINITY_30372_length_1263_cov_52.868509_g19474_i0	8.2	7.467	40.682	30.197	116.851	109.13
TRINITY_Contig12433	31.969	25.71	184.299	144.253	246.693	214.773
TRINITY_30609_length_1256_cov_27.197978_g19641_i0	20.886	23.331	5.398	4.577	3.141	3.331
TRINITY_Contig3537	4.743	5.774	30.777	30.218	4.886	4.367
TRINITY_DN14632_c0_g1_i11	69.169	66.387	9.651	7.798	13.711	11.685
TRINITY_DN16457_c1_g1_i16	63.045	66.7	8.113	15.409	2.803	1.793
TRINITY_BIN_24345_1	2.959	3.75	16.3	12.592	2.522	4.623
TRINITY_37796_length_1062_cov_24.088620_g23008_i1	14.247	13.088	1.188	1.201	0.259	0.267
TRINITY_BIN_1432_2	3.731	3.098	9.471	18.599	3.006	0
TRINITY_Contig14131	14.771	12.504	1.177	1.481	1.193	2.183
TRINITY_Contig12421	1.141	1.998	11.051	8.657	0.439	0.891
TRINITY_65408_length_577_cov_32.879921_g48572_i0	6.905	9.642	62.423	43.245	87.469	70.588

TRINITY_62788_length_610_cov_27.876155_g46046_i0	5.695	3.767	23.618	25.754	5.392	1.37
TRINITY_Contig7214	6.459	4.665	39.07	37.001	42.159	35.556
TRINITY_69262_length_534_cov_27.709677_g52341_i0	1.175	4.605	20.776	15.409	8.195	1.214
TRINITY_DN14275_c3_g2_i2	20.02	23.704	85.521	85.372	50.422	37.149
TRINITY_DN11129_c0_g2_i2	50.873	54.442	253.678	212.259	73.251	74.476
TRINITY_55064_length_724_cov_27.656489_g38808_i0	30.39	57.024	1.845	10.014	0.968	7.931
TRINITY_Contig17980	11.125	10.785	0.212	0.3	0.585	0.501
TRINITY_52383_length_769_cov_26.514286_g36450_i0	23.957	16.897	4.433	3.013	1.745	2.685
TRINITY_Contig12575	67.677	111.085	463.185	344.355	154.496	110.31
TRINITY_53374_length_752_cov_21.819912_g37303_i0	25.184	18.252	2.015	2.848	4.987	4.333
TRINITY_13996_length_1942_cov_29.030432_g2771_i3	34.405	31.39	8.251	8.191	5.798	6.026
TRINITY_13609_length_1966_cov_29.056405_g7710_i1	14.041	13.587	3.86	3.024	2.083	1.359
TRINITY_DN13209_c0_g3_i4	82.885	76.452	10.277	4.919	0	2.818
TRINITY_Contig849	16.031	15.492	62.009	63.252	113.361	120.67
TRINITY_Contig9287	12.72	9.837	2.227	2.154	1.069	1.359
TRINITY_Contig1057	58.971	53.629	1.633	2.299	4.008	1.604
TRINITY_87282_length_380_cov_33.813505_g70214_i0	50.891	56.508	275.122	272	89.665	84.991
TRINITY_67030_length_558_cov_24.044990_g50152_i0	38.805	31.644	8.983	7.611	5.921	4.534
TRINITY_DN20465_c1_g1_i4	68.046	66.573	7.965	4.536	4.942	1.704
TRINITY_91031_length_352_cov_27.409894_g73960_i0	14.05	14.942	67.174	81.478	30.429	16.876
TRINITY_81996_length_421_cov_27.676136_g64945_i0	16.28	10.091	58.615	36.566	9.175	8.31
TRINITY_BIN_5117_3	17.987	25.185	1.464	1.16	1.79	0.847
TRINITY_Contig12157	30.931	42.573	187.173	141.457	58.313	48.422
TRINITY_5504_length_2770_cov_36.719733_g3491_i0	3.86	5.02	19.005	17.729	19.002	18.58
TRINITY_Contig16170	7.437	6.265	52.051	31.491	105.763	78.308
TRINITY_DN18929_c2_g3_i4	10.542	9.371	1.336	0.88	1.385	0
TRINITY_Contig12664	431.288	439.659	1611.79	1334.733	3970.797	3480.137
TRINITY_DN14268_c0_g1_i2	11.279	15.661	2.938	1.823	0.574	1.582
TRINITY_77395_length_457_cov_42.881443_g60374_i0	5.361	7.026	25.612	20.39	82.516	79.7
TRINITY_Contig12690	3.68	6.73	21.497	21.115	76.358	54.604
TRINITY_70362_length_523_cov_11.724670_g53418_i0	4.958	5.824	27.128	21.933	26.871	31.969
TRINITY_47920_length_849_cov_32.901282_g32662_i0	10.43	11.335	44.394	42.872	26.68	18.558
TRINITY_46918_length_869_cov_21.746250_g31825_i0	1.638	1.727	19.429	15.347	32.781	31.156
TRINITY_Contig5385	18.15	15.018	2.641	4.039	5.696	6.539
TRINITY_BIN_10732_1	3.045	3.522	15.685	13.732	20.421	11.707
TRINITY_Contig8202	0.326	2.76	82.933	42.251	150.758	146.936
TRINITY_32389_length_1205_cov_22.623239_g20878_i0	1.604	1.134	29.748	21.384	55.645	51.73
TRINITY_Contig7842	8.063	6.324	147.138	140.764	0	3.019
TRINITY_Contig10335	2.342	3.259	12.535	11.464	26.241	28.059
TRINITY_DN19026_c3_g2_i4	35.786	28.614	3.203	2.951	6.45	9.969
TRINITY_Contig10863	2.916	3.945	21.942	12.271	2.105	1.771
TRINITY_BIN_2103_8	9.298	11.733	0.901	3.521	0	2.551
TRINITY_Contig12340	24.909	29.409	133.086	112.058	418.108	381.758
TRINITY_BIN_0_103	11.211	10.235	47.066	51.612	149.779	140.453
TRINITY_DN14639_c5_g1_i5	37.784	36.156	140.096	152.082	130.799	171.029
TRINITY_Contig1572	2.076	1.261	7.127	6.048	15.952	14.648
TRINITY_Contig1900	943.135	943.74	179.696	145.776	257.219	232.773
TRINITY_Contig16398	1.012	1.422	6.193	7.86	14.511	15.082
TRINITY_63182_length_605_cov_25.376866_g46425_i0	3.568	6.273	19.726	20.69	4.12	3.241
TRINITY_35675_length_1115_cov_30.729446_g23239_i0	57.504	54.154	0.159	0	140.908	137.846
TRINITY_18526_length_1705_cov_25.967604_g11646_i0	66.905	64.753	12.631	9.434	15.22	13.345
TRINITY_50268_length_806_cov_24.518318_g34625_i0	8.44	5.757	29.748	27.463	35.483	28.438
TRINITY_29544_length_1289_cov_52.859016_g18918_i0	3.019	3.996	21.751	14.198	52.651	47.898
TRINITY_62784_length_610_cov_31.695009_g46042_i0	50.848	55.483	235.31	205.641	95.8	82.919
TRINITY_BIN_11939_5	8.792	8.339	0	0.342	0	0
TRINITY_Contig10061	54.202	49.972	234.461	169.376	469.318	447.735
TRINITY_52786_length_762_cov_14.047619_g36801_i0	6.665	5.198	0	0.28	0	0
TRINITY_Contig17951	2.05	1.998	11.454	10.366	16.526	13.668
TRINITY_DN12250_c0_g1_i3	9.058	10.887	47.172	35.789	13.734	11.184
TRINITY_BIN_1443_3	10.473	11.894	0.615	2.672	0.63	0.88
TRINITY_49097_length_827_cov_34.362797_g33630_i0	3.38	4.334	16.884	18.267	29.393	20.05
TRINITY_DN13393_c2_g2_i7	0	0	54.967	37.177	0	0
TRINITY_Contig17571	17.335	12.394	2.832	2.651	2.589	2.506
TRINITY_87290_length_380_cov_28.405145_g70222_i0	74.65	68.613	11.008	10.459	2.713	1.738
TRINITY_Contig8136	0.515	1.126	4.581	4.774	12	11.819

TRINITY_Contig7277	105.212	96.135	19.185	18.34	18.293	18.079
TRINITY_90468_length_356_cov_57.822300_g73397_i0	11.134	15.856	111.334	89.39	166.395	162.597
TRINITY_DN14702_c3_g2_i4	9.452	4.665	29.78	29.71	48.53	68.806
TRINITY_DN21285_c0_g1_i16	31.016	39.322	3.309	0.331	1.576	2.762
TRINITY_Contig10086	5.189	7.348	35.263	32.113	0	0.111
TRINITY_53238_length_754_cov_25.849635_g37188_i0	36.575	35.64	7.445	8.502	4.064	5.247
TRINITY_DN15811_c1_g1_i4	291.328	298.123	52.867	57.235	94.719	76.615
TRINITY_Contig13050	91.66	98.344	20.468	17.998	18.676	23.336
TRINITY_DN19528_c4_g3_i1	8.595	2.701	29.971	55.061	47.022	31.746
TRINITY_DN16287_c0_g1_i2	13.827	8.449	0.901	2.226	0	0.769
TRINITY_5374_length_2792_cov_42.929857_g3410_i0	2.805	3.175	11.199	12.375	21.186	20.44
TRINITY_22599_length_1526_cov_31.847632_g14314_i0	2.676	5.24	17.34	17.812	1.306	1.236
TRINITY_Contig15838	268.125	278.254	1572.72	1181.812	2146.142	2079.264
TRINITY_Contig18607	37.269	27.251	0.817	0.621	15.85	9.658
TRINITY_60670_length_639_cov_22.564912_g44026_i0	3.997	3.115	12.705	16.331	2.454	2.495
TRINITY_DN22344_c2_g2_i18	12.12	10.768	2.132	2.765	2.465	1.281
TRINITY_54067_length_740_cov_28.119225_g37916_i0	10.971	13.536	3.839	1.75	3.265	4.144
TRINITY_Contig1270	1.33	2.201	15.802	10.501	27.873	23.425
TRINITY_Contig17960	69.066	62.569	4.613	7.011	3.141	3.765
TRINITY_54511_length_733_cov_28.352410_g38316_i0	26.625	35.014	4.2	3.272	4.267	1.938
TRINITY_Contig19955	0.918	0.356	5.547	3.593	9.805	8.711
TRINITY_90607_length_355_cov_71.762238_g73536_i0	58.628	95.39	338.923	194.478	581.283	554.403
TRINITY_Contig2279	24.283	25.684	97.441	99.714	286.78	268.296
TRINITY_53276_length_753_cov_68.157895_g37223_i0	13.124	5.951	69.825	52.172	201.461	195.658
TRINITY_Contig7272	2.419	2.54	8.802	10.19	15.648	14.559
TRINITY_70345_length_523_cov_19.814978_g53401_i0	9.907	11.158	2.354	2.309	0.957	0.635
TRINITY_48977_length_830_cov_13.180026_g33530_i0	7.291	7.644	0	0	0.383	0.267
TRINITY_Contig944	3.585	2.311	15.887	12.81	3.388	2.607
TRINITY_67474_length_553_cov_62.969008_g50588_i0	6.51	5.943	33.195	30.549	105.582	124.29
TRINITY_Contig3533	2.899	2.438	15.24	11.453	1.362	1.17
TRINITY_DN13783_c2_g1_i15	7.025	7.001	30.151	23.994	41.63	41.125
TRINITY_DN14279_c3_g2_i6	5.421	3.699	13.776	20.432	30.654	22.991
TRINITY_DN21758_c2_g1_i6	1.716	2.794	1.591	18.868	0	0.858
TRINITY_BIN_10626_1	31.994	31.28	6.321	5.789	6.563	3.932
TRINITY_54629_length_731_cov_29.590634_g38422_i0	98.625	89.396	20.818	25.382	17.64	13.98
TRINITY_Contig1283	5.001	3.217	21.062	12.965	58.234	51.574
TRINITY_5825_length_2717_cov_37.339124_g3671_i0	3.877	3.674	17.615	20.152	16.92	22.601
TRINITY_Contig10348	1.947	1.431	10.054	9.413	20.027	19.883
TRINITY_Contig15094	2.222	1.871	9.799	6.307	14.409	14.871
TRINITY_DN12804_c0_g2_i3	12.875	10.658	2.004	1.47	1.261	0.869
TRINITY_BIN_18001_1	38.144	42.641	4.815	3.883	7.666	5.603
TRINITY_DN20553_c2_g1_i5	19.325	25.659	178.879	106.818	142.506	95.841
TRINITY_46036_length_885_cov_19.969363_g31105_i0	0.678	0.711	7.307	5.934	9.873	14.303
TRINITY_Contig11431	1.673	1.888	9.311	5.457	0	0
TRINITY_Contig17073	28.563	16.609	91.333	96.732	139.951	118.108
TRINITY_65979_length_570_cov_30.265469_g49125_i0	10.13	15.052	73.293	69.652	28.143	21.008
TRINITY_BIN_858_4	367.625	322.089	37.606	24.097	11.482	13.4
TRINITY_DN18572_c1_g1_i1	8.363	6.849	0	0	0	0.423
TRINITY_13203_length_1992_cov_41.331773_g8267_i0	0	0	32.548	33.49	0	0
TRINITY_Contig11777	10.885	12.284	46.844	42.075	63.131	58.736
TRINITY_49538_length_819_cov_24.449333_g34003_i0	3.028	2.954	15.696	10.169	11.573	19.215
TRINITY_56140_length_706_cov_29.390895_g39779_i0	36.42	44.935	4.168	3.49	4.064	4.144
TRINITY_Contig6899	4.306	3.031	20.002	14.508	7.193	5.169
TRINITY_Contig6593	2.41	3.124	13.31	14.788	22.323	17.266
TRINITY_Contig11252	1.939	2.861	13.235	8.088	1.475	1.827
TRINITY_DN15636_c2_g3_i5	2.024	5.816	23.639	23.87	43.69	41.315
TRINITY_67558_length_552_cov_34.223602_g50672_i0	6.536	7.247	46.865	34.246	15.985	14.659
TRINITY_44507_length_915_cov_25.151300_g29871_i0	2.162	3.699	10.531	13.379	0.664	3.175
TRINITY_DN18316_c1_g2_i6	1.63	1.532	14.169	11.174	0	0
TRINITY_Contig12973	36.686	41.71	1.474	1.408	1065.101	883.34
TRINITY_49997_length_811_cov_33.514825_g34406_i0	3.148	4.597	20.892	15.15	1.193	0
TRINITY_17846_length_1738_cov_29.339125_g11206_i0	14.204	11.649	0.732	0.487	0.946	0.769
TRINITY_BIN_2591_2	3.74	4.14	26.248	13.959	243.293	220.309
TRINITY_28260_length_1330_cov_27.621729_g18041_i0	1.587	2.582	10.648	9.051	0.777	0.401
TRINITY_53646_length_747_cov_26.910029_g37540_i0	0.497	1.549	14.37	11.36	4.199	8.688

TRINITY_Contig12363	5.824	8.152	49.315	33.304	103.714	102.513
TRINITY_Contig9280	5.035	4.741	26.895	28.623	39.13	39.744
TRINITY_DN13007_c0_g1_i3	51.954	63.102	9.152	6.089	183.72	185.098
TRINITY_43965_length_926_cov_21.295216_g29443_i0	27.98	21.96	2.27	3.086	3.884	3.342
TRINITY_Contig12632	1.561	2.514	20.299	11.764	74.231	64.529
TRINITY_22890_length_1515_cov_27.502075_g14498_i0	17.49	18.81	0.53	0.735	0	0
TRINITY_17043_length_1776_cov_18.461629_g10702_i0	8.252	8.135	0	0	0	0.089
TRINITY_59057_length_661_cov_26.596284_g42507_i0	12.343	17.812	0	0.352	1.148	0.78
TRINITY_17744_length_1744_cov_20.575522_g11073_i1	10.971	11.479	0	0	0.417	0.1
TRINITY_60032_length_648_cov_15.336788_g43424_i0	9.324	6.366	0	0	1.193	0.401
TRINITY_Contig707	8.252	8.034	36.768	32.807	77.934	72.304
TRINITY_DN18844_c0_g1_i1	31.943	23.644	5.281	4.639	1.61	2.952
TRINITY_Contig859	9.341	8.787	0	0.321	0.326	0
TRINITY_DN9783_c0_g2_i2	4.786	5.917	30.257	16.103	5.843	7.719
TRINITY_DN21802_c2_g3_i1	12.926	9.769	0	2.63	0	1.192
TRINITY_DN13833_c1_g1_i11	2.813	4.013	16.014	13.038	32.5	22.222
TRINITY_75684_length_472_cov_29.022333_g58684_i0	36.583	51.708	256.33	195.534	76.1	58.436
TRINITY_8109_length_2413_cov_37.381399_g3974_i1	40.718	40.482	4.974	6.493	5.899	6.851
TRINITY_Contig2475	2.342	2.76	12.185	13.7	32.162	26.567
TRINITY_71771_length_509_cov_28.370455_g54812_i0	7.951	5.714	27.722	25.868	1.024	3.42
TRINITY_51271_length_788_cov_33.305981_g33374_i1	5.241	5.367	28.38	29.7	7.261	7.307
TRINITY_81495_length_425_cov_29.362360_g64447_i0	7.883	6.214	27.362	25.34	19.531	12.654
TRINITY_Contig18609	4.152	3.903	18.124	13.794	37.87	32.17
TRINITY_70330_length_523_cov_25.475771_g53386_i0	0	0	43.057	39.258	0	0
TRINITY_BIN_7_6	396.257	385.344	4001.445	2291.238	2753.137	2834.639
TRINITY_Contig5133	25.613	25.574	124.177	85.724	40.346	34.698
TRINITY_Contig19486	91.883	88.423	4.359	3.997	344.711	313.676
TRINITY_19469_length_1662_cov_39.137476_g12241_i0	1.347	1.642	12.97	8.502	33.772	33.206
TRINITY_2433_length_3521_cov_24.759560_g1555_i0	9.298	9.143	0	0	1.902	1.626
TRINITY_DN16992_c4_g1_i4	2.222	2.946	19.28	17.905	18.496	19.181
TRINITY_Contig8306	8.029	8.449	0.106	0.093	0.45	0.802
TRINITY_7910_length_2436_cov_27.240811_g5005_i0	6.15	7.094	0.583	0.849	0	0
TRINITY_Contig12509	1.347	1.871	13.797	12.447	1.993	2.751
TRINITY_43076_length_944_cov_13.602286_g28760_i0	6.588	5.613	0	1.999	0.315	0.212
TRINITY_36456_length_1095_cov_17.918129_g23786_i0	1.33	1.033	4.221	4.691	13.407	13.623
TRINITY_68424_length_543_cov_27.683544_g51528_i0	3.963	6.197	22.154	25.444	1.745	2.93
TRINITY_DN21725_c0_g1_i15	1.021	1.024	26.407	11.153	23.393	8.421
TRINITY_44602_length_913_cov_24.276066_g29946_i0	2.813	2.362	15.844	14.053	0.326	0
TRINITY_DN19858_c2_g2_i5	9.324	8.313	42.729	32.486	53.844	52.008
TRINITY_29224_length_1300_cov_17.233144_g18694_i0	6.579	9.515	54.554	46.776	54.834	52.198
TRINITY_DN10833_c0_g3_i1	8.003	9.685	43.726	38.813	18.586	12.999
TRINITY_51836_length_778_cov_26.496474_g24322_i1	25.415	25.143	3.298	1.274	5.133	4.277
TRINITY_DN21719_c0_g6_i1	17.85	17.354	0.997	0.642	0.754	1.158
TRINITY_DN16512_c0_g2_i1	3.448	4.063	27.69	23.942	19.52	24.428
TRINITY_31800_length_1221_cov_31.754340_g20474_i0	1.321	1.219	6.851	10.314	13.498	13.668
TRINITY_Contig12360	2.865	3.217	13.564	12.675	37.768	43.476
TRINITY_Contig8378	7.6	12.453	46.525	49.282	71.889	72.571
TRINITY_24064_length_1472_cov_12.982181_g15270_i0	0.223	0.432	3.691	3.49	9.163	10.805
TRINITY_Contig12126	18.107	11.005	1.241	1.636	2.657	0.446
TRINITY_Contig7926	2.35	1.837	13.013	10.604	42.271	30.878
TRINITY_Contig10944	9.727	11.564	1.559	1.077	0.484	1.437
TRINITY_Contig2204	58.971	71.644	0	0	85.286	61.376
TRINITY_DN21959_c1_g2_i1	0.978	3.056	9.163	15.616	25.487	21.454
TRINITY_DN14658_c0_g1_i2	19.677	21.333	2.418	1.512	0.507	0
TRINITY_Contig1936	34.087	30.713	0	0	2.184	3.932
TRINITY_DN10431_c0_g1_i1	3.903	3.158	13.151	15.647	0.417	10.137
TRINITY_Contig10545	43.317	43.166	11.92	7.694	7.835	6.416
TRINITY_Contig11502	1.355	1.236	14.784	10.739	54.621	44.3
TRINITY_Contig8054	25.089	24.762	104.154	99.093	327.521	318.533
TRINITY_Contig10929	18.287	25.05	87.515	78.889	29.033	21.532
TRINITY_47022_length_867_cov_21.624060_g31913_i0	51.851	44.046	7.084	2.496	2.51	2.952
TRINITY_DN10773_c0_g4_i1	34.516	47.306	187.894	163.246	64.932	48.366
TRINITY_Contig633	5.773	5.393	21.02	25.019	31.588	33.161
TRINITY_BIN_20500_1	2.779	2.709	14.678	13.669	1.767	1.214
TRINITY_62861_length_609_cov_35.333333_g46116_i0	49.698	44.673	201.394	157.55	48.744	38.853

TRINITY_56598_length_698_cov_31.243243_g40202_i0	9.127	12.918	42.845	43.991	18.327	7.263
TRINITY_Contig6503	0.343	0.737	6.098	5.323	20.218	19.805
TRINITY_Contig7651	4.949	7.153	36.111	28.188	89.158	71.379
TRINITY_DN16903_c0_g1_i9	19.866	13.655	0	1.771	3.873	6.795
TRINITY_Contig19759	2.728	3.31	23.523	17.76	26.466	25.353
TRINITY_Contig17316	32.706	47.424	192.496	157.819	54.53	41.85
TRINITY_DN14398_c1_g1_i15	3.225	2.557	77.694	73.991	0	0
TRINITY_Contig17572	20.269	22.019	128.409	106.932	322.556	327.645
TRINITY_Contig1754	0.815	0.957	5.547	4.857	15.209	14.169
TRINITY_Contig15185	3.585	4.698	24.445	21.032	35.652	45.381
TRINITY_35899_length_1110_cov_10.097022_g23400_i0	2.119	2.819	13.394	13.203	2.736	2.918
TRINITY_46552_length_875_cov_14.988834_g31517_i0	1.081	1.642	9.937	10.915	10.604	12.799
TRINITY_44022_length_925_cov_15.968458_g29486_i0	1.913	2.811	11.136	9.455	23.19	20.964
TRINITY_Contig11435	1.304	3.048	11.443	8.626	0.664	0.679
TRINITY_21825_length_1561_cov_14.347855_g13774_i0	5.198	6.933	0	0.104	0	0.111
TRINITY_63104_length_606_cov_28.420857_g46350_i0	3.551	5.553	21.37	27.774	4.109	5.536
TRINITY_BIN_18342_1	10.559	9.896	1.326	1.854	1.238	1.426
TRINITY_DN13209_c0_g3_i1	46.01	42.328	2.323	2.63	3.681	3.219
TRINITY_Contig5358	1.673	4.343	16.767	16.082	5.313	3.097
TRINITY_64848_length_584_cov_28.415534_g48029_i0	2.882	4.131	18.899	14.933	6.867	3.487
TRINITY_Contig6971	17.215	21.316	110.009	94.091	48.44	29.262
TRINITY_DN17176_c0_g1_i4	45.221	41.667	256.298	241.513	68.062	48.121
TRINITY_58645_length_667_cov_26.615385_g42113_i0	3.68	2.294	14.922	10.873	12.417	13.033
TRINITY_10300_length_2207_cov_78.101497_g6166_i4	174.407	173.425	25.081	28.892	14.049	16.486
TRINITY_45143_length_902_cov_21.211285_g30392_i0	2.385	5.308	19.917	15.047	6.552	4.623
TRINITY_Contig12026	388.546	378.546	89.127	97.684	88.167	81.371
TRINITY_DN13181_c0_g2_i1	87.611	95.441	404.093	291.375	134.39	115.345
TRINITY_Contig16117	3.903	3.513	15.431	18.309	27.558	23.682
TRINITY_Contig12488	1.209	1.575	14.667	10.376	33.693	26.032
TRINITY_Contig4042	10.773	9.803	2.238	1.885	2.51	1.392
TRINITY_Contig12458	2.573	3.065	17.35	11.319	35.922	34.954
TRINITY_54238_length_737_cov_40.712575_g38072_i0	56.577	33.744	7.063	4.474	4.57	2.941
TRINITY_59961_length_649_cov_10.224138_g43356_i0	4.263	3.327	13.129	19.189	30.946	35.545
TRINITY_Contig19652	1.381	5.096	16.979	8.75	21.67	17.956
TRINITY_Contig556	3.157	3.903	24.763	23.6	50.084	44.59
TRINITY_Contig3620	3.989	1.744	28.412	26.262	1.925	4.222
TRINITY_DN13181_c0_g8_i1	72.455	94.975	379.064	309.508	134.491	108.55
TRINITY_DN14345_c5_g1_i2	65.49	80.981	3.107	11.598	3.287	9.134
TRINITY_Contig10704	3.079	4.14	16.47	14.767	15.141	14.893
TRINITY_52819_length_761_cov_28.182081_g36830_i0	4.649	6.993	23.056	24.843	4.447	5.157
TRINITY_DN16975_c0_g2_i6	17.773	17.202	1.495	1.098	1.069	0.457
TRINITY_Contig1914	4.477	3.556	26.566	15.958	31.791	34.899
TRINITY_36799_length_1086_cov_26.382498_g24034_i0	43.394	38.671	3.553	6.949	5.077	4.6
TRINITY_DN18813_c1_g1_i3	1.818	2.37	8.665	9.776	13.554	14.024
TRINITY_65025_length_582_cov_25.727096_g48200_i0	16.941	13.24	3.256	1.823	5.223	3.008
TRINITY_Contig1568	2.856	5.266	41.223	38.875	76.741	73.952
TRINITY_29826_length_1281_cov_21.981023_g19120_i0	30.716	31.822	5.653	3.407	9.186	5.792
TRINITY_Contig8255	2.668	2.785	21.645	12.199	49.285	44.267
TRINITY_Contig16942	6.296	6.645	39.165	26.448	180.815	154.867
TRINITY_Contig11948	32.457	31.052	3.351	2.982	2.657	2.161
TRINITY_Contig12207	2.307	3.92	23.12	18.464	46.02	42.039
TRINITY_70529_length_521_cov_35.712389_g53582_i0	10.628	12.241	57.746	48.35	0.968	0.646
TRINITY_Contig15201	16.572	16.643	66.272	58.178	18.8	9.067
TRINITY_Contig18228	20.037	14.476	4.454	4.008	2.6	0.579
TRINITY_Contig2379	5.412	4.563	22.08	15.047	69.401	65.587
TRINITY_53593_length_748_cov_27.405007_g37493_i0	70.868	104.448	6.384	8.326	13.272	9.067
TRINITY_53585_length_748_cov_32.690722_g37487_i0	23.134	19.598	101.705	71.288	126.307	134.159
TRINITY_Contig12180	24.197	24.973	101.694	84.036	26.86	26.11
TRINITY_40180_length_1007_cov_15.253731_g26533_i0	2.445	2.15	9.683	10.977	13.88	12.665

Supplementary table 1. *In silico* expression matrix for DEGs in Root (control vs salt, control vs drought) and Leaves (control vs drought, control vs salt) and the UniProt Ids for the Unigenes.

Unigene ID	CONTROL1	CONTROL2	PEG_TREATED _LEAVES1	PEG_TREATED _LEAVES2	SALT_TREATED _LEAVES1	SALT_TREATED _LEAVES2
TRINITY_Contig10832	42.656	35.493	2.171	6.718	7.326	11.508
TRINITY_BIN_4886_7	16.193	20.631	1.466	1.439	12.327	60.889
TRINITY_25469_length_1421_cov_26.433432_g16200_i0	6.693	6.247	0.253	1.188	0.552	1.41
TRINITY_57587_length_683_cov_25.188925_g41125_i0	2.39	2.164	365.056	205.547	13.614	35.047
TRINITY_DN13139_c0_g1_i2	40.127	45.107	0	0.38	18.816	1.51
TRINITY_Contig7516	20.767	59.91	3.709	5.546	28.717	74.207
TRINITY_50925_length_794_cov_25.200000_g35189_i0	159.893	74.439	8.467	2.991	1.806	4.537
TRINITY_1325_length_4122_cov_24.586973_g857_i0	4.435	6.486	0.362	0.097	0	2.203
TRINITY_Contig7003	5.08	17.272	0.145	0	0.167	1.386
TRINITY_DN20129_c1_g1_i1	9.046	14.616	0.507	1.617	2.224	3.651
TRINITY_9543_length_2274_cov_27.739229_g5996_i0	29.579	25.858	0.941	6.904	1.873	8.265
TRINITY_4828_length_2893_cov_30.035765_g3070_i0	5.842	8.586	0	1.035	0.284	1.841
TRINITY_Contig448	4.501	5.755	0	0.647	0.535	2.126
TRINITY_82391_length_418_cov_29.868195_g65338_i0	7.059	14.29	0	0	0	15.22
TRINITY_DN22323_c2_g1_i3	14.91	9.896	1.194	0.639	0	0.886
TRINITY_BIN_16970_4	9.42	10.156	0.362	0.97	5.268	8.619
TRINITY_BIN_3238_3	1.29	2.802	237.231	118.7	28.416	10.268
TRINITY_DN21081_c2_g2_i3	9.786	8.6	0	0.137	0	0.531
TRINITY_DN17961_c0_g1_i1	7.382	7.84	0	1.019	1.455	2.072
TRINITY_DN18954_c2_g2_i4	35.267	46.996	4.451	7.115	3.88	16.615
TRINITY_37113_length_1078_cov_30.117939_g23146_i1	0.909	2.649	342.621	1610.565	623.754	237.857
TRINITY_BIN_3718_1	0	0.456	142.281	33.01	10.771	6.955
TRINITY_22888_length_1515_cov_28.957123_g14496_i0	3.277	6.913	0	0	2.158	5.492
TRINITY_47734_length_853_cov_25.942602_g32504_i0	1.503	1.397	425.431	182.045	9.299	3.358
TRINITY_BIN_16563_3	3.841	5.929	0	0	0	0
TRINITY_Contig13523	1.488	7.76	764.976	379.361	329.271	701.833
TRINITY_60378_length_643_cov_25.811847_g43753_i0	1.591	1.52	474.933	112.871	13.464	5.222
TRINITY_Contig17563	27.057	34.458	1.99	4.487	14.3	29.863
TRINITY_BIN_866_7	2.639	5.502	956.379	594.165	264.528	43.697
TRINITY_BIN_20507_2	8.408	17.62	0	1.019	0	0.254
TRINITY_1996_length_3708_cov_30.695796_g1277_i0	8.973	13.342	1.321	1.633	1.472	1.61
TRINITY_Contig9143	7.55	7.087	0	1.051	0.401	1.471
TRINITY_DN17363_c1_g2_i1	26.192	15.064	2.298	3.347	8.045	10.468
TRINITY_BIN_1717_7	0.337	0.478	442.221	379.539	80.332	137.415
TRINITY_BIN_14760_1	23.296	60.844	4.668	3.606	11.624	22.153
TRINITY_BIN_9764_3	49.913	93.818	2.841	13.704	3.83	25.796
TRINITY_23221_length_1502_cov_27.458479_g14712_i0	5.102	12.668	0.271	0.986	0	1.148
TRINITY_BIN_3017_2	1247.47	1467.602	158.04	327.546	180.985	775.994
TRINITY_17925_length_1734_cov_24.494895_g11252_i0	5.718	6.03	0.199	0.897	0	0.239
TRINITY_DN18773_c0_g1_i11	4.779	6.081	0	0.647	0.97	2.203
TRINITY_DN17266_c1_g2_i4	140.849	96.649	5.102	11.901	16.474	41.471
TRINITY_DN16027_c0_g1_i1	0.323	0.203	34.774	90.194	7.945	0.632
TRINITY_DN20537_c1_g1_i7	26.962	22.094	2.352	0.016	16.157	2.342
TRINITY_Contig2888	53.198	43.442	5.482	11.391	13.966	14.481
TRINITY_BIN_1602_10	9.896	6.899	0.832	1.091	0.702	1.171
TRINITY_Contig8909	2.94	12.285	0.47	0.105	0	0.185
TRINITY_DN20690_c3_g1_i1	127.61	55.328	7.418	8.562	10.219	11.215
TRINITY_BIN_6569_1	3.137	16.368	729.37	10913.893	646.4	188.306
TRINITY_BIN_6283_1	0.154	0	307.63	311.498	47.634	0.816
TRINITY_BIN_20631_1	0	0	77.021	22.751	0.903	0.069
TRINITY_DN22073_c1_g2_i8	2312.308	2192.036	56.431	251.355	250.328	913.502
TRINITY_BIN_11547_3	6.436	12.813	0	1.318	3.763	2.942
TRINITY_DN20488_c1_g1_i13	12.975	16.853	0.778	1.14	5.503	5.415
TRINITY_1533_length_3973_cov_27.862705_g941_i1	24.095	10.316	0	1.407	1.305	2.842

TRINITY_9683_length_2260_cov_41.896394_g6079_i0	0.198	0.398	81.598	48.792	15.655	14.389
TRINITY_DN22307_c0_g3_i1	5.359	5.263	0	0	0	0.439
TRINITY_23368_length_1496_cov_34.950946_g13703_i1	129.435	68.633	1.194	7.276	5.804	47.094
TRINITY_23371_length_1496_cov_27.297127_g14812_i0	3.489	8.738	0.561	0.34	0	0.285
TRINITY_DN15746_c0_g1_i4	4.999	7.232	0	0.146	0.318	1.34
TRINITY_Contig10698	6.553	7.087	0	0.808	0.502	0.747
TRINITY_Contig1009	88.604	18.93	0.543	4.39	1.806	1.086
TRINITY_Contig13120	37.122	32.424	4.451	7.721	4.633	6.709
TRINITY_33458_length_1176_cov_12.514905_g21625_i0	6.444	4.025	0.308	0.639	0	0.308
TRINITY_DN20844_c0_g2_i1	4.567	12.14	0	0.097	2.191	2.634
TRINITY_BIN_5115_6	162.943	136.796	16.374	42.17	31.36	97.615
TRINITY_DN21509_c2_g3_i2	0.125	2.28	203.868	164.614	68.975	108.268
TRINITY_DN14317_c1_g1_i1	122.669	51.47	0.326	1.488	1.104	5.091
TRINITY_40499_length_1000_cov_22.002148_g26779_i0	10.849	21.905	0	0.129	0	6.432
TRINITY_DN22005_c2_g1_i1	22.167	30.534	1.809	2.991	8.814	8.226
TRINITY_DN13872_c0_g1_i3	126.935	93.608	1.086	10.033	5.268	14.866
TRINITY_Contig15352	4.435	16.071	0.615	0.574	2.442	7.487
TRINITY_DN13227_c0_g1_i16	0.704	0.203	165.856	64.832	3.312	2.149
TRINITY_35304_length_1125_cov_27.677083_g22970_i0	1.422	2.744	394.095	169.052	140.259	234.738
TRINITY_19576_length_1657_cov_44.516373_g12312_i0	14.009	10.497	1.086	2.466	0	5.107
TRINITY_36620_length_1091_cov_31.309198_g23902_i0	0.242	0.883	194.569	250.296	18.063	13.287
TRINITY_DN11567_c0_g1_i1	0.095	1.404	277.867	77.808	15.371	32.382
TRINITY_DN19204_c1_g2_i4	0	0.398	397.695	119.331	9.115	0.955
TRINITY_6752_length_2581_cov_35.234873_g3991_i1	4.67	21.42	0.579	0.534	4.114	15.359
TRINITY_DN18728_c0_g5_i4	7.58	14.768	129.67	154.249	225.14	504.576
TRINITY_DN11954_c3_g2_i2	318.995	330.485	48.724	86.45	59.676	146.404
TRINITY_16880_length_1784_cov_31.650729_g10598_i0	8.071	5.574	0.38	0.784	0.636	0.47
TRINITY_29819_length_1281_cov_29.047030_g17545_i1	0.066	0.217	201.046	24.206	1.974	0.139
TRINITY_BIN_6546_2	21.566	26.278	2.877	3.727	11.507	15.39
TRINITY_BIN_24258_1	14.815	19.806	0	0	4.215	4.93
TRINITY_4092_length_3037_cov_27.128369_g2604_i0	10.255	5.943	0.416	0.922	0.351	1.641
TRINITY_Contig15930	111.365	61.112	5.355	9.322	4.114	5.831
TRINITY_22764_length_1520_cov_31.175052_g14411_i0	17.271	15.028	1.248	3.646	3.696	7.695
TRINITY_DN17626_c0_g2_i1	12.77	22.065	1.791	1.18	0	4.991
TRINITY_BIN_42_20	42.092	12.198	0.416	1.568	6.991	7.264
TRINITY_BIN_943_9	21.493	14.123	1.013	3.808	7.158	6.439
TRINITY_55328_length_720_cov_22.572965_g39043_i0	13.451	7.731	0.615	0	0	1.702
TRINITY_BIN_11398_2	95.868	59.505	3.564	5.15	7.744	17.462
TRINITY_51168_length_790_cov_19.793343_g35401_i0	2.661	17.54	0	0	1.823	2.688
TRINITY_DN13944_c0_g1_i9	537.605	217.432	14.8	52.761	6.707	8.858
TRINITY_BIN_8248_1	12.411	8.875	0.651	1.431	0.585	0.909
TRINITY_Contig3439	16.281	20.696	0.995	0.986	0.134	0.154
TRINITY_Contig15938	60.176	26.061	5.374	9.613	13.949	21.529
TRINITY_Contig12714	5.652	16.063	0.217	0	1.606	1.918
TRINITY_DN15786_c2_g2_i2	16.208	14.138	1.447	3.274	0.652	1.094
TRINITY_DN17363_c1_g2_i11	8.577	3.315	0.651	0.671	1.606	1.856
TRINITY_5027_length_2855_cov_19.676956_g1942_i1	13.268	5.979	0.507	0.711	0.619	0.408
TRINITY_DN18660_c1_g2_i26	157.951	99.211	10.946	30.342	46.864	43.196
TRINITY_6483_length_2616_cov_33.980762_g4098_i0	20.65	20.943	0.543	4.358	1.037	7.256
TRINITY_BIN_12007_1	1.114	4.814	667.312	344.75	34.237	23.747
TRINITY_BIN_912_4	0.33	0	187.856	33.657	2.559	0.693
TRINITY_Contig11942	82.417	72.173	3.6	3.525	3.596	3.697
TRINITY_DN18700_c0_g1_i10	0	0.101	25.239	23.43	33.551	45.33
TRINITY_Contig16328	0.469	0.478	157.334	50.465	1.84	1.479
TRINITY_17597_length_1750_cov_27.232005_g11053_i0	0.044	0.391	112.41	28.766	7.058	13.487
TRINITY_BIN_19022_2	3.211	8.643	0	0.566	0.418	1.587
TRINITY_BIN_4317_1	0.953	0.825	37.597	33.495	34.003	49.197
TRINITY_Contig9689	0	1.122	540.103	32.646	54.826	31.989
TRINITY_DN13722_c5_g5_i2	47.956	29.087	1.013	5.853	0.418	4.991
TRINITY_DN12706_c0_g1_i2	70.49	57.536	2.822	12.313	10.754	34.084
TRINITY_DN22383_c0_g1_i1	87.607	27.067	0.905	8.877	19.401	8.519
TRINITY_Contig17766	7.448	5.335	0.416	0.59	1.388	2.781
TRINITY_BIN_17558_5	5.813	19.871	0	0	0	0.339

TRINITY_Contig9265	20.122	22.6	2.461	4.342	5.854	19.395
TRINITY_53181_length_755_cov_23.173469_g37141_i0	77.227	96.953	11.416	6.743	127.832	139.618
TRINITY_Contig17597	0.169	0.601	78.595	53.02	24.084	75.994
TRINITY_BIN_2309_7	4.809	5.017	0.326	0	0	6.586
TRINITY_BIN_2745_2	90.972	109.013	8.757	26.542	9.149	37.974
TRINITY_Contig8667	28.728	47.561	0.633	1.9	2.007	24.094
TRINITY_DN19004_c0_g1_i1	529.82	457.074	0	0.21	1.405	0.308
TRINITY_Contig17998	9.845	13.486	0.452	1.294	3.529	4.93
TRINITY_Contig6883	0.117	1.651	126.902	88.366	53.856	70.579
TRINITY_BIN_14597_1	2.595	2.44	1329.178	549.804	68.691	26.482
TRINITY_DN22598_c6_g1_i6	47.011	59.621	2.587	11.472	16.29	22.176
TRINITY_DN20508_c0_g5_i3	5.205	9.556	0	0	0	3.975
TRINITY_BIN_6019_2	274.821	175.062	7.707	25.968	25.121	99.734
TRINITY_Contig9243	1.158	1.318	206.257	63.239	20.154	26.913
TRINITY_BIN_4564_7	2.382	3.207	90.192	312.169	132.766	85.399
TRINITY_10521_length_2190_cov_26.274399_g2377_i1	0	0.253	105.824	20.268	5.319	3.274
TRINITY_Contig6397	0.367	1.274	130.919	41.523	9.901	3.736
TRINITY_BIN_3400_2	0.059	0.203	77.961	20.851	1.422	0.285
TRINITY_Contig12789	1.078	0.804	104.123	39.664	0.518	0.801
TRINITY_44556_length_914_cov_25.604734_g29908_i0	31.36	69.198	0	1.447	6.857	25.057
TRINITY_BIN_7735_17	24.814	20.972	1.248	4.697	7.125	20.558
TRINITY_DN16486_c0_g3_i3	146.09	65.151	0.959	6.727	16.826	20.304
TRINITY_Contig13158	9.105	10.222	0.814	1.374	2.793	4.236
TRINITY_DN16117_c5_g2_i4	0.154	0.434	180.33	25.944	13.113	23.855
TRINITY_DN9469_c0_g1_i2	0.147	0.362	87.008	42.211	9.851	4.375
TRINITY_DN17534_c0_g2_i11	0.44	0.695	37.235	19.541	27.597	33.368
TRINITY_BIN_15103_2	0.433	0.514	161.043	48.695	11.724	14.096
TRINITY_4942_length_2873_cov_26.481455_g3145_i0	2.962	7.652	0.326	0.331	0.853	1.101
TRINITY_DN18037_c0_g1_i23	0	0.471	13.877	3.25	21.191	19.11
TRINITY_BIN_9932_5	11.318	16.512	0.923	3.113	2.676	5.515
TRINITY_Contig10204	27.262	11.192	0.615	0.323	0	0
TRINITY_DN16557_c0_g2_i2	4.45	7.485	0	0.243	0	0.239
TRINITY_DN16742_c2_g3_i2	32.086	25.728	0	0	144.022	64.702
TRINITY_Contig20107	0.447	2.027	174.938	62.107	31.042	36.233
TRINITY_9820_length_2247_cov_25.817723_g6171_i0	1.224	1.006	387.039	145.598	18.298	5.839
TRINITY_Contig3189	0.176	1.086	630.62	691.231	111.976	253.255
TRINITY_BIN_1703_7	2350.625	1916.539	160.952	486.638	249.375	503.845
TRINITY_BIN_11843_2	24.139	19.821	1.466	4.188	3.295	1.04
TRINITY_Contig17905	13.276	22.644	0.181	1.892	2.843	9.043
TRINITY_36657_length_1090_cov_26.702253_g1902_i2	0.777	2.497	145.501	56.359	71.517	74.176
TRINITY_BIN_1107_10	86.918	104.865	8.902	24.295	38.92	108.823
TRINITY_DN18492_c0_g1_i5	7.536	14.927	1.067	0.275	7.242	26.99
TRINITY_37307_length_1074_cov_24.689552_g24415_i0	3.599	8.006	0	0.267	1.957	4.367
TRINITY_Contig20078	13.166	13.038	1.701	3.145	1.254	3.767
TRINITY_13929_length_1946_cov_17.416622_g8719_i0	0.117	0.565	122.035	294.124	57.569	6.686
TRINITY_DN21167_c0_g2_i1	20.459	9.425	0	1.091	1.522	2.449
TRINITY_Contig1252	13.642	17.164	0.615	1.027	0.351	2.935
TRINITY_Contig17757	23.758	13.262	0.941	3.331	6.406	10.383
TRINITY_8846_length_2340_cov_24.682078_g5586_i0	5.241	4.575	0.543	0.21	0	1.433
TRINITY_BIN_8243_7	48.667	41.936	0	3.129	4.967	13.695
TRINITY_9542_length_2274_cov_29.067574_g5995_i0	6.062	4.553	0.145	0.146	0.636	2.257
TRINITY_8636_length_2358_cov_28.701616_g5459_i0	12.711	9.881	0.145	0.17	0.151	0.37
TRINITY_17849_length_1738_cov_25.905333_g11208_i0	5.219	1.723	0	0	9.249	13.141
TRINITY_43819_length_929_cov_19.138372_g29325_i0	20.225	15.6	2.316	2.199	3.178	19.403
TRINITY_BIN_13114_2	6.011	6.095	0	0	0	7.51
TRINITY_DN18423_c0_g1_i3	4.655	11.112	0.199	0	5.921	6.17
TRINITY_5300_length_2804_cov_28.563803_g3365_i0	16.977	21.898	0	1.019	0.251	5.407
TRINITY_BIN_2309_14	0.535	0.637	287.692	111.562	11.524	7.533
TRINITY_Contig10665	88.663	71.732	6.26	14.561	2.559	4.837
TRINITY_BIN_3323_2	0.066	0.195	63.994	143.027	10.035	6.085
TRINITY_8847_length_2340_cov_23.405108_g5587_i0	0	0.608	390.494	53.012	16.608	5.207
TRINITY_Contig360	7.243	4.865	0.308	1.003	0.167	0.67
TRINITY_DN19356_c0_g2_i2	3.445	7.471	1452.824	272.917	37.18	26.975

TRINITY_Contig12699	88.479	50.659	5.862	17.56	43.118	66.258
TRINITY_BIN_2948_2	0.579	0	212.137	40.125	0	0.732
TRINITY_53_length_8517_cov_24.605587_g29_i0	96.367	37.585	1.845	10.243	1.873	1.895
TRINITY_BIN_9927_1	69.948	36.391	8.594	4.77	1.422	2.403
TRINITY_1451_length_4022_cov_28.028586_g941_i0	10.703	4.799	0.452	0.752	0.301	0.77
TRINITY_29071_length_1305_cov_29.024272_g18592_i0	6.246	3.793	0	0.631	0.92	1.086
TRINITY_DN19720_c0_g6_i2	0	0.434	207.668	98.246	0	1.787
TRINITY_21197_length_1586_cov_38.420567_g13360_i0	1.635	5.965	14.637	33.649	106.406	82.657
TRINITY_BIN_19978_1	2.793	6.805	0	0.129	0	0.139
TRINITY_47946_length_849_cov_17.776923_g32684_i0	2.617	6.761	0	0.121	1.087	0.847
TRINITY_DN16989_c1_g1_i5	7.287	14.666	0.235	0.065	0	0.755
TRINITY_BIN_3639_11	5.71	8.665	0.489	0.825	2.258	2.064
TRINITY_16708_length_1793_cov_22.377030_g10491_i0	17.417	16.932	1.176	0.259	0	0.046
TRINITY_46902_length_869_cov_27.276250_g31811_i0	8.1	4.988	0	0.72	1.054	1.756
TRINITY_57881_length_678_cov_26.144499_g41397_i0	0.806	1.593	470.898	104.019	83.192	94.943
TRINITY_DN19424_c1_g1_i8	0.454	0.565	219.138	70.766	1.305	1.163
TRINITY_DN22308_c1_g5_i10	32.306	72.036	3.401	3.444	43.185	146.419
TRINITY_BIN_13823_3	14.412	7.623	0.597	0.768	0.284	5.199
TRINITY_DN14161_c0_g2_i3	2.485	3.953	768.016	284.883	540.914	25.357
TRINITY_BIN_20189_1	0	0.999	184.455	451.453	41.579	7.079
TRINITY_DN19722_c1_g1_i7	3.643	6.262	31.825	32.598	100.034	86.231
TRINITY_Contig7579	0	0	60.846	27.31	0.903	0.3
TRINITY_BIN_7439_3	18.773	18.119	0.362	3.703	6.556	7.656
TRINITY_5616_length_2753_cov_27.887481_g3547_i0	8.782	8.231	0.579	1.447	0.585	3.22
TRINITY_DN20204_c1_g1_i14	9.684	9.606	0	0	4.633	6.247
TRINITY_DN20137_c1_g2_i3	2.031	1.194	299.687	134.175	6.657	8.434
TRINITY_4103_length_3034_cov_28.572344_g2612_i0	17.527	15.216	1.429	4.536	7.827	7.841
TRINITY_Contig982	5.879	10.996	0.235	1.245	2.726	11.824
TRINITY_Contig16066	6.605	5.009	0.199	0.323	0.201	0.763
TRINITY_DN22120_c2_g3_i9	2.844	7.073	0.109	0.428	0.602	2.588
TRINITY_DN14937_c0_g3_i3	0.081	0.101	38.121	18.078	0	0.169
TRINITY_DN9995_c0_g1_i2	0	0.239	185.739	179.716	14.802	3.682
TRINITY_33143_length_1184_cov_24.504036_g21408_i0	8.152	2.881	0	0.307	0	0.193
TRINITY_3946_length_3071_cov_33.057295_g2507_i0	11.326	13.233	1.212	2.628	4.984	9.99
TRINITY_BIN_10690_3	0	0	97.375	33.01	25.222	0
TRINITY_11732_length_2093_cov_33.770257_g7338_i0	35.26	15.267	0.778	2.902	5.185	8.843
TRINITY_Contig17996	10.527	11.554	0	1.294	0.569	3.058
TRINITY_DN11273_c0_g1_i2	7.411	6.602	0.09	0.606	1.555	1.517
TRINITY_Contig12208	221.103	128.747	22.019	41.887	35.809	62.923
TRINITY_38427_length_1047_cov_16.186094_g25185_i0	0	0	119.774	49.155	4.466	6.147
TRINITY_Contig16022	39.592	40.17	454.524	135.379	17.177	8.966
TRINITY_BIN_2575_1	6.971	5.104	0	0.8	0.903	0.994
TRINITY_21652_length_1568_cov_26.823883_g13659_i0	67.06	68.995	0.868	0.275	0	0.216
TRINITY_26921_length_1373_cov_23.520706_g17146_i0	46.901	23.78	0	0.323	0.569	0.062
TRINITY_BIN_3951_27	2.954	1.477	52.65	62.293	59.124	57.462
TRINITY_DN18305_c2_g2_i2	59.912	43.948	3.908	5.328	3.847	10.221
TRINITY_DN15624_c0_g3_i6	6.942	8.846	0.941	1.156	0.201	3.52
TRINITY_60536_length_641_cov_19.125874_g43899_i0	9.405	11.792	0	0.574	0	0.377
TRINITY_BIN_3654_11	25.693	17.642	0	2.054	4.114	2.85
TRINITY_Contig816	13.965	15.13	0.525	2.142	1.455	2.334
TRINITY_DN21819_c0_g1_i5	0	0.42	216.407	84.575	87.724	0.562
TRINITY_BIN_5381_2	56.665	61.329	9.824	11.48	4.198	15.968
TRINITY_DN16037_c0_g1_i3	131.693	185.421	5.247	21.093	3.864	47.433
TRINITY_Contig14472	13.034	8.73	1.194	2.611	5.218	3.713
TRINITY_DN11762_c0_g1_i1	60.785	53.243	6.767	5.029	4.7	4.999
TRINITY_BIN_2956_3	28.838	17.627	0.742	1.908	1.238	8.966
TRINITY_16946_length_1781_cov_26.710864_g10641_i0	14.353	21.645	1.683	0.873	4.399	6.609
TRINITY_25561_length_1418_cov_29.464789_g8577_i3	37.422	32.069	4.577	8.667	14.919	7.672
TRINITY_BIN_390_6	8.797	10.178	0	1.269	2.291	3.397
TRINITY_52272_length_771_cov_21.150997_g36351_i0	7.748	4.872	0	0	0	0.555
TRINITY_Contig1421	1.833	19.618	0	0	0	0
TRINITY_BIN_1436_3	12.836	28.942	0	0.768	2.191	8.82
TRINITY_DN18933_c1_g4_i1	0.792	0.485	333.665	117.666	10.353	2.496

TRINITY_DN16940_c0_g4_i2	176.761	423	25.547	42.971	37.766	29.794
TRINITY_87124_length_381_cov_35.833333_g70056_i0	23.194	16.52	0	0	0	0
TRINITY_DN20690_c3_g1_i2	130.732	61.612	2.171	8.651	25.924	7.402
TRINITY_Contig8236	1.254	5.111	756.907	185.852	15.237	12.008
TRINITY_Contig12319	27.981	84.118	1.086	0	2.526	29.216
TRINITY_BIN_3704_2	6.392	5.263	0.941	0.784	4.114	3.882
TRINITY_Contig12895	26.346	24.779	1.719	2.87	3.579	5.685
TRINITY_DN16486_c0_g3_i4	19.704	17.801	0.669	2.207	3.027	3.358
TRINITY_7372_length_2502_cov_32.012741_g4658_i0	2.404	25.8	0.253	0.356	0.134	0.347
TRINITY_51831_length_778_cov_27.468265_g35969_i0	1.554	0.319	239.999	369.627	128.283	22.854
TRINITY_Contig10267	22.277	23.541	1.176	5.247	2.375	7.834
TRINITY_DN18272_c1_g1_i1	15.247	6.168	0	0.841	0.401	2.103
TRINITY_Contig4915	45.523	60.859	3.275	11.893	8.145	28.23
TRINITY_5299_length_2804_cov_29.469835_g3364_i0	8.247	22.513	0	0.032	0	0
TRINITY_7546_length_2480_cov_25.817503_g4782_i0	429.18	288.281	16.41	37.441	53.187	133.464
TRINITY_4116_length_3032_cov_26.498819_g2623_i0	7.448	6.609	0.308	0.291	4.583	4.021
TRINITY_Contig1225	9.42	15.687	0	1.221	1.154	2.989
TRINITY_BIN_6091_7	8.379	8.238	0	0.234	0.953	3.027
TRINITY_Contig8430	11.897	11.879	0.905	1.221	0	0.393
TRINITY_DN18700_c0_g1_i3	0	0.101	10.476	14.658	16.19	26.62
TRINITY_DN16990_c4_g1_i6	3.79	5.748	0	0.081	0	0.154
TRINITY_DN16634_c3_g9_i2	5.703	38.179	0	0.477	7.777	14.666
TRINITY_Contig7974	4.244	7.138	0.271	0.404	2.342	2.588
TRINITY_DN14304_c0_g3_i10	5.916	4.416	0	0.469	0	2.996
TRINITY_Contig510	9.258	21.015	0.543	0.186	0.401	2.057
TRINITY_39523_length_1021_cov_50.735294_g26020_i0	2747.323	4960.352	92.49	239.131	3594.422	3824.674
TRINITY_2609_length_3462_cov_30.345712_g1658_i0	8.569	9.852	0.308	0.251	0	0.909
TRINITY_DN20137_c1_g2_i1	0	0.152	139.657	34.158	0	2.08
TRINITY_BIN_11398_1	50.515	4.691	0	0	0	0.716
TRINITY_31910_length_1218_cov_28.061793_g20157_i1	0	2.954	431.727	78.826	23.131	12.394
TRINITY_DN18269_c1_g3_i2	13.195	8.919	0	0.453	0	0.208
TRINITY_Contig7673	51.599	20.436	0.579	5.037	1.723	0.578
TRINITY_BIN_15727_1	10.695	23.773	1.827	1.698	1.589	1.548
TRINITY_Contig13103	0.169	0.217	26.705	88.819	17.562	5.738
TRINITY_27499_length_1353_cov_29.495327_g17524_i0	891.816	793.719	55.128	173.628	46.613	155.37
TRINITY_Contig8093	78.063	40.756	0.633	7.066	19.702	8.696
TRINITY_DN19304_c2_g2_i1	4.882	7.608	0.416	0.752	0	2.288
TRINITY_BIN_6636_2	41.351	50.956	4.686	2.571	7.911	13.51
TRINITY_Contig6526	12.726	22.101	0.525	1.423	2.542	4.868
TRINITY_75569_length_473_cov_44.455446_g58569_i0	0.645	1.513	411.156	60.797	24.001	7.741
TRINITY_BIN_2441_5	49.928	47.908	6.007	13.267	15.237	26.382
TRINITY_Contig9585	96.939	261.699	14.547	40.254	57.334	255.389
TRINITY_BIN_2455_3	3.35	14.319	0	0.04	0	1.756
TRINITY_16286_length_1817_cov_27.038330_g10219_i0	7.001	6.949	0.362	0.752	3.479	3.743
TRINITY_DN17534_c0_g2_i6	0.301	0.369	8.775	8.23	16.424	19.287
TRINITY_DN21886_c1_g1_i4	6.575	7.065	0	0.348	0.903	1.448
TRINITY_Contig5428	21.977	24.077	0.76	0.978	0.217	4.806
TRINITY_Contig10478	0	0.68	28.406	30.908	86.972	99.002
TRINITY_DN7956_c0_g1_i3	40.567	77.784	4.831	7.721	0	7.079
TRINITY_DN18155_c1_g2_i3	8.144	7.985	0.796	1.795	0	2.087
TRINITY_17532_length_1753_cov_36.917458_g10813_i1	64.267	73.534	3.709	16.792	15.605	42.334
TRINITY_Contig9191	0.029	0.449	162.436	28.111	9.901	8.188
TRINITY_789_length_4703_cov_21.108114_g501_i0	5.307	6.168	0.326	0.154	0.435	0.57
TRINITY_DN22244_c1_g2_i13	0.117	0	142.643	130.941	0	0
TRINITY_37977_length_1058_cov_17.960566_g24867_i0	0.081	1.035	278.953	103	13.213	17.755
TRINITY_Contig17167	56.899	39.909	2.804	7.05	1.756	10.945
TRINITY_Contig881	4.648	5.654	0.253	0.833	0.268	2.58
TRINITY_BIN_13016_9	5.337	10.808	341.499	321.329	168.742	149.069
TRINITY_BIN_1943_8	13.598	10.345	0.308	1.496	2.342	4.106
TRINITY_Contig19061	59.656	36.166	1.665	0.849	0.368	0.501
TRINITY_BIN_25884_1	14.903	8.072	0	0.356	0	0.863
TRINITY_Contig9754	5.007	4.655	0.181	0.679	0.786	1.879
TRINITY_39367_length_1025_cov_29.965481_g25910_i0	25.085	34.103	3437.862	1706.806	511.193	889.323

TRINITY_28687_length_1316_cov_41.912590_g18334_i0	20.247	12.886	1.086	2.514	0.769	2.026
TRINITY_13314_length_1986_cov_28.293166_g8340_i0	4.516	7.275	0.326	0.251	0.184	2.55
TRINITY_DN19298_c0_g1_i3	0.953	3.439	802.012	3766.189	1190.726	418.946
TRINITY_BIN_7741_3	56.98	47.307	4.035	13.89	14.217	19.033
TRINITY_DN22137_c1_g1_i1	47.04	75.359	0.941	3.501	5.018	38.19
TRINITY_DN18492_c0_g1_i1	5.564	9.23	0.362	1.116	3.128	17.832
TRINITY_Contig11363	15.086	5.646	479.257	568.181	321.611	199.991
TRINITY_BIN_3323_5	0	0.347	125.075	165.204	15.237	5.738
TRINITY_4969_length_2868_cov_24.511969_g2709_i1	12.711	6.769	1.429	1.091	0.368	0.67
TRINITY_BIN_13589_2	0.147	0.181	62.112	57.822	18.415	53.117
TRINITY_Contig11958	4.926	7.21	0.145	0.8	2.358	0.108
TRINITY_11940_length_2078_cov_32.831757_g7461_i0	218.779	154.735	25.185	34.934	109.049	168.079
TRINITY_BIN_3276_1	8.613	6.559	0.416	0.962	2.342	2.195
TRINITY_46853_length_870_cov_22.659176_g31771_i0	65.689	30.99	1.393	0.356	7.878	41.355
TRINITY_11479_length_2112_cov_30.728830_g7176_i0	7.316	4.01	0.47	0.631	0	0.693
TRINITY_Contig17616	29.388	56.921	4.053	7.082	11.005	10.984
TRINITY_65434_length_577_cov_18.037402_g48598_i0	5.366	4.734	0	0	0	1.802
TRINITY_Contig14114	38.947	19.35	0.434	2.636	2.96	4.391
TRINITY_Contig9017	6.319	7.608	1.104	1.132	3.362	1.618
TRINITY_DN9347_c0_g1_i2	3.152	8.716	0	0	0	0
TRINITY_BIN_750_1	84.081	18.655	0	3.687	6.724	1.941
TRINITY_8742_length_2349_cov_23.646491_g5523_i0	0	0.246	124.912	20.422	7.008	5.045
TRINITY_14818_length_1896_cov_27.142310_g9295_i0	6.429	7.319	0.706	1.334	1.555	3.782
TRINITY_DN22186_c0_g1_i1	0	0	57.1	31.636	0	0.401
TRINITY_BIN_630_12	50.331	6.826	0	0	0	0
TRINITY_DN22191_c2_g1_i2	0	0	4.342	6.282	32.899	43.142
TRINITY_Contig9052	16.523	18.655	2525.81	1591.016	784.351	131.261
TRINITY_BIN_8421_4	16.2	6.942	0.543	0.55	0.602	0
TRINITY_51107_length_791_cov_15.894737_g35348_i0	22.615	17.815	0.525	1.779	2.425	0
TRINITY_DN19298_c0_g3_i1	0	0.224	71.611	97.898	12.511	2.919
TRINITY_77296_length_458_cov_24.843188_g60275_i0	6.29	5.487	78.052	20.624	0	0.362
TRINITY_46128_length_883_cov_38.030713_g31182_i0	0.828	0.673	443.651	145.251	6.305	0.701
TRINITY_DN15296_c0_g1_i5	21.376	72.144	2.316	6.597	8.864	10.229
TRINITY_DN22110_c0_g3_i7	23.267	2.288	0	0	0	10.391
TRINITY_860_length_4595_cov_28.621741_g550_i0	5.227	6.776	0.724	1.229	4.031	5.338
TRINITY_DN11840_c0_g1_i1	3.717	7.116	0	0.259	0	0.793
TRINITY_Contig2572	0.088	0.333	135.008	35.775	8.597	2.58
TRINITY_BIN_1709_4	13.884	8.122	0.253	1.795	1.104	4.321
TRINITY_DN18777_c1_g3_i2	1.224	3.518	516.636	80.662	27.781	9.135
TRINITY_DN18452_c0_g1_i6	9.061	4.886	0.47	0	0	2.003
TRINITY_64857_length_584_cov_26.221359_g48038_i0	177.479	250.044	21.621	30.803	11.975	25.01
TRINITY_DN13272_c0_g1_i12	0	0.543	24.226	34.328	37.364	31.434
TRINITY_DN18660_c1_g2_i14	13.972	6.892	0.688	1.415	1.84	2.58
TRINITY_DN21795_c0_g1_i1	0	0	62.221	21.95	21.057	22.445
TRINITY_BIN_4326_1	6.143	6.001	0	0.194	0	0
TRINITY_49153_length_826_cov_25.743725_g33679_i0	14.207	6.305	0	0.768	0	0
TRINITY_Contig16356	0.037	0.13	83.281	67.977	3.011	1.664
TRINITY_Contig19379	45.222	55.039	2.044	1.059	0	0
TRINITY_6426_length_2624_cov_32.417613_g4064_i0	2.595	3.381	666.57	140.19	37.331	16.907
TRINITY_DN16050_c0_g3_i1	9.258	17.381	1.556	1.423	1.388	5.376
TRINITY_Contig12983	11.245	13.117	0	0.412	2.074	0.632
TRINITY_DN15775_c0_g3_i1	237.758	287.492	31.97	46.051	130.708	498.068
TRINITY_BIN_7705_7	31.99	8.962	1.556	1.18	0	0.709
TRINITY_74234_length_485_cov_34.216346_g57241_i0	2.141	1.484	155.289	31.296	1.455	0.639
TRINITY_DN21450_c0_g9_i1	15.775	9.143	0.887	1.358	5.068	2.912
TRINITY_DN13464_c0_g1_i4	3.724	5.357	136.527	65.058	108.531	77.204
TRINITY_53107_length_756_cov_28.545852_g37076_i0	78.818	102.281	3.98	15.652	4.533	11.646
TRINITY_DN17266_c1_g2_i7	24.066	31.063	1.773	5.595	5.185	4.976
TRINITY_6808_length_2574_cov_27.122954_g4287_i0	10.856	14.898	1.176	0.736	3.78	7.988
TRINITY_21725_length_1565_cov_29.952540_g13703_i0	18.158	10.576	1.049	0.307	0.853	4.768
TRINITY_DN20418_c0_g1_i7	17.212	10.583	0.47	0.97	1.02	2.195
TRINITY_12044_length_2070_cov_32.029985_g6712_i1	3.445	7.427	0	0.049	0.385	0.67
TRINITY_17846_length_1738_cov_29.339125_g11206_i0	13.642	11.517	1.628	0.566	0	0.947

TRINITY_BIN_2817_1	0	0.594	73.999	111.327	43.77	3.536
TRINITY_Contig17915	79.324	54.908	5.12	16.776	26.158	52.902
TRINITY_6905_length_2559_cov_28.179518_g4348_i0	4.12	5.907	0.127	0.655	0.669	2.542
TRINITY_BIN_97_5	8.283	8.622	0.525	1.318	4.499	13.865
TRINITY_Contig13133	6.018	5.531	0	0.841	1.154	2.234
TRINITY_39052_length_1033_cov_27.057054_g25669_i0	1.547	1.498	154.168	112.734	89.397	66.535
TRINITY_7917_length_2435_cov_29.959425_g5008_i0	7.389	13.095	1.447	1.18	0.284	4.799
TRINITY_Contig116	25.627	23.295	0.615	5.085	4.466	8.789
TRINITY_BIN_1436_7	15.841	31.461	1.357	0.55	2.007	13.857
TRINITY_BIN_8105_8	4.684	8.868	0.362	0.364	2.04	5.823
TRINITY_Contig1134	205.526	128.471	13.823	44.304	64.61	101.536
TRINITY_DN18322_c0_g3_i1	6.7	3.25	0.253	0.582	0.552	0.316
TRINITY_DN19913_c0_g1_i7	17.329	1.918	0.163	0	0	0
TRINITY_DN17322_c1_g2_i6	0.073	0.927	150.857	246.391	12.343	9.259
TRINITY_DN21756_c2_g1_i7	12.257	7.695	0	0.986	2.893	2.372
TRINITY_DN22080_c2_g8_i1	23.054	24.077	0	0.469	0	0.524
TRINITY_Contig10122	13.261	16.802	0.434	1.965	0	1.202
TRINITY_BIN_3347_9	3.768	4.814	0	0.598	1.438	3.566
TRINITY_BIN_15544_2	4.376	25.909	0.561	0.073	0	4.498
TRINITY_DN15946_c1_g2_i4	0.059	0	68.318	24.222	10.788	7.264
TRINITY_DN15464_c2_g1_i5	0	0	265.094	314.877	81.686	107.937
TRINITY_DN21825_c1_g1_i6	4.53	6.87	0	0.081	0	0.886
TRINITY_Contig9385	155.656	109.94	19.196	29.598	64.51	88.773
TRINITY_DN20951_c1_g4_i1	0.433	0.492	103.327	29.024	4.783	2.295
TRINITY_12500_length_2039_cov_25.783249_g7812_i0	3.137	9.056	0	0.226	0	4.521
TRINITY_Contig7533	4.054	3.656	236.453	283.355	81.82	146.612
TRINITY_Contig15369	32.789	16.918	1.683	2.393	4.934	0.855
TRINITY_BIN_13043_9	43.785	25.988	4.306	3.937	2.827	6.401
TRINITY_Contig8669	18.282	17.497	1.502	4.487	2.124	5.785
TRINITY_63561_length_600_cov_29.361582_g46790_i0	6.356	7.84	0	0.711	0	3.821
TRINITY_Contig9647	5.505	7.246	1124.586	646.53	182.774	275.046
TRINITY_DN21756_c2_g1_i15	20.247	7.319	1.14	1.253	2.325	3.066
TRINITY_44878_length_907_cov_25.770883_g30171_i0	0	0.637	35.299	4.156	26.342	39.237
TRINITY_22226_length_1542_cov_31.294637_g2198_i1	38.163	25.054	0.887	2.151	0.284	0.539
TRINITY_Contig493	51.196	33.589	6.188	9.37	7.677	5.839
TRINITY_DN19996_c0_g1_i2	7.301	11.763	0.163	1.302	3.529	17.115
TRINITY_Contig11950	0.154	3.106	245.807	174.679	467.389	0.562
TRINITY_Contig6991	79.287	56.001	9.137	16.897	18.197	20.712
TRINITY_DN21909_c0_g2_i10	184.209	96.576	0.308	4.479	0.686	0.154
TRINITY_BIN_2887_1	38.111	63.964	0.163	1.787	0.385	0.493
TRINITY_DN21725_c0_g1_i15	0	1.122	396.103	43.795	0	6.532
TRINITY_Contig20011	41.08	85.5	9.969	11.351	16.09	11.2
TRINITY_Contig15543	5.183	9.997	0.181	0	0	2.858
TRINITY_BIN_6778_3	49.569	73.028	5.482	11.691	12.226	41.224
TRINITY_Contig19467	34.431	6.544	0.507	1.294	2.827	3.289
TRINITY_49700_length_816_cov_33.551539_g34146_i0	2.896	1.346	88.509	42.445	1.154	1.926
TRINITY_DN17265_c0_g3_i1	1.928	2.896	302.654	211.966	43.218	28.43
TRINITY_18953_length_1685_cov_24.115099_g1094_i2	0.396	0	7.165	35.5	25.673	17.593
TRINITY_BIN_2666_4	5.732	13.182	0	0.768	3.128	4.113
TRINITY_36487_length_1094_cov_29.361951_g10636_i1	67.932	45.157	5.102	15.07	13.932	38.274
TRINITY_16231_length_1820_cov_32.281553_g6995_i1	71.876	43.405	6.061	6.88	3.379	26.636
TRINITY_Contig18731	13.166	19.321	1.447	2.749	2.275	3.505
TRINITY_Contig18398	6.927	12.031	0.76	0.711	0.401	2.01
TRINITY_7703_length_2464_cov_22.626722_g4886_i0	4.398	5.357	0	0.695	0.284	0.585
TRINITY_Contig3367	0	0	60.864	37.748	3.445	4.013
TRINITY_DN17564_c0_g1_i12	11.311	13.088	0.416	1.536	1.539	2.003
TRINITY_Contig17457	175.39	73.925	2.099	11.561	5.302	16.445
TRINITY_27036_length_1369_cov_21.286923_g17220_i0	18.876	39.684	0.507	5.732	2.208	8.85
TRINITY_Contig16886	9.603	14.818	0.959	1.714	3.579	7.549
TRINITY_DN13918_c0_g8_i1	2.104	3.75	461.924	197.931	257.955	160.03
TRINITY_DN8691_c0_g1_i2	73.254	64.377	3.347	15.329	9.132	14.935
TRINITY_Contig898	0.088	1.433	220.17	65.753	10.219	6.909
TRINITY_DN18037_c0_g1_i1	0.029	0.152	26.922	9.75	18.95	22.954

TRINITY_32198_length_1210_cov_30.662577_g20743_i0	10.871	5.118	0.597	0.154	1.004	0
TRINITY_BIN_15641_2	220.495	137.817	5.41	35.896	86.57	80.916
TRINITY_Contig14673	254.376	215.058	7.49	10.631	9.065	5.03
TRINITY_15397_length_1864_cov_26.900836_g9656_i0	189.428	86.13	17.079	15.798	18.063	7.325
TRINITY_4588_length_2938_cov_31.908330_g1232_i1	16.736	16.143	1.031	3.873	4.499	20.004
TRINITY_2046_length_3684_cov_29.339419_g1306_i0	8.342	9.606	0.434	1.698	1.187	2.75
TRINITY_BIN_198_5	7.998	3.735	0.362	0.428	1.589	0.755
TRINITY_19795_length_1647_cov_30.508872_g10193_i1	7.309	16.65	1.013	1.14	8.614	23.786
TRINITY_BIN_727_2	54.832	35.862	2.75	10.906	4.616	40.847
TRINITY_DN18234_c2_g1_i6	8.833	13.001	1.013	0.526	0.485	4.776
TRINITY_DN17417_c1_g3_i2	56.746	49.768	8.359	13.857	15.153	33.907
TRINITY_Contig2917	24.257	21.978	2.768	3.436	5.954	9.135
TRINITY_BIN_18663_3	15.094	22.419	0	0.121	1.054	3.374
TRINITY_BIN_478_8	96.14	121.348	2.28	23.203	25.79	25.896
TRINITY_BIN_18355_1	12.55	4.785	130.611	74.461	2.71	4.021
TRINITY_Contig15483	12.161	9.628	1.429	1.568	9.048	6.57
TRINITY_Contig17611	92.452	24.461	1.411	8.909	10.805	7.703
TRINITY_BIN_968_2	32.386	33.973	374.283	119.614	11.808	10.337
TRINITY_3865_length_3090_cov_23.962264_g2463_i0	2.529	7.97	0.253	0.154	2.509	0.1
TRINITY_58582_length_668_cov_30.041736_g42051_i0	0.33	0.203	271.824	340.069	54.474	78.906
TRINITY_DN22582_c1_g2_i2	0	0	114.87	22.322	1.121	0.585
TRINITY_Contig17091	22.959	21.703	1.646	1.9	0.234	3.035
TRINITY_42067_length_963_cov_25.493289_g27976_i0	10.629	14.5	0.814	1.859	0.903	2.434
TRINITY_BIN_18860_2	1.979	5.111	1043.043	152.681	111.458	23.817
TRINITY_9507_length_2277_cov_42.851449_g5975_i0	47.179	43.717	6.513	10.171	13.096	9.89
TRINITY_Contig16764	5.095	5.313	0.706	0.485	0.318	2.265
TRINITY_DN15842_c0_g1_i2	0.359	0.514	153.733	69.957	7.577	6.27
TRINITY_22103_length_1548_cov_28.137255_g13972_i0	0.323	0.869	177.489	33.705	8.831	1.718
TRINITY_DN12427_c0_g2_i2	6.832	9.896	0	0.566	0	0
TRINITY_BIN_14328_1	24.777	52.389	2.424	0.461	0	3.551
TRINITY_Contig16403	4.977	7.891	0.398	0.881	0.151	0.454
TRINITY_27734_length_1346_cov_26.772122_g17673_i0	0.125	0.152	37.995	71.008	5.285	3.474
TRINITY_DN19315_c1_g1_i4	0	0	160.41	63.142	13.681	14.681
TRINITY_16911_length_1783_cov_23.957410_g5241_i1	4.193	7.702	0	0	0	0.37
TRINITY_19238_length_1673_cov_21.231920_g12106_i0	0.938	14.21	0	0	2.024	0
TRINITY_48787_length_833_cov_20.123037_g33371_i0	10.915	4.271	0.489	0	4.048	7.818
TRINITY_DN15708_c0_g1_i1	1.283	10.75	1290.044	691.595	170.632	123.042
TRINITY_BIN_15770_1	0.85	9.664	2736.734	565.788	171.468	58.925
TRINITY_DN20488_c1_g1_i7	26.551	36.412	3.456	3.929	7.643	12.632
TRINITY_Contig18732	25.151	19.849	247.562	66.796	5.887	6.247
TRINITY_DN21458_c1_g1_i1	20.54	16.664	1.628	4.495	2.944	4.945
TRINITY_DN19004_c0_g1_i8	6.722	5.321	0	0	0	0
TRINITY_DN19057_c0_g5_i4	37.979	22.405	1.321	6.419	0	0.986
TRINITY_Contig19436	231.952	119.966	13.298	40.877	55.244	89.805
TRINITY_Contig17465	8.188	18.626	1.393	0.501	0.719	3.066
TRINITY_1987_length_3713_cov_30.967069_g1272_i0	7.631	22.369	0.09	0	0.284	2.919

Supplementary table 1. *In silico* expression matrix for DEGs in Root (control vs salt, control vs drought) and Leaves (control vs drought, control vs salt) and the UniProt Ids for the Unigenes.

Unigene ID	CONTROL1	CONTROL2	PEG_TREATED _LEAVES1	PEG_TREATED _LEAVES2	SALT_TREATED _LEAVES1	SALT_TREATED_ LEAVES2
TRINITY_25469_length_1421_cov_26.433432_g16200_i0	6.693	6.247	0.253	1.188	0.552	1.41
TRINITY_57587_length_683_cov_25.188925_g41125_i0	2.39	2.164	365.056	205.547	13.614	35.047
TRINITY_50925_length_794_cov_25.200000_g35189_i0	159.893	74.439	8.467	2.991	1.806	4.537
TRINITY_1325_length_4122_cov_24.586973_g857_i0	4.435	6.486	0.362	0.097	0	2.203
TRINITY_Contig7003	5.08	17.272	0.145	0	0.167	1.386
TRINITY_9543_length_2274_cov_27.739229_g5996_i0	29.579	25.858	0.941	6.904	1.873	8.265
TRINITY_4828_length_2893_cov_30.035765_g3070_i0	5.842	8.586	0	1.035	0.284	1.841
TRINITY_DN22323_c2_g1_i3	14.91	9.896	1.194	0.639	0	0.886
TRINITY_BIN_3238_3	1.29	2.802	237.231	118.7	28.416	10.268
TRINITY_DN21081_c2_g2_i3	9.786	8.6	0	0.137	0	0.531
TRINITY_37113_length_1078_cov_30.117939_g23146_i1	0.909	2.649	342.621	1610.565	623.754	237.857
TRINITY_BIN_3718_1	0	0.456	142.281	33.01	10.771	6.955
TRINITY_BIN_16563_3	3.841	5.929	0	0	0	0
TRINITY_Contig13523	1.488	7.76	764.976	379.361	329.271	701.833
TRINITY_BIN_866_7	2.639	5.502	956.379	594.165	264.528	43.697
TRINITY_BIN_20507_2	8.408	17.62	0	1.019	0	0.254
TRINITY_1996_length_3708_cov_30.695796_g1277_i0	8.973	13.342	1.321	1.633	1.472	1.61
TRINITY_Contig9143	7.55	7.087	0	1.051	0.401	1.471
TRINITY_BIN_1717_7	0.337	0.478	442.221	379.539	80.332	137.415
TRINITY_BIN_9764_3	49.913	93.818	2.841	13.704	3.83	25.796
TRINITY_23221_length_1502_cov_27.458479_g14712_i0	5.102	12.668	0.271	0.986	0	1.148
TRINITY_17925_length_1734_cov_24.494895_g11252_i0	5.718	6.03	0.199	0.897	0	0.239
TRINITY_BIN_1602_10	9.896	6.899	0.832	1.091	0.702	1.171
TRINITY_Contig8909	2.94	12.285	0.47	0.105	0	0.185
TRINITY_DN20690_c3_g1_i1	127.61	55.328	7.418	8.562	10.219	11.215
TRINITY_BIN_6569_1	3.137	16.368	729.37	10913.893	646.4	188.306
TRINITY_1533_length_3973_cov_27.862705_g941_i1	24.095	10.316	0	1.407	1.305	2.842
TRINITY_9683_length_2260_cov_41.896394_g6079_i0	0.198	0.398	81.598	48.792	15.655	14.389
TRINITY_DN22307_c0_g3_i1	5.359	5.263	0	0	0	0.439
TRINITY_23371_length_1496_cov_27.297127_g14812_i0	3.489	8.738	0.561	0.34	0	0.285
TRINITY_DN15746_c0_g1_i4	4.999	7.232	0	0.146	0.318	1.34
TRINITY_Contig10698	6.553	7.087	0	0.808	0.502	0.747
TRINITY_Contig1009	88.604	18.93	0.543	4.39	1.806	1.086
TRINITY_Contig13120	37.122	32.424	4.451	7.721	4.633	6.709
TRINITY_33458_length_1176_cov_12.514905_g21625_i0	6.444	4.025	0.308	0.639	0	0.308
TRINITY_DN21509_c2_g3_i2	0.125	2.28	203.868	164.614	68.975	108.268
TRINITY_DN14317_c1_g1_i1	122.669	51.47	0.326	1.488	1.104	5.091
TRINITY_40499_length_1000_cov_22.002148_g26779_i0	10.849	21.905	0	0.129	0	6.432
TRINITY_DN13872_c0_g1_i3	126.935	93.608	1.086	10.033	5.268	14.866
TRINITY_35304_length_1125_cov_27.677083_g22970_i0	1.422	2.744	394.095	169.052	140.259	234.738
TRINITY_19576_length_1657_cov_44.516373_g12312_i0	14.009	10.497	1.086	2.466	0	5.107
TRINITY_36620_length_1091_cov_31.309198_g23902_i0	0.242	0.883	194.569	250.296	18.063	13.287
TRINITY_DN11567_c0_g1_i1	0.095	1.404	277.867	77.808	15.371	32.382
TRINITY_DN18728_c0_g5_i4	7.58	14.768	129.67	154.249	225.14	504.576
TRINITY_16880_length_1784_cov_31.650729_g10598_i0	8.071	5.574	0.38	0.784	0.636	0.47
TRINITY_4092_length_3037_cov_27.128369_g2604_i0	10.255	5.943	0.416	0.922	0.351	1.641
TRINITY_Contig15930	111.365	61.112	5.355	9.322	4.114	5.831
TRINITY_DN17626_c0_g2_i1	12.77	22.065	1.791	1.18	0	4.991
TRINITY_BIN_18639_1	0.073	0.55	3.166	38.492	52.518	58.517
TRINITY_55328_length_720_cov_22.572965_g39043_i0	13.451	7.731	0.615	0	0	1.702
TRINITY_BIN_11398_2	95.868	59.505	3.564	5.15	7.744	17.462
TRINITY_51168_length_790_cov_19.793343_g35401_i0	2.661	17.54	0	0	1.823	2.688
TRINITY_DN13944_c0_g1_i9	537.605	217.432	14.8	52.761	6.707	8.858
TRINITY_BIN_8248_1	12.411	8.875	0.651	1.431	0.585	0.909

TRINITY_Contig3439	16.281	20.696	0.995	0.986	0.134	0.154
TRINITY_Contig12714	5.652	16.063	0.217	0	1.606	1.918
TRINITY_DN15786_c2_g2_i2	16.208	14.138	1.447	3.274	0.652	1.094
TRINITY_5027_length_2855_cov_19.676956_g1942_i1	13.268	5.979	0.507	0.711	0.619	0.408
TRINITY_6483_length_2616_cov_33.980762_g4098_i0	20.65	20.943	0.543	4.358	1.037	7.256
TRINITY_BIN_12007_1	1.114	4.814	667.312	344.75	34.237	23.747
TRINITY_Contig11942	82.417	72.173	3.6	3.525	3.596	3.697
TRINITY_DN18700_c0_g1_i10	0	0.101	25.239	23.43	33.551	45.33
TRINITY_17597_length_1750_cov_27.232005_g11053_i0	0.044	0.391	112.41	28.766	7.058	13.487
TRINITY_BIN_19022_2	3.211	8.643	0	0.566	0.418	1.587
TRINITY_BIN_4317_1	0.953	0.825	37.597	33.495	34.003	49.197
TRINITY_Contig9689	0	1.122	540.103	32.646	54.826	31.989
TRINITY_DN13722_c5_g5_i2	47.956	29.087	1.013	5.853	0.418	4.991
TRINITY_BIN_17558_5	5.813	19.871	0	0	0	0.339
TRINITY_Contig17597	0.169	0.601	78.595	53.02	24.084	75.994
TRINITY_BIN_2745_2	90.972	109.013	8.757	26.542	9.149	37.974
TRINITY_DN19004_c0_g1_i1	529.82	457.074	0	0.21	1.405	0.308
TRINITY_Contig6883	0.117	1.651	126.902	88.366	53.856	70.579
TRINITY_BIN_14597_1	2.595	2.44	1329.178	549.804	68.691	26.482
TRINITY_Contig9243	1.158	1.318	206.257	63.239	20.154	26.913
TRINITY_BIN_4564_7	2.382	3.207	90.192	312.169	132.766	85.399
TRINITY_10521_length_2190_cov_26.274399_g2377_i1	0	0.253	105.824	20.268	5.319	3.274
TRINITY_Contig6397	0.367	1.274	130.919	41.523	9.901	3.736
TRINITY_DN16486_c0_g3_i3	146.09	65.151	0.959	6.727	16.826	20.304
TRINITY_DN16117_c5_g2_i4	0.154	0.434	180.33	25.944	13.113	23.855
TRINITY_DN17534_c0_g2_i11	0.44	0.695	37.235	19.541	27.597	33.368
TRINITY_BIN_15103_2	0.433	0.514	161.043	48.695	11.724	14.096
TRINITY_4942_length_2873_cov_26.481455_g3145_i0	2.962	7.652	0.326	0.331	0.853	1.101
TRINITY_DN18037_c0_g1_i23	0	0.471	13.877	3.25	21.191	19.11
TRINITY_Contig10204	27.262	11.192	0.615	0.323	0	0
TRINITY_DN16557_c0_g2_i2	4.45	7.485	0	0.243	0	0.239
TRINITY_DN16742_c2_g3_i2	32.086	25.728	0	0	144.022	64.702
TRINITY_Contig20107	0.447	2.027	174.938	62.107	31.042	36.233
TRINITY_9820_length_2247_cov_25.817723_g6171_i0	1.224	1.006	387.039	145.598	18.298	5.839
TRINITY_Contig3189	0.176	1.086	630.62	691.231	111.976	253.255
TRINITY_BIN_1703_7	2350.625	1916.539	160.952	486.638	249.375	503.845
TRINITY_BIN_11843_2	24.139	19.821	1.466	4.188	3.295	1.04
TRINITY_36657_length_1090_cov_26.702253_g1902_i2	0.777	2.497	145.501	56.359	71.517	74.176
TRINITY_Contig20078	13.166	13.038	1.701	3.145	1.254	3.767
TRINITY_13929_length_1946_cov_17.416622_g8719_i0	0.117	0.565	122.035	294.124	57.569	6.686
TRINITY_DN21167_c0_g2_i1	20.459	9.425	0	1.091	1.522	2.449
TRINITY_Contig1252	13.642	17.164	0.615	1.027	0.351	2.935
TRINITY_8846_length_2340_cov_24.682078_g5586_i0	5.241	4.575	0.543	0.21	0	1.433
TRINITY_BIN_8243_7	48.667	41.936	0	3.129	4.967	13.695
TRINITY_8636_length_2358_cov_28.701616_g5459_i0	12.711	9.881	0.145	0.17	0.151	0.37
TRINITY_5300_length_2804_cov_28.563803_g3365_i0	16.977	21.898	0	1.019	0.251	5.407
TRINITY_BIN_2309_14	0.535	0.637	287.692	111.562	11.524	7.533
TRINITY_Contig10665	88.663	71.732	6.26	14.561	2.559	4.837
TRINITY_BIN_3323_2	0.066	0.195	63.994	143.027	10.035	6.085
TRINITY_8847_length_2340_cov_23.405108_g5587_i0	0	0.608	390.494	53.012	16.608	5.207
TRINITY_Contig360	7.243	4.865	0.308	1.003	0.167	0.67
TRINITY_DN19356_c0_g2_i2	3.445	7.471	1452.824	272.917	37.18	26.975
TRINITY_53_length_8517_cov_24.605587_g29_i0	96.367	37.585	1.845	10.243	1.873	1.895
TRINITY_BIN_9927_1	69.948	36.391	8.594	4.77	1.422	2.403
TRINITY_1451_length_4022_cov_28.028586_g941_i0	10.703	4.799	0.452	0.752	0.301	0.77
TRINITY_29071_length_1305_cov_29.024272_g18592_i0	6.246	3.793	0	0.631	0.92	1.086
TRINITY_21197_length_1586_cov_38.420567_g13360_i0	1.635	5.965	14.637	33.649	106.406	82.657
TRINITY_BIN_19978_1	2.793	6.805	0	0.129	0	0.139
TRINITY_47946_length_849_cov_17.776923_g32684_i0	2.617	6.761	0	0.121	1.087	0.847
TRINITY_DN16989_c1_g1_i5	7.287	14.666	0.235	0.065	0	0.755
TRINITY_16708_length_1793_cov_22.377030_g10491_i0	17.417	16.932	1.176	0.259	0	0.046
TRINITY_46902_length_869_cov_27.276250_g31811_i0	8.1	4.988	0	0.72	1.054	1.756
TRINITY_57881_length_678_cov_26.144499_g41397_i0	0.806	1.593	470.898	104.019	83.192	94.943

TRINITY_DN14161_c0_g2_i3	2.485	3.953	768.016	284.883	540.914	25.357
TRINITY_BIN_20189_1	0	0.999	184.455	451.453	41.579	7.079
TRINITY_DN19722_c1_g1_i7	3.643	6.262	31.825	32.598	100.034	86.231
TRINITY_5616_length_2753_cov_27.887481_g3547_i0	8.782	8.231	0.579	1.447	0.585	3.22
TRINITY_DN20137_c1_g2_i3	2.031	1.194	299.687	134.175	6.657	8.434
TRINITY_Contig16066	6.605	5.009	0.199	0.323	0.201	0.763
TRINITY_33143_length_1184_cov_24.504036_g21408_i0	8.152	2.881	0	0.307	0	0.193
TRINITY_Contig17996	10.527	11.554	0	1.294	0.569	3.058
TRINITY_DN11273_c0_g1_i2	7.411	6.602	0.09	0.606	1.555	1.517
TRINITY_BIN_2575_1	6.971	5.104	0	0.8	0.903	0.994
TRINITY_21652_length_1568_cov_26.823883_g13659_i0	67.06	68.995	0.868	0.275	0	0.216
TRINITY_26921_length_1373_cov_23.520706_g17146_i0	46.901	23.78	0	0.323	0.569	0.062
TRINITY_BIN_3951_27	2.954	1.477	52.65	62.293	59.124	57.462
TRINITY_DN18305_c2_g2_i2	59.912	43.948	3.908	5.328	3.847	10.221
TRINITY_60536_length_641_cov_19.125874_g43899_i0	9.405	11.792	0	0.574	0	0.377
TRINITY_BIN_3654_11	25.693	17.642	0	2.054	4.114	2.85
TRINITY_Contig816	13.965	15.13	0.525	2.142	1.455	2.334
TRINITY_BIN_5381_2	56.665	61.329	9.824	11.48	4.198	15.968
TRINITY_DN16037_c0_g1_i3	131.693	185.421	5.247	21.093	3.864	47.433
TRINITY_DN11762_c0_g1_i1	60.785	53.243	6.767	5.029	4.7	4.999
TRINITY_BIN_2956_3	28.838	17.627	0.742	1.908	1.238	8.966
TRINITY_52272_length_771_cov_21.150997_g36351_i0	7.748	4.872	0	0	0	0.555
TRINITY_Contig1421	1.833	19.618	0	0	0	0
TRINITY_DN16940_c0_g4_i2	176.761	423	25.547	42.971	37.766	29.794
TRINITY_87124_length_381_cov_35.833333_g70056_i0	23.194	16.52	0	0	0	0
TRINITY_DN20690_c3_g1_i2	130.732	61.612	2.171	8.651	25.924	7.402
TRINITY_Contig8236	1.254	5.111	756.907	185.852	15.237	12.008
TRINITY_Contig12895	26.346	24.779	1.719	2.87	3.579	5.685
TRINITY_DN16486_c0_g3_i4	19.704	17.801	0.669	2.207	3.027	3.358
TRINITY_7372_length_2502_cov_32.012741_g4658_i0	2.404	25.8	0.253	0.356	0.134	0.347
TRINITY_51831_length_778_cov_27.468265_g35969_i0	1.554	0.319	239.999	369.627	128.283	22.854
TRINITY_Contig10267	22.277	23.541	1.176	5.247	2.375	7.834
TRINITY_DN18272_c1_g1_i1	15.247	6.168	0	0.841	0.401	2.103
TRINITY_5299_length_2804_cov_29.469835_g3364_i0	8.247	22.513	0	0.032	0	0
TRINITY_Contig1225	9.42	15.687	0	1.221	1.154	2.989
TRINITY_Contig8430	11.897	11.879	0.905	1.221	0	0.393
TRINITY_DN18700_c0_g1_i3	0	0.101	10.476	14.658	16.19	26.62
TRINITY_DN16990_c4_g1_i6	3.79	5.748	0	0.081	0	0.154
TRINITY_Contig510	9.258	21.015	0.543	0.186	0.401	2.057
TRINITY_2609_length_3462_cov_30.345712_g1658_i0	8.569	9.852	0.308	0.251	0	0.909
TRINITY_BIN_11398_1	50.515	4.691	0	0	0	0.716
TRINITY_31910_length_1218_cov_28.061793_g20157_i1	0	2.954	431.727	78.826	23.131	12.394
TRINITY_DN18269_c1_g3_i2	13.195	8.919	0	0.453	0	0.208
TRINITY_Contig7673	51.599	20.436	0.579	5.037	1.723	0.578
TRINITY_BIN_15727_1	10.695	23.773	1.827	1.698	1.589	1.548
TRINITY_Contig13103	0.169	0.217	26.705	88.819	17.562	5.738
TRINITY_27499_length_1353_cov_29.495327_g17524_i0	891.816	793.719	55.128	173.628	46.613	155.37
TRINITY_DN19304_c2_g2_i1	4.882	7.608	0.416	0.752	0	2.288
TRINITY_BIN_6636_2	41.351	50.956	4.686	2.571	7.911	13.51
TRINITY_Contig6526	12.726	22.101	0.525	1.423	2.542	4.868
TRINITY_BIN_2455_3	3.35	14.319	0	0.04	0	1.756
TRINITY_DN17534_c0_g2_i6	0.301	0.369	8.775	8.23	16.424	19.287
TRINITY_DN21886_c1_g1_i4	6.575	7.065	0	0.348	0.903	1.448
TRINITY_Contig5428	21.977	24.077	0.76	0.978	0.217	4.806
TRINITY_Contig10478	0	0.68	28.406	30.908	86.972	99.002
TRINITY_DN7956_c0_g1_i3	40.567	77.784	4.831	7.721	0	7.079
TRINITY_DN18155_c1_g2_i3	8.144	7.985	0.796	1.795	0	2.087
TRINITY_Contig9191	0.029	0.449	162.436	28.111	9.901	8.188
TRINITY_789_length_4703_cov_21.108114_g501_i0	5.307	6.168	0.326	0.154	0.435	0.57
TRINITY_37977_length_1058_cov_17.960566_g24867_i0	0.081	1.035	278.953	103	13.213	17.755
TRINITY_Contig17167	56.899	39.909	2.804	7.05	1.756	10.945
TRINITY_BIN_13016_9	5.337	10.808	341.499	321.329	168.742	149.069
TRINITY_Contig19061	59.656	36.166	1.665	0.849	0.368	0.501

TRINITY_BIN_25884_1	14.903	8.072	0	0.356	0	0.863
TRINITY_39367_length_1025_cov_29.965481_g25910_i0	25.085	34.103	3437.862	1706.806	511.193	889.323
TRINITY_28687_length_1316_cov_41.912590_g18334_i0	20.247	12.886	1.086	2.514	0.769	2.026
TRINITY_DN19298_c0_g1_i3	0.953	3.439	802.012	3766.189	1190.726	418.946
TRINITY_Contig11363	15.086	5.646	479.257	568.181	321.611	199.991
TRINITY_BIN_3323_5	0	0.347	125.075	165.204	15.237	5.738
TRINITY_4969_length_2868_cov_24.511969_g2709_i1	12.711	6.769	1.429	1.091	0.368	0.67
TRINITY_BIN_13589_2	0.147	0.181	62.112	57.822	18.415	53.117
TRINITY_Contig11958	4.926	7.21	0.145	0.8	2.358	0.108
TRINITY_11479_length_2112_cov_30.728830_g7176_i0	7.316	4.01	0.47	0.631	0	0.693
TRINITY_Contig14114	38.947	19.35	0.434	2.636	2.96	4.391
TRINITY_DN9347_c0_g1_i2	3.152	8.716	0	0	0	0
TRINITY_BIN_750_1	84.081	18.655	0	3.687	6.724	1.941
TRINITY_8742_length_2349_cov_23.646491_g5523_i0	0	0.246	124.912	20.422	7.008	5.045
TRINITY_BIN_630_12	50.331	6.826	0	0	0	0
TRINITY_DN22191_c2_g1_i2	0	0	4.342	6.282	32.899	43.142
TRINITY_Contig9052	16.523	18.655	2525.81	1591.016	784.351	131.261
TRINITY_BIN_8421_4	16.2	6.942	0.543	0.55	0.602	0
TRINITY_51107_length_791_cov_15.894737_g35348_i0	22.615	17.815	0.525	1.779	2.425	0
TRINITY_DN19298_c0_g3_i1	0	0.224	71.611	97.898	12.511	2.919
TRINITY_DN15296_c0_g1_i5	21.376	72.144	2.316	6.597	8.864	10.229
TRINITY_DN11840_c0_g1_i1	3.717	7.116	0	0.259	0	0.793
TRINITY_DN18777_c1_g3_i2	1.224	3.518	516.636	80.662	27.781	9.135
TRINITY_DN18452_c0_g1_i6	9.061	4.886	0.47	0	0	2.003
TRINITY_64857_length_584_cov_26.221359_g48038_i0	177.479	250.044	21.621	30.803	11.975	25.01
TRINITY_DN13272_c0_g1_i12	0	0.543	24.226	34.328	37.364	31.434
TRINITY_DN18660_c1_g2_i14	13.972	6.892	0.688	1.415	1.84	2.58
TRINITY_DN21795_c0_g1_i1	0	0	62.221	21.95	21.057	22.445
TRINITY_BIN_4326_1	6.143	6.001	0	0.194	0	0
TRINITY_49153_length_826_cov_25.743725_g33679_i0	14.207	6.305	0	0.768	0	0
TRINITY_Contig19379	45.222	55.039	2.044	1.059	0	0
TRINITY_6426_length_2624_cov_32.417613_g4064_i0	2.595	3.381	666.57	140.19	37.331	16.907
TRINITY_Contig12983	11.245	13.117	0	0.412	2.074	0.632
TRINITY_BIN_7705_7	31.99	8.962	1.556	1.18	0	0.709
TRINITY_DN13464_c0_g1_i4	3.724	5.357	136.527	65.058	108.531	77.204
TRINITY_53107_length_756_cov_28.545852_g37076_i0	78.818	102.281	3.98	15.652	4.533	11.646
TRINITY_DN17266_c1_g2_i7	24.066	31.063	1.773	5.595	5.185	4.976
TRINITY_21725_length_1565_cov_29.952540_g13703_i0	18.158	10.576	1.049	0.307	0.853	4.768
TRINITY_DN20418_c0_g1_i7	17.212	10.583	0.47	0.97	1.02	2.195
TRINITY_12044_length_2070_cov_32.029985_g6712_i1	3.445	7.427	0	0.049	0.385	0.67
TRINITY_17846_length_1738_cov_29.339125_g11206_i0	13.642	11.517	1.628	0.566	0	0.947
TRINITY_BIN_2817_1	0	0.594	73.999	111.327	43.77	3.536
TRINITY_39052_length_1033_cov_27.057054_g25669_i0	1.547	1.498	154.168	112.734	89.397	66.535
TRINITY_DN18322_c0_g3_i1	6.7	3.25	0.253	0.582	0.552	0.316
TRINITY_DN19913_c0_g1_i7	17.329	1.918	0.163	0	0	0
TRINITY_DN17322_c1_g2_i6	0.073	0.927	150.857	246.391	12.343	9.259
TRINITY_DN22080_c2_g8_i1	23.054	24.077	0	0.469	0	0.524
TRINITY_Contig10122	13.261	16.802	0.434	1.965	0	1.202
TRINITY_BIN_15544_2	4.376	25.909	0.561	0.073	0	4.498
TRINITY_DN15946_c1_g2_i4	0.059	0	68.318	24.222	10.788	7.264
TRINITY_DN15464_c2_g1_i5	0	0	265.094	314.877	81.686	107.937
TRINITY_DN21825_c1_g1_i6	4.53	6.87	0	0.081	0	0.886
TRINITY_DN20951_c1_g4_i1	0.433	0.492	103.327	29.024	4.783	2.295
TRINITY_Contig7533	4.054	3.656	236.453	283.355	81.82	146.612
TRINITY_Contig15369	32.789	16.918	1.683	2.393	4.934	0.855
TRINITY_BIN_13043_9	43.785	25.988	4.306	3.937	2.827	6.401
TRINITY_Contig8669	18.282	17.497	1.502	4.487	2.124	5.785
TRINITY_Contig9647	5.505	7.246	1124.586	646.53	182.774	275.046
TRINITY_DN21756_c2_g1_i15	20.247	7.319	1.14	1.253	2.325	3.066
TRINITY_44878_length_907_cov_25.770883_g30171_i0	0	0.637	35.299	4.156	26.342	39.237
TRINITY_22226_length_1542_cov_31.294637_g2198_i1	38.163	25.054	0.887	2.151	0.284	0.539
TRINITY_Contig493	51.196	33.589	6.188	9.37	7.677	5.839
TRINITY_Contig11950	0.154	3.106	245.807	174.679	467.389	0.562

TRINITY_DN21909_c0_g2_i10	184.209	96.576	0.308	4.479	0.686	0.154
TRINITY_BIN_2887_1	38.111	63.964	0.163	1.787	0.385	0.493
TRINITY_Contig20011	41.08	85.5	9.969	11.351	16.09	11.2
TRINITY_Contig15543	5.183	9.997	0.181	0	0	2.858
TRINITY_Contig19467	34.431	6.544	0.507	1.294	2.827	3.289
TRINITY_DN17265_c0_g3_i1	1.928	2.896	302.654	211.966	43.218	28.43
TRINITY_18953_length_1685_cov_24.115099_g1094_i2	0.396	0	7.165	35.5	25.673	17.593
TRINITY_Contig18731	13.166	19.321	1.447	2.749	2.275	3.505
TRINITY_Contig18398	6.927	12.031	0.76	0.711	0.401	2.01
TRINITY_7703_length_2464_cov_22.626722_g4886_i0	4.398	5.357	0	0.695	0.284	0.585
TRINITY_DN17564_c0_g1_i12	11.311	13.088	0.416	1.536	1.539	2.003
TRINITY_Contig17457	175.39	73.925	2.099	11.561	5.302	16.445
TRINITY_27036_length_1369_cov_21.286923_g17220_i0	18.876	39.684	0.507	5.732	2.208	8.85
TRINITY_DN13918_c0_g8_i1	2.104	3.75	461.924	197.931	257.955	160.03
TRINITY_DN8691_c0_g1_i2	73.254	64.377	3.347	15.329	9.132	14.935
TRINITY_Contig898	0.088	1.433	220.17	65.753	10.219	6.909
TRINITY_DN18037_c0_g1_i1	0.029	0.152	26.922	9.75	18.95	22.954
TRINITY_32198_length_1210_cov_30.662577_g20743_i0	10.871	5.118	0.597	0.154	1.004	0
TRINITY_Contig14673	254.376	215.058	7.49	10.631	9.065	5.03
TRINITY_15397_length_1864_cov_26.900836_g9656_i0	189.428	86.13	17.079	15.798	18.063	7.325
TRINITY_2046_length_3684_cov_29.339419_g1306_i0	8.342	9.606	0.434	1.698	1.187	2.75
TRINITY_BIN_198_5	7.998	3.735	0.362	0.428	1.589	0.755
TRINITY_BIN_18663_3	15.094	22.419	0	0.121	1.054	3.374
TRINITY_Contig17611	92.452	24.461	1.411	8.909	10.805	7.703
TRINITY_58582_length_668_cov_30.041736_g42051_i0	0.33	0.203	271.824	340.069	54.474	78.906
TRINITY_Contig17091	22.959	21.703	1.646	1.9	0.234	3.035
TRINITY_42067_length_963_cov_25.493289_g27976_i0	10.629	14.5	0.814	1.859	0.903	2.434
TRINITY_BIN_18860_2	1.979	5.111	1043.043	152.681	111.458	23.817
TRINITY_DN15842_c0_g1_i2	0.359	0.514	153.733	69.957	7.577	6.27
TRINITY_22103_length_1548_cov_28.137255_g13972_i0	0.323	0.869	177.489	33.705	8.831	1.718
TRINITY_DN12427_c0_g2_i2	6.832	9.896	0	0.566	0	0
TRINITY_BIN_14328_1	24.777	52.389	2.424	0.461	0	3.551
TRINITY_Contig16403	4.977	7.891	0.398	0.881	0.151	0.454
TRINITY_DN19315_c1_g1_i4	0	0	160.41	63.142	13.681	14.681
TRINITY_16911_length_1783_cov_23.957410_g5241_i1	4.193	7.702	0	0	0	0.37
TRINITY_19238_length_1673_cov_21.231920_g12106_i0	0.938	14.21	0	0	2.024	0
TRINITY_DN15708_c0_g1_i1	1.283	10.75	1290.044	691.595	170.632	123.042
TRINITY_BIN_15770_1	0.85	9.664	2736.734	565.788	171.468	58.925
TRINITY_DN21458_c1_g1_i1	20.54	16.664	1.628	4.495	2.944	4.945
TRINITY_DN19004_c0_g1_i8	6.722	5.321	0	0	0	0
TRINITY_DN19057_c0_g5_i4	37.979	22.405	1.321	6.419	0	0.986
TRINITY_Contig17465	8.188	18.626	1.393	0.501	0.719	3.066
TRINITY_1987_length_3713_cov_30.967069_g1272_i0	7.631	22.369	0.09	0	0.284	2.919

Supplementary table 1. *In silico* expression matrix for DEGs in Root (control vs salt, control vs drought) and Leaves (control vs drought, control vs salt) and the UniProt Ids for the Unigenes.

Unigene ID	UniProt SwissProt ID	% identity	E-value
TRINITY_Contig199	sp_Q3BAI2_YCX91_PHAAO_Uncharacterized_protein_ORF91_OS=Phalaenopsis_aphrodite_subsp._formosana_OX=308872_PE=4_SV=1	68.421	3.63E-13
TRINITY_Contig325	sp_Q8TGM6_TAR1_YEAST_Protein_TAR1_OS=Saccharomyces_cerevisiae_(strain_ATCC_204508_/_S288c)_OX=559292_GN=TAR1_PE=2_SV=1	53.982	6.69E-23
TRINITY_Contig360	sp_Q9ZNR0_C78A6_ARATH_Cytochrome_P450_78A6_OS=Arabidopsis_thaliana_OX=3702_GN=CYP78A6_PE=2_SV=1	57.547	1.16E-175
TRINITY_Contig379	sp_Q3BAI2_YCX91_PHAAO_Uncharacterized_protein_ORF91_OS=Phalaenopsis_aphrodite_subsp._formosana_OX=308872_PE=4_SV=1	71.429	2.25E-16
TRINITY_Contig394	sp_Q94AA4_PFKA3_ARATH_ATP-dependent_6-phosphofructokinase_3_OS=Arabidopsis_thaliana_OX=3702_GN=PFK3_PE=1_SV=1	77.523	0
TRINITY_Contig444	sp_O13755_OSM1_SCHPO_Fumarate_reductase_OS=Schizosaccharomyces_pombe_(strain_972_/_ATCC_24843)_OX=284812_GN=osm1_PE=3_SV=1	50.753	4.41E-144
TRINITY_Contig448	sp_Q9SUC6_BBE22_ARATH_Berberine_bridge_enzyme-like_22_OS=Arabidopsis_thaliana_OX=3702_GN=FAD-OXR_PE=2_SV=1	49.887	7.67E-105
TRINITY_Contig482	sp_B7KJX8_LEU1_CYAP7_2-isopropylmalate_synthase_OS=Cyanothece_sp._(strain_PCC_7424)_OX=65393_GN=leuA_PE=3_SV=1	55.229	0
TRINITY_Contig493	sp_Q9SGD6_AROD6_ARATH_Arogenate_dehydratase/prephenate_dehydratase_6,_chloroplastic_OS=Arabidopsis_thaliana_OX=3702_GN=ADT6_PE=1_SV=1	75.196	0
TRINITY_Contig502	sp_P10719_ATPB_RAT_ATP_synthase_subunit_beta,_mitochondrial_OS=Rattus_norvegicus_OX=10116_GN=Atp5f1b_PE=1_SV=2	75.862	0
TRINITY_Contig510	sp_Q9AR73_HQGT_RAUSE_Hydroquinone_glucosyltransferase_OS=Rauvolfia_serpentina_OX=4060_GN=AS_PE=1_SV=1	43.133	3.79E-99
TRINITY_Contig633	sp_Q3BAI2_YCX91_PHAAO_Uncharacterized_protein_ORF91_OS=Phalaenopsis_aphrodite_subsp._formosana_OX=308872_PE=4_SV=1	66.667	7.28E-13
TRINITY_Contig672	sp_P27939_AMY3C_ORYSJ_Alpha-amylase_isozyme_3C_OS=Oryza_sativa_subsp._japonica_OX=39947_GN=AMY1.7_PE=2_SV=2	87.872	0
TRINITY_Contig689	sp_Q10R54_SUT1_ORYSJ_Sucrose_transport_protein_SUT1_OS=Oryza_sativa_subsp._japonica_OX=39947_GN=SUT1_PE=1_SV=1	26.912	8.76E-19
TRINITY_Contig816	sp_C0LGH2_Y1561_ARATH_Probable_LRR_receptor-like_serine/threonine-protein_kinase_At1g56130_OS=Arabidopsis_thaliana_OX=3702_GN=At1g56130_PE=2_SV=2	49.407	3.99E-62
TRINITY_Contig816	sp_C0LGH2_Y1561_ARATH_Probable_LRR_receptor-like_serine/threonine-protein_kinase_At1g56130_OS=Arabidopsis_thaliana_OX=3702_GN=At1g56130_PE=2_SV=2	52.083	4.97E-31
TRINITY_Contig816	sp_C0LGH2_Y1561_ARATH_Probable_LRR_receptor-like_serine/threonine-protein_kinase_At1g56130_OS=Arabidopsis_thaliana_OX=3702_GN=At1g56130_PE=2_SV=2	57.576	1.27E-16
TRINITY_Contig849	sp_Q3BAI2_YCX91_PHAAO_Uncharacterized_protein_ORF91_OS=Phalaenopsis_aphrodite_subsp._formosana_OX=308872_PE=4_SV=1	68.421	6.37E-14
TRINITY_Contig852	sp_Q39757_1433_FUCVE_14-3-3-like_protein_OS=Fucus_vesiculosus_OX=49266_PE=2_SV=1	76.23	8.26E-123
TRINITY_Contig859	sp_Q8L970_P4H7_ARATH_Probable_prolyl_4-hydroxylase_7_OS=Arabidopsis_thaliana_OX=3702_GN=P4H7_PE=2_SV=1	62.454	1.09E-123
TRINITY_Contig869	sp_A9RHX1_THI41_PHYPA_Thiamine_thiazole_synthase_1,_chloroplastic_OS=Physcomitrella_patens_subsp._patens_OX=3218_GN=THI1-1_PE=3_SV=1	66.774	1.42E-149
TRINITY_Contig882	sp_Q55512_DHAS_SYNY3_Aspartate-semialdehyde_dehydrogenase_OS=Synechocystis_sp._(strain_PCC_6803_/_Kazusa)_OX=1111708_GN=asd_PE=3_SV=2	54.627	4.8E-104
TRINITY_Contig896	sp_Q9FPL6_METK2_SUASA_S-adenosylmethionine_synthase_2_OS=Suaeda_salsa_OX=126914_GN=METK2_PE=2_SV=1	72.539	0
TRINITY_Contig908	sp_P22131_ACT1_PHYIN_Actin-1_OS=Phytophthora_infestans_OX=4787_GN=ACTA_PE=2_SV=1	97.872	0
TRINITY_Contig913	sp_Q8DJB3_GRPE_THEEB_Protein_GrpE_OS=Thermosynechococcus_elongatus_(strain_BP-1)_OX=197221_GN=grpE_PE=3_SV=1	50	1.88E-41
TRINITY_Contig944	sp_P18124_RL7_HUMAN_60S_ribosomal_protein_L7_OS=Homo_sapiens_OX=9606_GN=RPL7_PE=1_SV=1	61.111	4.4E-100
TRINITY_Contig951	sp_Q84PB7_THF1_ORYSJ_Protein_THYLAKOID_FORMATION1,_chloroplastic_OS=Oryza_sativa_subsp._japonica_OX=39947_GN=THF1_PE=2_SV=1	86.411	2.74E-149
TRINITY_Contig960	sp_Q8LIL0_CUS_ORYSJ_Bisdemethoxycurcumin_synthase_OS=Oryza_sativa_subsp._japonica_OX=39947_GN=Os07g0271500_PE=1_SV=2	59.5	2.56E-130
TRINITY_Contig982	sp_Q9FFF6_DIOX5_ARATH_Probable_2-oxoglutarate-dependent_dioxygenase_At5g05600_OS=Arabidopsis_thaliana_OX=3702_GN=At5g05600_PE=2_SV=1	38.614	2.96E-58
TRINITY_Contig1009	sp_Q94CS9_TIP12_ORYSJ_Probable_aquaporin_TIP1-2_OS=Oryza_sativa_subsp._japonica_OX=39947_GN=TIP1-2_PE=2_SV=1	93.254	8.65E-142
TRINITY_Contig1057	sp_Q9ZUN4_ACCO1_ARATH_1-aminocyclopropane-1-carboxylate_oxidase_1_OS=Arabidopsis_thaliana_OX=3702_GN=ACO1_PE=2_SV=1	48.052	2.01E-92
TRINITY_Contig1106	sp_Q26365_ADT_DROME_ADP,ATP_carrier_protein_OS=Drosophila_melanogaster_OX=7227_GN=sesB_PE=2_SV=4	73.22	5.64E-136
TRINITY_Contig1134	sp_Q39096_ERD15_ARATH_Protein_EARLY_RESPONSIVE_TO_DEHYDRATION_15_OS=Arabidopsis_thaliana_OX=3702_GN=ERD15_PE=1_SV=1	50.725	2.83E-13

TRINITY_Contig1168	sp_Q8HXY0_ETFA_MACFA_Electron_transfer_flavoprotein_subunit_alpha,_mitochondrial_OS=Macaca_fascicularis_OX=9541_GN=ETFA_PE=2_SV=1	56.875	2.18E-117
TRINITY_Contig1198	sp_Q9DG68_RLA0_RANSY_60S_acidic_ribosomal_protein_P0_OS=Rana_sylvatica_OX=45438_GN=RPLP0_PE=2_SV=1	58.935	4.47E-103
TRINITY_Contig1200	sp_Q9UR38_G3P_LENED_Glyceraldehyde-3-phosphate_dehydrogenase_OS=Lentinula_edodes_OX=5353_GN=gpd_PE=2_SV=1	75.581	1.35E-167
TRINITY_Contig1225	sp_Q8S8Q6_TET8_ARATH_Tetraspanin-8_OS=Arabidopsis_thaliana_OX=3702_GN=TET8_PE=2_SV=1	52.814	7.27E-62
TRINITY_Contig1250	sp_P24631_HSP21_MAIZE_17.5_kDa_class_II_heat_shock_protein_OS=Zea_mays_OX=4577_PE=2_SV=1	86.713	1.47E-68
TRINITY_Contig1252	sp_F8WKW1_UGT2_GARJA_7-deoxyloganetin_glucosyltransferase_OS=Gardenia_jasminoides_OX=114476_GN=UGT85A24_PE=1_SV=1	47.774	5.2E-90
TRINITY_Contig1350	sp_P62755_RS6_RAT_40S_ribosomal_protein_S6_OS=Rattus_norvegicus_OX=10116_GN=Rps6_PE=1_SV=1	69.406	1.38E-107
TRINITY_Contig1358	sp_Q00988_CY1_KLULA_Cytochrome_c1,_heme_protein,_mitochondrial_OS=Kluyveromyces_lactis_(strain_ATCC_8585_/_CBS_2359_/_DSM_70799_/_NBRC_1267_/_NRRL_Y-1140_/_WM37)_OX=284590_GN=CYT1_PE=3_SV=1	59.402	6.05E-93
TRINITY_Contig1409	sp_Q9XS41_SODM_HORSE_Superoxide_dismutase_[Mn],_mitochondrial_OS=Equus_caballus_OX=9796_GN=SOD2_PE=2_SV=1	67.677	5.99E-90
TRINITY_Contig1409	sp_Q9XS41_SODM_HORSE_Superoxide_dismutase_[Mn],_mitochondrial_OS=Equus_caballus_OX=9796_GN=SOD2_PE=2_SV=1	75.758	4.47E-09
TRINITY_Contig1421	sp_Q00008_PRMS_MAIZE_Pathogenesis-related_protein_PRMS_OS=Zea_mays_OX=4577_GN=PRMS_PE=2_SV=1	63.571	5.12E-56
TRINITY_Contig1453	sp_Q9XG77_PSA6_TOBAC_Proteasome_subunit_alpha_type-6_OS=Nicotiana_tabacum_OX=4097_GN=PAA1_PE=2_SV=1	62.397	6.88E-103
TRINITY_Contig1529	sp_Q9FLF0_RS92_ARATH_40S_ribosomal_protein_S9-2_OS=Arabidopsis_thaliana_OX=3702_GN=RPS9C_PE=1_SV=1	67.47	1.38E-81
TRINITY_Contig1533	sp_Q75L11_H2A6_ORYSJ_Probable_histone_H2A.6_OS=Oryza_sativa_subsp._japonica_OX=39947_GN=Os05g0113900_PE=2_SV=2	100	2.27E-50
TRINITY_Contig1543	sp_D0N381_DRE2_PHYIT_Anamorsin_homolog_OS=Phytophthora_infestans_(strain_T30-4)_OX=403677_GN=PITG_05601_PE=3_SV=1	53.358	8.56E-70
TRINITY_Contig1591	sp_Q84Q77_HS17A_ORYSJ_17.9_kDa_class_I_heat_shock_protein_OS=Oryza_sativa_subsp._japonica_OX=39947_GN=HSP17.9A_PE=1_SV=1	85.714	4.1E-63
TRINITY_Contig1597	sp_Q8AWF2_NACA_ORENI_Nascent_polypeptide-associated_complex_subunit_alpha_OS=Oreochromis_niloticus_OX=8128_GN=naca_PE=2_SV=1	54.545	2.32E-39
TRINITY_Contig1625	sp_Q96252_ATP4_ARATH_ATP_synthase_subunit_delta',_mitochondrial_OS=Arabidopsis_thaliana_OX=3702_GN=At5g47030_PE=1_SV=1	44.444	5.35E-31
TRINITY_Contig1654	sp_O61231_RL10_DROME_60S_ribosomal_protein_L10_OS=Drosophila_melanogaster_OX=7227_GN=RpL10_PE=1_SV=1	70.874	4.43E-107
TRINITY_Contig1670	sp_P51413_RL172_ARATH_60S_ribosomal_protein_L17-2_OS=Arabidopsis_thaliana_OX=3702_GN=RPL17B_PE=2_SV=2	54.268	3.32E-57
TRINITY_Contig1685	sp_Q55GJ6_PSB6_DICDI_Proteasome_subunit_beta_type-6_OS=Dictyostelium_discoideum_OX=44689_GN=psmB6_PE=1_SV=1	62.264	3.43E-82
TRINITY_Contig1691	sp_Q8VY57_ARL8A_ARATH_ADP-ribosylation_factor-like_protein_8a_OS=Arabidopsis_thaliana_OX=3702_GN=ARL8A_PE=2_SV=1	43.017	3.97E-43
TRINITY_Contig1725	sp_P37515_MAA_BACSU_Probable_maltose_O-acetyltransferase_OS=Bacillus_subtilis_(strain_168)_OX=224308_GN=maa_PE=3_SV=1	53.261	1.56E-61
TRINITY_Contig1736	sp_Q71UH5_CALM_PYTSP_Calmodulin_OS=Pythium_splendens_OX=82926_PE=2_SV=1	100	9.86E-103
TRINITY_Contig1788	sp_P28799_GRN_HUMAN_Granulins_OS=Homo_sapiens_OX=9606_GN=GRN_PE=1_SV=2	42.667	1.78E-11
TRINITY_Contig1788	sp_P28799_GRN_HUMAN_Granulins_OS=Homo_sapiens_OX=9606_GN=GRN_PE=1_SV=2	49.275	1.34E-09
TRINITY_Contig1788	sp_P28799_GRN_HUMAN_Granulins_OS=Homo_sapiens_OX=9606_GN=GRN_PE=1_SV=2	57.447	2.47E-09
TRINITY_Contig1788	sp_P28799_GRN_HUMAN_Granulins_OS=Homo_sapiens_OX=9606_GN=GRN_PE=1_SV=2	45.714	5.78E-09
TRINITY_Contig1788	sp_P28799_GRN_HUMAN_Granulins_OS=Homo_sapiens_OX=9606_GN=GRN_PE=1_SV=2	39.655	0.00000113
TRINITY_Contig1788	sp_P28799_GRN_HUMAN_Granulins_OS=Homo_sapiens_OX=9606_GN=GRN_PE=1_SV=2	48.936	0.00000836
TRINITY_Contig1849	sp_Q8TGM7_ART2_YEAST_Putative_uncharacterized_protein_ART2_OS=Saccharomyces_cerevisiae_(strai	89.474	9.35E-27
TRINITY_Contig1854	sp_P29763_RLA1_CHLRE_60S_acidic_ribosomal_protein_P1_OS=Chlamydomonas_reinhardtii_OX=3055_PE=3_SV=1	51.724	3.28E-10
TRINITY_Contig1860	sp_P61866_RL12_CAEEL_60S_ribosomal_protein_L12_OS=Caenorhabditis_elegans_OX=6239_GN=rpl-12_PE=3_SV=1	67.485	3.38E-73
TRINITY_Contig1901	sp_Q9FT52_ATP5H_ARATH_ATP_synthase_subunit_d,_mitochondrial_OS=Arabidopsis_thaliana_OX=3702_GN=At3g52300_PE=1_SV=3	78.667	1.8E-70
TRINITY_Contig2061	sp_P84089_ERH_MOUSE_Enhancer_of_rudimentary_homolog_OS=Mus_musculus_OX=10090_GN=Erh_PE=1_SV=1	56.701	7.95E-34
TRINITY_Contig2187	sp_A4K526_TM256_BUFGR_Transmembrane_protein_256_homolog_OS=Bufo_gargarizans_OX=30331_PE=3_SV=1	46.429	2.07E-19
TRINITY_Contig2198	sp_Q9M7X7_RL291_ARATH_60S_ribosomal_protein_L29-1_OS=Arabidopsis_thaliana_OX=3702_GN=RPL29A_PE=1_SV=1	62.295	3.75E-18
TRINITY_Contig2221	sp_P36525_RM24_YEAST_54S_ribosomal_protein_L24,_mitochondrial_OS=Saccharomyces_cerevisiae_(strain_ATCC_204508_/_S288c)_OX=559292_GN=MRPL24_PE=1_SV=2	40.566	4E-15
TRINITY_Contig2273	sp_P48495_TPIS_PETHY_Triosephosphate_isomerase,_cytosolic_OS=Petunia_hybrida_OX=4102_GN=TPIP1_PE=2_SV=1	81.275	1.62E-149
TRINITY_Contig2475	sp_Q6ENQ6_YCF76_SACOF_Uncharacterized_protein_ycf76_OS=Saccharum_officinarum_OX=4547_GN=ycf76-A_PE=3_SV=1	98.824	2.96E-52

TRINITY_Contig2476	sp_Q8S2G0_ISPE_ORYSJ_4-diphosphocytidyl-2-C-methyl-D-erythritol_kinase,_chloroplastic_OS=Oryza_sativa_subsp._japonica_OX=39947_GN=ISPE_PE=2_SV=1	81.454	0
TRINITY_Contig2510	sp_B3CQN9_RL19_ORITI_50S_ribosomal_protein_L19_OS=Orientia_tsutsugamushi_(strain_Ikeda)_OX=334380_GN=rplS_PE=3_SV=1	39.583	1.01E-14
TRINITY_Contig2518	sp_Q9S9K4_ASPL2_ARATH_Aspartic_proteinase-like_protein_2_OS=Arabidopsis_thaliana_OX=3702_GN=At1g65240_PE=3_SV=2	47.867	2.33E-127
TRINITY_Contig2556	sp_O48651_ERG1_PANGI_Squalene_monooxygenase_OS=Panax_ginseng_OX=4054_PE=2_SV=1	83.964	0
TRINITY_Contig2733	sp_Q93W28_Y4554_ARATH_Uncharacterized_protein_At4g15545_OS=Arabidopsis_thaliana_OX=3702_GN=At4g15545_PE=1_SV=1	50.152	1.54E-72
TRINITY_Contig2740	sp_P42794_RL112_ARATH_60S_ribosomal_protein_L11-2_OS=Arabidopsis_thaliana_OX=3702_GN=RPL11B_PE=2_SV=2	75.294	7.26E-93
TRINITY_Contig2802	sp_P33198_IDHP_PIG_Isocitrate_dehydrogenase_[NADP],_mitochondrial_(Fragment)_OS=Sus_scrofa_OX=9823_GN=IDH2_PE=1_SV=1	67.813	0
TRINITY_Contig2888	sp_Q6JN46_EIX2_SOLLC_Receptor-like_protein_EIX2_OS=Solanum_lycopersicum_OX=4081_GN=EIX2_PE=1_SV=2	40.318	4.17E-50
TRINITY_Contig2888	sp_Q6JN46_EIX2_SOLLC_Receptor-like_protein_EIX2_OS=Solanum_lycopersicum_OX=4081_GN=EIX2_PE=1_SV=2	30.348	0.000000543
TRINITY_Contig2888	sp_Q6JN46_EIX2_SOLLC_Receptor-like_protein_EIX2_OS=Solanum_lycopersicum_OX=4081_GN=EIX2_PE=1_SV=2	29.762	0.00000372
TRINITY_Contig2892	sp_Q3BAI2_YCX91_PHAAO_Uncharacterized_protein_ORF91_OS=Phalaenopsis_aphrodite_subsp._formosana_OX=308872_PE=4_SV=1	68.421	3.48E-14
TRINITY_Contig2916	sp_P19536_COX5B_MOUSE_Cytochrome_c_oxidase_subunit_5B,_mitochondrial_OS=Mus_musculus_OX=10090_GN=Cox5b_PE=1_SV=1	29.63	0.000000014
TRINITY_Contig2917	sp_Q93YN1_CRPK1_ARATH_Cold-responsive_protein_kinase_1_OS=Arabidopsis_thaliana_OX=3702_GN=CRPK1_PE=1_SV=1	60.694	3.76E-144
TRINITY_Contig2954	sp_P40620_HMGL_VICFA_HMG1/2-like_protein_OS=Vicia_faba_OX=3906_PE=2_SV=1	65.517	6.43E-20
TRINITY_Contig3003	sp_B8ELF8_RL22_METSB_50S_ribosomal_protein_L22_OS=Methylocella_silvestris_(strain_DSM_15510_/_CIP_108128_/_LMG_27833_/_NCIMB_13906_/_BL2)_OX=395965_GN=rplV_PE=3_SV=1	43.966	4.69E-22
TRINITY_Contig3017	sp_Q94JQ4_RIDA_ARATH_Reactive_Intermediate_Deaminase_A,_chloroplastic_OS=Arabidopsis_thaliana_OX=3702_GN=RIDA_PE=1_SV=1	87.097	2.76E-71
TRINITY_Contig3023	sp_Q96565_AMNMT_HORVV_3-aminomethylindole_N-methyltransferase_OS=Hordeum_vulgare_subsp._vulgare_OX=112509_PE=1_SV=1	62.703	1.09E-169
TRINITY_Contig3069	sp_O22527_CLH1_ARATH_Chlorophyllase-1_OS=Arabidopsis_thaliana_OX=3702_GN=CLH1_PE=1_SV=1	48.993	4.17E-93
TRINITY_Contig3104	sp_O65282_CH20_ARATH_20_kDa_chaperonin,_chloroplastic_OS=Arabidopsis_thaliana_OX=3702_GN=CPN20_PE=1_SV=2	68.482	5.67E-117
TRINITY_Contig3132	sp_Q16698_DECR_HUMAN_2,4-dienoyl-CoA_reductase,_mitochondrial_OS=Homo_sapiens_OX=9606_GN=DECR1_PE=1_SV=1	61.672	2.13E-105
TRINITY_Contig3150	sp_Q96WZ1_EF1A_COCIM_Elongation_factor_1-alpha_OS=Coccidioides_immitis_(strain_RS)_OX=246410_GN=TEF_PE=2_SV=2	44.812	5.72E-131
TRINITY_Contig3165	sp_Q96253_ATP5E_ARATH_ATP_synthase_subunit_epsilon,_mitochondrial_OS=Arabidopsis_thaliana_OX=3702_GN=At1g51650_PE=1_SV=3	43.333	5.56E-11
TRINITY_Contig3167	sp_P15569_ELIB_PHYCI_Beta-elicitin_cinnamomin_OS=Phytophthora_cinnamomi_OX=4785_PE=1_SV=1	39.024	0.00000192
TRINITY_Contig3184	sp_P48279_YCF39_CYAPA_Uncharacterized_protein_ycf39_OS=Cyanophora_paradoxa_OX=2762_GN=ycf39_PE=3_SV=1	50.313	2.8E-114
TRINITY_Contig3189	sp_Q42376_LEA3_MAIZE_Late_embryogenesis_abundant_protein,_group_3_OS=Zea_mays_OX=4577_GN=MGL3_PE=2_SV=1	70.455	2.98E-49
TRINITY_Contig3266	sp_Q6NQE2_FQRL1_ARATH_Probable_NAD(P)H_dehydrogenase_(quinone)_FQR1-like_1_OS=Arabidopsis_thaliana_OX=3702_GN=At4g27270_PE=1_SV=1	58.586	1.76E-65
TRINITY_Contig3308	sp_P23403_RS20_XENLA_40S_ribosomal_protein_S20_OS=Xenopus_laevis_OX=8355_GN=rps20_PE=1_SV=1	80.374	3.91E-59
TRINITY_Contig3328	sp_Q9PP01_PCKA_CAMJE_Phosphoenolpyruvate_carboxykinase_(ATP)_OS=Campylobacter_jejuni_subsp._jejuni_serotype_O:2_(strain_ATCC_700819_/_NCTC_11168)_OX=192222_GN=pckA_PE=3_SV=1	60.115	0

TRINITY_Contig3367	sp_P46518_LEA14_GOSHI_Late_embryogenesis_abundant_protein_Lea14-A_OS=Gossypium_hirsutum_OX=3635_GN=LEA14-A_PE=2_SV=1	66.225	4.21E-73
TRINITY_Contig3397	sp_Q86WU2_LDHD_HUMAN_Probable_D-lactate_dehydrogenase,_mitochondrial_OS=Homo_sapiens_OX=9606_GN=LDHD_PE=1_SV=1	47.323	5.4E-151
TRINITY_Contig3430	sp_P0AB91_AROG_ECOLI_Phospho-2-dehydro-3-deoxyheptonate_aldolase,_Phe-sensitive_OS=Escherichia_coli_(strain_K12)_OX=83333_GN=aroG_PE=1_SV=1	58.529	9.57E-130
TRINITY_Contig3457	sp_Q94HF1_EIF3K_ORYSJ_Eukaryotic_translation_initiation_factor_3_subunit_K_OS=Oryza_sativa_subsp._japonica_OX=39947_GN=TIF3K1_PE=2_SV=1	27.885	7.74E-22
TRINITY_Contig3460	sp_P38542_RAN_BRUMA_GTP-binding_nuclear_protein_Ran_OS=Brugia_malayi_OX=6279_GN=Bm1_44725_PE=2_SV=2	87.027	2.81E-119
TRINITY_Contig3483	sp_P41827_HSP74_ANOAL_Heat_shock_protein_70_B2_OS=Anopheles_albimanus_OX=7167_GN=HSP70B2_PE=3_SV=1	43.436	9.08E-146
TRINITY_Contig3486	sp_Q1QS47_UBIE_NITHX_Ubiquinone/menaquinone_biosynthesis_C-methyltransferase_UbiE_OS=Nitrobacter_hamburgensis_(strain_DSM_10229_/_NCIMB_13809_/_X14)_OX=323097_GN=ubiE_PE=3_SV=1	35.616	1.42E-09
TRINITY_Contig3523	sp_P26222_GUN2_THEFU_Endoglucanase_E-2_OS=Thermobifida_fusca_OX=2021_GN=celB_PE=1_SV=2	33.594	1.25E-28
TRINITY_Contig3533	sp_P70684_PGDH_CAVPO_15-hydroxyprostaglandin_dehydrogenase_[NAD(+)]_OS=Cavia_porcellus_OX=10141_GN=HPGD_PE=2_SV=1	30.932	1.53E-26
TRINITY_Contig3541	sp_Q9RPT1_RHLG_PSEAE_Rhamnolipids_biosynthesis_3-oxoacyl-[acyl-carrier-protein]_reductase_OS=Pseudomonas_aeruginosa_(strain_ATCC_15692_/_DSM_22644_/_CIP_104116_/_JCM_14847_/_LMG_12228_/_1C_/_PRS_101_/_PAO1)_OX=208964_GN=rhlG_PE=1_SV=1	40.084	8.84E-52
TRINITY_Contig3552	sp_Q04013_YHM2_YEAST_Citrate/oxoglutarate_carrier_protein_OS=Saccharomyces_cerevisiae_(strain_ATCC_204508_/_S288c)_OX=559292_GN=YHM2_PE=1_SV=1	33.217	1.63E-37
TRINITY_Contig3608	sp_Q9M5K3_DLDH1_ARATH_Dihydrolipoyl_dehydrogenase_1,_mitochondrial_OS=Arabidopsis_thaliana_OX=3702_GN=LPD1_PE=1_SV=2	62.031	0
TRINITY_Contig3614	sp_O02640_MDHM_CAEEL_Probable_malate_dehydrogenase,_mitochondrial_OS=Caenorhabditis_elegans_OX=6239_GN=mdh-2_PE=3_SV=1	62.876	8.49E-126
TRINITY_Contig3647	sp_Q11HS7_RL17_CHESB_50S_ribosomal_protein_L17_OS=Chelativorans_sp._(strain_BNC1)_OX=266779_GN=rplQ_PE=3_SV=1	56.78	2.23E-39
TRINITY_Contig3663	sp_Q9SDG6_RL30_ORYSJ_60S_ribosomal_protein_L30_OS=Oryza_sativa_subsp._japonica_OX=39947_GN=RPL30_PE=3_SV=1	65.455	1.47E-51
TRINITY_Contig3665	sp_Q757G1_RS29_ASHGO_40S_ribosomal_protein_S29_OS=Ashbya_gossypii_(strain_ATCC_10895_/_CBS_109.51_/_FGSC_9923_/_NRRL_Y-1056)_OX=284811_GN=RPS29_PE=3_SV=1	69.231	6.83E-20
TRINITY_Contig3756	sp_Q33339_RDRP_CPMV1_RNA-directed_RNA_polymerase_OS=Cryphonectria_parasitica_mitovirus_1_(strain_American_chestnut_tree/New_Jersey/NB631)_OX=186769_PE=3_SV=1	42.411	1.51E-35
TRINITY_Contig3774	sp_P90543_H3_EUPCR_Histone_H3_OS=Euplotes_crassus_OX=5936_PE=3_SV=3	77.011	8.71E-42
TRINITY_Contig3792	sp_Q3T186_RPIA_BOVIN_Ribose-5-phosphate_isomerase_(Fragment)_OS=Bos_taurus_OX=9913_GN=RPIA_PE=2_SV=2	56.034	5.11E-78
TRINITY_Contig3852	sp_P55142_GRXC6_ORYSJ_Glutaredoxin-C6_OS=Oryza_sativa_subsp._japonica_OX=39947_GN=GRXC6_PE=1_SV=2	47.115	5.85E-26
TRINITY_Contig3865	sp_Q9P2R3_ANFY1_HUMAN_Rabankyrin-5_OS=Homo_sapiens_OX=9606_GN=ANKFY1_PE=1_SV=2	27.711	1.04E-10
TRINITY_Contig3876	sp_P37515_MAA_BACSU_Probable_maltose_O-acetyltransferase_OS=Bacillus_subtilis_(strain_168)_OX=224308_GN=maa_PE=3_SV=1	53.552	2.62E-55
TRINITY_Contig3882	sp_Q8WQI5_RS8_SPOFR_40S_ribosomal_protein_S8_OS=Spodoptera_frugiperda_OX=7108_GN=RpS8_PE=2_SV=1	66.99	7.04E-74
TRINITY_Contig3887	sp_P81102_YODC_BACSU_Putative_NAD(P)H_nitroreductase_YodC_OS=Bacillus_subtilis_(strain_168)_OX=224308_GN=yodC_PE=1_SV=3	30.542	6.88E-17
TRINITY_Contig3915	sp_P78946_RL26_SCHPO_60S_ribosomal_protein_L26_OS=Schizosaccharomyces_pombe_(strain_972_/_ATCC_24843)_OX=284812_GN=rpl26_PE=3_SV=1	67.925	1.31E-37
TRINITY_Contig3948	sp_Q4I8E9_PNS1_GIBZE_Protein_PNS1_OS=Gibberella_zeae_(strain_PH-1_/_ATCC_MYA-4620_/_FGSC_9075_/_NRRL_31084)_OX=229533_GN=PNS1_PE=3_SV=1	29.521	1.97E-35
TRINITY_Contig3963	sp_O95372_LYPA2_HUMAN_Acyl-protein_thioesterase_2_OS=Homo_sapiens_OX=9606_GN=LYPLA2_PE=1_SV=1	44.55	1.69E-59
TRINITY_Contig3970	sp_Q12626_EXG_PICAN_Glucan_1,3-beta-glucosidase_OS=Pichia_angusta_OX=870730_PE=3_SV=1	27.778	6.64E-34
TRINITY_Contig3989	sp_Q3TNA1_XYLB_MOUSE_Xylulose_kinase_OS=Mus_musculus_OX=10090_GN=Xylb_PE=1_SV=1	28.375	4.43E-24
TRINITY_Contig3993	sp_Q5M827_PIR_RAT_Pirin_OS=Rattus_norvegicus_OX=10116_GN=Pir_PE=1_SV=1	48.966	6.97E-90
TRINITY_Contig4006	sp_A0PFK7_CAPZB_PIG_F-actin-capping_protein_subunit_beta_OS=Sus_scrofa_OX=9823_GN=CAPZB_PE=2_SV=1	51.181	2.65E-82
TRINITY_Contig4016	sp_B0XT32_PLYA_ASPFC_Probable_pectate_lyase_A_OS=Neosartorya_fumigata_(strain_CEA10_/_CBS_144.89_/_FGSC_A1163)_OX=451804_GN=plyA_PE=3_SV=1	62.452	2.53E-106
TRINITY_Contig4020	sp_P29787_TRY5_AEDAE_Trypsin_5G1_OS=Aedes_aegypti_OX=7159_GN=AAEL013712_PE=2_SV=2	37.759	9.39E-33
TRINITY_Contig4066	sp_Q54F93_MPPA2_DICDI_Mitochondrial-processing_peptidase_subunit_alpha-2_OS=Dictyostelium_discoideum_OX=44689_GN=mppA2_PE=1_SV=1	27.34	6.98E-19
TRINITY_Contig4093	sp_B3QKG4_RS9_RHOPT_30S_ribosomal_protein_S9_OS=Rhodopseudomonas_palustris_(strain_TIE-1)_OX=395960_GN=rpsI_PE=3_SV=1	54.015	7.73E-38
TRINITY_Contig4097	sp_P97521_MCAT_RAT_Mitochondrial_carnitine/acylcarnitine_carrier_protein_OS=Rattus_norvegicus_OX=10116_GN=Slc25a20_PE=1_SV=1	51.667	1.16E-82
TRINITY_Contig4097	sp_P97521_MCAT_RAT_Mitochondrial_carnitine/acylcarnitine_carrier_protein_OS=Rattus_norvegicus_OX=10116_GN=Slc25a20_PE=1_SV=1	28.061	1.47E-09
TRINITY_Contig4162	sp_P34558_YNP5_CAEEL_Uncharacterized_protein_T05G5.5_OS=Caenorhabditis_elegans_OX=6239_GN=T05G5.5_PE=3_SV=2	48.691	4.98E-58

TRINITY_Contig4303	sp_Q8IWQ3_BRSK2_HUMAN_Serine/threonine-protein_kinase_BRSK2_OS=Homo_sapiens_OX=9606_GN=BRSK2_PE=1_SV=3	31.469	2.25E-37
TRINITY_Contig4707	sp_Q9VXX8_RL371_DROME_Probable_60S_ribosomal_protein_L37-A_OS=Drosophila_melanogaster_OX=7227_GN=RpL37a_PE=1_SV=1	73.913	3.47E-45
TRINITY_Contig4915	sp_Q9LT96_Y5977_ARATH_Probable_leucine-rich_repeat_receptor-like_protein_kinase_At5g49770_OS=Arabidopsis_thaliana_OX=3702_GN=At5g49770_PE=1_SV=1	46.433	0
TRINITY_Contig5133	sp_Q3T0L7_RL28_BOVIN_60S_ribosomal_protein_L28_OS=Bos_taurus_OX=9913_GN=RPL28_PE=2_SV=3	36.22	5.91E-11
TRINITY_Contig5140	sp_Q9SKP9_PRP2_ARATH_Proline-rich_protein_2_OS=Arabidopsis_thaliana_OX=3702_GN=PRP2_PE=2_SV=1	32.512	4.3E-14
TRINITY_Contig5192	sp_P79781_RS27A_CHICK_Ubiquitin-40S_ribosomal_protein_S27a_OS=Gallus_gallus_OX=9031_GN=RPS27A_PE=2_SV=3	84.868	2.44E-74
TRINITY_Contig5358	sp_Q43362_VATL_CHRCT_V-type_proton_ATPase_16_kDa_proteolipid_subunit_OS=Chrysotila_carterae_OX=13221_GN=VAP_PE=2_SV=1	72.393	5.12E-59
TRINITY_Contig5370	sp_Q9FT52_ATP5H_ARATH_ATP_synthase_subunit_d,_mitochondrial_OS=Arabidopsis_thaliana_OX=3702_GN=At3g52300_PE=1_SV=3	29.63	3.51E-10
TRINITY_Contig5385	sp_Q70E96_AL3F1_ARATH_Aldehyde_dehydrogenase_family_3_member_F1_OS=Arabidopsis_thaliana_OX=3702_GN=ALDH3F1_PE=2_SV=2	53.503	0
TRINITY_Contig5388	sp_Q9S7L9_CX6B1_ARATH_Cytochrome_c_oxidase_subunit_6b-1_OS=Arabidopsis_thaliana_OX=3702_GN=COX6B-1_PE=1_SV=1	51.429	1.65E-19
TRINITY_Contig5428	sp_A2RVM0_TIC32_ARATH_Short-chain_dehydrogenase_TIC_32,_chloroplastic_OS=Arabidopsis_thaliana_OX=3702_GN=TIC32_PE=2_SV=1	47.468	1.47E-68
TRINITY_Contig5459	sp_Q84Q77_HS17A_ORYSJ_17.9_kDa_class_I_heat_shock_protein_OS=Oryza_sativa_subsp._japonica_OX=39947_GN=HSP17.9A_PE=1_SV=1	87.578	3.47E-79
TRINITY_Contig5505	sp_Q9HZP6_ETFB_PSEAE_Electron_transfer_flavoprotein_subunit_beta_OS=Pseudomonas_aeruginosa_(strain_ATCC_15692_/_DSM_22644_/_CIP_104116_/_JCM_14847_/_LMG_12228_/_1C_/_PRS_101_/_PAO1)_OX=208964_GN=etfB_PE=3_SV=1	62.055	7.28E-103
TRINITY_Contig5765	sp_O42706_RL21B_SCHPO_60S_ribosomal_protein_L21-B_OS=Schizosaccharomyces_pombe_(strain_972_/_ATCC_24843)_OX=284812_GN=rpl2102_PE=2_SV=2	59.477	5.27E-60
TRINITY_Contig5773	sp_Q92354_RL24A_SCHPO_60S_ribosomal_protein_L24-A_OS=Schizosaccharomyces_pombe_(strain_972_/_ATCC_24843)_OX=284812_GN=rpl2401_PE=3_SV=1	52.525	5.98E-28
TRINITY_Contig5841	sp_Q67UF5_PDI23_ORYSJ_Protein_disulfide_isomerase-like_2-3_OS=Oryza_sativa_subsp._japonica_OX=39947_GN=PDIL2-3_PE=2_SV=1	90.998	0
TRINITY_Contig5841	sp_Q67UF5_PDI23_ORYSJ_Protein_disulfide_isomerase-like_2-3_OS=Oryza_sativa_subsp._japonica_OX=39947_GN=PDIL2-3_PE=2_SV=1	47.706	1.8E-13
TRINITY_Contig5870	sp_P08772_THN3_HORVU_Leaf-specific_thionin_DB4_OS=Hordeum_vulgare_OX=4513_GN=THI1.3_PE=2_SV=2	62.5	4.34E-48
TRINITY_Contig5876	sp_D0NVH9_PURA_PHYIT_Adenylosuccinate_synthetase_OS=Phytophthora_infestans_(strain_T30-4)_OX=403677_GN=PITG_16736_PE=3_SV=1	80.739	0
TRINITY_Contig5964	sp_O80929_RL361_ARATH_60S_ribosomal_protein_L36-1_OS=Arabidopsis_thaliana_OX=3702_GN=RPL36A_PE=3_SV=1	55.056	3.35E-26
TRINITY_Contig6250	sp_O82197_VP321_ARATH_Vacuolar_protein_sorting-associated_protein_32_homolog_1_OS=Arabidopsis_thaliana_OX=3702_GN=VPS32.1_PE=1_SV=1	83.66	1.51E-65
TRINITY_Contig6292	sp_P30821_P25_SCHPO_P25_protein_OS=Schizosaccharomyces_pombe_(strain_972_/_ATCC_24843)_OX=284812_GN=obr1_PE=1_SV=1	57.732	3.37E-75
TRINITY_Contig6397	sp_Q5UNY4_YL728_MIMIV_Uncharacterized_protein_L728_OS=Acanthamoeba_polyphaga_mimivirus_OX=212035_GN=MIMI_L728_PE=4_SV=1	33.333	4.39E-58
TRINITY_Contig6407	sp_Q3BAI2_YCX91_PHAAO_Uncharacterized_protein_ORF91_OS=Phalaenopsis_aphrodite_subsp._formosana_OX=308872_PE=4_SV=1	67.241	9.5E-19
TRINITY_Contig6427	sp_O23193_CBSX1_ARATH_CBS_domain-containing_protein_CBSX1,_chloroplastic_OS=Arabidopsis_thaliana_OX=3702_GN=CBSX1_PE=1_SV=2	68.263	1.97E-78
TRINITY_Contig6512	sp_Q6Z4N6_40G2_ORYSJ_Ricin_B-like_lectin_R40G2_OS=Oryza_sativa_subsp._japonica_OX=39947_GN=R40G2_PE=2_SV=1	82.029	0
TRINITY_Contig6526	sp_G3FIN9_UGTK5_MANES_Linamarin_synthase_2_OS=Manihot_esculenta_OX=3983_GN=UGT85K5_PE=1_SV=1	48.256	6.24E-52
TRINITY_Contig6727	sp_P31209_PABP_SCHPO_Polyadenylate-binding_protein,_cytoplasmic_and_nuclear_OS=Schizosaccharomyces_pombe_(strain_972_/_ATCC_24843)_OX=284812_GN=pab1_PE=1_SV=2	56.351	7.07E-157
TRINITY_Contig6727	sp_P31209_PABP_SCHPO_Polyadenylate-binding_protein,_cytoplasmic_and_nuclear_OS=Schizosaccharomyces_pombe_(strain_972_/_ATCC_24843)_OX=284812_GN=pab1_PE=1_SV=2	51.724	2.77E-08
TRINITY_Contig6747	sp_Q7KLV9_PSD11_DROME_26S_proteasome_non-ATPase_regulatory_subunit_11_OS=Drosophila_melanogaster_OX=7227_GN=Rpn6_PE=1_SV=1	51.436	5.56E-134
TRINITY_Contig6759	sp_A2XT03_PI21_ORYSI_Protein_PYRICULARIA_ORYZAE_RESISTANCE_21_OS=Oryza_sativa_subsp._indica_OX=39946_GN=PI21_PE=4_SV=1	56.923	3.23E-14
TRINITY_Contig6883	sp_Q10B67_MAN4_ORYSJ_Mannan_endo-1,4-beta-mannosidase_4_OS=Oryza_sativa_subsp._japonica_OX=39947_GN=MAN4_PE=2_SV=2	73.575	4.64E-75
TRINITY_Contig6894	sp_C4KZL3_RL13_EXISA_50S_ribosomal_protein_L13_OS=Exiguobacterium_sp._(strain_ATCC_BAA-1283_/_AT1b)_OX=360911_GN=rplM_PE=3_SV=1	49.265	4.33E-44

TRINITY_Contig6898	sp_O24143_NDUS8_TOBAC_NADH_dehydrogenase_[ubiquinone]_iron-sulfur_protein_8,_mitochondrial_OS=Nicotiana_tabacum_OX=4097_PE=2_SV=1	62.5	1.26E-96
TRINITY_Contig6899	sp_Q28IL6_RL22_XENTR_60S_ribosomal_protein_L22_OS=Xenopus_tropicalis_OX=8364_GN=rpl22_PE=2_SV=3	63.636	5.14E-24
TRINITY_Contig6932	sp_Q9FLX7_NDUA5_ARATH_Probable_NADH_dehydrogenase_[ubiquinone]_1_alpha_subcomplex_subunit_5,_mitochondrial_OS=Arabidopsis_thaliana_OX=3702_GN=At5g52840_PE=1_SV=1	50.98	5.46E-26
TRINITY_Contig6935	sp_B8GQQ2_RL27_THISH_50S_ribosomal_protein_L27_OS=Thioalkalivibrio_sulfidiphilus_(strain_HL-EbGR7)_OX=396588_GN=rpmA_PE=3_SV=1	68.293	1.89E-32
TRINITY_Contig6971	sp_Q69CJ9_RL35_OPHHA_60S_ribosomal_protein_L35_OS=Ophiophagus_hannah_OX=8665_GN=RPL35_PE=2_SV=3	68.595	1.32E-25
TRINITY_Contig6991	sp_Q8H0Y8_WRK41_ARATH_Probable_WRKY_transcription_factor_41_OS=Arabidopsis_thaliana_OX=3702_GN=WRKY41_PE=2_SV=2	48.765	8.97E-33
TRINITY_Contig7272	sp_Q3BAI2_YCX91_PHAAO_Uncharacterized_protein_ORF91_OS=Phalaenopsis_aphrodite_subsp._formosana_OX=308872_PE=4_SV=1	69.643	9.8E-14
TRINITY_Contig7275	sp_O31463_HMT_BACSU_Homocysteine_S-methyltransferase_YbgG_OS=Bacillus_subtilis_(strain_168)_OX=224308_GN=ybgG_PE=4_SV=1	40.836	3.51E-65
TRINITY_Contig7277	sp_Q8TGM5_ART3_YEAST_Putative_uncharacterized_protein_ART3_OS=Saccharomyces_cerevisiae_(strain_ATCC_204508_/_S288c)_OX=559292_GN=ART3_PE=5_SV=1	89.362	1.69E-19
TRINITY_Contig7290	sp_A8QW51_OMT2_SORBI_Probable_O-methyltransferase_2_OS=Sorghum_bicolor_OX=4558_GN=OMT2_PE=2_SV=1	79.893	0
TRINITY_Contig7516	sp_Q6BDZ9_KSL8_ORYSJ_Stemar-13-ene_synthase_OS=Oryza_sativa_subsp._japonica_OX=39947_GN=KSL8_PE=2_SV=2	55.112	7.51E-128
TRINITY_Contig7516	sp_Q6BDZ9_KSL8_ORYSJ_Stemar-13-ene_synthase_OS=Oryza_sativa_subsp._japonica_OX=39947_GN=KSL8_PE=2_SV=2	43.229	1.42E-78
TRINITY_Contig7533	sp_Q06398_GSTU6_ORYSJ_Probable_glutathione_S-transferase_GSTU6_OS=Oryza_sativa_subsp._japonica_OX=39947_GN=GSTU6_PE=2_SV=2	54.622	1.27E-51
TRINITY_Contig7535	sp_P18445_RL27A_RAT_60S_ribosomal_protein_L27a_OS=Rattus_norvegicus_OX=10116_GN=Rpl27a_PE=1_SV=3	70.161	3.35E-60
TRINITY_Contig7549	sp_Q9MUW8_MATK_PANCA_Maturase_K_OS=Panicum_capillare_OX=33109_GN=matK_PE=3_SV=1	99.018	0
TRINITY_Contig7587	sp_P46291_RL38_SOLLC_60S_ribosomal_protein_L38_OS=Solanum_lycopersicum_OX=4081_GN=RPL38_PE=3_SV=1	73.529	1.45E-27
TRINITY_Contig7974	sp_Q9LMN8_WAK3_ARATH_Wall-associated_receptor_kinase_3_OS=Arabidopsis_thaliana_OX=3702_GN=WAK3_PE=2_SV=2	44.276	2.03E-118
TRINITY_Contig8037	sp_P00827_ATPB_MAIZE_ATP_synthase_subunit_beta,_chloroplastic_OS=Zea_mays_OX=4577_GN=atpB_PE=3_SV=1	98.594	0
TRINITY_Contig8093	sp_Q6K1S6_MYB30_ORYSJ_Transcription_factor_MYB30_OS=Oryza_sativa_subsp._japonica_OX=39947_GN=MYB30_PE=1_SV=1	82.353	4.07E-70
TRINITY_Contig8164	sp_Q9SYM5_RHM1_ARATH_Trifunctional_UDP-glucose_4,6-dehydratase/UDP-4-keto-6-deoxy-D-glucose_3,5-epimerase/UDP-4-keto-L-rhamnose-reductase_RHM1_OS=Arabidopsis_thaliana_OX=3702_GN=RHM1_PE=1_SV=1	84.501	0

TRINITY_Contig8236	sp_Q5EAF2_IP5PB_ARATH_Type_IV_inositol_polyphosphate_5-phosphatase_11_OS=Arabidopsis_thaliana_OX=3702_GN=IP5P11_PE=1_SV=1	58.278	6.97E-115
TRINITY_Contig8268	sp_P46225_TPIC_SECCE_Triosephosphate_isomerase,_chloroplastic_OS=Secale_cereale_OX=4550_PE=1_SV=1	88.506	2.36E-170
TRINITY_Contig8306	sp_Q06572_AVP_HORVU_Pyrophosphate-energized_vacuolar_membrane_proton_pump_OS=Hordeum_vulgare_OX=4513_PE=2_SV=2	84.487	0
TRINITY_Contig8306	sp_Q06572_AVP_HORVU_Pyrophosphate-energized_vacuolar_membrane_proton_pump_OS=Hordeum_vulgare_OX=4513_PE=2_SV=2	94.012	0
TRINITY_Contig8378	sp_P62246_RS15A_RAT_40S_ribosomal_protein_S15a_OS=Rattus_norvegicus_OX=10116_GN=Rps15a_PE=1_SV=2	77.692	1.69E-72
TRINITY_Contig8405	sp_Q9FR62_P23A_BRANA_Co-chaperone_protein_p23-1_OS=Brassica_napus_OX=3708_GN=P23-1_PE=1_SV=1	62.963	4.28E-39
TRINITY_Contig8430	sp_O80920_PYL4_ARATH_Abscisic_acid_receptor_PYL4_OS=Arabidopsis_thaliana_OX=3702_GN=PYL4_PE=1_SV=1	71.613	1.8E-53
TRINITY_Contig8531	sp_Q8RWA7_NDADA_ARATH_NADH_dehydrogenase_[ubiquinone]_1_alpha_subcomplex_subunit_13-A_OS=Arabidopsis_thaliana_OX=3702_GN=MEE4_PE=2_SV=1	45.528	5.78E-19
TRINITY_Contig8667	sp_Q9MAP7_SBT35_ARATH_Subtilisin-like_protease_SBT3.5_OS=Arabidopsis_thaliana_OX=3702_GN=SBT3.5_PE=1_SV=1	42.911	3.04E-116
TRINITY_Contig8667	sp_Q9MAP7_SBT35_ARATH_Subtilisin-like_protease_SBT3.5_OS=Arabidopsis_thaliana_OX=3702_GN=SBT3.5_PE=1_SV=1	47.826	1.29E-52
TRINITY_Contig8669	sp_Q9LT96_Y5977_ARATH_Probable_leucine-rich_repeat_receptor-like_protein_kinase_At5g49770_OS=Arabidopsis_thaliana_OX=3702_GN=At5g49770_PE=1_SV=1	50	0
TRINITY_Contig8835	sp_Q23716_EF2_CRYPV_Elongation_factor_2_OS=Cryptosporidium_parvum_OX=5807_PE=3_SV=1	64.808	0
TRINITY_Contig8909	sp_Q9LMN8_WAK3_ARATH_Wall-associated_receptor_kinase_3_OS=Arabidopsis_thaliana_OX=3702_GN=WAK3_PE=2_SV=2	39.528	3.64E-160
TRINITY_Contig9017	sp_Q941F1_PLA15_ARATH_Phospholipase_A1-Igamma1,_chloroplastic_OS=Arabidopsis_thaliana_OX=3702_GN=At1g06800_PE=1_SV=2	52.982	3.12E-134
TRINITY_Contig9052	sp_P13917_7SB1_SOYBN_Basic_7S_globulin_OS=Glycine_max_OX=3847_GN=BG_PE=1_SV=2	33.784	5.61E-14
TRINITY_Contig9143	sp_Q9SLP1_C78A9_ARATH_Cytochrome_P450_78A9_OS=Arabidopsis_thaliana_OX=3702_GN=CYP78A9_PE=1_SV=1	64.842	0
TRINITY_Contig9243	sp_Q9LX85_ZAT8_ARATH_Zinc_finger_protein_ZAT8_OS=Arabidopsis_thaliana_OX=3702_GN=ZAT8_PE=2_SV=1	54.43	5.2E-18
TRINITY_Contig9265	sp_Q2R9D2_Y1176_ORYSJ_B3_domain-containing_protein_Os11g0197600_OS=Oryza_sativa_subsp._japonica_OX=39947_GN=Os11g0197600_PE=2_SV=1	51.09	3.09E-125
TRINITY_Contig9280	sp_Q3BAI2_YCX91_PHAAO_Uncharacterized_protein_ORF91_OS=Phalaenopsis_aphrodite_subsp._formosana_OX=308872_PE=4_SV=1	68.966	3.64E-16
TRINITY_Contig9287	sp_Q43207_FKB70_WHEAT_70_kDa_peptidyl-prolyl_isomerase_OS=Triticum_aestivum_OX=4565_GN=FKBP70_PE=1_SV=1	90.04	0
TRINITY_Contig9287	sp_Q43207_FKB70_WHEAT_70_kDa_peptidyl-prolyl_isomerase_OS=Triticum_aestivum_OX=4565_GN=FKBP70_PE=1_SV=1	36.323	2.19E-27
TRINITY_Contig9385	sp_O23522_PLA14_ARATH_Phospholipase_A1-Ibeta2,_chloroplastic_OS=Arabidopsis_thaliana_OX=3702_GN=At4g16820_PE=1_SV=2	47.639	6.39E-117
TRINITY_Contig9557	sp_Q9ZQG4_U73B5_ARATH_UDP-glycosyltransferase_73B5_OS=Arabidopsis_thaliana_OX=3702_GN=UGT73B5_PE=2_SV=1	31.504	1.66E-58
TRINITY_Contig9566	sp_Q10LR9_DCUP2_ORYSJ_Uroporphyrinogen_decarboxylase_2,_chloroplastic_OS=Oryza_sativa_subsp._japonica_OX=39947_GN=Os03g0337600_PE=3_SV=1	90.127	0
TRINITY_Contig9585	sp_Q9FMX6_DJC76_ARATH_Chaperone_protein_dnaJ_C76,_chloroplastic_OS=Arabidopsis_thaliana_OX=3702_GN=DJC76_PE=2_SV=1	46.804	1.26E-118
TRINITY_Contig9627	sp_Q7UJ69_ILVD_RHOBA_Dihydroxy-acid_dehydratase_OS=Rhodopirellula_baltica_(strain_DSM_10527_/_NCIMB_13988_/_SH1)_OX=243090_GN=ilvD_PE=3_SV=2	72.89	0
TRINITY_Contig9647	sp_Q84TB6_ADF3_ORYSJ_Actin-depolymerizing_factor_3_OS=Oryza_sativa_subsp._japonica_OX=39947_GN=ADF3_PE=1_SV=1	82.609	4.15E-56
TRINITY_Contig9689	sp_Q6YV88_C71Z7_ORYSJ_Ent-cassadiene_hydroxylase_OS=Oryza_sativa_subsp._japonica_OX=39947_GN=CYP71Z7_PE=1_SV=1	59.548	0
TRINITY_Contig9754	sp_Q9FIL7_CRCK1_ARATH_Calmodulin-binding_receptor-like_cytoplasmic_kinase_1_OS=Arabidopsis_thaliana_OX=3702_GN=CRCK1_PE=1_SV=1	63.717	6.04E-92
TRINITY_Contig9774	sp_Q552J0_TCPQ_DICDI_T-complex_protein_1_subunit_theta_OS=Dictyostelium_discoideum_OX=44689_GN=cct8_PE=3_SV=1	51.238	0
TRINITY_Contig9790	sp_Q3Y416_KKCC_CAEEL_Calcium/calmodulin-dependent_protein_kinase_kinase_OS=Caenorhabditis_elegans_OX=6239_GN=ckk-1_PE=1_SV=2	33.693	2.19E-47
TRINITY_Contig9816	sp_Q90512_ODO2_TAKRU_Dihydrolipoyllysine-residue_succinyltransferase_component_of_2-oxoglutarate_dehydrogenase_complex,_mitochondrial_(Fragment)_OS=Takifugu_rubripes_OX=31033_GN=dlst_PE=3_SV=1	55.066	3.04E-81
TRINITY_Contig9816	sp_Q90512_ODO2_TAKRU_Dihydrolipoyllysine-residue_succinyltransferase_component_of_2-oxoglutarate_dehydrogenase_complex,_mitochondrial_(Fragment)_OS=Takifugu_rubripes_OX=31033_GN=dlst_PE=3_SV=1	45.946	0.00000018
TRINITY_Contig9816	sp_Q90512_ODO2_TAKRU_Dihydrolipoyllysine-residue_succinyltransferase_component_of_2-oxoglutarate_dehydrogenase_complex,_mitochondrial_(Fragment)_OS=Takifugu_rubripes_OX=31033_GN=dlst_PE=3_SV=1	45.333	0.00000475
TRINITY_Contig9863	sp_P05307_PDIA1_BOVIN_Protein_disulfide-isomerase_OS=Bos_taurus_OX=9913_GN=P4HB_PE=1_SV=1	43.297	9.45E-114
TRINITY_Contig9863	sp_P05307_PDIA1_BOVIN_Protein_disulfide-isomerase_OS=Bos_taurus_OX=9913_GN=P4HB_PE=1_SV=1	39.344	1.12E-15

TRINITY_Contig9981	sp_Q9M9S6_UTR3_ARATH_UDP-galactose/UDP-glucose_transporter_3_OS=Arabidopsis_thaliana_OX=3702_GN=UTR3_PE=1_SV=1	77.955	6.15E-162
TRINITY_Contig10019	sp_Q9LUI1_LRX6_ARATH_Leucine-rich_repeat_extensin-like_protein_6_OS=Arabidopsis_thaliana_OX=3702_GN=LRX6_PE=2_SV=1	53.776	2.03E-120
TRINITY_Contig10086	sp_P16397_SUBF_BACSU_Bacillopeptidase_F_OS=Bacillus_subtilis_(strain_168)_OX=224308_GN=bpr_PE=1_SV=2	35.092	2.54E-48
TRINITY_Contig10122	sp_Q9M0B8_WTR37_ARATH_WAT1-related_protein_At4g30420_OS=Arabidopsis_thaliana_OX=3702_GN=At4g30420_PE=3_SV=1	42.74	3.25E-95
TRINITY_Contig10184	sp_Q8RWN9_ODP22_ARATH_Dihydrolipoyllysine-residue_acetyltransferase_component_2_of_pyruvate_dehydrogenase_complex,_mitochondrial_OS=Arabidopsis_thaliana_OX=3702_GN=At3g13930_PE=1_SV=2	42.082	3.07E-103
TRINITY_Contig10200	sp_Q9FKK7_XYLA_ARATH_Xylose_isomerase_OS=Arabidopsis_thaliana_OX=3702_GN=XYLA_PE=2_SV=2	52.273	3.73E-158
TRINITY_Contig10204	sp_Q41870_PIP11_MAIZE_Aquaporin_PIP1-1_OS=Zea_mays_OX=4577_GN=PIP1-1_PE=1_SV=1	64.545	2.52E-52
TRINITY_Contig10204	sp_Q41870_PIP11_MAIZE_Aquaporin_PIP1-1_OS=Zea_mays_OX=4577_GN=PIP1-1_PE=1_SV=1	55.422	2.52E-52
TRINITY_Contig10204	sp_Q41870_PIP11_MAIZE_Aquaporin_PIP1-1_OS=Zea_mays_OX=4577_GN=PIP1-1_PE=1_SV=1	62.921	1.37E-27
TRINITY_Contig10213	sp_P46436_GST1_ASCSU_Glutathione_S-transferase_1_OS=Ascaris_suum_OX=6253_GN=GST1_PE=1_SV=3	38.15	9.01E-21
TRINITY_Contig10213	sp_P46436_GST1_ASCSU_Glutathione_S-transferase_1_OS=Ascaris_suum_OX=6253_GN=GST1_PE=1_SV=3	32.407	1.7E-19
TRINITY_Contig10257	sp_Q8CFY5_COX10_MOUSE_Protoheme_IX_farnesyltransferase,_mitochondrial_OS=Mus_musculus_OX=10090_GN=Cox10_PE=2_SV=1	46.591	2.24E-67
TRINITY_Contig10267	sp_Q5ZPI9_SPO12_ORYSI_Meiotic_recombination_protein_SPO11-2_OS=Oryza_sativa_subsp._indica_OX=39946_GN=SPO11-2_PE=1_SV=1	85.638	0
TRINITY_Contig10416	sp_M1CZC0_EBP1_SOLTU_ERBB-3_BINDING_PROTEIN_1_OS=Solanum_tuberosum_OX=4113_GN=EBP1_PE=2_SV=1	50.152	2.94E-108
TRINITY_Contig10432	sp_Q3EAL1_OFP6_ARATH_Transcription_repressor_OFP6_OS=Arabidopsis_thaliana_OX=3702_GN=OFP6_PE=1_SV=1	62.963	4.27E-15
TRINITY_Contig10452	sp_P41367_ACADM_PIG_Medium-chain_specific_acyl-CoA_dehydrogenase,_mitochondrial_OS=Sus_scrofa_OX=9823_GN=ACADM_PE=1_SV=3	66.146	0
TRINITY_Contig10478	sp_Q9AV39_GLO1A_ORYSJ_Very-long-chain_aldehyde_decarbonylase_GL1-10_OS=Oryza_sativa_subsp._japonica_OX=39947_GN=GL1-10_PE=2_SV=1	59.797	2.98E-101
TRINITY_Contig10480	sp_P24792_ASO_CUCMA_L-ascorbate_oxidase_OS=Cucurbita_maxima_OX=3661_GN=AAO_PE=1_SV=2	47.721	4.48E-103
TRINITY_Contig10545	sp_P93788_REMO_SOLTU_Remorin_OS=Solanum_tuberosum_OX=4113_PE=1_SV=1	70.286	5.64E-29
TRINITY_Contig10552	sp_Q01294_CARP_NEUCR_Vacuolar_protease_A_OS=Neurospora_crassa_(strain_ATCC_24698_/_74-OR23-1A_/_CBS_708.71_/_DSM_1257_/_FGSC_987)_OX=367110_GN=pep-4_PE=3_SV=2	39.031	2.7E-75
TRINITY_Contig10665	sp_Q9FLB1_PYL5_ARATH_Abscisic_acid_receptor_PYL5_OS=Arabidopsis_thaliana_OX=3702_GN=PYL5_PE=1_SV=1	65.772	1.79E-51
TRINITY_Contig10678	sp_P22008_P5CR_PSEAE_Pyrroline-5-carboxylate_reductase_OS=Pseudomonas_aeruginosa_(strain_ATCC_15692_/_DSM_22644_/_CIP_104116_/_JCM_14847_/_LMG_12228_/_1C_/_PRS_101_/_PAO1)_OX=208964_GN=proC_PE=1_SV=2	39.925	1.29E-61
TRINITY_Contig10684	sp_Q38799_ODPB1_ARATH_Pyruvate_dehydrogenase_E1_component_subunit_beta-1,_mitochondrial_OS=Arabidopsis_thaliana_OX=3702_GN=PDH2_PE=1_SV=2	71.084	6.63E-173
TRINITY_Contig10698	sp_Q0JCR9_MST1_ORYSJ_Sugar_transport_protein_MST1_OS=Oryza_sativa_subsp._japonica_OX=39947_GN=MST1_PE=2_SV=1	56.512	1.36E-174
TRINITY_Contig10734	sp_Q93ZE8_SDF2_ARATH_Stromal_cell-derived_factor_2-like_protein_OS=Arabidopsis_thaliana_OX=3702_GN=SDF2_PE=1_SV=1	75.978	4.06E-101
TRINITY_Contig10753	sp_Q29471_ANX13_CANLF_Annexin_A13_OS=Canis_lupus_familiaris_OX=9615_GN=ANXA13_PE=1_SV=2	31.833	7.68E-44
TRINITY_Contig10793	sp_Q9FWV6_TIP31_ORYSJ_Probable_aquaporin_TIP3-1_OS=Oryza_sativa_subsp._japonica_OX=39947_GN=TIP3-1_PE=2_SV=1	61.728	2.91E-69
TRINITY_Contig10811	sp_P38661_PDIA6_MEDSA_Probable_protein_disulfide-isomerase_A6_OS=Medicago_sativa_OX=3879_PE=2_SV=1	48.943	5.88E-96
TRINITY_Contig10832	sp_F2VYU4_PIK1_ORYSJ_Disease_resistance_protein_Pik-1_OS=Oryza_sativa_subsp._japonica_OX=39947_GN=PIK-1_PE=1_SV=1	52.564	9.91E-14
TRINITY_Contig10863	sp_P24639_ANXA7_DICDI_Annexin_A7_OS=Dictyostelium_discoideum_OX=44689_GN=nxnA_PE=1_SV=1	36.156	5.21E-44
TRINITY_Contig10863	sp_P24639_ANXA7_DICDI_Annexin_A7_OS=Dictyostelium_discoideum_OX=44689_GN=nxnA_PE=1_SV=1	32.414	1.67E-09
TRINITY_Contig10927	sp_O01666_ATPG_DROME_ATP_synthase_subunit_gamma,_mitochondrial_OS=Drosophila_melanogaster_OX=7227_GN=ATPsyngamma_PE=2_SV=2	41.549	1.85E-61
TRINITY_Contig10929	sp_P62282_RS11_RAT_40S_ribosomal_protein_S11_OS=Rattus_norvegicus_OX=10116_GN=Rps11_PE=1_SV=3	63.58	3.97E-66
TRINITY_Contig10944	sp_P29421_IAAS_ORYSJ_Alpha-amylase/subtilisin_inhibitor_OS=Oryza_sativa_subsp._japonica_OX=39947_GN=RASI_PE=1_SV=2	55.814	1.02E-45
TRINITY_Contig11045	sp_P25387_GBLP_CHLRE_Guanine_nucleotide-binding_protein_subunit_beta-like_protein_OS=Chlamydomonas_reinhardtii_OX=3055_GN=GBLP_PE=2_SV=1	74.295	3.9E-168
TRINITY_Contig11056	sp_Q5XFW8_SEC13_RAT_Protein_SEC13_homolog_OS=Rattus_norvegicus_OX=10116_GN=Sec13_PE=1_SV=1	50.637	6.38E-105
TRINITY_Contig11062	sp_A8IB25_RSSA_CHLRE_40S_ribosomal_protein_SA_OS=Chlamydomonas_reinhardtii_OX=3055_GN=CHLREDRAFT_126059_PE=3_SV=1	68.528	5.63E-99
TRINITY_Contig11088	sp_Q16698_DECR_HUMAN_2,4-dienoyl-CoA_reductase,_mitochondrial_OS=Homo_sapiens_OX=9606_GN=DECR1_PE=1_SV=1	58.099	2.73E-104

TRINITY_Contig11110	sp_Q06398_GSTU6_ORYSJ_Probable_glutathione_S-transferase_GSTU6_OS=Oryza_sativa_subsp._japonica_OX=39947_GN=GSTU6_PE=2_SV=2	57.965	2.26E-46
TRINITY_Contig11121	sp_P31009_RS2_DROME_40S_ribosomal_protein_S2_OS=Drosophila_melanogaster_OX=7227_GN=RpS2_PE=1_SV=2	76.471	6.02E-123
TRINITY_Contig11151	sp_Q851K1_GL36_ORYSJ_Germin-like_protein_3-6_OS=Oryza_sativa_subsp._japonica_OX=39947_GN=Os03g0694000_PE=2_SV=1	81.659	1.64E-130
TRINITY_Contig11236	sp_Q852K6_SAP7_ORYSJ_Zinc_finger_A20_and_AN1_domain-containing_stress-associated_protein_7_OS=Oryza_sativa_subsp._japonica_OX=39947_GN=SAP7_PE=2_SV=1	34.932	4.71E-15
TRINITY_Contig11237	sp_P51412_RK21_ARATH_50S_ribosomal_protein_L21,_chloroplastic_OS=Arabidopsis_thaliana_OX=3702_GN=RPL21_PE=2_SV=1	76.147	1.31E-45
TRINITY_Contig11238	sp_Q8TGM6_TAR1_YEAST_Protein_TAR1_OS=Saccharomyces_cerevisiae_(strain_ATCC_204508_/_S288c)_OX=559292_GN=TAR1_PE=2_SV=1	51.429	8.83E-13
TRINITY_Contig11252	sp_P53575_ETFB_BRADU_Electron_transfer_flavoprotein_subunit_beta_OS=Bradyrhizobium_diazoefficiens_(strain_JCM_10833_/_IAM_13628_/_NBRC_14792_/_USDA_110)_OX=224911_GN=etfB_PE=3_SV=2	63.241	5.14E-104
TRINITY_Contig11266	sp_Q9LRN8_RK3B_ARATH_50S_ribosomal_protein_L3-2,_chloroplastic_OS=Arabidopsis_thaliana_OX=3702_GN=RPL3B_PE=2_SV=1	51.383	5.19E-82
TRINITY_Contig11289	sp_Q3BAI2_YCX91_PHAAO_Uncharacterized_protein_ORF91_OS=Phalaenopsis_aphrodite_subsp._formosana_OX=308872_PE=4_SV=1	65.789	0.000000227
TRINITY_Contig11296	sp_O81304_PAP11_ARATH_Probable_plastid-lipid-associated_protein_11,_chloroplastic_OS=Arabidopsis_thaliana_OX=3702_GN=PAP11_PE=2_SV=1	61.494	1.01E-73
TRINITY_Contig11305	sp_Q60E70_COBL3_ORYSJ_COBRA-like_protein_3_OS=Oryza_sativa_subsp._japonica_OX=39947_GN=BC1L4_PE=2_SV=1	89.976	0
TRINITY_Contig11363	sp_Q94A08_RFS2_ARATH_Probable_galactinol--sucrose_galactosyltransferase_2_OS=Arabidopsis_thaliana_OX=3702_GN=RFS2_PE=2_SV=2	57.774	0
TRINITY_Contig11431	sp_P29787_TRY5_AEDAE_Trypsin_5G1_OS=Aedes_aegypti_OX=7159_GN=AAEL013712_PE=2_SV=2	37.131	2.25E-37
TRINITY_Contig11435	sp_P29787_TRY5_AEDAE_Trypsin_5G1_OS=Aedes_aegypti_OX=7159_GN=AAEL013712_PE=2_SV=2	36.017	6.95E-32
TRINITY_Contig11454	sp_P82968_MCPI_MELCP_Four-domain_proteases_inhibitor_OS=Melithaea_caledonica_OX=156534_PE=1_SV=1	35.762	1.06E-11
TRINITY_Contig11474	sp_P85971_6PGL_RAT_6-phosphogluconolactonase_OS=Rattus_norvegicus_OX=10116_GN=Pgls_PE=1_SV=1	45.378	1.81E-55
TRINITY_Contig11475	sp_Q9SIP7_RS31_ARATH_40S_ribosomal_protein_S3-1_OS=Arabidopsis_thaliana_OX=3702_GN=RPS3A_PE=1_SV=1	78.341	7.11E-120
TRINITY_Contig11483	sp_A1UTH4_RL25_BARBK_50S_ribosomal_protein_L25_OS=Bartonella_bacilliformis_(strain_ATCC_35685_/_KC583)_OX=360095_GN=rplY_PE=3_SV=1	34.375	3.27E-31
TRINITY_Contig11504	sp_P34120_PSA7_DICDI_Proteasome_subunit_alpha_type-7_OS=Dictyostelium_discoideum_OX=44689_GN=psmA7_PE=3_SV=1	64.378	1.36E-101
TRINITY_Contig11536	sp_Q8RT64_RRF_BARBK_Ribosome-recycling_factor_OS=Bartonella_bacilliformis_(strain_ATCC_35685_/_KC583)_OX=360095_GN=frr_PE=3_SV=1	35.45	1.84E-21
TRINITY_Contig11541	sp_Q9FVR6_Y1222_ARATH_Uncharacterized_protein_At1g32220,_chloroplastic_OS=Arabidopsis_thaliana_OX=3702_GN=At1g32220_PE=1_SV=1	43.827	1.86E-31
TRINITY_Contig11545	sp_Q8VZB9_R10A1_ARATH_60S_ribosomal_protein_L10a-1_OS=Arabidopsis_thaliana_OX=3702_GN=RPL10AA_PE=1_SV=1	62.115	4.65E-86
TRINITY_Contig11585	sp_A9W4S1_RL15_METEP_50S_ribosomal_protein_L15_OS=Methylobacterium_extorquens_(strain_PA1)_OX=419610_GN=rplO_PE=3_SV=1	53.797	6.6E-38
TRINITY_Contig11635	sp_P41920_YRB1_YEAST_Ran-specific_GTPase-activating_protein_1_OS=Saccharomyces_cerevisiae_(strain_ATCC_204508_/_S288c)_OX=559292_GN=YRB1_PE=1_SV=1	51.748	2.99E-35
TRINITY_Contig11674	sp_Q5R9R4_RL7_PONAB_60S_ribosomal_protein_L7_OS=Pongo_abelii_OX=9601_GN=RPL7_PE=2_SV=1	61.966	2.15E-98
TRINITY_Contig11715	sp_Q9XI91_IF5A1_ARATH_Eukaryotic_translation_initiation_factor_5A-1_OS=Arabidopsis_thaliana_OX=3702_GN=ELF5A-1_PE=1_SV=1	60	1.14E-62
TRINITY_Contig11736	sp_Q02921_NO93_SOYBN_Early_nodulin-93_OS=Glycine_max_OX=3847_PE=2_SV=1	70.455	4.44E-25
TRINITY_Contig11750	sp_Q9CAX6_RS142_ARATH_40S_ribosomal_protein_S14-2_OS=Arabidopsis_thaliana_OX=3702_GN=RPS14B_PE=1_SV=1	84.677	3.96E-76
TRINITY_Contig11776	sp_Q9LL85_WHY1_SOLTU_Single-stranded_DNA-binding_protein_WHY1,_chloroplastic_OS=Solanum_tuberosum_OX=4113_GN=WHY1_PE=1_SV=1	28.488	0.000000403
TRINITY_Contig11777	sp_Q8S0S6_G2OX3_ORYSJ_Gibberellin_2-beta-dioxygenase_3_OS=Oryza_sativa_subsp._japonica_OX=39947_GN=GA2OX3_PE=1_SV=1	67.857	1.74E-35
TRINITY_Contig11791	sp_O07347_SRP54_THEAQ_Signal_recognition_particle_protein_OS=Thermus_aquaticus_OX=271_GN=ffh_PE=1_SV=3	27.778	0.000000497
TRINITY_Contig11814	sp_Q3BAI2_YCX91_PHAAO_Uncharacterized_protein_ORF91_OS=Phalaenopsis_aphrodite_subsp._formosana_OX=308872_PE=4_SV=1	76.471	1.01E-15
TRINITY_Contig11862	sp_Q04HQ3_RL9_OENOB_50S_ribosomal_protein_L9_OS=Oenococcus_oeni_(strain_ATCC_BAA-331_/_PSU-1)_OX=203123_GN=rplI_PE=3_SV=1	36.567	7.07E-17
TRINITY_Contig11865	sp_P50433_GLYM_SOLTU_Serine_hydroxymethyltransferase,_mitochondrial_OS=Solanum_tuberosum_OX=4113_PE=2_SV=1	87.14	0
TRINITY_Contig11874	sp_Q43844_NDUS7_SOLTU_NADH_dehydrogenase_[ubiquinone]_iron-sulfur_protein_7,_mitochondrial_OS=Solanum_tuberosum_OX=4113_PE=2_SV=1	73.913	2.36E-88
TRINITY_Contig11879	sp_Q75C01_GRPE_ASHGO_GrpE_protein_homolog,_mitochondrial_OS=Ashbya_gossypii_(strain_ATCC_10895_/_CBS_109.51_/_FGSC_9923_/_NRRL_Y-1056)_OX=284811_GN=mge1_PE=3_SV=1	42.222	5.4E-28
TRINITY_Contig11886	sp_Q9T0I5_PRP4_ARATH_Proline-rich_protein_4_OS=Arabidopsis_thaliana_OX=3702_GN=PRP4_PE=2_SV=1	40.698	6.4E-09

TRINITY_Contig11922	sp_Q84Q77_HS17A_ORYSJ_17.9_kDa_class_I_heat_shock_protein_OS=Oryza_sativa_subsp._japonica_OX=39947_GN=HSP17.9A_PE=1_SV=1	80.255	2.02E-58
TRINITY_Contig11942	sp_B7F7B9_SPSA2_ORYSJ_Probable_sucrose-phosphate_synthase_2_OS=Oryza_sativa_subsp._japonica_OX=39947_GN=SPS2_PE=2_SV=2	93.252	0
TRINITY_Contig11958	sp_Q9C7U5_E132_ARATH_Glucan_endo-1,3-beta-glucosidase_2_OS=Arabidopsis_thaliana_OX=3702_GN=At1g66250_PE=2_SV=2	38.288	9.15E-74
TRINITY_Contig11958	sp_Q9C7U5_E132_ARATH_Glucan_endo-1,3-beta-glucosidase_2_OS=Arabidopsis_thaliana_OX=3702_GN=At1g66250_PE=2_SV=2	31.792	1.06E-14
TRINITY_Contig11958	sp_Q9C7U5_E132_ARATH_Glucan_endo-1,3-beta-glucosidase_2_OS=Arabidopsis_thaliana_OX=3702_GN=At1g66250_PE=2_SV=2	40.476	6.61E-14
TRINITY_Contig12001	sp_P49633_RL40_ACACA_Ubiquitin-60S_ribosomal_protein_L40_OS=Acanthamoeba_castellanii_OX=5755_PE=2_SV=2	38.158	1.11E-09
TRINITY_Contig12026	sp_Q3BAI2_YCX91_PHAAO_Uncharacterized_protein_ORF91_OS=Phalaenopsis_aphrodite_subsp._formosana_OX=308872_PE=4_SV=1	71.053	0.000000453
TRINITY_Contig12088	sp_Q6K9A2_CCR1_ORYSJ_Cinnamoyl-CoA_reductase_1_OS=Oryza_sativa_subsp._japonica_OX=39947_GN=CCR1_PE=1_SV=1	88.037	0
TRINITY_Contig12157	sp_P61866_RL12_CAEEL_60S_ribosomal_protein_L12_OS=Caenorhabditis_elegans_OX=6239_GN=rpl-12_PE=3_SV=1	68.098	1.9E-74
TRINITY_Contig12180	sp_P62752_RL23A_RAT_60S_ribosomal_protein_L23a_OS=Rattus_norvegicus_OX=10116_GN=Rpl23a_PE=2_SV=1	75.397	1.53E-59
TRINITY_Contig12433	sp_Q962Q5_RS25_SPOFR_40S_ribosomal_protein_S25_OS=Spodoptera_frugiperda_OX=7108_GN=RpS25_PE=3_SV=1	53.846	8.19E-24
TRINITY_Contig12434	sp_Q9LTS6_RS16B_ARATH_30S_ribosomal_protein_S16-2,_chloroplastic/mitochondrial_OS=Arabidopsis_thaliana_OX=3702_GN=RPS16-2_PE=2_SV=1	56.322	2.17E-31
TRINITY_Contig12575	sp_O96269_RS30_PLAF7_40S_ribosomal_protein_S30_OS=Plasmodium_falciparum_(isolate_3D7)_OX=36329_GN=RPS30_PE=1_SV=1	72.881	7.8E-17
TRINITY_Contig12674	sp_Q3BAI2_YCX91_PHAAO_Uncharacterized_protein_ORF91_OS=Phalaenopsis_aphrodite_subsp._formosana_OX=308872_PE=4_SV=1	74.074	8.08E-17
TRINITY_Contig12714	sp_Q9LKA5_MORF8_ARATH_Multiple_organellar_RNA_editing_factor_8,_chloroplastic/mitochondrial_OS=Arabidopsis_thaliana_OX=3702_GN=MORF8_PE=1_SV=1	80.11	3.37E-75
TRINITY_Contig12789	sp_Q39613_CYPH_CATRO_Peptidyl-prolyl_cis-trans_isomerase_OS=Catharanthus_roseus_OX=4058_GN=PCKR1_PE=1_SV=1	62.644	6.98E-75
TRINITY_Contig12895	sp_Q9LXF8_AVT1J_ARATH_Amino_acid_transporter_AVT1J_OS=Arabidopsis_thaliana_OX=3702_GN=AVT1J_PE=2_SV=1	47.619	4.92E-93
TRINITY_Contig12983	sp_Q65XS5_BC10_ORYSJ_Glycosyltransferase_BC10_OS=Oryza_sativa_subsp._japonica_OX=39947_GN=BC10_PE=1_SV=1	36.213	8.23E-32
TRINITY_Contig13050	sp_Q9LYU3_EF113_ARATH_Ethylene-responsive_transcription_factor_ERF113_OS=Arabidopsis_thaliana_OX=3702_GN=ERF113_PE=2_SV=1	56.452	0.00000121
TRINITY_Contig13103	sp_Q8S9J6_ASPA_ARATH_Aspartyl_protease_family_protein_At5g10770_OS=Arabidopsis_thaliana_OX=3702_GN=At5g10770_PE=2_SV=1	35.682	6.97E-71
TRINITY_Contig13120	sp_C0LGH2_Y1561_ARATH_Probable_LRR_receptor-like_serine/threonine-protein_kinase_At1g56130_OS=Arabidopsis_thaliana_OX=3702_GN=At1g56130_PE=2_SV=2	53.493	0
TRINITY_Contig13133	sp_Q39096_ERD15_ARATH_Protein_EARLY_RESPONSIVE_TO_DEHYDRATION_15_OS=Arabidopsis_thaliana_OX=3702_GN=ERD15_PE=1_SV=1	69.048	9.18E-13
TRINITY_Contig13158	sp_Q04ZJ3_RIMP_LEPBL_Ribosome_maturation_factor_RimP_OS=Leptospira_borgpetersenii_serovar_Hardjo-bovis_(strain_L550)_OX=355276_GN=rimP_PE=3_SV=1	37.363	2.65E-08
TRINITY_Contig13250	sp_P24632_HSP22_MAIZE_17.8_kDa_class_II_heat_shock_protein_OS=Zea_mays_OX=4577_PE=2_SV=1	85.366	4.24E-74
TRINITY_Contig13338	sp_A2XFI3_PDC2_ORYSI_Pyruvate_decarboxylase_2_OS=Oryza_sativa_subsp._indica_OX=39946_GN=PDC2_PE=2_SV=2	95.238	0
TRINITY_Contig13523	sp_Q9ARD5_LT02_HORVU_Low_temperature-induced_protein_lt101.2_OS=Hordeum_vulgare_OX=4513_GN=LT101.2_PE=2_SV=1	81.481	1.56E-21
TRINITY_Contig13538	sp_Q8BH95_ECHM_MOUSE_Enoyl-CoA_hydratase,_mitochondrial_OS=Mus_musculus_OX=10090_GN=Echs1_PE=1_SV=1	64.103	3.4E-122
TRINITY_Contig13563	sp_Q3BAI2_YCX91_PHAAO_Uncharacterized_protein_ORF91_OS=Phalaenopsis_aphrodite_subsp._formosana_OX=308872_PE=4_SV=1	58.824	9.84E-21
TRINITY_Contig13657	sp_Q9SK27_ENL1_ARATH_Early_nodulin-like_protein_1_OS=Arabidopsis_thaliana_OX=3702_GN=At2g25060_PE=2_SV=2	40.678	3.02E-24
TRINITY_Contig13705	sp_Q00058_MI25_ORYSI_ATP_synthase_protein_MI25_OS=Oryza_sativa_subsp._indica_OX=39946_PE=3_SV=1	91.371	4.74E-115
TRINITY_Contig13804	sp_Q8H0M1_QORH_SPIOL_Quinone-oxidoreductase_homolog,_chloroplastic_OS=Spinacia_oleracea_OX=3562_GN=QOR_PE=1_SV=1	65.041	8.17E-38
TRINITY_Contig13864	sp_P12329_CB21_MAIZE_Chlorophyll_a-b_binding_protein_1,_chloroplastic_OS=Zea_mays_OX=4577_GN=CAB1_PE=1_SV=1	86.01	1.64E-117
TRINITY_Contig13935	sp_P35684_RL3_ORYSJ_60S_ribosomal_protein_L3_OS=Oryza_sativa_subsp._japonica_OX=39947_GN=RPL3_PE=2_SV=2	69.152	0
TRINITY_Contig13980	sp_Q8LB81_GDL79_ARATH_GDSL_esterase/lipase_At5g33370_OS=Arabidopsis_thaliana_OX=3702_GN=At5g33370_PE=2_SV=1	76.705	3.33E-104
TRINITY_Contig13980	sp_Q8LB81_GDL79_ARATH_GDSL_esterase/lipase_At5g33370_OS=Arabidopsis_thaliana_OX=3702_GN=At5g33370_PE=2_SV=1	55.208	3.33E-104
TRINITY_Contig14036	sp_Q9SL67_PRS4B_ARATH_26S_proteasome_regulatory_subunit_4_homolog_B_OS=Arabidopsis_thaliana_OX=3702_GN=RPT2B_PE=1_SV=1	78.924	0
TRINITY_Contig14050	sp_Q8LBK6_GRS15_ARATH_Monothiol_glutaredoxin-S15,_mitochondrial_OS=Arabidopsis_thaliana_OX=3702_GN=GRXS15_PE=1_SV=2	41.558	1.23E-36

TRINITY_Contig14073	sp_P37830_G6PD_SOLTU_Glucose-6-phosphate_1-dehydrogenase,_cytoplasmic_isoform_OS=Solanum_tuberosum_OX=4113_GN=G6PDH_PE=2_SV=1	79.596	0
TRINITY_Contig14100	sp_Q26365_ADT_DROME_ADP,ATP_carrier_protein_OS=Drosophila_melanogaster_OX=7227_GN=sesB_PE=2_SV=4	73.559	2.59E-145
TRINITY_Contig14114	sp_Q6L4H4_Y5498_ORYSJ_AP2/ERF_and_B3_domain-containing_protein_Os05g0549800_OS=Oryza_sativa_subsp._japonica_OX=39947_GN=Os05g0549800_PE=2_SV=1	84.375	3.71E-17
TRINITY_Contig14131	sp_Q65X92_C3H37_ORYSJ_Zinc_finger_CCCH_domain-containing_protein_37_OS=Oryza_sativa_subsp._japonica_OX=39947_GN=Os05g0525900_PE=2_SV=1	81.188	9.39E-70
TRINITY_Contig14251	sp_Q6K8J4_ISPG_ORYSJ_4-hydroxy-3-methylbut-2-en-1-yl_diphosphate_synthase_(ferredoxin),_chloroplastic_OS=Oryza_sativa_subsp._japonica_OX=39947_GN=ISPG_PE=2_SV=1	95.174	0
TRINITY_Contig14256	sp_Q3BAI2_YCX91_PHAAO_Uncharacterized_protein_ORF91_OS=Phalaenopsis_aphrodite_subsp._formosana_OX=308872_PE=4_SV=1	57.471	7.76E-20
TRINITY_Contig14402	sp_Q7XP65_G2OX6_ORYSJ_Gibberellin_2-beta-dioxygenase_6_OS=Oryza_sativa_subsp._japonica_OX=39947_GN=GA2OX6_PE=1_SV=1	67.412	1.07E-141
TRINITY_Contig14472	sp_Q7XQ12_AMT11_ORYSJ_Ammonium_transporter_1_member_1_OS=Oryza_sativa_subsp._japonica_OX=39947_GN=AMT1-1_PE=2_SV=1	93.429	0
TRINITY_Contig14514	sp_O82528_RL15_PETHY_60S_ribosomal_protein_L15_OS=Petunia_hybrida_OX=4102_GN=RPL15_PE=2_SV=1	72.139	1.02E-89
TRINITY_Contig14629	sp_Q6RYA0_SABP2_TOBAC_Salicylic_acid-binding_protein_2_OS=Nicotiana_tabacum_OX=4097_GN=SABP2_PE=1_SV=1	53.333	1.77E-64
TRINITY_Contig14655	sp_A2YNP2_TI10B_ORYSI_Protein_TIFY_10b_OS=Oryza_sativa_subsp._indica_OX=39946_GN=TIFY10B_PE=3_SV=1	72.951	6.25E-100
TRINITY_Contig14673	sp_Q96520_PER12_ARATH_Peroxidase_12_OS=Arabidopsis_thaliana_OX=3702_GN=PER12_PE=1_SV=1	51.475	6.35E-99
TRINITY_Contig14673	sp_Q96520_PER12_ARATH_Peroxidase_12_OS=Arabidopsis_thaliana_OX=3702_GN=PER12_PE=1_SV=1	49.367	6.52E-15
TRINITY_Contig14797	sp_P0C343_NDHK_ORYSJ_NAD(P)H-quinone_oxidoreductase_subunit_K,_chloroplastic_OS=Oryza_sativa_subsp._japonica_OX=39947_GN=ndhK_PE=3_SV=2	96.018	1.18E-142
TRINITY_Contig15094	sp_F2VYU4_PIK1_ORYSJ_Disease_resistance_protein_Pik-1_OS=Oryza_sativa_subsp._japonica_OX=39947_GN=PIK-1_PE=1_SV=1	60.606	1.3E-12
TRINITY_Contig15157	sp_O03982_HIP39_ARATH_Heavy_metal-associated_isoprenylated_plant_protein_39_OS=Arabidopsis_thaliana_OX=3702_GN=HIPP39_PE=2_SV=1	50	0.0000028
TRINITY_Contig15201	sp_Q9XGL4_RL31_CYAPA_60S_ribosomal_protein_L31_OS=Cyanophora_paradoxa_OX=2762_GN=RPL31_PE=3_SV=1	74.312	5.49E-55
TRINITY_Contig15239	sp_P93099_RL13A_CYAPA_60S_ribosomal_protein_L13a_OS=Cyanophora_paradoxa_OX=2762_PE=2_SV=1	61.307	2.74E-83
TRINITY_Contig15352	sp_O04647_PP399_ARATH_Pentatricopeptide_repeat-containing_protein_At5g27270_OS=Arabidopsis_thaliana_OX=3702_GN=EMB976_PE=2_SV=2	48.394	0
TRINITY_Contig15369	sp_Q6IEL0_WRK71_ORYSI_WRKY_transcription_factor_WRKY71_OS=Oryza_sativa_subsp._indica_OX=39946_GN=WRKY71_PE=1_SV=1	68.306	5.71E-74
TRINITY_Contig15483	sp_Q9LY50_ACCR3_ARATH_Putative_serine/threonine-protein_kinase-like_protein_CCR3_OS=Arabidopsis_thaliana_OX=3702_GN=CCR3_PE=2_SV=1	38.632	1.99E-88
TRINITY_Contig15483	sp_Q9LY50_ACCR3_ARATH_Putative_serine/threonine-protein_kinase-like_protein_CCR3_OS=Arabidopsis_thaliana_OX=3702_GN=CCR3_PE=2_SV=1	41.892	1.99E-88
TRINITY_Contig15543	sp_Q9SJL0_U86A1_ARATH_UDP-glycosyltransferase_86A1_OS=Arabidopsis_thaliana_OX=3702_GN=UGT86A1_PE=2_SV=1	54.393	0
TRINITY_Contig15699	sp_Q6K9N6_SUCB_ORYSJ_Succinate--CoA_ligase_[ADP-forming]_subunit_beta,_mitochondrial_OS=Oryza_sativa_subsp._japonica_OX=39947_GN=Os02g0621700_PE=1_SV=1	95.377	0
TRINITY_Contig15773	sp_P34106_ALA2_PANMI_Alanine_aminotransferase_2_OS=Panicum_miliaceum_OX=4540_PE=1_SV=1	97.303	0
TRINITY_Contig15784	sp_P19804_NDKB_RAT_Nucleoside_diphosphate_kinase_B_OS=Rattus_norvegicus_OX=10116_GN=Nme2_PE=1_SV=1	67.568	6.07E-73
TRINITY_Contig15838	sp_P0CC48_NU2C1_COFAR_NAD(P)H-quinone_oxidoreductase_subunit_2_A,_chloroplastic_OS=Coffea_arabica_OX=13443_GN=ndhB1_PE=3_SV=1	91.438	7.29E-161
TRINITY_Contig15838	sp_P0CC48_NU2C1_COFAR_NAD(P)H-quinone_oxidoreductase_subunit_2_A,_chloroplastic_OS=Coffea_arabica_OX=13443_GN=ndhB1_PE=3_SV=1	95.565	7.15E-114
TRINITY_Contig15930	sp_Q9SD85_F3PH_ARATH_Flavonoid_3'-monooxygenase_OS=Arabidopsis_thaliana_OX=3702_GN=CYP75B1_PE=1_SV=1	41.954	1.02E-132
TRINITY_Contig16004	sp_Q3BAI2_YCX91_PHAAO_Uncharacterized_protein_ORF91_OS=Phalaenopsis_aphrodite_subsp._formosana_OX=308872_PE=4_SV=1	69.767	4.53E-08
TRINITY_Contig16022	sp_Q9FR99_ACCO_MUSAC_1-aminocyclopropane-1-carboxylate_oxidase_OS=Musa_acuminata_OX=4641_GN=MAO1B_PE=3_SV=1	71.661	5.85E-159
TRINITY_Contig16054	sp_Q9LUQ6_RL192_ARATH_60S_ribosomal_protein_L19-2_OS=Arabidopsis_thaliana_OX=3702_GN=RPL19B_PE=2_SV=1	74.684	1.75E-74
TRINITY_Contig16066	sp_Q6AWW5_Y5262_ARATH_Ankyrin_repeat-containing_protein_At5g02620_OS=Arabidopsis_thaliana_OX=3702_GN=At5g02620_PE=1_SV=1	29.493	6.51E-14

TRINITY_Contig16066	sp_Q6AWW5_Y5262_ARATH_Ankyrin_repeat-containing_protein_At5g02620_OS=Arabidopsis_thaliana_OX=3702_GN=At5g02620_PE=1_SV=1	29.412	1.13E-12
TRINITY_Contig16118	sp_Q3BAI2_YCX91_PHAAO_Uncharacterized_protein_ORF91_OS=Phalaenopsis_aphrodite_subsp._formosana_OX=308872_PE=4_SV=1	64.912	2.19E-11
TRINITY_Contig16192	sp_P51427_RS52_ARATH_40S_ribosomal_protein_S5-2_OS=Arabidopsis_thaliana_OX=3702_GN=RPS5B_PE=1_SV=2	75.253	8.98E-110
TRINITY_Contig16276	sp_Q9GT45_RS26_ANOGA_40S_ribosomal_protein_S26_OS=Anopheles_gambiae_OX=7165_GN=RpS26_PE=3_SV=2	59.459	3.03E-23
TRINITY_Contig16296	sp_Q67VD7_BURP9_ORYSJ_BURP_domain-containing_protein_9_OS=Oryza_sativa_subsp._japonica_OX=39947_GN=BURP9_PE=2_SV=1	45.674	1.44E-132
TRINITY_Contig16296	sp_Q67VD7_BURP9_ORYSJ_BURP_domain-containing_protein_9_OS=Oryza_sativa_subsp._japonica_OX=39947_GN=BURP9_PE=2_SV=1	38.608	3.23E-21
TRINITY_Contig16328	sp_Q9FG13_CXE15_ARATH_Probable_carboxylesterase_15_OS=Arabidopsis_thaliana_OX=3702_GN=CXE15_PE=2_SV=1	45.033	2.73E-50
TRINITY_Contig16356	sp_Q9M088_E135_ARATH_Glucan_endo-1,3-beta-glucosidase_5_OS=Arabidopsis_thaliana_OX=3702_GN=At4g31140_PE=2_SV=1	58.716	4.58E-180
TRINITY_Contig16403	sp_Q84YK8_LOXC2_ORYSJ_Probable_lipoxygenase_8,_chloroplastic_OS=Oryza_sativa_subsp._japonica_OX=39947_GN=CM-LOX2_PE=2_SV=1	76.953	0
TRINITY_Contig16406	sp_Q6Z4N6_40G2_ORYSJ_Ricin_B-like_lectin_R40G2_OS=Oryza_sativa_subsp._japonica_OX=39947_GN=R40G2_PE=2_SV=1	82.8	2.42E-164
TRINITY_Contig16406	sp_Q6Z4N6_40G2_ORYSJ_Ricin_B-like_lectin_R40G2_OS=Oryza_sativa_subsp._japonica_OX=39947_GN=R40G2_PE=2_SV=1	80.612	2.42E-164
TRINITY_Contig16406	sp_Q6Z4N6_40G2_ORYSJ_Ricin_B-like_lectin_R40G2_OS=Oryza_sativa_subsp._japonica_OX=39947_GN=R40G2_PE=2_SV=1	66.883	2.41E-68
TRINITY_Contig16406	sp_Q6Z4N6_40G2_ORYSJ_Ricin_B-like_lectin_R40G2_OS=Oryza_sativa_subsp._japonica_OX=39947_GN=R40G2_PE=2_SV=1	56.25	1.26E-22
TRINITY_Contig16502	sp_Q9XF97_RL4_PRUAR_60S_ribosomal_protein_L4_OS=Prunus_armeniaca_OX=36596_GN=RPL4_PE=2_SV=1	67.266	3.73E-126
TRINITY_Contig16691	sp_Q12626_EXG_PICAN_Glucan_1,3-beta-glucosidase_OS=Pichia_angusta_OX=870730_PE=3_SV=1	27.224	1.5E-22
TRINITY_Contig16691	sp_Q12626_EXG_PICAN_Glucan_1,3-beta-glucosidase_OS=Pichia_angusta_OX=870730_PE=3_SV=1	29	9.01E-13
TRINITY_Contig16764	sp_Q688Y7_TLP10_ORYSJ_Tubby-like_F-box_protein_10_OS=Oryza_sativa_subsp._japonica_OX=39947_GN=TULP10_PE=2_SV=1	82.041	5.01E-133
TRINITY_Contig16764	sp_Q688Y7_TLP10_ORYSJ_Tubby-like_F-box_protein_10_OS=Oryza_sativa_subsp._japonica_OX=39947_GN=TULP10_PE=2_SV=1	85.526	1.36E-36
TRINITY_Contig16783	sp_Q5N8X6_BGAL3_ORYSJ_Beta-galactosidase_3_OS=Oryza_sativa_subsp._japonica_OX=39947_GN=Os01g0875500_PE=3_SV=1	83.252	0
TRINITY_Contig16886	sp_F8WLS6_UGT6_CATRO_7-deoxyloganetin_glucosyltransferase_OS=Catharanthus_roseus_OX=4058_GN=UGT85A23_PE=1_SV=1	53.191	7.07E-110
TRINITY_Contig16907	sp_P0DKK7_RL7A2_ORYSJ_60S_ribosomal_protein_L7a-2_OS=Oryza_sativa_subsp._japonica_OX=39947_GN=RPL7A-2_PE=2_SV=1	60	4.73E-90
TRINITY_Contig16972	sp_Q42290_MPPB_ARATH_Probable_mitochondrial-processing_peptidase_subunit_beta,_mitochondrial_OS=Arabidopsis_thaliana_OX=3702_GN=At3g02090_PE=1_SV=2	56.818	1.92E-168
TRINITY_Contig17091	sp_Q9FZ95_PUP3_ARATH_Purine_permease_3_OS=Arabidopsis_thaliana_OX=3702_GN=PUP3_PE=2_SV=1	40.184	9.27E-51
TRINITY_Contig17167	sp_P46032_PTR2_ARATH_Protein_NRT1/_PTR_FAMILY_8.3_OS=Arabidopsis_thaliana_OX=3702_GN=NPF8.3_PE=1_SV=1	43.427	1.34E-106
TRINITY_Contig17176	sp_O96269_RS30_PLAF7_40S_ribosomal_protein_S30_OS=Plasmodium_falciparum_(isolate_3D7)_OX=36329_GN=RPS30_PE=1_SV=1	71.186	1.61E-16
TRINITY_Contig17213	sp_Q8BH95_ECHM_MOUSE_Enoyl-CoA_hydratase,_mitochondrial_OS=Mus_musculus_OX=10090_GN=Echs1_PE=1_SV=1	63.37	3.98E-122
TRINITY_Contig17304	sp_Q43362_VATL_CHRCT_V-type_proton_ATPase_16_kDa_proteolipid_subunit_OS=Chrysotila_carterae_OX=13221_GN=VAP_PE=2_SV=1	72.393	3.49E-58
TRINITY_Contig17316	sp_P48166_RL36A_CAEEL_Ribosomal_protein_rpl-36.A_OS=Caenorhabditis_elegans_OX=6239_GN=rpl-36.A_PE=3_SV=2	75.728	4.7E-45
TRINITY_Contig17327	sp_Q9ZW03_TRNH3_ARATH_Tropinone_reductase_homolog_At2g29150_OS=Arabidopsis_thaliana_OX=3702_GN=At2g29150_PE=1_SV=1	50.249	7.1E-60
TRINITY_Contig17457	sp_F2VYU4_PIK1_ORYSJ_Disease_resistance_protein_Pik-1_OS=Oryza_sativa_subsp._japonica_OX=39947_GN=PIK-1_PE=1_SV=1	59.459	5.67E-17
TRINITY_Contig17465	sp_O64495_SBT12_ARATH_Subtilisin-like_protease_SBT1.2_OS=Arabidopsis_thaliana_OX=3702_GN=SBT1.2_PE=2_SV=1	45.271	4.25E-142
TRINITY_Contig17563	sp_Q3UDW8_HGNAT_MOUSE_Heparan-alpha-glucosaminide_N-acetyltransferase_OS=Mus_musculus_OX=10090_GN=Hgsnat_PE=1_SV=2	27.576	1.18E-30
TRINITY_Contig17563	sp_Q3UDW8_HGNAT_MOUSE_Heparan-alpha-glucosaminide_N-acetyltransferase_OS=Mus_musculus_OX=10090_GN=Hgsnat_PE=1_SV=2	38.182	1.18E-30
TRINITY_Contig17571	sp_P42736_RAP23_ARATH_Ethylene-responsive_transcription_factor_RAP2-3_OS=Arabidopsis_thaliana_OX=3702_GN=RAP2-3_PE=1_SV=2	52.174	4E-26
TRINITY_Contig17597	sp_Q9LTW4_NANA_ARATH_Aspartic_proteinase_NANA,_chloroplast_OS=Arabidopsis_thaliana_OX=3702_GN=NANA_PE=1_SV=1	45.103	7.86E-90
TRINITY_Contig17611	sp_Q0DKA5_AT7_ORYSJ_Acyl_transferase_7_OS=Oryza_sativa_subsp._japonica_OX=39947_GN=AT7_PE=2_SV=1	73.438	0
TRINITY_Contig17616	sp_Q9ZUF4_RIPK_ARATH_Serine/threonine-protein_kinase_RIPK_OS=Arabidopsis_thaliana_OX=3702_GN=RIPK_PE=1_SV=1	70.899	1.95E-87

TRINITY_Contig17688	sp_Q8LC83_RS242_ARATH_40S_ribosomal_protein_S24-2_OS=Arabidopsis_thaliana_OX=3702_GN=RPS24B_PE=2_SV=2	61.29	3.86E-49
TRINITY_Contig17757	sp_Q05JG2_ABAH1_ORYSJ_Abscisic_acid_8'-hydroxylase_1_OS=Oryza_sativa_subsp._japonica_OX=39947_GN=CYP707A5_PE=2_SV=1	87.079	1.22E-176
TRINITY_Contig17757	sp_Q05JG2_ABAH1_ORYSJ_Abscisic_acid_8'-hydroxylase_1_OS=Oryza_sativa_subsp._japonica_OX=39947_GN=CYP707A5_PE=2_SV=1	76.037	1.22E-176
TRINITY_Contig17766	sp_Q0D5R3_CRK6_ORYSJ_Cysteine-rich_receptor-like_protein_kinase_6_OS=Oryza_sativa_subsp._japonica_OX=39947_GN=CRK6_PE=1_SV=1	63.06	1.92E-112
TRINITY_Contig17766	sp_Q0D5R3_CRK6_ORYSJ_Cysteine-rich_receptor-like_protein_kinase_6_OS=Oryza_sativa_subsp._japonica_OX=39947_GN=CRK6_PE=1_SV=1	32.923	1.34E-29
TRINITY_Contig17806	sp_P06671_CB22_MAIZE_Chlorophyll_a-b_binding_protein,_chloroplastic_OS=Zea_mays_OX=4577_PE=2_SV=1	94.191	1.25E-157
TRINITY_Contig17905	sp_Q7XT97_UGT79_ORYSJ_UDP-glycosyltransferase_79_OS=Oryza_sativa_subsp._japonica_OX=39947_GN=UGT79_PE=1_SV=2	54.251	1.14E-78
TRINITY_Contig17905	sp_Q7XT97_UGT79_ORYSJ_UDP-glycosyltransferase_79_OS=Oryza_sativa_subsp._japonica_OX=39947_GN=UGT79_PE=1_SV=2	45.833	5.66E-33
TRINITY_Contig17915	sp_O03982_HIP39_ARATH_Heavy_metal-associated_isoprenylated_plant_protein_39_OS=Arabidopsis_thaliana_OX=3702_GN=HIPP39_PE=2_SV=1	61.364	0.00000224
TRINITY_Contig17951	sp_Q3BAI2_YCX91_PHAAO_Uncharacterized_protein_ORF91_OS=Phalaenopsis_aphrodite_subsp._formosana_OX=308872_PE=4_SV=1	67.273	4.05E-16
TRINITY_Contig17960	sp_Q3E813_YL154_YEAST_Uncharacterized_protein_YLR154C-G_OS=Saccharomyces_cerevisiae_(strain_ATCC_204508_/_S288c)_OX=559292_GN=YLR154C-G_PE=2_SV=1	65.854	3.95E-09
TRINITY_Contig17980	sp_Q5ZAJ0_RBOHB_ORYSJ_Respiratory_burst_oxidase_homolog_protein_B_OS=Oryza_sativa_subsp._japonica_OX=39947_GN=RBOHB_PE=1_SV=1	60.147	0
TRINITY_Contig17980	sp_Q5ZAJ0_RBOHB_ORYSJ_Respiratory_burst_oxidase_homolog_protein_B_OS=Oryza_sativa_subsp._japonica_OX=39947_GN=RBOHB_PE=1_SV=1	73.188	1.81E-60
TRINITY_Contig17998	sp_O65472_CRK12_ARATH_Putative_cysteine-rich_receptor-like_protein_kinase_12_OS=Arabidopsis_thaliana_OX=3702_GN=CRK12_PE=3_SV=3	63.333	4.11E-69
TRINITY_Contig18138	sp_P38560_GLNA2_MAIZE_Glutamine_synthetase_root_isozyme_2_OS=Zea_mays_OX=4577_GN=GLN2_PE=2_SV=1	89.071	0
TRINITY_Contig18227	sp_Q9T0I5_PRP4_ARATH_Proline-rich_protein_4_OS=Arabidopsis_thaliana_OX=3702_GN=PRP4_PE=2_SV=1	40.206	2.56E-10
TRINITY_Contig18228	sp_Q9T0I5_PRP4_ARATH_Proline-rich_protein_4_OS=Arabidopsis_thaliana_OX=3702_GN=PRP4_PE=2_SV=1	40.206	8.02E-10
TRINITY_Contig18236	sp_Q6CQE5_TAR1_KLULA_Protein_TAR1_OS=Kluyveromyces_lactis_(strain_ATCC_8585_/_CBS_2359_/_DSM_70799_/_NBRC_1267_/_NRRL_Y-1140_/_WM37)_OX=284590_GN=TAR1-A_PE=4_SV=2	59.615	1.74E-09
TRINITY_Contig18398	sp_Q6YZA6_GL85_ORYSJ_Germin-like_protein_8-5_OS=Oryza_sativa_subsp._japonica_OX=39947_GN=Os08g0189400_PE=2_SV=1	83.186	1.04E-131
TRINITY_Contig18445	sp_Q9LVE0_PTR33_ARATH_Protein_NRT1/_PTR_FAMILY_6.4_OS=Arabidopsis_thaliana_OX=3702_GN=NPF6.4_PE=1_SV=1	60.171	2.54E-123
TRINITY_Contig18448	sp_Q9LVE0_PTR33_ARATH_Protein_NRT1/_PTR_FAMILY_6.4_OS=Arabidopsis_thaliana_OX=3702_GN=NPF6.4_PE=1_SV=1	78.218	8.08E-33
TRINITY_Contig18675	sp_Q84MN7_HFA2A_ORYSJ_Heat_stress_transcription_factor_A-2a_OS=Oryza_sativa_subsp._japonica_OX=39947_GN=HSFA2A_PE=2_SV=1	68.794	2.35E-41
TRINITY_Contig18731	sp_Q9SY91_PBL15_ARATH_Probable_serine/threonine-protein_kinase_PBL15_OS=Arabidopsis_thaliana_OX=3702_GN=PBL15_PE=1_SV=1	56.327	1.28E-85
TRINITY_Contig18732	sp_Q0WPW4_ACCO5_ARATH_1-aminocyclopropane-1-carboxylate_oxidase_5_OS=Arabidopsis_thaliana_OX=3702_GN=At1g77330_PE=2_SV=1	72.464	5.73E-67
TRINITY_Contig18816	sp_Q96CT7_CC124_HUMAN_Coiled-coil_domain-containing_protein_124_OS=Homo_sapiens_OX=9606_GN=CCDC124_PE=1_SV=1	49.474	3.74E-10
TRINITY_Contig18835	sp_P27216_ANX13_HUMAN_Annexin_A13_OS=Homo_sapiens_OX=9606_GN=ANXA13_PE=1_SV=3	32.68	1.09E-45
TRINITY_Contig19061	sp_C5Y376_CSPLB_SORBI_CASP-like_protein_1U2_OS=Sorghum_bicolor_OX=4558_GN=Sb05g019440_PE=2_SV=1	66.12	4.86E-47
TRINITY_Contig19131	sp_P38560_GLNA2_MAIZE_Glutamine_synthetase_root_isozyme_2_OS=Zea_mays_OX=4577_GN=GLN2_PE=2_SV=1	81.141	0
TRINITY_Contig19168	sp_Q6CQE5_TAR1_KLULA_Protein_TAR1_OS=Kluyveromyces_lactis_(strain_ATCC_8585_/_CBS_2359_/_DSM_70799_/_NBRC_1267_/_NRRL_Y-1140_/_WM37)_OX=284590_GN=TAR1-A_PE=4_SV=2	57.292	5.63E-25
TRINITY_Contig19379	sp_Q651D5_PIP27_ORYSJ_Probable_aquaporin_PIP2-7_OS=Oryza_sativa_subsp._japonica_OX=39947_GN=PIP2-7_PE=2_SV=2	81.379	6.54E-77
TRINITY_Contig19436	sp_Q9FJH0_RAA1F_ARATH_Ras-related_protein_RABA1f_OS=Arabidopsis_thaliana_OX=3702_GN=RABA1F_PE=2_SV=1	79.535	2.07E-122
TRINITY_Contig19467	sp_Q42578_PER53_ARATH_Peroxidase_53_OS=Arabidopsis_thaliana_OX=3702_GN=PER53_PE=1_SV=1	60.518	5.39E-122
TRINITY_Contig19481	sp_O59950_RS4_YARLI_40S_ribosomal_protein_S4_OS=Yarrowia_lipolytica_(strain_CLIB_122_/_E_150)_OX=284591_GN=RPS4_PE=2_SV=1	68.992	1.23E-129
TRINITY_Contig19489	sp_Q3BAI2_YCX91_PHAAO_Uncharacterized_protein_ORF91_OS=Phalaenopsis_aphrodite_subsp._formosana_OX=308872_PE=4_SV=1	64.912	1.84E-11
TRINITY_Contig19744	sp_P29766_RL8_SOLLC_60S_ribosomal_protein_L8_OS=Solanum_lycopersicum_OX=4081_GN=RPL8_PE=2_SV=1	75.403	8.24E-141
TRINITY_Contig20011	sp_O64637_C76C2_ARATH_Cytochrome_P450_76C2_OS=Arabidopsis_thaliana_OX=3702_GN=CYP76C2_PE=2_SV=1	37.013	5.28E-93
TRINITY_Contig20013	sp_Q3BAI2_YCX91_PHAAO_Uncharacterized_protein_ORF91_OS=Phalaenopsis_aphrodite_subsp._formosana_OX=308872_PE=4_SV=1	84.483	3.34E-28

TRINITY_Contig20078	sp_Q9SY91_PBL15_ARATH_Probable_serine/threonine-protein_kinase_PBL15_OS=Arabidopsis_thaliana_OX=3702_GN=PBL15_PE=1_SV=1	67.58	6.51E-94
TRINITY_Contig20078	sp_Q9SY91_PBL15_ARATH_Probable_serine/threonine-protein_kinase_PBL15_OS=Arabidopsis_thaliana_OX=3702_GN=PBL15_PE=1_SV=1	61.957	1.06E-24
TRINITY_Contig20084	sp_P22988_LDHA_HORVU_L-lactate_dehydrogenase_A_OS=Hordeum_vulgare_OX=4513_PE=1_SV=1	81.513	0
TRINITY_Contig20107	sp_Q0DM48_MAN3_ORYSJ_Mannan_endo-1,4-beta-mannosidase_3_OS=Oryza_sativa_subsp._japonica_OX=39947_GN=MAN3_PE=2_SV=2	74.691	1.35E-148
TRINITY_Contig20107	sp_Q0DM48_MAN3_ORYSJ_Mannan_endo-1,4-beta-mannosidase_3_OS=Oryza_sativa_subsp._japonica_OX=39947_GN=MAN3_PE=2_SV=2	81.818	2.69E-24
TRINITY_789_length_4703_cov_21.108114_g501_i0	sp_Q7XBQ9_RGA2_SOLBU_Disease_resistance_protein_RGA2_OS=Solanum_bulbocastanum_OX=147425_GN=RGA2_PE=1_SV=1	30.152	3.55E-79
TRINITY_789_length_4703_cov_21.108114_g501_i0	sp_Q7XBQ9_RGA2_SOLBU_Disease_resistance_protein_RGA2_OS=Solanum_bulbocastanum_OX=147425_GN=RGA2_PE=1_SV=1	29.186	2.81E-19
TRINITY_860_length_4595_cov_28.621741_g550_i0	sp_Q2QZF1_PIK6_ORYSJ_Disease_resistance_protein_PIK6-NP_OS=Oryza_sativa_subsp._japonica_OX=39947_GN=PIK6-NP_PE=2_SV=1	27.388	4.38E-40
TRINITY_1451_length_4022_cov_28.028586_g941_i0	sp_O64477_Y2913_ARATH_G-type_lectin_S-receptor-like_serine/threonine-protein_kinase_At2g19130_OS=Arabidopsis_thaliana_OX=3702_GN=At2g19130_PE=2_SV=1	43.484	0
TRINITY_1782_length_3817_cov_28.259338_g1144_i0	sp_Q33339_RDRP_CPMV1_RNA-directed_RNA_polymerase_OS=Cryphonectria_parasitica_mitovirus_1_(strain_American_chestnut_tree/New_Jersey/NB631)_OX=186769_PE=3_SV=1	38.14	9.78E-33
TRINITY_1987_length_3713_cov_30.967069_g1272_i0	sp_Q7XA40_RGA3_SOLBU_Putative_disease_resistance_protein_RGA3_OS=Solanum_bulbocastanum_OX=147425_GN=RGA3_PE=2_SV=2	27.556	9.05E-88
TRINITY_1996_length_3708_cov_30.695796_g1277_i0	sp_Q7XA40_RGA3_SOLBU_Putative_disease_resistance_protein_RGA3_OS=Solanum_bulbocastanum_OX=147425_GN=RGA3_PE=2_SV=2	32.136	4.67E-130
TRINITY_2046_length_3684_cov_29.339419_g1306_i0	sp_C0LGH2_Y1561_ARATH_Probable_LRR_receptor-like_serine/threonine-protein_kinase_At1g56130_OS=Arabidopsis_thaliana_OX=3702_GN=At1g56130_PE=2_SV=2	51.888	0
TRINITY_2046_length_3684_cov_29.339419_g1306_i0	sp_C0LGH2_Y1561_ARATH_Probable_LRR_receptor-like_serine/threonine-protein_kinase_At1g56130_OS=Arabidopsis_thaliana_OX=3702_GN=At1g56130_PE=2_SV=2	54.157	0
TRINITY_2433_length_3521_cov_24.759560_g1555_i0	sp_Q33339_RDRP_CPMV1_RNA-directed_RNA_polymerase_OS=Cryphonectria_parasitica_mitovirus_1_(strain_American_chestnut_tree/New_Jersey/NB631)_OX=186769_PE=3_SV=1	46.635	1.34E-39
TRINITY_2609_length_3462_cov_30.345712_g1658_i0	sp_Q9ZU96_Y2168_ARATH_Ankyrin_repeat-containing_protein_At2g01680_OS=Arabidopsis_thaliana_OX=3702_GN=At2g01680_PE=2_SV=1	25.45	9.69E-22
TRINITY_2609_length_3462_cov_30.345712_g1658_i0	sp_Q9ZU96_Y2168_ARATH_Ankyrin_repeat-containing_protein_At2g01680_OS=Arabidopsis_thaliana_OX=3702_GN=At2g01680_PE=2_SV=1	25.824	5.73E-09
TRINITY_2655_length_3447_cov_11.604204_g1694_i0	sp_O94489_EF3_SCHPO_Elongation_factor_3_OS=Schizosaccharomyces_pombe_(strain_972_/_ATCC_24843)_OX=284812_GN=tef3_PE=1_SV=1	50.984	0
TRINITY_4308_length_2994_cov_35.929231_g2740_i0	sp_Q3BAI2_YCX91_PHAAO_Uncharacterized_protein_ORF91_OS=Phalaenopsis_aphrodite_subsp._formosana_OX=308872_PE=4_SV=1	68.966	7.17E-17
TRINITY_4969_length_2868_cov_24.511969_g2709_i1	sp_Q9SJZ2_PER17_ARATH_Peroxidase_17_OS=Arabidopsis_thaliana_OX=3702_GN=PER17_PE=2_SV=1	73.786	5.81E-35
TRINITY_4969_length_2868_cov_24.511969_g2709_i1	sp_Q9SJZ2_PER17_ARATH_Peroxidase_17_OS=Arabidopsis_thaliana_OX=3702_GN=PER17_PE=2_SV=1	82.857	1.56E-10
TRINITY_6426_length_2624_cov_32.417613_g4064_i0	sp_O64697_C7101_ARATH_Cytochrome_P450_710A1_OS=Arabidopsis_thaliana_OX=3702_GN=CYP710A1_PE=1_SV=1	63.41	0
TRINITY_6483_length_2616_cov_33.980762_g4098_i0	sp_Q651X6_CSLE6_ORYSJ_Cellulose_synthase-like_protein_E6_OS=Oryza_sativa_subsp._japonica_OX=39947_GN=CSLE6_PE=2_SV=1	75.728	0
TRINITY_6752_length_2581_cov_35.234873_g3991_i1	sp_P46032_PTR2_ARATH_Protein_NRT1/_PTR_FAMILY_8.3_OS=Arabidopsis_thaliana_OX=3702_GN=NPF8.3_PE=1_SV=1	47.331	4.39E-166
TRINITY_7865_length_2443_cov_8.386689_g1146_i3	sp_B7F7K7_BGL31_ORYSJ_Beta-glucosidase_31_OS=Oryza_sativa_subsp._japonica_OX=39947_GN=BGLU31_PE=2_SV=1	72.614	0
TRINITY_7917_length_2435_cov_29.959425_g5008_i0	sp_P93604_LRK10_WHEAT_Rust_resistance_kinase_Lr10_OS=Triticum_aestivum_OX=4565_GN=LRK10_PE=2_SV=1	54.142	1.17E-112
TRINITY_8636_length_2358_cov_28.701616_g5459_i0	sp_Q9LXA5_LRK91_ARATH_L-type_lectin-domain_containing_receptor_kinase_IX.1_OS=Arabidopsis_thaliana_OX=3702_GN=LECRK91_PE=1_SV=1	32.448	5.63E-69
TRINITY_8636_length_2358_cov_28.701616_g5459_i0	sp_Q9LXA5_LRK91_ARATH_L-type_lectin-domain_containing_receptor_kinase_IX.1_OS=Arabidopsis_thaliana_OX=3702_GN=LECRK91_PE=1_SV=1	39.459	5.63E-69
TRINITY_8636_length_2358_cov_28.701616_g5459_i0	sp_Q9LXA5_LRK91_ARATH_L-type_lectin-domain_containing_receptor_kinase_IX.1_OS=Arabidopsis_thaliana_OX=3702_GN=LECRK91_PE=1_SV=1	32	5.63E-69
TRINITY_8742_length_2349_cov_23.646491_g5523_i0	sp_Q6WNR0_C81E7_MEDTR_Isoflavone_2'-hydroxylase_OS=Medicago_truncatula_OX=3880_GN=CYP81E7_PE=1_SV=1	36.862	2.39E-102
TRINITY_8846_length_2340_cov_24.682078_g5586_i0	sp_Q6WG30_T5H_TAXCU_Taxadiene_5-alpha_hydroxylase_OS=Taxus_cuspidata_OX=99806_PE=1_SV=2	39.514	1.02E-111
TRINITY_9542_length_2274_cov_29.067574_g5995_i0	sp_Q7XHC4_AT15_ORYSJ_Acyl_transferase_15_OS=Oryza_sativa_subsp._japonica_OX=39947_GN=AT15_PE=2_SV=1	40.625	6.2E-56
TRINITY_9820_length_2247_cov_25.817723_g6171_i0	sp_P52837_F4ST_FLACH_Flavonol_4'-sulfotransferase_OS=Flaveria_chlorifolia_OX=4228_PE=1_SV=1	40.252	3.53E-68
TRINITY_10300_length_2207_cov_78.101497_g6166_i4	sp_Q8TGM6_TAR1_YEAST_Protein_TAR1_OS=Saccharomyces_cerevisiae_(strain_ATCC_204508_/_S288c)_OX=559292_GN=TAR1_PE=2_SV=1	61.165	2.7E-16
TRINITY_10786_length_2168_cov_17.008576_g6767_i0	sp_Q54GI9_PHB1_DICDI_Prohibitin-1,_mitochondrial_OS=Dictyostelium_discoideum_OX=44689_GN=phbA_PE=3_SV=1	57.091	2.32E-87

TRINITY_11479_length_2112_cov_30.728830_g7176_i0	sp_A3AVP1_CKX8_ORYSJ_Cytokinin_dehydrogenase_8_OS=Oryza_sativa_subsp._japonica_OX=39947_GN=CKX8_PE=3_SV=1	76.923	0
TRINITY_11940_length_2078_cov_32.831757_g7461_i0	sp_O23522_PLA14_ARATH_Phospholipase_A1-Ibeta2,_chloroplastic_OS=Arabidopsis_thaliana_OX=3702_GN=At4g16820_PE=1_SV=2	48.283	1.85E-119
TRINITY_12500_length_2039_cov_25.783249_g7812_i0	sp_Q9LMX7_C78A5_ARATH_Cytochrome_P450_78A5_OS=Arabidopsis_thaliana_OX=3702_GN=CYP78A5_PE=2_SV=1	47.316	1.39E-141
TRINITY_13148_length_1996_cov_25.654904_g8229_i0	sp_Q11KJ6_DNAK_CHESB_Chaperone_protein_DnaK_OS=Chelativorans_sp._(strain_BNC1)_OX=266779_GN=dnaK_PE=3_SV=1	69.92	0
TRINITY_13203_length_1992_cov_41.331773_g8267_i0	sp_Q3E811_RRT15_YEAST_Regulator_of_rDNA_transcription_protein_15_OS=Saccharomyces_cerevisiae_(strain_ATCC_204508_/_S288c)_OX=559292_GN=RRT15_PE=3_SV=1	57.407	2.36E-12
TRINITY_13314_length_1986_cov_28.293166_g8340_i0	sp_P09189_HSP7C_PETHY_Heat_shock_cognate_70_kDa_protein_OS=Petunia_hybrida_OX=4102_GN=HSP70_PE=2_SV=1	70.062	0
TRINITY_13314_length_1986_cov_28.293166_g8340_i0	sp_P09189_HSP7C_PETHY_Heat_shock_cognate_70_kDa_protein_OS=Petunia_hybrida_OX=4102_GN=HSP70_PE=2_SV=1	69.953	0
TRINITY_13668_length_1962_cov_12.956683_g8555_i0	sp_Q9HAS3_S28A3_HUMAN_Solute_carrier_family_28_member_3_OS=Homo_sapiens_OX=9606_GN=SLC28A3_PE=1_SV=1	37.473	9.59E-77
TRINITY_14577_length_1911_cov_22.877307_g9127_i0	sp_Q94EE9_TYDC_ORYSJ_Tyrosine_decarboxylase_OS=Oryza_sativa_subsp._japonica_OX=39947_GN=TYDC_PE=1_SV=1	77.674	0
TRINITY_14818_length_1896_cov_27.142310_g9295_i0	sp_Q69ST6_PID13_ORYSJ_Protein_disulfide_isomerase-like_1-3_OS=Oryza_sativa_subsp._japonica_OX=39947_GN=PDIL1-3_PE=2_SV=1	73.626	0
TRINITY_16010_length_1831_cov_24.440976_g10042_i0	sp_O81117_C94A1_VICSA_Cytochrome_P450_94A1_OS=Vicia_sativa_OX=3908_GN=CYP94A1_PE=2_SV=2	38.289	8.95E-116
TRINITY_16708_length_1793_cov_22.377030_g10491_i0	sp_Q651D5_PIP27_ORYSJ_Probable_aquaporin_PIP2-7_OS=Oryza_sativa_subsp._japonica_OX=39947_GN=PIP2-7_PE=2_SV=2	63.2	5.68E-83
TRINITY_16708_length_1793_cov_22.377030_g10491_i0	sp_Q651D5_PIP27_ORYSJ_Probable_aquaporin_PIP2-7_OS=Oryza_sativa_subsp._japonica_OX=39947_GN=PIP2-7_PE=2_SV=2	81.818	7.82E-42
TRINITY_16773_length_1789_cov_243.990698_g10536_i0	sp_Q3BAI2_YCX91_PHAAO_Uncharacterized_protein_ORF91_OS=Phalaenopsis_aphrodite_subsp._formosana_OX=308872_PE=4_SV=1	96.703	7.08E-55
TRINITY_16880_length_1784_cov_31.650729_g10598_i0	sp_O81020_NPC2_ARATH_Non-specific_phospholipase_C2_OS=Arabidopsis_thaliana_OX=3702_GN=NPC2_PE=2_SV=1	66.802	0
TRINITY_16882_length_1784_cov_29.045481_g10600_i0	sp_Q9FNN5_NDUV1_ARATH_NADH_dehydrogenase_[ubiquinone]_flavoprotein_1,_mitochondrial_OS=Arabidopsis_thaliana_OX=3702_GN=At5g08530_PE=1_SV=1	73.034	0
TRINITY_16908_length_1783_cov_27.085764_g10616_i0	sp_P16397_SUBF_BACSU_Bacillopeptidase_F_OS=Bacillus_subtilis_(strain_168)_OX=224308_GN=bpr_PE=1_SV=2	33.619	5.84E-51
TRINITY_17167_length_1770_cov_25.322163_g10782_i0	sp_O59682_LCB1_SCHPO_Serine_palmitoyltransferase_1_OS=Schizosaccharomyces_pombe_(strain_972_/_ATCC_24843)_OX=284812_GN=lcb1_PE=3_SV=1	36.998	2.14E-100
TRINITY_17639_length_1748_cov_28.599762_g11079_i0	sp_Q9SE26_ACEA_DENCR_Isocitrate_lyase_OS=Dendrobium_crumenatum_OX=51096_GN=ICL_PE=2_SV=1	53.247	0
TRINITY_19072_length_1680_cov_24.467412_g12002_i0	sp_Q5ATG5_APDG_EMENI_Acyl-CoA_dehydrogenase_apdG_OS=Emericella_nidulans_(strain_FGSC_A4_/_ATCC_38163_/_CBS_112.46_/_NRRL_194_/_M139)_OX=227321_GN=apdG_PE=2_SV=1	38.084	1.21E-83
TRINITY_19212_length_1674_cov_27.246729_g12093_i0	sp_P28998_DHE4_CHLSO_NADP-specific_glutamate_dehydrogenase_(Fragment)_OS=Chlorella_sorokiniana_OX=3076_PE=2_SV=1	46.756	9.44E-120
TRINITY_19576_length_1657_cov_44.516373_g12312_i0	sp_P27939_AMY3C_ORYSJ_Alpha-amylase_isozyme_3C_OS=Oryza_sativa_subsp._japonica_OX=39947_GN=AMY1.7_PE=2_SV=2	87.872	0
TRINITY_20316_length_1624_cov_24.274598_g12454_i1	sp_Q65X92_C3H37_ORYSJ_Zinc_finger_CCCH_domain-containing_protein_37_OS=Oryza_sativa_subsp._japonica_OX=39947_GN=Os05g0525900_PE=2_SV=1	82.5	1.7E-75
TRINITY_21825_length_1561_cov_14.347855_g13774_i0	sp_Q6CQE5_TAR1_KLULA_Protein_TAR1_OS=Kluyveromyces_lactis_(strain_ATCC_8585_/_CBS_2359_/_DSM_70799_/_NBRC_1267_/_NRRL_Y-1140_/_WM37)_OX=284590_GN=TAR1-A_PE=4_SV=2	53.846	1.82E-14
TRINITY_21936_length_1555_cov_54.466353_g3031_i1	sp_Q3BAI2_YCX91_PHAAO_Uncharacterized_protein_ORF91_OS=Phalaenopsis_aphrodite_subsp._formosana_OX=308872_PE=4_SV=1	69.643	1E-14
TRINITY_21957_length_1555_cov_10.988560_g13869_i0	sp_O80605_SUC3_ARATH_Sucrose_transport_protein_SUC3_OS=Arabidopsis_thaliana_OX=3702_GN=SUC3_PE=1_SV=1	30.22	1.44E-17
TRINITY_22139_length_1546_cov_38.231550_g13999_i0	sp_Q3BAI2_YCX91_PHAAO_Uncharacterized_protein_ORF91_OS=Phalaenopsis_aphrodite_subsp._formosana_OX=308872_PE=4_SV=1	69.565	2.09E-13
TRINITY_22139_length_1546_cov_38.231550_g13999_i0	sp_Q3BAI2_YCX91_PHAAO_Uncharacterized_protein_ORF91_OS=Phalaenopsis_aphrodite_subsp._formosana_OX=308872_PE=4_SV=1	58.621	2.09E-13
TRINITY_22285_length_1540_cov_10.903467_g14094_i0	sp_Q4PIP8_PNS1_USTMA_Protein_PNS1_OS=Ustilago_maydis_(strain_521_/_FGSC_9021)_OX=237631_GN=PNS1_PE=3_SV=2	35.714	3.31E-27
TRINITY_23048_length_1509_cov_11.136806_g14059_i1	sp_P79008_TBB_COPC7_Tubulin_beta_chain_OS=Coprinopsis_cinerea_(strain_Okayama-7_/_130_/_ATCC_MYA-4618_/_FGSC_9003)_OX=240176_GN=CC1G_04743_PE=3_SV=1	94.419	0
TRINITY_23221_length_1502_cov_27.458479_g14712_i0	sp_Q39224_SRG1_ARATH_Protein_SRG1_OS=Arabidopsis_thaliana_OX=3702_GN=SRG1_PE=2_SV=1	35.45	6.2E-37
TRINITY_23221_length_1502_cov_27.458479_g14712_i0	sp_Q39224_SRG1_ARATH_Protein_SRG1_OS=Arabidopsis_thaliana_OX=3702_GN=SRG1_PE=2_SV=1	39.56	6.2E-37
TRINITY_23368_length_1496_cov_34.950946_g13703_i1	sp_A7QEU4_PER5_VITVI_Peroxidase_5_OS=Vitis_vinifera_OX=29760_GN=GSVIVT00037159001_PE=1_SV=2	54.098	5.12E-90
TRINITY_25469_length_1421_cov_26.433432_g16200_i0	sp_O67577_TRMH_AQUAE_tRNA_(guanosine(18)-2'-O)-methyltransferase_OS=Aquifex_aeolicus_(strain_VF5)_OX=224324_GN=trmH_PE=1_SV=1	35.333	2.76E-25
TRINITY_25907_length_1406_cov_32.055348_g9321_i1	sp_Q8RUF8_NILP3_ARATH_Omega-amidase,_chloroplastic_OS=Arabidopsis_thaliana_OX=3702_GN=NLP3_PE=1_SV=1	80.528	1.08E-153
TRINITY_27021_length_1369_cov_34.769231_g17209_i0	sp_P27659_RL3_MOUSE_60S_ribosomal_protein_L3_OS=Mus_musculus_OX=10090_GN=Rpl3_PE=1_SV=3	69.347	0
TRINITY_27499_length_1353_cov_29.495327_g17524_i0	sp_Q8LGG8_USPAL_ARATH_Universal_stress_protein_A-like_protein_OS=Arabidopsis_thaliana_OX=3702_GN=At3g01520_PE=1_SV=2	37.681	2.14E-10

TRINITY_27734_length_1346_cov_26.772122_g17673_i0	sp_P93847_RL10_SOLME_60S_ribosomal_protein_L10_OS=Solanum_melongena_OX=4111_GN=RPL10_PE=2_SV=1	81.395	5.94E-126
TRINITY_28084_length_1335_cov_11.505529_g17924_i0	sp_O30992_FTSZ_RHIRD_Cell_division_protein_FtsZ_OS=Rhizobium_radiobacter_OX=358_GN=ftsZ_PE=3_SV=1	64.762	1.39E-85
TRINITY_28199_length_1332_cov_25.331750_g18001_i0	sp_Q9FIG6_DIR1_ARATH_Dirigent_protein_1_OS=Arabidopsis_thaliana_OX=3702_GN=DIR1_PE=2_SV=1	31.429	3.55E-12
TRINITY_29826_length_1281_cov_21.981023_g19120_i0	sp_Q9FGQ9_FLZ1_ARATH_FCS-Like_Zinc_finger_1_OS=Arabidopsis_thaliana_OX=3702_GN=FLZ1_PE=1_SV=1	53.488	1.93E-08
TRINITY_30223_length_1268_cov_27.085071_g19373_i0	sp_P0CH32_UBQ4_ARATH_Polyubiquitin_4_OS=Arabidopsis_thaliana_OX=3702_GN=UBQ4_PE=3_SV=1	98.656	0
TRINITY_30223_length_1268_cov_27.085071_g19373_i0	sp_P0CH32_UBQ4_ARATH_Polyubiquitin_4_OS=Arabidopsis_thaliana_OX=3702_GN=UBQ4_PE=3_SV=1	98.684	0
TRINITY_30349_length_1264_cov_32.415900_g19459_i0	sp_Q9LHT0_TRNHF_ARATH_Tropinone_reductase_homolog_At5g06060_OS=Arabidopsis_thaliana_OX=3702_GN=At5g06060_PE=2_SV=1	42.006	1.25E-72
TRINITY_30534_length_1258_cov_27.903280_g19581_i0	sp_Q9V3D2_HEM6_DROME_Oxygen-dependent_coproporphyrinogen-III_oxidase_OS=Drosophila_melanogaster_OX=7227_GN=Coprox_PE=2_SV=1	55.072	4.38E-127
TRINITY_31407_length_1231_cov_39.333046_g20200_i0	sp_P02576_ACTA_PHYPO_Actin,_plasmodial_isoform_OS=Physarum_polycephalum_OX=5791_GN=ARDA_PE=1_SV=2	97.872	0
TRINITY_31785_length_1222_cov_16.156982_g20461_i0	sp_A8FJE5_RS18_BACP2_30S_ribosomal_protein_S18_OS=Bacillus_pumilus_(strain_SAFR-032)_OX=315750_GN=rpsR_PE=3_SV=1	62.5	1.85E-12
TRINITY_33056_length_1186_cov_38.690242_g2617_i5	sp_A3BLC3_RRFC_ORYSJ_Ribosome-recycling_factor,_chloroplastic_OS=Oryza_sativa_subsp._japonica_OX=39947_GN=Os07g0570700_PE=2_SV=2	85.171	1.06E-129
TRINITY_33143_length_1184_cov_24.504036_g21408_i0	sp_P15490_VSPA_SOYBN_Stem_28_kDa_glycoprotein_OS=Glycine_max_OX=3847_GN=VSPA_PE=2_SV=1	49.138	1.71E-67
TRINITY_33167_length_1183_cov_40.830341_g21423_i0	sp_P59075_GMPR_PHYIN_GMP_reductase_OS=Phytophthora_infestans_OX=4787_PE=2_SV=1	85.227	0
TRINITY_34076_length_1159_cov_24.367890_g5130_i3	sp_O22386_RK12_ORYSJ_50S_ribosomal_protein_L12,_chloroplastic_OS=Oryza_sativa_subsp._japonica_OX=39947_GN=RPL12-2_PE=2_SV=2	90.476	2.67E-58
TRINITY_34320_length_1152_cov_32.090489_g22257_i0	sp_Q5BK63_NDUA9_RAT_NADH_dehydrogenase_[ubiquinone]_1_alpha_subcomplex_subunit_9,_mitochondrial_OS=Rattus_norvegicus_OX=10116_GN=Ndufa9_PE=1_SV=2	40.498	8.37E-59
TRINITY_34510_length_1147_cov_16.309833_g22399_i0	sp_Q9V3D2_HEM6_DROME_Oxygen-dependent_coproporphyrinogen-III_oxidase_OS=Drosophila_melanogaster_OX=7227_GN=Coprox_PE=2_SV=1	47.74	1.18E-109
TRINITY_34846_length_1138_cov_26.140318_g22654_i0	sp_O66607_LEU3_AQUAE_3-isopropylmalate_dehydrogenase_OS=Aquifex_aeolicus_(strain_VF5)_OX=224324_GN=leuB_PE=3_SV=1	49.171	3.08E-116
TRINITY_35939_length_1108_cov_41.467757_g23432_i0	sp_Q10N21_APX1_ORYSJ_L-ascorbate_peroxidase_1,_cytosolic_OS=Oryza_sativa_subsp._japonica_OX=39947_GN=APX1_PE=1_SV=1	90	1.22E-166
TRINITY_36311_length_1098_cov_33.822157_g20413_i1	sp_P0C8Z0_Y8359_ORYSI_Uncharacterized_protein_OsI_027940_OS=Oryza_sativa_subsp._indica_OX=39946_GN=OsI_027940_PE=1_SV=2	89.844	2.24E-60
TRINITY_36799_length_1086_cov_26.382498_g24034_i0	sp_P31110_TLP_ORYSJ_Thaumatin-like_protein_OS=Oryza_sativa_subsp._japonica_OX=39947_GN=Os12g0628600_PE=1_SV=1	71.141	2.18E-70
TRINITY_37307_length_1074_cov_24.689552_g24415_i0	sp_Q9FMR3_NAC90_ARATH_NAC_domain-containing_protein_90_OS=Arabidopsis_thaliana_OX=3702_GN=NAC090_PE=1_SV=1	56.667	2.32E-63
TRINITY_37441_length_1071_cov_20.983034_g24503_i0	sp_P02723_ADT_NEUCR_ADP,ATP_carrier_protein_OS=Neurospora_crassa_(strain_ATCC_24698_/_74-OR23-1A_/_CBS_708.71_/_DSM_1257_/_FGSC_987)_OX=367110_GN=aac_PE=3_SV=1	78.095	8.96E-169
TRINITY_37641_length_1066_cov_8.959880_g24640_i0	sp_O31826_YNGI_BACSU_Putative_acyl-CoA_synthetase_YngI_OS=Bacillus_subtilis_(strain_168)_OX=224308_GN=yngI_PE=3_SV=1	24.468	5.44E-19
TRINITY_37696_length_1064_cov_33.411055_g23900_i1	sp_P42736_RAP23_ARATH_Ethylene-responsive_transcription_factor_RAP2-3_OS=Arabidopsis_thaliana_OX=3702_GN=RAP2-3_PE=1_SV=2	52.206	5.47E-28
TRINITY_38231_length_1051_cov_39.189409_g13247_i1	sp_Q8H124_Y2446_ARATH_Uncharacterized_protein_At2g34460,_chloroplastic_OS=Arabidopsis_thaliana_OX=3702_GN=At2g34460_PE=1_SV=1	71.489	8.65E-119
TRINITY_38273_length_1050_cov_34.260958_g25070_i0	sp_Q29380_VDAC3_PIG_Voltage-dependent_anion-selective_channel_protein_3_OS=Sus_scrofa_OX=9823_GN=VDAC3_PE=2_SV=2	27.338	2.32E-22
TRINITY_39367_length_1025_cov_29.965481_g25910_i0	sp_Q43194_NLTP2_SORBI_Non-specific_lipid-transfer_protein_2_OS=Sorghum_bicolor_OX=4558_GN=LTP2_PE=3_SV=1	83.333	7.63E-34
TRINITY_39457_length_1023_cov_26.554507_g25971_i0	sp_Q12G86_RS5_POLSJ_30S_ribosomal_protein_S5_OS=Polaromonas_sp._(strain_JS666_/_ATCC_BAA-500)_OX=296591_GN=rpsE_PE=3_SV=1	38.462	2.51E-31
TRINITY_40668_length_996_cov_18.574973_g2381_i2	sp_Q3BAI2_YCX91_PHAAO_Uncharacterized_protein_ORF91_OS=Phalaenopsis_aphrodite_subsp._formosana_OX=308872_PE=4_SV=1	76.923	1.01E-10
TRINITY_41032_length_987_cov_19.518519_g27176_i0	sp_Q03161_YMY9_YEAST_Glucose-6-phosphate_1-epimerase_OS=Saccharomyces_cerevisiae_(strain_ATCC_204508_/_S288c)_OX=559292_GN=YMR099C_PE=1_SV=1	35.855	1.23E-48
TRINITY_41670_length_971_cov_30.574279_g27665_i0	sp_P29787_TRY5_AEDAE_Trypsin_5G1_OS=Aedes_aegypti_OX=7159_GN=AAEL013712_PE=2_SV=2	40.948	5.1E-35
TRINITY_42139_length_962_cov_15.804031_g28035_i0	sp_P50085_PHB2_YEAST_Prohibitin-2_OS=Saccharomyces_cerevisiae_(strain_ATCC_204508_/_S288c)_OX=559292_GN=PHB2_PE=1_SV=2	58.367	1.05E-102
TRINITY_43050_length_944_cov_30.869714_g28743_i0	sp_Q2EN81_ATPO_PIG_ATP_synthase_subunit_O,_mitochondrial_OS=Sus_scrofa_OX=9823_GN=ATP5PO_PE=1_SV=1	44.681	8.04E-49
TRINITY_43818_length_929_cov_20.308140_g29324_i0	sp_D4AM78_CDA_ARTBC_Chitin_deacetylase_ARB_04768_OS=Arthroderma_benhamiae_(strain_ATCC_MYA-4681_/_CBS_112371)_OX=663331_GN=ARB_04768_PE=3_SV=1	32.906	1.73E-26
TRINITY_44102_length_923_cov_32.492974_g29548_i0	sp_Q5RJH2_MCTP2_MOUSE_Multiple_C2_and_transmembrane_domain-containing_protein_2_OS=Mus_musculus_OX=10090_GN=Mctp2_PE=2_SV=1	35.115	5.35E-10

TRINITY_44173_length_922_cov_13.471278_g29605_i0	sp_Q9LTV6_DECR2_ARATH_Peroxisomal_2,4-dienoyl-CoA_reductase_OS=Arabidopsis_thaliana_OX=3702_GN=At3g12800_PE=2_SV=1	50.69	1.19E-78
TRINITY_44220_length_921_cov_10.535211_g29643_i0	sp_Q01890_YPT1_PHYIN_Ras-like_GTP-binding_protein_YPT1_OS=Phytophthora_infestans_OX=4787_GN=YPT1_PE=3_SV=1	92.118	4.58E-133
TRINITY_44507_length_915_cov_25.151300_g29871_i0	sp_Q8KWT4_BACC_BACIU_Dihydroanticapsin_7-dehydrogenase_OS=Bacillus_subtilis_OX=1423_GN=bacC_PE=1_SV=1	40.816	9.75E-36
TRINITY_44602_length_913_cov_24.276066_g29946_i0	sp_P29786_TRY3_AEDAE_Trypsin_3A1_OS=Aedes_aegypti_OX=7159_GN=AAEL007818_PE=2_SV=2	39.167	1.21E-43
TRINITY_44624_length_912_cov_35.240807_g29046_i1	sp_O82081_UCC1_ARATH_Uclacyanin_1_OS=Arabidopsis_thaliana_OX=3702_GN=UCC1_PE=1_SV=1	46.316	4.83E-22
TRINITY_44798_length_909_cov_12.097619_g30102_i0	sp_Q9R1Z0_VDAC3_RAT_Voltage-dependent_anion-selective_channel_protein_3_OS=Rattus_norvegicus_OX=10116_GN=Vdac3_PE=1_SV=2	27.046	1.12E-20
TRINITY_45254_length_900_cov_18.270758_g30482_i0	sp_Q20719_NDUV2_CAEEL_Probable_NADH_dehydrogenase_[ubiquinone]_flavoprotein_2,_mitochondrial_OS=Caenorhabditis_elegans_OX=6239_GN=F53F4.10_PE=3_SV=1	55.869	1.48E-79
TRINITY_45521_length_895_cov_23.578692_g30705_i0	sp_P29786_TRY3_AEDAE_Trypsin_3A1_OS=Aedes_aegypti_OX=7159_GN=AAEL007818_PE=2_SV=2	41.736	9.04E-44
TRINITY_45575_length_894_cov_14.859394_g30748_i0	sp_Q6CQE5_TAR1_KLULA_Protein_TAR1_OS=Kluyveromyces_lactis_(strain_ATCC_8585_/_CBS_2359_/_DSM_70799_/_NBRC_1267_/_NRRL_Y-1140_/_WM37)_OX=284590_GN=TAR1-A_PE=4_SV=2	55.357	2.66E-26
TRINITY_46079_length_884_cov_27.233129_g31144_i0	sp_Q84Q72_HS181_ORYSJ_18.1_kDa_class_I_heat_shock_protein_OS=Oryza_sativa_subsp._japonica_OX=39947_GN=HSP18.1_PE=2_SV=1	85.714	1.11E-63
TRINITY_46620_length_874_cov_9.279503_g31572_i0	sp_Q9ZUT9_RS51_ARATH_40S_ribosomal_protein_S5-1_OS=Arabidopsis_thaliana_OX=3702_GN=RPS5A_PE=1_SV=1	74.872	5.68E-107
TRINITY_46902_length_869_cov_27.276250_g31811_i0	sp_F7J0N2_RGA5R_ORYSJ_Disease_resistance_protein_RGA5_OS=Oryza_sativa_subsp._japonica_OX=39947_GN=RGA5_PE=1_SV=1	54.878	0.000000784
TRINITY_47022_length_867_cov_21.624060_g31913_i0	sp_Q02921_NO93_SOYBN_Early_nodulin-93_OS=Glycine_max_OX=3847_PE=2_SV=1	69.318	6.6E-25
TRINITY_47110_length_865_cov_11.727387_g31982_i0	sp_Q9SIP7_RS31_ARATH_40S_ribosomal_protein_S3-1_OS=Arabidopsis_thaliana_OX=3702_GN=RPS3A_PE=1_SV=1	79.279	1.09E-125
TRINITY_47920_length_849_cov_32.901282_g32662_i0	sp_Q8VZB9_R10A1_ARATH_60S_ribosomal_protein_L10a-1_OS=Arabidopsis_thaliana_OX=3702_GN=RPL10AA_PE=1_SV=1	62.719	3.25E-87
TRINITY_48328_length_842_cov_8.978008_g32983_i0	sp_P11069_GLB3_LUMTE_Extracellular_globin-3_OS=Lumbricus_terrestris_OX=6398_PE=1_SV=3	49.664	7.95E-33
TRINITY_48405_length_840_cov_25.081712_g33047_i0	sp_P27777_HS16A_ORYSJ_16.9_kDa_class_I_heat_shock_protein_1_OS=Oryza_sativa_subsp._japonica_OX=39947_GN=HSP16.9A_PE=1_SV=1	88.158	2.12E-69
TRINITY_48690_length_835_cov_13.203655_g33293_i0	sp_Q57625_ILVH_METJA_Probable_acetolactate_synthase_small_subunit_OS=Methanocaldococcus_jannaschii_(strain_ATCC_43067_/_DSM_2661_/_JAL-1_/_JCM_10045_/_NBRC_100440)_OX=243232_GN=ilvH_PE=3_SV=1	39.394	9.9E-38
TRINITY_49435_length_821_cov_24.875000_g33912_i0	sp_Q84Q72_HS181_ORYSJ_18.1_kDa_class_I_heat_shock_protein_OS=Oryza_sativa_subsp._japonica_OX=39947_GN=HSP18.1_PE=2_SV=1	78.986	1.42E-52
TRINITY_49677_length_817_cov_7.887701_g34126_i0	sp_P13181_GAL2_YEAST_Galactose_transporter_OS=Saccharomyces_cerevisiae_(strain_ATCC_204508_/_S288c)_OX=559292_GN=GAL2_PE=1_SV=3	33.333	5.14E-32
TRINITY_50285_length_806_cov_7.518318_g34638_i0	sp_Q6PR31_ECTC_VIRPA_L-ectoine_synthase_OS=Virgibacillus_pantothenticus_OX=1473_GN=ectC_PE=3_SV=1	28.889	5.19E-13
TRINITY_51040_length_792_cov_26.226833_g35292_i0	sp_Q9YGQ1_EF1B_CHICK_Elongation_factor_1-beta_OS=Gallus_gallus_OX=9031_GN=EEF1B_PE=2_SV=3	55.752	7.22E-68
TRINITY_51087_length_791_cov_30.051247_g35333_i0	sp_A1CU59_XYNB_ASPCL_Probable_endo-1,4-beta-xylanase_B_OS=Aspergillus_clavatus_(strain_ATCC_1007_/_CBS_513.65_/_DSM_816_/_NCTC_3887_/_NRRL_1)_OX=344612_GN=xlnB_PE=3_SV=2	59.276	3.55E-78
TRINITY_51271_length_788_cov_33.305981_g33374_i1	sp_P34091_RL6_MESCR_60S_ribosomal_protein_L6_OS=Mesembryanthemum_crystallinum_OX=3544_GN=RPL6_PE=2_SV=1	54.667	7.44E-64
TRINITY_51638_length_782_cov_14.040673_g35804_i0	sp_P61518_CAN_SHIFL_Carbonic_anhydrase_2_OS=Shigella_flexneri_OX=623_GN=can_PE=3_SV=1	39.801	1.95E-35
TRINITY_51831_length_778_cov_27.468265_g35969_i0	sp_Q10ST8_NLTPX_ORYSJ_Non-specific_lipid-transfer_protein_2_OS=Oryza_sativa_subsp._japonica_OX=39947_GN=LTP-2_PE=1_SV=1	52.727	5.15E-12
TRINITY_52383_length_769_cov_26.514286_g36450_i0	sp_Q9FIG6_DIR1_ARATH_Dirigent_protein_1_OS=Arabidopsis_thaliana_OX=3702_GN=DIR1_PE=2_SV=1	36.301	2.59E-16
TRINITY_53321_length_753_cov_7.992690_g37257_i0	sp_Q7ZUZ0_CSN8_DANRE_COP9_signalosome_complex_subunit_8_OS=Danio_rerio_OX=7955_GN=cops8_PE=2_SV=1	33.742	9.1E-26
TRINITY_53589_length_748_cov_31.410898_g37489_i0	sp_Q02921_NO93_SOYBN_Early_nodulin-93_OS=Glycine_max_OX=3847_PE=2_SV=1	79.032	1.09E-15
TRINITY_53589_length_748_cov_31.410898_g37489_i0	sp_Q02921_NO93_SOYBN_Early_nodulin-93_OS=Glycine_max_OX=3847_PE=2_SV=1	57.143	1.09E-15
TRINITY_53973_length_742_cov_16.849926_g37829_i0	sp_O14201_MUG86_SCHPO_Meiotically_up-regulated_gene_86_protein_OS=Schizosaccharomyces_pombe_(strain_972_/_ATCC_24843)_OX=284812_GN=mug86_PE=1_SV=1	36.364	9.57E-29
TRINITY_54202_length_738_cov_12.753363_g38037_i0	sp_Q9LY75_CYP63_ARATH_Peptidyl-prolyl_cis-trans_isomerase_CYP63_OS=Arabidopsis_thaliana_OX=3702_GN=CYP63_PE=1_SV=1	47.093	1.59E-37
TRINITY_54629_length_731_cov_29.590634_g38422_i0	sp_Q02921_NO93_SOYBN_Early_nodulin-93_OS=Glycine_max_OX=3847_PE=2_SV=1	68.182	5.95E-24
TRINITY_54844_length_728_cov_11.807284_g38608_i0	sp_Q12335_PST2_YEAST_Protoplast_secreted_protein_2_OS=Saccharomyces_cerevisiae_(strain_ATCC_204508_/_S288c)_OX=559292_GN=PST2_PE=1_SV=1	60.606	1.05E-74
TRINITY_55144_length_723_cov_15.974006_g38880_i0	sp_Q29RM3_REEP5_BOVIN_Receptor_expression-enhancing_protein_5_OS=Bos_taurus_OX=9913_GN=REEP5_PE=2_SV=1	48.905	1.8E-34

TRINITY_55261_length_721_cov_34.769939_g38988_i0	sp_Q0CGL5_NACB_ASPTN_Nascent_polypeptide-associated_complex_subunit_beta_OS=Aspergillus_terreus_(strain_NIH_2624_/_FGSC_A1156)_OX=341663_GN=egd1_PE=3_SV=1	63.014	1.19E-27
TRINITY_55657_length_714_cov_25.865116_g39340_i0	sp_Q9C3Y4_RHOA_EMENI_GTP-binding_protein_rhoA_OS=Emericella_nidulans_(strain_FGSC_A4_/_ATCC_38163_/_CBS_112.46_/_NRRL_194_/_M139)_OX=227321_GN=rhoA_PE=3_SV=1	89.143	9.18E-114
TRINITY_55667_length_714_cov_23.524031_g39350_i0	sp_P36397_ARF1_ARATH_ADP-ribosylation_factor_1_OS=Arabidopsis_thaliana_OX=3702_GN=ARF1_PE=1_SV=2	83.333	9.05E-114
TRINITY_55742_length_713_cov_11.625776_g39416_i0	sp_P05738_RL9A_YEAST_60S_ribosomal_protein_L9-A_OS=Saccharomyces_cerevisiae_(strain_ATCC_204508_/_S288c)_OX=559292_GN=RPL9A_PE=1_SV=2	59.067	1.12E-77
TRINITY_56012_length_708_cov_25.733959_g39660_i0	sp_Q8LDP4_CP19D_ARATH_Peptidyl-prolyl_cis-trans_isomerase_CYP19-4_OS=Arabidopsis_thaliana_OX=3702_GN=CYP19-4_PE=1_SV=2	79.07	6.02E-86
TRINITY_56315_length_703_cov_22.380126_g39937_i0	sp_Q39613_CYPH_CATRO_Peptidyl-prolyl_cis-trans_isomerase_OS=Catharanthus_roseus_OX=4058_GN=PCKR1_PE=1_SV=1	80.702	1.16E-89
TRINITY_57032_length_691_cov_47.041801_g40609_i0	sp_P02362_RS7_XENLA_40S_ribosomal_protein_S7_OS=Xenopus_laevis_OX=8355_GN=rps7_PE=2_SV=2	61.78	5.92E-75
TRINITY_57259_length_688_cov_24.528271_g40822_i0	sp_P14009_14KD_DAUCA_14_kDa_proline-rich_protein_DC2.15_OS=Daucus_carota_OX=4039_PE=2_SV=1	60.305	1.44E-38
TRINITY_57649_length_682_cov_23.876020_g25291_i1	sp_Q02921_NO93_SOYBN_Early_nodulin-93_OS=Glycine_max_OX=3847_PE=2_SV=1	68.478	2.64E-18
TRINITY_57894_length_678_cov_19.193760_g41409_i0	sp_P46295_RS14_CHLRE_40S_ribosomal_protein_S14_OS=Chlamydomonas_reinhardtii_OX=3055_GN=RPS14_PE=3_SV=1	91.129	1.2E-78
TRINITY_58163_length_674_cov_40.960331_g41660_i0	sp_O65059_RS15_PICMA_40S_ribosomal_protein_S15_OS=Picea_mariana_OX=3335_GN=RPS15_PE=2_SV=1	74.016	2.31E-55
TRINITY_58533_length_669_cov_16.348333_g42002_i0	sp_O96876_TCP17_TRYCR_Protein_TCP17_OS=Trypanosoma_cruzi_OX=5693_GN=TCP17_PE=1_SV=1	44.604	1.19E-28
TRINITY_58645_length_667_cov_26.615385_g42113_i0	sp_Q9LPK5_CML44_ARATH_Probable_calcium-binding_protein_CML44_OS=Arabidopsis_thaliana_OX=3702_GN=CML44_PE=2_SV=2	46.429	5.68E-08
TRINITY_59791_length_651_cov_30.013746_g43197_i0	sp_P35066_H2A_TRYCR_Histone_H2A_OS=Trypanosoma_cruzi_OX=5693_PE=3_SV=2	80	6.45E-27
TRINITY_60032_length_648_cov_15.336788_g43424_i0	sp_Q9LUQ6_RL192_ARATH_60S_ribosomal_protein_L19-2_OS=Arabidopsis_thaliana_OX=3702_GN=RPL19B_PE=2_SV=1	74.684	2.46E-75
TRINITY_60365_length_643_cov_32.642857_g43740_i0	sp_P80306_FER6_RHOCA_Ferredoxin-6_OS=Rhodobacter_capsulatus_OX=1061_GN=fdxE_PE=1_SV=2	25.21	0.00000176
TRINITY_61523_length_627_cov_38.087814_g44828_i0	sp_P90543_H3_EUPCR_Histone_H3_OS=Euplotes_crassus_OX=5936_PE=3_SV=3	77.011	4.58E-42
TRINITY_62611_length_612_cov_30.747698_g45876_i0	sp_P0DJ25_RL40_TETTS_Ubiquitin-60S_ribosomal_protein_L40_OS=Tetrahymena_thermophila_(strain_SB210)_OX=312017_GN=RPL40_PE=1_SV=1	86.992	9.31E-76
TRINITY_63030_length_607_cov_31.377323_g46280_i0	sp_Q9CQJ8_NDUB9_MOUSE_NADH_dehydrogenase_[ubiquinone]_1_beta_subcomplex_subunit_9_OS=Mus_musculus_OX=10090_GN=Ndufb9_PE=1_SV=3	42.391	2.69E-13
TRINITY_63104_length_606_cov_28.420857_g46350_i0	sp_O14602_IF1AY_HUMAN_Eukaryotic_translation_initiation_factor_1A,_Y-chromosomal_OS=Homo_sapiens_OX=9606_GN=EIF1AY_PE=1_SV=4	78.723	2.73E-47
TRINITY_63182_length_605_cov_25.376866_g46425_i0	sp_Q02369_NDUB9_BOVIN_NADH_dehydrogenase_[ubiquinone]_1_beta_subcomplex_subunit_9_OS=Bos_taurus_OX=9913_GN=NDUFB9_PE=1_SV=2	38.889	6.5E-14
TRINITY_63265_length_604_cov_23.091589_g46506_i0	sp_Q9HGX4_H2A_AGABI_Histone_H2A_OS=Agaricus_bisporus_OX=5341_PE=2_SV=3	81.6	1.52E-51
TRINITY_64539_length_588_cov_26.105973_g47730_i0	sp_P49211_RL321_ARATH_60S_ribosomal_protein_L32-1_OS=Arabidopsis_thaliana_OX=3702_GN=RPL32A_PE=2_SV=2	72.321	4.64E-54
TRINITY_65025_length_582_cov_25.727096_g48200_i0	sp_Q9SU35_ERLL1_ARATH_pEARLI1-like_lipid_transfer_protein_1_OS=Arabidopsis_thaliana_OX=3702_GN=AZI1_PE=1_SV=1	66.667	1.02E-12
TRINITY_65730_length_573_cov_26.823413_g48887_i0	sp_Q6GPM4_RM24_XENLA_Probable_39S_ribosomal_protein_L24,_mitochondrial_OS=Xenopus_laevis_OX=8355_GN=mrpl24_PE=2_SV=1	47.059	3.24E-27
TRINITY_65816_length_572_cov_27.196819_g48968_i0	sp_P62250_RS16_RAT_40S_ribosomal_protein_S16_OS=Rattus_norvegicus_OX=10116_GN=Rps16_PE=1_SV=2	79.433	1.88E-81
TRINITY_65979_length_570_cov_30.265469_g49125_i0	sp_P0DJ19_RL27_TETTH_60S_ribosomal_protein_L27_OS=Tetrahymena_thermophila_OX=5911_GN=RPL27_PE=1_SV=1	53.846	9.87E-29
TRINITY_66567_length_563_cov_32.196356_g49701_i0	sp_P0CT74_RS11B_SCHPO_40S_ribosomal_protein_S11-B_OS=Schizosaccharomyces_pombe_(strain_972_/_ATCC_24843)_OX=284812_GN=rps1102_PE=3_SV=1	65.823	2.58E-68
TRINITY_66665_length_562_cov_28.018256_g49797_i0	sp_Q962R9_RS10_SPOFR_40S_ribosomal_protein_S10_OS=Spodoptera_frugiperda_OX=7108_GN=RpS10_PE=2_SV=1	64.286	2.22E-44
TRINITY_67483_length_553_cov_29.580579_g50597_i0	sp_Q8J0N3_SODC_ISATE_Superoxide_dismutase_[Cu-Zn]_OS=Isaria_tenuipes_OX=45847_GN=SOD1_PE=3_SV=3	51.923	2.94E-42
TRINITY_67558_length_552_cov_34.223602_g50672_i0	sp_Q9SJ36_RS172_ARATH_40S_ribosomal_protein_S17-2_OS=Arabidopsis_thaliana_OX=3702_GN=RPS17B_PE=2_SV=3	74.51	1.73E-43
TRINITY_67661_length_551_cov_31.452282_g50772_i0	sp_O14317_RLA6_SCHPO_60S_acidic_ribosomal_protein_P2-C_OS=Schizosaccharomyces_pombe_(strain_972_/_ATCC_24843)_OX=284812_GN=rpp203_PE=1_SV=2	60	1.13E-19
TRINITY_67782_length_550_cov_19.070686_g50891_i0	sp_Q9W385_FRDA_DROME_Frataxin_homolog,_mitochondrial_OS=Drosophila_melanogaster_OX=7227_GN=fh_PE=2_SV=1	41.121	4.75E-21
TRINITY_67928_length_548_cov_31.467641_g51035_i0	sp_Q962R9_RS10_SPOFR_40S_ribosomal_protein_S10_OS=Spodoptera_frugiperda_OX=7108_GN=RpS10_PE=2_SV=1	64.286	4.9E-44

TRINITY_68424_length_543_cov_27.683544_g51528_i0	sp_Q54P95_COX6B_DICDI_Probable_cytochrome_c_oxidase_subunit_6B_OS=Dictyostelium_discoideum_OX=44689_GN=DDB_G0284693_PE=3_SV=1	44.231	2.45E-11
TRINITY_70529_length_521_cov_35.712389_g53582_i0	sp_P15569_ELIB_PHYCI_Beta-elicitin_cinnamomin_OS=Phytophthora_cinnamomi_OX=4785_PE=1_SV=1	41.975	2.67E-12
TRINITY_70538_length_521_cov_28.570796_g53591_i0	sp_P00427_COX6_YEAST_Cytochrome_c_oxidase_subunit_6,_mitochondrial_OS=Saccharomyces_cerevisiae_(strain_ATCC_204508_/_S288c)_OX=559292_GN=COX6_PE=1_SV=1	53.901	1.23E-47
TRINITY_71255_length_514_cov_46.017978_g54301_i0	sp_P99027_RLA2_MOUSE_60S_acidic_ribosomal_protein_P2_OS=Mus_musculus_OX=10090_GN=Rplp2_PE=1_SV=3	65.455	6.01E-18
TRINITY_71284_length_514_cov_20.253933_g54330_i0	sp_Q28IL6_RL22_XENTR_60S_ribosomal_protein_L22_OS=Xenopus_tropicalis_OX=8364_GN=rpl22_PE=2_SV=3	61.017	5.61E-27
TRINITY_71889_length_508_cov_19.735763_g54926_i0	sp_O93931_RS26_SCHCO_40S_ribosomal_protein_S26_OS=Schizophyllum_commune_OX=5334_GN=RPS26_PE=2_SV=1	94.624	2.24E-47
TRINITY_72660_length_500_cov_37.150812_g55688_i0	sp_Q69CJ9_RL35_OPHHA_60S_ribosomal_protein_L35_OS=Ophiophagus_hannah_OX=8665_GN=RPL35_PE=2_SV=3	68.595	2.94E-49
TRINITY_72987_length_497_cov_18.705607_g56008_i0	sp_Q68HB4_PROF_BOMMO_Profilin_OS=Bombyx_mori_OX=7091_PE=2_SV=1	27.82	0.0000044
TRINITY_73080_length_496_cov_25.309133_g56099_i0	sp_P62792_H4_PHACH_Histone_H4_OS=Phanerochaete_chrysosporium_OX=5306_GN=H4.1_PE=3_SV=2	100	4.73E-51
TRINITY_74342_length_484_cov_40.106024_g57346_i0	sp_P62832_RL23_RAT_60S_ribosomal_protein_L23_OS=Rattus_norvegicus_OX=10116_GN=Rpl23_PE=2_SV=1	87.681	1.03E-83
TRINITY_75684_length_472_cov_29.022333_g58684_i0	sp_Q9VXX8_RL371_DROME_Probable_60S_ribosomal_protein_L37-A_OS=Drosophila_melanogaster_OX=7227_GN=RpL37a_PE=1_SV=1	76.087	2.73E-45
TRINITY_75892_length_470_cov_29.094763_g58887_i0	sp_P46291_RL38_SOLLC_60S_ribosomal_protein_L38_OS=Solanum_lycopersicum_OX=4081_GN=RPL38_PE=3_SV=1	73.913	6.55E-22
TRINITY_85417_length_394_cov_26.606154_g68353_i0	sp_Q54UG4_RL37A_DICDI_60S_ribosomal_protein_L37a_OS=Dictyostelium_discoideum_OX=44689_GN=rpl37A_PE=3_SV=1	76.923	5.63E-35
TRINITY_86997_length_382_cov_30.827476_g69930_i0	sp_P33286_RS28_KLUMA_40S_ribosomal_protein_S28_OS=Kluyveromyces_marxianus_OX=4911_GN=RPS28_PE=3_SV=1	77.778	1.93E-17
TRINITY_87282_length_380_cov_33.813505_g70214_i0	sp_P33286_RS28_KLUMA_40S_ribosomal_protein_S28_OS=Kluyveromyces_marxianus_OX=4911_GN=RPS28_PE=3_SV=1	69.811	7.86E-19
TRINITY_88083_length_374_cov_25.714754_g71013_i0	sp_P48504_QCR6_SOLTU_Cytochrome_b-c1_complex_subunit_6_OS=Solanum_tuberosum_OX=4113_PE=1_SV=2	44.928	4.31E-14
TRINITY_91031_length_352_cov_27.409894_g73960_i0	sp_Q96253_ATP5E_ARATH_ATP_synthase_subunit_epsilon,_mitochondrial_OS=Arabidopsis_thaliana_OX=3702_GN=At1g51650_PE=1_SV=3	45.614	8.15E-12
TRINITY_BIN_1107_10	sp_Q10CE4_GLO1_ORYSJ_Peroxisomal_(S)-2-hydroxy-acid_oxidase_GLO1_OS=Oryza_sativa_subsp._japonica_OX=39947_GN=GLO1_PE=1_SV=1	90.786	0
TRINITY_BIN_11288_13	sp_Q5Z9J0_MPK12_ORYSJ_Mitogen-activated_protein_kinase_12_OS=Oryza_sativa_subsp._japonica_OX=39947_GN=MPK12_PE=1_SV=2	89.273	0
TRINITY_BIN_11479_1	sp_Q41140_PFPA_RICCO_Pyrophosphate--fructose_6-phosphate_1-phosphotransferase_subunit_alpha_OS=Ricinus_communis_OX=3988_GN=PFP-ALPHA_PE=3_SV=1	67.638	0
TRINITY_BIN_11487_1	sp_Q84VQ4_NDHU_ARATH_NAD(P)H-quinone_oxidoreductase_subunit_U,_chloroplastic_OS=Arabidopsis_thaliana_OX=3702_GN=ndhU_PE=1_SV=1	54.167	4.82E-33
TRINITY_BIN_11503_2	sp_Q9ZNT1_NB5R1_ARATH_NADH--cytochrome_b5_reductase_1_OS=Arabidopsis_thaliana_OX=3702_GN=CBR1_PE=1_SV=1	76.786	1.39E-151
TRINITY_BIN_1201_2	sp_Q54MK8_RL18A_DICDI_60S_ribosomal_protein_L18a_OS=Dictyostelium_discoideum_OX=44689_GN=rpl18a_PE=3_SV=2	58.046	1.78E-70
TRINITY_BIN_12695_3	sp_Q9LYU3_EF113_ARATH_Ethylene-responsive_transcription_factor_ERF113_OS=Arabidopsis_thaliana_OX=3702_GN=ERF113_PE=2_SV=1	56.452	0.00000322
TRINITY_BIN_13116_7	sp_P16397_SUBF_BACSU_Bacillopeptidase_F_OS=Bacillus_subtilis_(strain_168)_OX=224308_GN=bpr_PE=1_SV=2	34.318	7.89E-49
TRINITY_BIN_14439_1	sp_Q9SUU5_QCR71_ARATH_Cytochrome_b-c1_complex_subunit_7-1_OS=Arabidopsis_thaliana_OX=3702_GN=QCR7-1_PE=1_SV=1	39.175	9.86E-15
TRINITY_BIN_14504_2	sp_P02879_RICI_RICCO_Ricin_OS=Ricinus_communis_OX=3988_PE=1_SV=1	32.679	1.13E-67
TRINITY_BIN_15103_2	sp_Q2QSL4_CDKF2_ORYSJ_Putative_cyclin-dependent_kinase_F-2_OS=Oryza_sativa_subsp._japonica_OX=39947_GN=CDKF-2_PE=3_SV=1	40.924	2.49E-53
TRINITY_BIN_15300_1	sp_Q9C7S7_ERO1_ARATH_Endoplasmic_reticulum_oxidoreductin-1_OS=Arabidopsis_thaliana_OX=3702_GN=AERO1_PE=1_SV=1	75.188	0
TRINITY_BIN_15641_2	sp_Q9FMS6_CAF1K_ARATH_Probable_CCR4-associated_factor_1_homolog_11_OS=Arabidopsis_thaliana_OX=3702_GN=CAF1-11_PE=2_SV=1	51.128	7.25E-80
TRINITY_BIN_17586_1	sp_Q9N2W7_NDUAC_CAEEL_Probable_NADH_dehydrogenase_[ubiquinone]_1_alpha_subcomplex_subunit_12_OS=Caenorhabditis_elegans_OX=6239_GN=Y94H6A.8_PE=3_SV=2	36.047	5.57E-11
TRINITY_BIN_18031_1	sp_P51425_RL39_MAIZE_60S_ribosomal_protein_L39_OS=Zea_mays_OX=4577_GN=RPL39_PE=3_SV=1	76.471	1.99E-22
TRINITY_BIN_18342_1	sp_F2VYU4_PIK1_ORYSJ_Disease_resistance_protein_Pik-1_OS=Oryza_sativa_subsp._japonica_OX=39947_GN=PIK-1_PE=1_SV=1	42.857	5.83E-09
TRINITY_BIN_19022_2	sp_O65857_GSTF1_ORYSJ_Probable_glutathione_S-transferase_GSTF1_OS=Oryza_sativa_subsp._japonica_OX=39947_GN=GSTF1_PE=1_SV=2	67.361	6.74E-73
TRINITY_BIN_19022_2	sp_O65857_GSTF1_ORYSJ_Probable_glutathione_S-transferase_GSTF1_OS=Oryza_sativa_subsp._japonica_OX=39947_GN=GSTF1_PE=1_SV=2	75.472	6.74E-73
TRINITY_BIN_19291_1	sp_Q9P6C8_ADH1_NEUCR_Alcohol_dehydrogenase_1_OS=Neurospora_crassa_(strain_ATCC_24698_/_74-OR23-1A_/_CBS_708.71_/_DSM_1257_/_FGSC_987)_OX=367110_GN=adh-1_PE=3_SV=1	50.986	4.16E-120
TRINITY_BIN_19720_1	sp_B2IGK2_GCSH_BEII9_Glycine_cleavage_system_H_protein_OS=Beijerinckia_indica_subsp._indica_(strain_ATCC_9039_/_DSM_1715_/_NCIB_8712)_OX=395963_GN=gcvH_PE=3_SV=1	60.606	1.03E-37

TRINITY_BIN_2061_1	sp_P52032_GPX1_ARATH_Phospholipid_hydroperoxidase_glutathione_peroxidase_1,_chloroplastic_OS=Arabidopsis_thaliana_OX=3702_GN=GPX1_PE=2_SV=2	84.94	2.12E-101
TRINITY_BIN_21114_1	sp_Q9H8Y5_ANKZ1_HUMAN_Ankyrin_repeat_and_zinc_finger_domain-containing_protein_1_OS=Homo_sapiens_OX=9606_GN=ANKZF1_PE=1_SV=1	35.018	7.81E-26
TRINITY_BIN_2309_14	sp_P81713_IBB3_WHEAT_Bowman-Birk_type_trypsin_inhibitor_OS=Triticum_aestivum_OX=4565_PE=1_SV=1	72.727	7.39E-24
TRINITY_BIN_23317_1	sp_Q9XFR5_SR30_ARATH_Serine/arginine-rich_splicing_factor_SR30_OS=Arabidopsis_thaliana_OX=3702_GN=SR30_PE=1_SV=1	41.758	1.56E-32
TRINITY_BIN_234_2	sp_Q8TGM6_TAR1_YEAST_Protein_TAR1_OS=Saccharomyces_cerevisiae_(strain_ATCC_204508_/_S288c)_OX=559292_GN=TAR1_PE=2_SV=1	51.163	9.1E-15
TRINITY_BIN_24345_1	sp_A8XGH1_RPAB1_CAEBR_DNA-directed_RNA_polymerases_I,_II,_and_III_subunit_RPABC1_OS=Caenorhabditis_briggsae_OX=6238_GN=rpb-5_PE=3_SV=1	57.561	1.85E-87
TRINITY_BIN_2441_5	sp_Q2RBR1_PHT1B_ORYSJ_Phototropin-1B_OS=Oryza_sativa_subsp._japonica_OX=39947_GN=PHOT1B_PE=1_SV=2	84.457	0
TRINITY_BIN_2441_5	sp_Q2RBR1_PHT1B_ORYSJ_Phototropin-1B_OS=Oryza_sativa_subsp._japonica_OX=39947_GN=PHOT1B_PE=1_SV=2	56.41	7.89E-14
TRINITY_BIN_2591_2	sp_Q86FP7_RS23_DERVA_40S_ribosomal_protein_S23_OS=Dermacentor_variabilis_OX=34621_GN=RpS23_PE=2_SV=1	78.014	2.2E-77
TRINITY_BIN_26423_2	sp_Q2KIL4_COQ6_BOVIN_Ubiquinone_biosynthesis_monooxygenase_COQ6,_mitochondrial_OS=Bos_taurus_OX=9913_GN=COQ6_PE=2_SV=2	36.797	3.47E-70
TRINITY_BIN_26502_1	sp_P61291_PSME3_PIG_Proteasome_activator_complex_subunit_3_OS=Sus_scrofa_OX=9823_GN=PSME3_PE=2_SV=1	24.855	1.83E-09
TRINITY_BIN_27713_1	sp_P0CT65_RS16B_SCHPO_40S_ribosomal_protein_S16-B_OS=Schizosaccharomyces_pombe_(strain_972_/_ATCC_24843)_OX=284812_GN=rps1602_PE=3_SV=1	76.259	1.96E-73
TRINITY_BIN_288_4	sp_P26182_ACT_ACHBI_Actin_OS=Achlya_bisexualis_OX=4766_PE=3_SV=1	94.164	0
TRINITY_BIN_2887_1	sp_Q2QXM3_STLP2_ORYSJ_Sialyltransferase-like_protein_2_OS=Oryza_sativa_subsp._japonica_OX=39947_GN=STLP2_PE=2_SV=1	76.543	2.47E-162
TRINITY_BIN_29282_1	sp_P77493_YDJH_ECOLI_Uncharacterized_sugar_kinase_YdjH_OS=Escherichia_coli_(strain_K12)_OX=83333_GN=ydjH_PE=1_SV=2	24.606	8.32E-12
TRINITY_BIN_29296_3	sp_Q9HXI4_SUHB_PSEAE_Inositol-1-monophosphatase_OS=Pseudomonas_aeruginosa_(strain_ATCC_15692_/_DSM_22644_/_CIP_104116_/_JCM_14847_/_LMG_12228_/_1C_/_PRS_101_/_PAO1)_OX=208964_GN=suhB_PE=3_SV=1	38.462	1.58E-45
TRINITY_BIN_2964_5	sp_O80662_TCHQD_ARATH_Glutathione_S-transferase_TCHQD_OS=Arabidopsis_thaliana_OX=3702_GN=TCHQD_PE=2_SV=1	61.654	2.68E-117
TRINITY_BIN_30079_1	sp_P53184_PNC1_YEAST_Nicotinamidase_OS=Saccharomyces_cerevisiae_(strain_ATCC_204508_/_S288c)_OX=559292_GN=PNC1_PE=1_SV=1	26.275	1.28E-08
TRINITY_BIN_3016_28	sp_Q2QMX9_ACA10_ORYSJ_Calcium-transporting_ATPase_10,_plasma_membrane-type_OS=Oryza_sativa_subsp._japonica_OX=39947_GN=ACA10_PE=2_SV=1	79.216	0
TRINITY_BIN_3181_1	sp_Q1HQU2_RL5_AEDAE_60S_ribosomal_protein_L5_OS=Aedes_aegypti_OX=7159_GN=RpL5_PE=2_SV=1	58.163	4.88E-96
TRINITY_BIN_32460_1	sp_F1RBN2_SPAG1_DANRE_Sperm-associated_antigen_1A_OS=Danio_rerio_OX=7955_GN=spag1a_PE=2_SV=1	28.369	1.83E-19
TRINITY_BIN_32460_1	sp_F1RBN2_SPAG1_DANRE_Sperm-associated_antigen_1A_OS=Danio_rerio_OX=7955_GN=spag1a_PE=2_SV=1	41.525	5.56E-17
TRINITY_BIN_3276_1	sp_Q9FLV9_SLAH3_ARATH_S-type_anion_channel_SLAH3_OS=Arabidopsis_thaliana_OX=3702_GN=SLAH3_PE=1_SV=1	49.367	3.16E-19
TRINITY_BIN_3276_1	sp_Q9FLV9_SLAH3_ARATH_S-type_anion_channel_SLAH3_OS=Arabidopsis_thaliana_OX=3702_GN=SLAH3_PE=1_SV=1	57.576	3.16E-19
TRINITY_BIN_3276_1	sp_Q9FLV9_SLAH3_ARATH_S-type_anion_channel_SLAH3_OS=Arabidopsis_thaliana_OX=3702_GN=SLAH3_PE=1_SV=1	46.429	3.16E-19
TRINITY_BIN_3678_3	sp_P0DJ19_RL27_TETTH_60S_ribosomal_protein_L27_OS=Tetrahymena_thermophila_OX=5911_GN=RPL27_PE=1_SV=1	52.113	5E-33
TRINITY_BIN_4317_1	sp_B8BHF1_GLO15_ORYSI_Very-long-chain_aldehyde_decarbonylase_GL1-5_OS=Oryza_sativa_subsp._indica_OX=39946_GN=GL1-5_PE=2_SV=1	84.583	0
TRINITY_BIN_4326_1	sp_Q9HFQ4_RLA4_CANAX_60S_acidic_ribosomal_protein_P2-B_OS=Candida_albicans_OX=5476_GN=RPP2B_PE=1_SV=1	69.697	2.67E-21
TRINITY_BIN_4529_4	sp_P51204_YCF54_PORPU_Uncharacterized_protein_ycf54_OS=Porphyra_purpurea_OX=2787_GN=ycf54_PE=3_SV=1	38.462	4.54E-11
TRINITY_BIN_4819_4	sp_F4JQ95_DGK7_ARATH_Diacylglycerol_kinase_7_OS=Arabidopsis_thaliana_OX=3702_GN=DGK7_PE=1_SV=1	60.656	0
TRINITY_BIN_4977_1	sp_Q43814_OTC_PEA_Ornithine_carbamoyltransferase,_chloroplastic_OS=Pisum_sativum_OX=3888_GN=ARGF_PE=2_SV=1	83.113	0
TRINITY_BIN_5058_1	sp_P08927_RUBB_PEA_RuBisCO_large_subunit-binding_protein_subunit_beta,_chloroplastic_OS=Pisum_sativum_OX=3888_PE=1_SV=2	84.992	0
TRINITY_BIN_5117_3	sp_Q39129_STR16_ARATH_Thiosulfate_sulfurtransferase_16,_chloroplastic_OS=Arabidopsis_thaliana_OX=3702_GN=STR16_PE=1_SV=2	54.369	4.11E-33
TRINITY_BIN_5217_3	sp_O82197_VP321_ARATH_Vacuolar_protein_sorting-associated_protein_32_homolog_1_OS=Arabidopsis_thaliana_OX=3702_GN=VPS32.1_PE=1_SV=1	80.272	2.05E-77
TRINITY_BIN_5760_7	sp_Q6I581_GH35_ORYSJ_Jasmonic_acid-amido_synthetase_JAR1_OS=Oryza_sativa_subsp._japonica_OX=39947_GN=GH3.5_PE=2_SV=1	63.715	0
TRINITY_BIN_5972_3	sp_O23049_RK6_ARATH_50S_ribosomal_protein_L6,_chloroplastic_OS=Arabidopsis_thaliana_OX=3702_GN=RPL6_PE=2_SV=1	67.757	6.62E-103

TRINITY_BIN_5994_1	sp_Q9SWI6_RK29_MAIZE_50S_ribosomal_protein_L29,_chloroplastic_OS=Zea_mays_OX=4577_GN=RPL29_PE=2_SV=1	85.897	1.26E-81
TRINITY_BIN_6542_1	sp_Q8L803_RK9_WHEAT_50S_ribosomal_protein_L9,_chloroplastic_OS=Triticum_aestivum_OX=4565_GN=RPL9_PE=2_SV=1	90.062	5.12E-97
TRINITY_BIN_6546_2	sp_F7J0N2_RGA5R_ORYSJ_Disease_resistance_protein_RGA5_OS=Oryza_sativa_subsp._japonica_OX=39947_GN=RGA5_PE=1_SV=1	43.662	0.0000016
TRINITY_BIN_6636_2	sp_Q8LGS1_MARD1_ARATH_Protein_MARD1_OS=Arabidopsis_thaliana_OX=3702_GN=MARD1_PE=1_SV=2	56.757	7.04E-09
TRINITY_BIN_6778_3	sp_O23877_FENR3_ORYSJ_Ferredoxin--NADP_reductase,_embryo_isozyme,_chloroplastic_OS=Oryza_sativa_subsp._japonica_OX=39947_GN=Os07g0147900_PE=1_SV=1	94.737	0
TRINITY_BIN_6782_2	sp_Q9FVR6_Y1222_ARATH_Uncharacterized_protein_At1g32220,_chloroplastic_OS=Arabidopsis_thaliana_OX=3702_GN=At1g32220_PE=1_SV=1	74.231	1.32E-143
TRINITY_BIN_7439_3	sp_P80607_RGP1_MAIZE_Probable_UDP-arabinopyranose_mutase_1_OS=Zea_mays_OX=4577_GN=UPTG_PE=1_SV=2	91.964	2.1E-133
TRINITY_BIN_7439_3	sp_P80607_RGP1_MAIZE_Probable_UDP-arabinopyranose_mutase_1_OS=Zea_mays_OX=4577_GN=UPTG_PE=1_SV=2	98.824	2.1E-133
TRINITY_BIN_7439_3	sp_P80607_RGP1_MAIZE_Probable_UDP-arabinopyranose_mutase_1_OS=Zea_mays_OX=4577_GN=UPTG_PE=1_SV=2	100	2.1E-133
TRINITY_BIN_7735_17	sp_Q3UDW8_HGNAT_MOUSE_Heparan-alpha-glucosaminide_N-acetyltransferase_OS=Mus_musculus_OX=10090_GN=Hgsnat_PE=1_SV=2	28.205	1.65E-37
TRINITY_BIN_7739_1	sp_Q9FFB3_VP241_ARATH_Vacuolar_protein_sorting-associated_protein_24_homolog_1_OS=Arabidopsis_thaliana_OX=3702_GN=VPS24-1_PE=1_SV=1	73.82	1.01E-87
TRINITY_BIN_7741_3	sp_F4JTP5_STY46_ARATH_Serine/threonine-protein_kinase_STY46_OS=Arabidopsis_thaliana_OX=3702_GN=STY46_PE=1_SV=1	43.223	1.84E-142
TRINITY_BIN_8228_2	sp_Q39033_PLCD2_ARATH_Phosphoinositide_phospholipase_C_2_OS=Arabidopsis_thaliana_OX=3702_GN=PLC2_PE=1_SV=1	56.343	0
TRINITY_BIN_8556_1	sp_Q1XDE8_RK12_PYRYE_50S_ribosomal_protein_L12,_chloroplastic_OS=Pyropia_yezoensis_OX=2788_GN=rpl12_PE=3_SV=1	44.355	1.37E-19
TRINITY_BIN_866_7	sp_P13917_7SB1_SOYBN_Basic_7S_globulin_OS=Glycine_max_OX=3847_GN=BG_PE=1_SV=2	27.465	3.14E-21
TRINITY_BIN_9616_2	sp_Q940U6_FLU_ARATH_Protein_FLUORESCENT_IN_BLUE_LIGHT,_chloroplastic_OS=Arabidopsis_thaliana_OX=3702_GN=FLU_PE=1_SV=1	56.522	4.78E-105
TRINITY_DN22191_c2_g1_i2	sp_Q5VQG8_TAR2_ORYSJ_Tryptophan_aminotransferase-related_protein_2_OS=Oryza_sativa_subsp._japonica_OX=39947_GN=TAR2_PE=1_SV=1	66.01	3.77E-114
TRINITY_DN22191_c2_g1_i2	sp_Q5VQG8_TAR2_ORYSJ_Tryptophan_aminotransferase-related_protein_2_OS=Oryza_sativa_subsp._japonica_OX=39947_GN=TAR2_PE=1_SV=1	62.048	3.77E-114
TRINITY_DN13181_c0_g2_i1	sp_O01725_RLA2_BRAFL_60S_acidic_ribosomal_protein_P2_OS=Branchiostoma_floridae_OX=7739_PE=3_SV=1	68.254	1.13E-22
TRINITY_DN13181_c0_g8_i1	sp_P05389_RLA2_DROME_60S_acidic_ribosomal_protein_P2_OS=Drosophila_melanogaster_OX=7227_GN=RpLP2_PE=1_SV=1	51.613	3.48E-10
TRINITY_DN13113_c0_g1_i1	sp_Q55AI5_SUCB1_DICDI_Succinate--CoA_ligase_[ADP-forming]_subunit_beta,_mitochondrial_OS=Dictyostelium_discoideum_OX=44689_GN=scsC_PE=3_SV=1	55.402	1.72E-168
TRINITY_DN13194_c0_g2_i1	sp_A4R188_RSSA_MAGO7_40S_ribosomal_protein_S0_OS=Magnaporthe_oryzae_(strain_70-15_/_ATCC_MYA-4617_/_FGSC_8958)_OX=242507_GN=RPS0_PE=3_SV=1	67.692	3.43E-90
TRINITY_DN14113_c0_g1_i1	sp_Q94BU8_GSDA_ARATH_Guanosine_deaminase_OS=Arabidopsis_thaliana_OX=3702_GN=GSDA_PE=1_SV=1	84.946	1E-100
TRINITY_DN22308_c1_g5_i10	sp_Q0JA81_KSL2_ORYSJ_Ent-kaurene_synthase-like_2_OS=Oryza_sativa_subsp._japonica_OX=39947_GN=KSL2_PE=2_SV=2	53.295	0
TRINITY_DN22308_c1_g5_i10	sp_Q0JA81_KSL2_ORYSJ_Ent-kaurene_synthase-like_2_OS=Oryza_sativa_subsp._japonica_OX=39947_GN=KSL2_PE=2_SV=2	42.373	0
TRINITY_DN22307_c0_g3_i1	sp_O64483_SIRK_ARATH_Senescence-induced_receptor-like_serine/threonine-protein_kinase_OS=Arabidopsis_thaliana_OX=3702_GN=SIRK_PE=1_SV=1	46.465	1.37E-19
TRINITY_DN22355_c0_g5_i2	sp_Q7XJE6_MCA1_ARATH_Metacaspase-1_OS=Arabidopsis_thaliana_OX=3702_GN=AMC1_PE=1_SV=1	45.55	2.49E-47
TRINITY_DN22383_c0_g1_i1	sp_Q10SY2_EL2_ORYSJ_Cyclin-dependent_protein_kinase_inhibitor_EL2_OS=Oryza_sativa_subsp._japonica_OX=39947_GN=EL2_PE=1_SV=1	50	1.31E-12
TRINITY_DN22323_c2_g1_i3	sp_P10978_POLX_TOBAC_Retrovirus-related_Pol_polyprotein_from_transposon_TNT_1-94_OS=Nicotiana_tabacum_OX=4097_PE=2_SV=1	23.035	8.65E-22
TRINITY_DN21277_c4_g1_i4	sp_Q9LR30_GGT1_ARATH_Glutamate--glyoxylate_aminotransferase_1_OS=Arabidopsis_thaliana_OX=3702_GN=GGAT1_PE=1_SV=1	83.368	0
TRINITY_DN18492_c0_g1_i1	sp_Q9S9N9_CCR1_ARATH_Cinnamoyl-CoA_reductase_1_OS=Arabidopsis_thaliana_OX=3702_GN=CCR1_PE=1_SV=1	42.544	2.08E-47
TRINITY_DN18492_c0_g1_i1	sp_Q9S9N9_CCR1_ARATH_Cinnamoyl-CoA_reductase_1_OS=Arabidopsis_thaliana_OX=3702_GN=CCR1_PE=1_SV=1	49.438	2.08E-47
TRINITY_DN15899_c0_g3_i5	sp_Q942L2_PDI22_ORYSJ_Protein_disulfide_isomerase-like_2-2_OS=Oryza_sativa_subsp._japonica_OX=39947_GN=PDIL2-2_PE=2_SV=1	91.239	0
TRINITY_DN15899_c0_g3_i5	sp_Q942L2_PDI22_ORYSJ_Protein_disulfide_isomerase-like_2-2_OS=Oryza_sativa_subsp._japonica_OX=39947_GN=PDIL2-2_PE=2_SV=1	47.273	1.54E-25
TRINITY_DN11532_c0_g1_i1	sp_Q8L9C4_KCR1_ARATH_Very-long-chain_3-oxoacyl-CoA_reductase_1_OS=Arabidopsis_thaliana_OX=3702_GN=KCR1_PE=1_SV=1	42.045	5.24E-74
TRINITY_DN11514_c0_g1_i1	sp_Q6INR1_EIF3J_XENLA_Eukaryotic_translation_initiation_factor_3_subunit_J_OS=Xenopus_laevis_OX=8355_GN=eif3j_PE=2_SV=1	30.5	1.92E-12

TRINITY_DN16940_c0_g4_i2	sp_Q96520_PER12_ARATH_Peroxidase_12_OS=Arabidopsis_thaliana_OX=3702_GN=PER12_PE=1_SV=1	38.719	6.54E-57
TRINITY_DN16990_c4_g1_i6	sp_Q9LXA5_LRK91_ARATH_L-type_lectin-domain_containing_receptor_kinase_IX.1_OS=Arabidopsis_thaliana_OX=3702_GN=LECRK91_PE=1_SV=1	33.108	2.09E-68
TRINITY_DN20690_c3_g1_i1	sp_Q6K1S6_MYB30_ORYSJ_Transcription_factor_MYB30_OS=Oryza_sativa_subsp._japonica_OX=39947_GN=MYB30_PE=1_SV=1	53.804	6.21E-28
TRINITY_DN14370_c0_g2_i2	sp_Q3BAI2_YCX91_PHAAO_Uncharacterized_protein_ORF91_OS=Phalaenopsis_aphrodite_subsp._formosana_OX=308872_PE=4_SV=1	63.793	1.04E-16
TRINITY_DN18997_c2_g1_i1	sp_A1E9W2_RK16_SORBI_50S_ribosomal_protein_L16,_chloroplastic_OS=Sorghum_bicolor_OX=4558_GN=rpl16_PE=3_SV=1	99.248	1.35E-91
TRINITY_DN19161_c0_g4_i5	sp_O04616_CUT1A_ARATH_Protein_CURVATURE_THYLAKOID_1A,_chloroplastic_OS=Arabidopsis_thaliana_OX=3702_GN=CURT1A_PE=1_SV=1	41.237	3.56E-18
TRINITY_DN22249_c0_g1_i3	sp_Q9FQA3_GST23_MAIZE_Glutathione_transferase_GST_23_OS=Zea_mays_OX=4577_PE=2_SV=1	90.566	1.75E-62
TRINITY_DN13944_c0_g1_i9	sp_Q99090_CPRF2_PETCR_Light-inducible_protein_CPRF2_OS=Petroselinum_crispum_OX=4043_GN=CPRF2_PE=2_SV=2	40.385	3.82E-22
TRINITY_DN13918_c0_g8_i1	sp_Q42993_CHI1_ORYSJ_Chitinase_1_OS=Oryza_sativa_subsp._japonica_OX=39947_GN=Cht1_PE=2_SV=1	72.459	8.65E-137
TRINITY_DN10023_c0_g1_i1	sp_Q26998_UPP_TOXGO_Uracil_phosphoribosyltransferase_OS=Toxoplasma_gondii_OX=5811_GN=uprt_PE=1_SV=1	45.581	1.15E-54
TRINITY_DN10099_c0_g1_i3	sp_Q9C912_R35A3_ARATH_60S_ribosomal_protein_L35a-3_OS=Arabidopsis_thaliana_OX=3702_GN=RPL35AC_PE=3_SV=1	52.83	2.29E-39
TRINITY_DN11686_c0_g1_i1	sp_Q9FWR5_GCA1_ARATH_Gamma_carbonic_anhydrase_1,_mitochondrial_OS=Arabidopsis_thaliana_OX=3702_GN=GAMMACA1_PE=1_SV=1	44.811	1.59E-66
TRINITY_DN15798_c1_g1_i3	sp_F4JJE0_CCT2_ARATH_Choline-phosphate_cytidylyltransferase_2_OS=Arabidopsis_thaliana_OX=3702_GN=CCT2_PE=1_SV=1	79.339	3.96E-145
TRINITY_DN22582_c1_g2_i2	sp_Q6YV88_C71Z7_ORYSJ_Ent-cassadiene_hydroxylase_OS=Oryza_sativa_subsp._japonica_OX=39947_GN=CYP71Z7_PE=1_SV=1	57.292	5.08E-179
TRINITY_DN20129_c1_g1_i1	sp_Q9LUI1_LRX6_ARATH_Leucine-rich_repeat_extensin-like_protein_6_OS=Arabidopsis_thaliana_OX=3702_GN=LRX6_PE=2_SV=1	56.707	1.53E-127
TRINITY_DN20137_c1_g2_i1	sp_Q9LV58_MBF1C_ARATH_Multiprotein-bridging_factor_1c_OS=Arabidopsis_thaliana_OX=3702_GN=MBF1C_PE=1_SV=1	59.589	2.88E-36
TRINITY_DN18526_c2_g1_i3	sp_Q67UP9_P2C58_ORYSJ_Probable_protein_phosphatase_2C_58_OS=Oryza_sativa_subsp._japonica_OX=39947_GN=Os06g0651600_PE=2_SV=1	87.149	2.04E-167
TRINITY_DN12328_c0_g2_i3	sp_Q962R9_RS10_SPOFR_40S_ribosomal_protein_S10_OS=Spodoptera_frugiperda_OX=7108_GN=RpS10_PE=2_SV=1	64.286	1.86E-44
TRINITY_DN12369_c0_g1_i1	sp_O48529_STR9_ARATH_Rhodanese-like_domain-containing_protein_9,_chloroplastic_OS=Arabidopsis_thaliana_OX=3702_GN=STR9_PE=2_SV=1	69.417	2.56E-99
TRINITY_DN21758_c2_g1_i6	sp_Q53NF7_NAC71_ORYSJ_NAC_domain-containing_protein_71_OS=Oryza_sativa_subsp._japonica_OX=39947_GN=NAC071_PE=1_SV=1	41.618	3.71E-10
TRINITY_DN13209_c0_g3_i4	sp_Q02921_NO93_SOYBN_Early_nodulin-93_OS=Glycine_max_OX=3847_PE=2_SV=1	67.021	3.38E-27
TRINITY_DN13272_c0_g1_i12	sp_Q0JIL1_NRX2_ORYSJ_Probable_nucleoredoxin_2_OS=Oryza_sativa_subsp._japonica_OX=39947_GN=Os01g0794400_PE=2_SV=1	82.946	0
TRINITY_DN18844_c0_g1_i1	sp_H2DH22_C7A10_PANGI_Cytochrome_P450_CYP73A100_OS=Panax_ginseng_OX=4054_PE=2_SV=1	70.659	0
TRINITY_DN19057_c0_g5_i4	sp_Q766C2_NEP2_NEPGR_Aspartic_proteinase_nepenthesin-2_OS=Nepenthes_gracilis_OX=150966_GN=nep2_PE=1_SV=1	38.166	1.5E-60
TRINITY_DN19026_c3_g2_i5	sp_P42736_RAP23_ARATH_Ethylene-responsive_transcription_factor_RAP2-3_OS=Arabidopsis_thaliana_OX=3702_GN=RAP2-3_PE=1_SV=2	50.746	1.84E-29
TRINITY_DN19859_c0_g3_i1	sp_Q02028_HSP7S_PEA_Stromal_70_kDa_heat_shock-related_protein,_chloroplastic_OS=Pisum_sativum_OX=3888_GN=HSP70_PE=2_SV=1	89.72	0
TRINITY_DN14058_c0_g1_i2	sp_Q8RVH5_7SBG2_SOYBN_Basic_7S_globulin_2_OS=Glycine_max_OX=3847_PE=1_SV=1	30.806	2.28E-35
TRINITY_DN17534_c0_g2_i11	sp_Q0JAW2_SPXM2_ORYSJ_SPX_domain-containing_membrane_protein_Os04g0573000_OS=Oryza_sativa_subsp._japonica_OX=39947_GN=Os04g0573000_PE=2_SV=2	88.075	0
TRINITY_DN15636_c2_g3_i5	sp_Q6RYA0_SABP2_TOBAC_Salicylic_acid-binding_protein_2_OS=Nicotiana_tabacum_OX=4097_GN=SABP2_PE=1_SV=1	51.316	3.61E-48
TRINITY_DN17322_c1_g2_i6	sp_Q8H0W3_RVE6_ARATH_Protein_REVEILLE_6_OS=Arabidopsis_thaliana_OX=3702_GN=RVE6_PE=2_SV=1	59.322	6.77E-11
TRINITY_DN19722_c1_g1_i7	sp_Q33E23_DHE2_ORYSJ_Glutamate_dehydrogenase_2,_mitochondrial_OS=Oryza_sativa_subsp._japonica_OX=39947_GN=GDH2_PE=2_SV=1	90.123	0
TRINITY_DN10773_c0_g4_i1	sp_P62832_RL23_RAT_60S_ribosomal_protein_L23_OS=Rattus_norvegicus_OX=10116_GN=Rpl23_PE=2_SV=1	86.331	5.63E-84
TRINITY_DN11954_c3_g2_i2	sp_O48557_RL17_MAIZE_60S_ribosomal_protein_L17_OS=Zea_mays_OX=4577_GN=RPL17_PE=2_SV=1	95.679	3.03E-113
TRINITY_DN21819_c0_g1_i5	sp_Q9ATL7_TIP31_MAIZE_Aquaporin_TIP3-1_OS=Zea_mays_OX=4577_GN=TIP3-1_PE=2_SV=1	95.473	2.36E-125
TRINITY_DN21825_c1_g1_i6	sp_Q9FZB8_Y5181_ARATH_Probable_LRR_receptor-like_serine/threonine-protein_kinase_At1g51810_OS=Arabidopsis_thaliana_OX=3702_GN=At1g51810_PE=1_SV=1	33.333	9.16E-38
TRINITY_DN21825_c1_g1_i6	sp_Q9FZB8_Y5181_ARATH_Probable_LRR_receptor-like_serine/threonine-protein_kinase_At1g51810_OS=Arabidopsis_thaliana_OX=3702_GN=At1g51810_PE=1_SV=1	52.679	4.59E-29
TRINITY_DN12804_c0_g2_i3	sp_Q62785_HAP28_RAT_28_kDa_heat-_and_acid-stable_phosphoprotein_OS=Rattus_norvegicus_OX=10116_GN=Pdap1_PE=1_SV=1	62.338	3.53E-12
TRINITY_DN16290_c0_g1_i1	sp_Q9M1T1_SETH3_ARATH_Probable_arabinose_5-phosphate_isomerase_OS=Arabidopsis_thaliana_OX=3702_GN=SETH3_PE=2_SV=1	66.102	9.43E-154
TRINITY_DN15047_c0_g1_i5	sp_Q9SKI2_VPS2A_ARATH_Vacuolar_protein_sorting-associated_protein_2_homolog_1_OS=Arabidopsis_thaliana_OX=3702_GN=VPS2.1_PE=1_SV=2	85.068	5.45E-103

TRINITY_DN13853_c0_g1_i5	sp_Q94B38_GPT2_ARATH_Glucose-6-phosphate/phosphate_translocator_2,_chloroplastic_OS=Arabidopsis_thaliana_OX=3702_GN=GPT2_PE=2_SV=2	83.713	3.61E-164
TRINITY_DN13829_c0_g1_i3	sp_P02362_RS7_XENLA_40S_ribosomal_protein_S7_OS=Xenopus_laevis_OX=8355_GN=rps7_PE=2_SV=2	60.541	8.95E-70
TRINITY_DN13872_c0_g1_i3	sp_Q2R0D1_CSPLK_ORYSJ_CASP-like_protein_1U2_OS=Oryza_sativa_subsp._japonica_OX=39947_GN=Os11g0649700_PE=2_SV=1	62.5	2.27E-27
TRINITY_DN14275_c3_g2_i2	sp_P42794_RL112_ARATH_60S_ribosomal_protein_L11-2_OS=Arabidopsis_thaliana_OX=3702_GN=RPL11B_PE=2_SV=2	74.118	1.01E-91
TRINITY_DN14268_c0_g1_i2	sp_Q9LFD5_BPA1_ARATH_Binding_partner_of_ACD11_1_OS=Arabidopsis_thaliana_OX=3702_GN=BPA1_PE=1_SV=1	38.743	1.17E-29
TRINITY_DN14203_c1_g2_i2	sp_P04748_AMY4_HORVU_Alpha-amylase_type_B_isozyme_(Fragment)_OS=Hordeum_vulgare_OX=4513_GN=AMY1.4_PE=2_SV=1	78.689	5.24E-67
TRINITY_DN19360_c1_g1_i2	sp_Q9SYL9_RK13_ARATH_50S_ribosomal_protein_L13,_chloroplastic_OS=Arabidopsis_thaliana_OX=3702_GN=RPL13_PE=2_SV=1	76.44	1.09E-100
TRINITY_DN19304_c2_g2_i1	sp_Q9FNL7_PTR3_ARATH_Protein_NRT1/_PTR_FAMILY_5.2_OS=Arabidopsis_thaliana_OX=3702_GN=NPF5.2_PE=2_SV=1	66.667	0
TRINITY_DN19320_c1_g1_i1	sp_Q54I39_IST1L_DICDI_IST1-like_protein_OS=Dictyostelium_discoideum_OX=44689_GN=DDB_G0289029_PE=3_SV=1	30.846	1.53E-15
TRINITY_DN12685_c0_g1_i3	sp_Q9S7L9_CX6B1_ARATH_Cytochrome_c_oxidase_subunit_6b-1_OS=Arabidopsis_thaliana_OX=3702_GN=COX6B-1_PE=1_SV=1	95.588	5.66E-45
TRINITY_DN18155_c1_g2_i3	sp_Q0D5R3_CRK6_ORYSJ_Cysteine-rich_receptor-like_protein_kinase_6_OS=Oryza_sativa_subsp._japonica_OX=39947_GN=CRK6_PE=1_SV=1	45.993	1.07E-150
TRINITY_DN18183_c1_g7_i2	sp_A1E9U6_RR18_SORBI_30S_ribosomal_protein_S18,_chloroplastic_OS=Sorghum_bicolor_OX=4558_GN=rps18_PE=3_SV=1	96.403	8.15E-82
TRINITY_DN22097_c1_g1_i4	sp_Q6Z4G8_COBL1_ORYSJ_COBRA-like_protein_1_OS=Oryza_sativa_subsp._japonica_OX=39947_GN=BC1L6_PE=2_SV=1	89.189	0
TRINITY_DN15239_c3_g3_i3	sp_Q6CQE5_TAR1_KLULA_Protein_TAR1_OS=Kluyveromyces_lactis_(strain_ATCC_8585_/_CBS_2359_/_DSM_70799_/_NBRC_1267_/_NRRL_Y-1140_/_WM37)_OX=284590_GN=TAR1-A_PE=4_SV=2	61.538	2.42E-25
TRINITY_DN15262_c1_g1_i13	sp_Q9LTK0_IPP2_ARATH_Protein_phosphatase_inhibitor_2_OS=Arabidopsis_thaliana_OX=3702_GN=I-2_PE=1_SV=1	52.239	1.34E-30
TRINITY_DN16027_c0_g1_i1	sp_Q6L4D2_PM19L_ORYSJ_Membrane_protein_PM19L_OS=Oryza_sativa_subsp._japonica_OX=39947_GN=PM19L_PE=2_SV=1	47.531	8.74E-12
TRINITY_DN16050_c0_g3_i1	sp_Q9LMN8_WAK3_ARATH_Wall-associated_receptor_kinase_3_OS=Arabidopsis_thaliana_OX=3702_GN=WAK3_PE=2_SV=2	31.292	3.84E-92
TRINITY_DN16575_c2_g3_i1	sp_Q3E813_YL154_YEAST_Uncharacterized_protein_YLR154C-G_OS=Saccharomyces_cerevisiae_(strain_ATCC_204508_/_S288c)_OX=559292_GN=YLR154C-G_PE=2_SV=1	55.814	0.00000199
TRINITY_DN16840_c0_g1_i8	sp_Q6ENW9_ATPI_SACOF_ATP_synthase_subunit_a,_chloroplastic_OS=Saccharum_officinarum_OX=4547_GN=atpI_PE=3_SV=1	99.595	2.53E-141
TRINITY_DN16874_c0_g2_i5	sp_P49353_FPPS_MAIZE_Farnesyl_pyrophosphate_synthase_OS=Zea_mays_OX=4577_GN=FPS_PE=2_SV=1	86.471	0
TRINITY_DN18700_c0_g1_i3	sp_P51615_MAOX_VITVI_NADP-dependent_malic_enzyme_OS=Vitis_vinifera_OX=29760_PE=2_SV=1	80.142	0
TRINITY_DN18660_c1_g2_i26	sp_Q3SZX8_CCD25_BOVIN_Coiled-coil_domain-containing_protein_25_OS=Bos_taurus_OX=9913_GN=CCDC25_PE=2_SV=1	62.312	3.9E-69
TRINITY_DN11789_c0_g1_i5	sp_Q9SBK8_THN_BRARP_Thionin_OS=Brassica_rapa_subsp._pekinensis_OX=51351_GN=THI2_PE=2_SV=1	40.476	0.00000208
TRINITY_DN11762_c0_g1_i1	sp_Q9M897_DMP5_ARATH_Protein_DMP5_OS=Arabidopsis_thaliana_OX=3702_GN=DMP5_PE=2_SV=1	38.614	1.08E-36
TRINITY_DN16356_c0_g1_i1	sp_Q8RX77_PTR21_ARATH_Protein_NRT1/_PTR_FAMILY_2.13_OS=Arabidopsis_thaliana_OX=3702_GN=NPF2.13_PE=1_SV=1	48.342	3.89E-166
TRINITY_DN28970_c0_g1_i1	sp_P82953_LECH_HORVV_Horcolin_OS=Hordeum_vulgare_subsp._vulgare_OX=112509_PE=1_SV=2	33.582	1.57E-12
TRINITY_DN14950_c2_g4_i1	sp_Q8TGM6_TAR1_YEAST_Protein_TAR1_OS=Saccharomyces_cerevisiae_(strain_ATCC_204508_/_S288c)_OX=559292_GN=TAR1_PE=2_SV=1	50.909	0.000000359
TRINITY_1142_length_4255_cov_35.603679_g738_i0	sp_Q6AT26_CESA1_ORYSJ_Probable_cellulose_synthase_A_catalytic_subunit_1_[UDP-forming]_OS=Oryza_sativa_subsp._japonica_OX=39947_GN=CESA1_PE=2_SV=1	96.1	0
TRINITY_1533_length_3973_cov_27.862705_g941_i1	sp_O64477_Y2913_ARATH_G-type_lectin_S-receptor-like_serine/threonine-protein_kinase_At2g19130_OS=Arabidopsis_thaliana_OX=3702_GN=At2g19130_PE=2_SV=1	42.981	0
TRINITY_1555_length_3959_cov_33.626221_g1005_i0	sp_Q84ZN6_CESA8_ORYSJ_Probable_cellulose_synthase_A_catalytic_subunit_8_[UDP-forming]_OS=Oryza_sativa_subsp._japonica_OX=39947_GN=CESA8_PE=1_SV=1	97.595	0
TRINITY_3946_length_3071_cov_33.057295_g2507_i0	sp_P17801_KPRO_MAIZE_Putative_receptor_protein_kinase_ZmPK1_OS=Zea_mays_OX=4577_GN=PK1_PE=2_SV=2	54.134	0
TRINITY_4103_length_3034_cov_28.572344_g2612_i0	sp_Q7XQ12_AMT11_ORYSJ_Ammonium_transporter_1_member_1_OS=Oryza_sativa_subsp._japonica_OX=39947_GN=AMT1-1_PE=2_SV=1	92.813	0
TRINITY_4828_length_2893_cov_30.035765_g3070_i0	sp_O81905_SD18_ARATH_Receptor-like_serine/threonine-protein_kinase_SD1-8_OS=Arabidopsis_thaliana_OX=3702_GN=SD18_PE=1_SV=1	49.938	0
TRINITY_4942_length_2873_cov_26.481455_g3145_i0	sp_O81905_SD18_ARATH_Receptor-like_serine/threonine-protein_kinase_SD1-8_OS=Arabidopsis_thaliana_OX=3702_GN=SD18_PE=1_SV=1	38.869	1.24E-175
TRINITY_5027_length_2855_cov_19.676956_g1942_i1	sp_P82953_LECH_HORVV_Horcolin_OS=Hordeum_vulgare_subsp._vulgare_OX=112509_PE=1_SV=2	32.632	8.47E-20
TRINITY_5299_length_2804_cov_29.469835_g3364_i0	sp_P93844_PLDA2_ORYSJ_Phospholipase_D_alpha_2_OS=Oryza_sativa_subsp._japonica_OX=39947_GN=PLD2_PE=2_SV=2	62.836	0
TRINITY_5300_length_2804_cov_28.563803_g3365_i0	sp_Q8L7I2_SBT36_ARATH_Subtilisin-like_protease_SBT3.6_OS=Arabidopsis_thaliana_OX=3702_GN=SBT3.6_PE=2_SV=1	43.898	4.63E-110

TRINITY_5300_length_2804_cov_28.563803_g3365_i0	sp_Q8L7I2_SBT36_ARATH_Subtilisin-like_protease_SBT3.6_OS=Arabidopsis_thaliana_OX=3702_GN=SBT3.6_PE=2_SV=1	45.614	5.57E-53
TRINITY_5374_length_2792_cov_42.929857_g3410_i0	sp_Q3BAI2_YCX91_PHAAO_Uncharacterized_protein_ORF91_OS=Phalaenopsis_aphrodite_subsp._formosana_OX=308872_PE=4_SV=1	75.439	6.41E-20
TRINITY_5504_length_2770_cov_36.719733_g3491_i0	sp_Q3BAI2_YCX91_PHAAO_Uncharacterized_protein_ORF91_OS=Phalaenopsis_aphrodite_subsp._formosana_OX=308872_PE=4_SV=1	58.824	1.02E-20
TRINITY_5571_length_2759_cov_27.794796_g3523_i0	sp_Q117R7_METE_TRIEI_5-methyltetrahydropteroyltriglutamate--homocysteine_methyltransferase_OS=Trichodesmium_erythraeum_(strain_IMS101)_OX=203124_GN=metE_PE=3_SV=1	48.765	0
TRINITY_5616_length_2753_cov_27.887481_g3547_i0	sp_Q9LMN7_WAK5_ARATH_Wall-associated_receptor_kinase_5_OS=Arabidopsis_thaliana_OX=3702_GN=WAK5_PE=2_SV=1	42.398	2.08E-159
TRINITY_5825_length_2717_cov_37.339124_g3671_i0	sp_Q3BAI2_YCX91_PHAAO_Uncharacterized_protein_ORF91_OS=Phalaenopsis_aphrodite_subsp._formosana_OX=308872_PE=4_SV=1	62.069	1.12E-14
TRINITY_5840_length_2712_cov_37.498297_g3680_i0	sp_Q3BAI2_YCX91_PHAAO_Uncharacterized_protein_ORF91_OS=Phalaenopsis_aphrodite_subsp._formosana_OX=308872_PE=4_SV=1	73.684	5.9E-16
TRINITY_5956_length_2693_cov_37.538110_g3396_i1	sp_Q06572_AVP_HORVU_Pyrophosphate-energized_vacuolar_membrane_proton_pump_OS=Hordeum_vulgare_OX=4513_PE=2_SV=2	88.579	0
TRINITY_6905_length_2559_cov_28.179518_g4348_i0	sp_Q39191_WAK1_ARATH_Wall-associated_receptor_kinase_1_OS=Arabidopsis_thaliana_OX=3702_GN=WAK1_PE=1_SV=2	42.044	8.34E-151
TRINITY_7372_length_2502_cov_32.012741_g4658_i0	sp_Q9SZY3_SBT38_ARATH_Subtilisin-like_protease_SBT3.8_OS=Arabidopsis_thaliana_OX=3702_GN=SBT3.8_PE=3_SV=1	44.558	0
TRINITY_7546_length_2480_cov_25.817503_g4782_i0	sp_P19954_PRSP1_SPIOL_Ribosome-binding_factor_PSRP1,_chloroplastic_OS=Spinacia_oleracea_OX=3562_GN=PSRP1_PE=1_SV=2	51.502	4.79E-64
TRINITY_7703_length_2464_cov_22.626722_g4886_i0	sp_Q9ZSB1_SBT3A_ARATH_Subtilisin-like_protease_SBT3.10_OS=Arabidopsis_thaliana_OX=3702_GN=SBT3.10_PE=3_SV=2	45.515	0
TRINITY_7910_length_2436_cov_27.240811_g5005_i0	sp_P28516_PDC1_MAIZE_Pyruvate_decarboxylase_1_OS=Zea_mays_OX=4577_GN=PDC1_PE=2_SV=1	91.832	0
TRINITY_7910_length_2436_cov_27.240811_g5005_i0	sp_P28516_PDC1_MAIZE_Pyruvate_decarboxylase_1_OS=Zea_mays_OX=4577_GN=PDC1_PE=2_SV=1	93.671	0
TRINITY_8118_length_2412_cov_37.173282_g5128_i0	sp_F8RP11_HSOP_WHEAT_Hsp70-Hsp90_organizing_protein_OS=Triticum_aestivum_OX=4565_GN=HOP_PE=1_SV=1	86.585	0
TRINITY_8118_length_2412_cov_37.173282_g5128_i0	sp_F8RP11_HSOP_WHEAT_Hsp70-Hsp90_organizing_protein_OS=Triticum_aestivum_OX=4565_GN=HOP_PE=1_SV=1	29.949	1.58E-16
TRINITY_8847_length_2340_cov_23.405108_g5587_i0	sp_Q6WNR0_C81E7_MEDTR_Isoflavone_2'-hydroxylase_OS=Medicago_truncatula_OX=3880_GN=CYP81E7_PE=1_SV=1	39.167	2.77E-102
TRINITY_9507_length_2277_cov_42.851449_g5975_i0	sp_C0LGH2_Y1561_ARATH_Probable_LRR_receptor-like_serine/threonine-protein_kinase_At1g56130_OS=Arabidopsis_thaliana_OX=3702_GN=At1g56130_PE=2_SV=2	53.859	0
TRINITY_9507_length_2277_cov_42.851449_g5975_i0	sp_C0LGH2_Y1561_ARATH_Probable_LRR_receptor-like_serine/threonine-protein_kinase_At1g56130_OS=Arabidopsis_thaliana_OX=3702_GN=At1g56130_PE=2_SV=2	54.545	0
TRINITY_9543_length_2274_cov_27.739229_g5996_i0	sp_Q8H6H4_PHT11_ORYSJ_Inorganic_phosphate_transporter_1-1_OS=Oryza_sativa_subsp._japonica_OX=39947_GN=PHT1-1_PE=2_SV=1	88.566	0
TRINITY_9683_length_2260_cov_41.896394_g6079_i0	sp_Q69NX5_NCED4_ORYSJ_9-cis-epoxycarotenoid_dioxygenase_NCED4,_chloroplastic_OS=Oryza_sativa_subsp._japonica_OX=39947_GN=NCED4_PE=2_SV=1	85.417	0
TRINITY_11732_length_2093_cov_33.770257_g7338_i0	sp_Q9SIB2_KCS12_ARATH_3-ketoacyl-CoA_synthase_12_OS=Arabidopsis_thaliana_OX=3702_GN=KCS12_PE=2_SV=1	51.81	1.52E-155
TRINITY_12044_length_2070_cov_32.029985_g6712_i1	sp_Q0DV32_4CLL1_ORYSJ_4-coumarate--CoA_ligase-like_1_OS=Oryza_sativa_subsp._japonica_OX=39947_GN=4CLL1_PE=2_SV=2	74.492	0
TRINITY_12044_length_2070_cov_32.029985_g6712_i1	sp_Q0DV32_4CLL1_ORYSJ_4-coumarate--CoA_ligase-like_1_OS=Oryza_sativa_subsp._japonica_OX=39947_GN=4CLL1_PE=2_SV=2	84.211	1.43E-09
TRINITY_13077_length_2001_cov_25.057453_g8182_i0	sp_P16397_SUBF_BACSU_Bacillopeptidase_F_OS=Bacillus_subtilis_(strain_168)_OX=224308_GN=bpr_PE=1_SV=2	36.364	4.45E-60
TRINITY_13609_length_1966_cov_29.056405_g7710_i1	sp_Q9XGY7_FAR_SIMCH_Alcohol-forming_fatty_acyl-CoA_reductase_OS=Simmondsia_chinensis_OX=3999_PE=1_SV=1	46.029	3.19E-146
TRINITY_13929_length_1946_cov_17.416622_g8719_i0	sp_P81295_PRR3_JUNAS_Pathogenesis-related_protein_OS=Juniperus_ashei_OX=13101_PE=1_SV=1	60.396	2.78E-79
TRINITY_13996_length_1942_cov_29.030432_g2771_i3	sp_Q42521_DCE1_ARATH_Glutamate_decarboxylase_1_OS=Arabidopsis_thaliana_OX=3702_GN=GAD1_PE=1_SV=2	82.072	0
TRINITY_14077_length_1938_cov_22.678438_g8815_i0	sp_P43309_PPO_MALDO_Polyphenol_oxidase,_chloroplastic_OS=Malus_domestica_OX=3750_PE=2_SV=1	45.614	5.76E-143
TRINITY_14533_length_1913_cov_28.687636_g9105_i0	sp_Q9SP22_CALR_MAIZE_Calreticulin_OS=Zea_mays_OX=4577_GN=CRT_PE=2_SV=1	94.678	0
TRINITY_15397_length_1864_cov_26.900836_g9656_i0	sp_Q0DAJ3_WRK28_ORYSJ_WRKY_transcription_factor_WRKY28_OS=Oryza_sativa_subsp._japonica_OX=39947_GN=WRKY28_PE=2_SV=2	63.81	4.77E-59
TRINITY_15397_length_1864_cov_26.900836_g9656_i0	sp_Q0DAJ3_WRK28_ORYSJ_WRKY_transcription_factor_WRKY28_OS=Oryza_sativa_subsp._japonica_OX=39947_GN=WRKY28_PE=2_SV=2	66.225	4.01E-47
TRINITY_16231_length_1820_cov_32.281553_g6995_i1	sp_F4IK45_C70B2_ARATH_Cytochrome_P450_709B2_OS=Arabidopsis_thaliana_OX=3702_GN=CYP709B2_PE=2_SV=1	49.702	1.85E-174
TRINITY_16911_length_1783_cov_23.957410_g5241_i1	sp_F8WKW1_UGT2_GARJA_7-deoxyloganetin_glucosyltransferase_OS=Gardenia_jasminoides_OX=114476_GN=UGT85A24_PE=1_SV=1	58.111	0
TRINITY_16946_length_1781_cov_26.710864_g10641_i0	sp_Q9M3B4_RID3_ARATH_Protein_ROOT_INITIATION_DEFECTIVE_3_OS=Arabidopsis_thaliana_OX=3702_GN=RID3_PE=1_SV=1	41.244	3.22E-104
TRINITY_17490_length_1755_cov_25.852906_g10980_i0	sp_Q9FFT4_PDC2_ARATH_Pyruvate_decarboxylase_2_OS=Arabidopsis_thaliana_OX=3702_GN=PDC2_PE=2_SV=1	74.144	1.48E-160

TRINITY_17490_length_1755_cov_25.852906_g10980_i0	sp_Q9FFT4_PDC2_ARATH_Pyruvate_decarboxylase_2_OS=Arabidopsis_thaliana_OX=3702_GN=PDC2_PE=2_SV=1	83.824	1.48E-160
TRINITY_17490_length_1755_cov_25.852906_g10980_i0	sp_Q9FFT4_PDC2_ARATH_Pyruvate_decarboxylase_2_OS=Arabidopsis_thaliana_OX=3702_GN=PDC2_PE=2_SV=1	78.947	1.48E-160
TRINITY_17532_length_1753_cov_36.917458_g10813_i1	sp_O23877_FENR3_ORYSJ_Ferredoxin--NADP_reductase,_embryo_isozyme,_chloroplastic_OS=Oryza_sativa_subsp._japonica_OX=39947_GN=Os07g0147900_PE=1_SV=1	93.86	0
TRINITY_17846_length_1738_cov_29.339125_g11206_i0	sp_P04707_ADH2_MAIZE_Alcohol_dehydrogenase_2_OS=Zea_mays_OX=4577_GN=ADH2_PE=2_SV=1	95.251	0
TRINITY_18219_length_1719_cov_27.636970_g11446_i0	sp_I7C6E8_C7A52_PANGI_Beta-amyrin_28-monooxygenase_OS=Panax_ginseng_OX=4054_PE=1_SV=1	51.24	2.68E-52
TRINITY_18219_length_1719_cov_27.636970_g11446_i0	sp_I7C6E8_C7A52_PANGI_Beta-amyrin_28-monooxygenase_OS=Panax_ginseng_OX=4054_PE=1_SV=1	37.815	2.68E-52
TRINITY_18219_length_1719_cov_27.636970_g11446_i0	sp_I7C6E8_C7A52_PANGI_Beta-amyrin_28-monooxygenase_OS=Panax_ginseng_OX=4054_PE=1_SV=1	42.5	2.68E-52
TRINITY_18953_length_1685_cov_24.115099_g1094_i2	sp_Q654V6_ODPA2_ORYSJ_Pyruvate_dehydrogenase_E1_component_subunit_alpha-2,_mitochondrial_OS=Oryza_sativa_subsp._japonica_OX=39947_GN=Os06g0246500_PE=2_SV=1	85.64	0
TRINITY_19122_length_1678_cov_28.188937_g12037_i0	sp_Q0J6T3_CADH5_ORYSJ_Probable_cinnamyl_alcohol_dehydrogenase_5_OS=Oryza_sativa_subsp._japonica_OX=39947_GN=CAD5_PE=3_SV=2	82.059	0
TRINITY_19610_length_1656_cov_26.684310_g12334_i0	sp_P16397_SUBF_BACSU_Bacillopeptidase_F_OS=Bacillus_subtilis_(strain_168)_OX=224308_GN=bpr_PE=1_SV=2	34.018	4.37E-58
TRINITY_19795_length_1647_cov_30.508872_g10193_i1	sp_Q9S9N9_CCR1_ARATH_Cinnamoyl-CoA_reductase_1_OS=Arabidopsis_thaliana_OX=3702_GN=CCR1_PE=1_SV=1	41.667	3.83E-42
TRINITY_19795_length_1647_cov_30.508872_g10193_i1	sp_Q9S9N9_CCR1_ARATH_Cinnamoyl-CoA_reductase_1_OS=Arabidopsis_thaliana_OX=3702_GN=CCR1_PE=1_SV=1	55.556	3.83E-42
TRINITY_19795_length_1647_cov_30.508872_g10193_i1	sp_Q9S9N9_CCR1_ARATH_Cinnamoyl-CoA_reductase_1_OS=Arabidopsis_thaliana_OX=3702_GN=CCR1_PE=1_SV=1	42.593	3.83E-42
TRINITY_20204_length_1628_cov_35.821681_g12723_i0	sp_Q7XPL2_HEM6_ORYSJ_Oxygen-dependent_coproporphyrinogen-III_oxidase,_chloroplastic_OS=Oryza_sativa_subsp._japonica_OX=39947_GN=CPX_PE=2_SV=2	95.152	0
TRINITY_20429_length_1619_cov_24.490323_g12855_i0	sp_P16397_SUBF_BACSU_Bacillopeptidase_F_OS=Bacillus_subtilis_(strain_168)_OX=224308_GN=bpr_PE=1_SV=2	35.349	9.25E-46
TRINITY_20602_length_1612_cov_21.245625_g12967_i0	sp_Q3SYZ4_SYDC_BOVIN_Aspartate--tRNA_ligase,_cytoplasmic_OS=Bos_taurus_OX=9913_GN=DARS_PE=2_SV=1	55.04	0
TRINITY_20620_length_1611_cov_24.781453_g11960_i1	sp_Q0J1C1_ACCO1_ORYSJ_1-aminocyclopropane-1-carboxylate_oxidase_1_OS=Oryza_sativa_subsp._japonica_OX=39947_GN=ACO1_PE=2_SV=1	85.172	2.01E-154
TRINITY_20620_length_1611_cov_24.781453_g11960_i1	sp_Q0J1C1_ACCO1_ORYSJ_1-aminocyclopropane-1-carboxylate_oxidase_1_OS=Oryza_sativa_subsp._japonica_OX=39947_GN=ACO1_PE=2_SV=1	71.429	2.14E-12
TRINITY_21197_length_1586_cov_38.420567_g13360_i0	sp_Q7FAS1_GLO3_ORYSJ_Peroxisomal_(S)-2-hydroxy-acid_oxidase_GLO3_OS=Oryza_sativa_subsp._japonica_OX=39947_GN=GLO3_PE=2_SV=1	91.553	0
TRINITY_21218_length_1585_cov_36.085092_g13373_i0	sp_Q850M0_KCO1_ORYSJ_Two_pore_potassium_channel_a_OS=Oryza_sativa_subsp._japonica_OX=39947_GN=TPKA_PE=1_SV=1	76.437	1.65E-177
TRINITY_21347_length_1580_cov_33.256784_g13457_i0	sp_Q8W3M4_Y4744_ARATH_Uncharacterized_protein_At4g06744_OS=Arabidopsis_thaliana_OX=3702_GN=At4g06744_PE=2_SV=1	44.315	3.37E-84
TRINITY_21652_length_1568_cov_26.823883_g13659_i0	sp_P82953_LECH_HORVV_Horcolin_OS=Hordeum_vulgare_subsp._vulgare_OX=112509_PE=1_SV=2	47.5	4.4E-16
TRINITY_21652_length_1568_cov_26.823883_g13659_i0	sp_P82953_LECH_HORVV_Horcolin_OS=Hordeum_vulgare_subsp._vulgare_OX=112509_PE=1_SV=2	43.939	3.43E-09
TRINITY_21697_length_1566_cov_32.713427_g13361_i1	sp_Q8LJJ9_STAD1_ORYSJ_Stearoyl-[acyl-carrier-protein]_9-desaturase_1,_chloroplastic_OS=Oryza_sativa_subsp._japonica_OX=39947_GN=Os01g0880800_PE=2_SV=1	94.18	5.63E-176
TRINITY_21697_length_1566_cov_32.713427_g13361_i1	sp_Q8LJJ9_STAD1_ORYSJ_Stearoyl-[acyl-carrier-protein]_9-desaturase_1,_chloroplastic_OS=Oryza_sativa_subsp._japonica_OX=39947_GN=Os01g0880800_PE=2_SV=1	95.973	5.63E-176
TRINITY_21725_length_1565_cov_29.952540_g13703_i0	sp_A7QEU4_PER5_VITVI_Peroxidase_5_OS=Vitis_vinifera_OX=29760_GN=GSVIVT00037159001_PE=1_SV=2	55.41	5.01E-84
TRINITY_22103_length_1548_cov_28.137255_g13972_i0	sp_Q84QK0_OPR1_ORYSJ_12-oxophytodienoate_reductase_1_OS=Oryza_sativa_subsp._japonica_OX=39947_GN=OPR1_PE=2_SV=1	87.43	0
TRINITY_22226_length_1542_cov_31.294637_g2198_i1	sp_P84516_PER1_SORBI_Cationic_peroxidase_SPC4_OS=Sorghum_bicolor_OX=4558_GN=Sb03g046810_PE=1_SV=2	44.375	3.28E-73
TRINITY_22764_length_1520_cov_31.175052_g14411_i0	sp_Q6K669_AMPL2_ORYSJ_Leucine_aminopeptidase_2,_chloroplastic_OS=Oryza_sativa_subsp._japonica_OX=39947_GN=Os02g0794700_PE=2_SV=1	80.645	1.62E-79
TRINITY_22764_length_1520_cov_31.175052_g14411_i0	sp_Q6K669_AMPL2_ORYSJ_Leucine_aminopeptidase_2,_chloroplastic_OS=Oryza_sativa_subsp._japonica_OX=39947_GN=Os02g0794700_PE=2_SV=1	91.589	5.78E-57
TRINITY_23350_length_1497_cov_25.478291_g14798_i0	sp_Q680C0_GDL62_ARATH_GDSL_esterase/lipase_At4g10955_OS=Arabidopsis_thaliana_OX=3702_GN=At4g10955_PE=2_SV=1	31.234	4.16E-40
TRINITY_23380_length_1496_cov_10.729502_g14820_i0	sp_Q9ZRJ4_TBA_CHLVU_Tubulin_alpha_chain_OS=Chlorella_vulgaris_OX=3077_GN=TUBA_PE=3_SV=1	90.046	0
TRINITY_23883_length_1478_cov_26.500355_g15145_i0	sp_P86939_EF1A2_TRYB2_Elongation_factor_1-alpha_2_OS=Trypanosoma_brucei_brucei_(strain_927/4_GUTat10.1)_OX=185431_GN=Tb10.70.5670_PE=1_SV=1	83.565	0
TRINITY_24771_length_1446_cov_32.411038_g15738_i0	sp_A7HWP7_EFTU_PARL1_Elongation_factor_Tu_OS=Parvibaculum_lavamentivorans_(strain_DS-1_/_DSM_13023_/_NCIMB_13966)_OX=402881_GN=tuf1_PE=3_SV=1	76.864	0
TRINITY_24941_length_1440_cov_27.471189_g15849_i0	sp_Q55AI5_SUCB1_DICDI_Succinate--CoA_ligase_[ADP-forming]_subunit_beta,_mitochondrial_OS=Dictyostelium_discoideum_OX=44689_GN=scsC_PE=3_SV=1	55.402	5.82E-168

TRINITY_25493_length_1420_cov_37.376018_g14764_i1	sp_Q0D673_P2C62_ORYSJ_Probable_protein_phosphatase_2C_62_OS=Oryza_sativa_subsp._japonica_OX=39947_GN=Os07g0507000_PE=2_SV=1	91.379	0
TRINITY_25507_length_1420_cov_28.694301_g5628_i2	sp_Q9SXJ6_CLPP3_ARATH_ATP-dependent_Clp_protease_proteolytic_subunit_3,_chloroplastic_OS=Arabidopsis_thaliana_OX=3702_GN=CLPP3_PE=1_SV=1	66.4	9.23E-106
TRINITY_25561_length_1418_cov_29.464789_g8577_i3	sp_Q39134_AAP3_ARATH_Amino_acid_permease_3_OS=Arabidopsis_thaliana_OX=3702_GN=AAP3_PE=1_SV=2	69.668	0
TRINITY_26921_length_1373_cov_23.520706_g17146_i0	sp_Q42993_CHI1_ORYSJ_Chitinase_1_OS=Oryza_sativa_subsp._japonica_OX=39947_GN=Cht1_PE=2_SV=1	77.663	6.84E-135
TRINITY_26973_length_1371_cov_29.294931_g17176_i0	sp_A7NY33_PER4_VITVI_Peroxidase_4_OS=Vitis_vinifera_OX=29760_GN=GSVIVT00023967001_PE=1_SV=1	45.646	1.6E-90
TRINITY_27003_length_1370_cov_23.229055_g17194_i0	sp_Q40082_XYLA_HORVU_Xylose_isomerase_OS=Hordeum_vulgare_OX=4513_GN=XYLA_PE=1_SV=1	89.965	0
TRINITY_27101_length_1366_cov_84.599846_g17263_i0	sp_Q3BAI2_YCX91_PHAAO_Uncharacterized_protein_ORF91_OS=Phalaenopsis_aphrodite_subsp._formosana_OX=308872_PE=4_SV=1	68.421	4.1E-16
TRINITY_28260_length_1330_cov_27.621729_g18041_i0	sp_P07871_THIKB_RAT_3-ketoacyl-CoA_thiolase_B,_peroxisomal_OS=Rattus_norvegicus_OX=10116_GN=Acaa1b_PE=1_SV=2	65.873	6.91E-176
TRINITY_28625_length_1318_cov_31.466773_g11111_i2	sp_Q6RYA0_SABP2_TOBAC_Salicylic_acid-binding_protein_2_OS=Nicotiana_tabacum_OX=4097_GN=SABP2_PE=1_SV=1	50.769	2.48E-27
TRINITY_28625_length_1318_cov_31.466773_g11111_i2	sp_Q6RYA0_SABP2_TOBAC_Salicylic_acid-binding_protein_2_OS=Nicotiana_tabacum_OX=4097_GN=SABP2_PE=1_SV=1	55.738	1.22E-24
TRINITY_28673_length_1317_cov_23.326923_g18323_i0	sp_Q9M593_HBL_ZEAMP_Non-symbiotic_hemoglobin_OS=Zea_mays_subsp._parviglumis_OX=76912_GN=HB_PE=1_SV=1	64.634	1.2E-61
TRINITY_28673_length_1317_cov_23.326923_g18323_i0	sp_Q9M593_HBL_ZEAMP_Non-symbiotic_hemoglobin_OS=Zea_mays_subsp._parviglumis_OX=76912_GN=HB_PE=1_SV=1	86.364	7.56E-17
TRINITY_28977_length_1308_cov_16.570621_g18529_i0	sp_P14656_GLN11_ORYSJ_Glutamine_synthetase_cytosolic_isozyme_1-1_OS=Oryza_sativa_subsp._japonica_OX=39947_GN=GLN1-1_PE=1_SV=1	65.706	1.01E-153
TRINITY_29071_length_1305_cov_29.024272_g18592_i0	sp_Q9FG34_PER54_ARATH_Peroxidase_54_OS=Arabidopsis_thaliana_OX=3702_GN=PER54_PE=2_SV=1	54.276	8.92E-95
TRINITY_29819_length_1281_cov_29.047030_g17545_i1	sp_Q6EPZ0_WRK62_ORYSJ_WRKY_transcription_factor_WRKY62_OS=Oryza_sativa_subsp._japonica_OX=39947_GN=WRKY62_PE=1_SV=1	52.041	9.31E-64
TRINITY_30409_length_1262_cov_28.735960_g19501_i0	sp_Q9ZV04_PER24_ARATH_Peroxidase_24_OS=Arabidopsis_thaliana_OX=3702_GN=PER24_PE=2_SV=1	51.623	1.02E-100
TRINITY_30609_length_1256_cov_27.197978_g19641_i0	sp_Q9M886_LBD41_ARATH_LOB_domain-containing_protein_41_OS=Arabidopsis_thaliana_OX=3702_GN=LBD41_PE=2_SV=1	69.828	9.68E-47
TRINITY_31408_length_1231_cov_37.751291_g20201_i0	sp_P55440_Y4FB_SINFN_Uncharacterized_protein_y4fB_OS=Sinorhizobium_fredii_(strain_NBRC_101917_/_NGR234)_OX=394_GN=NGR_a03790_PE=4_SV=1	28.085	7.25E-08
TRINITY_32198_length_1210_cov_30.662577_g20743_i0	sp_Q9AST3_SBH2_ARATH_Sphinganine_C4-monooxygenase_2_OS=Arabidopsis_thaliana_OX=3702_GN=SBH2_PE=1_SV=1	51.538	7.34E-86
TRINITY_32293_length_1208_cov_27.000878_g20802_i0	sp_Q9BV79_MECR_HUMAN_Enoyl-[acyl-carrier-protein]_reductase,_mitochondrial_OS=Homo_sapiens_OX=9606_GN=MECR_PE=1_SV=2	50.166	1.6E-80
TRINITY_32360_length_1206_cov_23.992084_g20854_i0	sp_Q9LXG9_PCO1_ARATH_Plant_cysteine_oxidase_1_OS=Arabidopsis_thaliana_OX=3702_GN=PCO1_PE=1_SV=1	48.503	1.18E-33
TRINITY_32679_length_1197_cov_27.954787_g21080_i0	sp_P29022_CHIA_MAIZE_Endochitinase_A_OS=Zea_mays_OX=4577_GN=CHIA_PE=1_SV=1	82.377	6.5E-129
TRINITY_33006_length_1188_cov_25.395889_g21116_i2	sp_P29022_CHIA_MAIZE_Endochitinase_A_OS=Zea_mays_OX=4577_GN=CHIA_PE=1_SV=1	82.377	1.57E-131
TRINITY_34763_length_1140_cov_25.963585_g22597_i0	sp_Q9LYP4_BAG3_ARATH_BAG_family_molecular_chaperone_regulator_3_OS=Arabidopsis_thaliana_OX=3702_GN=BAG3_PE=1_SV=1	49.038	1.18E-12
TRINITY_35020_length_1133_cov_26.391917_g22781_i0	sp_P47917_ZRP4_MAIZE_O-methyltransferase_ZRP4_OS=Zea_mays_OX=4577_GN=ZRP4_PE=2_SV=1	61.168	1.05E-111
TRINITY_35099_length_1131_cov_25.828625_g18363_i2	sp_Q6ER94_BAS1_ORYSJ_2-Cys_peroxiredoxin_BAS1,_chloroplastic_OS=Oryza_sativa_subsp._japonica_OX=39947_GN=BAS1_PE=1_SV=1	99.153	7.15E-79
TRINITY_35304_length_1125_cov_27.677083_g22970_i0	sp_Q9ARD5_LT02_HORVU_Low_temperature-induced_protein_lt101.2_OS=Hordeum_vulgare_OX=4513_GN=LT101.2_PE=2_SV=1	53.659	1.14E-15
TRINITY_35675_length_1115_cov_30.729446_g23239_i0	sp_Q6CQE5_TAR1_KLULA_Protein_TAR1_OS=Kluyveromyces_lactis_(strain_ATCC_8585_/_CBS_2359_/_DSM_70799_/_NBRC_1267_/_NRRL_Y-1140_/_WM37)_OX=284590_GN=TAR1-A_PE=4_SV=2	59.633	2.07E-27
TRINITY_35958_length_1108_cov_20.987488_g23445_i0	sp_Q6DRC4_EIF3G_DANRE_Eukaryotic_translation_initiation_factor_3_subunit_G_OS=Danio_rerio_OX=7955_GN=eif3g_PE=1_SV=1	40.728	1.02E-50
TRINITY_36487_length_1094_cov_29.361951_g10636_i1	sp_Q9SDN0_DNJ20_ARATH_Chaperone_protein_dnaJ_20,_chloroplastic_OS=Arabidopsis_thaliana_OX=3702_GN=ATJ20_PE=1_SV=2	50.4	8.65E-32
TRINITY_36620_length_1091_cov_31.309198_g23902_i0	sp_Q9LJ97_LEA31_ARATH_Late_embryogenesis_abundant_protein_31_OS=Arabidopsis_thaliana_OX=3702_GN=RAB28_PE=1_SV=1	50.495	4.12E-12
TRINITY_36657_length_1090_cov_26.702253_g1902_i2	sp_Q9FF99_AAP7_ARATH_Probable_amino_acid_permease_7_OS=Arabidopsis_thaliana_OX=3702_GN=AAP7_PE=2_SV=1	50.698	1.21E-69
TRINITY_37796_length_1062_cov_24.088620_g23008_i1	sp_P29421_IAAS_ORYSJ_Alpha-amylase/subtilisin_inhibitor_OS=Oryza_sativa_subsp._japonica_OX=39947_GN=RASI_PE=1_SV=2	55.758	5.51E-43
TRINITY_38067_length_1055_cov_41.061866_g24926_i0	sp_B3FHT4_SUCA_BLAHN_Succinate--CoA_ligase_[ADP-forming]_subunit_alpha_OS=Blastocystis_sp._subtype_1_(strain_ATCC_50177_/_NandII)_OX=478820_GN=SCSa_PE=1_SV=1	73.883	2.34E-130
TRINITY_38445_length_1046_cov_43.795292_g25200_i0	sp_Q1HQU2_RL5_AEDAE_60S_ribosomal_protein_L5_OS=Aedes_aegypti_OX=7159_GN=RpL5_PE=2_SV=1	58.844	7.13E-97
TRINITY_39052_length_1033_cov_27.057054_g25669_i0	sp_Q94A08_RFS2_ARATH_Probable_galactinol--sucrose_galactosyltransferase_2_OS=Arabidopsis_thaliana_OX=3702_GN=RFS2_PE=2_SV=2	60.086	1.15E-91

TRINITY_39052_length_1033_cov_27.057054_g25669_i0	sp_Q94A08_RFS2_ARATH_Probable_galactinol--sucrose_galactosyltransferase_2_OS=Arabidopsis_thaliana_OX=3702_GN=RFS2_PE=2_SV=2	69.048	7.98E-36
TRINITY_39095_length_1032_cov_14.651090_g25702_i0	sp_Q54BC8_PSB5_DICDI_Proteasome_subunit_beta_type-5_OS=Dictyostelium_discoideum_OX=44689_GN=psmB5_PE=1_SV=1	51.709	5.82E-74
TRINITY_42166_length_961_cov_30.558296_g28056_i0	sp_Q764D2_RS3A_LENED_40S_ribosomal_protein_S1_OS=Lentinula_edodes_OX=5353_GN=RPS1_PE=2_SV=1	67.089	8.26E-116
TRINITY_43352_length_938_cov_34.561565_g28965_i0	sp_Q9FWR5_GCA1_ARATH_Gamma_carbonic_anhydrase_1,_mitochondrial_OS=Arabidopsis_thaliana_OX=3702_GN=GAMMACA1_PE=1_SV=1	45.283	1.37E-66
TRINITY_43407_length_937_cov_31.114055_g29007_i0	sp_Q2W2J2_RL4_MAGSA_50S_ribosomal_protein_L4_OS=Magnetospirillum_magneticum_(strain_AMB-1_/_ATCC_700264)_OX=342108_GN=rplD_PE=3_SV=1	51.724	3.05E-64
TRINITY_43501_length_935_cov_35.773672_g29083_i0	sp_P29787_TRY5_AEDAE_Trypsin_5G1_OS=Aedes_aegypti_OX=7159_GN=AAEL013712_PE=2_SV=2	41.991	1.39E-38
TRINITY_43907_length_927_cov_29.083916_g29398_i0	sp_P0DKK7_RL7A2_ORYSJ_60S_ribosomal_protein_L7a-2_OS=Oryza_sativa_subsp._japonica_OX=39947_GN=RPL7A-2_PE=2_SV=1	60	7.5E-90
TRINITY_44830_length_908_cov_27.209774_g30129_i0	sp_P82162_RR10_SPIOL_30S_ribosomal_protein_S10_alpha,_chloroplastic_OS=Spinacia_oleracea_OX=3562_GN=RPS10_PE=1_SV=2	78.74	1.47E-68
TRINITY_45075_length_903_cov_25.934053_g30337_i0	sp_P24632_HSP22_MAIZE_17.8_kDa_class_II_heat_shock_protein_OS=Zea_mays_OX=4577_PE=2_SV=1	83.537	4.99E-78
TRINITY_45143_length_902_cov_21.211285_g30392_i0	sp_O65059_RS15_PICMA_40S_ribosomal_protein_S15_OS=Picea_mariana_OX=3335_GN=RPS15_PE=2_SV=1	72.043	8.52E-32
TRINITY_45143_length_902_cov_21.211285_g30392_i0	sp_O65059_RS15_PICMA_40S_ribosomal_protein_S15_OS=Picea_mariana_OX=3335_GN=RPS15_PE=2_SV=1	78.378	4.34E-13
TRINITY_45320_length_899_cov_14.833735_g30534_i0	sp_P13228_TRP_NEUCR_Tryptophan_synthase_OS=Neurospora_crassa_(strain_ATCC_24698_/_74-OR23-1A_/_CBS_708.71_/_DSM_1257_/_FGSC_987)_OX=367110_GN=trp-3_PE=1_SV=1	48.117	8.17E-71
TRINITY_46015_length_885_cov_35.610294_g31093_i0	sp_Q06398_GSTU6_ORYSJ_Probable_glutathione_S-transferase_GSTU6_OS=Oryza_sativa_subsp._japonica_OX=39947_GN=GSTU6_PE=2_SV=2	70	9.22E-46
TRINITY_46015_length_885_cov_35.610294_g31093_i0	sp_Q06398_GSTU6_ORYSJ_Probable_glutathione_S-transferase_GSTU6_OS=Oryza_sativa_subsp._japonica_OX=39947_GN=GSTU6_PE=2_SV=2	73.913	4.16E-14
TRINITY_46128_length_883_cov_38.030713_g31182_i0	sp_M0Y4P1_UGT13_HORVV_UDP-glucosyltransferase_UGT13248_OS=Hordeum_vulgare_subsp._vulgare_OX=112509_PE=2_SV=1	75.342	5.23E-146
TRINITY_47440_length_858_cov_35.711027_g32255_i0	sp_B6IRP3_RL1_RHOCS_50S_ribosomal_protein_L1_OS=Rhodospirillum_centenum_(strain_ATCC_51521_/_SW)_OX=414684_GN=rplA_PE=3_SV=1	67.662	5.71E-81
TRINITY_47723_length_853_cov_40.008929_g32493_i0	sp_Q9C6B3_GCA2_ARATH_Gamma_carbonic_anhydrase_2,_mitochondrial_OS=Arabidopsis_thaliana_OX=3702_GN=GAMMACA2_PE=1_SV=1	48.684	1.75E-71
TRINITY_47734_length_853_cov_25.942602_g32504_i0	sp_Q9FG13_CXE15_ARATH_Probable_carboxylesterase_15_OS=Arabidopsis_thaliana_OX=3702_GN=CXE15_PE=2_SV=1	45.089	1.3E-39
TRINITY_48039_length_847_cov_27.322622_g32756_i0	sp_P35793_PR13_HORVU_Pathogenesis-related_protein_PRB1-3_OS=Hordeum_vulgare_OX=4513_PE=2_SV=1	47.093	6.61E-38
TRINITY_48406_length_840_cov_24.446174_g33048_i0	sp_Q9FJ45_GDL83_ARATH_GDSL_esterase/lipase_At5g45910_OS=Arabidopsis_thaliana_OX=3702_GN=At5g45910_PE=2_SV=1	48.023	9.82E-53
TRINITY_48460_length_839_cov_22.741558_g33092_i0	sp_Q66XS7_NDUS4_GEKJA_NADH_dehydrogenase_[ubiquinone]_iron-sulfur_protein_4,_mitochondrial_OS=Gekko_japonicus_OX=146911_GN=NDUFS4_PE=2_SV=1	44.545	3.77E-23
TRINITY_48509_length_838_cov_29.072822_g33139_i0	sp_O04616_CUT1A_ARATH_Protein_CURVATURE_THYLAKOID_1A,_chloroplastic_OS=Arabidopsis_thaliana_OX=3702_GN=CURT1A_PE=1_SV=1	81.319	2.03E-47
TRINITY_48717_length_834_cov_30.366013_g33315_i0	sp_P29787_TRY5_AEDAE_Trypsin_5G1_OS=Aedes_aegypti_OX=7159_GN=AAEL013712_PE=2_SV=2	41.667	2.43E-45
TRINITY_48787_length_833_cov_20.123037_g33371_i0	sp_O64792_PIGP_ARATH_Phosphatidylinositol_N-acetylglucosaminyltransferase_subunit_P_OS=Arabidopsis_thaliana_OX=3702_GN=At1g61280_PE=2_SV=1	51.938	1.05E-33
TRINITY_48941_length_830_cov_29.049934_g33499_i0	sp_Q7Z0T3_TEMPT_APLCA_Temptin_OS=Aplysia_californica_OX=6500_PE=1_SV=1	46.392	1.7E-18
TRINITY_48977_length_830_cov_13.180026_g33530_i0	sp_Q9ZUT9_RS51_ARATH_40S_ribosomal_protein_S5-1_OS=Arabidopsis_thaliana_OX=3702_GN=RPS5A_PE=1_SV=1	73.232	3.07E-108
TRINITY_49153_length_826_cov_25.743725_g33679_i0	sp_F2VYU4_PIK1_ORYSJ_Disease_resistance_protein_Pik-1_OS=Oryza_sativa_subsp._japonica_OX=39947_GN=PIK-1_PE=1_SV=1	59.091	7.91E-14
TRINITY_49361_length_822_cov_34.589641_g33852_i0	sp_P37841_UCRI_SOLTU_Cytochrome_b-c1_complex_subunit_Rieske,_mitochondrial_OS=Solanum_tuberosum_OX=4113_GN=FES1_PE=1_SV=1	63.874	5.05E-83
TRINITY_49533_length_819_cov_28.770667_g33999_i0	sp_P14897_ELI9_HORVU_Low_molecular_mass_early_light-inducible_protein_HV90,_chloroplastic_OS=Hordeum_vulgare_OX=4513_PE=2_SV=1	79.091	4.72E-47
TRINITY_49663_length_817_cov_19.518717_g34113_i0	sp_Q6ETW4_PMEI8_ORYSJ_Pectinesterase_inhibitor_8_OS=Oryza_sativa_subsp._japonica_OX=39947_GN=PMEI8_PE=2_SV=1	54.225	9.42E-27
TRINITY_49762_length_815_cov_30.804290_g34196_i0	sp_P34091_RL6_MESCR_60S_ribosomal_protein_L6_OS=Mesembryanthemum_crystallinum_OX=3544_GN=RPL6_PE=2_SV=1	53.097	3.18E-55
TRINITY_50011_length_811_cov_22.212938_g23964_i1	sp_P51204_YCF54_PORPU_Uncharacterized_protein_ycf54_OS=Porphyra_purpurea_OX=2787_GN=ycf54_PE=3_SV=1	38.043	8.67E-12
TRINITY_50188_length_808_cov_9.849797_g34554_i0	sp_P32907_ATO2_YEAST_Ammonia_transport_outward_protein_2_OS=Saccharomyces_cerevisiae_(strain_ATCC_204508_/_S288c)_OX=559292_GN=ATO2_PE=1_SV=1	53.571	3.99E-41
TRINITY_50726_length_798_cov_23.219479_g35018_i0	sp_Q7XJE6_MCA1_ARATH_Metacaspase-1_OS=Arabidopsis_thaliana_OX=3702_GN=AMC1_PE=1_SV=1	38.824	0.00000677
TRINITY_50808_length_796_cov_38.947730_g35087_i0	sp_Q90Z10_RL13_DANRE_60S_ribosomal_protein_L13_OS=Danio_rerio_OX=7955_GN=rpl13_PE=2_SV=3	58.201	1.42E-55
TRINITY_50839_length_796_cov_8.536451_g35117_i0	sp_O35587_TMEDA_MESAU_Transmembrane_protein_Tmp21_OS=Mesocricetus_auratus_OX=10036_GN=TMED10_PE=1_SV=1	38.674	9.03E-32

TRINITY_53374_length_752_cov_21.819912_g37303_i0	sp_O80594_ERG28_ARATH_Ergosterol_biosynthetic_protein_28_OS=Arabidopsis_thaliana_OX=3702_GN=At1g10030_PE=2_SV=2	56.667	8.27E-37
TRINITY_53381_length_752_cov_13.234261_g37309_i0	sp_Q0BPZ0_RS6_GRABC_30S_ribosomal_protein_S6_OS=Granulibacter_bethesdensis_(strain_ATCC_BAA-1260_/_CGDNIH1)_OX=391165_GN=rpsF_PE=3_SV=1	34.653	4.39E-08
TRINITY_53585_length_748_cov_32.690722_g37487_i0	sp_P51119_GLNA2_VITVI_Glutamine_synthetase_cytosolic_isozyme_2_OS=Vitis_vinifera_OX=29760_GN=GS1-2_PE=2_SV=1	66.265	3.86E-106
TRINITY_53642_length_747_cov_28.740413_g37537_i0	sp_P49199_RS8_ORYSJ_40S_ribosomal_protein_S8_OS=Oryza_sativa_subsp._japonica_OX=39947_GN=RPS8_PE=2_SV=2	68.571	3.55E-92
TRINITY_54238_length_737_cov_40.712575_g38072_i0	sp_Q10MW3_PDC2_ORYSJ_Pyruvate_decarboxylase_2_OS=Oryza_sativa_subsp._japonica_OX=39947_GN=PDC2_PE=2_SV=1	95.492	9.65E-151
TRINITY_54953_length_726_cov_16.487062_g33650_i1	sp_P52009_CYP1_CAEEL_Peptidyl-prolyl_cis-trans_isomerase_1_OS=Caenorhabditis_elegans_OX=6239_GN=cyn-1_PE=2_SV=1	69.006	1.44E-72
TRINITY_55328_length_720_cov_22.572965_g39043_i0	sp_P46032_PTR2_ARATH_Protein_NRT1/_PTR_FAMILY_8.3_OS=Arabidopsis_thaliana_OX=3702_GN=NPF8.3_PE=1_SV=1	47.573	5.36E-21
TRINITY_55595_length_715_cov_25.557276_g39282_i0	sp_P83506_NLTP2_MAIZE_Probable_non-specific_lipid-transfer_protein_2_OS=Zea_mays_OX=4577_PE=1_SV=1	54.545	2.34E-15
TRINITY_56313_length_703_cov_26.293375_g39935_i0	sp_P79781_RS27A_CHICK_Ubiquitin-40S_ribosomal_protein_S27a_OS=Gallus_gallus_OX=9031_GN=RPS27A_PE=2_SV=3	86.184	3.29E-66
TRINITY_56438_length_701_cov_16.892405_g40047_i0	sp_O61231_RL10_DROME_60S_ribosomal_protein_L10_OS=Drosophila_melanogaster_OX=7227_GN=RpL10_PE=1_SV=1	81.221	1.3E-130
TRINITY_56538_length_699_cov_30.077778_g40144_i0	sp_P27807_H2B1_WHEAT_Histone_H2B.1_OS=Triticum_aestivum_OX=4565_PE=1_SV=3	100	2.03E-59
TRINITY_56598_length_698_cov_31.243243_g40202_i0	sp_Q1DI23_NACB_COCIM_Nascent_polypeptide-associated_complex_subunit_beta_OS=Coccidioides_immitis_(strain_RS)_OX=246410_GN=EGD1_PE=3_SV=1	63.014	2.84E-26
TRINITY_57372_length_686_cov_35.113452_g40926_i0	sp_Q90YW0_RL9_ICTPU_60S_ribosomal_protein_L9_OS=Ictalurus_punctatus_OX=7998_GN=rpl9_PE=2_SV=1	55.556	8.88E-66
TRINITY_59057_length_661_cov_26.596284_g42507_i0	sp_P69326_UBIQ_WHEAT_Ubiquitin_OS=Triticum_aestivum_OX=4565_PE=1_SV=2	43.421	0.000000297
TRINITY_60722_length_638_cov_31.063269_g44074_i0	sp_P49211_RL321_ARATH_60S_ribosomal_protein_L32-1_OS=Arabidopsis_thaliana_OX=3702_GN=RPL32A_PE=2_SV=2	70	3.18E-60
TRINITY_60938_length_635_cov_29.932862_g44277_i0	sp_P0A9L5_PPIC_ECOLI_Peptidyl-prolyl_cis-trans_isomerase_C_OS=Escherichia_coli_(strain_K12)_OX=83333_GN=ppiC_PE=1_SV=2	44.565	1.83E-15
TRINITY_62084_length_619_cov_34.760000_g45366_i0	sp_Q1XDE8_RK12_PYRYE_50S_ribosomal_protein_L12,_chloroplastic_OS=Pyropia_yezoensis_OX=2788_GN=rpl12_PE=3_SV=1	42.4	1.51E-18
TRINITY_62784_length_610_cov_31.695009_g46042_i0	sp_Q56K03_RL27A_BOVIN_60S_ribosomal_protein_L27a_OS=Bos_taurus_OX=9913_GN=RPL27A_PE=2_SV=3	72.519	6.15E-66
TRINITY_63246_length_604_cov_37.749533_g46488_i0	sp_Q9M069_E137_ARATH_Glucan_endo-1,3-beta-glucosidase_7_OS=Arabidopsis_thaliana_OX=3702_GN=At4g34480_PE=1_SV=2	47.727	5.87E-23
TRINITY_63246_length_604_cov_37.749533_g46488_i0	sp_Q9M069_E137_ARATH_Glucan_endo-1,3-beta-glucosidase_7_OS=Arabidopsis_thaliana_OX=3702_GN=At4g34480_PE=1_SV=2	38.06	3.13E-22
TRINITY_63737_length_598_cov_28.483932_g46956_i0	sp_Q71UH5_CALM_PYTSP_Calmodulin_OS=Pythium_splendens_OX=82926_PE=2_SV=1	100	3.8E-103
TRINITY_63903_length_596_cov_31.576850_g47117_i0	sp_P62302_RS13_SOYBN_40S_ribosomal_protein_S13_OS=Glycine_max_OX=3847_GN=RPS13_PE=2_SV=1	82.119	4.47E-90
TRINITY_64848_length_584_cov_28.415534_g48029_i0	sp_Q5WZM0_RL7_LEGPL_50S_ribosomal_protein_L7/L12_OS=Legionella_pneumophila_(strain_Lens)_OX=297245_GN=rplL_PE=3_SV=1	45.763	9.58E-20
TRINITY_65041_length_582_cov_18.855750_g48215_i0	sp_P00073_CYC_SPIOL_Cytochrome_c_OS=Spinacia_oleracea_OX=3562_PE=1_SV=1	74.51	1.42E-49
TRINITY_65268_length_579_cov_15.229412_g48434_i0	sp_Q3BAI2_YCX91_PHAAO_Uncharacterized_protein_ORF91_OS=Phalaenopsis_aphrodite_subsp._formosana_OX=308872_PE=4_SV=1	67.568	0.000000292
TRINITY_65405_length_577_cov_33.877953_g48570_i0	sp_P62752_RL23A_RAT_60S_ribosomal_protein_L23a_OS=Rattus_norvegicus_OX=10116_GN=Rpl23a_PE=2_SV=1	73.81	9.58E-58
TRINITY_65719_length_573_cov_36.797619_g48876_i0	sp_P0DJ19_RL27_TETTH_60S_ribosomal_protein_L27_OS=Tetrahymena_thermophila_OX=5911_GN=RPL27_PE=1_SV=1	52.113	5.16E-33
TRINITY_66381_length_565_cov_32.981855_g49518_i0	sp_Q3T0L7_RL28_BOVIN_60S_ribosomal_protein_L28_OS=Bos_taurus_OX=9913_GN=RPL28_PE=2_SV=3	36.923	3.52E-16
TRINITY_66387_length_565_cov_28.356855_g49524_i0	sp_P49249_IN22_MAIZE_IN2-2_protein_OS=Zea_mays_OX=4577_GN=IN2-2_PE=2_SV=1	86.905	4.37E-102
TRINITY_67030_length_558_cov_24.044990_g50152_i0	sp_Q39817_CALX_SOYBN_Calnexin_homolog_OS=Glycine_max_OX=3847_PE=2_SV=1	79.07	1.56E-70
TRINITY_67666_length_551_cov_29.454357_g50777_i0	sp_Q3BAI2_YCX91_PHAAO_Uncharacterized_protein_ORF91_OS=Phalaenopsis_aphrodite_subsp._formosana_OX=308872_PE=4_SV=1	68.421	0.000000162
TRINITY_68916_length_538_cov_23.624733_g52008_i0	sp_Q8TGM7_ART2_YEAST_Putative_uncharacterized_protein_ART2_OS=Saccharomyces_cerevisiae_(strain_ATCC_204508_/_S288c)_OX=559292_GN=ART2_PE=5_SV=1	76.786	9.33E-20
TRINITY_68995_length_537_cov_34.965812_g52085_i0	sp_Q9SJ36_RS172_ARATH_40S_ribosomal_protein_S17-2_OS=Arabidopsis_thaliana_OX=3702_GN=RPS17B_PE=2_SV=3	74.49	1.33E-40
TRINITY_70530_length_521_cov_35.092920_g53583_i0	sp_P40525_RL34B_YEAST_60S_ribosomal_protein_L34-B_OS=Saccharomyces_cerevisiae_(strain_ATCC_204508_/_S288c)_OX=559292_GN=RPL34B_PE=1_SV=1	70.652	1.28E-39
TRINITY_71551_length_511_cov_31.798643_g54592_i0	sp_Q8TGM7_ART2_YEAST_Putative_uncharacterized_protein_ART2_OS=Saccharomyces_cerevisiae_(strain_ATCC_204508_/_S288c)_OX=559292_GN=ART2_PE=5_SV=1	67.273	1.34E-18
TRINITY_77296_length_458_cov_24.843188_g60275_i0	sp_Q0WPW4_ACCO5_ARATH_1-aminocyclopropane-1-carboxylate_oxidase_5_OS=Arabidopsis_thaliana_OX=3702_GN=At1g77330_PE=2_SV=1	84	0.000004

TRINITY_79784_length_438_cov_28.607046_g62748_i0	sp_P19984_PROF2_ACACA_Profilin-2_OS=Acanthamoeba_castellanii_OX=5755_PE=1_SV=3	60.317	1.98E-52
TRINITY_81865_length_422_cov_35.141643_g64814_i0	sp_Q9VMU4_RL37A_DROME_60S_ribosomal_protein_L37a_OS=Drosophila_melanogaster_OX=7227_GN=RpL37A_PE=1_SV=3	65.217	2.28E-40
TRINITY_82391_length_418_cov_29.868195_g65338_i0	sp_Q9SIV0_SUR1_ARATH_S-alkyl-thiohydroximate_lyase_SUR1_OS=Arabidopsis_thaliana_OX=3702_GN=SUR1_PE=1_SV=1	43.396	3.85E-18
TRINITY_87290_length_380_cov_28.405145_g70222_i0	sp_P17654_AMY1_ORYSJ_Alpha-amylase_OS=Oryza_sativa_subsp._japonica_OX=39947_GN=AMY1.1_PE=1_SV=2	90.722	4.04E-58
TRINITY_88096_length_374_cov_21.773770_g71026_i0	sp_Q9M0Z6_MSRB3_ARATH_Peptide_methionine_sulfoxide_reductase_B3_OS=Arabidopsis_thaliana_OX=3702_GN=MSRB3_PE=2_SV=2	73.077	0.00000784
TRINITY_92840_length_339_cov_18.951852_g75769_i0	sp_Q0JCR9_MST1_ORYSJ_Sugar_transport_protein_MST1_OS=Oryza_sativa_subsp._japonica_OX=39947_GN=MST1_PE=2_SV=1	63.265	3.36E-10
TRINITY_BIN_1005_1	sp_P27937_AMY3B_ORYSJ_Alpha-amylase_isozyme_3B_OS=Oryza_sativa_subsp._japonica_OX=39947_GN=AMY1.6_PE=2_SV=1	84.921	1.64E-74
TRINITY_BIN_10205_2	sp_Q8LJJ9_STAD1_ORYSJ_Stearoyl-[acyl-carrier-protein]_9-desaturase_1,_chloroplastic_OS=Oryza_sativa_subsp._japonica_OX=39947_GN=Os01g0880800_PE=2_SV=1	71.976	4.32E-178
TRINITY_BIN_10690_3	sp_Q9ATL7_TIP31_MAIZE_Aquaporin_TIP3-1_OS=Zea_mays_OX=4577_GN=TIP3-1_PE=2_SV=1	91.626	2.39E-92
TRINITY_BIN_11269_1	sp_Q9LXG9_PCO1_ARATH_Plant_cysteine_oxidase_1_OS=Arabidopsis_thaliana_OX=3702_GN=PCO1_PE=1_SV=1	45.349	9.86E-14
TRINITY_BIN_11269_1	sp_Q9LXG9_PCO1_ARATH_Plant_cysteine_oxidase_1_OS=Arabidopsis_thaliana_OX=3702_GN=PCO1_PE=1_SV=1	58.14	1.15E-08
TRINITY_BIN_11547_3	sp_Q9C5S8_CRK5_ARATH_Cysteine-rich_receptor-like_protein_kinase_5_OS=Arabidopsis_thaliana_OX=3702_GN=CRK5_PE=1_SV=1	36.429	3.16E-21
TRINITY_BIN_11843_2	sp_Q10BU2_GL37_ORYSJ_Germin-like_protein_3-7_OS=Oryza_sativa_subsp._japonica_OX=39947_GN=GER7_PE=2_SV=1	77.612	1.17E-111
TRINITY_BIN_11939_5	sp_Q9LRB7_EL5_ORYSJ_E3_ubiquitin-protein_ligase_EL5_OS=Oryza_sativa_subsp._japonica_OX=39947_GN=EL5.1_PE=1_SV=1	43.796	8.83E-17
TRINITY_BIN_13016_9	sp_O49627_ISU1_ARATH_Iron-sulfur_cluster_assembly_protein_1_OS=Arabidopsis_thaliana_OX=3702_GN=ISU1_PE=2_SV=1	87.302	3.74E-79
TRINITY_BIN_13043_9	sp_Q9FNL8_PTR4_ARATH_Protein_NRT1/_PTR_FAMILY_5.3_OS=Arabidopsis_thaliana_OX=3702_GN=NPF5.3_PE=2_SV=1	40.625	2.76E-36
TRINITY_BIN_13823_3	sp_F4IK45_C70B2_ARATH_Cytochrome_P450_709B2_OS=Arabidopsis_thaliana_OX=3702_GN=CYP709B2_PE=2_SV=1	48.693	3.05E-95
TRINITY_BIN_13823_3	sp_F4IK45_C70B2_ARATH_Cytochrome_P450_709B2_OS=Arabidopsis_thaliana_OX=3702_GN=CYP709B2_PE=2_SV=1	44.776	0.000000881
TRINITY_BIN_14087_1	sp_Q9ZU75_UB12L_ARATH_Probable_NEDD8-conjugating_enzyme_Ubc12-like_OS=Arabidopsis_thaliana_OX=3702_GN=RCE2_PE=2_SV=1	75	3.06E-87
TRINITY_BIN_1443_3	sp_P31110_TLP_ORYSJ_Thaumatin-like_protein_OS=Oryza_sativa_subsp._japonica_OX=39947_GN=Os12g0628600_PE=1_SV=1	73.333	5.46E-73
TRINITY_BIN_14597_1	sp_P52837_F4ST_FLACH_Flavonol_4'-sulfotransferase_OS=Flaveria_chlorifolia_OX=4228_PE=1_SV=1	40.881	1.07E-72
TRINITY_BIN_15544_2	sp_Q9SK55_NAC42_ARATH_Transcription_factor_JUNGBRUNNEN_1_OS=Arabidopsis_thaliana_OX=3702_GN=JUB1_PE=1_SV=1	67.485	4.19E-72
TRINITY_BIN_15727_1	sp_Q9LMP1_WAK2_ARATH_Wall-associated_receptor_kinase_2_OS=Arabidopsis_thaliana_OX=3702_GN=WAK2_PE=1_SV=1	32.46	2.44E-90
TRINITY_BIN_1602_10	sp_Q9AWS0_Y1410_ORYSJ_AP2/ERF_and_B3_domain-containing_protein_Os01g0141000_OS=Oryza_sativa_subsp._japonica_OX=39947_GN=Os01g0141000_PE=2_SV=1	75.342	5.96E-15
TRINITY_BIN_1703_7	sp_Q57951_Y531_METJA_Universal_stress_protein_MJ0531_OS=Methanocaldococcus_jannaschii_(strain_ATCC_43067_/_DSM_2661_/_JAL-1_/_JCM_10045_/_NBRC_100440)_OX=243232_GN=MJ0531_PE=3_SV=1	35.165	2.53E-11
TRINITY_BIN_1709_4	sp_Q9SIB2_KCS12_ARATH_3-ketoacyl-CoA_synthase_12_OS=Arabidopsis_thaliana_OX=3702_GN=KCS12_PE=2_SV=1	53.333	2.7E-150
TRINITY_BIN_1717_7	sp_Q42376_LEA3_MAIZE_Late_embryogenesis_abundant_protein,_group_3_OS=Zea_mays_OX=4577_GN=MGL3_PE=2_SV=1	87.5	3.09E-22
TRINITY_BIN_1717_7	sp_Q42376_LEA3_MAIZE_Late_embryogenesis_abundant_protein,_group_3_OS=Zea_mays_OX=4577_GN=MGL3_PE=2_SV=1	64	7.23E-09
TRINITY_BIN_18101_1	sp_P93788_REMO_SOLTU_Remorin_OS=Solanum_tuberosum_OX=4113_PE=1_SV=1	60.584	1.12E-12
TRINITY_BIN_18968_1	sp_O14317_RLA6_SCHPO_60S_acidic_ribosomal_protein_P2-C_OS=Schizosaccharomyces_pombe_(strain_972_/_ATCC_24843)_OX=284812_GN=rpp203_PE=1_SV=2	60	1.87E-19
TRINITY_BIN_1943_8	sp_Q0D5R3_CRK6_ORYSJ_Cysteine-rich_receptor-like_protein_kinase_6_OS=Oryza_sativa_subsp._japonica_OX=39947_GN=CRK6_PE=1_SV=1	54.574	7.41E-108
TRINITY_BIN_198_5	sp_Q6K1S6_MYB30_ORYSJ_Transcription_factor_MYB30_OS=Oryza_sativa_subsp._japonica_OX=39947_GN=MYB30_PE=1_SV=1	91.209	3.84E-53
TRINITY_BIN_19978_1	sp_Q9FEQ8_PER2_MAIZE_Peroxidase_2_OS=Zea_mays_OX=4577_GN=PER2_PE=2_SV=1	48.43	5.85E-65
TRINITY_BIN_20189_1	sp_P81295_PRR3_JUNAS_Pathogenesis-related_protein_OS=Juniperus_ashei_OX=13101_PE=1_SV=1	60.294	3.5E-54
TRINITY_BIN_2103_8	sp_Q9FRX6_AS1_ANTMA_Aureusidin_synthase_OS=Antirrhinum_majus_OX=4151_GN=AS1_PE=1_SV=1	37.526	4.97E-86
TRINITY_BIN_22453_1	sp_Q8TGM7_ART2_YEAST_Putative_uncharacterized_protein_ART2_OS=Saccharomyces_cerevisiae_(strain_ATCC_204508_/_S288c)_OX=559292_GN=ART2_PE=5_SV=1	67.442	5.1E-11
TRINITY_BIN_2309_7	sp_Q0PGJ6_AKRC9_ARATH_NADPH-dependent_aldo-keto_reductase,_chloroplastic_OS=Arabidopsis_thaliana_OX=3702_GN=AKR4C9_PE=1_SV=1	67.254	7.45E-131
TRINITY_BIN_2455_3	sp_C0LGP4_Y3475_ARATH_Probable_LRR_receptor-like_serine/threonine-protein_kinase_At3g47570_OS=Arabidopsis_thaliana_OX=3702_GN=At3g47570_PE=2_SV=1	40.22	4.06E-133

TRINITY_BIN_2455_3	sp_C0LGP4_Y3475_ARATH_Probable_LRR_receptor-like_serine/threonine-protein_kinase_At3g47570_OS=Arabidopsis_thaliana_OX=3702_GN=At3g47570_PE=2_SV=1	37.367	1.66E-35
TRINITY_BIN_2455_3	sp_C0LGP4_Y3475_ARATH_Probable_LRR_receptor-like_serine/threonine-protein_kinase_At3g47570_OS=Arabidopsis_thaliana_OX=3702_GN=At3g47570_PE=2_SV=1	35.039	2.9E-26
TRINITY_BIN_2455_3	sp_C0LGP4_Y3475_ARATH_Probable_LRR_receptor-like_serine/threonine-protein_kinase_At3g47570_OS=Arabidopsis_thaliana_OX=3702_GN=At3g47570_PE=2_SV=1	38.532	9.69E-23
TRINITY_BIN_2455_3	sp_C0LGP4_Y3475_ARATH_Probable_LRR_receptor-like_serine/threonine-protein_kinase_At3g47570_OS=Arabidopsis_thaliana_OX=3702_GN=At3g47570_PE=2_SV=1	36.207	3.1E-22
TRINITY_BIN_2455_3	sp_C0LGP4_Y3475_ARATH_Probable_LRR_receptor-like_serine/threonine-protein_kinase_At3g47570_OS=Arabidopsis_thaliana_OX=3702_GN=At3g47570_PE=2_SV=1	34.853	1.65E-21
TRINITY_BIN_2455_3	sp_C0LGP4_Y3475_ARATH_Probable_LRR_receptor-like_serine/threonine-protein_kinase_At3g47570_OS=Arabidopsis_thaliana_OX=3702_GN=At3g47570_PE=2_SV=1	29.487	8.71E-20
TRINITY_BIN_2455_3	sp_C0LGP4_Y3475_ARATH_Probable_LRR_receptor-like_serine/threonine-protein_kinase_At3g47570_OS=Arabidopsis_thaliana_OX=3702_GN=At3g47570_PE=2_SV=1	33.333	1.44E-19
TRINITY_BIN_2455_3	sp_C0LGP4_Y3475_ARATH_Probable_LRR_receptor-like_serine/threonine-protein_kinase_At3g47570_OS=Arabidopsis_thaliana_OX=3702_GN=At3g47570_PE=2_SV=1	34.375	1.67E-18
TRINITY_BIN_2455_3	sp_C0LGP4_Y3475_ARATH_Probable_LRR_receptor-like_serine/threonine-protein_kinase_At3g47570_OS=Arabidopsis_thaliana_OX=3702_GN=At3g47570_PE=2_SV=1	31.071	5.96E-18
TRINITY_BIN_2455_3	sp_C0LGP4_Y3475_ARATH_Probable_LRR_receptor-like_serine/threonine-protein_kinase_At3g47570_OS=Arabidopsis_thaliana_OX=3702_GN=At3g47570_PE=2_SV=1	34.756	1.51E-08
TRINITY_BIN_2562_1	sp_Q9SMI3_RS12_CYAPA_40S_ribosomal_protein_S12_OS=Cyanophora_paradoxa_OX=2762_GN=RPS12_PE=2_SV=1	70.085	2.95E-53
TRINITY_BIN_260_7	sp_Q9FG34_PER54_ARATH_Peroxidase_54_OS=Arabidopsis_thaliana_OX=3702_GN=PER54_PE=2_SV=1	48.701	4.5E-86
TRINITY_BIN_2817_1	sp_Q7GCM7_XIP1_ORYSJ_Xylanase_inhibitor_protein_1_OS=Oryza_sativa_subsp._japonica_OX=39947_GN=RIXI_PE=1_SV=1	63.604	8.89E-88
TRINITY_BIN_2956_3	sp_Q651X6_CSLE6_ORYSJ_Cellulose_synthase-like_protein_E6_OS=Oryza_sativa_subsp._japonica_OX=39947_GN=CSLE6_PE=2_SV=1	74.828	0
TRINITY_BIN_3188_1	sp_O59950_RS4_YARLI_40S_ribosomal_protein_S4_OS=Yarrowia_lipolytica_(strain_CLIB_122_/_E_150)_OX=284591_GN=RPS4_PE=2_SV=1	68.605	7.96E-129
TRINITY_BIN_3224_4	sp_Q9SF40_RL4A_ARATH_60S_ribosomal_protein_L4-1_OS=Arabidopsis_thaliana_OX=3702_GN=RPL4A_PE=1_SV=1	63.772	2.27E-133
TRINITY_BIN_3238_3	sp_Q9SVG5_BBE18_ARATH_Berberine_bridge_enzyme-like_18_OS=Arabidopsis_thaliana_OX=3702_GN=At4g20820_PE=3_SV=1	42.5	1.03E-85
TRINITY_BIN_3238_3	sp_Q9SVG5_BBE18_ARATH_Berberine_bridge_enzyme-like_18_OS=Arabidopsis_thaliana_OX=3702_GN=At4g20820_PE=3_SV=1	46.099	1.03E-85
TRINITY_BIN_3254_3	sp_Q8W3M4_Y4744_ARATH_Uncharacterized_protein_At4g06744_OS=Arabidopsis_thaliana_OX=3702_GN=At4g06744_PE=2_SV=1	42.899	1.97E-67
TRINITY_BIN_3323_2	sp_Q9LJ97_LEA31_ARATH_Late_embryogenesis_abundant_protein_31_OS=Arabidopsis_thaliana_OX=3702_GN=RAB28_PE=1_SV=1	44.554	0.000000169
TRINITY_BIN_3347_9	sp_Q2QY07_CCDA2_ORYSJ_Cytochrome_c-type_biogenesis_ccda-like_chloroplastic_protein_2_OS=Oryza_sativa_subsp._japonica_OX=39947_GN=CCDA2_PE=3_SV=1	83.516	2.53E-75
TRINITY_BIN_3347_9	sp_Q2QY07_CCDA2_ORYSJ_Cytochrome_c-type_biogenesis_ccda-like_chloroplastic_protein_2_OS=Oryza_sativa_subsp._japonica_OX=39947_GN=CCDA2_PE=3_SV=1	93.269	7.24E-58
TRINITY_BIN_3370_10	sp_Q8LGG8_USPAL_ARATH_Universal_stress_protein_A-like_protein_OS=Arabidopsis_thaliana_OX=3702_GN=At3g01520_PE=1_SV=2	44.444	1.02E-11
TRINITY_BIN_3400_2	sp_M0Y4P1_UGT13_HORVV_UDP-glucosyltransferase_UGT13248_OS=Hordeum_vulgare_subsp._vulgare_OX=112509_PE=2_SV=1	64.675	3.54E-159
TRINITY_BIN_3639_11	sp_Q9M0G7_MIK1_ARATH_MDIS1-interacting_receptor_like_kinase_1_OS=Arabidopsis_thaliana_OX=3702_GN=MIK1_PE=1_SV=1	38.727	1.03E-139
TRINITY_BIN_3639_11	sp_Q9M0G7_MIK1_ARATH_MDIS1-interacting_receptor_like_kinase_1_OS=Arabidopsis_thaliana_OX=3702_GN=MIK1_PE=1_SV=1	31.139	2.41E-30
TRINITY_BIN_3639_11	sp_Q9M0G7_MIK1_ARATH_MDIS1-interacting_receptor_like_kinase_1_OS=Arabidopsis_thaliana_OX=3702_GN=MIK1_PE=1_SV=1	35.507	8.37E-15
TRINITY_BIN_3639_11	sp_Q9M0G7_MIK1_ARATH_MDIS1-interacting_receptor_like_kinase_1_OS=Arabidopsis_thaliana_OX=3702_GN=MIK1_PE=1_SV=1	28.333	8.07E-10
TRINITY_BIN_3704_2	sp_O80470_GDL38_ARATH_GDSL_esterase/lipase_At2g23540_OS=Arabidopsis_thaliana_OX=3702_GN=At2g23540_PE=2_SV=1	69.706	3.37E-172
TRINITY_BIN_3907_5	sp_Q9SE93_PNAE_RAUSE_Polyneuridine-aldehyde_esterase_OS=Rauvolfia_serpentina_OX=4060_GN=PNAE_PE=1_SV=1	35.315	1.72E-29
TRINITY_BIN_3951_27	sp_Q9T0G0_HSD5_ARATH_11-beta-hydroxysteroid_dehydrogenase-like_5_OS=Arabidopsis_thaliana_OX=3702_GN=HSD5_PE=2_SV=1	67.093	2.99E-154
TRINITY_BIN_3983_4	sp_Q9SV68_QORH_ARATH_Chloroplast_envelope_quinone_oxidoreductase_homolog_OS=Arabidopsis_thaliana_OX=3702_GN=CEQORH_PE=1_SV=1	66.96	1.75E-93
TRINITY_BIN_3983_4	sp_Q9SV68_QORH_ARATH_Chloroplast_envelope_quinone_oxidoreductase_homolog_OS=Arabidopsis_thaliana_OX=3702_GN=CEQORH_PE=1_SV=1	71.667	2.08E-20
TRINITY_BIN_4110_9	sp_Q39817_CALX_SOYBN_Calnexin_homolog_OS=Glycine_max_OX=3847_PE=2_SV=1	82.086	0
TRINITY_BIN_42_20	sp_Q9FJH0_RAA1F_ARATH_Ras-related_protein_RABA1f_OS=Arabidopsis_thaliana_OX=3702_GN=RABA1F_PE=2_SV=1	77.465	7.1E-69
TRINITY_BIN_42_20	sp_Q9FJH0_RAA1F_ARATH_Ras-related_protein_RABA1f_OS=Arabidopsis_thaliana_OX=3702_GN=RABA1F_PE=2_SV=1	72.857	1.01E-25
TRINITY_BIN_4432_1	sp_O06431_DNAJ_NITEU_Chaperone_protein_DnaJ_OS=Nitrosomonas_europaea_(strain_ATCC_19718_/_CIP_103999_/_KCTC_2705_/_NBRC_14298)_OX=228410_GN=dnaJ_PE=3_SV=2	48.438	2.89E-08

TRINITY_BIN_4564_7	sp_O64624_PP163_ARATH_Pentatricopeptide_repeat-containing_protein_At2g18940,_chloroplastic_OS=Arabidopsis_thaliana_OX=3702_GN=At2g18940_PE=2_SV=1	57.804	0
TRINITY_BIN_4564_7	sp_O64624_PP163_ARATH_Pentatricopeptide_repeat-containing_protein_At2g18940,_chloroplastic_OS=Arabidopsis_thaliana_OX=3702_GN=At2g18940_PE=2_SV=1	23.864	7.47E-28
TRINITY_BIN_478_8	sp_Q9FJ45_GDL83_ARATH_GDSL_esterase/lipase_At5g45910_OS=Arabidopsis_thaliana_OX=3702_GN=At5g45910_PE=2_SV=1	43.75	4.69E-40
TRINITY_BIN_4886_7	sp_Q93V61_LCAT3_ARATH_Phospholipase_A(1)_LCAT3_OS=Arabidopsis_thaliana_OX=3702_GN=LCAT3_PE=1_SV=1	67.123	1.14E-72
TRINITY_BIN_4886_7	sp_Q93V61_LCAT3_ARATH_Phospholipase_A(1)_LCAT3_OS=Arabidopsis_thaliana_OX=3702_GN=LCAT3_PE=1_SV=1	83.721	1.14E-72
TRINITY_BIN_4886_7	sp_Q93V61_LCAT3_ARATH_Phospholipase_A(1)_LCAT3_OS=Arabidopsis_thaliana_OX=3702_GN=LCAT3_PE=1_SV=1	54.945	7.19E-20
TRINITY_BIN_4886_7	sp_Q93V61_LCAT3_ARATH_Phospholipase_A(1)_LCAT3_OS=Arabidopsis_thaliana_OX=3702_GN=LCAT3_PE=1_SV=1	66.265	2.89E-18
TRINITY_BIN_4886_7	sp_Q93V61_LCAT3_ARATH_Phospholipase_A(1)_LCAT3_OS=Arabidopsis_thaliana_OX=3702_GN=LCAT3_PE=1_SV=1	58.824	2.54E-11
TRINITY_BIN_4975_1	sp_Q0CGL5_NACB_ASPTN_Nascent_polypeptide-associated_complex_subunit_beta_OS=Aspergillus_terreus_(strain_NIH_2624_/_FGSC_A1156)_OX=341663_GN=egd1_PE=3_SV=1	63.014	8.66E-28
TRINITY_BIN_5_1	sp_Q6Z401_MST6_ORYSJ_Sugar_transport_protein_MST6_OS=Oryza_sativa_subsp._japonica_OX=39947_GN=MST6_PE=1_SV=1	82.31	0
TRINITY_BIN_5115_6	sp_Q9XGP7_OMT15_ORYSJ_Tricin_synthase_1_OS=Oryza_sativa_subsp._japonica_OX=39947_GN=ROMT-15_PE=1_SV=1	78.365	1.68E-105
TRINITY_BIN_5211_2	sp_Q2L6L1_CNPY1_DANRE_Protein_canopy-1_OS=Danio_rerio_OX=7955_GN=cnpy1_PE=1_SV=1	28.387	1.76E-11
TRINITY_BIN_5680_1	sp_P29787_TRY5_AEDAE_Trypsin_5G1_OS=Aedes_aegypti_OX=7159_GN=AAEL013712_PE=2_SV=2	41.667	4.39E-45
TRINITY_BIN_5696_1	sp_Q9LW57_PAP6_ARATH_Plastid-lipid-associated_protein_6,_chloroplastic_OS=Arabidopsis_thaliana_OX=3702_GN=PAP6_PE=1_SV=1	60.09	2.97E-80
TRINITY_BIN_5888_4	sp_P93788_REMO_SOLTU_Remorin_OS=Solanum_tuberosum_OX=4113_PE=1_SV=1	63.514	4.27E-23
TRINITY_BIN_6019_2	sp_P19954_PRSP1_SPIOL_Ribosome-binding_factor_PSRP1,_chloroplastic_OS=Spinacia_oleracea_OX=3562_GN=PSRP1_PE=1_SV=2	35.484	4.08E-30
TRINITY_BIN_6091_7	sp_Q6K498_HOX4_ORYSJ_Homeobox-leucine_zipper_protein_HOX4_OS=Oryza_sativa_subsp._japonica_OX=39947_GN=HOX4_PE=1_SV=1	86.131	7.94E-46
TRINITY_BIN_6485_4	sp_P29787_TRY5_AEDAE_Trypsin_5G1_OS=Aedes_aegypti_OX=7159_GN=AAEL013712_PE=2_SV=2	41.558	9.87E-38
TRINITY_BIN_7_6	sp_Q8TGM6_TAR1_YEAST_Protein_TAR1_OS=Saccharomyces_cerevisiae_(strain_ATCC_204508_/_S288c)_OX=559292_GN=TAR1_PE=2_SV=1	54.651	4.65E-21
TRINITY_BIN_7_6	sp_Q8TGM6_TAR1_YEAST_Protein_TAR1_OS=Saccharomyces_cerevisiae_(strain_ATCC_204508_/_S288c)_OX=559292_GN=TAR1_PE=2_SV=1	58.974	4.65E-21
TRINITY_BIN_727_2	sp_Q0J1C1_ACCO1_ORYSJ_1-aminocyclopropane-1-carboxylate_oxidase_1_OS=Oryza_sativa_subsp._japonica_OX=39947_GN=ACO1_PE=2_SV=1	87.5	5E-118
TRINITY_BIN_750_1	sp_Q9ZPE7_EXO_ARATH_Protein_EXORDIUM_OS=Arabidopsis_thaliana_OX=3702_GN=EXO_PE=2_SV=1	68.817	9.48E-40
TRINITY_BIN_7560_1	sp_Q9C912_R35A3_ARATH_60S_ribosomal_protein_L35a-3_OS=Arabidopsis_thaliana_OX=3702_GN=RPL35AC_PE=3_SV=1	52.83	3.53E-39
TRINITY_BIN_7705_7	sp_Q9LMP1_WAK2_ARATH_Wall-associated_receptor_kinase_2_OS=Arabidopsis_thaliana_OX=3702_GN=WAK2_PE=1_SV=1	37.701	2.97E-160
TRINITY_BIN_8105_8	sp_Q9SDN0_DNJ20_ARATH_Chaperone_protein_dnaJ_20,_chloroplastic_OS=Arabidopsis_thaliana_OX=3702_GN=ATJ20_PE=1_SV=2	55.952	1.03E-21
TRINITY_BIN_968_2	sp_Q9FR99_ACCO_MUSAC_1-aminocyclopropane-1-carboxylate_oxidase_OS=Musa_acuminata_OX=4641_GN=MAO1B_PE=3_SV=1	72.947	4.47E-109
TRINITY_BIN_97_5	sp_P32138_SQASE_ECOLI_Sulfoquinovosidase_OS=Escherichia_coli_(strain_K12)_OX=83333_GN=yihQ_PE=1_SV=3	36.333	2.88E-99
TRINITY_BIN_9764_3	sp_Q7EZ57_HS188_ORYSJ_18.8_kDa_class_V_heat_shock_protein_OS=Oryza_sativa_subsp._japonica_OX=39947_GN=HSP18.8_PE=2_SV=1	59.712	1.11E-37
TRINITY_BIN_9927_1	sp_Q9SJZ2_PER17_ARATH_Peroxidase_17_OS=Arabidopsis_thaliana_OX=3702_GN=PER17_PE=2_SV=1	72.391	9.74E-148
TRINITY_BIN_9932_5	sp_Q9LT96_Y5977_ARATH_Probable_leucine-rich_repeat_receptor-like_protein_kinase_At5g49770_OS=Arabidopsis_thaliana_OX=3702_GN=At5g49770_PE=1_SV=1	44.801	0
TRINITY_DN11840_c0_g1_i1	sp_Q9SZY3_SBT38_ARATH_Subtilisin-like_protease_SBT3.8_OS=Arabidopsis_thaliana_OX=3702_GN=SBT3.8_PE=3_SV=1	46.711	5.84E-41
TRINITY_DN22137_c1_g1_i1	sp_Q9SZY3_SBT38_ARATH_Subtilisin-like_protease_SBT3.8_OS=Arabidopsis_thaliana_OX=3702_GN=SBT3.8_PE=3_SV=1	43.421	2.41E-138
TRINITY_DN22120_c2_g3_i9	sp_Q9LMN7_WAK5_ARATH_Wall-associated_receptor_kinase_5_OS=Arabidopsis_thaliana_OX=3702_GN=WAK5_PE=2_SV=1	55.516	2.29E-107
TRINITY_DN22120_c2_g3_i9	sp_Q9LMN7_WAK5_ARATH_Wall-associated_receptor_kinase_5_OS=Arabidopsis_thaliana_OX=3702_GN=WAK5_PE=2_SV=1	31.25	2.29E-107
TRINITY_DN22133_c0_g2_i9	sp_Q10SC8_CIPK9_ORYSJ_CBL-interacting_protein_kinase_9_OS=Oryza_sativa_subsp._japonica_OX=39947_GN=CIPK9_PE=2_SV=1	89.37	8.48E-167
TRINITY_DN16117_c5_g2_i4	sp_Q0DBL6_HFC2B_ORYSJ_Heat_stress_transcription_factor_C-2b_OS=Oryza_sativa_subsp._japonica_OX=39947_GN=HSFC2B_PE=3_SV=1	78.723	2.97E-106
TRINITY_DN15174_c1_g1_i1	sp_Q9W5R8_RL5_DROME_60S_ribosomal_protein_L5_OS=Drosophila_melanogaster_OX=7227_GN=RpL5_PE=1_SV=2	63.927	1.54E-87
TRINITY_DN16475_c0_g3_i3	sp_P14133_ASO_CUCSA_L-ascorbate_oxidase_OS=Cucumis_sativus_OX=3659_PE=1_SV=1	48.244	1.38E-130
TRINITY_DN16475_c0_g3_i4	sp_P14133_ASO_CUCSA_L-ascorbate_oxidase_OS=Cucumis_sativus_OX=3659_PE=1_SV=1	49.389	6.96E-129

TRINITY_DN16486_c0_g3_i3	sp_F2VYU4_PIK1_ORYSJ_Disease_resistance_protein_Pik-1_OS=Oryza_sativa_subsp._japonica_OX=39947_GN=PIK-1_PE=1_SV=1	63.768	1.54E-19
TRINITY_DN16486_c0_g3_i4	sp_F2VYU4_PIK1_ORYSJ_Disease_resistance_protein_Pik-1_OS=Oryza_sativa_subsp._japonica_OX=39947_GN=PIK-1_PE=1_SV=1	63.768	5.7E-19
TRINITY_DN20731_c0_g1_i1	sp_Q9FR99_ACCO_MUSAC_1-aminocyclopropane-1-carboxylate_oxidase_OS=Musa_acuminata_OX=4641_GN=MAO1B_PE=3_SV=1	69.663	7.72E-89
TRINITY_DN20778_c0_g1_i2	sp_Q8GSI0_TI11A_ORYSJ_Protein_TIFY_11a_OS=Oryza_sativa_subsp._japonica_OX=39947_GN=TIFY11A_PE=1_SV=1	47.826	7.52E-16
TRINITY_DN17417_c1_g3_i2	sp_Q9XGP7_OMT15_ORYSJ_Tricin_synthase_1_OS=Oryza_sativa_subsp._japonica_OX=39947_GN=ROMT-15_PE=1_SV=1	78.333	1.36E-130
TRINITY_DN14127_c1_g2_i2	sp_Q8GYX8_DNJ10_ARATH_Chaperone_protein_dnaJ_10_OS=Arabidopsis_thaliana_OX=3702_GN=ATJ10_PE=2_SV=2	70.541	2.24E-169
TRINITY_DN14117_c0_g10_i1	sp_P29022_CHIA_MAIZE_Endochitinase_A_OS=Zea_mays_OX=4577_GN=CHIA_PE=1_SV=1	67.677	3.41E-23
TRINITY_DN14161_c0_g2_i3	sp_Q7GCM7_XIP1_ORYSJ_Xylanase_inhibitor_protein_1_OS=Oryza_sativa_subsp._japonica_OX=39947_GN=RIXI_PE=1_SV=1	59.273	1.46E-110
TRINITY_DN22340_c0_g1_i6	sp_Q9FXQ3_CDPKD_ORYSJ_Calcium-dependent_protein_kinase_13_OS=Oryza_sativa_subsp._japonica_OX=39947_GN=CPK13_PE=2_SV=1	98.315	2.74E-121
TRINITY_DN22344_c2_g2_i18	sp_O81024_EKI_ARATH_Probable_ethanolamine_kinase_OS=Arabidopsis_thaliana_OX=3702_GN=EMB1187_PE=2_SV=1	72.903	1.87E-74
TRINITY_DN17265_c0_g3_i1	sp_Q9LPV8_ERF1Y_ARATH_Eukaryotic_peptide_chain_release_factor_subunit_1-2_OS=Arabidopsis_thaliana_OX=3702_GN=ERF1-2_PE=1_SV=1	80.184	0
TRINITY_DN17266_c1_g2_i4	sp_P18123_CATA3_MAIZE_Catalase_isozyme_3_OS=Zea_mays_OX=4577_GN=CAT3_PE=2_SV=2	91.968	0
TRINITY_DN17266_c1_g2_i7	sp_P18123_CATA3_MAIZE_Catalase_isozyme_3_OS=Zea_mays_OX=4577_GN=CAT3_PE=2_SV=2	92.073	0
TRINITY_DN21347_c0_g3_i4	sp_Q9SP22_CALR_MAIZE_Calreticulin_OS=Zea_mays_OX=4577_GN=CRT_PE=2_SV=1	94.93	0
TRINITY_DN21347_c0_g3_i11	sp_Q9SLY8_CALR_ORYSJ_Calreticulin_OS=Oryza_sativa_subsp._japonica_OX=39947_GN=Os07g0246200_PE=1_SV=2	92.628	0
TRINITY_DN15946_c1_g2_i4	sp_Q8VZ80_PLT5_ARATH_Polyol_transporter_5_OS=Arabidopsis_thaliana_OX=3702_GN=PLT5_PE=1_SV=2	43.501	7.33E-64
TRINITY_DN15968_c0_g1_i8	sp_Q9SV68_QORH_ARATH_Chloroplast_envelope_quinone_oxidoreductase_homolog_OS=Arabidopsis_thaliana_OX=3702_GN=CEQORH_PE=1_SV=1	66.529	1.78E-98
TRINITY_DN15968_c0_g1_i8	sp_Q9SV68_QORH_ARATH_Chloroplast_envelope_quinone_oxidoreductase_homolog_OS=Arabidopsis_thaliana_OX=3702_GN=CEQORH_PE=1_SV=1	59.016	1.29E-14
TRINITY_DN20051_c0_g1_i1	sp_Q84R49_GUN10_ORYSJ_Endoglucanase_10_OS=Oryza_sativa_subsp._japonica_OX=39947_GN=GLU2_PE=2_SV=1	92.893	0
TRINITY_DN21458_c1_g1_i1	sp_Q9ZT07_RKS1_ARATH_G-type_lectin_S-receptor-like_serine/threonine-protein_kinase_RKS1_OS=Arabidopsis_thaliana_OX=3702_GN=RKS1_PE=3_SV=3	44.337	0
TRINITY_DN21484_c2_g2_i8	sp_P04783_CB25_PETSP_Chlorophyll_a-b_binding_protein_91R,_chloroplastic_OS=Petunia_sp._OX=4104_GN=CAB91R_PE=3_SV=1	93.137	3.17E-83
TRINITY_DN21484_c2_g2_i8	sp_P04783_CB25_PETSP_Chlorophyll_a-b_binding_protein_91R,_chloroplastic_OS=Petunia_sp._OX=4104_GN=CAB91R_PE=3_SV=1	98.413	3.17E-83
TRINITY_DN19996_c0_g1_i2	sp_P32138_SQASE_ECOLI_Sulfoquinovosidase_OS=Escherichia_coli_(strain_K12)_OX=83333_GN=yihQ_PE=1_SV=3	36.425	1.72E-101
TRINITY_DN19905_c0_g3_i2	sp_Q8L836_MA657_ARATH_65-kDa_microtubule-associated_protein_7_OS=Arabidopsis_thaliana_OX=3702_GN=MAP65-7_PE=2_SV=1	56.342	2.36E-112
TRINITY_DN19913_c0_g1_i7	sp_Q9SSG3_HIPL1_ARATH_HIPL1_protein_OS=Arabidopsis_thaliana_OX=3702_GN=HIPL1_PE=3_SV=2	60.062	0
TRINITY_DN18383_c0_g1_i16	sp_Q41328_PTI1_SOLLC_Pto-interacting_protein_1_OS=Solanum_lycopersicum_OX=4081_GN=PTI1_PE=1_SV=2	81.089	0
TRINITY_DN18322_c0_g3_i1	sp_Q1PEY5_HIBC2_ARATH_Probable_3-hydroxyisobutyryl-CoA_hydrolase_2_OS=Arabidopsis_thaliana_OX=3702_GN=At2g30650_PE=2_SV=1	70.27	4.79E-14
TRINITY_DN18322_c0_g3_i1	sp_Q1PEY5_HIBC2_ARATH_Probable_3-hydroxyisobutyryl-CoA_hydrolase_2_OS=Arabidopsis_thaliana_OX=3702_GN=At2g30650_PE=2_SV=1	42.105	4.79E-14
TRINITY_DN9994_c0_g1_i3	sp_Q9T043_RL142_ARATH_60S_ribosomal_protein_L14-2_OS=Arabidopsis_thaliana_OX=3702_GN=RPL14B_PE=1_SV=1	47.458	1.38E-31
TRINITY_DN18492_c0_g1_i5	sp_Q5XLY0_ANR_GINBI_Putative_anthocyanidin_reductase_OS=Ginkgo_biloba_OX=3311_PE=2_SV=1	42.69	1.41E-33
TRINITY_DN15880_c0_g5_i2	sp_P31110_TLP_ORYSJ_Thaumatin-like_protein_OS=Oryza_sativa_subsp._japonica_OX=39947_GN=Os12g0628600_PE=1_SV=1	70.548	9.69E-69
TRINITY_DN15837_c0_g1_i4	sp_Q7Y0E8_NRX11_ORYSJ_Probable_nucleoredoxin_1-1_OS=Oryza_sativa_subsp._japonica_OX=39947_GN=Os03g0405500_PE=2_SV=1	80.825	0
TRINITY_DN15837_c0_g1_i4	sp_Q7Y0E8_NRX11_ORYSJ_Probable_nucleoredoxin_1-1_OS=Oryza_sativa_subsp._japonica_OX=39947_GN=Os03g0405500_PE=2_SV=1	38.75	1.07E-57
TRINITY_DN15837_c0_g1_i4	sp_Q7Y0E8_NRX11_ORYSJ_Probable_nucleoredoxin_1-1_OS=Oryza_sativa_subsp._japonica_OX=39947_GN=Os03g0405500_PE=2_SV=1	42.138	1.08E-34
TRINITY_DN15837_c0_g1_i4	sp_Q7Y0E8_NRX11_ORYSJ_Probable_nucleoredoxin_1-1_OS=Oryza_sativa_subsp._japonica_OX=39947_GN=Os03g0405500_PE=2_SV=1	37.241	1.75E-27
TRINITY_DN11567_c0_g1_i1	sp_Q2QSL4_CDKF2_ORYSJ_Putative_cyclin-dependent_kinase_F-2_OS=Oryza_sativa_subsp._japonica_OX=39947_GN=CDKF-2_PE=3_SV=1	41.503	1.79E-55
TRINITY_DN12754_c0_g1_i3	sp_Q8ISQ3_RL24_BRABE_60S_ribosomal_protein_L24_OS=Branchiostoma_belcheri_OX=7741_GN=RPL24_PE=2_SV=1	50.538	1.43E-22
TRINITY_DN12706_c0_g1_i2	sp_Q9CAB8_PXG5_ARATH_Probable_peroxygenase_5_OS=Arabidopsis_thaliana_OX=3702_GN=PXG5_PE=2_SV=1	56.471	1.45E-59
TRINITY_DN12795_c0_g2_i3	sp_O61231_RL10_DROME_60S_ribosomal_protein_L10_OS=Drosophila_melanogaster_OX=7227_GN=RpL10_PE=1_SV=1	71.845	7.8E-109
TRINITY_DN16903_c0_g1_i9	sp_Q8TGM7_ART2_YEAST_Putative_uncharacterized_protein_ART2_OS=Saccharomyces_cerevisiae_(strain_ATCC_204508_/_S288c)_OX=559292_GN=ART2_PE=5_SV=1	71.698	1.77E-19

TRINITY_DN16975_c0_g2_i6	sp_Q0DHF6_PDC1_ORYSJ_Pyruvate_decarboxylase_1_OS=Oryza_sativa_subsp._japonica_OX=39947_GN=PDC1_PE=2_SV=1	93.701	0
TRINITY_DN16989_c1_g1_i5	sp_Q9LMN7_WAK5_ARATH_Wall-associated_receptor_kinase_5_OS=Arabidopsis_thaliana_OX=3702_GN=WAK5_PE=2_SV=1	47.042	1.15E-101
TRINITY_DN20690_c3_g1_i2	sp_Q6K1S6_MYB30_ORYSJ_Transcription_factor_MYB30_OS=Oryza_sativa_subsp._japonica_OX=39947_GN=MYB30_PE=1_SV=1	50.273	4.09E-30
TRINITY_DN20665_c1_g2_i18	sp_Q2HIV9_BH035_ARATH_Transcription_factor_bHLH35_OS=Arabidopsis_thaliana_OX=3702_GN=BHLH35_PE=2_SV=1	44	4.8E-37
TRINITY_DN20626_c0_g2_i1	sp_Q7XJE6_MCA1_ARATH_Metacaspase-1_OS=Arabidopsis_thaliana_OX=3702_GN=AMC1_PE=1_SV=1	58.108	4.62E-62
TRINITY_DN20626_c0_g3_i6	sp_Q7XJE6_MCA1_ARATH_Metacaspase-1_OS=Arabidopsis_thaliana_OX=3702_GN=AMC1_PE=1_SV=1	41.29	4E-27
TRINITY_DN14818_c1_g1_i2	sp_P31009_RS2_DROME_40S_ribosomal_protein_S2_OS=Drosophila_melanogaster_OX=7227_GN=RpS2_PE=1_SV=2	76.923	9.37E-125
TRINITY_DN21167_c0_g2_i1	sp_O64477_Y2913_ARATH_G-type_lectin_S-receptor-like_serine/threonine-protein_kinase_At2g19130_OS=Arabidopsis_thaliana_OX=3702_GN=At2g19130_PE=2_SV=1	43.433	2.79E-146
TRINITY_DN13722_c5_g5_i2	sp_Q9LPQ3_M2K7_ARATH_Mitogen-activated_protein_kinase_kinase_7_OS=Arabidopsis_thaliana_OX=3702_GN=MKK7_PE=1_SV=1	47.794	1.31E-57
TRINITY_DN14345_c5_g1_i2	sp_Q65X92_C3H37_ORYSJ_Zinc_finger_CCCH_domain-containing_protein_37_OS=Oryza_sativa_subsp._japonica_OX=39947_GN=Os05g0525900_PE=2_SV=1	72.222	6.33E-51
TRINITY_DN14379_c0_g4_i3	sp_Q9FGQ9_FLZ1_ARATH_FCS-Like_Zinc_finger_1_OS=Arabidopsis_thaliana_OX=3702_GN=FLZ1_PE=1_SV=1	69.231	1.99E-12
TRINITY_DN14317_c1_g1_i1	sp_Q9SJA7_SOX_ARATH_Probable_sarcosine_oxidase_OS=Arabidopsis_thaliana_OX=3702_GN=At2g24580_PE=2_SV=1	54.545	5.81E-141
TRINITY_DN14304_c0_g3_i10	sp_C0SV51_CB60C_ARATH_Calmodulin-binding_protein_60_C_OS=Arabidopsis_thaliana_OX=3702_GN=CBP60C_PE=2_SV=1	44.351	2.28E-52
TRINITY_DN18943_c1_g2_i3	sp_Q9SD85_F3PH_ARATH_Flavonoid_3'-monooxygenase_OS=Arabidopsis_thaliana_OX=3702_GN=CYP75B1_PE=1_SV=1	44.326	7.44E-74
TRINITY_DN18943_c1_g2_i3	sp_Q9SD85_F3PH_ARATH_Flavonoid_3'-monooxygenase_OS=Arabidopsis_thaliana_OX=3702_GN=CYP75B1_PE=1_SV=1	34.483	7.44E-74
TRINITY_DN18954_c2_g2_i4	sp_P80299_HYES_RAT_Bifunctional_epoxide_hydrolase_2_OS=Rattus_norvegicus_OX=10116_GN=Ephx2_PE=1_SV=1	32.765	9.92E-30
TRINITY_DN18929_c2_g3_i4	sp_Q9LXG9_PCO1_ARATH_Plant_cysteine_oxidase_1_OS=Arabidopsis_thaliana_OX=3702_GN=PCO1_PE=1_SV=1	54.098	8.13E-29
TRINITY_DN19190_c2_g1_i6	sp_Q6Z4N6_40G2_ORYSJ_Ricin_B-like_lectin_R40G2_OS=Oryza_sativa_subsp._japonica_OX=39947_GN=R40G2_PE=2_SV=1	81.884	2.69E-76
TRINITY_DN19190_c2_g1_i6	sp_Q6Z4N6_40G2_ORYSJ_Ricin_B-like_lectin_R40G2_OS=Oryza_sativa_subsp._japonica_OX=39947_GN=R40G2_PE=2_SV=1	64.167	1.59E-52
TRINITY_DN20418_c0_g1_i7	sp_O49621_MLO1_ARATH_MLO-like_protein_1_OS=Arabidopsis_thaliana_OX=3702_GN=MLO1_PE=1_SV=1	77.358	5.31E-18
TRINITY_DN20418_c0_g1_i7	sp_O49621_MLO1_ARATH_MLO-like_protein_1_OS=Arabidopsis_thaliana_OX=3702_GN=MLO1_PE=1_SV=1	65.909	2.03E-09
TRINITY_DN20465_c1_g1_i4	sp_Q8LJJ9_STAD1_ORYSJ_Stearoyl-[acyl-carrier-protein]_9-desaturase_1,_chloroplastic_OS=Oryza_sativa_subsp._japonica_OX=39947_GN=Os01g0880800_PE=2_SV=1	93.805	0
TRINITY_DN20488_c1_g1_i7	sp_O48723_PLP2_ARATH_Patatin-like_protein_2_OS=Arabidopsis_thaliana_OX=3702_GN=PLP2_PE=1_SV=1	58.571	8.49E-112
TRINITY_DN20488_c1_g1_i13	sp_O48723_PLP2_ARATH_Patatin-like_protein_2_OS=Arabidopsis_thaliana_OX=3702_GN=PLP2_PE=1_SV=1	63.063	1.03E-87
TRINITY_DN20488_c1_g1_i13	sp_O48723_PLP2_ARATH_Patatin-like_protein_2_OS=Arabidopsis_thaliana_OX=3702_GN=PLP2_PE=1_SV=1	56.725	1.02E-55
TRINITY_DN10665_c1_g1_i1	sp_P42791_RL182_ARATH_60S_ribosomal_protein_L18-2_OS=Arabidopsis_thaliana_OX=3702_GN=RPL18B_PE=1_SV=2	61.979	3.08E-78
TRINITY_DN13988_c1_g1_i3	sp_O82081_UCC1_ARATH_Uclacyanin_1_OS=Arabidopsis_thaliana_OX=3702_GN=UCC1_PE=1_SV=1	47.273	3.01E-12
TRINITY_DN11129_c0_g2_i2	sp_P40525_RL34B_YEAST_60S_ribosomal_protein_L34-B_OS=Saccharomyces_cerevisiae_(strain_ATCC_204508_/_S288c)_OX=559292_GN=RPL34B_PE=1_SV=1	69.565	2.15E-39
TRINITY_DN10833_c0_g3_i1	sp_P24922_IF5A2_NICPL_Eukaryotic_translation_initiation_factor_5A-2_OS=Nicotiana_plumbaginifolia_OX=4092_GN=EIF-5A2_PE=2_SV=1	61.538	6.68E-63
TRINITY_DN18272_c1_g1_i1	sp_Q9FKQ1_DTX27_ARATH_Protein_DETOXIFICATION_27_OS=Arabidopsis_thaliana_OX=3702_GN=DTX27_PE=2_SV=1	62.864	0
TRINITY_DN18272_c1_g1_i1	sp_Q9FKQ1_DTX27_ARATH_Protein_DETOXIFICATION_27_OS=Arabidopsis_thaliana_OX=3702_GN=DTX27_PE=2_SV=1	67.442	0.000000248
TRINITY_DN18269_c1_g3_i2	sp_P84516_PER1_SORBI_Cationic_peroxidase_SPC4_OS=Sorghum_bicolor_OX=4558_GN=Sb03g046810_PE=1_SV=2	46.23	1.27E-75
TRINITY_DN18234_c2_g1_i6	sp_Q9LHL5_PGTB2_ARATH_Geranylgeranyl_transferase_type-2_subunit_beta_2_OS=Arabidopsis_thaliana_OX=3702_GN=RGTB2_PE=1_SV=1	57.447	0.000000104
TRINITY_DN10991_c2_g1_i2	sp_Q8ISP0_RS18_BRABE_40S_ribosomal_protein_S18_OS=Branchiostoma_belcheri_OX=7741_GN=RPS18_PE=2_SV=1	68.243	7.18E-74
TRINITY_DN10930_c0_g2_i1	sp_P55833_RS27_HOMAM_40S_ribosomal_protein_S27_OS=Homarus_americanus_OX=6706_GN=RPS27_PE=3_SV=2	73.171	1.08E-38
TRINITY_DN15708_c0_g1_i1	sp_O26373_Y273_METTH_UPF0098_protein_MTH_273_OS=Methanothermobacter_thermautotrophicus_(strain_ATCC_29096_/_DSM_1053_/_JCM_10044_/_NBRC_100330_/_Delta_H)_OX=187420_GN=MTH_273_PE=3_SV=1	46.341	1.13E-23

TRINITY_DN15771_c1_g3_i4	sp_P48166_RL36A_CAEEL_Ribosomal_protein_rpl-36.A_OS=Caenorhabditis_elegans_OX=6239_GN=rpl-36.A_PE=3_SV=2	74.757	8.21E-47
TRINITY_DN15775_c0_g3_i1	sp_Q56Y11_DDPS2_ARATH_Dehydrodolichyl_diphosphate_synthase_2_OS=Arabidopsis_thaliana_OX=3702_GN=At5g58770_PE=2_SV=2	47.034	3.56E-66
TRINITY_DN15786_c2_g2_i2	sp_Q8H0Y8_WRK41_ARATH_Probable_WRKY_transcription_factor_41_OS=Arabidopsis_thaliana_OX=3702_GN=WRKY41_PE=2_SV=2	65.882	1.23E-32
TRINITY_DN22598_c6_g1_i6	sp_Q2QYY8_PHT1A_ORYSJ_Phototropin-1A_OS=Oryza_sativa_subsp._japonica_OX=39947_GN=PHOT1A_PE=1_SV=2	72.197	0
TRINITY_DN22598_c6_g1_i6	sp_Q2QYY8_PHT1A_ORYSJ_Phototropin-1A_OS=Oryza_sativa_subsp._japonica_OX=39947_GN=PHOT1A_PE=1_SV=2	37.838	4.67E-24
TRINITY_DN22598_c6_g1_i6	sp_Q2QYY8_PHT1A_ORYSJ_Phototropin-1A_OS=Oryza_sativa_subsp._japonica_OX=39947_GN=PHOT1A_PE=1_SV=2	92.105	3.64E-13
TRINITY_DN11093_c0_g1_i1	sp_Q9SRN0_NHL1_ARATH_NDR1/HIN1-like_protein_1_OS=Arabidopsis_thaliana_OX=3702_GN=NHL1_PE=2_SV=1	42.197	2.09E-40
TRINITY_DN19240_c1_g3_i4	sp_A7PZL3_PGLR_VITVI_Probable_polygalacturonase_OS=Vitis_vinifera_OX=29760_GN=GSVIVT00026920001_PE=1_SV=1	68.578	0
TRINITY_DN19204_c1_g2_i4	sp_M0Y4P1_UGT13_HORVV_UDP-glucosyltransferase_UGT13248_OS=Hordeum_vulgare_subsp._vulgare_OX=112509_PE=2_SV=1	64.532	9.73E-64
TRINITY_DN20137_c1_g2_i3	sp_Q9LV58_MBF1C_ARATH_Multiprotein-bridging_factor_1c_OS=Arabidopsis_thaliana_OX=3702_GN=MBF1C_PE=1_SV=1	56.164	1.23E-32
TRINITY_DN18572_c1_g1_i1	sp_Q6K9A2_CCR1_ORYSJ_Cinnamoyl-CoA_reductase_1_OS=Oryza_sativa_subsp._japonica_OX=39947_GN=CCR1_PE=1_SV=1	88.433	1.41E-163
TRINITY_DN16711_c1_g1_i11	sp_P51110_DFRA_VITVI_Dihydroflavonol_4-reductase_OS=Vitis_vinifera_OX=29760_GN=DFR_PE=1_SV=1	27.225	8.93E-20
TRINITY_DN16711_c1_g1_i11	sp_P51110_DFRA_VITVI_Dihydroflavonol_4-reductase_OS=Vitis_vinifera_OX=29760_GN=DFR_PE=1_SV=1	27.885	8.93E-20
TRINITY_DN21756_c2_g1_i7	sp_Q0D5R2_CRK10_ORYSJ_Cysteine-rich_receptor-like_protein_kinase_10_OS=Oryza_sativa_subsp._japonica_OX=39947_GN=CRK10_PE=2_SV=1	62.281	8.21E-37
TRINITY_DN21756_c2_g1_i7	sp_Q0D5R2_CRK10_ORYSJ_Cysteine-rich_receptor-like_protein_kinase_10_OS=Oryza_sativa_subsp._japonica_OX=39947_GN=CRK10_PE=2_SV=1	33.473	1.41E-14
TRINITY_DN21756_c2_g1_i15	sp_Q0D5R3_CRK6_ORYSJ_Cysteine-rich_receptor-like_protein_kinase_6_OS=Oryza_sativa_subsp._japonica_OX=39947_GN=CRK6_PE=1_SV=1	45.055	2.73E-106
TRINITY_DN21725_c0_g1_i15	sp_P49175_INV1_MAIZE_Beta-fructofuranosidase_1_OS=Zea_mays_OX=4577_GN=IVR1_PE=3_SV=1	86.944	0
TRINITY_DN21725_c0_g1_i15	sp_P49175_INV1_MAIZE_Beta-fructofuranosidase_1_OS=Zea_mays_OX=4577_GN=IVR1_PE=3_SV=1	89.286	0.000000126
TRINITY_DN21719_c0_g6_i1	sp_P04707_ADH2_MAIZE_Alcohol_dehydrogenase_2_OS=Zea_mays_OX=4577_GN=ADH2_PE=2_SV=1	95.094	0
TRINITY_DN21795_c0_g1_i1	sp_P29250_LOX2_ORYSJ_Linoleate_9S-lipoxygenase_2_OS=Oryza_sativa_subsp._japonica_OX=39947_GN=LOX1.1_PE=2_SV=2	75.93	0
TRINITY_DN9783_c0_g2_i2	sp_O82337_AGP16_ARATH_Arabinogalactan_protein_16_OS=Arabidopsis_thaliana_OX=3702_GN=AGP16_PE=1_SV=1	81.081	1.02E-12
TRINITY_DN13209_c0_g3_i1	sp_Q02921_NO93_SOYBN_Early_nodulin-93_OS=Glycine_max_OX=3847_PE=2_SV=1	64.286	2.39E-26
TRINITY_DN13227_c0_g1_i16	sp_Q9M060_IF62_ARATH_Eukaryotic_translation_initiation_factor_6-2_OS=Arabidopsis_thaliana_OX=3702_GN=EIF6-2_PE=2_SV=1	69.672	1.83E-128
TRINITY_DN20951_c1_g4_i1	sp_O48716_JGB_ARATH_Protein_JINGUBANG_OS=Arabidopsis_thaliana_OX=3702_GN=JGB_PE=1_SV=1	48.684	2.81E-96
TRINITY_DN16605_c1_g1_i2	sp_P42158_CKL1_ARATH_Casein_kinase_1-like_protein_1_OS=Arabidopsis_thaliana_OX=3702_GN=CKL1_PE=2_SV=2	88.837	8.26E-146
TRINITY_DN12956_c0_g3_i1	sp_P25387_GBLP_CHLRE_Guanine_nucleotide-binding_protein_subunit_beta-like_protein_OS=Chlamydomonas_reinhardtii_OX=3055_GN=GBLP_PE=2_SV=1	80.18	2.24E-127
TRINITY_DN12956_c0_g3_i1	sp_P25387_GBLP_CHLRE_Guanine_nucleotide-binding_protein_subunit_beta-like_protein_OS=Chlamydomonas_reinhardtii_OX=3055_GN=GBLP_PE=2_SV=1	28.022	5.12E-11
TRINITY_DN15464_c2_g1_i5	sp_Q42376_LEA3_MAIZE_Late_embryogenesis_abundant_protein,_group_3_OS=Zea_mays_OX=4577_GN=MGL3_PE=2_SV=1	67.273	3.37E-61
TRINITY_DN18882_c2_g4_i3	sp_O22259_ERF71_ARATH_Ethylene-responsive_transcription_factor_ERF071_OS=Arabidopsis_thaliana_OX=3702_GN=ERF071_PE=2_SV=1	46.067	4.77E-25
TRINITY_DN18813_c1_g1_i3	sp_Q5Z6F0_TIP22_ORYSJ_Probable_aquaporin_TIP2-2_OS=Oryza_sativa_subsp._japonica_OX=39947_GN=TIP2-2_PE=2_SV=1	93.137	1.7E-128
TRINITY_DN18813_c1_g1_i3	sp_Q5Z6F0_TIP22_ORYSJ_Probable_aquaporin_TIP2-2_OS=Oryza_sativa_subsp._japonica_OX=39947_GN=TIP2-2_PE=2_SV=1	91.111	5.34E-17
TRINITY_DN19001_c0_g1_i18	sp_P93798_SNAA_VITVI_Alpha-soluble_NSF_attachment_protein_OS=Vitis_vinifera_OX=29760_PE=2_SV=1	79.592	1.03E-50
TRINITY_DN19026_c3_g2_i4	sp_P42736_RAP23_ARATH_Ethylene-responsive_transcription_factor_RAP2-3_OS=Arabidopsis_thaliana_OX=3702_GN=RAP2-3_PE=1_SV=2	50.746	9.81E-30
TRINITY_DN20811_c1_g1_i15	sp_F4HRJ4_M3K3A_ARATH_Mitogen-activated_protein_kinase_kinase_kinase_3_OS=Arabidopsis_thaliana_OX=3702_GN=MAPKKK3_PE=1_SV=1	79.92	5.23E-148
TRINITY_DN19858_c2_g2_i5	sp_Q4W8D0_GLN13_ORYSJ_Glutamine_synthetase_cytosolic_isozyme_1-3_OS=Oryza_sativa_subsp._japonica_OX=39947_GN=GLN1-3_PE=2_SV=1	91.2	3.87E-155
TRINITY_DN19858_c2_g2_i5	sp_Q4W8D0_GLN13_ORYSJ_Glutamine_synthetase_cytosolic_isozyme_1-3_OS=Oryza_sativa_subsp._japonica_OX=39947_GN=GLN1-3_PE=2_SV=1	88.406	3.87E-155
TRINITY_DN19858_c2_g2_i15	sp_P38560_GLNA2_MAIZE_Glutamine_synthetase_root_isozyme_2_OS=Zea_mays_OX=4577_GN=GLN2_PE=2_SV=1	88.889	3.96E-149
TRINITY_DN19882_c0_g1_i7	sp_Q6K7H2_KCY4_ORYSJ_UMP-CMP_kinase_4_OS=Oryza_sativa_subsp._japonica_OX=39947_GN=Os02g0778400_PE=2_SV=1	94.33	3.46E-128
TRINITY_DN18037_c0_g1_i1	sp_P14749_AGAL_CYATE_Alpha-galactosidase_OS=Cyamopsis_tetragonoloba_OX=3832_PE=1_SV=1	72.189	1.28E-96
TRINITY_DN18037_c0_g1_i1	sp_P14749_AGAL_CYATE_Alpha-galactosidase_OS=Cyamopsis_tetragonoloba_OX=3832_PE=1_SV=1	65.385	1.28E-96

TRINITY_DN18037_c0_g1_i1	sp_P14749_AGAL_CYATE_Alpha-galactosidase_OS=Cyamopsis_tetragonoloba_OX=3832_PE=1_SV=1	56.667	1.28E-96
TRINITY_DN18037_c0_g1_i1	sp_P14749_AGAL_CYATE_Alpha-galactosidase_OS=Cyamopsis_tetragonoloba_OX=3832_PE=1_SV=1	59.091	1.28E-96
TRINITY_DN18037_c0_g1_i1	sp_P14749_AGAL_CYATE_Alpha-galactosidase_OS=Cyamopsis_tetragonoloba_OX=3832_PE=1_SV=1	56.25	3.28E-12
TRINITY_DN18037_c0_g1_i23	sp_Q9FT97_AGAL1_ARATH_Alpha-galactosidase_1_OS=Arabidopsis_thaliana_OX=3702_GN=AGAL1_PE=2_SV=1	71.795	1.74E-124
TRINITY_DN18037_c0_g1_i23	sp_Q9FT97_AGAL1_ARATH_Alpha-galactosidase_1_OS=Arabidopsis_thaliana_OX=3702_GN=AGAL1_PE=2_SV=1	56.552	1.74E-124
TRINITY_DN14058_c0_g1_i6	sp_Q8RVH5_7SBG2_SOYBN_Basic_7S_globulin_2_OS=Glycine_max_OX=3847_PE=1_SV=1	28.058	1.43E-21
TRINITY_DN14018_c0_g6_i1	sp_Q9SJ36_RS172_ARATH_40S_ribosomal_protein_S17-2_OS=Arabidopsis_thaliana_OX=3702_GN=RPS17B_PE=2_SV=3	74.49	6.42E-41
TRINITY_DN17564_c0_g1_i12	sp_F4IK45_C70B2_ARATH_Cytochrome_P450_709B2_OS=Arabidopsis_thaliana_OX=3702_GN=CYP709B2_PE=2_SV=1	47.143	5.29E-103
TRINITY_DN17534_c0_g2_i6	sp_Q0JAW2_SPXM2_ORYSJ_SPX_domain-containing_membrane_protein_Os04g0573000_OS=Oryza_sativa_subsp._japonica_OX=39947_GN=Os04g0573000_PE=2_SV=2	81.597	0
TRINITY_DN17520_c1_g1_i5	sp_Q9T0I5_PRP4_ARATH_Proline-rich_protein_4_OS=Arabidopsis_thaliana_OX=3702_GN=PRP4_PE=2_SV=1	40.698	3.13E-09
TRINITY_DN15636_c2_g3_i2	sp_Q9LFT6_HNL_ARATH_Alpha-hydroxynitrile_lyase_OS=Arabidopsis_thaliana_OX=3702_GN=HNL_PE=1_SV=1	52.222	1.87E-12
TRINITY_DN17363_c1_g2_i1	sp_Q6Z955_CKX11_ORYSJ_Cytokinin_dehydrogenase_11_OS=Oryza_sativa_subsp._japonica_OX=39947_GN=CKX11_PE=2_SV=1	73.622	3.74E-114
TRINITY_DN17363_c1_g2_i11	sp_Q6Z955_CKX11_ORYSJ_Cytokinin_dehydrogenase_11_OS=Oryza_sativa_subsp._japonica_OX=39947_GN=CKX11_PE=2_SV=1	81.72	3.07E-114
TRINITY_DN17363_c1_g2_i11	sp_Q6Z955_CKX11_ORYSJ_Cytokinin_dehydrogenase_11_OS=Oryza_sativa_subsp._japonica_OX=39947_GN=CKX11_PE=2_SV=1	73.077	3.19E-40
TRINITY_DN17363_c1_g2_i11	sp_Q6Z955_CKX11_ORYSJ_Cytokinin_dehydrogenase_11_OS=Oryza_sativa_subsp._japonica_OX=39947_GN=CKX11_PE=2_SV=1	81.609	2.11E-28
TRINITY_DN22469_c3_g1_i1	sp_Q9M2K6_PDCB5_ARATH_PLASMODESMATA_CALLOSE-BINDING_PROTEIN_5_OS=Arabidopsis_thaliana_OX=3702_GN=PDCB5_PE=2_SV=2	53.409	3.31E-24
TRINITY_DN13326_c0_g3_i6	sp_Q8VZM9_FLZ2_ARATH_FCS-Like_Zinc_finger_2_OS=Arabidopsis_thaliana_OX=3702_GN=FLZ2_PE=1_SV=1	68.627	3.17E-17
TRINITY_DN13377_c1_g1_i3	sp_Q6I587_CDPKF_ORYSJ_Calcium-dependent_protein_kinase_15_OS=Oryza_sativa_subsp._japonica_OX=39947_GN=CPK15_PE=2_SV=1	96.667	2.67E-161
TRINITY_DN17626_c0_g2_i1	sp_P27538_PR2_PETCR_Pathogenesis-related_protein_2_OS=Petroselinum_crispum_OX=4043_GN=PR2_PE=2_SV=1	28.571	0.0000003
TRINITY_DN12269_c0_g1_i1	sp_B3FHT4_SUCA_BLAHN_Succinate--CoA_ligase_[ADP-forming]_subunit_alpha_OS=Blastocystis_sp._subtype_1_(strain_ATCC_50177_/_NandII)_OX=478820_GN=SCSa_PE=1_SV=1	73.883	2.12E-130
TRINITY_DN12250_c0_g1_i1	sp_P49396_RS3A_BRACM_40S_ribosomal_protein_S3a_OS=Brassica_campestris_OX=3711_GN=RPS3A_PE=2_SV=2	71.064	9.67E-117
TRINITY_DN12250_c0_g1_i3	sp_Q764D2_RS3A_LENED_40S_ribosomal_protein_S1_OS=Lentinula_edodes_OX=5353_GN=RPS1_PE=2_SV=1	69.469	1.53E-116
TRINITY_DN21819_c0_g1_i2	sp_Q9ATL7_TIP31_MAIZE_Aquaporin_TIP3-1_OS=Zea_mays_OX=4577_GN=TIP3-1_PE=2_SV=1	95.473	7.53E-126
TRINITY_DN21886_c1_g1_i4	sp_Q9ZUF4_RIPK_ARATH_Serine/threonine-protein_kinase_RIPK_OS=Arabidopsis_thaliana_OX=3702_GN=RIPK_PE=1_SV=1	66.667	2.71E-65
TRINITY_DN21886_c1_g1_i4	sp_Q9ZUF4_RIPK_ARATH_Serine/threonine-protein_kinase_RIPK_OS=Arabidopsis_thaliana_OX=3702_GN=RIPK_PE=1_SV=1	80.328	2.71E-65
TRINITY_DN14423_c0_g1_i5	sp_Q9M069_E137_ARATH_Glucan_endo-1,3-beta-glucosidase_7_OS=Arabidopsis_thaliana_OX=3702_GN=At4g34480_PE=1_SV=2	39.716	3.23E-24
TRINITY_DN14423_c0_g1_i5	sp_Q9M069_E137_ARATH_Glucan_endo-1,3-beta-glucosidase_7_OS=Arabidopsis_thaliana_OX=3702_GN=At4g34480_PE=1_SV=2	44.578	5.01E-18
TRINITY_DN14493_c1_g1_i17	sp_Q7F8T6_OMT17_ORYSJ_Tricin_synthase_2_OS=Oryza_sativa_subsp._japonica_OX=39947_GN=ROMT-17_PE=1_SV=1	71.324	3.9E-105
TRINITY_DN17961_c0_g1_i1	sp_Q40702_MADS2_ORYSJ_MADS-box_transcription_factor_2_OS=Oryza_sativa_subsp._japonica_OX=39947_GN=MADS2_PE=2_SV=1	93.651	1.77E-130
TRINITY_DN16287_c0_g1_i2	sp_Q8LGG8_USPAL_ARATH_Universal_stress_protein_A-like_protein_OS=Arabidopsis_thaliana_OX=3702_GN=At3g01520_PE=1_SV=2	34.862	5.04E-13
TRINITY_DN16219_c5_g2_i3	sp_P27322_HSP72_SOLLC_Heat_shock_cognate_70_kDa_protein_2_OS=Solanum_lycopersicum_OX=4081_GN=HSC-2_PE=2_SV=1	93.519	6.74E-68
TRINITY_DN16219_c5_g2_i11	sp_P09189_HSP7C_PETHY_Heat_shock_cognate_70_kDa_protein_OS=Petunia_hybrida_OX=4102_GN=HSP70_PE=2_SV=1	93.514	3.87E-123
TRINITY_DN15010_c0_g2_i2	sp_Q6H6D2_HEM3_ORYSJ_Porphobilinogen_deaminase,_chloroplastic_OS=Oryza_sativa_subsp._japonica_OX=39947_GN=HEMC_PE=2_SV=1	82.54	1.56E-95
TRINITY_DN19356_c0_g2_i2	sp_O64697_C7101_ARATH_Cytochrome_P450_710A1_OS=Arabidopsis_thaliana_OX=3702_GN=CYP710A1_PE=1_SV=1	63.41	0
TRINITY_DN14618_c0_g1_i1	sp_Q55403_Y575_SYNY3_Thylakoid_membrane_protein_slr0575_OS=Synechocystis_sp._(strain_PCC_6803_/_Kazusa)_OX=1111708_GN=slr0575_PE=4_SV=1	40.816	2.34E-26
TRINITY_DN14632_c0_g1_i11	sp_Q8LGG8_USPAL_ARATH_Universal_stress_protein_A-like_protein_OS=Arabidopsis_thaliana_OX=3702_GN=At3g01520_PE=1_SV=2	44.444	3.28E-12
TRINITY_DN22005_c2_g1_i1	sp_Q9ZUF4_RIPK_ARATH_Serine/threonine-protein_kinase_RIPK_OS=Arabidopsis_thaliana_OX=3702_GN=RIPK_PE=1_SV=1	65.775	3.58E-84
TRINITY_DN22073_c1_g2_i8	sp_P19954_PRSP1_SPIOL_Ribosome-binding_factor_PSRP1,_chloroplastic_OS=Spinacia_oleracea_OX=3562_GN=PSRP1_PE=1_SV=2	60	1.55E-48
TRINITY_DN15299_c0_g2_i4	sp_P37834_PER1_ORYSJ_Peroxidase_1_OS=Oryza_sativa_subsp._japonica_OX=39947_GN=PRX74_PE=2_SV=2	72.642	1.84E-50

TRINITY_DN15296_c0_g1_i5	sp_Q09LL3_BBD_ORYMI_Bifunctional_nuclease_OS=Oryza_minuta_OX=63629_GN=BBD_PE=2_SV=1	75.543	1.68E-64
TRINITY_DN21081_c2_g2_i3	sp_Q651D5_PIP27_ORYSJ_Probable_aquaporin_PIP2-7_OS=Oryza_sativa_subsp._japonica_OX=39947_GN=PIP2-7_PE=2_SV=2	84.091	4.13E-48
TRINITY_DN21081_c2_g2_i3	sp_Q651D5_PIP27_ORYSJ_Probable_aquaporin_PIP2-7_OS=Oryza_sativa_subsp._japonica_OX=39947_GN=PIP2-7_PE=2_SV=2	77.5	4.13E-48
TRINITY_DN16037_c0_g1_i3	sp_Q7EZ57_HS188_ORYSJ_18.8_kDa_class_V_heat_shock_protein_OS=Oryza_sativa_subsp._japonica_OX=39947_GN=HSP18.8_PE=2_SV=1	56.429	5.5E-35
TRINITY_DN18728_c0_g5_i4	sp_Q9ARD5_LT02_HORVU_Low_temperature-induced_protein_lt101.2_OS=Hordeum_vulgare_OX=4513_GN=LT101.2_PE=2_SV=1	79.63	3.83E-20
TRINITY_DN18720_c2_g2_i6	sp_Q43062_PME_PRUPE_Pectinesterase/pectinesterase_inhibitor_PPE8B_OS=Prunus_persica_OX=3760_PE=2_SV=1	55.22	6.47E-158
TRINITY_DN18773_c0_g1_i11	sp_Q9P4X3_UTP7_SCHPO_Probable_U3_small_nucleolar_RNA-associated_protein_7_OS=Schizosaccharomyces_pombe_(strain_972_/_ATCC_24843)_OX=284812_GN=utp7_PE=3_SV=1	48.958	1.11E-87
TRINITY_DN16512_c0_g2_i1	sp_O81117_C94A1_VICSA_Cytochrome_P450_94A1_OS=Vicia_sativa_OX=3908_GN=CYP94A1_PE=2_SV=2	42.331	1.22E-25
TRINITY_DN16567_c0_g3_i5	sp_Q9LPN5_VP601_ARATH_Vacuolar_protein_sorting-associated_protein_60.1_OS=Arabidopsis_thaliana_OX=3702_GN=VPS60-1_PE=1_SV=1	77.35	3.5E-119
TRINITY_DN19481_c1_g1_i8	sp_Q8W034_RNP1_ARATH_Heterogeneous_nuclear_ribonucleoprotein_1_OS=Arabidopsis_thaliana_OX=3702_GN=RNP1_PE=1_SV=1	56.25	3.37E-69
TRINITY_DN19424_c1_g1_i8	sp_Q5EAF2_IP5PB_ARATH_Type_IV_inositol_polyphosphate_5-phosphatase_11_OS=Arabidopsis_thaliana_OX=3702_GN=IP5P11_PE=1_SV=1	58.442	8.54E-22
TRINITY_DN17828_c1_g2_i3	sp_Q93YU2_RUS6_ARATH_Protein_root_UVB_sensitive_6_OS=Arabidopsis_thaliana_OX=3702_GN=RUS6_PE=2_SV=1	72.843	2.9E-155
TRINITY_DN21909_c0_g2_i10	sp_P82953_LECH_HORVV_Horcolin_OS=Hordeum_vulgare_subsp._vulgare_OX=112509_PE=1_SV=2	45.255	3.72E-30
TRINITY_DN16874_c0_g2_i2	sp_P49353_FPPS_MAIZE_Farnesyl_pyrophosphate_synthase_OS=Zea_mays_OX=4577_GN=FPS_PE=2_SV=1	79.518	3E-42
TRINITY_DN11265_c0_g1_i2	sp_P51412_RK21_ARATH_50S_ribosomal_protein_L21,_chloroplastic_OS=Arabidopsis_thaliana_OX=3702_GN=RPL21_PE=2_SV=1	74.194	2.98E-09
TRINITY_DN11273_c0_g1_i2	sp_Q67IX6_PDI14_ORYSJ_Protein_disulfide_isomerase-like_1-4_OS=Oryza_sativa_subsp._japonica_OX=39947_GN=PDIL1-4_PE=2_SV=1	55.906	2.64E-30
TRINITY_DN18700_c0_g1_i10	sp_P51615_MAOX_VITVI_NADP-dependent_malic_enzyme_OS=Vitis_vinifera_OX=29760_PE=2_SV=1	80.142	0
TRINITY_DN18660_c1_g2_i14	sp_Q6GLE1_CCD25_XENTR_Coiled-coil_domain-containing_protein_25_OS=Xenopus_tropicalis_OX=8364_GN=ccdc25_PE=2_SV=1	60.44	1.81E-28
TRINITY_DN18660_c1_g2_i14	sp_Q6GLE1_CCD25_XENTR_Coiled-coil_domain-containing_protein_25_OS=Xenopus_tropicalis_OX=8364_GN=ccdc25_PE=2_SV=1	66.667	1.7E-09
TRINITY_DN16359_c2_g1_i3	sp_Q9SSA8_RA212_ARATH_Ethylene-responsive_transcription_factor_RAP2-12_OS=Arabidopsis_thaliana_OX=3702_GN=RAP2-12_PE=1_SV=1	38.191	0.00000068
TRINITY_DN12182_c0_g1_i1	sp_Q8LDL0_HHL1_ARATH_Protein_HHL1,_chloroplastic_OS=Arabidopsis_thaliana_OX=3702_GN=HHL1_PE=1_SV=1	72.973	1.09E-54
TRINITY_DN14998_c1_g5_i1	sp_Q39613_CYPH_CATRO_Peptidyl-prolyl_cis-trans_isomerase_OS=Catharanthus_roseus_OX=4058_GN=PCKR1_PE=1_SV=1	80.702	2.46E-90
TRINITY_DN14971_c0_g1_i2	sp_P49133_TPT_MAIZE_Triose_phosphate/phosphate_translocator,_chloroplastic_OS=Zea_mays_OX=4577_GN=TPT_PE=1_SV=1	87.853	0
TRINITY_DN14937_c0_g3_i3	sp_O81815_MO1_ARATH_Monooxygenase_1_OS=Arabidopsis_thaliana_OX=3702_GN=MO1_PE=2_SV=1	42.529	2.24E-33
TRINITY_DN14937_c0_g3_i3	sp_O81815_MO1_ARATH_Monooxygenase_1_OS=Arabidopsis_thaliana_OX=3702_GN=MO1_PE=2_SV=1	64.286	1.19E-13
TRINITY_DN14973_c0_g1_i1	sp_P02879_RICI_RICCO_Ricin_OS=Ricinus_communis_OX=3988_PE=1_SV=1	38.596	9.75E-48
TRINITY_DN14973_c0_g1_i1	sp_P02879_RICI_RICCO_Ricin_OS=Ricinus_communis_OX=3988_PE=1_SV=1	36.709	9.75E-48
TRINITY_DN14973_c0_g1_i1	sp_P02879_RICI_RICCO_Ricin_OS=Ricinus_communis_OX=3988_PE=1_SV=1	34.314	0.000000962
TRINITY_DN20537_c1_g1_i7	sp_Q39134_AAP3_ARATH_Amino_acid_permease_3_OS=Arabidopsis_thaliana_OX=3702_GN=AAP3_PE=1_SV=2	66.387	2.91E-52
TRINITY_DN20548_c0_g1_i7	sp_P22988_LDHA_HORVU_L-lactate_dehydrogenase_A_OS=Hordeum_vulgare_OX=4513_PE=1_SV=1	83.636	6.76E-127
TRINITY_DN21554_c1_g3_i3	sp_F4KFM8_LEA65_ARATH_Late_embryogenesis_abundant_protein_At5g17165_OS=Arabidopsis_thaliana_OX=3702_GN=At5g17165_PE=3_SV=1	40.698	3.06E-12
TRINITY_DN21575_c1_g5_i1	sp_P08440_ALF_MAIZE_Fructose-bisphosphate_aldolase,_cytoplasmic_isozyme_OS=Zea_mays_OX=4577_PE=2_SV=1	94.479	9.29E-110
TRINITY_DN21509_c2_g3_i2	sp_Q0DM48_MAN3_ORYSJ_Mannan_endo-1,4-beta-mannosidase_3_OS=Oryza_sativa_subsp._japonica_OX=39947_GN=MAN3_PE=2_SV=2	75.389	1.52E-155

Supplementary table 2. *In silico* expression matrix and UniProt annotations for DEGs that coincide in both root and leaf tissue under drought and salinity stress.

UNIGENE IDS	UniProt annotation	CONTROL_LEAF1	CONTROL_LEAF2	PEG_TREATED_LEAVES1	PEG_TREATED_LEAVES2	SALT_TREATED_LEAVES1	SALT_TREATED_LEAVES2	CONTROL_ROOT1	CONTROL_ROOT2	PEG_TREATED_ROOT1	PEG_TREATED_ROOT2	SALT_TREATED_ROOT1	SALT_TREATED_ROOT2
TRINITY_13371_length_1982_cov_27.060115_g8376_i0	--	46.64	38.37	905.2	303.8	148.79	157.52	37.78	39.34	118.2	103.8	111.8	90.85
TRINITY_21179_length_1587_cov_31.185771_g5866_i1	sp_P24805_TSJT1_TOBAC_Stem-specific_protein_TSJT1	3.944	3.808	127.5	56.54	21.994	36.426	12.08	10.44	17.48	19.81	29.94	29.83
TRINITY_26921_length_1373_cov_23.520706_g17146_i0	sp_Q42993_CHI1_ORYSJ_Chitinase_1	46.9	23.78	0	0.323	0.569	0.062	252.3	260.8	113.2	97.3	26.12	24.372
TRINITY_2861_length_3373_cov_28.611077_g1589_i1	sp_P24805_TSJT1_TOBAC_Stem-specific_protein_TSJT1	10.83	14.65	281	142.8	41.462	36.495	17.46	17.76	30.62	25.15	27.57	27.558
TRINITY_32727_length_1195_cov_41.831261_g21116_i0	sp_P29022_CHIA_MAIZE_Endochitinase_A	3.497	5.704	27.39	50.15	194.72	49.02	78.29	70.15	119.8	119.8	110.7	102.59
TRINITY_35304_length_1125_cov_27.677083_g22970_i0	sp_Q9ARD5_LT02_HORVU_Low_temperature-induced_protein_lt101.2	1.422	2.744	394.1	169.1	140.26	234.74	10.75	10.73	34.2	32.9	49.74	40.479
TRINITY_39367_length_1025_cov_29.965481_g25910_i0	sp_Q43194_NLTP2_SORBI_Non-specific_lipid-transfer_protein_2	25.09	34.1	3438	1707	511.19	889.32	1419	1301	638.6	533.6	455.6	366
TRINITY_43470_length_936_cov_24.776240_g29056_i0	-	17.09	25.62	807.2	227.4	70.547	90.937	39	44.83	85.28	81.48	100.6	83.632
TRINITY_51107_length_791_cov_15.894737_g35348_i0	-	22.62	17.82	0.525	1.779	2.425	0	56.31	55.97	103.6	89.09	98.2	75.456
TRINITY_53585_length_748_cov_32.690722_g37487_i0	sp_P51119_GLNA2_VITVI_Glutamine_synthetase_cytosolic_isozyme_2	2.463	2.512	22.38	46.92	26.192	49.597	23.13	19.6	101.7	71.29	126.3	134.16
TRINITY_6127_length_2666_cov_32.976126_g3875_i0	-	133	118.8	601.1	682.8	777.06	634.1	298	325.5	573.8	1826	733.4	948.18
TRINITY_6483_length_2616_cov_33.980762_g4098_i0	sp_Q651X6_CSLE6_ORYSJ_Cellulose_synthase-like_protein_E6	20.65	20.94	0.543	4.358	1.037	7.256	1.501	1.261	0.424	0.259	0.09	0.201
TRINITY_69571_length_531_cov_26.666667_g52643_i0	sp_A5H8G4_PER1_MAIZE_Peroxidase_1	14.62	26.78	233.5	164.4	168.49	78.097	62.26	47.31	149.4	111.4	140.1	147.7
TRINITY_8742_length_2349_cov_23.646491_g5523_i0	sp_Q6WNR0_C81E7_MEDTR_Isoflavone_2'-hydroxylase	0	0.246	124.9	20.42	7.008	5.045	2.788	2.921	1.188	1.191	1.227	0.869
TRINITY_BIN_12030_5	sp_Q7F2L3_NAC48_ORYSJ_NAC_domain-containing_protein_48	11.87	15.82	336	121.8	45.744	52.933	35.9	35.65	57.1	51.38	52.18	56.018
TRINITY_BIN_12545_6	sp_Q942D4_BURP3_ORYSJ_BURP_domain-containing_protein_3	12.95	5.516	86.54	109.1	60.813	36.58	22.52	24.7	9.672	12.26	10.11	7.441
TRINITY_BIN_1284_9	-	3.13	5.704	20.75	24.21	21.291	24.171	10.09	14.2	25.39	24.67	31.1	30.153
TRINITY_BIN_20507_2	-	8.408	17.62	0	1.019	0	0.254	36.06	33.51	12.92	11.08	4.357	6.995
TRINITY_BIN_25884_1	-	14.9	8.072	0	0.356	0	0.863	12.33	12.17	33.98	22.84	31.16	33.261
TRINITY_BIN_276_3	sp_A5H8G4_PER1_MAIZE_Peroxidase_1	4.068	6.884	84.13	66.56	66.5	27.652	15.92	13.16	40.08	30.34	38.31	42.551
TRINITY_BIN_4317_1	sp_B8BHF1_GLO15_ORYSI_Very-long-chain_aldehyde_decarbonylase_GL1-5	0.953	0.825	37.6	33.5	34.003	49.197	3.337	2.379	0.785	0.715	0.214	1.259
TRINITY_BIN_6163_1	-	16.98	11.23	364.4	168.1	73.809	81.394	41.83	46.49	72.85	69.7	111.8	116.86
TRINITY_BIN_6373_5	sp_Q6RVV4_TIC32_PEA_Short-chain_dehydrogenase_TIC_32,_chloroplastic	2.441	4.923	40.27	19.5	30.808	48.735	8.029	5.435	1.951	1.47	1.52	2.517
TRINITY_BIN_6942_3	sp_Q9FEB4_PSK5_ARATH_Phytosulfokines_5	3.115	0.239	25.47	15.46	14.852	12.085	12.26	12.92	28.78	29.57	37.84	53.947
TRINITY_Contig10696	sp_Q6Z4N4_40G3_ORYSJ_Ricin_B-like_lectin_R40G3	50.84	134.9	1936	2018	478.63	427.74	83.68	77.69	28.74	29.71	20.83	18.513
TRINITY_Contig10717	-	0.762	3.67	185.5	22.71	28.533	35.655	4.049	4.097	9.566	7.736	15.97	8.254
TRINITY_Contig11570	sp_Q5SMM8_PHT1_ORYSJ_Putrescine_hydroxycinnamoyltransferase_1	2.338	2.411	51.26	38.52	14.785	25.696	35.2	34.15	54.48	54.38	46.14	69.396
TRINITY_Contig11942	sp_B7F7B9_SPSA2_ORYSJ_Probable_sucrose-phosphate_synthase_2	82.42	72.17	3.6	3.525	3.596	3.697	59.29	55.21	22.23	19.37	26.64	22.668
TRINITY_Contig13523	sp_Q9ARD5_LT02_HORVU_Low_temperature-induced_protein_lt101.2	1.488	7.76	765	379.4	329.27	701.83	16.91	18.51	63.52	57.7	63.05	73.796
TRINITY_Contig14418	sp_Q10MX3_ASNS1_ORYSJ_Asparagine_synthetase_[glutamine-hydrolyzing]_1	4.706	18	163.4	91.09	123.88	208.19	43.55	42.02	87.61	63.8	84.46	74.743
TRINITY_Contig14687	sp_Q5N9N2_P2C09_ORYSJ_Probable_protein_phosphatase_2C_9	3.658	4.85	42.88	24.55	26.108	29.94	26.01	21.22	39.14	37.37	45.23	47.174
TRINITY_Contig16081	sp_P29022_CHIA_MAIZE_Endochitinase_A	0.902	0.384	8.811	15.95	53.086	11.646	59.35	64.28	142.1	96.66	114.6	96.721
TRINITY_Contig16806	sp_Q9LFR7_CXE17_ARATH_Probable_carboxylesterase_17	13.49	7.196	96.33	64.41	33.902	53.549	58.28	54.97	99.17	99.86	78.55	110.17
TRINITY_Contig17988	sp_A5H8G4_PER1_MAIZE_Peroxidase_1	21.9	28.15	323.5	245.1	321.13	118.61	125.1	120.6	264.4	232.3	380.5	359.84
TRINITY_Contig18138	sp_P38560_GLNA2_MAIZE_Glutamine_synthetase_root_isozyme_2	3.812	7.948	214.1	192.2	87.958	150.57	47.47	40.44	157.8	134.8	227.2	211.32
TRINITY_Contig18663	sp_B9EXZ6_BAT1_ORYSJ_Amino-acid_permease_BAT1_homolog	2.163	4.177	29.94	15.66	11.591	17.855	45.26	40.36	70.23	70.39	68.79	68.639
TRINITY_Contig18746	sp_A5H8G4_PER1_MAIZE_Peroxidase_1	8.386	14.67	202	123.5	132.3	50.606	23.79	26.45	80.51	58.24	74.96	83.777
TRINITY_Contig18784	-	8.958	5.567	53.25	41.91	25.874	31.327	20.01	18.51	47.4	53.17	56.01	54.336
TRINITY_Contig19061	sp_C5Y376_CSPLB_SORBI_CASP-like_protein_1U2	59.66	36.17	1.665	0.849	0.368	0.501	21.51	20.77	35.29	35.28	105.7	86.072
TRINITY_Contig19131	sp_P38560_GLNA2_MAIZE_Glutamine_synthetase_root_isozyme_2	0.52	1.665	22.8	46.6	14.267	36.672	9.238	11.36	43.14	26.8	52.58	48.7
TRINITY_Contig19851	sp_Q9FG13_CXE15_ARATH_Probable_carboxylesterase_15	1.671	1.578	64.36	28.01	9.818	11.107	29.93	31.6	53.46	50.26	59.65	65.832
TRINITY_Contig2477	sp_Q12PE6_TOLB_SHEDO_Tol-Pal_system_protein_TolB	5.732	18.47	577.2	333	173.54	152.58	134.2	129.4	218.5	194.8	306.4	309.15
TRINITY_Contig2587	sp_Q2QNE8_MT4B_ORYSJ_Metallothionein-like_protein_4B	22.12	96.62	8868	1714	1394.6	889.15	150.8	160.3	251.3	227.6	519.1	451.03

TRINITY_Contig2852	sp_Q7F2L3_NAC48_ORYSJ_NAC_domain-containing_protein_48	12.95	18.44	314.1	119.4	66.115	66.635	34.82	33.22	56.26	54.25	61.06	61.521
TRINITY_Contig3297	sp_Q9FT92_Y5843_ARATH_Uncharacterized_protein_At5g08430	3.753	3.366	63.56	18.77	17.127	19.218	14.7	15.31	25.16	22.24	30.88	29.597
TRINITY_Contig6665	sp_P14219_ADH1_PENAM_Alcohol_dehydrogenase_1	15.56	11.66	78.2	115.1	75.833	37.704	131.9	120.7	37.92	46.56	31.42	30.087
TRINITY_Contig6714	sp_Q9SMT7_4CLLA_ARATH_Oxalate--CoA_ligase	11.83	6.298	46.21	49.86	46.429	55.636	106.4	102.3	170.8	150.4	208.5	203.59
TRINITY_Contig898	-	0.088	1.433	220.2	65.75	10.219	6.909	25.3	29.6	55.65	39.88	44.82	43.064
TRINITY_DN12117_c0_g1_i4	sp_O82089_CCH_ARATH_Copper_transport_protein_CCH	19.33	55.39	721.5	235.6	136.16	187.95	204.9	207.8	101.7	93.14	75.03	65.832
TRINITY_DN13722_c5_g5_i2	sp_Q9LPQ3_M2K7_ARATH_Mitogen-activated_protein_kinase_kinase_7	47.96	29.09	1.013	5.853	0.418	4.991	39.56	42.29	19.46	25.64	17.6	23.593
TRINITY_DN13759_c0_g1_i6	sp_Q9ZVJ5_SGGP_ARATH_Haloacid_dehaloge	2.61	3.019	181.1	89.6	27.145	28.893	8.921	8.525	4.062	3.407	4.627	4.088
TRINITY_DN14299_c0_g1_i1	sp_O48604_HPPD_HORVU_4-hydroxyphenylpyruvate_dioxygenase	46.14	29.5	482.8	269.4	325.63	563.31	62.53	66.91	88.85	130.5	88.33	104.76
TRINITY_DN16117_c5_g2_i4	sp_Q0DBL6_HFC2B_ORYSJ_Heat_stress_transcription_factor_C-2b	0.154	0.434	180.3	25.94	13.113	23.855	9.675	7.771	15.49	22.66	21.21	26.255
TRINITY_DN16313_c0_g4_i1	sp_P01053_ICI2_HORVU_Subtilisin-chymotrypsin_inhibitor-2A	17.33	25.45	272.5	173.9	90.233	59.865	206.8	194.9	337	293	304.4	300.86
TRINITY_DN16831_c1_g5_i2	sp_A5H8G4_PER1_MAIZE_Peroxidase_1	51.59	78.5	624.9	493	634.76	281.02	299.1	305.2	561.1	674.9	949.6	980.23
TRINITY_DN17631_c1_g2_i3	sp_P19656_NLTP_MAIZE_Non-specific_lipid-transfer_protein	73.69	56.92	1699	1049	459.39	657.73	671.8	625	363.6	311.6	267.4	243.08
TRINITY_DN18135_c0_g3_i7	sp_B3SGL0_BCH_GENLU_Beta-carotene_3-hydroxylase,_chloroplastic	2.17	0.709	60.59	31.4	27.647	18.717	14.38	10.53	22.54	31.62	27.55	26.7
TRINITY_DN19209_c0_g2_i2	sp_Q9LXI7_PPA20_ARATH_Probable_purple_acid_phosphatase_20	1.217	2.331	132.2	21.61	16.257	19.357	13.89	15.65	24.4	22.73	26.49	25.52
TRINITY_DN19858_c2_g2_i5	sp_Q4W8D0_GLN13_ORYSJ_Glutamine_synthetase_cytosolic_isozyme_1-3	1.319	0.927	12.23	20.37	10.236	22.114	9.324	8.313	42.73	32.49	53.84	52.008
TRINITY_DN20439_c0_g4_i3	sp_Q9XIH7_PLT1_ARATH_Putative_polyol_transporter_1	0.418	1.426	40.96	16.43	33.033	13.503	23.51	21.73	37.74	52.27	57.06	61.956
TRINITY_DN21253_c1_g2_i12	sp_Q6Z4N4_40G3_ORYSJ_Ricin_B-like_lectin_R40G3	13.75	32.37	570.1	404.7	108.66	78.498	32.63	32.01	16.71	17.62	9.028	10.849

Supplementary table 3. Primer sequences used for qRT PCR.

Sl. No.	GENE	Unigene ID	Forward	Reverse
1	EF 1	TRINITY_BIN_256_14	GCTGACTGTGCTGTCCTTAT	GATCATCTGCTTCACTCCAAGA
2	UB 1	TRINITY_2200_length_3617_cov_29.580327_g1404_i0	AACACGGTCTGGTCCTACTA	CAAGACCACCGAGCAAACTA
3	lm wrky1	TRINITY_DN13115_c0_g4	AGGCTAGCAGCTAATTGTATGG	CTCTTCAGCAGCACCACTT
4	lm wrky2	TRINITY_2900_length_3356_cov_26.337085_g1863	TACTCGCGCAATGGTTCTT	ATCAGTCTCTACCCTGCCTATAA
5	lm wrky3	TRINITY_15397_length_1864_cov_26.900836_g9656	TTCGTCTCCAGAGGTTGAATG	GTGTTGCTGTTGATTGCTTCT
6	lm wrky4	TRINITY_29420_length_1293_cov_35.348856_g10026	TCTGGTTCCTGTATCATTGTATGG	ACAACAAACCTCTGGACTCTG
7	lm wrky5	TRINITY_47681_length_854_cov_27.751592_g32459	CCCTTGATAAGTGAGCAGCA	CTCGTCGTGGAAGTGGAAC
8	lm DEG1	TRINITY_34092_length_1159_cov_7.666055_g22087	GGTGCCTATCAGTATCAGATTCAA	GGGTTGTGGAGAAACGTAAGA
9	lm DEG2	TRINITY_42578_length_953_cov_15.270362_g28375	AAGAGCTTCCCTCGGTTTATTC	GAACAACATGATTCGAGCAACC
10	lm DEG3	TRINITY_DN13688_c1_g1	GGCAGCCCATGTGTAGAAT	ACCTGGGCATGAGAATTTAGG
11	lm DEG4	TRINITY_BIN_20091_5	CCTGTCCAACGAGGAGTTTAG	GTCACCGGTTATCCAGTCAAA
12	lm DEG5	TRINITY_Contig15518	GCACCTGCACCTACATCAA	GGCTCAATCGCTCACCTATC
13	lm DEG6	TRINITY_19238_length_1673_cov_21.231920_g12106	CTGGATCTGTCGACCAAGAAA	GCACCTGAAAGATGCTTGAATAG
14	lm DEG7	TRINITY_51168_length_790_cov_19.793343_g35401	TCTGAACATGAGGAAGGAATGAG	TGTTAGTGGATGTTTCGGTAACT
15	lm DEG8	TRINITY_DN17793_c2_g5	GGGATACTGCTTGCGTTCT	CTGCGAGGCTGGTAAGAATAG
16	lm DEG9	TRINITY_DN12427_c0_g2	CAGGTCACATGAGTTGGAAGT	TGCCAAATGCTCAACATGAATATAG
17	lm DEG10	TRINITY_BIN_15690_4	CTTGCTACAACGCTCTTTACAC	CCACGCATTCCTGAGATGAT
18	lm abc1	TRINITY_Contig1514	CCCTTCGTCAACGTGTAGATA	CACTACTTCCTCAGTTCCTCTAC
19	lm abc2	TRINITY_Contig9616	GCAAATGCTGATCCCGTAGA	CTACAGACTACCTCCTTGCTTTC
20	lm abc3	TRINITY_Contig13084	CTGACTGGTAGGTCGATCATTAC	GCTGAGATGAACCTGCTATCTT
21	lm abc4	TRINITY_DN22512_c4_g2	CCCATCCTGAGACATCATCTTC	TCACCTTCGCTGAGCAATAATA
22	lm abc5	TRINITY_DN15064_c0_g1	CTACAGACTACCTCCTTGCTTTC	GCAAATGCTGATCCCGTAGA
23	LMC1	TRINITY_Contig11942	TTGTTTGACGCCCTTGTTTG	GAATCGCTGCTGCTCTACTT
24	LMC2	TRINITY_6483_length_2616_cov_33.980762_g4098_i0	TCATAGGTGCAGGTTGCTAATG	GGCAGGAGCTTCAACAAAGA
25	LMC3	TRINITY_26921_length_1373_cov_23.520706_g17146_i0	ACAACCTGGACTGCTACAAC	GCAGCGAGCTCTGGTTATT
26	LMC4	TRINITY_DN13722_c5_g5_i2	CGCCACCTATGACAGGTTATT	GTAGGAGATCTAGGAGAGTCAGG
27	LMC5	TRINITY_Contig19061	CATTTGCCAAGGGCTTGTTG	TGCTCGAGGACGATGACTAATA
28	LMC6	TRINITY_53585_length_748_cov_32.690722_g37487_i0	AAGGAAGGCAAAGGTGAGAG	GGTTCAGCCCTTCATCAAGTA
29	LMC7	TRINITY_Contig6665	TTGCCTCTTTCCCTCACAAA	GATTCGCTTCGGTTTGGATTG
30	LMC8	TRINITY_Contig10696	CCCGTTTGTTTCGTCTTGTTT	CGCACGTTTGTTGCAGATT
31	LMC9	TRINITY_8742_length_2349_cov_23.646491_g5523_i0	CTAACCCTTGATGAGGCCATT	CCCACCATGACTCTTCTGTTT
32	LMC10	TRINITY_39367_length_1025_cov_29.965481_g25910_i0	TCATCTGAGAGAGACGGATCTT	AGGTAGTGGACAAGGTACAGA
33	LMC11	TRINITY_BIN_4317_1	GATTGGCTTGGGAGAAGGAA	TAAGGAGTCCGAGGCTAACA
34	LMC12	TRINITY_BIN_6373_5	TAGACTTGGCAGTGGCTTTC	CTGTCTAAAGATGGAGTCGAGATG
35	LMC13	TRINITY_DN13759_c0_g1_i6	CCATGAACTGCATAGCCTTCT	AGTTCTACAGCGCCAACATC
36	LMC14	TRINITY_BIN_12545_6	CTGCTAGGTTCTGCTGTATAGTT	GTGATCGATACTGGCTGAGAAC
37	LMC15	TRINITY_DN12117_c0_g1_i4	CGTCATGTTGTTGCCACTCTA	CTCCTGCAAGGGATGTGTTTAT

Supplementary Table 4. *In silico* expression matrix for DEGs containing SSR markers in Leaves (control vs drought, control vs salt)

Unigene ID	CONTROL1	CONTROL2	PEG_TREATED_LEAVES1	PEG_TREATED_LEAVES2	SALT_TREATED_LEAVES1	SALT_TREATED_LEAVES2
TRINITY_Contig10832	42.656	35.493	2.171	6.718	7.326	11.508
TRINITY_BIN_4886_7	16.193	20.631	1.466	1.439	12.327	60.889
TRINITY_Contig7516	20.767	59.91	3.709	5.546	28.717	74.207
TRINITY_DN20129_c1_g1_i1	9.046	14.616	0.507	1.617	2.224	3.651
TRINITY_9543_length_2274_cov_27.739229_g5996_i0	29.579	25.858	0.941	6.904	1.873	8.265
TRINITY_4828_length_2893_cov_30.035765_g3070_i0	5.842	8.586	0	1.035	0.284	1.841
TRINITY_BIN_3238_3	1.29	2.802	237.231	118.7	28.416	10.268
TRINITY_DN21081_c2_g2_i3	9.786	8.6	0	0.137	0	0.531
TRINITY_47734_length_853_cov_25.942602_g32504_i0	1.503	1.397	425.431	182.045	9.299	3.358
TRINITY_Contig13523	1.488	7.76	764.976	379.361	329.271	701.833
TRINITY_Contig9143	7.55	7.087	0	1.051	0.401	1.471
TRINITY_DN17363_c1_g2_i1	26.192	15.064	2.298	3.347	8.045	10.468
TRINITY_BIN_9764_3	49.913	93.818	2.841	13.704	3.83	25.796
TRINITY_23221_length_1502_cov_27.458479_g14712_i0	5.102	12.668	0.271	0.986	0	1.148
TRINITY_DN17266_c1_g2_i4	140.849	96.649	5.102	11.901	16.474	41.471
TRINITY_DN16027_c0_g1_i1	0.323	0.203	34.774	90.194	7.945	0.632
TRINITY_Contig2888	53.198	43.442	5.482	11.391	13.966	14.481
TRINITY_BIN_1602_10	9.896	6.899	0.832	1.091	0.702	1.171
TRINITY_DN20690_c3_g1_i1	127.61	55.328	7.418	8.562	10.219	11.215
TRINITY_1533_length_3973_cov_27.862705_g941_i1	24.095	10.316	0	1.407	1.305	2.842
TRINITY_Contig10698	6.553	7.087	0	0.808	0.502	0.747
TRINITY_BIN_5115_6	162.943	136.796	16.374	42.17	31.36	97.615
TRINITY_DN14317_c1_g1_i1	122.669	51.47	0.326	1.488	1.104	5.091
TRINITY_DN22005_c2_g1_i1	22.167	30.534	1.809	2.991	8.814	8.226
TRINITY_Contig15352	4.435	16.071	0.615	0.574	2.442	7.487
TRINITY_DN13227_c0_g1_i16	0.704	0.203	165.856	64.832	3.312	2.149
TRINITY_35304_length_1125_cov_27.677083_g22970_i0	1.422	2.744	394.095	169.052	140.259	234.738
TRINITY_DN11567_c0_g1_i1	0.095	1.404	277.867	77.808	15.371	32.382
TRINITY_DN19204_c1_g2_i4	0	0.398	397.695	119.331	9.115	0.955
TRINITY_DN18728_c0_g5_i4	7.58	14.768	129.67	154.249	225.14	504.576
TRINITY_DN11954_c3_g2_i2	318.995	330.485	48.724	86.45	59.676	146.404
TRINITY_16880_length_1784_cov_31.650729_g10598_i0	8.071	5.574	0.38	0.784	0.636	0.47
TRINITY_29819_length_1281_cov_29.047030_g17545_i1	0.066	0.217	201.046	24.206	1.974	0.139
TRINITY_BIN_6546_2	21.566	26.278	2.877	3.727	11.507	15.39
TRINITY_DN17626_c0_g2_i1	12.77	22.065	1.791	1.18	0	4.991
TRINITY_55328_length_720_cov_22.572965_g39043_i0	13.451	7.731	0.615	0	0	1.702
TRINITY_Contig12714	5.652	16.063	0.217	0	1.606	1.918
TRINITY_DN15786_c2_g2_i2	16.208	14.138	1.447	3.274	0.652	1.094
TRINITY_DN17363_c1_g2_i11	8.577	3.315	0.651	0.671	1.606	1.856
TRINITY_6483_length_2616_cov_33.980762_g4098_i0	20.65	20.943	0.543	4.358	1.037	7.256
TRINITY_Contig11942	82.417	72.173	3.6	3.525	3.596	3.697
TRINITY_DN18700_c0_g1_i10	0	0.101	25.239	23.43	33.551	45.33
TRINITY_Contig16328	0.469	0.478	157.334	50.465	1.84	1.479
TRINITY_Contig9689	0	1.122	540.103	32.646	54.826	31.989
TRINITY_DN13722_c5_g5_i2	47.956	29.087	1.013	5.853	0.418	4.991
TRINITY_DN12706_c0_g1_i2	70.49	57.536	2.822	12.313	10.754	34.084
TRINITY_DN22383_c0_g1_i1	87.607	27.067	0.905	8.877	19.401	8.519
TRINITY_Contig17766	7.448	5.335	0.416	0.59	1.388	2.781
TRINITY_Contig17597	0.169	0.601	78.595	53.02	24.084	75.994
TRINITY_Contig8667	28.728	47.561	0.633	1.9	2.007	24.094
TRINITY_Contig6883	0.117	1.651	126.902	88.366	53.856	70.579
TRINITY_DN22598_c6_g1_i6	47.011	59.621	2.587	11.472	16.29	22.176
TRINITY_Contig9243	1.158	1.318	206.257	63.239	20.154	26.913
TRINITY_BIN_4564_7	2.382	3.207	90.192	312.169	132.766	85.399
TRINITY_Contig12789	1.078	0.804	104.123	39.664	0.518	0.801
TRINITY_DN16486_c0_g3_i3	146.09	65.151	0.959	6.727	16.826	20.304
TRINITY_Contig13158	9.105	10.222	0.814	1.374	2.793	4.236

TRINITY_DN16117_c5_g2_i4	0.154	0.434	180.33	25.944	13.113	23.855
TRINITY_DN17534_c0_g2_i11	0.44	0.695	37.235	19.541	27.597	33.368
TRINITY_BIN_15103_2	0.433	0.514	161.043	48.695	11.724	14.096
TRINITY_4942_length_2873_cov_26.481455_g3145_i0	2.962	7.652	0.326	0.331	0.853	1.101
TRINITY_DN18037_c0_g1_i23	0	0.471	13.877	3.25	21.191	19.11
TRINITY_BIN_9932_5	11.318	16.512	0.923	3.113	2.676	5.515
TRINITY_Contig10204	27.262	11.192	0.615	0.323	0	0
TRINITY_Contig20107	0.447	2.027	174.938	62.107	31.042	36.233
TRINITY_Contig3189	0.176	1.086	630.62	691.231	111.976	253.255
TRINITY_BIN_1703_7	2350.625	1916.539	160.952	486.638	249.375	503.845
TRINITY_BIN_11843_2	24.139	19.821	1.466	4.188	3.295	1.04
TRINITY_Contig17905	13.276	22.644	0.181	1.892	2.843	9.043
TRINITY_36657_length_1090_cov_26.702253_g1902_i2	0.777	2.497	145.501	56.359	71.517	74.176
TRINITY_BIN_1107_10	86.918	104.865	8.902	24.295	38.92	108.823
TRINITY_37307_length_1074_cov_24.689552_g24415_i0	3.599	8.006	0	0.267	1.957	4.367
TRINITY_Contig20078	13.166	13.038	1.701	3.145	1.254	3.767
TRINITY_13929_length_1946_cov_17.416622_g8719_i0	0.117	0.565	122.035	294.124	57.569	6.686
TRINITY_Contig17757	23.758	13.262	0.941	3.331	6.406	10.383
TRINITY_8846_length_2340_cov_24.682078_g5586_i0	5.241	4.575	0.543	0.21	0	1.433
TRINITY_9542_length_2274_cov_29.067574_g5995_i0	6.062	4.553	0.145	0.146	0.636	2.257
TRINITY_8636_length_2358_cov_28.701616_g5459_i0	12.711	9.881	0.145	0.17	0.151	0.37
TRINITY_BIN_2309_14	0.535	0.637	287.692	111.562	11.524	7.533
TRINITY_Contig10665	88.663	71.732	6.26	14.561	2.559	4.837
TRINITY_8847_length_2340_cov_23.405108_g5587_i0	0	0.608	390.494	53.012	16.608	5.207
TRINITY_Contig360	7.243	4.865	0.308	1.003	0.167	0.67
TRINITY_DN19356_c0_g2_i2	3.445	7.471	1452.824	272.917	37.18	26.975
TRINITY_21197_length_1586_cov_38.420567_g13360_i0	1.635	5.965	14.637	33.649	106.406	82.657
TRINITY_BIN_3639_11	5.71	8.665	0.489	0.825	2.258	2.064
TRINITY_46902_length_869_cov_27.276250_g31811_i0	8.1	4.988	0	0.72	1.054	1.756
TRINITY_DN22308_c1_g5_i10	32.306	72.036	3.401	3.444	43.185	146.419
TRINITY_BIN_13823_3	14.412	7.623	0.597	0.768	0.284	5.199
TRINITY_DN19722_c1_g1_i7	3.643	6.262	31.825	32.598	100.034	86.231
TRINITY_DN20137_c1_g2_i3	2.031	1.194	299.687	134.175	6.657	8.434
TRINITY_4103_length_3034_cov_28.572344_g2612_i0	17.527	15.216	1.429	4.536	7.827	7.841
TRINITY_Contig982	5.879	10.996	0.235	1.245	2.726	11.824
TRINITY_DN22120_c2_g3_i9	2.844	7.073	0.109	0.428	0.602	2.588
TRINITY_DN14937_c0_g3_i3	0.081	0.101	38.121	18.078	0	0.169
TRINITY_33143_length_1184_cov_24.504036_g21408_i0	8.152	2.881	0	0.307	0	0.193
TRINITY_11732_length_2093_cov_33.770257_g7338_i0	35.26	15.267	0.778	2.902	5.185	8.843
TRINITY_21652_length_1568_cov_26.823883_g13659_i0	67.06	68.995	0.868	0.275	0	0.216
TRINITY_26921_length_1373_cov_23.520706_g17146_i0	46.901	23.78	0	0.323	0.569	0.062
TRINITY_Contig816	13.965	15.13	0.525	2.142	1.455	2.334
TRINITY_DN21819_c0_g1_i5	0	0.42	216.407	84.575	87.724	0.562
TRINITY_DN16037_c0_g1_i3	131.693	185.421	5.247	21.093	3.864	47.433
TRINITY_Contig14472	13.034	8.73	1.194	2.611	5.218	3.713
TRINITY_DN11762_c0_g1_i1	60.785	53.243	6.767	5.029	4.7	4.999
TRINITY_16946_length_1781_cov_26.710864_g10641_i0	14.353	21.645	1.683	0.873	4.399	6.609
TRINITY_DN20690_c3_g1_i2	130.732	61.612	2.171	8.651	25.924	7.402
TRINITY_BIN_3704_2	6.392	5.263	0.941	0.784	4.114	3.882
TRINITY_Contig12895	26.346	24.779	1.719	2.87	3.579	5.685
TRINITY_51831_length_778_cov_27.468265_g35969_i0	1.554	0.319	239.999	369.627	128.283	22.854
TRINITY_Contig10267	22.277	23.541	1.176	5.247	2.375	7.834
TRINITY_Contig4915	45.523	60.859	3.275	11.893	8.145	28.23
TRINITY_5299_length_2804_cov_29.469835_g3364_i0	8.247	22.513	0	0.032	0	0
TRINITY_7546_length_2480_cov_25.817503_g4782_i0	429.18	288.281	16.41	37.441	53.187	133.464
TRINITY_BIN_6091_7	8.379	8.238	0	0.234	0.953	3.027
TRINITY_Contig8430	11.897	11.879	0.905	1.221	0	0.393
TRINITY_DN18700_c0_g1_i3	0	0.101	10.476	14.658	16.19	26.62
TRINITY_DN16990_c4_g1_i6	3.79	5.748	0	0.081	0	0.154
TRINITY_BIN_15727_1	10.695	23.773	1.827	1.698	1.589	1.548
TRINITY_Contig13103	0.169	0.217	26.705	88.819	17.562	5.738
TRINITY_27499_length_1353_cov_29.495327_g17524_i0	891.816	793.719	55.128	173.628	46.613	155.37
TRINITY_Contig8093	78.063	40.756	0.633	7.066	19.702	8.696

TRINITY_BIN_6636_2	41.351	50.956	4.686	2.571	7.911	13.51
TRINITY_BIN_2441_5	49.928	47.908	6.007	13.267	15.237	26.382
TRINITY_Contig9585	96.939	261.699	14.547	40.254	57.334	255.389
TRINITY_DN21886_c1_g1_i4	6.575	7.065	0	0.348	0.903	1.448
TRINITY_Contig5428	21.977	24.077	0.76	0.978	0.217	4.806
TRINITY_Contig10478	0	0.68	28.406	30.908	86.972	99.002
TRINITY_DN18155_c1_g2_i3	8.144	7.985	0.796	1.795	0	2.087
TRINITY_17532_length_1753_cov_36.917458_g10813_i1	64.267	73.534	3.709	16.792	15.605	42.334
TRINITY_BIN_1943_8	13.598	10.345	0.308	1.496	2.342	4.106
TRINITY_Contig9754	5.007	4.655	0.181	0.679	0.786	1.879
TRINITY_39367_length_1025_cov_29.965481_g25910_i0	25.085	34.103	3437.862	1706.806	511.193	889.323
TRINITY_DN22137_c1_g1_i1	47.04	75.359	0.941	3.501	5.018	38.19
TRINITY_Contig11363	15.086	5.646	479.257	568.181	321.611	199.991
TRINITY_4969_length_2868_cov_24.511969_g2709_i1	12.711	6.769	1.429	1.091	0.368	0.67
TRINITY_11940_length_2078_cov_32.831757_g7461_i0	218.779	154.735	25.185	34.934	109.049	168.079
TRINITY_11479_length_2112_cov_30.728830_g7176_i0	7.316	4.01	0.47	0.631	0	0.693
TRINITY_Contig14114	38.947	19.35	0.434	2.636	2.96	4.391
TRINITY_Contig9017	6.319	7.608	1.104	1.132	3.362	1.618
TRINITY_14818_length_1896_cov_27.142310_g9295_i0	6.429	7.319	0.706	1.334	1.555	3.782
TRINITY_DN22191_c2_g1_i2	0	0	4.342	6.282	32.899	43.142
TRINITY_860_length_4595_cov_28.621741_g550_i0	5.227	6.776	0.724	1.229	4.031	5.338
TRINITY_BIN_1709_4	13.884	8.122	0.253	1.795	1.104	4.321
TRINITY_DN18660_c1_g2_i14	13.972	6.892	0.688	1.415	1.84	2.58
TRINITY_BIN_4326_1	6.143	6.001	0	0.194	0	0
TRINITY_49153_length_826_cov_25.743725_g33679_i0	14.207	6.305	0	0.768	0	0
TRINITY_6426_length_2624_cov_32.417613_g4064_i0	2.595	3.381	666.57	140.19	37.331	16.907
TRINITY_Contig12983	11.245	13.117	0	0.412	2.074	0.632
TRINITY_BIN_7705_7	31.99	8.962	1.556	1.18	0	0.709
TRINITY_DN17266_c1_g2_i7	24.066	31.063	1.773	5.595	5.185	4.976
TRINITY_DN20418_c0_g1_i7	17.212	10.583	0.47	0.97	1.02	2.195
TRINITY_12044_length_2070_cov_32.029985_g6712_i1	3.445	7.427	0	0.049	0.385	0.67
TRINITY_BIN_2817_1	0	0.594	73.999	111.327	43.77	3.536
TRINITY_Contig17915	79.324	54.908	5.12	16.776	26.158	52.902
TRINITY_BIN_97_5	8.283	8.622	0.525	1.318	4.499	13.865
TRINITY_Contig13133	6.018	5.531	0	0.841	1.154	2.234
TRINITY_7917_length_2435_cov_29.959425_g5008_i0	7.389	13.095	1.447	1.18	0.284	4.799
TRINITY_BIN_8105_8	4.684	8.868	0.362	0.364	2.04	5.823
TRINITY_Contig1134	205.526	128.471	13.823	44.304	64.61	101.536
TRINITY_DN19913_c0_g1_i7	17.329	1.918	0.163	0	0	0
TRINITY_DN17322_c1_g2_i6	0.073	0.927	150.857	246.391	12.343	9.259
TRINITY_BIN_3347_9	3.768	4.814	0	0.598	1.438	3.566
TRINITY_BIN_15544_2	4.376	25.909	0.561	0.073	0	4.498
TRINITY_DN15946_c1_g2_i4	0.059	0	68.318	24.222	10.788	7.264
TRINITY_DN15464_c2_g1_i5	0	0	265.094	314.877	81.686	107.937
TRINITY_Contig9385	155.656	109.94	19.196	29.598	64.51	88.773
TRINITY_DN20951_c1_g4_i1	0.433	0.492	103.327	29.024	4.783	2.295
TRINITY_Contig15369	32.789	16.918	1.683	2.393	4.934	0.855
TRINITY_Contig8669	18.282	17.497	1.502	4.487	2.124	5.785
TRINITY_Contig9647	5.505	7.246	1124.586	646.53	182.774	275.046
TRINITY_DN21756_c2_g1_i15	20.247	7.319	1.14	1.253	2.325	3.066
TRINITY_22226_length_1542_cov_31.294637_g2198_i1	38.163	25.054	0.887	2.151	0.284	0.539
TRINITY_Contig493	51.196	33.589	6.188	9.37	7.677	5.839
TRINITY_DN19996_c0_g1_i2	7.301	11.763	0.163	1.302	3.529	17.115
TRINITY_Contig6991	79.287	56.001	9.137	16.897	18.197	20.712
TRINITY_BIN_2887_1	38.111	63.964	0.163	1.787	0.385	0.493
TRINITY_Contig20011	41.08	85.5	9.969	11.351	16.09	11.2
TRINITY_Contig15543	5.183	9.997	0.181	0	0	2.858
TRINITY_18953_length_1685_cov_24.115099_g1094_i2	0.396	0	7.165	35.5	25.673	17.593
TRINITY_36487_length_1094_cov_29.361951_g10636_i1	67.932	45.157	5.102	15.07	13.932	38.274
TRINITY_16231_length_1820_cov_32.281553_g6995_i1	71.876	43.405	6.061	6.88	3.379	26.636
TRINITY_Contig18731	13.166	19.321	1.447	2.749	2.275	3.505
TRINITY_DN17564_c0_g1_i12	11.311	13.088	0.416	1.536	1.539	2.003
TRINITY_DN13918_c0_g8_i1	2.104	3.75	461.924	197.931	257.955	160.03

TRINITY_DN18037_c0_g1_i1	0.029	0.152	26.922	9.75	18.95	22.954
TRINITY_32198_length_1210_cov_30.662577_g20743_i0	10.871	5.118	0.597	0.154	1.004	0
TRINITY_BIN_15641_2	220.495	137.817	5.41	35.896	86.57	80.916
TRINITY_Contig14673	254.376	215.058	7.49	10.631	9.065	5.03
TRINITY_15397_length_1864_cov_26.900836_g9656_i0	189.428	86.13	17.079	15.798	18.063	7.325
TRINITY_2046_length_3684_cov_29.339419_g1306_i0	8.342	9.606	0.434	1.698	1.187	2.75
TRINITY_BIN_198_5	7.998	3.735	0.362	0.428	1.589	0.755
TRINITY_DN18234_c2_g1_i6	8.833	13.001	1.013	0.526	0.485	4.776
TRINITY_DN17417_c1_g3_i2	56.746	49.768	8.359	13.857	15.153	33.907
TRINITY_BIN_478_8	96.14	121.348	2.28	23.203	25.79	25.896
TRINITY_Contig15483	12.161	9.628	1.429	1.568	9.048	6.57
TRINITY_DN22582_c1_g2_i2	0	0	114.87	22.322	1.121	0.585
TRINITY_Contig17091	22.959	21.703	1.646	1.9	0.234	3.035
TRINITY_Contig16403	4.977	7.891	0.398	0.881	0.151	0.454
TRINITY_48787_length_833_cov_20.123037_g33371_i0	10.915	4.271	0.489	0	4.048	7.818
TRINITY_DN15708_c0_g1_i1	1.283	10.75	1290.044	691.595	170.632	123.042
TRINITY_Contig18732	25.151	19.849	247.562	66.796	5.887	6.247
TRINITY_Contig19436	231.952	119.966	13.298	40.877	55.244	89.805
TRINITY_Contig17465	8.188	18.626	1.393	0.501	0.719	3.066

Supplementary Table 4. *In silico* expression matrix for DEGs containing SSR markers in Roots (control vs drought, control vs salt)

Unigene ID	Control_1	Control_2	PEG_TREATED_ROOT1	PEG_TREATED_ROOT2	SALT_TREATED_ROOT1	SALT_TREATED_ROOT2
TRINITY_Contig4093	0	0	0	0	7.126	7.062
TRINITY_16882_length_1784_cov_29.045481_g10600_i0	0	0	0	0	24.71	19.014
TRINITY_Contig4609	0	0	0	0	5.145	3.999
TRINITY_Contig3993	0	0	0	0	5.471	8.51
TRINITY_29805_length_1282_cov_9.583677_g19103_i0	0	0	0	0	6.732	5.648
TRINITY_DN20533_c0_g1_i6	0	0	0	1.16	3.906	2.306
TRINITY_Contig11121	0	0	0	0	134.638	104.841
TRINITY_Contig928	1.39	1.702	0	0	4.683	3.253
TRINITY_Contig3970	0	0	0	0	8.072	8.655
TRINITY_22503_length_1531_cov_8.959644_g14247_i0	0	0	0	0	7.936	5.057
TRINITY_44102_length_923_cov_32.492974_g29548_i0	0	0	0	0	42.26	34.431
TRINITY_31785_length_1222_cov_16.156982_g20461_i0	0	0	0	0	9.355	12.175
TRINITY_BIN_209_9	0	0	0	0	2.116	5.124
TRINITY_8361_length_2386_cov_3.722486_g5290_i0	0	0	0	0	3.512	3.241
TRINITY_Contig1725	0	0	0	0	20.263	9.546
TRINITY_39095_length_1032_cov_14.651090_g25702_i0	0	0	0	0	9.265	5.792
TRINITY_32628_length_1199_cov_6.784956_g21043_i0	0	0	0	0	5.133	4.322
TRINITY_Contig3460	0	0	0	0	14.162	14.815
TRINITY_BIN_36566_1	0	0	0	0	3.332	3.286
TRINITY_DN16440_c2_g1_i3	4.958	3.386	5.313	1.667	0	0
TRINITY_DN13187_c0_g1_i1	0	0	0	0	5.392	6.004
TRINITY_43050_length_944_cov_30.869714_g28743_i0	0	0	0.202	0	45.243	39.343
TRINITY_12553_length_2036_cov_6.735638_g7850_i0	0	0.059	0	0	4.345	3.899
TRINITY_Contig5765	0.197	0.313	0	0	229.908	201.907
TRINITY_39457_length_1023_cov_26.554507_g25971_i0	0	0	0	0	16.74	19.805
TRINITY_Contig248	0	0	0	0	7.148	5.547
TRINITY_Contig10684	0	0	0	0	13.903	12.821
TRINITY_24771_length_1446_cov_32.411038_g15738_i0	0	0	0	0.114	29.213	22.523
TRINITY_Contig10184	0	0	0.18	0	8.105	6.405
TRINITY_31178_length_1239_cov_6.241880_g20042_i0	0	0	0	0	4.694	4.957
TRINITY_Contig6894	0	0	0	0	27.434	20.184
TRINITY_28003_length_1338_cov_7.430260_g17865_i0	0	0	0	0	4.818	4.667
TRINITY_21957_length_1555_cov_10.988560_g13869_i0	0	0	0	0	8.533	5.748
TRINITY_BIN_21114_1	0	0	0	0	14.511	11.273
TRINITY_Contig1854	0	0	0	0	519.436	452.703
TRINITY_Contig869	0	0	0	0	7.047	7.441
TRINITY_BIN_11939_5	8.792	8.339	0	0.342	0	0
TRINITY_Contig6727	0	0	0	0	61.015	51.763
TRINITY_DN15771_c1_g3_i4	0	0	0	0	319.1	286.252
TRINITY_38349_length_1049_cov_8.856122_g25132_i0	0	0	0	0	6.721	5.57
TRINITY_DN18519_c0_g5_i1	0	0	4.783	3.314	1.081	0
TRINITY_Contig9819	0	0	0	0	5.426	4.545
TRINITY_BIN_32975_1	0	0	0	0	5.933	4.099
TRINITY_16900_length_1784_cov_6.367930_g10611_i0	0	0	0	0	4.559	3.72
TRINITY_17608_length_1750_cov_6.378941_g11061_i0	0	0	0	0	3.017	5.436
TRINITY_Contig1198	0	0.254	0.318	0	334.849	280.07
TRINITY_Contig10927	0	0	0	0	44.275	43.71
TRINITY_11865_length_2085_cov_10.147817_g7421_i0	0	0	0	0	3.287	3.141
TRINITY_Contig12105	3.131	1.659	4.645	3.49	0	0
TRINITY_Contig11780	6.476	5.418	0.859	3.045	0	0
TRINITY_20263_length_1626_cov_8.107258_g12757_i0	0	0	0	0	5.854	5.001
TRINITY_Contig4563	0	0	0	0	5.629	3.197
TRINITY_10693_length_2177_cov_4.111006_g6703_i0	0	0	0	0	2.645	3.676
TRINITY_Contig9790	0	0	0	0	8.769	7.318
TRINITY_7910_length_2436_cov_27.240811_g5005_i0	6.15	7.094	0.583	0.849	0	0

TRINITY_Contig10452	0	0	0	0	7.824	5.803
TRINITY_32725_length_1196_cov_12.614020_g21114_i0	0	0	0	0.145	4.413	4.89
TRINITY_17092_length_1774_cov_8.583578_g10735_i0	0	0	0	0	5.246	4.489
TRINITY_6644_length_2595_cov_7.356690_g4198_i0	0	0	0	0	2.814	4.021
TRINITY_Contig1625	0	0	0	0	18.518	11.484
TRINITY_Contig9816	0	0	0	0	12.642	9.747
TRINITY_Contig798	0	0	0	0	4.12	3.776
TRINITY_DN15174_c1_g1_i1	0	0	0	0	40.74	34.798
TRINITY_DN18572_c1_g1_i1	8.363	6.849	0	0	0	0.423
TRINITY_60365_length_643_cov_32.642857_g43740_i0	0	0	0	0	50.309	30.8
TRINITY_BIN_4207_5	1.124	1.101	1.188	0	0	0
TRINITY_Contig7212	0	0	0	0	3.073	4.723
TRINITY_Contig5876	0	0	0	0	43.116	35.289
TRINITY_Contig17264	0.523	0	5.536	2.858	0	0
TRINITY_28977_length_1308_cov_16.570621_g18529_i0	0	0	0	0	10.672	9.858
TRINITY_62084_length_619_cov_34.760000_g45366_i0	0	0	0	0	23.044	29.496
TRINITY_71255_length_514_cov_46.017978_g54301_i0	0	0	0	0	528.565	425.591
TRINITY_51040_length_792_cov_26.226833_g35292_i0	0	0	0	0	38.613	34.386
TRINITY_Contig9774	0	0	0	0	10.571	9.056
TRINITY_Contig10416	0	0	0	0.114	11.584	9.802
TRINITY_Contig9005	0	0	0	0	4.706	3.832
TRINITY_Contig969	0	0	0	0	6.957	3.743
TRINITY_15167_length_1878_cov_6.398563_g9522_i0	0	0	0	0	4.537	4.901
TRINITY_Contig9627	0	0	0	0	7.193	8.031
TRINITY_Contig5505	0	0	0	0	11.708	9.39
TRINITY_Contig14036	0	0	0	0	9.096	9.234

Supplementary table 5.
Primer sequences for SSRs identified in the transcriptome of Little millet

Unigene ID	Number o	SSR typ	SSR	size	start	end	FORWARD PRIMER1 (5'-3')	Tm(°C)	REVERSE PRIMER1 (5'-3')	Tm(°C)	PRODUCT1 size (bp)
TRINITY_Contig1	1	p4	(ATTG)3	12	2614	2625	TCACGACGTTCTGAACCCAG	59.968	ACTGCAAGACTGACAGGTCG	59.967	253
TRINITY_Contig1	2	p4	(AATA)3	12	5365	5376	TCACGACGTTCTGAACCCAG	59.968	ACTGCAAGACTGACAGGTCG	59.967	253
TRINITY_Contig1	3	p4	(ATCC)3	12	12135	12146	TCACGACGTTCTGAACCCAG	59.968	ACTGCAAGACTGACAGGTCG	59.967	253
TRINITY_Contig1	4	p4	(TCGT)3	12	15614	15625	TCACGACGTTCTGAACCCAG	59.968	ACTGCAAGACTGACAGGTCG	59.967	253
TRINITY_Contig2	1	p3	(CGC)5	15	189	203	CCTACGGGACCTCTTCCTCA	60.033	GCAACAAGTGGGTGCTGAAG	59.969	174
TRINITY_Contig2	2	p3	(CAG)9	27	502	528	CCTACGGGACCTCTTCCTCA	60.033	GCAACAAGTGGGTGCTGAAG	59.969	174
TRINITY_Contig3	1	p3	(GGC)4	12	409	420	GCTTCCGCGCTTTCACATAG	59.973	ATATTTGGTGGGCGTCTGCA	60.035	214
TRINITY_Contig3	2	p3	(CCG)4	12	7578	7589	GCTTCCGCGCTTTCACATAG	59.973	ATATTTGGTGGGCGTCTGCA	60.035	214
TRINITY_Contig4	1	p4	(CATT)3	12	2349	2360	GAGTACGCGCGCTACAAAAG	59.977	GCCGCCTAGCTCGTTATTCT	59.968	243
TRINITY_Contig4	2	p4	(TCGC)3	12	6837	6848	GAGTACGCGCGCTACAAAAG	59.977	GCCGCCTAGCTCGTTATTCT	59.968	243
TRINITY_Contig6	1	p3	(CGC)4	12	238	249	CCTGGGTCCATTGCACTGAT	60.034	CTCGAGAAGCTAGACCTGCG	59.971	202
TRINITY_Contig6	2	p3	(CGG)5	15	1564	1578	CCTGGGTCCATTGCACTGAT	60.034	CTCGAGAAGCTAGACCTGCG	59.971	202
TRINITY_Contig6	3	p3	(GGA)4	12	1933	1944	CCTGGGTCCATTGCACTGAT	60.034	CTCGAGAAGCTAGACCTGCG	59.971	202
TRINITY_Contig6	4	c	(GGA)4(GGC)5	27	2659	2685	CCTGGGTCCATTGCACTGAT	60.034	CTCGAGAAGCTAGACCTGCG	59.971	202
TRINITY_Contig6	5	p3	(CGC)5	15	2983	2997	CCTGGGTCCATTGCACTGAT	60.034	CTCGAGAAGCTAGACCTGCG	59.971	202
TRINITY_Contig7	1	p4	(AACC)3	12	1225	1236	TTCACCGTGTCTTCCACTCG	59.968	TCACGTTGAGCTGTGAGGAC	59.968	165
TRINITY_Contig9	1	c	(CAGG)4aggagggggggcggaggagaccgggtgggggagtgggagactaacct(CGG)4	74	3332	3405	ATCCCCAATCCCTCAGAGCT	60.029	TACCCGGGCCATAAACCCTA	60.03	181
TRINITY_Contig13	1	p4	(GACA)3	12	216	227	AGATCCCATCTCGTCCACCA	60.032	CGCACGCAATTATTCCCTGG	59.971	175
TRINITY_Contig13	2	p2	(GC)6	12	491	502	AGATCCCATCTCGTCCACCA	60.032	CGCACGCAATTATTCCCTGG	59.971	175
TRINITY_Contig13	3	c	(GCA)5gttcgcgggcgtcgacctgcgccggcccaagggctacgccgcgccgccggcggcagcgcg(GGC)6	93	3178	3270	AGATCCCATCTCGTCCACCA	60.032	CGCACGCAATTATTCCCTGG	59.971	175
TRINITY_Contig13	4	p6	(CGCCGG)3	18	3953	3970	AGATCCCATCTCGTCCACCA	60.032	CGCACGCAATTATTCCCTGG	59.971	175
TRINITY_Contig13	5	p6	(GCGACG)3	18	5431	5448	AGATCCCATCTCGTCCACCA	60.032	CGCACGCAATTATTCCCTGG	59.971	175
TRINITY_Contig16	1	p4	(GGAA)3	12	183	194	AGATTGCATCAGCGGGAACA	60.035	GCATGTTTGCCATCGGATCC	59.969	118
TRINITY_Contig16	2	p3	(CGC)4	12	344	355	AGATTGCATCAGCGGGAACA	60.035	GCATGTTTGCCATCGGATCC	59.969	118
TRINITY_Contig17	1	p3	(GAA)4	12	1213	1224	GTCAAACACAACGTGTCGGG	59.973	GGTGTGCGTGTTACCCAAAC	59.97	161
TRINITY_Contig17	2	c*	(CTCCAC)2(CT<CCAC>CACCACCACCACCGCGGCCACGTCGACGGCGACCCCGGCCCCCTCCACCCCCGCTCGGACCGGGGGGAGGTGCGCCAAGGAGGGAGGTGGCAGGTGGCGTGGTGGAGGAAGTCGGTGGGGTGGTCACGAGAGCGGTAGAGGTGGAGGAGGAGGACCCGGTCGTGGAACTCCGGGTGATCTCCTCCAAAATGAGGTCATCCCGGACCTGCAGGAAGGTCGGGAAGGGCCTCTGCCTGGTGATCCAGGTCCGCAGGTGCTCGTAGGCGGCGTTGAGGCCACGGAGGACGTTGAGCACCAGGATCCGATCGGCCATCGGGTAGCCAAGATCGCTGAGAGCATCGGCCATGCCCTTCATCCTTCGGCAGTACTCGCCCACCGACAGATCCCCCTGCTCAAGCGTGCGGAAGGACGCATCGA	29	2084	2112	GTCAAACACAACGTGTCGGG	59.973	GGTGTGCGTGTTACCCAAAC	59.97	161
TRINITY_Contig17	3	p3	(GGA)4	12	2244	2255	GTCAAACACAACGTGTCGGG	59.973	GGTGTGCGTGTTACCCAAAC	59.97	161
TRINITY_Contig22	1	p4	(CTTG)3	12	4840	4851	TTACGCCCCCATCCAACTTC	60.035	TGGTGTGTGTGAGCTGTTGT	60.036	191
TRINITY_Contig23	1	p4	(CAAG)3	12	2560	2571	TCAGGGGCCAACAAACAGTT	60.033	GGATGGGCCTAGGATCATGC	60.035	225
TRINITY_Contig24	1	p3	(GCG)4	12	209	220	GCTAGATGTGTCGATGGCGA	59.969	AGTTGTCGTCACCTAGCAGC	60.038	139
TRINITY_Contig24	2	p3	(GCG)4	12	433	444	GCTAGATGTGTCGATGGCGA	59.969	AGTTGTCGTCACCTAGCAGC	60.038	139
TRINITY_Contig24	3	p3	(CTC)4	12	693	704	GCTAGATGTGTCGATGGCGA	59.969	AGTTGTCGTCACCTAGCAGC	60.038	139
TRINITY_Contig24	4	p3	(CGA)4	12	1173	1184	GCTAGATGTGTCGATGGCGA	59.969	AGTTGTCGTCACCTAGCAGC	60.038	139
TRINITY_Contig24	5	c	(GAGGC)3gtgccgcgtgcgcgcacggcaccgccccgggggccccggccgagagc(GCGTG)3gcggctgccgggcgctcgccgtcccagctgggaagggtgtgcgcgggcgtcgctctcgcgacagctcggcctgcccccaccgcacgcccatg(GGC)4tgtgggcgtggagcgataccgcgtgcgccgccgccggcgcatgtttctttattccattcggcgcgtggcgcggcaccaccgcaccca(CCAG)4ccgcctcgcccgcggtcgccgtcacctgccgctcggtgccagctccgtgtctcgtttccgtctgagctgagcccgtggacc(AG)7	379	1309	1687	GCTAGATGTGTCGATGGCGA	59.969	AGTTGTCGTCACCTAGCAGC	60.038	139
TRINITY_Contig24	6	p3	(CTT)4	12	3488	3499	GCTAGATGTGTCGATGGCGA	59.969	AGTTGTCGTCACCTAGCAGC	60.038	139
TRINITY_Contig24	7	p6	(TGTCCG)3	18	3682	3699	GCTAGATGTGTCGATGGCGA	59.969	AGTTGTCGTCACCTAGCAGC	60.038	139
TRINITY_Contig25	1	p3	(TCC)4	12	582	593	CGGGAGAAGGAGGAGGAGAA	60.033	CCGATCATACGACAGCGACA	59.971	159
TRINITY_Contig25	2	p3	(TGG)4	12	2104	2115	CGGGAGAAGGAGGAGGAGAA	60.033	CCGATCATACGACAGCGACA	59.971	159
TRINITY_Contig25	3	p4	(GCCG)4	16	2641	2656	CGGGAGAAGGAGGAGGAGAA	60.033	CCGATCATACGACAGCGACA	59.971	159
TRINITY_Contig25	4	p4	(CAGC)3	12	3816	3827	CGGGAGAAGGAGGAGGAGAA	60.033	CCGATCATACGACAGCGACA	59.971	159
TRINITY_Contig25	5	p6	(CCGGCG)3	18	4244	4261	CGGGAGAAGGAGGAGGAGAA	60.033	CCGATCATACGACAGCGACA	59.971	159
TRINITY_Contig25	6	c	(GGGTT)3ggatgacgagagggccgggggggaagcggac(ACCG)4agacaggacaggttggctggaggctggcgcgg(GGCT)3	106	4610	4715	CGGGAGAAGGAGGAGGAGAA	60.033	CCGATCATACGACAGCGACA	59.971	159
TRINITY_Contig26	1	p2	(AG)6	12	2070	2081	AAAACAAGGTTCCAGGGGCA	60.032	CTCGGCCGTTTTGCAGAAAA	59.97	263
TRINITY_Contig26	2	c	(GAGG)3aggcgcgcgagctgggcc(GGCT)3gggccgactgggccgcgggagggtaa(AG)19	106	2322	2427	AAAACAAGGTTCCAGGGGCA	60.032	CTCGGCCGTTTTGCAGAAAA	59.97	263
TRINITY_Contig28	1	p6	(ATGCTG)3	18	1219	1236	AGCCGACACACTCGTTGAAT	59.966	TCGTGAGGGGCATCTAAGGA	60.033	181
TRINITY_Contig28	2	p3	(TGC)5	15	2909	2923	AGCCGACACACTCGTTGAAT	59.966	TCGTGAGGGGCATCTAAGGA	60.033	181
TRINITY_Contig28	3	c	(TGT)4(TGC)8	36	3131	3166	AGCCGACACACTCGTTGAAT	59.966	TCGTGAGGGGCATCTAAGGA	60.033	181
TRINITY_Contig28	4	c	(GCT)6gcatatcctgcacagtatccat(CTG)4	52	3444	3495	AGCCGACACACTCGTTGAAT	59.966	TCGTGAGGGGCATCTAAGGA	60.033	181
TRINITY_Contig28	5	c	(AGC)4gggagcaaccgcggtcgccgggctccggctagggtttagcgt(CGC)4	66	4592	4657	AGCCGACACACTCGTTGAAT	59.966	TCGTGAGGGGCATCTAAGGA	60.033	181
TRINITY_Contig30	1	p3	(GCG)5	15	4634	4648	GCCAAACGGTCCAAAGACAC	59.97	CTTGCATATTGCTCGGTGGC	59.97	145
TRINITY_Contig30	2	p6	(GTGGGA)3	18	5109	5126	GCCAAACGGTCCAAAGACAC	59.97	CTTGCATATTGCTCGGTGGC	59.97	145
TRINITY_Contig31	1	c	(GGGGGC)3gt(GCGTGA)3	38	4580	4617	GATCACCTCCAGGATCGCAG	59.967	TGTATCCACCGGGTGCTAGA	60.033	219
TRINITY_Contig32	1	p3	(ACC)4	12	119	130	TGGGCGGTTACTATCCTGGA	60.032	ATCCTGCTCCTGGATCACCT	60.03	120
TRINITY_Contig32	2	p3	(AGG)4	12	3625	3636	TGGGCGGTTACTATCCTGGA	60.032	ATCCTGCTCCTGGATCACCT	60.03	120
TRINITY_Contig33	1	c	(CTTCTC)3ctcctcttc(CTT)4	39	4135	4173	TTTGGTGGACGAGACGGAAG	59.967	ACATCGACGGACCATATGGC	59.966	122
TRINITY_Contig34	1	p2	(AT)7	14	1111	1124	CCCAGAAGTCGTATCCGTCG	59.971	AAGCTCGCGGACAACTACAA	59.967	196
TRINITY_Contig34	2	p3	(CGG)4	12	3465	3476	CCCAGAAGTCGTATCCGTCG	59.971	AAGCTCGCGGACAACTACAA	59.967	196
TRINITY_Contig34	3	p3	(GCG)4	12	4077	4088	CCCAGAAGTCGTATCCGTCG	59.971	AAGCTCGCGGACAACTACAA	59.967	196
TRINITY_Contig34	4	p4	(GCCT)3	12	4778	4789	CCCAGAAGTCGTATCCGTCG	59.971	AAGCTCGCGGACAACTACAA	59.967	196
TRINITY_Contig35	1	p4	(GGAA)6	24	284	307	GAGGAAGACGAGATCTGCGG	59.97	GAGAGTCGTGCGTACCGTAG	59.974	122
TRINITY_Contig35	2	p3	(GCA)5	15	1295	1309	GAGGAAGACGAGATCTGCGG	59.97	GAGAGTCGTGCGTACCGTAG	59.974	122
TRINITY_Contig35	3	p3	(GGC)5	15	1857	1871	GAGGAAGACGAGATCTGCGG	59.97	GAGAGTCGTGCGTACCGTAG	59.974	122
TRINITY_Contig35	4	c	(CCT)4catgtgcgcgtgcctcgggggagggacgccgaa(GCCGGC)3	63	2262	2324	GAGGAAGACGAGATCTGCGG	59.97	GAGAGTCGTGCGTACCGTAG	59.974	122
TRINITY_Contig35	5	p2	(AT)12	24	3188	3211	GAGGAAGACGAGATCTGCGG	59.97	GAGAGTCGTGCGTACCGTAG	59.974	122
TRINITY_Contig35	6	p3	(GCC)6	18	4626	4643	GAGGAAGACGAGATCTGCGG	59.97	GAGAGTCGTGCGTACCGTAG	59.974	122
TRINITY_Contig35	7	p5	(GCCAA)3	15	4780	4794	GAGGAAGACGAGATCTGCGG	59.97	GAGAGTCGTGCGTACCGTAG	59.974	122
TRINITY_Contig38	1	p3	(CCG)4	12	4169	4180	CTACGCTACCTGGAGCATGG	59.967	CTAATCTCCTCGACCGTGGC	59.969	164
TRINITY_Contig39	1	p3	(GCC)4	12	3366	3377	GTGCCAACAAAAGGACCGAC	59.97	TCGTCCTTCTCCTCCTCTGG	60.034	173
TRINITY_Contig40	1	p3	(CCG)4	12	400	411	ATGAAGCTGTGGATTGCGGA	60.035	CTGATGCTACCGTCGTCCTC	59.97	170
TRINITY_Contig41	1	p3	(CTG)4	12	2113	2124	CAAACATGCACAGCCGGAAA	59.968	GAACGGTCTGTGTCTCTGCA	59.968	268

TRINITY_Contig41	2	p3	(GCG)4	12	2384	2395	CAAACATGCACAGCCGGAAA	59.968	GAACGGTCTGTGTCTCTGCA	59.968	268
TRINITY_Contig42	1	c	(CCGC)3gccggttggaaaaaaaggggtttc gctcgtccggagaaggcaagg(CGCC)3	69	147	215	TCTGAGGCTAATCCACCCGA	60.033	ACTGGTGGCCGTTCTGTTAG	59.965	188
TRINITY_Contig42	2	p3	(AGC)6	18	417	434	TCTGAGGCTAATCCACCCGA	60.033	ACTGGTGGCCGTTCTGTTAG	59.965	188
TRINITY_Contig44	1	p3	(CCG)4	12	136	147	CCATATGCTCCGTGTCCTCC	59.966	ATCTACCCAGCAACCACAGC	60.035	118
TRINITY_Contig44	2	c	(CCT)4ccc(CCT)4	27	410	436	CCATATGCTCCGTGTCCTCC	59.966	ATCTACCCAGCAACCACAGC	60.035	118
TRINITY_Contig44	3	p3	(CGC)4	12	889	900	CCATATGCTCCGTGTCCTCC	59.966	ATCTACCCAGCAACCACAGC	60.035	118
TRINITY_Contig44	4	p4	(CTGA)3	12	2358	2369	CCATATGCTCCGTGTCCTCC	59.966	ATCTACCCAGCAACCACAGC	60.035	118
TRINITY_Contig44	5	p4	(CCAG)3	12	2521	2532	CCATATGCTCCGTGTCCTCC	59.966	ATCTACCCAGCAACCACAGC	60.035	118
TRINITY_Contig44	6	c	(AGG)4g(GGA)4	25	4024	4048	CCATATGCTCCGTGTCCTCC	59.966	ATCTACCCAGCAACCACAGC	60.035	118
TRINITY_Contig44	7	p3	(CGG)4	12	4307	4318	CCATATGCTCCGTGTCCTCC	59.966	ATCTACCCAGCAACCACAGC	60.035	118
TRINITY_Contig46	1	p6	(GGGAGC)4	24	4977	5000	TCCGCGAGTACCTCTTCTGA	60.036	TGTTTGCGTTGTTGAGCGAG	59.973	210
TRINITY_Contig46	2	p3	(GGC)4	12	5149	5160	TCCGCGAGTACCTCTTCTGA	60.036	TGTTTGCGTTGTTGAGCGAG	59.973	210
TRINITY_Contig46	3	p3	(CAG)5	15	5928	5942	TCCGCGAGTACCTCTTCTGA	60.036	TGTTTGCGTTGTTGAGCGAG	59.973	210
TRINITY_Contig47	1	p4	(CTGC)3	12	2715	2726	CTGAAAACGCATGTGCCACA	59.969	TCCCTTAGATGGCTCAGGCT	60.031	128
TRINITY_Contig48	1	c	(TCGCA)3tcgcccacgcaagtagggttttcc ttcccttcccccttccacctcccctccgtcgagt cgatc(TCCG)3ccgcattctctcccgccccct cgccgccgccaacccaaaccctagtcccatgg ggcgcaagaagaacgtcgccat(CGA)4gta ctccttcccgcaggacgacgacgccgccgacc cag(CGC)4ccgccgcggagaaggagaagc ccaagaagggcggcaagaagggcaagaagg gaggcaaggccgccgcgcc(CGA)4ctacga accccctccgcccccgcccgccgcggacgagg aggatgacgacgagcccgtcaacctcgtcttc accggc(AAG)6	404	149	552	ATGTGGATGACAAGGCTGGG	60.034	TTTGCTGCCGCCTTTTTCTC	59.968	103
TRINITY_Contig48	2	p3	(GAA)4	12	717	728	ATGTGGATGACAAGGCTGGG	60.034	TTTGCTGCCGCCTTTTTCTC	59.968	103
TRINITY_Contig48	3	p3	(AGA)4	12	866	877	ATGTGGATGACAAGGCTGGG	60.034	TTTGCTGCCGCCTTTTTCTC	59.968	103
TRINITY_Contig48	4	c*	(AAG)6(<AAG>GAG)(AAGGAG)2	36	1426	1461	ATGTGGATGACAAGGCTGGG	60.034	TTTGCTGCCGCCTTTTTCTC	59.968	103
TRINITY_Contig48	5	p3	(GAG)4	12	1669	1680	ATGTGGATGACAAGGCTGGG	60.034	TTTGCTGCCGCCTTTTTCTC	59.968	103
TRINITY_Contig48	6	p4	(AGAA)3	12	3580	3591	ATGTGGATGACAAGGCTGGG	60.034	TTTGCTGCCGCCTTTTTCTC	59.968	103
TRINITY_Contig51	1	p3	(GCA)6	18	1115	1132	TCTAGGAATGTGGGGGAGGG	60.03	GCCCAGATTTGTCCAGGTGA	59.962	224
TRINITY_Contig52	1	c	(TGA)4accccc(CCA)4ccgctaccgccttt gcgggagggcggcggggaaggcgacgaacc tccgtcgctggagccctcgccgatgtgtagctt cctgaggaagttcttcat(GCC)7tccgagac cgaggtcgccgtcgaagctatcgcctcgcctg gtgttactggcgctggt(GGTG)3	215	3745	3959	ATCACCCTCTCCTCCACCTC	60.032	CGATCGCAGCAGGAAGAGAT	59.968	140
TRINITY_Contig53	1	p4	(GTTG)3	12	3194	3205	AGCATCTAATCGTCGCCCTG	59.967	CAGCAGCTCAACCAACCAAC	59.969	135
TRINITY_Contig54	1	p4	(CATG)3	12	512	523	AGGGGAAGAGGAGGCTGTAG	60.032	CACCTACTGGGCTCTCTCCT	60.033	140
TRINITY_Contig54	2	c	(AGC)4tgacctatacttactactaattaagct agcacaa(AATC)4	62	1043	1104	AGGGGAAGAGGAGGCTGTAG	60.032	CACCTACTGGGCTCTCTCCT	60.033	140
TRINITY_Contig54	3	p3	(CGA)4	12	1516	1527	AGGGGAAGAGGAGGCTGTAG	60.032	CACCTACTGGGCTCTCTCCT	60.033	140
TRINITY_Contig54	4	p6	(CGGCGC)3	18	2535	2552	AGGGGAAGAGGAGGCTGTAG	60.032	CACCTACTGGGCTCTCTCCT	60.033	140
TRINITY_Contig54	5	p3	(GAG)4	12	3775	3786	AGGGGAAGAGGAGGCTGTAG	60.032	CACCTACTGGGCTCTCTCCT	60.033	140
TRINITY_Contig54	6	c	(GATT)3gcaacgccggcgaggtgaggcgg gcgttgactttgcgaggcttgccgcgccgggg aggg(AGGAA)3gcgggcggcggagttgg acccggaacggaacccctgcctgctgcctgcct cgcggtcggcagccaag(ACC)4	165	3915	4079	AGGGGAAGAGGAGGCTGTAG	60.032	CACCTACTGGGCTCTCTCCT	60.033	140
TRINITY_Contig57	1	p3	(CTC)4	12	97	108	GCGATGGCGTTAGATCTGGA	59.968	TGCCGCAAACGAAAAGAGTG	59.971	272
TRINITY_Contig57	2	p6	(CCGGCG)3	18	239	256	GCGATGGCGTTAGATCTGGA	59.968	TGCCGCAAACGAAAAGAGTG	59.971	272
TRINITY_Contig57	3	p3	(GCA)4	12	1208	1219	GCGATGGCGTTAGATCTGGA	59.968	TGCCGCAAACGAAAAGAGTG	59.971	272
TRINITY_Contig57	4	p3	(TGC)4	12	1568	1579	GCGATGGCGTTAGATCTGGA	59.968	TGCCGCAAACGAAAAGAGTG	59.971	272
TRINITY_Contig57	5	p4	(CAAG)3	12	3907	3918	GCGATGGCGTTAGATCTGGA	59.968	TGCCGCAAACGAAAAGAGTG	59.971	272
TRINITY_Contig59	1	c*	(ACGC)3(A<CGC>GCGCGCGCGCCG CTGGGCATTCCCCGTGGAAGACGC TTCGGCGTCGCTGTCGCTGTCGCAT ACGCGGGCGCATCTCTCCGTCCGTG CCGCCCCGAAACACGCTTTGCCTTC CGTCCCAAGCTGTCGTGTCGGCGG TGCCGCCACCTCGCCCTCGGTAGCT AGTTACGTAGGTGAGGGGAGGCA CGGTAACCACCGCTTCGCTTACCTG ACGCGCCATTCCTTCGCGCCTCCCC TATTTTGACTTCTTGCCAGCCGCCG GCCAGCTGCCGCGCGGGAGGAGAT TTCCGGGTCGGATGAGATGAGGAG GCGCTGCGGCTGGCTCTCGTCCCTC TTCCGCCCTCGCCGGGGCGGGCGC GCGCTCCCGCAACCAGATCAACCGC GCCCGGATTCCGAGCCCGAGGTAG TAGATAAGCGTGGTGCTTCTAACG	25	100	124	CATGCATAGCGAGGCACAAC	59.971	GCAGTGACCACGAACCTGTA	59.967	194
TRINITY_Contig60	1	p5	(AATGC)3	15	2804	2818	GCCGCTTTAGACGCAATCTG	59.973	CCACCTCTTCCTCACGTTCC	60.037	234
TRINITY_Contig61	1	c	(CT)8tctggcccacttcaagtcccgtccaccc ttcgt(CTTC)5(CCGT)3	81	1	81	AAGGACCTCATCTACGCCCT	60.032	GGCGTTATACTCGTCCCAGG	59.968	183
TRINITY_Contig62	1	p3	(GGC)4	12	3644	3655	TCCGGCACTCCTTTGGATTC	60.035	CAACTTGCAGTGCCAGTTCC	59.969	115
TRINITY_Contig63	1	c	(CGA)4gagccacggcggcgccgagccgcg gcaccgcagcag(CGGCGC)3	66	3569	3634	GTTAGGAAAACGGCGCGATC	59.974	AAGGAGGCGAGGGATACACT	60.032	138
TRINITY_Contig65	1	p3	(GCG)5	15	374	388	CAAAGGGTGAAGCCAGTTGC	59.967	AGTACTCTGGCGGTCTAGCA	60.034	192
TRINITY_Contig65	2	p2	(AT)7	14	2742	2755	CAAAGGGTGAAGCCAGTTGC	59.967	AGTACTCTGGCGGTCTAGCA	60.034	192
TRINITY_Contig66	1	c	(CGA)4ccaccgctgcgactgcggctcgggct tgggcgccgc(CTG)4ggacgcctggatcatc gaccggagcggcgacacc(CGCGG)3	109	5850	5958	GCTTATCAGCTCGACCCTCC	59.967	CACTGCCGTTGACAACAGTG	59.972	155
TRINITY_Contig66	2	p3	(TCA)5	15	6585	6599	GCTTATCAGCTCGACCCTCC	59.967	CACTGCCGTTGACAACAGTG	59.972	155
TRINITY_Contig68	1	p3	(GCG)5	15	53	67	CGCTGGAGCTGGTAGATGAG	59.968	GCACAAGGCACAGCTCTTTC	60.04	259
TRINITY_Contig70	1	p6	(GGCGGT)3	18	3652	3669	TCGTCCACCAGAAGCTGTTC	59.966	GTTGGCTTGTTTGGAGCTGG	59.967	230
TRINITY_Contig75	1	p4	(ACCC)3	12	372	383	ATTCGCAGCTGATACCTCCG	59.967	TCAAGGCGGTTCATGACGAA	59.966	236
TRINITY_Contig75	2	c	(TCGC)3cgcgacgcgctgcgc(TGCGA)4	47	3622	3668	ATTCGCAGCTGATACCTCCG	59.967	TCAAGGCGGTTCATGACGAA	59.966	236
TRINITY_Contig76	1	p4	(TACA)3	12	396	407	CACGAGTCGGTCACAAGACA	59.97	TGGAAAGTGAGCTCGCCAAT	59.963	111
TRINITY_Contig79	1	p4	(GTCC)3	12	2829	2840	CCTGCTCCATGAGAAGTGCA	60.036	TCAATTTGCCCAAGTGCTGC	59.966	177
TRINITY_Contig79	2	p4	(TTCA)3	12	3610	3621	CCTGCTCCATGAGAAGTGCA	60.036	TCAATTTGCCCAAGTGCTGC	59.966	177
TRINITY_Contig80	1	c	(TTCCCC)3ttcctccttcccctcccttcctctg caggtttgctccatggcagcccggcgcgatgg gcctgctcggcgggccggcgtcaggggagac gcgg(TCCCCT)3	128	845	972	CGCTCACAAACACACATGCA	59.971	CTTTCAGTGGTAGGCGACGT	60.039	157
TRINITY_Contig81	1	p5	(CTGCT)3	15	15	29	GTGCACGAACAGTCCAACAC	59.972	AGCTGCTTTGCAACATGTGG	59.967	205
TRINITY_Contig82	1	c	(TCC)5gag(GCC)4	30	3378	3407	GAAGCATCGTCAACGCCATC	59.973	GGTGGTGATGCAGATTCCCA	60.034	251
TRINITY_Contig83	1	p3	(TGA)4	12	738	749	TCTGGCATCGGAGGAAGAGA	60.033	TTTGGGGATGAGGATTGGGC	60.032	136
TRINITY_Contig85	1	p3	(CGC)4	12	168	179	CATGGAGATCGAGACACCCG	59.969	GAAGACAGGCTGCCTTCCAT	60.035	261
TRINITY_Contig85	2	c	(GAT)4ggcgctgaagatct(TGA)4	38	3001	3038	CATGGAGATCGAGACACCCG	59.969	GAAGACAGGCTGCCTTCCAT	60.035	261
TRINITY_Contig87	1	p3	(GGC)4	12	3463	3474	AAGGGGTTGTGGAACTGCAA	60.033	TCGTGAAAAGGATCGGCACA	59.966	185
TRINITY_Contig88	1	p5	(AGGGC)3	15	420	434	GGCATCGCATTCTTCGTCAC	59.973	GACTTTCTGGTGGCTCGTCA	59.966	128
TRINITY_Contig88	2	p4	(CCGC)3	12	1214	1225	GGCATCGCATTCTTCGTCAC	59.973	GACTTTCTGGTGGCTCGTCA	59.966	128
TRINITY_Contig88	3	c	(GCT)5gggcgtgcgca(CCG)6atgcggag cgcgtcgggggacggcctcgcggtgagcgcg cggctgtcccagtcccacatcttggggttccag ttcccgtccc(CCG)4	138	5947	6084	GGCATCGCATTCTTCGTCAC	59.973	GACTTTCTGGTGGCTCGTCA	59.966	128
TRINITY_Contig88	4	p3	(CTC)5	15	6217	6231	GGCATCGCATTCTTCGTCAC	59.973	GACTTTCTGGTGGCTCGTCA	59.966	128
TRINITY_Contig88	5	p4	(GAGC)4	16	6390	6405	GGCATCGCATTCTTCGTCAC	59.973	GACTTTCTGGTGGCTCGTCA	59.966	128
TRINITY_Contig89	1	c	(CGCGAC)3aggtcggagccgccgtgccgg gagctgttctgcc(CGG)5	67	682	748	CGGAGGGAGAAGACGACATG	59.899	GCCTCGATTCTCTCTGCTCC	59.968	100

TRINITY_Contig89	2	p3	(CGC)4	12	1540	1551	CGGAGGGAGAAGACGACATG	59.899	GCCTCGATTCTCTCTGCTCC	59.968	100
TRINITY_Contig89	3	p3	(TCT)4	12	1817	1828	CGGAGGGAGAAGACGACATG	59.899	GCCTCGATTCTCTCTGCTCC	59.968	100
TRINITY_Contig89	4	p3	(CGT)4	12	3096	3107	CGGAGGGAGAAGACGACATG	59.899	GCCTCGATTCTCTCTGCTCC	59.968	100
TRINITY_Contig89	5	p6	(GGCGGT)3	18	3237	3254	CGGAGGGAGAAGACGACATG	59.899	GCCTCGATTCTCTCTGCTCC	59.968	100
TRINITY_Contig89	6	c	(CA)6agaaaaaaaaaagagagagggagat gatgagc(TGA)4	56	3409	3464	CGGAGGGAGAAGACGACATG	59.899	GCCTCGATTCTCTCTGCTCC	59.968	100
TRINITY_Contig90	1	p3	(GCG)4	12	3405	3416	TTAGCTGCTGCAGAACCTCC	60.036	GCCAAGGGAGGAGCTTGATT	60.034	254
TRINITY_Contig95	1	p3	(CCG)4	12	278	289	CCATAGGCCGGTCGTTCTAC	59.968	GGCTCGATAAACGCCGTAGA	59.97	154
TRINITY_Contig95	2	c	(GGA)4gggaggcgccggggcgggcgcggc cgggccaaggcggccgca(GCG)4	66	434	499	CCATAGGCCGGTCGTTCTAC	59.968	GGCTCGATAAACGCCGTAGA	59.97	154
TRINITY_Contig97	1	p2	(CT)19	38	59	96	TCGGTTGATGCGAAGGGAAA	59.964	TCGACGCCATTAGCATCTCC	59.968	109
TRINITY_Contig97	2	p3	(TGA)4	12	1018	1029	TCGGTTGATGCGAAGGGAAA	59.964	TCGACGCCATTAGCATCTCC	59.968	109
TRINITY_Contig99	1	p3	(GGT)4	12	2654	2665	TTAGGCGCAAGAATGGAGCA	60.036	ACTGAACAGTGGGGGCAAAA	60.033	174
TRINITY_Contig99	2	p3	(GCG)5	15	2862	2876	TTAGGCGCAAGAATGGAGCA	60.036	ACTGAACAGTGGGGGCAAAA	60.033	174
TRINITY_Contig99	3	c	(GCG)4gggacgcgtgcctgcacgacctcga cctcgacgccgcgcggcgcggcgagcgggag gcgcggcgcgggc(CCG)4	93	3040	3132	TTAGGCGCAAGAATGGAGCA	60.036	ACTGAACAGTGGGGGCAAAA	60.033	174
TRINITY_Contig100	1	p4	(ACGC)3	12	1835	1846	CCCTGAGATTGCCCACATGT	60.034	GGGATACACACAACCAGCGA	60.037	191
TRINITY_Contig100	2	p3	(TCC)5	15	2278	2292	CCCTGAGATTGCCCACATGT	60.034	GGGATACACACAACCAGCGA	60.037	191
TRINITY_Contig100	3	p3	(CCG)4	12	2645	2656	CCCTGAGATTGCCCACATGT	60.034	GGGATACACACAACCAGCGA	60.037	191
TRINITY_Contig101	1	p3	(GCG)4	12	2896	2907	TGACGATCTGCAACGGTTGA	59.967	GAATGGATCTCGGAGGCCTG	59.965	141
TRINITY_Contig102	1	c	(AAGC)3aatcgcttaaccacacaaaaccttc acctct(TTTC)3	55	25	79	CCCTCCTCTCCTGTAGCCTT	60.032	ACGCAGTGATCAGCTGAGAC	60.109	113
TRINITY_Contig102	2	p2	(GT)6	12	210	221	CCCTCCTCTCCTGTAGCCTT	60.032	ACGCAGTGATCAGCTGAGAC	60.109	113
TRINITY_Contig103	1	p3	(CAC)4	12	2979	2990	CACCTTCGCCGTGATATCGA	59.97	TCGATGCTCTTGGTGGCATT	60.035	120
TRINITY_Contig104	1	p2	(AG)6	12	2625	2636	TCGAACCCAGGAAAGCTGAC	59.965	TCCACCTCGTCTTCAACAGC	59.966	192
TRINITY_Contig105	1	p2	(CT)12	24	396	419	AGAATCTACGAGCGCACGAG	59.972	TCGAAGAGAGGGAGGTGAGG	60.034	199
TRINITY_Contig106	1	p4	(GGCC)3	12	1111	1122	ATCTTCTTCACGGCCACCTG	60.036	CGAGGACATGACCAACAGCT	60.037	105
TRINITY_Contig106	2	p3	(AGG)5	15	2904	2918	ATCTTCTTCACGGCCACCTG	60.036	CGAGGACATGACCAACAGCT	60.037	105
TRINITY_Contig107	1	c	(GCT)7aactggctaataagccgagctgactg tgagttgagctacttgttgctgctagc(AGA)4 (TGGG)3	98	2741	2838	AAATAGCCCACCCTCTTGCC	60.033	AATTTGGGTTGGTGGTGGGT	60.031	104
TRINITY_Contig107	2	p4	(ATGG)3	12	3035	3046	AAATAGCCCACCCTCTTGCC	60.033	AATTTGGGTTGGTGGTGGGT	60.031	104
TRINITY_Contig109	1	c	(ATAG)3acagacagataaac(AG)6	38	310	347	AAAAAGTGCGACCGGAAAGC	59.97	CGGATGTGACCCGGTTACAA	60.037	219
TRINITY_Contig109	2	p4	(ATAC)3	12	3203	3214	AAAAAGTGCGACCGGAAAGC	59.97	CGGATGTGACCCGGTTACAA	60.037	219
TRINITY_Contig111	1	p3	(GCC)6	18	1915	1932	TTCCTGAGCCTGTGCATGAG	60.036	GACCCGTTTCTTGTGCACAC	59.971	181
TRINITY_Contig112	1	p4	(AGCC)3	12	541	552	GCATGGAGCTCCTTCCACTT	60.035	AGGGAGATCAGACCAGCCAT	60.03	107
TRINITY_Contig114	1	p3	(CAT)7	21	3035	3055	CCAAAACACACCTTCTGCCG	59.969	GTGGCGACTGGTGTACAAGA	59.967	145
TRINITY_Contig115	1	p3	(CTC)4	12	311	322	AGGTGGCGGTGAATACTGTG	60.036	GGCGTAAAGGTTTGCAGCAA	59.969	137
TRINITY_Contig117	1	c	(GGC)5gacccgcaccacgaggacttccagc tcaaggacacgaacccgctcctcggcgagcaa tggcccaagggcgcggctggcccggcgcgccc cgcg(GGC)4	120	616	735	CTGAGACCTTGTGCTGCAGA	59.966	GTTCGTGTCCTTGAGCTGGA	59.966	142
TRINITY_Contig117	2	p3	(CGC)4	12	1230	1241	CTGAGACCTTGTGCTGCAGA	59.966	GTTCGTGTCCTTGAGCTGGA	59.966	142
TRINITY_Contig118	1	p3	(CCG)6	18	1746	1763	AGGAGGTGGGGCATTCAATG	60.033	CTGCATGTCCCTCACGTTCT	60.037	268
TRINITY_Contig118	2	p3	(CCG)4	12	2860	2871	AGGAGGTGGGGCATTCAATG	60.033	CTGCATGTCCCTCACGTTCT	60.037	268
TRINITY_Contig119	1	p3	(ATC)5	15	1361	1375	GATGGCTGGCTTCCCCTATC	59.963	TCTGCTGCTGAGAATTGGGG	60.035	198
TRINITY_Contig120	1	p3	(CGC)4	12	340	351	TCGAGCTCAACACAGGTCAC	59.968	GGCACTGCACCTTCAACAAG	59.969	201
TRINITY_Contig120	2	p3	(CGA)4	12	2836	2847	TCGAGCTCAACACAGGTCAC	59.968	GGCACTGCACCTTCAACAAG	59.969	201
TRINITY_Contig120	3	p4	(AGCT)3	12	3002	3013	TCGAGCTCAACACAGGTCAC	59.968	GGCACTGCACCTTCAACAAG	59.969	201
TRINITY_Contig121	1	p3	(TCG)4	12	1955	1966	TGTGTGCTGGATCAGGTGTC	59.965	AGACTTTCGCTCACCTGGTG	59.966	121
TRINITY_Contig122	1	p3	(TGC)4	12	393	404	GACTGGGTGCACTCACATCA	59.965	GAGCGGGAGAAGACCAACAA	59.965	235
TRINITY_Contig122	2	p3	(TAA)4	12	4884	4895	GACTGGGTGCACTCACATCA	59.965	GAGCGGGAGAAGACCAACAA	59.965	235
TRINITY_Contig123	1	p3	(CGG)5	15	567	581	CCACGCCGCTGAAAAGAAAA	59.97	GAATCTCCCTCTCTGTCGCG	59.97	183
TRINITY_Contig123	2	p3	(AGG)4	12	1214	1225	CCACGCCGCTGAAAAGAAAA	59.97	GAATCTCCCTCTCTGTCGCG	59.97	183
TRINITY_Contig124	1	p3	(GCC)4	12	528	539	TGCATTTGTGAACGCAGGTG	59.969	CAGCAGCAGACAAACACCAC	59.97	185
TRINITY_Contig124	2	p3	(CGA)4	12	2951	2962	TGCATTTGTGAACGCAGGTG	59.969	CAGCAGCAGACAAACACCAC	59.97	185
TRINITY_Contig125	1	p6	(CCGTGG)3	18	2846	2863	CTGGCTCCAATGTGGGTGAT	60.034	CCTGAGTATGGTGGCTGCAA	60.035	230
TRINITY_Contig126	1	p3	(AAG)4	12	1534	1545	CGAGTTCCAGACGGAGATCG	59.972	GATGTTCTCCGAGTAGCCCG	59.969	253
TRINITY_Contig126	2	p3	(GCA)4	12	1770	1781	CGAGTTCCAGACGGAGATCG	59.972	GATGTTCTCCGAGTAGCCCG	59.969	253
TRINITY_Contig126	3	p3	(CGT)4	12	2801	2812	CGAGTTCCAGACGGAGATCG	59.972	GATGTTCTCCGAGTAGCCCG	59.969	253
TRINITY_Contig126	4	p3	(GAC)4	12	2989	3000	CGAGTTCCAGACGGAGATCG	59.972	GATGTTCTCCGAGTAGCCCG	59.969	253
TRINITY_Contig129	1	p3	(CGA)4	12	3164	3175	AAGGCACCAACTATCCACGG	60.035	CGATCCCGGAGAAGGATGTG	59.967	227
TRINITY_Contig130	1	p4	(GCCT)3	12	942	953	TCCGTCTTTTCGATCCTGGC	60.108	GTCGAGATCGGTTCGGACTC	59.972	128
TRINITY_Contig130	2	p5	(CCGCG)3	15	1549	1563	TCCGTCTTTTCGATCCTGGC	60.108	GTCGAGATCGGTTCGGACTC	59.972	128
TRINITY_Contig130	3	p3	(GGC)5	15	2344	2358	TCCGTCTTTTCGATCCTGGC	60.108	GTCGAGATCGGTTCGGACTC	59.972	128
TRINITY_Contig130	4	p2	(GC)6	12	2545	2556	TCCGTCTTTTCGATCCTGGC	60.108	GTCGAGATCGGTTCGGACTC	59.972	128
TRINITY_Contig130	5	p3	(TCG)4	12	2997	3008	TCCGTCTTTTCGATCCTGGC	60.108	GTCGAGATCGGTTCGGACTC	59.972	128
TRINITY_Contig131	1	p4	(AAAG)3	12	732	743	AATGATCGCGAGCTCCTCAG	59.968	TAGACTGCTTGTCCGGGAGA	59.961	222
TRINITY_Contig132	1	p3	(CCG)6	18	234	251	TCGTGTGCTCACCTCAGTTC	59.968	TGCAGCCGGTTGACATACAT	60.036	122
TRINITY_Contig133	1	p4	(GCTT)3	12	2620	2631	AATTACAGTTCAGCCCCCGG	60.034	AGGTGGGATAGGTGGTCCTC	60.031	271
TRINITY_Contig134	1	c	(GAC)5aagggcaaggac(AAG)5	42	1629	1670	CCGAGAAGGAGATCAGCGAC	59.97	TCCACGGAGAAGTGCTTCAC	59.966	265
TRINITY_Contig135	1	p3	(CTG)6	18	735	752	TCATCCTCAAGGTCGGGGAT	60.031	GATGTCGTAGCAGAGCTCCC	59.969	197
TRINITY_Contig137	1	p3	(TGG)4	12	861	872	GCCCGGATAGACTCTCTTGC	59.967	GCTGGGAGTCCTGAACAGTC	60.037	251
TRINITY_Contig138	1	p4	(GGCG)3	12	82	93	GAAGCGGATATGGCTCGTCA	59.968	AGCGCCATGATCTTGTGGAA	60.035	267
TRINITY_Contig138	2	p3	(CCG)4	12	224	235	GAAGCGGATATGGCTCGTCA	59.968	AGCGCCATGATCTTGTGGAA	60.035	267
TRINITY_Contig139	1	p5	(GGGGA)3	15	865	879	CGGACCACGGGACTCATATG	59.968	ATGAAGGGCGTGATGAAGCA	60.035	224
TRINITY_Contig139	2	p3	(GTC)4	12	2269	2280	CGGACCACGGGACTCATATG	59.968	ATGAAGGGCGTGATGAAGCA	60.035	224
TRINITY_Contig141	1	p5	(GAGGA)3	15	21	35	GGACGAGAAACCCTGTCCTG	60.037	CCATGGCTCCAAGTCCACAT	60.034	259
TRINITY_Contig141	2	p3	(CGC)5	15	291	305	GGACGAGAAACCCTGTCCTG	60.037	CCATGGCTCCAAGTCCACAT	60.034	259
TRINITY_Contig141	3	p3	(TGG)4	12	702	713	GGACGAGAAACCCTGTCCTG	60.037	CCATGGCTCCAAGTCCACAT	60.034	259
TRINITY_Contig142	1	p3	(GCC)6	18	31	48	GAGTTCACCAGATGGCACCA	59.963	AGCTGATCTGTGCAACGACA	59.966	220
TRINITY_Contig143	1	p3	(CAG)5	15	567	581	CGGCAGTGATTTCGATGCAG	59.973	TGGTGTGGCCCTAGCTATCT	60.031	238
TRINITY_Contig144	1	p5	(CGAGA)4	20	544	563	AGTGAAGGTTTCGTACGCGT	59.969	GCTCCGACCTTGTGTCTGAA	59.966	123
TRINITY_Contig144	2	p4	(GATC)4	16	772	787	AGTGAAGGTTTCGTACGCGT	59.969	GCTCCGACCTTGTGTCTGAA	59.966	123
TRINITY_Contig144	3	p3	(CGG)5	15	949	963	AGTGAAGGTTTCGTACGCGT	59.969	GCTCCGACCTTGTGTCTGAA	59.966	123
TRINITY_Contig144	4	p3	(GCG)4	12	1111	1122	AGTGAAGGTTTCGTACGCGT	59.969	GCTCCGACCTTGTGTCTGAA	59.966	123
TRINITY_Contig146	1	p3	(CCG)5	15	822	836	CTAGCTGCTACAGAGTCGGC	59.97	TCATCATCATCCCGTCGCTG	59.967	176
TRINITY_Contig146	2	p3	(TCG)4	12	1139	1150	CTAGCTGCTACAGAGTCGGC	59.97	TCATCATCATCCCGTCGCTG	59.967	176
TRINITY_Contig146	3	p3	(CGC)4	12	1576	1587	CTAGCTGCTACAGAGTCGGC	59.97	TCATCATCATCCCGTCGCTG	59.967	176
TRINITY_Contig146	4	p3	(GAT)5	15	1853	1867	CTAGCTGCTACAGAGTCGGC	59.97	TCATCATCATCCCGTCGCTG	59.967	176
TRINITY_Contig147	1	p3	(CCG)5	15	49	63	AGATGGATTGGGAGAGGCCT	60.029	AGGGTATGTCGCATGCCAAA	60.035	192
TRINITY_Contig147	2	p3	(GCG)4	12	272	283	AGATGGATTGGGAGAGGCCT	60.029	AGGGTATGTCGCATGCCAAA	60.035	192
TRINITY_Contig147	3	p4	(CCTC)3	12	1265	1276	AGATGGATTGGGAGAGGCCT	60.029	AGGGTATGTCGCATGCCAAA	60.035	192
TRINITY_Contig149	1	p5	(TTGGG)3	15	2176	2190	GCCTTTGTTGTTCGTCGCTT	59.971	AACAATGTTGCACCGCTTCC	59.968	261
TRINITY_Contig150	1	p3	(TCC)6	18	162	179	GGATTATCTGGCCCAGGAGC	59.963	GAGACCACAAGCCGAACTCA	59.966	110
TRINITY_Contig152	1	p3	(CGC)4	12	2436	2447	ACTTCCTCAAGCTCACCGTG	59.966	GACCTCGAAGTGGAAGCACA	59.966	140
TRINITY_Contig152	2	p4	(GATG)3	12	2804	2815	ACTTCCTCAAGCTCACCGTG	59.966	GACCTCGAAGTGGAAGCACA	59.966	140
TRINITY_Contig152	3	p4	(AAGC)3	12	2978	2989	ACTTCCTCAAGCTCACCGTG	59.966	GACCTCGAAGTGGAAGCACA	59.966	140
TRINITY_Contig153	1	p3	(ACG)4	12	533	544	TTCTTCTGCAACGCAACTGC	59.971	GCTGAACCGACTCTGACACA	59.968	259
TRINITY_Contig154	1	p3	(GCG)5	15	2944	2958	ATTCCTCCTCTTTGCAGCCC	60.034	GCACTGGAGGTTCTCATGCT	60.036	117
TRINITY_Contig159	1	p6	(GCGCCG)3	18	2660	2677	CCCAGGACGTATCTGCCATC	59.966	CCAAGTTGTTGTGCGACAGG	59.971	243
TRINITY_Contig160	1	c	(GGC)5agcgcttcgtggagc(GGA)5ggg ggcccttgtgctgtcccgcggccgcgctacccc cgcccctcggagtcggtgatggccccgaccgc ggagccacctt(CGC)5	139	305	443	CAGGAGGATGCACACGAGTT	60.037	ACTTCACCTGACTGAGCAGC	59.965	187
TRINITY_Contig161	1	p3	(CGC)4	12	344	355	TCACCTACAACGTCGTGCTC	60.04	GCAGCGCATTGTAGGTGATG	59.971	231
TRINITY_Contig162	1	p3	(ATA)10	30	921	950	CTGCATGCCCTTCCTTCAGA	60.035	GAACATCGCATGCACCTTCC	59.901	152
TRINITY_Contig162	2	p2	(GT)8	16	2477	2492	CTGCATGCCCTTCCTTCAGA	60.035	GAACATCGCATGCACCTTCC	59.901	152
TRINITY_Contig162	3	p4	(AAAC)4	16	2872	2887	CTGCATGCCCTTCCTTCAGA	60.035	GAACATCGCATGCACCTTCC	59.901	152
TRINITY_Contig163	1	p3	(AAT)4	12	975	986	AACACAGCAGCAACACCAAC	59.827	ATGCCTTCCTCCTGCACTTC	60.035	232
TRINITY_Contig163	2	p2	(GT)8	16	2478	2493	AACACAGCAGCAACACCAAC	59.827	ATGCCTTCCTCCTGCACTTC	60.035	232
TRINITY_Contig163	3	p4	(AAAC)4	16	2873	2888	AACACAGCAGCAACACCAAC	59.827	ATGCCTTCCTCCTGCACTTC	60.035	232
TRINITY_Contig164	1	p3	(CCG)4	12	2722	2733	TGATTGAGCTGCTTGGTCGT	59.965	CCCTGGGAAGGTCTACCGTA	60.033	163
TRINITY_Contig166	1	p2	(AG)11	22	2899	2920	CTCCAGTGCCTGCTTGATGA	60.036	CCCTCAGGTTGGTTCAGGTC	59.963	230

TRINITY_Contig167	1	p6	(GCGCCA)3	18	1492	1509	GGTGGTGATCTCCTCCTCCT	60.032	AATGGCTGGTGTTCGTCGAT	60.036	277
TRINITY_Contig167	2	p2	(TC)7	14	2827	2840	GGTGGTGATCTCCTCCTCCT	60.032	AATGGCTGGTGTTCGTCGAT	60.036	277
TRINITY_Contig169	1	p2	(TG)8	16	2828	2843	GCAATTGGACACAGCTGCAA	59.968	GTACGGGGCTTTCACTTCCA	59.964	196
TRINITY_Contig172	1	p3	(CCA)6	18	390	407	TCCATCGGGCAAGAAGGAAC	60.035	GGCTCCTCCTTCAAATGGCT	60.034	175
TRINITY_Contig172	2	p4	(AAAG)3	12	1217	1228	TCCATCGGGCAAGAAGGAAC	60.035	GGCTCCTCCTTCAAATGGCT	60.034	175
TRINITY_Contig172	3	p3	(CGC)4	12	2655	2666	TCCATCGGGCAAGAAGGAAC	60.035	GGCTCCTCCTTCAAATGGCT	60.034	175
TRINITY_Contig172	4	p5	(AATGG)3	15	2911	2925	TCCATCGGGCAAGAAGGAAC	60.035	GGCTCCTCCTTCAAATGGCT	60.034	175
TRINITY_Contig173	1	p3	(CAG)8	24	847	870	ACGGTCGAGTCCTGGATGTA	60.035	GCGCACTGCACTTTATAGCC	59.972	146
TRINITY_Contig173	2	p3	(GGC)5	15	2871	2885	ACGGTCGAGTCCTGGATGTA	60.035	GCGCACTGCACTTTATAGCC	59.972	146
TRINITY_Contig176	1	p3	(CCG)4	12	4527	4538	GAAGAGTGTGCGTTCAGGGA	59.966	CCTGACAATCGGGCTCTTGT	60.036	222
TRINITY_Contig177	1	p3	(CCG)4	12	145	156	GTATCCATCGGCGTTGGGAT	59.965	CACGTGTTGGCTGGGAAAAG	59.969	152
TRINITY_Contig178	1	p3	(AGC)4	12	157	168	ACATGGCTCGTGATGTGGTT	59.963	CTTAGCCAGTCCTGTGCACA	59.965	148
TRINITY_Contig178	2	p6	(AAAGGA)3	18	1848	1865	ACATGGCTCGTGATGTGGTT	59.963	CTTAGCCAGTCCTGTGCACA	59.965	148
TRINITY_Contig179	1	p3	(CGC)5	15	669	683	CATGGCGTGTCAAGTATGCG	59.973	AGAAGAAGACGAGCACAGCC	60.038	133
TRINITY_Contig179	2	p3	(AGA)4	12	1584	1595	CATGGCGTGTCAAGTATGCG	59.973	AGAAGAAGACGAGCACAGCC	60.038	133
TRINITY_Contig181	1	p3	(TGC)4	12	2749	2760	CCTTGTGAGGTTGTTGCTGC	59.969	ATTGCAAGCTGCCAGTGTTG	59.967	170
TRINITY_Contig182	1	p3	(CAT)4	12	636	647	TTCTGCAGGTGGAGCTTCAG	59.964	CGTGCGCAACCTAATGATGG	59.972	170
TRINITY_Contig183	1	p3	(GAA)4	12	1019	1030	CAGCAACATGCCATCGGATG	59.97	CAAGTGTTTGCCCCTGAAGC	59.967	122
TRINITY_Contig184	1	p3	(CGC)5	15	312	326	CCGTGACGTGTTAGGTTCCA	59.968	CAACGGAAGCCATTATGCCG	59.971	113
TRINITY_Contig184	2	p4	(CACG)3	12	720	731	CCGTGACGTGTTAGGTTCCA	59.968	CAACGGAAGCCATTATGCCG	59.971	113
TRINITY_Contig184	3	p3	(ACG)7	21	967	987	CCGTGACGTGTTAGGTTCCA	59.968	CAACGGAAGCCATTATGCCG	59.971	113
TRINITY_Contig184	4	p4	(CAGC)3	12	1717	1728	CCGTGACGTGTTAGGTTCCA	59.968	CAACGGAAGCCATTATGCCG	59.971	113
TRINITY_Contig184	5	p3	(CGC)5	15	2100	2114	CCGTGACGTGTTAGGTTCCA	59.968	CAACGGAAGCCATTATGCCG	59.971	113
TRINITY_Contig184	6	p3	(GCC)5	15	3834	3848	CCGTGACGTGTTAGGTTCCA	59.968	CAACGGAAGCCATTATGCCG	59.971	113
TRINITY_Contig185	1	p3	(CCT)4	12	210	221	GCCAGGAGAACAAGGTCCTC	60.036	TAGACTCTCTGCCTGCCCAT	60.032	122
TRINITY_Contig185	2	p3	(TCC)4	12	457	468	GCCAGGAGAACAAGGTCCTC	60.036	TAGACTCTCTGCCTGCCCAT	60.032	122
TRINITY_Contig185	3	p3	(CCG)5	15	940	954	GCCAGGAGAACAAGGTCCTC	60.036	TAGACTCTCTGCCTGCCCAT	60.032	122
TRINITY_Contig186	1	p4	(CCCT)3	12	172	183	TCCTCCTCCCTCTCCTCTCT	59.953	AGCAAGCGATCGAGATAGCC	60.039	120
TRINITY_Contig186	2	c*	(CGCTCC)3(CG<CTCC>TCCTCCTCC<br>TCCTCCTGCGGTGCGCCCCTGAGCC<br>GGCCGCCGCGGTGCGGTTCGACTA<br>CGCGACGCTGACGCTCGGCAGCCT<br>CAAGCTGCTCGGCGACGCGCACCT<br>CAAGAACGGCACCATCCGCCTCTCC<br>CGGGACCTGCCCGTCCCGACCTCCG<br>GCGCCGGCCGCGCGCTCTACGCCTC<br>CGCCGTCCCCGTTCGCGCCGGCTTC<br>TCCACCCAGTTCGCCTTCACCGTCG<br>CCACGCTCAACCCCTCCTCCGTCGG<br>CGGCGGGCTCGCCTTCGTCGTCGCC<br>GCCGACGACGAGTCACTCGGCGAC<br>GCCGGGCCCTACATCGGCGTCGCC<br>GCCGGGACCGACGCCGCCGCGGTC<br>GAGTTCGACACGCTCATGGACGTG<br>CAGTTCGGGGACGTCAACGGGAAC<br>CACGTGGGGCTGGATCTCGGCTCT	38	393	430	TCCTCCTCCCTCTCCTCTCT	59.953	AGCAAGCGATCGAGATAGCC	60.039	120
TRINITY_Contig187	1	p3	(GCA)4	12	2052	2063	TCCACTCCATCGAGGTGGAT	60.032	CCAATCTCTACGGCGAGACC	59.969	130
TRINITY_Contig187	2	p3	(GGC)4	12	2325	2336	TCCACTCCATCGAGGTGGAT	60.032	CCAATCTCTACGGCGAGACC	59.969	130
TRINITY_Contig187	3	p5	(GGAGT)4	20	2755	2774	TCCACTCCATCGAGGTGGAT	60.032	CCAATCTCTACGGCGAGACC	59.969	130
TRINITY_Contig188	1	p6	(ACGCCG)5	30	51	80	AACCACGGCGCTTTCTTTTC	59.97	GTGCGGCATGGCTAAAGATG	59.97	103
TRINITY_Contig188	2	p3	(AAG)4	12	2403	2414	AACCACGGCGCTTTCTTTTC	59.97	GTGCGGCATGGCTAAAGATG	59.97	103
TRINITY_Contig189	1	p5	(CGCCG)3	15	259	273	AGGCTGGGCTATCTCCAGAA	60.031	ACCACGCTGAAGTTCTCCTG	59.966	191
TRINITY_Contig190	1	p4	(CTCC)3	12	42	53	CTCCAACTCCAAGTCCCCAC	59.963	TCCCGATGCTACCAGAAGGA	60.033	180
TRINITY_Contig190	2	p3	(AGC)4	12	272	283	CTCCAACTCCAAGTCCCCAC	59.963	TCCCGATGCTACCAGAAGGA	60.033	180
TRINITY_Contig190	3	p3	(ACT)4	12	2821	2832	CTCCAACTCCAAGTCCCCAC	59.963	TCCCGATGCTACCAGAAGGA	60.033	180
TRINITY_Contig191	1	p3	(CTG)4	12	114	125	CTTAAGGAGATCGCCGGTCC	59.968	GGCACCTCTGGCATCTTTCT	60.035	239
TRINITY_Contig191	2	p3	(CGG)4	12	440	451	CTTAAGGAGATCGCCGGTCC	59.968	GGCACCTCTGGCATCTTTCT	60.035	239
TRINITY_Contig191	3	p3	(GCC)6	18	1254	1271	CTTAAGGAGATCGCCGGTCC	59.968	GGCACCTCTGGCATCTTTCT	60.035	239
TRINITY_Contig193	1	p3	(TCC)4	12	58	69	AACCTCTTGTGTGGCCCAAA	60.033	GTGGGCAAAACACATCCACC	59.967	265
TRINITY_Contig193	2	p3	(CGG)4	12	433	444	AACCTCTTGTGTGGCCCAAA	60.033	GTGGGCAAAACACATCCACC	59.967	265
TRINITY_Contig193	3	p3	(GCG)5	15	1096	1110	AACCTCTTGTGTGGCCCAAA	60.033	GTGGGCAAAACACATCCACC	59.967	265
TRINITY_Contig194	1	p4	(GAAG)3	12	196	207	GGGACTCCACCAAAGCATCA	59.962	CACTTGAGCCTGCAACAACC	59.969	253
TRINITY_Contig194	2	p3	(CGG)4	12	418	429	GGGACTCCACCAAAGCATCA	59.962	CACTTGAGCCTGCAACAACC	59.969	253
TRINITY_Contig195	1	p4	(AGAA)3	12	920	931	CAGTGTCGTTGGAGCTCTGT	59.967	TACTACATCCTGCTGGGGCT	60.031	119
TRINITY_Contig195	2	c*	(GCGCCC)2(GCGC<CC>G)(CCG)4	31	1476	1506	CAGTGTCGTTGGAGCTCTGT	59.967	TACTACATCCTGCTGGGGCT	60.031	119
TRINITY_Contig197	1	c	(CAT)5(CAG)4	27	1479	1505	GATCACTGTTCCCTGCTGCT	60.036	GCTGGTGTGGCTGTTTGAAG	59.969	196
TRINITY_Contig203	1	c	(TCGC)4gtcgtcgctcccgaccggtgctccc<br>gcctccgccgatccgcgcacgcagatcgacaa<br>gcagcccggcggcgcaggggcaaaggggag<br>aaggggagc(GAGG)3	124	221	344	TATCCCCTGGAACGCAGGTA	60.032	CCAAGCACAACAGTTCACCG	59.971	168
TRINITY_Contig204	1	p3	(GAG)4	12	2799	2810	GCTGCTGCTGTTGATATGGC	59.97	CCCGAAACAGTAGTGGCAGT	59.965	138
TRINITY_Contig204	2	p3	(GCT)5	15	3006	3020	GCTGCTGCTGTTGATATGGC	59.97	CCCGAAACAGTAGTGGCAGT	59.965	138
TRINITY_Contig204	3	c	(GCC)4gggggtcgccc(CCG)6	41	3802	3842	GCTGCTGCTGTTGATATGGC	59.97	CCCGAAACAGTAGTGGCAGT	59.965	138
TRINITY_Contig205	1	p4	(CGAT)4	16	142	157	TTCTTCGCGCACACTGTTTG	59.973	CTCCTTCATGCTAGCTCCCG	59.967	152
TRINITY_Contig205	2	p3	(GCG)5	15	1210	1224	TTCTTCGCGCACACTGTTTG	59.973	CTCCTTCATGCTAGCTCCCG	59.967	152
TRINITY_Contig207	1	p3	(CGC)4	12	1187	1198	CTCCTCGGGAGTCTTGATGC	59.896	GTCGATGGACCAGCTCTACG	59.97	133
TRINITY_Contig207	2	c	(GCC)4cccgccgcggccgccgtcctcctcct<br>gcgggttct(CCCG)3	59	2373	2431	CTCCTCGGGAGTCTTGATGC	59.896	GTCGATGGACCAGCTCTACG	59.97	133
TRINITY_Contig208	1	p5	(CGAAG)3	15	26	40	TTGGATGCGTTATGGTGCCT	60.035	GCCAAACTGGACGCAACAAT	59.968	222
TRINITY_Contig208	2	p4	(AATT)3	12	876	887	TTGGATGCGTTATGGTGCCT	60.035	GCCAAACTGGACGCAACAAT	59.968	222
TRINITY_Contig209	1	p3	(GCC)6	18	621	638	CTCCTTGAAGCTGCTGGACA	59.964	ATGCAGGACACGAACCAGAG	60.037	204
TRINITY_Contig209	2	p3	(CGC)5	15	944	958	CTCCTTGAAGCTGCTGGACA	59.964	ATGCAGGACACGAACCAGAG	60.037	204
TRINITY_Contig209	3	c	(CCG)4ctcgtcgtcctcgtcgtcgaacgcgtc<br>cagccgcaccggccggctccacagcgacacc<br>gccgacgccgaccccagctc(CGCCGA)3	108	2216	2323	CTCCTTGAAGCTGCTGGACA	59.964	ATGCAGGACACGAACCAGAG	60.037	204
TRINITY_Contig209	4	p4	(CGAC)4	16	2468	2483	CTCCTTGAAGCTGCTGGACA	59.964	ATGCAGGACACGAACCAGAG	60.037	204
TRINITY_Contig210	1	p4	(GTCA)3	12	677	688	GAACGGTCAGTCAGTCAGCA	59.968	CGCTCAATCGTGATGCACTG	59.974	264
TRINITY_Contig210	2	p3	(GAC)4	12	988	999	GAACGGTCAGTCAGTCAGCA	59.968	CGCTCAATCGTGATGCACTG	59.974	264
TRINITY_Contig210	3	p3	(CGG)4	12	2159	2170	GAACGGTCAGTCAGTCAGCA	59.968	CGCTCAATCGTGATGCACTG	59.974	264
TRINITY_Contig212	1	p5	(TCACC)3	15	623	637	GATTGGTCCTTCTGGGGGTG	60.034	TAACCAACTCCTGCCGTGTC	59.966	163
TRINITY_Contig212	2	p3	(CCG)6	18	885	902	GATTGGTCCTTCTGGGGGTG	60.034	TAACCAACTCCTGCCGTGTC	59.966	163
TRINITY_Contig212	3	p4	(AAGC)3	12	2033	2044	GATTGGTCCTTCTGGGGGTG	60.034	TAACCAACTCCTGCCGTGTC	59.966	163
TRINITY_Contig217	1	p3	(CGG)5	15	414	428	AACTGCCGAGATGTTCACGT	59.966	ACAAATCGACCCGGATGCTT	60.035	201
TRINITY_Contig217	2	p3	(CGT)4	12	1489	1500	AACTGCCGAGATGTTCACGT	59.966	ACAAATCGACCCGGATGCTT	60.035	201
TRINITY_Contig217	3	p3	(AGC)5	15	1964	1978	AACTGCCGAGATGTTCACGT	59.966	ACAAATCGACCCGGATGCTT	60.035	201
TRINITY_Contig218	1	p4	(CGGC)3	12	313	324	AAAGCTTCTCCAGGCATCCC	60.034	CCGCTAGCTACTGTACCAGC	59.969	111
TRINITY_Contig218	2	p4	(ACAT)3	12	757	768	AAAGCTTCTCCAGGCATCCC	60.034	CCGCTAGCTACTGTACCAGC	59.969	111
TRINITY_Contig218	3	p3	(TCG)4	12	1581	1592	AAAGCTTCTCCAGGCATCCC	60.034	CCGCTAGCTACTGTACCAGC	59.969	111
TRINITY_Contig221	1	p3	(AGC)7	21	532	552	TCGAAGGGGTAGAGGAAGGG	60.032	CTGTCTGCGCCTATGAGGAG	59.968	273
TRINITY_Contig221	2	p5	(GGAGG)3	15	1284	1298	TCGAAGGGGTAGAGGAAGGG	60.032	CTGTCTGCGCCTATGAGGAG	59.968	273
TRINITY_Contig222	1	p3	(TGA)5	15	343	357	CACCAAGTGGAAGCAACTGC	59.969	CTCAACCTCCTTGGCTTCGT	59.965	106
TRINITY_Contig223	1	c	(GCC)5ctcggacggcaccgggtccgacc(A<br>CG)4acaaggggaagaagccg(TCC)4	79	333	411	ATCATCAGGATCGCTGCTGG	59.965	AACTTTGGCACGCAATCCAC	59.968	147
TRINITY_Contig224	1	p3	(CGG)4	12	2165	2176	GCGATGGAGACGAAGACGAT	59.97	GTCACTGCTCCCTGTGGTAC	60.037	151
TRINITY_Contig225	1	p6	(CGCCGT)4	24	146	169	AAGACGATCCAACCAACGCT	59.965	CGTCGAACTTATTGCCGCTG	59.975	198
TRINITY_Contig225	2	p4	(TCAT)5	20	537	556	AAGACGATCCAACCAACGCT	59.965	CGTCGAACTTATTGCCGCTG	59.975	198

TRINITY_Contig225	3	c*	(GGCTGC)2(G<GCTGC>TGCTGCTGCGGCGGCGGCGGCGGCGCAGCAGGCCGGCGACTCGCCCGGCGCGGAGTCGCTCCGGCAGGAGCTCAAGACGCAGCGGCTCAAGGTGAAGCGCCTCAAGGACGAGTCCCTGTGGAACCAGAGCTACGAGAAGGCCGTCGGCCTCATGGCGCGCGCCGCCTGCGCGGTGTTCGTCCGCATCTGCGCCATCTTCGGCCCGTTCGTGCCCGGCCTCCCGCCGCCGTTGCCGGCGGCCACCACGGACAGCGTCCAGACCCGGCTGTCGAAGCTGCTGCACCCGCGGTCGGGGAGGGCGAGGGCGTCATCGGGGCCGATCACGCGTCGCGACGGGCCGTCCCGCGTCCACCCGCCGATGAGCAACTCGTGCCCGATCATTGGCCTCCGCCCCTCCGGCCAGAAATCCCCCACCGACT	43	1089	1131	AAGACGATCCAACCAACGCT	59.965	CGTCGAACTTATTGCCGCTG	59.975	198
TRINITY_Contig225	4	p3	(AGC)5	15	2224	2238	AAGACGATCCAACCAACGCT	59.965	CGTCGAACTTATTGCCGCTG	59.975	198
TRINITY_Contig226	1	p3	(GCG)4	12	2576	2587	TGACAGCAGGCAGACTTCTG	59.966	ATGCCAGCAATTGCACAAGG	60.037	145
TRINITY_Contig228	1	p5	(CATCA)3	15	165	179	CCCAGCAGCGAGTAGATCAG	59.968	TTCAAGAACCAGTCCTCGGC	59.965	236
TRINITY_Contig230	1	p4	(CTGC)3	12	1036	1047	AGCTGGCCTGGAGTTTTACC	59.962	AGCAGGTGGGTTCGCAATAA	59.962	220
TRINITY_Contig230	2	p4	(TGAA)3	12	2356	2367	AGCTGGCCTGGAGTTTTACC	59.962	AGCAGGTGGGTTCGCAATAA	59.962	220
TRINITY_Contig231	1	p3	(CCG)4	12	2169	2180	AGGTTGACGTGCTGGATGAG	60.037	GGAGTCCTCCCTGGTAGTGT	59.959	216
TRINITY_Contig232	1	p3	(CGC)4	12	165	176	ATATTGGAGGAGCAGCGAGC	59.965	CTGACGGCCCTCATTTCCTT	60.035	204
TRINITY_Contig232	2	p6	(CTGCTC)3	18	520	537	ATATTGGAGGAGCAGCGAGC	59.965	CTGACGGCCCTCATTTCCTT	60.035	204
TRINITY_Contig232	3	p4	(TGGT)3	12	2181	2192	ATATTGGAGGAGCAGCGAGC	59.965	CTGACGGCCCTCATTTCCTT	60.035	204
TRINITY_Contig233	1	p3	(GCT)4	12	993	1004	AAATTTTGTTCGGGGGCAGC	59.965	CGATAAGTTGCACGTCGCTG	59.976	192
TRINITY_Contig233	2	p3	(CTA)5	15	1441	1455	AAATTTTGTTCGGGGGCAGC	59.965	CGATAAGTTGCACGTCGCTG	59.976	192
TRINITY_Contig233	3	p3	(CTG)7	21	1592	1612	AAATTTTGTTCGGGGGCAGC	59.965	CGATAAGTTGCACGTCGCTG	59.976	192
TRINITY_Contig233	4	p4	(GCGA)3	12	2388	2399	AAATTTTGTTCGGGGGCAGC	59.965	CGATAAGTTGCACGTCGCTG	59.976	192
TRINITY_Contig233	5	p4	(GGTG)3	12	2510	2521	AAATTTTGTTCGGGGGCAGC	59.965	CGATAAGTTGCACGTCGCTG	59.976	192
TRINITY_Contig237	1	p3	(TGG)4	12	1346	1357	AAGCAGCTCATCTTGCCCAT	60.033	CGCCAACGTTTCGGAAAGTT	59.972	107
TRINITY_Contig237	2	p4	(CAGT)3	12	2144	2155	AAGCAGCTCATCTTGCCCAT	60.033	CGCCAACGTTTCGGAAAGTT	59.972	107
TRINITY_Contig239	1	p4	(CCGG)4	16	685	700	CTGACCGGTCGACAGAACAA	59.968	CCTAGCTACCTACACGTGCG	59.971	220
TRINITY_Contig239	2	p3	(GCA)4	12	1027	1038	CTGACCGGTCGACAGAACAA	59.968	CCTAGCTACCTACACGTGCG	59.971	220
TRINITY_Contig239	3	p4	(GGAT)3	12	2172	2183	CTGACCGGTCGACAGAACAA	59.968	CCTAGCTACCTACACGTGCG	59.971	220
TRINITY_Contig240	1	p4	(TTCC)3	12	622	633	GCAGCACGATGAGCTTCATG	59.972	GCCCCTGAAGAACGTCAAGA	59.965	194
TRINITY_Contig240	2	p5	(GCGGC)3	15	1858	1872	GCAGCACGATGAGCTTCATG	59.972	GCCCCTGAAGAACGTCAAGA	59.965	194
TRINITY_Contig241	1	p3	(CCG)4	12	898	909	CCAACGAGACAGACGGTTCA	59.968	TGACCAAGTCCGTGTACGTG	59.969	163
TRINITY_Contig241	2	c	(CCG)4cgcgcggaggagggggagaagga(GCC)5	50	1552	1601	CCAACGAGACAGACGGTTCA	59.968	TGACCAAGTCCGTGTACGTG	59.969	163
TRINITY_Contig241	3	c	(CTGC)4ctgtgggtgtgtcggt(GCG)4	44	1986	2029	CCAACGAGACAGACGGTTCA	59.968	TGACCAAGTCCGTGTACGTG	59.969	163
TRINITY_Contig242	1	p4	(AAAC)5	20	75	94	AGACAGAGTGTGCTGCTGTC	59.967	CGTAGGTGGGTGGAGTAGGA	60.034	201
TRINITY_Contig243	1	p3	(CCG)6	18	52	69	CCAGAGCGTGAACCTCAAGT	59.966	CGAAGTCGACGAGGTAGACG	59.975	254
TRINITY_Contig244	1	p3	(CCG)4	12	2068	2079	TCCTCCGACTTCTGCAGAGA	59.962	GGTGCTTCCTGCTCTTGTCT	59.964	248
TRINITY_Contig244	2	p3	(CCG)4	12	2258	2269	TCCTCCGACTTCTGCAGAGA	59.962	GGTGCTTCCTGCTCTTGTCT	59.964	248
TRINITY_Contig245	1	p6	(GCTCCC)3	18	143	160	CTTCTTCCTCCTCAAGGCGG	60.108	AGGAGCACAGAGGAGGTAGG	60.033	161
TRINITY_Contig246	1	p2	(GA)15	30	4	33	GCTTTGCGCTCTTCACACAA	59.971	TTTCTCAAGGCGCTTTGTGC	59.969	122
TRINITY_Contig247	1	p3	(GAG)4	12	1386	1397	ATGGACCTGCGAATTCAGCA	60.035	TCGTTCTCATGGTGCTTGCT	59.965	142
TRINITY_Contig248	1	p3	(GCG)4	12	187	198	AGAGACCAACAGCGACACAG	59.967	CCAGATCTTCTCGTCGACCG	59.972	140
TRINITY_Contig249	1	p4	(TCCC)3	12	124	135	CAAATCGCTCCACCTTCCCT	60.035	GGAAAACCGAGAGCCCTACC	60.108	239
TRINITY_Contig249	2	p3	(GGC)4	12	581	592	CAAATCGCTCCACCTTCCCT	60.035	GGAAAACCGAGAGCCCTACC	60.108	239
TRINITY_Contig250	1	p3	(AAG)4	12	118	129	GTGCGGACCATCTACGAGAG	59.97	ACATCGTGCATGGTCACCAT	60.035	212
TRINITY_Contig250	2	c	(AGA)5ggaagaaggacgaggaaaccgatgatggtgatgcttctgttcctgttaagaagatcaagactgaggaagctgctgagacagtggaagggaaaagagtg(AGA)5aaaagaaagataaggctaaatcaggatcagcggaagtgaaggctgatgtggctgatgacaaggatggaagtgaaaagaaa(AAG)5	224	1457	1680	GTGCGGACCATCTACGAGAG	59.97	ACATCGTGCATGGTCACCAT	60.035	212
TRINITY_Contig251	1	p5	(GGGAG)4	20	2461	2480	AGATGCCTTGCACGGTAACA	59.964	GTCCCTACTCCCTAGTGGCA	60.032	152
TRINITY_Contig252	1	p3	(ACA)4	12	174	185	GTGGGATTGTCTCCATGGCA	60.034	TGGAGGATCGCAGCATCATC	59.966	270
TRINITY_Contig252	2	c	(GCG)6aggaccccattggcgacgcgagggctagctggc(CCCT)3	63	2314	2376	GTGGGATTGTCTCCATGGCA	60.034	TGGAGGATCGCAGCATCATC	59.966	270
TRINITY_Contig254	1	p3	(AGC)4	12	1950	1961	GACGCACTGCCTCTAGGATC	59.969	AGTGCACACCGAACACCTAG	59.966	102
TRINITY_Contig256	1	p3	(CGC)5	15	209	223	TGCACTAGAGCCTGAGGGAT	60.032	CATCGGCAATGGCACTCATG	59.97	254
TRINITY_Contig256	2	c	(TCC)4ttcctgcctcctcctcttcttcctattccccaccgctgacacgagcaaccttgctcggcgccggcgccgctcgcgccgcgcgagc(CCT)4	109	510	618	TGCACTAGAGCCTGAGGGAT	60.032	CATCGGCAATGGCACTCATG	59.97	254
TRINITY_Contig257	1	p3	(GTC)4	12	150	161	GTGCGGTTCAATGGCAACTT	59.968	GACTTCAGACCTGCGTGTCA	59.968	211
TRINITY_Contig258	1	p3	(TGC)4	12	68	79	TTCCTGTCGAACACCAGCTC	59.966	CTCAAGAAGAAGGGGCTGCA	59.963	200
TRINITY_Contig258	2	c	(AGT)4tgagcggcta(CTAG)3	34	788	821	TTCCTGTCGAACACCAGCTC	59.966	CTCAAGAAGAAGGGGCTGCA	59.963	200
TRINITY_Contig258	3	p3	(CGG)4	12	963	974	TTCCTGTCGAACACCAGCTC	59.966	CTCAAGAAGAAGGGGCTGCA	59.963	200
TRINITY_Contig258	4	p3	(GGC)5	15	2043	2057	TTCCTGTCGAACACCAGCTC	59.966	CTCAAGAAGAAGGGGCTGCA	59.963	200
TRINITY_Contig258	5	c	(CGG)4cttcccatccatgacggcacc(GTG)6	51	2159	2209	TTCCTGTCGAACACCAGCTC	59.966	CTCAAGAAGAAGGGGCTGCA	59.963	200
TRINITY_Contig261	1	p3	(AAG)4	12	1909	1920	CACGTCGAGGAACAGGTTGA	59.968	TTCTGCAAATGGCCCCTAGG	60.033	193
TRINITY_Contig262	1	p4	(CTCG)4	16	359	374	GCAGGGGAGAAGAAATGGCT	60.034	GGCTAGGCTAGGCTGAATGG	59.964	223
TRINITY_Contig262	2	c	(ACC)5(AGC)6	33	774	806	GCAGGGGAGAAGAAATGGCT	60.034	GGCTAGGCTAGGCTGAATGG	59.964	223
TRINITY_Contig264	1	p3	(AGG)5	15	431	445	CGCCTGGTGAATGAGAACCT	60.036	GGAGGAGGAGGCTCTTCTGA	60.033	123
TRINITY_Contig264	2	p3	(CCG)6	18	1851	1868	CGCCTGGTGAATGAGAACCT	60.036	GGAGGAGGAGGCTCTTCTGA	60.033	123
TRINITY_Contig265	1	p3	(CCG)5	15	114	128	GCCCAGCAACTACCCTGAAT	60.034	CGGCGAGCTGATTCCCTATT	59.966	202
TRINITY_Contig266	1	p3	(GCC)4	12	83	94	CTTTTGCAACTGTGGCTGCT	59.896	AAAGATGAGCCATGCCCGAA	60.034	181
TRINITY_Contig267	1	p3	(CCG)5	15	248	262	ACATGATTTCCAGCTGGGGG	60.033	CGAAACGAATAACGCCCGTC	59.976	256
TRINITY_Contig269	1	p3	(GAC)6	18	522	539	TCGACACCGTGAACAGACAG	59.97	ACACATGATGGCCTGCTTCA	59.961	168
TRINITY_Contig270	1	c	(CAT)4agctagcgtcttgtcaagacgagtcaaaaggtcgagggtggaggaaaggaaaaga(TC)7	81	636	716	CGACCTTTCATCTGGGTGCT	60.036	CTAGCTTCCCTCCTGTCCCT	60.032	129
TRINITY_Contig270	2	p3	(CGG)4	12	1155	1166	CGACCTTTCATCTGGGTGCT	60.036	CTAGCTTCCCTCCTGTCCCT	60.032	129
TRINITY_Contig270	3	p3	(GGC)4	12	1831	1842	CGACCTTTCATCTGGGTGCT	60.036	CTAGCTTCCCTCCTGTCCCT	60.032	129
TRINITY_Contig274	1	p3	(CTC)4	12	196	207	CAGCCTCTTCTCCAACGTGT	59.966	GCCGAGAATCCGACGTACAT	59.97	255
TRINITY_Contig274	2	p3	(GCC)4	12	1524	1535	CAGCCTCTTCTCCAACGTGT	59.966	GCCGAGAATCCGACGTACAT	59.97	255
TRINITY_Contig274	3	p3	(GCC)4	12	1863	1874	CAGCCTCTTCTCCAACGTGT	59.966	GCCGAGAATCCGACGTACAT	59.97	255
TRINITY_Contig275	1	c	(GGC)4gggtggccgtagtagtgcaggtggt(GCG)8ggggcg(GGT)4	79	1574	1652	GCAACCTCTGTGCCATTTGG	60.038	TGCATTCGCATCTTTCCCCT	60.034	247
TRINITY_Contig275	2	c	(GGCGTC)3gagggggaggacgagctgttgga(CGG)4cgcggcggtgggcggcgacggcggcggggctgcagccgtgccgttgggcggcggggaggtcgccggcggcgg(CGA)4	137	1940	2076	GCAACCTCTGTGCCATTTGG	60.038	TGCATTCGCATCTTTCCCCT	60.034	247
TRINITY_Contig275	3	p3	(GGA)4	12	2429	2440	GCAACCTCTGTGCCATTTGG	60.038	TGCATTCGCATCTTTCCCCT	60.034	247
TRINITY_Contig278	1	p4	(CGCC)3	12	2039	2050	CCATACTCTAAGCGGCCCAG	59.966	CTTCCGGACATCCTGACCAC	60.108	202
TRINITY_Contig279	1	p4	(CCTC)4	16	344	359	TAACAGGTGGTCCAAGCACC	59.89	ACGTGGTCGATGTTTCCCTC	60.038	271
TRINITY_Contig279	2	p4	(GGAG)4	16	2034	2049	TAACAGGTGGTCCAAGCACC	59.89	ACGTGGTCGATGTTTCCCTC	60.038	271
TRINITY_Contig280	1	p3	(GCC)4	12	1956	1967	GCTGGAAAGCAACTGGAAGC	60.039	TCGTTTGCCGGTCTTGATGA	59.966	110
TRINITY_Contig281	1	p3	(GCG)4	12	2185	2196	TATCGCCACGAGGATGCTTC	59.968	GATGTGTCGGGTGAAGGTGT	59.965	103
TRINITY_Contig282	1	p3	(TCC)5	15	656	670	CATTGGATGCTCGCGTTCTG	59.973	CTAGCCCACTGGTCAACTGG	60.036	176
TRINITY_Contig282	2	p3	(TGG)4	12	1060	1071	CATTGGATGCTCGCGTTCTG	59.973	CTAGCCCACTGGTCAACTGG	60.036	176
TRINITY_Contig282	3	p3	(CGC)4	12	1582	1593	CATTGGATGCTCGCGTTCTG	59.973	CTAGCCCACTGGTCAACTGG	60.036	176
TRINITY_Contig284	1	p4	(AGAA)3	12	1929	1940	AACTCAGCACAGGAGTCAGC	59.965	GAGTCCACTGTGCAGCTTCT	59.965	141
TRINITY_Contig284	2	p5	(AACCA)3	15	2851	2865	AACTCAGCACAGGAGTCAGC	59.965	GAGTCCACTGTGCAGCTTCT	59.965	141
TRINITY_Contig285	1	p3	(GGA)4	12	2350	2361	CTCGTCATCCTTGCCAACCT	60.036	CAGCTTCCTCTCCTCCTCCT	60.032	259
TRINITY_Contig288	1	p3	(TCC)4	12	1967	1978	CCGTTGGCATCCTTCTGAGT	60.036	GTGGGTGTCCTGTCCGTAAG	60.038	220
TRINITY_Contig288	2	p3	(CCG)4	12	2214	2225	CCGTTGGCATCCTTCTGAGT	60.036	GTGGGTGTCCTGTCCGTAAG	60.038	220

TRINITY_Contig289	1	p3	(CCG)4	12	2062	2073	CCCGTAGTCTTTTTCCGGCT	60.037	CCAACTTGGTGCCCAATGTG	59.966	125
TRINITY_Contig289	2	p2	(GC)7	14	2328	2341	CCCGTAGTCTTTTTCCGGCT	60.037	CCAACTTGGTGCCCAATGTG	59.966	125
TRINITY_Contig290	1	c	(CTT)4ccttg(GA)6	29	2689	2717	CCGCATAATCCGAGTCCGAA	59.968	ATGTGTGCACGGTTTCAAGC	59.969	154
TRINITY_Contig290	2	p3	(ATC)4	12	7251	7262	CCGCATAATCCGAGTCCGAA	59.968	ATGTGTGCACGGTTTCAAGC	59.969	154
TRINITY_Contig290	3	c	(TCA)6ctctcggagtcggagctttcatcggaatcccattcaacaaaataggcttgtccatctctcttcttcttgaagaa(CTT)4	104	7405	7508	CCGCATAATCCGAGTCCGAA	59.968	ATGTGTGCACGGTTTCAAGC	59.969	154
TRINITY_Contig291	1	p3	(ATC)4	12	1505	1516	GCAGTTTCCTCCCCCACTAC	60.035	AGGAACACTATGTGGTGCCG	60.036	130
TRINITY_Contig292	1	p3	(CGC)5	15	811	825	GATGAAGCTTTCGGGGTCCA	60.035	ATCGTGATGCTCGACGAGTC	59.972	138
TRINITY_Contig292	2	c	(CGT)4cgccggcgcctacctcctccaagtccaactcttctgccatcagctcccttctcctccgcttgct(CCG)4	88	1186	1273	GATGAAGCTTTCGGGGTCCA	60.035	ATCGTGATGCTCGACGAGTC	59.972	138
TRINITY_Contig292	3	p3	(GGC)6	18	1386	1403	GATGAAGCTTTCGGGGTCCA	60.035	ATCGTGATGCTCGACGAGTC	59.972	138
TRINITY_Contig292	4	p3	(GGC)4	12	2178	2189	GATGAAGCTTTCGGGGTCCA	60.035	ATCGTGATGCTCGACGAGTC	59.972	138
TRINITY_Contig292	5	p5	(AGTCA)4	20	2320	2339	GATGAAGCTTTCGGGGTCCA	60.035	ATCGTGATGCTCGACGAGTC	59.972	138
TRINITY_Contig293	1	c	(GCG)4cggcggtggcggagtacacgagccacgcctgcacccgcagcatcccgggccactacgtcgtgtactgggagctgctggccaccgccgagc(AGG)4	114	1622	1735	CTACGTCGTGTACTGGGAGC	59.902	GCGTAGTCCATGAGCTCCTC	59.969	207
TRINITY_Contig293	2	p4	(GTGG)3	12	2246	2257	CTACGTCGTGTACTGGGAGC	59.902	GCGTAGTCCATGAGCTCCTC	59.969	207
TRINITY_Contig294	1	p3	(GAG)4	12	1318	1329	ACAGGGTGGATGAATGCCTG	60.034	ACCGGGTACTCTGTCCTCTC	60.034	272
TRINITY_Contig294	2	p3	(CTG)4	12	2006	2017	ACAGGGTGGATGAATGCCTG	60.034	ACCGGGTACTCTGTCCTCTC	60.034	272
TRINITY_Contig295	1	p3	(TCC)4	12	121	132	ATTGCCCGGAACTGGTTGAT	59.96	CTCGTTCAGGACCTGGTTCC	60.037	152
TRINITY_Contig295	2	p3	(GCC)4	12	250	261	ATTGCCCGGAACTGGTTGAT	59.96	CTCGTTCAGGACCTGGTTCC	60.037	152
TRINITY_Contig295	3	c	(AGG)4cggtggtggatgccgtgccgctcgcggtggcgccgccgccggtgccggtgccggggaggaagagggcgagggtggaggtggtggacgt(GGA)4cgagggggaggacgacggcgac(GAG)4gccctggggtgggtcgagctgggcgcggggcggcgcgcgcccggggaggtggtggtggaggtgctgcggcgggtagcgccccgcgccgt(GGC)4	247	479	725	ATTGCCCGGAACTGGTTGAT	59.96	CTCGTTCAGGACCTGGTTCC	60.037	152
TRINITY_Contig298	1	p4	(ACCT)4	16	143	158	GCAGTTACAGGTGCCAGTCT	59.965	TGAGGCACGGCATTGTTTTG	59.968	254
TRINITY_Contig299	1	p5	(GGGAG)4	20	2305	2324	GACCCGAACTAGATGCCTCG	59.969	GTCCCTACTCCCTAGTGGCA	60.032	162
TRINITY_Contig301	1	p6	(GTCGCC)3	18	439	456	AGAAGGAAACGCATGGCAGA	59.963	CGGTGCAAACAAGATGCCAA	59.968	231
TRINITY_Contig301	2	p3	(TCG)4	12	1036	1047	AGAAGGAAACGCATGGCAGA	59.963	CGGTGCAAACAAGATGCCAA	59.968	231
TRINITY_Contig302	1	p3	(CAG)4	12	1604	1615	GAGTCCGTTTGTGCCTCAGA	59.966	TGAGACAGAAGCTGGCAGTG	59.966	145
TRINITY_Contig304	1	p3	(TTG)4	12	1189	1200	AGCCAAGTGCCCATCGTAAA	59.962	AAAGCGGCAAAGCAACAGAG	59.969	180
TRINITY_Contig306	1	p2	(GA)9	18	1836	1853	CCTGATCGCCTTCGTCTCTC	59.97	TGGGGTCTTTTGGCAGTGTT	60.033	136
TRINITY_Contig308	1	p4	(GCAG)3	12	96	107	TGCTTGTTCATCGTCGGTGA	59.967	AGATCCTTTGCCCCTTCAGC	60.034	155
TRINITY_Contig309	1	p3	(GGC)5	15	33	47	TGCCAAGAAGCAGTCTGAGG	59.964	GAGCAAACATAGCGCATGGG	59.97	242
TRINITY_Contig309	2	p3	(AGA)4	12	1706	1717	TGCCAAGAAGCAGTCTGAGG	59.964	GAGCAAACATAGCGCATGGG	59.97	242
TRINITY_Contig310	1	c	(CGCT)3ccctcac(CCG)5	34	58	91	AGTTGCAGATTGGGTACGGG	60.035	ACTCTGTTCGGCGGCTAAAA	59.966	176
TRINITY_Contig310	2	p6	(GCGCCC)5	30	618	647	AGTTGCAGATTGGGTACGGG	60.035	ACTCTGTTCGGCGGCTAAAA	59.966	176
TRINITY_Contig310	3	p3	(AAG)7	21	1054	1074	AGTTGCAGATTGGGTACGGG	60.035	ACTCTGTTCGGCGGCTAAAA	59.966	176
TRINITY_Contig310	4	p3	(GGC)4	12	1567	1578	AGTTGCAGATTGGGTACGGG	60.035	ACTCTGTTCGGCGGCTAAAA	59.966	176
TRINITY_Contig311	1	p4	(CATC)3	12	205	216	CACCCAGCACCACGAAAAAG	59.969	GTTCGTTCGTCTCGCATTCG	59.978	103
TRINITY_Contig311	2	p6	(GGCGCG)3	18	503	520	CACCCAGCACCACGAAAAAG	59.969	GTTCGTTCGTCTCGCATTCG	59.978	103
TRINITY_Contig311	3	p3	(GCC)4	12	631	642	CACCCAGCACCACGAAAAAG	59.969	GTTCGTTCGTCTCGCATTCG	59.978	103
TRINITY_Contig311	4	c	(GTGCTG)3c(TGG)4tgctgcgggtggaggcgcgacaggccggcgt(GGCG)3	74	850	923	CACCCAGCACCACGAAAAAG	59.969	GTTCGTTCGTCTCGCATTCG	59.978	103
TRINITY_Contig311	5	c	(AGC)5gaggaaacggagcaggactcaccagagggaacgggagggaggccggactccaacca(GGC)5	86	2202	2287	CACCCAGCACCACGAAAAAG	59.969	GTTCGTTCGTCTCGCATTCG	59.978	103
TRINITY_Contig313	1	p5	(CTTCC)3	15	133	147	CTGTTGCCTGTTGGATCCCT	59.961	GAAGGGAAGAAGACCGGTGG	60.036	130
TRINITY_Contig314	1	c	(GCG)4cggggtccctccgctcggccgcccacgacggct(CGG)4	57	1778	1834	AGAGCCAGCCATCCTTTCAC	60.035	TGGCGCAAACAAATGACCAG	59.968	169
TRINITY_Contig314	2	c	(CCTCGC)3cctccc(CCT)4	36	2069	2104	AGAGCCAGCCATCCTTTCAC	60.035	TGGCGCAAACAAATGACCAG	59.968	169
TRINITY_Contig314	3	p3	(CGG)4	12	2237	2248	AGAGCCAGCCATCCTTTCAC	60.035	TGGCGCAAACAAATGACCAG	59.968	169
TRINITY_Contig315	1	p6	(GGGAGC)3	18	183	200	CGATCTGCGAGCTAGCTTGA	59.97	GACTTGTCCCTCGTCCACAG	60.038	120
TRINITY_Contig315	2	p5	(CCGCG)3	15	1628	1642	CGATCTGCGAGCTAGCTTGA	59.97	GACTTGTCCCTCGTCCACAG	60.038	120
TRINITY_Contig315	3	p6	(TCCATG)3	18	1915	1932	CGATCTGCGAGCTAGCTTGA	59.97	GACTTGTCCCTCGTCCACAG	60.038	120
TRINITY_Contig316	1	p3	(CGC)5	15	398	412	CAGGGTTTTGCTTCCATGGC	60.037	TGCAGCACCAGTTCCTAGTG	59.965	173
TRINITY_Contig316	2	c	(CGG)5(CCG)4	27	517	543	CAGGGTTTTGCTTCCATGGC	60.037	TGCAGCACCAGTTCCTAGTG	59.965	173
TRINITY_Contig316	3	p6	(CGCTGC)3	18	697	714	CAGGGTTTTGCTTCCATGGC	60.037	TGCAGCACCAGTTCCTAGTG	59.965	173
TRINITY_Contig317	1	p3	(GCC)4	12	171	182	GTTCCAGAGCTACGGCAACT	60.037	CGGAGGTGTTGTGGTACTCC	60.038	173
TRINITY_Contig317	2	p3	(GGC)4	12	1322	1333	GTTCCAGAGCTACGGCAACT	60.037	CGGAGGTGTTGTGGTACTCC	60.038	173
TRINITY_Contig318	1	p3	(GAG)4	12	2087	2098	TTGCCTTCAGTGCCAAATGC	59.966	GTCATCTCCAGGGCAGATCG	59.967	173
TRINITY_Contig319	1	c	(GC)6gtgcacggctggggcgatgacgccctccgccgcgtcgggttccacgagctcgacgtcc(CCG)4	82	943	1024	CTCATCAGCGTGTGGTCCTT	60.037	GCGTGCAGTTTCGATGATCC	59.973	101
TRINITY_Contig323	1	p5	(CGCGT)3	15	26	40	GACCAATTTCCCGCTCTGGA	60.035	CGTCCCACAAATTTGCAGCA	59.968	252
TRINITY_Contig323	2	c	(CCT)5cgccgccgcctcgctcgcgcatggccgcgccatccccgccggcgcgacggtcctccggctcccgtcggaggaggcc(GCG)5	106	346	451	GACCAATTTCCCGCTCTGGA	60.035	CGTCCCACAAATTTGCAGCA	59.968	252
TRINITY_Contig327	1	p3	(CTC)5	15	649	663	GATAATGCCGAACCCAACGC	59.971	AAAGCAGGCAGAGCAAAAGC	59.967	252
TRINITY_Contig327	2	p3	(GCG)4	12	3327	3338	GATAATGCCGAACCCAACGC	59.971	AAAGCAGGCAGAGCAAAAGC	59.967	252
TRINITY_Contig328	1	p6	(GCCTCA)4	24	938	961	CCAGGTTCCTGGTCTTCACC	59.963	CTAGCGTGAAATTGTCGGCG	59.975	274
TRINITY_Contig328	2	p3	(TGG)4	12	2211	2222	CCAGGTTCCTGGTCTTCACC	59.963	CTAGCGTGAAATTGTCGGCG	59.975	274
TRINITY_Contig329	1	p6	(GCCGGC)3	18	1289	1306	TCATCCCTCGCTCTCCTCAA	60.033	TCTCGAGTTCTTGGTTGGGC	59.965	188
TRINITY_Contig329	2	c	(GTGA)3ggccgtgtggcgtgaaccacaca(GCCG)3	47	1940	1986	TCATCCCTCGCTCTCCTCAA	60.033	TCTCGAGTTCTTGGTTGGGC	59.965	188
TRINITY_Contig330	1	c	(CCG)4tcccctccgccaccatcacctcctccgccgtctcctccacctccatccccatcaccaccgccaccttcacctcctccgccct(CTC)4catccccatcaccacctcctccttctcccccaccgccgtctcctccacctccatccccatcc(CCT)4	180	1312	1491	CCTCAACTCCAACCGCTTCT	59.965	TCCCTCGAACTCGTTGAAGC	60.039	169
TRINITY_Contig330	2	p3	(CCT)4	12	1687	1698	CCTCAACTCCAACCGCTTCT	59.965	TCCCTCGAACTCGTTGAAGC	60.039	169
TRINITY_Contig331	1	p2	(GC)6	12	119	130	TCAACAGCCTCAGCAGGAAG	59.964	TGGTTGATCGTGTGGCTACC	60.037	106
TRINITY_Contig331	2	p6	(TCCCCG)3	18	297	314	TCAACAGCCTCAGCAGGAAG	59.964	TGGTTGATCGTGTGGCTACC	60.037	106
TRINITY_Contig331	3	p3	(GCG)4	12	552	563	TCAACAGCCTCAGCAGGAAG	59.964	TGGTTGATCGTGTGGCTACC	60.037	106
TRINITY_Contig333	1	p3	(CGT)4	12	398	409	CGATCAGACGCTCATCGACA	59.972	CCGTCGATAGCTAACCCACC	59.968	223
TRINITY_Contig333	2	p4	(ATTG)3	12	1991	2002	CGATCAGACGCTCATCGACA	59.972	CCGTCGATAGCTAACCCACC	59.968	223
TRINITY_Contig333	3	p4	(AATC)3	12	2388	2399	CGATCAGACGCTCATCGACA	59.972	CCGTCGATAGCTAACCCACC	59.968	223
TRINITY_Contig333	4	p3	(CGG)4	12	4177	4188	CGATCAGACGCTCATCGACA	59.972	CCGTCGATAGCTAACCCACC	59.968	223
TRINITY_Contig335	1	p4	(TTTG)3	12	1735	1746	CGAGCCCTAATGTTGGTGGT	60.035	GCACGCATCCATCTCGAAAC	59.973	114
TRINITY_Contig336	1	p4	(AAGG)3	12	2028	2039	AATGGCACTAACAGGAGGGC	60.034	GAGAAGACACCAGGAAGGCC	60.036	244
TRINITY_Contig337	1	p3	(GGA)4	12	2169	2180	GCAGACCGTTGAGTTGAGGA	59.966	ACGCATTTTGTTGGACTGCC	59.968	170
TRINITY_Contig339	1	p4	(CGAA)3	12	57	68	GACACCAGGGAAGCGAAGAA	59.965	GGTTGCAGCAAAGTCACTGG	59.969	174
TRINITY_Contig339	2	p3	(CCG)5	15	1606	1620	GACACCAGGGAAGCGAAGAA	59.965	GGTTGCAGCAAAGTCACTGG	59.969	174
TRINITY_Contig339	3	p4	(CGAT)3	12	1867	1878	GACACCAGGGAAGCGAAGAA	59.965	GGTTGCAGCAAAGTCACTGG	59.969	174
TRINITY_Contig340	1	p3	(AGA)4	12	1032	1043	AATGGGAGGAGAAAAGCGGG	60.034	ATGGGAAAAGGGGAGAAGCG	60.034	248
TRINITY_Contig340	2	p3	(CGG)4	12	1541	1552	AATGGGAGGAGAAAAGCGGG	60.034	ATGGGAAAAGGGGAGAAGCG	60.034	248
TRINITY_Contig340	3	c	(AGG)4tggagcagctgcttcgggagcaccagctccgagtagcccaccgggaagcccatgccgcggccgggacggga(CGCT)4cgtccggatttgctggcgtgtctgtctcccggctctc(GGT)5	151	2002	2152	AATGGGAGGAGAAAAGCGGG	60.034	ATGGGAAAAGGGGAGAAGCG	60.034	248
TRINITY_Contig342	1	p3	(TCC)5	15	203	217	TCTCTGCCTCCCTCTCTCAC	60.034	TTCAGCTGGCCTAGGGTTTG	59.962	237
TRINITY_Contig344	1	p3	(CGG)5	15	2121	2135	AGATGCACTTGACATGGGGG	60.034	TGCACGATGCCTTCTGAAGT	59.965	138
TRINITY_Contig345	1	p3	(AGA)4	12	2395	2406	GAACTCGTCCACCTCAGCAA	59.966	TGGTGCAAGAGGCTGAGAAG	59.964	224
TRINITY_Contig347	1	p3	(CGG)6	18	594	611	TCTGAAGGTTCCCCGCAATC	60.035	GCAGCGGAAATGCGAACTAG	59.973	101
TRINITY_Contig347	2	p3	(GCC)4	12	1899	1910	TCTGAAGGTTCCCCGCAATC	60.035	GCAGCGGAAATGCGAACTAG	59.973	101
TRINITY_Contig348	1	p3	(CCG)4	12	52	63	AGGAAAAGAATCTGCCCCCG	60.034	GCATTGTGCAACTGAAGCCA	59.968	262

TRINITY_Contig348	2	p3	(GCC)4	12	320	331	AGGAAAAGAATCTGCCCCCG	60.034	GCATTGTGCAACTGAAGCCA	59.968	262
TRINITY_Contig349	1	p3	(CCG)4	12	278	289	GAGATGGCGGAAGTAGACGG	59.969	CCCCTCTCCATCCTCAGACA	60.032	231
TRINITY_Contig349	2	p3	(GGA)5	15	1065	1079	GAGATGGCGGAAGTAGACGG	59.969	CCCCTCTCCATCCTCAGACA	60.032	231
TRINITY_Contig350	1	p4	(CCGG)3	12	1256	1267	CGAGTACATCTCCCGGAACG	59.971	CGAGACGAAGGAGGTGATCG	59.972	172
TRINITY_Contig351	1	p4	(GGAC)4	16	834	849	CAGACAACCGACACCCATCA	59.965	CACTCCAGCCTACACTGCAA	59.965	179
TRINITY_Contig353	1	c	(CCCTT)3ccctccgatctcctccttcct(CCG)4	48	30	77	TGTGGAATAACTCGGCCACC	60.036	ATTCTGTGTTGGTTGCGTGC	59.969	105
TRINITY_Contig354	1	p3	(GCG)5	15	869	883	GTTCTTCCGATGTGAGGCCA	60.036	ATCTCACCACTCACCAAGCG	60.037	236
TRINITY_Contig354	2	p3	(GGC)5	15	1561	1575	GTTCTTCCGATGTGAGGCCA	60.036	ATCTCACCACTCACCAAGCG	60.037	236
TRINITY_Contig355	1	p4	(GCGA)3	12	1399	1410	AATTTTGTTCGCCCATGCCC	60.036	ATTTTCCGACGACAGCGACT	60.039	175
TRINITY_Contig356	1	p3	(ATC)4	12	1634	1645	TGGAGAGCCATCCTGGAAGA	59.957	GTGGACTTTTCAGGAGGCGA	59.965	228
TRINITY_Contig357	1	p3	(GCA)4	12	1423	1434	TTCCTACGCTACCTCGACCA	60.035	CGCTTCTTCTCGTAGGCGAT	59.97	275
TRINITY_Contig357	2	p6	(CCCGCA)3	18	1585	1602	TTCCTACGCTACCTCGACCA	60.035	CGCTTCTTCTCGTAGGCGAT	59.97	275
TRINITY_Contig360	1	p3	(CGC)5	15	1801	1815	AGGGATAGGTACAGCAGCCA	60.031	CCTCCAGTTCGAAGCCTCAG	60.108	119
TRINITY_Contig360	2	p3	(CGC)4	12	2028	2039	AGGGATAGGTACAGCAGCCA	60.031	CCTCCAGTTCGAAGCCTCAG	60.108	119
TRINITY_Contig361	1	c	(CCA)4ctaccatagccacctccgccaaag(CA)7	57	643	699	GTGGAATGGGGGAAGAGTGG	60.034	TCGTGGACTTAACTGGCACC	59.966	223
TRINITY_Contig361	2	p3	(CCT)4	12	1282	1293	GTGGAATGGGGGAAGAGTGG	60.034	TCGTGGACTTAACTGGCACC	59.966	223
TRINITY_Contig362	1	p4	(TCCC)3	12	340	351	AGCTTTTATTCCCCTGGGCC	60.032	GATTAGCGCGACCTGCTAGT	59.969	147
TRINITY_Contig362	2	p3	(CGC)4	12	1512	1523	AGCTTTTATTCCCCTGGGCC	60.032	GATTAGCGCGACCTGCTAGT	59.969	147
TRINITY_Contig364	1	p3	(GGC)4	12	552	563	CACCGTTGCTGTTGTGGAAG	59.971	CTGGTCCCTTTGATCTGCGT	60.036	231
TRINITY_Contig364	2	c	(CTG)4ttgtggaggaagaggcggatatcgtggccattgcgc(CTG)5	63	889	951	CACCGTTGCTGTTGTGGAAG	59.971	CTGGTCCCTTTGATCTGCGT	60.036	231
TRINITY_Contig364	3	p3	(GCT)4	12	1077	1088	CACCGTTGCTGTTGTGGAAG	59.971	CTGGTCCCTTTGATCTGCGT	60.036	231
TRINITY_Contig365	1	p3	(GGA)4	12	1771	1782	TAGTACCACTCCTCCACGGG	60.034	GGCAGGGGATTCCACATTGA	60.033	203
TRINITY_Contig365	2	c	(GCGG)3ggaccagcggggaggcgagcaccgcggcgcgcacggccgtgatctccgcgggggtgagcgggtcgagcgggtgggggcgcgaggag(GCC)4gctgtgacggagagcacggc(GAG)4	142	1962	2103	TAGTACCACTCCTCCACGGG	60.034	GGCAGGGGATTCCACATTGA	60.033	203
TRINITY_Contig366	1	p3	(CGG)4	12	1325	1336	ATTCAGCCAGAGTGCCTGAC	60.036	GACACTGCAATGAGGTGGGA	59.963	173
TRINITY_Contig369	1	p3	(CTG)6	18	486	503	GCTTGCTCAGAATCGCGAAG	59.974	AGCTCCACCAAGAATGAGGC	60.035	148
TRINITY_Contig370	1	p5	(GAAGA)4	20	1819	1838	AGGAGATCACAGAGGACCCC	60.032	GATCTCCTTCTCCCTGGGGT	60.031	116
TRINITY_Contig371	1	p3	(GGC)4	12	2072	2083	ACAGCAGCAAACGATCCTCA	59.965	AACTTGCTAGAGCTTGGCGT	59.965	263
TRINITY_Contig372	1	p3	(CCT)6	18	1712	1729	ACGGGAGCTACAATTGTGCA	59.964	TTCAAGCCACGCTCGTGTAT	60.038	146
TRINITY_Contig374	1	p3	(GCC)5	15	113	127	TGATGCTGTTGTTGCTGCAC	59.969	CTGGCAACACACGGTTCAAG	59.971	251
TRINITY_Contig374	2	c	(CCGCG)3ctggcggcggccgcgctcctgctcttcctcgtcgccgccgtcgcgctgctcttcgtctccccgccgccggtctcggaccaccccgtggtcgcctcctc(CCG)5ctcggcccatgctttgctgaacagctc(CGG)5	170	235	404	TGATGCTGTTGTTGCTGCAC	59.969	CTGGCAACACACGGTTCAAG	59.971	251
TRINITY_Contig375	1	p3	(GGC)5	15	957	971	TTCGGCGGTTGTGAAGATGA	59.966	GAAGATGGAGGAGTACCGCG	59.969	259
TRINITY_Contig375	2	p3	(GCC)5	15	1235	1249	TTCGGCGGTTGTGAAGATGA	59.966	GAAGATGGAGGAGTACCGCG	59.969	259
TRINITY_Contig375	3	p2	(TC)7	14	2123	2136	TTCGGCGGTTGTGAAGATGA	59.966	GAAGATGGAGGAGTACCGCG	59.969	259
TRINITY_Contig376	1	c	(TCGGC)3ttcccctcctggatctgccgcccggtggtacctcgccgccggtggcaagcatggcgcgggcggagaagaagcgcgccgctatct(CGG)5	114	28	141	CGGCTGGATTCCTCTACGTC	59.969	CATGGAGCAATGTCATGGCG	59.97	240
TRINITY_Contig378	1	p3	(CGC)4	12	1348	1359	CCACCACTTTCTTCTCGCCT	59.965	CCCGAGGAGAACTTCACCAC	60.037	214
TRINITY_Contig381	1	c*	(GGC)3(<GGC>GGA)(GGCGGA)2gcggctcatcgcgcggctgccgctgttcacgctgtcctcgtcgctagcgtccgtgcccaagtcgtcgcgcgactgcgccgtgtgccagagcgcgttccg(CGA)4	138	668	805	GGAGCATAGCACTCTCGACC	59.969	GAGAGGAGTGCCCACTTCAC	60.037	196
TRINITY_Contig381	2	p3	(CAG)5	15	1149	1163	GGAGCATAGCACTCTCGACC	59.969	GAGAGGAGTGCCCACTTCAC	60.037	196
TRINITY_Contig381	3	p3	(CGG)4	12	1559	1570	GGAGCATAGCACTCTCGACC	59.969	GAGAGGAGTGCCCACTTCAC	60.037	196
TRINITY_Contig385	1	p4	(TTGC)3	12	886	897	TAGTGAAGGCGAGCATTGCA	60.036	AAATACCAGGGAGCCAAGGC	60.033	203
TRINITY_Contig386	1	p3	(CGC)4	12	1048	1059	CCCGCACCTCAAAACTCTCT	59.965	TTTGTACGCCTTTTGCCGTG	59.97	107
TRINITY_Contig386	2	c	(CCGG)3attccctcctgcgctgcggatcggcaggggaataaagagcaaagggacggaaggaa(GGCG)4cggtggggaaggaaggggaagagggaggaggagtggccgcagtaaagacggggctctttaaaaagaggagcagaaatggtgcaggagcagg(AGC)4	186	1429	1614	CCCGCACCTCAAAACTCTCT	59.965	TTTGTACGCCTTTTGCCGTG	59.97	107
TRINITY_Contig386	3	c*	(GCG)3(GCGGTGGCGGTGGCGGTGGCGGCGACGTGAGGTGGAGGCCGAGGAGTGGAGCGCTTGACGGGATGGGAGTTTCTGGGGGTTTGGGGAGGTTTTTG<GGCG>GT)(GGCGGT)2	26	1992	2017	CCCGCACCTCAAAACTCTCT	59.965	TTTGTACGCCTTTTGCCGTG	59.97	107
TRINITY_Contig390	1	p3	(CTT)4	12	888	899	GTGTCCTTGTTAGGGGTCGG	60.037	CCTTGACAACTGGCACAACG	59.971	153
TRINITY_Contig390	2	p6	(GACGGC)3	18	1639	1656	GTGTCCTTGTTAGGGGTCGG	60.037	CCTTGACAACTGGCACAACG	59.971	153
TRINITY_Contig391	1	c	(GACCA)3cccagcagccagagctactactagtagtggtagtcgtt(TTC)4	65	152	216	CAGCAGCACCTCCTTCATCA	60.036	TAGCAGCACCATTGACCTGG	60.035	208
TRINITY_Contig391	2	p3	(AGC)4	12	329	340	CAGCAGCACCTCCTTCATCA	60.036	TAGCAGCACCATTGACCTGG	60.035	208
TRINITY_Contig391	3	c	(TCC)5tggagatcttcttcgacat(CTG)5ctccaggtcaatggtgctgctactcccagccccagcc(GCA)4	98	471	568	CAGCAGCACCTCCTTCATCA	60.036	TAGCAGCACCATTGACCTGG	60.035	208
TRINITY_Contig391	4	p3	(CTT)4	12	1105	1116	CAGCAGCACCTCCTTCATCA	60.036	TAGCAGCACCATTGACCTGG	60.035	208
TRINITY_Contig391	5	p4	(ATGC)3	12	2073	2084	CAGCAGCACCTCCTTCATCA	60.036	TAGCAGCACCATTGACCTGG	60.035	208
TRINITY_Contig392	1	p3	(TCA)8	24	3351	3374	TCCGCTGATGAGATCGCTTC	59.969	ATGAGGAGGATGAGGTGCCT	60.03	108
TRINITY_Contig392	2	p3	(CTC)4	12	3668	3679	TCCGCTGATGAGATCGCTTC	59.969	ATGAGGAGGATGAGGTGCCT	60.03	108
TRINITY_Contig392	3	p3	(TCG)4	12	4332	4343	TCCGCTGATGAGATCGCTTC	59.969	ATGAGGAGGATGAGGTGCCT	60.03	108
TRINITY_Contig392	4	p4	(GGGA)3	12	4639	4650	TCCGCTGATGAGATCGCTTC	59.969	ATGAGGAGGATGAGGTGCCT	60.03	108
TRINITY_Contig394	1	p3	(CGC)4	12	228	239	CTGATCGCTAGGAGCATGCA	59.967	CTTGAGCGTGATGGGGAAGT	60.036	138
TRINITY_Contig394	2	p3	(AGG)4	12	1683	1694	CTGATCGCTAGGAGCATGCA	59.967	CTTGAGCGTGATGGGGAAGT	60.036	138
TRINITY_Contig397	1	p3	(CGG)5	15	2014	2028	CATTTGCCTCTGGAGCTGGA	60.035	GCGACGGCTGCATTAAGAAG	59.973	220
TRINITY_Contig398	1	p3	(GGC)5	15	571	585	TGTCAATCAGGCCGGACTTC	60.036	TCCATCACCTCTTTCCACGC	60.036	241
TRINITY_Contig398	2	p3	(GCG)6	18	739	756	TGTCAATCAGGCCGGACTTC	60.036	TCCATCACCTCTTTCCACGC	60.036	241
TRINITY_Contig398	3	c	(CGT)4cgacctccggcgggggtcctgaacggctgaactcccttgattctttgggaacatgatcgcattgcgtgcccccgctat(TGC)5	105	1556	1660	TGTCAATCAGGCCGGACTTC	60.036	TCCATCACCTCTTTCCACGC	60.036	241
TRINITY_Contig399	1	p3	(CGG)5	15	276	290	CTCCCCCATGATGCTCGATC	60.037	CATCGATCCATCCTTCCCG	60.037	128
TRINITY_Contig399	2	p4	(GGAG)3	12	1927	1938	CTCCCCCATGATGCTCGATC	60.037	CATCGATCCATCCTTCCCG	60.037	128
TRINITY_Contig400	1	c	(GCG)6(GCA)6	36	82	117	GCGTAGTGGTTGCAAGTGTG	60.041	TCTTCTTCGACGCGACACAA	59.97	138
TRINITY_Contig400	2	p4	(TCAT)3	12	2020	2031	GCGTAGTGGTTGCAAGTGTG	60.041	TCTTCTTCGACGCGACACAA	59.97	138
TRINITY_Contig401	1	c	(CGG)5(CGA)4	27	214	240	AATGCATCTGGGCTTGCTCT	60.033	TAGCCAGCCTATGTGGGACT	60.031	114
TRINITY_Contig402	1	p3	(GAC)4	12	629	640	GGACTTCAGGAACGTGCTGA	59.966	ACCTGCTTCATCCTCAAGCC	60.035	111
TRINITY_Contig402	2	c	(GCG)4cctggaaggggaa(GGC)4	37	843	879	GGACTTCAGGAACGTGCTGA	59.966	ACCTGCTTCATCCTCAAGCC	60.035	111
TRINITY_Contig402	3	p3	(GTG)4	12	1484	1495	GGACTTCAGGAACGTGCTGA	59.966	ACCTGCTTCATCCTCAAGCC	60.035	111
TRINITY_Contig403	1	p3	(CAG)5	15	1189	1203	ACGTTCTTCTGTGACCGCAT	59.966	TATAGCTAGTGTGGCGCGTG	59.97	224
TRINITY_Contig408	1	p3	(CGC)7	21	142	162	GAACAAGCTCTGACCCGTCA	59.966	TTGACCCTCATGTTCTCCGC	60.036	266
TRINITY_Contig408	2	p6	(CGGCCC)3	18	354	371	GAACAAGCTCTGACCCGTCA	59.966	TTGACCCTCATGTTCTCCGC	60.036	266
TRINITY_Contig409	1	p3	(ACG)4	12	390	401	AGCATGGATGGGAGTCTCCT	60.03	GTCACTTGGCTTCAGGCTCT	59.964	198
TRINITY_Contig409	2	c	(CCAT)3ggatctacctgctctatttttactctgctgctgctatcctttgcgtacgcggtgcatccgggaaaaggggaggaaaatttcgcg(TTC)5	111	908	1018	AGCATGGATGGGAGTCTCCT	60.03	GTCACTTGGCTTCAGGCTCT	59.964	198
TRINITY_Contig411	1	p4	(GGAT)3	12	461	472	CTCACCCCTCCACCAATGAC	60.035	ATGCTGGTGCTGTCTGAGTC	60.037	270
TRINITY_Contig412	1	p4	(CTAG)3	12	108	119	GAAACGCATGATCCTTCCGC	59.972	GAGGGGAGGAACAACCATGG	60.034	176
TRINITY_Contig414	1	p5	(ACCCA)3	15	150	164	TCCCCAACAAACCGCTAGTC	59.964	TGCCACAGCTAGAGCACTTC	60.037	206
TRINITY_Contig414	2	p3	(CGG)5	15	552	566	TCCCCAACAAACCGCTAGTC	59.964	TGCCACAGCTAGAGCACTTC	60.037	206
TRINITY_Contig414	3	p3	(AGC)4	12	1281	1292	TCCCCAACAAACCGCTAGTC	59.964	TGCCACAGCTAGAGCACTTC	60.037	206
TRINITY_Contig414	4	p4	(CATC)3	12	1511	1522	TCCCCAACAAACCGCTAGTC	59.964	TGCCACAGCTAGAGCACTTC	60.037	206
TRINITY_Contig418	1	p5	(AGAGG)5	25	1970	1994	ACCGAATCGAGCACCAAAGT	59.965	TTGGGCATCAACTTTGCACG	59.968	117

TRINITY_Contig421	1	p3	(AGC)4	12	70	81	CGACCACAGGAAGACGAACA	59.968	ATGTTGTCCCGTGTGAGCTT	59.891	129
TRINITY_Contig424	1	p3	(GGT)4	12	990	1001	GGTGGTCTCTTTCTGGGAGC	60.036	CTGTCGATGTCATCCGGGAG	59.969	116
TRINITY_Contig424	2	p3	(GCC)5	15	1876	1890	GGTGGTCTCTTTCTGGGAGC	60.036	CTGTCGATGTCATCCGGGAG	59.969	116
TRINITY_Contig425	1	p6	(TCTCCG)3	18	485	502	AGCACAGCATCCCACTTCTC	60.036	ACTTGCGGATTAACCAGCCA	59.962	238
TRINITY_Contig425	2	c	(CGG)6ccttcttcttgttcttggcagcggcggcgggcctgctcttgcgcgccttctgcttctt(CGGC)3	88	674	761	AGCACAGCATCCCACTTCTC	60.036	ACTTGCGGATTAACCAGCCA	59.962	238
TRINITY_Contig425	3	p5	(GGGCG)3	15	1227	1241	AGCACAGCATCCCACTTCTC	60.036	ACTTGCGGATTAACCAGCCA	59.962	238
TRINITY_Contig426	1	p3	(ACC)6	18	1732	1749	GCAAAACAGCCCTTGGTGAG	59.967	AAGATGTGGGGCGAGAACTG	60.036	103
TRINITY_Contig429	1	p3	(GCG)4	12	1752	1763	TTTGCGGCTGATGATGTCCT	60.035	CAAGCTGCTCTACAAGGCCT	60.036	168
TRINITY_Contig430	1	p6	(GTTCGA)3	18	1365	1382	AGGCGTCGTCATTCCACAAT	60.036	ACAAGGCCAAGGAGATGCTC	60.035	262
TRINITY_Contig432	1	p3	(GCG)4	12	196	207	TGAGTGAGGGGCTTCGTTTC	59.965	GCAACGGATCGGAGCATAGA	59.968	223
TRINITY_Contig432	2	p4	(AGCA)4	16	773	788	TGAGTGAGGGGCTTCGTTTC	59.965	GCAACGGATCGGAGCATAGA	59.968	223
TRINITY_Contig433	1	p3	(GGC)4	12	289	300	AAGTAGCTTGCGGAGTTGCT	59.965	GCCACCGGAAGAGTCTTCAA	59.965	253
TRINITY_Contig434	1	p3	(CGC)4	12	1455	1466	GAGACTGGATGATGGAGCGG	59.967	TTTGCTTCTCATGTCCGGCT	59.963	170
TRINITY_Contig434	2	c	(GGA)4tggagacggc(GCCT)3	34	1679	1712	GAGACTGGATGATGGAGCGG	59.967	TTTGCTTCTCATGTCCGGCT	59.963	170
TRINITY_Contig435	1	c	(TCCC)3caacatgatgggccgtccgtccggtgcgcggagcgcgct(CCGG)3	63	1280	1342	AATCCAGGGAGCTGGGATCT	60.029	TTGCCCACAATTGCACGAAG	59.968	216
TRINITY_Contig435	2	c	(TCG)4a(GGAT)3	25	1879	1903	AATCCAGGGAGCTGGGATCT	60.029	TTGCCCACAATTGCACGAAG	59.968	216
TRINITY_Contig436	1	p3	(GGC)5	15	389	403	AACTGCCTGTTCTCCGACAG	59.966	ATTCGTCGCGGCATGAAATG	59.972	234
TRINITY_Contig438	1	p4	(CAGC)3	12	73	84	ATCACTCTCCAGTCCTCCCC	60.032	CTTGACCTTGTGCTTGCACC	59.969	251
TRINITY_Contig438	2	c	(CAG)4agcaacaacctcatggcgagcatgcaggggtaccatcatcacccagcggcgacggcggcgatgatgaacaatttgaggggcctgaacagcaac(ATG)4	117	1355	1471	ATCACTCTCCAGTCCTCCCC	60.032	CTTGACCTTGTGCTTGCACC	59.969	251
TRINITY_Contig438	3	p5	(CTTTT)7	35	1731	1765	ATCACTCTCCAGTCCTCCCC	60.032	CTTGACCTTGTGCTTGCACC	59.969	251
TRINITY_Contig439	1	c	(CGGC)3ggcgtgggagagcgacggggagcgggcga(GTGCGT)3	59	1723	1781	ATAGCAGTGGCCGGTACATG	59.893	CCCTCGTCGGAGATTTAGCC	59.968	166
TRINITY_Contig440	1	p5	(GGAGC)3	15	1842	1856	AGGGCTGTACTGTGAGGACA	60.179	GAGGGATGCGCTGTCGATTA	59.968	200
TRINITY_Contig442	1	p3	(AAG)5	15	1588	1602	TCCTAAACCCTAGCCCCTCC	60.03	CGGTCATGATCCCTTCGGAG	59.967	264
TRINITY_Contig443	1	p3	(GTC)5	15	1526	1540	CCACTGGAGCCACTGACATT	59.963	ACTTGGTGACTGCTTCTCCG	59.966	100
TRINITY_Contig447	1	p3	(CCG)4	12	435	446	AGGAACACCGTCATCTGCTG	60.037	AGGGAGCTCCTCAAGACGAT	60.033	269
TRINITY_Contig449	1	p3	(GGC)4	12	1273	1284	GACTACGTATGCGACCTGGG	59.97	CGTCCGGATCAAGATGGAGG	59.967	103
TRINITY_Contig449	2	p4	(CCGC)3	12	3024	3035	GACTACGTATGCGACCTGGG	59.97	CGTCCGGATCAAGATGGAGG	59.967	103
TRINITY_Contig451	1	c	(GCA)6gccacaggcgcggcaaccaccttcccgttcgtctcctccttccaccgcccgcgcctgcgtccctgc(CCGCG)3	99	451	549	GTCCATGAGATCAGCCCGAG	59.967	GATCGCGGGGAGTAAGTGAG	59.969	200
TRINITY_Contig451	2	p3	(GGT)4	12	876	887	GTCCATGAGATCAGCCCGAG	59.967	GATCGCGGGGAGTAAGTGAG	59.969	200
TRINITY_Contig452	1	p3	(CTG)4	12	1707	1718	GAGAGCAGGATCTGGATGCC	59.965	GCATACCTCGGGGTTTCCAA	60.035	212
TRINITY_Contig452	2	p4	(CGGC)3	12	1893	1904	GAGAGCAGGATCTGGATGCC	59.965	GCATACCTCGGGGTTTCCAA	60.035	212
TRINITY_Contig456	1	p6	(CTCCAA)3	18	140	157	AGGGACGCCATCAGAACATG	60.108	TCCGCCATTACTCATCTCGC	59.968	145
TRINITY_Contig456	2	p4	(GGAA)3	12	686	697	AGGGACGCCATCAGAACATG	60.108	TCCGCCATTACTCATCTCGC	59.968	145
TRINITY_Contig456	3	p3	(GCG)4	12	1069	1080	AGGGACGCCATCAGAACATG	60.108	TCCGCCATTACTCATCTCGC	59.968	145
TRINITY_Contig456	4	p3	(TGG)4	12	1271	1282	AGGGACGCCATCAGAACATG	60.108	TCCGCCATTACTCATCTCGC	59.968	145
TRINITY_Contig456	5	p6	(CGTCTT)3	18	1470	1487	AGGGACGCCATCAGAACATG	60.108	TCCGCCATTACTCATCTCGC	59.968	145
TRINITY_Contig458	1	p3	(TTC)4	12	645	656	GCCTGGCGTTTGTTTAAGCA	59.969	GCGTAATGCGGGTGGATAGA	59.967	120
TRINITY_Contig458	2	p3	(GTC)6	18	1256	1273	GCCTGGCGTTTGTTTAAGCA	59.969	GCGTAATGCGGGTGGATAGA	59.967	120
TRINITY_Contig458	3	c	(CTT)5gagggactcgagcgccgcgaacacgttgccggtgttgagcttcacctcgtccg(CCGCC)3	83	1415	1497	GCCTGGCGTTTGTTTAAGCA	59.969	GCGTAATGCGGGTGGATAGA	59.967	120
TRINITY_Contig460	1	p3	(CGT)4	12	374	385	GTCTGGTGACCGTAACACGT	59.969	TTCCTTTCCATGGGTCAGGC	59.96	159
TRINITY_Contig461	1	p5	(GAAAA)5	25	1874	1898	CTGGAAGCCACACAACAAGC	59.969	CCCTCGACAAGAACCTTGCT	59.965	255
TRINITY_Contig464	1	p3	(CCG)4	12	559	570	AGCAATGGAGCACGGAATCA	60.035	AGCTTGTGTTCGTCGTGGAT	59.966	111
TRINITY_Contig464	2	p3	(CCG)5	15	1298	1312	AGCAATGGAGCACGGAATCA	60.035	AGCTTGTGTTCGTCGTGGAT	59.966	111
TRINITY_Contig465	1	c	(TCC)5(GCG)4	27	392	418	CGCGACTTCACCTACATCGA	59.902	GGTGTTGCCGAGGTTGTAGA	59.966	135
TRINITY_Contig465	2	p3	(CGT)4	12	864	875	CGCGACTTCACCTACATCGA	59.902	GGTGTTGCCGAGGTTGTAGA	59.966	135
TRINITY_Contig465	3	c	(AAG)4gccgccgccgcggccatgtcggcggcgtcgtgatcaatcgatcacagcacacagagacagag(CAC)4	86	1544	1629	CGCGACTTCACCTACATCGA	59.902	GGTGTTGCCGAGGTTGTAGA	59.966	135
TRINITY_Contig465	4	p3	(GAT)4	12	1827	1838	CGCGACTTCACCTACATCGA	59.902	GGTGTTGCCGAGGTTGTAGA	59.966	135
TRINITY_Contig466	1	p3	(TCC)4	12	538	549	TCTTTGATGCGGGACCTCAC	60.036	CACCCATGCCAACAAGGTTG	59.966	222
TRINITY_Contig466	2	p3	(CGC)4	12	863	874	TCTTTGATGCGGGACCTCAC	60.036	CACCCATGCCAACAAGGTTG	59.966	222
TRINITY_Contig466	3	p6	(GCCGGA)4	24	1461	1484	TCTTTGATGCGGGACCTCAC	60.036	CACCCATGCCAACAAGGTTG	59.966	222
TRINITY_Contig467	1	p4	(CCCA)3	12	48	59	TCGTGGGACTCATACGGTCT	60.035	TCCGGACTGAAACCAAGCTC	59.965	273
TRINITY_Contig467	2	p3	(GCG)4	12	2803	2814	TCGTGGGACTCATACGGTCT	60.035	TCCGGACTGAAACCAAGCTC	59.965	273
TRINITY_Contig468	1	p4	(CCGC)3	12	437	448	CTCAGTTGAGAGCTGGTGGG	60.037	CCCTTGCCCTCCAGTACTTG	60.035	197
TRINITY_Contig470	1	p3	(CGC)4	12	1284	1295	AGATGGAATGCTTTGGCCGA	60.034	CCCTTGTCCCACATGATGCT	60.034	116
TRINITY_Contig470	2	p5	(ACACA)3	15	1577	1591	AGATGGAATGCTTTGGCCGA	60.034	CCCTTGTCCCACATGATGCT	60.034	116
TRINITY_Contig472	1	p6	(GAGGCC)4	24	1619	1642	GCGTCCAAACACAGCACAAT	59.969	ACATTCCGTGCTCCGAACTT	59.965	120
TRINITY_Contig473	1	p5	(CCCCG)3	15	146	160	ATGGAGTGCCCCTTCTTTCG	60.035	GTGCCAAGTCTCTCTGGACC	60.037	157
TRINITY_Contig473	2	p4	(ATTA)3	12	1632	1643	ATGGAGTGCCCCTTCTTTCG	60.035	GTGCCAAGTCTCTCTGGACC	60.037	157
TRINITY_Contig474	1	p3	(CGG)5	15	336	350	TAGTTGCTGGCGGCTATAGC	59.966	GCCTGTTGTTTGCTACCAGC	60.039	107
TRINITY_Contig475	1	p3	(CGC)4	12	671	682	CTACATCCACCTCGTCGACG	59.972	GAGTCCAGACCATCAGCTCG	59.898	279
TRINITY_Contig476	1	p3	(CGG)5	15	477	491	CCTTGTTTCTGCGGTGTGTG	59.971	AGCAACAGTGTCGACCGAAT	59.966	141
TRINITY_Contig476	2	p3	(CAC)4	12	1309	1320	CCTTGTTTCTGCGGTGTGTG	59.971	AGCAACAGTGTCGACCGAAT	59.966	141
TRINITY_Contig478	1	p3	(GAG)4	12	1697	1708	GGATCGTCAGAATGCGTTGC	59.973	AGGATGCATTGTCGCAAGGA	60.035	219
TRINITY_Contig480	1	p2	(CT)11	22	29	50	AGCGGTTGGTTCTGCGATTA	60.037	CCATCAAAGGGTACACGCCT	60.035	215
TRINITY_Contig481	1	p3	(CCG)4	12	808	819	TCATGACCTCCTCCCCTCTG	60.032	TCAAGGTCGAGCCCTTCAAC	59.965	163
TRINITY_Contig483	1	p3	(CGC)5	15	426	440	ACTCCGGCCTGTCATCTTTG	60.036	GGATCCCATCGTGGACATCC	59.965	192
TRINITY_Contig484	1	p2	(CG)6	12	106	117	TCCAGCCAAGCCAAGATCTG	60.035	GGACGCACCAGGAATACACA	60.037	109
TRINITY_Contig485	1	p6	(GCCACG)3	18	1493	1510	TGCTGAGTTGACAAAACGCG	59.973	CAAAGATGGAACGCTCCCCT	60.035	191
TRINITY_Contig486	1	c	(CGG)4ccatggcgcatcagaacccggcgccgttcgtctacctcga(CGC)4	64	108	171	CCTCAAGTTCTGGCAGGAGG	60.036	CCTGGGCACTCTGGAAGAAG	60.036	131
TRINITY_Contig487	1	c	(CGG)4tgccgccggcgcccgcgcagaagaggg(TGA)4	51	1498	1548	TCCACACGACCACACGTTAG	59.969	ACAGGAAGGCGATGCAATGA	60.035	127
TRINITY_Contig487	2	p4	(CTGC)3	12	1803	1814	TCCACACGACCACACGTTAG	59.969	ACAGGAAGGCGATGCAATGA	60.035	127
TRINITY_Contig488	1	p5	(CTTTT)3	15	1844	1858	GACGAAGAGAACCTTGCGGA	60.039	GGTTCCTGTTCGTGGTGGAT	59.964	266
TRINITY_Contig489	1	p3	(GGC)5	15	787	801	ATATAGTGCTTGGGCGGCTC	59.965	GAAATCCCTGGAGCCGGAAA	60.034	150
TRINITY_Contig491	1	c	(ACACCC)3acagttggtgtgctgtggtgtgcaaaaata(TCTCG)3tgagctgaggtaccagatcaccaagc(TGTGGG)3tgttgtggccgtcggctcccctcgctgccccgccgttggttggtctatccggaatccatccgccccgcgcgcgtctccgccacccaaat(CCCTC)3ttcttatcctccaaatcccagaccacgca(CGCC)3accagcttccctcccagtccaagcctcc(AAGCA)3	295	1	295	CCTTTGACACACGCTGTTGG	59.971	AAGTACAACTCAGCCACCCG	59.965	231
TRINITY_Contig491	2	p3	(TTC)4	12	605	616	CCTTTGACACACGCTGTTGG	59.971	AAGTACAACTCAGCCACCCG	59.965	231
TRINITY_Contig493	1	p5	(GAGCA)3	15	158	172	CGACATCTCGCTCACCAAGA	59.829	TGAGGAGGAGATGGTGGAGG	60.032	259
TRINITY_Contig495	1	p5	(CTCCC)3	15	69	83	TCGGATCATATTCCACGGCG	60.039	TCCAACCAACGCCATTACGA	59.965	153
TRINITY_Contig495	2	p3	(GGC)5	15	250	264	TCGGATCATATTCCACGGCG	60.039	TCCAACCAACGCCATTACGA	59.965	153
TRINITY_Contig495	3	c	(GCCG)3tgcccgctcgacgcgctctgggcgccctacgacct(CCG)4	59	1138	1196	TCGGATCATATTCCACGGCG	60.039	TCCAACCAACGCCATTACGA	59.965	153
TRINITY_Contig498	1	p3	(GCC)4	12	545	556	GTACAGGTTTCCGGTGGAGG	60.037	ACGCCCTTCTGTTGGACAAT	59.89	135
TRINITY_Contig500	1	p3	(GTC)4	12	1783	1794	TGGATGACCGCCAGAGAGTA	60.034	GATTCTACTGCCTCGGGAGC	59.967	100
TRINITY_Contig501	1	p3	(GCC)4	12	1865	1876	TCATTTCCTGGATGCGCAGT	60.035	AAGCAAGACCATCCTCCTGC	60.035	185
TRINITY_Contig503	1	p3	(GCC)6	18	175	192	ACAAGAGCAGACACGCTCTC	60.039	AGGATCCATAGCACCAGGGT	60.03	133
TRINITY_Contig503	2	p3	(TCT)4	12	666	677	ACAAGAGCAGACACGCTCTC	60.039	AGGATCCATAGCACCAGGGT	60.03	133
TRINITY_Contig503	3	p3	(CGC)4	12	895	906	ACAAGAGCAGACACGCTCTC	60.039	AGGATCCATAGCACCAGGGT	60.03	133
TRINITY_Contig507	1	p3	(AGC)4	12	803	814	CGGGCTGAACTGTGTCTCTT	59.966	TCTCCATGTACCCAGCGGTA	60.033	207
TRINITY_Contig507	2	p3	(GCC)8	24	1316	1339	CGGGCTGAACTGTGTCTCTT	59.966	TCTCCATGTACCCAGCGGTA	60.033	207

TRINITY_Contig507	3	c*	(CTCC)2(CTCCTCCTCCTCCCGGTCC<br>CGGCCCGGCACCTCTCTCTGACCAA<br>CGTTGCAAGCCCGGCAAGAGCTGC<br>CGCGCGCACCGGTAAGGAGCAGCA<br>GGCGGTCGCTCCCTCTAATCAGAA<br>GACGCAAGGTGCGTGGCGCTCCTC<br>GCGGCGGGCTGAACTGTGTCTCTT<br>CTCTTCCTCTCGCTCGCTCTCTTCCT<br>CCGCCCTCAAAAGACGAGAGGGGG<br>CGTAGGTTTATAAGCTGGACGCCG<br>GGTGGTTGCCGTCTTGGGTGCAGT<br>TTGCCGGTTCGTTCGCTCCCCTCCT<br>GTATGGGCATGGGCGCATGGCTTC<br>CTGTCTGCTCCTACGCTGCGTACCG<br>CTGGGTACATGGAGAGAGGCTAGC<br>TGTCTAAATCATCGTCTCGTTGGGC<br>CTGTCGCGGATTAGCGGAGCACGG<br>ACGGAC<CCTCC>TCCTCCTCCCGG	19	1446	1464	CGGGCTGAACTGTGTCTCTT	59.966	TCTCCATGTACCCAGCGGTA	60.033	207
TRINITY_Contig511	1	p3	(GCT)4	12	818	829	AAATGAACGCCGACCTGTCT	59.965	AGCAGCAGCCTTGTTGAGAT	59.962	247
TRINITY_Contig512	1	c	(GCCGCG)3gcgccggcggcggtggagga<br>gaggagtgggggcgtcggggaggcggggtgc<br>gggcgggtgtcgaggaggaggaccgggcgct<br>tccgcgg(GGC)4	119	1498	1616	AGGCAGCAATCTTAGCAGCA	60.035	TGGGTTCGTGGAGATCAAGC	60.036	188
TRINITY_Contig514	1	p3	(GCG)4	12	1826	1837	TGGTCGGTTGTTGTGGTGAA	60.035	TGTAGCAATGCGGACTGGTT	59.964	242
TRINITY_Contig516	1	p3	(CCG)4	12	15	26	GGGCTCCTACACCATCATCG	59.966	TTCTCGACTGTGAGCACCAC	59.968	114
TRINITY_Contig516	2	p6	(GAGGCC)3	18	451	468	GGGCTCCTACACCATCATCG	59.966	TTCTCGACTGTGAGCACCAC	59.968	114
TRINITY_Contig516	3	p3	(AAG)4	12	1618	1629	GGGCTCCTACACCATCATCG	59.966	TTCTCGACTGTGAGCACCAC	59.968	114
TRINITY_Contig517	1	p5	(GCGCG)3	15	538	552	TGACGAAGGTGACCTTGACG	59.968	CAAGCTCAAACCACTGCCAC	59.969	166
TRINITY_Contig523	1	p3	(CTG)4	12	271	282	TCGTCTCAGGCTTGGACAAC	59.966	ATTGGCATGGGACAACCGAT	60.033	211
TRINITY_Contig524	1	p3	(CGC)4	12	1602	1613	CATCGAGCAAAGCCATGACG	59.973	CTACAGAGGGCTGAGGAGGT	60.033	277
TRINITY_Contig525	1	p3	(CCG)4	12	863	874	ACGAATCCCTTGACGCCAAT	60.035	AGAGGCTGGCCAAGATGTTC	60.035	178
TRINITY_Contig525	2	p3	(GAG)5	15	1622	1636	ACGAATCCCTTGACGCCAAT	60.035	AGAGGCTGGCCAAGATGTTC	60.035	178
TRINITY_Contig526	1	p4	(AAGA)4	16	78	93	CCAGCCGTGTGAGACTCTTT	59.966	AGCACACGATGGCTTACACA	59.965	122
TRINITY_Contig526	2	p3	(CCG)4	12	469	480	CCAGCCGTGTGAGACTCTTT	59.966	AGCACACGATGGCTTACACA	59.965	122
TRINITY_Contig526	3	p3	(CGC)4	12	704	715	CCAGCCGTGTGAGACTCTTT	59.966	AGCACACGATGGCTTACACA	59.965	122
TRINITY_Contig527	1	p4	(CTGG)3	12	1594	1605	CCGGATTCTAGGCACCGATC	60.038	AACGCGAGCACAGATCAGAA	60.038	116
TRINITY_Contig528	1	p3	(GCC)5	15	1509	1523	CGATGTTGTTGAGCACCCAC	59.762	ACAAGTGTCGCCATCCTAGC	60.108	278
TRINITY_Contig531	1	p3	(GTG)4	12	1500	1511	GACAAGCAGCCCAGAGGAAT	60.035	GTGGTGAGTGGTGACAGAGG	59.966	161
TRINITY_Contig532	1	p2	(AG)6	12	162	173	CTGGATTCCGTCGAGTCTCG	59.972	ACCTCTCCACACTAGGACCC	59.959	194
TRINITY_Contig532	2	c	(GTCGA)3ggcgagcagtacgcagccgccg<br>cgc(CCCCCG)3	58	510	567	CTGGATTCCGTCGAGTCTCG	59.972	ACCTCTCCACACTAGGACCC	59.959	194
TRINITY_Contig533	1	p3	(GGC)4	12	594	605	GTCCGGACTGCTCAACTTCA	59.966	GTATCTCCGGGAACAGCTCG	59.969	221
TRINITY_Contig533	2	p3	(ACC)4	12	766	777	GTCCGGACTGCTCAACTTCA	59.966	GTATCTCCGGGAACAGCTCG	59.969	221
TRINITY_Contig535	1	p4	(GGCG)3	12	1727	1738	TTGAGCGGTGGAAGGAGTTC	59.965	ATGGATTTCCCGAGCCGATC	59.965	181
TRINITY_Contig536	1	p3	(CGC)5	15	35	49	TCAAAGCTGCGTTTCAAGCC	59.969	ACCTTCTGGCAATCCCATGG	60.033	153
TRINITY_Contig537	1	p3	(GGA)6	18	1684	1701	CCCTTTTTGGCGGTTCAGTG	59.968	TCCGTTGTCCATGGAACCTG	59.964	191
TRINITY_Contig539	1	p6	(ATGCCC)3	18	597	614	TCTGGTGGCAAGTCGAAGTC	59.966	TCCGTCTCGACCAACAAAGG	59.967	209
TRINITY_Contig540	1	p3	(GCG)5	15	89	103	TGACTGTGCAAGCGTGATCT	59.966	CAGTGCCACGCAACAGAAAA	59.9	241
TRINITY_Contig540	2	p4	(CCGC)3	12	464	475	TGACTGTGCAAGCGTGATCT	59.966	CAGTGCCACGCAACAGAAAA	59.9	241
TRINITY_Contig541	1	p6	(ATCAGC)3	18	246	263	GGGCTTGTACTTGACGTCCA	59.966	AGCCCAGGGTTAGGAGGTAG	60.031	115
TRINITY_Contig541	2	p2	(CT)6	12	449	460	GGGCTTGTACTTGACGTCCA	59.966	AGCCCAGGGTTAGGAGGTAG	60.031	115
TRINITY_Contig541	3	c	(GCG)5gggacaccgaccgctcctcgtcgtac<br>tcggagccgtcc(GAGTCG)3	71	1096	1166	GGGCTTGTACTTGACGTCCA	59.966	AGCCCAGGGTTAGGAGGTAG	60.031	115
TRINITY_Contig542	1	p3	(GCG)6	18	177	194	CAAGGGTTGAGTGCACTTGC	59.969	TCAATGGCTGCCTAAGTGGG	60.034	240
TRINITY_Contig543	1	p6	(CAGCCA)3	18	1027	1044	TTGCAAGATGACGGACGTGA	59.967	ACCCTTCCCGCAATGCTTAA	59.961	188
TRINITY_Contig543	2	p3	(CTC)5	15	1312	1326	TTGCAAGATGACGGACGTGA	59.967	ACCCTTCCCGCAATGCTTAA	59.961	188
TRINITY_Contig543	3	p3	(GCG)4	12	1644	1655	TTGCAAGATGACGGACGTGA	59.967	ACCCTTCCCGCAATGCTTAA	59.961	188
TRINITY_Contig546	1	p3	(CGA)4	12	129	140	CGAGAACATCCAGCCATCGA	59.897	CGGGCGCTTTTGAGTTTTGA	59.97	162
TRINITY_Contig546	2	p3	(GAG)5	15	815	829	CGAGAACATCCAGCCATCGA	59.897	CGGGCGCTTTTGAGTTTTGA	59.97	162
TRINITY_Contig550	1	p4	(TGGC)3	12	285	296	CGCACTGTGAGTCCATGACT	60.038	TTCCGTCCGTTGCAGTCTAC	60.039	189
TRINITY_Contig550	2	p3	(TGG)5	15	674	688	CGCACTGTGAGTCCATGACT	60.038	TTCCGTCCGTTGCAGTCTAC	60.039	189
TRINITY_Contig550	3	p3	(CCG)4	12	1286	1297	CGCACTGTGAGTCCATGACT	60.038	TTCCGTCCGTTGCAGTCTAC	60.039	189
TRINITY_Contig550	4	p4	(CCTC)3	12	1445	1456	CGCACTGTGAGTCCATGACT	60.038	TTCCGTCCGTTGCAGTCTAC	60.039	189
TRINITY_Contig551	1	p3	(CCG)4	12	210	221	GCATCCTCCTCCTCATGCTC	59.965	AGGTCATCTTGTGCGAGCAA	59.965	159
TRINITY_Contig552	1	p3	(GCG)5	15	773	787	CAACGAACAAGCGGATCGAC	59.907	ACGGATGGAGGGTAGAGCAT	60.105	133
TRINITY_Contig552	2	p4	(GGCG)3	12	1610	1621	CAACGAACAAGCGGATCGAC	59.907	ACGGATGGAGGGTAGAGCAT	60.105	133
TRINITY_Contig553	1	p3	(GAG)6	18	1661	1678	CCATAAGATGGCAAAGGCGC	59.969	TCACGGGAAGCTTCAACCTC	59.965	118
TRINITY_Contig555	1	p3	(CGG)4	12	1504	1515	TGGACAGGGAGATCAGGAGG	60.032	CCCGGCGAAGAAGAAGTTCT	60.037	188
TRINITY_Contig557	1	p3	(CCG)5	15	59	73	CGTCTTCGTACTCGCCTCTC	59.973	CATCAGCCTGAATGCCATGC	59.968	194
TRINITY_Contig559	1	p4	(CCCG)3	12	339	350	CGAGTTCACCGTGGTCATGA	60.039	AGCTTCAGGTTGCTACGACC	60.037	243
TRINITY_Contig559	2	p3	(CGG)4	12	877	888	CGAGTTCACCGTGGTCATGA	60.039	AGCTTCAGGTTGCTACGACC	60.037	243
TRINITY_Contig560	1	p3	(GCA)4	12	101	112	CGGCTCCATGCATGACATTG	59.97	CTTGAGACGCCGTACCTTGT	60.039	103
TRINITY_Contig560	2	p3	(CGC)4	12	278	289	CGGCTCCATGCATGACATTG	59.97	CTTGAGACGCCGTACCTTGT	60.039	103
TRINITY_Contig560	3	p6	(CGCGCT)3	18	461	478	CGGCTCCATGCATGACATTG	59.97	CTTGAGACGCCGTACCTTGT	60.039	103
TRINITY_Contig561	1	p3	(GAC)5	15	371	385	GGAAGAGGAGGAGGACGGAT	60.106	GGCAGGAGAGGCATGTTTCT	60.035	254
TRINITY_Contig561	2	p3	(CGG)5	15	871	885	GGAAGAGGAGGAGGACGGAT	60.106	GGCAGGAGAGGCATGTTTCT	60.035	254
TRINITY_Contig561	3	p3	(GAG)5	15	1547	1561	GGAAGAGGAGGAGGACGGAT	60.106	GGCAGGAGAGGCATGTTTCT	60.035	254
TRINITY_Contig562	1	p4	(CTTT)3	12	3259	3270	CCACCCGAGCTGGAAAGAAT	60.035	AGCAGAGCACGCTTCTCTTT	59.965	116
TRINITY_Contig562	2	c	(GCG)4gccat(GGCGC)3	32	4015	4046	CCACCCGAGCTGGAAAGAAT	60.035	AGCAGAGCACGCTTCTCTTT	59.965	116
TRINITY_Contig565	1	p3	(CGC)5	15	47	61	AAGTCCGGGATCAGCTACCT	60.032	TGCCCATACGTTCACGAACA	59.966	156
TRINITY_Contig567	1	p3	(AGA)5	15	84	98	CATCGGAATCCCCCTCATCG	60.037	TCCGGATGGAGGCCTTAGAA	60.032	192
TRINITY_Contig567	2	p3	(TAA)4	12	795	806	CATCGGAATCCCCCTCATCG	60.037	TCCGGATGGAGGCCTTAGAA	60.032	192
TRINITY_Contig569	1	p4	(GCAG)3	12	780	791	GTCTGCTGCAAGAACAAGCC	60.04	GTACGAAACGAGGCGATTGC	59.975	222
TRINITY_Contig572	1	p3	(TCC)5	15	276	290	GCCATGATCATCTCGCCGTA	60.038	GACAGGCTGACACTTCTCCC	60.037	253
TRINITY_Contig573	1	p4	(CGCA)3	12	1759	1770	CTGCGTCTGCCGTTTTGAAA	59.971	TGTTGACCTGTGATGGCTCC	59.963	107
TRINITY_Contig574	1	p3	(GGT)4	12	1309	1320	ATTGCCACACATGCAAGCAG	60.038	GTCCGATTCAGCCGAGAGAG	59.97	241
TRINITY_Contig574	2	p3	(GCG)4	12	1522	1533	ATTGCCACACATGCAAGCAG	60.038	GTCCGATTCAGCCGAGAGAG	59.97	241
TRINITY_Contig575	1	p4	(GGAA)3	12	1742	1753	TGTAGGGCACGGTGTAGAGA	59.961	TTCGTGGAGCTCATGGTCAC	60.037	136
TRINITY_Contig576	1	p6	(CCTCCA)3	18	1215	1232	TCGAGAGCTTGTGTTGTCCC	59.966	CTCCGTGATCGTCTTCCTCG	59.972	273
TRINITY_Contig579	1	p3	(TCT)4	12	409	420	GGACACAGAACGAGCTTCCA	59.966	TTACTGGATGGCTTGGGCTG	60.034	113
TRINITY_Contig579	2	p4	(TGTA)3	12	1409	1420	GGACACAGAACGAGCTTCCA	59.966	TTACTGGATGGCTTGGGCTG	60.034	113
TRINITY_Contig580	1	p3	(TCT)4	12	97	108	TGCGTGCTCCACCTTCTTAG	60.037	CGTAGCTGCTAGTGGAGTCG	59.972	193
TRINITY_Contig580	2	p3	(GGC)5	15	1379	1393	TGCGTGCTCCACCTTCTTAG	60.037	CGTAGCTGCTAGTGGAGTCG	59.972	193
TRINITY_Contig581	1	p3	(CGG)5	15	819	833	TCCCTTCCACAGCTTCGTTC	59.965	CGCTGCTGGATGGATCATCT	59.965	194
TRINITY_Contig581	2	p2	(AT)6	12	1572	1583	TCCCTTCCACAGCTTCGTTC	59.965	CGCTGCTGGATGGATCATCT	59.965	194
TRINITY_Contig583	1	p3	(GCG)5	15	422	436	GACGACAACTGCAAGATCGC	59.906	ATCGAGCAGGTGCCAAATCA	60.035	121
TRINITY_Contig584	1	p3	(CTC)4	12	319	330	TCGCGATACTCTGCGTCTTC	59.972	CTTGCTCACGCCTTTGCTTT	59.969	241
TRINITY_Contig587	1	p3	(TCA)6	18	1166	1183	CAGCAACACACCCACACATG	59.969	GAGAAGGGGACTGTTGGAGC	60.036	126
TRINITY_Contig587	2	c	(CGG)4ggcctggggctcctctagcggcgcg<br>cccgcgttctccgacgaggaggccatgggcgg<br>cgtgcaga(GCG)8gattccccttctcagcgc<br>ggtgagtggggaagg(GGAA)3	146	1633	1778	CAGCAACACACCCACACATG	59.969	GAGAAGGGGACTGTTGGAGC	60.036	126
TRINITY_Contig588	1	p3	(CGG)4	12	611	622	CGCTCAGCAAAAATCCCGAG	59.902	CACTCACTCGATGGATCCGG	59.969	234
TRINITY_Contig588	2	p3	(GCG)4	12	1156	1167	CGCTCAGCAAAAATCCCGAG	59.902	CACTCACTCGATGGATCCGG	59.969	234
TRINITY_Contig589	1	p6	(CCGGCG)3	18	944	961	CTTGCATTGGGGAGCCCTAA	60.033	CTTCCGTTTTTGAGGCCACG	60.04	212
TRINITY_Contig589	2	p3	(TCG)4	12	1288	1299	CTTGCATTGGGGAGCCCTAA	60.033	CTTCCGTTTTTGAGGCCACG	60.04	212
TRINITY_Contig593	1	p3	(GCC)4	12	816	827	GACACCGTCATCAGGCTCAA	60.037	TCCTGTCCGAGTCCATCCAT	60.032	233
TRINITY_Contig594	1	p3	(CTC)4	12	553	564	TGTGCAGCGGATGAACTCAT	60.036	GCTGCCGCGAAGATAAGTTG	59.973	227
TRINITY_Contig594	2	p5	(GAGGG)4	20	1671	1690	TGTGCAGCGGATGAACTCAT	60.036	GCTGCCGCGAAGATAAGTTG	59.973	227
TRINITY_Contig596	1	p3	(CGC)5	15	146	160	GCCAAAGACACCAAGCTGTG	59.969	AGCTGAGAAGGACACACTGC	59.965	168
TRINITY_Contig597	1	p3	(TCC)4	12	111	122	AGATGCAGCAGCTTGGATGT	60.034	GCAAGAACCTTCAGTGCTGC	60.04	109
TRINITY_Contig601	1	p3	(CGC)5	15	881	895	TGGTCCCCGGAATACCATCT	60.031	CCTGCAAACATGCAACTGCT	59.967	212

TRINITY_Contig601	2	c	(GCA)6gtagttcgggcacgcgtcctgcgtgcgcgcccgcgccatggccgcgaaccgcgtggttccgtccgctccatggcggattcgaga(TTCC)9	138	1298	1435	TGGTCCCCGGAATACCATCT	60.031	CCTGCAAACATGCAACTGCT	59.967	212
TRINITY_Contig603	1	p4	(CCAT)3	12	359	370	CATTCGATTTCAACCCCGGC	59.899	ACATGTGCCACAGTGAGGAG	59.964	170
TRINITY_Contig603	2	p6	(GACGGG)3	18	1362	1379	CATTCGATTTCAACCCCGGC	59.899	ACATGTGCCACAGTGAGGAG	59.964	170
TRINITY_Contig605	1	p2	(CT)22	44	87	130	TGTCGAAGCACTCCAACTCC	59.966	AGCAAAACAAAGACGGCGAC	59.971	269
TRINITY_Contig605	2	c	(CGA)5ggaggtggtggtggagggcgagggcgagggtagtaaggcgggggattgt(GGCGCC)3	82	327	408	TGTCGAAGCACTCCAACTCC	59.966	AGCAAAACAAAGACGGCGAC	59.971	269
TRINITY_Contig605	3	p3	(CGC)6	18	960	977	TGTCGAAGCACTCCAACTCC	59.966	AGCAAAACAAAGACGGCGAC	59.971	269
TRINITY_Contig607	1	p6	(CGGAGG)3	18	1373	1390	AAGATGAACGGCCAGCATCA	60.035	GGTTGTGAAAGAGGCGGAGA	59.965	212
TRINITY_Contig608	1	p3	(GGC)5	15	1631	1645	TTGCAGTAGCCCAAGACGAG	60.037	CACATACGGGACCCTCATCG	59.968	243
TRINITY_Contig609	1	p3	(CGA)4	12	208	219	CAACTGCTTCAGGGGGAGGAG	60.036	CCACTGCTTGACATGGACCT	59.963	250
TRINITY_Contig610	1	c	(GCC)4accgtct(CCG)5	34	94	127	GCTGCTGCTATTGCCATGTC	59.97	ACTGTGTTGGTGAAAGCCCA	60.034	275
TRINITY_Contig611	1	p4	(AAGA)3	12	323	334	CTTGACACGGTCATCAGGCT	60.037	TCCAATATCGCGCTCTTGCT	59.895	254
TRINITY_Contig613	1	p2	(CT)13	26	1638	1663	GTATCTGGACTCGTCGCTGG	59.97	AGGAGATGGAGGAGGTGGTC	60.032	110
TRINITY_Contig614	1	p2	(GC)6	12	43	54	CTCGGCACTGGAGGATTACC	59.895	ACACCAATAGCTTCCGCGAA	60.037	176
TRINITY_Contig614	2	p3	(TGT)4	12	743	754	CTCGGCACTGGAGGATTACC	59.895	ACACCAATAGCTTCCGCGAA	60.037	176
TRINITY_Contig616	1	p4	(GATT)3	12	144	155	TGCTTTAGCTTCCGTGCTCA	59.965	TGAACAGGACGTCCTCTTGC	59.966	111
TRINITY_Contig618	1	p3	(GGA)6	18	115	132	TTCCATGACAACGAGTGGGG	59.964	AGCAACTCACGTGCGTTTTC	59.972	263
TRINITY_Contig618	2	p6	(ACGGCC)3	18	331	348	TTCCATGACAACGAGTGGGG	59.964	AGCAACTCACGTGCGTTTTC	59.972	263
TRINITY_Contig620	1	p3	(CCA)4	12	417	428	GAGTCGATCGTCTCCGGAAC	59.972	GTCGACACTGACCGTTCCTT	59.968	227
TRINITY_Contig621	1	p3	(CGC)4	12	1374	1385	TGAGCAGCATGTCGTCTTGT	59.966	AACGATTGAGCCACGTGACT	59.966	216
TRINITY_Contig627	1	p3	(CGC)4	12	406	417	CGGTGAGAGGATGAAGACGG	59.899	CCGGCGCCTCTTCTTCTTTA	60.108	167
TRINITY_Contig627	2	c	(GCAGCC)3gcagagctgaacacgtcgccgcgagggttgacctgccgcctgcggcggtggccggcggtgcggggaggtcgatgcat(CTC)5	110	1203	1312	CGGTGAGAGGATGAAGACGG	59.899	CCGGCGCCTCTTCTTCTTTA	60.108	167
TRINITY_Contig628	1	p4	(GATC)3	12	63	74	GATCGGAGAGCATGCACGTA	59.969	CAGGCTCAGGATGTCGTTGT	60.037	264
TRINITY_Contig628	2	p3	(GGC)4	12	194	205	GATCGGAGAGCATGCACGTA	59.969	CAGGCTCAGGATGTCGTTGT	60.037	264
TRINITY_Contig628	3	p3	(CGG)4	12	634	645	GATCGGAGAGCATGCACGTA	59.969	CAGGCTCAGGATGTCGTTGT	60.037	264
TRINITY_Contig628	4	c	(CGG)5tttcggca(GCG)4tgaggtcatcgccaacatcatcaaacacgaa(CGG)4	78	998	1075	GATCGGAGAGCATGCACGTA	59.969	CAGGCTCAGGATGTCGTTGT	60.037	264
TRINITY_Contig629	1	p4	(CTCC)4	16	280	295	GATCGTCTTCTCGCACGGAT	59.97	GTCGAAGCCCACCATGTACA	60.037	100
TRINITY_Contig629	2	p3	(CGC)6	18	518	535	GATCGTCTTCTCGCACGGAT	59.97	GTCGAAGCCCACCATGTACA	60.037	100
TRINITY_Contig630	1	p3	(CCG)4	12	224	235	AGGAAACAGCCGGTGCAATA	59.962	GTCCTCCAGAAGACAGGCAC	60.037	209
TRINITY_Contig631	1	p3	(CGC)4	12	1307	1318	CCATTTTGTCCTTGCAGGCC	60.037	CCAGTGAAGTCCCTATCGCG	60.249	141
TRINITY_Contig634	1	c	(GAGCAG)3gagggg(CGT)4	36	448	483	CCACGCCTCAAGGTTGTAGT	59.965	CGTCGATCTGGTCTCTGCTC	59.971	130
TRINITY_Contig636	1	p3	(CTT)4	12	1556	1567	CCGCACAAGGTTTTTCAGGG	59.968	GCTCAAGACTTCCAGCACCT	59.964	237
TRINITY_Contig637	1	c	(GCT)4agtggctg(TGC)4	32	26	57	AGGAAGAAGAGGATCCCGCT	60.032	GGGGATTCTCGATCACGGTC	59.968	129
TRINITY_Contig638	1	c	(CGA)6(CGC)6	36	1402	1437	GACGGTGGTGACGAAAGAGT	59.968	CAACAGGTCTCTGCTACCCG	60.109	154
TRINITY_Contig640	1	p4	(GGGT)4	16	128	143	CAACAACCAGTCACAAGGCG	59.971	GTAGCTGCTATTGCCGTTGC	59.972	232
TRINITY_Contig640	2	p3	(GCG)4	12	1420	1431	CAACAACCAGTCACAAGGCG	59.971	GTAGCTGCTATTGCCGTTGC	59.972	232
TRINITY_Contig641	1	p3	(CCA)4	12	58	69	GGAGTTCTGCAGGCCTCATT	60.035	CTCCGTGATATCACCCCTGC	59.966	160
TRINITY_Contig643	1	p5	(CCTCC)3	15	65	79	AGATGGGCATGACCAAGGTG	60.034	CCGGTATGGGCATAGTCCAC	59.965	106
TRINITY_Contig643	2	c	(TCC)4accaccagcgccccaagtcgctcctgccccgcatcctcgccgtcgacccggcgtcgggccgccgcgtgctgctc(CCG)4	98	522	619	AGATGGGCATGACCAAGGTG	60.034	CCGGTATGGGCATAGTCCAC	59.965	106
TRINITY_Contig644	1	p3	(GGC)4	12	1455	1466	CGGACCTCGTTTGCTAGGTT	60.038	AGAGTTCGAAGGGAGGAGCT	59.96	161
TRINITY_Contig646	1	p4	(CCGG)4	16	28	43	ATCTTCCTGGTCATGGCTGC	60.107	TCCTCCTCCTCCTTCGATCC	59.814	230
TRINITY_Contig646	2	p3	(CGC)4	12	372	383	ATCTTCCTGGTCATGGCTGC	60.107	TCCTCCTCCTCCTTCGATCC	59.814	230
TRINITY_Contig646	3	p3	(AGG)4	12	686	697	ATCTTCCTGGTCATGGCTGC	60.107	TCCTCCTCCTCCTTCGATCC	59.814	230
TRINITY_Contig648	1	p3	(TGC)4	12	377	388	TGATCTTGGCCTTCCACGTC	60.036	TCTCTAGGGAGCTTGGGACC	60.032	173
TRINITY_Contig649	1	p3	(CGC)5	15	1117	1131	CTCGGTTTCCACGGACTTGA	59.967	ACCAACGGCAGGTACAAGAG	59.965	252
TRINITY_Contig650	1	p3	(CGC)4	12	260	271	AGAAGCACAGTGAGGTCGTG	59.967	ATCGAGATCCTTGCGCGAAT	59.967	161
TRINITY_Contig652	1	p3	(GCC)4	12	1630	1641	CTGCTTCAGCTTGAGGTCCA	59.964	CGATGGCAAGCATTTCGAGG	59.971	256
TRINITY_Contig653	1	p4	(TGGT)3	12	1649	1660	AGCAATGCCACCTCCAATGA	59.96	GACAAGCGCCTCTCTGAAGT	60.038	259
TRINITY_Contig655	1	p3	(GCT)4	12	441	452	CTCCCCCTCTCTCCACAAGA	59.958	CTTCTTGCTCGTGGAGTCGT	60.039	137
TRINITY_Contig655	2	p3	(CGT)4	12	639	650	CTCCCCCTCTCTCCACAAGA	59.958	CTTCTTGCTCGTGGAGTCGT	60.039	137
TRINITY_Contig655	3	p4	(CCAC)3	12	1641	1652	CTCCCCCTCTCTCCACAAGA	59.958	CTTCTTGCTCGTGGAGTCGT	60.039	137
TRINITY_Contig656	1	p5	(GCCGG)3	15	1324	1338	GAAATCGCGATGCTTTCCCC	59.971	CAAATCGCAGCGGTCAAACA	60.041	236
TRINITY_Contig657	1	p3	(GCC)7	21	574	594	CTAGGCGAGTAAGGCGTGAG	59.97	TCCTCCTTCTCCCCCATCTG	60.031	195
TRINITY_Contig657	2	p3	(GCC)4	12	760	771	CTAGGCGAGTAAGGCGTGAG	59.97	TCCTCCTTCTCCCCCATCTG	60.031	195
TRINITY_Contig657	3	p3	(GGC)6	18	1567	1584	CTAGGCGAGTAAGGCGTGAG	59.97	TCCTCCTTCTCCCCCATCTG	60.031	195
TRINITY_Contig658	1	p3	(CCG)6	18	1643	1660	TCGAGCGCAACTAAAGGGTT	59.966	AGACAGGCCATCCAGGGTAT	60.03	160
TRINITY_Contig659	1	p2	(AG)6	12	271	282	GCCTTGCTTCATGGTTGCAA	59.966	TCTCAGCAGCCCTAGGATGT	60.032	280
TRINITY_Contig659	2	p3	(CGA)4	12	761	772	GCCTTGCTTCATGGTTGCAA	59.966	TCTCAGCAGCCCTAGGATGT	60.032	280
TRINITY_Contig661	1	p4	(ACGG)3	12	960	971	CTTGCCACCCTTCTTAGCGA	60.036	GTAGTCCGTGATCGCCAGAG	59.97	280
TRINITY_Contig662	1	p3	(GCT)4	12	2156	2167	CATCCCCAGCAAGAACCTGT	59.961	GGTTGAGGGCGTTGTTGAAC	59.97	164
TRINITY_Contig663	1	p4	(AAAC)3	12	57	68	CAAACCGCCTGCAATGTCAA	59.968	GGTCTCGTGACAGCAGAACA	59.968	115
TRINITY_Contig664	1	p4	(CAGC)3	12	6	17	TGTATGCAACGACCCCCTTC	60.036	CAGGATCAGAGCTAGCTGGC	59.966	148
TRINITY_Contig667	1	p3	(CTG)5	15	209	223	ATGGGATGGGAAGCAAAGGG	60.032	CGTTTTGATGGATTCGGGGC	59.899	173
TRINITY_Contig667	2	p3	(AAG)4	12	526	537	ATGGGATGGGAAGCAAAGGG	60.032	CGTTTTGATGGATTCGGGGC	59.899	173
TRINITY_Contig667	3	p6	(CGCCCG)3	18	710	727	ATGGGATGGGAAGCAAAGGG	60.032	CGTTTTGATGGATTCGGGGC	59.899	173
TRINITY_Contig667	4	p3	(TGC)7	21	1566	1586	ATGGGATGGGAAGCAAAGGG	60.032	CGTTTTGATGGATTCGGGGC	59.899	173
TRINITY_Contig670	1	p3	(GCC)4	12	148	159	TTGTGGACATTGGAGCCTCC	59.962	GCACCTTGGGAAACTCGAGA	59.965	224
TRINITY_Contig675	1	p3	(CCG)5	15	234	248	CACCAGCCAATCAAAAGGGC	60.037	TGACCATTACAACACGCCGA	59.966	269
TRINITY_Contig675	2	p3	(CAC)4	12	426	437	CACCAGCCAATCAAAAGGGC	60.037	TGACCATTACAACACGCCGA	59.966	269
TRINITY_Contig677	1	p3	(CGC)4	12	940	951	AGGAGAGGTGTGGAGGAGTC	59.96	CCGAAATCCTAGCCCACACA	59.748	144
TRINITY_Contig678	1	c	(CGG)4cgaggcgcacccggaggacagcgacgacgcggtggaggcctccgaggaggacgagtc(GCG)4	81	700	780	CTCCAGAACTCGATCCACCG	59.899	CTCGACGTGCTCCACTTCTT	60.039	176
TRINITY_Contig680	1	p6	(CGGCTG)6	36	658	693	TTCCACATCCCGACCAATGG	60.035	AAGACCTCTCCTGCCAAAGC	59.963	239
TRINITY_Contig681	1	p4	(GGGA)3	12	1452	1463	GGCACCTGTCGTTCTACCAA	59.966	TAGGGTGTTCGTTCACTGGC	59.966	145
TRINITY_Contig683	1	p4	(CGCT)3	12	88	99	GGTGCAAAGCATTGGAACGT	59.968	GAGTGAGGTTTGGAGCGGAA	59.965	150
TRINITY_Contig683	2	p3	(GGC)4	12	397	408	GGTGCAAAGCATTGGAACGT	59.968	GAGTGAGGTTTGGAGCGGAA	59.965	150
TRINITY_Contig685	1	p3	(GGC)8	24	1320	1343	TGCCCCCAATTTCAAAAGCG	59.965	CCCTCTTCGTCCTCACCATG	59.823	203
TRINITY_Contig696	1	p3	(ATC)5	15	199	213	CAAAGTCGCCTAATGCTCGC	59.973	GATCTCGATCTCGCCCGTTT	59.969	248
TRINITY_Contig697	1	c	(AAAAG)3gagagagaagtagagacaaattcagaagacgagatccccaacccgaccaaaaaggcagcgaaaagaccgtacgccg(GA)6	103	335	437	ACCAAACCAAAACGATGGCG	59.969	TTTCGCTGCCTTTTTGGTCG	59.97	163
TRINITY_Contig697	2	p4	(CCGG)4	16	644	659	ACCAAACCAAAACGATGGCG	59.969	TTTCGCTGCCTTTTTGGTCG	59.97	163
TRINITY_Contig697	3	c	(CTG)4cccggccgccgccaccggggcgggctgcaacgatacggctgcggcggcg(GCT)4	73	942	1014	ACCAAACCAAAACGATGGCG	59.969	TTTCGCTGCCTTTTTGGTCG	59.97	163
TRINITY_Contig697	4	p3	(CGC)5	15	1310	1324	ACCAAACCAAAACGATGGCG	59.969	TTTCGCTGCCTTTTTGGTCG	59.97	163
TRINITY_Contig700	1	p3	(CCG)4	12	145	156	CGAACAAGGTGTCGTGCAAG	60.042	GGAGACGTGCTTGGTGATGA	60.037	167
TRINITY_Contig700	2	c	(CCG)4ccacccacaccgccgcca(CCG)5	45	282	326	CGAACAAGGTGTCGTGCAAG	60.042	GGAGACGTGCTTGGTGATGA	60.037	167
TRINITY_Contig701	1	c	(CGG)4(CCT)4	24	496	519	ATTCGCCCGAAACATCCCTT	60.034	GGAGAAGGCGTACGAGAAGG	59.899	217
TRINITY_Contig701	2	p3	(CGA)4	12	1319	1330	ATTCGCCCGAAACATCCCTT	60.034	GGAGAAGGCGTACGAGAAGG	59.899	217
TRINITY_Contig701	3	c	(AT)7acttatatagatggtaagtagctggtggtggagcagttcttgccggccctaatatatagga g(TGGAGC)3	94	1504	1597	ATTCGCCCGAAACATCCCTT	60.034	GGAGAAGGCGTACGAGAAGG	59.899	217
TRINITY_Contig702	1	p3	(GCC)5	15	1338	1352	ATCACCGTCAGAGCACCTTG	60.037	CTCTTGGGGGATGGTTCCAC	60.034	117
TRINITY_Contig705	1	p2	(CT)7	14	36	49	CGCAAGCTTTTCCTGCTGTT	59.969	GTGTGCGCACAAGTGGAAAT	59.969	180
TRINITY_Contig706	1	p3	(GCG)4	12	1859	1870	GCAGTAGCCCAGTAACTGCA	60.036	GCCGTCAAGAGGATATCGCA	59.968	114
TRINITY_Contig708	1	p4	(TGCG)3	12	1359	1370	ATGTGACCGTGTCCGAACTC	60.039	GCCTCGAGCTATTCAGGACC	59.967	223
TRINITY_Contig710	1	c	(CCA)4cgacgcgctgtgggcgaagctgcacgagctggaggtgcagctcgcggcgtacaagctcctgcgcgccgcgc(GCG)4	95	237	331	ATCTCCGGATGAGGAAGCCT	60.105	TTCATTCTCCCAAGCGCAGT	59.963	119
TRINITY_Contig710	2	c	(CGC)5tgtccatcccgagcaccccgaggagggacgccgcggcgctgccgaggagcaggaccgtgac(CGG)4	88	629	716	ATCTCCGGATGAGGAAGCCT	60.105	TTCATTCTCCCAAGCGCAGT	59.963	119

TRINITY_Contig711	1	p2	(CA)6	12	597	608	ATGTCAACCCACAGACGGAC	59.965	AGACAGAGTGGCCTGAGCTA	59.96	154
TRINITY_Contig712	1	p5	(AGAGT)3	15	564	578	AAGAGGTAGGGGGCATAGCA	60.03	CAGAGGAGGAGGAGGAAGCT	60.032	106
TRINITY_Contig712	2	c	(TCGTG)4(CGCC)3	32	1305	1336	AAGAGGTAGGGGGCATAGCA	60.03	CAGAGGAGGAGGAGGAAGCT	60.032	106
TRINITY_Contig712	3	p3	(TAG)4	12	1440	1451	AAGAGGTAGGGGGCATAGCA	60.03	CAGAGGAGGAGGAGGAAGCT	60.032	106
TRINITY_Contig712	4	c	(GGGC)3g(CACGA)4	33	1794	1826	AAGAGGTAGGGGGCATAGCA	60.03	CAGAGGAGGAGGAGGAAGCT	60.032	106
TRINITY_Contig712	5	c	(GCATA)3tatggcgccactgccgagccact tcggtgcatccatcaactgctcaacacttcaatt cgagct(TCC)4	90	2330	2419	AAGAGGTAGGGGGCATAGCA	60.03	CAGAGGAGGAGGAGGAAGCT	60.032	106
TRINITY_Contig715	1	p3	(CTG)4	12	162	173	GATACTAGGTGTGCCTCGCC	59.968	TGAGTGCCGACAGGAAGAAC	59.966	237
TRINITY_Contig715	2	p6	(TTGGAG)3	18	387	404	GATACTAGGTGTGCCTCGCC	59.968	TGAGTGCCGACAGGAAGAAC	59.966	237
TRINITY_Contig715	3	p3	(CAG)4	12	3113	3124	GATACTAGGTGTGCCTCGCC	59.968	TGAGTGCCGACAGGAAGAAC	59.966	237
TRINITY_Contig716	1	c	(GCG)4gtgagaggagatcgagcacggact agtatcgatc(GCCG)3	58	209	266	TCCACTGGAAGCTTCAGCAG	59.964	GAGCTGTCCATGAACCACGA	60.037	208
TRINITY_Contig716	2	p3	(CAG)4	12	395	406	TCCACTGGAAGCTTCAGCAG	59.964	GAGCTGTCCATGAACCACGA	60.037	208
TRINITY_Contig718	1	p3	(CGG)7	21	2502	2522	ACAGCAAGACTCTCACCACG	59.967	TTGCGTCCATTTTACCCCGA	59.964	144
TRINITY_Contig721	1	p3	(CAC)4	12	1400	1411	CACAACCTGCAGAAGCCAAC	59.969	ACTGGTAGTGCTTGTGGTCG	59.966	129
TRINITY_Contig722	1	p2	(AG)10	20	1527	1546	CAAGCTGCTTCTCTCAGGCT	60.036	TGTTCGTCATCGTTCAGCCA	59.967	112
TRINITY_Contig723	1	p3	(CCG)4	12	185	196	TGAGGTGGAGTTCAATGGGC	59.962	TTGCTCTGGGACACCGTTAC	59.966	234
TRINITY_Contig724	1	c	(CGC)4caccgtgcccgcagcagctgccgca gcctacgccctcc(CCT)5	65	244	308	TTGTGCCAGCGAATTTTGCA	59.896	CAAGCTCCAGGGAGAACAGG	60.036	144
TRINITY_Contig725	1	p3	(CGC)4	12	765	776	GACTCCTTGTCCAGCGTCTC	60.109	TGGTGTACGTGCAGACTCAC	59.968	232
TRINITY_Contig726	1	p3	(CTT)4	12	592	603	TTCTTGGAGGTCACTGCGAC	59.966	CCTGTCCCTTTTCCCTCACC	59.962	121
TRINITY_Contig726	2	p3	(AGC)5	15	1131	1145	TTCTTGGAGGTCACTGCGAC	59.966	CCTGTCCCTTTTCCCTCACC	59.962	121
TRINITY_Contig727	1	c	(CGGAT)5ccgcgaccacccccg(CGC)7	61	70	130	CCGCAACATCGGTTTTGTGT	59.97	CCAAGCTTGGTTGCAAGGAC	59.967	228
TRINITY_Contig728	1	p2	(CA)9	18	172	189	GGTCAGCTGCCCAGTGTTAT	60.035	TGGCTAGGGAGACAGCATCT	60.032	212
TRINITY_Contig729	1	p3	(GCA)7	21	976	996	CAACTTCGCTGCGGAAAACA	59.971	CGATGAGCTTCTCCGTCTCC	59.97	221
TRINITY_Contig729	2	p3	(CGC)4	12	1238	1249	CAACTTCGCTGCGGAAAACA	59.971	CGATGAGCTTCTCCGTCTCC	59.97	221
TRINITY_Contig729	3	p5	(GACTG)3	15	1352	1366	CAACTTCGCTGCGGAAAACA	59.971	CGATGAGCTTCTCCGTCTCC	59.97	221
TRINITY_Contig732	1	p3	(GCC)4	12	690	701	CATATGCAGAGGCCATCGGT	59.964	TTCTACACGCCGGGGTACTA	60.035	195
TRINITY_Contig732	2	c	(CCGTCG)3ccaccgccgccgctcgggtgga ggcgccggacctcgggcgtggggtgcggcgg catggcgctggcgagcgtggcgccgtcg(GC C)4	111	1141	1251	CATATGCAGAGGCCATCGGT	59.964	TTCTACACGCCGGGGTACTA	60.035	195
TRINITY_Contig735	1	p4	(AGGA)3	12	1576	1587	CCGTGTTGTTTGCAGCATCA	59.969	AGGCATGGTCCCACTTGATG	60.034	137
TRINITY_Contig736	1	p3	(GGC)4	12	548	559	AGAAGGCGATGGTGGTTCTG	60.036	GCAAAGTCTCAGCCTTGTGC	60.04	267
TRINITY_Contig738	1	p3	(CGG)5	15	1480	1494	CACGCAACAGATGATGCTCG	59.974	ATCTCAAGTGCCAGGCTTCC	60.035	277
TRINITY_Contig739	1	p4	(TCGC)3	12	189	200	GTCTTCGCCTCGTCACTCTC	60.179	GGGGAGGTAGGTCTAACGGT	60.033	200
TRINITY_Contig739	2	p3	(CGG)5	15	1225	1239	GTCTTCGCCTCGTCACTCTC	60.179	GGGGAGGTAGGTCTAACGGT	60.033	200
TRINITY_Contig741	1	p3	(GCC)8	24	1383	1406	AAGGGGAATGCAGCTGGATC	60.106	GCATTTGTGCAGTGGCTTCA	59.968	128
TRINITY_Contig742	1	p3	(GCC)5	15	134	148	CAACAATGCGCTGATCCGAG	59.973	TCAGGCAAGGCATGATTGGT	59.96	244
TRINITY_Contig742	2	c	(CGG)4c(GAG)4gccgaggtgttcagcacc cctcctcttacacagggccagcagagccaaca gagccagcagggtcagggggta(GAG)4	109	417	525	CAACAATGCGCTGATCCGAG	59.973	TCAGGCAAGGCATGATTGGT	59.96	244
TRINITY_Contig745	1	c	(AGG)5gggaggtctccagca(CCG)4	42	994	1035	GTGGACGGTGTACTGCTACC	60.109	GCTGCTGACGTGGTAGTTCT	60.038	209
TRINITY_Contig746	1	p3	(GCG)4	12	326	337	GGGAAGACCTACCGGCTCTA	60.106	GAGCTGGTACGTCACGAACA	60.04	198
TRINITY_Contig747	1	p3	(GAG)4	12	1450	1461	CGAGGAGATGAGGTCCATGC	59.967	CCGAGGTGTCCATCGAGATG	59.969	224
TRINITY_Contig749	1	p3	(AGG)4	12	624	635	GCGAGGGAAGAGAAAAGGCT	60.036	GGAGGACGAAGATCAGCGAG	59.97	147
TRINITY_Contig749	2	p4	(GAAC)3	12	799	810	GCGAGGGAAGAGAAAAGGCT	60.036	GGAGGACGAAGATCAGCGAG	59.97	147
TRINITY_Contig751	1	p3	(TGC)4	12	576	587	TCCTGCTGTACTGCCAGTTG	59.965	AACGTCCCCTTGTTGCTAGG	59.964	172
TRINITY_Contig751	2	p3	(GGA)5	15	1217	1231	TCCTGCTGTACTGCCAGTTG	59.965	AACGTCCCCTTGTTGCTAGG	59.964	172
TRINITY_Contig751	3	c	(GAGGGA)3gaggaagcaga(CGGT)3	41	1437	1477	TCCTGCTGTACTGCCAGTTG	59.965	AACGTCCCCTTGTTGCTAGG	59.964	172
TRINITY_Contig755	1	p3	(GCC)4	12	1224	1235	GCGGTGGTCATTTCCTGAAC	59.478	CGCTGTTCTACGGGGAGTAC	59.9	198
TRINITY_Contig756	1	p3	(GGC)5	15	280	294	TCACCATCATGGAGCTTCCG	59.821	TCCCTTGTGGCTGAACGTTT	60.107	109
TRINITY_Contig757	1	p3	(GAG)4	12	631	642	GGGACAACCAACGCTGTTTC	59.97	GGGGCCCTGATTCTTTGTCA	59.96	165
TRINITY_Contig758	1	p3	(GCC)4	12	293	304	CGCGTCGTTCAACCAAACTT	59.973	TGTTCTCCGCGTTTCCATCA	59.966	249
TRINITY_Contig758	2	c	(AGCT)3actactc(GAA)4	31	1245	1275	CGCGTCGTTCAACCAAACTT	59.973	TGTTCTCCGCGTTTCCATCA	59.966	249
TRINITY_Contig758	3	p4	(GGTG)3	12	1482	1493	CGCGTCGTTCAACCAAACTT	59.973	TGTTCTCCGCGTTTCCATCA	59.966	249
TRINITY_Contig759	1	p3	(TCG)4	12	1332	1343	CGGGGAGATCTTCTTCGTCG	59.97	TTGGCGGCGATGAGTATCTC	59.968	121
TRINITY_Contig760	1	p3	(CGA)4	12	848	859	TCTTGTTCTTGTCCGAGGCC	59.965	CTTCTTCCCGTGCAACTTGC	60.04	228
TRINITY_Contig760	2	p3	(GCG)4	12	1251	1262	TCTTGTTCTTGTCCGAGGCC	59.965	CTTCTTCCCGTGCAACTTGC	60.04	228
TRINITY_Contig761	1	p3	(AAG)5	15	829	843	TTGCTGTGGAAGTCCGGATC	60.036	CCAGCACCAAGAACATGTGC	60.039	276
TRINITY_Contig764	1	p3	(CCG)6	18	1066	1083	GTCGACTTCAGAAGGTGGCA	59.966	GGATGAGCTTCGCGATGAGA	59.969	247
TRINITY_Contig765	1	p3	(ACT)4	12	1326	1337	GCATGTCGCTGCTGTACATG	59.973	CCTCTTCCCACTCCACGTTC	60.037	243
TRINITY_Contig766	1	p4	(TAGC)3	12	261	272	CTAGCGAGGCGATCTTGGAG	60.04	CCATTTGACCGAGAGCGAGA	59.827	112
TRINITY_Contig766	2	p3	(TGG)5	15	1452	1466	CTAGCGAGGCGATCTTGGAG	60.04	CCATTTGACCGAGAGCGAGA	59.827	112
TRINITY_Contig767	1	p4	(CCAG)4	16	225	240	CACCAAACCTCTGACCCCTC	59.963	CTTCCTGCCTCCCTCTCTCT	60.032	201
TRINITY_Contig767	2	p6	(GCCGGC)3	18	622	639	CACCAAACCTCTGACCCCTC	59.963	CTTCCTGCCTCCCTCTCTCT	60.032	201
TRINITY_Contig767	3	p2	(TC)11	22	4615	4636	CACCAAACCTCTGACCCCTC	59.963	CTTCCTGCCTCCCTCTCTCT	60.032	201
TRINITY_Contig768	1	c	(GCG)4tacctggtggtggacc(CGG)4	40	274	313	CTCAATCTCCCCGCCAGATC	59.965	GTCATGTCGTTCAATGGCGG	59.903	267
TRINITY_Contig768	2	p4	(CATA)3	12	486	497	CTCAATCTCCCCGCCAGATC	59.965	GTCATGTCGTTCAATGGCGG	59.903	267
TRINITY_Contig770	1	p3	(TCC)4	12	338	349	CAGCCAGGCATCTTCTTCCA	60.035	GCTGGACTGGAAGTGAGGAC	60.037	208
TRINITY_Contig770	2	p3	(CGG)5	15	1405	1419	CAGCCAGGCATCTTCTTCCA	60.035	GCTGGACTGGAAGTGAGGAC	60.037	208
TRINITY_Contig773	1	p2	(AG)6	12	1411	1422	TGCGATCGGTGAACTGTTGA	59.967	CTCTTTCCGGCCTTCCATGT	60.035	160
TRINITY_Contig775	1	p3	(CGC)4	12	886	897	ACCCGTATGTGTTGAGCTGG	60.036	CAACAACAGCTTCGTCGTCG	60.112	113
TRINITY_Contig775	2	p3	(GCC)4	12	1226	1237	ACCCGTATGTGTTGAGCTGG	60.036	CAACAACAGCTTCGTCGTCG	60.112	113
TRINITY_Contig775	3	p3	(CGT)4	12	1456	1467	ACCCGTATGTGTTGAGCTGG	60.036	CAACAACAGCTTCGTCGTCG	60.112	113
TRINITY_Contig776	1	p3	(ACT)4	12	90	101	TGGGGTCGAGGAAGTAGGAG	60.033	CTCTTCCATCCTCTCCCCCA	60.031	250
TRINITY_Contig778	1	p6	(GCAGCG)3	18	49	66	ATTGCGAAACGGATGATGCG	59.972	CCAGGGTCTCTTTGCAGGAG	60.036	168
TRINITY_Contig778	2	c	(GCG)5atcgatggacccgta(CGG)4	42	213	254	ATTGCGAAACGGATGATGCG	59.972	CCAGGGTCTCTTTGCAGGAG	60.036	168
TRINITY_Contig780	1	p4	(CTTG)3	12	1315	1326	GTGTCCAAGAAGTCCGACGT	59.968	GTCGTCGTACCCCTCTCCTA	59.821	177
TRINITY_Contig781	1	p4	(TAGA)3	12	91	102	GAGGGAGGAGACGAGGGATT	60.106	GCATCGATCGCCTGCAAATT	59.97	105
TRINITY_Contig781	2	p3	(GCA)4	12	234	245	GAGGGAGGAGACGAGGGATT	60.106	GCATCGATCGCCTGCAAATT	59.97	105
TRINITY_Contig781	3	p3	(CAG)4	12	825	836	GAGGGAGGAGACGAGGGATT	60.106	GCATCGATCGCCTGCAAATT	59.97	105
TRINITY_Contig781	4	p3	(ACG)4	12	1319	1330	GAGGGAGGAGACGAGGGATT	60.106	GCATCGATCGCCTGCAAATT	59.97	105
TRINITY_Contig781	5	p4	(TAGC)4	16	1499	1514	GAGGGAGGAGACGAGGGATT	60.106	GCATCGATCGCCTGCAAATT	59.97	105
TRINITY_Contig783	1	p3	(TGG)4	12	1486	1497	CGGTCTGCATCAAGGGAAGT	60.036	ACCGTCGACCTGCAAGAAAT	59.965	242
TRINITY_Contig784	1	p3	(CCG)4	12	520	531	GAACCCTCGCGGCCATATAA	59.966	AGGCGCAGGGAAATTTCTCA	59.962	150
TRINITY_Contig784	2	p5	(GAGGA)4	20	1125	1144	GAACCCTCGCGGCCATATAA	59.966	AGGCGCAGGGAAATTTCTCA	59.962	150
TRINITY_Contig784	3	p4	(CCCT)3	12	1404	1415	GAACCCTCGCGGCCATATAA	59.966	AGGCGCAGGGAAATTTCTCA	59.962	150
TRINITY_Contig786	1	p3	(GGC)4	12	355	366	TCTCCTCATCGTCAGCCTCA	60.034	AGGGTAGGAGGTGCTGAGAG	60.033	264
TRINITY_Contig788	1	p2	(CT)6	12	6	17	ATGGTGGAAAGGAGGCGAAG	60.035	CAGAGCCTAACAGCAGCAGT	60.037	176
TRINITY_Contig788	2	p3	(GAC)4	12	1228	1239	ATGGTGGAAAGGAGGCGAAG	60.035	CAGAGCCTAACAGCAGCAGT	60.037	176
TRINITY_Contig789	1	p3	(GCC)6	18	1031	1048	AACAGATGCACCCGTACCAG	60.036	CCACATACTGGGGTTGCTGT	59.962	246
TRINITY_Contig794	1	p3	(GAG)5	15	94	108	CGGTGAGCCTGGAGAAGATG	60.179	TCGAACAGCGTACCCATGTC	60.109	178
TRINITY_Contig794	2	p3	(GCC)4	12	973	984	CGGTGAGCCTGGAGAAGATG	60.179	TCGAACAGCGTACCCATGTC	60.109	178
TRINITY_Contig794	3	p3	(CGG)4	12	1116	1127	CGGTGAGCCTGGAGAAGATG	60.179	TCGAACAGCGTACCCATGTC	60.109	178
TRINITY_Contig795	1	p3	(GGC)4	12	1372	1383	CTTGCTTCTTTGGTGCCCAC	59.967	ATTGCTGAGGCCATCACTCC	60.107	163
TRINITY_Contig797	1	p3	(TCT)9	27	1345	1371	TCTTTACACGTCGGTCGCAA	59.969	GCTCAAGAACCTCGTCGACA	60.039	117
TRINITY_Contig798	1	p2	(TC)8	16	20	35	TGGGGTTAGCGATACCCTCA	60.032	TTGACGACAAGGTCACGGAG	59.968	108
TRINITY_Contig800	1	p4	(CACG)3	12	62	73	CGTCCCTGAGGCTGAAACTT	59.965	AGCCTAAAGATCTCTCGCGC	59.969	250
TRINITY_Contig801	1	p3	(CTC)5	15	130	144	GCCAACGACGACGATGATTG	59.975	TACCACCTGACGTTCAGCAC	59.967	166
TRINITY_Contig801	2	p3	(CCG)4	12	366	377	GCCAACGACGACGATGATTG	59.975	TACCACCTGACGTTCAGCAC	59.967	166
TRINITY_Contig802	1	p3	(GGC)9	27	1348	1374	GGCGTTTCCTGTTGGGTAGA	59.964	GGATGTAAGGGACGCTCCTG	59.895	186
TRINITY_Contig803	1	p2	(CT)8	16	356	371	ACAGTATGAGGGCCCTGCTA	60.031	ATACAGTGGCTCGGCAAACA	59.964	174
TRINITY_Contig804	1	p3	(CCA)4	12	296	307	TCCCCACCCATCATCATCCT	60.029	GGAGGAGGACCAGATGGAGT	60.032	189
TRINITY_Contig804	2	p6	(CGGCAA)3	18	618	635	TCCCCACCCATCATCATCCT	60.029	GGAGGAGGACCAGATGGAGT	60.032	189
TRINITY_Contig804	3	p4	(CTTG)3	12	779	790	TCCCCACCCATCATCATCCT	60.029	GGAGGAGGACCAGATGGAGT	60.032	189
TRINITY_Contig804	4	p4	(GTAT)3	12	2033	2044	TCCCCACCCATCATCATCCT	60.029	GGAGGAGGACCAGATGGAGT	60.032	189
TRINITY_Contig805	1	p3	(CAG)4	12	1468	1479	ATCCTTGGAGGCAGGGAGAT	60.029	GGACAAAATCGCTCTCCCCA	60.035	114
TRINITY_Contig807	1	p3	(CCG)4	12	193	204	CGACCAAGTGGGAGCTCTTT	59.965	AACACCAGGCTCATCAGTGG	59.963	173
TRINITY_Contig811	1	p6	(TCCTGT)3	18	343	360	AGATAAATGTGGCGGCCCAA	60.034	ACAGGAACAGGAGCAGAAGC	59.964	211
TRINITY_Contig811	2	p3	(GCC)4	12	2678	2689	AGATAAATGTGGCGGCCCAA	60.034	ACAGGAACAGGAGCAGAAGC	59.964	211
TRINITY_Contig813	1	p2	(GT)8	16	184	199	TGGTGTGTGTGTGTGTGTCA	59.965	GACCACTAGCATGGACCTCG	59.897	263
TRINITY_Contig814	1	p3	(AGC)4	12	239	250	GGTGTTGAAGGCACTGCTTG	59.969	TCAAGTGCATGAGAGGCCTG	60.036	217

TRINITY_Contig814	2	p4	(AGAC)3	12	1090	1101	GGTGTTGAAGGCACTGCTTG	59.969	TCAAGTGCATGAGAGGCCTG	60.036	217
TRINITY_Contig816	1	p4	(CGAT)3	12	31	42	CAGGCCTCAACTGTCTCCAG	60.037	CTTCCATTGGGGGCCTCATT	60.032	223
TRINITY_Contig817	1	p6	(CCCCTG)3	18	159	176	CTGCTCCTGTAGGTGTCACG	60.109	ACGAGCAGAGCTACAACCAC	60.038	255
TRINITY_Contig817	2	c	(CGCT)3cgtgtgctctcgctcgcttgcgcgcgccgggaaggcagtgggtgcctggtcggttctcgcccgcgagggtggtgg(TGC)4	99	654	752	CTGCTCCTGTAGGTGTCACG	60.109	ACGAGCAGAGCTACAACCAC	60.038	255
TRINITY_Contig820	1	c	(CTGTCG)3ccgtcgcgagagtggggaactgctcacacaggcccaccaccgggagcttctctatgagaa(GCG)4	90	515	604	ACCACCGGGAGCTTCTCTAT	60.032	GGTGCTGCTGACTCCTTCAT	60.036	242
TRINITY_Contig821	1	p3	(GTG)4	12	2244	2255	CTTTGATTCAGCGAGCGAGC	59.974	GACGAGCGAGCTACTTCCTC	59.972	280
TRINITY_Contig823	1	p4	(GTAT)3	12	1341	1352	TGTTGTGGCTTGCATGATGC	60.038	GGTCGGACTTCCTGAACTCG	60.109	131
TRINITY_Contig825	1	p3	(GAG)4	12	1310	1321	ATTGGGAGCACCTGCATGAA	59.96	ACCCCCTTCTCTCCATCCTC	60.031	271
TRINITY_Contig826	1	c	(CCTTC)5cgatatctcctc(CCG)4	49	122	170	AGCTTGTGGGGGAAGTTAGC	59.962	GTCCTTCGCCCTTCACTGAA	59.965	114
TRINITY_Contig827	1	p3	(TGG)5	15	1296	1310	TTTCTGTTGGCTCGATGGCT	59.963	GCAATCCTCCCTAATCCCGG	59.963	149
TRINITY_Contig828	1	p3	(CGC)4	12	194	205	TCGGATTGGAGCAGTGTGAC	60.037	AGCTAGCTCCCTCACCATCA	60.032	152
TRINITY_Contig829	1	p3	(CCG)4	12	636	647	CTGCCGATCTCGAGTTCCTC	59.97	GCGTCGATGGTGGAGATGAT	59.967	272
TRINITY_Contig831	1	p3	(CCG)4	12	523	534	GTCTTCCCCCGAAACAGAGG	60.036	CCAACGTGGCTCCTCTGATT	60.036	100
TRINITY_Contig833	1	c*	(TGGT)2(TG<GT>)(GT)6	24	1414	1437	ACGTATCACACCGACACACC	60.039	AGCAGTTCTGCCAGGACTTC	59.964	115
TRINITY_Contig835	1	p3	(GCG)4	12	198	209	TGCTGCCTTCTCAGAACCTG	59.964	ACCGTTTTCCTTGCAATGGC	59.967	123
TRINITY_Contig839	1	p3	(GGC)5	15	136	150	GAGAGCTTACGATGGGTCCG	59.969	GACATCTTTGCCAGAGCCCT	60.035	156
TRINITY_Contig840	1	p3	(CGC)4	12	288	299	AACATGCTAGGCGGAGATCG	59.967	CGCGCTGCTAGTAGACTACC	60.041	156
TRINITY_Contig844	1	p3	(CGG)4	12	1341	1352	CTTGGAGGACCGGAAGTGTC	60.037	TCTACAACACGGAGCCCAAC	59.966	277
TRINITY_Contig845	1	p4	(CCAG)3	12	588	599	CAGGGTGCTTGGCTTTGTTC	59.967	TTTGTGCTGGCCTGAAATGC	59.966	196
TRINITY_Contig845	2	p3	(GGC)4	12	1392	1403	CAGGGTGCTTGGCTTTGTTC	59.967	TTTGTGCTGGCCTGAAATGC	59.966	196
TRINITY_Contig846	1	p2	(GC)6	12	1243	1254	AACAGCTTCACCATCCTGGG	59.961	ACCCCGATCTCATCTCCCAA	60.031	202
TRINITY_Contig847	1	p3	(GAC)5	15	1301	1315	AGACCACTCGCTCCTTGTTG	59.966	GACCTCAAGAACGAGCCCAA	59.965	244
TRINITY_Contig848	1	p3	(TTG)4	12	582	593	CGATATACTGCGCCCAAGGT	59.966	CAGTAAGCGCATCCGAAAGC	59.973	258
TRINITY_Contig851	1	p3	(AAG)4	12	1004	1015	TGTCTCCGGTGTTAACGTGG	59.968	TCGCCATCCCTGAGAGAGAA	60.033	237
TRINITY_Contig853	1	c	(GCA)5(CCA)4	27	422	448	ATTGTTCTGCTCCTCCCCG	60.035	ATTCTTTCCCTCCCGGCTTG	60.034	200
TRINITY_Contig855	1	p3	(AAT)4	12	222	233	TTACATTCAGGCCGGCAAT	60.034	TGGCTCATCATGACCGCTTT	60.035	229
TRINITY_Contig857	1	p3	(TCG)4	12	837	848	TCGCCGTAGAAGAGGAGGAA	60.035	GTCTTCGCCTTCTTCCTGCT	60.037	233
TRINITY_Contig858	1	p4	(TGTT)4	16	1367	1382	GGCCCGTCTGGTGACATAAA	60.036	GATGTCGCTGACAGGAGAGG	59.898	221
TRINITY_Contig860	1	p3	(GCT)4	12	1291	1302	CCAAAACCCCTCAAAAGCGG	59.967	CTGCGACGATCTCCTCTTCC	59.97	167
TRINITY_Contig863	1	p3	(GGA)5	15	310	324	CATGTACCGATGCTACGCCT	59.968	TCAACGGTCCTGGTTAACGG	59.966	252
TRINITY_Contig863	2	p3	(CGC)4	12	730	741	CATGTACCGATGCTACGCCT	59.968	TCAACGGTCCTGGTTAACGG	59.966	252
TRINITY_Contig863	3	c	(CTC)4ttctttctctttttttagttctctgaagaaaagcagctactttttca(TCT)5	73	1023	1095	CATGTACCGATGCTACGCCT	59.968	TCAACGGTCCTGGTTAACGG	59.966	252
TRINITY_Contig863	4	p3	(CAG)4	12	1233	1244	CATGTACCGATGCTACGCCT	59.968	TCAACGGTCCTGGTTAACGG	59.966	252
TRINITY_Contig864	1	p3	(GCG)4	12	1158	1169	ATCCTCACGCTTACCCTCCT	60.032	GTGGACACTAGGCTGCAGTT	59.965	109
TRINITY_Contig864	2	p4	(GGCG)3	12	1310	1321	ATCCTCACGCTTACCCTCCT	60.032	GTGGACACTAGGCTGCAGTT	59.965	109
TRINITY_Contig866	1	p3	(CGG)5	15	1009	1023	CTATCCCGGTCCCGATCTCT	59.962	AACCAGCAAACCCTACCTCG	59.964	177
TRINITY_Contig866	2	p5	(AGAGG)3	15	1348	1362	CTATCCCGGTCCCGATCTCT	59.962	AACCAGCAAACCCTACCTCG	59.964	177
TRINITY_Contig867	1	p4	(CATG)3	12	1121	1132	ATTGCTCCTGGATATGCGGG	59.963	CCGACCTCGAGTGCAGATAC	59.97	153
TRINITY_Contig867	2	p6	(GAGCGG)3	18	2188	2205	ATTGCTCCTGGATATGCGGG	59.963	CCGACCTCGAGTGCAGATAC	59.97	153
TRINITY_Contig867	3	p3	(GGC)4	12	2331	2342	ATTGCTCCTGGATATGCGGG	59.963	CCGACCTCGAGTGCAGATAC	59.97	153
TRINITY_Contig868	1	p3	(CGC)4	12	1075	1086	CGCCCGGAGTCTTTCTTCTT	60.037	CGAGACGGACCATACACAGG	59.899	171
TRINITY_Contig869	1	p4	(TGGC)3	12	1121	1132	GACGAGGCAAGGAGACACAA	59.966	CATGTGCTGACTGGCTGAGA	60.037	109
TRINITY_Contig870	1	p3	(CGG)5	15	575	589	TACCGTGGCGAGTTTCTTCC	60.038	GTGTTTGCTAGGTCGGTCGA	60.039	200
TRINITY_Contig871	1	p3	(GCC)8	24	294	317	CCATCGAGCATCATCCTCCC	60.037	CCGATCGTGCTGTCCGATTA	59.97	216
TRINITY_Contig871	2	p4	(CTGC)3	12	940	951	CCATCGAGCATCATCCTCCC	60.037	CCGATCGTGCTGTCCGATTA	59.97	216
TRINITY_Contig872	1	p2	(CT)8	16	6	21	ACGAATCCAGTGCCCAAGAG	60.036	CTCGCGGCGACAGTTATTTG	59.975	220
TRINITY_Contig872	2	p3	(TCT)4	12	149	160	ACGAATCCAGTGCCCAAGAG	60.036	CTCGCGGCGACAGTTATTTG	59.975	220
TRINITY_Contig872	3	p3	(ATG)4	12	518	529	ACGAATCCAGTGCCCAAGAG	60.036	CTCGCGGCGACAGTTATTTG	59.975	220
TRINITY_Contig872	4	p3	(TCT)4	12	3003	3014	ACGAATCCAGTGCCCAAGAG	60.036	CTCGCGGCGACAGTTATTTG	59.975	220
TRINITY_Contig875	1	p4	(ATGT)3	12	295	306	CCTGCGAACTGTGATGCCTA	60.108	GGAGACCCCAGCAATTCACA	59.962	184
TRINITY_Contig875	2	p3	(CGC)5	15	626	640	CCTGCGAACTGTGATGCCTA	60.108	GGAGACCCCAGCAATTCACA	59.962	184
TRINITY_Contig875	3	p3	(CGC)6	18	1105	1122	CCTGCGAACTGTGATGCCTA	60.108	GGAGACCCCAGCAATTCACA	59.962	184
TRINITY_Contig879	1	p3	(CGG)4	12	82	93	GCTAACCTGTCTTGCTGGGA	59.675	CGCACACGAGCCAAACTATG	59.905	251
TRINITY_Contig880	1	p3	(GCG)4	12	1394	1405	TGGTACTGCTGTTCCACGTC	59.967	AGGAGAAGAAGCACCTGCAC	59.964	100
TRINITY_Contig881	1	c	(ACC)4gctggtgcgcggagtccccccgc(GCGG)4	50	144	193	TGCCATCCGGGTTTCTCTTC	60.035	CTTCTCCCCTCTTGCTGCTC	60.108	241
TRINITY_Contig881	2	p3	(GCG)4	12	376	387	TGCCATCCGGGTTTCTCTTC	60.035	CTTCTCCCCTCTTGCTGCTC	60.108	241
TRINITY_Contig882	1	p3	(GCC)4	12	77	88	GTAGCGGTGGGTAGGATTCG	59.968	GACTCAAGCCCGTCTCACAA	59.966	275
TRINITY_Contig884	1	p3	(GAG)4	12	434	445	ACATCCATGTCAGGGCAAGG	60.034	GGGCTCAGGGAAGCAATTCT	60.034	226
TRINITY_Contig885	1	p2	(CG)6	12	87	98	ACAACGTGTCCCAAGTGTGT	60.036	CATCTTCAAATCCGCCGCAG	59.971	212
TRINITY_Contig885	2	p3	(CTG)4	12	1082	1093	ACAACGTGTCCCAAGTGTGT	60.036	CATCTTCAAATCCGCCGCAG	59.971	212
TRINITY_Contig886	1	p4	(CGGC)3	12	1222	1233	GGATCAACATGTGCGCCATC	59.97	TCCGTTTGTGATGTCCTGGG	59.964	110
TRINITY_Contig887	1	p6	(GCAAGG)3	18	968	985	TTGATCACGATGTGCTGGCT	60.036	GACTGCTACATCGTCTCCGG	59.97	101
TRINITY_Contig887	2	p3	(TTG)4	12	1092	1103	TTGATCACGATGTGCTGGCT	60.036	GACTGCTACATCGTCTCCGG	59.97	101
TRINITY_Contig887	3	c	(GGC)4cggacggcggcagcaggattaggctgtggaggtggattgggaggtggaggcagcggccatcatggcgtgctggcgtcgggggcgggaccggg(ATGGA)3	119	2955	3073	TTGATCACGATGTGCTGGCT	60.036	GACTGCTACATCGTCTCCGG	59.97	101
TRINITY_Contig888	1	p4	(CTAG)3	12	222	233	GAGGCCCATTCATCCAACCA	60.033	GTCGTAGAACTGCGGGAACA	60.039	257
TRINITY_Contig889	1	p4	(TAGC)3	12	1210	1221	TGTACTTTAGAACGGCCCGG	59.753	GCGCTTGTGATGATCTGCAG	59.972	165
TRINITY_Contig890	1	p4	(ACGC)3	12	1146	1157	GAGTACTGCGAGGCGATGAA	59.899	GGGTAGTAGTTGCACCGCAT	60.108	134
TRINITY_Contig893	1	p3	(CCT)5	15	1090	1104	CGTCCAGCATCCTTGGTCTT	60.036	TGCCTTTGCAATTCCGTGTG	59.968	176
TRINITY_Contig894	1	p3	(AAC)7	21	385	405	ATCCTCAATCGCCAGCAACA	60.035	GCCAACACGAACTCTCTCCA	59.966	119
TRINITY_Contig894	2	p5	(CGCCG)3	15	1106	1120	ATCCTCAATCGCCAGCAACA	60.035	GCCAACACGAACTCTCTCCA	59.966	119
TRINITY_Contig894	3	p3	(CCA)7	21	1314	1334	ATCCTCAATCGCCAGCAACA	60.035	GCCAACACGAACTCTCTCCA	59.966	119
TRINITY_Contig895	1	p4	(TTGA)3	12	711	722	ACAAGAAAATGCTGCGCGTT	59.969	GTTGGGTTGGGCTCAGATGA	59.962	147
TRINITY_Contig897	1	p2	(CA)10	20	2	21	TCCAAGGAGGAAGAGGACGT	59.885	GCAGCTTGCTGATGTCGATG	59.972	205
TRINITY_Contig897	2	p3	(GAG)4	12	149	160	TCCAAGGAGGAAGAGGACGT	59.885	GCAGCTTGCTGATGTCGATG	59.972	205
TRINITY_Contig897	3	p3	(CGC)4	12	644	655	TCCAAGGAGGAAGAGGACGT	59.885	GCAGCTTGCTGATGTCGATG	59.972	205
TRINITY_Contig897	4	p3	(GCC)4	12	759	770	TCCAAGGAGGAAGAGGACGT	59.885	GCAGCTTGCTGATGTCGATG	59.972	205
TRINITY_Contig898	1	p3	(CCG)4	12	381	392	CGGAAGTCAGGTACGGATCG	59.971	CTCCATGATCTCGGGCTTCC	59.965	227
TRINITY_Contig898	2	p5	(CCCGG)3	15	551	565	CGGAAGTCAGGTACGGATCG	59.971	CTCCATGATCTCGGGCTTCC	59.965	227
TRINITY_Contig898	3	p3	(CCG)4	12	731	742	CGGAAGTCAGGTACGGATCG	59.971	CTCCATGATCTCGGGCTTCC	59.965	227
TRINITY_Contig898	4	p2	(AT)23	46	1315	1360	CGGAAGTCAGGTACGGATCG	59.971	CTCCATGATCTCGGGCTTCC	59.965	227
TRINITY_Contig899	1	p3	(TGC)5	15	634	648	TGAGGCAGCTGTTCCAGAAG	59.964	TGCGCTGGATCAACTACCTG	60.108	174
TRINITY_Contig899	2	p3	(CGC)4	12	1103	1114	TGAGGCAGCTGTTCCAGAAG	59.964	TGCGCTGGATCAACTACCTG	60.108	174
TRINITY_Contig899	3	p4	(CTGC)3	12	1256	1267	TGAGGCAGCTGTTCCAGAAG	59.964	TGCGCTGGATCAACTACCTG	60.108	174
TRINITY_Contig901	1	p4	(TGCA)3	12	126	137	AAGGCGTCGTGGAGAAGAAG	60.038	CGCCAACACCAACTCCTACT	59.965	126
TRINITY_Contig903	1	p3	(TGG)4	12	29	40	GTGGCTTTATCCCTGCCCTT	60.033	CGTACGACCATGGTCCAACA	60.038	262
TRINITY_Contig904	1	p5	(GCATG)3	15	598	612	TCTTCCCTCTCCTCCGTCTG	60.034	CAGGTTCGACGACTTGGACA	59.968	254
TRINITY_Contig905	1	p5	(GAGAT)3	15	1300	1314	GTCGTAGTAGTTCTCCGCCG	59.973	CTCTAGGCTCACACCAGCAC	60.108	155
TRINITY_Contig906	1	p3	(CGG)4	12	145	156	AGCCAGAACCCACCAAACAA	60.033	GTCCTTCTTGGCCCTCTTCC	60.035	220
TRINITY_Contig910	1	c	(TGGAT)3ctgcacatctatctattctatctgttcacaggaataataataaccccctgtgtatgtgctgctgccctgacctggctt(AGG)6gtgggtgctgggaatttctgttagggccaggagctcagggcaggagcaaacagatccctgc(AAAG)3	183	357	539	CGGGGAGGAGAGATTCATGC	59.965	AGATCAAATGGCACCTGGGG	60.033	140
TRINITY_Contig912	1	p3	(GGC)4	12	1080	1091	TGCAGTCTCATGCCGTCATT	60.036	GGACCTGCTGTAGTGCTGTT	59.965	116
TRINITY_Contig915	1	c	(GCT)4gaggaggcggccaagaaacagcac(CCG)4	48	1052	1099	CAACAGACTGCTGCCTCTGA	59.966	CTGGGGCAAGTCTAGCAACA	59.963	200
TRINITY_Contig916	1	p6	(CGCCCG)4	24	128	151	GTTTGGCTCCAGGTTGCATG	60.038	GGGAAAACGGGCAAAATCCC	60.037	124
TRINITY_Contig917	1	p3	(GCG)5	15	401	415	CATCCTGTAGTGGTTGCCGT	60.036	ATCACTTACGCGTGCAGGAA	60.038	210
TRINITY_Contig917	2	p3	(GCC)4	12	535	546	CATCCTGTAGTGGTTGCCGT	60.036	ATCACTTACGCGTGCAGGAA	60.038	210
TRINITY_Contig918	1	c	(GCG)4(GCT)4	24	1121	1144	CGTGCCCCACTGATAACCAT	60.107	GGCCCTGACTTCCATGTGAA	59.962	197
TRINITY_Contig918	2	p3	(CGC)5	15	1316	1330	CGTGCCCCACTGATAACCAT	60.107	GGCCCTGACTTCCATGTGAA	59.962	197
TRINITY_Contig920	1	p3	(CGC)4	12	1797	1808	TGAAGGTCGAACACTTCCGG	59.967	TGCATGTCAGGTCAGGTGTC	59.965	113
TRINITY_Contig920	2	p4	(TATC)3	12	1981	1992	TGAAGGTCGAACACTTCCGG	59.967	TGCATGTCAGGTCAGGTGTC	59.965	113
TRINITY_Contig923	1	p3	(GAC)4	12	661	672	GCTCTTGTCCATGAAGGGCT	60.035	GCCCATCTATCTGGCCAGAC	59.964	154
TRINITY_Contig924	1	p6	(CTGGCT)3	18	308	325	CTCACCTCTGGGATCCTGGA	60.032	CATCGGGGTATAGCCAGCTG	60.037	136

TRINITY_Contig924	2	p3	(GCG)4	12	717	728	CTCACCTCTGGGATCCTGGA	60.032	CATCGGGGTATAGCCAGCTG	60.037	136
TRINITY_Contig924	3	p3	(CGA)4	12	1241	1252	CTCACCTCTGGGATCCTGGA	60.032	CATCGGGGTATAGCCAGCTG	60.037	136
TRINITY_Contig926	1	p3	(GCC)4	12	714	725	GGCAGTAGCTCGTGTCAGAG	60.179	ATGCCCAAGGACTGTCATGG	60.034	135
TRINITY_Contig926	2	p2	(GC)6	12	860	871	GGCAGTAGCTCGTGTCAGAG	60.179	ATGCCCAAGGACTGTCATGG	60.034	135
TRINITY_Contig926	3	p4	(CCGG)4	16	1066	1081	GGCAGTAGCTCGTGTCAGAG	60.179	ATGCCCAAGGACTGTCATGG	60.034	135
TRINITY_Contig927	1	c	(GCC)4gctcttccgccgccgtgaagcgagaagacgcaggagaagatcaaccacctctggagtgctgtcgacggcgacattgcgaacaactcaa(GAC)4acggccggtgc(TAGCT)6	153	1187	1339	ATGAAGTCGTTGGTGCCGAT	60.036	GCCTACTTCGAGGAGTACGC	59.971	113
TRINITY_Contig928	1	p3	(GCC)4	12	787	798	GAGCACGACTTCAAGGGTCA	59.966	TGGTGATGAGCTTTCCGGTC	60.036	139
TRINITY_Contig929	1	p3	(GCG)4	12	383	394	TGGTGGTCGAAGCTGAACTC	59.966	GAGCGAGAGGTGGAAGTGTC	60.109	104
TRINITY_Contig933	1	p3	(CGC)4	12	269	280	TTTCGTGTTTTACAGCGCCG	60.042	CGTACCCCAGTTCACTGTCC	60.038	215
TRINITY_Contig933	2	p4	(CTTT)3	12	1018	1029	TTTCGTGTTTTACAGCGCCG	60.042	CGTACCCCAGTTCACTGTCC	60.038	215
TRINITY_Contig934	1	p3	(AAG)4	12	112	123	ACAGGATGGAGCACAGGTTG	59.963	ACTCCGTCTGCGAATCCATC	59.897	135
TRINITY_Contig935	1	p4	(AGGG)3	12	875	886	GCAGGGAGAGAGAGAAGGGA	60.033	CCCTTCTTCCCTCCTCCTCA	59.957	119
TRINITY_Contig935	2	p2	(GA)6	12	1332	1343	GCAGGGAGAGAGAGAAGGGA	60.033	CCCTTCTTCCCTCCTCCTCA	59.957	119
TRINITY_Contig936	1	p3	(AGC)4	12	280	291	CTTGCTTGGGTTGGGTTTCG	59.968	CGAGGGTACGGACGAAGATG	59.971	262
TRINITY_Contig936	2	p4	(AGCA)3	12	482	493	CTTGCTTGGGTTGGGTTTCG	59.968	CGAGGGTACGGACGAAGATG	59.971	262
TRINITY_Contig936	3	c	(CGCCT)3cgccgaccggggttgggatgtcgggcacggaaccgcgac(CCAA)3	66	651	716	CTTGCTTGGGTTGGGTTTCG	59.968	CGAGGGTACGGACGAAGATG	59.971	262
TRINITY_Contig938	1	c	(CAC)4(CAG)4	24	94	117	AGCGAAAAGGAGCACGAGAA	59.966	GGTTTCCTCCACATCCACGT	59.964	259
TRINITY_Contig938	2	p4	(AGAT)3	12	759	770	AGCGAAAAGGAGCACGAGAA	59.966	GGTTTCCTCCACATCCACGT	59.964	259
TRINITY_Contig938	3	c	(AGC)5aga(AGACG)3agagaacagaactagtataagtcaagcttaccatcttgctataacaagtggtggagtgtgtgtggaggcgacgacgacggaca(GATCAG)4	140	1158	1297	AGCGAAAAGGAGCACGAGAA	59.966	GGTTTCCTCCACATCCACGT	59.964	259
TRINITY_Contig938	4	p4	(CGGC)3	12	2844	2855	AGCGAAAAGGAGCACGAGAA	59.966	GGTTTCCTCCACATCCACGT	59.964	259
TRINITY_Contig940	1	c	(GCGCCG)3ccgctgcctgggtggcgtaggaggacgcggaggcgacccatttggccgcgacggggagcggccggggt(GGGGGC)3gggtgcgggaggcgccg(GGC)4	133	1075	1207	AGTCACACTCACACGAGCAG	59.969	TTCCGAGCACTCCTGAAACC	59.965	195
TRINITY_Contig943	1	p4	(CCCG)3	12	103	114	CAATGAAATGTGGCGTGCCA	60.038	GTCCTCGCTTCACCCTTGAA	59.965	248
TRINITY_Contig946	1	p5	(AGCAC)4	20	22	41	CTTCTCCTTCACAGGCAGCA	59.964	GATCGGAGGAGGGATCGAGA	59.963	151
TRINITY_Contig946	2	p3	(GGT)4	12	486	497	CTTCTCCTTCACAGGCAGCA	59.964	GATCGGAGGAGGGATCGAGA	59.963	151
TRINITY_Contig946	3	p3	(CGC)4	12	656	667	CTTCTCCTTCACAGGCAGCA	59.964	GATCGGAGGAGGGATCGAGA	59.963	151
TRINITY_Contig946	4	p4	(ATAC)3	12	1218	1229	CTTCTCCTTCACAGGCAGCA	59.964	GATCGGAGGAGGGATCGAGA	59.963	151
TRINITY_Contig949	1	p3	(CCT)5	15	556	570	CCTTGATGCCAATGATGCCG	59.968	TATAATACACACCGCCCGCC	59.966	144
TRINITY_Contig953	1	c	(TGC)5cgccgcgctgccgccggcgtacaccggcgtgctgtactacacg(CAC)4	70	456	525	CGTCGTCTTCCTCTACGTCG	59.975	AGGTAGCACAGGACTTGCAC	59.965	277
TRINITY_Contig955	1	p5	(AGAGG)4	20	30	49	TGCTTCTTCAGGCAGTGGAG	59.964	GCCTGAAGAACAGCACTCCT	59.964	114
TRINITY_Contig956	1	p5	(CAGAG)3	15	1220	1234	GAGAAGGCCTCTACGCTCAC	59.898	ATCAGCCATCAGGTGCTTCC	60.107	199
TRINITY_Contig959	1	p6	(GGGGCT)4	24	31	54	GATGAAAGGTGGGAGGGTGG	60.034	GCCCCTCATACGGTAGCTTC	59.966	131
TRINITY_Contig961	1	p6	(GCCGGC)3	18	693	710	GGTCCTTCCTCTGTTCCAGC	60.036	GGTACGCTAACCCATACGCA	59.897	244
TRINITY_Contig962	1	p3	(GCG)5	15	427	441	GTCCTCCTCCTCCTTCACCT	59.958	GCAAGAACCGCGTCGATTAC	59.975	197
TRINITY_Contig962	2	p3	(TCC)4	12	760	771	GTCCTCCTCCTCCTTCACCT	59.958	GCAAGAACCGCGTCGATTAC	59.975	197
TRINITY_Contig962	3	p3	(CGG)6	18	873	890	GTCCTCCTCCTCCTTCACCT	59.958	GCAAGAACCGCGTCGATTAC	59.975	197
TRINITY_Contig962	4	p4	(TGGC)3	12	1224	1235	GTCCTCCTCCTCCTTCACCT	59.958	GCAAGAACCGCGTCGATTAC	59.975	197
TRINITY_Contig963	1	p3	(GAG)7	21	853	873	AAAGCAGGCAGAGCAAAAGC	59.967	TTTTATCCCCTCCGATCGCG	59.967	176
TRINITY_Contig963	2	p3	(AGC)4	12	1105	1116	AAAGCAGGCAGAGCAAAAGC	59.967	TTTTATCCCCTCCGATCGCG	59.967	176
TRINITY_Contig966	1	p3	(CGC)5	15	475	489	ACGAAAACCAGCTCCCTCTG	59.965	ATCGATCAGCTTCTCTGGCG	59.968	197
TRINITY_Contig966	2	p3	(CCG)4	12	1098	1109	ACGAAAACCAGCTCCCTCTG	59.965	ATCGATCAGCTTCTCTGGCG	59.968	197
TRINITY_Contig969	1	p3	(CTT)5	15	508	522	CCAACGGAATCCAGTCGTCA	60.038	CGCGGCAAGAACTTTTTGGA	59.97	253
TRINITY_Contig970	1	p2	(GT)9	18	298	315	TGTGTGACCGAGTTCAGTCG	59.97	TGCACGCCAGTTACATGGAT	60.036	148
TRINITY_Contig970	2	c	(GC)7tgctccccgcctcctcgacctcgccctcccccg(TC)6	59	1147	1205	TGTGTGACCGAGTTCAGTCG	59.97	TGCACGCCAGTTACATGGAT	60.036	148
TRINITY_Contig971	1	p3	(GTT)4	12	1172	1183	AAGGGGTGCTCACTGTGATG	59.963	TCGTCCTTGGAACCTTCAGC	59.965	121
TRINITY_Contig972	1	p3	(GCG)4	12	280	291	AGTACTCGTCGCGCAAGAAA	60.04	TGGCGATGATGTTGGAGGTC	60.108	188
TRINITY_Contig972	2	p5	(GGAGC)3	15	865	879	AGTACTCGTCGCGCAAGAAA	60.04	TGGCGATGATGTTGGAGGTC	60.108	188
TRINITY_Contig973	1	p3	(GAG)4	12	1173	1184	AGCCAAATTGGAGGGATGGG	60.032	AACACCAGGATCGTGCAGAG	60.037	193
TRINITY_Contig975	1	c	(GCGCG)3tcacggcacgccaccttcgcgaacccctcgtcgtctcccccgccaccatgcggccgcattaggaagtaggagtaggagcgagagaccgag(GACC)3	117	54	170	CCCCGTGTCATCATCCTACG	59.968	ATTGTCAAGGAGCACGACGT	59.966	148
TRINITY_Contig975	2	c	(GGCG)3agcgggcaggcagccaggcaggcaagcggtgcccgtcgcccgctcccсttccacctgttgcgtgctcccggcctcaagaaa(CCG)4	105	371	475	CCCCGTGTCATCATCCTACG	59.968	ATTGTCAAGGAGCACGACGT	59.966	148
TRINITY_Contig975	3	p2	(TC)6	12	796	807	CCCCGTGTCATCATCCTACG	59.968	ATTGTCAAGGAGCACGACGT	59.966	148
TRINITY_Contig977	1	c	(TCG)4tcctggtccgccgcctccgcgccgcccacccccacgtcggccgcggacgccaagaacccgcgccgcgcctcggcgtcctcgccgcgctccaa(CAGCGG)3	122	444	565	TGCAGCGACAATGATCCACT	60.036	TACCCCATCTCCATGCTCCA	60.03	168
TRINITY_Contig977	2	p3	(CCG)4	12	759	770	TGCAGCGACAATGATCCACT	60.036	TACCCCATCTCCATGCTCCA	60.03	168
TRINITY_Contig977	3	p3	(ACG)4	12	1059	1070	TGCAGCGACAATGATCCACT	60.036	TACCCCATCTCCATGCTCCA	60.03	168
TRINITY_Contig977	4	p3	(AGA)4	12	1429	1440	TGCAGCGACAATGATCCACT	60.036	TACCCCATCTCCATGCTCCA	60.03	168
TRINITY_Contig977	5	p3	(GCG)4	12	1805	1816	TGCAGCGACAATGATCCACT	60.036	TACCCCATCTCCATGCTCCA	60.03	168
TRINITY_Contig977	6	c*	(GGACGA)2(G<GACGA>CGACGACGAGCGCTTGGCCTTGCGGCAGCCGCCGCCGACGGGGACGTTGCGGAGGACGCCGCCCTTGGTCCAGTACCGGCGGCAGCTCTTGCAGAAGTGGCGCGGCTGCGACAGGTTGTAGTTGTTGTAGTAGCAGAACTTGGTGTTGGTGGACTCGCAGCGCGGGCACTTGACCGCCTCGGGCGCCGGCGACACCCCGCCGCACCGCGCCCCGGGGCCCTGCTGCGCCTGCGCGCGCCGGAGGTCCGCCGCCGCGGCTCCGCCGAACAGCCGCCCCGCCAGGCCCGGGATCGACTGGAACTCCTGCATGCTGGCGGCAAGCGGTGTTGGCCTCCGCGCGCGACGTGCTCGCTAGGATATCCCCCCTGGCTGGATCAGCCTCAGCTTAGTGGGTGTGTGTTTGCCGAGGACTAGA	25	2106	2130	TGCAGCGACAATGATCCACT	60.036	TACCCCATCTCCATGCTCCA	60.03	168
TRINITY_Contig978	1	p3	(GCT)4	12	1317	1328	GGTCGCAACCAATCCCTGTA	60.036	GGGGGCTCTTCTACCCAAAC	60.034	141
TRINITY_Contig980	1	c	(CGA)5cgcgcagccgccgcggacgccgcccgcgggcgccagcggtatgcacgacggcgtgttccccatacccg(CCAA)3	95	808	902	TGGTTCCCGAGCACAAAACT	60.107	GCTTTGGTTTCGCTGGTGTT	59.898	178
TRINITY_Contig981	1	p4	(CCCG)3	12	175	186	TTGTGGCTTTGAATGCAGGC	59.966	ATCAGTGTAGACAGCGCACC	60.109	172
TRINITY_Contig982	1	p3	(CCG)4	12	920	931	CCGGTGAAAACCCAGGATCA	59.962	GCAGCAGACCCCTGAAAGAT	60.035	124
TRINITY_Contig983	1	c	(CCG)4ga(GCC)5	29	784	812	TTTTCGCCCCTGAGAAGTCC	59.964	GTCAGAAGCAGATGGGCCTT	60.035	105
TRINITY_Contig983	2	p3	(CTG)4	12	1079	1090	TTTTCGCCCCTGAGAAGTCC	59.964	GTCAGAAGCAGATGGGCCTT	60.035	105
TRINITY_Contig984	1	p3	(CGC)4	12	81	92	AAGCTGAAGGGGAACGTGAG	59.965	TGTCTTGTTCTTGGCCAGCA	60.107	114
TRINITY_Contig984	2	c	(TCCGGC)3agcggaccggcgccgcggtgcagcccggaccaggagaacatgccgctggccgtggactcgctgcagttcgagcgcaccggcagcgcgagcgcg(GCC)4	123	925	1047	AAGCTGAAGGGGAACGTGAG	59.965	TGTCTTGTTCTTGGCCAGCA	60.107	114
TRINITY_Contig985	1	p3	(CTC)5	15	74	88	GGTTTCGGTGGATGCAGGTA	60.036	TCCAGCCGACGAAATCACAA	59.966	111
TRINITY_Contig985	2	p3	(CGC)4	12	252	263	GGTTTCGGTGGATGCAGGTA	60.036	TCCAGCCGACGAAATCACAA	59.966	111
TRINITY_Contig985	3	p3	(GCA)4	12	965	976	GGTTTCGGTGGATGCAGGTA	60.036	TCCAGCCGACGAAATCACAA	59.966	111
TRINITY_Contig989	1	p3	(AAG)5	15	1180	1194	TATCGACACCATGCCATCGG	59.967	AGGTCACCGAACTTGCAGAG	59.966	222
TRINITY_Contig990	1	p3	(GCT)4	12	838	849	CAGATGCCGGTGGAATACGA	59.896	ATCCTCCCATCTCCCTGCAT	60.104	195
TRINITY_Contig991	1	p6	(GGCGGA)4	24	731	754	GGCGTCATCCAGATCCTCAG	59.967	TGTGGAAGTACACGAGCACC	59.967	208
TRINITY_Contig992	1	p5	(CCGTC)3	15	76	90	AGGTATCTTTGGCACGGCAA	59.962	CTGGGTGCCATCTAAGTGCA	60.035	105
TRINITY_Contig992	2	p3	(GCG)4	12	227	238	AGGTATCTTTGGCACGGCAA	59.962	CTGGGTGCCATCTAAGTGCA	60.035	105

TRINITY_Contig993	1	p3	(CCG)4	12	464	475	ACCTACGGGGATCTTCGGAA	60.033	TTACAGGGAGTGGCGCATTT	59.962	223
TRINITY_Contig994	1	p3	(CGA)6	18	435	452	CCTAGGCTGCTCAACGACAA	60.037	AGAACACCATGAGTCCGCAG	60.037	106
TRINITY_Contig994	2	p3	(ACC)6	18	928	945	CCTAGGCTGCTCAACGACAA	60.037	AGAACACCATGAGTCCGCAG	60.037	106
TRINITY_Contig995	1	p5	(CAGCA)3	15	289	303	ACACTCATCACACACGTCCC	59.966	TTTTGGAGGCTTGCGCTTTC	59.968	254
TRINITY_Contig996	1	p3	(AGC)4	12	714	725	GGGGCATAAGCTGGACAGTT	60.034	ATGGCCTTCTCCTACCTCGT	60.032	256
TRINITY_Contig996	2	c	(CCG)4gcccccaagc(CGCCGA)3cgccca(GCG)4	58	837	894	GGGGCATAAGCTGGACAGTT	60.034	ATGGCCTTCTCCTACCTCGT	60.032	256
TRINITY_Contig996	3	p3	(GAT)5	15	1231	1245	GGGGCATAAGCTGGACAGTT	60.034	ATGGCCTTCTCCTACCTCGT	60.032	256
TRINITY_Contig997	1	c	(GCT)4g(CGA)5	28	210	237	ACGTCATGTACAAGCGACGT	60.04	TCATCATCGTCGTCGTCGTC	59.974	187
TRINITY_Contig997	2	p6	(GCTGGA)3	18	981	998	ACGTCATGTACAAGCGACGT	60.04	TCATCATCGTCGTCGTCGTC	59.974	187
TRINITY_Contig998	1	c*	(GCC)4actggccagc(CGGG)3(GGC)4*	44	159	202	TCGTTCTGGTGTTGCCCTAC	59.966	AAGGGAGAAGAGGCGGAGAT	60.032	135
TRINITY_Contig998	2	c	(GCC)6acctgctcgggagcaagcttcggtgtcttgggtgggcgtggactgtggctgcgt(CGC)5	87	433	519	TCGTTCTGGTGTTGCCCTAC	59.966	AAGGGAGAAGAGGCGGAGAT	60.032	135
TRINITY_Contig999	1	p4	(TACA)3	12	1106	1117	CGCGATGCTGTGGATCTACT	59.969	AGGATCTTGGCCGTGAACAG	60.036	153
TRINITY_Contig1001	1	p4	(GGAG)3	12	699	710	AGGAAGGGTTGGCTACATGC	60.034	CACTGCTCTCCGCTCTATGG	59.968	182
TRINITY_Contig1003	1	c	(CT)18(CGCT)3	48	43	90	CGCACAATCTTATGGTGCCG	59.972	CTTCCGCCTTTTGCACTGTC	60.04	261
TRINITY_Contig1005	1	p3	(GCC)4	12	715	726	GATCGAGAGCATGGAGAGCC	60.038	GTCAACCTCCCCGGCAATAA	60.035	162
TRINITY_Contig1005	2	p3	(CCG)5	15	895	909	GATCGAGAGCATGGAGAGCC	60.038	GTCAACCTCCCCGGCAATAA	60.035	162
TRINITY_Contig1005	3	p4	(TCGA)3	12	1267	1278	GATCGAGAGCATGGAGAGCC	60.038	GTCAACCTCCCCGGCAATAA	60.035	162
TRINITY_Contig1008	1	p3	(CCG)4	12	2037	2048	GGTAGTCTCCGAGCATGACG	59.97	GTCTACCTCACCCTCATGCG	59.897	244
TRINITY_Contig1008	2	p3	(GCG)4	12	2262	2273	GGTAGTCTCCGAGCATGACG	59.97	GTCTACCTCACCCTCATGCG	59.897	244
TRINITY_Contig1010	1	p6	(GCCGGC)3	18	1030	1047	ATGGCTAGCCGCTCTTCATC	59.965	GGAAGCCGATGATCGAGGAG	60.039	136
TRINITY_Contig1010	2	p4	(AGCT)3	12	1394	1405	ATGGCTAGCCGCTCTTCATC	59.965	GGAAGCCGATGATCGAGGAG	60.039	136
TRINITY_Contig1011	1	p3	(GCC)4	12	19	30	TACTCCGACGACACCCTGAT	60.035	CTCCGTCCTGTACTCATCGC	59.97	274
TRINITY_Contig1011	2	c	(AGC)4gaggactggacgttcctccgcaccacggcgctccactc(GCT)7	71	1155	1225	TACTCCGACGACACCCTGAT	60.035	CTCCGTCCTGTACTCATCGC	59.97	274
TRINITY_Contig1013	1	p3	(GAC)4	12	785	796	CTTCATGGCCTCCTGCTCTC	60.179	TCGCTCCGCCGCTTTTATAA	60.179	164
TRINITY_Contig1013	2	p3	(CGC)4	12	969	980	CTTCATGGCCTCCTGCTCTC	60.179	TCGCTCCGCCGCTTTTATAA	60.179	164
TRINITY_Contig1017	1	c	(GAG)4(GTG)5	27	1263	1289	CCGATCCAGACGTACTCAGC	59.97	CCTCCTCCTCTCCACCATGA	60.032	105
TRINITY_Contig1018	1	p3	(CGG)5	15	192	206	TCCCTTTCCACTCAGGTTGC	59.89	GCCAGCCTCCTCTTCCATTT	60.034	266
TRINITY_Contig1019	1	c	(CCT)6cgacggcggcggacgcctcggcggcagcg(TCC)6	65	890	954	GCCCATCCAAACTCTGTCCA	59.962	AAGCAGCTCCTAGAGGCTCT	60.032	267
TRINITY_Contig1019	2	c	(GGGA)4ggggaaggggataaga(GAGG)3	44	1236	1279	GCCCATCCAAACTCTGTCCA	59.962	AAGCAGCTCCTAGAGGCTCT	60.032	267
TRINITY_Contig1020	1	p3	(TGC)4	12	1197	1208	TCATGCGTATAGGCTGCTGG	59.966	CTTGTTGTGGTTGTGGCTCG	59.971	172
TRINITY_Contig1020	2	p3	(CCG)5	15	5268	5282	TCATGCGTATAGGCTGCTGG	59.966	CTTGTTGTGGTTGTGGCTCG	59.971	172
TRINITY_Contig1020	3	c	(GTG)4cggcgaaccaattcgccggaacggcaa(AGGG)3	51	5396	5446	TCATGCGTATAGGCTGCTGG	59.966	CTTGTTGTGGTTGTGGCTCG	59.971	172
TRINITY_Contig1021	1	c	(GGC)4ggaatgatggaggccggcggagggggagc(GGGA)3	53	83	135	ACCGATGCACAGCAGAAAGA	59.965	CAAAAACAACCCGCTCCTGG	59.968	188
TRINITY_Contig1023	1	p4	(CATG)3	12	76	87	GTAGTTGGTCCACCTCAGCC	60.036	AGACCTCCGACTTCACTCGA	59.964	279
TRINITY_Contig1023	2	p3	(TCG)4	12	574	585	GTAGTTGGTCCACCTCAGCC	60.036	AGACCTCCGACTTCACTCGA	59.964	279
TRINITY_Contig1023	3	p3	(CCT)4	12	815	826	GTAGTTGGTCCACCTCAGCC	60.036	AGACCTCCGACTTCACTCGA	59.964	279
TRINITY_Contig1024	1	p3	(CTC)6	18	42	59	CAACGACTACCACCTGTCCC	60.038	GACGATCAGCCTCTCGATGG	60.04	106
TRINITY_Contig1031	1	p4	(GCCG)4	16	537	552	GCGACACAAGGAGTCAAGGA	59.966	ACTACTGTGTCTGCCCGTTG	59.966	178
TRINITY_Contig1032	1	p4	(ACCG)3	12	427	438	TCGCGCTAGTTAACCCGATC	59.97	CGCCCTGGTTGATTCTGTCT	60.036	110
TRINITY_Contig1032	2	c	(CGG)5tgggggcgggtacgcgaggcggcctcagcctcacgaggaggagacccacggccccgtgggctctgggtacggctacgggaggaagggac(ACG)6agcagaaggcgtaccggaagcccgaagccggcgtacggg(GAC)4	172	721	892	TCGCGCTAGTTAACCCGATC	59.97	CGCCCTGGTTGATTCTGTCT	60.036	110
TRINITY_Contig1033	1	p3	(GGC)4	12	375	386	GTGCTTGTGCGTGATTTGGT	59.969	TTAGCCCAGCACTCCTCTCT	59.958	247
TRINITY_Contig1033	2	p3	(AGC)4	12	511	522	GTGCTTGTGCGTGATTTGGT	59.969	TTAGCCCAGCACTCCTCTCT	59.958	247
TRINITY_Contig1037	1	p3	(GCC)8	24	799	822	TGGACTCGTCTCCGATCCTT	60.034	CATGCCGAGCTCCAGGTTTA	60.108	223
TRINITY_Contig1038	1	p3	(CGA)4	12	356	367	CACCTCCAAGGACGTTGTCA	59.894	AAGAGGAGGATCGGGAGCTT	60.032	114
TRINITY_Contig1038	2	p6	(GAGGGC)3	18	1015	1032	CACCTCCAAGGACGTTGTCA	59.894	AAGAGGAGGATCGGGAGCTT	60.032	114
TRINITY_Contig1039	1	p4	(AAGA)3	12	622	633	TCGTGTAGTTGCGAACACGA	59.971	GTAATGCAGCGAAGGCGAAG	59.973	152
TRINITY_Contig1039	2	p3	(GCC)6	18	770	787	TCGTGTAGTTGCGAACACGA	59.971	GTAATGCAGCGAAGGCGAAG	59.973	152
TRINITY_Contig1039	3	c	(ACG)4cgccgccgccgtcctccatcgccatggcgatcctcgcgagcctca(TCT)4	69	1123	1191	TCGTGTAGTTGCGAACACGA	59.971	GTAATGCAGCGAAGGCGAAG	59.973	152
TRINITY_Contig1040	1	p3	(CCG)4	12	759	770	GTGAGCAGGTACGGGATGAG	59.897	CCCGCGGGTCATATAAAGCT	59.965	255
TRINITY_Contig1042	1	p3	(GAC)5	15	1038	1052	CAGGAACAAACCAACGGCTG	59.969	AGCAAGAAGATGGTGCGGAA	59.963	272
TRINITY_Contig1043	1	p3	(AGC)4	12	800	811	ACACAAACGAAACGAGCACG	59.974	ACCTGCAATGCAAGCACATG	60.038	157
TRINITY_Contig1044	1	p4	(AACC)3	12	74	85	TGCAGTGCTTCCTGATCTCG	60.108	CGCCAACCAGGACATCTTCT	60.036	104
TRINITY_Contig1045	1	p4	(TCCC)3	12	153	164	GTACACCGAGTTCACAGGCA	59.967	TCAAGAAAGGCCACCGAGAC	59.965	228
TRINITY_Contig1045	2	p6	(GCGCCG)3	18	415	432	GTACACCGAGTTCACAGGCA	59.967	TCAAGAAAGGCCACCGAGAC	59.965	228
TRINITY_Contig1048	1	p3	(GCG)6	18	625	642	TGGATCTTCTTGCCGGTGAC	60.036	ATCTAGGGTTTTAGCCGCCG	59.894	233
TRINITY_Contig1048	2	p3	(TGC)4	12	746	757	TGGATCTTCTTGCCGGTGAC	60.036	ATCTAGGGTTTTAGCCGCCG	59.894	233
TRINITY_Contig1050	1	p3	(CTC)4	12	1024	1035	TGAAGCCGCTTGAAGCAAAC	59.969	TCAAAGAAGCGCTGAGAGCA	59.966	168
TRINITY_Contig1050	2	p2	(TC)19	38	1219	1256	TGAAGCCGCTTGAAGCAAAC	59.969	TCAAAGAAGCGCTGAGAGCA	59.966	168
TRINITY_Contig1052	1	p3	(CGC)4	12	65	76	TCATCGTCTGGCCAGTTCAC	60.037	GACCATCAAGTCTGCACCCA	59.963	272
TRINITY_Contig1053	1	p4	(CGCC)3	12	1070	1081	TTCCACCCCGAACCATTAGC	60.035	CCACACGCACAACTCAACTG	59.972	212
TRINITY_Contig1056	1	p3	(TGG)4	12	944	955	TGATGGTGGTGGTGGTGATG	59.962	TTGGTATCGGCGGATGGATG	59.965	205
TRINITY_Contig1056	2	p4	(CATC)3	12	1122	1133	TGATGGTGGTGGTGGTGATG	59.962	TTGGTATCGGCGGATGGATG	59.965	205
TRINITY_Contig1057	1	p3	(GCG)5	15	926	940	CCCTACAGGTTCGGGGACTA	60.033	AGCACCATTAGTCAACCGCA	59.964	274
TRINITY_Contig1057	2	p3	(TGA)4	12	1122	1133	CCCTACAGGTTCGGGGACTA	60.033	AGCACCATTAGTCAACCGCA	59.964	274
TRINITY_Contig1058	1	p3	(CGC)4	12	386	397	ACGAACGGGAACTCGAACTC	60.04	AAAAGCGTCGTCGGAAAAGC	60.042	199
TRINITY_Contig1058	2	p6	(GAACTC)3	18	1023	1040	ACGAACGGGAACTCGAACTC	60.04	AAAAGCGTCGTCGGAAAAGC	60.042	199
TRINITY_Contig1060	1	p3	(CAG)5	15	1101	1115	GAAGCAGTCGTGGAAGTGGA	59.966	CTCACGGCTACTACCCTCCT	60.107	170
TRINITY_Contig1061	1	c	(CGCCGG)3catcgtcccttctcctttctcctgctcgcctcgaaggccacggcgaggcggcgagctgctgctccccctgc(GCG)6	107	1192	1298	GCACGACGATGGCAATAACC	59.972	GATCGTGCTCCTCCTTCTCG	59.97	158
TRINITY_Contig1067	1	p3	(CAG)4	12	369	380	GGCCCACTAAGCACTTCCAT	60.034	GACGTGCAAATCGATCGCAA	59.905	242
TRINITY_Contig1068	1	p3	(GAG)4	12	480	491	GGGTTGTAGACATGGCTGCT	60.035	CATAGCTGCGTCGCTGAAAC	59.974	110
TRINITY_Contig1068	2	p3	(ACC)7	21	1183	1203	GGGTTGTAGACATGGCTGCT	60.035	CATAGCTGCGTCGCTGAAAC	59.974	110
TRINITY_Contig1069	1	p5	(AGCCA)5	25	196	220	CGAGTTCCGTCAAGTCCACA	59.968	GCACGATCGATGGATTTGGC	60.041	121
TRINITY_Contig1071	1	p3	(GCG)4	12	1118	1129	AACTCAGCCCAATCCGTGAG	60.036	ATCCCGCTATCCCGCTAGAT	60.033	272
TRINITY_Contig1076	1	c	(CCG)5cgccgctcaggcggccctgcgcgatctggaagaccaaaagatggccatcgacacccagccgtgcgacggagccggcagcgccgcggccaggaa(CAG)8	133	853	985	GTAGAAGTCATCCTCGGCCG	59.969	GTGGGGTTTACCTCCGACAG	60.037	120
TRINITY_Contig1076	2	p2	(AG)6	12	1130	1141	GTAGAAGTCATCCTCGGCCG	59.969	GTGGGGTTTACCTCCGACAG	60.037	120
TRINITY_Contig1078	1	p3	(AGA)4	12	946	957	CCCGTCTCTTTCGTTTGGGA	59.966	TGACGATCTGGAGCTCAGGA	60.034	122
TRINITY_Contig1080	1	p3	(GCC)4	12	24	35	CTCGACTCGCTCATCATCCC	60.04	ATAAGCCCAGCACAGGTTCC	60.034	134
TRINITY_Contig1081	1	p5	(CGTGG)3	15	1139	1153	CTTCATGCGATCAAGCCAGC	59.971	GATCACCGCCTACTTCCTCG	59.969	226
TRINITY_Contig1082	1	p2	(TC)6	12	66	77	CAACCTGGCGTGTGTTTCTG	59.971	GTTGGGGTCATCCAGAGGTG	60.035	196
TRINITY_Contig1083	1	p3	(AGA)4	12	778	789	CAAGTGTCCCATAGCCAGCA	60.035	TCCGGTGAAAAATCCGCTGA	59.964	209
TRINITY_Contig1085	1	p4	(TTTA)4	16	832	847	AGTGCAGAGTTAGCTGGCAG	60.037	GCGCGACGTTGTTCTTTGAT	60.111	173
TRINITY_Contig1087	1	p5	(GCGGC)4	20	99	118	TCCTCTCCCAGCTGCTGTAT	60.032	TCCAGGCTCAAGCATCTTGG	60.035	142
TRINITY_Contig1091	1	p3	(CCT)4	12	726	737	GTGAGATTGTGCGCGACATC	59.974	ACGGATGTCAATGTCGCACT	60.037	254
TRINITY_Contig1092	1	p4	(TCAT)3	12	1192	1203	ACCTTGTCCATGCTCAGAGC	60.036	TCCTTGACCTTGAGTTCCGC	59.965	193
TRINITY_Contig1094	1	p4	(GAGG)3	12	2144	2155	CATCATCGCCATCGTCCTCA	59.967	CTACCACCTACCTGCCTCCT	60.032	235
TRINITY_Contig1097	1	p4	(TTCC)3	12	118	129	AAGAATTGAGCCCTGGCCTC	60.034	ACGGCAGGAACTTGAGGAAG	59.965	106
TRINITY_Contig1097	2	p3	(CCA)4	12	572	583	AAGAATTGAGCCCTGGCCTC	60.034	ACGGCAGGAACTTGAGGAAG	59.965	106
TRINITY_Contig1098	1	p5	(CATCT)3	15	39	53	TGGTGGTGTACGAGTGCTTC	59.967	GGCCCAGATGAAACCATCCA	60.033	107
TRINITY_Contig1098	2	p2	(TC)21	42	1172	1213	TGGTGGTGTACGAGTGCTTC	59.967	GGCCCAGATGAAACCATCCA	60.033	107
TRINITY_Contig1100	1	c	(GCTG)3ctcagcgcggc(GCG)5	38	414	451	GCCCTCGCATGAGATCATCA	59.966	CGGCGTTAACTGAGTCAGGT	60.039	125
TRINITY_Contig1102	1	p3	(GGC)4	12	253	264	TGAATTGCTGCCCCCAAGAT	59.958	TGAATGCCCATGGAAGCACT	59.96	253
TRINITY_Contig1104	1	p4	(GAGG)3	12	213	224	AACCACGAGACTCTGCAGTG	59.967	AGAAGCTTCCTTTCCCGTCG	60.037	107
TRINITY_Contig1104	2	p2	(CA)6	12	524	535	AACCACGAGACTCTGCAGTG	59.967	AGAAGCTTCCTTTCCCGTCG	60.037	107
TRINITY_Contig1105	1	p4	(AGCT)3	12	246	257	CAAGAAGGTTCCCGTCCTCC	60.036	TCCAACACTGCATGCTCGAT	60.036	252
TRINITY_Contig1108	1	p3	(GAG)5	15	561	575	CCCATTCCTGTTGGAGGCTT	59.96	CTCCGTATCTGCCGTCAGTC	59.97	211
TRINITY_Contig1110	1	p3	(GTC)4	12	412	423	GCTCCAGGACGGATAGAAGC	59.967	AGGAGGAGCAAGAAGGGACA	60.179	116

TRINITY_Contig1110	2	p3	(CTA)4	12	1047	1058	GCTCCAGGACGGATAGAAGC	59.967	AGGAGGAGCAAGAAGGGACA	60.179	116
TRINITY_Contig1112	1	p3	(GGC)5	15	294	308	GCTGATCGAGGAGGATGGTG	59.967	GTTGACTGCAGCCTTGTTGG	59.969	177
TRINITY_Contig1115	1	c*	(CAT)3(C<AT>CG)(ATCG)2	22	1	22	GGAGGTATCCTGAGGAGGCA	60.105	ACGTGCAAGCTACCGTACAA	59.968	199
TRINITY_Contig1115	2	p3	(CGC)4	12	475	486	GGAGGTATCCTGAGGAGGCA	60.105	ACGTGCAAGCTACCGTACAA	59.968	199
TRINITY_Contig1116	1	c	(GGGA)3agcggtagaa(CGG)4	34	281	314	TTCGACAGGAACTTGGCCTC	59.965	CCGCCTCCAATCTGATCTCC	59.965	156
TRINITY_Contig1116	2	p6	(GGAGGG)3	18	1097	1114	TTCGACAGGAACTTGGCCTC	59.965	CCGCCTCCAATCTGATCTCC	59.965	156
TRINITY_Contig1119	1	p3	(CGC)4	12	114	125	TCATCATCGCCTCGCTCTTC	59.969	CCCGAAGACAAAAGACCGGA	59.966	262
TRINITY_Contig1123	1	p4	(GCTC)3	12	117	128	CTGCTTTTCTATGCGTCGCC	59.973	TGTCTGCTCAGTATCCGGGA	60.034	101
TRINITY_Contig1124	1	p3	(GCG)5	15	1047	1061	AGTGGTAGAGCCGATCCAGT	60.033	ATCGACGTGGTTCCTTGCTT	59.965	182
TRINITY_Contig1125	1	p3	(GCA)4	12	12	23	CCTACCCTGGATCTTGGGGA	60.03	TCTTGTCGTGCACAGACTCC	59.968	140
TRINITY_Contig1126	1	p3	(CCG)7	21	47	67	CGGAGGCGTACGTCTTCTAC	59.973	TGATGAAGAAGAACGGGCCC	60.035	276
TRINITY_Contig1126	2	p3	(CGG)4	12	391	402	CGGAGGCGTACGTCTTCTAC	59.973	TGATGAAGAAGAACGGGCCC	60.035	276
TRINITY_Contig1126	3	p3	(CGG)6	18	659	676	CGGAGGCGTACGTCTTCTAC	59.973	TGATGAAGAAGAACGGGCCC	60.035	276
TRINITY_Contig1128	1	c	(CAC)6ccagctttgccttgcctcctcc(TCT)4	52	8	59	ATGAGCAATCAAGCAGCCCT	60.033	GAACATCGGAATTCCTGCGC	59.972	270
TRINITY_Contig1128	2	p4	(CGGC)3	12	581	592	ATGAGCAATCAAGCAGCCCT	60.033	GAACATCGGAATTCCTGCGC	59.972	270
TRINITY_Contig1128	3	p3	(GGC)4	12	707	718	ATGAGCAATCAAGCAGCCCT	60.033	GAACATCGGAATTCCTGCGC	59.972	270
TRINITY_Contig1129	1	p3	(CGG)4	12	877	888	TTGGATTGACGCATGGCTCT	60.035	TCGATCCAAGGAAAACGGCA	59.964	148
TRINITY_Contig1130	1	p4	(GGCG)3	12	658	669	CGATGGGTCGTCGTACAGAG	59.972	TTTGGGAGGCTTCCGGAATC	60.034	161
TRINITY_Contig1134	1	p4	(TGGA)3	12	861	872	AGCGAGTGGAAACTGGGAAG	59.965	CCAGTAGCACCAGCAAGGAA	59.963	128
TRINITY_Contig1138	1	c	(GCT)4ccgcgcctccattgcatctcttcttcctatgagcttgcggtatccatacctgatttctagaaaaccgttgtcaatcttggtcgtcgccactt(TCG)6	122	420	541	GAGCAAACCTCAAAGGCTGC	60.039	AAGTGGCGACGACCAAGATT	59.965	119
TRINITY_Contig1141	1	p4	(CTCA)3	12	46	57	CACCGACAACGAGGTCAAGA	59.968	GGCTCGCGTCGTAGTAGTAG	59.768	209
TRINITY_Contig1141	2	p3	(GCG)4	12	185	196	CACCGACAACGAGGTCAAGA	59.968	GGCTCGCGTCGTAGTAGTAG	59.768	209
TRINITY_Contig1141	3	c	(GCC)5gctgccgatgatgcttctcg(CTA)4	47	614	660	CACCGACAACGAGGTCAAGA	59.968	GGCTCGCGTCGTAGTAGTAG	59.768	209
TRINITY_Contig1146	1	p4	(CCCT)3	12	169	180	CCTTCTCCTCCCTAGCTGGT	60.032	CGGCAAATCAAGCGGAGATG	59.971	202
TRINITY_Contig1146	2	c	(CGG)4cccact(CTC)4	30	675	704	CCTTCTCCTCCCTAGCTGGT	60.032	CGGCAAATCAAGCGGAGATG	59.971	202
TRINITY_Contig1147	1	p2	(CT)6	12	130	141	GCCACCGTGATGTACAGGAA	60.037	CTCTCGCTCTCTTCGCTAGC	60.041	228
TRINITY_Contig1150	1	p4	(CCGG)3	12	1130	1141	ATTGCCAGGATGCTTGCTCT	60.033	GCCTCAAATCGCTAGCAAGC	59.971	103
TRINITY_Contig1151	1	p3	(CAT)4	12	159	170	GTTGAGCATAACGTCGTCGC	59.977	GGATCCTGGCCTTGGTCTTC	60.107	227
TRINITY_Contig1153	1	p3	(GCT)6	18	662	679	GTGTGGATGGTGGTTGCCTA	59.962	GAAGGAGATCTACGCGTGGG	59.969	178
TRINITY_Contig1153	2	p4	(GCTA)3	12	1113	1124	GTGTGGATGGTGGTTGCCTA	59.962	GAAGGAGATCTACGCGTGGG	59.969	178
TRINITY_Contig1156	1	p4	(CCGC)3	12	442	453	GCGCCTCGAAAACAAGAGTC	59.836	AACTTGGGACTCGCTGAAGG	59.965	142
TRINITY_Contig1157	1	p3	(TCA)4	12	300	311	GGCAAGCAACTGAAGTGCAA	59.897	ATCCGCGAAGTCAAAAGGGT	59.964	228
TRINITY_Contig1157	2	p3	(GGC)4	12	877	888	GGCAAGCAACTGAAGTGCAA	59.897	ATCCGCGAAGTCAAAAGGGT	59.964	228
TRINITY_Contig1158	1	p3	(GGA)4	12	386	397	GCTCCGCATCCCTATGGTAC	60.037	GCCGAACCACCCATAGGAAA	60.035	236
TRINITY_Contig1159	1	p4	(CGGA)3	12	160	171	CGGACAGGCTGGTGGTTTAT	60.035	CTCCGGCTGGATGAATCTCC	59.965	276
TRINITY_Contig1159	2	c	(CGG)5ccacgagggccacgaggagggcgacgtgcttggccatcgccatcgctaggac(GCTA)3	79	1072	1150	CGGACAGGCTGGTGGTTTAT	60.035	CTCCGGCTGGATGAATCTCC	59.965	276
TRINITY_Contig1163	1	p3	(CGC)4	12	491	502	TCACAATGTGGCGCAGTACT	59.965	GAGGTGAGCAAGTGGAAGCT	59.964	252
TRINITY_Contig1165	1	p3	(GCA)5	15	229	243	GGCAAAGTCCTCTGGCTTCT	59.963	CGCAGAAGATCGTCTCCCTC	59.97	242
TRINITY_Contig1166	1	p4	(CACG)3	12	457	468	GAGGACACGAGGAACACCTG	60.038	CACCGACTTGAGCTACGTGA	59.76	105
TRINITY_Contig1166	2	p3	(CGG)4	12	1004	1015	GAGGACACGAGGAACACCTG	60.038	CACCGACTTGAGCTACGTGA	59.76	105
TRINITY_Contig1167	1	c*	(GCTGCC)2(GCT<GCC>)(GCC)5tt(CTC)4cctcctctctctacacttgggcctctgcctctctctctatctctctctcat(GGC)4	110	9	118	CCAGTGAGATCGGGGCTTTT	60.035	CATTGACCAGCGGCGTAATG	59.972	264
TRINITY_Contig1170	1	p3	(GAC)4	12	37	48	CAAGCTCACTGGGTTTGTGC	59.969	CCCGAGCTTGAGGTCAACTT	59.965	142
TRINITY_Contig1171	1	p3	(GGT)4	12	454	465	CACACAAGCAAGACACTGCC	59.97	GCTCCTGACACCTCCTCCTA	60.033	145
TRINITY_Contig1171	2	c	(GCC)5gcggcgtcgccagcctcctcgcgcgggcgcttgccgaggacggtctgctggtggtagtgtgg(GGA)4	89	579	667	CACACAAGCAAGACACTGCC	59.97	GCTCCTGACACCTCCTCCTA	60.033	145
TRINITY_Contig1171	3	p3	(TGG)4	12	1125	1136	CACACAAGCAAGACACTGCC	59.97	GCTCCTGACACCTCCTCCTA	60.033	145
TRINITY_Contig1173	1	c	(GCCGC)3cgctcgccgccggcgccgacgcctccgtcgccgtcgcaacctccattttatt(CGAG)3	79	1049	1127	TCCACCACAAAACAAGGCCT	60.033	TGGAACCGGACATCTGTGTG	59.965	108
TRINITY_Contig1178	1	p3	(CGC)4	12	722	733	ATAGGGACCTCACCTGGGTG	60.327	GTGTCCATGACAGGTGGGAC	60.322	184
TRINITY_Contig1181	1	c	(CG)6ctcgctcgcctc(GCCGTG)4(GGCGT)5(GACCGA)4	97	180	276	CGGCTACTTTCCTTGGTCGT	60.038	CAAGGTTTGACGCGTTCCAG	60.041	122
TRINITY_Contig1184	1	p6	(GCCGGC)4	24	2937	2960	GTTGGTGTGAGCAAGCTGTG	59.97	GGCCCTATCCGGAATCGAAG	60.037	272
TRINITY_Contig1184	2	p2	(GA)7	14	3326	3339	GTTGGTGTGAGCAAGCTGTG	59.97	GGCCCTATCCGGAATCGAAG	60.037	272
TRINITY_Contig1185	1	p5	(GAGCC)3	15	72	86	CCGTATACGCATCCTGCAGT	59.968	AAACCTCCTGCTTCGCATCA	59.963	234
TRINITY_Contig1185	2	p3	(CCG)5	15	413	427	CCGTATACGCATCCTGCAGT	59.968	AAACCTCCTGCTTCGCATCA	59.963	234
TRINITY_Contig1188	1	p3	(GGC)4	12	659	670	GACGACAGCTTCCTCTACCG	59.901	GAGGAGGTGGAGGTCGTAGT	60.034	195
TRINITY_Contig1190	1	p3	(GCC)4	12	246	257	GGAGCGAGGTGAAGTAGAGC	59.898	TTCTCGTCCCCTTCCCTCAA	60.179	226
TRINITY_Contig1190	2	p3	(GGC)4	12	852	863	GGAGCGAGGTGAAGTAGAGC	59.898	TTCTCGTCCCCTTCCCTCAA	60.179	226
TRINITY_Contig1193	1	c	(CCG)4catcctgttcgtgcagtccagccgagcg(CTC)4accctcctctctctcttcccccctctccggtcaccca(CACC)3	101	241	341	GGGACCGTAGTTGCAGTGAA	59.966	GATGATCGGAGCAGGCGTTA	59.968	234
TRINITY_Contig1194	1	p3	(GCG)7	21	626	646	AATGACAAGCACCGTGTTGC	59.969	CATGTGCTCCATCTGCCTCA	60.107	276
TRINITY_Contig1194	2	p3	(CCG)4	12	770	781	AATGACAAGCACCGTGTTGC	59.969	CATGTGCTCCATCTGCCTCA	60.107	276
TRINITY_Contig1194	3	p3	(GGC)4	12	922	933	AATGACAAGCACCGTGTTGC	59.969	CATGTGCTCCATCTGCCTCA	60.107	276
TRINITY_Contig1195	1	p3	(GGA)5	15	974	988	CCATCGAGAGCCATCCTGAC	59.967	AAGATTGCCCTCTGGAAGGC	60.034	193
TRINITY_Contig1196	1	p3	(CGC)5	15	8	22	CACGAGCGGAGGAAGATCTC	59.97	CGCTATCTGCTCCCCTCATG	60.038	129
TRINITY_Contig1197	1	p3	(AGG)4	12	671	682	CGAGATGAGACCCGGAATGG	59.967	CCCCAAATCCTTCCCCTTCC	60.032	156
TRINITY_Contig1198	1	p3	(CGG)4	12	991	1002	GCAGGAACGTAAGCAGGAGT	60.037	TGTTGAACACCTTGCGCATG	59.969	182
TRINITY_Contig1199	1	p3	(CGA)4	12	732	743	CGTAGAGGAGCTTGCTCAGG	59.898	ATCCACCAGTGCGACGATTT	60.036	109
TRINITY_Contig1201	1	p3	(CGA)4	12	518	529	CCATCGTCGCTGACCCATTA	59.896	CGGCACAATCCAACCAACAG	60.039	115
TRINITY_Contig1202	1	p4	(AGCA)4	16	1878	1893	CTTGAGCAAGCAAGCAAGCA	59.968	TCACGCGACGAGAACAGAAA	59.97	168
TRINITY_Contig1202	2	c	(GCC)4(ATC)7	33	2089	2121	CTTGAGCAAGCAAGCAAGCA	59.968	TCACGCGACGAGAACAGAAA	59.97	168
TRINITY_Contig1202	3	c	(GCGGG)3gccggcgaaggag(CTC)4	40	2316	2355	CTTGAGCAAGCAAGCAAGCA	59.968	TCACGCGACGAGAACAGAAA	59.97	168
TRINITY_Contig1202	4	p5	(AGCTG)4	20	2672	2691	CTTGAGCAAGCAAGCAAGCA	59.968	TCACGCGACGAGAACAGAAA	59.97	168
TRINITY_Contig1203	1	p3	(CGG)4	12	433	444	GCAGGGTCTTCAGCTGACAT	60.036	GAAACTGGCCTCTCCACGAA	59.965	196
TRINITY_Contig1204	1	p3	(CCA)4	12	436	447	AAACAGAGCTAGCCGGTCAC	60.037	TCTGAACAACCTCGTCCACG	59.968	252
TRINITY_Contig1208	1	p3	(GGC)4	12	158	169	TCTGCACTCCGATGTCCCTA	60.034	GCTACCAGCATCAACGCATG	59.971	120
TRINITY_Contig1211	1	p3	(CGC)6	18	55	72	AAAAGGAAAAATGGCGGGGC	59.964	GCCACAAAACCCTTGTCGAC	59.97	279
TRINITY_Contig1218	1	p4	(GGAG)3	12	910	921	GGATCCACGAAACCACCCAT	60.034	AAGGAAGAAACAAACGGCGC	59.97	157
TRINITY_Contig1224	1	p5	(ATTTG)3	15	848	862	TTTTTCCTCCCTCGAGCACC	59.964	ATGCACTAGCTGAACGACCC	60.108	105
TRINITY_Contig1228	1	p4	(GGGA)3	12	861	872	GAGTTGAGGTTGTGGAGGGG	59.963	TTTCTTCCCCCGCATCTCAC	60.035	141
TRINITY_Contig1229	1	p3	(CGC)7	21	1700	1720	CTAATCGAGCAGAGCCTCGG	60.04	CGCCTCAAGAAATCAGCACG	59.904	219
TRINITY_Contig1230	1	p2	(GA)8	16	872	887	ATAGAACGAGTGCACGGCAA	60.038	GAGCGATGAGACCGGAAGAG	59.97	217
TRINITY_Contig1231	1	c	(GCCG)3gcgctgctgccgacttgccacgccgcgcacgcgcgcgcata(CGGC)4gg(CGGAT)3	86	681	766	ACTGTGTCTCCATGCTCACG	60.038	GTCAGACCGGAGCTAGCATC	59.969	105
TRINITY_Contig1232	1	p3	(CCG)5	15	982	996	CTATAGCTAGCCTCGCTCGC	59.902	TGATTATCTCGGACGCTCGC	60.04	123
TRINITY_Contig1234	1	p4	(GCGG)3	12	2773	2784	CTCCTCATGCCTAGACGCTG	59.968	CGGGCCAACTCCTTCTTCTT	59.963	141
TRINITY_Contig1236	1	p3	(GAG)5	15	80	94	GTCCACCGCCTCCTAATCAC	60.179	CGAACAGGAAGTCCACCTCC	60.037	139
TRINITY_Contig1236	2	p3	(CGA)5	15	657	671	GTCCACCGCCTCCTAATCAC	60.179	CGAACAGGAAGTCCACCTCC	60.037	139
TRINITY_Contig1236	3	p5	(GTACA)3	15	800	814	GTCCACCGCCTCCTAATCAC	60.179	CGAACAGGAAGTCCACCTCC	60.037	139
TRINITY_Contig1239	1	p3	(CCG)7	21	598	618	GCCTCCGTCGTGTTCTTGTA	60.039	TGTTCCAGGCGGACATGTAC	60.037	130
TRINITY_Contig1239	2	p3	(TCT)4	12	1034	1045	GCCTCCGTCGTGTTCTTGTA	60.039	TGTTCCAGGCGGACATGTAC	60.037	130
TRINITY_Contig1240	1	p3	(GAC)4	12	276	287	CTTGAGCGATCCTCCGAGAC	59.97	CACCGCTCCTTCTTCTTCGT	60.038	187
TRINITY_Contig1241	1	p3	(CAG)4	12	1248	1259	GCTTGATGAGTGGCACTCCT	60.036	TAGCTTGTGTTCCGTCCCAC	59.966	112
TRINITY_Contig1243	1	p5	(GGCCC)3	15	994	1008	GAACATGGCTCTCACCACCA	59.963	AGATGACAGCATGGGTGTGG	60.035	146
TRINITY_Contig1244	1	p4	(CCTC)3	12	33	44	ATAATCATCCGGCTGGGCAG	59.963	TCCGTTCACACTCTACACGC	60.04	101
TRINITY_Contig1246	1	p4	(CACC)3	12	322	333	TCGAAGAACAGCAGCAGGAG	60.038	TTTGAAGTTCTCTCGCCGCT	59.966	146
TRINITY_Contig1247	1	p3	(CGG)4	12	605	616	CCACATCTTCGTCGCGGATA	59.97	GATTCCATCTCCTCCCAGCG	59.965	212
TRINITY_Contig1248	1	p3	(GGC)4	12	751	762	CACATTCTCCTGCCACCTGT	59.963	AGTACTTGCACTGGCTGGTC	59.965	277
TRINITY_Contig1248	2	p4	(CGGC)3	12	1057	1068	CACATTCTCCTGCCACCTGT	59.963	AGTACTTGCACTGGCTGGTC	59.965	277
TRINITY_Contig1249	1	p3	(GCA)4	12	191	202	CAAACAACGTACACGCAGCA	59.973	GCGGAGAAGACGAGATCGAG	60.042	102
TRINITY_Contig1249	2	p3	(TCT)4	12	379	390	CAAACAACGTACACGCAGCA	59.973	GCGGAGAAGACGAGATCGAG	60.042	102

TRINITY_Contig1249	3	c	(CCG)4ctcgcccacgtacaccgcgaagtggccacgcggcacgtccgacggaagcccggacgtctcctccgcctcgtcgtactcctcctcgtacgacccgccg(TGC)5	124	761	884	CAAACAACGTACACGCAGCA	59.973	GCGGAGAAGACGAGATCGAG	60.042	102
TRINITY_Contig1250	1	p2	(AG)16	32	245	276	AACCTTCCATCGCCTTCCAG	60.035	CGCGGATCTTGGACTCTCTC	59.97	189
TRINITY_Contig1251	1	c*	(CCG)3(CC<G>ACGGA)(GACGGA)2	29	970	998	ACAGATTCAGCCTTCCAGCC	60.035	GCAAGTTGACCTGCCCAAAG	59.967	219
TRINITY_Contig1255	1	p6	(CGACGG)3	18	1025	1042	AAGGCAACAACAGTGGGGAA	60.033	GACTGAAGCTGCAGACGTCT	60.039	211
TRINITY_Contig1257	1	p6	(TCGCCG)3	18	539	556	CGATTGGGACGGGATGAGAG	59.967	CGCATACGCCCATTTCACTG	59.972	161
TRINITY_Contig1259	1	c	(CGG)5agcgaggagatgggagggtcgggtgccgagagtggcg(GGC)4	64	943	1006	GCACATTGCGCAAACCAAAC	60.042	AAGGAGGACGCCATGAAGTG	60.036	262
TRINITY_Contig1260	1	p4	(CGGC)3	12	388	399	GCTCGTGGTGAACCTTGAGA	59.966	CAAAATCCAGGACCGGGTCA	59.962	220
TRINITY_Contig1260	2	p4	(AAGA)3	12	834	845	GCTCGTGGTGAACCTTGAGA	59.966	CAAAATCCAGGACCGGGTCA	59.962	220
TRINITY_Contig1261	1	p2	(TA)6	12	329	340	GCACCAAGGAACAACAGCAG	59.969	GGAAGTTCGCGATGTGCATC	59.973	181
TRINITY_Contig1265	1	p3	(TCG)4	12	21	32	CCGTTTCCTTCTTCCCTCCC	60.035	CGGTCTGGGCAAAAGGAGAT	60.035	134
TRINITY_Contig1265	2	p3	(GCG)6	18	295	312	CCGTTTCCTTCTTCCCTCCC	60.035	CGGTCTGGGCAAAAGGAGAT	60.035	134
TRINITY_Contig1265	3	p2	(AT)15	30	826	855	CCGTTTCCTTCTTCCCTCCC	60.035	CGGTCTGGGCAAAAGGAGAT	60.035	134
TRINITY_Contig1266	1	c	(GCC)4ccgggcgcggcgggcgctggcacagctccgccggcgcgcgcgccgccaccgccaggaacgcggcgctgaacaccatccccccggtccgcc(CCG)4gttggtcgga(TGCT)3	137	557	693	CAGGCAGAAGAAGGCGAAGA	60.037	CAAGCAAGCATCCGACCAAC	60.11	227
TRINITY_Contig1271	1	p3	(GAG)4	12	809	820	ACCGCGAGTCCTTCTTCAAG	60.038	ATGAACTCCCGGCTGAAGTG	60.036	160
TRINITY_Contig1272	1	p4	(CCGG)4	16	173	188	AGAGAGCACCACGTTGACAG	59.967	GGATCAAGTGGAAGGTCGGG	60.108	244
TRINITY_Contig1272	2	c	(GCTCT)3(GGTCT)3ctcctctgctccgcggcgcagt(GGTG)3	64	857	920	AGAGAGCACCACGTTGACAG	59.967	GGATCAAGTGGAAGGTCGGG	60.108	244
TRINITY_Contig1273	1	p4	(GATC)3	12	48	59	GACATCTGGTAGTACCCGCG	59.97	GCTGCAGGACCTCAACATCT	60.036	270
TRINITY_Contig1273	2	p3	(GCG)4	12	942	953	GACATCTGGTAGTACCCGCG	59.97	GCTGCAGGACCTCAACATCT	60.036	270
TRINITY_Contig1273	3	p5	(CGCGC)3	15	1443	1457	GACATCTGGTAGTACCCGCG	59.97	GCTGCAGGACCTCAACATCT	60.036	270
TRINITY_Contig1273	4	p3	(CGC)4	12	3916	3927	GACATCTGGTAGTACCCGCG	59.97	GCTGCAGGACCTCAACATCT	60.036	270
TRINITY_Contig1274	1	p3	(TGG)6	18	1019	1036	CAGCCACGATAATTGCCAGC	59.97	GCAAAGAGCTGATTAGGCGC	59.971	272
TRINITY_Contig1278	1	c	(AGA)4(AGG)4	24	595	618	CTGCCACAACACACAGCTTC	59.97	ATAGCGTACGCACACTAGCC	59.97	128
TRINITY_Contig1285	1	p3	(CGG)5	15	256	270	ACGACCAGATGGCTCCTTTG	60.036	CCATGTACAGAGCCGCCTAG	59.967	239
TRINITY_Contig1285	2	c	(GCG)4gtgcacgacgacgcccaacgaccagatggctcctttgatcgtcgtcacgac(GAGCCG)3	81	662	742	ACGACCAGATGGCTCCTTTG	60.036	CCATGTACAGAGCCGCCTAG	59.967	239
TRINITY_Contig1287	1	p3	(GCC)4	12	138	149	GGTGCGCCCTTCTCTTAACT	60.036	TGCATCGCGATTAGGACAGT	59.54	119
TRINITY_Contig1289	1	p4	(GATC)3	12	872	883	CAATTGCCGTTGGTGACTGG	60.039	GGACAAGAACGTCGTCAGGT	59.968	162
TRINITY_Contig1294	1	p3	(GGC)5	15	980	994	CAGCATCCGAATTGCCATCG	60.041	GAAATGGAAGAACGTGCGGG	59.832	116
TRINITY_Contig1295	1	p4	(GCTA)3	12	77	88	GGGGCTACTTCTACGCGTAC	59.97	GACCTGGCACGTTGATGAGA	60.037	215
TRINITY_Contig1298	1	p3	(ACG)4	12	796	807	CATCATCCCCTTCCTCGAGC	59.965	CCTTGAGGAGCTCGACCTTC	59.825	221
TRINITY_Contig1303	1	p4	(GGCG)3	12	2429	2440	GATCACCCACACCAACGACT	59.965	GCAGCGCTTTGGTAGTATGC	59.972	145
TRINITY_Contig1305	1	p6	(GCCTCA)5	30	117	146	GACTGTCCAAGGACCAGTCG	60.038	TTCAGGAACTCGCAGTCCAC	59.966	187
TRINITY_Contig1305	2	p4	(CTAG)3	12	883	894	GACTGTCCAAGGACCAGTCG	60.038	TTCAGGAACTCGCAGTCCAC	59.966	187
TRINITY_Contig1306	1	p3	(GCC)4	12	2717	2728	GGATTCCCCTAGTAACGGCG	59.967	TGCCAGTTCTTGCATCTCGT	59.965	274
TRINITY_Contig1306	2	p3	(CTC)6	18	3004	3021	GGATTCCCCTAGTAACGGCG	59.967	TGCCAGTTCTTGCATCTCGT	59.965	274
TRINITY_Contig1307	1	p5	(CCTCC)3	15	2130	2144	TACTTTGGGCTGTCCGACAC	59.966	TGCGGAAGAGGAAACCTGTC	59.965	187
TRINITY_Contig1310	1	c	(AG)14caga(AGC)4atgcaaggctcaaaggaatcgtactcaccaccacaagggcacgactacttgtacccgcacgcgcagccagggctgtaccct(CCG)5gcgaacccgcaggggggc(TACCAG)3	176	203	378	CGGCCCTGGGTTCTTGTATT	60.034	TACTGGTACTGGTAGCCCCC	60.032	258
TRINITY_Contig1310	2	p4	(GATC)3	12	576	587	CGGCCCTGGGTTCTTGTATT	60.034	TACTGGTACTGGTAGCCCCC	60.032	258
TRINITY_Contig1311	1	p3	(GCA)4	12	722	733	GCATAGCACAGCAGAAGCAC	59.902	AGTCGTCAAGCCCAAGGATG	60.036	279
TRINITY_Contig1311	2	p3	(GCT)4	12	993	1004	GCATAGCACAGCAGAAGCAC	59.902	AGTCGTCAAGCCCAAGGATG	60.036	279
TRINITY_Contig1312	1	p4	(CACG)3	12	110	121	ACGAGAGAGAGGGTGTGTGA	59.889	TGGACCAGGACCTCTACCAG	59.959	266
TRINITY_Contig1312	2	p3	(CTC)4	12	495	506	ACGAGAGAGAGGGTGTGTGA	59.889	TGGACCAGGACCTCTACCAG	59.959	266
TRINITY_Contig1314	1	p3	(CCG)5	15	2590	2604	AGGATGGTGTAGGGCCTCAT	60.03	GGATGCAAATGCGGGTGATC	59.969	202
TRINITY_Contig1315	1	c	(GGC)8(GAC)5	39	2268	2306	GTGTTACAAGACGGGTCGGT	59.967	ACAAGAAAGCGGCGAGAGAA	59.966	214
TRINITY_Contig1315	2	p3	(GAC)4	12	2505	2516	GTGTTACAAGACGGGTCGGT	59.967	ACAAGAAAGCGGCGAGAGAA	59.966	214
TRINITY_Contig1315	3	p3	(GGA)6	18	2642	2659	GTGTTACAAGACGGGTCGGT	59.967	ACAAGAAAGCGGCGAGAGAA	59.966	214
TRINITY_Contig1317	1	p3	(CTG)6	18	614	631	CAATCTAACATGGCGAGCGC	60.042	GATCTGCCTGGACCTCAACC	60.107	114
TRINITY_Contig1317	2	p3	(GCT)4	12	838	849	CAATCTAACATGGCGAGCGC	60.042	GATCTGCCTGGACCTCAACC	60.107	114
TRINITY_Contig1319	1	p3	(GCC)4	12	481	492	GCACATTGCATCCTCTCCCT	60.107	TTGAGACCATTTCGGAGGCC	60.035	200
TRINITY_Contig1320	1	p3	(GAA)8	24	1385	1408	AGGTGATCTCGGTCAGAGCT	60.034	GATCCATGGCTGGTATGCGA	59.965	187
TRINITY_Contig1321	1	c	(CCG)5cattccccgcaggccgcagcaccagccatcaggcgcagccggcagccgtccgtcaccgccgcaccgtc(CCT)9	110	56	165	GGATCTCATTGACGCCGCTA	59.968	TGAAGAGCTGTACGGCCTTG	60.037	108
TRINITY_Contig1323	1	p3	(GTC)4	12	232	243	CACGTAGTTGCAGAGCAGGA	60.038	TCCATCCCTCCCTCGATCTC	59.887	161
TRINITY_Contig1323	2	p3	(GCG)7	21	900	920	CACGTAGTTGCAGAGCAGGA	60.038	TCCATCCCTCCCTCGATCTC	59.887	161
TRINITY_Contig1325	1	c	(TTA)4tgacatagtacaagaagaaatgatagtac(AAG)4	53	74	126	CGGGGATCTTCTTGTGCACT	60.036	ACAGCACTGCCAATACAGCT	59.963	224
TRINITY_Contig1325	2	c*	(GCT)5(TG)7tctatac(AT)17(ATGT)8*	100	1027	1126	CGGGGATCTTCTTGTGCACT	60.036	ACAGCACTGCCAATACAGCT	59.963	224
TRINITY_Contig1327	1	p4	(CCGG)3	12	564	575	GCGCTTTGAAAGATGCCCAA	60.038	CGACGGCGGCTTAGATTTTG	59.974	116
TRINITY_Contig1327	2	c	(CTT)4ggtggcggtggtggtgctgt(CGG)4	44	1468	1511	GCGCTTTGAAAGATGCCCAA	60.038	CGACGGCGGCTTAGATTTTG	59.974	116
TRINITY_Contig1328	1	c	(GATC)3gagcgagcgagagaagcgcggcggaagagacggggacgatggcggcttccaggctcgcggtcacggtgctggcggtgct(GGC)4	103	21	123	CTTCCTCGAGAAGCAGGACG	60.179	GCTCTCCGACGATGATCAGG	60.04	100
TRINITY_Contig1328	2	p6	(GCGCAG)4	24	426	449	CTTCCTCGAGAAGCAGGACG	60.179	GCTCTCCGACGATGATCAGG	60.04	100
TRINITY_Contig1328	3	p4	(GTCA)3	12	935	946	CTTCCTCGAGAAGCAGGACG	60.179	GCTCTCCGACGATGATCAGG	60.04	100
TRINITY_Contig1331	1	p3	(GGC)4	12	894	905	TCAGGTCCGGACAGTCTCAT	59.96	CATGCAAAGATGGCGTACGG	59.972	174
TRINITY_Contig1333	1	p3	(CTG)4	12	638	649	TGTCATACTCCTCCGGCAGA	60.034	GAGAACGCCTTCCTCCTCAC	60.109	100
TRINITY_Contig1333	2	p6	(GGCGAC)3	18	875	892	TGTCATACTCCTCCGGCAGA	60.034	GAGAACGCCTTCCTCCTCAC	60.109	100
TRINITY_Contig1335	1	p3	(GCC)4	12	215	226	GTGGTGGAGAATGGAGACGG	60.108	CAAGGAGGGAGAGGGATGGA	60.031	141
TRINITY_Contig1335	2	c	(CGA)4tgaccgggaagacgacgcggaggaac(ACG)4atgactgcc(GCG)4	71	433	503	GTGGTGGAGAATGGAGACGG	60.108	CAAGGAGGGAGAGGGATGGA	60.031	141
TRINITY_Contig1336	1	p3	(CGC)7	21	666	686	CGCTCATAGCCATTGCCAAC	59.97	GCTGCCTACATTGCCTCTCA	60.108	100
TRINITY_Contig1340	1	p4	(CTAG)3	12	567	578	CACGTCAGGATGTCAGTGCT	60.038	CTTGGTGGGTGCTACTGGAG	60.036	111
TRINITY_Contig1342	1	p4	(GGAG)3	12	3259	3270	CTCCGGAGCGTGAACAATCT	60.109	CGTCTTCAACCGGGTGTACA	59.968	202
TRINITY_Contig1343	1	c	(GCG)6cccggccccggcaccgcggctgt(GGA)4	53	640	692	CCTGCTCGATTTCTCCTCCC	59.894	GACCTCCTCCAGTGTGTTCG	60.038	277
TRINITY_Contig1343	2	p3	(CCT)4	12	917	928	CCTGCTCGATTTCTCCTCCC	59.894	GACCTCCTCCAGTGTGTTCG	60.038	277
TRINITY_Contig1344	1	c	(TCGA)5tggatcatcgtccgagacgtctaataacctttggtaattggtatcgatctcgacta(CTAG)3	88	552	639	GTGACATCCCGATCATCGCT	59.967	TCGGCGATCATCATCAGTGG	59.967	143
TRINITY_Contig1344	2	p3	(CGC)5	15	913	927	GTGACATCCCGATCATCGCT	59.967	TCGGCGATCATCATCAGTGG	59.967	143
TRINITY_Contig1344	3	p6	(GCGCCG)3	18	1047	1064	GTGACATCCCGATCATCGCT	59.967	TCGGCGATCATCATCAGTGG	59.967	143
TRINITY_Contig1345	1	c	(CAG)4aggattcctctcctccttcccctgcgcctgctcgtttcttctctagcgtttgttcccttgtccccttttccatccagtcctgctccatttgttcgtt(TCC)4	121	173	293	TTCCTCTCCTCCTTCCCCTG	59.957	TAAATCAGCAGGGGGAGGGA	59.954	137
TRINITY_Contig1347	1	p4	(CGGC)3	12	226	237	GATCGAGAGCATGGAGAGCC	60.038	CCAGACCATCTGCCTCTTCG	60.179	202
TRINITY_Contig1351	1	c	(CGC)4gagtggctcctgcttcggcgccggcgcgaccagataggcgccagcttggtgctgctccggccaccctgcggcctga(TGC)4	100	528	627	GGTGTGGCAGATCATGACCA	60.035	TTCGCCGACCTGTTCTACAC	60.039	200
TRINITY_Contig1352	1	p3	(CGT)4	12	170	181	ACCTCGTTCTCCTCCTCCTC	60.034	ATGTAGAGCAGGGAGGTGGT	59.957	262
TRINITY_Contig1352	2	p5	(TCCCC)3	15	296	310	ACCTCGTTCTCCTCCTCCTC	60.034	ATGTAGAGCAGGGAGGTGGT	59.957	262
TRINITY_Contig1352	3	c	(CTC)4gcggtgtccgtgcccgtcgccttcctctgggcgcacatggagccgctgctcctgctctgcggccaggacgcggcgat(CGC)4	101	923	1023	ACCTCGTTCTCCTCCTCCTC	60.034	ATGTAGAGCAGGGAGGTGGT	59.957	262
TRINITY_Contig1352	4	p3	(CTC)4	12	1247	1258	ACCTCGTTCTCCTCCTCCTC	60.034	ATGTAGAGCAGGGAGGTGGT	59.957	262
TRINITY_Contig1352	5	p3	(GAC)4	12	2009	2020	ACCTCGTTCTCCTCCTCCTC	60.034	ATGTAGAGCAGGGAGGTGGT	59.957	262

TRINITY_Contig1353	1	c	(TCGCG)3ccgt(CGCGC)3	34	623	656	ATCCTTGCCTCGGTGAACTG	60.036	GAAGAAGACGACGACGGTGA	59.763	143
TRINITY_Contig1353	2	p4	(GCCG)3	12	800	811	ATCCTTGCCTCGGTGAACTG	60.036	GAAGAAGACGACGACGGTGA	59.763	143
TRINITY_Contig1362	1	p2	(GA)7	14	97	110	CTCGAATCGAAGTTGCAGCG	59.976	GAACAATGGCCATCCGAACG	59.901	248
TRINITY_Contig1362	2	c	(CCGCG)3taacgcaggggcggccccgggcacgcagctcttcgcgaagatga(CGG)4	71	411	481	CTCGAATCGAAGTTGCAGCG	59.976	GAACAATGGCCATCCGAACG	59.901	248
TRINITY_Contig1363	1	p3	(GCG)6	18	170	187	AAGAAGACATGGCCGGTCAG	60.036	TTTTATGACCGGCTCCCCAC	60.035	109
TRINITY_Contig1363	2	p6	(GGGGCG)3	18	474	491	AAGAAGACATGGCCGGTCAG	60.036	TTTTATGACCGGCTCCCCAC	60.035	109
TRINITY_Contig1363	3	p6	(CCGCAG)3	18	686	703	AAGAAGACATGGCCGGTCAG	60.036	TTTTATGACCGGCTCCCCAC	60.035	109
TRINITY_Contig1366	1	p5	(CGGCG)3	15	562	576	GCGACATGCACAAGCTATGG	59.971	CTTCCCCAGTCGACATTGCT	60.036	257
TRINITY_Contig1367	1	p4	(CCTC)4	16	189	204	ATCACATGGCGATGGTGGAG	60.179	AGGAGGGCGAGGAAGATCTT	60.032	279
TRINITY_Contig1367	2	c	(GCC)4gtcgccgccgatgctggtgccccctccacagctccccccgccggcagcagtcgtgccaccaccgctggcatttccat(CGC)4	101	658	758	ATCACATGGCGATGGTGGAG	60.179	AGGAGGGCGAGGAAGATCTT	60.032	279
TRINITY_Contig1369	1	p3	(GGC)4	12	551	562	CGGTTGCGATAGACGATGGA	59.97	CGGTTGCGATAGACGATGGA	59.97	134
TRINITY_Contig1370	1	p2	(CT)7	14	71	84	AGCTACTACCGTGGAGCTCA	60.034	TGCCATGGTCATGAATGCCT	60.033	273
TRINITY_Contig1371	1	p3	(TCC)4	12	403	414	TCTGCTGCACGATCAACTGT	59.966	GGAGGATGCTACTGGCGATC	60.038	152
TRINITY_Contig1371	2	p3	(GCC)4	12	544	555	TCTGCTGCACGATCAACTGT	59.966	GGAGGATGCTACTGGCGATC	60.038	152
TRINITY_Contig1372	1	p3	(CCG)4	12	854	865	CGTCGGACGAGAGATCCTTG	59.972	AAGTGTCCAGATGCTCAGGC	60.036	225
TRINITY_Contig1373	1	p3	(CCG)6	18	1119	1136	ATTACGCAGAGCTCTACCGC	59.969	ACTCTGTCCTCTTGCACGTG	59.967	120
TRINITY_Contig1376	1	p3	(CGC)4	12	2109	2120	CAGGATGGCGAGCATGATCT	59.965	GAACCTGTACCGGTTCCTGG	60.037	136
TRINITY_Contig1376	2	p3	(GTG)4	12	3103	3114	CAGGATGGCGAGCATGATCT	59.965	GAACCTGTACCGGTTCCTGG	60.037	136
TRINITY_Contig1378	1	p3	(CGG)5	15	94	108	GGCTACTTCCGCGTCAACTA	59.828	CCGGAAGACGTAGAGGAAGC	59.899	129
TRINITY_Contig1380	1	p4	(GCCT)3	12	25	36	CCGGAGCAATCCTAGCTCAG	59.967	TGCAGGAAGGCTGAAGTCTG	59.964	148
TRINITY_Contig1380	2	p5	(GCGGC)3	15	217	231	CCGGAGCAATCCTAGCTCAG	59.967	TGCAGGAAGGCTGAAGTCTG	59.964	148
TRINITY_Contig1380	3	p3	(CAG)4	12	371	382	CCGGAGCAATCCTAGCTCAG	59.967	TGCAGGAAGGCTGAAGTCTG	59.964	148
TRINITY_Contig1381	1	p4	(GCCC)3	12	693	704	TCGTGGCTGTAACCTTGGAC	59.966	AGTCTGGCGATCGACTCTCT	60.107	222
TRINITY_Contig1381	2	p5	(GGCTC)3	15	849	863	TCGTGGCTGTAACCTTGGAC	59.966	AGTCTGGCGATCGACTCTCT	60.107	222
TRINITY_Contig1382	1	p3	(CCG)5	15	249	263	AGATGGCTGGATCACCCTCT	60.03	ACGGTACATCAACTGCTGGG	60.036	100
TRINITY_Contig1384	1	c	(GT)6aggttcttgcta(TC)6	36	736	771	AGGCCGAGGAAGAGGATCTT	60.032	AGGCCGAGGAAGAGGATCTT	60.032	195
TRINITY_Contig1385	1	c	(GAG)4(CGG)4	24	308	331	CGGATTCTCTCTCGGTCACG	59.972	TTGTATGTCCCGCCCAAGAC	60.036	107
TRINITY_Contig1388	1	p4	(CCTC)3	12	189	200	GGTACTGAAGGACGGCGTAG	59.9	ACGCAGTGTCGACTCTTCAG	60.04	126
TRINITY_Contig1388	2	p3	(CGG)4	12	446	457	GGTACTGAAGGACGGCGTAG	59.9	ACGCAGTGTCGACTCTTCAG	60.04	126
TRINITY_Contig1389	1	p5	(CGCCC)3	15	37	51	CGAAGAAGCAGGAGGGGTC	59.781	GAAGAAGTCGTACTCCGGGC	60.179	200
TRINITY_Contig1389	2	p3	(CGC)4	12	186	197	CGAAGAAGCAGGAGGGGTC	59.781	GAAGAAGTCGTACTCCGGGC	60.179	200
TRINITY_Contig1389	3	p3	(GCG)4	12	308	319	CGAAGAAGCAGGAGGGGTC	59.781	GAAGAAGTCGTACTCCGGGC	60.179	200
TRINITY_Contig1391	1	p4	(GAGG)3	12	86	97	CCTATGCGCCTTCTGGTCAT	59.893	AGATCAGGGGACACGACTGA	59.96	128
TRINITY_Contig1391	2	p3	(GCG)5	15	710	724	CCTATGCGCCTTCTGGTCAT	59.893	AGATCAGGGGACACGACTGA	59.96	128
TRINITY_Contig1394	1	p3	(CGG)4	12	273	284	AGCAGGAGACGAGCAAGTTC	60.038	AGGCCTTCCAATCCTTGTCG	60.035	279
TRINITY_Contig1398	1	p4	(AAGG)3	12	856	867	CGGACTCATCGTACTCCTGC	59.97	GCATCATGAAGTGCGACGTC	59.974	255
TRINITY_Contig1401	1	p4	(GCCT)3	12	799	810	ATAAGCCCTTCCTCCCACCT	59.954	CAGACTGAGGCGGAGATGAC	59.898	127
TRINITY_Contig1402	1	p3	(GCA)4	12	175	186	CACCTTGTTTGTGCGATGCA	59.969	AGGCTACCGTCGTACAGAGT	60.036	169
TRINITY_Contig1402	2	p3	(CGG)7	21	855	875	CACCTTGTTTGTGCGATGCA	59.969	AGGCTACCGTCGTACAGAGT	60.036	169
TRINITY_Contig1410	1	p4	(CAAA)4	16	106	121	GATGAAAGTGGTGGAGGGGG	60.034	CAAGAGCCTGGTTCAGAGCA	59.964	140
TRINITY_Contig1414	1	p3	(GGC)4	12	497	508	CTCATCCAACAGCCTGCTGA	60.036	GACGGTGAGCTCAAGGTCTC	60.109	102
TRINITY_Contig1416	1	p2	(GA)7	14	882	895	AAGGACGAGCTGAACGAGTG	60.039	TCCTCTCTCCTGCTGCTTCT	59.959	122
TRINITY_Contig1418	1	p3	(CGC)4	12	864	875	GTTCAGGGTGCTTGGCAAAG	59.967	ACAAGGAGGCCTACGAAAGC	60.036	222
TRINITY_Contig1419	1	p2	(CT)6	12	130	141	GCTTGGTTGGCTGACTGTTG	59.969	TTATCATCTGAGCCGTCCGC	59.968	100
TRINITY_Contig1419	2	p3	(CCA)4	12	3513	3524	GCTTGGTTGGCTGACTGTTG	59.969	TTATCATCTGAGCCGTCCGC	59.968	100
TRINITY_Contig1419	3	p4	(GGGA)3	12	5064	5075	GCTTGGTTGGCTGACTGTTG	59.969	TTATCATCTGAGCCGTCCGC	59.968	100
TRINITY_Contig1420	1	p5	(CCCTG)4	20	47	66	CAAGCAGCGCATCAGATTCC	59.971	CGCCGAGCCATATATCTCCC	59.897	122
TRINITY_Contig1432	1	p3	(CCG)5	15	369	383	TGCATTCAATTCGGCACACC	59.757	GGAGGAGTGGTTCGAAAGCA	59.965	129
TRINITY_Contig1435	1	p3	(GGA)4	12	689	700	TCAAGCGGCTGGGAAATCAT	60.034	CTTCCAGGTGATTGTGGGCT	59.961	159
TRINITY_Contig1436	1	p3	(GCC)6	18	1738	1755	CAACGGAAGACAGCCTAGCA	60.037	CACGAAGCCAAACCCCTTTG	59.968	107
TRINITY_Contig1437	1	p4	(TTCT)3	12	889	900	ACGGTCAAGCAGTTCGACAT	59.966	CACGTAGCCCTTGAAGTCGT	60.039	117
TRINITY_Contig1439	1	p3	(GAC)4	12	36	47	CAAGGGGCACAAGAACAAGC	59.967	TAGTCCCTGAGCCTCTGGTC	60.033	140
TRINITY_Contig1439	2	c	(CCATGT)3ccatg(CAC)5	38	476	513	CAAGGGGCACAAGAACAAGC	59.967	TAGTCCCTGAGCCTCTGGTC	60.033	140
TRINITY_Contig1440	1	p2	(CG)7	14	1	14	TCGAAAGTTTGAGGCGTCGA	59.969	AGGAACTGCGTGAAGAAGGG	59.965	253
TRINITY_Contig1442	1	p2	(TC)6	12	149	160	GCGACGGTACCAGGTTCTAG	59.9	AGCACGCAAATCAAGCACAG	60.04	155
TRINITY_Contig1443	1	p6	(AGGCAG)4	24	104	127	AAAAGGGAGGATGACAGCGG	60.035	AGATCTTGCTCCGAGACCCT	60.033	119
TRINITY_Contig1444	1	p3	(CCG)5	15	802	816	GAGGCGCTCCTTCATCAGAA	59.823	GGGAGATTAGGCGTCGCAAT	60.25	125
TRINITY_Contig1445	1	p3	(GCG)8	24	790	813	GGGTAATCGGCTTCACCCAA	60.035	CCGGACGGGGATCTACTACT	59.89	268
TRINITY_Contig1446	1	p4	(GGCC)4	16	1678	1693	CCCGTATCATCTGCTCCACC	59.966	TTCGACAAGGACCAGAACGG	59.967	106
TRINITY_Contig1447	1	p3	(CCG)7	21	119	139	CCTTGTCTCCAAGCTGCTGA	59.964	TAGTAGGGCTGGTCTGACCC	60.032	196
TRINITY_Contig1447	2	p3	(CCG)5	15	445	459	CCTTGTCTCCAAGCTGCTGA	59.964	TAGTAGGGCTGGTCTGACCC	60.032	196
TRINITY_Contig1448	1	p3	(CGC)4	12	219	230	TTGTTGTTGCTTCCATGCCG	59.968	CATCCCCGGGAAGATCAAGG	59.891	130
TRINITY_Contig1448	2	p2	(CG)6	12	644	655	TTGTTGTTGCTTCCATGCCG	59.968	CATCCCCGGGAAGATCAAGG	59.891	130
TRINITY_Contig1451	1	p3	(GCC)5	15	519	533	GTCCAGAGGTCGAACATCCG	60.179	GACTTCGGGTTTGGCGTCTA	60.038	200
TRINITY_Contig1457	1	p3	(GCA)4	12	209	220	CAACCGGACGAGGATTCTCC	60.179	TTTGCTGTTGCGAACTTGCA	59.828	204
TRINITY_Contig1461	1	p5	(CCCCT)3	15	147	161	AGAGGCTGGAGGAGGAGAAG	60.032	AGCACCCGAACCAGAAAGAG	59.965	164
TRINITY_Contig1464	1	p3	(GTC)4	12	780	791	AGCGTCATGGCGATGAAGAT	59.894	AGTGGTGTGGTCGTTCTTCC	59.894	123
TRINITY_Contig1466	1	p5	(CGATG)3	15	156	170	CGCATCGATCTCCGTCTGAA	59.971	CTGATCGATCCAGTCTGGCC	59.967	231
TRINITY_Contig1466	2	p4	(CCGG)3	12	306	317	CGCATCGATCTCCGTCTGAA	59.971	CTGATCGATCCAGTCTGGCC	59.967	231
TRINITY_Contig1467	1	p3	(GCC)4	12	39	50	TGACAAGAACCGGGACATCG	60.038	GGGTGGCCTACAAGAACCTC	60.035	152
TRINITY_Contig1468	1	p3	(AGG)4	12	2126	2137	AATGCTCGTGAGGGTTGAGG	60.036	GAACCACGCATACCTGGTGA	60.037	106
TRINITY_Contig1472	1	c	(CCG)4cca(CCG)4	27	129	155	GCAAGTCGGAGATCCTGGAG	59.896	TATTGCCGATCTGCATCCCC	59.963	145
TRINITY_Contig1474	1	c	(GCC)5ctgagcgcggcttggcgcagcccgaggccgccagcccgcggtgcggcacggattgccggccgttggcc(CGGCG)3	98	496	593	TCCATTCCCCTGTCTCGTCT	59.959	GCGATGAACAACAATCGCCA	59.832	220
TRINITY_Contig1475	1	p6	(GGCGGA)3	18	1635	1652	CTAGCACGTCTCATCCGCTT	59.899	AGTGGAGAAGCGGACATTGG	60.036	252
TRINITY_Contig1477	1	p3	(GCC)4	12	115	126	CGAGGAGTTCTTCGAGGTCG	59.903	AGCTATGGCCCTCTCAGTCA	60.032	271
TRINITY_Contig1481	1	p3	(CGG)4	12	512	523	CTCATCAGCCAGCTGTCCAA	60.036	CCCCCACATCATGAACAGCT	60.034	176
TRINITY_Contig1482	1	p3	(GCG)5	15	557	571	CCCAAAAACCAGATGCGCAA	59.967	TCCACCCCCATGGAGTAGAG	60.031	167
TRINITY_Contig1482	2	p4	(CGAC)3	12	686	697	CCCAAAAACCAGATGCGCAA	59.967	TCCACCCCCATGGAGTAGAG	60.031	167
TRINITY_Contig1482	3	p6	(GCGGCC)3	18	861	878	CCCAAAAACCAGATGCGCAA	59.967	TCCACCCCCATGGAGTAGAG	60.031	167
TRINITY_Contig1484	1	p5	(CATGC)3	15	954	968	AGGGCAAACATAGCTCCACC	60.034	AGTGCACATGAACGGATGGT	59.963	104
TRINITY_Contig1485	1	p3	(GAG)4	12	1720	1731	ATCAGGCTGGATCCCTTCCT	60.029	TGCAAGCACAATGTCTGCAC	59.969	117
TRINITY_Contig1485	2	c	(GGA)4gca(CGG)7	36	1974	2009	ATCAGGCTGGATCCCTTCCT	60.029	TGCAAGCACAATGTCTGCAC	59.969	117
TRINITY_Contig1486	1	p3	(GGC)5	15	551	565	AGGATCCTGCTGGAAAACCG	60.035	CCCCCACTAGTTGGTGCAAT	59.96	251
TRINITY_Contig1486	2	p6	(CCTGCC)3	18	682	699	AGGATCCTGCTGGAAAACCG	60.035	CCCCCACTAGTTGGTGCAAT	59.96	251
TRINITY_Contig1492	1	p6	(GGCTGT)4	24	1042	1065	TCGAGCGAGATCTCCATTGC	59.969	GTGGGTTGCATCGTTTTGCT	59.968	148
TRINITY_Contig1492	2	p3	(CCA)5	15	1363	1377	TCGAGCGAGATCTCCATTGC	59.969	GTGGGTTGCATCGTTTTGCT	59.968	148
TRINITY_Contig1493	1	c	(GGC)4tgggcgtccaa(CTC)4	35	724	758	CGCATGTACAGTAGGCCAGT	59.824	CCAACGGCTACATCGAGGAG	60.249	129
TRINITY_Contig1494	1	p4	(GGAG)3	12	811	822	GGGGCCCACATTAACGGTAA	60.034	GGATCCCCCTAGAATTGGCG	59.963	181
TRINITY_Contig1497	1	p3	(CGG)6	18	125	142	ACTAGTGTACAAACGCGCCA	59.968	TGATGAGTGCAGCTTGTGGT	59.891	107
TRINITY_Contig1502	1	p4	(GGAG)3	12	670	681	GGCTTGAGAAAAGGGTGGGA	59.888	CTCCCACCTCTCTCCTCCTC	60.106	168
TRINITY_Contig1503	1	p2	(AG)6	12	88	99	GGCACAGGGGAAATTCCAGA	59.96	AGGATTGGCAGTGTTCCTCG	60.036	276
TRINITY_Contig1503	2	p3	(GCA)4	12	446	457	GGCACAGGGGAAATTCCAGA	59.96	AGGATTGGCAGTGTTCCTCG	60.036	276
TRINITY_Contig1504	1	p3	(CGC)5	15	417	431	CCCGAGGTCGATAGTCTCCT	59.891	TTCTTTGCAGTACTCGCCGT	59.967	198
TRINITY_Contig1504	2	p3	(CCG)5	15	565	579	CCCGAGGTCGATAGTCTCCT	59.891	TTCTTTGCAGTACTCGCCGT	59.967	198
TRINITY_Contig1504	3	c	(TGC)6(TGG)4	30	742	771	CCCGAGGTCGATAGTCTCCT	59.891	TTCTTTGCAGTACTCGCCGT	59.967	198
TRINITY_Contig1505	1	p4	(CTCC)3	12	60	71	CGAATCGAAGTCAAGCGCAG	59.976	GGTCCTGCTCTACGACGATG	59.97	184
TRINITY_Contig1505	2	p3	(CCG)5	15	3701	3715	CGAATCGAAGTCAAGCGCAG	59.976	GGTCCTGCTCTACGACGATG	59.97	184
TRINITY_Contig1506	1	p3	(GTG)4	12	531	542	AGGGCATTTCGGAAAGGGAG	60.034	TTAGCCAGGTCTCCATCCCA	59.956	146
TRINITY_Contig1507	1	p3	(CCG)4	12	31	42	CTCGCCGTACAGGTCATCTC	59.97	TGATGCTGCGGTTGAAGGAT	60.035	174
TRINITY_Contig1508	1	c	(CCG)4ccccggccggagccgccggggagccgccgccggactcggaagcggatccagacgct(GCC)5	83	2222	2304	TGCAACAGGACGCATTTGTG	59.969	CGGATGATGCTACTCGTGCT	59.969	140
TRINITY_Contig1510	1	c	(ACG)4agaccgaca(CGG)4	33	384	416	CCTGCTCGTACACCGTTCTT	60.039	CTGCGTCACGTCGAAGTACT	60.11	146
TRINITY_Contig1510	2	p5	(CGGCG)3	15	613	627	CCTGCTCGTACACCGTTCTT	60.039	CTGCGTCACGTCGAAGTACT	60.11	146
TRINITY_Contig1512	1	p3	(GCC)4	12	631	642	AGAACTTGGGCGTCAGGATG	60.036	CACCAGGAGCACGGAGATTT	60.036	221
TRINITY_Contig1518	1	p3	(GTC)4	12	562	573	TGGACCGGAGCTTGAACTTC	59.965	ACTCGGTGATCATCGGCATC	59.967	147
TRINITY_Contig1518	2	p3	(GCG)4	12	1536	1547	TGGACCGGAGCTTGAACTTC	59.965	ACTCGGTGATCATCGGCATC	59.967	147
TRINITY_Contig1520	1	p3	(AGG)4	12	892	903	TCCAAGACTCTTGCTGCTCG	60.038	CTGCTTCTCCTCCTCCTCCT	60.032	265
TRINITY_Contig1531	1	p3	(ACC)4	12	464	475	AAGGAGCAGAATTCCCCAGC	60.034	ATTTAATTCGGGGGCGACGT	60.108	217
TRINITY_Contig1532	1	p4	(GGCG)3	12	128	139	GCTAGCTTCCGACTTGTCGA	59.829	CTAGCACGTCGCCTAAACCT	59.827	247

TRINITY_Contig1534	1	p3	(GCG)4	12	2336	2347	TGGCTGTCGCAAAAACCATG	59.968	AGATGAAGGCACCTGGTTCG	60.036	184
TRINITY_Contig1538	1	p3	(GGC)4	12	575	586	GGCGGCCGCAAATTAAAGAT	59.898	GAACCAATGACTCCTGGCCA	59.962	102
TRINITY_Contig1538	2	c	(CGCT)3cgtggactcgcagt(CGC)4	38	692	729	GGCGGCCGCAAATTAAAGAT	59.898	GAACCAATGACTCCTGGCCA	59.962	102
TRINITY_Contig1540	1	p5	(TTTTG)3	15	190	204	CTCCACCTATCTGCAGAGCG	59.968	AGGCCGTCGAGAAGAACAAG	60.038	113
TRINITY_Contig1540	2	p3	(GGC)4	12	505	516	CTCCACCTATCTGCAGAGCG	59.968	AGGCCGTCGAGAAGAACAAG	60.038	113
TRINITY_Contig1542	1	p2	(AT)9	18	88	105	GGAAGAAGCGGTTCAGTCCA	59.965	CATGCAGAAGAGGCAGGACA	60.036	209
TRINITY_Contig1542	2	c	(CGC)5gttcctggccgcggcctcgtccttgacgcaacggctgagcacgccgcacgccaccacgaaccgccgccggccgccgcaatccgct(GCC)4	112	653	764	GGAAGAAGCGGTTCAGTCCA	59.965	CATGCAGAAGAGGCAGGACA	60.036	209
TRINITY_Contig1544	1	p3	(GGC)5	15	90	104	CATGCTTCTCCTCTGCACGA	60.108	ACGAGTCCATCCACGTGAAC	60.039	161
TRINITY_Contig1546	1	p3	(TCC)4	12	1075	1086	AGCAAAACAAAGCCGCTCAG	59.969	TTTGCAAGCGAGCCTTTGAC	59.969	149
TRINITY_Contig1546	2	c	(CCG)4aatgcggcgggtgagga(GGC)5	44	1640	1683	AGCAAAACAAAGCCGCTCAG	59.969	TTTGCAAGCGAGCCTTTGAC	59.969	149
TRINITY_Contig1550	1	p3	(AGC)4	12	80	91	GGCTCGCTGCTGCATTATTC	60.041	GGCTCGCTGCTGCATTATTC	60.041	137
TRINITY_Contig1550	2	c	(TCGCCG)3ccgctgcgtgtcgatcagtgcagtcgctggctcgctgctgcattattcgtggatg(TATCA)3tcgggttattttcttggattatgtacgaacatgaaaacaatcacagaccccg(ATGAT)3acatccacgaataatgcagcagcgagccagcgactgcactgatcgacacgcagcgg(CGGCGA)3	229	630	858	GGCTCGCTGCTGCATTATTC	60.041	GGCTCGCTGCTGCATTATTC	60.041	137
TRINITY_Contig1550	3	p3	(GCT)4	12	1366	1377	GGCTCGCTGCTGCATTATTC	60.041	GGCTCGCTGCTGCATTATTC	60.041	137
TRINITY_Contig1551	1	p3	(CCG)4	12	309	320	GGTGGCCTCGCGATAACATA	59.967	ATCATGTGCGAGGCTAGAGC	59.967	227
TRINITY_Contig1551	2	p6	(CCGGCG)3	18	2627	2644	GGTGGCCTCGCGATAACATA	59.967	ATCATGTGCGAGGCTAGAGC	59.967	227
TRINITY_Contig1552	1	p3	(GCG)4	12	621	632	GCCCTTTTCGGCTTCCAAAG	60.038	CCACAAGTTCCCTGCCATCT	59.961	156
TRINITY_Contig1552	2	p3	(GCG)4	12	754	765	GCCCTTTTCGGCTTCCAAAG	60.038	CCACAAGTTCCCTGCCATCT	59.961	156
TRINITY_Contig1553	1	p4	(CGGC)3	12	908	919	AGCTGTTCGGCCATCAATGA	60.035	ACGAGGAACCACCAACGTAC	59.967	137
TRINITY_Contig1553	2	p3	(GCT)5	15	2506	2520	AGCTGTTCGGCCATCAATGA	60.035	ACGAGGAACCACCAACGTAC	59.967	137
TRINITY_Contig1553	3	p3	(GAC)5	15	2992	3006	AGCTGTTCGGCCATCAATGA	60.035	ACGAGGAACCACCAACGTAC	59.967	137
TRINITY_Contig1555	1	p3	(GGC)4	12	110	121	GTGCTGTGCTCTTGGGTTTG	59.969	ATGAGAAGACGACGACGACG	59.905	101
TRINITY_Contig1555	2	p3	(TCG)4	12	326	337	GTGCTGTGCTCTTGGGTTTG	59.969	ATGAGAAGACGACGACGACG	59.905	101
TRINITY_Contig1558	1	c	(CCG)4cccccggcgcggaggcga(AGC)4	42	113	154	TCGACTTCGACCTGCTCAAG	59.759	CAACCCTTTTTGCTCTCGGC	60.039	195
TRINITY_Contig1560	1	c	(GCC)4cccgccacgcacgcgctgctctctgct(GCC)4tgcgctactgcttttgctatctgcggccacaccgtccccaacgccaagcgctgttgctgctgcctccgcctccacccctgacgccgagc(GCT)4	152	295	446	TGGCACTTGAGACTCACGAC	59.968	AGGACCGATGTGAGCCAAAG	60.036	139
TRINITY_Contig1560	2	p3	(AGG)4	12	574	585	TGGCACTTGAGACTCACGAC	59.968	AGGACCGATGTGAGCCAAAG	60.036	139
TRINITY_Contig1561	1	c	(AGA)4gc(AAG)5	29	726	754	AGACCACCGTAGAGGAGACC	60.034	CTTGGTCTCCTCGACGATCG	59.972	218
TRINITY_Contig1564	1	p4	(GCTA)3	12	273	284	GCACATGGCATGAGCAGATG	59.969	CCTGGCAGCCTTCAATCTCA	60.035	105
TRINITY_Contig1565	1	p4	(GCAG)3	12	263	274	ATCGAGGAGTCGGAACTGGA	60.034	GTCGGTAGGCTGAAGCTTGT	60.037	110
TRINITY_Contig1565	2	p3	(ATG)8	24	2009	2032	ATCGAGGAGTCGGAACTGGA	60.034	GTCGGTAGGCTGAAGCTTGT	60.037	110
TRINITY_Contig1566	1	p3	(CGC)4	12	208	219	TGACCGTCACAGAACTTGGG	59.894	CTTCTGTGGTCACCGTGCTC	60.946	101
TRINITY_Contig1569	1	p3	(GCG)4	12	752	763	CCCTGGCACTTGACATCCAT	60.034	TGGTGCTCCAGAACGACATC	60.037	121
TRINITY_Contig1574	1	p3	(CAC)5	15	2790	2804	GGGGGAGAACAAGACGTGAG	60.037	CGCAACTACCGCTGAATTCG	59.975	154
TRINITY_Contig1578	1	p3	(CGG)6	18	243	260	CGAGGTCGATGTCCATCTCG	60.041	AATTTCCAACGGGCAACAGC	59.967	115
TRINITY_Contig1583	1	p3	(CAG)5	15	410	424	ACATCCCCGGACCATAGACA	60.032	CGAAACTCTCTGGTGGCAGT	59.966	195
TRINITY_Contig1585	1	p4	(GATG)5	20	353	372	TGAAGATCACCACCGCCTTC	60.036	TCCGGCACTTAAGCACACAA	60.179	166
TRINITY_Contig1585	2	p3	(CGG)4	12	826	837	TGAAGATCACCACCGCCTTC	60.036	TCCGGCACTTAAGCACACAA	60.179	166
TRINITY_Contig1588	1	p3	(GAG)6	18	671	688	TTTTCCAGCCCACCAAACCT	60.032	TCATGTCCCCTCGTGCTCTA	60.034	149
TRINITY_Contig1589	1	c	(ATCC)3cggcgcatcgaaacccagcacttctacccttagggcaaacattccaagtgctgagtccacccaattctcatgggcgaaagtcattagtagcctcc(GTCCTT)3	125	252	376	AGGACCGTGGTGTTGGAATC	59.964	GACAAGGACAAGGACGGAGG	60.037	139
TRINITY_Contig1596	1	p3	(GCG)6	18	84	101	TACCTGAGGCAAGGCATTGG	60.034	CTTGGTCCAGATCACGCCTT	60.036	208
TRINITY_Contig1598	1	p3	(GCC)5	15	360	374	AGCTCATCGTCACCGTCATC	59.899	GGTCCTCCTTGCACTCTGTC	60.037	104
TRINITY_Contig1599	1	c	(TCGA)3gatggcggagcccaggcgcacgcggccgtcgacgttccggtgcgcggcggcgacgtgcctggcgctgctggtc(GTG)4	97	81	177	TAGTGGGCGGCGAAACTTAG	60.109	GGTCCACGGAGATGTAGAGC	59.614	180
TRINITY_Contig1602	1	c	(CGC)4cacttcggcgcctctcgccactcctgtgccgcaagctgccgcggacgatgccagtacggccatgctgctgccaccaagtcctgcgccgagcaa(CGCTGC)3	123	196	318	GATTGCGTCGACATCAGCAC	59.974	CTCATCGTCCCTGATCACGG	59.969	129
TRINITY_Contig1604	1	p3	(GGC)4	12	707	718	CATCGGGAGCAGGTGAGTTT	60.036	TCCAGTGGTTCTTGACGTCG	59.968	240
TRINITY_Contig1604	2	p3	(CTT)5	15	931	945	CATCGGGAGCAGGTGAGTTT	60.036	TCCAGTGGTTCTTGACGTCG	59.968	240
TRINITY_Contig1606	1	p3	(CCG)4	12	676	687	CTTCGTCGTCTCCGTAGTCG	59.975	CAGTCCGTGGTTCACATCGA	60.039	182
TRINITY_Contig1609	1	p3	(GCA)4	12	51	62	ACGACGAAAAGCAGAGCAGA	59.968	AGAGAGGAGCAGAGGACGAG	60.107	168
TRINITY_Contig1609	2	p3	(GCG)4	12	241	252	ACGACGAAAAGCAGAGCAGA	59.968	AGAGAGGAGCAGAGGACGAG	60.107	168
TRINITY_Contig1609	3	p4	(TGTA)3	12	691	702	ACGACGAAAAGCAGAGCAGA	59.968	AGAGAGGAGCAGAGGACGAG	60.107	168
TRINITY_Contig1610	1	p3	(CGT)5	15	218	232	CGAAGGCGTAGTCGTAGTCC	59.973	CAGCTTCTACGACGTCTGCA	60.11	272
TRINITY_Contig1610	2	p3	(TGC)4	12	628	639	CGAAGGCGTAGTCGTAGTCC	59.973	CAGCTTCTACGACGTCTGCA	60.11	272
TRINITY_Contig1611	1	p3	(CCG)5	15	509	523	AGAACGGGAACACGATCGAC	60.109	TTTCCTGTTCGTCTGCGTGA	59.897	103
TRINITY_Contig1611	2	p3	(GCG)4	12	631	642	AGAACGGGAACACGATCGAC	60.109	TTTCCTGTTCGTCTGCGTGA	59.897	103
TRINITY_Contig1612	1	c	(TC)7cctccc(TCTT)3	32	699	730	TTACATGTGCTGTGCCGACT	59.965	TGAGTACATCTGCGCGCTAG	59.971	126
TRINITY_Contig1613	1	p2	(CA)6	12	14	25	GGAGAACCACCTGCACAAGA	59.891	TCGTCGTGTCCTTGGTCTTG	59.968	185
TRINITY_Contig1615	1	p6	(GCGCGG)3	18	567	584	GGCAATCCCTGACTCAACCA	59.962	CAAGACCTCCCGCTGTTCAA	60.25	207
TRINITY_Contig1616	1	p3	(CGG)4	12	711	722	GGCTGCTGACAACCAAAAGG	59.967	GCCGCAAAAAGGAAAGCAGA	59.968	200
TRINITY_Contig1616	2	p3	(GGC)5	15	904	918	GGCTGCTGACAACCAAAAGG	59.967	GCCGCAAAAAGGAAAGCAGA	59.968	200
TRINITY_Contig1619	1	p3	(GGA)4	12	512	523	CGAAAGGGAGGAGGAGGAGA	60.033	CGCCTGCTGATTATTGCCAC	59.97	199
TRINITY_Contig1620	1	p3	(CCG)4	12	182	193	CTTGATCCGGCGAACCTTCT	60.108	TCAAGAAGGCCGTGGAGTTC	59.965	263
TRINITY_Contig1622	1	p3	(GAG)5	15	696	710	CGAACTTGAAGCTCGGCATG	59.904	ACGTCTTCTGGCAGAGCTTC	60.038	279
TRINITY_Contig1623	1	c	(GCAG)3tttcagcgctcgccgct(CTCC)3	41	669	709	GCAACCGGTAGCTAGGGTTT	60.036	TGTGGGGGAAAAGGATCGTG	59.962	143
TRINITY_Contig1624	1	p4	(CGGC)3	12	128	139	CTTCTCTCGTTGCAGCTCCA	60.038	CCTCCGACGGTCAACATAGG	59.898	278
TRINITY_Contig1625	1	p3	(CGG)4	12	665	676	GGGTCAGTAATACCTCCGCG	59.968	TCAAGCTCGAAGACGTGGAC	60.039	219
TRINITY_Contig1626	1	p3	(GTG)5	15	219	233	TCGAGGTGGATGTGGTTGTG	59.965	GGGAGATTCCGCGAGATCTG	60.039	249
TRINITY_Contig1626	2	c*	(GGT)3(<GGT>CGA)(GGTCGA)3ggtggatgtggttgtggacttggaggacggcgcgaggaggtagcgggacgagacggccttggccttgggcttgggccgcgcgtcgccggcgtcggc(GGCGTT)4	153	386	538	TCGAGGTGGATGTGGTTGTG	59.965	GGGAGATTCCGCGAGATCTG	60.039	249
TRINITY_Contig1629	1	c	(CGG)4ccgggagcgggttccgaggatcgccgccggagttcgagggcggcagga(CGG)4	72	268	339	TCGTAGCTGCACAGTCGTAC	59.833	AGAGACGAGGCAACAAGTGG	59.966	252
TRINITY_Contig1630	1	p3	(ACC)4	12	178	189	GAAACGAGGGAAGGGAGCAA	59.964	AGGATTTCCTGCTGAAGCCC	60.034	229
TRINITY_Contig1630	2	p3	(CGC)6	18	599	616	GAAACGAGGGAAGGGAGCAA	59.964	AGGATTTCCTGCTGAAGCCC	60.034	229
TRINITY_Contig1635	1	p4	(CCTC)3	12	468	479	TGCTTGCCATTCACCTCGAT	60.035	GGAGGACTGAGTGTTGGTGG	59.965	152
TRINITY_Contig1637	1	p3	(GGC)5	15	383	397	CCCACCATTGAATCGACCCA	60.035	TTTCCTCCCTCGTGCAATCC	60.035	266
TRINITY_Contig1640	1	p6	(GCGCCG)3	18	631	648	CAAACCAAATGGCAGCTCCC	60.037	TCTTCCTGGTGTCTTTCGGC	59.965	181
TRINITY_Contig1640	2	p5	(AAAAG)4	20	1324	1343	CAAACCAAATGGCAGCTCCC	60.037	TCTTCCTGGTGTCTTTCGGC	59.965	181
TRINITY_Contig1641	1	c	(GCGA)3aaatcgcagcatcgttcgatcaccatattacgggtatggcgaggcatcagctgaaggtgtggccttcttcgtgggtgt(CGG)4	102	94	195	GCGAGGAGATCTACGTGGTG	59.97	GATGACGGCGATGGCTCTAA	59.968	184
TRINITY_Contig1656	1	p3	(GCA)4	12	105	116	GTAGCTGGAGTGACGCCTTT	60.037	CCCCTGGCACCGAACATAAT	60.107	132
TRINITY_Contig1658	1	c	(GAGC)4g(GGC)4	29	73	101	CCCGAAGAATCCGTATGGGG	59.965	ACGACCTTCTTGGGCTCATG	60.036	249
TRINITY_Contig1658	2	p4	(CGCC)3	12	376	387	CCCGAAGAATCCGTATGGGG	59.965	ACGACCTTCTTGGGCTCATG	60.036	249
TRINITY_Contig1659	1	p5	(GGAGG)3	15	2827	2841	TCAGGAAGCTGAGTGCCATG	60.036	GACCCAAGTTAACCCTCCGG	60.036	222
TRINITY_Contig1665	1	p4	(TATG)3	12	703	714	ACGTGCTGGACCTCATCAAG	60.037	GATGAGGCTGAGGTCGATGG	59.967	167
TRINITY_Contig1667	1	p4	(TCCC)3	12	277	288	CGTTCACCCTCTATTCCGCT	59.54	CTGCAGTACACGGATAGCGT	59.899	110
TRINITY_Contig1671	1	p2	(GA)6	12	40	51	CAAAGGCAGTCAAACCACGG	59.969	CAAAGGCAGTCAAACCACGG	59.969	185
TRINITY_Contig1671	2	p3	(CCG)4	12	530	541	CAAAGGCAGTCAAACCACGG	59.969	CAAAGGCAGTCAAACCACGG	59.969	185
TRINITY_Contig1672	1	p6	(CGCCGA)3	18	318	335	ACCAGGGACTACGACGAGAA	59.963	ACGAGTCTTGCATGAGCCAA	59.965	210
TRINITY_Contig1674	1	p3	(GGC)5	15	420	434	TGTATTCCTTGCCCGGATCG	59.894	CGTCTAATTTGCTGCCGTCG	59.975	139

TRINITY_Contig1679	1	c	(AC)7ggtcaacattgtacaccaccgtcgtcctccaccgtccacccccacacgatccgcaggctcgaatagcaaacggc(AGCT)3	100	94	193	GCGGCCCCATTGATTTGATC	59.967	GCTGCCGTTTGCTATTCGAG	59.973	150
TRINITY_Contig1679	2	p3	(GCC)4	12	387	398	GCGGCCCCATTGATTTGATC	59.967	GCTGCCGTTTGCTATTCGAG	59.973	150
TRINITY_Contig1680	1	p3	(GCC)4	12	425	436	GACCAAGGAAAAGGCGAGGA	59.964	GGTTGTGGTGCAAAGCTGAG	59.969	117
TRINITY_Contig1682	1	c	(GCT)4caacgagccacatgctagtcgactagtcttagtacctagct(AG)10	73	53	125	GGACCAAGTTCACCGAGGAG	60.037	GTGGTTGTGCATCCAGACCT	60.251	173
TRINITY_Contig1682	2	p3	(CGG)6	18	403	420	GGACCAAGTTCACCGAGGAG	60.037	GTGGTTGTGCATCCAGACCT	60.251	173
TRINITY_Contig1682	3	p3	(GGC)6	18	791	808	GGACCAAGTTCACCGAGGAG	60.037	GTGGTTGTGCATCCAGACCT	60.251	173
TRINITY_Contig1686	1	p3	(GCG)5	15	457	471	GTGGTGGTCCATCTTGAGCA	59.963	TGCCTCTTGTCCAACGACTC	59.966	192
TRINITY_Contig1690	1	p4	(CGAT)3	12	1366	1377	ATCGATTCGATTCCCGGTGG	59.966	GGGCACGACTCGTTGTAGAA	60.039	192
TRINITY_Contig1692	1	p6	(GTCGGC)3	18	271	288	AGGCACCCCAACAGTGAAAA	60.033	GTTGGAGCCTTGCACACTTG	59.969	244
TRINITY_Contig1692	2	p6	(CTTCCT)3	18	3289	3306	AGGCACCCCAACAGTGAAAA	60.033	GTTGGAGCCTTGCACACTTG	59.969	244
TRINITY_Contig1692	3	p3	(GGC)5	15	3472	3486	AGGCACCCCAACAGTGAAAA	60.033	GTTGGAGCCTTGCACACTTG	59.969	244
TRINITY_Contig1693	1	c	(GCG)6gccgccgagccgcgctaccacctcaccgcggcgtcccgcctcctggtt(GAC)4	78	340	417	CATGTCTCCACTCTACGCCG	60.249	CACGGATTTGAGGTAGCCGA	59.825	107
TRINITY_Contig1694	1	p2	(CG)7	14	426	439	ATTTTCGTCTGGGGCTCGAG	60.108	TTTGCCCCTTGCCAAACATG	59.892	220
TRINITY_Contig1696	1	p2	(CA)7	14	1216	1229	TTTGGTCATGGAGTACGCCC	60.036	GGTTGGGGGAATAATGGCCA	60.032	245
TRINITY_Contig1700	1	p3	(ATG)4	12	453	464	TGAACAGGGACGAAAGGACG	59.967	TACTCGGACCATCCTCTGCA	60.034	249
TRINITY_Contig1701	1	p4	(ATAA)3	12	14	25	TTGAAGCATTCGTGAGGCCT	59.963	GAGGAGAACTGGGCTTCCAC	60.036	171
TRINITY_Contig1701	2	p3	(CGC)9	27	320	346	TTGAAGCATTCGTGAGGCCT	59.963	GAGGAGAACTGGGCTTCCAC	60.036	171
TRINITY_Contig1702	1	p4	(GCTC)3	12	694	705	TGCCTGAGCAGATGTTTCCA	59.599	GTGACGGCGATGACCTCTAG	59.97	185
TRINITY_Contig1706	1	p5	(CCCAA)3	15	118	132	GCGGAGTGTTTTGTTGGCAT	59.968	ATTGGCATGGCATGGAAGGA	60.032	126
TRINITY_Contig1714	1	p3	(GCC)4	12	405	416	TGTGCTTTGCCAAGGATTGC	59.966	GCGGCTGATCCTTTGTGTTG	60.11	226
TRINITY_Contig1715	1	c	(GC)9tcgcgggggaggtgggggcgtggaggcgcccgacggtg(GCA)4gcgacgcggaggcgggtgggctggtgg(GGT)6	113	8377	8489	TGACTTGGGTGTTCGTACGG	59.968	GTGCTGCTCCTTGTTTGGTG	59.969	109
TRINITY_Contig1719	1	p3	(CAG)4	12	160	171	TTGCCCCTGAACTACACGAC	59.966	GCCTACGGCTACGGATGATC	60.109	115
TRINITY_Contig1719	2	p3	(CGG)4	12	459	470	TTGCCCCTGAACTACACGAC	59.966	GCCTACGGCTACGGATGATC	60.109	115
TRINITY_Contig1719	3	p4	(GCCT)3	12	662	673	TTGCCCCTGAACTACACGAC	59.966	GCCTACGGCTACGGATGATC	60.109	115
TRINITY_Contig1721	1	p3	(CGC)5	15	106	120	TCGTGAACTCTCCGTCTCCT	59.964	AGGGCAGAGAGAGCTCATGA	60.032	228
TRINITY_Contig1723	1	p4	(AAAG)3	12	5	16	TAACCATCAGCAACCGCAGT	59.964	GGTGTCCGTGGATCCATGTT	60.035	261
TRINITY_Contig1724	1	c	(CGT)4ccccgccgcgacgaaggt(GGC)5	45	166	210	GGACCCCATCCTCAACAAGG	60.034	TCGCGAGCCTTTCACTCTTT	59.966	153
TRINITY_Contig1725	1	p2	(AG)10	20	41	60	TTGCCCGTGACGATTGAAGA	59.966	ATGAGCGTTCTGTCATGGCA	60.036	245
TRINITY_Contig1728	1	p3	(TCA)4	12	1333	1344	CACTTCATTGCGTTTGGCCA	59.968	GGGCCTACAGTACCCTCAGA	60.032	183
TRINITY_Contig1728	2	c	(GGC)5gggatgagcagcggcggatctagggtttggggcgccggggagcgcgaatccgcgggcgggcccgcgcgaatccg(GGC)4cgaattgggcggccggggagggcggggcggatcgggagggcaggcgagcggatcgaattgggatcgcgaggcgggggaggcaggaagggggg(CGCA)3	205	2865	3069	CACTTCATTGCGTTTGGCCA	59.968	GGGCCTACAGTACCCTCAGA	60.032	183
TRINITY_Contig1731	1	p4	(GCTA)3	12	98	109	GTGAAAATGCTGCTGCTGCT	60.039	GTGCGGATCATGACTAGCCA	59.895	203
TRINITY_Contig1731	2	p3	(TGC)4	12	453	464	GTGAAAATGCTGCTGCTGCT	60.039	GTGCGGATCATGACTAGCCA	59.895	203
TRINITY_Contig1741	1	p3	(CGG)6	18	161	178	AACGTCGGCATGAACCAGAT	60.036	AGGAAGGTAGCAGAGGGAGG	60.032	260
TRINITY_Contig1744	1	p3	(GAC)4	12	526	537	TGGAAACCGATCTTCAGGCC	60.035	AGCAGGCAGGTTCAGATGAC	60.036	119
TRINITY_Contig1745	1	p2	(TC)6	12	34	45	CGAGCTCTCCTCTATCCCGA	59.964	GGGCTGGACAAGGAGAGTTC	60.036	137
TRINITY_Contig1750	1	p6	(GAGCGC)3	18	529	546	GCACATCTTGGGTTCCCTCA	59.962	GCCCAACGACTTCCAGAAGA	59.965	211
TRINITY_Contig1751	1	c	(CGT)4catggagaggcaaat(CGG)5	42	98	139	GCTCAGCCTAGCTCTTCTCG	59.969	TGCACGAGGTCCTTGAATCC	60.036	175
TRINITY_Contig1760	1	p5	(TCGGA)3	15	348	362	GCAGTGTACAGCGCACAAAA	59.971	CAGCAAGTTCTGGAGCAGGA	59.964	202
TRINITY_Contig1760	2	p4	(TGCG)3	12	1666	1677	GCAGTGTACAGCGCACAAAA	59.971	CAGCAAGTTCTGGAGCAGGA	59.964	202
TRINITY_Contig1761	1	p3	(CAG)6	18	218	235	ACTGCCAATGTGCCATAGCT	60.034	TGTGTCTTGCTCTGCTGGAG	59.966	277
TRINITY_Contig1761	2	p2	(TC)6	12	618	629	ACTGCCAATGTGCCATAGCT	60.034	TGTGTCTTGCTCTGCTGGAG	59.966	277
TRINITY_Contig1764	1	p3	(CGC)4	12	148	159	TTCCAGAAGACGTTGGAGCC	59.965	CTCGAGAAGAGCAAGGCCAA	60.037	233
TRINITY_Contig1766	1	c	(CGG)5gcaccgagctttgcggcatggcgatgcggtt(GGC)4	58	94	151	GGAGAAGAAGATCGAGCGCA	59.899	TGACACCACGCAATTCGAGA	59.967	234
TRINITY_Contig1767	1	p3	(CAA)4	12	1664	1675	CACAAGGTCTGAGCGGAGTT	59.966	CCAGACATGCTCCATGGTGT	60.035	106
TRINITY_Contig1767	2	c	(GCT)6tccgcgcctg(CGC)4ggcggccgacgaggaggacgtcgcgggccccgccgccccgccgccggacgcggcg(GCC)4	107	2264	2370	CACAAGGTCTGAGCGGAGTT	59.966	CCAGACATGCTCCATGGTGT	60.035	106
TRINITY_Contig1771	1	p3	(GGC)4	12	540	551	CTCTCCCTCAGCTGACCTCT	60.033	GGCTGTAGCTGGTGTCACTT	59.965	246
TRINITY_Contig1771	2	p5	(GCGCG)3	15	2627	2641	CTCTCCCTCAGCTGACCTCT	60.033	GGCTGTAGCTGGTGTCACTT	59.965	246
TRINITY_Contig1771	3	p3	(GCC)4	12	3881	3892	CTCTCCCTCAGCTGACCTCT	60.033	GGCTGTAGCTGGTGTCACTT	59.965	246
TRINITY_Contig1773	1	p2	(CA)6	12	1043	1054	GAGCAAGTGGACGGTCAAGA	59.966	TTGGCCGTCGATTCTCCAAA	59.964	255
TRINITY_Contig1774	1	c	(GGTC)3gcct(CGC)6	34	1755	1788	AAACAGCCCCCAAACACAGA	60.033	TTGGGCCAGTCACCAGTTTT	60.033	179
TRINITY_Contig1777	1	p4	(CTTC)3	12	139	150	TGACCGTCGTGCTAATCTCG	59.902	CCCCATGACGGATCAACCAA	60.035	268
TRINITY_Contig1778	1	p3	(TCC)6	18	458	475	CACCCCTTTCGTCTCCCTTC	60.036	CGGTAATCGGACGGTAGTGG	59.97	139
TRINITY_Contig1782	1	p2	(CG)6	12	110	121	GCTGACGTGGATCTGGTGAA	60.037	GAAGAGCGTTTCAGTCGTGC	59.838	262
TRINITY_Contig1782	2	p4	(TCCG)3	12	314	325	GCTGACGTGGATCTGGTGAA	60.037	GAAGAGCGTTTCAGTCGTGC	59.838	262
TRINITY_Contig1784	1	p2	(AT)29	58	620	677	TTGTGGGGCACAGGTTGAAT	60.106	GGAAGCATCGCAGGGGAATA	59.891	154
TRINITY_Contig1787	1	p5	(GCGCG)3	15	9	23	CGCCGTCTCTCTCTCTCTCT	60.179	CTATTCCCAGCGGAGTAGCG	60.039	280
TRINITY_Contig1787	2	p2	(TC)12	24	355	378	CGCCGTCTCTCTCTCTCTCT	60.179	CTATTCCCAGCGGAGTAGCG	60.039	280
TRINITY_Contig1788	1	p3	(GGA)5	15	245	259	ATAGCAACACCACCAGAGCC	60.035	GCTCCACCTACTCCCAAACC	60.035	169
TRINITY_Contig1789	1	p4	(ACCA)3	12	381	392	GGCATGTCTGTACACCGCTA	59.825	CAGTTTGTCACACATGGCCG	60.04	172
TRINITY_Contig1796	1	p2	(AT)6	12	1442	1453	GGCGCCTAGATGTTATGCCT	59.965	GACGATGCTGCAAGATGCTG	59.972	156
TRINITY_Contig1799	1	p3	(CGG)4	12	483	494	GCGCCGTTTCTGAAAAGGTT	59.97	ATGGTTCAGGCACAGGAAGG	59.961	145
TRINITY_Contig1799	2	c	(CGG)4ggtgaaggaggccgc(CGG)4	39	601	639	GCGCCGTTTCTGAAAAGGTT	59.97	ATGGTTCAGGCACAGGAAGG	59.961	145
TRINITY_Contig1802	1	c	(GCC)4gcgacgcgcgtccgccgcctctgctccttctgccacgcaaccatgcgctcatcccactccctgtccatgcggcacctcgcagcagccgctctgcgct(CCG)5	124	217	340	TCATCCCACTCCCTGTCCAT	59.955	CCCGAGAAATGGAAGGGGAC	60.107	259
TRINITY_Contig1804	1	p3	(GCG)4	12	423	434	ATCGGTTGCAGAGCACAAGA	59.965	GAACGCAGAGTGATCCACCA	60.037	109
TRINITY_Contig1806	1	p2	(AC)6	12	150	161	CAGGCCGTCGAAGAACTTCT	60.038	CTTACCGACACCGCCTTCTT	60.038	229
TRINITY_Contig1808	1	p3	(GGC)6	18	575	592	GGACACACACACAAGCACAC	59.901	CGTGTACAACATCGACCCCA	60.038	269
TRINITY_Contig1809	1	p4	(CCCT)3	12	243	254	GAGCTGCACCAGAAGTTCCT	59.964	CCGATCGTCTCTCCAACTCG	59.972	152
TRINITY_Contig1811	1	p5	(AAAGC)3	15	136	150	CAATGCACGCGTCGTAGAAG	59.976	TCTGCTCTTTGCGTGCCTAA	59.965	172
TRINITY_Contig1811	2	p3	(CCG)5	15	1263	1277	CAATGCACGCGTCGTAGAAG	59.976	TCTGCTCTTTGCGTGCCTAA	59.965	172
TRINITY_Contig1813	1	p3	(GGC)4	12	205	216	TGCCTCTACTCCTGCTGCTA	60.033	CGACATCCCCGGATTCATGT	59.893	251
TRINITY_Contig1813	2	p5	(CCGCC)3	15	515	529	TGCCTCTACTCCTGCTGCTA	60.033	CGACATCCCCGGATTCATGT	59.893	251
TRINITY_Contig1814	1	p2	(CG)6	12	281	292	CGCACACACCTGACAACTTG	59.972	GAGTGATGATAGGGAGCGGC	60.038	205
TRINITY_Contig1814	2	p4	(GCTA)3	12	582	593	CGCACACACCTGACAACTTG	59.972	GAGTGATGATAGGGAGCGGC	60.038	205
TRINITY_Contig1822	1	p6	(GCGCCG)3	18	639	656	CTCCGGTACAGGAAGCGATC	59.969	CAATAATGGCGAGCGGATGC	60.11	153
TRINITY_Contig1823	1	c	(CGC)4cggaacgggggctctggtcaagctcgtgctgctctgagggag(CGC)4	66	483	548	TCTTCACCAAGATGACGGCC	60.036	GATCCGTCCTCACGTAGCTG	59.97	204
TRINITY_Contig1829	1	p5	(ACATC)3	15	755	769	TTCCTCGACCGTCTAGCAGA	60.036	CGCAGTCATCAATCTGTGCG	59.974	217
TRINITY_Contig1829	2	p3	(GCC)4	12	1925	1936	TTCCTCGACCGTCTAGCAGA	60.036	CGCAGTCATCAATCTGTGCG	59.974	217
TRINITY_Contig1830	1	p3	(CGG)4	12	620	631	TTGCACTTTTTCGGCGACAG	59.971	CTACCAGCGATGACGACTCC	59.97	206
TRINITY_Contig1831	1	p3	(GGC)5	15	599	613	AGGCATGCGATCTCCATCAG	59.965	AAAGCGGTCGCAAACTGATG	59.762	138
TRINITY_Contig1832	1	c	(TCCC)3ggtcccgggccgatcgagccgatgggggtggccgtgg(CGCTGC)3	67	1786	1852	TTTACACCAAAAAGGCCGCG	59.969	CGAGAGCTCAAGGTTGGTGT	59.966	101
TRINITY_Contig1836	1	p4	(GCTG)3	12	470	481	TCTCCACCCTTTCGTGAAGC	59.965	AAGGAGACTGAAAGCCAGCC	59.963	280
TRINITY_Contig1837	1	p3	(CGG)5	15	1444	1458	CGAGTCCTTATCCTGTCGCC	59.969	TGACACGGTCAATGTCCCTG	59.965	259
TRINITY_Contig1840	1	p3	(CAC)4	12	2261	2272	CGACACCATTTGGATCCCCA	60.035	GATGGCGATGAAGCTGCATG	60.04	268
TRINITY_Contig1841	1	p4	(CGGC)3	12	158	169	TCATCTTCTGCTTCTCCGCC	59.823	GCAGCTGGGCTACTAGGATG	59.965	213
TRINITY_Contig1843	1	c	(TCCCC)3ggagaccgggcaggacggcaaggtaagcggcgaacgccgggccggcacgagctctcgcc(CCA)4	86	34	119	AGGTCTATGTAGGGCGTGGT	60.032	ATCTCAGGGACCTTTGCAGC	60.035	159
TRINITY_Contig1844	1	p3	(CCG)4	12	364	375	TGATTTACTGCGCCGCCTTA	60.108	GAGCTCCAGGTTTCCCTTCC	60.035	205
TRINITY_Contig1846	1	p4	(AGAA)3	12	44	55	GCTCCCCAGTTCTCGACTTC	60.109	GCCATCCTCTGCCACTTCTT	60.035	110
TRINITY_Contig1848	1	p4	(GCCG)3	12	149	160	AGAGCGGCAGAAGCTTTTCT	59.964	ACGGCCACCATGTAGTTCTG	60.036	138
TRINITY_Contig1850	1	p3	(TCA)4	12	1259	1270	TAGTCATCAGCCGCATCGTC	59.969	CAGGATGATGGGCGGGATAC	60.036	127
TRINITY_Contig1854	1	p3	(GCC)4	12	207	218	GACGCTTAGTAGTCCGAGCC	59.971	CATCGACCTGATCTCGTCGG	60.041	177
TRINITY_Contig1862	1	p3	(TGC)4	12	1571	1582	ACAGACTCCACTGCAACTCG	59.967	TCTCCGTCTTCCACATTGCC	60.036	221
TRINITY_Contig1862	2	p3	(CGC)6	18	3245	3262	ACAGACTCCACTGCAACTCG	59.967	TCTCCGTCTTCCACATTGCC	60.036	221

TRINITY_Contig1867	1	p3	(CCG)4	12	1145	1156	AATGGCAGACTGAGACCGTG	60.037	ATCTCTCCATCCTTGGCCCT	60.029	150
TRINITY_Contig1869	1	p3	(GGC)4	12	354	365	TTACTCGCGAAGATCCTCGC	59.971	CACTGCTCGACGGATCTCTC	59.971	193
TRINITY_Contig1869	2	p2	(GA)11	22	575	596	TTACTCGCGAAGATCCTCGC	59.971	CACTGCTCGACGGATCTCTC	59.971	193
TRINITY_Contig1870	1	p2	(TA)8	16	565	580	GTCCTTCTACGACTGCAGCA	59.756	CTCGTCGGTGAAGGTGTTGA	59.968	190
TRINITY_Contig1871	1	c	(CCG)4ttctgatccgccttgtccacgtccatgctcttgagcttccgcgacgcctcctcgtcctcccgcccgccggcgcgcatt(CTCG)3	102	1179	1280	AGCAGCATGGAATGGAAGCT	60.033	GGTTAGTGCTGACGGGTTCA	59.966	159
TRINITY_Contig1875	1	p3	(TCG)4	12	822	833	AGACGACTCTGCTGTGGTTG	59.967	AGTCTGCTGCCACTAAGCTG	60.037	221
TRINITY_Contig1875	2	p3	(TGC)4	12	996	1007	AGACGACTCTGCTGTGGTTG	59.967	AGTCTGCTGCCACTAAGCTG	60.037	221
TRINITY_Contig1876	1	p6	(CGCTTG)3	18	503	520	ACTAAAGCCCATCACGTGCA	59.964	CCTCATGCTCCACCTCGATC	59.967	114
TRINITY_Contig1878	1	p3	(GGC)4	12	568	579	CGGATTGTGCTCCTGACAGT	60.037	AAGAAGGCTGGAATCGTCGG	60.108	236
TRINITY_Contig1881	1	p4	(GTCA)7	28	409	436	GAGTTCGCACAGTTCGCATC	59.906	ATCTTCTGGGCAAGTGACGG	60.036	136
TRINITY_Contig1881	2	p2	(TG)7	14	563	576	GAGTTCGCACAGTTCGCATC	59.906	ATCTTCTGGGCAAGTGACGG	60.036	136
TRINITY_Contig1883	1	p6	(TTGCTG)4	24	57	80	TTGCTGTTGCTGTTGCTGTC	59.899	GCAACAGGATGGGGAACGTA	60.036	181
TRINITY_Contig1886	1	c	(CGG)4ttgcaggcagcagcggactgtgtcccgaccccctggtgcggccc(TCCCT)3	71	2752	2822	CTCTTCGTACTCGACCTCGC	59.973	AGGAAGCAGAAGCAGCTCTG	60.036	132
TRINITY_Contig1888	1	p3	(CGC)5	15	2157	2171	CATGGCCTCTCCTTTGCTGA	60.035	CCCTTGGCAATTCGAGTCCT	60.035	146
TRINITY_Contig1890	1	p3	(CGG)5	15	263	277	CTCATCATCCCCACCACCAC	60.107	CAGGGTGTTCGACGAAATGC	59.834	117
TRINITY_Contig1892	1	p6	(CCCGCG)3	18	120	137	TGACCGTGAACCTGTCCATG	59.965	ACGTAGGTAGCGTGACGTTG	60.11	102
TRINITY_Contig1896	1	p3	(CGG)5	15	1138	1152	AGTGGTAGAAGGGCGTGTTG	59.965	CTACACGCAGCTCTACCTCG	59.972	170
TRINITY_Contig1897	1	p3	(CCT)6	18	14	31	GAAGGAGGAGGAGGAGGAGG	60.106	GTCCCACATCTCCCTCTCCT	60.032	234
TRINITY_Contig1897	2	p4	(GCAG)3	12	149	160	GAAGGAGGAGGAGGAGGAGG	60.106	GTCCCACATCTCCCTCTCCT	60.032	234
TRINITY_Contig1897	3	p3	(AGG)8	24	372	395	GAAGGAGGAGGAGGAGGAGG	60.106	GTCCCACATCTCCCTCTCCT	60.032	234
TRINITY_Contig1898	1	p3	(CTC)4	12	1976	1987	GCTCCTGTAACCCAAGACCC	60.035	AGTATGCCGAAGCTACTCGC	59.969	210
TRINITY_Contig1898	2	p5	(GGAGC)3	15	2131	2145	GCTCCTGTAACCCAAGACCC	60.035	AGTATGCCGAAGCTACTCGC	59.969	210
TRINITY_Contig1899	1	p4	(GACC)3	12	19	30	CATCCATCGATCCCAGCTCC	60.037	CTTCTCCTCCCACGGCAAAT	60.035	205
TRINITY_Contig1899	2	p3	(CGG)4	12	515	526	CATCCATCGATCCCAGCTCC	60.037	CTTCTCCTCCCACGGCAAAT	60.035	205
TRINITY_Contig1901	1	p3	(GCC)4	12	1013	1024	GAAAGCGCGATGTACTGCAG	59.974	GGGTTGTTGGTCGTCGTTG	59.972	141
TRINITY_Contig1902	1	p3	(TCA)4	12	2626	2637	TCCTCATTCTCCAGCTCCGA	60.033	AAGACCCGAGGCCTTTTCAG	59.963	136
TRINITY_Contig1902	2	p3	(GTC)5	15	3309	3323	TCCTCATTCTCCAGCTCCGA	60.033	AAGACCCGAGGCCTTTTCAG	59.963	136
TRINITY_Contig1905	1	c	(TCG)4ttgtcgatgggcgcgcctcctcttgcgggtgcccgccgcctgctgga(CGT)4tgtagatgggcgcgcctcctcttgcgggtacccgccgcctgctgga(CGT)4cggactcggacgacgacaagatgatgagatcgtcggggttggagttcgccgcccg(GTC)5	203	387	589	TGGCCCTGTTGTACATGTCC	59.962	ATGGTCTTTCAGCGACGAGG	60.109	218
TRINITY_Contig1907	1	p2	(GC)6	12	4076	4087	AGCCCACCTGCTGTAATGAC	60.035	AGGGACCAACACGTGATGAC	59.965	118
TRINITY_Contig1907	2	p3	(GCC)4	12	4213	4224	AGCCCACCTGCTGTAATGAC	60.035	AGGGACCAACACGTGATGAC	59.965	118
TRINITY_Contig1907	3	p5	(GAGGC)3	15	4350	4364	AGCCCACCTGCTGTAATGAC	60.035	AGGGACCAACACGTGATGAC	59.965	118
TRINITY_Contig1913	1	p3	(AGC)4	12	340	351	AATTCGGTCGCTTCAGCTGA	60.037	CTGCATCGCACATGGACATG	59.971	153
TRINITY_Contig1913	2	p3	(CGC)4	12	1662	1673	AATTCGGTCGCTTCAGCTGA	60.037	CTGCATCGCACATGGACATG	59.971	153
TRINITY_Contig1915	1	p3	(CCG)4	12	1127	1138	GGCAGCCAGGTACTAAGCAA	60.035	GACTCAAAGCAAGCTGGTGC	60.04	254
TRINITY_Contig1917	1	p3	(TCG)4	12	132	143	TATCCAACCTCGCTCAGCAC	59.824	AGCTTCAGCAAAGACGGACA	59.893	152
TRINITY_Contig1922	1	p2	(GC)7	14	197	210	AACCCGGGACTCTACGAGAA	59.961	ATGAGAAACGTGGACGGGTC	60.038	218
TRINITY_Contig1923	1	p3	(CCG)4	12	109	120	GTGCCTCCTAGGTGTGCAAT	60.035	TGCTGCGTCAGATTCCAAGT	59.965	265
TRINITY_Contig1924	1	p3	(CGC)4	12	93	104	CGTCAGCTTCATGCAGATGC	59.972	CAGTCGTTTCGCCTCTTCCT	60.038	214
TRINITY_Contig1930	1	p3	(GGA)4	12	286	297	GAGTACGCGATGAACGGCTA	59.971	ACATGCCGGACGATGTTCTT	60.036	104
TRINITY_Contig1931	1	p4	(CCTC)3	12	1921	1932	TCAACGCTGTTGACCCTCTC	59.966	AGCCGGAAGATGTCAGTTGG	60.036	182
TRINITY_Contig1934	1	p4	(GGGA)3	12	154	165	TCCTGTACAAGCACGTCCAC	59.967	GCGCTGTTGTTGCTGAAGAA	59.971	253
TRINITY_Contig1938	1	p3	(CCG)6	18	117	134	GCAACTCTGGAAAGGTCGGA	59.965	TCTTGGAGACTGTGTCACGC	59.968	189
TRINITY_Contig1940	1	p4	(AAAT)3	12	538	549	TGTACAAACCCTCTTCGCCC	59.964	CTCCTGCAACTGCTCTCCAA	59.964	158
TRINITY_Contig1940	2	p4	(GCGG)3	12	876	887	TGTACAAACCCTCTTCGCCC	59.964	CTCCTGCAACTGCTCTCCAA	59.964	158
TRINITY_Contig1942	1	p3	(CGC)5	15	513	527	GAAGTAGAACTCCGGCGAGG	59.899	CCGGTGAACATCTCCACCAA	59.964	180
TRINITY_Contig1942	2	c	(GGCG)3gcctcgtgagtgggtgtggtcggggcggggtgggttggctttgcgttggtgcgccgctgcccaccgccggtcgcgtcgggttggcgtgc(GCGCAG)3gcgctgccgtggcctcgtgccacggatgggggattggggacgggccgccgaaagggt(CGTCC)4	196	1940	2135	GAAGTAGAACTCCGGCGAGG	59.899	CCGGTGAACATCTCCACCAA	59.964	180
TRINITY_Contig1944	1	c	(CGA)5cgcgtgtctatcgaggcggcggaagcagacgcaggcattagtagggccgccagccttgagcacgacag(CGC)4ccagcggtcgtctct(GCA)4	122	359	480	GTGACGATACCGACTCGTCC	59.973	ACTAATGCCTGCGTCTGCTT	60.036	272
TRINITY_Contig1945	1	p5	(GCCGT)3	15	121	135	GCTTGCTTCTGATGGCATCG	59.971	GGCATTGCTGGCATCAGATG	59.968	247
TRINITY_Contig1945	2	p3	(GCG)5	15	427	441	GCTTGCTTCTGATGGCATCG	59.971	GGCATTGCTGGCATCAGATG	59.968	247
TRINITY_Contig1947	1	p5	(AGAGA)3	15	144	158	GACAAGGAGTCCGGCACC	60.047	CGCCCGTTCTCTCTTCTCTT	59.472	156
TRINITY_Contig1950	1	p5	(TCTCC)3	15	120	134	CCTCTCCTCTCCCCCAATCA	60.031	GAGGAGCCTTACCGGATTCG	59.968	185
TRINITY_Contig1950	2	p3	(CCG)6	18	255	272	CCTCTCCTCTCCCCCAATCA	60.031	GAGGAGCCTTACCGGATTCG	59.968	185
TRINITY_Contig1957	1	p3	(CCG)4	12	1134	1145	TGTCGCTGGTTTCATGCTCT	59.965	AATGTGCTCTTCATCGGCCA	60.035	165
TRINITY_Contig1957	2	p3	(GGA)4	12	1262	1273	TGTCGCTGGTTTCATGCTCT	59.965	AATGTGCTCTTCATCGGCCA	60.035	165
TRINITY_Contig1957	3	p3	(GCG)4	12	1646	1657	TGTCGCTGGTTTCATGCTCT	59.965	AATGTGCTCTTCATCGGCCA	60.035	165
TRINITY_Contig1962	1	p3	(GCC)4	12	322	333	GGTTCCTCCCGATCGATTCC	59.966	TCACATCGTCTCCCCTGGAT	60.032	248
TRINITY_Contig1963	1	p5	(GGATT)3	15	4560	4574	AAGATGATCCTGGCCTCCCT	60.029	GGCTTCTACTGACCGATCCG	59.969	194
TRINITY_Contig1963	2	c	(CCTCT)4ccttccgttggaaggccaaaacgcatcgtgtgcgtgacccgcgattc(CCA)6	85	4731	4815	AAGATGATCCTGGCCTCCCT	60.029	GGCTTCTACTGACCGATCCG	59.969	194
TRINITY_Contig1964	1	p4	(GGGC)3	12	220	231	TCGTTAGCCGCAGTGTTTCT	59.967	AGAATGACAAGGCGAGGCAA	59.963	153
TRINITY_Contig1967	1	p3	(CCT)4	12	2206	2217	TGCTCGTTTCGGTCAATCCA	59.966	CTGAAGGTTGAGGATGCGGT	60.036	257
TRINITY_Contig1967	2	p4	(GGGT)3	12	2758	2769	TGCTCGTTTCGGTCAATCCA	59.966	CTGAAGGTTGAGGATGCGGT	60.036	257
TRINITY_Contig1969	1	c	(AC)10(AG)19	58	471	528	CGAGGTGCCCTTTTGTCTCT	59.965	CATTGGGACCAACGCTCTCT	60.036	220
TRINITY_Contig1973	1	p3	(GAG)4	12	115	126	GAGGCAGGCAGAGCTGATAG	59.966	GGGGCGATTCTGAAAGGGAA	60.034	224
TRINITY_Contig1977	1	p4	(CGGC)4	16	360	375	TCGTCGTCGTTGGTTTGCTA	59.969	CTTGTGCCAAGTTCTTGGGC	59.967	207
TRINITY_Contig1978	1	p3	(GCG)4	12	234	245	GGTGGTGTACTCGCTCTACG	59.902	AAGAGAAGAAAAGGGCGCGA	59.965	100
TRINITY_Contig1978	2	p3	(TGA)4	12	429	440	GGTGGTGTACTCGCTCTACG	59.902	AAGAGAAGAAAAGGGCGCGA	59.965	100
TRINITY_Contig1980	1	p3	(CTT)4	12	2555	2566	GGTCCTCGGCATACACTAGC	59.968	TCTGTTCCTCACACCTCGC	59.966	114
TRINITY_Contig1985	1	c	(TCAT)3ttgtttagtgaagtccaatgcatctgtaagcaattcattgttgtctttggctcttattccatgagacctgatcttg(GATTT)3	103	1631	1733	ACCAATCCCCAAATCCGGAC	60.034	CCCAGGACGCTTACAGTTGT	59.965	188
TRINITY_Contig1986	1	p4	(AGGG)3	12	545	556	CCAGTCCCAACATGGCATCT	60.034	GGTGCGGATGGGAAGATCAA	60.107	192
TRINITY_Contig1989	1	p3	(GCC)4	12	163	174	GTACGCCAAGGAGTTGTGGA	59.966	GACGCCCTATTGCCCTACTC	59.966	214
TRINITY_Contig1992	1	p3	(CGC)4	12	861	872	CTATTGCTGCCCACACCTCA	60.035	GGAACCAACCAGAAGGGAGG	59.962	280
TRINITY_Contig2000	1	c	(GAC)4(CAC)4	24	69	92	GCCTTCAATTCCTCTGGGCT	60.034	GATCTGATGAGCAGCGACGA	59.97	133
TRINITY_Contig2000	2	p4	(CCGG)3	12	267	278	GCCTTCAATTCCTCTGGGCT	60.034	GATCTGATGAGCAGCGACGA	59.97	133
TRINITY_Contig2000	3	p5	(ATCAG)3	15	417	431	GCCTTCAATTCCTCTGGGCT	60.034	GATCTGATGAGCAGCGACGA	59.97	133
TRINITY_Contig2001	1	p3	(ATT)4	12	258	269	GTTGAGACCGACAGCAGTCA	59.968	AGAATGCCCCCAAGATGGTG	60.033	109
TRINITY_Contig2003	1	p3	(GTC)4	12	438	449	TGGCACATGCACTCGTAGTT	59.965	ACAACGACGTCTGCATCCTT	59.966	185
TRINITY_Contig2004	1	p5	(GGGCG)3	15	218	232	GGTCCGGCTACTCATCGAAG	59.969	CGATCCGCCAGATAGAGTGG	59.758	260
TRINITY_Contig2012	1	p4	(CATG)3	12	92	103	AAGGTAGCTACGTACGCAGC	59.9	TGCTCGATCCTTTGTCGCTT	60.037	140
TRINITY_Contig2013	1	p4	(TCCG)3	12	266	277	GTGGCCATGATCAAGGAGCT	60.107	TCTTTCGTGCTGAGCTCGTT	59.968	257
TRINITY_Contig2014	1	p3	(GCC)4	12	3547	3558	AGTCCCAAGAACTACAGCGC	60.037	GGGGAGGGGTACTTCTGGAT	60.03	197
TRINITY_Contig2014	2	p2	(AG)6	12	4080	4091	AGTCCCAAGAACTACAGCGC	60.037	GGGGAGGGGTACTTCTGGAT	60.03	197
TRINITY_Contig2015	1	p2	(TC)8	16	324	339	AGCTTCTCCCCCATACCCAT	60.029	CAGCAGAACCAAGTGTGTGC	59.97	168
TRINITY_Contig2017	1	p4	(GGAG)3	12	6	17	TGGAGGAGATGCAGGGTGTA	59.957	GACGACCTGACAAGCTTGGA	59.966	260
TRINITY_Contig2018	1	p3	(CAG)4	12	61	72	TCGTCGCGATTTATGAGGGG	59.968	AGCCTCCCGTAGTCTCACAA	60.252	161
TRINITY_Contig2018	2	c	(GGAT)3gcaacaacacggggccccgatcgaggccgatctcgaagtctcgtcgcgatttatgag(GGGC)3gcgtgcacctccgtccacatgcgtgcacgtcaggctagcggtgcgacgaccc(CGCT)4	149	251	399	TCGTCGCGATTTATGAGGGG	59.968	AGCCTCCCGTAGTCTCACAA	60.252	161
TRINITY_Contig2019	1	p4	(TTGC)3	12	225	236	CGCTGTTGTCCTCCTTCCTT	59.965	TGCCTTGCCATGTATGCAGA	60.034	122
TRINITY_Contig2021	1	c	(CAGCT)3cagttcatctactcgcttgaagccactcggcggctgatagctgctgcttcttctgacgcgatggtgtttcggtcct(TGC)5tggcgtgcgccgccgtggcgat(GGC)4	140	319	458	GACGTTCAAGAGGGTGCTGA	59.966	GCTGTAGTCATCCGAGCTCC	59.969	190
TRINITY_Contig2022	1	p4	(TCAT)3	12	26	37	CTAAGGCAGAGACGTAGCCG	59.97	AAGTGGTGGCTCTAATGGCC	60.034	138
TRINITY_Contig2026	1	p5	(CTCCG)3	15	161	175	GGTGGTCCGGATGTTGGATT	60.034	CCCTCAATGGCTCGGTTTCT	60.035	101

TRINITY_Contig2029	1	p3	(TTC)4	12	20	31	CCACGGCTCCTAATCTGCAA	60.108	GCCTCCAGTTACTCGGACAG	59.826	124
TRINITY_Contig2030	1	c	(CG)8atcccgatcagcgccggcgggctccgatccgtggggttgaggcacgccgcacgcgacgcctgccatct(TCG)4	96	225	320	TGCATCTTGGTGATTCCCCC	60.033	GATGATTCCCACCGGCAGAA	60.107	206
TRINITY_Contig2031	1	c	(CGG)8aaatcgcacccgagtacgaaccggagtcctgggctgcggagagggcggaaccggcgcaggcctccggagtcctttatgttggtggccaccagggcat(GGC)5	136	153	288	AGGCCTCCGGAGTCCTTTAT	60.031	GCATGGTGTTCTCCTCTCCC	60.107	215
TRINITY_Contig2032	1	p3	(CGC)4	12	289	300	GCTGGATGTACTCGATCGGG	60.039	CTTCGCGACGATCTACGACA	59.974	170
TRINITY_Contig2035	1	p5	(CATCT)3	15	104	118	GTCACCACCACCTTCTGGAG	59.965	TGGACGGGTTTCTTCCATGG	59.962	144
TRINITY_Contig2036	1	c	(CGG)5(TCG)4	27	531	557	TTCCCGTAGCTGGATCTCCA	60.033	AGAGGAAGAAGAACGTGCCG	60.038	121
TRINITY_Contig2036	2	p4	(AACC)4	16	1196	1211	TTCCCGTAGCTGGATCTCCA	60.033	AGAGGAAGAAGAACGTGCCG	60.038	121
TRINITY_Contig2038	1	p3	(CCA)4	12	5753	5764	TGGGATGGGAGTGGGATGAT	60.029	CAAGCCTCCAAACAACACCG	59.969	194
TRINITY_Contig2038	2	c	(CTGCT)3tgctgcg(GGT)5gggatgggagtgggatgatggccctgcgctgcgctcgtg(GCT)7	97	7487	7583	TGGGATGGGAGTGGGATGAT	60.029	CAAGCCTCCAAACAACACCG	59.969	194
TRINITY_Contig2038	3	p3	(GAG)4	12	7759	7770	TGGGATGGGAGTGGGATGAT	60.029	CAAGCCTCCAAACAACACCG	59.969	194
TRINITY_Contig2039	1	c*	(ATATA)3atttgccgatgatatatgatgaacaagggcaatagcatacaagatggatggatcgatcatataatatagtggc(AAGCT)4ct(CTAG)3(CTAGC)3*	133	249	381	TAGTTGACGGTGACCTTGCC	59.966	CACCTTCAAGAACAACGCCG	60.041	213
TRINITY_Contig2039	2	c	(TTGCA)4tgc(CCA)4	35	1036	1070	TAGTTGACGGTGACCTTGCC	59.966	CACCTTCAAGAACAACGCCG	60.041	213
TRINITY_Contig2043	1	p3	(CCG)4	12	132	143	TGCTTGAGAGAGGAAGCTGC	60.037	AAAAGCAAACAGACGCGTCC	59.971	130
TRINITY_Contig2049	1	p3	(CGC)4	12	48	59	GCGATGTCCGGAGTACGTTT	60.457	AGCATCCCCATAAGCATCGG	59.963	102
TRINITY_Contig2055	1	p4	(CGGC)3	12	116	127	TGCGTACCAGGCTTCCAAAT	59.962	CATGTCTGCTCGATCGCTCT	59.969	212
TRINITY_Contig2055	2	p3	(AGC)4	12	550	561	TGCGTACCAGGCTTCCAAAT	59.962	CATGTCTGCTCGATCGCTCT	59.969	212
TRINITY_Contig2055	3	p3	(AGC)4	12	736	747	TGCGTACCAGGCTTCCAAAT	59.962	CATGTCTGCTCGATCGCTCT	59.969	212
TRINITY_Contig2058	1	p3	(CGC)4	12	75	86	TTGATCGTCAGCGGGTAGTG	59.827	TGTGCCTGTACGTGCAGCAC	60.301	112
TRINITY_Contig2059	1	c	(AACG)4cgcgtgcgcacgaatcgacctgccgtgatgga(CGC)4	60	353	412	GCCTGTCCACCCTTCTCTTC	60.036	TGCCGCGAGATTAAGATCCC	59.966	141
TRINITY_Contig2067	1	p3	(GGT)6	18	202	219	AATCCCACATCGCGAACTGT	60.036	AGGATTCTGCGAAGTTGGCA	59.963	212
TRINITY_Contig2067	2	p4	(TCCA)3	12	2635	2646	AATCCCACATCGCGAACTGT	60.036	AGGATTCTGCGAAGTTGGCA	59.963	212
TRINITY_Contig2077	1	p3	(CGG)5	15	37	51	TCGGATTCCTGGACGAGTCT	60.034	GTCTCTCGAGGACGCAGATG	59.971	210
TRINITY_Contig2079	1	p2	(CT)7	14	790	803	GATAGCCGTGATGGATCGGG	60.109	CTGACACTGAAACTCGCCCT	59.966	120
TRINITY_Contig2080	1	p6	(GTGCCG)3	18	31	48	CAGAGCGACCGGGATCTC	59.275	AGAACTATCACAGCCCACCG	59.463	273
TRINITY_Contig2088	1	p3	(GCG)4	12	2958	2969	CAGGCCCATGACTGCTTGTA	60.035	CGTGGATTGCTGTTCGTTCC	59.834	270
TRINITY_Contig2089	1	p3	(CAC)8	24	106	129	CAAGTGCAACCAACCGACTG	59.971	CCAGTCACTCAACACCCCTC	59.965	164
TRINITY_Contig2092	1	p6	(GAAGGA)3	18	345	362	GCCTTCCTATGTATCGGGCC	60.036	CCTTCACCTTCACCTCCACC	59.963	155
TRINITY_Contig2093	1	p3	(AGA)8	24	354	377	TTTCCCTCGTTGACGGTGAG	59.967	TACGTGCTCATCGACATCCG	59.971	119
TRINITY_Contig2093	2	p4	(TTGC)3	12	1082	1093	TTTCCCTCGTTGACGGTGAG	59.967	TACGTGCTCATCGACATCCG	59.971	119
TRINITY_Contig2095	1	p3	(TCC)4	12	170	181	CCTCTTCCACCCAAGCAGAG	60.036	AGGAGGAGGAAGAGGACGAC	60.034	148
TRINITY_Contig2096	1	p4	(CTCC)3	12	1774	1785	GGACAAGACGAGGTTCCTGG	60.037	CACCAATTGCGTCAACAGCA	59.969	138
TRINITY_Contig2098	1	p3	(AGG)6	18	436	453	GACTTGCCAATGCCGTTTGT	59.968	CGTTCATCTCCGAGGAGCTC	59.97	208
TRINITY_Contig2102	1	p5	(CGCGG)3	15	225	239	GTTTTTAATCTCCGGCGCCC	59.9	GTCGATCTGAGCTCCGACG	60.011	189
TRINITY_Contig2103	1	p4	(CGCT)4	16	167	182	TGTTCGGGTGTAAACCTCCG	59.966	AACCCCCAAACCCAAACCAT	60.03	194
TRINITY_Contig2105	1	p3	(GCG)4	12	293	304	CCCCGATACGAAGCTCTTCC	59.968	AGTATGCCGATCATGCCGAG	60.038	222
TRINITY_Contig2106	1	p4	(CTCC)3	12	9	20	TCCCTCCGCTTCCAAGTCTA	59.959	CAACCACGCCAACTACTCCT	59.965	195
TRINITY_Contig2111	1	c	(GCTC)3acgagcacgcccccgtgcgccgcacgacctgccagag(AGAGA)3	64	298	361	ACGACCTGCCAGAGAGAGAA	60.251	CCTCTCCTCAGATCCGGAGT	59.815	152
TRINITY_Contig2115	1	p5	(TCTGA)3	15	306	320	ACACCTGATGGCTGGTTCAG	59.963	CATGTACCTTGCTTGACGCG	59.905	102
TRINITY_Contig2115	2	c	(GT)12atg(TA)8tgtatgtactag(TA)6aagaacgtaggaacagatatgcaacgaaagacgaacctagcattgaaatcgtagcaagacagaaagtaaagctaactaatca(AT)6	161	436	596	ACACCTGATGGCTGGTTCAG	59.963	CATGTACCTTGCTTGACGCG	59.905	102
TRINITY_Contig2115	3	c	(CTG)4ttggtgacgatgctggcttgccgcggtggtggacgcgcctcatcgagctcggcgtcgtcctcgttgt(CGC)4	92	822	913	ACACCTGATGGCTGGTTCAG	59.963	CATGTACCTTGCTTGACGCG	59.905	102
TRINITY_Contig2115	4	p3	(CGC)5	15	1038	1052	ACACCTGATGGCTGGTTCAG	59.963	CATGTACCTTGCTTGACGCG	59.905	102
TRINITY_Contig2116	1	p3	(GGC)4	12	377	388	TCGAGATCGATCGGGAGGG	60.303	ATTTGCTCAGGCTCACGTCA	59.965	133
TRINITY_Contig2121	1	p4	(GATG)3	12	208	219	ATGGATGTCTGGTGCAGTGG	60.035	CTGGACTTGATGCAGTCGGT	60.037	196
TRINITY_Contig2123	1	p3	(CGA)4	12	67	78	AGCTTGAGGATCTCGATGCG	59.968	CGAGGGGGATGGACTGGTA	60.076	184
TRINITY_Contig2123	2	p3	(GGA)4	12	181	192	AGCTTGAGGATCTCGATGCG	59.968	CGAGGGGGATGGACTGGTA	60.076	184
TRINITY_Contig2123	3	p5	(GCGCG)3	15	400	414	AGCTTGAGGATCTCGATGCG	59.968	CGAGGGGGATGGACTGGTA	60.076	184
TRINITY_Contig2124	1	p3	(CGC)4	12	220	231	AGTACTTGCACGTCTGTGGG	59.966	TTGAAGTGGAGGGTGATGCC	59.962	117
TRINITY_Contig2127	1	c	(ATAC)3acacatacacacgcacgatgggacggagcg(GAGC)3	54	92	145	CGCGCAATTATTTGGGGAGG	59.969	ACCTCATAATGCGCGAGAGG	59.967	150
TRINITY_Contig2127	2	p3	(CGC)7	21	887	907	CGCGCAATTATTTGGGGAGG	59.969	ACCTCATAATGCGCGAGAGG	59.967	150
TRINITY_Contig2130	1	p3	(GCC)4	12	330	341	TTCTCCGGCGTCAAGATCTG	59.827	TCCCCGAGATGGAGATGGAC	60.47	124
TRINITY_Contig2132	1	p3	(CGC)4	12	433	444	TCACCTCCACAACAGGATGC	59.963	CGGTTCGCGGTTTGTAGTTG	60.111	101
TRINITY_Contig2147	1	p6	(GGCTGC)3	18	421	438	GGAGGGAAGGAGAAAGCGTC	60.108	CCTCCCTCGTCTCCCTCTAC	60.179	184
TRINITY_Contig2154	1	p5	(AGCCA)4	20	302	321	TGTTCCACAAGACCGTCGAG	59.968	GTTGTCCTTGAGGTACCCCG	60.037	122
TRINITY_Contig2156	1	p3	(GCA)4	12	431	442	AAGCGTGTCGTGTAGTGGAG	60.039	TTCGACTTGCTGAGCGAGAG	60.11	137
TRINITY_Contig2162	1	p3	(CGG)4	12	73	84	GCTATCGCGTACTGGAGGAC	60.04	AACCTCAGAGGGCAAACTGG	59.889	140
TRINITY_Contig2166	1	c	(GCC)4ttgatggccgtaactccctctgcctcccattgccatgtgatgctgctgttga(TGC)4ggcggg(GGC)4(TGC)5	109	295	403	TCCCATTGCCATGTGATGCT	60.033	TCGATCAGATTGAGCCGACG	59.971	173
TRINITY_Contig2166	2	p3	(CGC)5	15	706	720	TCCCATTGCCATGTGATGCT	60.033	TCGATCAGATTGAGCCGACG	59.971	173
TRINITY_Contig2167	1	p3	(CCA)7	21	171	191	CTTCCTCCCCACCAACTCAC	59.963	GGCTGCCGCAAAATCTGAAA	60.038	265
TRINITY_Contig2167	2	c	(ACCG)3gcccccgagctgcagcgagccaccttcccccgacatgacgccgcatctgcccgccgcatcctcctccg(CCA)5cctcccg(CGC)7gcgccaccaccgccacca(CCG)4	153	300	452	CTTCCTCCCCACCAACTCAC	59.963	GGCTGCCGCAAAATCTGAAA	60.038	265
TRINITY_Contig2168	1	c	(CT)14(CGCT)3	40	50	89	CGCTCGCTCTTCCTCTACTG	59.971	TCGTCATGGAGTGCCTCAAC	60.037	178
TRINITY_Contig2170	1	p4	(GCAT)3	12	73	84	CGTAGTACTGCTGCCCGTAG	59.971	ATGATGATGGCGCCGATGAT	60.036	208
TRINITY_Contig2170	2	c	(GCCGTG)5cccgtggtgcgcgtgcccgccgctggccgccggcttcttgcccatgtcgaagtcgtcgaagtcgaagccgtcgtccagcat(GTC)8	135	764	898	CGTAGTACTGCTGCCCGTAG	59.971	ATGATGATGGCGCCGATGAT	60.036	208
TRINITY_Contig2172	1	p4	(GCCC)3	12	125	136	GGCTCTGCTTCCAGGCATAA	60.107	CCCTCCAGATCTCGGTGGTA	60.106	114
TRINITY_Contig2173	1	p3	(CCG)5	15	183	197	ACAAACCCACCGATCTGTCC	59.964	AGTTGATCCGCTCGAACCAG	60.109	150
TRINITY_Contig2174	1	p4	(ATAC)4	16	300	315	TTGGGGGATGATGCTCAACC	60.033	GCCTAATTGAACCCACCGGA	60.035	101
TRINITY_Contig2177	1	p4	(CACG)5	20	166	185	CCAGGCCGCAGTTAAGAAGA	60.036	GGTTATCACTCCACCCCACG	60.108	123
TRINITY_Contig2179	1	p3	(CGC)4	12	141	152	CAAGCATCGACGCCAAAACA	60.041	CCTCCTTCACCACCACATCC	60.035	257
TRINITY_Contig2189	1	p3	(GCC)5	15	51	65	GTTGAGGTTTGGGGTTTGGC	59.894	CCAATCCAACCCAATCCCCA	59.958	113
TRINITY_Contig2190	1	p3	(GGA)6	18	334	351	TCTCGCGCACACTAACCAAT	60.038	GATCATACATGCCGCCAACG	59.764	182
TRINITY_Contig2192	1	p4	(CTCC)3	12	204	215	TCGTTCAGGTTCAGGCTCAC	59.966	ACGGAAGAGGAATGTGCTGT	59.313	104
TRINITY_Contig2195	1	c	(AG)13cgagagagcgagagg(GCAGA)3	56	28	83	CTGGGGCAGTTCATCCATGT	60.034	GGGCCTTCTTGCTGCTAAGA	60.035	157
TRINITY_Contig2195	2	p3	(GAA)4	12	281	292	CTGGGGCAGTTCATCCATGT	60.034	GGGCCTTCTTGCTGCTAAGA	60.035	157
TRINITY_Contig2195	3	p3	(GCC)4	12	2187	2198	CTGGGGCAGTTCATCCATGT	60.034	GGGCCTTCTTGCTGCTAAGA	60.035	157
TRINITY_Contig2206	1	p2	(TC)7	14	323	336	CGTCGGGAAGATTCGTCGAT	59.971	CCATCGACGCAGCTAATCAG	59.973	112
TRINITY_Contig2207	1	p3	(CGC)4	12	290	301	CTTCACAGCTCTCGTGTGGT	59.967	TTACGATTACCCGTCCGCAG	59.899	148
TRINITY_Contig2212	1	p5	(TTCCA)3	15	171	185	TCCACTCGATGATTCCATTCGA	59.307	TGGAATCGAATGGAATCACCGA	59.83	242
TRINITY_Contig2213	1	p3	(GCC)4	12	325	336	AAGTTCTTCTTCCTCCGGGC	59.675	CGTGCACTGGTGTCCTAAGG	60.671	265
TRINITY_Contig2215	1	p3	(TGC)4	12	2276	2287	TGAAGCCTATGTTGCTGGGG	60.034	GCATGAGCGTTTGCATAGGG	59.97	213
TRINITY_Contig2215	2	p5	(GAGAG)3	15	3353	3367	TGAAGCCTATGTTGCTGGGG	60.034	GCATGAGCGTTTGCATAGGG	59.97	213
TRINITY_Contig2219	1	p3	(AAG)15	45	140	184	TAGGTGAACTTGACGGTGGC	59.966	CAGATCACAGAAGCCGCAGA	60.108	129
TRINITY_Contig2219	2	p3	(GCT)7	21	463	483	TAGGTGAACTTGACGGTGGC	59.966	CAGATCACAGAAGCCGCAGA	60.108	129
TRINITY_Contig2223	1	p3	(GTG)4	12	272	283	TGGGAAAACTTGGCACCCTT	60.032	TTGCAAGTAGAGCTCGTGCA	59.967	120
TRINITY_Contig2223	2	p3	(CGG)6	18	428	445	TGGGAAAACTTGGCACCCTT	60.032	TTGCAAGTAGAGCTCGTGCA	59.967	120
TRINITY_Contig2227	1	p3	(AGG)5	15	347	361	TTGGATCCTGGAATGGTGCC	60.033	TGTGCCACTGTAGTCGACAC	59.968	267
TRINITY_Contig2230	1	p2	(TC)8	16	52	67	GACAGGTGCTTCCTTGAGCT	59.964	TTGGCAAAGTTGGTGAGGGT	60.033	200
TRINITY_Contig2239	1	p3	(GGC)4	12	902	913	TTCTTGTCCGCCTTGAGGTC	59.965	TTCACAGGCCGAAGACCTTC	59.965	184
TRINITY_Contig2240	1	p3	(TGC)6	18	4646	4663	GGCAGGAAGTGACGACTCAA	59.966	TCTTATCCAGGAGCGGGGAA	60.032	199
TRINITY_Contig2240	2	p6	(GAGCCG)3	18	5099	5116	GGCAGGAAGTGACGACTCAA	59.966	TCTTATCCAGGAGCGGGGAA	60.032	199

TRINITY_Contig2240	3	p4	(CCTC)3	12	5590	5601	GGCAGGAAGTGACGACTCAA	59.966	TCTTATCCAGGAGCGGGGAA	60.032	199
TRINITY_Contig2248	1	p3	(AAG)4	12	24	35	CCAACACACACACGCTAAGC	60.041	TCGACCTGATCCCCTTCCAT	60.031	224
TRINITY_Contig2248	2	p3	(GAG)6	18	556	573	CCAACACACACACGCTAAGC	60.041	TCGACCTGATCCCCTTCCAT	60.031	224
TRINITY_Contig2250	1	p3	(CTC)5	15	1324	1338	TTCTTTCCTCGCAGGTCCAC	59.965	ACTGGCAGTCGAGGTCCTAT	60.033	137
TRINITY_Contig2251	1	p3	(GGC)4	12	471	482	CCGTCACCTCACTCAAGCTT	59.966	GGTGGCAGCTGGTAAGGAAT	60.034	136
TRINITY_Contig2254	1	p3	(CCG)6	18	11	28	CCTGCTCCATGAGAAGTGCA	60.036	TCAATTTGCCCAAGTGCTGC	59.966	177
TRINITY_Contig2254	2	p4	(TTCA)3	12	1511	1522	CCTGCTCCATGAGAAGTGCA	60.036	TCAATTTGCCCAAGTGCTGC	59.966	177
TRINITY_Contig2258	1	p2	(CT)10	20	1	20	GGTGCTTGGCAGAAAAGTGG	59.967	GCTCGGTTCTGGTCTTGACA	59.966	209
TRINITY_Contig2262	1	p3	(CGG)4	12	292	303	CTGGTGGTCAGGAAGATGGC	60.394	CAAACCCTCGTCCGTCTAGG	59.827	145
TRINITY_Contig2263	1	p3	(CCT)4	12	139	150	GGGGATCGTTTGGTCGGATT	60.107	CACGAGCCCTGAATCTCCTC	59.896	139
TRINITY_Contig2266	1	p3	(GGC)4	12	395	406	ATCCTGTAGCGGTTCCCTCT	60.032	CAAGGCAATGGTAACCGTGC	60.109	212
TRINITY_Contig2268	1	c	(CGC)6(CGA)4	30	1322	1351	GCTCACACACATTTGCTGCA	59.969	TGCCTTCATCGGAGCATTGT	60.035	129
TRINITY_Contig2274	1	c	(GACC)4gtcggttgctcggctccgctctgcc ctgcctcgccctcgcgtcgcatccaccgacccc cacccacatggaaattggtggggga(AGG TG)5	125	50	174	GTGTACCCAGATCGAGAGCG	59.97	AAAATCCCGGTCTGCTCCAG	60.035	122
TRINITY_Contig2274	2	c	(TGAT)3tcctttggcatactcctatttgggtg gccatttttgtagacctgataaaaagtac(TA) 8	83	1800	1882	GTGTACCCAGATCGAGAGCG	59.97	AAAATCCCGGTCTGCTCCAG	60.035	122
TRINITY_Contig2283	1	p3	(CGG)4	12	212	223	GCTACTCCATCCGCCAGATC	60.038	CTCACGACGACGATGTTGGC	61.675	166
TRINITY_Contig2286	1	p3	(TGC)5	15	386	400	TGCCAGCAAAGAGGTCTCTG	59.964	CACAGAGCAGCATCGCAAAG	60.179	265
TRINITY_Contig2287	1	p5	(AGCAG)3	15	1546	1560	AGATGCAGTGGTTGATGGGG	60.034	CTGAGGGCTTATCGGCTGAG	59.967	160
TRINITY_Contig2287	2	p3	(GCT)5	15	1695	1709	AGATGCAGTGGTTGATGGGG	60.034	CTGAGGGCTTATCGGCTGAG	59.967	160
TRINITY_Contig2287	3	p5	(TGCCA)3	15	1892	1906	AGATGCAGTGGTTGATGGGG	60.034	CTGAGGGCTTATCGGCTGAG	59.967	160
TRINITY_Contig2288	1	p3	(CCA)4	12	522	533	TCCTGTTCGTTCGCTCAGAC	60.039	AACGTGCAGACCAGGATCAG	60.037	217
TRINITY_Contig2290	1	p4	(GAAT)3	12	187	198	TGTGAACAATCTCGACGGCA	59.967	TGGGGTATGGAGAGTGAGGG	60.031	254
TRINITY_Contig2292	1	p4	(CTTG)3	12	1221	1232	GCTCAATAGCCCGTGCATTG	59.97	GCGAAGATCTCCGCGTTAGA	59.971	208
TRINITY_Contig2292	2	c	(AGC)5(GCC)5	30	1534	1563	GCTCAATAGCCCGTGCATTG	59.97	GCGAAGATCTCCGCGTTAGA	59.971	208
TRINITY_Contig2297	1	p5	(CTCCG)3	15	186	200	CGGAGACCTCTGCACCAAAT	60.036	ATCCCGTAGCTTTCCCTCCT	60.031	240
TRINITY_Contig2297	2	c	(CTC)4(GGC)4gttcgtgccgccgccgtcgc cgtccgacggggagctgctccgttcgctgcacc gcctcgcgcgggacctgt(CCG)5	110	336	445	CGGAGACCTCTGCACCAAAT	60.036	ATCCCGTAGCTTTCCCTCCT	60.031	240
TRINITY_Contig2308	1	c	(CGC)6cctccgatcgggctcgaatccgcgcc gctgaagcgaagccgaccccgcgccgatcccg gagcggact(CGC)4	97	1472	1568	TAACCAGAAGGACCAACCGC	59.964	AGGAGAGGAGGGGAATGGAC	60.031	193
TRINITY_Contig2310	1	p3	(TCG)6	18	185	202	AGAGCATCTCAACGATCCGC	60.249	CGAATCAATCCGCTCCTCGA	59.969	193
TRINITY_Contig2311	1	p4	(CGCC)3	12	259	270	TGTTGTGGTACGAACACGCT	60.179	CTACAGCTACGCCTACGACG	60.042	110
TRINITY_Contig2314	1	p6	(GAGAGG)4	24	370	393	CGTCTTCGATCCCTAGCACC	59.969	GCACTGTCCTTTCCCTCTCC	60.036	112
TRINITY_Contig2328	1	p3	(CCG)5	15	1246	1260	ATCGACGGGAATCATGCCTC	59.966	AGAGGTCACGTCTGCTGTTG	59.967	190
TRINITY_Contig2328	2	p5	(GCGGC)4	20	1743	1762	ATCGACGGGAATCATGCCTC	59.966	AGAGGTCACGTCTGCTGTTG	59.967	190
TRINITY_Contig2331	1	p3	(CGT)4	12	56	67	CGTCGTCGTCGTTGAACATG	59.911	CGATGGAGAAGGTGACGGTG	60.459	276
TRINITY_Contig2332	1	c	(GCG)4gtggagggagcgccgcggttcactt gggggcggcg(GGA)5	62	1796	1857	TTTGTGCACCCCACTTCAGT	60.034	GAATGGATTGCGGCTCGAAC	59.972	212
TRINITY_Contig2333	1	p3	(CGC)4	12	132	143	TTACGCCGCAATACAATCGC	59.698	GGGACGAGTGGAGACCATTC	59.824	107
TRINITY_Contig2336	1	p3	(CGC)4	12	269	280	ACCAAGATCCTTGCCATGCA	59.96	AAGAGTGGCTTCTGCAGGAC	59.964	128
TRINITY_Contig2336	2	c	(CCA)4ggtcgccgctgtcgaggaggacc(G CG)4	47	594	640	ACCAAGATCCTTGCCATGCA	59.96	AAGAGTGGCTTCTGCAGGAC	59.964	128
TRINITY_Contig2336	3	p4	(CTTT)3	12	909	920	ACCAAGATCCTTGCCATGCA	59.96	AAGAGTGGCTTCTGCAGGAC	59.964	128
TRINITY_Contig2339	1	p4	(ATTT)3	12	1436	1447	CAAAAAGCCCCAACCTTCGG	59.967	CCTTGGATGTGGTAGCCGTT	60.035	249
TRINITY_Contig2342	1	p3	(CGG)8	24	250	273	AGACACACTTGCTGGGATCG	60.037	TTGACCAACTCGCACCCTAC	59.966	225
TRINITY_Contig2343	1	p2	(GC)7	14	139	152	TTACAGGGCAGGGAGAAGGT	60.179	CCTCGTTCCGGTCTCGATTT	59.827	277
TRINITY_Contig2345	1	p3	(GAT)4	12	122	133	TGCTCCATGGCAACTAGGTG	60.035	ACTGCAGTCGACTTGTGGAG	59.967	119
TRINITY_Contig2348	1	p3	(CGC)4	12	585	596	ATCAAGTCGAGGGAGGTCGA	60.034	GGCTTCCTCGACCTGTGAAA	59.965	103
TRINITY_Contig2348	2	p6	(GGACGG)3	18	891	908	ATCAAGTCGAGGGAGGTCGA	60.034	GGCTTCCTCGACCTGTGAAA	59.965	103
TRINITY_Contig2349	1	p3	(CGC)4	12	973	984	CAGGGTTGGAGGATTTCCCC	60.033	GATCCCGGTGACAGCAAGAA	60.036	175
TRINITY_Contig2350	1	p3	(GCG)4	12	789	800	TTAGGCGGTGGAAGAGGAGA	59.959	GGGAGAGAGAGAAGGGGGAG	60.106	249
TRINITY_Contig2365	1	p3	(CCT)4	12	33	44	ATTAATCGCGGTGTGACCCG	60.808	GTGATCCTCCACGCCAAGAA	60.036	203
TRINITY_Contig2365	2	c	(GC)6ggcgccggacggatcgctctccttccc gggccccggctaccccggcaggggatcctgtg (GCCGC)3	86	207	292	ATTAATCGCGGTGTGACCCG	60.808	GTGATCCTCCACGCCAAGAA	60.036	203
TRINITY_Contig2368	1	p3	(CGC)5	15	233	247	CATGTACACGTCGTCCTCCG	60.523	GTTCTCGTGAACGCCTCCG	61.087	129
TRINITY_Contig2370	1	p2	(TC)6	12	362	373	AGAATATCCTGCAGCGAGCC	59.965	AGAACTCGCCATGTTGCTCA	59.965	201
TRINITY_Contig2372	1	c	(CTC)6ggccgccgctcccagattcacct(CG C)4	53	50	102	GGGCATCGACGCTATCTTCA	59.968	CACACTGCACCTCATCCCAT	60.035	162
TRINITY_Contig2374	1	p3	(GGC)7	21	34	54	CGGAGGCAGTCAATCCTTGT	60.036	GAGCGAGGAGGAGTAGGGAA	60.107	274
TRINITY_Contig2374	2	p4	(CTCG)3	12	273	284	CGGAGGCAGTCAATCCTTGT	60.036	GAGCGAGGAGGAGTAGGGAA	60.107	274
TRINITY_Contig2377	1	p3	(GCC)4	12	243	254	GTTGCCGTCACTGTCGTCTA	60.04	GGAGCCTCCTTGTAGGCTTG	60.107	180
TRINITY_Contig2385	1	p3	(AGA)4	12	2176	2187	CTATGCGAGTCCGTGATGCT	59.969	TCGGTTAAGCACAGTGTCCC	59.966	251
TRINITY_Contig2395	1	p3	(CGG)4	12	1107	1118	GGTTGAGAGGAGGTGAAGGC	60.036	AACCCTAATCCTCCTCCCCC	60.029	235
TRINITY_Contig2402	1	p3	(TTC)4	12	488	499	ATAGACCAACACCAGGCAGC	60.035	CGCCGGGTTTAGTTCTCTGT	60.038	106
TRINITY_Contig2408	1	p2	(TC)8	16	193	208	GCTCTAGGCCTGCTAGCTTC	59.967	GGCAAGCAAAGACCAGTTGG	59.967	128
TRINITY_Contig2408	2	p6	(TACACA)3	18	314	331	GCTCTAGGCCTGCTAGCTTC	59.967	GGCAAGCAAAGACCAGTTGG	59.967	128
TRINITY_Contig2408	3	p5	(AGTTC)3	15	2611	2625	GCTCTAGGCCTGCTAGCTTC	59.967	GGCAAGCAAAGACCAGTTGG	59.967	128
TRINITY_Contig2408	4	p2	(AG)13	26	2810	2835	GCTCTAGGCCTGCTAGCTTC	59.967	GGCAAGCAAAGACCAGTTGG	59.967	128
TRINITY_Contig2409	1	p3	(CGA)4	12	1728	1739	CCTCATCCTTCCTCGTACGC	59.969	GGGTGAATTCCCCATTCGGT	60.034	142
TRINITY_Contig2414	1	p3	(GGT)4	12	6	17	GGCGATGTACGAGATGGAGG	60.039	CTTCCTCGTCAGGGGCAATG	60.746	151
TRINITY_Contig2423	1	p3	(TTC)4	12	977	988	GATCTCCCATCTTGAGGCCG	59.965	AGGATGTTCGTACCATCGCC	59.896	122
TRINITY_Contig2424	1	p5	(CAGTA)3	15	116	130	ACTCGGAGATCTGGTCGTCA	60.035	GCATTGTACGCCATGAGCAG	59.971	187
TRINITY_Contig2424	2	p3	(CGA)4	12	1482	1493	ACTCGGAGATCTGGTCGTCA	60.035	GCATTGTACGCCATGAGCAG	59.971	187
TRINITY_Contig2427	1	p3	(AGC)4	12	295	306	TCCGTGATCTCGAGCAATCG	59.971	CTTCGGCAAGCTCCTGATCA	60.108	102
TRINITY_Contig2430	1	p2	(TG)18	36	1	36	CCCAGGCGGAAACTGAATCT	60.035	GTCGCCTCCCTCATCGTAAG	59.969	184
TRINITY_Contig2430	2	c	(CCG)4ttctgatccgccttgtccacgtccatg ctcttgagcttccgcgacgcctcctcgtcctccc gcccgccggcgcgcatt(CTCG)3	102	1284	1385	CCCAGGCGGAAACTGAATCT	60.035	GTCGCCTCCCTCATCGTAAG	59.969	184
TRINITY_Contig2434	1	p3	(GGC)4	12	2062	2073	TGAAGCTCCAGTGCCTCATG	60.036	CTTTCTGGTGAGTCGGCCAT	60.036	122
TRINITY_Contig2435	1	p5	(GCGGC)3	15	2024	2038	GACCGCTAACTATGACGGGG	59.968	GCCCGAATCCGATCCATTCT	59.965	265
TRINITY_Contig2436	1	c	(CT)13cccgtcgtcgccatttattactcctctt cgccgcaaaattccagtcgcaataaagggtcc tgcgtccaccgtccacgtgggcggctgct(GC G)4	127	157	283	TGCCTACCCAGCTTTGGATG	60.034	TGCCCCAGCCAACTTTCTAG	59.962	190
TRINITY_Contig2442	1	p2	(CA)8	16	179	194	CAGATCAGGGTCAGCCATGG	60.179	GGGACAAAGACAAGCCTCCA	59.89	161
TRINITY_Contig2444	1	p2	(GA)10	20	648	667	TGTCCTTGCTGACGAAGGAC	59.966	TGCAGCTGGGATTTCCGATT	60.034	185
TRINITY_Contig2446	1	c	(GGC)4ggggtgcgcgacgcaccccgcggcg tgctcgcgggagacggcggcgtgatgaagac ggtgaggttcccggccctcccgatcatg(GGC)5	111	879	989	CGCCCACTGGTACTTGGATT	60.035	CTCAGTACGAGAAGGCGTCC	59.901	155
TRINITY_Contig2447	1	p2	(CT)8	16	304	319	CCCTCGCGTTTATAGCTCGT	59.97	GGTTGGGTTCTGGTCTGGAG	59.963	254
TRINITY_Contig2447	2	c	(GGCCT)3ggcccccaccgtccaccccctcca acccaagcaaggcacatatgcctacgctccgt cagagaacgaagc(TGCT)3ttttaaaaggg acccaaacccaaacccccttctttgtcagcatca gcaccgagcgccaccaccatggccatggctcc gccg(CTC)4	187	628	814	CCCTCGCGTTTATAGCTCGT	59.97	GGTTGGGTTCTGGTCTGGAG	59.963	254
TRINITY_Contig2447	3	p3	(CTC)4	12	1228	1239	CCCTCGCGTTTATAGCTCGT	59.97	GGTTGGGTTCTGGTCTGGAG	59.963	254
TRINITY_Contig2447	4	p3	(CGT)4	12	2235	2246	CCCTCGCGTTTATAGCTCGT	59.97	GGTTGGGTTCTGGTCTGGAG	59.963	254
TRINITY_Contig2447	5	p5	(TCTCA)3	15	2622	2636	CCCTCGCGTTTATAGCTCGT	59.97	GGTTGGGTTCTGGTCTGGAG	59.963	254
TRINITY_Contig2447	6	p4	(TTTC)3	12	3407	3418	CCCTCGCGTTTATAGCTCGT	59.97	GGTTGGGTTCTGGTCTGGAG	59.963	254
TRINITY_Contig2448	1	p4	(GGAA)3	12	153	164	CTCTCGCCATCGGAAGGAAG	60.249	GATCAGCAAACGAATCGGCC	59.972	112
TRINITY_Contig2450	1	p3	(GGC)5	15	2685	2699	CGGAGAGTCCTGGAGGAGAA	60.034	CGTTCAGATCGGATCGAGCA	59.971	110
TRINITY_Contig2450	2	p4	(GGGC)3	12	2944	2955	CGGAGAGTCCTGGAGGAGAA	60.034	CGTTCAGATCGGATCGAGCA	59.971	110
TRINITY_Contig2451	1	p3	(ACC)4	12	1011	1022	CCACCATGTAGGGCCGATAC	59.965	ACGTCCCATCTTCCTCTCA	59.959	190
TRINITY_Contig2451	2	p3	(CGG)4	12	1364	1375	CCACCATGTAGGGCCGATAC	59.965	ACGTCCCATCTTCCTCTCA	59.959	190
TRINITY_Contig2460	1	p4	(TCCA)3	12	193	204	TCCTTGACGTCGTACTTGGC	60.039	CTGAACCAGGACATCCACCC	60.035	113

TRINITY_Contig2460	2	c	(CCGCG)3cccgagcccagcagcggcgtcc gcagcccttgcccctgcgccgccgcccgcggc gacaccctcct(CGACGC)3	98	647	744	TCCTTGACGTCGTACTTGGC	60.039	CTGAACCAGGACATCCACCC	60.035	113
TRINITY_Contig2461	1	p5	(CCGTC)3	15	76	90	AGCTGTTGAAAGCAAAGGCG	59.969	TGTGCGACTTTTTCAGCAGC	59.971	263
TRINITY_Contig2461	2	p3	(GCG)5	15	619	633	AGCTGTTGAAAGCAAAGGCG	59.969	TGTGCGACTTTTTCAGCAGC	59.971	263
TRINITY_Contig2469	1	c	(CCATT)3ccgttccgttccggg(GGA)4	42	333	374	CGTTTAGGCCTCGCGAATTG	59.974	GAAGATTGCACGTACGCCAC	59.905	118
TRINITY_Contig2473	1	p4	(GAAT)3	12	1156	1167	TGGCATATTTCATTGGCGCG	59.969	GCAAACAGCCAAACGAGAGG	60.04	257
TRINITY_Contig2475	1	c	(TC)11tt(TC)11(TG)11(TA)11	90	10	99	AAGGAGAAATCCGCCCAAGG	60.034	GTGGCTGATCATCCTCTCGG	59.967	107
TRINITY_Contig2476	1	p6	(GGCAGC)3	18	125	142	CCTGTCCCAGAGCCTCTACT	60.033	GGTCCGCAAGTTGGTTGAAC	59.97	227
TRINITY_Contig2477	1	p4	(CACG)3	12	302	313	CGAACTGGGCGTTGAAGTTG	60.041	GCCAACACACAAAGCTCCAG	59.969	209
TRINITY_Contig2477	2	p5	(CCCGC)3	15	1763	1777	CGAACTGGGCGTTGAAGTTG	60.041	GCCAACACACAAAGCTCCAG	59.969	209
TRINITY_Contig2478	1	p4	(CCAC)3	12	178	189	CGGTAGAACTGGATGACGGG	59.898	AGGGAAGAAGGAAATGGCGG	60.034	165
TRINITY_Contig2478	2	p3	(GCG)5	15	748	762	CGGTAGAACTGGATGACGGG	59.898	AGGGAAGAAGGAAATGGCGG	60.034	165
TRINITY_Contig2478	3	p3	(TGG)4	12	1771	1782	CGGTAGAACTGGATGACGGG	59.898	AGGGAAGAAGGAAATGGCGG	60.034	165
TRINITY_Contig2481	1	p4	(CCGC)4	16	2213	2228	CAGTGCCTCCCAACATCTGT	59.963	AAGAAAGAGGCAACGGAGGG	59.963	180
TRINITY_Contig2482	1	p4	(CCGA)4	16	2733	2748	CATGATCGAGCGTGTACCGA	59.971	CTGGTGATGGGGTCCTGTTC	60.035	210
TRINITY_Contig2484	1	p3	(GGA)5	15	1343	1357	CAATGTCCTGTTGCACTGCC	60.039	ATCCTCCCCCTCCTCCTTTC	60.03	204
TRINITY_Contig2485	1	p3	(GCG)4	12	141	152	CCTCGTCGTCTGGATCATGG	59.969	TTGTTCCACGTCCGGGTTAG	59.966	181
TRINITY_Contig2486	1	p3	(GCT)4	12	753	764	CCCCGAGTTGAAGCTTGTCT	59.965	GCGATGGTAGTAGTGGAGGC	59.968	273
TRINITY_Contig2489	1	p3	(GGC)4	12	1344	1355	ATCCTCCTCCATGGCCTTCT	60.029	CGACACCGAGCCTAGCTTAG	59.97	277
TRINITY_Contig2490	1	c	(CTGC)3tgc(CTCG)3	27	3965	3991	GGGTGCAACAGCAACTTCAG	59.969	CCAAAGCCGACTCAGTCAGT	59.966	236
TRINITY_Contig2491	1	p4	(GGAG)3	12	2187	2198	GATGTGAACGTGGCGATGTG	59.905	TGCTAATTGGGCGGGTGATT	60.034	199
TRINITY_Contig2492	1	p3	(GGC)4	12	2124	2135	CCAAACAGATACATGCCGCG	59.972	AAGTCAGATCACGAGGGGGA	59.959	235
TRINITY_Contig2497	1	p4	(TGAA)3	12	765	776	TCCAGTTTCTTCACCTCCGC	59.965	TCAATGGGTAGGGGGAGAGG	60.03	173
TRINITY_Contig2497	2	p2	(GA)12	24	1245	1268	TCCAGTTTCTTCACCTCCGC	59.965	TCAATGGGTAGGGGGAGAGG	60.03	173
TRINITY_Contig2500	1	p5	(GAGGG)3	15	1711	1725	TCAGATTCAGCAGCCTGGTG	60.036	GTTAGATCTGAGCACCCCCG	59.895	208
TRINITY_Contig2501	1	p3	(CCT)4	12	1794	1805	GTCTTGGCACTGTACTGGCT	59.965	AACGGAGCAACCAAATTGGC	59.967	175
TRINITY_Contig2504	1	p3	(GCA)4	12	2978	2989	CAGATCCATTGCCATGCACG	59.97	CTCATCATCAGGCCGTCCTC	59.967	222
TRINITY_Contig2505	1	p4	(ACGG)3	12	1230	1241	GGCATCCCATCAAACAGGGA	60.033	CATCTCCCTCTCATCTACCC	59.967	138
TRINITY_Contig2506	1	p5	(TCAAC)3	15	4873	4887	CAACAAGTGTGTTCAGGGCG	59.971	CCAAGTGCCAGCTAGTGTGA	59.965	109
TRINITY_Contig2508	1	c	(CACGCG)4cacagcgaagaaatggcgatg gcgatgcagccatcttagccccct(CCCCTC) 3	87	1210	1296	AACGAGTTCCTGCTGGAGTG	59.966	TCCATCCCCTCCATGTCCAT	60.029	161
TRINITY_Contig2508	2	c	(GCG)4g(CAGCGC)3	31	1398	1428	AACGAGTTCCTGCTGGAGTG	59.966	TCCATCCCCTCCATGTCCAT	60.029	161
TRINITY_Contig2509	1	p3	(GGC)4	12	2037	2048	TCAAACGACAGCAGGAGCAT	59.965	CCGTAGGTTCTATGGACCGC	59.968	141
TRINITY_Contig2511	1	p4	(CTTT)3	12	2871	2882	TTCTGCTCTGTGCGAATCGT	60.038	GTCCATCTCCAGGTCCTCCT	60.032	264
TRINITY_Contig2512	1	p6	(CTCTTC)3	18	975	992	ACCACAATCCCTGCTTCGAG	60.036	AGAGAGAGCTGGAGTCTGGG	60.033	106
TRINITY_Contig2513	1	p3	(CGG)4	12	197	208	GTGCAGTCCCCTTGCATACT	60.035	CAAACTCAAGTGCAGTGGCC	59.969	157
TRINITY_Contig2513	2	p2	(CT)11	22	1345	1366	GTGCAGTCCCCTTGCATACT	60.035	CAAACTCAAGTGCAGTGGCC	59.969	157
TRINITY_Contig2516	1	p3	(CCG)4	12	60	71	AGCATCTGTGTACACGCCAA	59.965	TCTGTACCAGGTGACAACGC	59.967	256
TRINITY_Contig2516	2	p2	(GA)18	36	4359	4394	AGCATCTGTGTACACGCCAA	59.965	TCTGTACCAGGTGACAACGC	59.967	256
TRINITY_Contig2517	1	p3	(CGC)6	18	46	63	AGTTGCAGATGGCTCATGCT	60.034	CAGTCAACGCCCAAACCTTG	59.969	191
TRINITY_Contig2518	1	p4	(GCGT)3	12	2710	2721	TATGCCAATGTTGTCCCGCT	60.035	CTGGCTGCTGCTAGGAAAGT	60.036	254
TRINITY_Contig2519	1	p6	(TCGCCG)3	18	48	65	GTTTCTGGACGTTTCGTGGC	60.042	GCATGGAACCGAATCGAAGC	59.972	140
TRINITY_Contig2519	2	p3	(ACG)5	15	196	210	GTTTCTGGACGTTTCGTGGC	60.042	GCATGGAACCGAATCGAAGC	59.972	140
TRINITY_Contig2521	1	p3	(CGG)5	15	2428	2442	GCCTGAAAGCACGCTCAAAA	59.969	TCGCGAGTTGAGTGCTTTCT	59.968	115
TRINITY_Contig2521	2	p4	(CCGC)3	12	4552	4563	GCCTGAAAGCACGCTCAAAA	59.969	TCGCGAGTTGAGTGCTTTCT	59.968	115
TRINITY_Contig2522	1	p6	(CGGCGA)3	18	333	350	GGCGGCATTTAGAGGTCGTA	59.896	AGCTTGAGGTTCGCAGTGAA	59.893	229
TRINITY_Contig2523	1	p3	(CCG)4	12	100	111	GCTTTCTTCCCCCTTCCCTC	60.034	TTCTTGGAGGGTCGTCTTGC	59.965	219
TRINITY_Contig2524	1	p3	(CGG)4	12	2090	2101	GAGGTGCCTGGAGTGAGTTC	60.037	GAGGAGAGTATGCACCGTCG	59.97	259
TRINITY_Contig2525	1	p3	(GCC)6	18	1503	1520	GAGCACCCCTCAACTTCTCC	60.036	TTCTTGGTCGGGAAGGAAGC	59.964	153
TRINITY_Contig2525	2	p3	(TGG)4	12	1879	1890	GAGCACCCCTCAACTTCTCC	60.036	TTCTTGGTCGGGAAGGAAGC	59.964	153
TRINITY_Contig2525	3	p4	(GGGA)4	16	2023	2038	GAGCACCCCTCAACTTCTCC	60.036	TTCTTGGTCGGGAAGGAAGC	59.964	153
TRINITY_Contig2527	1	p3	(CTG)4	12	473	484	CACGATGACCTGGCTCAGTT	60.037	TCTCGGCTGTTGCTCATTGT	59.965	158
TRINITY_Contig2527	2	p6	(CCACCT)3	18	4264	4281	CACGATGACCTGGCTCAGTT	60.037	TCTCGGCTGTTGCTCATTGT	59.965	158
TRINITY_Contig2528	1	p2	(GA)16	32	2186	2217	CCTCTACCTCCACTTCCGGA	60.033	ACCTCATTGCATCCCAAGGG	60.033	117
TRINITY_Contig2529	1	p4	(AGCA)3	12	117	128	CCAGCACAAGAACTTGGTGC	59.969	ATGTTGCCTTGCAGGATCGA	60.035	124
TRINITY_Contig2529	2	p3	(TGC)5	15	4269	4283	CCAGCACAAGAACTTGGTGC	59.969	ATGTTGCCTTGCAGGATCGA	60.035	124
TRINITY_Contig2530	1	c	(GCG)5gaggtcgcgccggccccgccccctc(CCG)4	51	174	224	CATCCAGAGGTGAGGGAGGA	60.032	AGCTAGCCCATCTGCTGTTG	60.107	150
TRINITY_Contig2531	1	p6	(GCCGGA)3	18	2128	2145	GTCGGAGATGCTGCTCTACC	59.969	TCGTTACATGCACACACGGA	59.968	247
TRINITY_Contig2531	2	p3	(TGC)4	12	2279	2290	GTCGGAGATGCTGCTCTACC	59.969	TCGTTACATGCACACACGGA	59.968	247
TRINITY_Contig2532	1	p6	(CGCCTC)4	24	65	88	TGCTGTCTTCCCGTGTGTAC	59.967	GGAAAACTTGGCTGCACCTG	59.967	250
TRINITY_Contig2532	2	p3	(GAA)5	15	1555	1569	TGCTGTCTTCCCGTGTGTAC	59.967	GGAAAACTTGGCTGCACCTG	59.967	250
TRINITY_Contig2533	1	c	(GCG)4cgctccgacgccggaagctcgagga cgaggagtactcatccgccgcgggagctgagg gggagtacggcagcggcggcgggtcccagag ct(GCG)4	114	1316	1429	AGGAAGACGAGGAGGAGGAC	60.034	CGTCGTCCTCTTCTTCCTCG	59.903	157
TRINITY_Contig2535	1	p5	(CGACG)4	20	1492	1511	TGCTTGTCGATGTCCTGGTC	60.037	CTCTTATCCTGCTCCAGCCG	59.967	202
TRINITY_Contig2535	2	p4	(GGGA)4	16	1683	1698	TGCTTGTCGATGTCCTGGTC	60.037	CTCTTATCCTGCTCCAGCCG	59.967	202
TRINITY_Contig2536	1	p4	(CGGG)3	12	1041	1052	ACAGACGAGACGAGGTGAGA	59.965	TTTAGGTGTCGTGTTCGCGA	59.969	111
TRINITY_Contig2539	1	p3	(CCT)4	12	43	54	TGGTCATCAACTGCTCGCAT	60.036	GACCTGCCAATCACCTCGAA	60.036	180
TRINITY_Contig2540	1	p5	(GTGCG)3	15	1871	1885	GCTCATCTCGGCATGCAAAG	59.971	CTGCGAAATGGCGTTTGTGA	60.041	195
TRINITY_Contig2541	1	p4	(GCGA)4	16	151	166	CGTTTTGGCGTGAAAGCAGA	59.971	CCTTCGCTCCCATCATCTCC	59.965	235
TRINITY_Contig2541	2	p3	(GGC)6	18	398	415	CGTTTTGGCGTGAAAGCAGA	59.971	CCTTCGCTCCCATCATCTCC	59.965	235
TRINITY_Contig2544	1	p3	(CAA)4	12	488	499	AAGCAATCGAGACCAAGGCA	59.963	ATGGAGAGGAGATGGTGGCT	60.03	112
TRINITY_Contig2549	1	p3	(CGC)5	15	1721	1735	CTTGCAGGCAAGCGTATTCC	59.901	TCTCCCTCCATAATGGCCCA	60.029	210
TRINITY_Contig2549	2	p5	(GCGCC)4	20	1931	1950	CTTGCAGGCAAGCGTATTCC	59.901	TCTCCCTCCATAATGGCCCA	60.029	210
TRINITY_Contig2550	1	p3	(CGC)4	12	252	263	AGTTCGGTGTCGACGCTATC	59.902	CCCAAGACTGCACAACAACG	59.971	136
TRINITY_Contig2552	1	p3	(CCG)4	12	178	189	GAAGTCGAGCAGGTAGACGG	59.901	TGAAGCTGTGCTACCACCTG	59.965	233
TRINITY_Contig2552	2	p5	(AGCTG)3	15	639	653	GAAGTCGAGCAGGTAGACGG	59.901	TGAAGCTGTGCTACCACCTG	59.965	233
TRINITY_Contig2553	1	p3	(CGC)4	12	1220	1231	CATGCGCTAGTATCAGCCCA	59.966	GCGAGCTGTTTCTTGGCAAA	59.969	165
TRINITY_Contig2554	1	p3	(GCG)6	18	103	120	AAGCTGGCATGTACCAACGA	59.964	TGAACCTCATCTGCAGCCAG	60.036	248
TRINITY_Contig2554	2	p3	(CCG)4	12	339	350	AAGCTGGCATGTACCAACGA	59.964	TGAACCTCATCTGCAGCCAG	60.036	248
TRINITY_Contig2554	3	p3	(GGC)4	12	1030	1041	AAGCTGGCATGTACCAACGA	59.964	TGAACCTCATCTGCAGCCAG	60.036	248
TRINITY_Contig2555	1	p5	(GCCGC)3	15	1372	1386	ATTGGGGATGGAGGACCGTA	60.031	TCCTCCATAAGCCCTGACGA	60.033	184
TRINITY_Contig2555	2	p3	(TGA)4	12	3429	3440	ATTGGGGATGGAGGACCGTA	60.031	TCCTCCATAAGCCCTGACGA	60.033	184
TRINITY_Contig2556	1	p3	(TCG)4	12	2030	2041	CCTCCTCCCCTTCCCCATAA	60.029	TGTGTGGCTTCTTTGGGGTT	60.033	162
TRINITY_Contig2557	1	p2	(CT)6	12	112	123	AGTGGCTGGTATTGGTGTCG	60.036	TCCGAATAATCCAGAGCGCC	59.966	222
TRINITY_Contig2558	1	p3	(TGC)4	12	2377	2388	ATGCTGAGACGTCTGTTGCA	59.966	CTCAGGTGCCATTGCTGGTA	60.035	106
TRINITY_Contig2561	1	p3	(GGC)5	15	88	102	ACTGGATGCTAGGGGCCTTA	60.03	TCCATTATGCGTGGCCTTGT	60.035	186
TRINITY_Contig2562	1	c	(CCT)4tctcgtgctcctccttcacgccgcca(T CGTCC)3tccgacccatccgag(GCCCCC) 3	89	1253	1341	CATGCATCATCACTGCGTGG	59.971	ATGGAAGCTGGTCAGTGAGC	60.036	247
TRINITY_Contig2562	2	p3	(GGC)5	15	1638	1652	CATGCATCATCACTGCGTGG	59.971	ATGGAAGCTGGTCAGTGAGC	60.036	247
TRINITY_Contig2565	1	c	(TCG)4gcgtcggcggcgtcggcgtccgttc accgccgcg(GGCGCC)3	65	1848	1912	CTTCCCCGCACCAAACTTTG	59.968	AGAGCGGCTTCAATTCGGAA	60.036	187
TRINITY_Contig2566	1	c	(GGC)4(GAG)4	24	759	782	TTGCTGGAGAGGATGCGTTT	59.963	TGAACACTGACACGTCTCGG	59.97	246
TRINITY_Contig2567	1	p5	(CGCTC)3	15	1	15	GCTCGCTCTTGCATTCCATG	59.971	GCTAAAAAGCTCTGCCTCGC	59.902	148
TRINITY_Contig2571	1	p3	(GAC)4	12	380	391	GAGGGTGGTGTCTGTCTGTG	59.966	ACACCAAGATTCTCCCTGCG	60.036	154
TRINITY_Contig2571	2	p3	(CGC)4	12	554	565	GAGGGTGGTGTCTGTCTGTG	59.966	ACACCAAGATTCTCCCTGCG	60.036	154
TRINITY_Contig2572	1	p3	(CGG)4	12	325	336	GCGATCATCAGAGGGGTGAG	59.967	CCTCCGGATGAATTCCTCCG	59.966	279
TRINITY_Contig2573	1	p3	(GCA)6	18	657	674	TCAAGCACTCACTTCGGACC	59.966	TTCCCTCAGAGCGAACAACC	59.965	240
TRINITY_Contig2573	2	p3	(CCG)4	12	1210	1221	TCAAGCACTCACTTCGGACC	59.966	TTCCCTCAGAGCGAACAACC	59.965	240
TRINITY_Contig2573	3	c	(TCG)6tccacgtcga(CCT)4catcc(TCG) 4	57	6460	6516	TCAAGCACTCACTTCGGACC	59.966	TTCCCTCAGAGCGAACAACC	59.965	240
TRINITY_Contig2573	4	p3	(GGA)5	15	6653	6667	TCAAGCACTCACTTCGGACC	59.966	TTCCCTCAGAGCGAACAACC	59.965	240
TRINITY_Contig2575	1	p3	(GAT)7	21	726	746	CACTGTTGCGTACCACCTCT	59.966	TCTCAATCAGCGTGCCTGTT	59.965	212
TRINITY_Contig2576	1	p4	(GTCT)3	12	207	218	TCCTATGGCATCGTGACACG	59.898	CGCAGCTGCAAGACATCTTC	59.903	223
TRINITY_Contig2576	2	p4	(GCAT)3	12	747	758	TCCTATGGCATCGTGACACG	59.898	CGCAGCTGCAAGACATCTTC	59.903	223
TRINITY_Contig2577	1	p2	(GA)6	12	510	521	CCACTTGCTTGTCACAAGGC	59.969	CCGACGAGTACCAGATGAGC	59.97	226
TRINITY_Contig2577	2	p4	(GAGG)3	12	3087	3098	CCACTTGCTTGTCACAAGGC	59.969	CCGACGAGTACCAGATGAGC	59.97	226
TRINITY_Contig2577	3	p3	(GTG)4	12	3292	3303	CCACTTGCTTGTCACAAGGC	59.969	CCGACGAGTACCAGATGAGC	59.97	226
TRINITY_Contig2578	1	p3	(TTC)4	12	626	637	TTCGTGGAGCTGGTGAAGAC	59.966	GAGATCTTTGGCCTTGGCCT	60.034	168
TRINITY_Contig2579	1	p3	(CGG)4	12	839	850	ACTGCCCTATCAAGGCACAC	60.035	GGCCGAGCGTCTAATAGCTT	59.968	267

TRINITY_Contig2581	1	c	(CCA)4ccg(CCT)4	27	127	153	TAAACCCTTTCGCCACCTCC	59.963	GTATCGGGAGGCTTTCGGAG	59.968	227
TRINITY_Contig2583	1	p3	(CCG)4	12	554	565	ATCACCTCCTCTCCTTCCCC	60.031	GCCCTGATTGCTCCTGTTCT	60.035	125
TRINITY_Contig2585	1	p3	(CCG)4	12	1322	1333	GCCGATCACGACCGTCTTAT	59.97	GTCTGGTCCTATCCATGGCG	59.966	167
TRINITY_Contig2588	1	c	(CGC)7cacgccaacgcccctcctacgcgatatgggcgcgcaggagacggcggtggaggaggtgctgcg(CGC)4	96	57	152	ATCGCAAAGCTGAACGAGGA	60.037	CGTAGGTTTGCCGCAACAAA	59.97	267
TRINITY_Contig2590	1	p3	(CTC)4	12	6217	6228	TCAGTTCTCCGCACATCCAC	60.037	CGCAGATCATGAAACAGCGG	59.973	153
TRINITY_Contig2591	1	p2	(CG)6	12	289	300	GTAGCCGAAGAGAAGGTGCA	59.753	GACATTCTGAGGGCTTGGCT	60.035	119
TRINITY_Contig2592	1	p3	(CTC)4	12	128	139	ATTCACCAGGGTGGCTGAAG	59.961	AGCTGCCTAGATGTGGGAGA	60.032	139
TRINITY_Contig2593	1	p2	(GT)6	12	409	420	CGCACACGCCTAATAAAGCC	59.972	TTTGTAACCTGCGCAAACCG	59.97	245
TRINITY_Contig2594	1	p3	(CCG)4	12	2581	2592	GTTCTGAAGGAGCCGTGTCA	59.966	GAGCAGAACGTGTGGAGAA	59.966	108
TRINITY_Contig2595	1	p4	(GCGG)3	12	5721	5732	TCTACATCCATGAGGGGGCA	60.03	GCGTGCTGTACAACTTGCAA	59.971	190
TRINITY_Contig2595	2	c	(GC)7gtgtgtg(TGTA)3	33	6009	6041	TCTACATCCATGAGGGGGCA	60.03	GCGTGCTGTACAACTTGCAA	59.971	190
TRINITY_Contig2595	3	p5	(GGGAC)5	25	6250	6274	TCTACATCCATGAGGGGGCA	60.03	GCGTGCTGTACAACTTGCAA	59.971	190
TRINITY_Contig2597	1	p5	(GACGC)4	20	522	541	GGAAAGGAGAAGTGCGTGGA	59.965	TCGGGCTCGGCTAATCTTTC	59.896	102
TRINITY_Contig2597	2	p3	(GCT)5	15	657	671	GGAAAGGAGAAGTGCGTGGA	59.965	TCGGGCTCGGCTAATCTTTC	59.896	102
TRINITY_Contig2600	1	p3	(GGA)4	12	56	67	CATGTGCTTCATGCTCGACG	59.974	CCTCCAGGGGTTATCCAGGA	60.03	268
TRINITY_Contig2600	2	p4	(CCAT)3	12	341	352	CATGTGCTTCATGCTCGACG	59.974	CCTCCAGGGGTTATCCAGGA	60.03	268
TRINITY_Contig2600	3	p3	(ACC)5	15	669	683	CATGTGCTTCATGCTCGACG	59.974	CCTCCAGGGGTTATCCAGGA	60.03	268
TRINITY_Contig2600	4	p3	(CTG)4	12	3367	3378	CATGTGCTTCATGCTCGACG	59.974	CCTCCAGGGGTTATCCAGGA	60.03	268
TRINITY_Contig2602	1	p3	(GGC)6	18	1719	1736	GGCAGGGGGATCAAATGGAA	60.032	TCAGCAGAGGAGGAGCTCAT	60.032	214
TRINITY_Contig2602	2	p2	(GA)16	32	1870	1901	GGCAGGGGGATCAAATGGAA	60.032	TCAGCAGAGGAGGAGCTCAT	60.032	214
TRINITY_Contig2604	1	c*	(TCAGC)4(TCAG<C>TCC)(CTCC)2aatgtgtctcacaaggcagagaggggaagaag(AGGA)3aaaagaccttttctgacc(ACAA)3	110	292	401	AGGAGCAAATCGTGTCCTGG	60.036	CTGAGCTGATGCACCTGGAA	60.036	242
TRINITY_Contig2604	2	c	(CTGCTT)3ctgcacctgcttgttgaggagattgagaacaataactagtctgctagtcaccaattgttggtagctcagttcagctct(TATG)4c(ATGTAT)4	137	524	660	AGGAGCAAATCGTGTCCTGG	60.036	CTGAGCTGATGCACCTGGAA	60.036	242
TRINITY_Contig2605	1	c	(CGC)4gccgcgcgacgaggagggggtaaaggggaatg(GCGA)3	54	1412	1465	CGGCGAGTATGAGTGAGGAC	59.97	TGTGTGTCAGAGAAGGACGC	59.968	107
TRINITY_Contig2606	1	p6	(GGAGCG)3	18	1508	1525	TTGCTCGGGTCCAAGAAGAC	59.965	GAGGAAGTGCGTGGAGAACA	59.966	221
TRINITY_Contig2606	2	p3	(GGA)4	12	1738	1749	TTGCTCGGGTCCAAGAAGAC	59.965	GAGGAAGTGCGTGGAGAACA	59.966	221
TRINITY_Contig2607	1	p3	(GCG)5	15	444	458	CCGCTGTGCCTATCCAAGAA	60.108	CTCTAATTCCGGGTCTCGCC	59.968	116
TRINITY_Contig2607	2	p3	(CCA)4	12	610	621	CCGCTGTGCCTATCCAAGAA	60.108	CTCTAATTCCGGGTCTCGCC	59.968	116
TRINITY_Contig2607	3	p3	(CGG)4	12	856	867	CCGCTGTGCCTATCCAAGAA	60.108	CTCTAATTCCGGGTCTCGCC	59.968	116
TRINITY_Contig2608	1	p4	(GTGC)3	12	278	289	GCCTTTCTGTGCGTTACTGC	60.11	GATCAGAGCCAGAGCAGCAA	60.108	279
TRINITY_Contig2609	1	p3	(AGC)4	12	1368	1379	AAAAGGTACTCGTCGCGGTT	59.967	CTGATGTTCAACGCCCTCCT	60.036	236
TRINITY_Contig2611	1	p3	(TGC)4	12	389	400	CTACCCTTCGCTTCCCTTGG	60.108	GTCGGATTTAGCGAGGGGAG	59.968	101
TRINITY_Contig2611	2	p5	(CCCGT)3	15	756	770	CTACCCTTCGCTTCCCTTGG	60.108	GTCGGATTTAGCGAGGGGAG	59.968	101
TRINITY_Contig2613	1	p4	(GGAC)4	16	271	286	AGCAGCGTCTCAAGAACTCC	60.038	GAACGAACAAGCCGCTGTTT	59.971	200
TRINITY_Contig2613	2	c	(CCT)7tttcctccaggatcgcacagcttccacccgctcccaggcccccgacagagaagccgcgggaggacgacgaaccgccgccgtcgtcgtc(GGA)5	124	685	808	AGCAGCGTCTCAAGAACTCC	60.038	GAACGAACAAGCCGCTGTTT	59.971	200
TRINITY_Contig2614	1	p4	(AGCT)3	12	1356	1367	CCTGGCTCCTGGAACTTCAG	60.036	CGGAGATGGTGGACTCATCG	59.969	147
TRINITY_Contig2615	1	p3	(CGC)5	15	235	249	GTAGTGACAGCCCCGTGAAA	59.966	TGCCGTGGGGTTAACTGATC	60.036	107
TRINITY_Contig2616	1	p3	(GCC)5	15	331	345	TCCACCCCCACGTATGATCT	60.032	GATCCGCCTTCTTCTCCTCG	59.969	275
TRINITY_Contig2616	2	p3	(CCG)4	12	659	670	TCCACCCCCACGTATGATCT	60.032	GATCCGCCTTCTTCTCCTCG	59.969	275
TRINITY_Contig2616	3	p3	(GCC)5	15	805	819	TCCACCCCCACGTATGATCT	60.032	GATCCGCCTTCTTCTCCTCG	59.969	275
TRINITY_Contig2617	1	p6	(CCGTCG)3	18	425	442	GACTGAAGCTGCAGACGTCT	60.039	ACAGAAGGAGCATGACTGGC	60.036	178
TRINITY_Contig2618	1	c	(GA)7aacaggccgagactgcggggaggacgagatggccgacggggagga(CGC)4	71	47	117	TCATGCTGCTGGTATTGCCA	60.034	CGAATTGCAGCCATACCAGC	59.97	123
TRINITY_Contig2619	1	p5	(AAGGA)3	15	11	25	CGGAGGGAGGAGGAAGAAGA	60.033	TTCAGGTCATTCTTCCCGCC	60.035	198
TRINITY_Contig2619	2	p4	(GGCG)3	12	448	459	CGGAGGGAGGAGGAAGAAGA	60.033	TTCAGGTCATTCTTCCCGCC	60.035	198
TRINITY_Contig2620	1	c	(TTGA)3aga(ATC)4	27	2098	2124	CGCTTGAAGATGCATCACCG	59.973	ATGCAGGATTTGGGATGCCA	60.032	104
TRINITY_Contig2621	1	p3	(TGG)5	15	699	713	TCTCGCCGAAGTTGCTTCTT	59.966	GCTTCACGCTCTGCAATAGC	59.973	121
TRINITY_Contig2621	2	p3	(TGA)4	12	6225	6236	TCTCGCCGAAGTTGCTTCTT	59.966	GCTTCACGCTCTGCAATAGC	59.973	121
TRINITY_Contig2624	1	p3	(GGC)4	12	304	315	ACTCAACAGCCCCATGATGG	60.034	AGGGAAAGGGGAGGCTACAT	59.954	236
TRINITY_Contig2627	1	p2	(CT)6	12	181	192	TGGTTGCGAGGGAGATGAAC	60.036	CGCATTCGAAGCCATCAGTG	59.973	119
TRINITY_Contig2628	1	p3	(GCC)4	12	2092	2103	ATGTGTATCTGGAGGGCCCT	60.03	CATTTCGAGGCCCACTCCTT	60.035	134
TRINITY_Contig2629	1	p3	(GGA)4	12	646	657	GGAGAATGAGATGGCGAGGG	59.965	CCGACCGGACAACTTTCTCA	59.967	200
TRINITY_Contig2631	1	p3	(GCT)4	12	1221	1232	GCTTCATCCCGTCGAGGTAG	59.969	AGGACGAGCAACAGGACAAG	59.966	153
TRINITY_Contig2631	2	p4	(CCCT)3	12	2630	2641	GCTTCATCCCGTCGAGGTAG	59.969	AGGACGAGCAACAGGACAAG	59.966	153
TRINITY_Contig2631	3	p3	(GCG)4	12	2763	2774	GCTTCATCCCGTCGAGGTAG	59.969	AGGACGAGCAACAGGACAAG	59.966	153
TRINITY_Contig2632	1	p3	(CGC)4	12	1328	1339	AGCCTCAAGGAAAGATGCCC	60.034	CGTTACGGTGTGGAACAGGA	59.968	280
TRINITY_Contig2634	1	p5	(TTTTA)3	15	977	991	GGGAAGCACTTTCGAAGGGA	59.964	CGGCTAATCCTGGCAACTGA	60.108	203
TRINITY_Contig2635	1	p3	(GGA)5	15	932	946	GCTCGCAACAAGAAAGCTCC	60.11	CATAGGGACGGCTGGACATC	59.966	200
TRINITY_Contig2636	1	p3	(CCG)4	12	1334	1345	GCAAAACAGACCACACGGAC	59.971	AAGCAGCACAGCAGAGAGAG	60.037	164
TRINITY_Contig2636	2	c	(GAG)4ggatggtttagcgtgacgggccgggggaggagggag(GAGC)3	60	1473	1532	GCAAAACAGACCACACGGAC	59.971	AAGCAGCACAGCAGAGAGAG	60.037	164
TRINITY_Contig2636	3	c	(TGCC)3tgcgagcgtaggtccagggatagcaggtcgtacagttggctggtagtaacaacttgggatcggattcactgaccattcatggattgattgagttggttg(CTTTC)3	127	1672	1798	GCAAAACAGACCACACGGAC	59.971	AAGCAGCACAGCAGAGAGAG	60.037	164
TRINITY_Contig2636	4	p3	(GCG)5	15	1946	1960	GCAAAACAGACCACACGGAC	59.971	AAGCAGCACAGCAGAGAGAG	60.037	164
TRINITY_Contig2636	5	p3	(TGT)4	12	3152	3163	GCAAAACAGACCACACGGAC	59.971	AAGCAGCACAGCAGAGAGAG	60.037	164
TRINITY_Contig2637	1	p5	(CGCCC)3	15	362	376	CGCTTGGTTTTTGAGTGGCA	59.898	AGCACCACTGGTCCAAGATG	59.963	246
TRINITY_Contig2638	1	p3	(GCG)4	12	4616	4627	CAACCCAACTTCACAGCAGC	59.969	GACTTGGATGGAGGGTGGTG	60.035	103
TRINITY_Contig2642	1	p4	(TCCC)3	12	26	37	GGAGGCAAGCTGGAGTTGAT	60.035	GAAAGTCGTGTCCCAGAGCA	59.966	268
TRINITY_Contig2642	2	p3	(CCG)5	15	173	187	GGAGGCAAGCTGGAGTTGAT	60.035	GAAAGTCGTGTCCCAGAGCA	59.966	268
TRINITY_Contig2642	3	p3	(CCG)5	15	342	356	GGAGGCAAGCTGGAGTTGAT	60.035	GAAAGTCGTGTCCCAGAGCA	59.966	268
TRINITY_Contig2644	1	c	(TC)7ctttccccactagccgccccg(CCCCT)3	50	265	314	TGGTGCTTCATCGAATGGCT	60.035	GCCTTCGGGTTTTCAGAGGA	59.964	192
TRINITY_Contig2646	1	p3	(TTC)4	12	37	48	AACTGCAGAAAAGCGTGCTG	59.97	GCGTCTCTTCCAACCAGACA	59.966	206
TRINITY_Contig2646	2	c	(TCC)4tctaagggaggagcagca(GCT)4	42	224	265	AACTGCAGAAAAGCGTGCTG	59.97	GCGTCTCTTCCAACCAGACA	59.966	206
TRINITY_Contig2647	1	p5	(TGCAT)3	15	43	57	GGCCGAATCCATCTCGTTCT	59.895	ATGTTTTCGACAAGGCGTGC	60.04	122
TRINITY_Contig2647	2	p3	(GGC)4	12	1130	1141	GGCCGAATCCATCTCGTTCT	59.895	ATGTTTTCGACAAGGCGTGC	60.04	122
TRINITY_Contig2648	1	p4	(ATAG)3	12	164	175	AGTCTCGAGGAGTCGGTTGA	59.964	CCTTGCTGTGTCCAATGTGC	60.039	187
TRINITY_Contig2649	1	p3	(AGC)5	15	532	546	CGAATCTGCGGCGAGAAAAG	59.974	CAAAAAGTTGGGGACGCCTG	59.968	214
TRINITY_Contig2649	2	p3	(CGC)5	15	1800	1814	CGAATCTGCGGCGAGAAAAG	59.974	CAAAAAGTTGGGGACGCCTG	59.968	214
TRINITY_Contig2649	3	p2	(GA)6	12	2134	2145	CGAATCTGCGGCGAGAAAAG	59.974	CAAAAAGTTGGGGACGCCTG	59.968	214
TRINITY_Contig2650	1	p3	(GCG)4	12	983	994	TCCCTGGCCTACGGTATTGA	60.032	ACCAACTGAATGAGCGCTCA	59.965	177
TRINITY_Contig2650	2	p5	(GCCGC)4	20	1648	1667	TCCCTGGCCTACGGTATTGA	60.032	ACCAACTGAATGAGCGCTCA	59.965	177
TRINITY_Contig2650	3	p4	(TTCT)4	16	3086	3101	TCCCTGGCCTACGGTATTGA	60.032	ACCAACTGAATGAGCGCTCA	59.965	177
TRINITY_Contig2651	1	p3	(ATG)4	12	1084	1095	CAATCCCTCTCGATGCCCTC	59.965	TCCGCCAACCCTAAATTCCC	60.034	201
TRINITY_Contig2651	2	c	(CTC)4ttcatctaa(CTC)5	36	1331	1366	CAATCCCTCTCGATGCCCTC	59.965	TCCGCCAACCCTAAATTCCC	60.034	201
TRINITY_Contig2654	1	p3	(GCG)4	12	3101	3112	CACCGAGATCTACACCGACG	59.972	GTGAGTATCGGAAGGAGGCG	59.969	149
TRINITY_Contig2656	1	p3	(GCT)5	15	309	323	TTGGGCATGGCTAGGAAGTG	60.034	GCTCCATAGTAGGCAGCTGG	59.965	227
TRINITY_Contig2656	2	p4	(CATG)3	12	1367	1378	TTGGGCATGGCTAGGAAGTG	60.034	GCTCCATAGTAGGCAGCTGG	59.965	227
TRINITY_Contig2658	1	p3	(TCC)4	12	215	226	GAGTTCGGCATCTCGGTAGG	59.969	TTGTGCATCTTCGACCGACA	59.967	172
TRINITY_Contig2661	1	p4	(CCGC)3	12	3	14	GTAGATCCTGGGTGCGCAAT	60.179	CCTTCTGTAACCCAGCACGT	59.965	252
TRINITY_Contig2662	1	p3	(ACC)4	12	1227	1238	CGTATGAGGTCGTCAGTCCG	59.972	GGCTGAACTTCTCCACCTCC	60.036	147
TRINITY_Contig2666	1	c	(TC)6aaaaaaaag(GAA)4	33	36	68	CTCTTCTCTACTGCTCCGCG	59.971	TTGTCAACCTCTTCGGACGG	59.967	230
TRINITY_Contig2668	1	p3	(GAT)4	12	896	907	CTTCTCCCTACCTGCCTCCT	60.032	AAGTGGCTCAGAATCAGGGC	60.035	202
TRINITY_Contig2669	1	c	(CGC)6gcgagaggtgctgcgcgcagcgcgc(CCG)5	57	1164	1220	GATGATCCGCTGCTGCAAAG	59.971	TATCCCATCACCTGTGCTGC	59.819	250
TRINITY_Contig2671	1	c	(GCG)5ggagcgaggagaagaggcgcccgccggcgacccccggcgtccggggcgc(AGG)5	79	1296	1374	CGGCACCTTTCTGCAACTTC	60.04	TCCAGTCCCGAAGAGAGGTT	59.885	150
TRINITY_Contig2671	2	p4	(GGGC)3	12	1531	1542	CGGCACCTTTCTGCAACTTC	60.04	TCCAGTCCCGAAGAGAGGTT	59.885	150
TRINITY_Contig2671	3	p3	(CGC)4	12	1714	1725	CGGCACCTTTCTGCAACTTC	60.04	TCCAGTCCCGAAGAGAGGTT	59.885	150
TRINITY_Contig2673	1	p3	(TGA)4	12	3824	3835	TCAGCTGCGTGACTTGCTTA	59.967	CGGGCAACAACACAGTTCAG	59.971	127

TRINITY_Contig2674	1	p3	(GCG)5	15	1725	1739	CACATGTCGCCAAGAGCATG	59.902	CCTTGGGTACTCTGGCCTTG	60.035	212
TRINITY_Contig2675	1	p3	(CAT)4	12	421	432	CTCACAGCAGCGGTCAAGTA	60.038	TCCTGACAACATGGAGCACC	59.963	208
TRINITY_Contig2676	1	c	(AGC)4gcagccgagcgccttccccatcatgctcagtcaagtccaagcgcacgccgccggcctcgtccctggtctctctgcct(GGCC)4	105	1868	1972	TGGTCTGCAGTTACGTCACC	59.967	CAGGTTGAAGGTGCAGTTGC	59.969	110
TRINITY_Contig2677	1	p2	(TC)9	18	47	64	TTCAGCCGTCACTGCATTCT	59.965	AACGTAGTGACAGCCATGGG	60.036	237
TRINITY_Contig2677	2	p3	(CTG)4	12	658	669	TTCAGCCGTCACTGCATTCT	59.965	AACGTAGTGACAGCCATGGG	60.036	237
TRINITY_Contig2679	1	p3	(GAG)4	12	635	646	AGGCCAATGTGAGCTCTGAC	60.036	CCACGCTGATGCTGTATTGC	59.971	228
TRINITY_Contig2680	1	p4	(AAAT)3	12	1736	1747	GACTAGCAGGGCTGTCGATC	59.969	TGTGGATTTTGGTGCTTGCG	59.968	253
TRINITY_Contig2680	2	p3	(GCC)9	27	4150	4176	GACTAGCAGGGCTGTCGATC	59.969	TGTGGATTTTGGTGCTTGCG	59.968	253
TRINITY_Contig2680	3	p3	(CCG)5	15	4280	4294	GACTAGCAGGGCTGTCGATC	59.969	TGTGGATTTTGGTGCTTGCG	59.968	253
TRINITY_Contig2680	4	p2	(TG)6	12	5635	5646	GACTAGCAGGGCTGTCGATC	59.969	TGTGGATTTTGGTGCTTGCG	59.968	253
TRINITY_Contig2680	5	c	(GGA)5gaagcagtggagaggaggagatggaggagcctatctaagaaggttgttgtctgatttctttcgcatccttgtcatacttt(TC)9	113	6005	6117	GACTAGCAGGGCTGTCGATC	59.969	TGTGGATTTTGGTGCTTGCG	59.968	253
TRINITY_Contig2682	1	p4	(GCGA)3	12	1005	1016	CGTATGCGGCGAAAGAAAGG	59.974	AAGATGCAGAAGGAGCACCC	60.035	237
TRINITY_Contig2684	1	p3	(CGC)5	15	1057	1071	TCTCGCAACTGCATCCATGT	60.036	GCAGCCAGAAAGGAGCAAAC	60.039	264
TRINITY_Contig2686	1	p3	(CGG)4	12	1130	1141	GAGAGGTGGAGGTCCCTCAT	60.032	GTGGAGACGAAAGCCAGGAA	59.965	181
TRINITY_Contig2687	1	p6	(TGCCGA)3	18	156	173	CCCTCCCTTTCCTCTAGGCT	60.031	ATCACCCCAAAGCTCACGAG	60.036	143
TRINITY_Contig2687	2	p3	(GCC)4	12	437	448	CCCTCCCTTTCCTCTAGGCT	60.031	ATCACCCCAAAGCTCACGAG	60.036	143
TRINITY_Contig2688	1	p3	(GCT)4	12	180	191	AGTTCGGCTTCAACCTCCTG	59.965	CACATCATGCGCGTCTTCTG	59.974	175
TRINITY_Contig2688	2	c*	(GCCACC)3(G<CCACC>ACCACCAGCAGCAGCCATGGCCTCCTCCGCGTCCCTGCAGCGCTTCCTCCCTCCCTCCGCGCACGCGGCGTCGTCGTCCTCGTCCCGCCGGCGCGCCGGCCGCGCCCGGGCGGCCGTCTCCGTGCCGTCGTCCCAGGCGGCGGCGGGCGAGGTCCCCGCGGAGCGGCTGGAGCCGAGGGTCGAGCAGCGGGAGGGAGGGTACTGGGTGCTCAAGGAGAAGTACCGCACGGGGCTGAACCCTCAGGAGAAGGTGAAGCTGGAGAAGGAGCCCATGGGGCTGTTCATGGAGGGCGGCATCAAGGACCTCGCCAAGATCCCCATGGAGGAGATCGACGCCGCCAAGCTCACCAAGGACGACGTCGACGTACGCCTCAAGTGGCTCGGCCTCTTCCACCGCCGCAAGCACCAGTACGGGCGGT	81	416	496	AGTTCGGCTTCAACCTCCTG	59.965	CACATCATGCGCGTCTTCTG	59.974	175
TRINITY_Contig2689	1	p4	(TTGT)3	12	780	791	GGGGTCATTTTCCCACCGAT	60.034	AGCCATCTCTCTCCCCCAAT	60.029	192
TRINITY_Contig2692	1	p3	(CCT)10	30	219	248	GCCTAACGTAGAGCGGTCTC	59.971	CAGTTTCCGTTTGGTGGCAG	59.969	277
TRINITY_Contig2692	2	p3	(CGC)4	12	494	505	GCCTAACGTAGAGCGGTCTC	59.971	CAGTTTCCGTTTGGTGGCAG	59.969	277
TRINITY_Contig2692	3	p5	(CTCTG)3	15	681	695	GCCTAACGTAGAGCGGTCTC	59.971	CAGTTTCCGTTTGGTGGCAG	59.969	277
TRINITY_Contig2692	4	c	(GATG)3gtgccatggtttcgcggcgggcaagtca(CCTCC)3	55	2234	2288	GCCTAACGTAGAGCGGTCTC	59.971	CAGTTTCCGTTTGGTGGCAG	59.969	277
TRINITY_Contig2696	1	p3	(GTC)4	12	557	568	CTCATTCCTCTCCCGGAAGC	59.894	CGGCAGGGTCTATGTGCTAG	59.967	120
TRINITY_Contig2696	2	p3	(GGC)4	12	721	732	CTCATTCCTCTCCCGGAAGC	59.894	CGGCAGGGTCTATGTGCTAG	59.967	120
TRINITY_Contig2697	1	p3	(CGC)4	12	166	177	TCCATCCACATTTGCCCCTC	60.033	CAGCTTCACAAATCCCGCAC	60.11	127
TRINITY_Contig2698	1	c	(GCGA)3cggcatgggtagcaccagcaacagg(GGC)5	52	202	253	GGTACTACATCCTGGTCGCG	59.97	CCCGTCTTGAAGTCCTGCAT	60.036	235
TRINITY_Contig2700	1	p3	(CCG)5	15	126	140	AGAGCGAGTTCACAACCCTG	59.966	TATGGCGTTCCACCTCCAAC	60.036	172
TRINITY_Contig2701	1	p4	(CGGC)3	12	171	182	CTGACAACACAACAGCCGTG	59.972	GATCAGGATATGTGCCGGCA	59.965	202
TRINITY_Contig2701	2	p3	(AGG)5	15	3182	3196	CTGACAACACAACAGCCGTG	59.972	GATCAGGATATGTGCCGGCA	59.965	202
TRINITY_Contig2702	1	p3	(GGA)4	12	84	95	AGGAACATCGTAGCCTCCCT	60.032	TCGATCTTCGGAACCCTCCT	60.033	110
TRINITY_Contig2703	1	p6	(GCTCGC)3	18	150	167	TCAAGGAGAGGTACCCGAGG	60.033	TCAGATCTACCGCCTGGGAA	60.033	165
TRINITY_Contig2703	2	p3	(AGG)6	18	378	395	TCAAGGAGAGGTACCCGAGG	60.033	TCAGATCTACCGCCTGGGAA	60.033	165
TRINITY_Contig2704	1	p4	(CCAA)3	12	98	109	ACACTTCACCTGCAGAGTCG	59.967	TGACTAACTAGAGGCCGGCT	60.033	253
TRINITY_Contig2704	2	p4	(ATCA)3	12	3598	3609	ACACTTCACCTGCAGAGTCG	59.967	TGACTAACTAGAGGCCGGCT	60.033	253
TRINITY_Contig2706	1	p4	(CTCC)3	12	36	47	TCCTCCAGTGCACGAGAAAC	59.966	GAAGCTCCTGATGGCAACCT	60.035	116
TRINITY_Contig2707	1	p5	(CCACG)3	15	219	233	TGGGTTGCAGAAGGTTGGTT	60.033	GGCCGTGGGTTTTCTAGTCA	59.964	248
TRINITY_Contig2708	1	p4	(AAAC)3	12	2296	2307	AACACCTAGAGGATCGGGCT	60.032	AGGGGCAGTTCGAAGTCTTG	59.965	179
TRINITY_Contig2709	1	p5	(GGCGC)3	15	981	995	GCCGGATCATGCCTCGATAA	60.037	GTTTTGGACGGCCAGGTTTC	59.968	188
TRINITY_Contig2709	2	p2	(GA)16	32	1928	1959	GCCGGATCATGCCTCGATAA	60.037	GTTTTGGACGGCCAGGTTTC	59.968	188
TRINITY_Contig2710	1	c	(AGGCAG)3gaggcaggcgca(GCG)4acatctgcatctgcatctccgccccgtccaccctgcatctcatctg(CTGCT)3	103	18	120	CTCATCCAGTACGACCTCGC	59.97	AGCAGAATGAGCAACCCTCC	60.035	200
TRINITY_Contig2710	2	p3	(CGC)4	12	242	253	CTCATCCAGTACGACCTCGC	59.97	AGCAGAATGAGCAACCCTCC	60.035	200
TRINITY_Contig2712	1	p3	(GAC)4	12	3659	3670	ACGATGTCAGGGGAGCTTTG	60.036	GCAAAGCTGCGACTTGGAAA	59.969	230
TRINITY_Contig2714	1	p3	(CCA)4	12	57	68	TGATGTCAATGGTGGGAGGC	60.034	CGGTAGGAACAAGGACACCC	60.037	164
TRINITY_Contig2714	2	p6	(GCAACG)3	18	464	481	TGATGTCAATGGTGGGAGGC	60.034	CGGTAGGAACAAGGACACCC	60.037	164
TRINITY_Contig2715	1	p3	(CGC)4	12	991	1002	GGAAGGACTCAGTGACGTGG	60.038	CCATCTCCATCCGCATCCTC	60.037	116
TRINITY_Contig2715	2	p3	(GCC)4	12	1378	1389	GGAAGGACTCAGTGACGTGG	60.038	CCATCTCCATCCGCATCCTC	60.037	116
TRINITY_Contig2717	1	c	(CAAG)3ccgctcccacccccactcccggagatgatgagg(GGC)4gagtac(CAG)5	79	551	629	GTCGGGCTCATCATCGTCAT	59.967	GCGACGTCCTGTAGTAGAGC	59.972	175
TRINITY_Contig2719	1	c	(GTG)4gtcgacggccttcatagctccggggacgcggtaggagtggcagcgggggaaggagtggactgtcg(ACC)4	89	750	838	TTCCACGACTGCTCAACCTC	59.966	GATCCGCTCATCGACGAACT	59.97	239
TRINITY_Contig2720	1	p3	(CGC)5	15	740	754	GCTTCTCCTTGACCTCACCC	60.036	AGCATCTGAGGCCAAATGCT	60.033	278
TRINITY_Contig2722	1	p3	(CGG)5	15	1883	1897	TCCGTATCGAGGTCCCTTGT	60.034	CGCTGAGATCCTGGGAATCC	59.965	100
TRINITY_Contig2724	1	p3	(GCT)4	12	1244	1255	GTCGCACCGTAAATTCGAGC	59.975	AGTTTGGAGCCAGGCTTCTC	59.963	198
TRINITY_Contig2725	1	c	(CCTC)3tcctctccgccccgcccagcgctaccctaggccgcggcccgcgccccccttaccgccgcacgatgagctcctccccagccgcagccgcgt(CGC)4	114	207	320	TGATCTGGAACTGGTGGTGC	59.963	ACCACGCAGTTCACGGTTAT	59.966	105
TRINITY_Contig2727	1	p3	(CTG)4	12	587	598	CAGATGCAGCGTGTATTGCC	59.971	AAGGATGAGGAGACCGCTCT	60.033	247
TRINITY_Contig2728	1	p4	(CCTC)3	12	1310	1321	GTGTAGCCGTGCAACTTTGG	60.04	AGGAAGCAAGGTCGCTGTAC	60.037	204
TRINITY_Contig2728	2	p4	(CCCG)3	12	1516	1527	GTGTAGCCGTGCAACTTTGG	60.04	AGGAAGCAAGGTCGCTGTAC	60.037	204
TRINITY_Contig2729	1	p2	(AG)6	12	148	159	CGTCGCTCGGAGACATCTAC	60.042	TGTATTCATTGCTGTGCGCG	59.903	138
TRINITY_Contig2729	2	c	(CGC)5(GAG)4	27	287	313	CGTCGCTCGGAGACATCTAC	60.042	TGTATTCATTGCTGTGCGCG	59.903	138
TRINITY_Contig2729	3	p3	(CGA)5	15	611	625	CGTCGCTCGGAGACATCTAC	60.042	TGTATTCATTGCTGTGCGCG	59.903	138
TRINITY_Contig2730	1	p6	(CTGAAT)3	18	2457	2474	AAATTCTTGCGCTAGGGCCT	60.034	TGGACAAGACGTGCTGACTC	59.968	264
TRINITY_Contig2730	2	p3	(GCC)5	15	3272	3286	AAATTCTTGCGCTAGGGCCT	60.034	TGGACAAGACGTGCTGACTC	59.968	264
TRINITY_Contig2731	1	p3	(CGG)4	12	1533	1544	CATTGAACACCTCCAAGCGC	60.11	TGAAAGTTTTCGGCGCACTG	59.971	222
TRINITY_Contig2736	1	p3	(GCG)4	12	41	52	CTCACCCTGCCATGTTCTGT	59.963	CTGGAACTGGCCACAAATGC	60.038	198
TRINITY_Contig2736	2	p4	(TCTA)3	12	915	926	CTCACCCTGCCATGTTCTGT	59.963	CTGGAACTGGCCACAAATGC	60.038	198
TRINITY_Contig2737	1	p5	(GAGCA)3	15	87	101	TCTTGATCACCATGGTGCCC	60.034	CATCCGTGTGGTGTCACTCA	59.967	248
TRINITY_Contig2738	1	p2	(CT)6	12	210	221	TGATACTCCGCCAAGGGAGA	60.033	TTGTTGCTGTTTGATGCCCG	59.968	195
TRINITY_Contig2739	1	p3	(CCG)4	12	2153	2164	TTCTGCAGTGCAAAGAACGC	59.971	ACCGTTGCGAGGACATTTCT	59.965	110
TRINITY_Contig2741	1	p3	(CGC)8	24	735	758	TCTCTTGTGCGGTTGGTCTC	59.966	ACCAGACTTGCCCTCCAATG	59.961	121
TRINITY_Contig2741	2	p3	(GGC)4	12	1334	1345	TCTCTTGTGCGGTTGGTCTC	59.966	ACCAGACTTGCCCTCCAATG	59.961	121
TRINITY_Contig2741	3	p3	(CTG)6	18	3385	3402	TCTCTTGTGCGGTTGGTCTC	59.966	ACCAGACTTGCCCTCCAATG	59.961	121
TRINITY_Contig2741	4	p3	(CTC)5	15	3823	3837	TCTCTTGTGCGGTTGGTCTC	59.966	ACCAGACTTGCCCTCCAATG	59.961	121
TRINITY_Contig2741	5	p2	(GA)12	24	3969	3992	TCTCTTGTGCGGTTGGTCTC	59.966	ACCAGACTTGCCCTCCAATG	59.961	121
TRINITY_Contig2745	1	p3	(GAG)6	18	1666	1683	CGACCCTTTGAGCTCCTTGT	59.965	GTCTACGCCTCCATTAGGGC	59.966	189
TRINITY_Contig2749	1	p4	(CCCG)3	12	529	540	CCTACGAGACTCACATCGCC	59.97	GGGCCACGATGATGATCAGT	59.893	164
TRINITY_Contig2750	1	p3	(GAA)4	12	2262	2273	AACAGCTCCGAGACTGGTTG	59.966	CGCAGTACTGTCCCAACTGT	59.966	172
TRINITY_Contig2752	1	p2	(GA)9	18	1231	1248	GAGGATCTTGAGCTCGACCG	59.97	TTCATGTTTACGGGCCTCCC	60.035	138
TRINITY_Contig2753	1	c	(ACC)4(GCC)4	24	214	237	TCGGAGGTGACCATCAGGAT	60.032	CCACCAGATCCTAGTCCCGA	60.106	269
TRINITY_Contig2754	1	p3	(GCC)5	15	2044	2058	CTGCAAGAAGCCTCCATGGA	60.035	CGTCATTGGTTGGCTTGCAA	59.968	236
TRINITY_Contig2758	1	p3	(CCA)5	15	2571	2585	TACCTGGGGCAGTATTCCGA	60.032	TCTATCAGCTGGAGGCCACT	60.032	171
TRINITY_Contig2758	2	p6	(CCACCG)3	18	2700	2717	TACCTGGGGCAGTATTCCGA	60.032	TCTATCAGCTGGAGGCCACT	60.032	171
TRINITY_Contig2758	3	p3	(ACC)4	12	3344	3355	TACCTGGGGCAGTATTCCGA	60.032	TCTATCAGCTGGAGGCCACT	60.032	171
TRINITY_Contig2759	1	p3	(CCG)4	12	627	638	CAATGCAACGTGGAGCAACA	59.969	GCCTTGGCTTCCTCAGACAT	60.035	243
TRINITY_Contig2760	1	p5	(TAGTC)3	15	643	657	GGAGCTTGGAGGTCACCTTC	60.036	GATGTCTCGCCGCTATGACA	59.969	167
TRINITY_Contig2761	1	p3	(GCG)4	12	781	792	TGATCAACCATTCGCAACGC	59.832	GCGTCCTGCCACTGTACATA	59.825	180

TRINITY_Contig2765	1	p3	(CCA)6	18	322	339	CGTTAGCAAATCGAGGCGTG	59.975	GGCGGTTATTGCATGAGCTG	59.97	136
TRINITY_Contig2766	1	p3	(CCG)4	12	95	106	TTGTAGCTTCAGCGGCTGAA	59.965	ATGAAGTTAGCCCTTGGGCC	60.033	143
TRINITY_Contig2767	1	p5	(CGGCT)3	15	1155	1169	CTTCTCTCAAGGCAGCGTCA	60.038	CATCGTCTCGCCAGTACCTC	59.97	146
TRINITY_Contig2767	2	p3	(CCG)4	12	1977	1988	CTTCTCTCAAGGCAGCGTCA	60.038	CATCGTCTCGCCAGTACCTC	59.97	146
TRINITY_Contig2768	1	p3	(GCG)4	12	73	84	CAGCAGTGCTACAAGGGACA	59.965	ATCGCTGGATTTGGCTTGGA	60.034	134
TRINITY_Contig2769	1	p3	(GCG)6	18	2796	2813	ACGTAGGCATGACCCCAAAG	60.035	TTGCGGCTGACATTGTTTGG	59.968	197
TRINITY_Contig2770	1	p2	(AG)9	18	562	579	CACCGACCCACAAAGCTTTG	59.969	CAACAACCCACAAAGCCTCG	59.969	167
TRINITY_Contig2772	1	p4	(AGGC)3	12	14	25	CGTCTGAAACCTTCGTGGGA	59.967	CTCTCAGTATCGCGTTCCCC	59.969	236
TRINITY_Contig2773	1	p3	(CCT)5	15	2943	2957	CACAAAGTGCACCCTGAAGC	59.969	CTTGGGCCAGTTTCTGCAAC	59.967	136
TRINITY_Contig2774	1	p4	(ACCG)3	12	236	247	GCGGATCAAGTGGTTCGAGA	60.109	GTCAGCTTCGAAGAGGTGCT	60.038	267
TRINITY_Contig2774	2	p3	(CCT)4	12	732	743	GCGGATCAAGTGGTTCGAGA	60.109	GTCAGCTTCGAAGAGGTGCT	60.038	267
TRINITY_Contig2774	3	p3	(CCG)4	12	1095	1106	GCGGATCAAGTGGTTCGAGA	60.109	GTCAGCTTCGAAGAGGTGCT	60.038	267
TRINITY_Contig2783	1	p3	(CGC)6	18	121	138	CTGTTCAGGGATCACTGCGT	60.037	CCATCTGCACCCACACTGAT	60.035	221
TRINITY_Contig2783	2	p3	(CGC)6	18	532	549	CTGTTCAGGGATCACTGCGT	60.037	CCATCTGCACCCACACTGAT	60.035	221
TRINITY_Contig2784	1	p6	(GACAAG)3	18	166	183	AAGACCGCCAAGGAGTGAAG	59.965	CACGACACGATACAGACGGT	59.833	128
TRINITY_Contig2785	1	p3	(CTT)4	12	3	14	AGCAGAAGAATGTCTCCGCC	60.108	ATGAAGCTGCTCTCCAACCC	60.035	157
TRINITY_Contig2785	2	c	(GGC)4ttgctgtaggcggcggcgggggact tggtcttcttgttg(TTC)4	63	558	620	AGCAGAAGAATGTCTCCGCC	60.108	ATGAAGCTGCTCTCCAACCC	60.035	157
TRINITY_Contig2785	3	p3	(CGT)4	12	772	783	AGCAGAAGAATGTCTCCGCC	60.108	ATGAAGCTGCTCTCCAACCC	60.035	157
TRINITY_Contig2785	4	p3	(CCG)4	12	1293	1304	AGCAGAAGAATGTCTCCGCC	60.108	ATGAAGCTGCTCTCCAACCC	60.035	157
TRINITY_Contig2787	1	p3	(GCC)7	21	178	198	CCCTCGTCCTCACCCTAAGA	60.033	GGCAAGCAAAACAGCATCCA	59.966	218
TRINITY_Contig2788	1	p6	(AGCGGC)3	18	2926	2943	ATCTGCCCAGAGTGGAGGAT	60.03	GCAACAGTGGATTGCAGCAA	59.968	186
TRINITY_Contig2789	1	p3	(CAG)4	12	157	168	ATACGCACGGCCATGATCAT	59.965	AGATGCGTCGACGAAAAGGT	60.039	272
TRINITY_Contig2789	2	p4	(GGAG)3	12	325	336	ATACGCACGGCCATGATCAT	59.965	AGATGCGTCGACGAAAAGGT	60.039	272
TRINITY_Contig2789	3	p4	(TGGA)3	12	783	794	ATACGCACGGCCATGATCAT	59.965	AGATGCGTCGACGAAAAGGT	60.039	272
TRINITY_Contig2789	4	p4	(AACC)4	16	990	1005	ATACGCACGGCCATGATCAT	59.965	AGATGCGTCGACGAAAAGGT	60.039	272
TRINITY_Contig2789	5	p4	(GCCC)3	12	1776	1787	ATACGCACGGCCATGATCAT	59.965	AGATGCGTCGACGAAAAGGT	60.039	272
TRINITY_Contig2790	1	p4	(GGGC)3	12	260	271	GCGGACATGCTTAGATCGGA	59.968	TCCAAAGCGTGATCAACCGA	59.966	220
TRINITY_Contig2790	2	p3	(GCC)6	18	1235	1252	GCGGACATGCTTAGATCGGA	59.968	TCCAAAGCGTGATCAACCGA	59.966	220
TRINITY_Contig2790	3	p3	(TTC)4	12	1605	1616	GCGGACATGCTTAGATCGGA	59.968	TCCAAAGCGTGATCAACCGA	59.966	220
TRINITY_Contig2792	1	p3	(GCG)4	12	1104	1115	GGGTGCAAGCAAAGCTACAC	60.039	CACGGCTAGTCAAAGGCTGA	60.037	189
TRINITY_Contig2792	2	p3	(GCG)4	12	1263	1274	GGGTGCAAGCAAAGCTACAC	60.039	CACGGCTAGTCAAAGGCTGA	60.037	189
TRINITY_Contig2792	3	p5	(CGGCA)3	15	1464	1478	GGGTGCAAGCAAAGCTACAC	60.039	CACGGCTAGTCAAAGGCTGA	60.037	189
TRINITY_Contig2795	1	p2	(CT)7	14	20	33	GAACGTCAGGACACTGCAGA	59.968	TATGACCAGCAAGCCCAGTG	60.035	222
TRINITY_Contig2796	1	c	(GCC)4gcggccat(CTG)5gtgctgcattgg ttgcat(CTG)4	65	2139	2203	TTGAACAAAAGCTCCGCACG	59.971	CCCCGACAAAGCAAAAGTGG	59.968	243
TRINITY_Contig2796	2	p3	(GCT)6	18	2362	2379	TTGAACAAAAGCTCCGCACG	59.971	CCCCGACAAAGCAAAAGTGG	59.968	243
TRINITY_Contig2796	3	p3	(TGC)5	15	2484	2498	TTGAACAAAAGCTCCGCACG	59.971	CCCCGACAAAGCAAAAGTGG	59.968	243
TRINITY_Contig2796	4	p3	(CCG)4	12	4531	4542	TTGAACAAAAGCTCCGCACG	59.971	CCCCGACAAAGCAAAAGTGG	59.968	243
TRINITY_Contig2798	1	p3	(CCG)4	12	423	434	TGGTTGAACCTTTTGCCCCT	60.032	CGCACAAGGAAGAGGAGTGT	59.966	241
TRINITY_Contig2800	1	p5	(CGCTG)3	15	2462	2476	CGATTATGGACCCGCCGTTA	59.968	GCTCAGTTGGTGCAGTGTTG	59.97	270
TRINITY_Contig2801	1	p3	(CGG)4	12	1198	1209	CGACGATGAGGAAGAGGACG	59.972	TCTGGGTCTCCCTCCTCATG	60.032	138
TRINITY_Contig2801	2	p4	(GGCG)4	16	1797	1812	CGACGATGAGGAAGAGGACG	59.972	TCTGGGTCTCCCTCCTCATG	60.032	138
TRINITY_Contig2804	1	p3	(GGC)5	15	80	94	GCGCGCTGGAATCAATCAAT	59.97	CTTTGCCTGCCAAAACTCCC	59.966	254
TRINITY_Contig2804	2	p3	(CGT)4	12	968	979	GCGCGCTGGAATCAATCAAT	59.97	CTTTGCCTGCCAAAACTCCC	59.966	254
TRINITY_Contig2804	3	p4	(AATC)3	12	1096	1107	GCGCGCTGGAATCAATCAAT	59.97	CTTTGCCTGCCAAAACTCCC	59.966	254
TRINITY_Contig2804	4	p3	(GCG)4	12	1554	1565	GCGCGCTGGAATCAATCAAT	59.97	CTTTGCCTGCCAAAACTCCC	59.966	254
TRINITY_Contig2805	1	p3	(TTG)4	12	3064	3075	TGCTGCGTTCAAAGTGTTCG	59.973	AATCACAAGCAGCCCATCGA	60.035	253
TRINITY_Contig2806	1	p3	(GAA)4	12	4095	4106	ACGAAACAGCGAAACTTGCC	59.971	ACACGAATCGGATCGAGAGC	59.97	162
TRINITY_Contig2806	2	p4	(CTGG)3	12	7964	7975	ACGAAACAGCGAAACTTGCC	59.971	ACACGAATCGGATCGAGAGC	59.97	162
TRINITY_Contig2807	1	p3	(CGC)4	12	486	497	AGGTTCGACTCAACTGGCAG	59.966	CCGGCGGTAGAACAAGGTAG	60.179	198
TRINITY_Contig2808	1	p3	(CAA)4	12	1560	1571	AGCAGAAGGATGGTCACTGC	60.036	TATCTGTTGGGCCCAAGCTG	60.034	136
TRINITY_Contig2809	1	p3	(CGC)4	12	561	572	AGGCTGAGGAACGGGTAGAT	60.032	GCCCTTCCTGTCGTCTTTCA	59.965	273
TRINITY_Contig2810	1	p3	(CAC)6	18	98	115	GTTGGAGCTGATCTGGCTGT	60.036	GGTTGCAATTTCCCACGCTT	59.967	141
TRINITY_Contig2811	1	c	(CCG)4ccctccccccagcgagccagccatgg cggagatcgccgagcgggccaccgcgggcga gctgccggaggagccgcggccgccgcccggg gg(GGA)4c(GAG)6	133	300	432	GGAAAGCCCCCTGCCTTTAT	60.032	ACGCGCTTAAGATCGAGAGG	59.97	211
TRINITY_Contig2813	1	p3	(CGT)4	12	1657	1668	TGGGGCGCTTGTACATTGAT	60.035	TTCTTCAGGATTCCGGCCAC	60.035	184
TRINITY_Contig2813	2	p4	(GCAG)3	12	1841	1852	TGGGGCGCTTGTACATTGAT	60.035	TTCTTCAGGATTCCGGCCAC	60.035	184
TRINITY_Contig2815	1	p3	(TCA)4	12	71	82	ATTTGGTGCGTGACCCAGAT	59.962	CCCTCCATCGAGATCCTCCT	59.886	141
TRINITY_Contig2815	2	p6	(GCGCCG)3	18	246	263	ATTTGGTGCGTGACCCAGAT	59.962	CCCTCCATCGAGATCCTCCT	59.886	141
TRINITY_Contig2815	3	p3	(GCT)5	15	792	806	ATTTGGTGCGTGACCCAGAT	59.962	CCCTCCATCGAGATCCTCCT	59.886	141
TRINITY_Contig2817	1	p4	(ACAT)3	12	280	291	CCATGGATGATCGGACGGAG	60.038	CTTTCTGTCATCGCCCCCTT	60.035	198
TRINITY_Contig2817	2	p3	(AGC)5	15	738	752	CCATGGATGATCGGACGGAG	60.038	CTTTCTGTCATCGCCCCCTT	60.035	198
TRINITY_Contig2817	3	p3	(GAT)4	12	905	916	CCATGGATGATCGGACGGAG	60.038	CTTTCTGTCATCGCCCCCTT	60.035	198
TRINITY_Contig2817	4	p4	(GCTA)3	12	1186	1197	CCATGGATGATCGGACGGAG	60.038	CTTTCTGTCATCGCCCCCTT	60.035	198
TRINITY_Contig2818	1	p5	(TTTTG)4	20	125	144	GCGCGCTTGCTTTTGTTTTT	59.974	CTCTGAACTGTGAGCCCCTG	60.037	132
TRINITY_Contig2819	1	c	(TCACC)3gcgagattctccgtccaaacgcca gaaccagagccccttaaaagccagggagctc ctgctcccgagttcccccaaacacttcacc(AG CT)3	113	210	322	TACAGAAGGTGCATGCAGGG	60.035	AAGTGCGATGACTGCACAGA	59.966	113
TRINITY_Contig2820	1	p3	(GGC)4	12	73	84	CTCGACGCTGACCTCATCTC	59.971	TGCTGCATCCTTTTGTGTGC	59.968	264
TRINITY_Contig2821	1	p3	(CGG)5	15	1607	1621	AGCTCAAAGAGGTTGGAGGC	59.963	CCCTTGAACCCCACTTCCTC	59.962	179
TRINITY_Contig2823	1	p3	(GAA)4	12	253	264	GTTGCCTCTGAAAGCAAGCC	60.039	GAGTATCTGCCTCGTCACGG	59.97	246
TRINITY_Contig2823	2	p3	(TGC)5	15	753	767	GTTGCCTCTGAAAGCAAGCC	60.039	GAGTATCTGCCTCGTCACGG	59.97	246
TRINITY_Contig2824	1	p2	(AC)6	12	6	17	TCTGGCATGTGACACTGGAC	59.965	CTGGCTGACTTGTTTGCCAC	59.969	113
TRINITY_Contig2828	1	c	(CCG)6cggcgcactcttcccacgaccgcatc gatccaaccgtcgcgctagttttt(CCGC)3	80	418	497	AGGCAAAGCTCTGGATGCAT	60.033	TTCCCATCTGCCAACTGGTC	59.962	140
TRINITY_Contig2829	1	p3	(CGC)4	12	5000	5011	TCTGCGCTGGTCCAAATGAT	60.035	GCTTTGGCGTTTGTGCTACA	59.97	114
TRINITY_Contig2830	1	p4	(ACGA)3	12	174	185	CAATCCAAATCCGGGGAGGG	60.468	ATTTAACGGCGAGGACCAGG	60.108	192
TRINITY_Contig2832	1	p4	(GTTT)3	12	1698	1709	ATGACTGAGGAGGGGGAGTC	60.032	AATCGAGGGCAATTGCAGGA	60.034	156
TRINITY_Contig2834	1	p5	(GTTCG)3	15	1112	1126	CGGGCTCACAGAAGTTCAGT	59.966	CATCCGTGGAGATGAGGAGC	59.967	159
TRINITY_Contig2837	1	p3	(GGC)4	12	1112	1123	GAATGATTGCGGCCGTCTTC	59.972	GGGAGAGGGACCAGCTCTTA	60.032	225
TRINITY_Contig2837	2	c	(GCA)4acct(CGG)5	31	1485	1515	GAATGATTGCGGCCGTCTTC	59.972	GGGAGAGGGACCAGCTCTTA	60.032	225
TRINITY_Contig2838	1	p3	(GAG)4	12	1618	1629	CTTCTCCTTGGCGTTGTCCT	59.965	CCCGGAGATGGACAAGATCG	59.967	145
TRINITY_Contig2839	1	c	(CAC)5cattccatccggcacacatggagcac cgccccatccgcggcttctacctgtacagcgac tacgccgccggcgccggcgccgcgcga(CGT)7	120	64	183	CTCTCAGCACCTTCACCGTT	59.966	TCCATTGCCTGGGTTGGATC	60.033	184
TRINITY_Contig2839	2	p3	(GGC)4	12	339	350	CTCTCAGCACCTTCACCGTT	59.966	TCCATTGCCTGGGTTGGATC	60.033	184
TRINITY_Contig2839	3	p3	(CGT)5	15	631	645	CTCTCAGCACCTTCACCGTT	59.966	TCCATTGCCTGGGTTGGATC	60.033	184
TRINITY_Contig2839	4	p4	(CAGC)3	12	753	764	CTCTCAGCACCTTCACCGTT	59.966	TCCATTGCCTGGGTTGGATC	60.033	184
TRINITY_Contig2839	5	p3	(GCC)5	15	2290	2304	CTCTCAGCACCTTCACCGTT	59.966	TCCATTGCCTGGGTTGGATC	60.033	184
TRINITY_Contig2840	1	p5	(AAACC)4	20	1455	1474	GGATCCACCCAAACGACCAT	60.034	AGTTACTAGCCCGACCCCAT	60.031	123
TRINITY_Contig2841	1	p3	(CGA)4	12	437	448	CGATCGAAAGGGTTGGGTGA	60.037	ACGAGCAAGCTGATGACACA	59.966	140
TRINITY_Contig2841	2	p6	(CAACGG)3	18	905	922	CGATCGAAAGGGTTGGGTGA	60.037	ACGAGCAAGCTGATGACACA	59.966	140
TRINITY_Contig2843	1	p3	(CGC)4	12	1079	1090	ACGTCTTCAAGCAGGACTGG	59.966	TGGAGGTGACGACGAACTTG	59.968	168
TRINITY_Contig2847	1	p4	(CGAG)5	20	1327	1346	GCTTCTGTTTGCACCTTGGG	59.967	GAGGTGACAGACTTGTGGGG	59.965	248
TRINITY_Contig2848	1	p3	(GAG)4	12	2678	2689	TCGGGAACATTTCTGCCCTC	60.035	CTGGATTGGAACCCCAGCAT	60.033	280
TRINITY_Contig2848	2	p3	(GCA)4	12	2792	2803	TCGGGAACATTTCTGCCCTC	60.035	CTGGATTGGAACCCCAGCAT	60.033	280
TRINITY_Contig2849	1	p6	(CGCCGG)3	18	533	550	AGAGAGGCAGTACTCCAGGG	60.033	CAAGGGCTTCAGATGCAGGA	60.035	131
TRINITY_Contig2849	2	p3	(GCC)6	18	702	719	AGAGAGGCAGTACTCCAGGG	60.033	CAAGGGCTTCAGATGCAGGA	60.035	131
TRINITY_Contig2852	1	p3	(GCG)5	15	1216	1230	TCAGCAGTGACTGAAGCCAG	59.966	CGGGCGTTTGTTTCGTCTTT	59.972	243
TRINITY_Contig2852	2	p6	(GTGGCG)3	18	1831	1848	TCAGCAGTGACTGAAGCCAG	59.966	CGGGCGTTTGTTTCGTCTTT	59.972	243
TRINITY_Contig2852	3	p3	(GGC)5	15	2175	2189	TCAGCAGTGACTGAAGCCAG	59.966	CGGGCGTTTGTTTCGTCTTT	59.972	243
TRINITY_Contig2852	4	p4	(CCTT)4	16	2378	2393	TCAGCAGTGACTGAAGCCAG	59.966	CGGGCGTTTGTTTCGTCTTT	59.972	243

TRINITY_Contig2853	1	c*	(TC)6atccg(TCCC)3act(TGAGC)3(AGC)4*tggagctgagagctcactccccaccacaaacaacagacggatcccatcccaaacaaaagaa(AG)7atggatccttcgtctactctagcatgagatgagatgaaatactgacaagggaggaaggaagagcacttccatccgttccagctccagctttc(CATCT)3cgctcaccttttggttttggccggcaagggaaggattcctcccccgcccctccccgcctgct(GCATC)3	316	150	465	GTGGCGTATGATGACAACGC	59.974	CCCGCTCAGGAGATCATCAC	59.967	221
TRINITY_Contig2853	2	c	(CAG)4tgaccgaccc(GTCT)4	38	2549	2586	GTGGCGTATGATGACAACGC	59.974	CCCGCTCAGGAGATCATCAC	59.967	221
TRINITY_Contig2853	3	p5	(ATTCT)3	15	2734	2748	GTGGCGTATGATGACAACGC	59.974	CCCGCTCAGGAGATCATCAC	59.967	221
TRINITY_Contig2853	4	p3	(TTC)4	12	3270	3281	GTGGCGTATGATGACAACGC	59.974	CCCGCTCAGGAGATCATCAC	59.967	221
TRINITY_Contig2853	5	p3	(GCG)5	15	3758	3772	GTGGCGTATGATGACAACGC	59.974	CCCGCTCAGGAGATCATCAC	59.967	221
TRINITY_Contig2853	6	p5	(GGAGT)3	15	3880	3894	GTGGCGTATGATGACAACGC	59.974	CCCGCTCAGGAGATCATCAC	59.967	221
TRINITY_Contig2853	7	p3	(GCG)5	15	5628	5642	GTGGCGTATGATGACAACGC	59.974	CCCGCTCAGGAGATCATCAC	59.967	221
TRINITY_Contig2853	8	c	(CGG)5t(GGC)6	34	5965	5998	GTGGCGTATGATGACAACGC	59.974	CCCGCTCAGGAGATCATCAC	59.967	221
TRINITY_Contig2853	9	p3	(GCG)7	21	6285	6305	GTGGCGTATGATGACAACGC	59.974	CCCGCTCAGGAGATCATCAC	59.967	221
TRINITY_Contig2854	1	p3	(CAC)5	15	183	197	GGAAGTCCTGGGGAGGAGAT	60.031	CCTGACGGGCAGTTTCTTCT	59.965	133
TRINITY_Contig2854	2	c	(CTA)4cgaccccgctgccggcgtcgga(GGC)5	49	332	380	GGAAGTCCTGGGGAGGAGAT	60.031	CCTGACGGGCAGTTTCTTCT	59.965	133
TRINITY_Contig2855	1	p3	(GGA)4	12	1088	1099	TCGAACAGCTGCCTGAAGAG	60.038	ATATAACCAGGCGAGCCACG	59.966	199
TRINITY_Contig2856	1	p4	(GGAT)3	12	1450	1461	GACTTTGACTCTGGTGCGGA	59.966	CGCAATCGCAAGGAATCCAG	59.971	154
TRINITY_Contig2857	1	p3	(GTC)4	12	219	230	GACGGCATCATCTACCCCTG	59.966	CCGTGCTTACCCTTGTGGAT	60.035	182
TRINITY_Contig2858	1	p2	(GA)6	12	945	956	GGAAGAGCGAGAGGGAGAGA	60.107	TTTACTCCCCATGCCGTGTC	60.036	155
TRINITY_Contig2859	1	p3	(CGG)5	15	714	728	CGGCATGTTGGCACCATATG	59.969	ATGTGCATCCTGGCCTTTCA	59.96	128
TRINITY_Contig2859	2	p3	(GCT)4	12	906	917	CGGCATGTTGGCACCATATG	59.969	ATGTGCATCCTGGCCTTTCA	59.96	128
TRINITY_Contig2860	1	p3	(CCT)4	12	86	97	TCTGCCAAAGAGCTGCAAGA	59.891	CTCCACGGCCTTCTCAAACT	59.965	246
TRINITY_Contig2865	1	p2	(CT)16	32	70	101	GCAAGGTGCAAGTGACAGTG	59.97	GTTGCATCCTGGAGCTCCTT	60.035	228
TRINITY_Contig2866	1	p3	(GCG)7	21	4146	4166	AGGAAGACCGGCTTGAACTG	59.965	TCGTGTCCAAGTCTCACACG	59.97	109
TRINITY_Contig2870	1	c	(AGG)4ccattgg(CCAC)3	31	2471	2501	ACACGCACACATCAGTACGT	59.968	GCATGCGTGCGTGTTAGTAG	59.975	108
TRINITY_Contig2871	1	c	(CCTTC)3cccgcacgacgactgacgacgaccaccccccaaccccacgccgggaagccgcaccaaatctcatcccctcccctctagggcggctgtaaaaatc(TGG)4	121	9	129	CACACTTCGACGTGGTGAGA	59.97	ATCTGGCTGAAACTGGGTCG	60.036	249
TRINITY_Contig2871	2	p3	(GCT)4	12	309	320	CACACTTCGACGTGGTGAGA	59.97	ATCTGGCTGAAACTGGGTCG	60.036	249
TRINITY_Contig2872	1	p3	(GGA)4	12	270	281	CCTCCTGTTCCTGCAATTGC	59.471	CCCTCCCCTCCCTCCAATTA	60.029	108
TRINITY_Contig2872	2	p3	(TTC)7	21	572	592	CCTCCTGTTCCTGCAATTGC	59.471	CCCTCCCCTCCCTCCAATTA	60.029	108
TRINITY_Contig2873	1	p3	(CGG)4	12	1278	1289	GCGATGCGGATAATGCACAG	60.111	CAGTTCCAATCCCACCTCCC	60.034	277
TRINITY_Contig2874	1	p3	(GAG)4	12	2008	2019	ACCAGAGCTTGAACCGGAAG	59.965	CAGGGTAGGACGAAGGAGGA	60.033	262
TRINITY_Contig2874	2	c	(CGG)4ttaaatccgttgctcgggtcgtgcgctccagctagctgccgacgggaccggatcggatt(GGGC)3	83	2160	2242	ACCAGAGCTTGAACCGGAAG	59.965	CAGGGTAGGACGAAGGAGGA	60.033	262
TRINITY_Contig2874	3	p3	(GCG)5	15	2474	2488	ACCAGAGCTTGAACCGGAAG	59.965	CAGGGTAGGACGAAGGAGGA	60.033	262
TRINITY_Contig2879	1	c	(TGC)6tggaactgcgaaccgaacga(CTG)5cggcctgctcgccgagccgaatccaaacgggttgggctt(CTG)6	110	182	291	CGACAGTGAACCTGGAAGCT	59.966	TTGCACAAATCTGCCAAGGC	59.966	220
TRINITY_Contig2879	2	p3	(GCA)4	12	5087	5098	CGACAGTGAACCTGGAAGCT	59.966	TTGCACAAATCTGCCAAGGC	59.966	220
TRINITY_Contig2881	1	p3	(CGG)4	12	186	197	TTGAGTTTGCCTTTCTGCGC	59.969	TCTTCCCTTCCATTGCCCAC	59.96	138
TRINITY_Contig2882	1	c	(CAC)4caaggccacttccaggccttcaccctgcccaaggatcccccgatcctgttcccctttgtgat(CAG)4	86	241	326	CCGTGTCACTGTTCGAGTCA	59.97	CTAGCCCCAGCACACAGAAA	59.963	210
TRINITY_Contig2882	2	p3	(CGC)6	18	996	1013	CCGTGTCACTGTTCGAGTCA	59.97	CTAGCCCCAGCACACAGAAA	59.963	210
TRINITY_Contig2883	1	p3	(CGT)7	21	181	201	TCTGTCTTCACGGCACTGTC	59.968	TTGTGAGACGACAGCCAGTC	59.968	112
TRINITY_Contig2885	1	c	(GTCCGC)3gccctccccgcccgccggcgagccgccctcccca(CCG)4	64	336	399	GATTGAGCCAGTGGTTCCGA	60.036	GAAAACCATTGGCTCCGCTG	60.109	149
TRINITY_Contig2885	2	c	(CGGCTC)4a(TCC)4gagctctccgacgaccc(CGA)6	72	576	647	GATTGAGCCAGTGGTTCCGA	60.036	GAAAACCATTGGCTCCGCTG	60.109	149
TRINITY_Contig2886	1	p3	(CCG)4	12	116	127	CGCCAAGATCGCAATCAGTG	59.973	GATCCAGCTTGAGGTGTGCT	60.036	118
TRINITY_Contig2887	1	p4	(GAGC)3	12	221	232	AGGGGAGAGAGAGGAGCTTG	60.032	ATAACACAGCCGGCGGTTAA	60.036	207
TRINITY_Contig2887	2	p3	(GAG)4	12	409	420	AGGGGAGAGAGAGGAGCTTG	60.032	ATAACACAGCCGGCGGTTAA	60.036	207
TRINITY_Contig2887	3	p3	(GCG)5	15	587	601	AGGGGAGAGAGAGGAGCTTG	60.032	ATAACACAGCCGGCGGTTAA	60.036	207
TRINITY_Contig2888	1	c	(GTC)4gaggctgacgtcgaagaagtcatcgccatcgaggtccatgctcatcgccaggtggtctgaggacagtagcaggaga(CAGAGG)3	106	1591	1696	AGAGGCAGAGGCAGATGAGA	60.032	TGCTCCAAATTCCACGACGA	59.966	202
TRINITY_Contig2889	1	p3	(GAT)4	12	594	605	CAGCTCTTGGGAGGAAGGTG	60.036	AGCTGCTTGGCATGGATTCT	60.033	103
TRINITY_Contig2890	1	p4	(ACAT)3	12	23	34	TCAGCGCAGGAAATCACTGT	59.965	TCAGAAGTTGAATGCGGGCT	59.963	134
TRINITY_Contig2890	2	p3	(GCC)4	12	2111	2122	TCAGCGCAGGAAATCACTGT	59.965	TCAGAAGTTGAATGCGGGCT	59.963	134
TRINITY_Contig2891	1	p5	(TTGGA)3	15	324	338	ATGCTTCAATCACCCACGGT	59.962	CGGAGCAGGCCTTAGTTGAA	60.036	142
TRINITY_Contig2891	2	p6	(CGTGCG)3	18	464	481	ATGCTTCAATCACCCACGGT	59.962	CGGAGCAGGCCTTAGTTGAA	60.036	142
TRINITY_Contig2891	3	p3	(CCT)5	15	1388	1402	ATGCTTCAATCACCCACGGT	59.962	CGGAGCAGGCCTTAGTTGAA	60.036	142
TRINITY_Contig2891	4	p3	(GCC)5	15	1817	1831	ATGCTTCAATCACCCACGGT	59.962	CGGAGCAGGCCTTAGTTGAA	60.036	142
TRINITY_Contig2891	5	p3	(GGC)5	15	2404	2418	ATGCTTCAATCACCCACGGT	59.962	CGGAGCAGGCCTTAGTTGAA	60.036	142
TRINITY_Contig2891	6	p3	(CAG)4	12	2813	2824	ATGCTTCAATCACCCACGGT	59.962	CGGAGCAGGCCTTAGTTGAA	60.036	142
TRINITY_Contig2895	1	p5	(AGCGC)4	20	365	384	TTCCTGATGCAGTTTCGGCT	59.963	GCTTCAACTATCGCAGCTGC	59.973	231
TRINITY_Contig2895	2	p3	(GGC)4	12	1133	1144	TTCCTGATGCAGTTTCGGCT	59.963	GCTTCAACTATCGCAGCTGC	59.973	231
TRINITY_Contig2898	1	p3	(CCA)4	12	33	44	CATGCGCCCTGATCGATACT	60.038	CTCTACCATCCGCCCATGTC	59.966	280
TRINITY_Contig2899	1	p3	(CAC)4	12	356	367	ATTGGAGGGTGCTCGACAAG	60.036	TGTACGTTGTGCAGCAGCTA	59.967	202
TRINITY_Contig2901	1	p4	(TCAT)3	12	1977	1988	AGGCTTGGTTGTTGGTGGAA	60.033	AGAAGCATCCAGTCCAAGGC	60.035	210
TRINITY_Contig2903	1	p3	(GGA)4	12	281	292	TACTGTCAGCTCCACTTGCG	60.038	TGGTGTTCCAAGAACCTCCG	59.892	218
TRINITY_Contig2905	1	p4	(TTTG)4	16	229	244	CCACTCAACGGACCCCTAAC	60.037	GCAAGACCACGAACCACCTA	59.966	130
TRINITY_Contig2907	1	c	(GGC)8ggggagccgctgccgtcgctggggcgccgccgggtgccgcgcgtcgtcacggcgcccggcattgtgccgggcctcgacgcctc(CGA)4	119	260	378	AACTCGAGAAACACGGTCCC	59.967	CGAGAAACCTACGCGATCGA	59.973	126
TRINITY_Contig2907	2	p4	(TCGC)3	12	2947	2958	AACTCGAGAAACACGGTCCC	59.967	CGAGAAACCTACGCGATCGA	59.973	126
TRINITY_Contig2907	3	p5	(GGGAG)3	15	3577	3591	AACTCGAGAAACACGGTCCC	59.967	CGAGAAACCTACGCGATCGA	59.973	126
TRINITY_Contig2907	4	c	(GCCGA)3cgtcccgcgcgtcaggatcgtggtggcgataaggatccaccgccggaaccggccgcgcgggtggggcagggccgggtccgcgcga(CGTCG)3	115	3905	4019	AACTCGAGAAACACGGTCCC	59.967	CGAGAAACCTACGCGATCGA	59.973	126
TRINITY_Contig2908	1	p3	(AAG)5	15	1895	1909	AGGTACACATGGAGCAAGGC	60.035	AGAGTCACAACCAGCTCTGC	59.965	184
TRINITY_Contig2910	1	p3	(CTC)5	15	88	102	AAACGTTGGCCCAATGGTTG	59.894	AGGATAGTACACTGGCGGGT	60.032	133
TRINITY_Contig2911	1	p6	(GCGGAA)3	18	1350	1367	GGGATCTGGATTCTGGAGCG	59.965	CGAAGAAGACCAGGGAAGGG	59.749	235
TRINITY_Contig2912	1	p4	(CCTG)3	12	320	331	CTGCCGTATCTACACCGGTC	59.97	GGAACGAAGGTCCCAGGAAG	60.036	187
TRINITY_Contig2913	1	c	(CGCCA)3cgcttcc(ATCG)3	34	355	388	GAGCATTTAAGCGGCGAGTG	59.973	ATCCACCTTCTCCCACGAGA	59.959	263
TRINITY_Contig2913	2	p2	(AG)8	16	1822	1837	GAGCATTTAAGCGGCGAGTG	59.973	ATCCACCTTCTCCCACGAGA	59.959	263
TRINITY_Contig2914	1	p5	(TCCCG)4	20	882	901	CACAGGAAGGCAAGCACAAC	59.969	CCCTGGACTGGAGATCTGGA	60.032	213
TRINITY_Contig2915	1	p3	(CCG)4	12	39	50	CCTGGAGCAGATCGACCATC	59.967	TCCTGACCAGCGAACTTGAC	59.966	266
TRINITY_Contig2918	1	p6	(CGGAGC)3	18	361	378	CCTCTTCCTCCTCTGCCTCT	60.032	CAGGAGGAGATGATGGGGGA	60.105	175
TRINITY_Contig2919	1	p3	(GGC)4	12	141	152	ATGCCTTGCGGAAACTACCA	59.962	ACTGAACTGCCAGCAGGTAC	59.965	100
TRINITY_Contig2919	2	p3	(GGC)4	12	468	479	ATGCCTTGCGGAAACTACCA	59.962	ACTGAACTGCCAGCAGGTAC	59.965	100
TRINITY_Contig2919	3	c	(GCAGAG)4aattttgaagtatcatatcaaaagagcaaggttagatttgcgaggcaggat(GAA)4gatgggggggagggttctgtaattgagcagatgacagaaagtctggctcgccgtgacccgcttcgtgctcgcccaga(GCG)4	176	1230	1405	ATGCCTTGCGGAAACTACCA	59.962	ACTGAACTGCCAGCAGGTAC	59.965	100
TRINITY_Contig2924	1	p6	(CGCTGG)3	18	5258	5275	ACAGTGCTCAGTGACATGGG	59.964	GACGGGTGGCAAACTTGAAC	59.97	280
TRINITY_Contig2925	1	p5	(CGCCC)3	15	15	29	CCTCCGATACCTTCGTCAGC	59.969	GAGCCAGTTGTCACCGAAGA	59.966	155
TRINITY_Contig2925	2	p3	(GCG)4	12	484	495	CCTCCGATACCTTCGTCAGC	59.969	GAGCCAGTTGTCACCGAAGA	59.966	155
TRINITY_Contig2926	1	p3	(CTC)6	18	649	666	GCTCGACCAAAAACCCCAAC	59.968	GAGGTTGGGGAATGGGGAAG	60.033	257
TRINITY_Contig2930	1	p3	(GCG)4	12	371	382	AAGTTCCCAGCCCGAACAAA	60.106	CGGTGAGGCAATCGAAGACT	60.109	272
TRINITY_Contig2931	1	p4	(CTCC)3	12	157	168	CTGCATTCCTGGTGGATGGT	60.034	ACGATGATAGCGTGCTGGAG	59.969	197
TRINITY_Contig2931	2	p3	(CTG)4	12	2045	2056	CTGCATTCCTGGTGGATGGT	60.034	ACGATGATAGCGTGCTGGAG	59.969	197
TRINITY_Contig2932	1	p5	(GAAGG)3	15	1819	1833	CCAGCAGGTTCAGGCATGTA	60.035	GTTTTGGGGATCTGAGGGCA	59.96	247

TRINITY_Contig2935	1	p3	(CGG)4	12	2224	2235	GCTCCATGTCTACTCGCCTC	59.969	TCCACTAGTCCGTGTGCAAC	59.967	216
TRINITY_Contig2935	2	p3	(CCG)6	18	2846	2863	GCTCCATGTCTACTCGCCTC	59.969	TCCACTAGTCCGTGTGCAAC	59.967	216
TRINITY_Contig2937	1	p3	(CCA)4	12	22	33	CAGCTGCCTAACACCAAGGA	59.963	AGCCTGTACGCGATCATGAG	59.969	159
TRINITY_Contig2939	1	p3	(GGC)4	12	115	126	GCTCCAGTTCTTCCAGGTCC	60.036	AAAGATCCAGCCCTGCTTCC	60.034	107
TRINITY_Contig2940	1	p2	(GC)6	12	1461	1472	GTGTCTTGCCACCAACGTTC	59.971	TTACCACTGCCAGTCGACAC	59.967	280
TRINITY_Contig2941	1	c	(GGGC)3cgccgccggcgtggcgcacgcggtggccgcgggggacatggcga(TGGG)3	68	2239	2306	GCCAGCTTTCCTCACTCTGT	59.964	TCTGGTTCTCTTTGACCGGC	59.965	249
TRINITY_Contig2942	1	p3	(ATG)4	12	1503	1514	CGCATTCGCATTGGTCAGAG	59.973	GCCGGTCTTTCAAAAGCCTG	60.039	126
TRINITY_Contig2947	1	p3	(GCG)6	18	721	738	ACGCAGTCAAGACAGGTCTG	59.967	GACGCTGTCAAACCACAACC	59.971	238
TRINITY_Contig2948	1	p3	(TTC)4	12	56	67	AAGATCGAGGTACCAGCCCT	60.032	ACCTGTGTGCTGTGTGGAAA	60.035	160
TRINITY_Contig2948	2	p2	(GC)7	14	1466	1479	AAGATCGAGGTACCAGCCCT	60.032	ACCTGTGTGCTGTGTGGAAA	60.035	160
TRINITY_Contig2950	1	c	(TCT)5ttttcttttcacttccatccttgtcatcagccacatcagccttcacttccgctgatcctgatttagccttatctttcttt(TTC)5tcactcttttccccttccactgtctcagcagcttcctcagtcttgatcttcttaacaggaacagaagcatcaccatcatcggtttcctcgtccttcttcc(TCT)5	225	806	1030	ACATCGTGCATGGTCACCAT	60.035	GTGCGGACCATCTACGAGAG	59.97	212
TRINITY_Contig2950	2	c	(GCG)4gggtcgtccttcgacttgtg(CTT)4	44	2329	2372	ACATCGTGCATGGTCACCAT	60.035	GTGCGGACCATCTACGAGAG	59.97	212
TRINITY_Contig2953	1	p4	(TATG)3	12	1515	1526	AGCTGGAAGAACTTGCCTCC	59.963	CGCGCAGATTCTTGAGTTCG	59.976	100
TRINITY_Contig2955	1	p3	(CCG)4	12	1630	1641	TGGTGCCACGTCTAGTCAAC	59.967	GCGTGATCGATCTGAGCTGA	59.97	198
TRINITY_Contig2957	1	p4	(GTTT)5	20	1075	1094	TGGTTCAACGAGACCTTCCG	59.967	GGCGATCGATCAATCCCCTT	59.965	111
TRINITY_Contig2957	2	p3	(TCG)4	12	1291	1302	TGGTTCAACGAGACCTTCCG	59.967	GGCGATCGATCAATCCCCTT	59.965	111
TRINITY_Contig2958	1	c	(GTG)4gcggcccggccgctt(GCC)4	39	461	499	CAGGGGATCGAGAGGTGAGA	60.106	GAGCTGCTCTCCGAGAAGAC	59.899	255
TRINITY_Contig2958	2	c	(CGG)5cattgttgctgtcgctcttgttgaggttcttgatgcaccagacgtcctcgtcgcaggagaagcggcgccagcgcggga(CGG)4	105	832	936	CAGGGGATCGAGAGGTGAGA	60.106	GAGCTGCTCTCCGAGAAGAC	59.899	255
TRINITY_Contig2959	1	p3	(AGG)5	15	2226	2240	ATGGAAGCAAGCACAGCTCT	59.962	ATTTCGAGCTGGTGTGCTGA	59.965	198
TRINITY_Contig2960	1	p3	(CGC)4	12	232	243	GGAAAAGGGGGATGGGTGAG	60.033	TCTCCCCATCGACTGCAAAC	60.036	117
TRINITY_Contig2961	1	p4	(GAGG)3	12	1864	1875	TCCTGGTGTCTTCCTCCCAA	60.105	ATCACACCGTCGTCTTCCAC	60.039	165
TRINITY_Contig2962	1	p3	(CGC)5	15	518	532	GTTGCACTGGGCTTTCATGG	60.038	TCGCTCTGCACAATGTCCAT	60.036	113
TRINITY_Contig2963	1	c	(AAGC)3(GCAG)3	24	32	55	ATTTCGCTTCAATCCGGGGT	60.034	GTGGTCACGACACTCGAGTT	59.97	273
TRINITY_Contig2964	1	p4	(CCTT)3	12	63	74	GTGACCAAGGCGAAGAAGGA	59.965	GCGCACCGGTGATCATTAAC	59.972	183
TRINITY_Contig2964	2	p5	(GTTGC)3	15	2969	2983	GTGACCAAGGCGAAGAAGGA	59.965	GCGCACCGGTGATCATTAAC	59.972	183
TRINITY_Contig2965	1	p3	(ATC)4	12	747	758	AGGAGGGAAGGAGGGGATTC	60.03	ACACACAGGCCACAGTGAAA	60.035	139
TRINITY_Contig2968	1	p3	(CTT)4	12	198	209	TTGTAGCTGTAGGGCTTGGC	60.035	GCCTTCTTTGACTCCTCCCC	60.035	258
TRINITY_Contig2969	1	p4	(CCTT)3	12	247	258	CCTGCGGCGTACAATGATTG	59.972	AAACTTCAGCTAGCGGTCCC	60.036	184
TRINITY_Contig2970	1	c	(CTG)7cacaacagcccagcaggagagaaaga(GAGC)3gaggagaggggcggcagcg(GCA)4	91	4	94	GCATCAGGGAGACTACGTCG	59.97	CTGATGAACTCGGTGGTGCT	60.037	108
TRINITY_Contig2973	1	p3	(CAG)5	15	1839	1853	GAGCGCCTGTAGTATCACCC	59.968	CATTGGCCATGCTGAGATGC	59.968	268
TRINITY_Contig2974	1	p3	(AGC)4	12	25	36	CCATCTACGGATCCGCCTTC	60.038	AGGCGAACCTCCATTCCATG	60.107	217
TRINITY_Contig2976	1	p3	(CCG)4	12	1583	1594	TGATCGCAGCAGAAGCATCA	60.108	TGGCCAAGTGCATAGGTGAG	60.035	225
TRINITY_Contig2977	1	p3	(CTC)4	12	2512	2523	CGAGTCTCTTCGTACGCCTC	59.973	GGTTCGGCTTCAGTGTCTGA	59.966	209
TRINITY_Contig2979	1	c	(CCG)4ccctcgcccgcctcgcgcacgcgatcctcggcggcgccagcggctggtgggcgctcctcttcatgcaactctgccccagcccgacgtcggccgccgcg(ACC)4	122	150	271	CCCAATTGGCCTTCTACGGT	60.034	GAACTCGCTGTCTGTGGTCA	59.968	263
TRINITY_Contig2979	2	p3	(GAA)5	15	881	895	CCCAATTGGCCTTCTACGGT	60.034	GAACTCGCTGTCTGTGGTCA	59.968	263
TRINITY_Contig2980	1	p3	(CTG)4	12	517	528	TTGTGGAGGAGAAGCAGCAG	59.964	GCAAGGAACACCAAGCAGTG	59.969	208
TRINITY_Contig2980	2	p3	(GCC)7	21	3215	3235	TTGTGGAGGAGAAGCAGCAG	59.964	GCAAGGAACACCAAGCAGTG	59.969	208
TRINITY_Contig2981	1	p3	(CGC)4	12	597	608	ATCCCTGAACTATCGCTGCG	59.967	AGAACGGCCTCTCCAGAGAT	60.033	119
TRINITY_Contig2983	1	p4	(GCAT)3	12	310	321	CAGGAGATGACACAGCTGCA	60.037	CCTCAAGCCCGTCAACTTCT	59.965	265
TRINITY_Contig2983	2	p4	(GCCC)3	12	584	595	CAGGAGATGACACAGCTGCA	60.037	CCTCAAGCCCGTCAACTTCT	59.965	265
TRINITY_Contig2983	3	p2	(GC)6	12	2267	2278	CAGGAGATGACACAGCTGCA	60.037	CCTCAAGCCCGTCAACTTCT	59.965	265
TRINITY_Contig2990	1	p3	(AGA)4	12	191	202	CGGTGGAATCGAAGGTCCAA	60.037	GGGCGCGAGATCGAATAGAA	60.039	214
TRINITY_Contig2991	1	p3	(AGC)4	12	126	137	GGGAATGGAGCACACGTACA	60.037	CTCCAAGGAAGGAAGCTGGG	60.035	230
TRINITY_Contig2991	2	p3	(CTC)4	12	1004	1015	GGGAATGGAGCACACGTACA	60.037	CTCCAAGGAAGGAAGCTGGG	60.035	230
TRINITY_Contig2991	3	c	(GTC)4tgcgtcgcagcgttttgttacg(GGGGC)3	49	1409	1457	GGGAATGGAGCACACGTACA	60.037	CTCCAAGGAAGGAAGCTGGG	60.035	230
TRINITY_Contig2999	1	p3	(CGG)4	12	248	259	TCCGGTTGGTCTGTTGGATG	59.964	TAGCATGGACCTCAGAGGCT	60.032	274
TRINITY_Contig3000	1	p4	(GCAC)3	12	131	142	GCCAGCAGCAGTAGATCGAT	59.967	CACGACGTGAGGAAGGTGAA	59.968	247
TRINITY_Contig3000	2	p4	(GATC)3	12	842	853	GCCAGCAGCAGTAGATCGAT	59.967	CACGACGTGAGGAAGGTGAA	59.968	247
TRINITY_Contig3001	1	p3	(GCG)4	12	16	27	CGCTCAGGTACTCATCCGTC	59.97	CGGAATCCTCGAACTCGGAG	59.97	221
TRINITY_Contig3002	1	p4	(ACGC)3	12	266	277	AGCAGAAGAGGAGCAGCTTG	60.036	CGGGATCATGGACGAGAAGG	59.967	254
TRINITY_Contig3002	2	p3	(GCC)4	12	1245	1256	AGCAGAAGAGGAGCAGCTTG	60.036	CGGGATCATGGACGAGAAGG	59.967	254
TRINITY_Contig3004	1	c	(GGC)4ttgacgggggcgaggaggcggtcgtagtcgacgtcgtagtcctgctcgttgtcgat(GTC)4	80	1078	1157	TGGTGCACCTCACGTACTTC	59.967	ACAAGATCAGGCTGCTCCAC	60.036	165
TRINITY_Contig3004	2	p4	(TTCC)3	12	1410	1421	TGGTGCACCTCACGTACTTC	59.967	ACAAGATCAGGCTGCTCCAC	60.036	165
TRINITY_Contig3004	3	p4	(GAGC)3	12	2942	2953	TGGTGCACCTCACGTACTTC	59.967	ACAAGATCAGGCTGCTCCAC	60.036	165
TRINITY_Contig3006	1	c	(CGAA)3caccaggtcacc(ACG)6	42	331	372	CACACAGCAGAGACACGAGT	59.969	TGGTGTTCGTTCGTTCGGAT	59.968	188
TRINITY_Contig3009	1	p4	(CAAC)3	12	3340	3351	GGAATGACCTGATGTGGCCA	60.034	CTGCCATGCTGAAGATTCGC	59.971	272
TRINITY_Contig3011	1	p3	(GGC)4	12	641	652	TGGCACAGAGACTGCAAGAG	59.966	GATGTCCATCCTGCACACCA	60.035	178
TRINITY_Contig3011	2	p3	(CCG)4	12	1101	1112	TGGCACAGAGACTGCAAGAG	59.966	GATGTCCATCCTGCACACCA	60.035	178
TRINITY_Contig3011	3	p3	(CGT)5	15	1276	1290	TGGCACAGAGACTGCAAGAG	59.966	GATGTCCATCCTGCACACCA	60.035	178
TRINITY_Contig3011	4	p3	(GGC)4	12	1440	1451	TGGCACAGAGACTGCAAGAG	59.966	GATGTCCATCCTGCACACCA	60.035	178
TRINITY_Contig3011	5	p4	(AAGC)4	16	1707	1722	TGGCACAGAGACTGCAAGAG	59.966	GATGTCCATCCTGCACACCA	60.035	178
TRINITY_Contig3011	6	c	(GGA)6agaagaaga(CTAG)3	39	1847	1885	TGGCACAGAGACTGCAAGAG	59.966	GATGTCCATCCTGCACACCA	60.035	178
TRINITY_Contig3012	1	p3	(GGC)4	12	140	151	GCGGAGAGACTCGATAAGGC	60.04	CCCACGTGGTAAGAGCAGTT	59.965	251
TRINITY_Contig3013	1	p5	(CCGCC)3	15	84	98	CTCACGTTGAAGGTGGAGCT	59.966	TCCATCCGGCTCAAGTATGC	59.893	229
TRINITY_Contig3017	1	c	(TGG)4tagtagtagggttctagccatggcgtggagcgccgccgccgtctccaggttcgccgccgcctcccccgccgcggcgcccgtctccgccaggctcgg(CGC)4	120	111	230	TGGATAAGTGGTGTCGTGGC	60.037	CAGCAAGAAGGGAAGGGAGG	60.035	218
TRINITY_Contig3018	1	p5	(GTTTG)3	15	150	164	GGAGAATGCCGAGGATCCAG	59.965	CTGCTGTGCCAAGGGACATA	60.035	107
TRINITY_Contig3019	1	p3	(GGC)4	12	2464	2475	GGTTCTTAGCCCAGGTGGAC	60.035	TCCGGCTGCTTCAACTGATT	59.963	155
TRINITY_Contig3020	1	p4	(ACAT)3	12	1719	1730	GGGTAGTCGGGTCAAAACGT	59.966	CCACCATCAGCGACAGAACT	60.037	278
TRINITY_Contig3022	1	p4	(CCTC)3	12	165	176	GCATCTCTCTTCGGGAGACG	59.97	TCCGAGAAAATTGGCCCTCC	60.034	113
TRINITY_Contig3024	1	c*	(GAGG)2(GAGGAGGAGGAGGATTCCTTTACAACTTTGTTGTGTTTGTTTGGCTTG<GGAGG>AGGAGGAGGATTCCTTTACAACTTTGTTGTGTTTGTTTGGCTT)(GGA)4	22	3247	3268	CAGCATAAACCCATCTGCGC	59.97	AACGTGCCAACCTGCAATTC	59.968	173
TRINITY_Contig3026	1	c	(CTCC)3cagacagcgacatgacctcg(CGC)4	44	290	333	GGTCCTCTCCCTTGGGATCT	60.031	CAACAGTGTGCTGCAGTTCC	59.97	109
TRINITY_Contig3027	1	p6	(CGCTGG)3	18	1791	1808	TTGGACTCCCATCAATGGCC	60.033	GCAGCAGGTACTCCTTGTGT	59.965	185
TRINITY_Contig3030	1	p3	(GCC)4	12	877	888	TGCTGCCCTTGGAGTATGTG	60.035	GCATCATCCAACTCCCCACA	60.034	150
TRINITY_Contig3030	2	p3	(CGC)4	12	3027	3038	TGCTGCCCTTGGAGTATGTG	60.035	GCATCATCCAACTCCCCACA	60.034	150
TRINITY_Contig3031	1	p4	(GGGT)3	12	5472	5483	CCCACATAAGCTGCAGGTGA	60.035	GGGGCTCAGTACCTCCAGTA	60.032	249
TRINITY_Contig3032	1	p4	(ATGG)3	12	42	53	AGCTTGGTTTTTGGGGGTGA	60.032	GCAGCCTAGCAGGAAAGGAA	60.035	113
TRINITY_Contig3032	2	p4	(AAGG)3	12	1447	1458	AGCTTGGTTTTTGGGGGTGA	60.032	GCAGCCTAGCAGGAAAGGAA	60.035	113
TRINITY_Contig3034	1	p2	(CT)8	16	50	65	TCAGCTGAGAAGAACTGCGG	60.038	TGAAGTCCATGAGCCGCATT	60.035	255
TRINITY_Contig3034	2	p3	(GTC)8	24	1401	1424	TCAGCTGAGAAGAACTGCGG	60.038	TGAAGTCCATGAGCCGCATT	60.035	255
TRINITY_Contig3035	1	p4	(GCTC)3	12	149	160	ATCTGTCCTTCCGGCAAAGG	60.035	CTACTCCGATGGCAGCTCAG	59.968	222
TRINITY_Contig3037	1	p4	(AGGG)4	16	207	222	GCTACGATCGGATTCAGCCA	59.968	GCGCATGCATCGAAGGATTT	59.97	151
TRINITY_Contig3039	1	p4	(CTGA)3	12	1646	1657	CCTGATGGTGATGCCTGGTT	60.034	GCAGGGCATCTTCAGTCAGT	60.036	100
TRINITY_Contig3041	1	p3	(GGC)4	12	94	105	CATCTCCGTGCTGCTCTAGG	59.968	TCGGGCAACTCTTCAAGCAT	59.963	128
TRINITY_Contig3042	1	p3	(CCG)5	15	1609	1623	CGATGTATCGGTTCCCCTGG	59.966	TCACCGTGTCCAACAGCTAC	59.967	255
TRINITY_Contig3044	1	p3	(CCG)5	15	249	263	TGCCAGCTAATCCCATGCAA	60.033	GGGCTAGTGAGGTCAGAGGA	60.033	108
TRINITY_Contig3047	1	p3	(GAT)4	12	2237	2248	GGAAGAGGCACCAGCTCAAT	60.035	TTTACGCCCAACTTGTTGCG	59.97	168
TRINITY_Contig3048	1	p5	(ACCAA)3	15	819	833	GGCCATTGAGCCCTTCTTCT	60.034	AGCTCTGCCTTTGTAGCTGG	60.036	178
TRINITY_Contig3049	1	p3	(CTT)4	12	87	98	TCCCCGGTGGATATCCCTTT	60.029	GCTGGGCTCCACTGATCTTT	60.035	124
TRINITY_Contig3050	1	p3	(CCG)8	24	233	256	GATAGGAACGCTACCACCGG	59.968	GTATGAGCATGGGGTCGAGG	59.966	230

TRINITY_Contig3050	2	p6	(GAGGAC)3	18	697	714	GATAGGAACGCTACCACCGG	59.968	GTATGAGCATGGGGTCGAGG	59.966	230
TRINITY_Contig3050	3	c	(CCG)4gtccggag(CCT)4cgtcctcctggtcaaaaggaagaggaggaa(GGC)4accatcaaagcccgcgatggcggcggcgcggccaatcagttgaggtattacggcaga(GGC)4	143	1187	1329	GATAGGAACGCTACCACCGG	59.968	GTATGAGCATGGGGTCGAGG	59.966	230
TRINITY_Contig3050	4	p4	(CTTG)3	12	1458	1469	GATAGGAACGCTACCACCGG	59.968	GTATGAGCATGGGGTCGAGG	59.966	230
TRINITY_Contig3051	1	p3	(ATG)4	12	775	786	GCTGGTCACGCACACAATTT	59.969	CACACAACGCCTTCATTGCA	59.969	188
TRINITY_Contig3052	1	p3	(GCG)4	12	185	196	CATCTGGAAATGGGGGCCTT	60.032	TTGACACCACCTGATCCAGC	59.963	190
TRINITY_Contig3054	1	p3	(TTC)4	12	259	270	ATCGCAGGACTTCAATGCCA	60.035	TGACATACCACTGTCGGCAC	60.038	174
TRINITY_Contig3056	1	p3	(CGG)4	12	264	275	GCATTGACTATGGCTTGGCG	59.97	CGAGGAGGGGAATACATCGC	60.038	116
TRINITY_Contig3057	1	p5	(CGGCA)3	15	2001	2015	GAGAGCACGTCTTTCCTGCT	60.038	CCCGCGATAGATTTCCTCCC	60.037	204
TRINITY_Contig3059	1	p3	(GCC)4	12	1419	1430	CCATTTCGCTGATCGCTTGG	59.971	GATGCCCTGGTGCCTACTTT	60.034	195
TRINITY_Contig3060	1	p3	(TGA)5	15	2075	2089	AGCCATTGGGTCTCACATGG	60.034	AGAGGTGGACCATCTTTGCG	60.036	234
TRINITY_Contig3062	1	p3	(CGA)4	12	109	120	AAACATTGTGGCAGCTGCTG	59.967	TTGCGTTCTCCTTTCTCGCT	59.966	267
TRINITY_Contig3062	2	p3	(GCC)4	12	374	385	AAACATTGTGGCAGCTGCTG	59.967	TTGCGTTCTCCTTTCTCGCT	59.966	267
TRINITY_Contig3064	1	p3	(GCC)4	12	70	81	CTAGCACCTGTCTATGCGGG	59.967	CAGGTTGCAGCAACAGTTCC	59.969	139
TRINITY_Contig3067	1	p5	(CAGCG)4	20	207	226	CTGCTTCTGCTTGAGCTTGC	60.11	TGGTGTCCTGCAGCTACAAG	59.965	122
TRINITY_Contig3067	2	p3	(CGG)5	15	339	353	CTGCTTCTGCTTGAGCTTGC	60.11	TGGTGTCCTGCAGCTACAAG	59.965	122
TRINITY_Contig3070	1	p4	(GAAG)3	12	2041	2052	CTTGAGGAAGAAGCCGCTCA	60.037	GGAGAAGAAGATCGCCGAGG	59.969	226
TRINITY_Contig3071	1	c	(CTCC)3caagaatgccagattatccactgcggcctcctcttct(TCC)4	61	4	64	TGGGACCTTGGCATTGATCC	60.033	CGCGCACAATGAGGAAGATG	59.973	131
TRINITY_Contig3071	2	p3	(CCG)5	15	213	227	TGGGACCTTGGCATTGATCC	60.033	CGCGCACAATGAGGAAGATG	59.973	131
TRINITY_Contig3072	1	c	(CTCC)3tggcggaaccttgcggtactccctcgcgttcacgtacacgttgaacttgacaaagtccgcggcatctgcctcgatgccctcgaccaccagca(CCT)4	116	365	480	CTATCGTCACCTTCGGCTCC	59.969	CCTTCACGACCCTGAAGCAT	60.036	109
TRINITY_Contig3072	2	p3	(GCG)4	12	1863	1874	CTATCGTCACCTTCGGCTCC	59.969	CCTTCACGACCCTGAAGCAT	60.036	109
TRINITY_Contig3073	1	p3	(GCG)4	12	2221	2232	CCCCACCGCCAAAGTAATCT	60.034	AAAGCAGAGGATTCCACCGG	60.035	197
TRINITY_Contig3076	1	p6	(GGCAGC)3	18	33	50	AAATACCGTGCTGTCCGGTT	59.964	GCTCCAAGTTCTCCAGCCTT	59.963	104
TRINITY_Contig3076	2	p3	(CCA)4	12	198	209	AAATACCGTGCTGTCCGGTT	59.964	GCTCCAAGTTCTCCAGCCTT	59.963	104
TRINITY_Contig3076	3	p5	(AGTGT)3	15	1146	1160	AAATACCGTGCTGTCCGGTT	59.964	GCTCCAAGTTCTCCAGCCTT	59.963	104
TRINITY_Contig3077	1	p2	(GC)9	18	342	359	TCGGACGTGGTGTTCAAGAG	59.968	CTCCTCCTCTGCTGCTGATG	59.895	168
TRINITY_Contig3077	2	p3	(GGA)4	12	1625	1636	TCGGACGTGGTGTTCAAGAG	59.968	CTCCTCCTCTGCTGCTGATG	59.895	168
TRINITY_Contig3078	1	p4	(TGTA)3	12	1874	1885	AAGAGATCGAGAGCGCCATG	59.968	CAGGTGTGACATGTCGTGGA	59.967	215
TRINITY_Contig3079	1	p3	(GCT)5	15	2137	2151	TCACCCTCCATGGTTTGTCG	59.964	CGGCAACCAATTCAGCAACA	59.968	243
TRINITY_Contig3082	1	c	(GGT)5ggggattggatgccgcctcctcccctctcctctccttccgttggaaggccaaaacgcatcgtgttcgcgacccgcgattcccacct(CCA)4	113	306	418	CCCTCCTCAACATGGTGCTT	59.961	CACCTCTGGCAAGCACACTA	59.965	219
TRINITY_Contig3082	2	p3	(GCG)6	18	1590	1607	CCCTCCTCAACATGGTGCTT	59.961	CACCTCTGGCAAGCACACTA	59.965	219
TRINITY_Contig3084	1	p3	(CCA)4	12	1378	1389	CTGCGAGAAGAACACCTGGT	59.966	GACCTCGATGATTTTGCGGC	59.972	234
TRINITY_Contig3084	2	p4	(GAGG)4	16	2933	2948	CTGCGAGAAGAACACCTGGT	59.966	GACCTCGATGATTTTGCGGC	59.972	234
TRINITY_Contig3086	1	p3	(CGA)4	12	257	268	CAACATCGTCACCCAGGTCA	59.965	GCATCCTCCGTGACAGGAAA	60.036	171
TRINITY_Contig3086	2	p4	(AGAT)3	12	1354	1365	CAACATCGTCACCCAGGTCA	59.965	GCATCCTCCGTGACAGGAAA	60.036	171
TRINITY_Contig3087	1	p3	(GGT)4	12	1020	1031	TCAGCTGAGCTTCATGTGCA	59.964	GTTTGGTAATCGGCATCGCC	59.971	109
TRINITY_Contig3088	1	p3	(GCT)4	12	101	112	ATTGTGGTGGGGTTCCGATC	60.034	TCGAAGCGGACGGAGAAAAG	60.388	125
TRINITY_Contig3088	2	p6	(AGGGCC)4	24	427	450	ATTGTGGTGGGGTTCCGATC	60.034	TCGAAGCGGACGGAGAAAAG	60.388	125
TRINITY_Contig3089	1	c	(GGGGT)4ggggagacctttcgcgctacctgttgagaacttgagatgaacgaaaaggtagacgatgctgctactaggcctctgttatttgttggggcatg(GATC)3	124	2143	2266	TCCGTTCAGAAGCATGGTCC	60.036	TCGCACTCCTCCTGTTGAAC	59.966	242
TRINITY_Contig3089	2	p4	(CTAG)3	12	2444	2455	TCCGTTCAGAAGCATGGTCC	60.036	TCGCACTCCTCCTGTTGAAC	59.966	242
TRINITY_Contig3091	1	p3	(CGC)4	12	955	966	CCAGCCAACGAACATGGTTG	60.039	ATTGGGTTCTTCCTGCGGAG	60.035	247
TRINITY_Contig3092	1	p3	(CGG)4	12	1075	1086	CCAAATTGGCCAAGCAGCTT	59.964	CAAGAAGTTCCCCGAGCAGT	59.965	157
TRINITY_Contig3093	1	p5	(TGCAA)3	15	1274	1288	TCCATGCAGCCAGTTCGATT	60.035	GGGTTGTTCCTCCCGTCAAT	59.962	272
TRINITY_Contig3094	1	p3	(TGA)4	12	2078	2089	GATCAGAATGCGGCGAACAC	59.973	TCCAACTCATGCATGTCCCC	60.034	113
TRINITY_Contig3095	1	p3	(CAC)4	12	2670	2681	GTGCTTGCACAAGTCACCTG	59.97	CACGTGATGCGCAGACAAAA	60.041	116
TRINITY_Contig3096	1	p5	(CTCCA)3	15	40	54	CGTACCCCTACTTCGCGTAC	59.972	TCAGGTTGCCGTTGTACCTC	59.966	276
TRINITY_Contig3096	2	p3	(CGA)4	12	896	907	CGTACCCCTACTTCGCGTAC	59.972	TCAGGTTGCCGTTGTACCTC	59.966	276
TRINITY_Contig3097	1	p3	(CTA)6	18	2741	2758	GGTCTCATGTCGCGATGGAT	59.967	GGAGTGGGCATCGAGACAAA	60.036	105
TRINITY_Contig3099	1	c	(TCG)4tcctccacggcctcagcgcagaggcgacgcgcgga(GCC)5gcaggtcgtcccggcgacgccggggcggccgatgcagttcttcgcgagccccgcg(CAC)4cagctcgtcgcgccgaggaggtcggtgccgtccaagtgggaggacgccgagaaatggctgcggcactcgtcggactccg(ACC)4	220	359	578	GTCCTCCACACGACGAAACT	59.968	GGTCTCCTTGAGGTTCAGGC	60.036	136
TRINITY_Contig3099	2	p3	(GCG)4	12	827	838	GTCCTCCACACGACGAAACT	59.968	GGTCTCCTTGAGGTTCAGGC	60.036	136
TRINITY_Contig3099	3	p4	(GCCG)3	12	1005	1016	GTCCTCCACACGACGAAACT	59.968	GGTCTCCTTGAGGTTCAGGC	60.036	136
TRINITY_Contig3099	4	p3	(TCC)6	18	1400	1417	GTCCTCCACACGACGAAACT	59.968	GGTCTCCTTGAGGTTCAGGC	60.036	136
TRINITY_Contig3100	1	p3	(GAA)4	12	371	382	CTCCTCAATCCGTCGTCGAG	59.972	GCCTCTCTAACACACACGCT	60.038	271
TRINITY_Contig3105	1	p4	(GGTA)4	16	155	170	TAGAGATGGGTTCAGCGGGA	60.033	CGTGCACTGGTCAAAACAGG	59.971	262
TRINITY_Contig3105	2	c	(AG)8ggggcgctcggctccccccacacgcaggcaggcgcaacaaacagacagagtagaggagagagaaagagggt(AG)7	101	2085	2185	TAGAGATGGGTTCAGCGGGA	60.033	CGTGCACTGGTCAAAACAGG	59.971	262
TRINITY_Contig3106	1	p4	(TTAT)3	12	2761	2772	TTCCGCGACTTTGATCAGCT	60.037	GAGAATCCTCCTGCGGTACG	59.969	273
TRINITY_Contig3108	1	p2	(TC)7	14	1	14	AAGGAGGAGATGGAGTGGCT	59.956	GCCTCCCATTTCCACGATGA	60.107	153
TRINITY_Contig3108	2	p4	(GCCG)3	12	1073	1084	AAGGAGGAGATGGAGTGGCT	59.956	GCCTCCCATTTCCACGATGA	60.107	153
TRINITY_Contig3110	1	p3	(AGC)4	12	1225	1236	ACCAGATGCTGCTCCAAGTC	60.036	ACCTGAAGCAGACTGTGACG	59.967	121
TRINITY_Contig3110	2	p3	(TCC)5	15	1748	1762	ACCAGATGCTGCTCCAAGTC	60.036	ACCTGAAGCAGACTGTGACG	59.967	121
TRINITY_Contig3110	3	p3	(GGA)6	18	1870	1887	ACCAGATGCTGCTCCAAGTC	60.036	ACCTGAAGCAGACTGTGACG	59.967	121
TRINITY_Contig3112	1	p4	(CCCA)3	12	44	55	AATGCTTCATTCGGGCCTCA	60.034	CCTGGATGAAGGCATGGGTT	60.033	139
TRINITY_Contig3115	1	p3	(CGG)4	12	540	551	CCGAAACTCCGAATCCACCA	60.037	CAAACCAACAGCCAACGGAG	59.969	194
TRINITY_Contig3115	2	p3	(CGG)4	12	2330	2341	CCGAAACTCCGAATCCACCA	60.037	CAAACCAACAGCCAACGGAG	59.969	194
TRINITY_Contig3115	3	p3	(CGC)4	12	2594	2605	CCGAAACTCCGAATCCACCA	60.037	CAAACCAACAGCCAACGGAG	59.969	194
TRINITY_Contig3116	1	c	(CCTCCG)3ctcctcttcc(CCT)4	40	343	382	TGGTGAAGGCCTACTGCAAG	59.963	CGGAGTAGATGTCGAGCACC	59.97	261
TRINITY_Contig3116	2	p3	(GAG)4	12	1124	1135	TGGTGAAGGCCTACTGCAAG	59.963	CGGAGTAGATGTCGAGCACC	59.97	261
TRINITY_Contig3117	1	p2	(AG)7	14	200	213	ACGGCTCAGTACTCCAGACT	59.962	GCTGTCCCGATAAGGCTCTC	59.967	128
TRINITY_Contig3117	2	c	(CGC)4ccgcgccgcatcgtcgtcatacgccgccgggcttctcgctcatctcgtggagcccggagcccacgga(GCC)4	91	567	657	ACGGCTCAGTACTCCAGACT	59.962	GCTGTCCCGATAAGGCTCTC	59.967	128
TRINITY_Contig3117	3	p3	(GTG)5	15	832	846	ACGGCTCAGTACTCCAGACT	59.962	GCTGTCCCGATAAGGCTCTC	59.967	128
TRINITY_Contig3119	1	p4	(GAGG)3	12	329	340	ACTGATTCTGCAGCATGGCT	60.034	TGCAGCCTGTTTCCCCATAG	60.034	125
TRINITY_Contig3120	1	p3	(CGC)4	12	3290	3301	GCTAGCATGGCCATGTTTCG	59.97	TTGGGGAACTTGGGCACTTT	60.032	230
TRINITY_Contig3121	1	p3	(CGC)4	12	4186	4197	CCACTCCAGGGACCCATAGA	60.031	ATGGCCAAGCAGTACAGTCC	60.035	238
TRINITY_Contig3122	1	p3	(GCA)4	12	970	981	AGTTGCCATCCCATGCATCA	60.033	CTCAACAGTGCTGCCCAAAC	59.969	108
TRINITY_Contig3124	1	p5	(TGCTT)4	20	1802	1821	CAGAAACTGCAGTGCCAACC	59.969	GCAGTTGCTCAAGTGGTGTG	59.97	198
TRINITY_Contig3125	1	p6	(AAGAAA)3	18	1779	1796	AAACGCCAGAGGATTCAGGG	60.035	CCAGCCGGATGATGATCTCC	60.037	165
TRINITY_Contig3127	1	p4	(GATG)3	12	953	964	ATGAGTCGGGATCCCACTCA	60.032	CCGGTAGGAGCTATGCTGTG	59.967	154
TRINITY_Contig3127	2	p3	(GCT)4	12	1276	1287	ATGAGTCGGGATCCCACTCA	60.032	CCGGTAGGAGCTATGCTGTG	59.967	154
TRINITY_Contig3127	3	c	(GCG)4ctggaggtctcgacggtgtgccagccccctcctgaagacggtggccccgccgccccccgag(AGC)4	84	1676	1759	ATGAGTCGGGATCCCACTCA	60.032	CCGGTAGGAGCTATGCTGTG	59.967	154
TRINITY_Contig3128	1	p3	(CAG)4	12	556	567	TCTCCTTCCTCCTCGGACTG	60.034	CGACGGTGACTGATACCGAG	59.972	273
TRINITY_Contig3129	1	p3	(GCC)4	12	590	601	GCCATGCTCTACGACATCGA	59.969	TCAAGGAACCGGAACTGCTC	59.965	113
TRINITY_Contig3129	2	p3	(TTC)4	12	968	979	GCCATGCTCTACGACATCGA	59.969	TCAAGGAACCGGAACTGCTC	59.965	113
TRINITY_Contig3130	1	p5	(TGCTC)3	15	6128	6142	TCCGCTCCCTAGGATGAACA	60.033	CGTATGATGACCCGAAGCGA	59.97	172
TRINITY_Contig3131	1	p3	(CTG)4	12	751	762	CAGCCACACAGCCAAAAGTC	59.969	TAGGCCCGACATTCCTGGTA	60.032	225
TRINITY_Contig3133	1	p3	(GGC)4	12	38	49	ATCCACCTACCCGGAGAGTC	60.106	TCCCAAGACAGAGCACAAGC	60.25	120
TRINITY_Contig3134	1	p2	(GA)8	16	252	267	GCTGGAAGGTTACAGGCTGT	59.963	AAGGCCAATTTTCCGTGCAC	59.967	225
TRINITY_Contig3136	1	p3	(GGA)5	15	533	547	GAGTTCGCCGCTGATATGGA	59.968	ACATCGCTTCCGTCGATCTC	59.97	155
TRINITY_Contig3136	2	p3	(GAC)4	12	873	884	GAGTTCGCCGCTGATATGGA	59.968	ACATCGCTTCCGTCGATCTC	59.97	155
TRINITY_Contig3136	3	p3	(GCC)5	15	1440	1454	GAGTTCGCCGCTGATATGGA	59.968	ACATCGCTTCCGTCGATCTC	59.97	155

TRINITY_Contig3137	1	c	(GCT)7tgcgccgcgccgcggcggagcgcggatcccgagatccccggcggcgccgccccggagctcgc(CGG)4	95	153	247	AGGGTCCTGTTGGCAAACAA	60.033	ATTGCCTGCTGCCATCTTCT	60.033	152
TRINITY_Contig3137	2	c	(CCG)4(CCA)4	24	3058	3081	AGGGTCCTGTTGGCAAACAA	60.033	ATTGCCTGCTGCCATCTTCT	60.033	152
TRINITY_Contig3141	1	p3	(TCC)4	12	38	49	GTTCCTGCAACAAGAAGCCG	60.04	CCCACTTGCATACCTGGTGT	59.962	184
TRINITY_Contig3141	2	p3	(CTC)6	18	170	187	GTTCCTGCAACAAGAAGCCG	60.04	CCCACTTGCATACCTGGTGT	59.962	184
TRINITY_Contig3142	1	p3	(GCC)5	15	2049	2063	TGATCGGCAACAGCTTGTCT	59.965	CCGTCGAGGAGGGTTTGAAA	59.966	199
TRINITY_Contig3143	1	p4	(TAAA)3	12	230	241	CGTGAAGCTTGCGTATCACG	59.976	TCGGGGAAGAACACTCCTCT	59.885	154
TRINITY_Contig3143	2	p3	(CCG)4	12	1622	1633	CGTGAAGCTTGCGTATCACG	59.976	TCGGGGAAGAACACTCCTCT	59.885	154
TRINITY_Contig3145	1	p3	(GCC)4	12	32	43	AGGATGGTGGGTCATTGCTG	60.034	TAGGCACTTGCAACCTACGG	60.037	192
TRINITY_Contig3150	1	p4	(ATCA)3	12	129	140	CGAGGTAGATGACGTCACCG	59.972	TCTCCCGGTCTCGAACATCT	60.034	251
TRINITY_Contig3152	1	c	(GCG)4gctcggatcagcggcgcgcagtgggcggagcggaggcggacggaccctccctgcgacctccgctagccc(CT)6	94	1080	1173	GTGGAGGTCGAGTAGGAGGT	60.034	GCCACCTCCTTTCTTCCTCC	60.035	139
TRINITY_Contig3153	1	p4	(GCCG)3	12	42	53	ATAGTGCTACTGGTGGCGTG	59.824	AAATCTCACGCACACGGACT	59.966	210
TRINITY_Contig3154	1	p3	(TGG)4	12	295	306	AAGAGATGGCGGCAAATCCA	60.034	GGAGATCCCTAACGGCAAGG	59.893	262
TRINITY_Contig3154	2	p3	(TAC)4	12	1347	1358	AAGAGATGGCGGCAAATCCA	60.034	GGAGATCCCTAACGGCAAGG	59.893	262
TRINITY_Contig3156	1	p3	(GGA)4	12	400	411	TCATCGTCGTGTCCGTCTTC	59.833	GTGAGCCTGAGGTCTGACAC	60.038	171
TRINITY_Contig3157	1	c	(GGC)4cgtccgcgcggcggtgaagaggcagttgccgtcggcgtcgacctccccgcgtggtcgaggcggaagaccacggg(CGC)4	99	1090	1188	TCAGTCGCCAGGCATTCAAT	60.035	CACCCTGCAGTACACAGAGG	60.037	105
TRINITY_Contig3157	2	c	(GCG)4gggcgggggcgggggacggcggcagagcctcggcga(CGG)4	60	1356	1415	TCAGTCGCCAGGCATTCAAT	60.035	CACCCTGCAGTACACAGAGG	60.037	105
TRINITY_Contig3158	1	p6	(GGGGAC)3	18	219	236	AAGCTATCCTGCAGGGGAGA	60.031	GCAGGGGGTGTACCAAGTAC	60.036	161
TRINITY_Contig3159	1	p2	(AG)7	14	41	54	CTAGCGCGATCGATCTCTCC	59.904	AGGGCGCCCATGATGATATG	60.035	217
TRINITY_Contig3159	2	p3	(CTC)8	24	941	964	CTAGCGCGATCGATCTCTCC	59.904	AGGGCGCCCATGATGATATG	60.035	217
TRINITY_Contig3159	3	c	(CCT)5gctgctcgcgcacggcgacgcgccgg(TGCCGC)3	59	1847	1905	CTAGCGCGATCGATCTCTCC	59.904	AGGGCGCCCATGATGATATG	60.035	217
TRINITY_Contig3160	1	p4	(CCGC)3	12	1219	1230	TGTGGGTTGGCTCTTGGATC	59.962	AGGAATGGTTCTCACAGCGG	60.036	126
TRINITY_Contig3161	1	p3	(CCG)4	12	1675	1686	GAACACCGCTGTCAGAGTGA	59.968	TCCATTGGCCTGAACATCCC	60.033	240
TRINITY_Contig3164	1	p3	(CAT)4	12	234	245	TTGGAGATTTGCGCCTGGAT	60.034	GCTTCGTCGGGAGTAGGATG	59.969	167
TRINITY_Contig3164	2	c	(GAA)6(GAT)8	42	619	660	TTGGAGATTTGCGCCTGGAT	60.034	GCTTCGTCGGGAGTAGGATG	59.969	167
TRINITY_Contig3168	1	p2	(TG)11	22	3105	3126	TAGCGGCTTCAGTCTTGTGG	60.037	TGAGAATCTGGGAGAGGGGG	60.031	115
TRINITY_Contig3169	1	p3	(TCG)4	12	31	42	AAGGTTCAGCTGTGGTACCG	59.965	TTTGGGTTCGTCCTCGTCAG	59.967	227
TRINITY_Contig3169	2	p3	(CGG)4	12	166	177	AAGGTTCAGCTGTGGTACCG	59.965	TTTGGGTTCGTCCTCGTCAG	59.967	227
TRINITY_Contig3171	1	p3	(GAA)4	12	655	666	CACCCTAAACCTCGTCGGAG	59.827	AACACTTCACCAGGCCAACA	60.034	142
TRINITY_Contig3175	1	p3	(AGG)4	12	1914	1925	TTCTGAATGATGGGTGGGGC	60.033	TGTAGCAGGGTTCATGCCAG	60.035	217
TRINITY_Contig3176	1	p6	(GGCGGA)3	18	1544	1561	TCGCCAAACATCCTCGCTCAT	60.035	TCTTCGCATCACTGGTCACC	60.037	238
TRINITY_Contig3177	1	c	(AGC)5gggcggag(GCC)4	35	2018	2052	TGCTTCCCGTCGACATGAAA	59.966	ACATTGGGGCACATCAGAG	60.034	172
TRINITY_Contig3178	1	p4	(AATG)3	12	1026	1037	GCCAACAACAACATCCCACC	59.967	ACTGTCGGGGTTTGGACATC	59.964	266
TRINITY_Contig3180	1	p3	(CGG)6	18	3313	3330	CCGTGGGTCATAGCGTAGTC	59.97	TTGCAGAAAACAAGAGCGCC	59.969	114
TRINITY_Contig3180	2	c	(TCT)7tcagttcgtcgttggatccaactccaaggtccaaaccacaccag(AGCC)3	77	4360	4436	CCGTGGGTCATAGCGTAGTC	59.97	TTGCAGAAAACAAGAGCGCC	59.969	114
TRINITY_Contig3182	1	p3	(CCA)4	12	1248	1259	TGAGCACCTTGTGACGGTAC	59.967	TCAGCAAGGGCCAGGTTATG	60.034	195
TRINITY_Contig3183	1	p3	(CGC)4	12	1076	1087	GGTACTCGCAAACACAGGGA	59.966	GCTCAAAGCTTCAAACCGCA	59.969	101
TRINITY_Contig3183	2	p3	(CGC)4	12	1201	1212	GGTACTCGCAAACACAGGGA	59.966	GCTCAAAGCTTCAAACCGCA	59.969	101
TRINITY_Contig3185	1	p3	(TGC)4	12	893	904	TACCCTGATTGGCGCAACAT	60.035	TTCACGGAGGCGAACATGAA	59.966	177
TRINITY_Contig3185	2	p3	(GCG)4	12	1091	1102	TACCCTGATTGGCGCAACAT	60.035	TTCACGGAGGCGAACATGAA	59.966	177
TRINITY_Contig3189	1	p4	(AGCA)3	12	337	348	GCAACGCCAAACTTACAGCA	59.97	AGTAGACGTGCTTGCTTGCT	59.966	138
TRINITY_Contig3190	1	p2	(AT)7	14	52	65	GGTGTTTGTGTTTCCCTCGC	59.97	ATGACCGACCGTCGTTTCTC	60.109	205
TRINITY_Contig3190	2	p4	(AGAA)3	12	788	799	GGTGTTTGTGTTTCCCTCGC	59.97	ATGACCGACCGTCGTTTCTC	60.109	205
TRINITY_Contig3191	1	p4	(CAAG)3	12	310	321	AAGGTTGGGGCAACAAGGAA	60.032	AATGCTTCCTTCCTCTGGCC	60.034	125
TRINITY_Contig3191	2	p4	(GCCG)3	12	430	441	AAGGTTGGGGCAACAAGGAA	60.032	AATGCTTCCTTCCTCTGGCC	60.034	125
TRINITY_Contig3191	3	p3	(GCC)4	12	559	570	AAGGTTGGGGCAACAAGGAA	60.032	AATGCTTCCTTCCTCTGGCC	60.034	125
TRINITY_Contig3191	4	p6	(GACGCG)3	18	724	741	AAGGTTGGGGCAACAAGGAA	60.032	AATGCTTCCTTCCTCTGGCC	60.034	125
TRINITY_Contig3191	5	p3	(GCT)4	12	2842	2853	AAGGTTGGGGCAACAAGGAA	60.032	AATGCTTCCTTCCTCTGGCC	60.034	125
TRINITY_Contig3192	1	p5	(AGACC)3	15	118	132	AATGCTTCCTTCCTCTGGCC	60.034	AAGGTTGGGGCAACAAGGAA	60.032	125
TRINITY_Contig3192	2	p3	(CAG)4	12	863	874	AATGCTTCCTTCCTCTGGCC	60.034	AAGGTTGGGGCAACAAGGAA	60.032	125
TRINITY_Contig3192	3	p6	(GCGTCC)3	18	2947	2964	AATGCTTCCTTCCTCTGGCC	60.034	AAGGTTGGGGCAACAAGGAA	60.032	125
TRINITY_Contig3195	1	p3	(GCG)4	12	2132	2143	TACCGCTTCTTCTTGCCGTT	59.966	ACCACCGCCTCCCTAATACT	60.031	269
TRINITY_Contig3195	2	p3	(GCG)4	12	2517	2528	TACCGCTTCTTCTTGCCGTT	59.966	ACCACCGCCTCCCTAATACT	60.031	269
TRINITY_Contig3195	3	p3	(TCG)7	21	3435	3455	TACCGCTTCTTCTTGCCGTT	59.966	ACCACCGCCTCCCTAATACT	60.031	269
TRINITY_Contig3195	4	c	(TCA)4ttgaagccgaggaggaacgggtcgggtacctgcgacgaggacctcgccggcgcggccaacgcca(GCG)4gccgggtccctgctgctggtgctggtacccgtaggcgcacgctgtcgcggccatgcacgccacccgggcgtg(GC)6gtagtgaggcgaactcgccctcgtccttgttgcgggcgtgccgggccctgctgtgcccgtctggcgccctgtattataggtgggcgt(GGGGGC)4	284	3891	4174	TACCGCTTCTTCTTGCCGTT	59.966	ACCACCGCCTCCCTAATACT	60.031	269
TRINITY_Contig3197	1	p3	(AGC)5	15	2311	2325	ACCCAACCCTCGGATGGATA	60.031	TGAGGGAGAGACGGAGACAG	60.035	184
TRINITY_Contig3197	2	c	(GTTT)3cgttggtctatatataattgttgctgcagtggatggcaatggcatgtttcttcctgcaggcagcatgcatgacttgtctgccaacccaa(CCAAAC)3ccaaccctcggatggatatacagctagctaggca(GTTT)3atattaagccaagcaaggca(TGCC)4cttcaa(TTCC)3	219	2747	2965	ACCCAACCCTCGGATGGATA	60.031	TGAGGGAGAGACGGAGACAG	60.035	184
TRINITY_Contig3197	3	p3	(ACG)4	12	3234	3245	ACCCAACCCTCGGATGGATA	60.031	TGAGGGAGAGACGGAGACAG	60.035	184
TRINITY_Contig3198	1	p4	(CCTC)3	12	14	25	GTGGACCTATTGTCGCCCAA	60.036	TGCAGTGTGCCTTCTCTCAG	59.966	162
TRINITY_Contig3201	1	p4	(CCCG)3	12	1243	1254	CGATGGAACGATGCCTGAGA	59.897	AAGCGTACAGGTTCCTCGTG	60.039	273
TRINITY_Contig3201	2	p4	(CCGC)3	12	1371	1382	CGATGGAACGATGCCTGAGA	59.897	AAGCGTACAGGTTCCTCGTG	60.039	273
TRINITY_Contig3202	1	c	(CAAGG)3ggcgatgaatggtgatgcaccgactgatgctgggacgcagcgcggctttacttaaacaagtggtggctat(TGCGGC)3tgctgccgcggccgcgcgggagggggagcgtgggtctgcggcgcctgcggcggcgggggctgcggccgctgctgcttgggcttggggcggtta(TGC)4	208	1335	1542	AAGCAAGAGGAAAGACCCCG	59.963	GTGCATCACCATTCATCGCC	59.97	156
TRINITY_Contig3202	2	p3	(GGC)4	12	1702	1713	AAGCAAGAGGAAAGACCCCG	59.963	GTGCATCACCATTCATCGCC	59.97	156
TRINITY_Contig3204	1	p4	(AAAG)3	12	5	16	TCAGTATCCTGGCATCCCCA	60.03	TGGCAAAGAACAATGGCACG	59.968	144
TRINITY_Contig3206	1	p3	(GGA)4	12	1885	1896	GTCATGCTTAGGCGGCAATG	59.97	ACGACAAGGAGGGTTCGTTC	59.967	117
TRINITY_Contig3207	1	p4	(CCTT)3	12	404	415	TGCTTGATTGCGATCCCCTT	60.034	AGTCTCGCATGGCATCCAAA	60.035	115
TRINITY_Contig3209	1	c	(CGG)4(AGG)4	24	2106	2129	GCATCAATCTCAGCGTGCAG	59.972	CGTTGGAGCTGCATGAATCG	59.973	190
TRINITY_Contig3212	1	p3	(CCG)4	12	921	932	GGCAATTGCGAGAATCCGTC	59.972	CTCCGCCTTGGTGAAGATGT	60.036	130
TRINITY_Contig3212	2	p3	(GAC)4	12	1125	1136	GGCAATTGCGAGAATCCGTC	59.972	CTCCGCCTTGGTGAAGATGT	60.036	130
TRINITY_Contig3214	1	p3	(CGC)4	12	1105	1116	GCCCCACCATCTCCTTGTAC	60.107	CTGGAGCCCAAGGTGATCTC	59.82	220
TRINITY_Contig3215	1	p3	(GAC)4	12	439	450	GCCGAGCTCGTACATCTTCA	59.899	ACTACCAGGACCTCCCGTAC	60.034	164
TRINITY_Contig3215	2	p3	(GGT)5	15	1442	1456	GCCGAGCTCGTACATCTTCA	59.899	ACTACCAGGACCTCCCGTAC	60.034	164
TRINITY_Contig3215	3	p3	(CGC)4	12	3063	3074	GCCGAGCTCGTACATCTTCA	59.899	ACTACCAGGACCTCCCGTAC	60.034	164
TRINITY_Contig3215	4	p2	(CG)6	12	3349	3360	GCCGAGCTCGTACATCTTCA	59.899	ACTACCAGGACCTCCCGTAC	60.034	164
TRINITY_Contig3215	5	p4	(GCAC)3	12	3519	3530	GCCGAGCTCGTACATCTTCA	59.899	ACTACCAGGACCTCCCGTAC	60.034	164
TRINITY_Contig3217	1	c	(CCT)5cagcactcctatatgtcctcaagagcacgcgcaccatccccagggacccg(GCC)4	77	1394	1470	CAAGAACCACAGCTGCCAAC	59.969	CATGTTCATCCGCTGCTTCG	59.973	207
TRINITY_Contig3218	1	p2	(TG)7	14	2434	2447	AAGCAAGTCGAGAAGAGCCC	60.037	GTTGAGGGCTTCTCTGGACC	60.036	237
TRINITY_Contig3219	1	p3	(CCG)5	15	300	314	CTGATCATTGCGCTTCTGGC	59.971	ACTCCAAAAGGACCGTCGAC	59.967	131
TRINITY_Contig3219	2	p3	(CGC)5	15	2167	2181	CTGATCATTGCGCTTCTGGC	59.971	ACTCCAAAAGGACCGTCGAC	59.967	131
TRINITY_Contig3220	1	c	(TGA)4tgctgtactctcaagtctcaactagcagacgagtcaggtaacaccaccccctactcttctcgcaaacgcaaat(CAC)7	106	114	219	CGCATGAAAGCACGTATGGG	59.972	GGGTGGTGTTACCTGACTCG	60.038	169
TRINITY_Contig3220	2	p4	(GCGG)3	12	1612	1623	CGCATGAAAGCACGTATGGG	59.972	GGGTGGTGTTACCTGACTCG	60.038	169
TRINITY_Contig3221	1	p4	(CCAG)5	20	187	206	CGACAATACTCTCCTCGCCC	59.969	GGGAGGAAAGAACGAGGTGG	60.036	133
TRINITY_Contig3221	2	p3	(GGC)5	15	413	427	CGACAATACTCTCCTCGCCC	59.969	GGGAGGAAAGAACGAGGTGG	60.036	133

TRINITY_Contig3223	1	p3	(CCG)4	12	412	423	GTTGCTGTGGATGTTTGCGT	59.969	GCCCACACAGTCTGCATTTG	60.039	271
TRINITY_Contig3223	2	p4	(ATTT)3	12	3018	3029	GTTGCTGTGGATGTTTGCGT	59.969	GCCCACACAGTCTGCATTTG	60.039	271
TRINITY_Contig3224	1	p5	(CCTCG)3	15	59	73	GGCCTTCAAATGGTTGCCTG	60.037	GTACCTCATGCGGGAGTACG	59.97	219
TRINITY_Contig3225	1	p5	(TCCCC)3	15	8	22	CAGGCAACCACTTTTCAGCC	59.967	GGGTTCAGGACGCTCAAAGA	59.965	258
TRINITY_Contig3225	2	p4	(AAGG)5	20	223	242	CAGGCAACCACTTTTCAGCC	59.967	GGGTTCAGGACGCTCAAAGA	59.965	258
TRINITY_Contig3225	3	p3	(CAG)4	12	3245	3256	CAGGCAACCACTTTTCAGCC	59.967	GGGTTCAGGACGCTCAAAGA	59.965	258
TRINITY_Contig3225	4	p3	(ATG)4	12	6343	6354	CAGGCAACCACTTTTCAGCC	59.967	GGGTTCAGGACGCTCAAAGA	59.965	258
TRINITY_Contig3227	1	p3	(CCG)4	12	744	755	TCGTCGAAGAAGCCGTTGAA	59.969	TCGAGCCTGACAACCCAATC	60.036	190
TRINITY_Contig3228	1	p3	(CCT)5	15	8	22	CATCACGAGGCATGTGCATG	59.971	TTGTGGTCTGTACTCCGCAC	59.967	149
TRINITY_Contig3228	2	p5	(CACCT)3	15	233	247	CATCACGAGGCATGTGCATG	59.971	TTGTGGTCTGTACTCCGCAC	59.967	149
TRINITY_Contig3228	3	p3	(CCG)6	18	1189	1206	CATCACGAGGCATGTGCATG	59.971	TTGTGGTCTGTACTCCGCAC	59.967	149
TRINITY_Contig3229	1	p2	(GA)6	12	4902	4913	CACCAGTGGGCCACAATTTG	59.966	TCTGCTGGAGTTCCTTGCTG	59.964	195
TRINITY_Contig3230	1	p3	(CGG)5	15	912	926	TGCGTCAGAACTCACTCACC	59.968	CACGAAGACCAAAACGGCTG	60.041	108
TRINITY_Contig3232	1	p3	(TCC)4	12	109	120	CGTCGTACAAGGTGACACCA	59.969	TTTGCTTGTTCTGCGCCTTC	59.969	258
TRINITY_Contig3233	1	p3	(GCT)4	12	1593	1604	TGCGGCTGTTTGTCATTGTG	59.969	TTGACCCTGATCGGATCCT	60.031	132
TRINITY_Contig3235	1	p3	(CGC)5	15	229	243	TGCCATACCATCCTCTCCCA	60.03	ATTAGGGAGGCCATGGGAGT	60.029	224
TRINITY_Contig3236	1	p3	(GGC)5	15	3637	3651	GTCGCACCCAACAACAACTC	59.971	AGTTGTTGTCAGGGCTCGAG	59.966	208
TRINITY_Contig3236	2	p3	(CCG)4	12	4299	4310	GTCGCACCCAACAACAACTC	59.971	AGTTGTTGTCAGGGCTCGAG	59.966	208
TRINITY_Contig3237	1	c	(TC)8cccttccgcgttcccgtccgcgcgagcg gtgcgtccacacggtctgatgccggcgccgcc cggccagtaaagcgagagggacacgccgcgg gggc(GCGA)3gcggggagcgggagcgcg atga(CGC)4	157	78	234	TCCCTCATGGAGCACTACGA	60.034	CCGCCAGGGAAGTTGATCTT	60.035	215
TRINITY_Contig3237	2	c	(TC)8cccttccgcgttcccgtccgcgcgagcg gtgcgtccacacggtctgatgccggcgccgcc cggccagtaaagcgagagggacacgccgcgg gggc(GCGA)3gcggggagcgggagcgcg atga(CGC)4	157	1841	1997	TCCCTCATGGAGCACTACGA	60.034	CCGCCAGGGAAGTTGATCTT	60.035	215
TRINITY_Contig3238	1	p2	(AG)7	14	63	76	CCTCAGCTCCCCTCCACTAT	60.105	TGCTATGCTATCGGCTTGG	59.965	154
TRINITY_Contig3238	2	c	(CAG)4caagct(CAC)4	30	253	282	CCTCAGCTCCCCTCCACTAT	60.105	TGCTATGCTATCGGCTTGG	59.965	154
TRINITY_Contig3238	3	p3	(CGG)5	15	456	470	CCTCAGCTCCCCTCCACTAT	60.105	TGCTATGCTATCGGCTTGG	59.965	154
TRINITY_Contig3238	4	c	(CAC)5cagcagctcgagcacgagcatcagc gcaccaagcatgtcacagcactacagctatttt(CGGCAG)3	90	786	875	CCTCAGCTCCCCTCCACTAT	60.105	TGCTATGCTATCGGCTTGG	59.965	154
TRINITY_Contig3243	1	p3	(CGC)4	12	147	158	TGATGCAGCAGTGAACTCGT	59.966	GTCGCCCACACTTTTGGAAC	59.97	253
TRINITY_Contig3245	1	p3	(CGC)4	12	937	948	CTGGTGAACTGCGACCTCAT	60.037	CGGCCGATATCATCACACGA	60.04	153
TRINITY_Contig3247	1	c	(GCC)4atgcctcccgctcccgcacacgagct cgcg(GCC)4	54	360	413	CAAGGTCGCTGTGATCACCT	60.037	GCTACACACAAGCAGGGCTA	60.036	130
TRINITY_Contig3248	1	p3	(CGC)4	12	3937	3948	TGACATGCCAAGGTAGCAG	60.035	TGACAAGGCATCAGAGCGTT	59.965	229
TRINITY_Contig3248	2	p3	(CGG)5	15	4183	4197	TGACATGCCAAGGTAGCAG	60.035	TGACAAGGCATCAGAGCGTT	59.965	229
TRINITY_Contig3250	1	p4	(GGAG)4	16	619	634	TACGGTATCGATTGGGCACG	59.969	AGATACCGGCGCGTTTAGAG	59.97	150
TRINITY_Contig3253	1	p5	(GGAAG)3	15	2897	2911	CCGCTCGCTCTCTCTAGTTG	59.971	AAACTTTTCTCACGCGCACC	59.971	123
TRINITY_Contig3254	1	p3	(GGC)4	12	1135	1146	CTCAGCGCAAAGTCCAAACC	60.04	AGGTGGGGACAATGATGCTG	60.034	162
TRINITY_Contig3254	2	p3	(GGC)4	12	1322	1333	CTCAGCGCAAAGTCCAAACC	60.04	AGGTGGGGACAATGATGCTG	60.034	162
TRINITY_Contig3256	1	p3	(TCT)4	12	146	157	TACACGGACGTTGCATTGGA	59.966	AACACGGCCATCCATCATGT	60.034	174
TRINITY_Contig3256	2	p3	(GGC)4	12	539	550	TACACGGACGTTGCATTGGA	59.966	AACACGGCCATCCATCATGT	60.034	174
TRINITY_Contig3258	1	p3	(GGC)6	18	2494	2511	CAACGGGCACAATTCAGCAA	59.968	CCCACTGCGATTGACCTCTT	60.036	231
TRINITY_Contig3261	1	p3	(GGC)6	18	333	350	TTTGCAACGAAGCCATGTGG	59.968	GTATCATTTGGCGACCGTGC	59.972	235
TRINITY_Contig3261	2	p3	(CCG)4	12	3368	3379	TTTGCAACGAAGCCATGTGG	59.968	GTATCATTTGGCGACCGTGC	59.972	235
TRINITY_Contig3261	3	p5	(GCCGC)3	15	3805	3819	TTTGCAACGAAGCCATGTGG	59.968	GTATCATTTGGCGACCGTGC	59.972	235
TRINITY_Contig3263	1	p3	(AAG)4	12	1313	1324	TTGGCATTAACCTGGACGCT	59.962	ATCTCTCACACATCCCCCGA	60.032	104
TRINITY_Contig3264	1	p6	(AGAATC)3	18	1167	1184	ACCATCCATCGCACGAACTT	60.036	GCGTGCTATTGTCGACGTTC	59.977	138
TRINITY_Contig3264	2	p3	(GCG)9	27	3796	3822	ACCATCCATCGCACGAACTT	60.036	GCGTGCTATTGTCGACGTTC	59.977	138
TRINITY_Contig3265	1	c	(AGT)4tagcacctagcagtagaactgtaga agtagtaccaacccaacccaaatccgctgttg aaattggtggcgcggcaagtgaaagtttggta gg(GGGC)3	115	446	560	GCTTCAAGCACACCAACCAG	59.969	GATGTCGATGATGCCCGAGT	59.967	272
TRINITY_Contig3267	1	p6	(AGGCGA)3	18	1431	1448	ACTGCCTCGAGGTTTACTGC	60.037	GAAGCGCGCTTGGTAAACAA	60.041	206
TRINITY_Contig3268	1	p3	(CGC)4	12	133	144	AGCAACCTCCAGGATCGTTG	60.036	TGCGGTGCACAAAGTGATTG	59.969	107
TRINITY_Contig3269	1	p3	(CGG)4	12	1540	1551	GAAGTGCCATCTCCTGTGCT	60.036	GAAACAAAGCTTGGGGCCTG	59.966	137
TRINITY_Contig3271	1	p3	(CGG)4	12	3125	3136	CTAGCTGACCCGAATCGACC	59.969	ATCCAACAGCACCATCCAGG	60.034	185
TRINITY_Contig3271	2	p4	(GCCG)4	16	3343	3358	CTAGCTGACCCGAATCGACC	59.969	ATCCAACAGCACCATCCAGG	60.034	185
TRINITY_Contig3273	1	p5	(CAAGG)3	15	1762	1776	TACATCCCCCTCGCTCAAGA	60.033	TCTGCAGACTGTGGAACGAC	59.968	148
TRINITY_Contig3280	1	p3	(CCG)4	12	3622	3633	AGGAGACGAGAAGGGAGGTC	60.034	TTGAGGAACGATGTGGGGTG	59.964	151
TRINITY_Contig3285	1	p2	(TG)8	16	429	444	AGAGCTAGCTAGGTGGCACT	60.033	GCACAACTCCACTTCGAGGA	59.966	254
TRINITY_Contig3287	1	p4	(CCGG)3	12	5758	5769	GTTTGCCAAGGCTGTCAAGG	59.967	CGACGATGTTGGGCGATTTC	59.974	242
TRINITY_Contig3290	1	c	(GCAAGG)3gcaaagccatggccagcatcg tggaa(GAG)4	56	184	239	ATCGCGATGATGACGACGAA	59.971	TCCGGGCTAATCTCCTCCAA	60.032	120
TRINITY_Contig3290	2	p3	(CTC)4	12	1203	1214	ATCGCGATGATGACGACGAA	59.971	TCCGGGCTAATCTCCTCCAA	60.032	120
TRINITY_Contig3292	1	p4	(GCTC)3	12	2518	2529	GAACTCTCACTGGTTCCGCA	59.966	TGGACCTTTGGTGTTTGGCT	60.033	269
TRINITY_Contig3293	1	p3	(GAA)4	12	502	513	ATCCACGAACCGATACTCGC	59.97	TGATCGGTACCCTCACTGGT	59.959	195
TRINITY_Contig3297	1	p2	(GT)7	14	270	283	GAAAGCGGAGGAAGGAACCA	59.964	TGCTCTCCTCCTCCACTACC	60.033	155
TRINITY_Contig3297	2	p2	(AC)7	14	730	743	GAAAGCGGAGGAAGGAACCA	59.964	TGCTCTCCTCCTCCACTACC	60.033	155
TRINITY_Contig3297	3	p3	(TCC)6	18	1009	1026	GAAAGCGGAGGAAGGAACCA	59.964	TGCTCTCCTCCTCCACTACC	60.033	155
TRINITY_Contig3297	4	p3	(GCG)5	15	1990	2004	GAAAGCGGAGGAAGGAACCA	59.964	TGCTCTCCTCCTCCACTACC	60.033	155
TRINITY_Contig3299	1	p3	(CGG)5	15	1517	1531	CTGCACGTTTCCAGTGGTTG	59.971	AGGTACCGGCATAGCGATTG	59.966	100
TRINITY_Contig3300	1	c	(CGG)4aaggaggcccccgccgctgctgcg gtcgtggctcttggg(CGA)5	67	2384	2450	TAACCCGATCCCCATCCCTT	60.029	CGATGGCCTGGGTGTAGATC	59.966	210
TRINITY_Contig3300	2	c	(CGC)5ggggaaggagatcatcacctcgcgc tcgcgcgcgtcgtcccccgccgcgcgcgccac ca(GCG)4tgaccggcagcaggaacggcttg cggtccaggtcgaggtactccaggtcgtacag cagccccgcgcacttggccgc(CGCCGG)3	180	3264	3443	TAACCCGATCCCCATCCCTT	60.029	CGATGGCCTGGGTGTAGATC	59.966	210
TRINITY_Contig3301	1	p3	(GCG)4	12	1061	1072	TCAGCTGGCTCTTCTTGCTC	60.037	CTCGACCTGGATCGTAAGCC	59.969	217
TRINITY_Contig3301	2	p4	(CGCC)3	12	1624	1635	TCAGCTGGCTCTTCTTGCTC	60.037	CTCGACCTGGATCGTAAGCC	59.969	217
TRINITY_Contig3301	3	p2	(AC)6	12	1754	1765	TCAGCTGGCTCTTCTTGCTC	60.037	CTCGACCTGGATCGTAAGCC	59.969	217
TRINITY_Contig3302	1	p3	(GCG)4	12	1730	1741	CGAGGAGGTGAGAAGGAGGA	60.034	TTCGTCCATCCAATCCCAGC	60.107	128
TRINITY_Contig3303	1	p3	(CGA)4	12	625	636	ACGTGTGGGAGCTCATGAAG	60.037	TGCCCGGGAACATCTGAATC	60.107	252
TRINITY_Contig3303	2	p3	(GCC)4	12	1129	1140	ACGTGTGGGAGCTCATGAAG	60.037	TGCCCGGGAACATCTGAATC	60.107	252
TRINITY_Contig3304	1	p5	(CACTC)4	20	50	69	ATCAGTCTCCGTCTCGTGGA	60.035	CTTAACACTGGACCCACGCT	59.965	205
TRINITY_Contig3304	2	p3	(TCC)4	12	228	239	ATCAGTCTCCGTCTCGTGGA	60.035	CTTAACACTGGACCCACGCT	59.965	205
TRINITY_Contig3307	1	p4	(AGCA)3	12	2154	2165	GGACACTACCGCACAGTTCA	59.967	ACACCAAAAAGCCTCCCACA	60.033	275
TRINITY_Contig3309	1	p4	(CGCC)3	12	139	150	CTAGCTAAGCTAACCCGGCC	59.966	AGCGGTAGCAAACCCTAACC	60.036	110
TRINITY_Contig3310	1	p3	(CCG)5	15	404	418	TTCGGTTCAGAGCCTGTTCC	59.965	GGGGTCAAGCCGTGTCTAAA	59.964	112
TRINITY_Contig3314	1	p3	(CAG)4	12	1491	1502	AGGCAGAGGCAGGGTATGTA	60.031	AGCATTTGTGCTGGCTTTGG	59.966	127
TRINITY_Contig3314	2	c	(GCC)4acgcaccgctggacgcccaccggcg cgcacgggggccgccgtcgccgggacgcgcg agccccgcagccggctcatgcgggggt(CGC)4	107	2829	2935	AGGCAGAGGCAGGGTATGTA	60.031	AGCATTTGTGCTGGCTTTGG	59.966	127
TRINITY_Contig3314	3	p4	(GGCC)3	12	3135	3146	AGGCAGAGGCAGGGTATGTA	60.031	AGCATTTGTGCTGGCTTTGG	59.966	127
TRINITY_Contig3314	4	p4	(CTCG)3	12	3249	3260	AGGCAGAGGCAGGGTATGTA	60.031	AGCATTTGTGCTGGCTTTGG	59.966	127
TRINITY_Contig3315	1	p4	(GTGC)3	12	288	299	GCAGCACAACATCACACGTT	59.971	TGACGATGACGAGTGCAACA	59.969	123
TRINITY_Contig3315	2	p4	(GCGG)3	12	530	541	GCAGCACAACATCACACGTT	59.971	TGACGATGACGAGTGCAACA	59.969	123
TRINITY_Contig3316	1	p6	(GCTGAA)4	24	988	1011	CTGGACCCAAGCGTCATCTT	60.036	TTCCCCGCTATTTTTCCCCC	60.033	151
TRINITY_Contig3317	1	p4	(GGAG)3	12	2851	2862	AGTCTGTGCTCCGAGATCCT	60.034	TGTGATTGCTCAGTCGGCTT	59.965	122
TRINITY_Contig3318	1	p3	(GCC)4	12	54	65	AAAAGCCGGTATGGAGTGG	60.034	CAGCGTTTATCGGCACTTCG	59.975	190
TRINITY_Contig3319	1	p5	(AGAGG)3	15	887	901	CACCAAGGCAAAGCACCTTC	59.967	CAACAAGGCTGGCAAGTTCC	59.967	116
TRINITY_Contig3323	1	p3	(CTT)4	12	1549	1560	GCCTGGCTGTTCATGGTAGT	60.035	GAAGAAGAAGCCCATCCCCC	60.106	237
TRINITY_Contig3325	1	p3	(CTG)4	12	601	612	GTTGGAAGATCGCCAGTGGA	60.036	AGTTGGCTTCACTTCAGGGG	59.889	184
TRINITY_Contig3326	1	p3	(GCC)4	12	457	468	ACGAAAAAGCCTGAGACGGT	59.894	CTCTCCCTGCTCTTGGTGTG	60.037	140
TRINITY_Contig3330	1	p4	(AATA)3	12	2126	2137	TTGGGAGGCACTAAACGTCC	59.964	AGCGGAGGTCATGAAAGGTG	60.036	206
TRINITY_Contig3331	1	p6	(GACGAT)3	18	569	586	GGCGAATTTTGAGCAGCCAA	60.038	ATGCGATGTTTGAGAGCCCA	60.035	113
TRINITY_Contig3331	2	p3	(CGC)4	12	1026	1037	GGCGAATTTTGAGCAGCCAA	60.038	ATGCGATGTTTGAGAGCCCA	60.035	113
TRINITY_Contig3331	3	p4	(CGGC)3	12	1441	1452	GGCGAATTTTGAGCAGCCAA	60.038	ATGCGATGTTTGAGAGCCCA	60.035	113

TRINITY_Contig3335	1	c	(TCAA)5gatcgcagcaaacagcga(CCT)4	50	1397	1446	GGCGTCTCTCCTCCATGATG	59.967	GCCTCGATTTCTTTGACGCC	59.903	158
TRINITY_Contig3336	1	p3	(GCC)5	15	464	478	TGACCTTCCTGTCGTGCTTC	59.966	CAGAGGCCGAAGATCATCCC	59.965	241
TRINITY_Contig3339	1	p3	(GCG)5	15	1379	1393	TGCCCTTGCAGCATCAGTTA	59.962	TGGTATGGCCAGAACAGCTG	60.035	130
TRINITY_Contig3340	1	p3	(CGG)4	12	1862	1873	AAACTCGGCCTGCCATTGTA	59.962	GGAATGCCAGGTGAAGCTCT	60.035	198
TRINITY_Contig3340	2	c	(TGCC)3tgcgtcgctcgctgct(CTTCC)3	43	2049	2091	AAACTCGGCCTGCCATTGTA	59.962	GGAATGCCAGGTGAAGCTCT	60.035	198
TRINITY_Contig3343	1	p5	(CGACA)4	20	230	249	CATTCACCGGGATCCACACA	60.036	AGTGATGTGCTGTGTCGTGT	59.895	243
TRINITY_Contig3343	2	p2	(TC)21	42	920	961	CATTCACCGGGATCCACACA	60.036	AGTGATGTGCTGTGTCGTGT	59.895	243
TRINITY_Contig3344	1	p3	(CGC)4	12	3032	3043	CAGCTGGTAATAAACGGCGC	59.972	GCTGAACCCTCTCCAACTCC	60.036	172
TRINITY_Contig3345	1	p3	(CCA)4	12	805	816	TGCCCTCTAATCCTGGAGCT	60.031	CCGCCCTAGGATAGCAGTTG	59.966	179
TRINITY_Contig3346	1	p3	(AAC)4	12	1309	1320	AACTGCCACCACTACTCACG	59.966	GGCATACTCCAGGCGCTAAT	59.965	154
TRINITY_Contig3346	2	p5	(AATCT)3	15	3073	3087	AACTGCCACCACTACTCACG	59.966	GGCATACTCCAGGCGCTAAT	59.965	154
TRINITY_Contig3346	3	c	(GCG)5agccccag(GAC)4	35	3206	3240	AACTGCCACCACTACTCACG	59.966	GGCATACTCCAGGCGCTAAT	59.965	154
TRINITY_Contig3346	4	p3	(CCG)4	12	3736	3747	AACTGCCACCACTACTCACG	59.966	GGCATACTCCAGGCGCTAAT	59.965	154
TRINITY_Contig3347	1	p3	(CGG)7	21	1804	1824	CGTCCGACAGAAACTCCCAA	59.967	ACCTTGCCTGATGAATGGGG	60.033	174
TRINITY_Contig3348	1	p6	(GTGCCC)3	18	1878	1895	CTCAAGCCATGCCTGACTCA	60.036	CACGTTCGAAGGGGTGGTTA	59.966	117
TRINITY_Contig3351	1	p3	(TCT)5	15	85	99	GCCCGCAAATGATCATCACC	59.969	GGTGCAAACGCTTCTCTTGG	60.04	230
TRINITY_Contig3352	1	p3	(GCT)6	18	44	61	AGCCAGGACTCGTTGGATTG	60.036	TCTCAGGCAGTAGCAGGCTA	60.033	135
TRINITY_Contig3352	2	c	(ACGT)3gtcccatc(CAT)4	32	997	1028	AGCCAGGACTCGTTGGATTG	60.036	TCTCAGGCAGTAGCAGGCTA	60.033	135
TRINITY_Contig3352	3	p4	(GTGC)3	12	1209	1220	AGCCAGGACTCGTTGGATTG	60.036	TCTCAGGCAGTAGCAGGCTA	60.033	135
TRINITY_Contig3353	1	c	(TCC)4ccataaccctcacca(TCC)5	42	102	143	ATGCACGACGGACTGAAAGT	59.966	GATACGCAGCTGCCATTGTG	59.971	146
TRINITY_Contig3357	1	p3	(CCG)5	15	49	63	ATCCTCTTCTTCCCCCTCCC	60.03	GGAAGGAGAAGGTCAACGGG	60.036	263
TRINITY_Contig3357	2	p5	(CCGAG)3	15	376	390	ATCCTCTTCTTCCCCCTCCC	60.03	GGAAGGAGAAGGTCAACGGG	60.036	263
TRINITY_Contig3364	1	p3	(AGG)4	12	428	439	CGTCACGCCGTGAACTTTTT	59.973	CCTCAATACCGACGGACTCG	59.971	241
TRINITY_Contig3365	1	p3	(TTA)4	12	147	158	CTTAACATCAGGCGGCATGC	59.97	GCGTCTCTGCATCTCGATCA	59.97	174
TRINITY_Contig3365	2	c	(CGC)5tcctcctgccggcgatcctgctgcccctgctcctggcggcgctcgcgtctgccgcggccatggcgggcctgaaccggagccgtctcggtgc(GCT)5	121	489	609	CTTAACATCAGGCGGCATGC	59.97	GCGTCTCTGCATCTCGATCA	59.97	174
TRINITY_Contig3365	3	p3	(CGG)4	12	2303	2314	CTTAACATCAGGCGGCATGC	59.97	GCGTCTCTGCATCTCGATCA	59.97	174
TRINITY_Contig3365	4	p4	(GGAG)3	12	2471	2482	CTTAACATCAGGCGGCATGC	59.97	GCGTCTCTGCATCTCGATCA	59.97	174
TRINITY_Contig3366	1	p4	(ATCG)4	16	421	436	CTCCGGCAGATCTACGGAAC	59.969	CTCGGACGGGATAAGGAAGC	59.968	232
TRINITY_Contig3366	2	c	(GCCG)3gcagcatcgatccgtcgctgtacgtcgatcgctctcgccaccccggccgcgcgtcgccgtcggcggcgaacaggatagaaag(GCGGCA)3	112	813	924	CTCCGGCAGATCTACGGAAC	59.969	CTCGGACGGGATAAGGAAGC	59.968	232
TRINITY_Contig3366	3	p4	(GCCG)3	12	1294	1305	CTCCGGCAGATCTACGGAAC	59.969	CTCGGACGGGATAAGGAAGC	59.968	232
TRINITY_Contig3366	4	p4	(GGCC)3	12	2340	2351	CTCCGGCAGATCTACGGAAC	59.969	CTCGGACGGGATAAGGAAGC	59.968	232
TRINITY_Contig3366	5	p4	(CTTG)3	12	2712	2723	CTCCGGCAGATCTACGGAAC	59.969	CTCGGACGGGATAAGGAAGC	59.968	232
TRINITY_Contig3369	1	p2	(GC)6	12	496	507	AAGAAGCACTTCCCGCTGAA	59.891	GCTGCTGTTCCAGTACGAGT	60.038	126
TRINITY_Contig3370	1	p3	(GCG)4	12	483	494	ATTGCTAGCGATCAGGACCG	59.967	CTGGCTGTTGCGATCGAATG	59.973	213
TRINITY_Contig3370	2	p3	(GCG)4	12	1045	1056	ATTGCTAGCGATCAGGACCG	59.967	CTGGCTGTTGCGATCGAATG	59.973	213
TRINITY_Contig3371	1	p2	(CT)8	16	20	35	TCGTCCCTCTACTGGAAGGG	60.033	CCCACCAAGGCCACCATATT	60.032	273
TRINITY_Contig3376	1	c	(GCC)4gtctcctgcttagcttctaccgga(GCC)6	54	1941	1994	ACCAGAACATTTTGTGCGGC	59.968	CGGCTCCGGTAGAAGCTAAG	59.969	260
TRINITY_Contig3376	2	c	(ACC)4gccgcacggaggaattcgtcccgtgcgccgccgggatcccagcgaaatatctcatgggtccaacccgaaacggataaacacgttgc(GCGG)3	110	2220	2329	ACCAGAACATTTTGTGCGGC	59.968	CGGCTCCGGTAGAAGCTAAG	59.969	260
TRINITY_Contig3380	1	p3	(CGG)4	12	1183	1194	TCTTAGTGACAAGCTGCGCA	59.967	TCCAACCGCAAGTGCCTAAT	59.962	169
TRINITY_Contig3381	1	c	(CCG)4cctcttccttcgccctcaacttcttcc(CCG)4	51	208	258	ATTCCACTGCTCAACTCGGG	60.036	GGGAAGAAGTTGAGGGCGAA	59.964	131
TRINITY_Contig3381	2	p3	(CGC)6	18	843	860	ATTCCACTGCTCAACTCGGG	60.036	GGGAAGAAGTTGAGGGCGAA	59.964	131
TRINITY_Contig3381	3	p5	(TCAGG)3	15	1147	1161	ATTCCACTGCTCAACTCGGG	60.036	GGGAAGAAGTTGAGGGCGAA	59.964	131
TRINITY_Contig3388	1	p3	(GCG)4	12	534	545	GGGAGTGAGCGAGGTTTTGA	59.965	GGAGCAGGACAGCCTCTTTT	59.963	151
TRINITY_Contig3388	2	p6	(GGAGCA)3	18	735	752	GGGAGTGAGCGAGGTTTTGA	59.965	GGAGCAGGACAGCCTCTTTT	59.963	151
TRINITY_Contig3390	1	p3	(GTG)4	12	1132	1143	GCTTAGCATGCTTCTTCGCC	59.971	GGTCAAGAAGCCTGTCGTGA	59.966	227
TRINITY_Contig3390	2	p3	(GCC)4	12	1307	1318	GCTTAGCATGCTTCTTCGCC	59.971	GGTCAAGAAGCCTGTCGTGA	59.966	227
TRINITY_Contig3391	1	p3	(GCC)4	12	484	495	CGACATCCTTCCTTGCACCT	60.036	AGTTCAGAGGATTTGGGGCG	60.035	223
TRINITY_Contig3391	2	p4	(ATCC)3	12	819	830	CGACATCCTTCCTTGCACCT	60.036	AGTTCAGAGGATTTGGGGCG	60.035	223
TRINITY_Contig3392	1	p4	(ATGC)3	12	64	75	TGGCCCTCCTATTCCTTGGA	59.954	CCATAGCCACCGAGCATAGG	60.037	170
TRINITY_Contig3392	2	p4	(TAGC)5	20	320	339	TGGCCCTCCTATTCCTTGGA	59.954	CCATAGCCACCGAGCATAGG	60.037	170
TRINITY_Contig3392	3	p3	(CGC)4	12	780	791	TGGCCCTCCTATTCCTTGGA	59.954	CCATAGCCACCGAGCATAGG	60.037	170
TRINITY_Contig3393	1	p4	(CTCG)3	12	8	19	AGGGACTCCTGAGGCATCAT	60.03	ATTGTCAGTGAGCCAGGGTG	59.963	215
TRINITY_Contig3393	2	c*	(CTC)3(<CTC>TCTCTCTCTCTCTCTCTCTCTCGGCCTTCTTCGTTCCAAGCGGCGACGACAGGGGAGTGGCGCCGCCTTGCCAGCTTTTCGTCGGGCGCTCCGCCGGCCGACAAGCATATTCTTTTCCCAGTATGAGTAAATGCAGACCATAAAGCGCTTGCCGGAGAGGGTTCATAGCTCCTTGCGTTCAAGCATTGTTCTGTCTGACCTGCCAAGGGTTGTTGAGGAGTTGATATATAATAGCATTGATGCGAATGCGACCAAGATTGACATTGCAATAAACATCAGAGCATGCTATGTAAAAGTGGAAGATGATGGTTGTGGTATTACACGAGATGAATTGGTTCTCTTGGGAGAAAAATATACAACATCCAAATTTCATAATGTGATGGCGGATGGGGAACTTAGTCCTAGAAGTTTTGGACTAAATGGAGAAGCAC	33	127	159	AGGGACTCCTGAGGCATCAT	60.03	ATTGTCAGTGAGCCAGGGTG	59.963	215
TRINITY_Contig3396	1	c	(AGCACC)3agccccagcacc(AGCACA)4	54	2716	2769	CAGTGTTTGTGAGGCTTGCC	59.969	TTGATGCGGATGTGCTCTGT	60.036	233
TRINITY_Contig3398	1	p4	(GGCC)3	12	36	47	CGATCCGATCCGATCCGATC	59.972	AATCATGGCGCTGTTCTCCA	60.035	245
TRINITY_Contig3398	2	p5	(TTGGC)3	15	195	209	CGATCCGATCCGATCCGATC	59.972	AATCATGGCGCTGTTCTCCA	60.035	245
TRINITY_Contig3398	3	p4	(GCAC)4	16	389	404	CGATCCGATCCGATCCGATC	59.972	AATCATGGCGCTGTTCTCCA	60.035	245
TRINITY_Contig3398	4	p4	(ATAG)3	12	753	764	CGATCCGATCCGATCCGATC	59.972	AATCATGGCGCTGTTCTCCA	60.035	245
TRINITY_Contig3398	5	p3	(GCT)4	12	953	964	CGATCCGATCCGATCCGATC	59.972	AATCATGGCGCTGTTCTCCA	60.035	245
TRINITY_Contig3398	6	p5	(ATCCG)4	20	1074	1093	CGATCCGATCCGATCCGATC	59.972	AATCATGGCGCTGTTCTCCA	60.035	245
TRINITY_Contig3400	1	c	(CGAGGA)3gatgggggtca(AGG)4acggcgac(GAG)6	67	841	907	TGCCCTTCCGAGCAAATCAT	60.034	GTTGCTTGCTTCCATTGGCA	59.966	124
TRINITY_Contig3402	1	p3	(CGG)5	15	1765	1779	AGGCGTGTCAATCACTGGAG	60.037	TCTGGCTGTTGTCGTCTGTC	59.968	224
TRINITY_Contig3402	2	p4	(TCGG)3	12	2007	2018	AGGCGTGTCAATCACTGGAG	60.037	TCTGGCTGTTGTCGTCTGTC	59.968	224
TRINITY_Contig3404	1	p3	(CGC)4	12	292	303	CGGGAAAGAAAGGACGGACA	59.966	GCTCCTTCAGCTCCTCGATC	59.968	105
TRINITY_Contig3405	1	p3	(GCA)4	12	606	617	AGAGTGGCAACGACTTCCTG	59.966	CAGGAATTCCCGATCCCTCG	59.966	167
TRINITY_Contig3407	1	p6	(AGAGGG)3	18	2636	2653	GCAAGTGCGGAAAATGTGGT	59.968	ATCAGGTCCCACCATCGAGA	60.032	182
TRINITY_Contig3409	1	p3	(TAT)4	12	948	959	TGGTCTCCACGTTCTTGTCG	59.968	CACAGGCTCTGCTTCAGACA	59.966	233
TRINITY_Contig3409	2	p4	(GTTG)3	12	3185	3196	TGGTCTCCACGTTCTTGTCG	59.968	CACAGGCTCTGCTTCAGACA	59.966	233
TRINITY_Contig3409	3	p2	(TG)9	18	3845	3862	TGGTCTCCACGTTCTTGTCG	59.968	CACAGGCTCTGCTTCAGACA	59.966	233
TRINITY_Contig3411	1	c	(GCG)4atgacggccaccg(GCT)4	37	42	78	CTGGCCATGTTATGCGTTCG	59.972	CTCTGACGCATAGCCAGGAG	59.968	278
TRINITY_Contig3412	1	p3	(CGC)4	12	2452	2463	TGCCGAGCAACATTCTGACT	59.965	ATTGCTGCCCAAGAGCATCT	60.033	143
TRINITY_Contig3412	2	p3	(GAG)6	18	3722	3739	TGCCGAGCAACATTCTGACT	59.965	ATTGCTGCCCAAGAGCATCT	60.033	143
TRINITY_Contig3414	1	p3	(CCT)4	12	305	316	GACGAGCTCCAATCTCCAGG	59.896	TAGCCTCATCAGTGGTCGGA	60.034	239
TRINITY_Contig3415	1	p4	(GAGG)3	12	200	211	AGCATCACGATGGCAAGACA	60.036	AGATGCTTGGACTCCTCCCT	59.956	193
TRINITY_Contig3416	1	p3	(GCC)5	15	92	106	TCAGGGCAGATGATTTGCGT	60.035	CTCGCATGCTGATTTCTGGC	59.971	223
TRINITY_Contig3417	1	p5	(CATCG)3	15	123	137	ACACTGGCCCTGTTGGAAAA	60.033	CCTCAAACTGCCTTGCCAAC	59.967	135
TRINITY_Contig3417	2	p6	(CGGAGA)3	18	9681	9698	ACACTGGCCCTGTTGGAAAA	60.033	CCTCAAACTGCCTTGCCAAC	59.967	135
TRINITY_Contig3418	1	p4	(GGAT)3	12	1403	1414	TGGATCTGTCGGGGATGACT	60.032	TCGAAAGTGTGCGAGCTTCT	59.968	204
TRINITY_Contig3419	1	p3	(GCA)5	15	574	588	CCCGCATGTACTACCTCGAC	59.97	CGAAGGAGGAATTGCGCATG	59.971	192
TRINITY_Contig3423	1	p4	(CAAT)3	12	35	46	TGAGGTAGTACCGGCATCCA	60.033	TGCCAACAATGTTGCACGAG	59.969	114
TRINITY_Contig3427	1	p5	(AATAA)3	15	236	250	GAGCCATCTTTGCCTCCACT	60.035	GATCCCAAAGAGGAACGCCA	60.035	176
TRINITY_Contig3427	2	p3	(GGC)5	15	2940	2954	GAGCCATCTTTGCCTCCACT	60.035	GATCCCAAAGAGGAACGCCA	60.035	176
TRINITY_Contig3427	3	p3	(GGC)5	15	3064	3078	GAGCCATCTTTGCCTCCACT	60.035	GATCCCAAAGAGGAACGCCA	60.035	176

TRINITY_Contig3428	1	p3	(AGG)4	12	2144	2155	GACAACTGTTGCACTGCAGG	59.97	GCCTGTGCTGCTGAAGAGTA	60.037	236
TRINITY_Contig3431	1	p6	(CCACGC)3	18	322	339	ACATGGGTCCAAACGTCCTC	59.964	GCTACTACCGGCTCGAGTTC	59.971	178
TRINITY_Contig3431	2	p3	(GCC)4	12	1539	1550	ACATGGGTCCAAACGTCCTC	59.964	GCTACTACCGGCTCGAGTTC	59.971	178
TRINITY_Contig3433	1	c	(GAGG)4catgagcc(GAG)4	36	608	643	CTTGGAGTGAAGAGGCTGCA	59.964	CCTTATCGGAAGCAGGGACC	59.893	114
TRINITY_Contig3434	1	p3	(CCG)6	18	940	957	AGGTAGAGGGAGTGAGCTGG	60.033	CTCCACCTCCACTTGAGCTG	60.037	108
TRINITY_Contig3435	1	p4	(GTAG)3	12	456	467	AGAGGGGCTCTGTTCCAGAT	59.956	AAACCCTCCTAGCGATCCCT	60.031	111
TRINITY_Contig3438	1	p6	(CCTCCG)3	18	101	118	CCAACAGCCCGACTTTTGTG	59.969	CTTGGCCATCGTTCACTCCT	60.036	101
TRINITY_Contig3438	2	p3	(AAG)4	12	804	815	CCAACAGCCCGACTTTTGTG	59.969	CTTGGCCATCGTTCACTCCT	60.036	101
TRINITY_Contig3441	1	p3	(GAA)6	18	15	32	GGTAGTAGTACCGCGACTGC	59.971	AGCAACACACGCACAGTAGA	59.896	264
TRINITY_Contig3441	2	c	(CGA)4ccgcgacccgtcgtcggaggtcgac cacgccgccggc(GGT)4	61	605	665	GGTAGTAGTACCGCGACTGC	59.971	AGCAACACACGCACAGTAGA	59.896	264
TRINITY_Contig3441	3	p3	(GCC)4	12	770	781	GGTAGTAGTACCGCGACTGC	59.971	AGCAACACACGCACAGTAGA	59.896	264
TRINITY_Contig3442	1	p3	(CGC)5	15	545	559	ATGGCACCACAGATCCACAG	60.035	TTGTCCGATCTCTGCTTCCG	59.827	124
TRINITY_Contig3442	2	p4	(GGGA)3	12	692	703	ATGGCACCACAGATCCACAG	60.035	TTGTCCGATCTCTGCTTCCG	59.827	124
TRINITY_Contig3444	1	p2	(CG)8	16	168	183	TCTCCTCTCCCATCGCTGAA	60.033	CCGTTATAGAGCCGAAGCGT	59.97	113
TRINITY_Contig3445	1	p3	(CGG)4	12	89	100	GCGCATGTTCGAGAAGGTTG	60.179	TCCACCCTTTGGTCATCACG	59.964	224
TRINITY_Contig3445	2	c	(CAG)4gaggc(CTT)4cccacaggcggcg gccttgcaggtgcagatgcagcagcgcttggt ggtggcgccgccgcgcgtcgtgatctccatga(AAG)6	124	555	678	GCGCATGTTCGAGAAGGTTG	60.179	TCCACCCTTTGGTCATCACG	59.964	224
TRINITY_Contig3447	1	p5	(GGGGA)4	20	1540	1559	GTGGAGCAGCAACAGATCCT	60.036	ACCCTCCTCCTCCAAGATCC	60.031	104
TRINITY_Contig3448	1	p3	(GCC)5	15	978	992	CTTGGGCTAGGGTTTGAGGG	60.034	CCTTGAGGGTGATGTGGTCC	60.035	226
TRINITY_Contig3449	1	c	(GCG)4acggcggcggcgt(GGCAC)3	40	2755	2794	CCGTGAAGCTCCCTTCCATT	60.035	TTCCTCCTCGATCACTCCGT	60.034	120
TRINITY_Contig3451	1	c	(GCT)5t(CGG)6	34	322	355	CTTTCCACTTTGTGCAGCCC	59.967	ACATGGAAGCAGGCTCAGTC	60.036	130
TRINITY_Contig3452	1	c	(ACGC)3ccaccgcgccgccacaggcgcccc aagccacaccagcgacgtcgcgcg(GCCA)4 tgtccacggagcccctcctccccggcctcgccg gcgacaaggcggcggcgg(AGC)8	151	170	320	GAAGTGGAAGGACAGTGCGA	59.966	GTTGTGGGCAGAAGCAACAG	59.969	272
TRINITY_Contig3452	2	p3	(TCG)4	12	4246	4257	GAAGTGGAAGGACAGTGCGA	59.966	GTTGTGGGCAGAAGCAACAG	59.969	272
TRINITY_Contig3453	1	p4	(GCGT)4	16	170	185	CCACGTCAGGAGCTGCTTTA	60.037	GGCGACCAGTACTCCTCCTA	60.107	117
TRINITY_Contig3456	1	p3	(GCG)4	12	5686	5697	ATTCTGGCCAATCCCACCTG	60.033	GGTGAAGCTGGCCTTTTTGG	59.966	205
TRINITY_Contig3458	1	p3	(GCG)5	15	348	362	CAGAGGTGCCTCGATCGATC	60.04	GGACGAGGAGCATGATCAGG	59.967	183
TRINITY_Contig3460	1	p3	(GCG)4	12	409	420	GATCTGCTTGGCCTTGACCT	60.035	CGATGTGACCTCGCGTATCA	59.971	139
TRINITY_Contig3461	1	p4	(AAAC)3	12	64	75	GTGGAAATAAGGGGTGGGGG	60.033	CCCTCCCTCGTTTCCAACTC	60.036	137
TRINITY_Contig3461	2	p4	(GAGG)3	12	1523	1534	GTGGAAATAAGGGGTGGGGG	60.033	CCCTCCCTCGTTTCCAACTC	60.036	137
TRINITY_Contig3461	3	p4	(GAGG)3	12	1714	1725	GTGGAAATAAGGGGTGGGGG	60.033	CCCTCCCTCGTTTCCAACTC	60.036	137
TRINITY_Contig3462	1	c	(CGGCCA)3tgtccag(CAC)4cat(CAC) 5catcaccaccacccgtggggatcctccctca gcgccgtcgtcg(CGC)4	110	277	386	GGTGAAGTTCAGGGAGGAGC	60.036	CAGGGAGCTCTGTCTCTCCT	60.033	176
TRINITY_Contig3462	2	p3	(TCG)4	12	527	538	GGTGAAGTTCAGGGAGGAGC	60.036	CAGGGAGCTCTGTCTCTCCT	60.033	176
TRINITY_Contig3462	3	p3	(AAG)4	12	1043	1054	GGTGAAGTTCAGGGAGGAGC	60.036	CAGGGAGCTCTGTCTCTCCT	60.033	176
TRINITY_Contig3463	1	p2	(CT)6	12	1079	1090	CGAAGCCCCTTCACATCCTT	60.035	TGGTCAAGAAGAAGGTCGGC	59.965	142
TRINITY_Contig3464	1	p3	(CGC)5	15	2396	2410	AGGGTCCTCCAGCATCATCT	60.03	GAGGCTGTTGAAGGATGGCT	60.035	133
TRINITY_Contig3465	1	p2	(AT)6	12	849	860	AGCTTCTTCCCATCTGGCAC	60.035	TGAGATGCAGCGGAAAACCT	59.963	275
TRINITY_Contig3469	1	p3	(CCT)4	12	378	389	ATCTGCACTGCGACCAAAGA	59.965	GAGGGCGTTGTTGTTTTGCT	59.898	245
TRINITY_Contig3469	2	p3	(CCG)5	15	1016	1030	ATCTGCACTGCGACCAAAGA	59.965	GAGGGCGTTGTTGTTTTGCT	59.898	245
TRINITY_Contig3470	1	p4	(GCGG)3	12	149	160	GGCCTCTCTTGTTCCTGCTT	59.963	AGGCTACAGCTACAAGGGGA	59.957	215
TRINITY_Contig3470	2	p3	(ACC)4	12	434	445	GGCCTCTCTTGTTCCTGCTT	59.963	AGGCTACAGCTACAAGGGGA	59.957	215
TRINITY_Contig3470	3	p3	(ACG)5	15	580	594	GGCCTCTCTTGTTCCTGCTT	59.963	AGGCTACAGCTACAAGGGGA	59.957	215
TRINITY_Contig3474	1	p3	(CGC)4	12	314	325	GCAGCTACTACAGGCCTACG	59.969	GACATGGAGGACGTCGAGAG	59.621	144
TRINITY_Contig3474	2	c	(GGC)4gatagttctcgtggccatggacatgg agcagcgaacaagcagctagctagcgaagac tggtcagc(GAAG)3	89	669	757	GCAGCTACTACAGGCCTACG	59.969	GACATGGAGGACGTCGAGAG	59.621	144
TRINITY_Contig3476	1	p3	(TCT)4	12	1421	1432	CTCGTCGTTCCTGGAAACCA	59.967	CGCTGATTGCAGATGTCGTG	59.974	146
TRINITY_Contig3478	1	p4	(CCTT)3	12	1283	1294	AGGAGCTAGGAGGGTCTTGG	60.032	CATCCCAATCCAATGCTGCG	59.968	272
TRINITY_Contig3478	2	p3	(AGG)5	15	2327	2341	AGGAGCTAGGAGGGTCTTGG	60.032	CATCCCAATCCAATGCTGCG	59.968	272
TRINITY_Contig3481	1	c	(CGG)5cgcgtcgtgcgagggatcggcggcg gcagcgcgtcgtgcgagggattggcggcgccg cgtcgtgcgctggat(CGG)4	99	1598	1696	ATCCAAAGCACCCTGATGCA	59.96	TCCCATTTGCAGGCATCCAT	60.032	112
TRINITY_Contig3482	1	p3	(CGC)4	12	1109	1120	GACGTAGATGCACGTTTCGC	59.977	TCACGGTGGGAAAGGAAAGG	59.89	268
TRINITY_Contig3482	2	p3	(TCG)4	12	1263	1274	GACGTAGATGCACGTTTCGC	59.977	TCACGGTGGGAAAGGAAAGG	59.89	268
TRINITY_Contig3484	1	p3	(CGC)7	21	985	1005	CGGGCGTCCCTTCTAGAATC	59.968	GGCGAGATGAGTACAGGAGC	59.969	116
TRINITY_Contig3487	1	p3	(GTC)4	12	858	869	TTGTTGGCCTAGCAAAACGC	59.969	CCCTGGTGGAAGAGTTGGTC	59.963	204
TRINITY_Contig3488	1	p3	(CGA)5	15	3530	3544	ATAGGTTCCCTGGGCATCCT	60.029	TGGTGACCAGAATTGTCCGG	59.964	190
TRINITY_Contig3489	1	c	(GGA)4gaggagcgaggccaga(GCG)6	46	1152	1197	TGGCGCTTACAGTTCGAGTT	59.967	ATCTTTACCCGTGGCTGTGG	60.035	154
TRINITY_Contig3489	2	c	(GGT)5gatggtgcggcgggggcggcgggc ccgggtaggaggagtagtggtgcggcggcgg g(GGC)4gggtagtagtcgtcc(CGG)4ctc gacgaggcgcccatggcgcagcgcg(GGCG A)3	153	1318	1470	TGGCGCTTACAGTTCGAGTT	59.967	ATCTTTACCCGTGGCTGTGG	60.035	154
TRINITY_Contig3489	3	p4	(GGGC)3	12	1584	1595	TGGCGCTTACAGTTCGAGTT	59.967	ATCTTTACCCGTGGCTGTGG	60.035	154
TRINITY_Contig3491	1	c	(CGG)6cagtggaggcgcggccggtggcggt gctccctctgatgccgggcgcggacgtgcccgt cgcgcacga(GGAGGC)3	103	1988	2090	CTCTGTCTCATCGATGCCCC	59.967	TCTCCACAATGGTGTCACCG	59.965	177
TRINITY_Contig3491	2	p4	(GATT)3	12	2456	2467	CTCTGTCTCATCGATGCCCC	59.967	TCTCCACAATGGTGTCACCG	59.965	177
TRINITY_Contig3494	1	p3	(CCG)4	12	250	261	GCTGCGGCAGATCAGTATCT	59.967	TGAACTCGCCTGTCCAGAAC	59.966	259
TRINITY_Contig3496	1	p3	(CTG)4	12	616	627	CCGTGCTCACTATCCTCGTC	59.97	CTTCCTTGGTGCAGGGAGAG	60.036	240
TRINITY_Contig3496	2	p4	(GAGC)3	12	888	899	CCGTGCTCACTATCCTCGTC	59.97	CTTCCTTGGTGCAGGGAGAG	60.036	240
TRINITY_Contig3498	1	p3	(GCA)4	12	331	342	CGGTTCTACATCGTCGGGAG	59.971	GTTCCCCTTCTCCTTGAGCC	60.035	165
TRINITY_Contig3498	2	p2	(TA)6	12	1836	1847	CGGTTCTACATCGTCGGGAG	59.971	GTTCCCCTTCTCCTTGAGCC	60.035	165
TRINITY_Contig3498	3	p4	(TTTC)3	12	2242	2253	CGGTTCTACATCGTCGGGAG	59.971	GTTCCCCTTCTCCTTGAGCC	60.035	165
TRINITY_Contig3509	1	p3	(CGG)4	12	187	198	CTTCCTCCCCGGGTTTCTTC	60.035	CAGTTGACTTGACCGACCGA	59.968	108
TRINITY_Contig3509	2	p3	(CCT)4	12	417	428	CTTCCTCCCCGGGTTTCTTC	60.035	CAGTTGACTTGACCGACCGA	59.968	108
TRINITY_Contig3509	3	p4	(CGGT)3	12	843	854	CTTCCTCCCCGGGTTTCTTC	60.035	CAGTTGACTTGACCGACCGA	59.968	108
TRINITY_Contig3514	1	c	(GCG)4gcaggccgc(GGA)4	33	484	516	AAAGGAGCGAACAAGAGGGG	59.963	TGCAGCAGGTAGGAGGAGAT	60.032	143
TRINITY_Contig3517	1	p3	(CTT)4	12	191	202	GAGGAGGCGCATATCAGGAC	60.038	TTGGATCTACACGTTGCCCC	60.036	254
TRINITY_Contig3517	2	p2	(TG)6	12	782	793	GAGGAGGCGCATATCAGGAC	60.038	TTGGATCTACACGTTGCCCC	60.036	254
TRINITY_Contig3519	1	p3	(GCG)5	15	825	839	CCATATCCGGCTCTGCATGT	59.964	ACGTTGCACATGTTTTGGGG	59.896	211
TRINITY_Contig3522	1	p3	(TCA)4	12	1555	1566	AGCCCATCAAACATCAGGGG	60.033	TGCACGTTCATTGCCAAAGG	59.968	115
TRINITY_Contig3527	1	p4	(TGCG)5	20	593	612	TGGGGGAGTCCAAGATCGAT	60.031	GTCCATGGCTGGCTCTAGAC	59.894	112
TRINITY_Contig3529	1	p4	(AAGA)3	12	278	289	ATGATGAGGCCATGCACCAA	60.033	GCTCAGCATAGGCCATGTCA	60.179	217
TRINITY_Contig3529	2	p3	(CGA)4	12	1027	1038	ATGATGAGGCCATGCACCAA	60.033	GCTCAGCATAGGCCATGTCA	60.179	217
TRINITY_Contig3529	3	p3	(CGG)4	12	1140	1151	ATGATGAGGCCATGCACCAA	60.033	GCTCAGCATAGGCCATGTCA	60.179	217
TRINITY_Contig3530	1	p3	(CGC)4	12	458	469	GCCACTGTCACAGCAAACAG	59.97	CTGGAGCTGGAGCACATTGA	60.036	174
TRINITY_Contig3531	1	p3	(CCT)4	12	325	336	TCCGTTTGTCTCCTTGCCTC	59.965	TCGAGATCGGCGCTAAAGTC	59.971	210
TRINITY_Contig3531	2	c	(CCG)5ccaa(CTC)4	31	525	555	TCCGTTTGTCTCCTTGCCTC	59.965	TCGAGATCGGCGCTAAAGTC	59.971	210
TRINITY_Contig3532	1	c	(GCGCCA)3ccgcgcgccgccgccatccgg cggcgcaccacggcgtacgccacaccgacag ccaccag(CGC)4	89	243	331	AGGAGAAGGAGCTCGCAAAC	60.037	CATCGATCGACAGCGTCTCA	59.972	229
TRINITY_Contig3534	1	p3	(TCT)4	12	2984	2995	GTATGCTCTCCCTGCCCATC	59.964	CATACGAAACACTCAGCGCG	59.976	151
TRINITY_Contig3536	1	c	(CCG)5ccatccgggcgagccccagcgcgcg ccgcctccgcgcgctggcccgccccgtccggg cgtctcagcagcc(GCA)5	100	235	334	TCACTGGAGACGTGAGCAAC	59.968	TACCACGCTGCTGGCTTTTA	59.964	176
TRINITY_Contig3545	1	p3	(GAA)5	15	519	533	TTCAACCAGATCGGCAAGCT	59.963	TGACACCCTCGTTGTCGAAG	59.968	178
TRINITY_Contig3548	1	p3	(CGC)4	12	45	56	TCGTTATCGTCGACGTCGTC	59.976	TCAGCTTCTTGCCCCATGAG	60.035	207
TRINITY_Contig3550	1	p6	(GGCCGC)3	18	550	567	AGGTATCTGCGCATGGATGG	59.964	GAGCACATCCAATCCACCCA	60.034	120
TRINITY_Contig3553	1	p2	(AC)7	14	1096	1109	TCCATCCAGCAAGCACAGAG	60.036	GGAAGCAGCCATCCTCAAGT	60.035	255
TRINITY_Contig3564	1	p3	(GCA)4	12	510	521	TGGACGGCGTCATTGTCTAC	60.109	GACACACACTCCTCTGAGCC	60.038	145
TRINITY_Contig3565	1	p3	(GCC)4	12	366	377	ACGGCCCAATAAACATCGGT	60.035	GAACCCGACAAGCTCCAAGA	59.965	255
TRINITY_Contig3570	1	p4	(TCTT)3	12	1137	1148	CCTGTGCTAGTCTGCTGGTC	60.108	AAGCGTAGACACAGCAGGAC	60.038	110
TRINITY_Contig3570	2	p4	(ATGG)3	12	1287	1298	CCTGTGCTAGTCTGCTGGTC	60.108	AAGCGTAGACACAGCAGGAC	60.038	110
TRINITY_Contig3573	1	p3	(GAG)4	12	310	321	TTGTACTCCGACGCCATTCC	60.109	GGATGCATGGACATGTTGCC	59.896	103
TRINITY_Contig3579	1	p4	(GCGA)4	16	976	991	CGTGTTGAGCTGCTTGATGG	59.833	CCAGTATCCAGCCAAAGCCA	60.034	252
TRINITY_Contig3582	1	p3	(AAT)4	12	803	814	CCGGGATAAGAAGGGAAGCC	59.891	ATGGTTGCCTCCCTTTCGAG	60.035	111
TRINITY_Contig3583	1	p3	(CGC)6	18	92	109	ACAGTGGTGCTACCGTGAAG	59.966	CCTGCCACTGGAACATGGAT	60.034	141

TRINITY_Contig3586	1	p3	(CGC)4	12	1035	1046	AAGAGCTCGAGGTTGCTGTC	60.038	TGGAAGTCGAGGAGGACGAT	60.034	265
TRINITY_Contig3587	1	p3	(CAG)4	12	371	382	CACGAGGCTACGCTCTACAG	59.972	ATCGAATCCCCGTCGTGATC	59.686	253
TRINITY_Contig3587	2	c	(CCG)4ctccggcctccggcggcgccggaagaggtccctcacgattcgcgagcggaacgccgcg(GACC)3	82	550	631	CACGAGGCTACGCTCTACAG	59.972	ATCGAATCCCCGTCGTGATC	59.686	253
TRINITY_Contig3589	1	c	(CTC)4gccatcgccg(GCC)4	34	308	341	CTTGCTTTCCTGCCACCAAC	59.967	ATGCCTCTCCCCATCTTCCT	60.029	158
TRINITY_Contig3589	2	p3	(GCC)4	12	638	649	CTTGCTTTCCTGCCACCAAC	59.967	ATGCCTCTCCCCATCTTCCT	60.029	158
TRINITY_Contig3590	1	c	(CGG)5cgccggcggaa(GAC)4	38	754	791	ACCTGGCAGTTCTGATGCTC	60.036	TCTTCGCGTCTCCTTTTGCT	59.966	123
TRINITY_Contig3590	2	p3	(TGA)5	15	2483	2497	ACCTGGCAGTTCTGATGCTC	60.036	TCTTCGCGTCTCCTTTTGCT	59.966	123
TRINITY_Contig3592	1	p3	(CGC)4	12	399	410	GATGAAGGAGAGGCTCGTCG	59.97	ACTTGCTCTTGGTGACGGAG	59.966	131
TRINITY_Contig3593	1	c	(AGT)4cctccattatacttccttggcttgtcactgcttttcccactcgctgtttattggccgggacaaggacaag(GGA)6	100	808	907	ACAAAAACTGGGCCAAGGGA	60.032	GCGCATGTTGTCGAATCCTC	59.973	214
TRINITY_Contig3595	1	p3	(GCG)4	12	1527	1538	ATTTCACCCTGACAGCCTCG	60.036	GGTCGACAAGTGCCAGAAGA	59.966	125
TRINITY_Contig3595	2	p3	(GCG)4	12	1689	1700	ATTTCACCCTGACAGCCTCG	60.036	GGTCGACAAGTGCCAGAAGA	59.966	125
TRINITY_Contig3599	1	p3	(GGA)4	12	2294	2305	CGAATGTTGCTGTTGCCACA	59.969	GAGTGCAGAGTGCTGTGGAT	60.037	101
TRINITY_Contig3599	2	p5	(GTGCA)4	20	2527	2546	CGAATGTTGCTGTTGCCACA	59.969	GAGTGCAGAGTGCTGTGGAT	60.037	101
TRINITY_Contig3602	1	p6	(CTCCGC)3	18	359	376	ACACCTCCTTCTCCTCCTCC	59.958	TCCCTCTCGTTCCAGCAGTA	59.961	229
TRINITY_Contig3602	2	p3	(CGG)4	12	575	586	ACACCTCCTTCTCCTCCTCC	59.958	TCCCTCTCGTTCCAGCAGTA	59.961	229
TRINITY_Contig3602	3	p3	(GCG)4	12	798	809	ACACCTCCTTCTCCTCCTCC	59.958	TCCCTCTCGTTCCAGCAGTA	59.961	229
TRINITY_Contig3604	1	p3	(CTC)4	12	812	823	CTCCTTGATCTCGTCGTCCG	59.972	CATCTCATTCCTCGAGGCCC	59.965	112
TRINITY_Contig3612	1	p6	(TGCGAG)3	18	1772	1789	CAGTCGACGACAAGCTTCCT	60.039	TTCTACAGCTCGGGGATCCA	60.033	161
TRINITY_Contig3617	1	p3	(GGC)4	12	536	547	CAGTCAATCAGCAACGCACC	60.11	ATGCTGAGGACAATGGCCTC	60.107	117
TRINITY_Contig3622	1	p3	(GCT)5	15	1324	1338	TTGCTCACGGAGGCAATGAT	60.035	AAACCCACCCCAAGCAAGAA	60.032	128
TRINITY_Contig3623	1	c	(CGC)5cagaga(AGG)4	33	2339	2371	AGCAGCAGAGTTTAGGCCAG	60.036	CCATTCCCAATCGCATGCTG	59.968	190
TRINITY_Contig3624	1	p3	(CGC)4	12	239	250	CGCCATGGAAGGATGCATTG	59.968	CAGCATGCAAGCTCGTCATC	59.972	123
TRINITY_Contig3625	1	p4	(TCCC)4	16	66	81	ACAGATTCCCTCCCTCCCTC	60.031	GCCCCTTCTGAGCTGGATTT	60.034	194
TRINITY_Contig3625	2	p3	(ATG)4	12	983	994	ACAGATTCCCTCCCTCCCTC	60.031	GCCCCTTCTGAGCTGGATTT	60.034	194
TRINITY_Contig3627	1	p2	(GA)8	16	129	144	CTTCCACAACTACCGCGTCT	60.039	ACTTGAACTTTTGCGGACGC	59.971	233
TRINITY_Contig3627	2	p3	(CCG)4	12	1013	1024	CTTCCACAACTACCGCGTCT	60.039	ACTTGAACTTTTGCGGACGC	59.971	233
TRINITY_Contig3628	1	c	(GA)8ggaagatgagcttcaacaagagcaggggagtggggat(CGG)4	65	1	65	GTCAGCTCTACGCCATCTCC	59.969	GCTCGACGTCTAGACCGATG	60.042	123
TRINITY_Contig3635	1	p3	(AGG)4	12	175	186	TCACACACTCGCACACTCTC	59.969	TCTTATCGTACGTCACCGCG	59.973	170
TRINITY_Contig3638	1	p3	(TCG)5	15	91	105	GTCACCCTCGCTAGCCATAC	59.968	AGGCTGGTGACTACGAGGAT	60.033	188
TRINITY_Contig3639	1	p2	(GA)6	12	963	974	CCAGCGAAGAGGAAGAAGCA	60.037	ATCTCCTGCTTGCGCTTCTT	60.036	106
TRINITY_Contig3640	1	p6	(GGACGC)3	18	115	132	GGGGAGTGAGGGACTTCTCT	59.958	ATCGGCTGGGTCCGAAATTT	60.034	137
TRINITY_Contig3640	2	p3	(CGG)4	12	481	492	GGGGAGTGAGGGACTTCTCT	59.958	ATCGGCTGGGTCCGAAATTT	60.034	137
TRINITY_Contig3640	3	p2	(GA)6	12	1103	1114	GGGGAGTGAGGGACTTCTCT	59.958	ATCGGCTGGGTCCGAAATTT	60.034	137
TRINITY_Contig3640	4	p4	(TTTA)3	12	1324	1335	GGGGAGTGAGGGACTTCTCT	59.958	ATCGGCTGGGTCCGAAATTT	60.034	137
TRINITY_Contig3644	1	p3	(CCT)4	12	84	95	CCATTTCCACCACCTCCCTC	60.034	CACAGGCACAGTACACGACT	59.968	100
TRINITY_Contig3644	2	p3	(TGA)4	12	882	893	CCATTTCCACCACCTCCCTC	60.034	CACAGGCACAGTACACGACT	59.968	100
TRINITY_Contig3646	1	c	(CCG)5aggcagccgcccagcagggacctccacgccgacgaattctccttcctcgccctcatcgcggagctgagcagcgatgtcgaa(GCGAAG)3gtgaaagcgagagggtgatctgagttgttgcgtgcggtgggaggatggt(GTG)4	175	1625	1799	AGCGAGAGGGTGATCTGAGT	60.034	AAGCTCTCAAGAAACCCCCG	59.963	224
TRINITY_Contig3648	1	p2	(TC)7	14	488	501	ATTCTTAACAGCCCCAGCCC	60.033	TCCAAGTGGGCTAGCATTGG	60.034	187
TRINITY_Contig3648	2	p3	(TGC)5	15	897	911	ATTCTTAACAGCCCCAGCCC	60.033	TCCAAGTGGGCTAGCATTGG	60.034	187
TRINITY_Contig3648	3	p3	(CGC)4	12	1669	1680	ATTCTTAACAGCCCCAGCCC	60.033	TCCAAGTGGGCTAGCATTGG	60.034	187
TRINITY_Contig3648	4	p3	(GCT)4	12	1865	1876	ATTCTTAACAGCCCCAGCCC	60.033	TCCAAGTGGGCTAGCATTGG	60.034	187
TRINITY_Contig3648	5	p2	(CG)6	12	2711	2722	ATTCTTAACAGCCCCAGCCC	60.033	TCCAAGTGGGCTAGCATTGG	60.034	187
TRINITY_Contig3648	6	p2	(AG)6	12	2903	2914	ATTCTTAACAGCCCCAGCCC	60.033	TCCAAGTGGGCTAGCATTGG	60.034	187
TRINITY_Contig3649	1	p4	(ACAA)3	12	139	150	TGCCTTTCGCTTCTTCTCGT	59.966	AGCTACACGACAAGCGGAAA	59.967	186
TRINITY_Contig3649	2	p6	(TTCTGC)3	18	2775	2792	TGCCTTTCGCTTCTTCTCGT	59.966	AGCTACACGACAAGCGGAAA	59.967	186
TRINITY_Contig3650	1	p3	(GCC)4	12	1094	1105	CTCTTGATGGCCACCCACAT	60.034	TGTACGACAACATGGAGGCC	60.037	211
TRINITY_Contig3652	1	p3	(CCT)4	12	1624	1635	CAAACAACTTTCCGCGCTCA	59.971	AGCTGCTGAACTACTGCCAG	60.037	120
TRINITY_Contig3655	1	p3	(GCC)4	12	1565	1576	CAAAGGGAAAACTGCCACCG	59.968	CGTATTTGAAGCCAGCGACG	59.975	170
TRINITY_Contig3658	1	p3	(CTT)4	12	260	271	ATCATACGCATCGCCGAGTT	59.968	CAGGTGCGATCCGTAGAACA	59.827	124
TRINITY_Contig3660	1	p4	(CACC)3	12	32	43	ACTACCTGCGGCAATGTGAA	59.964	AAGTAGCCCAGCGTCTCAAC	60.037	167
TRINITY_Contig3660	2	p3	(ACG)4	12	304	315	ACTACCTGCGGCAATGTGAA	59.964	AAGTAGCCCAGCGTCTCAAC	60.037	167
TRINITY_Contig3661	1	c	(CCG)4ccaccgccaccgccgtcgctcatcactgtcgcggtgccagaccctgatgacccgctagaa(GCC)5	87	34	120	CGGATGTGGGATCTGCTCTC	59.967	ACATTCATCGCCTCCGTGTT	60.036	170
TRINITY_Contig3662	1	p3	(CGC)5	15	1481	1495	GGAGCACAAGATCCTGGACC	60.107	GCTTGATGATGAGCAGCAGC	59.97	167
TRINITY_Contig3666	1	p2	(TC)9	18	1975	1992	GCGCAGGTAGCTTACGAGAT	59.969	GCTTGTGGACTGGAGAGGAC	60.037	139
TRINITY_Contig3671	1	c	(CGG)5ctgggactacgaggcgcagctgcgggagctgcagcggcagcgcgactggtacctgatgagcacc(GCG)4	91	656	746	TATGGACGTGCAAGTTCCCC	60.036	CGCTGTCAGGAGGAAGGTTT	59.965	123
TRINITY_Contig3672	1	p3	(GTC)4	12	150	161	TTGTCGTCGTCGTCACCAAT	59.969	GCGAGGAACACCATGAAGGA	60.036	107
TRINITY_Contig3672	2	p3	(CCG)4	12	868	879	TTGTCGTCGTCGTCACCAAT	59.969	GCGAGGAACACCATGAAGGA	60.036	107
TRINITY_Contig3672	3	p3	(CGC)4	12	1002	1013	TTGTCGTCGTCGTCACCAAT	59.969	GCGAGGAACACCATGAAGGA	60.036	107
TRINITY_Contig3672	4	c	(AGCA)3cagtatcatcgatctaccacacccgggtgtgccctggccggtgtcgatcaattagttggttaattgttggatcattgtcgtcttgtactc(TG)8(CG)9gattaatgaagtagtagtttgcattggtgatgaatcccagccagtcagtaataaaagttgtggtagagtgtagctgctatgtt(TG)8	235	1134	1368	TTGTCGTCGTCGTCACCAAT	59.969	GCGAGGAACACCATGAAGGA	60.036	107
TRINITY_Contig3677	1	c	(GGC)4agccagcagcgcacgcctatggagcagccgagcaggagggccgagccaccgag(GGAAA)3	80	660	739	AAATCGAGCCCTATCGCCTG	59.966	AAAACTCTAGGCGCGGAGTC	60.109	246
TRINITY_Contig3679	1	p4	(GCAG)3	12	1880	1891	CGACGTAGGACATGACGAGG	59.972	CCCTCCCTTATAGCACGCAG	59.966	243
TRINITY_Contig3680	1	p3	(GGC)5	15	127	141	CGTGGTGTCTGTGTTCCTCA	59.896	CAGCCACACTAGCCATCGAT	59.894	140
TRINITY_Contig3683	1	c	(CCG)4tccctcgccctgctgccgctgcccacgccgcccccgccgccgtacctggagctggtgct(GCC)5	86	303	388	GGAGACAGTAGCAGCGGATC	59.969	TCGAATCGGATGCCACCAAA	60.036	271
TRINITY_Contig3685	1	p5	(GCGCG)3	15	432	446	CCACCTGCATGCGTTTTCTT	59.685	TTTTCTCATGGGAGGGTGCG	60.322	106
TRINITY_Contig3687	1	c	(TC)9(AC)14	46	852	897	CATCCTCCCTTATCCACGGC	59.965	CTGCATGCAAGCACAGTGTT	59.968	142
TRINITY_Contig3689	1	p4	(AAAG)4	16	891	906	AGCAACGCCAGTACAATCCA	59.964	CACTTGCCAGGTCGAGTTCT	59.966	115
TRINITY_Contig3691	1	p2	(AG)8	16	2265	2280	TTGGGGATCAGGGCAATCAC	60.033	GGAGTAGGAGTCAATCGCCG	59.969	101
TRINITY_Contig3694	1	p4	(TATC)3	12	886	897	AGGTATGTGCCCGTACGTTG	60.109	GGGAGAGGAGGGATCTTGGT	60.031	177
TRINITY_Contig3696	1	p3	(CCG)4	12	1310	1321	GACTATCGCCTCCTTTCGGG	59.968	CGACAGGGAGATGGAGATGC	59.967	227
TRINITY_Contig3697	1	p5	(CGCAC)3	15	18	32	CTGCAAGAAGGAAGGCCAGA	59.963	CAGCTCCTCCCTGTAGTCCT	60.033	160
TRINITY_Contig3697	2	p3	(TCC)5	15	149	163	CTGCAAGAAGGAAGGCCAGA	59.963	CAGCTCCTCCCTGTAGTCCT	60.033	160
TRINITY_Contig3697	3	p3	(GCG)5	15	827	841	CTGCAAGAAGGAAGGCCAGA	59.963	CAGCTCCTCCCTGTAGTCCT	60.033	160
TRINITY_Contig3698	1	p2	(AC)6	12	58	69	GATGAAGTGCTGCTCCTGGT	60.036	ACGGGATCCTACACCACTGA	59.959	243
TRINITY_Contig3698	2	p4	(TGGA)3	12	202	213	GATGAAGTGCTGCTCCTGGT	60.036	ACGGGATCCTACACCACTGA	59.959	243
TRINITY_Contig3698	3	c	(ATGG)3aatcctgcttgcaccatcagccttctctctctcttgtttttctctcacttcatgcctttttgctctc(CACTCT)3ctcctattccttttacctacacggctacacaacaaggacacctttgccagaggaacgtactcccgtgctcgctcgctttgcgtccacgta(CGGC)3ccgcctacatgaagtccacctcgaacagattgtgcatgct(GCC)4gttggtgttggtggtggtgttctggtcggagacctcgccgttggccgacggcagcgcgctggagcacaccgacgagcacgacaggatgttgtt(GCC)7	366	386	751	GATGAAGTGCTGCTCCTGGT	60.036	ACGGGATCCTACACCACTGA	59.959	243
TRINITY_Contig3698	4	p3	(GCT)4	12	859	870	GATGAAGTGCTGCTCCTGGT	60.036	ACGGGATCCTACACCACTGA	59.959	243
TRINITY_Contig3698	5	p3	(CGG)4	12	1107	1118	GATGAAGTGCTGCTCCTGGT	60.036	ACGGGATCCTACACCACTGA	59.959	243

TRINITY_Contig3698	6	c	(GCG)4ccatgggcgcgaggctggacggcagcgtgagcagc(TGG)4ggcgccgccgcctcctggaactgcagcgccgcggcgaagtcgagcccgtcgtagaggtggtgctgcatcccgccgccgcctg(GCC)6tcccaccacgccgtgctcgaacccgacgtagggcg(GCT)5	210	1600	1809	GATGAAGTGCTGCTCCTGGT	60.036	ACGGGATCCTACACCACTGA	59.959	243
TRINITY_Contig3698	7	p4	(GGGA)4	16	1927	1942	GATGAAGTGCTGCTCCTGGT	60.036	ACGGGATCCTACACCACTGA	59.959	243
TRINITY_Contig3702	1	p3	(GTC)4	12	453	464	GTTTAGCACAAGTCGGGGGA	59.964	CTAGGTGAGGGAGGTCGTCA	60.034	127
TRINITY_Contig3702	2	p3	(GGC)4	12	2001	2012	GTTTAGCACAAGTCGGGGGA	59.964	CTAGGTGAGGGAGGTCGTCA	60.034	127
TRINITY_Contig3705	1	p3	(GGC)4	12	3355	3366	GCATCATAACCGACACACGC	59.974	TCGTATGCAGGTTGCTCGTT	60.038	244
TRINITY_Contig3706	1	p3	(CTT)4	12	476	487	AGAGCCGGTATAAAGACGCG	59.97	AGGCCGAAGAAGAAGAAGCC	60.036	108
TRINITY_Contig3707	1	c	(GCC)4caagcagggatggggctcgtgg(TGC)4	46	945	990	GCGAGGGATGATTCAAACGC	59.972	GCACAGGACACCACTACTCC	60.037	202
TRINITY_Contig3708	1	p3	(GTG)5	15	297	311	TTTCCACGAGCAGCAGCATA	60.036	ATGATCGATCGTTCCGGTGG	59.968	135
TRINITY_Contig3708	2	p4	(GCAA)3	12	1186	1197	TTTCCACGAGCAGCAGCATA	60.036	ATGATCGATCGTTCCGGTGG	59.968	135
TRINITY_Contig3709	1	c	(AG)8ctgtgctgcctgtcattcttgtaatttagggaggggcgcaatcgccgcagctttgtga(TGGT)3	86	188	273	CATAAACCAGCTCGAACGCG	59.975	CGCCAGGAGCTTGAAGAAGA	60.037	182
TRINITY_Contig3711	1	p3	(CGG)4	12	1797	1808	ACTACAGCCTGGTAGCGGTA	60.034	ACGCGATCCAAGATCTCGAC	59.97	201
TRINITY_Contig3712	1	p3	(CCG)4	12	561	572	TGGCAGCGAAGACTACACAG	60.038	GCTCATCAGCGACATGGTCT	60.179	203
TRINITY_Contig3713	1	p4	(TCCC)3	12	4047	4058	GTTGTTGGAACTTGCCGACC	59.97	GGCAACACGCATTCGATCTC	59.973	161
TRINITY_Contig3714	1	p3	(TGC)7	21	2044	2064	CCTGGGATCATGCCACAGTT	60.034	GCACAAACAAGCCCTCCAAG	59.967	251
TRINITY_Contig3717	1	p6	(CACGTG)3	18	344	361	TCATCCAGAACAACGGACCG	60.038	TCGTCCTTGGTGATCATGGC	60.108	127
TRINITY_Contig3718	1	p2	(TC)7	14	206	219	AACTCGTCGCTACTGTACA	59.405	ATCACCATCAACCGGAGCAG	60.108	259
TRINITY_Contig3719	1	p6	(GGACGC)3	18	993	1010	CAGGTTCATGGTGGTCGTCA	59.965	AGGAGGGGAAGTGCAAATCG	60.035	254
TRINITY_Contig3719	2	p3	(CTC)4	12	1463	1474	CAGGTTCATGGTGGTCGTCA	59.965	AGGAGGGGAAGTGCAAATCG	60.035	254
TRINITY_Contig3720	1	p3	(CGT)4	12	968	979	GCAGAGGTTTGGAGAACGGA	59.965	ACATGGCCCCAGAAGTGATG	60.034	177
TRINITY_Contig3721	1	p6	(GGCGGA)3	18	2179	2196	CCCACATGGTCAGGTTCCTC	60.035	TTACCTTGAGGCCCATGCTG	60.034	254
TRINITY_Contig3723	1	p6	(GTGAGA)4	24	64	87	ACCTTGATGCAACAGAGGGG	59.961	GGGAGGAAGGGAATCTGCAG	59.818	116
TRINITY_Contig3724	1	p6	(CCTCTC)3	18	310	327	GGACACTTGCTAGCTCCGTT	60.037	GAATTGCCAAACTGCAGGGG	60.037	253
TRINITY_Contig3724	2	c	(GAG)5gaagatggcggc(GCAGGA)3	45	501	545	GGACACTTGCTAGCTCCGTT	60.037	GAATTGCCAAACTGCAGGGG	60.037	253
TRINITY_Contig3724	3	p3	(GCG)4	12	1046	1057	GGACACTTGCTAGCTCCGTT	60.037	GAATTGCCAAACTGCAGGGG	60.037	253
TRINITY_Contig3724	4	p3	(TCT)4	12	1234	1245	GGACACTTGCTAGCTCCGTT	60.037	GAATTGCCAAACTGCAGGGG	60.037	253
TRINITY_Contig3726	1	p2	(GA)6	12	1206	1217	ATACAGAAAGCCGCCACACA	59.964	GGTTGTTCGATCTTACGCGC	59.975	123
TRINITY_Contig3727	1	p5	(AGGCG)3	15	17	31	CAACTGGAGCTGAGAGCCTC	60.108	TCGAATTTCCGAAGCCCCAA	59.963	203
TRINITY_Contig3727	2	p4	(TGCC)4	16	187	202	CAACTGGAGCTGAGAGCCTC	60.108	TCGAATTTCCGAAGCCCCAA	59.963	203
TRINITY_Contig3727	3	p4	(TATG)3	12	568	579	CAACTGGAGCTGAGAGCCTC	60.108	TCGAATTTCCGAAGCCCCAA	59.963	203
TRINITY_Contig3730	1	p3	(CTC)4	12	42	53	ACTTCTCCTCCTCCTCCCAC	59.958	CAGATCCGGGCCAATATCCC	60.035	124
TRINITY_Contig3735	1	p3	(CAG)6	18	447	464	TTTTGGTGATGTTGCTGGCG	59.968	CTTGGAGTGTGGCCCTGTAG	60.036	144
TRINITY_Contig3735	2	p6	(GGAGGG)3	18	2261	2278	TTTTGGTGATGTTGCTGGCG	59.968	CTTGGAGTGTGGCCCTGTAG	60.036	144
TRINITY_Contig3740	1	p3	(CCG)5	15	70	84	GCTACAAAACCAAGCACCCG	60.04	GTACTTGGAGCAGCGAACCT	60.037	228
TRINITY_Contig3741	1	p3	(GCC)6	18	1518	1535	GATACTCTCCGGAACAGCGG	59.969	CTCTGTCAGTCACACGCCTT	59.967	100
TRINITY_Contig3741	2	p5	(GCGCG)3	15	1796	1810	GATACTCTCCGGAACAGCGG	59.969	CTCTGTCAGTCACACGCCTT	59.967	100
TRINITY_Contig3742	1	c	(CTGCG)3cctccccaccacccacga(CGCAC)3	48	162	209	TTTGGCGCATTGTTGTCAGG	59.968	GCCACACAACCACAATGGAC	59.968	234
TRINITY_Contig3742	2	c	(GA)8gggagggagaggaagcaacaaagaac(AACA)3	54	343	396	TTTGGCGCATTGTTGTCAGG	59.968	GCCACACAACCACAATGGAC	59.968	234
TRINITY_Contig3745	1	p2	(CT)10	20	1066	1085	AGCCCCAAGGACCAAAAACA	60.032	GAGTTGTGCTGCTAAGGGGT	59.963	142
TRINITY_Contig3745	2	p4	(CCAT)3	12	2330	2341	AGCCCCAAGGACCAAAAACA	60.032	GAGTTGTGCTGCTAAGGGGT	59.963	142
TRINITY_Contig3746	1	p4	(ACAA)3	12	428	439	GTTTTGAGTGCAGCCACCTG	59.969	GCTTCTCCAAGGTCCATGCT	60.035	146
TRINITY_Contig3747	1	p3	(CTG)4	12	450	461	GCAGGTAAACGTCCCTCACA	59.966	TCGAGAGTGTGTGTGTGTCG	59.971	131
TRINITY_Contig3747	2	p2	(AC)6	12	1058	1069	GCAGGTAAACGTCCCTCACA	59.966	TCGAGAGTGTGTGTGTGTCG	59.971	131
TRINITY_Contig3752	1	p3	(TGT)4	12	614	625	GAAACCAACACCGAACTGCC	59.97	CAAGTCCGACCTCGTCAACA	59.968	118
TRINITY_Contig3753	1	p3	(CAG)4	12	569	580	TGCTTGCACTCGTCTCTGAG	60.039	TCGGAGCCTCAGACTTCAGA	59.962	255
TRINITY_Contig3754	1	p6	(CCGGCG)3	18	951	968	CGAAGGTCGAACCCATCCAA	60.037	CTTGTCATGGGCGCAAACAA	59.968	203
TRINITY_Contig3755	1	p3	(GCG)4	12	602	613	CTACGCGGTCCCTCTTTCTC	59.899	CTGTCCTTCTCCATCGCTCC	59.896	102
TRINITY_Contig3757	1	p3	(GCG)5	15	610	624	CGATCGATCAGTAGGGGCAC	60.039	GGCAACGGCTACTTCCTCAT	60.108	253
TRINITY_Contig3758	1	p4	(TTCC)3	12	1003	1014	CGCACTTGGCATACATGTCG	59.973	AAATGGGGTCTGGCCAGATG	60.033	167
TRINITY_Contig3758	2	p3	(CGG)4	12	2376	2387	CGCACTTGGCATACATGTCG	59.973	AAATGGGGTCTGGCCAGATG	60.033	167
TRINITY_Contig3759	1	p3	(AGC)4	12	196	207	CTACCACCTACGCGACCATC	59.97	TTGAGACTGACGGGGTTGTG	59.894	104
TRINITY_Contig3760	1	c	(GCG)5ct(GCCAGC)3ttcgagcagcacgcgaagcggcgtcccccggcgtcgtc(GCA)5	88	146	233	CTTCCTCCACTTCCCACACC	59.963	CTGCTCATCGTGGTGGAGAG	60.179	115
TRINITY_Contig3761	1	p6	(GCGGCA)3	18	508	525	GAGACTAACACTACGGCCGG	59.9	CGCGGTCTGATTGTTGCATT	59.831	121
TRINITY_Contig3764	1	p3	(CGG)4	12	920	931	GGCAATGCCTTTTTGGGGTT	59.89	TCTGTTGGCTGCGTCATTCT	59.965	163
TRINITY_Contig3766	1	p3	(CCG)4	12	111	122	GGCTGGGGATCACCATCATC	60.251	AGCTTTCTCCTGTTGACCCG	59.965	179
TRINITY_Contig3766	2	c	(AGC)5tccacgaggctagtgtgagtagccactgtagctcatggctacagtagtctccctctctctgatgcatgcccgt(TGCG)3ccattcccgaggctggggatcaccatcatcgctacctagcagctcgctcgcgcgctcatcgtatctgcgcgcctccatgcgcgcgtcacctcccgcagca(TC)6	214	619	832	GGCTGGGGATCACCATCATC	60.251	AGCTTTCTCCTGTTGACCCG	59.965	179
TRINITY_Contig3768	1	p4	(TGTA)3	12	1976	1987	GGGGAAGGATTGATGGCCAA	60.032	TCAAGCATAGCAGTGGCACA	59.963	203
TRINITY_Contig3772	1	c	(CCTC)3gcaacactactgtcccatccatcgtctacctcccacctcatcact(TCCC)3	69	44	112	ATCACTTCCCTCCCTCCCTC	60.031	TGCTCCATGGAAGCAAGGAG	60.035	147
TRINITY_Contig3775	1	c	(CT)10tcatctatcaccattgcacgcccttcacacgacgcaaaagggtgcacacacacctcaagctcgcacatacactcccctctctccctctctatcc(AG)6	126	128	253	CATCACGGCATCTCTCTCCC	59.967	CGAAGTCGTCGTCCTCGTAG	59.975	247
TRINITY_Contig3775	2	p3	(TTC)4	12	911	922	CATCACGGCATCTCTCTCCC	59.967	CGAAGTCGTCGTCCTCGTAG	59.975	247
TRINITY_Contig3776	1	p3	(CCG)4	12	496	507	TCAGTCGGAGGTGACAAAGC	59.966	CTGCTAGCTGATCGGTCTCG	60.041	100
TRINITY_Contig3780	1	p6	(CGCCGT)4	24	244	267	CGGCGCCTGATAAGGAGATT	59.966	CCTTTCCCTCCACAGCTGAG	60.036	206
TRINITY_Contig3781	1	p4	(GAAT)3	12	910	921	TCTGCTCTGCACCATTCTGG	60.036	TGTGGGAAACTTGTGTGGCT	60.034	110
TRINITY_Contig3785	1	p3	(CGG)4	12	1467	1478	GTAGCTCGCGCATTGTTCAG	59.974	GCTCACCAAGTACTGCACCT	59.965	198
TRINITY_Contig3787	1	p4	(TCCC)3	12	338	349	CTTCCTCCTCCCTCCTCCTC	60.106	ATATATGCGTGAGGCGGACG	60.109	127
TRINITY_Contig3790	1	p4	(AGAT)5	20	1	20	TTGTTGCATCGTCTCTGGCT	59.965	CGGCGTTTCCAGCAAAAGAA	59.97	101
TRINITY_Contig3791	1	c	(GCG)4tcgctgccccgcctgcgcctgcgc(CTCCCT)3	54	82	135	ACACTAGACGAGCGCACAAA	59.968	TTATGGGCCGGTGACCTAGA	60.032	167
TRINITY_Contig3796	1	p4	(TCTT)3	12	995	1006	TGCCCGACAAGATCCACATC	60.108	TGTAAACGGATCGGCTACGG	59.899	175
TRINITY_Contig3797	1	p3	(CGG)8	24	2543	2566	CCCTCGTCAGAGCCACATTT	60.036	CAAGGCGCGGAGGAAGATAT	59.966	112
TRINITY_Contig3798	1	p6	(CCAACG)3	18	319	336	CGGAGCCAATCGTGATCGTA	59.97	CACGAGCTTCTCCTTGTGGT	59.966	200
TRINITY_Contig3800	1	p6	(TGAGGT)3	18	502	519	ATGTGTAGCTTCCTGGCCAC	60.035	AATGAAAGCGGGCTCTGACA	59.963	211
TRINITY_Contig3800	2	p3	(GCC)4	12	2990	3001	ATGTGTAGCTTCCTGGCCAC	60.035	AATGAAAGCGGGCTCTGACA	59.963	211
TRINITY_Contig3800	3	p3	(CGA)4	12	3343	3354	ATGTGTAGCTTCCTGGCCAC	60.035	AATGAAAGCGGGCTCTGACA	59.963	211
TRINITY_Contig3800	4	p3	(GGC)4	12	3509	3520	ATGTGTAGCTTCCTGGCCAC	60.035	AATGAAAGCGGGCTCTGACA	59.963	211
TRINITY_Contig3800	5	p3	(GCC)4	12	3692	3703	ATGTGTAGCTTCCTGGCCAC	60.035	AATGAAAGCGGGCTCTGACA	59.963	211
TRINITY_Contig3800	6	p3	(GAG)4	12	3972	3983	ATGTGTAGCTTCCTGGCCAC	60.035	AATGAAAGCGGGCTCTGACA	59.963	211
TRINITY_Contig3801	1	p4	(ACGC)3	12	173	184	GCTACGTGCTTGCCAAAGTC	60.11	TCTTTCGTTGGAGCAAGGCT	59.891	274
TRINITY_Contig3802	1	p4	(AGAT)3	12	153	164	AACTGCGCAGTGCTCAATTG	60.04	GGTGCGCCTTGAATTGTGTT	59.968	273
TRINITY_Contig3805	1	p3	(AGC)4	12	475	486	CAGGATCTGAGGGATGTCGC	59.967	CCGGCAGCATAATTCTGCAC	59.97	209
TRINITY_Contig3805	2	p4	(CGGC)3	12	698	709	CAGGATCTGAGGGATGTCGC	59.967	CCGGCAGCATAATTCTGCAC	59.97	209
TRINITY_Contig3808	1	p4	(TGCG)3	12	807	818	TCGACCGAACTCACCTGTTG	59.968	GTGCACCGACTTCTTCTCCA	59.966	215
TRINITY_Contig3810	1	p3	(AGC)4	12	56	67	ACGAAAACAAGCAGCAGCAG	59.97	AACTGGGCAGAACTCTGAGC	59.964	193
TRINITY_Contig3817	1	p3	(GCT)4	12	109	120	CACTCTTCGTCTGGATCCCG	59.899	ATTCTTGACTGCGCTCTCCC	60.108	189
TRINITY_Contig3819	1	p3	(GGC)4	12	1198	1209	CGTGGGATGGGGGATTTAGG	59.89	TCTCTCCTATCGGGCTACGG	59.964	108
TRINITY_Contig3821	1	p2	(CT)7	14	41	54	ACAAAAGAGTACGACGCGGT	59.969	CAAGACGCTGATCGAGACGA	59.903	243
TRINITY_Contig3828	1	p3	(AAG)4	12	1239	1250	CCCACCAAAGTTGCTGAAGC	59.967	ACAGGCCATCATGACACCTG	60.035	136
TRINITY_Contig3831	1	p4	(GAGG)5	20	737	756	ATCAACCGGGAACATGCCAT	60.033	TCGCAGACTTCTTGGTGGAC	59.966	151
TRINITY_Contig3836	1	p3	(GCG)4	12	106	117	CTACTCTGGCCCCTTCTCCT	60.032	GTGCAGCAACCTCAACAGTG	59.97	273
TRINITY_Contig3836	2	p3	(CCA)4	12	1014	1025	CTACTCTGGCCCCTTCTCCT	60.032	GTGCAGCAACCTCAACAGTG	59.97	273
TRINITY_Contig3836	3	p3	(GCG)4	12	1206	1217	CTACTCTGGCCCCTTCTCCT	60.032	GTGCAGCAACCTCAACAGTG	59.97	273
TRINITY_Contig3840	1	p3	(CAG)4	12	569	580	CTCGTTCTCATCCTCCGACG	59.972	CTGCAGTACAAGGAGGTGCA	59.965	273
TRINITY_Contig3840	2	p3	(CGA)4	12	899	910	CTCGTTCTCATCCTCCGACG	59.972	CTGCAGTACAAGGAGGTGCA	59.965	273
TRINITY_Contig3840	3	p3	(CCG)4	12	3141	3152	CTCGTTCTCATCCTCCGACG	59.972	CTGCAGTACAAGGAGGTGCA	59.965	273

TRINITY_Contig3840	4	p3	(CCT)4	12	3285	3296	CTCGTTCTCATCCTCCGACG	59.972	CTGCAGTACAAGGAGGTGCA	59.965	273
TRINITY_Contig3840	5	c	(GCG)4aga(AGG)4	27	3585	3611	CTCGTTCTCATCCTCCGACG	59.972	CTGCAGTACAAGGAGGTGCA	59.965	273
TRINITY_Contig3841	1	p4	(TCCC)3	12	575	586	TTGTGGCCTTCTCAGCTGAG	59.964	CTCCTTGTATCGACGGAGGC	59.969	107
TRINITY_Contig3843	1	p3	(CGC)5	15	132	146	CTTCGAAGCCACCAGGAACT	59.965	TCTCATGTGCCTGAAACGCT	59.965	265
TRINITY_Contig3843	2	p6	(AAGGAG)3	18	1049	1066	CTTCGAAGCCACCAGGAACT	59.965	TCTCATGTGCCTGAAACGCT	59.965	265
TRINITY_Contig3844	1	p3	(CCT)4	12	454	465	GGCACTACTGACCTGTCCAC	60.037	AGATGCACTTGGTCTCCTGC	60.036	232
TRINITY_Contig3844	2	p4	(TTCT)3	12	831	842	GGCACTACTGACCTGTCCAC	60.037	AGATGCACTTGGTCTCCTGC	60.036	232
TRINITY_Contig3847	1	p6	(CTGCGC)4	24	656	679	TTTCGACCAGACAACCTCCG	59.967	CCATCAACCCTGACAGGCAT	60.034	145
TRINITY_Contig3847	2	p2	(GC)6	12	3260	3271	TTTCGACCAGACAACCTCCG	59.967	CCATCAACCCTGACAGGCAT	60.034	145
TRINITY_Contig3850	1	p3	(GTC)5	15	1510	1524	GAACTCAGTTTGGGCGGAGA	59.965	CACGTCGCCTTTTGCTATCG	59.975	119
TRINITY_Contig3851	1	p3	(CCT)4	12	2660	2671	AACACACGAAGCAATGGCAC	59.969	CTGCCACCTCTGTGACTGAG	60.038	203
TRINITY_Contig3857	1	p3	(TTC)4	12	524	535	CCGTTTCCATCAGCCACTCT	60.036	GACCTAAGAACGTTGGGCCA	59.964	215
TRINITY_Contig3860	1	p4	(GACT)3	12	2	13	AAACACGTTCGCTCCAAAGC	59.971	TTCTGGTCGTACTTCGGCAC	60.039	264
TRINITY_Contig3862	1	c	(CCG)4ccatctcatgagaacacagacagcggcggcgggcgcgcacgcacacgaa(CGG)4	73	213	285	TGACCTTCTTCGCGGTCTTC	60.039	TTGGTTTCTTGCCAACCCCT	60.032	279
TRINITY_Contig3866	1	p6	(CCCTCC)3	18	373	390	ATGCTGTTCGTCTTCGGGTT	59.965	CCCCAGGCCTTTCCATTTCT	59.958	106
TRINITY_Contig3868	1	p3	(GCC)5	15	109	123	ATTAACCCTCCACTTGCCCG	60.034	ACCAACAGCGTCCTCAAGAG	59.966	251
TRINITY_Contig3868	2	c	(GCCG)3cgcgctttatgggccgagctggccggccgcggtggtaggtagttggcttgtggctgcgccgcgcccgtgccccgtggtggatggtccgggttcatcaagg(CTGCC)5ctgctcctcctcaaccgcttccacgaaccggggagaggcggcgcgagatggaaggggcgaagcagaagcggcggccgctgacggtgatgg(CCT)4	239	639	877	ATTAACCCTCCACTTGCCCG	60.034	ACCAACAGCGTCCTCAAGAG	59.966	251
TRINITY_Contig3868	3	p3	(CGC)5	15	5040	5054	ATTAACCCTCCACTTGCCCG	60.034	ACCAACAGCGTCCTCAAGAG	59.966	251
TRINITY_Contig3868	4	c	(GGA)4aggaagcacgc(AAGG)3	35	5440	5474	ATTAACCCTCCACTTGCCCG	60.034	ACCAACAGCGTCCTCAAGAG	59.966	251
TRINITY_Contig3869	1	p4	(GATG)3	12	16	27	TGGAACCAGTAGTGACGTGC	59.967	TCTTCTTCTCTGCTGCCTGC	60.037	125
TRINITY_Contig3869	2	c	(AGA)4aaggcag(AGGC)3	31	217	247	TGGAACCAGTAGTGACGTGC	59.967	TCTTCTTCTCTGCTGCCTGC	60.037	125
TRINITY_Contig3869	3	p3	(CAG)4	12	775	786	TGGAACCAGTAGTGACGTGC	59.967	TCTTCTTCTCTGCTGCCTGC	60.037	125
TRINITY_Contig3869	4	c*	(AT)6(<AT>GC)(ATGC)2	24	953	976	TGGAACCAGTAGTGACGTGC	59.967	TCTTCTTCTCTGCTGCCTGC	60.037	125
TRINITY_Contig3873	1	p3	(TGC)4	12	1142	1153	AGGGCTGGGAGATGGAGAAT	60.029	CTATTGGGGGAGGGGGTGTA	60.029	221
TRINITY_Contig3878	1	p3	(CCG)5	15	858	872	CAGCTGCACCTTCTTCTGGA	59.964	CCGATGGAATCAGACGCTGA	59.897	266
TRINITY_Contig3879	1	p3	(CGC)4	12	308	319	CTGTCAGATCTTGCTGGGCA	60.036	AGACACAGCCACGCATAACA	59.965	261
TRINITY_Contig3879	2	p3	(GGT)4	12	845	856	CTGTCAGATCTTGCTGGGCA	60.036	AGACACAGCCACGCATAACA	59.965	261
TRINITY_Contig3883	1	p3	(GAG)5	15	1524	1538	ACAACCCATGCCAGTGCTAA	59.888	TTCCCCCATTGTCATCTGGC	60.033	200
TRINITY_Contig3886	1	p3	(GCC)4	12	1338	1349	GTGGGGGCGGAGTATTTGAA	60.035	ACCACCGCCGTTTGGATATT	60.035	207
TRINITY_Contig3886	2	p3	(GCC)4	12	1797	1808	GTGGGGGCGGAGTATTTGAA	60.035	ACCACCGCCGTTTGGATATT	60.035	207
TRINITY_Contig3886	3	p3	(CCG)4	12	2002	2013	GTGGGGGCGGAGTATTTGAA	60.035	ACCACCGCCGTTTGGATATT	60.035	207
TRINITY_Contig3886	4	p3	(GCG)6	18	2280	2297	GTGGGGGCGGAGTATTTGAA	60.035	ACCACCGCCGTTTGGATATT	60.035	207
TRINITY_Contig3889	1	p4	(AGCT)3	12	652	663	CGGCTTATTTTACGGCAGCC	59.971	CACGCGCGGGATAGAAAAAG	59.974	102
TRINITY_Contig3889	2	p3	(CTA)4	12	829	840	CGGCTTATTTTACGGCAGCC	59.971	CACGCGCGGGATAGAAAAAG	59.974	102
TRINITY_Contig3890	1	p4	(GCCG)3	12	274	285	TCTCCCCAGTATGCAGAGCT	60.032	GAGCTCATCAGGTACGAGGC	59.969	260
TRINITY_Contig3890	2	p3	(GCG)4	12	642	653	TCTCCCCAGTATGCAGAGCT	60.032	GAGCTCATCAGGTACGAGGC	59.969	260
TRINITY_Contig3890	3	p3	(GCT)5	15	1169	1183	TCTCCCCAGTATGCAGAGCT	60.032	GAGCTCATCAGGTACGAGGC	59.969	260
TRINITY_Contig3891	1	p3	(GCC)5	15	51	65	GGCCCACTATTCGCTCCTAC	59.966	TATGTGATGACATCCGGCGG	59.967	124
TRINITY_Contig3896	1	p4	(GGGA)3	12	473	484	CATTCGACGTTGCTTTCGCT	59.836	GGATCTTGGGCGGCTATCTC	60.037	203
TRINITY_Contig3896	2	p4	(GGGA)3	12	795	806	CATTCGACGTTGCTTTCGCT	59.836	GGATCTTGGGCGGCTATCTC	60.037	203
TRINITY_Contig3897	1	p3	(CGG)4	12	56	67	AGAGAGACAGAGGGCGATGT	60.034	GAGGAGGAGTCGGAGTCAGT	60.035	156
TRINITY_Contig3898	1	p6	(GGCGCA)3	18	3356	3373	GCACAGCAACACAGATGCAA	59.969	GAGGGGAAGGAGCTGCAATT	60.034	120
TRINITY_Contig3899	1	p3	(AGC)4	12	143	154	CACAACACTCCGGTGCTTTG	59.971	CTCCCTGAGGCCTTTCTTGG	60.035	190
TRINITY_Contig3905	1	p3	(GGA)4	12	399	410	CCATGATCGCAGTCGGATGA	59.967	GCATGACTCGGTGGATAGGG	59.966	174
TRINITY_Contig3905	2	p3	(CGC)4	12	1922	1933	CCATGATCGCAGTCGGATGA	59.967	GCATGACTCGGTGGATAGGG	59.966	174
TRINITY_Contig3906	1	c	(GAA)4gagtcgcccgcgaagaaagaaaacctccccgcgaaggaagcgaagccaag(GAC)4	74	2201	2274	CGAACTCTGTCCACCAACGA	59.968	CACGAAAAAGCAGCGGAACA	59.971	259
TRINITY_Contig3908	1	p6	(CGGCCT)3	18	121	138	TCCCGTTATCTCTGATGCGC	59.968	GACGAGTGCTCTAGCCGTAC	59.972	252
TRINITY_Contig3909	1	p6	(TCGTGG)3	18	454	471	CAAGTGCGACCCTGGTTTTG	59.969	CGCTCAAGCACGTTAAGAGC	59.906	152
TRINITY_Contig3909	2	c	(TCA)4ccgtactcacgttcctcgtcgtcatcaccttcgtcatcctca(TCGTGG)3	72	778	849	CAAGTGCGACCCTGGTTTTG	59.969	CGCTCAAGCACGTTAAGAGC	59.906	152
TRINITY_Contig3909	3	p3	(GGC)4	12	1113	1124	CAAGTGCGACCCTGGTTTTG	59.969	CGCTCAAGCACGTTAAGAGC	59.906	152
TRINITY_Contig3910	1	p3	(CGC)4	12	719	730	AGGCGGGTTGTGGGATAAAG	60.034	GCTCGGTCTTCAGACACTCC	60.109	102
TRINITY_Contig3916	1	p3	(CGA)4	12	721	732	GTTGAACTGCTTGCCCTTGG	59.967	CCTCCTGTAATCTGGGCGTC	59.895	206
TRINITY_Contig3918	1	p4	(CTCG)3	12	217	228	GCGTGTGTTGCAGTTGCTAA	59.971	AGGCAAAGATCCAGCGAACA	59.963	128
TRINITY_Contig3919	1	p5	(CCCTC)3	15	23	37	CGATGATGACATTGCTGGCG	60.042	AGCACACCAATCGAGAGACA	59.031	158
TRINITY_Contig3920	1	p3	(CGC)5	15	1040	1054	ACCTTCAGTGGGTGCGTAAG	59.965	TACGAGGTGCTCTCAGACCA	59.962	182
TRINITY_Contig3921	1	p3	(GAG)5	15	923	937	CAACGAGGAGGAGGAGGAGA	60.034	TTCCCTCCAAAAACACCGCT	60.106	181
TRINITY_Contig3922	1	p3	(AGC)4	12	933	944	CTGGTTTCAGGTCGAGGGTC	60.037	GGCTGGGTTCAGGACGTTTA	59.964	149
TRINITY_Contig3922	2	p6	(AGGGGA)3	18	1080	1097	CTGGTTTCAGGTCGAGGGTC	60.037	GGCTGGGTTCAGGACGTTTA	59.964	149
TRINITY_Contig3923	1	p3	(CGC)4	12	400	411	CAGTGGGTTCTCCGTTCTCC	60.037	GGAATGGACATCCTAGCCCG	59.965	164
TRINITY_Contig3929	1	p3	(CTT)4	12	454	465	CGTACCCAGGTCACGATACG	59.971	TTAACTCCGCCTGTGTGGAC	59.966	265
TRINITY_Contig3930	1	p4	(TTTG)3	12	166	177	CCTCGTGAATCATGCATGCG	60.042	TTCACGGTCTCAGCAGTGTC	59.968	267
TRINITY_Contig3931	1	p3	(TGA)5	15	3	17	ACGTAGAAGGTGACGCCAAG	60.039	TCTTGAGCCACTTGTCGACC	59.966	207
TRINITY_Contig3931	2	p3	(GGA)4	12	703	714	ACGTAGAAGGTGACGCCAAG	60.039	TCTTGAGCCACTTGTCGACC	59.966	207
TRINITY_Contig3932	1	p5	(CTTTT)4	20	678	697	AGCGACCATTTCAGACCGTT	59.965	CAGCAAGCAGAGCAGCAAAA	59.968	118
TRINITY_Contig3935	1	c	(CTCC)4ctcgccggtgggccgcgggagctccgagcagtggccgcggcggcccggcgcacggcgtccggatcaggcgcg(GGC)4	100	286	385	GGAGCTCGTCCGCTTCTTTA	59.827	GTGCTATTTCACCCGTCCCA	60.036	163
TRINITY_Contig3936	1	p3	(TGC)7	21	423	443	CATTTTCCTGGCGTTGGGTG	60.038	GAGCAGGAACACCACGAGAA	59.966	136
TRINITY_Contig3936	2	p3	(GCT)4	12	849	860	CATTTTCCTGGCGTTGGGTG	60.038	GAGCAGGAACACCACGAGAA	59.966	136
TRINITY_Contig3937	1	p3	(CCT)5	15	465	479	AAAATAGAAATCCCGCGCGC	59.971	GCAAGTTGTTTCGTGCGACT	59.972	177
TRINITY_Contig3940	1	p5	(GCGCG)3	15	1160	1174	ACGACTAACTCCGATCCCCA	60.034	ATGGCTGGCTGCTACCATTT	60.033	243
TRINITY_Contig3943	1	p3	(CAC)4	12	549	560	CCACCGAGCAAGCAACTCTA	60.037	GCAGAGTCTAGCTAGCCACG	59.97	100
TRINITY_Contig3943	2	p4	(GCCG)3	12	3455	3466	CCACCGAGCAAGCAACTCTA	60.037	GCAGAGTCTAGCTAGCCACG	59.97	100
TRINITY_Contig3943	3	p4	(CGGC)3	12	4905	4916	CCACCGAGCAAGCAACTCTA	60.037	GCAGAGTCTAGCTAGCCACG	59.97	100
TRINITY_Contig3946	1	p3	(ATG)4	12	850	861	CTGGTGGCATCAGCACAAAC	60.039	CCTCGATGATCCTGCCAGTC	59.967	125
TRINITY_Contig3947	1	p4	(GCCG)3	12	625	636	TGAAGGGCAGCTTGTACTCG	60.037	GTGGTCAAGCTCGAGAACCA	59.966	151
TRINITY_Contig3947	2	p3	(TGG)5	15	1761	1775	TGAAGGGCAGCTTGTACTCG	60.037	GTGGTCAAGCTCGAGAACCA	59.966	151
TRINITY_Contig3950	1	p3	(CGT)4	12	795	806	CCTCCTCGTGGAAGAACACC	60.037	GTGCACCAAACCTTCCGTTC	59.97	159
TRINITY_Contig3950	2	c	(GTC)4gtactagcgcctgcca(TC)11	50	1448	1497	CCTCCTCGTGGAAGAACACC	60.037	GTGCACCAAACCTTCCGTTC	59.97	159
TRINITY_Contig3953	1	p3	(TCT)4	12	136	147	TTCTTGGTCGTTGGGACTGG	59.892	ACGAGGAGAACGACGTGAAC	60.041	217
TRINITY_Contig3954	1	p4	(ACAA)3	12	268	279	TCCGAACCCACAAACCTAGC	59.964	TCTCGTCATGGTCGTGTTGG	60.039	189
TRINITY_Contig3954	2	p3	(GGC)6	18	804	821	TCCGAACCCACAAACCTAGC	59.964	TCTCGTCATGGTCGTGTTGG	60.039	189
TRINITY_Contig3956	1	c	(GTC)4(CTC)5cttctgctcgtcggatgcttgaggacggtcgt(GGC)5	74	4095	4168	CTCCTCCTCCTCCTTCTGCT	60.032	CTCCCTCATCGCCTTCATCC	59.965	175
TRINITY_Contig3956	2	p2	(AG)17	34	4492	4525	CTCCTCCTCCTCCTTCTGCT	60.032	CTCCCTCATCGCCTTCATCC	59.965	175
TRINITY_Contig3960	1	p5	(AAGAA)3	15	209	223	GAAGAGTGTGCGGGACTTGA	59.966	CAGCTTGAAAGGCCAGAGGA	59.963	199
TRINITY_Contig3961	1	p3	(TGC)4	12	2151	2162	TCCTGTAGGTGGCGATCTCA	60.034	GAACTCAACCGCATGATCGC	59.973	253
TRINITY_Contig3966	1	p3	(CTC)4	12	300	311	GCCTGCACAGCGACATATTG	59.971	TGTGTGACGAGTTGGACGAG	59.97	121
TRINITY_Contig3967	1	p3	(GCA)4	12	2201	2212	TGGTACAGCATGGAACGGTC	60.037	ACAGCCGTGCTTAAGTACCC	60.037	222
TRINITY_Contig3968	1	p2	(CG)6	12	133	144	GCATTCTTGCGATTCGGCAT	59.97	CGCGATCCAATGGAAGCAAG	59.971	165
TRINITY_Contig3970	1	p3	(CAG)5	15	1276	1290	ACTGGAAGACGTTCGTCACC	59.968	TTGTACTTGACGCCCCACTC	59.966	196
TRINITY_Contig3971	1	c	(CCG)4gat(CCG)4	27	272	298	GCCGGGTTCGATGATCTGAT	59.966	AAACTATCCGCAGCAGCCTT	60.035	257
TRINITY_Contig3971	2	c	(CGC)4cagcgt(ACG)4	30	611	640	GCCGGGTTCGATGATCTGAT	59.966	AAACTATCCGCAGCAGCCTT	60.035	257
TRINITY_Contig3976	1	p3	(CCG)4	12	143	154	GGAGAAGTGGGTCAAGGAGC	60.036	GGCTTCAGCTTGCGAATACG	59.973	246
TRINITY_Contig3977	1	p3	(CGC)4	12	455	466	GAGAGACGCTCAACCTCGAC	60.179	ATCGTCTGGTCCATTTGCGT	60.036	136
TRINITY_Contig3978	1	c	(GCG)4agggaggggcgggtgacaggtggggcccgcccccccccgagcctcgccctcccgcctccgcccctgacgagcgggccccacctgtcacccgcccctccct(CGC)4	124	1023	1146	CCCCTTTGGTGAGATGCCAT	60.033	CGCTCGGCCATCAATTTGAG	59.971	138
TRINITY_Contig3978	2	p4	(TTGG)3	12	6331	6342	CCCCTTTGGTGAGATGCCAT	60.033	CGCTCGGCCATCAATTTGAG	59.971	138
TRINITY_Contig3979	1	p4	(CCGG)3	12	28	39	TGCTGCGATTTACCACCTGT	59.964	CGGAGAAGATGAGGACGACG	59.972	252
TRINITY_Contig3979	2	p4	(TACA)3	12	239	250	TGCTGCGATTTACCACCTGT	59.964	CGGAGAAGATGAGGACGACG	59.972	252
TRINITY_Contig3979	3	p3	(TCG)5	15	607	621	TGCTGCGATTTACCACCTGT	59.964	CGGAGAAGATGAGGACGACG	59.972	252
TRINITY_Contig3979	4	p3	(GAG)4	12	2871	2882	TGCTGCGATTTACCACCTGT	59.964	CGGAGAAGATGAGGACGACG	59.972	252

TRINITY_Contig3980	1	p3	(GCC)5	15	1526	1540	AACCCAGAGCTTCCAAGACG	59.965	GTCCGCTTTGGGTTGGTTTC	59.968	242
TRINITY_Contig3984	1	p3	(TCC)8	24	114	137	TTTGGGGGCTGGTTCAAACT	60.032	TCCCACAGTTTCTCTCGCAC	59.966	221
TRINITY_Contig3985	1	p3	(CGC)4	12	440	451	TTCCTCTTCGCCATGTTCCC	60.035	TGCAGCCACCGGTATCATTT	60.035	200
TRINITY_Contig3987	1	p3	(CTC)4	12	1508	1519	CTTCTCTGCGACGGAGGATC	59.97	TGCTCGGTATGTGTGTGCTT	59.965	111
TRINITY_Contig3987	2	p3	(GGC)4	12	2307	2318	CTTCTCTGCGACGGAGGATC	59.97	TGCTCGGTATGTGTGTGCTT	59.965	111
TRINITY_Contig3987	3	p2	(CT)8	16	2553	2568	CTTCTCTGCGACGGAGGATC	59.97	TGCTCGGTATGTGTGTGCTT	59.965	111
TRINITY_Contig3990	1	p4	(GCTC)5	20	12	31	GCCCCTAGACAACAGACACC	60.036	TGGCATTCTAGCGTGTTGGT	59.964	178
TRINITY_Contig3990	2	p3	(GCT)4	12	1284	1295	GCCCCTAGACAACAGACACC	60.036	TGGCATTCTAGCGTGTTGGT	59.964	178
TRINITY_Contig3992	1	p3	(CGG)4	12	863	874	AACCTCACTCGCCTCACAAG	59.966	TTCCGGGATAGCTCCACTGA	60.033	204
TRINITY_Contig3992	2	p3	(GCC)4	12	1275	1286	AACCTCACTCGCCTCACAAG	59.966	TTCCGGGATAGCTCCACTGA	60.033	204
TRINITY_Contig3992	3	c	(GGC)4cttctcccgcaccttctcccgcgcgccctcgctccgcgcgacgcccacattctcccgcgtcgcgtccacccgggtcggccccag(CTC)4ccgctccac(CTCCAG)3	135	1445	1579	AACCTCACTCGCCTCACAAG	59.966	TTCCGGGATAGCTCCACTGA	60.033	204
TRINITY_Contig3992	4	c	(GAC)5gaggat(GGC)6gcgcgcaagaccggcgacgg(CGA)5	74	1746	1819	AACCTCACTCGCCTCACAAG	59.966	TTCCGGGATAGCTCCACTGA	60.033	204
TRINITY_Contig3992	5	p3	(TCT)7	21	2091	2111	AACCTCACTCGCCTCACAAG	59.966	TTCCGGGATAGCTCCACTGA	60.033	204
TRINITY_Contig3993	1	p3	(CGC)4	12	1081	1092	TGGTCTTTGAAGTCGAGGGC	59.965	GGAGGCGATCAGTAGCTGTC	59.969	244
TRINITY_Contig3994	1	c	(GCCG)3gcaccccgcggccgcgcgaggcgatgcccggcccgatcgcggcgtgcttccgctgcgcc(GCG)4	83	237	319	AGGCTTGCAGAAGAGGTGAC	59.964	TCCAATTGTGTCTGAGCCCC	59.962	104
TRINITY_Contig4001	1	p3	(CGC)6	18	1363	1380	CACTCTTCAGGCGTCACAGT	59.967	TTGATGGGAAGCCACCATCC	60.033	232
TRINITY_Contig4001	2	c	(CACG)3cgctcgtgcccgcgcgagctggaaccgaagccgcagggaagggtttacagct(CG)6	76	1728	1803	CACTCTTCAGGCGTCACAGT	59.967	TTGATGGGAAGCCACCATCC	60.033	232
TRINITY_Contig4002	1	p3	(CCG)4	12	306	317	TCCACCGGAGCTTCTTGAAC	59.965	GACGTCTACAGGGTGCTGAG	59.829	127
TRINITY_Contig4002	2	p3	(GCT)4	12	1036	1047	TCCACCGGAGCTTCTTGAAC	59.965	GACGTCTACAGGGTGCTGAG	59.829	127
TRINITY_Contig4008	1	p3	(GGC)4	12	217	228	GGATCTCGTGAGCCTCATCG	60.04	CGCTGCATCGCCTTCTTTAC	59.973	234
TRINITY_Contig4009	1	p4	(TCTT)3	12	659	670	TTGTTCTGAGTCCGCCCTTC	59.965	AGCTACGACGACTAGCATGC	59.97	249
TRINITY_Contig4009	2	p3	(GCT)4	12	2592	2603	TTGTTCTGAGTCCGCCCTTC	59.965	AGCTACGACGACTAGCATGC	59.97	249
TRINITY_Contig4012	1	c	(CGGCGT)3cgggcgacatccgattggtggacgggagggg(AGC)5	64	2573	2636	GATTCGCATTGTCCGTCAGC	59.973	GATGCCCCAAGTGGAAGTGA	59.962	185
TRINITY_Contig4013	1	c	(CATA)3ctggaagtatacatacatatagtacatgcatgatgatcacagacagacacacac(AG)7aaacaaaaaaaaactagaaataaaagatcacatgggcaggaggtactggaattcgtagtccagggggatg(ACATCC)3	168	1	168	TCCACATCCTCCGTCTCCAT	60.032	CATCTCCAGCACTCCGGATC	59.967	274
TRINITY_Contig4013	2	c	(CATGGC)3tgcttctgcttct(TTA)5	46	1218	1263	TCCACATCCTCCGTCTCCAT	60.032	CATCTCCAGCACTCCGGATC	59.967	274
TRINITY_Contig4013	3	p3	(CCT)5	15	1372	1386	TCCACATCCTCCGTCTCCAT	60.032	CATCTCCAGCACTCCGGATC	59.967	274
TRINITY_Contig4013	4	p3	(CAG)9	27	1790	1816	TCCACATCCTCCGTCTCCAT	60.032	CATCTCCAGCACTCCGGATC	59.967	274
TRINITY_Contig4014	1	p4	(TGGT)3	12	130	141	TAAAAGGCGTTGGCAAAGGC	59.967	GTTTCAGGTCGTCGGTGGAT	60.038	191
TRINITY_Contig4014	2	p5	(CCCTC)3	15	1030	1044	TAAAAGGCGTTGGCAAAGGC	59.967	GTTTCAGGTCGTCGGTGGAT	60.038	191
TRINITY_Contig4015	1	p5	(CCCGG)3	15	50	64	GCTTCGCCTTTTCCTTTCCC	59.756	AGGAGAATTGGAAAGCGGGG	60.034	100
TRINITY_Contig4015	2	p2	(GC)6	12	412	423	GCTTCGCCTTTTCCTTTCCC	59.756	AGGAGAATTGGAAAGCGGGG	60.034	100
TRINITY_Contig4017	1	p4	(CTCC)3	12	21	32	ACCCTACCCAGTCCCATCTC	60.031	GGTTTGTTCCTCTCCTCTCGG	60.036	259
TRINITY_Contig4017	2	p3	(CGA)4	12	526	537	ACCCTACCCAGTCCCATCTC	60.031	GGTTTGTTCCTCTCCTCTCGG	60.036	259
TRINITY_Contig4017	3	p6	(GAACTC)3	18	1080	1097	ACCCTACCCAGTCCCATCTC	60.031	GGTTTGTTCCTCTCCTCTCGG	60.036	259
TRINITY_Contig4023	1	p3	(TCG)4	12	871	882	GGAGGTTGGGGGTGAGAAAG	59.962	TCGTGCCATCTGGGTTGTAC	60.037	133
TRINITY_Contig4027	1	p3	(CCG)7	21	4096	4116	GCTGCAGCAATCGTCATGAG	59.972	GGAACGCCTGGTATAGCCTC	59.966	209
TRINITY_Contig4027	2	p3	(GCC)6	18	4218	4235	GCTGCAGCAATCGTCATGAG	59.972	GGAACGCCTGGTATAGCCTC	59.966	209
TRINITY_Contig4027	3	p3	(CTG)5	15	5355	5369	GCTGCAGCAATCGTCATGAG	59.972	GGAACGCCTGGTATAGCCTC	59.966	209
TRINITY_Contig4027	4	p4	(CGGG)3	12	5564	5575	GCTGCAGCAATCGTCATGAG	59.972	GGAACGCCTGGTATAGCCTC	59.966	209
TRINITY_Contig4027	5	p2	(GA)6	12	5898	5909	GCTGCAGCAATCGTCATGAG	59.972	GGAACGCCTGGTATAGCCTC	59.966	209
TRINITY_Contig4028	1	p3	(GTC)4	12	3398	3409	TCGCTCCTTCCACAAGTTCC	59.965	CGACTGTGCCACTCAGAACT	59.967	249
TRINITY_Contig4029	1	p3	(ACG)4	12	1033	1044	ACAGGACGAGCGTGGAAAAT	59.965	CTCTCTCTCCCTCCACTGCT	60.033	263
TRINITY_Contig4029	2	p3	(CCT)4	12	1337	1348	ACAGGACGAGCGTGGAAAAT	59.965	CTCTCTCTCCCTCCACTGCT	60.033	263
TRINITY_Contig4029	3	p4	(GGCT)4	16	1918	1933	ACAGGACGAGCGTGGAAAAT	59.965	CTCTCTCTCCCTCCACTGCT	60.033	263
TRINITY_Contig4032	1	p3	(CGT)4	12	70	81	CTCCTGGTTCCGTCAGTCTG	59.755	CACACACACTTTACTCGCGC	60.111	152
TRINITY_Contig4032	2	p3	(GCC)4	12	405	416	CTCCTGGTTCCGTCAGTCTG	59.755	CACACACACTTTACTCGCGC	60.111	152
TRINITY_Contig4032	3	c	(CGT)4catcctggagcagccgccggtggtgaactgccttggccacggcgctcgcgaagccggcggccgacacgcttg(CGC)4	96	1093	1188	CTCCTGGTTCCGTCAGTCTG	59.755	CACACACACTTTACTCGCGC	60.111	152
TRINITY_Contig4032	4	c	(TGG)4cgcttgtgcccgtggcattgggttgtgccggcggtggtcaggaggaggagcgc(GAG)4	77	1776	1852	CTCCTGGTTCCGTCAGTCTG	59.755	CACACACACTTTACTCGCGC	60.111	152
TRINITY_Contig4033	1	p3	(GCC)4	12	569	580	ACTTGATCAGTGTGAGCGCA	59.966	TCACTGAGCTGGCTAGGCTA	60.033	175
TRINITY_Contig4036	1	p5	(CGGCG)4	20	83	102	TGTAATGATTCACGGGCGCT	60.108	AACTATCGGTGCTGCTAGGC	59.895	146
TRINITY_Contig4037	1	p3	(CCG)4	12	88	99	TTGACTGGGCGAGAATGACC	60.036	AGATCTTCTGCACCCCAAGC	60.035	176
TRINITY_Contig4038	1	p3	(CTG)4	12	586	597	AAGGATGGCCACCTTGTAGC	60.034	GTCGCCATACTGTACGGAGG	59.97	262
TRINITY_Contig4039	1	p2	(TC)8	16	26	41	TCTACGGTTTGACCTTGGGC	59.964	CATCTCCCCAAGTTCCCCAC	60.034	175
TRINITY_Contig4043	1	p2	(CA)6	12	1	12	CCAGTTCAACCAGTGCAAGC	59.969	CCCCAAGACAGAATGCAGGT	59.961	149
TRINITY_Contig4044	1	p3	(ATC)4	12	421	432	AACTCATGAACCTCGCCGTT	59.965	ACTCTTCTTGGTGCCTGTCG	59.966	236
TRINITY_Contig4046	1	p3	(GAC)5	15	256	270	TGCCTTGCGAGTTCACTGAT	59.965	TTCCCCTGGTTGGTGCTTTT	60.032	238
TRINITY_Contig4046	2	c	(CGT)4aggcgccgacaccgagcacggcgccggcgtcttcgtcgacgccgtcgtaggtgctgccgccgc(CGT)4	87	386	472	TGCCTTGCGAGTTCACTGAT	59.965	TTCCCCTGGTTGGTGCTTTT	60.032	238
TRINITY_Contig4047	1	p4	(GGCC)3	12	280	291	TCACGATCCTTGGCGTTTGA	59.966	TCTTCGACAAGCTGTGGGTC	59.966	264
TRINITY_Contig4047	2	p3	(CCG)5	15	436	450	TCACGATCCTTGGCGTTTGA	59.966	TCTTCGACAAGCTGTGGGTC	59.966	264
TRINITY_Contig4050	1	p2	(TC)6	12	666	677	CCGTATTGCGTCAACAGCAG	59.905	CTAGAAGACCGCACTTGCCA	60.037	109
TRINITY_Contig4055	1	p5	(TGGGA)4	20	342	361	TCCGTTCCCAGCTTCCATTC	60.035	ATCCCATCCCATCCAGGAA	60.028	200
TRINITY_Contig4057	1	p3	(GCG)4	12	349	360	CCAATGCTACGGAGGGGATC	59.965	CGTGGAGAAAACGTCTGGGA	59.967	275
TRINITY_Contig4058	1	p3	(CGC)5	15	131	145	CGAAGACGACGAGGAGGATG	59.972	CCTTCCACTGGGTGAGGTTC	59.963	245
TRINITY_Contig4061	1	c	(GTCG)3ggttggctcgcgagcgcggagctctct(CTCC)3cccgccggcgagtagggtttagcgaaggggccggccgcgcgacggcgggcttt(GCGG)3	116	927	1042	TCTCGAACTTGATGCGGGAC	60.109	CAAGCATTTCTAGCGCCGTC	59.973	163
TRINITY_Contig4062	1	p3	(GAC)4	12	80	91	CTGAGTGCGGTCCTCGTATC	59.97	CAACCCAGTGCTTGTGCTTC	59.969	219
TRINITY_Contig4062	2	c	(CAC)5tac(GAA)4	30	1067	1096	CTGAGTGCGGTCCTCGTATC	59.97	CAACCCAGTGCTTGTGCTTC	59.969	219
TRINITY_Contig4063	1	p3	(GCC)6	18	38	55	TCAGGCCACCAGATCTAGCT	60.032	CTCAGCTCAGCTCAGTCTCG	59.901	219
TRINITY_Contig4063	2	c	(GCAG)3gctatagggggggagaggcgaga(CTGAG)3ctgcgactctgcgcgagacggagagaggcctcaggccggccgggagaagagaaggag(AGCC)3	119	535	653	TCAGGCCACCAGATCTAGCT	60.032	CTCAGCTCAGCTCAGTCTCG	59.901	219
TRINITY_Contig4064	1	c	(TTCGA)3ttcaacgccgggaccttgtcaa(AGC)4	49	1619	1667	TGGAGGAGAGGATGTCCAGG	60.032	TGTAGGACGAAGAGGAGGGG	60.033	242
TRINITY_Contig4073	1	p2	(GA)6	12	52	63	ACAGACCCAAATGAGCTGCA	59.889	TGTTGTACCCGAGCCAAGAC	59.966	278
TRINITY_Contig4074	1	p2	(GA)6	12	2079	2090	GCTACTTGAACGAGGCAGGT	60.037	TGTTGGTGCGATTTTGGCTG	59.968	151
TRINITY_Contig4077	1	p4	(CCGG)3	12	815	826	AACACCATGCCGATCAGTGT	59.963	TCGACACACATCGCTGAACA	59.969	116
TRINITY_Contig4082	1	c	(GCG)4gccggcctcacacgggcgcgccctcctcgctccggcggaggacggaggacggcgcgcgcagaggccggtcggcccctcctctca(GGC)4	108	1879	1986	CAAAGCATCGATTCCGGCAG	59.971	GATTTGTGTTGCAGGCGTGT	59.969	163
TRINITY_Contig4082	2	p4	(CTCC)3	12	2665	2676	CAAAGCATCGATTCCGGCAG	59.971	GATTTGTGTTGCAGGCGTGT	59.969	163
TRINITY_Contig4085	1	p3	(CGG)4	12	177	188	CCCCGTGGCACTCCTAAAAT	60.034	CACAGTCATGCCAGGGTGAT	60.035	123
TRINITY_Contig4086	1	p3	(GCC)4	12	284	295	GACTACGGCTACCATGTCCG	59.97	CAAGAAGGGAGGGCTGAGC	60.078	211
TRINITY_Contig4086	2	p3	(CCG)4	12	414	425	GACTACGGCTACCATGTCCG	59.97	CAAGAAGGGAGGGCTGAGC	60.078	211
TRINITY_Contig4089	1	p4	(AATA)5	20	3546	3565	ATATGCCTTCGCCAGGTACG	59.966	TGTGCTCACCTGCAAAATGC	59.968	201
TRINITY_Contig4092	1	p3	(GCG)7	21	11	31	CATCTCTTCCTCGTCCTCGC	59.97	CGACGCCATTGATTGAGCAG	59.973	209
TRINITY_Contig4092	2	p3	(GAG)4	12	284	295	CATCTCTTCCTCGTCCTCGC	59.97	CGACGCCATTGATTGAGCAG	59.973	209
TRINITY_Contig4093	1	p3	(GTC)4	12	691	702	CCACTCGGGCTCAAAGTTCT	59.965	GTGTGGATCAAGAAGGCCGA	60.036	240
TRINITY_Contig4093	2	p3	(TCG)4	12	818	829	CCACTCGGGCTCAAAGTTCT	59.965	GTGTGGATCAAGAAGGCCGA	60.036	240
TRINITY_Contig4094	1	p3	(CGC)4	12	508	519	AGGGGCTCAAGTTCATCAGC	60.035	TTTGGTCCCAGAACTCGAGC	59.965	231
TRINITY_Contig4094	2	p3	(CGA)4	12	665	676	AGGGGCTCAAGTTCATCAGC	60.035	TTTGGTCCCAGAACTCGAGC	59.965	231
TRINITY_Contig4094	3	p4	(GGTT)3	12	3779	3790	AGGGGCTCAAGTTCATCAGC	60.035	TTTGGTCCCAGAACTCGAGC	59.965	231
TRINITY_Contig4099	1	p3	(CGC)4	12	128	139	TGGCGTCCATCATCAAGACC	60.108	CATCCTCTCCTTCGCGGTC	59.933	200

TRINITY_Contig4101	1	p4	(AAAC)3	12	49	60	ACCGCCAGTAGGACCCTATT	60.031	GAGCTGAGGAAGATACCGGC	59.967	193
TRINITY_Contig4103	1	p3	(CCG)4	12	128	139	CGATGACACCGAGGCTACTC	59.97	TAACCTCCAGTGACGGCAAC	59.966	122
TRINITY_Contig4104	1	p3	(CAC)5	15	389	403	CATCGCAAAGAAGGAAGCCG	59.902	CCCTGCGTCAAGTCTCACTT	59.966	117
TRINITY_Contig4104	2	p6	(CCGCAG)3	18	629	646	CATCGCAAAGAAGGAAGCCG	59.902	CCCTGCGTCAAGTCTCACTT	59.966	117
TRINITY_Contig4104	3	p3	(CGC)4	12	1330	1341	CATCGCAAAGAAGGAAGCCG	59.902	CCCTGCGTCAAGTCTCACTT	59.966	117
TRINITY_Contig4105	1	p3	(AGC)5	15	3922	3936	ACGCTCCACACCAGTAACAG	59.966	TGCCATTGAGGAAGCTGAGG	60.035	214
TRINITY_Contig4106	1	p3	(GGA)5	15	1229	1243	GCAGCATCGTCGCATTGATT	59.971	AGGGAATGCCAGTGAAGGTG	59.961	115
TRINITY_Contig4111	1	p3	(CGG)4	12	533	544	ATCAAATAGAGCCCGAGCCG	59.966	TCAACCTCCTCCTCCAACGA	60.179	215
TRINITY_Contig4112	1	p3	(CGG)4	12	753	764	CGGTTCTTGGCTTGGTGTTG	59.969	GTCTTGTTGGTGTTGTCGGC	59.971	226
TRINITY_Contig4115	1	p3	(TCC)6	18	42	59	ATTGTCACCACCATCCCCAC	59.96	AGCGAACACCAAGAGGACAG	59.966	252
TRINITY_Contig4116	1	p3	(TAT)6	18	5051	5068	CGGACCACGGGACTCATATG	59.968	ATGAAGGGCGTGATGAAGCA	60.035	224
TRINITY_Contig4120	1	p3	(CTC)4	12	120	131	ACTCACAAGTCAGCGGTCAG	59.967	TGGACTGGTACGTGCTGTTC	59.967	223
TRINITY_Contig4120	2	p3	(CGA)4	12	488	499	ACTCACAAGTCAGCGGTCAG	59.967	TGGACTGGTACGTGCTGTTC	59.967	223
TRINITY_Contig4120	3	p3	(CGC)4	12	856	867	ACTCACAAGTCAGCGGTCAG	59.967	TGGACTGGTACGTGCTGTTC	59.967	223
TRINITY_Contig4121	1	p3	(CCG)4	12	2292	2303	GGAAATTCGAAATCCCGGCG	59.971	CTTCCTTTCCCAGTTCCCCC	59.96	129
TRINITY_Contig4121	2	p2	(GT)7	14	2672	2685	GGAAATTCGAAATCCCGGCG	59.971	CTTCCTTTCCCAGTTCCCCC	59.96	129
TRINITY_Contig4122	1	p3	(CCT)4	12	299	310	GTGGCTGTCTTGCTCATCCT	60.036	CGTAAGCATCCCCCATCCTC	59.965	272
TRINITY_Contig4128	1	p3	(CCT)4	12	2440	2451	CGCGATACCGACAGTCATCA	59.971	TGGAATGGGGTCGGATCTCT	60.031	265
TRINITY_Contig4128	2	p5	(GAGCA)3	15	2792	2806	CGCGATACCGACAGTCATCA	59.971	TGGAATGGGGTCGGATCTCT	60.031	265
TRINITY_Contig4130	1	p3	(GCG)4	12	348	359	CTAATGGCGGAGGAGTCGAC	59.969	AATCAGTGCATCAGGCTGCT	60.034	236
TRINITY_Contig4132	1	p3	(CGA)5	15	452	466	CCCATGGATCCTTTGGTGCT	60.033	AGCTCTCCGGTCTTGTGTTG	59.966	201
TRINITY_Contig4137	1	c	(GTCG)3gagtgcctagcgctcggccgccgg<br>tccgccgtctgccgccagccgccgaccaccgtc<br>cgctgccgtcagagctgaaacctagggttcgg<br>ctcggaggggg(GA)8	128	1030	1157	CAAACACCAAAGCCGTCCAG	59.969	TGACCATAACTCGGCACCAC	60.037	187
TRINITY_Contig4144	1	p3	(CGG)5	15	830	844	CTCCAGTGGCACAAGACAGT	59.893	AGGAAGGCCCAGGAGTACAT	59.955	202
TRINITY_Contig4145	1	p5	(CCCTT)3	15	115	129	ATGGCGTCATGTGACCCAAT	60.034	ATTGCCTAACACTGGAGCCC	60.034	180
TRINITY_Contig4145	2	p2	(AC)6	12	657	668	ATGGCGTCATGTGACCCAAT	60.034	ATTGCCTAACACTGGAGCCC	60.034	180
TRINITY_Contig4145	3	p4	(ATCC)4	16	957	972	ATGGCGTCATGTGACCCAAT	60.034	ATTGCCTAACACTGGAGCCC	60.034	180
TRINITY_Contig4148	1	p3	(TCG)4	12	423	434	CGGAGTCGGTCATGGAGATG	59.969	ATCGAGTACGGCACTGCAAA	60.038	226
TRINITY_Contig4148	2	p6	(GACGGC)3	18	680	697	CGGAGTCGGTCATGGAGATG	59.969	ATCGAGTACGGCACTGCAAA	60.038	226
TRINITY_Contig4150	1	p3	(TCG)6	18	1230	1247	CCTGTGTGTACGGCATGAGT	60.037	GATCGACTCGAACTCGTCCC	59.972	194
TRINITY_Contig4152	1	c	(GCG)4ggttcgtcgcggatctcatgctcctg<br>gcgcgacgcctccatgga(ATGG)3	68	570	637	GCCGAGGGTGAACAGAAAGA	59.965	CTTCTGGGGATGCTTGGGAG	60.107	280
TRINITY_Contig4153	1	p3	(CTT)5	15	109	123	GCACTATCTCGTCCTCGGTG	59.97	CAAGGGACCATGGAAGGACC	60.034	125
TRINITY_Contig4153	2	p3	(GCT)4	12	369	380	GCACTATCTCGTCCTCGGTG	59.97	CAAGGGACCATGGAAGGACC	60.034	125
TRINITY_Contig4158	1	p5	(GAAGA)3	15	5	19	TGCCACAAAATCGTGCAAGG	59.968	CACAGCTCTCCTCAATGGCA	60.036	106
TRINITY_Contig4167	1	p3	(GCC)5	15	20	34	GAGATCTGCAGGAGGGTGGT	60.98	CGAGCAGATGGAGGTCATGG	60.25	103
TRINITY_Contig4167	2	p3	(CAG)4	12	340	351	GAGATCTGCAGGAGGGTGGT	60.98	CGAGCAGATGGAGGTCATGG	60.25	103
TRINITY_Contig4169	1	p4	(GGAA)3	12	1717	1728	ACCTGCTTCTCAAGGGCATC	60.035	GCCGTGGCGATGTCAATAAC	59.972	235
TRINITY_Contig4170	1	p3	(TCG)5	15	2430	2444	GTCCTCGAACAGCTTGTCGA	60.039	CATGTCCACCAAAGACCCGA	59.964	211
TRINITY_Contig4170	2	p3	(CGC)4	12	2774	2785	GTCCTCGAACAGCTTGTCGA	60.039	CATGTCCACCAAAGACCCGA	59.964	211
TRINITY_Contig4170	3	c	(CGA)6gcgtctcaacgacgaggccgaagaa<br>aggacgaagggaggaggacgaagaagcaag<br>agagggcttcg(TGC)4	96	2942	3037	GTCCTCGAACAGCTTGTCGA	60.039	CATGTCCACCAAAGACCCGA	59.964	211
TRINITY_Contig4173	1	c	(CGCT)3tgcgcgcgccgggaaggcagtggg<br>tgcctggtcggttctcgcccgcgagggtggtgg<br>(TGC)4	81	468	548	CGATGTTCTCGGTCTCCTCG	59.972	CTCGGAACTGATCGATGGGG	59.967	171
TRINITY_Contig4173	2	p4	(TCGC)3	12	1556	1567	CGATGTTCTCGGTCTCCTCG	59.972	CTCGGAACTGATCGATGGGG	59.967	171
TRINITY_Contig4173	3	p4	(CCGG)4	16	2901	2916	CGATGTTCTCGGTCTCCTCG	59.972	CTCGGAACTGATCGATGGGG	59.967	171
TRINITY_Contig4175	1	p4	(TTGG)3	12	1048	1059	AGATCCAGATCTCCCACCCC	60.105	ACAAGGCAAGGAAGGGATCG	60.035	242
TRINITY_Contig4176	1	p2	(GC)6	12	153	164	CCTTGGTGGTGCTCATCGT	60.003	TAGTCGTTCAGGCAGATGGC	59.824	232
TRINITY_Contig4177	1	p3	(GGT)6	18	2547	2564	GCTGTTCGTCTCCAACCTCA	59.966	CCGCTGAAGAGGTTGAGGTT	59.965	123
TRINITY_Contig4182	1	p3	(GCG)4	12	65	76	TAAGCATGCCTGTGTCGTGT	59.965	CAAATCCAACAGCACGCACA	59.969	217
TRINITY_Contig4183	1	p3	(CGC)4	12	3262	3273	GCCTCAACGCACTACCTTCT	60.037	CGTCGAGATGGCAATGCAAG	59.973	158
TRINITY_Contig4185	1	p3	(GCC)4	12	393	404	TGGGACGACGTGAATCTTGG	60.038	GTCCATCTTCCACCAGCTGG	60.394	208
TRINITY_Contig4186	1	p3	(GCG)4	12	410	421	CTCCCCAGTCTCTCTCTCCC	60.106	TCTTGCGCTGCAGAAAGAGA	59.966	193
TRINITY_Contig4186	2	p4	(CCCT)3	12	601	612	CTCCCCAGTCTCTCTCTCCC	60.106	TCTTGCGCTGCAGAAAGAGA	59.966	193
TRINITY_Contig4189	1	c*	(CCG)3(CCGGCCGGCCGGCCGCCCT<br>CTTGCCTGCCCGGCTCGCTCCGATC<br>CTTTCTCCAGAAATCCTCGCAACTG<br>CGATGCCGTAAGCGGCAAGGCCGC<br>GGTGCACGCGCGGTTGGGTACCAC<br>CGCTAGCTTGCCGTCCGGAGCCGG<br>GACGGAGCCAACGCGAGCAAGGA<br>AGCGCGGGTGCAGGCGGAACGGA<br>AGAGCGCGAGGGATCTTCACGGAG<br>GCGCCGTGTCTGCTGCCTCGTTGAG<br>CGAGAGGCAAGGATGCCGGGAGC<br>GGATTCCGGCGGGGCCTCTGTGCT<br>GTGCAGGTGGAAAGGTGGGCGAG<br>CGCAAA<GCCG>)(GCCG)3	24	196	219	GGCCCGTATTAGCCCATCTC	60.036	GGAGAAAGGATCGGAGCGAG	59.969	151
TRINITY_Contig4190	1	p2	(GA)6	12	115	126	CGGTGACATGGACCAGAACA	59.965	ATCAATACCACGGGCAAGCA	60.035	103
TRINITY_Contig4190	2	p3	(CGC)4	12	274	285	CGGTGACATGGACCAGAACA	59.965	ATCAATACCACGGGCAAGCA	60.035	103
TRINITY_Contig4195	1	c	(GCC)4gct(GCC)5	30	130	159	GCAGTACCAGCTTCCACAGT	59.965	GGTGGATCAGTCAGTGCACA	59.965	259
TRINITY_Contig4197	1	p3	(CGA)4	12	496	507	AAGAAGCAGTCGCCCATTGA	59.963	CAAAGTGCTCGCCGCTAATC	59.973	229
TRINITY_Contig4208	1	p4	(GGAG)3	12	2954	2965	CGTCTCGTCAAGTGGGTCAA	59.968	AAAGGAGCGCGTTTTTGGAC	59.97	106
TRINITY_Contig4209	1	c	(GCG)4gctggtgctggtagtgttggtggtgg<br>(TGCGGC)3	56	1819	1874	GAGAACTCGAGTCTACGGCG	59.973	CGCATAGGAACTCCTCGCAT	59.967	129
TRINITY_Contig4212	1	p5	(CGACG)3	15	327	341	ATTTTCGGTCTCGGTGCTGT	59.965	TGAACACATTCGAAGGCGGA	59.966	260
TRINITY_Contig4219	1	p4	(TTTA)3	12	974	985	GTAAATGCCAGCATGCCGAG	59.97	GATGTCGGTAATAGGCGGCA	59.967	126
TRINITY_Contig4230	1	p3	(CCG)4	12	48	59	TGTCCCTCGACGTTCTTTGG	59.967	TGATCATCCTCAGCCTGGGA	60.031	246
TRINITY_Contig4232	1	p3	(TTC)4	12	158	169	TCAGTTCCAGCGTCCTTGTC	59.966	TGGGATTTGTGGACCTGTCG	59.964	159
TRINITY_Contig4239	1	p3	(GGC)4	12	261	272	CCAGATTGGCCAGCTCAAGA	60.035	GGTGAGTGAGGTCAGGTTGG	59.965	172
TRINITY_Contig4247	1	p3	(GGC)4	12	548	559	CGCTCAAAACATGCACACCA	59.969	GGCATGAGTTTGCAAAGCCA	59.966	238
TRINITY_Contig4247	2	c	(AACA)4gttctctcccgatggccaaatctttg<br>gcgctctacagaacaaacacttttctcccgaag<br>accaaag(CT)6	94	1546	1639	CGCTCAAAACATGCACACCA	59.969	GGCATGAGTTTGCAAAGCCA	59.966	238
TRINITY_Contig4247	3	p6	(ACTTCC)3	18	1875	1892	CGCTCAAAACATGCACACCA	59.969	GGCATGAGTTTGCAAAGCCA	59.966	238
TRINITY_Contig4247	4	p3	(CGC)6	18	2818	2835	CGCTCAAAACATGCACACCA	59.969	GGCATGAGTTTGCAAAGCCA	59.966	238
TRINITY_Contig4248	1	p3	(CTG)5	15	98	112	AACTTCGCCACCGGATTCTT	59.964	GGATGGCGAGGACGTAGAAG	59.969	277
TRINITY_Contig4249	1	p4	(CAAG)3	12	1021	1032	CGTGCTGGGAAATCTCACCT	60.036	GTCAAGCTTTTGTGCAGCGA	59.971	128
TRINITY_Contig4253	1	p2	(GA)7	14	111	124	CTAATCTAGCGGCGGGGAAG	60.038	GGTCCGCTCTTCAACTCCAA	59.965	119
TRINITY_Contig4253	2	p5	(AGGTG)4	20	384	403	CTAATCTAGCGGCGGGGAAG	60.038	GGTCCGCTCTTCAACTCCAA	59.965	119
TRINITY_Contig4264	1	c*	(GCA)3(<GCA>GCC)(GCAGCC)2	27	1593	1619	CCCGATATAGCAGGCCCAAG	60.036	TGGTCCATCAACATGCCCTC	60.034	233
TRINITY_Contig4266	1	p3	(GCC)5	15	1094	1108	AATGACGTTTTCAGCGTGGC	60.04	TGAAAGGTACAGCAGCCAGG	59.963	225
TRINITY_Contig4267	1	p3	(TGC)4	12	1174	1185	ATTGAACAGTGAGACGGCGT	59.966	CGGATATCAGTCTGCAGCGT	59.969	258
TRINITY_Contig4271	1	p3	(TGC)6	18	1029	1046	GTGACCTGGAACACTGGAGG	59.965	GAAGCTGCTGCAGGGAAAAC	60.039	221
TRINITY_Contig4272	1	p5	(TTGCA)4	20	18	37	GGATGGGCCGAGATAATGGG	60.035	TGTGCATGCATGTCTCGACT	60.037	176
TRINITY_Contig4277	1	p4	(GCCG)3	12	1362	1373	TGGTCAATGGCCACATCCTC	60.034	GTTCTCCCGCCTCATACTCG	59.969	270
TRINITY_Contig4280	1	p3	(CAG)4	12	101	112	CCACACCTGAACGAAGACGA	59.968	AGCATCTCCGAGTATTGCGG	59.967	105
TRINITY_Contig4282	1	p3	(TGC)4	12	678	689	CTTGAGCCTCGTGTTCCAGT	59.966	GAACTACCTCCGGCCAAACA	59.964	166
TRINITY_Contig4283	1	c	(GCA)4cgtgcgcgccgcggcgggtgtgtctc<br>tcctcgttggaggcggcagc(GGA)4	70	1810	1879	GTCTCTCGTCAGCAAGCCTT	60.038	TTCACACACTGCGGATGGAA	59.892	168
TRINITY_Contig4291	1	p3	(GCG)4	12	1308	1319	CAACCAGAACCCGACCATGA	59.964	AGGCTAATCAACCCAGCACC	60.034	105
TRINITY_Contig4296	1	c	(CCT)5ctccggttgat(CCG)4tcaacaaca<br>accacctccgcgcctcca(CCG)9	92	270	361	CCTCCACATACGGCATCCTC	59.966	GATGGGGATGGGGATTCGTC	59.963	159
TRINITY_Contig4300	1	p4	(GCCG)3	12	706	717	TCGTAAAATCGTGGGGTGGG	60.036	TCTTGATCGCTTGTCCACCC	60.036	231
TRINITY_Contig4301	1	p4	(CCGC)3	12	1680	1691	GGGAAAACCAAAACCTCGCC	59.967	GAGGCTGTTGGTCTTCACGA	59.966	122
TRINITY_Contig4302	1	p3	(CCT)4	12	281	292	AAAGATTCGGCCAGAGTGCA	59.963	GCGGTTGTGTCCCTTTTCAC	59.97	147
TRINITY_Contig4320	1	p5	(GAATC)3	15	1	15	CACGCGAGGAGTGGTTAACT	60.039	ATTAGTGGAGTCCAACCGCC	59.748	131
TRINITY_Contig4320	2	c	(AGC)4gggagtggg(AGC)4	33	926	958	CACGCGAGGAGTGGTTAACT	60.039	ATTAGTGGAGTCCAACCGCC	59.748	131
TRINITY_Contig4326	1	p3	(TGC)4	12	14	25	GCAAAGGGCATGCTTGTCAA	59.966	TCGTCAAAAGTCGAGCCGAA	59.969	134
TRINITY_Contig4328	1	p3	(GCC)8	24	862	885	CTGGACCTAGGCTGAGACCT	60.033	GCATGGGTTGTCAACGTCAC	60.04	249

TRINITY_Contig4329	1	c	(TTTC)3ctttgtccttgttttttctactatatatgtaaatatttatctcagcagagc(AGAGG)3	78	788	865	TTTTCGTCGCATTGGGAGGA	59.964	GTGATAGCATCCACCTCGGG	59.966	218
TRINITY_Contig4330	1	c	(GCG)5cggctgcgtcctccctctccgaaactttccaacttttattattggagtggagttggtacctgctggc(TGGA)3	96	931	1026	TTAGGCCACCAGAGTGCTTG	59.963	GCATCCTTAAGGCCCATGGT	60.106	239
TRINITY_Contig4331	1	c	(GGGA)3cggcggcgcgcgt(GGC)6	44	228	271	GCTTTTGGCAAGGATCACGG	60.109	TCACCAAGCCCCAACCAAAT	60.105	157
TRINITY_Contig4335	1	p6	(GGCGTC)3	18	622	639	AGGTCATTGTGATTGGCGGT	59.962	GAGCTCACGAGTCCGATCTG	59.971	157
TRINITY_Contig4341	1	p3	(CGC)4	12	792	803	AGTTGATGGATGCATGCCCA	60.033	AGTCGTGGTCAGACAAGCTG	59.967	153
TRINITY_Contig4341	2	p3	(CCG)4	12	3642	3653	AGTTGATGGATGCATGCCCA	60.033	AGTCGTGGTCAGACAAGCTG	59.967	153
TRINITY_Contig4341	3	p5	(TGAGC)3	15	3914	3928	AGTTGATGGATGCATGCCCA	60.033	AGTCGTGGTCAGACAAGCTG	59.967	153
TRINITY_Contig4342	1	p3	(GCG)4	12	422	433	CAATGAGATGAAGCGCCGTG	59.973	ATCGCATCCTTGGAAAGCCA	60.034	165
TRINITY_Contig4345	1	p3	(CGC)4	12	1000	1011	GGCGAATTCCAGATCGCAAC	59.972	TGTTCTGGGATGTCCATGGC	60.034	271
TRINITY_Contig4346	1	p4	(CGGC)3	12	281	292	TGGGCATTGGACTGATGGAC	60.034	GCAACAACATCACTGCGGTT	59.969	187
TRINITY_Contig4350	1	c	(CCT)4cgtccccacctccctctcgctgtcaccgcctccctctcctcaccacgcacctccctctcg(CTTGC)3	87	932	1018	AGGAGGGCTGAGCTGATACA	60.032	AGGAGGGCTGAGCTGATACA	60.032	258
TRINITY_Contig4350	2	c	(TC)6ttttttttttattttttttgtccccctc(CCCT)3	51	1998	2048	AGGAGGGCTGAGCTGATACA	60.032	AGGAGGGCTGAGCTGATACA	60.032	258
TRINITY_Contig4350	3	p3	(CGG)4	12	2799	2810	AGGAGGGCTGAGCTGATACA	60.032	AGGAGGGCTGAGCTGATACA	60.032	258
TRINITY_Contig4353	1	p3	(GCC)4	12	4234	4245	TCGTCCTCCTCCTCGTCAAT	60.034	CTTCCTGAACACCCGAAGCT	59.965	140
TRINITY_Contig4354	1	p3	(CAA)4	12	631	642	CCACTCCTCCACAATGGTCC	60.035	CATCTCAGCCGCGATCTTCT	59.968	232
TRINITY_Contig4355	1	p4	(GCGA)3	12	70	81	CGAGCGAGAAAGCCAGAGAT	59.898	CCCTCGCGGTACAACTTGAG	60.736	213
TRINITY_Contig4359	1	p6	(TCTGCG)3	18	983	1000	GGCTTCTTGGTGTGCATGTG	60.039	GTTCACGACGTACATGCTGC	59.907	144
TRINITY_Contig4362	1	p4	(TTTC)3	12	1056	1067	CACCAGAAAGTTTGGGCTGC	59.967	GGACTGGATGATCTCTGGCG	59.967	189
TRINITY_Contig4366	1	p3	(CTC)4	12	1360	1371	CCACCACCTGGATCAGCAAT	60.034	GATTTCAGTGGCAGGGGTGA	59.962	137
TRINITY_Contig4367	1	c	(GCG)4tgcaacctgcgcaacacgacgggccagacgacgtgcgacctcggcggtgtgaactacaccaccgtgtgctcgtacaacaacgtgattgcgccgggc(ACG)4	120	469	588	GGGGTCCAACATCCAGAAGG	60.034	CGTTGTTGTACGAGCACACG	60.112	119
TRINITY_Contig4368	1	c	(GGC)4(AGC)5	27	113	139	CTGGCGGTGTGTAGAGATCC	59.897	GGAAGCCGGAGATGATGGAG	59.965	100
TRINITY_Contig4371	1	p3	(GCC)5	15	102	116	ATGGCGCTCAATTCCCTCAA	60.034	TGAAGGAACGCAAGACTCCC	59.965	148
TRINITY_Contig4373	1	p3	(CGG)4	12	17	28	GAGGAGGAAGGGAGGAAGGT	59.957	CCCTCTCGCAAAAGGCAGTA	60.036	184
TRINITY_Contig4380	1	c	(GGCGTC)4ca(GCG)4	38	837	874	GCCGTCAGTGTCTTTCCTCA	59.966	GATCGTGTACAATGGCACGC	59.974	268
TRINITY_Contig4380	2	c	(GCC)4gctggacgccgaggcggaggccggcgcggacggcaaggacgccggccgcgggagcgggagcccgaccgcggcctccg(GCT)6	107	6104	6210	GCCGTCAGTGTCTTTCCTCA	59.966	GATCGTGTACAATGGCACGC	59.974	268
TRINITY_Contig4380	3	c	(AG)7cgcgcagcgcgctgtgtgtgt(AG)9	53	6402	6454	GCCGTCAGTGTCTTTCCTCA	59.966	GATCGTGTACAATGGCACGC	59.974	268
TRINITY_Contig4385	1	p3	(CGG)4	12	660	671	GGACAAATCAGTCGCCGGTA	60.109	ACGGGCACGGGTATCATTTT	60.035	249
TRINITY_Contig4391	1	p4	(TTTC)3	12	636	647	GCCTGTCGAAACCCCAGTAA	59.964	GGCCGACATGCTGCAATAAG	59.97	181
TRINITY_Contig4394	1	p6	(GCAGGC)4	24	62	85	AGGTTTCGAAATCCCGGGTC	60.036	CCGCTCGTCATCATCCATCA	59.967	237
TRINITY_Contig4394	2	p4	(CGGC)4	16	255	270	AGGTTTCGAAATCCCGGGTC	60.036	CCGCTCGTCATCATCCATCA	59.967	237
TRINITY_Contig4394	3	c	(CG)8cagcgcaggggccggggatcgctccggtgatggatgatgacgag(CGGCGA)3cgacacccgagtccgaccgctggtgcgggtggctggcatgggggcggcgcaacgaccgggtcgtcgc(CG)6	158	687	844	AGGTTTCGAAATCCCGGGTC	60.036	CCGCTCGTCATCATCCATCA	59.967	237
TRINITY_Contig4394	4	p5	(CGAGG)3	15	1283	1297	AGGTTTCGAAATCCCGGGTC	60.036	CCGCTCGTCATCATCCATCA	59.967	237
TRINITY_Contig4395	1	p4	(AGCT)3	12	161	172	CTTGGCGCAGGAAAATAGGC	59.899	CAGTGGATCATCTCGGGCTC	59.967	112
TRINITY_Contig4395	2	p3	(ATG)4	12	1021	1032	CTTGGCGCAGGAAAATAGGC	59.899	CAGTGGATCATCTCGGGCTC	59.967	112
TRINITY_Contig4397	1	p3	(CCG)5	15	751	765	CGGCGACTGATCAATCGGTA	59.97	CTTGCCCATCCTTACTGCCA	60.034	149
TRINITY_Contig4397	2	p3	(TGG)4	12	1574	1585	CGGCGACTGATCAATCGGTA	59.97	CTTGCCCATCCTTACTGCCA	60.034	149
TRINITY_Contig4400	1	p2	(GA)7	14	23	36	CGGCTCATGAGGATGTACCC	59.966	GATCGACGCGTCTAGCTTCA	59.972	192
TRINITY_Contig4408	1	p3	(AAG)4	12	350	361	AGAACGCCATTGATGAGGCA	60.035	GTGCCATCTCTGCCTGAAGT	60.036	270
TRINITY_Contig4408	2	p3	(GTT)4	12	1072	1083	AGAACGCCATTGATGAGGCA	60.035	GTGCCATCTCTGCCTGAAGT	60.036	270
TRINITY_Contig4409	1	p3	(CGA)4	12	1884	1895	AGTTACTGGTAAGCGCCACC	60.037	CGATCTTCCAGCGTAGGCAT	59.967	146
TRINITY_Contig4412	1	p4	(GGAG)3	12	22	33	ATGCAGCGAATCTAGTCCGG	59.967	CAACCCAAGATCCTCGCTGT	60.036	121
TRINITY_Contig4412	2	p5	(AAAAC)3	15	2139	2153	ATGCAGCGAATCTAGTCCGG	59.967	CAACCCAAGATCCTCGCTGT	60.036	121
TRINITY_Contig4414	1	p3	(GGC)5	15	3178	3192	GTAGGCACGGATGAGGTAGC	59.968	GGGACGTATACTTCCGCCAG	59.968	182
TRINITY_Contig4420	1	p3	(GCC)5	15	23	37	ATGGTGAGCATCTGCAGCTT	60.034	AGTCAATCCCTTTGCCTCCG	60.035	263
TRINITY_Contig4422	1	c	(TCGACG)3gccgcgcgctcgt(CGC)5	46	390	435	GATCCCCAGGATGAAGAGCG	59.965	GTCCTCTCGCAGATCAGCTC	59.969	120
TRINITY_Contig4428	1	p3	(GCG)5	15	210	224	AATGGCCATGACCACTCCAG	60.034	TGGTGATGAAACATCGGCGA	60.037	174
TRINITY_Contig4432	1	p2	(CT)10	20	397	416	CGACGACGATGAAAATGGCC	59.974	GCTACCCCAAGATCTCGCTC	59.967	258
TRINITY_Contig4437	1	p3	(CGT)4	12	69	80	TCGTCTTCTGCATCGCTCTC	59.9	CGCTCTGGAAGACGAACGTA	59.833	178
TRINITY_Contig4438	1	c	(CTGGCT)3ctcggcctccgaggaggaggacgtgacgcagctgtcggcggcgcggaggcgcggaggggacggcgagggc(GAG)4	100	757	856	ACACCACCACCAATCATCCC	59.96	AGCTCACCAGAGATCGACCT	60.034	124
TRINITY_Contig4438	2	c	(GAGC)4agg(GAC)5ttactgtcggcgccgccgagctgggccacggcgagcacgaagcggtcgtgcagctggcgcgtccacctgagccgcgccctggcctcgcggcc(CTG)6	144	1545	1688	ACACCACCACCAATCATCCC	59.96	AGCTCACCAGAGATCGACCT	60.034	124
TRINITY_Contig4444	1	p3	(CTC)4	12	168	179	GCGAACCCGTCTCACAACTA	60.039	ATTCACCGCCATTACTCCGG	60.179	169
TRINITY_Contig4447	1	p5	(GCGCC)3	15	303	317	TGGACACCCATTCCTCAAGC	59.962	CTGTAATGTGCAGCGATGCC	59.971	179
TRINITY_Contig4458	1	p3	(TTG)4	12	75	86	TGGCATGCTTGTTGTTGTTGT	59.793	TGCTAGTATAGGATGGGAGGAT	57.025	160
TRINITY_Contig4459	1	p3	(CTG)4	12	1531	1542	TATGGCCCCAACATCCCAAC	60.033	GCTGAAGCTCCCCATTGTCT	60.035	131
TRINITY_Contig4465	1	p5	(ACTGG)3	15	745	759	GAGTAGGCGCTAGCGGAAAT	59.968	AGTCGTGAAGTCACCGCATT	59.966	270
TRINITY_Contig4468	1	p4	(ACCT)3	12	285	296	TGCTTACTGCTGGGTGTGAG	59.965	CCGCATCCCACCACATAGTT	60.107	132
TRINITY_Contig4468	2	p3	(GCT)4	12	400	411	TGCTTACTGCTGGGTGTGAG	59.965	CCGCATCCCACCACATAGTT	60.107	132
TRINITY_Contig4468	3	p3	(CGG)5	15	814	828	TGCTTACTGCTGGGTGTGAG	59.965	CCGCATCCCACCACATAGTT	60.107	132
TRINITY_Contig4473	1	p3	(CGC)4	12	648	659	CGAGCCGTCATCATCCATGA	59.967	CTGGTTGGCGGGTATCCTTT	60.034	243
TRINITY_Contig4475	1	p3	(GCG)5	15	1391	1405	ATCCATCCTTGAGCCCTCCT	60.029	TTGACCAAATGCCGCACAAG	59.968	183
TRINITY_Contig4480	1	p3	(GGC)4	12	2407	2418	TGCACTGCTGCCAAAATGAC	59.968	ACCGGCAAGATCAAGAAGCA	59.963	167
TRINITY_Contig4481	1	p3	(CCG)4	12	262	273	AGATAGAGTGCTGCCCAGGA	60.032	TATGGGTGCCAAGTGGTGAC	59.962	275
TRINITY_Contig4483	1	p3	(CTT)4	12	144	155	TGTGGCGATGCAGAACTTCT	59.965	GTGGAAAGCGGGTGGTTTTC	59.968	119
TRINITY_Contig4486	1	p5	(TTGAT)3	15	2386	2400	GCCTTTGCTTGAAGCTGCTT	59.967	GCCAAGGAGGACAGCTTCAT	60.035	196
TRINITY_Contig4492	1	p3	(ACA)4	12	101	112	ACGTTTACAGCTTCGGGGTT	59.893	GCCTCTCGTCCCCTTTGAAA	59.964	231
TRINITY_Contig4495	1	p5	(CGCGC)3	15	140	154	GCGTTCGCGTAAATGATGCT	59.974	GCTGCTTCCCAAGGATCACT	60.035	114
TRINITY_Contig4496	1	p4	(ACAG)3	12	319	330	CACCGGTTGACGTTCTGGTA	59.968	CGCACTTGCTGACGAAAACA	59.973	139
TRINITY_Contig4499	1	p3	(GTC)5	15	374	388	ATCACACGCCTCGATCATCC	59.967	GAGCATCTCCCGTCTGACAG	59.898	141
TRINITY_Contig4499	2	p3	(CTC)4	12	494	505	ATCACACGCCTCGATCATCC	59.967	GAGCATCTCCCGTCTGACAG	59.898	141
TRINITY_Contig4501	1	p4	(CCGG)3	12	567	578	CCTCCACTAACCACCACCAC	59.964	TCCAATCCATCTCGTGCCAC	60.108	125
TRINITY_Contig4506	1	p4	(CCTC)3	12	120	131	CTGCCCGTTCATCAGGTTCT	60.036	GAAGATGGTCATCAACGCGC	59.973	100
TRINITY_Contig4506	2	p3	(CCG)4	12	240	251	CTGCCCGTTCATCAGGTTCT	60.036	GAAGATGGTCATCAACGCGC	59.973	100
TRINITY_Contig4506	3	p3	(CGC)4	12	1888	1899	CTGCCCGTTCATCAGGTTCT	60.036	GAAGATGGTCATCAACGCGC	59.973	100
TRINITY_Contig4506	4	c	(CACC)3gcccggcgcccggcggctcgcgg(CAGTGC)3	53	3283	3335	CTGCCCGTTCATCAGGTTCT	60.036	GAAGATGGTCATCAACGCGC	59.973	100
TRINITY_Contig4510	1	p3	(TCG)5	15	438	452	TAGCTTGATCGCCGGATGAC	59.968	AGAAGAGCAAGAGCACCACG	60.32	128
TRINITY_Contig4510	2	p3	(GCA)4	12	658	669	TAGCTTGATCGCCGGATGAC	59.968	AGAAGAGCAAGAGCACCACG	60.32	128
TRINITY_Contig4510	3	c	(GCG)5gcaggggcggatcgaacccgaacaccccgagctgccccgcgcgcccccgt(CGC)4	78	1039	1116	TAGCTTGATCGCCGGATGAC	59.968	AGAAGAGCAAGAGCACCACG	60.32	128
TRINITY_Contig4518	1	p4	(GACT)3	12	789	800	GAAGGTCGAATGCGCACATC	59.973	CGTCGTCAGAGATGGAGTCG	59.973	138
TRINITY_Contig4521	1	p3	(TCT)5	15	909	923	ATCACATTGCAGATGGCCCA	60.033	AGGTCATGCCCAGTGTGATG	60.035	205
TRINITY_Contig4522	1	c	(CCTC)3ctcccgcggctgc(CT)6	37	123	159	TGATGGGCTCTCTCTCTCCC	60.106	TCCAGTACTCCACCACCGAT	59.959	222
TRINITY_Contig4523	1	p3	(TCG)4	12	1916	1927	TTCGCTCTGAATCTGCTGCA	60.037	CGCGTCTCAGCTCTATCGTT	59.972	201
TRINITY_Contig4523	2	c	(GCG)5cggctgatgctgaacgtgccgacgccgccgggcagcgcgtgctggcggagcggcttctccgcggcgggtcgcga(CGG)4aggagactgcgacgacggccgctccagcggctgcaggaagtcgtgcgtcttgaggaagaaaccttgatgagcggc(CGG)5	191	3223	3413	TTCGCTCTGAATCTGCTGCA	60.037	CGCGTCTCAGCTCTATCGTT	59.972	201
TRINITY_Contig4528	1	p3	(GTC)4	12	1817	1828	ATGAACCCTCTTTGCGTCGT	59.965	TGAGAGTAGTCCCTCCGGTG	60.034	131
TRINITY_Contig4529	1	p3	(GCC)4	12	412	423	AAGAAGTACCACGCCTGCTC	60.037	GCTCATCGACTCCTACCTGC	59.969	156

TRINITY_Contig4529	2	p5	(GCGGC)3	15	1316	1330	AAGAAGTACCACGCCTGCTC	60.037	GCTCATCGACTCCTACCTGC	59.969	156
TRINITY_Contig4530	1	p3	(ACA)4	12	362	373	CCTGGATAACCTGGTCGTGG	59.822	CAGGCTACCCTACACAGCAG	59.823	106
TRINITY_Contig4533	1	p3	(CCT)4	12	324	335	AGCACAGGTACCACAAGCTC	59.965	ACCCATCGCCATCATTGTGT	60.034	230
TRINITY_Contig4536	1	p3	(GAT)4	12	371	382	TTGTGTTGGTGACCACGACA	60.036	GGTCCTTCAAGAGCGTTGGA	59.965	117
TRINITY_Contig4536	2	p3	(GTG)4	12	714	725	TTGTGTTGGTGACCACGACA	60.036	GGTCCTTCAAGAGCGTTGGA	59.965	117
TRINITY_Contig4541	1	p3	(GCG)4	12	478	489	GTTGGTTGGCATCAAGGCTG	60.038	AACCCCACGATGGAGAACAC	59.964	123
TRINITY_Contig4542	1	p3	(AAC)4	12	331	342	AATTCGGCGGCTAACCATCA	60.107	CGCCGCCATTGTTGTTGTTA	60.04	145
TRINITY_Contig4543	1	p4	(TTTC)3	12	1096	1107	AGCTCCCCTTCTTCAACACG	59.965	GGACAGCATGGCAGAGAACT	60.036	185
TRINITY_Contig4543	2	c	(CTGTCC)3ctgtggaggcgga(TGGAT)4	51	1758	1808	AGCTCCCCTTCTTCAACACG	59.965	GGACAGCATGGCAGAGAACT	60.036	185
TRINITY_Contig4544	1	p3	(AAG)4	12	295	306	CCACTGTCGGTACCCTGAAC	60.038	ATCCAGCCAAGCAGAAGGTC	60.035	180
TRINITY_Contig4546	1	p3	(CAG)4	12	1078	1089	CTTTGCCAGTCACGTTGTGG	59.971	ATGGTATGCAGCCGGATCTG	59.964	273
TRINITY_Contig4549	1	p6	(CAAGGC)3	18	271	288	TATGATCGTCGGTGCCTTCG	59.97	ATTTTGCTGGGGTATCCGCA	60.034	147
TRINITY_Contig4553	1	p3	(GCT)4	12	179	190	GTTTCGAGGAGGAGGTGTGG	60.037	GCATATCCTTCGGCCTCACA	59.893	106
TRINITY_Contig4554	1	p4	(CGCC)3	12	551	562	GGAAGCCGAGGGATGGTATG	59.965	TTCTCCTCCCTCCTTCCTCG	60.033	181
TRINITY_Contig4557	1	p2	(AG)12	24	1181	1204	TGGGAGTGGGATGGAAGCTA	59.956	TGAGTGGCGGCAGTAAACAT	59.964	269
TRINITY_Contig4559	1	p3	(CGG)6	18	542	559	GAATTTGCGGTGGTGATGCA	59.757	CCGCCTAATCCTGGTCCATC	59.965	133
TRINITY_Contig4560	1	p3	(TGC)4	12	1079	1090	TGGACCAGCTGCTGAATCAG	60.036	CCTGCCTCCCCAATGTCATT	60.033	233
TRINITY_Contig4560	2	p3	(GCT)4	12	1767	1778	TGGACCAGCTGCTGAATCAG	60.036	CCTGCCTCCCCAATGTCATT	60.033	233
TRINITY_Contig4561	1	c	(CCG)4cagccgcagcggcg(GCC)4gcga cctccgccgcgcggggcacgctctcgagcgcc gtgat(GC)6	91	503	593	TGACACACTCACATGGCCTC	59.965	CACCGCCATCTCCTGAGATC	59.967	185
TRINITY_Contig4561	2	c	(CGC)4(AAC)5	27	1401	1427	TGACACACTCACATGGCCTC	59.965	CACCGCCATCTCCTGAGATC	59.967	185
TRINITY_Contig4561	3	p3	(CGC)4	12	1559	1570	TGACACACTCACATGGCCTC	59.965	CACCGCCATCTCCTGAGATC	59.967	185
TRINITY_Contig4561	4	p2	(TG)9	18	2256	2273	TGACACACTCACATGGCCTC	59.965	CACCGCCATCTCCTGAGATC	59.967	185
TRINITY_Contig4562	1	c	(CCG)4agcacacgacgacgaccgtgctcttc agggccgcccccgc(CGG)4	64	827	890	CTGTGGGAGGTGAAGCTCAG	60.037	GTGTCAGCTGCACGAAGAAC	59.767	187
TRINITY_Contig4563	1	p3	(AGA)4	12	60	71	CGTCCTGCATCTCGTAGTCC	59.97	CTTCTTCTTCCCCACCGTCC	60.036	278
TRINITY_Contig4563	2	p3	(CGC)4	12	826	837	CGTCCTGCATCTCGTAGTCC	59.97	CTTCTTCTTCCCCACCGTCC	60.036	278
TRINITY_Contig4567	1	p3	(GGC)4	12	1261	1272	ACACCACTACGGCAAGAAGG	59.965	CCGCCATCGTAGGTAAGTCC	59.968	197
TRINITY_Contig4576	1	p3	(GTC)4	12	960	971	ACATGGTGGGCAATCTCAGG	60.034	CTCGAGGATGATGGCAGCAT	59.965	169
TRINITY_Contig4579	1	p3	(TCT)4	12	289	300	CGGGTACAGCGTCTTGAAGT	60.039	TGGCATCAACATTGTGCAGC	60.038	109
TRINITY_Contig4581	1	p6	(CGAGGA)3	18	1022	1039	TCTTGGGCATCACGAAGACC	60.036	TCCCCCGTTCGAGTAGACAT	60.034	217
TRINITY_Contig4584	1	p4	(CCCG)3	12	168	179	TCAGATTCTGCATGCAGCCA	60.035	TGTGGGTTTCTGGTGTTGCT	60.034	236
TRINITY_Contig4588	1	p3	(GAC)5	15	225	239	TAGAACTCCTTCTCGCCCGA	60.035	CCGCTACAGTGGGGAAAGAG	60.108	217
TRINITY_Contig4589	1	p3	(GAC)4	12	361	372	ATTTTGGTCAACAACGCCGG	59.969	TGCGTCCGAGGAAATGTTGA	59.966	177
TRINITY_Contig4591	1	p6	(GGGGAG)3	18	5751	5768	TAGATTCGCCCCCTTCTGGA	60.032	GGCCACTGCACAATTGGAAG	60.038	126
TRINITY_Contig4593	1	p3	(TCG)4	12	184	195	GATAGAGAAGAGCGCGCTGT	59.97	CAGAACCTCCACGAAGCTGT	59.966	246
TRINITY_Contig4595	1	p3	(GCA)4	12	2131	2142	CCCGCGGCAAAAACCTAAAA	59.968	CCGGTCAAGCTTCCTTCCAT	60.035	273
TRINITY_Contig4599	1	p4	(GGAA)3	12	234	245	GAAGGAAGGAAGGAGGAGGC	59.456	AGTCGGTCAGTCCTCTCCTC	60.035	209
TRINITY_Contig4600	1	p3	(CGC)4	12	20	31	CAAGGGCGGTGTTTGACAAG	59.969	GAACAGTTCCAGAGCCTCCC	60.036	107
TRINITY_Contig4602	1	p4	(AAAT)3	12	780	791	CAATGTAATCAGGGCACGCG	59.972	GGTCGTTACCAGGCCAAAGA	59.964	149
TRINITY_Contig4605	1	p3	(CTT)4	12	595	606	ATTGCAGGCGATTGTGAGGA	60.035	GTCTTGCGACTTGCTATGCG	59.974	102
TRINITY_Contig4605	2	c	(TCG)4acggcgccggcgcggtggcg(GGC)4	44	2102	2145	ATTGCAGGCGATTGTGAGGA	60.035	GTCTTGCGACTTGCTATGCG	59.974	102
TRINITY_Contig4605	3	p3	(CGT)4	12	2558	2569	ATTGCAGGCGATTGTGAGGA	60.035	GTCTTGCGACTTGCTATGCG	59.974	102
TRINITY_Contig4605	4	p3	(CCG)4	12	2671	2682	ATTGCAGGCGATTGTGAGGA	60.035	GTCTTGCGACTTGCTATGCG	59.974	102
TRINITY_Contig4605	5	p2	(GC)7	14	2875	2888	ATTGCAGGCGATTGTGAGGA	60.035	GTCTTGCGACTTGCTATGCG	59.974	102
TRINITY_Contig4605	6	p4	(GGAA)3	12	3076	3087	ATTGCAGGCGATTGTGAGGA	60.035	GTCTTGCGACTTGCTATGCG	59.974	102
TRINITY_Contig4607	1	c	(GACCT)3gatggcgggg(CTC)4	37	463	499	CAAAACCCAACCAGCAGACG	59.969	ATGTCTCGCATGCATGGCTA	59.893	280
TRINITY_Contig4607	2	c	(GCG)4gacc(GCG)5	31	629	659	CAAAACCCAACCAGCAGACG	59.969	ATGTCTCGCATGCATGGCTA	59.893	280
TRINITY_Contig4607	3	p5	(CTGAG)4	20	1099	1118	CAAAACCCAACCAGCAGACG	59.969	ATGTCTCGCATGCATGGCTA	59.893	280
TRINITY_Contig4607	4	c*	(CT)18(CTTCTTCTTCTTCTTCTTCTT CTTCTTAACCCCCTCTCCCATTAATA TTAATATATCATGACAGATTACTAT TTCACGGATTTCCATTATAATTAGC GGTTGGCTTTGAGATTTATAATTAA TGGTTGGCGTTGAGATTTCTTGTGA GCACAATCA<TCT>)(TCT)8	62	1487	1548	CAAAACCCAACCAGCAGACG	59.969	ATGTCTCGCATGCATGGCTA	59.893	280
TRINITY_Contig4609	1	p3	(TGC)4	12	43	54	TGCTCGTGCTCCAGAAGAAG	60.038	GCAAAAGAGAAACAGCGCCA	59.969	137
TRINITY_Contig4612	1	p3	(GCG)4	12	347	358	ATGCGGGTAACTTTCAGGGG	60.034	CTGCCTATTTTCTTCGCCGC	59.972	242
TRINITY_Contig4614	1	p4	(TTTC)4	16	2190	2205	GTGCTCCAACGACGCTTTTT	59.971	ACTACACACAGCCTCAACCG	59.966	145
TRINITY_Contig4614	2	c	(GAGGG)3gaagcgggcgcggccgctgtgg atgga(GGCG)3	54	2574	2627	GTGCTCCAACGACGCTTTTT	59.971	ACTACACACAGCCTCAACCG	59.966	145
TRINITY_Contig4616	1	p3	(CCT)4	12	111	122	GCTTAGTAGTCCTCGTCGCC	59.971	AGAACGTGGACGAGCTGATC	59.829	199
TRINITY_Contig4621	1	p4	(GCCG)3	12	1337	1348	CATGCGGCAAGACGAATCAG	59.973	GCTCTGTCCTCCGCTTCTAC	59.898	164
TRINITY_Contig4626	1	p4	(CGCA)3	12	230	241	ATCTCCCCAGCTTCACTTGC	60.035	GCGGCAGAATAGAGCTTTGC	59.971	191
TRINITY_Contig4633	1	p3	(TTC)4	12	459	470	CCTCATACCTTCCCCCTCCA	60.03	TATGGTGGGGGCAGGTATGA	60.03	148
TRINITY_Contig4633	2	p3	(CCA)4	12	636	647	CCTCATACCTTCCCCCTCCA	60.03	TATGGTGGGGGCAGGTATGA	60.03	148
TRINITY_Contig4633	3	p3	(CCA)6	18	864	881	CCTCATACCTTCCCCCTCCA	60.03	TATGGTGGGGGCAGGTATGA	60.03	148
TRINITY_Contig4633	4	p3	(CTC)4	12	1082	1093	CCTCATACCTTCCCCCTCCA	60.03	TATGGTGGGGGCAGGTATGA	60.03	148
TRINITY_Contig4636	1	c	(CGC)4ggcaggaggcggctggtcgggacgt cctgccgtggcggttgcgcccgccagcggcaa attactccccg(TCC)4	92	465	556	CCGACCTGGACCTCATGTTC	60.108	TCCTTGCAGTAGCGGAACAG	60.037	103
TRINITY_Contig4636	2	p3	(CAC)4	12	953	964	CCGACCTGGACCTCATGTTC	60.108	TCCTTGCAGTAGCGGAACAG	60.037	103
TRINITY_Contig4637	1	p3	(CAA)4	12	317	328	TGGACCAGGATAAGGAGGGG	60.03	ATAGTATGTACGCCGCGGTG	60.04	242
TRINITY_Contig4637	2	p3	(TGG)4	12	1717	1728	TGGACCAGGATAAGGAGGGG	60.03	ATAGTATGTACGCCGCGGTG	60.04	242
TRINITY_Contig4640	1	p3	(CCA)4	12	457	468	CCGTCGTGAATGTGCGATTC	59.975	GTAGAAGGTGTCACGGGTGG	60.038	181
TRINITY_Contig4641	1	p3	(CGA)5	15	497	511	GCCTTCGCCAATTGTACGAC	59.904	TCAATACCGTGTCGTTGGCA	59.966	141
TRINITY_Contig4642	1	c	(GCACG)4gacccatcatccatccctctctcg cagtcgcaggcgcagcaggggggctcctg(T GGC)3	85	2	86	AGGAGGATTATGCCCCTGGT	60.029	GCTATGTGCTGCATTGAGCC	59.97	271
TRINITY_Contig4642	2	p3	(AGC)6	18	2229	2246	AGGAGGATTATGCCCCTGGT	60.029	GCTATGTGCTGCATTGAGCC	59.97	271
TRINITY_Contig4642	3	p4	(GGAT)3	12	2431	2442	AGGAGGATTATGCCCCTGGT	60.029	GCTATGTGCTGCATTGAGCC	59.97	271
TRINITY_Contig4642	4	p4	(ATCC)3	12	2836	2847	AGGAGGATTATGCCCCTGGT	60.029	GCTATGTGCTGCATTGAGCC	59.97	271
TRINITY_Contig4653	1	c	(GCG)4gtgcccggagtcgtcggacgaggac gccggcgagcctgac(GAG)4	64	67	130	CAGCAAGAGGAAGGTGGAGG	60.036	GGCATCCTGTAGTAGTCGCC	59.968	183
TRINITY_Contig4653	2	p3	(ACG)4	12	585	596	CAGCAAGAGGAAGGTGGAGG	60.036	GGCATCCTGTAGTAGTCGCC	59.968	183
TRINITY_Contig4660	1	c	(CGCC)3ctgctgccgctaccgctcgcgcccg gcgcgtcgtggctcctcttcagcctcttcggcg g(CCT)4	83	1419	1501	ATCAGCAACCGCAATCAGGA	60.035	GCTTGCATGTCTCCCTCAGT	60.036	146
TRINITY_Contig4661	1	p4	(CTCC)3	12	844	855	CCGGCGAATCGGTGAAATTC	59.972	TGATTGAGGAGGAGGGAGGG	60.031	222
TRINITY_Contig4665	1	p3	(TCC)4	12	191	202	GCACAGTTCTGACGACAGGA	59.968	CGACGTTTCCTGTCTCCACA	59.968	107
TRINITY_Contig4665	2	p3	(GGA)4	12	633	644	GCACAGTTCTGACGACAGGA	59.968	CGACGTTTCCTGTCTCCACA	59.968	107
TRINITY_Contig4665	3	p3	(GCC)5	15	1212	1226	GCACAGTTCTGACGACAGGA	59.968	CGACGTTTCCTGTCTCCACA	59.968	107
TRINITY_Contig4668	1	c	(AATT)3aatctgccacttgcatcgtcggt(G A)7	49	75	123	GCATCGTCGGTGAGAGAGAG	59.971	ATTGATTTCGGAGCCAGCCA	60.034	126
TRINITY_Contig4670	1	p3	(AAC)5	15	47	61	CGTCTACGACCTCATGACCG	59.972	AAAGTGGCTGGGTTGTTCCA	60.033	110
TRINITY_Contig4672	1	p3	(TCC)4	12	114	125	CCAGGTCCCAATCCAACTCC	60.034	CTCTTTTCTCTGGGGCTGGG	60.035	180
TRINITY_Contig4672	2	p4	(TTTC)3	12	237	248	CCAGGTCCCAATCCAACTCC	60.034	CTCTTTTCTCTGGGGCTGGG	60.035	180
TRINITY_Contig4672	3	p3	(CGA)5	15	655	669	CCAGGTCCCAATCCAACTCC	60.034	CTCTTTTCTCTGGGGCTGGG	60.035	180
TRINITY_Contig4676	1	p3	(GGC)4	12	115	126	AGAGGTAGCACGAGGAGGAG	60.107	GTAGGAATAGCGACTGGCCC	59.966	112
TRINITY_Contig4679	1	p3	(GCA)4	12	63	74	AGGAGACCGAGGAGCTTGAT	60.033	CTTGGCGTTTTCCACCTTGG	59.968	188
TRINITY_Contig4680	1	p4	(CCTG)3	12	353	364	TTGGCTGATCGTGTTTCCGA	59.966	CATCTCCTCCACGAATCCGG	59.967	100
TRINITY_Contig4680	2	p2	(TC)6	12	609	620	TTGGCTGATCGTGTTTCCGA	59.966	CATCTCCTCCACGAATCCGG	59.967	100
TRINITY_Contig4680	3	p2	(AG)7	14	899	912	TTGGCTGATCGTGTTTCCGA	59.966	CATCTCCTCCACGAATCCGG	59.967	100
TRINITY_Contig4680	4	p3	(AAG)6	18	2023	2040	TTGGCTGATCGTGTTTCCGA	59.966	CATCTCCTCCACGAATCCGG	59.967	100
TRINITY_Contig4684	1	p3	(TCT)4	12	195	206	CAACCACCAGGACTCAGGAC	59.965	GTGGTCCTCCTTCACGAGTG	60.038	269
TRINITY_Contig4687	1	p4	(GAAA)3	12	655	666	TGGCTTTGCAACAACCCTTG	59.822	GCCAAAGAAAGCCAAGGCAT	59.677	250
TRINITY_Contig4691	1	p3	(GAG)5	15	1135	1149	CCGAGCTACCGGTCTTCATC	59.969	CGTATGAGGACGGTGAGCTC	59.97	102
TRINITY_Contig4703	1	p3	(GCC)5	15	505	519	CTCCACGTACCACCAGTCAC	60.039	TGGAGGACCAGGATCCAGAG	60.032	180
TRINITY_Contig4709	1	c	(GCG)4gccctggaccagagcaaca(GCT) 4	43	450	492	ATGCAAAGCCTTGGCACTTG	59.966	TGCCAACAACAGCAACATGG	59.896	254
TRINITY_Contig4713	1	p3	(ATA)6	18	2444	2461	GAACGTGCCTGGAGTTGGTA	59.966	ATGGGAAGGCTGGAATGTGG	60.033	130

TRINITY_Contig4714	1	p3	(CCG)4	12	119	130	TCCCGCTCTTCTCAATCGAC	59.544	AGAAGTGGAGGAGTGCGTTG	59.966	267
TRINITY_Contig4719	1	p3	(GAA)4	12	454	465	GGTTGCAATCGATCAGACGC	59.973	CAGGTGGTCGGACATCAACA	59.965	157
TRINITY_Contig4731	1	p5	(TGTGC)3	15	65	79	CGCAGGACAAAACTGTGTGG	59.971	CCTACCCCAAGCCTTGTTGT	59.888	236
TRINITY_Contig4734	1	p3	(CGC)4	12	231	242	GGAGTCTGTGGTGGAGGGTA	60.252	CAAGGACCATCGTCACAGCT	60.037	179
TRINITY_Contig4734	2	p4	(CCGC)3	12	717	728	GGAGTCTGTGGTGGAGGGTA	60.252	CAAGGACCATCGTCACAGCT	60.037	179
TRINITY_Contig4737	1	p3	(TTC)4	12	443	454	GCATGGGCGTGAAAAGAACA	59.686	GTGCCAATCCCGCAACAAAA	60.25	120
TRINITY_Contig4745	1	p6	(GGACGA)3	18	1070	1087	GCTCTGGTACCACATGCACT	60.036	ACTGCTCAACTGTACGGTGG	59.966	139
TRINITY_Contig4747	1	p6	(GCCCCG)3	18	483	500	AAGACCCTCATCCTTGCTGC	60.035	CGATGACCTGCTTGCAGTTG	59.833	214
TRINITY_Contig4749	1	p2	(CA)11	22	215	236	CACAGGGATCCGACCGTATG	59.968	CGGCGAAAGTGCAAACTCAA	59.971	210
TRINITY_Contig4750	1	p3	(GGC)7	21	2657	2677	AAATTTGGTGTCGCCGTGTG	59.97	AGGACTGTGTGCCATGTCTG	59.964	237
TRINITY_Contig4750	2	p3	(CGC)4	12	2963	2974	AAATTTGGTGTCGCCGTGTG	59.97	AGGACTGTGTGCCATGTCTG	59.964	237
TRINITY_Contig4751	1	p3	(CGT)5	15	734	748	GGACGACCTCTACTCCACCT	60.034	GGGAACGACGACTTCTTGGT	59.967	192
TRINITY_Contig4751	2	p3	(CAG)5	15	1394	1408	GGACGACCTCTACTCCACCT	60.034	GGGAACGACGACTTCTTGGT	59.967	192
TRINITY_Contig4752	1	p2	(TC)10	20	846	865	GCGAGGTCTTCTATCCGACG	60.041	TACAACAGTACAAGCCGCGT	59.968	173
TRINITY_Contig4753	1	p3	(GAC)4	12	1036	1047	TCCAGTTGAACCTGTCGAGC	59.966	GACTTCCCCCACAAGTACGG	60.037	214
TRINITY_Contig4753	2	p4	(GGCC)3	12	1187	1198	TCCAGTTGAACCTGTCGAGC	59.966	GACTTCCCCCACAAGTACGG	60.037	214
TRINITY_Contig4755	1	p3	(CTC)4	12	439	450	TCGTTGGAGTACTTGCGCTT	59.967	AACTTCAAGGCTGTCCTCGG	59.965	254
TRINITY_Contig4762	1	p4	(TTAT)4	16	1326	1341	GTCTGGTGCACATCTCTGCT	60.037	TTTTCCAACGCCATTGCCTG	59.967	223
TRINITY_Contig4765	1	p6	(CGTCGG)3	18	86	103	TCGTGCTTTGTCGCTGTAGT	59.968	GGGACAAACGGCTTTTCGAC	60.041	121
TRINITY_Contig4773	1	p4	(TTGT)3	12	504	515	TTCGGATTCGATTAGGCGGG	59.967	GATTAGCGGGAGGAGGAAGC	59.966	204
TRINITY_Contig4773	2	p4	(TAGC)3	12	819	830	TTCGGATTCGATTAGGCGGG	59.967	GATTAGCGGGAGGAGGAAGC	59.966	204
TRINITY_Contig4774	1	p4	(GCCT)4	16	88	103	ATTTGGGCGAGCCTATCAGG	59.891	CCAACCACCACCCTCTGATC	60.035	190
TRINITY_Contig4777	1	c	(GCC)4ggggccgcacaagaggcatcgcag ggagga(CGG)4	54	244	297	GAACTACATTCGCGTTCGCC	59.975	CCAACCCCACTTGCCATTTG	59.965	230
TRINITY_Contig4779	1	p3	(CGC)4	12	1220	1231	GCAGTACTTGACGAGCCTGT	60.038	TGGTGCTCAATCCCATCACC	60.034	155
TRINITY_Contig4780	1	p3	(CCG)4	12	110	121	CGCAGTACCACATGTTTGGC	60.11	GCTGCCGCTTAAAGTCGATG	59.973	225
TRINITY_Contig4786	1	p3	(CAC)4	12	805	816	CACACGTTGTTCGCGAGTTT	59.974	GTCATCGACATGTTGAGCGC	59.974	143
TRINITY_Contig4791	1	p2	(TC)20	40	1199	1238	AAGAACTTCGAGCTCGGCAA	59.966	GCGTGGAGCTTCTTGTTGTG	60.041	221
TRINITY_Contig4802	1	p5	(CTCAG)4	20	479	498	AGAGCCCAATTTCCCTGCTC	60.034	GCCATGGACAGTTGACTCCA	59.963	268
TRINITY_Contig4802	2	c	(AGG)4agctccttcgccg(GCCCC)3	40	815	854	AGAGCCCAATTTCCCTGCTC	60.034	GCCATGGACAGTTGACTCCA	59.963	268
TRINITY_Contig4803	1	p3	(GAC)4	12	188	199	AACGAGGAAACCAGCGTGAT	59.965	TCAGCTGCCTCTTTCAGTGG	59.964	137
TRINITY_Contig4809	1	p3	(GGC)5	15	3472	3486	GAACAATCATCACGAGCCGC	59.973	GCGTTGAATTCTGCAGGCAA	60.039	251
TRINITY_Contig4826	1	p3	(CGG)6	18	476	493	CTCGACCTCGACGCGATTTA	59.973	CTGGAAGCGTCTTGGACAGT	59.966	133
TRINITY_Contig4832	1	p2	(GC)6	12	355	366	CAGGCGAAAAGAGAACCCCT	59.963	TTATGGGCTTTGTGGGAGCTG	60.034	134
TRINITY_Contig4837	1	p4	(AACC)3	12	476	487	GGAAGCATCCCCAACCGTTA	60.035	TGATGCAACTCGAAGGGCAT	60.035	119
TRINITY_Contig4837	2	p5	(CCCTC)3	15	716	730	GGAAGCATCCCCAACCGTTA	60.035	TGATGCAACTCGAAGGGCAT	60.035	119
TRINITY_Contig4838	1	p4	(CGCT)3	12	264	275	ATGATCTTCGTCAGCGCGAT	59.969	CTTTGGAGCTGTGGCACAAC	59.969	213
TRINITY_Contig4838	2	p3	(TGG)4	12	623	634	ATGATCTTCGTCAGCGCGAT	59.969	CTTTGGAGCTGTGGCACAAC	59.969	213
TRINITY_Contig4846	1	c	(GGGA)3ggaacagcaaacacacatggcat gttag(GCA)4agtgctc(GT)7	73	85	157	CAAGTGCTCGTGTGTGTGTG	59.973	GCAGATGTGGTAGGGGACAC	60.108	130
TRINITY_Contig4846	2	p4	(CGGC)4	16	452	467	CAAGTGCTCGTGTGTGTGTG	59.973	GCAGATGTGGTAGGGGACAC	60.108	130
TRINITY_Contig4850	1	p4	(ATAA)3	12	739	750	TCATCGACCATGGCTTTGCT	60.035	TGGTGTTGGAGCTGTTTGGT	60.034	259
TRINITY_Contig4850	2	c	(GCA)4gatggcggcgcagcatcctttccaga agccgtcgccgccgcggctggtggtctccg(G CT)4	80	1165	1244	TCATCGACCATGGCTTTGCT	60.035	TGGTGTTGGAGCTGTTTGGT	60.034	259
TRINITY_Contig4850	3	p3	(CGT)4	12	1567	1578	TCATCGACCATGGCTTTGCT	60.035	TGGTGTTGGAGCTGTTTGGT	60.034	259
TRINITY_Contig4850	4	c	(CTGAC)3ttgggaacaagggcccaccattg tggagccctaccaaacagctccaacaccaatc cgagaaggagaagaacagttgtttcatgag(T TATT)3	115	1733	1847	TCATCGACCATGGCTTTGCT	60.035	TGGTGTTGGAGCTGTTTGGT	60.034	259
TRINITY_Contig4852	1	p4	(AAAT)3	12	441	452	AAAATTTTCCACGCTCCCGC	60.039	AAAAGTGATGGCAAACGGGC	59.967	159
TRINITY_Contig4856	1	p5	(GAAAG)3	15	31	45	GCAGTCCCACTAGTTGGCAT	60.035	TCATGCATATCCATGTCGTCCA	59.632	102
TRINITY_Contig4857	1	p6	(GGGGCG)3	18	1373	1390	CTGATTCGCTAGTCCCCGTC	59.969	CTCCTGCGGCTGATAGGAAG	59.967	276
TRINITY_Contig4857	2	p3	(GCG)6	18	1657	1674	CTGATTCGCTAGTCCCCGTC	59.969	CTCCTGCGGCTGATAGGAAG	59.967	276
TRINITY_Contig4861	1	p4	(CGCT)4	16	502	517	CGCCCTGAATGGAAAAGTGC	60.109	TGGAGAATGCCGTGCTCAAT	60.035	213
TRINITY_Contig4862	1	p3	(GCT)5	15	286	300	TCCCGTGTGCCCCTAGATTA	60.032	GGTTTGACATTTCAGCGCGT	60.04	102
TRINITY_Contig4864	1	p3	(CGC)4	12	1896	1907	GATTGAGCCCGCGTTGATTC	59.972	CTGGCATGCTGAGTGAAGGA	60.036	148
TRINITY_Contig4865	1	p3	(CGC)7	21	16	36	GGTCTCACTTGGGGAGCTTC	60.036	AACTTGAGCGCTCACCATCA	59.965	259
TRINITY_Contig4865	2	p3	(GAT)4	12	371	382	GGTCTCACTTGGGGAGCTTC	60.036	AACTTGAGCGCTCACCATCA	59.965	259
TRINITY_Contig4866	1	p5	(AAACT)3	15	35	49	GCTACATCTCGTCGGTGAGG	59.97	TCCCCAAGAAGCAGTTCGTC	59.965	211
TRINITY_Contig4866	2	p3	(CGC)4	12	289	300	GCTACATCTCGTCGGTGAGG	59.97	TCCCCAAGAAGCAGTTCGTC	59.965	211
TRINITY_Contig4866	3	p3	(GGA)4	12	1199	1210	GCTACATCTCGTCGGTGAGG	59.97	TCCCCAAGAAGCAGTTCGTC	59.965	211
TRINITY_Contig4867	1	p3	(GAG)6	18	3161	3178	CTGCCGCTAAGCTACGATCA	59.969	CTCCACGTTCAAGCCTGAGT	59.966	140
TRINITY_Contig4871	1	p3	(CCG)5	15	360	374	GTCAGGAACCTCGTGCTCAA	59.966	CCTTGCTGCTTCGCAAACTT	59.969	143
TRINITY_Contig4881	1	p3	(AGC)4	12	744	755	ACTTGCGCGTCTGAAGAGAA	59.968	GCTTCTAACTGCATCTGCGC	59.973	271
TRINITY_Contig4881	2	p3	(GCC)4	12	944	955	ACTTGCGCGTCTGAAGAGAA	59.968	GCTTCTAACTGCATCTGCGC	59.973	271
TRINITY_Contig4881	3	p3	(AGC)4	12	3970	3981	ACTTGCGCGTCTGAAGAGAA	59.968	GCTTCTAACTGCATCTGCGC	59.973	271
TRINITY_Contig4882	1	p3	(GCC)5	15	161	175	CCCAAATCAGCCCACTGTCT	59.961	GGCAGGGTACTCTGAGCAAG	60.108	137
TRINITY_Contig4882	2	p3	(GGC)4	12	406	417	CCCAAATCAGCCCACTGTCT	59.961	GGCAGGGTACTCTGAGCAAG	60.108	137
TRINITY_Contig4883	1	p3	(TGG)4	12	921	932	CTAGGGGTCTAGGGCTGGTT	60.031	TTTCTCCTCTCCCTCGCTCA	59.96	154
TRINITY_Contig4883	2	p4	(TCCT)3	12	1053	1064	CTAGGGGTCTAGGGCTGGTT	60.031	TTTCTCCTCTCCCTCGCTCA	59.96	154
TRINITY_Contig4886	1	p3	(GGC)5	15	630	644	CACAAGGTGGAGTTCGACGA	59.968	ACGATGTTCTTGTGCCGGAT	60.036	242
TRINITY_Contig4891	1	p4	(GGGA)3	12	1571	1582	GGAGTCAACCACGGCAAAAC	59.97	CGTCCCCGGATTTCTAGTCG	59.97	143
TRINITY_Contig4891	2	p3	(CTT)4	12	2460	2471	GGAGTCAACCACGGCAAAAC	59.97	CGTCCCCGGATTTCTAGTCG	59.97	143
TRINITY_Contig4898	1	p3	(GCC)4	12	65	76	CCTCTTGCTGCTGCTTCTCT	60.036	ACAGGAATCACAGGATGCGG	60.108	129
TRINITY_Contig4899	1	p2	(GA)11	22	1626	1647	CTCACCTACCAATGCAGCCA	60.035	TGGTCTGTGTGTTGGCCTTT	60.034	116
TRINITY_Contig4900	1	p3	(CTC)4	12	261	272	GGACCCGGTATGCTTCACTC	60.179	CTCAGCCCGACCGAATCTAC	59.969	200
TRINITY_Contig4902	1	p3	(CAG)4	12	452	463	GCGAAGGCTATGGGATTCGA	59.966	GGCGAAAGTGGAGTCCAGAA	59.965	138
TRINITY_Contig4902	2	p3	(GGC)4	12	1054	1065	GCGAAGGCTATGGGATTCGA	59.966	GGCGAAAGTGGAGTCCAGAA	59.965	138
TRINITY_Contig4902	3	p6	(TCTTCC)3	18	1855	1872	GCGAAGGCTATGGGATTCGA	59.966	GGCGAAAGTGGAGTCCAGAA	59.965	138
TRINITY_Contig4903	1	p3	(GAG)5	15	4073	4087	GGAGGGTGGACGACAAACAT	59.964	TTGTTGCTTGGTGAATGGCG	59.968	113
TRINITY_Contig4903	2	p3	(GGA)4	12	4200	4211	GGAGGGTGGACGACAAACAT	59.964	TTGTTGCTTGGTGAATGGCG	59.968	113
TRINITY_Contig4904	1	p3	(CCG)4	12	55	66	TGGTATTTTGCCGCTCACCT	59.962	GTGAGGCCCTCATCTTGCTT	60.035	163
TRINITY_Contig4905	1	p4	(ATCG)3	12	1178	1189	CCTCCTAGACCTGGACGACA	60.034	GGTAAGGGACACAGGGAACG	60.037	195
TRINITY_Contig4912	1	p4	(TCTT)3	12	384	395	CCAACCGAGCACCACTAAGT	59.965	TTGCGCAACACACAAGAAGG	59.9	266
TRINITY_Contig4912	2	c	(ATCG)4accgtcagttgtagccggc(CGG) 4	47	623	669	CCAACCGAGCACCACTAAGT	59.965	TTGCGCAACACACAAGAAGG	59.9	266
TRINITY_Contig4912	3	p5	(TGGAA)4	20	999	1018	CCAACCGAGCACCACTAAGT	59.965	TTGCGCAACACACAAGAAGG	59.9	266
TRINITY_Contig4915	1	p3	(GCC)4	12	43	54	TTCCTTCTGGCAACGCAGAT	59.963	CTGACGTTGGGTTTGTGCTG	59.971	220
TRINITY_Contig4915	2	p4	(TTGG)3	12	2353	2364	TTCCTTCTGGCAACGCAGAT	59.963	CTGACGTTGGGTTTGTGCTG	59.971	220
TRINITY_Contig4918	1	p3	(GAA)4	12	889	900	TTTTGGGTGCTGGTCGCTAT	59.962	ACCAACACGCAGGATCACAT	59.963	276
TRINITY_Contig4927	1	p4	(CGGC)3	12	293	304	TCCAAAAAGTCCTCGCCCTC	59.964	CAACATAGAGCAGGTGGGCA	60.035	193
TRINITY_Contig4930	1	p5	(GGTTT)3	15	201	215	TGGATGGGGAGGAGATGGAG	60.105	CGGTCCGGATTTCTCCAACA	60.037	254
TRINITY_Contig4930	2	p4	(AGGG)3	12	4565	4576	TGGATGGGGAGGAGATGGAG	60.105	CGGTCCGGATTTCTCCAACA	60.037	254
TRINITY_Contig4932	1	p4	(CCTC)3	12	2659	2670	CTCAAGTGGGTTTGCACTGC	59.969	TTCAGAACCGGTGAAGGTCG	59.967	250
TRINITY_Contig4936	1	p5	(CGGGG)3	15	176	190	CTAACCATGTCTGTCGCGGT	60.109	CAACGTTTTGGCTCTGGACG	60.041	147
TRINITY_Contig4937	1	p4	(TGGA)3	12	1061	1072	TGGTGTGTAAGGTTCTCGCC	59.966	CCATTAGAGAAGCGCGGGAT	59.966	232
TRINITY_Contig4938	1	p3	(CCG)4	12	4007	4018	GCTGCCCAAATAAGCGGATG	59.969	AGCTGTTGCAATGGAGACGA	59.965	183
TRINITY_Contig4939	1	p3	(AAT)4	12	612	623	GTGGTGCTGGATTCACTCGA	60.037	TTGTCGTCCCTTTGCTCTCC	59.965	260
TRINITY_Contig4942	1	p4	(TGTC)3	12	1091	1102	AAGAAGATGAACAGGGGCGG	60.035	CAACGATAGATCCGCCGTCA	59.97	266
TRINITY_Contig4945	1	p3	(GAG)6	18	1130	1147	GGGTATCCGGATCCGTTCAC	59.967	CGCTCGTACCTACCGGTAAC	59.972	220
TRINITY_Contig4948	1	p3	(TAC)4	12	208	219	GCAGACTCAGACGACCAACA	59.968	AGCTCATTGGGGCAGATGTC	60.107	174
TRINITY_Contig4951	1	p3	(CGG)4	12	555	566	GGTGGAAGAGTGGAGTGGTG	59.965	TTTTGTTGGCTTCGGCTTCG	59.97	109
TRINITY_Contig4951	2	p2	(AG)10	20	1409	1428	GGTGGAAGAGTGGAGTGGTG	59.965	TTTTGTTGGCTTCGGCTTCG	59.97	109
TRINITY_Contig4951	3	c	(AGGGG)3ctgccgctgcgcccggccgggg aggaggccgaatcgggcgcccgaggaggccg aggcc(GCG)4	85	2843	2927	GGTGGAAGAGTGGAGTGGTG	59.965	TTTTGTTGGCTTCGGCTTCG	59.97	109
TRINITY_Contig4953	1	p3	(CGG)4	12	1375	1386	GTTGCCCATCTCCTTGAGCT	60.035	AAGATGGCCAATGAGCGGAA	60.034	147
TRINITY_Contig4964	1	p3	(GGC)4	12	823	834	ACGAGCCCAAAAATGTTGCC	59.967	GATCTACATCACGGCTGCCA	59.895	141
TRINITY_Contig4967	1	p3	(GGC)4	12	1574	1585	TCCGGGTGTAGACCGTAGTT	59.962	TTTCTTGTCGACATCCCCCG	60.037	270
TRINITY_Contig4969	1	p3	(GCA)4	12	1130	1141	AGATCTAGAAGGGGGCAGCA	60.031	ACAGCCCATAAAAGCCCTCC	60.033	215
TRINITY_Contig4971	1	c	(CAT)4a(ATC)5	28	2447	2474	GGATGGTCAACCTCAGTGCA	59.963	GGAGCAGGATTAGCGAGACC	59.967	178
TRINITY_Contig4971	2	p3	(GGT)8	24	4174	4197	GGATGGTCAACCTCAGTGCA	59.963	GGAGCAGGATTAGCGAGACC	59.967	178
TRINITY_Contig4971	3	c	(GCG)4(CGC)5	27	4724	4750	GGATGGTCAACCTCAGTGCA	59.963	GGAGCAGGATTAGCGAGACC	59.967	178
TRINITY_Contig4974	1	p3	(TCG)4	12	430	441	CACCAGTCGTTTCTTCGGGA	59.967	TCGGAGTTTCTCGCGTTCAA	59.969	126

TRINITY_Contig4976	1	c	(CTCGC)3gtcgccg(CGT)4	34	172	205	TTCGAGAGGAGGGTCTCTGG	60.034	TTGAGCGTTGCAGCAAACTC	59.971	214
TRINITY_Contig4976	2	p3	(CGC)4	12	12729	12740	TTCGAGAGGAGGGTCTCTGG	60.034	TTGAGCGTTGCAGCAAACTC	59.971	214
TRINITY_Contig4979	1	p3	(CGA)4	12	142	153	CCCGAGATCGAGACGTCTTG	59.972	TCAGACGTGGAAGCGTTCTC	60.039	104
TRINITY_Contig4981	1	c	(GAG)4cgcgtagccggcgtacttgcggaggtcggcgtcggaggagacgggggagggggccgaggagagcgcgcggaggagcagga(GGT)4	104	1784	1887	TCTTCAGCACACGCGTTACT	59.968	CAATTACAGGAGCATGGCGC	59.97	256
TRINITY_Contig4984	1	c	(ATGG)3accgac(AGCA)3	30	138	167	GGTTGAAGGTGAGGGAGTCG	60.037	CGAGCGACTGTACAGCCTAG	59.972	146
TRINITY_Contig4984	2	c	(AGC)5acca(CCG)5	34	1316	1349	GGTTGAAGGTGAGGGAGTCG	60.037	CGAGCGACTGTACAGCCTAG	59.972	146
TRINITY_Contig4986	1	c	(CGC)4cgtgcgtgtcgtcctggatgacgtggatgtcggcgaggcggcgcacgtcgttcttcttgacg(GCCGAC)3	92	574	665	GCGGCACCAAAATGTCCTTT	59.967	AAGCAGACCAAGCTCGACAA	59.893	133
TRINITY_Contig4987	1	p3	(GCG)5	15	210	224	TCAACGAATCGACCGTTCGT	60.04	CTGCCTGCTGATGTTCTGGA	60.036	127
TRINITY_Contig4991	1	c	(GGCA)3accatgggcaagcacggcaagtgcagccacgacgccgaggcgtgctacccgccgggcggcgtcccg(GGC)4	90	172	261	ATCATGATGGTCTTCGCGCT	59.894	TTGACAGGCAGCCTTCTCAG	59.964	259
TRINITY_Contig4991	2	p3	(TGG)4	12	611	622	ATCATGATGGTCTTCGCGCT	59.894	TTGACAGGCAGCCTTCTCAG	59.964	259
TRINITY_Contig4992	1	p3	(GGC)5	15	25	39	CCAACAACGTCGTGTGCATT	59.971	GAAGTTGAAGATGGCGACGC	59.905	227
TRINITY_Contig4995	1	p3	(GGA)5	15	1389	1403	ACGGTTGAGTCCCATTGTCC	59.964	AAGATTCAACCGAGCTGGGG	60.035	126
TRINITY_Contig5002	1	p3	(CGC)4	12	641	652	CGGCTAGCTCTCCATGTGAG	59.968	GCCATCGAGAGGACCTGATG	59.967	269
TRINITY_Contig5011	1	p3	(GGA)4	12	499	510	GTCGTTGGAGTTGGAGGAGG	60.037	TCCAAGTCCAATGAGCCGAC	60.036	100
TRINITY_Contig5013	1	p3	(CGG)4	12	3159	3170	CACCATTCTCCCGTGACACA	59.965	GGAGAGAGTGATGTCCCCCT	60.032	227
TRINITY_Contig5013	2	p3	(GGA)4	12	3464	3475	CACCATTCTCCCGTGACACA	59.965	GGAGAGAGTGATGTCCCCCT	60.032	227
TRINITY_Contig5015	1	p5	(CTCCT)3	15	314	328	CGTCACACGTCCCTTTCAGA	59.968	GCGCAGGAGGTAAGGTGTAA	59.752	148
TRINITY_Contig5015	2	p4	(CGAT)4	16	535	550	CGTCACACGTCCCTTTCAGA	59.968	GCGCAGGAGGTAAGGTGTAA	59.752	148
TRINITY_Contig5017	1	p4	(GCTA)3	12	809	820	GGGAGGAGAGGTGGATGGAT	60.105	CAGCTCGCCGAATACAAAGC	59.973	119
TRINITY_Contig5019	1	p2	(TA)9	18	31	48	GATCAGATCCCTGTGGCGAG	59.967	ACAGCAGCCATTACCGTCAA	59.964	243
TRINITY_Contig5019	2	p3	(TGG)4	12	512	523	GATCAGATCCCTGTGGCGAG	59.967	ACAGCAGCCATTACCGTCAA	59.964	243
TRINITY_Contig5020	1	c	(CGC)5agcatcgtcctcctcgcccgagaactcctcctccgcgccgccgtccaggaagcgcggcagg(AAC)4	88	1162	1249	GTGGTCACCCAATACCCCTG	60.034	GGGGCCACCTCTCATACAAC	60.107	120
TRINITY_Contig5020	2	p4	(TTCT)3	12	1874	1885	GTGGTCACCCAATACCCCTG	60.034	GGGGCCACCTCTCATACAAC	60.107	120
TRINITY_Contig5028	1	p4	(TCGC)3	12	1078	1089	GGTCCCATTCACCCACCAAT	59.959	CCCAACGTAACTCCACCTCC	60.037	108
TRINITY_Contig5035	1	p4	(GCCA)3	12	183	194	AGCAGATTGAGGAGATCGCG	59.968	ACCAACATCCCCGCTTGATT	59.96	118
TRINITY_Contig5039	1	p5	(CTGCT)3	15	2482	2496	ATGAGACTGACTGCCCAACG	60.037	AGGTTCCCTGGTTTTTGGCA	60.032	122
TRINITY_Contig5041	1	p4	(TATT)3	12	1209	1220	GGATCGAGTAGCTTACCGCC	60.039	CACAGCTTTCATGCGATCCG	59.973	253
TRINITY_Contig5044	1	p3	(CGC)4	12	346	357	GATGCAAGCTTCATCGCCAG	59.971	AACCAAGTCCTGCTTCCTCG	59.965	237
TRINITY_Contig5053	1	p4	(CCGC)3	12	282	293	CAGGGTCACAAGGGGACAAA	59.815	AGACCACGGAACCAAACACA	59.746	154
TRINITY_Contig5054	1	p3	(CCG)4	12	41	52	ATGTTCATGCGGCTACCCAA	60.035	TGCAGACGAGGCAAATCTGT	59.965	190
TRINITY_Contig5054	2	p6	(GTTTTT)3	18	714	731	ATGTTCATGCGGCTACCCAA	60.035	TGCAGACGAGGCAAATCTGT	59.965	190
TRINITY_Contig5055	1	p3	(GTC)4	12	1488	1499	TCGTAGGTGTAGAGCCAGCT	60.034	AGAGTCCCTGCTCCATGGAT	60.03	138
TRINITY_Contig5059	1	p3	(GAC)4	12	62	73	TCAGGTCAACACGCAGTACC	59.967	TTCGAACAGTACTGGGCCAC	59.966	147
TRINITY_Contig5059	2	p4	(GCGA)3	12	227	238	TCAGGTCAACACGCAGTACC	59.967	TTCGAACAGTACTGGGCCAC	59.966	147
TRINITY_Contig5063	1	p6	(CGGCGT)3	18	1153	1170	TGCCTTCAGAGAAGAAGCGG	60.037	AACCTGGACCACGACATGTC	59.965	194
TRINITY_Contig5065	1	p3	(TTC)4	12	998	1009	CGATGTCGTAGGTGAGGTCG	59.972	CCCTTCTCGCGGAGGAATAC	59.968	177
TRINITY_Contig5065	2	p3	(AGC)4	12	2767	2778	CGATGTCGTAGGTGAGGTCG	59.972	CCCTTCTCGCGGAGGAATAC	59.968	177
TRINITY_Contig5069	1	p3	(AGG)4	12	124	135	ACGGGTACGAGAGGATTCCA	60.034	ACCGTGGTTTCTTCGTCTCC	59.967	171
TRINITY_Contig5077	1	p5	(TTTCC)3	15	562	576	GGGAATCCGACCCCAATTGT	60.034	CTTCACAGCGTGGTGGTTTG	59.971	180
TRINITY_Contig5077	2	p4	(GCAC)3	12	1499	1510	GGGAATCCGACCCCAATTGT	60.034	CTTCACAGCGTGGTGGTTTG	59.971	180
TRINITY_Contig5077	3	p3	(GGC)5	15	1639	1653	GGGAATCCGACCCCAATTGT	60.034	CTTCACAGCGTGGTGGTTTG	59.971	180
TRINITY_Contig5081	1	c	(CCA)5aaaaagctgctaaagatgtcctgaatgttcatcccgccg(CCT)4	66	981	1046	TACCCTTGGTGCCGATTTCC	60.035	TGGGCGCTTCAGAAGAGAAG	60.037	131
TRINITY_Contig5082	1	p4	(AGGC)3	12	439	450	AAGAAGGGCATGAACGCTCA	59.963	GCTGAGCCTTTCGGAAGTCT	60.037	136
TRINITY_Contig5085	1	c	(GCG)4cgctggacgccgacgacagcacatccgtgttgtcgtcgtcctcgagcagcagcgagtcgtccagggagacggcc(GCG)5	101	892	992	CCCTGGTACGTGTCGACAAA	59.968	TCCATCAGTTCGCTAGTCGC	59.899	263
TRINITY_Contig5086	1	p3	(GGC)4	12	176	187	CCCTCTTCACCAAGCTCCTC	59.748	TCCTCCAGTCGCATTGGTTC	60.036	220
TRINITY_Contig5092	1	p4	(CGCT)3	12	73	84	CGCTGATTTCATCGCGTCAG	60.044	CGTCTCACTCGCCTGATCTC	59.971	116
TRINITY_Contig5092	2	p4	(GTTC)3	12	280	291	CGCTGATTTCATCGCGTCAG	60.044	CGTCTCACTCGCCTGATCTC	59.971	116
TRINITY_Contig5094	1	p4	(ATTT)3	12	562	573	TGTTCCTGATGTCCTGGTGC	59.963	TCGCAGCTTCTCTTCCACAG	60.038	164
TRINITY_Contig5099	1	p3	(CGC)4	12	1581	1592	TCATGGCAAGGTACGACCAC	60.037	TTGCATTTTCCTTTGCCGGG	59.965	194
TRINITY_Contig5100	1	p4	(AAGA)3	12	421	432	AAGTCAAGTCCGCCGAGAAG	60.038	TAGCTTCACTGCTAGCGCTC	59.899	228
TRINITY_Contig5104	1	p6	(GGAGGG)3	18	2800	2817	ATGAGCAGCTTGAGCATGGT	60.034	TCGCTGCAGCTCTTCTTCAA	59.966	227
TRINITY_Contig5105	1	p3	(CGC)4	12	378	389	TTTGTCGACGATGGCCTTGA	59.966	CTACTTCAGAGGCCACCTGG	59.461	234
TRINITY_Contig5107	1	p3	(GAG)4	12	2529	2540	ATTCTCCTCCTGCTCCCCAT	60.029	AGTGGCTCAGCTGCATGATT	60.034	271
TRINITY_Contig5107	2	p3	(CAG)4	12	3127	3138	ATTCTCCTCCTGCTCCCCAT	60.029	AGTGGCTCAGCTGCATGATT	60.034	271
TRINITY_Contig5107	3	c	(GCCG)4ggcgtcttagcttactactccgcgtacgtacgtgtgttttgtggtatatacagcaaacggtttttattaacagcagatcagatcagaatagggaggc(GA)9	131	3330	3460	ATTCTCCTCCTGCTCCCCAT	60.029	AGTGGCTCAGCTGCATGATT	60.034	271
TRINITY_Contig5107	4	p5	(TGGGA)3	15	4087	4101	ATTCTCCTCCTGCTCCCCAT	60.029	AGTGGCTCAGCTGCATGATT	60.034	271
TRINITY_Contig5107	5	p4	(TCCA)4	16	4246	4261	ATTCTCCTCCTGCTCCCCAT	60.029	AGTGGCTCAGCTGCATGATT	60.034	271
TRINITY_Contig5108	1	p3	(CGC)4	12	155	166	ACTCTGGAGATGAGGGGGAC	60.032	TCCGTCGCTCATCATGTAGC	59.969	166
TRINITY_Contig5108	2	p3	(GCC)4	12	3540	3551	ACTCTGGAGATGAGGGGGAC	60.032	TCCGTCGCTCATCATGTAGC	59.969	166
TRINITY_Contig5108	3	p3	(GCC)5	15	3708	3722	ACTCTGGAGATGAGGGGGAC	60.032	TCCGTCGCTCATCATGTAGC	59.969	166
TRINITY_Contig5115	1	c	(CTG)4tggctggccccacg(GCT)4	38	110	147	CTCAGCTTGTCCTGGAACGT	59.966	AACGTCAAGATCGTGGGCAT	60.036	209
TRINITY_Contig5115	2	p3	(GCC)4	12	599	610	CTCAGCTTGTCCTGGAACGT	59.966	AACGTCAAGATCGTGGGCAT	60.036	209
TRINITY_Contig5123	1	c*	(GGC)7gggttcgcgttgctgctgccgctgccgccgccgttcgtgtttgacgacgttggcgatgatctccca(CCG)4ctgccgaaa(CCG)5(GCCGAT)3*cccggagccgatgccgaggccgccgcctacgccgacccccagc(CCG)5aggctgacgcccaggtccttccgcgcaatgtggcggccctcagccgcggcaaccaa(CGC)4	263	2127	2389	CGGACCGTCAGTTCAACAGA	59.968	CAGTTGCTCTCAGTGGCGTA	60.038	253
TRINITY_Contig5123	2	p4	(GCCG)4	16	2661	2676	CGGACCGTCAGTTCAACAGA	59.968	CAGTTGCTCTCAGTGGCGTA	60.038	253
TRINITY_Contig5129	1	p3	(GAG)4	12	455	466	ACGATGAGGCATACGCTGAG	59.969	CTCTGCCTCCTCCTCCTCTT	60.032	149
TRINITY_Contig5133	1	p3	(CGA)4	12	79	90	CGAGTTGCGGTTAAGGAGGT	60.038	TCCAACGACACCCTTACGTG	59.968	111
TRINITY_Contig5137	1	c	(CTT)4ccacctgcaa(GAGT)3	34	604	637	TAGCATCGCCGATCCACTTC	59.968	CTGCCCAGATGTAGCTCGAG	59.968	172
TRINITY_Contig5137	2	c	(CTT)4ccacctgcaa(GAGT)3	34	5355	5388	TAGCATCGCCGATCCACTTC	59.968	CTGCCCAGATGTAGCTCGAG	59.968	172
TRINITY_Contig5138	1	p3	(CTT)5	15	2107	2121	AGCCATCCCCAGTTTTAGGC	60.033	AAAGGCTGTGATGGAGGCTC	60.035	221
TRINITY_Contig5140	1	p4	(TCCA)3	12	63	74	CGTGATCCCCCATTTCCACA	60.035	GAGTGGGTGGCTTGTACTCC	60.036	149
TRINITY_Contig5145	1	p3	(CTC)4	12	1108	1119	TCTTGTTCGCACTGGGGTAC	59.966	GTACGAGCGCTCAGAATGGA	59.899	133
TRINITY_Contig5147	1	p3	(CGC)4	12	535	546	AATCCATTTCGTGCGGTTCG	59.554	GCTGCACTCCATCTTCAGGT	60.036	210
TRINITY_Contig5149	1	p2	(TC)6	12	76	87	CCAGCAGCCTTTTGTCACAC	59.969	GACGAGGAGAGGGAGGAGTT	60.034	133
TRINITY_Contig5156	1	p5	(GTCGT)3	15	3788	3802	CCTTCGATGTCTCGCTCCTC	59.97	CGATCTCGATGGTGGTCTGG	59.969	243
TRINITY_Contig5156	2	p4	(GGTC)3	12	3911	3922	CCTTCGATGTCTCGCTCCTC	59.97	CGATCTCGATGGTGGTCTGG	59.969	243
TRINITY_Contig5162	1	p5	(GTCGC)3	15	658	672	GGCTTCGAGGACTATCACGG	59.969	AGATGACCAGCAGCTTGGAC	60.036	268
TRINITY_Contig5162	2	p3	(GCG)4	12	1170	1181	GGCTTCGAGGACTATCACGG	59.969	AGATGACCAGCAGCTTGGAC	60.036	268
TRINITY_Contig5167	1	p4	(AAGG)3	12	1131	1142	CTTTGCTGGCAAGGACCAAC	59.967	GTGTTCTCCCCCTGAAGAGC	60.036	156
TRINITY_Contig5172	1	p6	(ATCACC)3	18	94	111	TTTCCGCGGGAAGAATTCGA	60.037	AAGTTCCCTCGATCAGCAGC	60.108	125
TRINITY_Contig5172	2	p3	(CCT)4	12	227	238	TTTCCGCGGGAAGAATTCGA	60.037	AAGTTCCCTCGATCAGCAGC	60.108	125
TRINITY_Contig5172	3	p3	(CGC)4	12	348	359	TTTCCGCGGGAAGAATTCGA	60.037	AAGTTCCCTCGATCAGCAGC	60.108	125
TRINITY_Contig5172	4	p3	(CCG)5	15	487	501	TTTCCGCGGGAAGAATTCGA	60.037	AAGTTCCCTCGATCAGCAGC	60.108	125
TRINITY_Contig5173	1	p6	(CTCCCG)3	18	3605	3622	TAAACATAGGAGGGCCCGGA	60.031	GGAGGTTCCATGGCCATCAA	60.033	177
TRINITY_Contig5175	1	p3	(GCC)4	12	252	263	AGTGGAGGTTGTGGAGCATG	59.963	TGTGCATCCTCATCCACCAC	60.035	141
TRINITY_Contig5176	1	p5	(CAGCG)3	15	146	160	GTGATCACAGCCGCGAAATC	59.973	CCCGCAGACAGGAAAGACAT	60.036	177
TRINITY_Contig5188	1	p3	(GAG)4	12	2223	2234	GAGGAGGAGTTGGGGGAGAT	60.031	CCTCGCTCATCATCCTCACC	59.967	102
TRINITY_Contig5190	1	p3	(CTG)4	12	388	399	AGTGGTAGGGGAGGCATCAT	60.03	GCACTGGTTTCCTCTAGCCA	59.675	183
TRINITY_Contig5197	1	p3	(CGG)6	18	2202	2219	CCAGCAAAGTTCTGCACCAC	59.969	GTAGATGAGGCAGACGAGGC	59.969	261
TRINITY_Contig5199	1	p4	(CAAG)3	12	2153	2164	AGTCTGAACCTGCGAGTTGG	59.966	TGCTCCGGCGACTTAAAACT	59.966	188
TRINITY_Contig5204	1	p3	(ACG)4	12	189	200	GGAAGACGGCGAGATCGATT	59.969	GAAGTGAGCGTTGGAGACCA	59.966	139

TRINITY_Contig5208	1	c	(TCC)4tcggattcgctgtccccctgtccccagaaacgggatccctgcatggt(TGC)4ttttgcttcgacttggctcaagctctggagttcggg(GGC)6	125	3027	3151	AAGCAGCGCTTCAAAACTGG	59.969	TGGATACCAGAGGGACAGGG	60.031	220
TRINITY_Contig5211	1	p3	(GGC)4	12	432	443	GTCGTAGAAGATGAGCGCGA	59.972	CCACAACCACCACTCCTCTC	59.965	206
TRINITY_Contig5211	2	p3	(GGC)4	12	920	931	GTCGTAGAAGATGAGCGCGA	59.972	CCACAACCACCACTCCTCTC	59.965	206
TRINITY_Contig5212	1	c	(CTT)4(CTG)4	24	579	602	GCCGCAGAGAAGCTAGGAAA	60.108	CCCCATAAACAGCAGGAGCA	60.034	116
TRINITY_Contig5214	1	c	(CG)6cacgggccaccggagcaaagcgaggtggcgcggggtgcgctcggcgctgccggccgccaccgccaccgtgccggt(GCCGAC)4	111	1184	1294	CGGAACAAGGAGAAGGTGCT	59.965	GCGGTTCCCTTACTCTGCTT	60.036	140
TRINITY_Contig5225	1	p3	(TCG)5	15	201	215	ATCCCTTGGGCTTGGATGTG	60.033	ATGAGATGCTCGACGACGAC	59.972	119
TRINITY_Contig5231	1	p3	(TGC)4	12	87	98	CGGTGGAGTCGAGTATTCCG	59.971	AAGAGCATGGGGTTCGTGAG	60.036	133
TRINITY_Contig5231	2	p3	(ACG)7	21	708	728	CGGTGGAGTCGAGTATTCCG	59.971	AAGAGCATGGGGTTCGTGAG	60.036	133
TRINITY_Contig5243	1	p3	(TCG)4	12	351	362	GCACCAGCCTTCTTGTTGTG	59.969	TAAAGCCACCTGGATGGCTG	60.034	255
TRINITY_Contig5243	2	p3	(GCC)5	15	3499	3513	GCACCAGCCTTCTTGTTGTG	59.969	TAAAGCCACCTGGATGGCTG	60.034	255
TRINITY_Contig5243	3	p3	(TCC)5	15	4109	4123	GCACCAGCCTTCTTGTTGTG	59.969	TAAAGCCACCTGGATGGCTG	60.034	255
TRINITY_Contig5244	1	c	(CGC)4c(GGA)4ggcgcaggggcgcgcacggg(CGGGGC)4	71	3559	3629	TATGCACCCCACATGGCAAT	60.032	TTTTCTTGCAGCGAACCGTG	59.971	223
TRINITY_Contig5244	2	p4	(GGGC)3	12	3850	3861	TATGCACCCCACATGGCAAT	60.032	TTTTCTTGCAGCGAACCGTG	59.971	223
TRINITY_Contig5244	3	p3	(GGC)8	24	4133	4156	TATGCACCCCACATGGCAAT	60.032	TTTTCTTGCAGCGAACCGTG	59.971	223
TRINITY_Contig5252	1	p3	(CGT)4	12	538	549	ATCGGGGATTGGGGACACTA	60.031	ATGCAGCTTTGCCAAGGTTG	59.966	143
TRINITY_Contig5254	1	p3	(CGG)6	18	882	899	CCGGAGAGTCTTCCTGGAGA	60.034	ATACCTGTTGCCACGGTCAG	60.036	180
TRINITY_Contig5258	1	p5	(TAGCT)3	15	433	447	CTGCACCAGAAAGCTCCAGA	59.964	GAACATGATCGCTTGCTGCC	60.248	244
TRINITY_Contig5261	1	c	(AAAAG)3aaaactattctgccacaggaatctgtgcagtttcttcaatcagaa(GAAAAG)3	78	758	835	AAGATCGGGGTGAGGTAGCT	60.032	CAAGGCACCAGCTGTTGTTC	59.969	168
TRINITY_Contig5267	1	p4	(TGCT)3	12	878	889	AGTAGAACGCCACCAGGTTG	59.965	CTGAAGCTGTCCGTGTTCCT	59.966	235
TRINITY_Contig5267	2	p3	(CCG)4	12	1134	1145	AGTAGAACGCCACCAGGTTG	59.965	CTGAAGCTGTCCGTGTTCCT	59.966	235
TRINITY_Contig5267	3	c	(ACT)4atctaactgtcgtgt(TACA)3	39	3237	3275	AGTAGAACGCCACCAGGTTG	59.965	CTGAAGCTGTCCGTGTTCCT	59.966	235
TRINITY_Contig5272	1	p5	(CGAGC)3	15	1270	1284	CAGCACTCCCATGGTCATGT	60.035	GTGACCGAGCTAAAGCACCT	60.037	280
TRINITY_Contig5275	1	c	(CCG)4cgcgcgccttcatggccaccttggcagggccgttcttcccctt cttcttccctttgc(TCT)4	81	3159	3239	CCAGCTGCTCTTTCCTGGAA	59.963	GCTTGCAGCTGAAAACCGAA	59.969	238
TRINITY_Contig5275	2	p5	(GGCGC)3	15	3367	3381	CCAGCTGCTCTTTCCTGGAA	59.963	GCTTGCAGCTGAAAACCGAA	59.969	238
TRINITY_Contig5276	1	p3	(TCG)4	12	472	483	CACGGTGCGGTAAATGTTGG	60.11	GGCCGAGACCAAGACGTTTA	60.038	247
TRINITY_Contig5277	1	p3	(TCA)4	12	632	643	CTTGCTCGTCGCTCAATTCG	59.976	CGTGAATAAGAGCGCTGTGC	59.974	224
TRINITY_Contig5293	1	p4	(GGGA)3	12	696	707	TGGGGGTACACGTACAGCTA	59.961	GCCTAACCCCAGGGAAAGTC	60.034	162
TRINITY_Contig5296	1	p3	(GCG)4	12	236	247	TCGATCTCCGACCACTCCTC	60.464	CTTCCTCCAGGAGCTCCTCT	60.032	150
TRINITY_Contig5299	1	p3	(GCC)4	12	240	251	CTTGCACACCACGAAACCTG	59.971	ATTCCGCAGCAGGATAGAGC	59.965	270
TRINITY_Contig5300	1	p3	(TCG)4	12	8	19	GCCAACACCACCTTCACAAC	59.898	ATGCTGTTGTAGTAGCCGGG	59.822	188
TRINITY_Contig5302	1	p2	(CG)6	12	275	286	TTCCTAGCGACCTTGCAGTG	60.037	GGCAAACACTCCTCCGTACA	59.966	120
TRINITY_Contig5302	2	p3	(CGG)4	12	397	408	TTCCTAGCGACCTTGCAGTG	60.037	GGCAAACACTCCTCCGTACA	59.966	120
TRINITY_Contig5306	1	p3	(TCC)4	12	1417	1428	CCAAAGGAATCGGTGAGCCT	60.035	TGAAGCTCCACCAGATGCTG	60.036	261
TRINITY_Contig5313	1	p3	(GAA)4	12	186	197	GGCAGTGACCTTCATCGACA	60.037	CCCCAGTCGAAGAAGTGGTC	60.037	147
TRINITY_Contig5319	1	p4	(AGCC)3	12	498	509	TTCTTTGCAGCATGCTTCCC	59.395	TGGTGAGCAGGATCCTCCAT	60.327	222
TRINITY_Contig5320	1	p3	(CCG)5	15	1535	1549	TCCTAAACACACGGTGAGGC	59.966	GCAAACTGTGACAGCAAGGG	59.969	112
TRINITY_Contig5322	1	c	(CGC)7aaagcgccatacaaacacctcgcacagctcgaatccgtcacacaacctta(CCGC)3	83	556	638	CGGAGATTCGTGTCTCGGAG	59.972	CGGTAAGGTTGTGTGACGGA	59.968	151
TRINITY_Contig5322	2	c	(CCG)4aggccctggagcgcctccatctcgaccccgcttctgatggtgatcca(CCG)4	71	784	854	CGGAGATTCGTGTCTCGGAG	59.972	CGGTAAGGTTGTGTGACGGA	59.968	151
TRINITY_Contig5322	3	p6	(CTGATG)4	24	1225	1248	CGGAGATTCGTGTCTCGGAG	59.972	CGGTAAGGTTGTGTGACGGA	59.968	151
TRINITY_Contig5325	1	p3	(GCC)4	12	383	394	CAGTGACCACCAAAGTTGCG	59.971	AAACGAGCAGAGACAAGGGG	59.965	177
TRINITY_Contig5327	1	c	(GAG)5cggtacgtgcaggtggagtcgaggttcttccgggtgaagccgc(GCG)4	70	277	346	CACGACCCTGAGATCTACGC	59.97	CGTCATCATCACCCTCCTCG	59.969	148
TRINITY_Contig5327	2	p3	(GCG)6	18	538	555	CACGACCCTGAGATCTACGC	59.97	CGTCATCATCACCCTCCTCG	59.969	148
TRINITY_Contig5334	1	p5	(ACCAA)3	15	1100	1114	ACGTGACCAGCAAGAGTCTG	59.967	GAGCTTTGACAGGTCCAGCT	59.964	225
TRINITY_Contig5336	1	p4	(TCCC)3	12	2109	2120	GTCCCTCCCTCCCCATAGTT	60.03	ACGTCGCGATCATCCCTTAC	59.97	202
TRINITY_Contig5337	1	p3	(CCT)4	12	1391	1402	TGCACAGTGAAGCATCAGGT	59.891	CTTTGCGCTGCACAGAAGTT	59.97	161
TRINITY_Contig5345	1	p3	(TCG)4	12	554	565	CGAGGAGCTTGGACTCGATC	59.97	ACAAGGACTTCTGTGAGGCG	59.966	105
TRINITY_Contig5346	1	p3	(TCG)4	12	2908	2919	GGTGTCAGTACGAAGGGGTG	60.038	AGAAAGCTTTTCGCCTCGGA	59.965	100
TRINITY_Contig5350	1	p3	(CGC)5	15	54	68	GAGCAGATCAAGTCGCTCGA	59.9	GTGGAGGACGTCGAACTTGT	59.968	198
TRINITY_Contig5350	2	p3	(GGC)5	15	187	201	GAGCAGATCAAGTCGCTCGA	59.9	GTGGAGGACGTCGAACTTGT	59.968	198
TRINITY_Contig5350	3	c	(AGC)4tgcaa(CGG)4	29	559	587	GAGCAGATCAAGTCGCTCGA	59.9	GTGGAGGACGTCGAACTTGT	59.968	198
TRINITY_Contig5350	4	p2	(TA)6	12	1170	1181	GAGCAGATCAAGTCGCTCGA	59.9	GTGGAGGACGTCGAACTTGT	59.968	198
TRINITY_Contig5357	1	p2	(TC)8	16	121	136	TCTATCCTGGGCTCGGAACA	60.033	GAAAAAGAAAGGCGCCGGTT	59.968	275
TRINITY_Contig5357	2	p5	(TGGAT)3	15	324	338	TCTATCCTGGGCTCGGAACA	60.033	GAAAAAGAAAGGCGCCGGTT	59.968	275
TRINITY_Contig5357	3	p3	(ACG)5	15	666	680	TCTATCCTGGGCTCGGAACA	60.033	GAAAAAGAAAGGCGCCGGTT	59.968	275
TRINITY_Contig5357	4	p2	(CT)6	12	936	947	TCTATCCTGGGCTCGGAACA	60.033	GAAAAAGAAAGGCGCCGGTT	59.968	275
TRINITY_Contig5358	1	p4	(TGCT)3	12	741	752	GATCAACGCCACGATCAAGC	59.973	TGTGCTGTGGTTTGAGTGGT	60.035	164
TRINITY_Contig5359	1	p3	(GCG)5	15	166	180	CTGGGTTAGTTCTGCCGTGT	59.965	TATGTGACAACGGCAGCCAT	60.036	234
TRINITY_Contig5360	1	p3	(CGC)4	12	14	25	AGCAAGTCGTACGAGCCAAA	59.967	CGTTTGTGCTGACGCTGAAA	59.973	115
TRINITY_Contig5361	1	p3	(GAT)4	12	455	466	CACCACCCTGACCTTCAACA	59.817	GGGACCAGTGTGAGACTTGG	59.965	103
TRINITY_Contig5364	1	p3	(GCG)5	15	278	292	GGAGGGAAGGAGCAGTTTCC	60.035	CACCAGCTTTGTTCCACAGC	59.969	205
TRINITY_Contig5366	1	p5	(CGCCG)3	15	69	83	AACTGGAGCTCAGAATGCCC	60.035	ATAACGGTGGTGTCAGCCTG	60.036	180
TRINITY_Contig5366	2	p6	(CCATCC)3	18	228	245	AACTGGAGCTCAGAATGCCC	60.035	ATAACGGTGGTGTCAGCCTG	60.036	180
TRINITY_Contig5368	1	p6	(CTCGCT)3	18	456	473	GACTTCCTCGCGGACGTTAT	59.9	CGAAGGCTGTCTCTTGCAGA	60.038	130
TRINITY_Contig5373	1	p3	(CCG)4	12	209	220	GCAAGTGGATTTGTGCGGTT	59.968	ACTGCGAACCTGACAGTGAG	59.967	127
TRINITY_Contig5374	1	p4	(GCGA)3	12	31	42	GCCGTAGCTTCCTCTTCCTC	59.897	AGCAGCGCCAATTAGGGAAT	60.107	102
TRINITY_Contig5374	2	p3	(CTC)4	12	346	357	GCCGTAGCTTCCTCTTCCTC	59.897	AGCAGCGCCAATTAGGGAAT	60.107	102
TRINITY_Contig5375	1	p3	(CGG)4	12	1445	1456	TGGTAGAAGAGGCCGATGGA	60.033	GCTCGAGTATCTTGGAGGCC	59.967	184
TRINITY_Contig5381	1	p3	(CGG)4	12	47	58	AGATCACCGTGCAGTCATCG	60.179	AGTTCACCTTGTCGAGCACC	60.25	108
TRINITY_Contig5382	1	p5	(CCAAT)4	20	102	121	AAAGATGAGGTGGTCCTGCG	60.036	ATGGTGAGGTGCTTGATGGG	60.034	267
TRINITY_Contig5382	2	p5	(AATGC)4	20	1476	1495	AAAGATGAGGTGGTCCTGCG	60.036	ATGGTGAGGTGCTTGATGGG	60.034	267
TRINITY_Contig5384	1	p3	(TGC)7	21	2184	2204	TGGCGAATCTGCTGTGAGTT	59.965	GGATGATGCGGAGGAAGAGG	59.965	267
TRINITY_Contig5384	2	p3	(GTG)5	15	2387	2401	TGGCGAATCTGCTGTGAGTT	59.965	GGATGATGCGGAGGAAGAGG	59.965	267
TRINITY_Contig5384	3	p3	(GCC)5	15	2753	2767	TGGCGAATCTGCTGTGAGTT	59.965	GGATGATGCGGAGGAAGAGG	59.965	267
TRINITY_Contig5385	1	p4	(AGCG)4	16	58	73	CTCGAGGACAAGGAGGAGGA	60.034	CGCCCAGTTCTTGAGGTTCT	59.965	138
TRINITY_Contig5385	2	p4	(TGAA)3	12	1607	1618	CTCGAGGACAAGGAGGAGGA	60.034	CGCCCAGTTCTTGAGGTTCT	59.965	138
TRINITY_Contig5387	1	p3	(CGT)6	18	293	310	GTGGGTCAACGACTTCGACT	59.968	GGCTCACCTCCTCAAGAACC	60.036	179
TRINITY_Contig5389	1	p2	(GT)7	14	183	196	GTGTGTGTGTGTGTGGCATC	59.971	ACTGACCGACCATACCCGTA	60.034	126
TRINITY_Contig5389	2	c	(TGC)6ggtgatg(GCTCCT)3	43	346	388	GTGTGTGTGTGTGTGGCATC	59.971	ACTGACCGACCATACCCGTA	60.034	126
TRINITY_Contig5389	3	c	(GGC)5ctcctcctccttcctctccgct(CTC)5	52	1291	1342	GTGTGTGTGTGTGTGGCATC	59.971	ACTGACCGACCATACCCGTA	60.034	126
TRINITY_Contig5394	1	p3	(CGG)4	12	372	383	TCTCCCACCTGTCGTTTTCG	59.967	TCTCCCCAATCCTAGACGCA	60.033	201
TRINITY_Contig5394	2	p5	(CGAGA)3	15	3050	3064	TCTCCCACCTGTCGTTTTCG	59.967	TCTCCCCAATCCTAGACGCA	60.033	201
TRINITY_Contig5399	1	p3	(GCG)5	15	184	198	CCTCAATACGCGCATACCCT	59.966	CACTGTACTCATGGGGTGGG	59.747	221
TRINITY_Contig5400	1	p3	(TTG)6	18	727	744	TTGATTCGGCTCCCCAGAAC	60.035	AGAAGGACAAGGACAAGCCG	59.965	244
TRINITY_Contig5401	1	p3	(TCT)4	12	483	494	TTCTCGGTATGGCGATGACG	59.97	AGAAGGACGAAGCACACGAG	60.039	106
TRINITY_Contig5403	1	p2	(CT)13	26	26	51	GGCAATGGCATTTTGGCTGA	60.036	CCCGTCACCAGGCTGTTAAT	60.035	104
TRINITY_Contig5410	1	p4	(CACG)3	12	22	33	GTCCGAGTCCATGAGCTACG	59.97	AGGCACACGCATCTTAACCA	59.964	194
TRINITY_Contig5418	1	c	(CGG)4cgtccat(GGGGAC)3	37	1695	1731	GCAGCAATTGGAGCATAGCC	59.968	GCAAGGTTGGCTTTTGAGGG	59.966	143
TRINITY_Contig5418	2	c	(GCC)4gctgccagtcggcgacgcgacggacgcggggcacgggtccatgtagtagcagccgtcg(TCC)4	82	2221	2302	GCAGCAATTGGAGCATAGCC	59.968	GCAAGGTTGGCTTTTGAGGG	59.966	143
TRINITY_Contig5418	3	p3	(CAT)4	12	2455	2466	GCAGCAATTGGAGCATAGCC	59.968	GCAAGGTTGGCTTTTGAGGG	59.966	143
TRINITY_Contig5423	1	p3	(GCC)4	12	52	63	GGTAGGCGCTGAAGAAGGAG	60.179	ACTCGTGTGATTTCCGGACG	60.388	195
TRINITY_Contig5426	1	p2	(CT)7	14	56	69	GACGCGTCGCTCCATTAAAC	59.975	GGAGAGGTGGGAGAGGTCAT	60.032	275
TRINITY_Contig5428	1	p3	(CTG)4	12	1404	1415	TCCAAATCCCGAGGCTCCTA	60.032	AAGTACTTCGCCGACTGCAA	59.967	212
TRINITY_Contig5436	1	p4	(ATCA)3	12	210	221	GTCGAAGAACAACGCACCAG	59.768	TCCTGGTACATGCCGGTAGA	60.033	189
TRINITY_Contig5437	1	c	(GAG)4gccgagttggcttcgtgcggtgcggg cacgtcgttgctgact(TGC)5	69	454	522	CCTGCCGCTGAAAGGAAGTA	60.036	ACGTAACAGGTCGACCAACC	59.967	155
TRINITY_Contig5437	2	c	(CGA)4ctagcgagtaaccaaaacattgtgtccaatccactcacgagcgcgg(CGGC)3	70	1823	1892	CCTGCCGCTGAAAGGAAGTA	60.036	ACGTAACAGGTCGACCAACC	59.967	155
TRINITY_Contig5438	1	p3	(ATT)4	12	247	258	GTCATGCCGGATCACCAGAA	60.108	CACGGGTTGCCTCCAAATTT	59.321	100

TRINITY_Contig5445	1	p4	(CTAG)4	16	379	394	CCGGACCAAACTCTCAACGA	59.967	GGCTGCTTGAGGGTGAATCT	60.035	189
TRINITY_Contig5445	2	p3	(GGA)4	12	985	996	CCGGACCAAACTCTCAACGA	59.967	GGCTGCTTGAGGGTGAATCT	60.035	189
TRINITY_Contig5446	1	p3	(TGC)4	12	700	711	TGTTTTTCGCATGCTGCACA	59.898	TGATCTGTCGTTTTGGCCGA	59.966	244
TRINITY_Contig5446	2	p3	(GCC)5	15	1049	1063	TGTTTTTCGCATGCTGCACA	59.898	TGATCTGTCGTTTTGGCCGA	59.966	244
TRINITY_Contig5447	1	p3	(GAC)4	12	44	55	GTTCTTCCCCGTCTTCCAGG	60.036	ACAGTGCTCAATGACGTCGT	59.968	121
TRINITY_Contig5449	1	p4	(GGTC)3	12	578	589	GAGCCACCAGACGTCAAAGA	59.966	CGGCATTCTCCACCAAAAGC	60.109	178
TRINITY_Contig5459	1	p3	(GCA)5	15	162	176	AACGTGTTCGACCCCTTCTC	59.967	CGACCTCCACCTTCACATCC	60.108	226
TRINITY_Contig5460	1	c	(TC)9tggaatggttgtgt(AGGC)3a(GGAG)4	61	88	148	GGCTGCAACACAATCTGCAA	59.968	GGCCTTTGTGTACGTGGAGA	59.966	156
TRINITY_Contig5463	1	p3	(GCT)5	15	334	348	GGCTGGATGACGAGATGGAG	59.967	CCTCATCAGACTCCTCCCCA	60.032	177
TRINITY_Contig5463	2	c	(CGCGAG)3cgctcgtcctcgcggcaccaccgcgacgacggcgaccgg(GACCGC)3	75	1184	1258	GGCTGGATGACGAGATGGAG	59.967	CCTCATCAGACTCCTCCCCA	60.032	177
TRINITY_Contig5463	3	p3	(CAC)5	15	1508	1522	GGCTGGATGACGAGATGGAG	59.967	CCTCATCAGACTCCTCCCCA	60.032	177
TRINITY_Contig5464	1	p3	(GGC)4	12	3140	3151	GGGTCAGTTCCTTCTCAGCC	60.036	GCAGAATGCTTCATGAGGCG	59.971	133
TRINITY_Contig5465	1	p3	(CGC)4	12	139	150	CGAACGTCGTTGTGTAGGGA	60.04	CCGCCGTACAAGAACACCAT	60.672	130
TRINITY_Contig5466	1	p4	(CGTG)3	12	44	55	TGGATCGTGGTGGAGGAGAT	60.032	GCATCTGCCCCATTTTTGCA	60.036	279
TRINITY_Contig5466	2	p3	(GAC)4	12	812	823	TGGATCGTGGTGGAGGAGAT	60.032	GCATCTGCCCCATTTTTGCA	60.036	279
TRINITY_Contig5466	3	p5	(GATCT)3	15	1117	1131	TGGATCGTGGTGGAGGAGAT	60.032	GCATCTGCCCCATTTTTGCA	60.036	279
TRINITY_Contig5469	1	p5	(GCCGA)3	15	176	190	ACCCATTAAAATCGCGTGCG	59.902	TCCGTTGGAGTTCAGACTGC	59.966	115
TRINITY_Contig5478	1	p3	(GCG)4	12	1428	1439	GCCACGCCCATTTACGATTC	59.971	CGTGAAGCAGGAGGTCATGT	60.037	126
TRINITY_Contig5480	1	p4	(CCAT)3	12	303	314	ACAGACCGGACAGCAATACG	60.109	ATCGGCATCTGCGAATCTGT	59.894	192
TRINITY_Contig5488	1	p4	(AAAG)5	20	183	202	AGCTGGGGCACTGATAGGTA	60.031	CTGGGCCACGTATGAACCTT	60.035	205
TRINITY_Contig5488	2	p3	(CGC)4	12	3848	3859	AGCTGGGGCACTGATAGGTA	60.031	CTGGGCCACGTATGAACCTT	60.035	205
TRINITY_Contig5489	1	p3	(GGT)4	12	3878	3889	AAGTGCCATCATCTGCTGCT	60.034	CTGTCAAAACTGCACCTGGC	59.969	212
TRINITY_Contig5489	2	p6	(GCGGCT)3	18	4160	4177	AAGTGCCATCATCTGCTGCT	60.034	CTGTCAAAACTGCACCTGGC	59.969	212
TRINITY_Contig5489	3	p4	(GGTT)3	12	5530	5541	AAGTGCCATCATCTGCTGCT	60.034	CTGTCAAAACTGCACCTGGC	59.969	212
TRINITY_Contig5490	1	p5	(AGGAA)3	15	1159	1173	TGCCTCAGCCAGTAGGGTAT	60.031	TTATGCGTGGGACTGCCAAT	60.035	102
TRINITY_Contig5495	1	p2	(AT)10	20	3265	3284	TGGATTGTCCTGCGGAACTC	60.036	GTCTGGTGGGAGAAAGCCTC	60.036	139
TRINITY_Contig5495	2	p3	(CCG)4	12	3828	3839	TGGATTGTCCTGCGGAACTC	60.036	GTCTGGTGGGAGAAAGCCTC	60.036	139
TRINITY_Contig5495	3	p4	(GCCG)3	12	4009	4020	TGGATTGTCCTGCGGAACTC	60.036	GTCTGGTGGGAGAAAGCCTC	60.036	139
TRINITY_Contig5497	1	p4	(CCGG)3	12	833	844	TAGCTAGCTGCAGTGGGAGA	60.033	CGGGAAGCAGCTTCACCTTA	60.036	156
TRINITY_Contig5497	2	p3	(TGC)5	15	1232	1246	TAGCTAGCTGCAGTGGGAGA	60.033	CGGGAAGCAGCTTCACCTTA	60.036	156
TRINITY_Contig5497	3	p3	(GCG)5	15	1581	1595	TAGCTAGCTGCAGTGGGAGA	60.033	CGGGAAGCAGCTTCACCTTA	60.036	156
TRINITY_Contig5497	4	c	(GCG)4gaagggaggaggcgattgggtgggcgagtgga(TAGC)4	60	1809	1868	TAGCTAGCTGCAGTGGGAGA	60.033	CGGGAAGCAGCTTCACCTTA	60.036	156
TRINITY_Contig5497	5	p3	(GGC)4	12	2022	2033	TAGCTAGCTGCAGTGGGAGA	60.033	CGGGAAGCAGCTTCACCTTA	60.036	156
TRINITY_Contig5497	6	p5	(GTGGA)3	15	2137	2151	TAGCTAGCTGCAGTGGGAGA	60.033	CGGGAAGCAGCTTCACCTTA	60.036	156
TRINITY_Contig5500	1	p3	(CGC)4	12	1755	1766	TCGCATTCTTGGCGAGTTCT	60.037	AGGCCCTCAAATGCAGAGTC	60.035	169
TRINITY_Contig5505	1	p3	(AAG)4	12	616	627	ACGTGGACCAGTTGATCGAC	60.039	GTAAGGGGTGGGTGGAATGG	60.034	117
TRINITY_Contig5505	2	p2	(CT)6	12	911	922	ACGTGGACCAGTTGATCGAC	60.039	GTAAGGGGTGGGTGGAATGG	60.034	117
TRINITY_Contig5508	1	p3	(GGT)4	12	2349	2360	GGTCTTCACGGATGGAAGCA	60.036	TTGAAGGTGGTGCTGGTTGT	60.034	250
TRINITY_Contig5519	1	p2	(AG)9	18	220	237	AGCCCTGGGATGCTTACCTA	60.03	TCTCTCCTTCCACCCTCGAG	60.034	240
TRINITY_Contig5521	1	p2	(AG)6	12	4261	4272	ATCTCGTCCTCGTCCACTGA	60.035	CAAGGCTTCCAGAGAGCGA	60.037	160
TRINITY_Contig5521	2	p3	(GCC)4	12	4806	4817	ATCTCGTCCTCGTCCACTGA	60.035	CAAGGCTTCCAGAGAGCGA	60.037	160
TRINITY_Contig5521	3	p4	(AGGG)3	12	5088	5099	ATCTCGTCCTCGTCCACTGA	60.035	CAAGGCTTCCAGAGAGCGA	60.037	160
TRINITY_Contig5521	4	p3	(AGC)5	15	5644	5658	ATCTCGTCCTCGTCCACTGA	60.035	CAAGGCTTCCAGAGAGCGA	60.037	160
TRINITY_Contig5524	1	p5	(AAATC)3	15	91	105	CAACCACACTGCTTGCAGAC	59.97	GCAGAACAGTGCACCAACTG	59.97	188
TRINITY_Contig5524	2	p4	(GGGA)3	12	4510	4521	CAACCACACTGCTTGCAGAC	59.97	GCAGAACAGTGCACCAACTG	59.97	188
TRINITY_Contig5525	1	c	(GGCG)3ggccggggatccggagcgcctggagcagggaggag(CCT)4ttctct(CTCC)3	77	3409	3485	CCGGAGTTCTTGCTCATGGT	60.036	GTTGATGCGATTCCAGCGAC	59.973	184
TRINITY_Contig5525	2	p5	(GGGAG)3	15	3667	3681	CCGGAGTTCTTGCTCATGGT	60.036	GTTGATGCGATTCCAGCGAC	59.973	184
TRINITY_Contig5526	1	p3	(TTG)4	12	369	380	GTGATGAGGGTGGCGTAGAG	59.897	CTGCCCACGGATGATCAGTT	60.108	146
TRINITY_Contig5528	1	p5	(AAAAG)3	15	44	58	TCGCGAGGTGTATGATGTCG	59.971	CTGGTTTCATGTGCTTGGCC	60.038	277
TRINITY_Contig5535	1	p3	(CCG)4	12	435	446	ACAAGGCCTTTCCCATAGGC	60.033	ATGGAAAGCTGGGATGGTGG	60.033	262
TRINITY_Contig5538	1	p3	(AGG)7	21	1569	1589	GCATCAACTCCTGGCCTCTT	60.035	TTGAAGACCATGTCAGGGGC	59.962	175
TRINITY_Contig5541	1	p3	(CGC)6	18	1016	1033	CTTGGGCTTCGACTGGATGT	60.036	AAAAACCCTCGAAAAGCGGC	59.968	187
TRINITY_Contig5544	1	p3	(CCG)4	12	1049	1060	GTGTAGCATGCGCATGACTG	59.973	CGTACGTGCCAATTCCGTTC	59.906	214
TRINITY_Contig5545	1	p6	(AAAATC)3	18	1506	1523	CAACTGGCACCCTACGAACT	59.965	AGGGTTGTATGTGCCCATGG	60.033	177
TRINITY_Contig5545	2	p6	(GGGGGA)4	24	3240	3263	CAACTGGCACCCTACGAACT	59.965	AGGGTTGTATGTGCCCATGG	60.033	177
TRINITY_Contig5552	1	p3	(CGC)4	12	311	322	GGGGTTGAGACTTGGACTCG	60.037	ATCCCCCTGAACAAACGACC	59.962	191
TRINITY_Contig5555	1	p3	(CGC)5	15	1469	1483	AGCGGACGACCTAGTAGTGT	60.036	AATTGTTGAACCAGCACGGC	59.968	111
TRINITY_Contig5557	1	p3	(GCG)4	12	195	206	CATGTCACCCCAGGAAGCTT	59.961	GTCAGGACGAGGCTGGAAAA	59.965	203
TRINITY_Contig5561	1	p5	(GTCCC)3	15	54	68	GCCCGAGTACGAGAACTACG	59.973	CTGGCGCTCGTAGCTTATGA	59.969	100
TRINITY_Contig5561	2	p3	(GGC)4	12	597	608	GCCCGAGTACGAGAACTACG	59.973	CTGGCGCTCGTAGCTTATGA	59.969	100
TRINITY_Contig5562	1	p4	(CCAG)3	12	270	281	CTCCTCGCTCTGTCGGAATC	59.97	GGAGAGATGTAGGTGGGGGT	60.031	224
TRINITY_Contig5562	2	p3	(GCA)5	15	534	548	CTCCTCGCTCTGTCGGAATC	59.97	GGAGAGATGTAGGTGGGGGT	60.031	224
TRINITY_Contig5564	1	p4	(TGCT)3	12	245	256	AGAACCTGCTTCGTCAGGTG	59.966	TCCTGTAACACACCGTCTGC	59.967	134
TRINITY_Contig5564	2	p3	(CGC)5	15	855	869	AGAACCTGCTTCGTCAGGTG	59.966	TCCTGTAACACACCGTCTGC	59.967	134
TRINITY_Contig5564	3	p3	(CCG)4	12	994	1005	AGAACCTGCTTCGTCAGGTG	59.966	TCCTGTAACACACCGTCTGC	59.967	134
TRINITY_Contig5564	4	p3	(GGC)4	12	1203	1214	AGAACCTGCTTCGTCAGGTG	59.966	TCCTGTAACACACCGTCTGC	59.967	134
TRINITY_Contig5564	5	p3	(GCG)4	12	1489	1500	AGAACCTGCTTCGTCAGGTG	59.966	TCCTGTAACACACCGTCTGC	59.967	134
TRINITY_Contig5568	1	p3	(AAT)4	12	803	814	GCCTGCTCCATTCTTGGTCT	60.035	CAGATTCCGAGGACGACGAG	59.972	199
TRINITY_Contig5575	1	p4	(ACTG)3	12	1587	1598	AGGCGAAGAATGCACACTCA	59.965	ACACCCTAGAGGCTATGGCA	60.031	103
TRINITY_Contig5575	2	p3	(GCG)7	21	4271	4291	AGGCGAAGAATGCACACTCA	59.965	ACACCCTAGAGGCTATGGCA	60.031	103
TRINITY_Contig5575	3	p3	(CGG)4	12	4591	4602	AGGCGAAGAATGCACACTCA	59.965	ACACCCTAGAGGCTATGGCA	60.031	103
TRINITY_Contig5585	1	c	(AAG)4agcaaaagctcaatgagcgagctgacaccgctgattgtcaagggcgatgagaagag(GAA)4	80	158	237	TATCCGCTGCCAGCAGTATG	59.966	TTTCCTCCTCGCTCGTTGAC	60.039	246
TRINITY_Contig5588	1	p4	(CGAG)3	12	541	552	GAGTTGTTGCTGTTGCTGGG	59.969	GACGCGTCTACCGAATCCAT	59.97	270
TRINITY_Contig5592	1	p6	(GCCGCT)3	18	174	191	TAGAGGTCACCATGCGGAGA	60.034	TCTGAGCAAGGTGACTGCAG	59.966	165
TRINITY_Contig5592	2	p3	(TGG)4	12	3990	4001	TAGAGGTCACCATGCGGAGA	60.034	TCTGAGCAAGGTGACTGCAG	59.966	165
TRINITY_Contig5595	1	p3	(ACT)4	12	13	24	GCTGTCTCTTGGGCTGTCTT	59.964	GCAGTTGCATATGCTGTCGG	59.971	175
TRINITY_Contig5602	1	p3	(AGG)4	12	517	528	TCGAGCATGATGATGGCCTC	59.966	CCGATTCCTCGTAGCAGACC	59.969	223
TRINITY_Contig5602	2	p4	(CGTG)3	12	1351	1362	TCGAGCATGATGATGGCCTC	59.966	CCGATTCCTCGTAGCAGACC	59.969	223
TRINITY_Contig5603	1	c*	(CGG)3(CGGGCGGGCGGCGGCGGCGGCGGTGCGAAGAACACGGCGACCGTGTTCGTCCTTTTGGCCTTTTTTTTTTCTAGACTAGATCCCCTATCGAATACCATACCATGTGTACATTAAGTGCCACGCTTTGATATTAGTACTTCGAACAGGGTACATCCGATCCGACGATG<GGCGG>GCGGGCGGCGGCGGCGGCGGTGCGAAGAACACGGCGACCGTGTTCGTCCTTTTGGCCTTTTTTTTTTCTAGACTAGATCCCCTATCGAATACCATACCATGTGTACATTAAGTGCCACGCTTTGATATTAGTACTTCGAACAGGGTACATCCGATCCGACGATGA)(GGCG)2(GGC)5*	30	4108	4137	GGACGCCGGTATTCTCTCTG	59.969	CCAGCTCCAGTTGTGCAAAC	59.969	140
TRINITY_Contig5607	1	p4	(ATAG)3	12	158	169	AGCATGGCTCAAGGCTCAAT	60.033	GTGGTCTGCCCTTGTTCTCA	59.891	162
TRINITY_Contig5608	1	p3	(GCG)4	12	177	188	GTCAGGCTGTCCCTTCCATC	60.107	TAGAAGAGCCAGGGCAGGAT	60.031	188
TRINITY_Contig5609	1	p4	(CTCG)3	12	101	112	GCTGTTTTTGCCCATGCTCA	59.966	CGGGCCTCCAAGTACTGTTT	59.964	122
TRINITY_Contig5609	2	p4	(CCAA)5	20	2845	2864	GCTGTTTTTGCCCATGCTCA	59.966	CGGGCCTCCAAGTACTGTTT	59.964	122
TRINITY_Contig5616	1	c	(GAC)4gtcgtcgaggtcgtcggcgaggaccaccgcccccagctccca(GCAGCC)3	71	174	244	CATCATACGCTCCTGCTCGT	59.969	AGAGGTGCTTCTTCGTCAGC	60.038	181
TRINITY_Contig5618	1	p3	(CCG)4	12	83	94	TTCTCCTCCTCAGCTCCTCC	60.033	GTCGAGCTCTTTACGGGGAG	59.899	115
TRINITY_Contig5619	1	p5	(TGTTT)4	20	651	670	GAGGATTTCCCAGCTGCACT	60.035	TGACGTCACGGTCATGTCTG	60.04	126
TRINITY_Contig5619	2	p3	(CTC)4	12	1448	1459	GAGGATTTCCCAGCTGCACT	60.035	TGACGTCACGGTCATGTCTG	60.04	126
TRINITY_Contig5620	1	p4	(TCGC)3	12	169	180	TCGACAAGGCCAGACGAATC	60.109	TTCCTCTGCCTTCCTTCCCT	59.883	237
TRINITY_Contig5633	1	p2	(CA)8	16	176	191	TTGCCCAGGAACTCGAAGTC	59.965	CATGCACATGAAGCTCGTCG	59.974	108
TRINITY_Contig5633	2	c	(CGC)5ccgcgtcgccgacagcgctgctagagtcgctatggcggcggccagcagcagccaccgcgcggccgccatcctctc(CT)6	102	1059	1160	TTGCCCAGGAACTCGAAGTC	59.965	CATGCACATGAAGCTCGTCG	59.974	108

TRINITY_Contig5634	1	p3	(GGC)4	12	507	518	GTCGTTCCTCTTGCCTTCCA	59.965	TGCCGTCTCTTCAGTTCACC	59.966	119
TRINITY_Contig5634	2	p2	(AG)7	14	1582	1595	GTCGTTCCTCTTGCCTTCCA	59.965	TGCCGTCTCTTCAGTTCACC	59.966	119
TRINITY_Contig5636	1	p5	(CCAAG)3	15	75	89	CGGAGGAACCTACCATACGC	59.968	TACAGACCAGGCGGAGTGTA	59.961	253
TRINITY_Contig5640	1	p3	(CTC)7	21	71	91	GTCGCGTACTCCTACTTCCG	59.973	CGTTGTGCCTGGTACTCTGT	59.966	221
TRINITY_Contig5640	2	p6	(CGCCGA)3	18	232	249	GTCGCGTACTCCTACTTCCG	59.973	CGTTGTGCCTGGTACTCTGT	59.966	221
TRINITY_Contig5642	1	p3	(TAG)4	12	329	340	GCCGGCGCATGTATTTGATT	59.969	ATGCCATGTATGACGCCGAT	59.965	110
TRINITY_Contig5642	2	p3	(CTT)6	18	1318	1335	GCCGGCGCATGTATTTGATT	59.969	ATGCCATGTATGACGCCGAT	59.965	110
TRINITY_Contig5644	1	p3	(AGG)5	15	1486	1500	GTAGAGCGTGAACTCCGAGG	59.901	CGCTGTCGTCGTATCGATCA	60.042	190
TRINITY_Contig5645	1	p3	(CGC)5	15	623	637	GCGGTATGATGCTCTCGACA	59.969	TGCAGAAACTCGCCCATCTT	59.963	147
TRINITY_Contig5646	1	p3	(AGG)7	21	205	225	TCTAGGAGCAAGCACACAGC	60.037	TCTCGTTCTCGGCGATGATG	59.971	253
TRINITY_Contig5646	2	p6	(GGAGCT)3	18	418	435	TCTAGGAGCAAGCACACAGC	60.037	TCTCGTTCTCGGCGATGATG	59.971	253
TRINITY_Contig5647	1	p3	(CCG)4	12	1907	1918	CATGCCTTGTAATGCTCCGC	59.97	CGCAGGCTTTTTATCCGCTC	59.972	197
TRINITY_Contig5648	1	p5	(AGTTG)3	15	70	84	CCCCAAAATCCATTGCTGGC	60.108	TGTCCATGGCTGAAGCTGAG	60.036	190
TRINITY_Contig5648	2	p5	(ATGAG)3	15	346	360	CCCCAAAATCCATTGCTGGC	60.108	TGTCCATGGCTGAAGCTGAG	60.036	190
TRINITY_Contig5648	3	p3	(GCG)4	12	1381	1392	CCCCAAAATCCATTGCTGGC	60.108	TGTCCATGGCTGAAGCTGAG	60.036	190
TRINITY_Contig5650	1	p3	(CCT)4	12	156	167	GCTGACTCCTTTGCATTGCC	60.109	TGAGTCCAGAAGGCAGCATG	60.036	209
TRINITY_Contig5651	1	p3	(CGG)4	12	331	342	ATTTAGGTGGCCTGCCTGAC	60.034	CGAGCAAGAAGTATGCACGC	59.974	154
TRINITY_Contig5651	2	p3	(TGG)4	12	4860	4871	ATTTAGGTGGCCTGCCTGAC	60.034	CGAGCAAGAAGTATGCACGC	59.974	154
TRINITY_Contig5652	1	p6	(CCGTCG)3	18	168	185	ATTTAGGTGGCCTGCCTGAC	60.034	CGAGCAAGAAGTATGCACGC	59.974	154
TRINITY_Contig5652	2	p3	(TGG)4	12	1158	1169	ATTTAGGTGGCCTGCCTGAC	60.034	CGAGCAAGAAGTATGCACGC	59.974	154
TRINITY_Contig5654	1	p3	(GCT)4	12	275	286	TAGCTGTTGTTGCTGCGAGA	59.967	ACCAGCTAGCCATGGTGAAC	60.035	167
TRINITY_Contig5656	1	p6	(GGGCGA)3	18	1530	1547	TACCGCTGCTGCAATTGAGA	60.036	TTTCTGGTATGTTGGCCCCC	59.96	225
TRINITY_Contig5657	1	p3	(AGC)4	12	1001	1012	TGGGAGTGAGATGGAGAGGG	60.032	AAGTGAGTTGCTACCCTGCC	59.963	205
TRINITY_Contig5658	1	c	(TGGAT)3ctgcacatctatctattctatctgttcacaggaataataataacccctgtgtatgtgctgctgccctgacctggctt(AGG)6gtgggtgctgggaatttctgttagggccaggagctcagggcaggagcaaacagatccctgc(AAAG)3	183	149	331	GCACACCTGCCAGTACTTCT	59.965	TGCACCAGATGGATTCCACC	60.034	236
TRINITY_Contig5660	1	p3	(CTC)4	12	341	352	TTGCTTGCTCCAGAGTCCAG	59.964	CCACTACGGGAGAGGAGGAA	60.033	103
TRINITY_Contig5661	1	p3	(CGC)4	12	1947	1958	TTTCACATGTTCCCCGAGGG	59.962	GCCAATGCCATGCTAACGAG	59.97	178
TRINITY_Contig5661	2	p3	(AGC)4	12	2145	2156	TTTCACATGTTCCCCGAGGG	59.962	GCCAATGCCATGCTAACGAG	59.97	178
TRINITY_Contig5662	1	p5	(CGCCT)3	15	140	154	CTGTATGTTGACATGCCGCG	59.973	AAGTTCCTCATGACGCCTGG	60.036	189
TRINITY_Contig5662	2	p6	(CCGCTC)3	18	258	275	CTGTATGTTGACATGCCGCG	59.973	AAGTTCCTCATGACGCCTGG	60.036	189
TRINITY_Contig5662	3	p3	(GCC)6	18	403	420	CTGTATGTTGACATGCCGCG	59.973	AAGTTCCTCATGACGCCTGG	60.036	189
TRINITY_Contig5662	4	p3	(CAG)4	12	3037	3048	CTGTATGTTGACATGCCGCG	59.973	AAGTTCCTCATGACGCCTGG	60.036	189
TRINITY_Contig5663	1	p4	(ACCG)3	12	1121	1132	GGGACCGCCAAGATTAAGCT	60.107	GGGGGTAAGTGCGATTGTCA	60.036	170
TRINITY_Contig5665	1	p3	(CTC)5	15	536	550	CAAGAGGCCAGCAAACACAC	59.969	ACGAGGAGGAGGAGGAGAAC	60.034	135
TRINITY_Contig5665	2	p3	(CAG)4	12	674	685	CAAGAGGCCAGCAAACACAC	59.969	ACGAGGAGGAGGAGGAGAAC	60.034	135
TRINITY_Contig5666	1	p3	(CCG)4	12	177	188	AGAACAAGAGGAAGGCAGCC	59.963	GGTCCATCACGTAGTAGCCG	59.97	150
TRINITY_Contig5666	2	c*	(CAGAG)2(C<AGAG>AGAGAGAGAGCCCTTCTTCCTCCTCCCCGGCCGCCGGCGGCGAGAGATCGACCAGCGAGCGAGATGGTGCTGTCCCACGGCGTCAGCGGCTCCGATGAGTCGGTGCACTCGACGTTCGCGTCCCGCTACGTCCGCGCCTCCCTCCCCAGGTTCCGGATGCCGGAGCAGTCGATCCCGAAGGAGGCGGCGTACCAGATCATCAACGACGAGCTGATGCTGGACGGGAACCCGCGGCTGAACCTGGCGTCCTTCGTCACCACGTGGATGGAGCCCGAGTGCGACAAGCTCATCAAGGCCGCCATCAACAAGAACTACGTCGACATGGACGAGTACCCCGTCACCACCGAGCTCCAGAACCGGTGCGTGAACATCATCGCGCAGCTGTTCAACGCCCCGCTCGGCGACGCCGAAAAGGCCGTCGG	25	341	365	AGAACAAGAGGAAGGCAGCC	59.963	GGTCCATCACGTAGTAGCCG	59.97	150
TRINITY_Contig5668	1	p3	(GTC)4	12	80	91	GTTGGTGGGTGTCATTGTGC	59.968	TGGACAGGAAAGACGAACCG	59.967	192
TRINITY_Contig5668	2	c*	(GTG)3(GTGTGGTGTGGTTGGTGTGACGATGGGGATGGGATGCTCCGATCCTTTTCGTACGTGTCGGCACTGTCGGTTGGGTGGTGTTGGTGGGTGTCATTGTGCGTGCGTGTCCGTGTCCCGATCTGGCGTGCAGCGGCACGGCCGTGTGGCCTGTGGCCATGCTGGCATGCTGCGCACTCCAGCGTCGGGCACCACGGCTGAATAAAGAAAGGATTTTCCATGGATTTTCAGGATTTGTGTTACAGCAGCGGGTTCGGTTCGTCTTTCCTGTCCAGACCATGGCCAAGGGGGATCGGACAGACCTAGGTGATCCACGTAT<GTGGTG>TGGTGTGGTTGGTGTGACGATGGGGATGGGATGCTCCGATCCTTTTCGTACGTGTCGGCACTGTCGGTTGGGTGGTGTTGGTGGGTGTCATTGTGCGTGCGTGTCC	21	799	819	GTTGGTGGGTGTCATTGTGC	59.968	TGGACAGGAAAGACGAACCG	59.967	192
TRINITY_Contig5669	1	p4	(TGCA)3	12	2164	2175	CTGGCTGCTACTTCGGTTGA	60.037	CCATGCATCATCCAGTTGCG	59.97	261
TRINITY_Contig5671	1	c*	(ATCC)4agcacctctcctctgctcctcatgcaaaagcaaaacgccctcccca(CTC)4(TCCTCT)3*tctctcccaatcca(AATCCC)3aataaaaaggcaa(GCAG)4cctctacctagctagcttcgttcaccccacacgcacaaaacagagactactcttccctag(CCTT)4	224	177	400	ATCCATCCAGCACCTCTCCT	60.03	CTCTGTTTTGTGCGTGTGGG	59.971	185
TRINITY_Contig5671	2	p3	(CCG)4	12	793	804	ATCCATCCAGCACCTCTCCT	60.03	CTCTGTTTTGTGCGTGTGGG	59.971	185
TRINITY_Contig5671	3	p3	(CGC)4	12	980	991	ATCCATCCAGCACCTCTCCT	60.03	CTCTGTTTTGTGCGTGTGGG	59.971	185
TRINITY_Contig5675	1	p3	(CGC)4	12	3015	3026	GGCCATGGTATTTCCCCACA	60.033	CGGTGAAGTGCAACAACAGG	59.971	201
TRINITY_Contig5675	2	p6	(CCGCTG)3	18	3383	3400	GGCCATGGTATTTCCCCACA	60.033	CGGTGAAGTGCAACAACAGG	59.971	201
TRINITY_Contig5676	1	p3	(GCC)4	12	2944	2955	TGGATTTCCTCAGCTGCTGG	60.035	GCTGAGATTGAGCAGGTGGT	60.036	222
TRINITY_Contig5678	1	p3	(CAC)4	12	310	321	TCCCGGACTGATGCTCACTA	60.034	TACCGGGGAGGATGCTTACA	60.032	188
TRINITY_Contig5679	1	c	(ATCC)4attgctcgtctccctcccaaattcgatgcctttgtagcgctgccgcttcgcccctgcgcctgcgt(CGCGC)3	96	92	187	TGCTGGAGGACATCGTCAAC	60.037	GCTCGCGTGTACATCTCGTA	59.973	156
TRINITY_Contig5679	2	p3	(GAC)4	12	575	586	TGCTGGAGGACATCGTCAAC	60.037	GCTCGCGTGTACATCTCGTA	59.973	156
TRINITY_Contig5680	1	p4	(TCAC)3	12	873	884	AGCTCGCACGACAAGAAGAA	59.968	GCCTTCCTCCTCTGCAACAT	60.035	273
TRINITY_Contig5680	2	p3	(CTC)6	18	2051	2068	AGCTCGCACGACAAGAAGAA	59.968	GCCTTCCTCCTCTGCAACAT	60.035	273
TRINITY_Contig5680	3	p3	(CGC)4	12	2260	2271	AGCTCGCACGACAAGAAGAA	59.968	GCCTTCCTCCTCTGCAACAT	60.035	273
TRINITY_Contig5681	1	p4	(ACCG)3	12	28	39	CATCGCTCATGTGAAGCAGC	59.972	TTTGCCAGGGTGGTGACTTT	60.033	189
TRINITY_Contig5681	2	p2	(AG)7	14	2251	2264	CATCGCTCATGTGAAGCAGC	59.972	TTTGCCAGGGTGGTGACTTT	60.033	189
TRINITY_Contig5682	1	p3	(CGC)4	12	248	259	CGCTGAGAACGTGTTTGCAA	59.973	ACCCCTCTGCTCTCATTTGC	60.035	131
TRINITY_Contig5682	2	p5	(GGGTC)4	20	3706	3725	CGCTGAGAACGTGTTTGCAA	59.973	ACCCCTCTGCTCTCATTTGC	60.035	131
TRINITY_Contig5682	3	p3	(GCC)4	12	3945	3956	CGCTGAGAACGTGTTTGCAA	59.973	ACCCCTCTGCTCTCATTTGC	60.035	131
TRINITY_Contig5685	1	p4	(AAAG)3	12	1529	1540	TCTCTATCTCCACCCACGCA	60.034	CATCCTTGAGCCGGACTCTC	59.896	278
TRINITY_Contig5686	1	c	(CCG)4tagtagtcgtcggaattggggtagtacttgtaggtcggggagaaggcgatgtcgccctcgctccagccccggaacg(CGC)4	100	930	1029	GCAGCCGTAGTTCTCTTGGT	60.037	CAGCTCAAGGGGAACGGATT	60.035	255
TRINITY_Contig5686	2	p4	(GCCG)3	12	2076	2087	GCAGCCGTAGTTCTCTTGGT	60.037	CAGCTCAAGGGGAACGGATT	60.035	255
TRINITY_Contig5690	1	c	(GGTG)3cggtggctagctggctggtggt(GGC)4	46	1425	1470	CTGCCGAAAACAGCTATCGC	59.973	AGCATTGCAGGTGAGATGCT	60.034	121
TRINITY_Contig5690	2	p3	(GCG)5	15	1617	1631	CTGCCGAAAACAGCTATCGC	59.973	AGCATTGCAGGTGAGATGCT	60.034	121
TRINITY_Contig5692	1	c*	(CGGC)4agcgggtgccgcgccggggcggtg(GGCT)3(TGGCT)3*	62	2109	2170	GCGCGCTCATGAATCACAAT	59.971	CTTTAGCCAGCCGAGGAACA	60.036	132
TRINITY_Contig5693	1	p3	(AAG)6	18	208	225	AGAAGAAGAAGGGGTGCTGC	59.963	TCCGATCCACCTCAGGCTAA	60.033	215
TRINITY_Contig5694	1	c	(CGC)4gttgcccccacg(GCC)4	36	279	314	TGTCATGCAAGGCAAACAGC	59.968	CGGTATTCAAGCCCACTGGT	60.035	189
TRINITY_Contig5694	2	p3	(GAA)4	12	572	583	TGTCATGCAAGGCAAACAGC	59.968	CGGTATTCAAGCCCACTGGT	60.035	189
TRINITY_Contig5694	3	p4	(TTTA)3	12	1719	1730	TGTCATGCAAGGCAAACAGC	59.968	CGGTATTCAAGCCCACTGGT	60.035	189

TRINITY_Contig5695	1	p3	(CAC)5	15	244	258	TGCATTGGACGAGCTTGGAT	60.035	ACGCAGATCATAGAGCAGGC	59.967	137
TRINITY_Contig5695	2	p4	(AAAG)3	12	1467	1478	TGCATTGGACGAGCTTGGAT	60.035	ACGCAGATCATAGAGCAGGC	59.967	137
TRINITY_Contig5697	1	p6	(CGCCGG)3	18	258	275	GGAGGGAAGGGAATGGTTGG	60.033	TGCGCACCTGTTGAACAATG	59.969	111
TRINITY_Contig5697	2	p3	(CCT)4	12	425	436	GGAGGGAAGGGAATGGTTGG	60.033	TGCGCACCTGTTGAACAATG	59.969	111
TRINITY_Contig5697	3	p2	(GA)9	18	3232	3249	GGAGGGAAGGGAATGGTTGG	60.033	TGCGCACCTGTTGAACAATG	59.969	111
TRINITY_Contig5698	1	p4	(GCAA)3	12	1	12	CCACCAACGAAACCTTGCAG	59.969	CTTCCTTGGCTTGCGCTTTT	59.967	173
TRINITY_Contig5698	2	p5	(CCTCC)3	15	221	235	CCACCAACGAAACCTTGCAG	59.969	CTTCCTTGGCTTGCGCTTTT	59.967	173
TRINITY_Contig5698	3	p6	(GATGAA)5	30	3017	3046	CCACCAACGAAACCTTGCAG	59.969	CTTCCTTGGCTTGCGCTTTT	59.967	173
TRINITY_Contig5699	1	p3	(GCT)4	12	155	166	GTGAAAATTCCGATCGGCCG	59.972	CTGATTCGGATCGGTGGAGG	59.967	155
TRINITY_Contig5699	2	p6	(CTGCTC)3	18	2268	2285	GTGAAAATTCCGATCGGCCG	59.972	CTGATTCGGATCGGTGGAGG	59.967	155
TRINITY_Contig5701	1	c	(CGG)4ccatcgctggcggcgcacgtggt(CGG)5	50	1087	1136	CCATTGCGCAATAAGGCTCC	59.969	GCAAGAGGTCACTGGAGTCC	60.037	275
TRINITY_Contig5703	1	p3	(CGG)4	12	343	354	CTTCTTCGCGTCGATCGAGA	59.974	CTAGCCATCCATCGTTCCCC	59.965	210
TRINITY_Contig5704	1	p3	(GCA)4	12	350	361	GGTGCCGACCAAAGAAACAC	59.97	CTGGTGGATTGGTGGCATCT	60.034	269
TRINITY_Contig5705	1	p3	(GAG)4	12	250	261	TGTGCTTTTCTTTGCTCGGC	59.969	GGAGGGGAAGGGAGGAAGAT	60.03	119
TRINITY_Contig5705	2	p3	(CTC)5	15	3942	3956	TGTGCTTTTCTTTGCTCGGC	59.969	GGAGGGGAAGGGAGGAAGAT	60.03	119
TRINITY_Contig5705	3	p3	(GCC)4	12	4185	4196	TGTGCTTTTCTTTGCTCGGC	59.969	GGAGGGGAAGGGAGGAAGAT	60.03	119
TRINITY_Contig5705	4	p3	(CCT)4	12	4439	4450	TGTGCTTTTCTTTGCTCGGC	59.969	GGAGGGGAAGGGAGGAAGAT	60.03	119
TRINITY_Contig5707	1	c	(CCCT)4tcctcccttccccctgagccgcgg(CCCT)3	52	247	298	ACAATCTGGAGAAGGCTGGC	60.035	AGGCGAATACTAAGAGGCGC	59.968	160
TRINITY_Contig5708	1	p3	(GCG)5	15	249	263	GTCGTACTCCCCATGGCAAA	60.036	GCTCAAACTCCCCTGCTTCT	59.963	108
TRINITY_Contig5709	1	p3	(GGA)4	12	2471	2482	TGGGAAGCAGTATGAGCAGC	60.108	CTGCAAAAGGGGCTGCATTT	59.964	155
TRINITY_Contig5709	2	p4	(GCTA)4	16	2606	2621	TGGGAAGCAGTATGAGCAGC	60.108	CTGCAAAAGGGGCTGCATTT	59.964	155
TRINITY_Contig5710	1	p4	(CGCC)4	16	49	64	CCTAAGGGGGCTAAGGAGGT	60.03	AGCTGGACTCTCACAAACGG	59.966	180
TRINITY_Contig5711	1	p4	(CTCC)3	12	726	737	TCCAGCGATGCTTTGTCCAT	60.035	TGTTCGTTCGGTGGAGAAGG	59.967	125
TRINITY_Contig5713	1	p3	(CGG)5	15	148	162	GGCCACATGACTCTGACACA	59.965	AACCAGGTCATTGCACGCTA	59.964	246
TRINITY_Contig5714	1	p3	(GCG)4	12	3589	3600	ATGAACACGGCCCATCCATT	60.033	TTTCCACACTGGCGGCATAT	60.035	110
TRINITY_Contig5716	1	p3	(CTA)4	12	110	121	CCACCAAGCAACAACGTCTG	59.971	AGCCTATGACAGCCTCCTCA	60.032	126
TRINITY_Contig5716	2	p2	(TC)10	20	496	515	CCACCAAGCAACAACGTCTG	59.971	AGCCTATGACAGCCTCCTCA	60.032	126
TRINITY_Contig5717	1	c	(GGCG)3agcgcgagcgagcggacgaacggcgtagggctggccggagagattaggcgggc(AGGG)3	77	758	834	TCAGCTTCGCGGATCATCTC	59.969	CTTCCCCGAGTTCCTCAACC	60.036	195
TRINITY_Contig5718	1	p3	(GAC)4	12	1880	1891	TATCACTCGCGACGAAGCTC	59.972	CATGTGGAAGGTGGTCGACA	59.965	161
TRINITY_Contig5718	2	p3	(GCG)5	15	2031	2045	TATCACTCGCGACGAAGCTC	59.972	CATGTGGAAGGTGGTCGACA	59.965	161
TRINITY_Contig5718	3	p6	(GCCTGA)6	36	2319	2354	TATCACTCGCGACGAAGCTC	59.972	CATGTGGAAGGTGGTCGACA	59.965	161
TRINITY_Contig5719	1	p3	(CGC)5	15	424	438	CAAACACCACTCAAAGGGCG	59.969	CCTCCTCAGATGGACCAGGA	60.032	266
TRINITY_Contig5720	1	p3	(CGC)6	18	365	382	CAGGTCGTCGTAGTAGTCGC	59.974	AAGGCGACGACCAAGAAGAG	60.038	159
TRINITY_Contig5720	2	p3	(GCC)4	12	668	679	CAGGTCGTCGTAGTAGTCGC	59.974	AAGGCGACGACCAAGAAGAG	60.038	159
TRINITY_Contig5723	1	p3	(GCC)4	12	502	513	CCTCGATCGATGGTGGTCAG	59.969	AGCTCATCGCCAAGAACCTC	60.108	105
TRINITY_Contig5724	1	p3	(CGG)5	15	1806	1820	AGGCAGTAGTGGTGAGTTGC	59.965	ACCTCTTCAAGATGCTGGCC	60.035	106
TRINITY_Contig5727	1	p3	(CGC)4	12	1343	1354	CCCCGCAGTCACAACAAAAG	59.969	CTCGACAGCTCTACGTCGAC	59.975	252
TRINITY_Contig5728	1	p3	(CTC)4	12	3437	3448	AGGTCTATGGCTGGCAACAC	60.035	TGTCCCAGCAAAGGTTGTGT	60.034	160
TRINITY_Contig5731	1	p3	(GGC)4	12	101	112	CTGCCGATAAGTGCAATGCC	59.97	TCGTTGCACATCGGTTCTGA	59.967	162
TRINITY_Contig5732	1	c	(GCGGC)3gccatcgtaagctaactgcctacctaacctaccaccacccggttggtccgcgtcggcgtgtgggtgtggcgcttggctgctgctcggctgcc(CGTGCT)4	131	1689	1819	GTTCGTTGACCTGATCGGGT	60.038	CACAACAAGAACCAGGTGCG	59.971	149
TRINITY_Contig5733	1	p3	(TCG)5	15	4568	4582	TCGTCGTCATGGTACTCCCT	60.035	GATGATGACGACGACGACGA	59.974	194
TRINITY_Contig5733	2	p6	(GTAGGA)3	18	5179	5196	TCGTCGTCATGGTACTCCCT	60.035	GATGATGACGACGACGACGA	59.974	194
TRINITY_Contig5736	1	p6	(CCTCCA)4	24	100	123	CTTCCGGAAGTCCAGCAACT	59.965	TTCTTTTGACAGCGCCTTGC	59.969	105
TRINITY_Contig5736	2	p6	(GGCTCC)3	18	541	558	CTTCCGGAAGTCCAGCAACT	59.965	TTCTTTTGACAGCGCCTTGC	59.969	105
TRINITY_Contig5737	1	p4	(GCCC)3	12	252	263	GCTCATGTGGACAAGCAACG	60.11	GTACCGCTCATCTCTGACCG	59.97	158
TRINITY_Contig5737	2	p4	(GCTG)3	12	400	411	GCTCATGTGGACAAGCAACG	60.11	GTACCGCTCATCTCTGACCG	59.97	158
TRINITY_Contig5738	1	p3	(AGG)5	15	511	525	TCCTCTCCCTACTCTGCCAC	60.033	CTCCTCCTCCTCCTGCTTCT	60.032	271
TRINITY_Contig5740	1	p3	(CTG)6	18	668	685	ACCAGTGCGCACTTATTCCA	59.964	AGGCTGAACACGAACACGAT	59.966	137
TRINITY_Contig5740	2	p3	(CCG)4	12	1090	1101	ACCAGTGCGCACTTATTCCA	59.964	AGGCTGAACACGAACACGAT	59.966	137
TRINITY_Contig5744	1	p3	(CCG)5	15	199	213	GATCAGTCAAACGCGCATCC	59.973	CAGAACCTGCTGTCTGCTCA	59.966	136
TRINITY_Contig5744	2	p3	(TGG)4	12	3348	3359	GATCAGTCAAACGCGCATCC	59.973	CAGAACCTGCTGTCTGCTCA	59.966	136
TRINITY_Contig5744	3	p4	(AAAG)3	12	3986	3997	GATCAGTCAAACGCGCATCC	59.973	CAGAACCTGCTGTCTGCTCA	59.966	136
TRINITY_Contig5744	4	p3	(AAG)4	12	5887	5898	GATCAGTCAAACGCGCATCC	59.973	CAGAACCTGCTGTCTGCTCA	59.966	136
TRINITY_Contig5744	5	p5	(TTTTG)3	15	7046	7060	GATCAGTCAAACGCGCATCC	59.973	CAGAACCTGCTGTCTGCTCA	59.966	136
TRINITY_Contig5746	1	p3	(GCG)5	15	456	470	ACCCACCAGCGAATCTCAAG	60.036	CAAGGGATTCGGTTGGCTCT	60.035	188
TRINITY_Contig5750	1	p3	(CGC)5	15	671	685	CAATTTCAGCAGCGGATCGG	59.971	AAGTTCTGGCCGACGATGTT	59.965	234
TRINITY_Contig5750	2	p3	(CGA)4	12	1474	1485	CAATTTCAGCAGCGGATCGG	59.971	AAGTTCTGGCCGACGATGTT	59.965	234
TRINITY_Contig5751	1	p4	(TCGG)3	12	2671	2682	TTTCCCTTAGCATGGGCCAG	60.033	GGCGTCGATACTGAAGGAG	59.969	252
TRINITY_Contig5752	1	p4	(ACAG)3	12	141	152	CGACAGGATCTGATCGCGAA	59.971	GCACTTCAGCTCGTCCTCTT	60.038	214
TRINITY_Contig5752	2	c	(GAG)4ccccggtcgccggcgccggcga(GCG)4	46	1389	1434	CGACAGGATCTGATCGCGAA	59.971	GCACTTCAGCTCGTCCTCTT	60.038	214
TRINITY_Contig5756	1	p3	(TCA)4	12	2674	2685	AAGCAGCTACAAGGAGGCAG	60.036	ACCACACAAGGAAGAGCGAG	59.966	268
TRINITY_Contig5758	1	p4	(TCGA)3	12	127	138	GAAGGAGGTGTACTTGGCCC	60.035	CCGTGATCCTCTCGTACGTG	59.972	182
TRINITY_Contig5758	2	c	(CGCC)4ggcgccgcggatctcggttcgccgcggtc(GCCG)3	57	282	338	GAAGGAGGTGTACTTGGCCC	60.035	CCGTGATCCTCTCGTACGTG	59.972	182
TRINITY_Contig5758	3	p3	(CGG)4	12	1777	1788	GAAGGAGGTGTACTTGGCCC	60.035	CCGTGATCCTCTCGTACGTG	59.972	182
TRINITY_Contig5758	4	p3	(CGC)5	15	2276	2290	GAAGGAGGTGTACTTGGCCC	60.035	CCGTGATCCTCTCGTACGTG	59.972	182
TRINITY_Contig5759	1	p2	(GA)6	12	362	373	CAAAGGCAGTCAAACCACGG	59.969	AGTAGAAGTAGGAGGCGGCA	60.033	205
TRINITY_Contig5759	2	p3	(CCG)4	12	854	865	CAAAGGCAGTCAAACCACGG	59.969	AGTAGAAGTAGGAGGCGGCA	60.033	205
TRINITY_Contig5762	1	p3	(CGG)4	12	57	68	ATGACACACGGCCATTCGAT	60.108	CTTTGTCGCAGCATGTCAGG	59.833	221
TRINITY_Contig5765	1	p2	(AG)6	12	98	109	CCCTTGTGGATAGCACCGTT	60.035	AATGGCGTGCCTTTCAACAC	59.968	231
TRINITY_Contig5766	1	p3	(TCA)4	12	2378	2389	GAACCTGTTCAGGACGCTGA	59.966	AACTTGGCGTCAGTCTCTGG	59.966	162
TRINITY_Contig5766	2	c	(GGA)5tgctggccctggtcgccatgcccat(CG)6tcccaccg(CGGC)3	72	3693	3764	GAACCTGTTCAGGACGCTGA	59.966	AACTTGGCGTCAGTCTCTGG	59.966	162
TRINITY_Contig5768	1	p3	(CGC)4	12	316	327	TCCCCTCCTCTCGTCGTTAG	60.107	CGCCCTTCTATCGCCATCTT	59.966	171
TRINITY_Contig5769	1	p3	(CAC)5	15	423	437	TCATCAGCAAACGGAGTCCC	60.036	TGAACAGGAACACCGAGTGG	59.894	196
TRINITY_Contig5769	2	p3	(TCG)4	12	1055	1066	TCATCAGCAAACGGAGTCCC	60.036	TGAACAGGAACACCGAGTGG	59.894	196
TRINITY_Contig5775	1	p3	(GGC)5	15	172	186	GTGCGTCAAGGTTGCACATT	59.969	AGTGATTCGAAGGCTTGGGG	60.035	113
TRINITY_Contig5777	1	p4	(AAAT)3	12	431	442	TTTTGCTGTGCGTGTGCTAC	59.971	CGTTTCTCCGCAGCAAAACA	59.971	137
TRINITY_Contig5778	1	p3	(CCG)5	15	114	128	TCAGATCGAGAGGGAACGGT	60.034	GGAATTGGGGTGATGGGGAG	60.106	243
TRINITY_Contig5779	1	p3	(CCG)6	18	177	194	TGGTAGGGCAGCAAATGGAG	60.034	AGTTGCCTTAGGGTCCATGC	60.034	232
TRINITY_Contig5780	1	p5	(CCAGA)3	15	1	15	GAGGAGGCCCTACTCTGGAA	60.032	AGAGAGCATGGCAACTGGAC	60.036	165
TRINITY_Contig5781	1	p2	(CG)6	12	582	593	GTCCCCTGCTTCCTGAAGAC	60.036	ACAGTGTCCATGCTCGACTG	60.038	138
TRINITY_Contig5781	2	p3	(TGC)4	12	906	917	GTCCCCTGCTTCCTGAAGAC	60.036	ACAGTGTCCATGCTCGACTG	60.038	138
TRINITY_Contig5782	1	p3	(CGG)5	15	2626	2640	TCTCGTTTCTTGAGTGCCCC	59.965	CGCTGTGGTTTGACTGGTTG	59.971	248
TRINITY_Contig5782	2	c	(CCT)5ctgcctttatggctgtggccgccgtcaaaccagccgcgttt(CTTC)3	68	3001	3068	TCTCGTTTCTTGAGTGCCCC	59.965	CGCTGTGGTTTGACTGGTTG	59.971	248
TRINITY_Contig5784	1	p3	(CGC)4	12	516	527	ACGTACGGCCGTTACAGTTT	59.968	ACACACACAGCTTATCCGCA	59.965	223
TRINITY_Contig5784	2	p5	(GCGCC)3	15	856	870	ACGTACGGCCGTTACAGTTT	59.968	ACACACACAGCTTATCCGCA	59.965	223
TRINITY_Contig5784	3	p3	(GGC)4	12	1209	1220	ACGTACGGCCGTTACAGTTT	59.968	ACACACACAGCTTATCCGCA	59.965	223
TRINITY_Contig5785	1	p3	(TCC)4	12	415	426	ATCCCCCAGAACTCCTACCC	60.03	TTGATCTGCCAGCTGAAGGG	60.035	204
TRINITY_Contig5785	2	c	(CCT)4cgccgcgcgcgccctcttgttccgttgctcgtcgctgcg(CT)20cccccctccgccttggaggtcgcgttggttcgtgcccgttgctggctctgtgctctggctcggcggttttct(CTCTC)3	177	3330	3506	ATCCCCCAGAACTCCTACCC	60.03	TTGATCTGCCAGCTGAAGGG	60.035	204
TRINITY_Contig5786	1	p5	(ATCCC)3	15	398	412	TCGCGGGCAGATAAGATTCC	59.966	GCTGAGGCTGGAAATCAGGT	60.035	145
TRINITY_Contig5786	2	p6	(ATGCTG)3	18	523	540	TCGCGGGCAGATAAGATTCC	59.966	GCTGAGGCTGGAAATCAGGT	60.035	145
TRINITY_Contig5787	1	p3	(CCT)4	12	2282	2293	TGCATCTCCTCCTGCCTAGT	60.032	ACCTAGGAAAAAGCGGCGAA	59.964	258
TRINITY_Contig5789	1	p6	(GAGACA)3	18	3554	3571	CAGCATCCGTCTCTGGATCC	59.967	GGAGCCAGCTCTCGTATGAC	59.969	105
TRINITY_Contig5790	1	p3	(TGC)5	15	681	695	CCGTTGGAGTCGTGGAGAAA	59.967	GCTTGGACACGTACCACTGA	59.967	193
TRINITY_Contig5791	1	p3	(GGC)4	12	1532	1543	ATCATCCCCAGCACCAACAG	60.034	CGAGGAGCTGGTGGCATTAT	59.893	207
TRINITY_Contig5791	2	p3	(GGC)4	12	1834	1845	ATCATCCCCAGCACCAACAG	60.034	CGAGGAGCTGGTGGCATTAT	59.893	207
TRINITY_Contig5792	1	p6	(GCCGCG)3	18	4069	4086	AGCTAGAGCCACCCTAAGCT	60.032	CGCTGCTTGACGTCATGATG	59.974	147
TRINITY_Contig5792	2	p3	(TGG)4	12	4223	4234	AGCTAGAGCCACCCTAAGCT	60.032	CGCTGCTTGACGTCATGATG	59.974	147
TRINITY_Contig5793	1	c	(CCCGCC)3gccgcg(GCA)4	36	4448	4483	CCCACTGATCAGGTGTCCAC	60.036	GATGATTCGGCGTCAAAGGC	59.972	176
TRINITY_Contig5796	1	p3	(CGC)4	12	720	731	GCGCTATCACCCACCGATAA	59.967	CAGCGAGTGGTAAACAGGGT	59.965	133
TRINITY_Contig5799	1	p3	(ATC)5	15	1339	1353	CAAGTTTGTGGGCGCGTTTA	59.97	GGAACCGTTGCACTGTTCAC	59.971	213

TRINITY_Contig5799	2	p3	(GCC)7	21	2414	2434	CAAGTTTGTGGGCGCGTTTA	59.97	GGAACCGTTGCACTGTTCAC	59.971	213
TRINITY_Contig5801	1	p3	(GCC)6	18	1498	1515	CAAATGTTGCGGCAACCAGA	59.968	GTGATTCAGGAGCACAGCCT	60.036	123
TRINITY_Contig5802	1	p5	(GTGGA)3	15	301	315	CGGGATATTGAGTCGCTGCT	59.967	GGCAACTCAGCAACTTGGTG	59.969	215
TRINITY_Contig5803	1	p3	(CAT)5	15	714	728	ATCCCAGCATGGTCCAGTTG	60.034	GTGTGATCCTCCTCCCCTCT	60.032	260
TRINITY_Contig5804	1	c	(GGC)4gcggtggacggcatcttgagcgccatggcgtcggcatcggcgcgctcccacgg(GGC)4	77	1698	1774	ACAACACCTACGACGTCCAC	59.969	CTTGGGGTTGAGGTCCTTCC	59.962	260
TRINITY_Contig5804	2	p3	(CGC)4	12	1968	1979	ACAACACCTACGACGTCCAC	59.969	CTTGGGGTTGAGGTCCTTCC	59.962	260
TRINITY_Contig5804	3	p3	(GCG)4	12	2166	2177	ACAACACCTACGACGTCCAC	59.969	CTTGGGGTTGAGGTCCTTCC	59.962	260
TRINITY_Contig5804	4	p3	(GCC)4	12	2337	2348	ACAACACCTACGACGTCCAC	59.969	CTTGGGGTTGAGGTCCTTCC	59.962	260
TRINITY_Contig5804	5	c	(GCG)4gaggcctcagaggtgtctccggcagacgccgcactggggcaggcgcagttcgagaaggtggcgagcatgctgaagctgccggagg(AGC)4	109	2940	3048	ACAACACCTACGACGTCCAC	59.969	CTTGGGGTTGAGGTCCTTCC	59.962	260
TRINITY_Contig5806	1	p4	(AGCG)3	12	980	991	CAAAAAGGCCTGTCCACAGC	59.967	CGGATCTGGTGTTCTGGCTT	60.036	183
TRINITY_Contig5806	2	p4	(CGAG)3	12	1353	1364	CAAAAAGGCCTGTCCACAGC	59.967	CGGATCTGGTGTTCTGGCTT	60.036	183
TRINITY_Contig5808	1	c	(GCC)4gcgccaccggcgcct(CGC)5	42	179	220	TTCGCATCGTCCTCCCAAAA	59.964	CGGGGGATTGGGTGGATTAG	59.89	110
TRINITY_Contig5810	1	p3	(CGG)4	12	2094	2105	GGCCCTCAGGTTTTGCTTTG	59.966	TCACCACCAAAGATCCAGCC	59.962	118
TRINITY_Contig5811	1	p4	(GCCG)3	12	397	408	CTAGAGGTAGGGGCCACAGT	60.032	GGGGCAGTAATCAGCACAGT	60.035	246
TRINITY_Contig5811	2	c	(GGT)4tg(GT)6	26	1763	1788	CTAGAGGTAGGGGCCACAGT	60.032	GGGGCAGTAATCAGCACAGT	60.035	246
TRINITY_Contig5811	3	p3	(TTA)4	12	2491	2502	CTAGAGGTAGGGGCCACAGT	60.032	GGGGCAGTAATCAGCACAGT	60.035	246
TRINITY_Contig5811	4	p4	(CTAT)3	12	2636	2647	CTAGAGGTAGGGGCCACAGT	60.032	GGGGCAGTAATCAGCACAGT	60.035	246
TRINITY_Contig5814	1	p3	(CGG)7	21	710	730	ACTCCCCTAGTGGATGCCAT	60.03	TAAAGATCGACCGCCGGATG	59.968	149
TRINITY_Contig5814	2	p3	(GCG)4	12	1144	1155	ACTCCCCTAGTGGATGCCAT	60.03	TAAAGATCGACCGCCGGATG	59.968	149
TRINITY_Contig5815	1	c	(CAGGCG)4gagccctcgacgccgacgtcggaggcgaccaggctgcgggcgccggcggcgtcgcccgcggcgggaggagaggatca(GGC)4	113	288	400	GCTTGCAGGCACGGTAAAAA	59.969	TCTCCTCATCCTCACCCCTG	60.032	119
TRINITY_Contig5816	1	p3	(TGC)5	15	790	804	GTCACGTTTGATATGGCGCC	59.972	CCACCAACAAGAAGCACGTG	59.971	181
TRINITY_Contig5816	2	p5	(TTTTC)3	15	2374	2388	GTCACGTTTGATATGGCGCC	59.972	CCACCAACAAGAAGCACGTG	59.971	181
TRINITY_Contig5817	1	p3	(AAG)5	15	791	805	TAGCGCCAAAATCCCCCATT	60.033	CATCGCAGAGTTCATGCACG	59.974	127
TRINITY_Contig5819	1	p2	(CT)8	16	2571	2586	CTGAAAGTCGAGGGTGGCAT	60.036	GAACTGTACCCTTGCGTCA	59.966	220
TRINITY_Contig5821	1	p4	(TGAT)3	12	225	236	CCCTGCTCAATTCCGGTTCT	60.035	CTTCGGAGCCCCCAGAAAAT	60.034	207
TRINITY_Contig5823	1	c	(GCCG)3ggtcagcagcgagcactagccgccgcctagcccccggcgcggctcttcgagggcgatcgaggcagccgca(CG)7	96	617	712	GTTCCGCCCTGCAATTGTTT	59.967	ATTACCGGACAAGGTGCGTT	59.964	102
TRINITY_Contig5823	2	p3	(GTG)4	12	1045	1056	GTTCCGCCCTGCAATTGTTT	59.967	ATTACCGGACAAGGTGCGTT	59.964	102
TRINITY_Contig5824	1	p3	(CCT)4	12	89	100	GGCCTTCCTCATCGGATCTG	59.965	GTAGTTCACCGCTCCACCAA	59.966	142
TRINITY_Contig5824	2	p3	(GGC)4	12	819	830	GGCCTTCCTCATCGGATCTG	59.965	GTAGTTCACCGCTCCACCAA	59.966	142
TRINITY_Contig5825	1	p3	(CTT)4	12	310	321	TGCAAACTCGTTGAACAGCG	59.973	CATGCGATGATTGTGCTGGG	59.97	101
TRINITY_Contig5825	2	p3	(GAC)4	12	488	499	TGCAAACTCGTTGAACAGCG	59.973	CATGCGATGATTGTGCTGGG	59.97	101
TRINITY_Contig5828	1	p4	(AACG)4	16	21	36	TTTGGTGGTGTGCCTCTGTT	60.034	CGAACAGGAACAGAGCTGGT	59.966	139
TRINITY_Contig5828	2	p3	(TCG)4	12	411	422	TTTGGTGGTGTGCCTCTGTT	60.034	CGAACAGGAACAGAGCTGGT	59.966	139
TRINITY_Contig5828	3	p2	(GA)6	12	741	752	TTTGGTGGTGTGCCTCTGTT	60.034	CGAACAGGAACAGAGCTGGT	59.966	139
TRINITY_Contig5828	4	p3	(GCT)6	18	895	912	TTTGGTGGTGTGCCTCTGTT	60.034	CGAACAGGAACAGAGCTGGT	59.966	139
TRINITY_Contig5829	1	p5	(CGCCG)3	15	1500	1514	AGATGTGGCGCTTCATTCCA	60.035	TGCAGAGGACGTTCCATTCC	60.036	228
TRINITY_Contig5830	1	p3	(GCC)5	15	919	933	GCCAGATCGTTCACCTTCCA	60.036	GGCCAATGCGTCAGTTGTTT	59.968	170
TRINITY_Contig5830	2	p5	(GGGGT)4	20	1715	1734	GCCAGATCGTTCACCTTCCA	60.036	GGCCAATGCGTCAGTTGTTT	59.968	170
TRINITY_Contig5831	1	p2	(GA)8	16	251	266	AGCACCAAACTTTCCACCCA	60.033	CGTGTATGCGCGTTCGAATT	59.976	177
TRINITY_Contig5831	2	p3	(CGG)6	18	471	488	AGCACCAAACTTTCCACCCA	60.033	CGTGTATGCGCGTTCGAATT	59.976	177
TRINITY_Contig5831	3	c	(GGGA)3gagagacagcagctggc(GCCG)3	41	1594	1634	AGCACCAAACTTTCCACCCA	60.033	CGTGTATGCGCGTTCGAATT	59.976	177
TRINITY_Contig5831	4	p3	(TTC)4	12	1766	1777	AGCACCAAACTTTCCACCCA	60.033	CGTGTATGCGCGTTCGAATT	59.976	177
TRINITY_Contig5832	1	p3	(GCA)6	18	20	37	TTCCCCATCAGCATGTCCAC	60.034	TCGAAGCAGCACTGAACACA	60.179	262
TRINITY_Contig5832	2	p4	(ATTA)3	12	555	566	TTCCCCATCAGCATGTCCAC	60.034	TCGAAGCAGCACTGAACACA	60.179	262
TRINITY_Contig5833	1	p3	(AGC)4	12	1474	1485	CACCCATGACACATCTGGCT	60.035	TCGTCCGTGAACAAGCATCA	59.967	183
TRINITY_Contig5833	2	c	(CCAAT)3ccagcgggatccaatccaacgcgc(CCCTT)4cccgcgcagcgcacggccccgctctccgagct(CTG)5	106	2619	2724	CACCCATGACACATCTGGCT	60.035	TCGTCCGTGAACAAGCATCA	59.967	183
TRINITY_Contig5833	3	c	(GCA)4gttgcagcgcctgaaccaccacg(TCC)5	50	3002	3051	CACCCATGACACATCTGGCT	60.035	TCGTCCGTGAACAAGCATCA	59.967	183
TRINITY_Contig5833	4	p4	(GCTT)3	12	5222	5233	CACCCATGACACATCTGGCT	60.035	TCGTCCGTGAACAAGCATCA	59.967	183
TRINITY_Contig5833	5	c	(CAT)4cagctcatcatctccctctctctttttttttct(TG)6	58	5732	5789	CACCCATGACACATCTGGCT	60.035	TCGTCCGTGAACAAGCATCA	59.967	183
TRINITY_Contig5833	6	p3	(ACG)4	12	6073	6084	CACCCATGACACATCTGGCT	60.035	TCGTCCGTGAACAAGCATCA	59.967	183
TRINITY_Contig5833	7	p3	(GGC)4	12	7619	7630	CACCCATGACACATCTGGCT	60.035	TCGTCCGTGAACAAGCATCA	59.967	183
TRINITY_Contig5834	1	p4	(TTTC)3	12	1277	1288	ACCCCCATCCTCTTCTCTCC	60.031	GGTAACGCTGTGGAAGTCCA	59.966	249
TRINITY_Contig5834	2	c	(TGG)4gcgcgcgggagggggtggtggtcggaggggaggtacgggatcgggtgggagtcgtactcgtagtcgtacctagaccgcggaggggcaacgccc(CCG)4	117	6084	6200	ACCCCCATCCTCTTCTCTCC	60.031	GGTAACGCTGTGGAAGTCCA	59.966	249
TRINITY_Contig5834	3	c	(GCG)6(GCT)5	33	6447	6479	ACCCCCATCCTCTTCTCTCC	60.031	GGTAACGCTGTGGAAGTCCA	59.966	249
TRINITY_Contig5835	1	p3	(GCG)6	18	179	196	CTCTCTCTCCTCCTCCACCC	60.106	TGTCCGTCAAGATCTACGCG	59.902	235
TRINITY_Contig5835	2	p2	(CT)6	12	336	347	CTCTCTCTCCTCCTCCACCC	60.106	TGTCCGTCAAGATCTACGCG	59.902	235
TRINITY_Contig5835	3	p3	(GCA)4	12	458	469	CTCTCTCTCCTCCTCCACCC	60.106	TGTCCGTCAAGATCTACGCG	59.902	235
TRINITY_Contig5837	1	p3	(TCC)5	15	515	529	TCACGTGCGAGTCACATTCA	59.969	TCCAGTAGGTGCTCCGATCA	60.034	133
TRINITY_Contig5837	2	p3	(CCG)5	15	938	952	TCACGTGCGAGTCACATTCA	59.969	TCCAGTAGGTGCTCCGATCA	60.034	133
TRINITY_Contig5838	1	p3	(TGA)4	12	1113	1124	AGGCCATTGAGAGAACTGGC	60.035	TGACACAGTAGCAACCGACC	59.967	100
TRINITY_Contig5839	1	p4	(TGGA)3	12	1911	1922	CACAACAGCAAGCTGGTCAC	59.97	GACATTAGCATGTGCAGCGG	59.971	136
TRINITY_Contig5840	1	p3	(CCT)4	12	423	434	CTCGCTCTTCTCTAGCACCG	59.971	TTGTCCACCAGGAGCATCAC	59.963	120
TRINITY_Contig5841	1	p3	(GCG)4	12	3676	3687	CGTATGCGCCTTTCTTCACG	59.975	TGTCATGGAGAAGTGCGCTT	59.965	251
TRINITY_Contig5841	2	p4	(GAGC)3	12	3845	3856	CGTATGCGCCTTTCTTCACG	59.975	TGTCATGGAGAAGTGCGCTT	59.965	251
TRINITY_Contig5843	1	p3	(CTC)4	12	334	345	CTCCCGCGCTAGCTTTATCA	59.968	AGGAGAGGCGGAGGTAGAAG	60.106	197
TRINITY_Contig5844	1	p4	(GCTC)4	16	110	125	CAGCCAGACAAAACAGCACC	59.969	CGTCTCTAGCGGCTGTGTAG	59.972	210
TRINITY_Contig5846	1	c	(CCA)4ccgcctgctggtgaccctcgctcgccatggccgctt(CGC)4	60	5629	5688	GAACGGAAAAGGTTGTGGGC	59.968	GAGCCACTTGCAACAACTGG	59.969	196
TRINITY_Contig5846	2	p3	(CCG)5	15	5871	5885	GAACGGAAAAGGTTGTGGGC	59.968	GAGCCACTTGCAACAACTGG	59.969	196
TRINITY_Contig5847	1	p3	(GGT)4	12	1466	1477	GGCCCAGATCTTTGCTGAGT	60.035	AGGACACTACGGGATGCTCT	60.033	138
TRINITY_Contig5847	2	p3	(TCC)4	12	2147	2158	GGCCCAGATCTTTGCTGAGT	60.035	AGGACACTACGGGATGCTCT	60.033	138
TRINITY_Contig5847	3	p3	(GGC)4	12	2962	2973	GGCCCAGATCTTTGCTGAGT	60.035	AGGACACTACGGGATGCTCT	60.033	138
TRINITY_Contig5848	1	p4	(CATC)3	12	84	95	GTGCCTTCGTAGTGCTGACT	60.038	TTGTAGCAAACGCACAAGGC	59.97	120
TRINITY_Contig5849	1	p4	(CCGC)3	12	106	117	CGTAGCCGGTGAGGAAGATC	59.969	CCAGTACGAGGGTGGAGAGA	60.034	191
TRINITY_Contig5849	2	p5	(CTTCC)3	15	354	368	CGTAGCCGGTGAGGAAGATC	59.969	CCAGTACGAGGGTGGAGAGA	60.034	191
TRINITY_Contig5850	1	p3	(GGC)4	12	490	501	GGGTAGCTCTGACTGCGTTT	60.037	TTCGTTCGATCGTCGGGATC	59.971	190
TRINITY_Contig5852	1	p4	(CCAT)3	12	841	852	TGCCTGTACTTGTACGCGTT	59.968	CATCGCTGCAAGCTCAATCC	59.971	168
TRINITY_Contig5853	1	p3	(GGC)4	12	526	537	ACCGGACGAGGGTTGTTTAC	59.966	CAGTCGATCGAACCTCCTCG	59.972	207
TRINITY_Contig5854	1	p3	(CGC)4	12	140	151	TACAACTCGGTCCTGCACAC	59.967	CCTGGTCTCGTCATCCATCG	59.969	102
TRINITY_Contig5856	1	p6	(GACGGC)4	24	4170	4193	GCTCCAATCTTGTGGCCTCT	60.035	GAACACAGCTGTCCTTCGGA	59.966	251
TRINITY_Contig5856	2	p3	(CGG)5	15	4354	4368	GCTCCAATCTTGTGGCCTCT	60.035	GAACACAGCTGTCCTTCGGA	59.966	251
TRINITY_Contig5856	3	p5	(GCTGA)3	15	4646	4660	GCTCCAATCTTGTGGCCTCT	60.035	GAACACAGCTGTCCTTCGGA	59.966	251
TRINITY_Contig5858	1	p3	(CCT)5	15	256	270	AGGAGCACCACTATCTCGGT	60.033	TCGCCTTCGCTATCGTTACC	59.97	222
TRINITY_Contig5858	2	p3	(GGT)4	12	1561	1572	AGGAGCACCACTATCTCGGT	60.033	TCGCCTTCGCTATCGTTACC	59.97	222
TRINITY_Contig5859	1	p3	(CGC)4	12	3659	3670	CGGAAGATCTCGGACGAGTG	59.972	TCCTCCCTACCAGTCACGAG	60.034	200
TRINITY_Contig5860	1	p4	(TCCC)3	12	1261	1272	AGAGCTACGCTGGGCATTAC	59.895	CGCGTTCCCGATCATGAAAC	59.974	113
TRINITY_Contig5862	1	p3	(CCG)4	12	989	1000	CAGTTGACCGTTTGGCACAG	59.971	AGTCATGGAGAAACGGCTGG	60.036	168
TRINITY_Contig5862	2	p2	(CG)6	12	1220	1231	CAGTTGACCGTTTGGCACAG	59.971	AGTCATGGAGAAACGGCTGG	60.036	168
TRINITY_Contig5862	3	p5	(GCGCT)3	15	1377	1391	CAGTTGACCGTTTGGCACAG	59.971	AGTCATGGAGAAACGGCTGG	60.036	168
TRINITY_Contig5863	1	p3	(TAC)4	12	394	405	CGATGGCTCGGTACAGGTAC	59.97	CACAACAAGTGCTTGCTCCC	59.969	168
TRINITY_Contig5863	2	c	(CGAT)3ggctcggtacaggtactgtagtgtaaaccctagcctagcttgctagaaaactcttgctggagaagga(AGGG)3(AGGAA)3	106	1561	1666	CGATGGCTCGGTACAGGTAC	59.97	CACAACAAGTGCTTGCTCCC	59.969	168
TRINITY_Contig5864	1	p3	(CCG)4	12	1087	1098	CGGTAGACTCAGATGCGCAT	59.969	CCATGAGACAGGCAACGACT	60.037	134
TRINITY_Contig5865	1	p3	(CGC)5	15	486	500	GTCGATGAAGAGTCGGTCCC	59.899	CGCTTCCGCTACTCCTACTG	59.97	153
TRINITY_Contig5865	2	p3	(GGC)5	15	662	676	GTCGATGAAGAGTCGGTCCC	59.899	CGCTTCCGCTACTCCTACTG	59.97	153
TRINITY_Contig5865	3	p3	(CCG)4	12	1257	1268	GTCGATGAAGAGTCGGTCCC	59.899	CGCTTCCGCTACTCCTACTG	59.97	153

TRINITY_Contig5866	1	c	(TCCC)3cagcgtacgccttctccgccgttcctctcgtctcctcccttcgaatcttcccttccttcgatccatctctcgcct(CG)6	99	1	99	AGGGAGCTGTCATGGATCCT	60.03	GGATGATGCTGAGGTCCGAG	59.967	165
TRINITY_Contig5867	1	p3	(CTG)5	15	3673	3687	CTGGCCTGAGAACGATGTGT	60.037	GTCATGGCGAGCGATGTTTC	59.973	177
TRINITY_Contig5867	2	p3	(CTC)4	12	3836	3847	CTGGCCTGAGAACGATGTGT	60.037	GTCATGGCGAGCGATGTTTC	59.973	177
TRINITY_Contig5867	3	p3	(GGA)5	15	3985	3999	CTGGCCTGAGAACGATGTGT	60.037	GTCATGGCGAGCGATGTTTC	59.973	177
TRINITY_Contig5867	4	p4	(CAAG)4	16	4176	4191	CTGGCCTGAGAACGATGTGT	60.037	GTCATGGCGAGCGATGTTTC	59.973	177
TRINITY_Contig5868	1	p3	(TGC)4	12	859	870	GAGTAGGGGAGTGGGGGAAT	60.03	TCACACACGCTCCTCCTTTC	59.966	115
TRINITY_Contig5868	2	p3	(CCG)4	12	1399	1410	GAGTAGGGGAGTGGGGGAAT	60.03	TCACACACGCTCCTCCTTTC	59.966	115
TRINITY_Contig5868	3	c	(GCC)5agcatcgtg(GCC)4	36	1552	1587	GAGTAGGGGAGTGGGGGAAT	60.03	TCACACACGCTCCTCCTTTC	59.966	115
TRINITY_Contig5874	1	c	(TGGC)4aacaagaacacaaa(AGGC)3agctatgtcgatccg(TGGCT)3gcatcta(TCTT)3ttt(TTTC)3	106	106	211	ACAGGTACTTCGTTGCCCTG	59.965	CGTTGTTGTTGGCCCTGAAG	59.969	219
TRINITY_Contig5874	2	p4	(CTTC)3	12	334	345	ACAGGTACTTCGTTGCCCTG	59.965	CGTTGTTGTTGGCCCTGAAG	59.969	219
TRINITY_Contig5874	3	c	(ATCC)3atcg(ATGGCA)3	34	502	535	ACAGGTACTTCGTTGCCCTG	59.965	CGTTGTTGTTGGCCCTGAAG	59.969	219
TRINITY_Contig5874	4	c*	(TGGT)2(TG<GT>)(GT)6	24	2511	2534	ACAGGTACTTCGTTGCCCTG	59.965	CGTTGTTGTTGGCCCTGAAG	59.969	219
TRINITY_Contig5875	1	p4	(AACA)3	12	596	607	GCAGACAGGACTCCAGTGAC	60.038	CCAGATGGACCTCCCTCAGA	60.032	272
TRINITY_Contig5876	1	c	(CCGAGC)3agcgccgcgaccggcagcacgacgcggcgctgctcgcttccctcgacggcggcaatcgcgcgcagcgcgtggaacgacgccacgc(GCT)4	115	1604	1718	TTCGTACTCGACCTCCGACT	60.037	TCAACCTGACCAAGCCTCGAC	59.966	137
TRINITY_Contig5877	1	p3	(GCG)5	15	32	46	ATTGAAGCCCCTGCACTACC	60.034	CGGTCCCACATGTCAGTGAA	59.965	117
TRINITY_Contig5880	1	p3	(CCG)4	12	1223	1234	GTGAAAGGCAGCCAGGTACT	59.963	CTCCCGACTCGAAGCAAACT	60.038	265
TRINITY_Contig5881	1	p3	(GCC)4	12	126	137	CCAGATGGTTCGAAGTGCCT	60.036	AGTAAGCATGTGCAGGGCAT	60.034	183
TRINITY_Contig5882	1	p4	(TCTT)3	12	244	255	GCCTTCTTGCCTTCTCCACT	59.963	GATGAGGGGCTGATGGTGAG	59.892	265
TRINITY_Contig5883	1	p3	(CCA)4	12	234	245	ATGGCACGAACTGATGAGCA	60.036	CGAGGAAGGGGAGGATGAGA	60.106	231
TRINITY_Contig5883	2	c	(TTC)6ctcttgatggttgcgtgaccgatgaagatgatctcggccatttgatat(TCAG)4	82	611	692	ATGGCACGAACTGATGAGCA	60.036	CGAGGAAGGGGAGGATGAGA	60.106	231
TRINITY_Contig5883	3	p3	(GCG)5	15	1307	1321	ATGGCACGAACTGATGAGCA	60.036	CGAGGAAGGGGAGGATGAGA	60.106	231
TRINITY_Contig5884	1	c	(GAGCAG)3cggcaggaggaggcggggaggaggcatgacgatggcgggcggatgcgcgcgcggattggtggcctc(GCG)4(GTG)4	108	2465	2572	GAGGATCATCGTGGCCAGAG	59.967	AACCATGCATCCCGTAGCAA	60.035	134
TRINITY_Contig5884	2	c	(ACG)4gc(GCAT)3	26	2789	2814	GAGGATCATCGTGGCCAGAG	59.967	AACCATGCATCCCGTAGCAA	60.035	134
TRINITY_Contig5885	1	p4	(AGGA)3	12	1495	1506	CATCTTCCCAGGGTGTGACC	60.035	AGCCCATCAAGGAGCAGTTC	60.035	259
TRINITY_Contig5886	1	p4	(CTTT)3	12	3281	3292	AAGCCGGAAGAATGACCCTG	60.035	TATAGGCTCAACCCAGGCCT	60.03	271
TRINITY_Contig5889	1	p3	(CCG)4	12	65	76	CGTCACCTTCGTTGAGGTCA	59.968	CCACTTTTTGCTTACCGCCC	60.039	164
TRINITY_Contig5891	1	p3	(CTG)4	12	2008	2019	CGGGGATGATGTAGACGGTG	59.968	TGGGAGAAGGTGATCGGGAT	60.031	172
TRINITY_Contig5891	2	p3	(GGC)4	12	2195	2206	CGGGGATGATGTAGACGGTG	59.968	TGGGAGAAGGTGATCGGGAT	60.031	172
TRINITY_Contig5892	1	p3	(CCA)4	12	360	371	GCGGGATTGTGGGAGGTTTA	60.035	GGATCAGACACTGCAGCTT	60.036	214
TRINITY_Contig5892	2	p3	(GGC)4	12	3369	3380	GCGGGATTGTGGGAGGTTTA	60.035	GGATCAGACACTGCAGCTT	60.036	214
TRINITY_Contig5895	1	p3	(CTC)4	12	343	354	TGCACGGTAACTTCAGGGAC	59.966	CACAGCAAGCAACAGACACC	59.97	174
TRINITY_Contig5896	1	p3	(GGC)6	18	1620	1637	ATATCCGGCGTCACGTTCTC	59.97	TGGACCCCTTCCCATCGTAT	60.031	189
TRINITY_Contig5897	1	p4	(AGCC)3	12	162	173	CCTCACTTCGACCAGCTGTT	59.966	CTCGTGCGCGATGTAGTTTG	59.976	270
TRINITY_Contig5899	1	p4	(GAGG)3	12	343	354	TGTACCACGAGTTCAGCGAC	60.04	ACCTTGTAGTTGGTGAGCCG	59.965	148
TRINITY_Contig5899	2	p3	(GGC)4	12	2028	2039	TGTACCACGAGTTCAGCGAC	60.04	ACCTTGTAGTTGGTGAGCCG	59.965	148
TRINITY_Contig5899	3	p5	(CTGAC)3	15	2162	2176	TGTACCACGAGTTCAGCGAC	60.04	ACCTTGTAGTTGGTGAGCCG	59.965	148
TRINITY_Contig5900	1	p3	(CCG)4	12	2213	2224	CAATGTCACCACAGCCAAGC	60.039	GCATATGCAGCAGTGTCAGC	59.971	138
TRINITY_Contig5901	1	p3	(CGG)4	12	2450	2461	CCCACAGCAAAACAGACAGC	59.969	CTCCAGCAGTGTGGTCTCTG	60.038	141
TRINITY_Contig5902	1	p3	(CGC)4	12	202	213	ATGCCGAACTTCTCACCCTG	60.036	GCTAGCAACCTGGACACCAT	60.035	195
TRINITY_Contig5904	1	c	(CAG)4ccaccg(CCT)4gggaacccctggcctcctcaccacgcctccggccagcccccgccgcctagctaccct(CCG)4	99	367	465	TACAGCCCCTCCGTATCACA	60.033	ACATGAACGTACTGGCAGCA	59.965	105
TRINITY_Contig5904	2	p2	(CT)9	18	3027	3044	TACAGCCCCTCCGTATCACA	60.033	ACATGAACGTACTGGCAGCA	59.965	105
TRINITY_Contig5905	1	c	(GCG)4c(AGG)4	25	1647	1671	TGCCCTCCACATCGGTAGTA	60.033	CCCCTGGAGCAAGAAGAAGG	60.035	273
TRINITY_Contig5907	1	p3	(CCG)4	12	164	175	ACCAGATCGTCAAGCAGGTG	60.037	GTGATGTCCTGAGTGCAGCT	60.037	186
TRINITY_Contig5907	2	p4	(ACAA)3	12	1181	1192	ACCAGATCGTCAAGCAGGTG	60.037	GTGATGTCCTGAGTGCAGCT	60.037	186
TRINITY_Contig5908	1	p2	(CT)7	14	12	25	CTTTGTGGCTTGTCAGGTGC	59.969	CTTCCTCAGCTTGATGGCCA	60.035	249
TRINITY_Contig5908	2	p3	(TGG)5	15	154	168	CTTTGTGGCTTGTCAGGTGC	59.969	CTTCCTCAGCTTGATGGCCA	60.035	249
TRINITY_Contig5909	1	c	(AAGAGC)3aagaggataacgcgcgacggcgggagggtgaccgtgcagggcgcacgcgcgcctctgtgtgcg(TTGGC)3	96	3377	3472	TGCCCATTTTCAACAGCACG	59.968	AGGCACGAGAGAAGTTGTGG	59.966	187
TRINITY_Contig5910	1	p3	(CGC)6	18	1151	1168	AGTCCATGTGAGCAGAGCAC	60.037	TCGACAACCCCCAACCAAAA	60.033	117
TRINITY_Contig5911	1	c	(GCC)4gcggcgcgcgacgacgagcggagggcgaggcgtcaggcgtcgaaccgcgagtcggcgcg(GC)7ggcggaggggccagctggacgtgctgtcgtcgcgcgtcgcggagctccgcgccgcgaacgcgcggctggccgtggagctgaaccgcgtggcggc(CG)6ggcggcgcgggagggcgcgcggctcagggaggaggcgcgcgccctgcgggagaggctcgacgccgccgagcaggcgcagcac(GCG)4agggaaaaggaagccggcggtgaggaagccgg(GAC)4	329	430	758	ATGCCTGCAACTTTAGGGCT	59.96	GCATGATTGCCTAGAAGCGC	60.041	218
TRINITY_Contig5911	2	p6	(CGGCGA)3	18	1190	1207	ATGCCTGCAACTTTAGGGCT	59.96	GCATGATTGCCTAGAAGCGC	60.041	218
TRINITY_Contig5913	1	p4	(CCTC)3	12	24	35	TCAAAGCCTGCTGGGATCAG	60.035	CAACCCACACTGGAAAAGCG	59.969	161
TRINITY_Contig5914	1	p5	(GGAGG)3	15	2896	2910	CCGACCGGATCAGCGATTTA	59.968	CGAGGATGACGGTGATGGAG	59.969	195
TRINITY_Contig5919	1	p3	(CCG)4	12	60	71	ATGGCTGGCATGATGGTGAA	60.033	AGCTGATCTTCTTTGGCGCT	60.036	144
TRINITY_Contig5919	2	p3	(GGC)5	15	209	223	ATGGCTGGCATGATGGTGAA	60.033	AGCTGATCTTCTTTGGCGCT	60.036	144
TRINITY_Contig5920	1	p3	(GGC)5	15	105	119	ACAAGGTCGAGGAATGGCTG	60.036	TGTACTCAACAGCCGGTGAC	59.967	144
TRINITY_Contig5922	1	c	(TCT)4ccttctccccctcctcctgctcattctcaactaccgaatcgccatcgccactaaagccctcgttatcattcacattcc(TCT)4	102	2425	2526	TCAGCCTGCCTTTCACTCAG	59.964	CACGCTTAGCCACATCATGC	59.971	174
TRINITY_Contig5922	2	p5	(GGCGG)4	20	5787	5806	TCAGCCTGCCTTTCACTCAG	59.964	CACGCTTAGCCACATCATGC	59.971	174
TRINITY_Contig5923	1	c	(GGA)4agccacctgccacgccacccgctcccgccatacccgccgccaccatgatctccgcgcag(GCC)5	86	286	371	GCAAGAAGGTTTGGCTGGTG	59.967	TCGAACAAGGACGTCACAGG	59.968	118
TRINITY_Contig5924	1	p5	(CCTCG)3	15	70	84	GAGATCATGAGCGGCGTACA	59.969	TACTTGGTTCTGCTCAGGCG	60.037	156
TRINITY_Contig5926	1	p4	(CCTG)4	16	344	359	AAGTCCATGCCGCGTTTTTC	60.04	AGCTGGAGAGGCTATCCACA	60.032	223
TRINITY_Contig5927	1	p3	(TCG)4	12	547	558	TCTATCTCGAACGCAGCGAC	59.972	GGCCTACATCCAGCAGCTAG	59.965	165
TRINITY_Contig5927	2	c	(GCC)4gcggcgcgccggcgcggccgctcgggcggcggcgggggcgggggcttgggcttgctgttgcacgcgcggggcaggcgcgtcttccgcggggccggctg(CTC)4cgccggcagcgccgcccgcttccccgccttccccggcggcgcctccgccg(CCT)7ccctcgcgagccttatcttcttc(TCCG)3	228	1829	2056	TCTATCTCGAACGCAGCGAC	59.972	GGCCTACATCCAGCAGCTAG	59.965	165
TRINITY_Contig5927	3	p3	(CGG)4	12	2290	2301	TCTATCTCGAACGCAGCGAC	59.972	GGCCTACATCCAGCAGCTAG	59.965	165
TRINITY_Contig5927	4	p2	(CT)10	20	2468	2487	TCTATCTCGAACGCAGCGAC	59.972	GGCCTACATCCAGCAGCTAG	59.965	165
TRINITY_Contig5928	1	c	(CGG)4cgactccgccccgtcctcct(CGC)4	44	535	578	TGTGTTAAGCTAAGCGGGGG	60.036	ATGCTTGCCCCATCTGGTAC	60.107	112
TRINITY_Contig5928	2	c	(GACGAG)3cacggcgacggaccgacgttcctctgcgcccgctggtgctgccgctgcggcggcgggggcgtcgacga(CGC)4	98	1136	1233	TGTGTTAAGCTAAGCGGGGG	60.036	ATGCTTGCCCCATCTGGTAC	60.107	112
TRINITY_Contig5928	3	p3	(TGG)4	12	1671	1682	TGTGTTAAGCTAAGCGGGGG	60.036	ATGCTTGCCCCATCTGGTAC	60.107	112
TRINITY_Contig5928	4	c*	(CTGC)4(<CTGC>G)(CTGCG)2	31	1925	1955	TGTGTTAAGCTAAGCGGGGG	60.036	ATGCTTGCCCCATCTGGTAC	60.107	112
TRINITY_Contig5929	1	p3	(CGG)5	15	322	336	TCTTGGTGATGCAAGAGCGT	59.965	GGCCGACACTTTCTACACCA	59.966	217
TRINITY_Contig5933	1	p5	(AGCCG)3	15	100	114	CCTGGACCGGAGTCACTAGA	60.034	ATCTGAAGGGCTGGCTCCTA	60.031	177
TRINITY_Contig5935	1	c	(CCA)4aacctgctgccacctccacgg(CCA)5	48	86	133	CGCCGAAAGCCCATATGTTG	59.971	GCAGGCCTCCAAGTGTTTTG	59.967	101
TRINITY_Contig5935	2	p3	(GCG)4	12	2249	2260	CGCCGAAAGCCCATATGTTG	59.971	GCAGGCCTCCAAGTGTTTTG	59.967	101
TRINITY_Contig5935	3	p5	(ATCTC)3	15	4720	4734	CGCCGAAAGCCCATATGTTG	59.971	GCAGGCCTCCAAGTGTTTTG	59.967	101
TRINITY_Contig5935	4	p5	(AAAAG)3	15	5609	5623	CGCCGAAAGCCCATATGTTG	59.971	GCAGGCCTCCAAGTGTTTTG	59.967	101
TRINITY_Contig5936	1	p3	(CGC)4	12	146	157	TCTTGCCGTTCAGAAGACCC	59.965	CTGCTGCGGTCTACTACGAG	59.972	171
TRINITY_Contig5936	2	p3	(ACG)4	12	5898	5909	TCTTGCCGTTCAGAAGACCC	59.965	CTGCTGCGGTCTACTACGAG	59.972	171
TRINITY_Contig5938	1	p3	(GCG)4	12	766	777	TGAGCAAGGGGAATGACCAC	59.962	TGGTCCTGTTATGGGTGTGC	59.962	129

TRINITY_Contig5939	1	p3	(CGC)4	12	287	298	TAGCAAACCTGTCAGCTGGG	59.963	TTCCACTGGTGATGATGGGC	60.034	107
TRINITY_Contig5939	2	p3	(CAG)4	12	870	881	TAGCAAACCTGTCAGCTGGG	59.963	TTCCACTGGTGATGATGGGC	60.034	107
TRINITY_Contig5941	1	p3	(CGG)5	15	546	560	CGACGACTCATCCTCTACGC	60.042	GTCCTGACGACACGGTTCTT	59.968	234
TRINITY_Contig5944	1	p3	(CCG)4	12	6	17	GAGGTGTTGGCAGATGCTCT	60.036	GAGCAAATGTGGCCAAACGT	59.968	100
TRINITY_Contig5947	1	p3	(GCC)4	12	7100	7111	TCGCGACGTAGATCTGCTTC	59.972	ACTGGTACTGGCAACGAGTG	59.966	112
TRINITY_Contig5948	1	p3	(GGC)6	18	300	317	GCTACGCGGTTATCTGCTCT	59.969	GCCAGCCTACTTGACACAGT	59.965	196
TRINITY_Contig5948	2	p2	(CT)9	18	505	522	GCTACGCGGTTATCTGCTCT	59.969	GCCAGCCTACTTGACACAGT	59.965	196
TRINITY_Contig5949	1	p3	(GAC)4	12	1202	1213	TCGATCTCCTCCTCGGTGTT	60.034	TCGAGGTGTGGAACAAGTCG	59.968	118
TRINITY_Contig5950	1	p3	(CCT)4	12	1557	1568	CACCACCACTGGGATCTGTC	60.036	CAGAAGCTCCTCTATGGGCG	59.967	254
TRINITY_Contig5951	1	c	(CGGCGT)4ggggggctgcgcaggcggtgg cttgtgctgcttcttggagt(GGC)4	77	435	511	CAGATGACCATCGCCTACGG	60.319	CTCCAAGAAGCAGCACAAGC	59.76	146
TRINITY_Contig5952	1	p3	(GAG)4	12	74	85	TGTGGTTGACAGGTTGCAGT	60.035	ATTGAATGCCCTGCTCAGCT	60.033	264
TRINITY_Contig5952	2	p3	(CGG)5	15	320	334	TGTGGTTGACAGGTTGCAGT	60.035	ATTGAATGCCCTGCTCAGCT	60.033	264
TRINITY_Contig5953	1	p6	(GGGCGT)3	18	113	130	CTCGTCATCGACCTCTTCCG	59.972	CACTGGTACGAGTGGTTGCT	59.966	141
TRINITY_Contig5958	1	p3	(GGC)4	12	286	297	GTCGGAGAAGGCGTCATAGG	59.969	CACCAAACGCCTGGATAGGT	60.035	193
TRINITY_Contig5962	1	p3	(GCC)9	27	924	950	TCACCTGAAGACTTGCCGTC	59.966	TCGCGGAGGATGATGATGTG	59.967	130
TRINITY_Contig5963	1	p6	(CGCGGC)3	18	160	177	ACAAGGCGTGGGAGAATGAG	60.036	TTTGGTGCTTGTGCGTGATG	59.969	148
TRINITY_Contig5966	1	p3	(CGT)5	15	1704	1718	GATCGTGGAGCTTTGGGTGA	60.036	CGGTGTCTCCACGAGTTTCA	59.968	118
TRINITY_Contig5967	1	p3	(CTC)5	15	104	118	GCATGCATGCGTACTGGAAG	59.971	CCCAACGTCCTCAATCCACA	59.964	108
TRINITY_Contig5967	2	p4	(GGAG)5	20	7003	7022	GCATGCATGCGTACTGGAAG	59.971	CCCAACGTCCTCAATCCACA	59.964	108
TRINITY_Contig5968	1	p4	(TCCA)4	16	155	170	GGGAAGGGGTAGGGAGTGAT	60.03	TCCTTAACTCCCTCCTCCGG	60.032	127
TRINITY_Contig5973	1	p3	(GCG)4	12	74	85	TCCAATGCCCAGTGCTAGTG	60.035	GCAAGTGCCCACAACTCTTG	59.969	126
TRINITY_Contig5973	2	p3	(GAG)4	12	200	211	TCCAATGCCCAGTGCTAGTG	60.035	GCAAGTGCCCACAACTCTTG	59.969	126
TRINITY_Contig5973	3	p3	(GCC)4	12	2332	2343	TCCAATGCCCAGTGCTAGTG	60.035	GCAAGTGCCCACAACTCTTG	59.969	126
TRINITY_Contig5976	1	p4	(GGAT)3	12	15	26	TATCCACCGGTGACTGGGAT	60.032	TCAGGTCGCTCTTGCATTGT	59.965	175
TRINITY_Contig5976	2	p3	(GCC)5	15	449	463	TATCCACCGGTGACTGGGAT	60.032	TCAGGTCGCTCTTGCATTGT	59.965	175
TRINITY_Contig5978	1	p3	(TGG)7	21	1459	1479	TCCAATCCCGTCCTAGCTGA	60.033	GAGGGGAGTTCAAGTCCTGC	60.036	191
TRINITY_Contig5978	2	p3	(AGC)4	12	2074	2085	TCCAATCCCGTCCTAGCTGA	60.033	GAGGGGAGTTCAAGTCCTGC	60.036	191
TRINITY_Contig5979	1	p3	(CGC)6	18	1201	1218	TCAGGAAGTCCAGCAGCTTG	59.964	GAGATCATGGCGCTGATCCA	59.966	106
TRINITY_Contig5981	1	p4	(CTCC)3	12	119	130	GATGAATGGTGAGCGTGCAC	59.902	GCTTGCACCCTCTCAGAGTT	59.964	201
TRINITY_Contig5984	1	p3	(GCG)4	12	208	219	TCTCTAGGCAGCCAGGATGT	60.032	CCTATGAAGCTCTCGCCCAG	59.967	173
TRINITY_Contig5984	2	c	(AGC)4aggcgaagagcggcgccggcgatc agctccagatggggtcgt(CGA)4	66	982	1047	TCTCTAGGCAGCCAGGATGT	60.032	CCTATGAAGCTCTCGCCCAG	59.967	173
TRINITY_Contig5985	1	p4	(CATC)3	12	129	140	GCAGTTGTTTCCAGGCCAAG	59.967	ATGCCAGAGGTGGTGTCATG	60.035	247
TRINITY_Contig5986	1	p3	(GCC)5	15	183	197	CTTGTTGTTGCTTTCCCCGG	59.968	CGAGAAGAGGAGGAAGGGGA	60.033	133
TRINITY_Contig5986	2	p3	(CGC)5	15	395	409	CTTGTTGTTGCTTTCCCCGG	59.968	CGAGAAGAGGAGGAAGGGGA	60.033	133
TRINITY_Contig5986	3	p3	(CCA)4	12	694	705	CTTGTTGTTGCTTTCCCCGG	59.968	CGAGAAGAGGAGGAAGGGGA	60.033	133
TRINITY_Contig5986	4	p3	(GAA)4	12	2504	2515	CTTGTTGTTGCTTTCCCCGG	59.968	CGAGAAGAGGAGGAAGGGGA	60.033	133
TRINITY_Contig5986	5	c	(AGCC)3agagccagcgaggcaaagcc(CA TA)3tgtgtactttgcaaattggcccagcagc agatgtctgtctatgggcccggcggcccgt(G AA)6	120	2867	2986	CTTGTTGTTGCTTTCCCCGG	59.968	CGAGAAGAGGAGGAAGGGGA	60.033	133
TRINITY_Contig5986	6	c	(CGG)6ccacgcatcctcctccccctcctccac ctgctgctgcctgtgagtgtga(CCTCTC)3	85	3096	3180	CTTGTTGTTGCTTTCCCCGG	59.968	CGAGAAGAGGAGGAAGGGGA	60.033	133
TRINITY_Contig5987	1	p3	(GCG)4	12	1530	1541	ACATTGTGTGTTGCCATGGC	59.966	GCTGCTGTCGCTTATGTTGG	59.902	107
TRINITY_Contig5989	1	p3	(CGG)5	15	1216	1230	TTCTCGTCCGCTTCGATAGC	59.971	GAACTAAGGCTGTCCGCACT	60.037	122
TRINITY_Contig5989	2	p3	(CCG)4	12	1546	1557	TTCTCGTCCGCTTCGATAGC	59.971	GAACTAAGGCTGTCCGCACT	60.037	122
TRINITY_Contig5990	1	p3	(CGG)4	12	270	281	TTCTCGTCCGCTTCGATAGC	59.971	GAACTAAGGCTGTCCGCACT	60.037	122
TRINITY_Contig5991	1	p3	(TGC)4	12	1363	1374	CTGTCCAAGTGTGTCCTCCC	59.965	GGGATGGGTGGAACAGTGAG	60.035	147
TRINITY_Contig5993	1	p4	(ATGA)4	16	2402	2417	CGTCTTCACAGCAGTGTCCT	59.967	TTCCGCTGTGTTGGCAAATG	59.968	252
TRINITY_Contig5994	1	p6	(GAAGCA)3	18	3159	3176	AAATGCGGTCCAGTAAGGGG	60.034	TTTGGCTTTTGACTCGCGTG	59.971	101
TRINITY_Contig5996	1	c	(GAGCAG)4ccccgtggtcgcttcgccggcg ttgacctctcaccaccagcacggccacctccct cccccg(CCT)5ct(TCC)5	117	2452	2568	CTCCTGCACCATGTTAGCCA	60.035	TTCCCTTGCCCAATGATCCC	60.032	204
TRINITY_Contig5998	1	c	(CCT)5g(GCC)4	28	407	434	GCTCCACGATGTTCACCTGA	60.037	CAAGCACACTTGTGGCCATC	60.039	142
TRINITY_Contig5998	2	c	(CG)6gcgtcggcgtacgtgggccggctgt(G CG)5gggcggcggcgaaggggaag(GGC) 4	84	624	707	GCTCCACGATGTTCACCTGA	60.037	CAAGCACACTTGTGGCCATC	60.039	142
TRINITY_Contig5999	1	p3	(CGG)4	12	1120	1131	GGTTGAGAGGAGGTGAAGGC	60.036	AACCCTAATCCTCCTCCCCC	60.029	244
TRINITY_Contig6000	1	p5	(AATCT)4	20	757	776	GGAACGAAGCGTACGATTGC	59.975	ATCGATCATGGCCATCGCAT	60.036	214
TRINITY_Contig6000	2	c	(CCG)4gcggcgctgctcttcttctggcacttg tccttgatccactggaacccgtgcgccgtgccc tccttcaccttcctgagcccggt(CGC)4	107	1622	1728	GGAACGAAGCGTACGATTGC	59.975	ATCGATCATGGCCATCGCAT	60.036	214
TRINITY_Contig6000	3	p3	(CGA)4	12	1887	1898	GGAACGAAGCGTACGATTGC	59.975	ATCGATCATGGCCATCGCAT	60.036	214
TRINITY_Contig6001	1	p3	(TGC)4	12	1663	1674	CGCTGGCTTGCTGAGTTTTT	59.969	TCAGCAGTCCTCACAGCATG	60.037	140
TRINITY_Contig6001	2	p4	(ATCA)3	12	1802	1813	CGCTGGCTTGCTGAGTTTTT	59.969	TCAGCAGTCCTCACAGCATG	60.037	140
TRINITY_Contig6002	1	p3	(AAT)4	12	22	33	AGGTCCTCCCATTCCGATCA	60.031	CGGTGATATGGGTGCTCCTC	59.966	205
TRINITY_Contig6002	2	p3	(CTG)5	15	555	569	AGGTCCTCCCATTCCGATCA	60.031	CGGTGATATGGGTGCTCCTC	59.966	205
TRINITY_Contig6002	3	p3	(CGC)4	12	2249	2260	AGGTCCTCCCATTCCGATCA	60.031	CGGTGATATGGGTGCTCCTC	59.966	205
TRINITY_Contig6003	1	p3	(GAC)4	12	350	361	GGCGACCAGACAAGTCTTGA	59.966	GATGGTCGTCGATGCTTTGC	59.973	100
TRINITY_Contig6004	1	c	(GGT)5tgaaatgcatcaggatgcgggaacg gg(GGT)6	60	929	988	CATTTGGGGTGGCATTGTGG	60.036	ATCCTCCATCACCAGAGCCT	60.03	101
TRINITY_Contig6005	1	p4	(GGGA)3	12	1061	1072	CCAATTGAGCAGGGCAGAGA	60.035	GCCACGGGTGAAACCTTTTC	59.968	273
TRINITY_Contig6005	2	p4	(GGCT)3	12	1260	1271	CCAATTGAGCAGGGCAGAGA	60.035	GCCACGGGTGAAACCTTTTC	59.968	273
TRINITY_Contig6005	3	p3	(CAC)6	18	1514	1531	CCAATTGAGCAGGGCAGAGA	60.035	GCCACGGGTGAAACCTTTTC	59.968	273
TRINITY_Contig6012	1	p3	(CGC)5	15	50	64	CTGCCGCGCCTAAAGAATTC	59.972	TCCACTACCACAACCTTCGC	59.966	203
TRINITY_Contig6012	2	p6	(GACCGC)3	18	422	439	CTGCCGCGCCTAAAGAATTC	59.972	TCCACTACCACAACCTTCGC	59.966	203
TRINITY_Contig6012	3	p6	(GGAGCA)3	18	2052	2069	CTGCCGCGCCTAAAGAATTC	59.972	TCCACTACCACAACCTTCGC	59.966	203
TRINITY_Contig6013	1	p3	(TCG)4	12	698	709	CGAGAAGCATGCCATGTGTG	59.902	CGCAGCATCTTTAGCAGCAG	59.972	159
TRINITY_Contig6014	1	p3	(GCC)4	12	1566	1577	GTAGGGGAGAAGGAGACGGT	60.033	TGGCGACAAAGAAACGCAAG	59.971	169
TRINITY_Contig6015	1	p4	(GAGG)3	12	320	331	GCAGAGCGGCTCAAACAAAA	59.969	TGGAGAAGAAATCGGTGGCC	60.035	278
TRINITY_Contig6015	2	p3	(GCA)5	15	580	594	GCAGAGCGGCTCAAACAAAA	59.969	TGGAGAAGAAATCGGTGGCC	60.035	278
TRINITY_Contig6016	1	p3	(CGG)4	12	1272	1283	AACAGGCTCTTCAACTCCGG	59.965	CAGGGCTCAGTTCCTGGAAG	60.036	126
TRINITY_Contig6017	1	c	(CT)9cctctcgcaagaagacgaggcaaaag agagaaacaaggaacgtttcgtttcaactccc gaggcaacgcaccaaccagcccaggcagctc agcctcgg(GTCTC)4	135	5	139	ATGGCGCCACACATTCTGTA	60.036	GCAGTAGACTGTGGCACCTT	59.965	151
TRINITY_Contig6017	2	c	(CAG)5(CAA)5	30	2169	2198	ATGGCGCCACACATTCTGTA	60.036	GCAGTAGACTGTGGCACCTT	59.965	151
TRINITY_Contig6017	3	p3	(CAG)4	12	2382	2393	ATGGCGCCACACATTCTGTA	60.036	GCAGTAGACTGTGGCACCTT	59.965	151
TRINITY_Contig6017	4	p3	(CAC)5	15	2551	2565	ATGGCGCCACACATTCTGTA	60.036	GCAGTAGACTGTGGCACCTT	59.965	151
TRINITY_Contig6017	5	p5	(TGCTG)3	15	3876	3890	ATGGCGCCACACATTCTGTA	60.036	GCAGTAGACTGTGGCACCTT	59.965	151
TRINITY_Contig6019	1	p3	(ATC)4	12	1334	1345	ACTTTCTAGCGCCCCTTGAC	60.036	TTGCGCTAGGATCCTGCAAA	60.036	177
TRINITY_Contig6019	2	p3	(CCG)4	12	3796	3807	ACTTTCTAGCGCCCCTTGAC	60.036	TTGCGCTAGGATCCTGCAAA	60.036	177
TRINITY_Contig6019	3	p3	(CTT)4	12	3941	3952	ACTTTCTAGCGCCCCTTGAC	60.036	TTGCGCTAGGATCCTGCAAA	60.036	177
TRINITY_Contig6021	1	p3	(GCG)4	12	364	375	CTGATGCCCTTATGTTGCGC	59.97	AACCAACAGCAGGTCTCGAG	59.966	219
TRINITY_Contig6022	1	p3	(ACT)4	12	367	378	GGTTCGCAAACTTGGCATGT	59.968	CATGATCAGACGCAAGCAGC	59.972	117
TRINITY_Contig6022	2	p6	(TGATCA)3	18	640	657	GGTTCGCAAACTTGGCATGT	59.968	CATGATCAGACGCAAGCAGC	59.972	117
TRINITY_Contig6023	1	p4	(ACCA)3	12	209	220	GAGGATGAGACAGACGAGCG	59.971	GCCACGATGAGATCCAGAGG	59.967	236
TRINITY_Contig6023	2	c	(CGTGGC)3cgtgggcatggtggtgctcgtg gtccgcgccgccaccgccgcagctgtgcccgt gcccgtggtcgtgcgggtgctc(GCCGTG)3	112	2206	2317	GAGGATGAGACAGACGAGCG	59.971	GCCACGATGAGATCCAGAGG	59.967	236
TRINITY_Contig6024	1	p3	(CGG)4	12	3285	3296	TTAGCTCTGAACGCACAGCA	59.967	GGCGTTATTGCTGAGCCATG	59.97	137
TRINITY_Contig6028	1	p3	(AGT)4	12	2179	2190	TCAGCCAATTTGCTCTCCGT	59.963	GTTGGAGTTGCTGCCTTTGG	59.967	107
TRINITY_Contig6028	2	c	(GAG)5caggggtattggaaggccagagg(CGGCGA)3	56	2639	2694	TCAGCCAATTTGCTCTCCGT	59.963	GTTGGAGTTGCTGCCTTTGG	59.967	107
TRINITY_Contig6030	1	p3	(GGC)4	12	416	427	CGAATCAGTCCTCCGACGAG	59.972	GTGGCGCTGCGGTTATAAAG	59.972	147
TRINITY_Contig6031	1	c	(GCACA)3gcacgcgccattcacag(CTC)9	59	47	105	GGAGCGGAATCAGCTACCTC	59.967	TAACAAGTGGTTGCGTTGCG	59.972	278
TRINITY_Contig6031	2	p6	(GCTCAA)3	18	222	239	GGAGCGGAATCAGCTACCTC	59.967	TAACAAGTGGTTGCGTTGCG	59.972	278
TRINITY_Contig6031	3	c	(CTCTGC)3ctcggggg(TAGC)3tag(AT TC)3cac(ACGCA)3	71	621	691	GGAGCGGAATCAGCTACCTC	59.967	TAACAAGTGGTTGCGTTGCG	59.972	278

TRINITY_Contig6031	4	c	(CAA)4tcttctccggaagagtccatcgtctctgcttcatctccatgtcttgtttcctgtcgc(CAA)4	81	1475	1555	GGAGCGGAATCAGCTACCTC	59.967	TAACAAGTGGTTGCGTTGCG	59.972	278
TRINITY_Contig6031	5	c	(GAGACG)3gagggggagagcc(GGAGCA)3	49	2896	2944	GGAGCGGAATCAGCTACCTC	59.967	TAACAAGTGGTTGCGTTGCG	59.972	278
TRINITY_Contig6032	1	p3	(GGA)4	12	64	75	GATAAGGCACGGGGTCTGAG	59.895	CTTCTTGGCTGGTCCTCCTG	60.036	189
TRINITY_Contig6032	2	c	(AGG)4tggaggtggcaaagcaggaggcgtccggcgagtcggcggccgcgtccaaggagagcagcgacgtgcggagctcggcgagcctgt(GCC)4	108	724	831	GATAAGGCACGGGGTCTGAG	59.895	CTTCTTGGCTGGTCCTCCTG	60.036	189
TRINITY_Contig6032	3	p6	(CCGAGC)4	24	1411	1434	GATAAGGCACGGGGTCTGAG	59.895	CTTCTTGGCTGGTCCTCCTG	60.036	189
TRINITY_Contig6032	4	c	(GAC)4gcagccgcc(ACG)4	33	1578	1610	GATAAGGCACGGGGTCTGAG	59.895	CTTCTTGGCTGGTCCTCCTG	60.036	189
TRINITY_Contig6032	5	p3	(GCT)4	12	1776	1787	GATAAGGCACGGGGTCTGAG	59.895	CTTCTTGGCTGGTCCTCCTG	60.036	189
TRINITY_Contig6034	1	p3	(CGT)4	12	372	383	CCACGAAACCTGACCGAAGA	59.967	CTCCTCTCGATCTTGGACGC	59.97	156
TRINITY_Contig6035	1	p4	(CTGC)3	12	179	190	CCGGTCTGTGCCCCATATAC	59.965	CTCATCGAGATGCAGACCCC	59.967	262
TRINITY_Contig6035	2	p4	(TGCT)3	12	2106	2117	CCGGTCTGTGCCCCATATAC	59.965	CTCATCGAGATGCAGACCCC	59.967	262
TRINITY_Contig6036	1	p2	(GC)7	14	481	494	GAAGCAGTCGTGGAACTGGA	59.966	CTCAAGGTCGGGTACTACGC	59.9	132
TRINITY_Contig6036	2	p5	(CCGCG)3	15	749	763	GAAGCAGTCGTGGAACTGGA	59.966	CTCAAGGTCGGGTACTACGC	59.9	132
TRINITY_Contig6036	3	c*	(CGGC)2(<CGGC>T)(CGGCT)2	23	1006	1028	GAAGCAGTCGTGGAACTGGA	59.966	CTCAAGGTCGGGTACTACGC	59.9	132
TRINITY_Contig6037	1	p3	(CCG)7	21	3785	3805	GTTTGGGTGCACAAGAGCAG	59.969	GGATGGCGGATACTTGAGG	59.965	196
TRINITY_Contig6040	1	c	(CAG)4cacgcgtctcctccccctcttggcccCgaatcgatctcccccaccaaacaggagcacccgatccccggaggccggagccggcggagcagcga(GC)6ggccagcccactcgtccatccaccatgctcagggtcgcgggcaggcgcctctcgtccgccctcgcctggcgcc(CCG)4tcacccggggccccctcgccggcgcccttcccggccg(CGA)5	254	72	325	GAGCGTGTCAAGAACCCTGA	59.966	AGATGTCATAGTGCGAGCCG	59.969	130
TRINITY_Contig6041	1	p3	(CAG)5	15	326	340	ACGAGGAAAGCGAACTCCAG	60.038	CAACTTGCCCAGGGTAGAGG	60.035	143
TRINITY_Contig6041	2	c	(GGA)4cctggcgcggcgcgcgctgcggggcagggagcgctgggtgtactgcagcagcggctcctcgccgacggctaccaccgccgccgcca(CGT)4	110	508	617	ACGAGGAAAGCGAACTCCAG	60.038	CAACTTGCCCAGGGTAGAGG	60.035	143
TRINITY_Contig6041	3	p3	(GGC)4	12	829	840	ACGAGGAAAGCGAACTCCAG	60.038	CAACTTGCCCAGGGTAGAGG	60.035	143
TRINITY_Contig6042	1	p3	(TCC)4	12	1811	1822	GCAAGAGCAACTGATAGCGC	59.973	CTGCAGGTGTCTGTGGTGAT	59.964	254
TRINITY_Contig6043	1	p3	(CAG)8	24	4585	4608	AGGCTCCTGATGCCTCCTAA	60.031	TGCCAAGAGTACTGCACAGG	59.965	215
TRINITY_Contig6043	2	p6	(GGCGAT)3	18	4747	4764	AGGCTCCTGATGCCTCCTAA	60.031	TGCCAAGAGTACTGCACAGG	59.965	215
TRINITY_Contig6044	1	c	(AGC)4cggagct(GAA)4	31	1182	1212	GGTCATCTGCTACATCCCGG	59.966	GGTCACGGACATGGTGTTCT	59.965	112
TRINITY_Contig6045	1	p4	(CTCC)3	12	47	58	GCTGGACTTGGCTGACTCTT	59.964	GCTAGACGGCTTCATCCTCC	59.967	138
TRINITY_Contig6047	1	p2	(AG)7	14	1034	1047	GGCCGACATGCAATTGCTAG	59.97	TGGCAGTGCACTCAGGTTAG	59.965	270
TRINITY_Contig6047	2	p3	(CTG)6	18	2078	2095	GGCCGACATGCAATTGCTAG	59.97	TGGCAGTGCACTCAGGTTAG	59.965	270
TRINITY_Contig6047	3	p3	(CGG)5	15	2255	2269	GGCCGACATGCAATTGCTAG	59.97	TGGCAGTGCACTCAGGTTAG	59.965	270
TRINITY_Contig6047	4	p6	(TGCTGG)3	18	2826	2843	GGCCGACATGCAATTGCTAG	59.97	TGGCAGTGCACTCAGGTTAG	59.965	270
TRINITY_Contig6048	1	p3	(AGC)5	15	466	480	CAACGAAAGACTTGGCAGCC	60.04	CGGGCCCTTTGGATCTTTCT	60.034	166
TRINITY_Contig6049	1	p4	(ACGG)3	12	452	463	GCCGCTGCCCTAATTTAACG	59.971	TGCGTGTCTTGCATGTTTGG	59.969	157
TRINITY_Contig6049	2	p3	(CGG)4	12	3362	3373	GCCGCTGCCCTAATTTAACG	59.971	TGCGTGTCTTGCATGTTTGG	59.969	157
TRINITY_Contig6050	1	p4	(TGGC)3	12	3220	3231	CCAGTTCGAACCAGACGTGA	59.968	TGTGGTTGCTGGTTGAAGGT	60.034	130
TRINITY_Contig6051	1	p4	(CAGA)4	16	30	45	GTCCAAGAAGAAGGACCGCA	59.965	AGCTTCCTGCACATCAGCAT	60.034	145
TRINITY_Contig6051	2	p6	(AGGGAA)3	18	154	171	GTCCAAGAAGAAGGACCGCA	59.965	AGCTTCCTGCACATCAGCAT	60.034	145
TRINITY_Contig6051	3	p3	(GCC)4	12	552	563	GTCCAAGAAGAAGGACCGCA	59.965	AGCTTCCTGCACATCAGCAT	60.034	145
TRINITY_Contig6051	4	p4	(AGGC)3	12	1521	1532	GTCCAAGAAGAAGGACCGCA	59.965	AGCTTCCTGCACATCAGCAT	60.034	145
TRINITY_Contig6053	1	p3	(CGC)4	12	37	48	ACATTGTTTTCCTGCGTGCC	59.968	AGGATTGAACTTCCGCGGTT	59.964	203
TRINITY_Contig6053	2	p3	(AGA)4	12	3635	3646	ACATTGTTTTCCTGCGTGCC	59.968	AGGATTGAACTTCCGCGGTT	59.964	203
TRINITY_Contig6054	1	p4	(CTGA)4	16	291	306	TTCTGCAAAACGCTCACTGC	59.971	GACTGCATCCCCTGAAACCA	59.962	255
TRINITY_Contig6056	1	p3	(TCT)4	12	571	582	CCTCAGACTGCTTCTTGGCA	59.964	GTTAGCAAGGCCCCCAAGAT	60.033	109
TRINITY_Contig6056	2	p3	(GGC)6	18	1225	1242	CCTCAGACTGCTTCTTGGCA	59.964	GTTAGCAAGGCCCCCAAGAT	60.033	109
TRINITY_Contig6057	1	p3	(GGC)5	15	2495	2509	GCCTATCTGCCTGCCTAGTG	59.965	AAACAGCTGAGACGACTGGG	59.966	111
TRINITY_Contig6057	2	p3	(GAG)4	12	2663	2674	GCCTATCTGCCTGCCTAGTG	59.965	AAACAGCTGAGACGACTGGG	59.966	111
TRINITY_Contig6058	1	p3	(TCA)7	21	1677	1697	CGTCCATATCCTCGCCTTCC	60.038	CGAGGACGTGTACGAGTACG	59.976	155
TRINITY_Contig6059	1	p2	(CG)6	12	235	246	TGTTGAGCTCGTGTCTGGTC	59.968	CCTTCACACGTGGCAGAGAT	60.037	128
TRINITY_Contig6064	1	p3	(GCC)4	12	2907	2918	AAGACGCATCAACCTGCTCA	59.965	CACACTGGACCAAAGCAAGC	59.969	276
TRINITY_Contig6065	1	p3	(CGT)4	12	84	95	AGGAGCATGCAATCCGACAA	60.035	GTTCAAAAGACCACGGCACC	59.97	109
TRINITY_Contig6066	1	p3	(TCA)5	15	3052	3066	ATCCTCCTGGCCTTCCTCAT	60.029	TTATGCTCAGGTGCCCAAGG	60.034	134
TRINITY_Contig6068	1	p3	(GAC)4	12	2101	2112	TAGGGTTAAGGGAGGGGACG	60.032	TAGTTGACAGGAACACGCCC	59.966	268
TRINITY_Contig6074	1	p3	(CGG)7	21	1082	1102	CAATTCAACCAGGCCACAGC	60.038	AGCCTGCCTTTTTGCCATTG	59.964	170
TRINITY_Contig6075	1	p3	(GCC)5	15	363	377	CATTGTCGTACGCAAGCGAG	59.976	GAACACTGAAGGCAGCAAGC	60.04	185
TRINITY_Contig6075	2	p3	(CTC)4	12	691	702	CATTGTCGTACGCAAGCGAG	59.976	GAACACTGAAGGCAGCAAGC	60.04	185
TRINITY_Contig6076	1	p3	(CGC)5	15	2820	2834	GCTACTACCGCACCCTCATC	59.968	CTGCTCGAGCTCGTAGAAGG	59.971	248
TRINITY_Contig6077	1	p3	(GAA)14	42	18	59	TGTTTTCGCTGCGTTCTTGG	59.971	TTGAGGGCATCTTCGGCATT	60.034	141
TRINITY_Contig6077	2	c*	(CGCC)2(CGCCGCCGCCGCCGCCGCCACAATGCTCGGCTTCACCGCCGCAGGATCCTGCCTTTCCTCTCCGGCACCGCCGAGACACACGGCCGGGACGGCGCCTCCCCTCGTCCGCATCCTTCCCCTCACCTCCCACGGCCGCCGCCGCCCGTGCCGTGCCAGGCTCCTCCGGCCGCTCGGGGCGGCCCCCACCACGCCGCCGCCCGCGCCGTCCTCGCGCCCGGCGAGGGACCGCGTGATCGAGTTCGGCAAGCACAAGGGCCAGATGCTGGGCACGCTGCCGCCGTCGTACCTGCGCTGGGTCGCGGCGGAGCTCGACTACGGGGACACCGCGCCGTGGGCTGGCCTCGCGCGCGACGTCCTCGACGACCCCGTCTACGTCGACCGCGTCGAGTGGGAGCACGCGCACCGCTTCCTCCGCGGCGACGCCGACTACGAC	25	573	597	TGTTTTCGCTGCGTTCTTGG	59.971	TTGAGGGCATCTTCGGCATT	60.034	141
TRINITY_Contig6077	3	p3	(GCC)4	12	713	724	TGTTTTCGCTGCGTTCTTGG	59.971	TTGAGGGCATCTTCGGCATT	60.034	141
TRINITY_Contig6078	1	p2	(AC)8	16	1792	1807	TTTACTGCCTCCTGCCCATG	60.034	CTCTCCCTGGTCATCCTCCA	60.032	137
TRINITY_Contig6080	1	c	(CGC)4cgtgctccgcctgctcctccaggcgcagcaaccgcgcctcctcctccgccgccgtggcagcgggaa(CCG)4	90	1224	1313	ATCTGTCAACGAGGGCACTG	60.037	AGGAGGAAGAGGGCTCAGAG	60.032	168
TRINITY_Contig6081	1	p4	(CCAG)3	12	130	141	AGCCTTCTCCCGAGTCTTCT	59.96	CCAGCCCCAAATTCACCTCT	59.96	215
TRINITY_Contig6081	2	p3	(CCG)4	12	257	268	AGCCTTCTCCCGAGTCTTCT	59.96	CCAGCCCCAAATTCACCTCT	59.96	215
TRINITY_Contig6081	3	p3	(CCG)5	15	377	391	AGCCTTCTCCCGAGTCTTCT	59.96	CCAGCCCCAAATTCACCTCT	59.96	215
TRINITY_Contig6081	4	p4	(ACAA)3	12	1769	1780	AGCCTTCTCCCGAGTCTTCT	59.96	CCAGCCCCAAATTCACCTCT	59.96	215
TRINITY_Contig6082	1	p3	(CGA)4	12	383	394	TCAACCCTTTCGACGACTGG	59.967	TTGATCTTGAGCCACCCGAC	60.036	271
TRINITY_Contig6083	1	c	(CCGA)3ccccagcggcgcaccccaccag(CCA)4	46	15	60	GTCCATGCTCAGCCTCTACC	59.894	CGGACAGGGAGGAATCATCG	59.967	183
TRINITY_Contig6083	2	p3	(ACC)4	12	182	193	GTCCATGCTCAGCCTCTACC	59.894	CGGACAGGGAGGAATCATCG	59.967	183
TRINITY_Contig6083	3	p3	(TCG)4	12	352	363	GTCCATGCTCAGCCTCTACC	59.894	CGGACAGGGAGGAATCATCG	59.967	183
TRINITY_Contig6084	1	p3	(AGA)5	15	193	207	AAGATGTCCTCGTGCTCGTG	60.109	AGAAGCTGGAGGGCAATGAC	60.035	166
TRINITY_Contig6084	2	p3	(GCG)4	12	519	530	AAGATGTCCTCGTGCTCGTG	60.109	AGAAGCTGGAGGGCAATGAC	60.035	166
TRINITY_Contig6084	3	p3	(CCG)4	12	1260	1271	AAGATGTCCTCGTGCTCGTG	60.109	AGAAGCTGGAGGGCAATGAC	60.035	166
TRINITY_Contig6085	1	p3	(GGT)4	12	1224	1235	AAACCAAACCCCCTGCTTGA	60.032	GGTTTTGTTGACCGGCCTTC	59.968	266
TRINITY_Contig6085	2	p3	(CGC)6	18	1389	1406	AAACCAAACCCCCTGCTTGA	60.032	GGTTTTGTTGACCGGCCTTC	59.968	266
TRINITY_Contig6085	3	c	(CCT)5cccgccgcagca(GCC)4	39	2095	2133	AAACCAAACCCCCTGCTTGA	60.032	GGTTTTGTTGACCGGCCTTC	59.968	266
TRINITY_Contig6088	1	p3	(CCG)4	12	1693	1704	AACCAAAATTCTGCCGGTGC	59.967	ACCGAGGGAAGCTTGACAAG	59.965	125
TRINITY_Contig6090	1	p5	(CCGGG)3	15	2373	2387	AGGGAGCATCAGCAAAGCAT	60.033	CTTCAGCCAGGAATCAGGCA	60.035	204
TRINITY_Contig6092	1	p4	(CTAG)5	20	59	78	GTGAGGCCATCTGCAAGTCT	60.036	GATCGGTCCAAGAGGCACAA	60.036	270
TRINITY_Contig6092	2	p3	(CAG)4	12	1496	1507	GTGAGGCCATCTGCAAGTCT	60.036	GATCGGTCCAAGAGGCACAA	60.036	270
TRINITY_Contig6092	3	p3	(GGC)4	12	2206	2217	GTGAGGCCATCTGCAAGTCT	60.036	GATCGGTCCAAGAGGCACAA	60.036	270
TRINITY_Contig6093	1	p3	(GAG)4	12	56	67	GCCCGCTGATGGCTAGTAAT	59.965	TTGGAGCCCATGGTAAGCAG	60.034	124
TRINITY_Contig6093	2	p3	(GGC)6	18	184	201	GCCCGCTGATGGCTAGTAAT	59.965	TTGGAGCCCATGGTAAGCAG	60.034	124
TRINITY_Contig6093	3	p3	(GCG)4	12	347	358	GCCCGCTGATGGCTAGTAAT	59.965	TTGGAGCCCATGGTAAGCAG	60.034	124

TRINITY_Contig6093	4	p5	(CGCCG)3	15	553	567	GCCCGCTGATGGCTAGTAAT	59.965	TTGGAGCCCATGGTAAGCAG	60.034	124
TRINITY_Contig6094	1	c	(GAG)4ggtggggaggagatggtggccgagacgggctcgacgcgggttggggttttggt(GGC)6	83	1439	1521	CTGGCCAATAGTTCCTGCCA	60.034	TCTCCCTACCAGTACCGTGG	60.034	252
TRINITY_Contig6098	1	p3	(GGC)5	15	1051	1065	CGAAATCGAACAGCCTCCCT	60.108	TTCGACCGCTACCAGGAGTA	60.035	148
TRINITY_Contig6100	1	p2	(CG)6	12	4000	4011	AGGATCGATTGCTTGCCCAA	60.034	GTGCAACTATCGCGCGAAAT	59.974	104
TRINITY_Contig6101	1	p4	(AAGA)3	12	15	26	CAGTGGAGAAGATGGCCGTT	60.036	TCCTTCAGTTACAACGCCCC	59.964	259
TRINITY_Contig6104	1	p3	(CAA)4	12	1596	1607	TCACCACTCAACGCTGTCTC	59.968	TTGGCACCTCTTAGCTCTGC	60.036	161
TRINITY_Contig6108	1	p3	(CGC)4	12	115	126	GAATCGGGGAGAGCTCGAAG	59.969	TGGCATCAGCATAGTGCCAA	60.034	200
TRINITY_Contig6108	2	p3	(GCC)4	12	288	299	GAATCGGGGAGAGCTCGAAG	59.969	TGGCATCAGCATAGTGCCAA	60.034	200
TRINITY_Contig6109	1	c	(CCG)5cccgcggcgtccaccggcgccaccggcttctccattatcaactccatcaccacagttagctagcta(CGTG)3	93	2696	2788	CGAAGATCATGGCGACGAGA	59.971	GCGCTACCTGTCCTTCAACT	60.037	140
TRINITY_Contig6110	1	p3	(CTG)4	12	2375	2386	ATCTTCTGCTGCGAGTGCTT	60.037	CACGGCGTTGGTCAAAACAT	59.97	234
TRINITY_Contig6111	1	p3	(GCC)4	12	172	183	TCGTGTGACTCTCAAGCCAC	59.968	CAGGATTCCAACCGGCTTCT	60.035	256
TRINITY_Contig6111	2	c	(GCTC)3gcctcgcgcgtgccgccaccgccagcctcccccacctcgcgcctagtagttcccccgcctccacctgtcgctcgacgcaccc(GAGG)3	107	371	477	TCGTGTGACTCTCAAGCCAC	59.968	CAGGATTCCAACCGGCTTCT	60.035	256
TRINITY_Contig6113	1	c	(TTGG)3(CTT)4	24	874	897	TCTCACTTGTCTGCAGCAGG	59.966	TGTCCACCGTTGTAACTCGG	59.968	152
TRINITY_Contig6114	1	p3	(CGC)4	12	242	253	TGGTTGTCGCTTCAGGTACC	59.966	GGATCTGCTGCTCTCGAGTC	59.969	263
TRINITY_Contig6115	1	p2	(TC)14	28	122	149	CTCATCTCCCTTCTCCCCCA	60.031	GTGATTTCAATCCTCGGCGC	59.972	205
TRINITY_Contig6116	1	c*	(CTC)3(CT<C>TCGCC)(CTCGCC)3ctccccagtcccacctcccaccgccgcgccaccggcccggtcgcccacctcctc(CCCT)3	102	158	259	GCGCAACACTTGTACGTTGT	59.973	AAGATCAGCAGCGTGGGAAA	59.963	176
TRINITY_Contig6116	2	p3	(GCC)5	15	416	430	GCGCAACACTTGTACGTTGT	59.973	AAGATCAGCAGCGTGGGAAA	59.963	176
TRINITY_Contig6117	1	p3	(GGA)4	12	309	320	GTGCCTTCCTGTTGCACTTG	59.969	CGCTCCTCTCCGTCAAGATC	59.97	185
TRINITY_Contig6117	2	c	(CTT)4catcattatca(TCG)4	35	577	611	GTGCCTTCCTGTTGCACTTG	59.969	CGCTCCTCTCCGTCAAGATC	59.97	185
TRINITY_Contig6117	3	p2	(AG)6	12	3821	3832	GTGCCTTCCTGTTGCACTTG	59.969	CGCTCCTCTCCGTCAAGATC	59.97	185
TRINITY_Contig6119	1	p3	(GCG)4	12	198	209	TCGCCTTTTCCCCACTCTTC	59.964	CATTTCAGCTCCGAGGTGGT	60.036	117
TRINITY_Contig6121	1	p6	(CCTCGC)3	18	341	358	GGTCTCGGAGTTGCTATCCG	59.969	GGAGGGGAAGACCGAAACAG	60.036	102
TRINITY_Contig6122	1	c	(CTCAC)4ctccgtcccccattgcaaggctgcttctgctgtccccccccccccc(TC)6tagattccaatactttcctctgttcctgggcaaaccccatcatgaggtgaggggagggggaggcgctgcccagtctactctagcctgggtctg(GA)6	184	186	369	CTTCGAGAGCTCCGTCATCC	59.97	AGCGCTGGATCTTGAAACCA	59.963	131
TRINITY_Contig6125	1	p4	(GGCG)3	12	18	29	TTGAGCAACTTTGGCGCTTC	59.969	AGGATTGAAGCTGGCCTGAC	60.035	106
TRINITY_Contig6127	1	p3	(GAC)4	12	2563	2574	GAGCCGGTCAAACACTCAGA	59.966	AGTCGGAGCCATCTACCTGT	60.033	213
TRINITY_Contig6127	2	p3	(CCG)5	15	2682	2696	GAGCCGGTCAAACACTCAGA	59.966	AGTCGGAGCCATCTACCTGT	60.033	213
TRINITY_Contig6128	1	p3	(CCG)5	15	2757	2771	CTTGCGAAAACAGCACGTGA	59.973	TCGACCGGCAGAAACAATGA	59.966	231
TRINITY_Contig6129	1	p3	(GCG)5	15	41	55	AATGCCCCAAGGTGGAAGAG	59.96	GTTTCAGCGGTGCCTTCTTG	60.04	173
TRINITY_Contig6131	1	p3	(GGA)4	12	362	373	ACAACATTACCCCTGCCGAG	60.035	CCCCCGAGTTTCAGCAGATT	60.035	252
TRINITY_Contig6134	1	p3	(ATC)5	15	199	213	CAAAGTCGCCTAATGCTCGC	59.973	CCCGTTTCAGTTCCTTCCGA	59.966	234
TRINITY_Contig6135	1	p3	(CCA)5	15	42	56	GACACCTTCTTCCTGTCGCA	59.966	ACTTGAGCTGGTACATGGGC	60.035	238
TRINITY_Contig6135	2	p3	(GGA)6	18	196	213	GACACCTTCTTCCTGTCGCA	59.966	ACTTGAGCTGGTACATGGGC	60.035	238
TRINITY_Contig6140	1	p4	(GCCA)3	12	2	13	ACCTGCTGGACTGGCATTAC	60.035	AGAGCCGATTTGACCCTTGG	60.035	135
TRINITY_Contig6140	2	p5	(TACTC)3	15	244	258	ACCTGCTGGACTGGCATTAC	60.035	AGAGCCGATTTGACCCTTGG	60.035	135
TRINITY_Contig6140	3	p3	(GAT)4	12	969	980	ACCTGCTGGACTGGCATTAC	60.035	AGAGCCGATTTGACCCTTGG	60.035	135
TRINITY_Contig6141	1	p4	(ATCC)4	16	226	241	GGCAACCATCGGTGTAGTGA	60.037	AGAACGACCTCAAGCCATGG	60.036	228
TRINITY_Contig6143	1	p3	(TCA)4	12	2165	2176	AACAGCATAGCCAGGCACAT	60.034	CTCAGGCCTCCCCTTTTCAG	60.035	176
TRINITY_Contig6145	1	c	(GGA)4ggcttcctggtgctgaccactgacgcctggtatgg(TCGC)3	59	590	648	AACCTTCTCCCTGCTGATGC	60.035	CTACCCCTGCTGCTGAACAA	59.963	100
TRINITY_Contig6146	1	p3	(GGC)6	18	2047	2064	CAAAACCCAACACGACCGTC	59.972	AGAAGGAACCCTGTGCTTCG	59.965	280
TRINITY_Contig6146	2	p4	(TCCC)3	12	2211	2222	CAAAACCCAACACGACCGTC	59.972	AGAAGGAACCCTGTGCTTCG	59.965	280
TRINITY_Contig6148	1	p3	(TGA)4	12	1630	1641	CTGACGAACTAGCCGCATCT	59.899	GTGCTGCTTCTTCCCCATCT	60.035	179
TRINITY_Contig6149	1	p3	(ATG)4	12	3750	3761	ACGGGTGAGGCTATGTACCT	60.032	CTAGTGATGCTGCCGAGGAG	59.968	262
TRINITY_Contig6150	1	p4	(CCTC)4	16	98	113	TGGATGAGCTGGCAACCATT	59.96	ACCGCTTCGCTACCAAGAAA	59.966	109
TRINITY_Contig6151	1	p2	(CA)7	14	1373	1386	ACATGTGCGTCTCTGCTTGA	59.966	GTTTGTGGTGGAGCGTTCAC	59.971	105
TRINITY_Contig6158	1	p3	(CGT)4	12	426	437	TGGAAACTTCTCCCCACACG	59.892	CTGTGGTGTGTGCATATGCG	59.903	161
TRINITY_Contig6159	1	p6	(GAGGAC)3	18	5563	5580	CACCATAAGCCCTAGCCTCG	59.966	ATTCAGACGACAAAGGGCGT	59.965	152
TRINITY_Contig6159	2	p3	(CGC)4	12	5750	5761	CACCATAAGCCCTAGCCTCG	59.966	ATTCAGACGACAAAGGGCGT	59.965	152
TRINITY_Contig6160	1	c	(CGA)4ggccgacggtgaaccgcggttgggca(GCG)5	53	309	361	GAAGCTGTACGACTTGCCCT	60.037	TCGAGGTGGTTGTTCTGAGC	59.966	157
TRINITY_Contig6161	1	p6	(CCATCC)3	18	15	32	CCTATTTGCGGCTGCCATTC	59.969	TTGTACACCTTCCCGGATGC	60.036	204
TRINITY_Contig6161	2	p6	(GTGCCG)4	24	137	160	CCTATTTGCGGCTGCCATTC	59.969	TTGTACACCTTCCCGGATGC	60.036	204
TRINITY_Contig6161	3	p3	(CCT)4	12	263	274	CCTATTTGCGGCTGCCATTC	59.969	TTGTACACCTTCCCGGATGC	60.036	204
TRINITY_Contig6163	1	p3	(CGC)4	12	278	289	AGGCAACCGCTATGTCGAAA	60.037	TTTTGGCTGATCCGGCTGAT	60.034	265
TRINITY_Contig6164	1	p3	(CCG)4	12	212	223	GACAGGTGAGGAGACGCAAA	59.966	TTTCGCATGCAGCAGCTTTT	59.968	200
TRINITY_Contig6165	1	p3	(GGA)4	12	489	500	ATTGGAGGAGGAGGATCCCC	60.104	CTTCACAACGCTGCGACAAA	59.973	120
TRINITY_Contig6166	1	p3	(CAT)5	15	2240	2254	GCAGTTTCCGGGCACAAAAT	59.967	AGTCACCGAGCAAAACCGAT	59.965	118
TRINITY_Contig6167	1	p3	(TCG)4	12	2223	2234	TCGTCGTCGTCGTCATCATC	59.974	AGGAGAAGGAGGAGGAGCTG	60.032	127
TRINITY_Contig6169	1	p4	(CCTC)3	12	101	112	AACCCACACCACACCCAAAT	60.032	TTCCCACATCGAGGAGGACT	59.959	251
TRINITY_Contig6169	2	p3	(GCG)4	12	437	448	AACCCACACCACACCCAAAT	60.032	TTCCCACATCGAGGAGGACT	59.959	251
TRINITY_Contig6171	1	p4	(GCTC)3	12	149	160	GTACTCATCCACTACCCGCG	59.97	GGAGATGAACCCCCTGTTGG	60.034	243
TRINITY_Contig6172	1	p3	(GCT)4	12	971	982	CTGCGGTTTGCCATAGATGC	59.97	TGCTGCTCTCCCATTGAAGG	60.035	132
TRINITY_Contig6172	2	p6	(TGCTGT)3	18	1183	1200	CTGCGGTTTGCCATAGATGC	59.97	TGCTGCTCTCCCATTGAAGG	60.035	132
TRINITY_Contig6173	1	p4	(GAAA)3	12	1353	1364	CCCACCCAAGCGGATTTACT	60.034	ACTGGCTCTGAAAGGGCATC	60.035	188
TRINITY_Contig6173	2	p4	(TAAA)3	12	1668	1679	CCCACCCAAGCGGATTTACT	60.034	ACTGGCTCTGAAAGGGCATC	60.035	188
TRINITY_Contig6177	1	p3	(GGC)4	12	489	500	CTGCTCCACCAGGATGATCC	59.892	GGTTGGGGAAGAGAAGGTGG	59.962	223
TRINITY_Contig6177	2	p3	(CGT)4	12	857	868	CTGCTCCACCAGGATGATCC	59.892	GGTTGGGGAAGAGAAGGTGG	59.962	223
TRINITY_Contig6177	3	c	(CGA)5cgccgggccgtaaccccgcacggtggg(CGC)4	54	1152	1205	CTGCTCCACCAGGATGATCC	59.892	GGTTGGGGAAGAGAAGGTGG	59.962	223
TRINITY_Contig6179	1	p4	(AGGG)3	12	1456	1467	GCAAAGAAACAAGCCCTGGG	59.966	CAGTCTCGGCCTGTTGGATT	60.036	266
TRINITY_Contig6181	1	p5	(CGCGG)3	15	751	765	GCAAAATCCGCTCGATTCCC	59.971	AGAGCTAGGAGTTCGACGGT	60.035	188
TRINITY_Contig6184	1	p4	(CGGC)3	12	11	22	CAGCCTTTCAAACCACACCG	59.969	GTCGCGAGGAGGAGAATGAG	59.97	111
TRINITY_Contig6185	1	p5	(GGCGC)3	15	504	518	CCTGAATCCCCCAAATCCCC	60.106	CTCCGAAACCCTAGATCCGC	59.968	163
TRINITY_Contig6188	1	p3	(AGC)4	12	750	761	GATCGGAGCTCCTCAGAACG	59.97	TACACGCTGGTCTACGAGGA	60.036	112
TRINITY_Contig6188	2	p3	(GCA)5	15	1029	1043	GATCGGAGCTCCTCAGAACG	59.97	TACACGCTGGTCTACGAGGA	60.036	112
TRINITY_Contig6189	1	c	(CGC)5cgtcgcaaaccataaaaaaccccccaacacccctctccgccggcgaaaccgggccg(GGC)4	84	39	122	TTTGGCCTGTTGGTCTGGTT	60.033	CCGATAGTCCTTCCGAACCG	59.97	100
TRINITY_Contig6191	1	p6	(GCCACT)3	18	711	728	CTTCCTCTCCCTCTCCCTCC	60.106	TGATAGTGGCAGTGGCAGTG	60.036	173
TRINITY_Contig6191	2	p4	(GCGG)4	16	980	995	CTTCCTCTCCCTCTCCCTCC	60.106	TGATAGTGGCAGTGGCAGTG	60.036	173
TRINITY_Contig6192	1	c	(ACC)6ccgcaccctcctcctcgcccctcgcca(CCG)5	60	293	352	AGAAGGCGCGTGTGAAAGTA	59.967	GCATGCAAGTGCTTGTCCAA	59.968	229
TRINITY_Contig6194	1	p3	(TCA)4	12	56	67	GTGTAGTAGTCGTACCGGCG	59.973	GGACTTTTACCCCGTGGAGG	60.036	135
TRINITY_Contig6194	2	p3	(GCC)4	12	1911	1922	GTGTAGTAGTCGTACCGGCG	59.973	GGACTTTTACCCCGTGGAGG	60.036	135
TRINITY_Contig6197	1	p4	(GTGA)3	12	55	66	CAGTCTTTGCTGCCCTGTTTC	60.001	AGACAAGACCTCAAGCAGCC	59.964	144
TRINITY_Contig6198	1	p4	(GTTG)4	16	1510	1525	CACACACGACGTCACTCTCA	59.971	CAAAAACTGGGGCGATGCAA	59.967	255
TRINITY_Contig6199	1	p3	(CAT)4	12	411	422	GATCTCCAAGAGGCGACGAG	59.97	GTCGGGGCGCAATAAAATCC	59.97	112
TRINITY_Contig6199	2	c	(GCC)4atc(GAG)4	27	2426	2452	GATCTCCAAGAGGCGACGAG	59.97	GTCGGGGCGCAATAAAATCC	59.97	112
TRINITY_Contig6199	3	p5	(TTTTC)3	15	2747	2761	GATCTCCAAGAGGCGACGAG	59.97	GTCGGGGCGCAATAAAATCC	59.97	112
TRINITY_Contig6200	1	p2	(AT)7	14	241	254	AGCCCCAGAAAGAACAGCTC	59.963	CTTCTTCCTCGCGCAATTCG	59.974	193
TRINITY_Contig6201	1	p3	(TTG)4	12	1552	1563	TGGAAGCTCAAAGGGTCGAC	59.965	AGCCTTGTACACGGTGTCAG	59.966	223
TRINITY_Contig6202	1	p3	(AGG)6	18	1202	1219	GCAGCGGCTATTTCTCAACG	59.973	TGGACAACTTCCTCATGGGC	59.962	139
TRINITY_Contig6202	2	p3	(CTG)5	15	1388	1402	GCAGCGGCTATTTCTCAACG	59.973	TGGACAACTTCCTCATGGGC	59.962	139
TRINITY_Contig6205	1	p5	(TTTTC)3	15	3005	3019	TCCCCAGGCAGATCAGATCA	60.031	TCCCTCACCCATGCCTGATA	60.03	159
TRINITY_Contig6208	1	p6	(GGGCGG)3	18	129	146	AGTACAACGTGTCGCTGGAG	60.039	GATCATCTGTCAGGGCTCCG	59.967	212
TRINITY_Contig6208	2	p4	(CCAC)3	12	253	264	AGTACAACGTGTCGCTGGAG	60.039	GATCATCTGTCAGGGCTCCG	59.967	212
TRINITY_Contig6208	3	p3	(GCG)5	15	1408	1422	AGTACAACGTGTCGCTGGAG	60.039	GATCATCTGTCAGGGCTCCG	59.967	212
TRINITY_Contig6208	4	c	(GA)7gcgtgtccaaggttaatttacacacacaaacactgtaa(ATTG)3	64	2041	2104	AGTACAACGTGTCGCTGGAG	60.039	GATCATCTGTCAGGGCTCCG	59.967	212
TRINITY_Contig6208	5	p4	(ATGT)3	12	2339	2350	AGTACAACGTGTCGCTGGAG	60.039	GATCATCTGTCAGGGCTCCG	59.967	212
TRINITY_Contig6211	1	p6	(GGCGCC)3	18	2081	2098	TTCATGGCGAATGTCCACCA	59.963	GAGTCAGAGGAGATGCTCGC	59.969	156
TRINITY_Contig6212	1	p3	(GAG)5	15	346	360	GTGGGAGAAGAACAGCGGAA	59.965	CCCCGATTCCGTGAAAGTGA	60.037	106

TRINITY_Contig6212	2	p4	(TTTG)3	12	1589	1600	GTGGGAGAAGAACAGCGGAA	59.965	CCCCGATTCCGTGAAAGTGA	60.037	106
TRINITY_Contig6213	1	c	(TG)7cgc(GA)8ggaagaaagtgagagctctgttgagaagc(AGCT)3	74	517	590	CCCCTCGACCTTTTGACGAA	59.966	CTCTGTGCCACACTGCAAAC	59.97	129
TRINITY_Contig6213	2	c	(GATC)3ggtggccgctgcggatggcgtgcgaggtgccaagcatggccctca(TC)21(AC)19	137	816	952	CCCCTCGACCTTTTGACGAA	59.966	CTCTGTGCCACACTGCAAAC	59.97	129
TRINITY_Contig6213	3	p2	(TA)7	14	2567	2580	CCCCTCGACCTTTTGACGAA	59.966	CTCTGTGCCACACTGCAAAC	59.97	129
TRINITY_Contig6216	1	p3	(TCC)4	12	182	193	CAGCAGCGGCATCTTTTCTC	59.902	AGGGCAGAAGGGTCATTTGG	59.96	117
TRINITY_Contig6216	2	p3	(CGC)4	12	777	788	CAGCAGCGGCATCTTTTCTC	59.902	AGGGCAGAAGGGTCATTTGG	59.96	117
TRINITY_Contig6217	1	p2	(CT)12	24	27	50	AGTGGATTTTTGTTCGCGCC	60.04	GCCCCTCGCTCATAGATCAC	60.038	130
TRINITY_Contig6217	2	p3	(CGG)6	18	259	276	AGTGGATTTTTGTTCGCGCC	60.04	GCCCCTCGCTCATAGATCAC	60.038	130
TRINITY_Contig6217	3	p3	(GAC)6	18	1598	1615	AGTGGATTTTTGTTCGCGCC	60.04	GCCCCTCGCTCATAGATCAC	60.038	130
TRINITY_Contig6219	1	p3	(CCT)4	12	150	161	GTTGCTTTGTCTGAGCGCAA	59.971	GCCAGGTTTCAGTGCTCTCT	59.964	145
TRINITY_Contig6222	1	p3	(CGG)4	12	292	303	AACGAATTCCCCAATCCCCC	60.033	GCGCGCATAATACGAAGCAA	60.043	124
TRINITY_Contig6223	1	c	(CTT)4caaactacgacacgagcgaacgacgactcgacgagaacgaaag(ACT)4	67	167	233	TCTCCACACACGCCTTCTTC	59.966	CCTCCTGGCTCTTGCATCAA	60.035	137
TRINITY_Contig6223	2	c	(GCC)4gtagcca(CCG)5	34	415	448	TCTCCACACACGCCTTCTTC	59.966	CCTCCTGGCTCTTGCATCAA	60.035	137
TRINITY_Contig6224	1	c	(GCC)4accgtagccgccctcacggcgaccgccaccgtagccgccaccgcttccgccgtaa(CCG)7taacctccctggcgaccgccgccgta(GCC)5	129	1271	1399	AGGAGATGCGCATCCTCATG	59.965	CGGCAATCATCAAGCCTTCG	59.971	265
TRINITY_Contig6225	1	p3	(GGC)4	12	61	72	CAACCTGGCAAAGCACATCC	60.038	CGCGCTGTTTGATGACTACG	59.976	203
TRINITY_Contig6227	1	p3	(CCG)6	18	39	56	GCAGGATGAAACCAGGGACA	59.962	GGGCGTCGCAAAAGAACTTT	59.97	228
TRINITY_Contig6230	1	p3	(CCG)5	15	2170	2184	AGTCATTGCAGAACGCTGGA	59.965	GCAGCTGCTTCAAGGGAGTA	60.036	171
TRINITY_Contig6233	1	c	(GAAT)3gctctcaa(TC)8	36	1726	1761	TAGACAGGTGCCATGCCAAG	60.035	AAGTCGAGAATGCCACCTGG	60.036	145
TRINITY_Contig6236	1	p2	(GC)6	12	1244	1255	CAAGCAAGCTAGCAGGGTCT	60.036	TAGACTGGGACGGAGGAAGG	60.033	275
TRINITY_Contig6237	1	p5	(ACAAG)3	15	379	393	TACCCTCCCGATCGGAACTT	60.033	CCTCTCCTGCTGTTGCTTGA	59.964	107
TRINITY_Contig6238	1	p5	(ACAAG)3	15	410	424	GCTGTCGTGGAAGTCAAGGA	59.966	CCCGAAAGAGCTGCTTCAGA	60.037	175
TRINITY_Contig6239	1	p3	(GCC)4	12	499	510	CGATCCGCTGCAAACATGAG	59.973	GGGCTCAACCTACACCTGTC	60.036	145
TRINITY_Contig6240	1	p3	(CGC)4	12	618	629	ATCTCAGACTTTGGCCTGGC	60.035	TTTCTTCGCCCGGTCACTAC	60.038	182
TRINITY_Contig6241	1	p4	(AAAT)3	12	1926	1937	TTTGACAAAGCTGTGCTCGC	59.971	AGGAGGCCCCTCAAGATGAT	60.029	239
TRINITY_Contig6241	2	p2	(TC)6	12	3858	3869	TTTGACAAAGCTGTGCTCGC	59.971	AGGAGGCCCCTCAAGATGAT	60.029	239
TRINITY_Contig6243	1	p4	(GCCG)3	12	65	76	ACTCGCAGAAGGGCAAGAAG	60.321	GAGGATCAGGAAGGGGAGGT	60.031	161
TRINITY_Contig6243	2	p3	(CAG)6	18	301	318	ACTCGCAGAAGGGCAAGAAG	60.321	GAGGATCAGGAAGGGGAGGT	60.031	161
TRINITY_Contig6243	3	p3	(GCG)4	12	466	477	ACTCGCAGAAGGGCAAGAAG	60.321	GAGGATCAGGAAGGGGAGGT	60.031	161
TRINITY_Contig6243	4	p3	(CGA)5	15	687	701	ACTCGCAGAAGGGCAAGAAG	60.321	GAGGATCAGGAAGGGGAGGT	60.031	161
TRINITY_Contig6243	5	p4	(CGAA)3	12	1509	1520	ACTCGCAGAAGGGCAAGAAG	60.321	GAGGATCAGGAAGGGGAGGT	60.031	161
TRINITY_Contig6245	1	p6	(GCAGGA)3	18	471	488	TGGTTGGAAAGCACGCAAAG	59.898	GTCGGGAGCAGAAGATCGAG	59.97	168
TRINITY_Contig6248	1	c	(CTG)4cggcggcatgcaggtgccgctgttggtgctgcagtgcggctgcggatcggcgccggtggcgggctcgct(GGC)4	93	443	535	GGCAGCATACACGCAAACAA	60.04	TATCGGGTAACTGGGCTGGA	60.032	155
TRINITY_Contig6248	2	p3	(CCG)4	12	1390	1401	GGCAGCATACACGCAAACAA	60.04	TATCGGGTAACTGGGCTGGA	60.032	155
TRINITY_Contig6249	1	p4	(GCCC)3	12	2067	2078	TTGCTAGGTTCACCGACGAC	60.039	AGCACGATTTCATCCGTGGT	60.036	179
TRINITY_Contig6250	1	p3	(CCG)4	12	98	109	CTCGTGCGTTTTGCTGGAAA	59.971	CCGTCGGACTGGAAAAGGAA	59.966	255
TRINITY_Contig6252	1	c	(CGC)7ggatcgacgaag(GCC)4ggcgcggccactagctgtggcc(CTG)4	79	217	295	TAGCCTGATCCTGCTGGACT	60.032	ATCGACCTCTGAACGCGATC	59.97	124
TRINITY_Contig6252	2	c	(GGA)5cgaggacggaggctgctgc(TGG)4	46	1646	1691	TAGCCTGATCCTGCTGGACT	60.032	ATCGACCTCTGAACGCGATC	59.97	124
TRINITY_Contig6253	1	p5	(CAAAC)3	15	141	155	GCACCTCAAGAAGTCGGACA	59.966	AGCCGCATCTCCATGTTGAA	60.035	261
TRINITY_Contig6253	2	p3	(GCG)4	12	359	370	GCACCTCAAGAAGTCGGACA	59.966	AGCCGCATCTCCATGTTGAA	60.035	261
TRINITY_Contig6253	3	p3	(GCG)5	15	501	515	GCACCTCAAGAAGTCGGACA	59.966	AGCCGCATCTCCATGTTGAA	60.035	261
TRINITY_Contig6253	4	p6	(CGGCGC)3	18	1179	1196	GCACCTCAAGAAGTCGGACA	59.966	AGCCGCATCTCCATGTTGAA	60.035	261
TRINITY_Contig6257	1	p2	(GT)6	12	1783	1794	TGTGGGGAAGCAACAAGTGT	60.034	CACCATGCGCACCTTTTGAA	59.968	140
TRINITY_Contig6259	1	p3	(GGC)4	12	113	124	ACAGGTACATGCCACCAGTG	59.963	TTCCGCAGCTGCAAACAATC	60.039	209
TRINITY_Contig6260	1	p3	(CCG)5	15	2212	2226	TGGGATCATTAGGGGGAGCA	60.029	CACCAAGGGATGCACCTGAT	60.034	258
TRINITY_Contig6261	1	p3	(GGA)4	12	531	542	GAATGCATGCTCATGGGCAG	59.968	AAACAGGCTCAGAGGATGGC	60.035	117
TRINITY_Contig6262	1	p4	(GAGG)3	12	1458	1469	AGCATTCAGGGCTGCAAGAT	60.033	CATCCTCGTCAATCCCCTCG	59.967	155
TRINITY_Contig6263	1	p3	(CTC)4	12	1808	1819	CCCAACTCCTTGACGAGCTT	59.965	GCGCACAACTTCTGCTTTCA	59.971	279
TRINITY_Contig6265	1	c*	(GGAG)4(G<GAG>AGAGAGAGAGAGAGAGAGAGAGAGTTCTCGTCTACTACTTTCTCGAGAGTTCATTAACAAAAGGAAATTAACCGATGGATTATGACCAGCTAGCGAGCTAAGCGATGGAGCTGCTGGTGGTTTACTGGATGGAACGGTGGACGGACGTGTCACTGGTTTTGGCAGGTGCGAAGCTTCCAAGCGGCGCAATCGAGCGGAGGCCGCGCGCGTGCCATGCTTTCGCCATCGATCGAGAGCGGAGGCCGCACACAGGACAAGGAGGAATGCTAAGCTTGCTTGCATCGATCGAGGAGACGGATGGATGGCTACGTACCTTGCTCCTCCTCGGCGGCGGCGTGGCCGCCGGGGTGGTGCGGCGGCGCTGATTGTTGCTGGTGGCTCGTGGCGCCGGCGGTGCCGTAGAAGGCCTGCGGCGGGAGC	41	236	276	GACGTGTCACTGGTTTTGGC	59.971	CGTCTCCTCGATCGATGCAA	59.971	153
TRINITY_Contig6268	1	c*	(AG)5(<AG>C)(AGC)4	25	2011	2035	CTCCAGGGATCCCTTCTCCA	60.031	ACGACTGATGGGGTGTGTTC	59.965	183
TRINITY_Contig6269	1	c	(GCGG)3gggagggagcggc(GGAG)4	41	65	105	CTGGTCGACTCCAAGGACAC	60.038	AAACCCACCAACTCCAAGCA	60.033	183
TRINITY_Contig6271	1	c	(CGG)4tggccgcggcggcgcagccgcagg ggcgcggcccagcgcggcggcag(GGC)6	77	1016	1092	TGGCGGTGTTCTGGTACTTC	59.966	CATATCCATTGGCTCGCCCT	59.963	110
TRINITY_Contig6272	1	p4	(TTCC)3	12	3637	3648	TCCGTGTTGCCTCAAGGTAC	59.966	TTTCCTGCTGTGCCTTCCAA	60.106	269
TRINITY_Contig6273	1	p6	(GCGCCC)3	18	1911	1928	GCGATCGTAGTGCGGTAGAA	59.971	GGCAGCTAATCCGGTCCTAC	59.966	208
TRINITY_Contig6273	2	p3	(CGC)5	15	2086	2100	GCGATCGTAGTGCGGTAGAA	59.971	GGCAGCTAATCCGGTCCTAC	59.966	208
TRINITY_Contig6273	3	c	(CCG)4cagccgcagtacatcccgatgccgcagtacgtgccgcagccgtacccctgcaacccct(GCG)4	82	2259	2340	GCGATCGTAGTGCGGTAGAA	59.971	GGCAGCTAATCCGGTCCTAC	59.966	208
TRINITY_Contig6276	1	p3	(TGC)6	18	1268	1285	CCTCCATGTCTCCTCCTCCA	60.032	CGGTCACTTCTCCTCGATCG	59.972	178
TRINITY_Contig6277	1	p3	(ATA)4	12	36	47	CTAGCTGAGCTCGAGGAAGC	59.969	GCTCCTGTTGGATGAACCGA	60.036	250
TRINITY_Contig6280	1	p3	(CCT)4	12	327	338	CTGTGAGCATGTTGGGCAAC	60.039	CCCAAGGCGTCAAGTGTTTG	59.969	249
TRINITY_Contig6284	1	p3	(CCG)4	12	189	200	GCCCGGTGTTCTATCTCGAG	59.969	TCCCCATTCTCCAGGTCGAT	60.031	184
TRINITY_Contig6284	2	p6	(GAGGAA)3	18	1333	1350	GCCCGGTGTTCTATCTCGAG	59.969	TCCCCATTCTCCAGGTCGAT	60.031	184
TRINITY_Contig6286	1	p3	(GAC)4	12	317	328	TGAGAGGGCTGAGGCTACAT	60.032	GGTAGATGCTCTTCTCCGCC	59.967	239
TRINITY_Contig6286	2	p3	(GCG)5	15	987	1001	TGAGAGGGCTGAGGCTACAT	60.032	GGTAGATGCTCTTCTCCGCC	59.967	239
TRINITY_Contig6286	3	c	(CCGG)3cggggcaatgatgatatggttcttgatcccgtgggggcagcgacggcggggcgaataaatggaggagggggcacgggtggcggagccgagcctgcctg(GGGA)3agggaatctcgaacgagcggaagagagag(GGT)4	163	1445	1607	TGAGAGGGCTGAGGCTACAT	60.032	GGTAGATGCTCTTCTCCGCC	59.967	239
TRINITY_Contig6287	1	p3	(GGC)7	21	185	205	CTGCTACTCAATTCACGCGC	59.974	CTAGCTCTTCCTCCAAGGCG	59.896	269
TRINITY_Contig6288	1	p6	(GAAGAG)3	18	11	28	GACAGATAGCAGATCCCGGC	60.038	GGTGTAGGCGATTACGGGAG	59.968	104
TRINITY_Contig6290	1	p4	(GCGG)3	12	1402	1413	GGGCACATGCACAAACAGAG	60.039	GATTGGCCACGATGTGATGC	59.97	121
TRINITY_Contig6291	1	c	(CCG)6ctgccgcctatc(CCT)4	42	1581	1622	AGTAGCTCCTGGGGTAAGGG	60.031	AAAAAGGCGGACGAGGATGT	59.964	232
TRINITY_Contig6293	1	p5	(GGGGA)3	15	893	907	TCTTCGACGAGAGCGCATAC	59.972	GCTCCGTTCCCGAGGATTAG	59.968	269
TRINITY_Contig6293	2	p3	(GAA)4	12	1229	1240	TCTTCGACGAGAGCGCATAC	59.972	GCTCCGTTCCCGAGGATTAG	59.968	269
TRINITY_Contig6294	1	p3	(GCC)7	21	3505	3525	CTTTCGCTGCTGATGGATGC	59.971	ACATCTGACCTCCCTACGCT	60.033	157
TRINITY_Contig6295	1	p3	(GCG)5	15	2603	2617	TGGAGTAAGGGGGTGCCATA	59.955	CACGCCAGCCATTCAACAAA	59.968	173
TRINITY_Contig6298	1	p3	(GGC)4	12	955	966	TGAGGTTCCGTTTCTGGACG	59.967	CCCCAGTTCAGACCATGGAC	60.035	102
TRINITY_Contig6301	1	p3	(GCG)4	12	1727	1738	CTGCTGGGTAGTAGCCATCG	59.967	ATAAGAAGGTGGCAGCGACC	60.108	104
TRINITY_Contig6302	1	p3	(GGC)5	15	1480	1494	CGGCGTACAGATCCTTCTCC	59.969	GATCATCTCGATGTGCCGGT	59.967	197
TRINITY_Contig6302	2	p3	(GGA)4	12	2353	2364	CGGCGTACAGATCCTTCTCC	59.969	GATCATCTCGATGTGCCGGT	59.967	197
TRINITY_Contig6305	1	p4	(CGGC)3	12	18	29	CTGCTACTGCGACTACTCCG	59.972	CGTTGCGCTTGATGTACTCG	59.976	107
TRINITY_Contig6305	2	c	(GT)6actgcgatgggctggttcatccgtcctgttgctgatgctgctattatgaattgccgattcagtagtagtggtggtggtagtgt(TG)9	113	1743	1855	CTGCTACTGCGACTACTCCG	59.972	CGTTGCGCTTGATGTACTCG	59.976	107

TRINITY_Contig6306	1	p2	(CT)6	12	449	460	GGGGCTTCAACTTTTGGCAG	59.966	GCCAACGTTGCCCAAATTCT	59.967	213
TRINITY_Contig6306	2	p6	(CGGCGA)3	18	1647	1664	GGGGCTTCAACTTTTGGCAG	59.966	GCCAACGTTGCCCAAATTCT	59.967	213
TRINITY_Contig6306	3	p3	(TTC)5	15	2446	2460	GGGGCTTCAACTTTTGGCAG	59.966	GCCAACGTTGCCCAAATTCT	59.967	213
TRINITY_Contig6307	1	p3	(CCG)4	12	154	165	CAACTGCCTTGCAACAGCAT	59.967	TTCGTTGACGGCTGTGATCA	59.967	247
TRINITY_Contig6308	1	p2	(AG)30	60	1148	1207	TGGAGGAGGAGTCCACGTAG	60.034	GCAAGGAGAGAGAAAGGCGT	60.037	115
TRINITY_Contig6309	1	c*	(TCG)3(TC<G>GCGTG)(GGCGTG)2	29	65	93	CTCTTGCAGCTTCTCGGTCA	60.038	GCTCGCAGGTAACCCTTCTT	60.036	141
TRINITY_Contig6309	2	c	(CGC)5ggccggctcctcgacgtcatcggccaaggaagccttgacgtccaccgccggcagccccg(CGGCGT)4	98	376	473	CTCTTGCAGCTTCTCGGTCA	60.038	GCTCGCAGGTAACCCTTCTT	60.036	141
TRINITY_Contig6309	3	p3	(AGG)4	12	1389	1400	CTCTTGCAGCTTCTCGGTCA	60.038	GCTCGCAGGTAACCCTTCTT	60.036	141
TRINITY_Contig6310	1	p3	(GAA)4	12	2234	2245	CTTAGTGCTCGCCATCCGAT	59.967	CTGTTGGAGGCCGAGTTCTT	59.965	139
TRINITY_Contig6310	2	c	(CGC)4ggagtgggagcaggcagcagccagggggcggccgcgcctaccaccaccagaggctga(GAGC)3	81	2374	2454	CTTAGTGCTCGCCATCCGAT	59.967	CTGTTGGAGGCCGAGTTCTT	59.965	139
TRINITY_Contig6311	1	p6	(TCTTTT)3	18	671	688	TTGCTGTGATGCTGGTTTGC	59.968	TCCTTGACCTCCTATGCGGA	60.033	110
TRINITY_Contig6312	1	p4	(TCTG)3	12	846	857	GTGACCGTGACAAGCAGGTA	59.967	GTGTGTGTGAACTGCTGCTG	59.971	241
TRINITY_Contig6312	2	p3	(CAG)4	12	3155	3166	GTGACCGTGACAAGCAGGTA	59.967	GTGTGTGTGAACTGCTGCTG	59.971	241
TRINITY_Contig6312	3	p3	(GGA)5	15	3902	3916	GTGACCGTGACAAGCAGGTA	59.967	GTGTGTGTGAACTGCTGCTG	59.971	241
TRINITY_Contig6313	1	p4	(TGGC)3	12	854	865	GGCAGCATCAACTTGCACAA	59.968	AGCAACAAGACGGTGAGGAG	59.966	207
TRINITY_Contig6315	1	p3	(TTC)4	12	3431	3442	GGCTTCTCTCACTTGCCCAT	60.035	CGTGCGCTTTAAGGAGATGC	59.973	226
TRINITY_Contig6315	2	p3	(GGC)4	12	4500	4511	GGCTTCTCTCACTTGCCCAT	60.035	CGTGCGCTTTAAGGAGATGC	59.973	226
TRINITY_Contig6316	1	c	(GCA)5gcttcgcgcgcgtccaagcgg(GCC)5	50	399	448	GTACATGTGCCCACCCATGA	60.035	TATCACCATCCCGTCCCAGT	60.032	165
TRINITY_Contig6318	1	p3	(GGC)4	12	4289	4300	ATGCACACATCCGTCCACTT	59.963	ATGGCGGTCGTTGTTGTTTG	59.97	238
TRINITY_Contig6319	1	c	(CTCTG)3ccctctcgctctctcg(CT)12	55	2309	2363	TGATGGATGGTTGGGCCTTC	60.033	CTGGGGCAGTTCATCCATGT	60.034	169
TRINITY_Contig6320	1	p3	(GGT)6	18	594	611	AACCGGCTTCTTCTTCTCGG	60.037	GTGGTCGTGTTCGAGGACTT	59.968	228
TRINITY_Contig6320	2	p3	(TGC)5	15	1780	1794	AACCGGCTTCTTCTTCTCGG	60.037	GTGGTCGTGTTCGAGGACTT	59.968	228
TRINITY_Contig6321	1	p6	(CCCTCG)3	18	81	98	TCCATTCTTCCCTGCTGCAG	60.035	CGCAGTCAGATATCCGCCAT	60.038	129
TRINITY_Contig6324	1	p2	(GA)6	12	475	486	GCAGGCACGTTCTGGAATTC	59.831	CCTCTCTCTCCCCGTCTTGA	60.034	181
TRINITY_Contig6324	2	p3	(CCT)5	15	833	847	GCAGGCACGTTCTGGAATTC	59.831	CCTCTCTCTCCCCGTCTTGA	60.034	181
TRINITY_Contig6324	3	p3	(GCG)4	12	1279	1290	GCAGGCACGTTCTGGAATTC	59.831	CCTCTCTCTCCCCGTCTTGA	60.034	181
TRINITY_Contig6325	1	p4	(CTGT)3	12	583	594	TCTGTCTGTGGCTTCGTGTC	59.968	GCGATATCAGCACTCAGGCT	59.967	153
TRINITY_Contig6325	2	p6	(CGTCCC)3	18	2980	2997	TCTGTCTGTGGCTTCGTGTC	59.968	GCGATATCAGCACTCAGGCT	59.967	153
TRINITY_Contig6326	1	p2	(GC)6	12	373	384	AATCTGTGACCTCACTGCGG	60.037	ATGAAGTCGTTGGTGCCGAT	60.036	178
TRINITY_Contig6327	1	c	(GA)9ggcgtc(TGCG)3	36	36	71	TTGTGCTCAGTTGTGCTTGC	59.899	GAGCCATGACCCAGTCCTTC	60.107	143
TRINITY_Contig6328	1	p3	(GGC)4	12	405	416	CCTACGACTCGCTCTTCGTC	59.973	AGATGGATGGGCGTTGTTGT	59.962	261
TRINITY_Contig6328	2	p3	(GGC)4	12	555	566	CCTACGACTCGCTCTTCGTC	59.973	AGATGGATGGGCGTTGTTGT	59.962	261
TRINITY_Contig6328	3	p4	(ATCG)3	12	1565	1576	CCTACGACTCGCTCTTCGTC	59.973	AGATGGATGGGCGTTGTTGT	59.962	261
TRINITY_Contig6329	1	p3	(CGG)7	21	751	771	AGCTACCATCGGTCTGACCT	60.033	CCACCGTTACGCATCTGTCT	60.109	157
TRINITY_Contig6330	1	p6	(GTCGGA)3	18	1386	1403	TGACATTCTGAACGTCCGCA	59.967	CGCACGAGCAAGAAACCAAA	59.971	264
TRINITY_Contig6330	2	p3	(GCG)7	21	1800	1820	TGACATTCTGAACGTCCGCA	59.967	CGCACGAGCAAGAAACCAAA	59.971	264
TRINITY_Contig6331	1	p4	(GCGT)3	12	2207	2218	CGATTTCTCCTCGGCTCCTC	59.969	GTCTGAAGGGTACGTGTGCA	59.967	223
TRINITY_Contig6333	1	p4	(CCCT)3	12	155	166	CGAACGAGGCTATAGCTGCA	59.969	AGGGCTCTTGTATGTGGCAC	60.035	257
TRINITY_Contig6333	2	p3	(CAG)4	12	2186	2197	CGAACGAGGCTATAGCTGCA	59.969	AGGGCTCTTGTATGTGGCAC	60.035	257
TRINITY_Contig6334	1	p3	(GGC)4	12	2666	2677	CTTGGGTGGCAGGATGGATT	60.033	TGAGCTGCCTGAAGGGAAAG	59.963	212
TRINITY_Contig6335	1	p5	(CGTCT)3	15	3	17	CCCTACATGCGCTCACCTAG	59.967	CGATCATAAGCGGGGCTTCT	59.966	173
TRINITY_Contig6335	2	p3	(CCG)4	12	1656	1667	CCCTACATGCGCTCACCTAG	59.967	CGATCATAAGCGGGGCTTCT	59.966	173
TRINITY_Contig6336	1	p3	(AGC)6	18	989	1006	CGCATTGCTTGGCATAGTCC	59.97	ACAAGCTGTCGATTGGAGCA	59.965	231
TRINITY_Contig6337	1	c	(TCA)5atagtgttgtcttcaccaccatcatcatcagtgttgtcttcaccaccgtcatcatcagtgttgcgttcgtcatcatccttgacatct(TCA)4	114	1334	1447	TGGATGCCCCATCAGGTTTC	60.033	CAAGAGGAGTGCAGCCTTCA	59.964	149
TRINITY_Contig6337	2	p3	(CCT)4	12	1860	1871	TGGATGCCCCATCAGGTTTC	60.033	CAAGAGGAGTGCAGCCTTCA	59.964	149
TRINITY_Contig6338	1	c	(CCG)4cctctcctccacctgaactttcttctcttctgctgcgcca(CCG)4	64	23	86	GTACGAGTACGCGAGGAAGG	59.973	ATGACATTGTGGAAGGCGGT	59.962	270
TRINITY_Contig6338	2	c	(CCG)4cctctcctccacctgaactttcttctcttctgctgcgcca(CCG)4	64	240	303	GTACGAGTACGCGAGGAAGG	59.973	ATGACATTGTGGAAGGCGGT	59.962	270
TRINITY_Contig6339	1	p3	(GCC)4	12	310	321	GAGAAAGCTCACAGGGCCAT	60.035	CACCGCACTCCATGTACAGT	60.037	137
TRINITY_Contig6341	1	p2	(GA)16	32	1	32	AGGAGTACCTCAGCCTGGAG	60.033	CATCAGGTCGGAGTAGACGC	59.97	239
TRINITY_Contig6341	2	p3	(CAG)4	12	1253	1264	AGGAGTACCTCAGCCTGGAG	60.033	CATCAGGTCGGAGTAGACGC	59.97	239
TRINITY_Contig6341	3	p3	(GCA)7	21	1633	1653	AGGAGTACCTCAGCCTGGAG	60.033	CATCAGGTCGGAGTAGACGC	59.97	239
TRINITY_Contig6341	4	p4	(CATC)4	16	2014	2029	AGGAGTACCTCAGCCTGGAG	60.033	CATCAGGTCGGAGTAGACGC	59.97	239
TRINITY_Contig6341	5	c	(GAC)5cat(GGC)4	30	2319	2348	AGGAGTACCTCAGCCTGGAG	60.033	CATCAGGTCGGAGTAGACGC	59.97	239
TRINITY_Contig6342	1	p3	(GAT)4	12	1417	1428	TGTTGTGCAGCTCCCATGAT	59.961	CCCCAGGCACAGATGAACTT	59.961	144
TRINITY_Contig6346	1	p3	(CCT)6	18	143	160	CCTCGATCTCCTCCCGATCT	59.962	TATTGCCCTTCTCGATCGCC	59.966	226
TRINITY_Contig6350	1	p5	(TAGCC)3	15	1929	1943	TCTGAATGATGCTTGGCCGT	60.035	GCCTCAAGCGAGATCGATCA	59.969	114
TRINITY_Contig6350	2	p6	(CTGAAT)4	24	2069	2092	TCTGAATGATGCTTGGCCGT	60.035	GCCTCAAGCGAGATCGATCA	59.969	114
TRINITY_Contig6353	1	p3	(TCC)5	15	229	243	GGGAAGCCACTGGTCTTCTC	60.036	GCTGGGATGAGACCGATGAG	59.967	209
TRINITY_Contig6354	1	p3	(GTG)5	15	485	499	TGTCCTTGGTGCCAAAGTGT	60.034	CTGAGCTTGATCCTTGGCCA	60.035	265
TRINITY_Contig6355	1	p6	(TCGGGG)5	30	1070	1099	TCGGTGATGACCTTTGCTCC	60.036	TCGAGCTGGTGCAATGACAT	60.036	144
TRINITY_Contig6356	1	p3	(ACC)5	15	246	260	CTTTGCTTGAAGCTGCCAGG	60.039	GACCAGCTCCTTCTATCGCC	59.967	201
TRINITY_Contig6359	1	c	(CGC)4cggtgggtcactgaaggaggtgcgcaagggagt(GCG)4	57	792	848	AACTGAGCAGTAGTGGCGTC	60.038	TTGGAGAAGAGGGCAGCAAG	59.963	141
TRINITY_Contig6360	1	p3	(CTC)4	12	350	361	TTGCATCTCCAGCTCAAGGG	60.035	GAATGTGGACCCCAAGGAGG	60.034	205
TRINITY_Contig6361	1	p6	(GAGAGG)4	24	452	475	CTACTGCTCCATGGCCAACA	60.035	TTTTCTCCGCTTGCCTCCAT	59.962	140
TRINITY_Contig6371	1	p3	(GCG)4	12	2159	2170	GCGCATTTGCAGTGTTCTCA	60.04	ATGCCAGCAATTGCACAAGG	60.037	264
TRINITY_Contig6372	1	p2	(CG)6	12	480	491	GCACGCGTCTAGTACTCCTC	59.972	CGACCACAAGTTCGACCTCA	59.968	192
TRINITY_Contig6374	1	p3	(CGG)4	12	1503	1514	TCCAATTGCTGCTGGAGAGG	60.035	TCTGAACATGTTCCCCACCG	59.964	163
TRINITY_Contig6377	1	p5	(GGTCG)4	20	409	428	TTCATCATCTCTCTCGCGCC	59.969	GTTGCGGTGTTGCCTTTCAT	59.968	204
TRINITY_Contig6377	2	c	(AT)8gagtatatatactataattttgcatcgattagtga(AAAT)3	63	1670	1732	TTCATCATCTCTCTCGCGCC	59.969	GTTGCGGTGTTGCCTTTCAT	59.968	204
TRINITY_Contig6377	3	p4	(AAAT)4	16	2245	2260	TTCATCATCTCTCTCGCGCC	59.969	GTTGCGGTGTTGCCTTTCAT	59.968	204
TRINITY_Contig6377	4	p4	(TTGT)3	12	3044	3055	TTCATCATCTCTCTCGCGCC	59.969	GTTGCGGTGTTGCCTTTCAT	59.968	204
TRINITY_Contig6378	1	p5	(CGCCA)3	15	473	487	CGGACGGCTTCTACCTCATC	59.969	TGGAGTCAGCACGGATCCTA	60.034	219
TRINITY_Contig6379	1	p4	(TTTC)3	12	4238	4249	CGCCACCACTTCAGTCTTCT	59.966	GGGATCCACTCAGATGCTCG	59.967	119
TRINITY_Contig6383	1	c	(GCCTG)3a(CGCTC)3	31	39	69	GGGCTCGGCTACTTGGAATT	60.107	GTGGAGCTCCTTGGCAATCT	60.035	122
TRINITY_Contig6388	1	p4	(GGGA)3	12	1071	1082	CTCAGTGTCAGCTCCGGATC	59.898	AGAGCAGGGGGTCATCTTCT	59.956	119
TRINITY_Contig6391	1	p3	(CGA)4	12	2314	2325	CGTTGATCATGCTCTGTGCG	59.974	GTTGGCTTGAAGCTTGGTGG	59.967	107
TRINITY_Contig6393	1	p3	(TTA)4	12	53	64	ATAGCCACCTTGTGATGCCC	60.107	CATGCAAGAGTCGCATGAGC	59.972	109
TRINITY_Contig6394	1	p4	(TTTC)3	12	147	158	AGAGCTCAGCTTTTGTCGCT	59.965	CACGCCACCCACTTCAAAAG	59.969	116
TRINITY_Contig6399	1	c	(GGC)6acagcacagctcagctcactcagcgcgccgcggcacccgcag(GCCT)4	76	2048	2123	GTCGAACCTCATGTGCTCCA	60.037	AGGGGGTTTGACAAAGTGCA	60.033	184
TRINITY_Contig6399	2	p3	(GGT)7	21	2317	2337	GTCGAACCTCATGTGCTCCA	60.037	AGGGGGTTTGACAAAGTGCA	60.033	184
TRINITY_Contig6402	1	p5	(GACTC)4	20	309	328	ATGATTCCAAAGAGCGCCCA	60.034	CATGAGGTATGTTTGCGGCG	59.972	217
TRINITY_Contig6402	2	c	(GAGC)3tttgggcgaggtggttctcgagggaagctgctagaaggagggggacgaggagtgtggactgcgctttggcc(TTGCT)3ttgtgctgcctcgctttgcttagcc(TGGT)3	135	635	769	ATGATTCCAAAGAGCGCCCA	60.034	CATGAGGTATGTTTGCGGCG	59.972	217
TRINITY_Contig6402	3	p3	(GCG)4	12	979	990	ATGATTCCAAAGAGCGCCCA	60.034	CATGAGGTATGTTTGCGGCG	59.972	217
TRINITY_Contig6404	1	p4	(CTCG)3	12	91	102	GGAACCACGGCAGAGATCTG	60.461	TCACATCTATGCCTGCTGCC	60.179	241
TRINITY_Contig6404	2	p3	(CGG)4	12	390	401	GGAACCACGGCAGAGATCTG	60.461	TCACATCTATGCCTGCTGCC	60.179	241
TRINITY_Contig6408	1	p3	(AAT)4	12	8	19	AGGTCCTCCCATTCCGATCA	60.031	CGGTGATATGGGTGCTCCTC	59.966	205
TRINITY_Contig6408	2	p3	(CGC)4	12	2175	2186	AGGTCCTCCCATTCCGATCA	60.031	CGGTGATATGGGTGCTCCTC	59.966	205
TRINITY_Contig6410	1	p4	(TCCT)3	12	3541	3552	CAGGGCAGGGGATTTGGATT	60.032	TCCAATTCAGACCACCCAGC	59.962	231
TRINITY_Contig6410	2	p6	(CTGCCG)3	18	3673	3690	CAGGGCAGGGGATTTGGATT	60.032	TCCAATTCAGACCACCCAGC	59.962	231
TRINITY_Contig6411	1	p4	(GCAG)3	12	760	771	AATATCCGCCAAAATGCCGC	59.968	CTCTGCTGCCACAAAAGCTG	60.039	130
TRINITY_Contig6411	2	c	(CCG)6cccaaggaggagccccct(CCG)5	51	1747	1797	AATATCCGCCAAAATGCCGC	59.968	CTCTGCTGCCACAAAAGCTG	60.039	130
TRINITY_Contig6414	1	p4	(ATCC)3	12	2148	2159	GGGGAGAGAGAAGGGGGAAT	60.03	CTTGCTTGCTGAAAACCCCC	59.966	193
TRINITY_Contig6414	2	p4	(AGCA)3	12	4686	4697	GGGGAGAGAGAAGGGGGAAT	60.03	CTTGCTTGCTGAAAACCCCC	59.966	193
TRINITY_Contig6415	1	p2	(GC)6	12	1123	1134	CTCCGTTCTTGGTGAGGTCC	60.037	TCTTTGGAGCAGGTGCAGAG	59.964	196
TRINITY_Contig6416	1	c	(AGC)6gcacgcggccgccgccccgcctgtgagacaactgagaggaactgccc(TCCCT)4	85	80	164	TCGCTCGTGGTTTTGTAGCT	59.967	CAGCCGTGAACTTCCTCCAT	60.036	258

TRINITY_Contig6418	1	p3	(CCT)4	12	913	924	TCGTGTAGTTGCGAACACGA	59.971	GTAATGCAGCGAAGGCGAAG	59.973	152
TRINITY_Contig6418	2	p5	(GAGGA)3	15	1294	1308	TCGTGTAGTTGCGAACACGA	59.971	GTAATGCAGCGAAGGCGAAG	59.973	152
TRINITY_Contig6418	3	p3	(CTC)5	15	2948	2962	TCGTGTAGTTGCGAACACGA	59.971	GTAATGCAGCGAAGGCGAAG	59.973	152
TRINITY_Contig6418	4	p3	(GCG)4	12	3139	3150	TCGTGTAGTTGCGAACACGA	59.971	GTAATGCAGCGAAGGCGAAG	59.973	152
TRINITY_Contig6418	5	p3	(CCT)5	15	3720	3734	TCGTGTAGTTGCGAACACGA	59.971	GTAATGCAGCGAAGGCGAAG	59.973	152
TRINITY_Contig6419	1	p4	(CACC)3	12	45	56	CGGAAACCTCGAGGCATTCT	60.108	TTGATGAACATGACCGGCGA	60.037	123
TRINITY_Contig6420	1	p3	(GCG)6	18	980	997	TGGGGGCTGTTCTTTTGGTT	60.032	CTGTTTTGGCACATCGCACA	59.969	248
TRINITY_Contig6423	1	p4	(CGGA)3	12	57	68	GACTCGAACCCGGACAAGG	60.08	TTGCTCTTTGCTCCGACTCC	60.321	279
TRINITY_Contig6424	1	c	(GCA)5ggcgccaccggcacaaatactaccagcagcagcgccgccgcagtactaccagccgggcgccgccccgcagccgtggggc(CAG)4	106	143	248	TTGGATCGGGGATCTCCAGT	60.031	CCTTCAGACTGCCCACTCTG	60.037	126
TRINITY_Contig6424	2	c	(AGC)4aacaggcagcacctcaacagcaaccaccgccagcacaaccagcacaggaggagcaacagcagcagcctccacca(CAG)6	104	1429	1532	TTGGATCGGGGATCTCCAGT	60.031	CCTTCAGACTGCCCACTCTG	60.037	126
TRINITY_Contig6425	1	p3	(GCA)6	18	330	347	AGCCTGTGGAAGTCTTTCGG	59.965	AGTACTCGGGCAGGATTCCT	60.032	194
TRINITY_Contig6426	1	p4	(GATC)3	12	1165	1176	GCCTTCACCCACCTCTCTTC	60.036	AGGAGGGCTAGAGGAAGTGG	60.032	127
TRINITY_Contig6427	1	p3	(CGT)6	18	79	96	CAGAAGTCGAGCGTCCATCA	59.829	CTAGCTGGTGGGGAAGCAAA	59.962	151
TRINITY_Contig6428	1	c	(TGCCG)3tgccacctgaggtcaccgcgcagcggagcggaggcgcgccacgcaccacacgtct(GCCC)3	82	54	135	ACGAAGCCTTTTTGGTTGCC	59.896	TCTTCTGAGCTCCTCCTCCC	60.033	112
TRINITY_Contig6428	2	c*	(CCTCCC)2(CCTCCCTCCCTCATGTCGGCGGCCCCCACCCAACTTGCCGCACACGTAAGCCGTGAACCGCCTCTCCCCCCCACCACCGCACAGCCAGCACAACCTATATAACCGCTCGCGACTCCACCGTCCGCTCCCAACACACAACCAGAACGACGAAGCCTTTTTGGTTGCCCCGCATCGCATCACACCTGCAGTGCAGATCAACAACAAGCCAGCCAATCCATCAATCCAGCCACAGGAGGAGGGGAGGAGGAGCTCAGAAGATGAAACATCCGAGGGCGGAGCAGCAGCAGCAGGAGGAGGTGTCGCTGGCGCTGGCGCTGAGCACGACGGACTCGTGCACGACCTCCAACTCGTCCGCCGCGGCGCGGCGCCCCCGGCGGCCAAGCGCGCGCGCCGGCGGAGGGGCCCGGTGGTGGCCACGTCCGGGGAGGGGG	23	273	295	ACGAAGCCTTTTTGGTTGCC	59.896	TCTTCTGAGCTCCTCCTCCC	60.033	112
TRINITY_Contig6428	3	p3	(AGC)4	12	561	572	ACGAAGCCTTTTTGGTTGCC	59.896	TCTTCTGAGCTCCTCCTCCC	60.033	112
TRINITY_Contig6428	4	p4	(CATC)3	12	1096	1107	ACGAAGCCTTTTTGGTTGCC	59.896	TCTTCTGAGCTCCTCCTCCC	60.033	112
TRINITY_Contig6429	1	p5	(GAGCA)3	15	817	831	CCCCACAATGGTTACGGTCA	59.963	CGTGCATATTAGGCGGAGGT	59.966	129
TRINITY_Contig6431	1	p3	(GCC)4	12	788	799	AGGGAGATGATGACCGGGAA	60.031	AGGACGAGCCACCTTCTTTG	59.965	100
TRINITY_Contig6432	1	p4	(ATGG)3	12	2825	2836	CAGAAGGAAGAGGGGCCAAG	60.035	ACACCCGCTCATTTTTGCAC	59.968	202
TRINITY_Contig6433	1	p6	(GAAACA)3	18	102	119	AACTGAAGCAGCACCATCGA	59.965	GCAAGCGAATCTACTGCTGC	59.973	270
TRINITY_Contig6433	2	p3	(GGC)4	12	727	738	AACTGAAGCAGCACCATCGA	59.965	GCAAGCGAATCTACTGCTGC	59.973	270
TRINITY_Contig6434	1	p3	(CGC)7	21	118	138	CTTCTCCACCGACCTGACAC	60.038	GGACACGATCGGGATGAGAC	59.969	214
TRINITY_Contig6435	1	c	(CGC)4acacctgcgaggcctacttcggcaacggcttctcccgcgcgtggaggtcctccccgcgc(GCG)7	93	406	498	GCAAGGCACAATTGGAGCAA	59.966	TGAAAGAATGGCGTGTCGGA	59.966	101
TRINITY_Contig6435	2	p3	(GAG)4	12	630	641	GCAAGGCACAATTGGAGCAA	59.966	TGAAAGAATGGCGTGTCGGA	59.966	101
TRINITY_Contig6436	1	c	(CCGC)3gccgccgtccgccacaccgcttctccggccgactgagaggcgcgcc(CG)6	70	23	92	TGGTGAAGCGGATACCCAAC	60.036	AGCAATCCTCCCAAATGCGA	60.034	208
TRINITY_Contig6437	1	p3	(GGC)4	12	216	227	CGAGCTCTACTTCCACAGCC	60.179	AGGATGGACCGGATGAGGAA	60.031	132
TRINITY_Contig6437	2	p3	(CTG)4	12	1112	1123	CGAGCTCTACTTCCACAGCC	60.179	AGGATGGACCGGATGAGGAA	60.031	132
TRINITY_Contig6437	3	p3	(AAT)4	12	1515	1526	CGAGCTCTACTTCCACAGCC	60.179	AGGATGGACCGGATGAGGAA	60.031	132
TRINITY_Contig6438	1	p3	(CCA)4	12	5	16	TCTTCCTCCTCTACCGGGTG	60.033	CGTACCTGCTAGGCTGCTAC	59.969	195
TRINITY_Contig6438	2	p3	(CTC)4	12	751	762	TCTTCCTCCTCTACCGGGTG	60.033	CGTACCTGCTAGGCTGCTAC	59.969	195
TRINITY_Contig6438	3	p3	(GCG)4	12	1291	1302	TCTTCCTCCTCTACCGGGTG	60.033	CGTACCTGCTAGGCTGCTAC	59.969	195
TRINITY_Contig6438	4	p4	(CCGG)3	12	1854	1865	TCTTCCTCCTCTACCGGGTG	60.033	CGTACCTGCTAGGCTGCTAC	59.969	195
TRINITY_Contig6439	1	p4	(GTGA)3	12	1059	1070	CGTTCCAAGTCTGAGCGAGT	60.039	GGCAGCATAAACATCGGCAG	59.97	161
TRINITY_Contig6440	1	p3	(CGG)4	12	1857	1868	GGACACACCAGCTGCAAAAG	59.969	CTTGGCGTTGGGTCTTCTCT	59.965	197
TRINITY_Contig6441	1	p3	(GGA)5	15	1938	1952	ACCTTCAAAATGCACCACGC	59.968	ACCAGCTTGCCTCATGGTAC	60.035	188
TRINITY_Contig6441	2	c	(CGGG)3gcctaccgctgccggcccccctgtggacgtgagc(CG)6	57	2163	2219	ACCTTCAAAATGCACCACGC	59.968	ACCAGCTTGCCTCATGGTAC	60.035	188
TRINITY_Contig6442	1	p2	(GC)6	12	439	450	GCGGTGAAGACGAAGAGGAA	60.039	GAGAGAGACAACTTGCGCCA	60.32	132
TRINITY_Contig6446	1	p3	(GCT)4	12	325	336	TTCACGGTGTACTACGGCAC	60.039	CCTTCTCCACCTTGGACACC	59.963	142
TRINITY_Contig6447	1	p4	(GAGC)3	12	1199	1210	ACAGCACGAAACTGCAGAGA	59.895	TTGGAAAGAGGCTGGTCGAC	59.965	199
TRINITY_Contig6448	1	p5	(GGTCG)3	15	1061	1075	AAGGAGAGCGGGAGTGTACT	59.96	AGCTTCATCTTGCTGCCTGT	59.962	230
TRINITY_Contig6453	1	p6	(CGTGCC)3	18	1560	1577	ATTTGGAGCAATCCCCCGAG	60.107	AGATTTGACCGGGCGATACC	59.894	164
TRINITY_Contig6457	1	p3	(GTG)4	12	1699	1710	GAGGAATCAGGGCTGCTGTT	60.035	GGCATGACCATCCAAGGACA	60.034	104
TRINITY_Contig6458	1	p3	(TTG)4	12	102	113	GTCAAATACATCGGTGCCGC	59.972	TGATTTGGGCCTTTTGCTGC	59.965	245
TRINITY_Contig6458	2	p5	(CCCCT)3	15	2678	2692	GTCAAATACATCGGTGCCGC	59.972	TGATTTGGGCCTTTTGCTGC	59.965	245
TRINITY_Contig6459	1	p6	(GCGGTG)3	18	594	611	GAAAAGTGCTTGCCCCACAG	59.967	ACCAATCACCAATCCCCGAC	60.034	224
TRINITY_Contig6459	2	p3	(GCC)4	12	866	877	GAAAAGTGCTTGCCCCACAG	59.967	ACCAATCACCAATCCCCGAC	60.034	224
TRINITY_Contig6460	1	p3	(CCG)5	15	300	314	ACATGGATGAACTGGCCCAG	60.034	CGATGTTAAAGTCGCTGGCG	59.975	207
TRINITY_Contig6460	2	p3	(CGG)4	12	467	478	ACATGGATGAACTGGCCCAG	60.034	CGATGTTAAAGTCGCTGGCG	59.975	207
TRINITY_Contig6460	3	p4	(GGAG)3	12	2739	2750	ACATGGATGAACTGGCCCAG	60.034	CGATGTTAAAGTCGCTGGCG	59.975	207
TRINITY_Contig6460	4	p4	(CGCT)3	12	2885	2896	ACATGGATGAACTGGCCCAG	60.034	CGATGTTAAAGTCGCTGGCG	59.975	207
TRINITY_Contig6461	1	p4	(TAGC)4	16	96	111	CCTGGCTTGGCTTACATGGA	60.034	TTCCACTGCTTCGACTGGTC	59.966	207
TRINITY_Contig6463	1	p3	(CTT)4	12	54	65	ATGTTCCAATGCGAGGGGTT	59.96	CTCGGCGAACAACAAAAGCA	59.971	146
TRINITY_Contig6463	2	p3	(CGA)4	12	313	324	ATGTTCCAATGCGAGGGGTT	59.96	CTCGGCGAACAACAAAAGCA	59.971	146
TRINITY_Contig6463	3	p3	(AGT)4	12	585	596	ATGTTCCAATGCGAGGGGTT	59.96	CTCGGCGAACAACAAAAGCA	59.971	146
TRINITY_Contig6464	1	p5	(GCGTT)3	15	2720	2734	TCTCCCAAGCGAACCAGAAC	59.965	CTTCACCATATCCCCGCTCC	59.965	152
TRINITY_Contig6465	1	p2	(CG)6	12	745	756	GACAACCAGGCAACGTTGAC	59.971	ATGTGCTCTCCGGTGAACTG	60.037	164
TRINITY_Contig6465	2	p6	(GGCGAC)3	18	1991	2008	GACAACCAGGCAACGTTGAC	59.971	ATGTGCTCTCCGGTGAACTG	60.037	164
TRINITY_Contig6466	1	p2	(GA)14	28	35	62	GCGAAGACGTGATGGCAATC	59.973	GCCACATCAGAGCTCCTCAG	60.179	129
TRINITY_Contig6467	1	c	(GGC)5(GGA)7	36	405	440	CGCCCCGGCAATAGATTACT	59.965	CGGTGGAATGGGGTGATGAA	60.035	192
TRINITY_Contig6467	2	p3	(GGC)4	12	547	558	CGCCCCGGCAATAGATTACT	59.965	CGGTGGAATGGGGTGATGAA	60.035	192
TRINITY_Contig6471	1	p3	(GCC)4	12	487	498	GCAGGAGGATAGTTACGCCC	59.966	GCACCTCATTTCCCGAGACA	60.036	195
TRINITY_Contig6472	1	c*	(GCG)3(GCGGTGGCGGTGGCGGTGGCGCCACCCGCGTCGTCCTCCGCCGTGGACGAGGCCGGGGGCGCGGTGGAGCAGGCCAGGACGCTCATCGGCGCGCTCAACCTGCTCTCCCGCAACCTCCCGCTTCCCCCGACGTCCTGCGCGCCGTTTCGTCCATCTACCACGACGAAGGCGCCGACGACGGGGAGGCGGAGGAGGGGGAGGCGGAGAAGGAAGGGGATGAGGAGATGGCGGCCGCGGATGCCGCGGAGGAGGGCTGCGCGGAGGGCGCCGATGACGGTGCTGCCGAGGGGGCTACCTTGATTGAAGAGCTTGAAGATGCGATATTTAAGAATCAAAGAATGCCCGTATCCTACTCAGAATTGGCTGCTCTGAAGGAAGGACGATTCAATGCATCCATCCAGCATCGGCTAGCTGAACTCGAAGG	26	234	259	TTGGCTGCTCTGAAGGAAGG	59.963	GTATCCGCATCATCCCCCAG	60.036	241
TRINITY_Contig6473	1	c	(GGAG)3gctgggcggtgaagggaggcc(GGAG)5gcct(GA)9	75	712	786	TCTATCCCATACGCAACGCC	59.967	CCTCTTCTCCTTCCGTTCCG	59.826	197
TRINITY_Contig6475	1	p4	(TTGC)3	12	851	862	ATCTATCAAGGCGGCGTCTG	59.967	CATTGGGCTCCATGTCTGGT	60.034	162
TRINITY_Contig6476	1	c	(AGC)4acg(TTC)4	27	118	144	TTCCCGTAGCTGGATCTCCA	60.033	CTGGCTGGCTAGAGACGATG	59.968	194
TRINITY_Contig6476	2	p3	(CGG)7	21	546	566	TTCCCGTAGCTGGATCTCCA	60.033	CTGGCTGGCTAGAGACGATG	59.968	194

TRINITY_Contig6478	1	c*	(CGCTTC)3ctccacctccagcgcgaggcgcggggcggttccggcgggccgcggcgacccgccctcaggtgccgcggcgggtccccgc(CTG)10ccgcgggggcggc(GGAG)3gatggcc(GCG)4(GGAG)3*	182	83	264	TGCTCAAGCTTCCTTCTGGG	59.963	AAGTGGTTCCTGGCCCAAAA	60.032	126
TRINITY_Contig6479	1	p3	(GCG)6	18	62	79	TTGCAAACCATGACAGCTGC	59.968	TTTGATGCCTCGTGGATGCT	60.035	118
TRINITY_Contig6479	2	p3	(CCG)4	12	357	368	TTGCAAACCATGACAGCTGC	59.968	TTTGATGCCTCGTGGATGCT	60.035	118
TRINITY_Contig6480	1	c	(GGGA)3gagattattgtggttacccggagtagtggttggaggggaaggagccgtggaacccggggaggccggtgacgaggtggtcgtcgggc(GCG)5ggcgtgcggaggaggag(CGC)4	142	1613	1754	CCTTGGGCTTGTACTCAGGG	60.035	GTTGTCGATAAATGGCGCCC	59.971	130
TRINITY_Contig6481	1	c	(AT)7(TA)14	42	950	991	CCAACTTTGAGGACCTCCCC	59.962	CTGCTACGGAGATGAGCCAG	59.968	243
TRINITY_Contig6481	2	c	(AT)7(TA)17	48	1109	1156	CCAACTTTGAGGACCTCCCC	59.962	CTGCTACGGAGATGAGCCAG	59.968	243
TRINITY_Contig6484	1	p4	(CACG)3	12	25	36	GGGAACAGCGAACAGATCCA	60.036	CCAACACGTGCCGAAAATGT	59.97	128
TRINITY_Contig6487	1	p3	(GAT)4	12	1668	1679	CGGGTTCAGCTGAGTTGAGT	59.966	ATCACGCAGCTTCCTCAACA	59.965	182
TRINITY_Contig6489	1	p3	(AGG)4	12	3680	3691	AGAAGGAGGAGGAGGAGCAG	60.032	AGTCCTTCTTCAGCACCACG	59.966	157
TRINITY_Contig6491	1	p4	(CGCC)3	12	267	278	CCTGGCTGGATGATCGAGTC	59.967	GGTTGTCTCCACCATCTGGG	60.035	135
TRINITY_Contig6493	1	p3	(CTG)4	12	190	201	GCCGACTACGCCATCATGTA	59.968	CCAGCTTCTGCGGTAGTGAA	60.037	143
TRINITY_Contig6495	1	p3	(GCA)7	21	334	354	GTTGACCGAGAAGGCCTTGA	59.965	TAAGTGCGTCGCTGTAGTCC	59.83	245
TRINITY_Contig6499	1	p3	(CCG)4	12	113	124	TGAGAGTGAGGTCTGGGAGG	59.96	TCGACAACTGCAAGGATCCC	60.036	126
TRINITY_Contig6501	1	p3	(GGA)4	12	808	819	GTGTGGCTGACCTGAGTACC	60.037	CACACTTGTCTCGTCGTCCA	59.97	118
TRINITY_Contig6501	2	p3	(CGC)4	12	1149	1160	GTGTGGCTGACCTGAGTACC	60.037	CACACTTGTCTCGTCGTCCA	59.97	118
TRINITY_Contig6501	3	p6	(TGGCGC)3	18	1488	1505	GTGTGGCTGACCTGAGTACC	60.037	CACACTTGTCTCGTCGTCCA	59.97	118
TRINITY_Contig6504	1	p4	(GCGG)4	16	92	107	CGAGTTCGTGGTGGTGTACA	59.969	CGAAGGTGGCGAAGAAGAGT	60.038	173
TRINITY_Contig6504	2	p5	(CGCCG)3	15	254	268	CGAGTTCGTGGTGGTGTACA	59.969	CGAAGGTGGCGAAGAAGAGT	60.038	173
TRINITY_Contig6504	3	p4	(GGGC)3	12	591	602	CGAGTTCGTGGTGGTGTACA	59.969	CGAAGGTGGCGAAGAAGAGT	60.038	173
TRINITY_Contig6505	1	p3	(GTT)4	12	215	226	AGGTGCTATTCCCCTGCCTA	60.03	CGCAGTAGAACACTCCTGCA	60.038	206
TRINITY_Contig6506	1	p3	(CTC)4	12	256	267	AGTGGTGCTTCAGATGCCTC	60.036	ATGTGTGGAGGCTGACATGG	60.035	130
TRINITY_Contig6507	1	p3	(CGC)5	15	64	78	CAGACTCCGCACAAGTTCCT	59.966	TTGACGGCGAGAACGAGTAG	59.833	169
TRINITY_Contig6507	2	p6	(CGGCGC)3	18	310	327	CAGACTCCGCACAAGTTCCT	59.966	TTGACGGCGAGAACGAGTAG	59.833	169
TRINITY_Contig6507	3	p3	(CGC)5	15	948	962	CAGACTCCGCACAAGTTCCT	59.966	TTGACGGCGAGAACGAGTAG	59.833	169
TRINITY_Contig6508	1	p5	(ACAGC)5	25	83	107	GCCAAACGTCACACCTTCAC	59.971	TGGAGTACAGGAACGGCAAC	59.966	119
TRINITY_Contig6508	2	p3	(CGG)5	15	1538	1552	GCCAAACGTCACACCTTCAC	59.971	TGGAGTACAGGAACGGCAAC	59.966	119
TRINITY_Contig6508	3	p3	(CCG)6	18	1858	1875	GCCAAACGTCACACCTTCAC	59.971	TGGAGTACAGGAACGGCAAC	59.966	119
TRINITY_Contig6510	1	c	(GCCG)4ggcgctcgtggcgcgaaaacaaatcgagctgccggccgtggccggcaagggggccggacggcacgcgccccgcaggtg(GGGA)3	106	741	846	ATGCCACATCCGGTTTCCTT	59.96	TGCGATCGGAAGGATGGATG	59.966	233
TRINITY_Contig6510	2	p4	(ACGC)3	12	1267	1278	ATGCCACATCCGGTTTCCTT	59.96	TGCGATCGGAAGGATGGATG	59.966	233
TRINITY_Contig6511	1	p3	(GCG)4	12	812	823	GGTAGCCGGGGATCTTGATG	59.965	GGCCAGTACATCATCGAGGG	59.966	199
TRINITY_Contig6512	1	p4	(CCAT)3	12	39	50	ACACTGTGGCTCGAAGTCTG	59.967	CCTCCCCTGGTAGATGGTGA	60.031	134
TRINITY_Contig6512	2	p4	(CTAG)3	12	235	246	ACACTGTGGCTCGAAGTCTG	59.967	CCTCCCCTGGTAGATGGTGA	60.031	134
TRINITY_Contig6512	3	p3	(CCT)4	12	1083	1094	ACACTGTGGCTCGAAGTCTG	59.967	CCTCCCCTGGTAGATGGTGA	60.031	134
TRINITY_Contig6512	4	p4	(GGCT)3	12	1545	1556	ACACTGTGGCTCGAAGTCTG	59.967	CCTCCCCTGGTAGATGGTGA	60.031	134
TRINITY_Contig6513	1	p4	(TTGC)3	12	2528	2539	TAACCAACTCCTGCCGTGTC	59.966	GATTGGTCCTTCTGGGGGTG	60.034	163
TRINITY_Contig6518	1	p5	(GAGCC)3	15	1616	1630	ATACGACCCAAACCTGGCTG	60.035	CTGCCATGGTTGGGGAGAAT	60.033	115
TRINITY_Contig6519	1	c	(AGG)4agcatgagcttcctccaacacgcaccgagc(AGG)4	54	2329	2382	CATTGGAATGCGCCATCTGG	59.968	CCCCTCATGCTGTTCCCATT	60.033	146
TRINITY_Contig6519	2	p3	(TCA)4	12	2870	2881	CATTGGAATGCGCCATCTGG	59.968	CCCCTCATGCTGTTCCCATT	60.033	146
TRINITY_Contig6519	3	p3	(GGT)6	18	3291	3308	CATTGGAATGCGCCATCTGG	59.968	CCCCTCATGCTGTTCCCATT	60.033	146
TRINITY_Contig6521	1	c	(TTTGAC)3ccccatgacctgtccccaatatagcagcttcgtcagaagccaagcaagctccaaggcaaggctgcagcagcaatgg(CCT)4	106	104	209	TCGTCGCTGAAGGTGTACAC	60.04	CATGATCTCCACCTCCGTCG	59.969	162
TRINITY_Contig6521	2	p6	(CCGCCT)3	18	950	967	TCGTCGCTGAAGGTGTACAC	60.04	CATGATCTCCACCTCCGTCG	59.969	162
TRINITY_Contig6521	3	p3	(CGG)4	12	1195	1206	TCGTCGCTGAAGGTGTACAC	60.04	CATGATCTCCACCTCCGTCG	59.969	162
TRINITY_Contig6523	1	p3	(GGC)4	12	1255	1266	AAACTGACTCCTACAGGCGC	60.037	ATAAGTCAGCCCGTTTGCCA	59.962	154
TRINITY_Contig6525	1	c	(CCCTCC)3cgcgc(CGG)7	44	51	94	CACTGCTGTCTAAGGCCTCC	60.108	TGTTGCCCTTTTGGTCTGGT	60.033	211
TRINITY_Contig6527	1	p5	(CTTTT)3	15	3067	3081	GCACCCATTAGCGTTTGCAA	60.039	GCGCTATGTAGCCAAGTTGC	59.972	259
TRINITY_Contig6529	1	p3	(TGC)4	12	40	51	ATCTCCCCTTCCCTTCCCTC	60.03	CCTCAAACACACTTGCTCGC	60.041	233
TRINITY_Contig6530	1	p6	(GCCGGC)3	18	385	402	CGCAGTACCTGAGAGGCTTC	60.179	CACGCACACAACTCAAAGCA	59.901	263
TRINITY_Contig6531	1	p3	(GGC)4	12	558	569	TCGGCAAAAGGACCAGTCTC	59.965	TCATCGCCATGAGAGCTTCC	59.894	133
TRINITY_Contig6532	1	p4	(AGGC)3	12	435	446	CCGATCGTGCTGTCCGATTA	59.97	TTCGTCGAGGCAACCATCAA	59.966	229
TRINITY_Contig6532	2	p3	(GGC)7	21	1098	1118	CCGATCGTGCTGTCCGATTA	59.97	TTCGTCGAGGCAACCATCAA	59.966	229
TRINITY_Contig6532	3	c	(GCCGCG)3gc(CCAGCA)3	38	1296	1333	CCGATCGTGCTGTCCGATTA	59.97	TTCGTCGAGGCAACCATCAA	59.966	229
TRINITY_Contig6533	1	p2	(GA)6	12	583	594	GCAAGAGCTGTAAATCGCCG	59.973	TAGCCATGCAACTGCCATCA	60.034	207
TRINITY_Contig6533	2	p4	(TCAA)3	12	3246	3257	GCAAGAGCTGTAAATCGCCG	59.973	TAGCCATGCAACTGCCATCA	60.034	207
TRINITY_Contig6535	1	p6	(CCTCTC)4	24	1	24	TGATGTACAGCAGCAGCACA	59.965	GTGCCATAGACTGCAATGCG	59.971	269
TRINITY_Contig6535	2	c	(GCA)5gctccaacagaacccacagcaacagcagcagctccaacagaaccca(CAG)14caaatacaa(CAG)5ctacaacaacagct(GCA)5gctgcag(CAA)5atggggtctccgttacagcaggcgcaggtgggctcacctgctggctcacagcagtcaatgatgat(GCA)5	258	3873	4130	TGATGTACAGCAGCAGCACA	59.965	GTGCCATAGACTGCAATGCG	59.971	269
TRINITY_Contig6537	1	p3	(CGG)4	12	391	402	AGGCTCGTCACCTTTCTTGG	59.965	TGCAAGTCCTAGCTGTGCTC	60.037	212
TRINITY_Contig6537	2	p2	(GA)15	30	705	734	AGGCTCGTCACCTTTCTTGG	59.965	TGCAAGTCCTAGCTGTGCTC	60.037	212
TRINITY_Contig6537	3	p3	(AGG)4	12	931	942	AGGCTCGTCACCTTTCTTGG	59.965	TGCAAGTCCTAGCTGTGCTC	60.037	212
TRINITY_Contig6538	1	p5	(ATTTT)3	15	2265	2279	AAGGGCCGATTTCCTGATGG	60.107	GAGCAACATCCCAAGAGCCT	60.035	146
TRINITY_Contig6540	1	p4	(CTGC)4	16	21	36	ACCCTGCCTACCCATCTCAT	60.03	TTCAGCAGGTTCTTCGGGTC	59.965	192
TRINITY_Contig6542	1	p3	(TGC)6	18	619	636	CAACAACTGTGCCTGCTGAC	59.97	CAGGAGCCCTCTTCCGTTTT	59.963	235
TRINITY_Contig6543	1	p5	(GCTCC)4	20	2499	2518	GGATACCCACTTTCACCGCA	60.036	CCGGGCTTTAAGACTCCGTT	60.037	211
TRINITY_Contig6544	1	p2	(CA)9	18	510	527	AAAACACAAGTGCTCGCGAC	59.972	ATTCCACTTTGCGTTGTGCC	59.968	125
TRINITY_Contig6544	2	p3	(GCC)4	12	1324	1335	AAAACACAAGTGCTCGCGAC	59.972	ATTCCACTTTGCGTTGTGCC	59.968	125
TRINITY_Contig6545	1	p4	(GAGC)4	16	1267	1282	CGGAACACACGAACCTCAGA	59.968	TGGTGAAGTTCGAGGTCGTG	59.968	120
TRINITY_Contig6546	1	p3	(CTC)5	15	234	248	ACATCAGCATGAGCCAAGCT	60.034	CGCCAAATATTCTGCGTGGG	59.971	279
TRINITY_Contig6546	2	p3	(GGA)4	12	4100	4111	ACATCAGCATGAGCCAAGCT	60.034	CGCCAAATATTCTGCGTGGG	59.971	279
TRINITY_Contig6547	1	p6	(CGCCCG)3	18	248	265	GGAAGAGGAACAGCAGGACC	60.036	GAAGCTGGTTGACCGGATGA	60.036	146
TRINITY_Contig6547	2	p3	(CGG)4	12	392	403	GGAAGAGGAACAGCAGGACC	60.036	GAAGCTGGTTGACCGGATGA	60.036	146
TRINITY_Contig6548	1	p3	(TCG)5	15	412	426	CCTCGACCGAATGAACCACA	60.038	ACAAGTGGTACGACCTGCAG	59.966	254
TRINITY_Contig6548	2	p2	(GA)14	28	586	613	CCTCGACCGAATGAACCACA	60.038	ACAAGTGGTACGACCTGCAG	59.966	254
TRINITY_Contig6549	1	p4	(CAAT)3	12	355	366	CCACTGAACACTGGGAGCAT	59.963	TACACACGACCAAAGCCTCC	59.966	152
TRINITY_Contig6550	1	c	(TG)6(GGT)4	24	225	248	GGCCATCCGAACACCACTAA	60.036	CCTGGACAGCTTCTTCCAGG	60.036	206
TRINITY_Contig6551	1	p3	(GCT)5	15	914	928	CCGTGACAGGGAGAAGCTTT	59.965	GGAGGAGTGTGCTTCAGCTT	59.964	249
TRINITY_Contig6552	1	p3	(GCC)6	18	552	569	CGGTGCTGGGATGTCTTCTT	60.036	AACACTTACGGACAGCTGGG	59.965	222
TRINITY_Contig6553	1	p3	(GAG)4	12	3040	3051	AAGAGGAGGAGGAGGCGAAT	60.032	AGCCACTAATCCCCAAAGCC	60.033	104
TRINITY_Contig6554	1	p3	(GCG)4	12	2073	2084	TGGAATGTTCTGCTGCTCGT	59.965	ATGGCGCGGATAGAGAACTG	59.967	102
TRINITY_Contig6555	1	p2	(GA)6	12	208	219	TGAAAGGCGGCAATCGTAGT	60.037	GTTACGACAGTTGGCCCTCA	59.966	102
TRINITY_Contig6556	1	p3	(GCG)7	21	1605	1625	TTGGCGAAGACGAACGTGTA	59.969	GACGGCTGATGCGAATTGTC	59.973	246
TRINITY_Contig6556	2	p4	(GGAT)4	16	1961	1976	TTGGCGAAGACGAACGTGTA	59.969	GACGGCTGATGCGAATTGTC	59.973	246
TRINITY_Contig6559	1	c	(CCTC)3cgtcgaggtggtctacggcgacgtcaccgacgaggagtcgctcgccaccgccttccgcgggcgcgacgccgtgttccact(CCG)4	104	349	452	TGTCTGAAGTCTGAACCGGC	59.966	GTACGGATGCTGCTCGATGA	59.969	136
TRINITY_Contig6562	1	p3	(TCA)4	12	948	959	ACCATGCCTCTGTTCAGAGC	60.036	AGAAACTGGTGTCTGGCGAG	59.966	153
TRINITY_Contig6564	1	p6	(GGCGAG)3	18	1078	1095	CGCTGGAAACACACCCAAAG	59.969	CGTCCCGTGTGAAGATGACA	60.039	238
TRINITY_Contig6565	1	p2	(GC)6	12	349	360	GCAATTCTTTGGCCACTGCA	59.966	TCGGTCAGTGTCTGCTTTCC	59.966	170
TRINITY_Contig6567	1	p3	(CGC)4	12	154	165	GTGTGGTGGCCCATGTCATA	60.035	CCACAGACAAGCAAGCCAAC	59.969	240
TRINITY_Contig6568	1	p3	(AGC)4	12	362	373	TCCGCTACAAGTTCAGCCAG	60.037	CTGCAGTCCCTTGTTTGTGC	59.969	146
TRINITY_Contig6569	1	p6	(CGCGCC)3	18	58	75	GGCATAACCGCGATGACAAC	59.972	CCCACCAGGATGAGGCATTT	60.033	200
TRINITY_Contig6569	2	p3	(CGG)6	18	310	327	GGCATAACCGCGATGACAAC	59.972	CCCACCAGGATGAGGCATTT	60.033	200
TRINITY_Contig6569	3	p4	(TTTC)3	12	1758	1769	GGCATAACCGCGATGACAAC	59.972	CCCACCAGGATGAGGCATTT	60.033	200
TRINITY_Contig6571	1	p4	(GCGG)3	12	588	599	AACGGCTACCACATCCAAGG	60.035	ACCAGACTTGCCCTCCAATG	59.961	165

TRINITY_Contig6573	1	c	(TC)10gatctcgaacctcgcttcgccgcacggcacaaaaaagcccggccgcgccctccccccgcgctctcatcccctccacacgtttccgcctccccgatccaca(GCC)4gacacagcaccgcccccgccgtcccctcccagccttcgccgcagccgagg(CCGA)3ccagg(CAGC)3cgggctccttcctcgccaggcggcgcggcgcgcgccgg(GAGC)3	262	60	321	TTCCTTGTGTTCTCGGAGCC	59.965	AGAGACTCATCGTCAGGGCT	60.034	131
TRINITY_Contig6578	1	p2	(AG)6	12	70	81	TACTGCTGGTTCTGGTGCTG	59.965	AGCTTGCAAGTCGCTCTCAT	60.037	244
TRINITY_Contig6580	1	p3	(CTG)4	12	647	658	CTGCACCAGAGGACTTGGAG	60.037	GCTCAGGATTTTGTTGCCGG	60.109	145
TRINITY_Contig6581	1	p3	(GGC)4	12	17	28	GCACATTACCTGCAGATGCG	59.971	GCCACAGCAGAATTGAAGCC	60.109	117
TRINITY_Contig6582	1	p3	(CGG)5	15	2848	2862	GCTGCTGCAACAATGTGTCA	59.969	GAGGCTCTGAGGGTTAGGGA	60.032	247
TRINITY_Contig6585	1	p3	(GCG)4	12	186	197	AGTTCATGTCGACCAGGCTG	60.037	GCTCGTTGTTGCTCGCATAG	59.974	132
TRINITY_Contig6586	1	c	(CCT)4ccgccagcgccgctcgctcggtcgctgattagtccgtt(GCC)7	71	884	954	TTCTCACGCCCAGGGAAATC	60.035	GCCGATGAGAACGTGCATTC	59.973	197
TRINITY_Contig6587	1	p3	(GTT)4	12	852	863	TGGCGAACGCTCAAGTTACT	59.967	CCTTCCCCTGTGCGTACAAT	60.035	277
TRINITY_Contig6588	1	p3	(TGG)4	12	857	868	CGGCCTCCTTGAAGGTCAAT	60.035	TGTTGTAGCCCCTGAGCTTG	59.963	246
TRINITY_Contig6592	1	p3	(GAA)4	12	2224	2235	ATTCGCTCCCTTCACCACAG	60.036	CTCGTCAACCTGTCGGTCAA	59.968	157
TRINITY_Contig6595	1	p3	(TCA)4	12	1728	1739	GACACACGAGAGGAACTGCA	59.968	AAGTTGGCAGGGTTCACACA	60.034	105
TRINITY_Contig6596	1	p3	(AGG)4	12	444	455	GTAGCCCTGGCACTCCAAAT	60.034	CACTGTGTTGGATCCTCCCC	60.035	118
TRINITY_Contig6598	1	p3	(TGC)10	30	631	660	TCCTCCCACTCCTTCTCGAG	60.034	TACAAGGGGATCCACGGCTA	60.032	260
TRINITY_Contig6598	2	p3	(TCA)5	15	859	873	TCCTCCCACTCCTTCTCGAG	60.034	TACAAGGGGATCCACGGCTA	60.032	260
TRINITY_Contig6600	1	p3	(CCG)6	18	1630	1647	CTTATGTAGCGCGGGATGGT	59.966	GGAGGAGATGGATCGTGCAG	59.967	159
TRINITY_Contig6602	1	p2	(AC)6	12	52	63	GCGGTATCATCCCTGTCAGG	59.966	CAGCAGCTGGAGTCACTTGA	59.966	127
TRINITY_Contig6607	1	c	(GGA)4gtgggacgagtcccggcgggactcgcggaacaggcgagccatcgcggcggcggaggtgtcggcgtcggcgggtggcctcgcggcttgc(CT)6	112	3478	3589	CATCAGCGCAGTTATGGCAC	59.971	CCATGTTCTCTGGGACACCC	60.035	145
TRINITY_Contig6608	1	c	(ACC)4gccgccg(CCA)5	34	1101	1134	TTGATCCTTCCAACCTGGCC	59.96	CTATAGTGGTCGGTCCGTGC	59.97	264
TRINITY_Contig6611	1	p3	(GGC)4	12	2472	2483	CCTCCAAATTTGCCACCAGC	60.037	AGCATTGGATGGAGTTCGCA	60.035	273
TRINITY_Contig6613	1	p3	(GCC)4	12	89	100	GTGGGAATGTCCGTCCAAGT	59.964	GCAAGGCTTAACCGCAAACA	59.969	223
TRINITY_Contig6615	1	c	(GGA)4ggcagacatcgcggcgcggcggc(GCGG)3cgcggacgctcgggtttcgagcggggcgaggcacagagagggtgga(GGC)4	105	1310	1414	TCCAATGGAGGAGGAGGAGG	60.031	CTCCACTCCCACCAGTCAAC	59.965	165
TRINITY_Contig6616	1	p3	(GCC)4	12	1005	1016	GCGCAGTTGAACTCTTTGCA	59.971	TTTCTTCGGGTGTGGAGACG	59.967	119
TRINITY_Contig6617	1	p3	(CGC)4	12	1316	1327	TCCATGAAGTCGACAGTGGC	60.037	CTATCGTTGCGGCATCCTCT	59.967	262
TRINITY_Contig6618	1	p3	(CGC)4	12	1040	1051	GAGACAAGTTGCTGGAGGCT	59.964	TGTTCCAGCCAGCAGAAGAG	59.964	271
TRINITY_Contig6618	2	p3	(CCT)6	18	1555	1572	GAGACAAGTTGCTGGAGGCT	59.964	TGTTCCAGCCAGCAGAAGAG	59.964	271
TRINITY_Contig6619	1	c	(CCCTCT)4ctctagactagactctct(CCTC)4	58	119	176	CCTCCCTTCTCCTCTCCTCC	60.106	GGATTAATAGGCGGCGGGAA	59.964	255
TRINITY_Contig6619	2	c	(TTGCC)4gcctacacccgcgggaatggagattcccgccgcctattaatccttcctcggagggc(CCTTCT)3	94	434	527	CCTCCCTTCTCCTCTCCTCC	60.106	GGATTAATAGGCGGCGGGAA	59.964	255
TRINITY_Contig6619	3	p3	(TTC)5	15	718	732	CCTCCCTTCTCCTCTCCTCC	60.106	GGATTAATAGGCGGCGGGAA	59.964	255
TRINITY_Contig6623	1	p4	(ATAC)3	12	1121	1132	CCAGTCAGCAAGCACCAAAC	59.969	CAAGTTCAAGGTGCCCAAGC	59.967	159
TRINITY_Contig6623	2	p4	(GGGA)3	12	2040	2051	CCAGTCAGCAAGCACCAAAC	59.969	CAAGTTCAAGGTGCCCAAGC	59.967	159
TRINITY_Contig6623	3	p3	(GGC)4	12	2161	2172	CCAGTCAGCAAGCACCAAAC	59.969	CAAGTTCAAGGTGCCCAAGC	59.967	159
TRINITY_Contig6623	4	p4	(GATG)3	12	2461	2472	CCAGTCAGCAAGCACCAAAC	59.969	CAAGTTCAAGGTGCCCAAGC	59.967	159
TRINITY_Contig6624	1	p3	(GTT)7	21	305	325	TCATTCCCCTCCTCCCTCAG	60.031	CACCATGAGCCTTTGGGGAT	60.033	116
TRINITY_Contig6626	1	p4	(CGAT)3	12	220	231	ACACGTACACCTTGAGGCAG	59.966	ATCTGGAGGATCGACCCCAA	60.031	278
TRINITY_Contig6627	1	p2	(TC)8	16	16	31	CTGGACGATGACATCTCCCG	59.969	CAACTTCCACTGCCAACTGC	59.969	247
TRINITY_Contig6627	2	c	(CCG)4cagcatcagcaacagccggtg(CTTC)3	45	143	187	CTGGACGATGACATCTCCCG	59.969	CAACTTCCACTGCCAACTGC	59.969	247
TRINITY_Contig6627	3	p3	(GGC)7	21	458	478	CTGGACGATGACATCTCCCG	59.969	CAACTTCCACTGCCAACTGC	59.969	247
TRINITY_Contig6630	1	p6	(GTCCCG)3	18	868	885	AGGCGATGACGATCACGATC	60.04	GATAGGGATCGGGATCGGGA	60.033	179
TRINITY_Contig6630	2	p3	(GCG)5	15	1606	1620	AGGCGATGACGATCACGATC	60.04	GATAGGGATCGGGATCGGGA	60.033	179
TRINITY_Contig6631	1	p4	(AGCA)3	12	158	169	GCTTGTAGCACTGGAGCAGA	60.037	TAAAAAGCAGTGCACGGTGC	59.97	260
TRINITY_Contig6631	2	p3	(GCG)5	15	343	357	GCTTGTAGCACTGGAGCAGA	60.037	TAAAAAGCAGTGCACGGTGC	59.97	260
TRINITY_Contig6631	3	p3	(CTG)4	12	561	572	GCTTGTAGCACTGGAGCAGA	60.037	TAAAAAGCAGTGCACGGTGC	59.97	260
TRINITY_Contig6633	1	p5	(TCACC)3	15	7	21	AGGTTGAAGAGGGCATCTGC	60.035	TTGATGAATGCCCCGAGCTT	60.034	187
TRINITY_Contig6634	1	p4	(CCTC)3	12	17	28	GGGTGTAGAAGCCATGCTGT	60.035	CATGGTCCGCTAAACCCCTT	60.034	215
TRINITY_Contig6636	1	p5	(GGATG)4	20	1692	1711	TGACATGCCATCCGATCTGG	59.893	GAAGAAGCCATGGAGCCCTT	60.034	159
TRINITY_Contig6637	1	c	(CAGAG)3cag(GCGGC)3ag(CATC)3gatcgatcccgtccgcgcaccaaccccaacgcctgtgtacttgctggtgcacgagcgccgcttccgtccgtcccgacctgctccactctactctac(GA)7	157	203	359	ACTACCTCCTCGTGGTCCTC	60.034	TGAACGCCGTCTGGTAGAAC	60.039	141
TRINITY_Contig6637	2	p5	(CGCAT)3	15	497	511	ACTACCTCCTCGTGGTCCTC	60.034	TGAACGCCGTCTGGTAGAAC	60.039	141
TRINITY_Contig6642	1	c	(CCG)4cagtagtggtggccgagtgcgcgaagccgcagaccggcctccatctcgctcgcggtgcccgcgggtggctcggttcgtaatccgacgcgc(CGG)4	114	1024	1137	AACAGAATCCCGTGTGGTCC	59.964	TATCAACCTGAGCTCCCGGA	60.033	176
TRINITY_Contig6645	1	p6	(TCGCCG)3	18	543	560	CTTGTGGAAACTGCAGCACC	59.969	AAGGGCTTGAGGATGGCTTC	60.034	144
TRINITY_Contig6649	1	p4	(CATC)3	12	19	30	CGTGTGTCGTGTGTGAGAGA	59.971	ATGGCGACGAACTTCCCAAT	60.035	200
TRINITY_Contig6649	2	p3	(GGC)4	12	717	728	CGTGTGTCGTGTGTGAGAGA	59.971	ATGGCGACGAACTTCCCAAT	60.035	200
TRINITY_Contig6649	3	p3	(GAG)4	12	1024	1035	CGTGTGTCGTGTGTGAGAGA	59.971	ATGGCGACGAACTTCCCAAT	60.035	200
TRINITY_Contig6649	4	p3	(GAG)7	21	1333	1353	CGTGTGTCGTGTGTGAGAGA	59.971	ATGGCGACGAACTTCCCAAT	60.035	200
TRINITY_Contig6649	5	p3	(CCA)4	12	1547	1558	CGTGTGTCGTGTGTGAGAGA	59.971	ATGGCGACGAACTTCCCAAT	60.035	200
TRINITY_Contig6649	6	c*	(CCGG)2(CCG<G>CC)(GCC)7gcacctcccattcatgcttcaacgagtgatgatgatgctctctgtttgcctgcctcaggtgta(ACGT)3	110	1793	1902	CGTGTGTCGTGTGTGAGAGA	59.971	ATGGCGACGAACTTCCCAAT	60.035	200
TRINITY_Contig6649	7	p3	(CTA)4	12	2066	2077	CGTGTGTCGTGTGTGAGAGA	59.971	ATGGCGACGAACTTCCCAAT	60.035	200
TRINITY_Contig6650	1	p3	(GAG)4	12	857	868	TCCTGATGGTGGTGTTCTGC	59.963	TAACCACCTTCCCTGTTCGC	59.964	169
TRINITY_Contig6654	1	p3	(CAA)4	12	763	774	AGCAGAAGGATGGTCACTGC	60.036	TATCTGTTGGGCCCAAGCTG	60.034	136
TRINITY_Contig6655	1	p3	(CCG)4	12	1123	1134	ATGTCAGGCCCATATAGCGC	60.036	AGAAGGTTGGGGATTCAGCG	60.035	217
TRINITY_Contig6655	2	p2	(CG)6	12	1284	1295	ATGTCAGGCCCATATAGCGC	60.036	AGAAGGTTGGGGATTCAGCG	60.035	217
TRINITY_Contig6656	1	p3	(CGC)4	12	1419	1430	AAGTCAGCAGCACAGGAGAC	59.965	GGCAGGTTTGGGTTTCGTTC	59.968	269
TRINITY_Contig6658	1	p3	(GCC)5	15	840	854	CGGCTCAAACCACATGCAAA	59.968	CGTTCTTGGAGAGGTGTCCC	60.037	113
TRINITY_Contig6659	1	c	(CGGC)5ggggaagatttata(TATATG)3tatacatatatatacacctggtgatcgatctcttcgaccattaatcgcagtggtgattagagtatcatctagtagcagcagggtagaac(GAA)4	153	19	171	GCTTCTGCGCCATCTTCATG	59.971	CGGCGAACCCAAGAAAAAGG	60.04	205
TRINITY_Contig6660	1	c	(GGC)4(TGC)5gggtcggagcaggacgagggcggggacggcggcaccgcgtgcgccgcggcgcccagatcggcgta(CGG)4	104	471	574	GCAGCAAAGTTGAGCTTCCC	60.039	ACCTCGTCAACGCCTACATG	60.109	235
TRINITY_Contig6661	1	c	(CGG)5ttcctctg(CCT)4	35	665	699	TGCTCGCCTCACTTTCTTGT	59.893	CGGCCCAACAAGAGGAGATT	60.035	186
TRINITY_Contig6663	1	p4	(GCTC)3	12	96	107	AACGTGCCTTGCAATGTTCC	59.968	ACGTTGCACATTGATGCACC	60.039	245
TRINITY_Contig6663	2	p3	(CGG)5	15	388	402	AACGTGCCTTGCAATGTTCC	59.968	ACGTTGCACATTGATGCACC	60.039	245
TRINITY_Contig6666	1	p5	(TCCGT)3	15	207	221	AACGCGACAAGACACAAAGC	59.972	GATCCCCTGCTTGACTGCTT	60.035	252
TRINITY_Contig6669	1	p4	(GCAG)3	12	173	184	ATCTGTGCCTGTGTGATGCA	59.963	CAATTGCTCCTACGCACAGC	59.902	106
TRINITY_Contig6670	1	p4	(GTAC)3	12	94	105	GCAACCTCAAGTCCGTCTCA	59.966	CGTCGTAGATCCCTTCCTGC	59.969	171
TRINITY_Contig6670	2	p3	(CTA)5	15	306	320	GCAACCTCAAGTCCGTCTCA	59.966	CGTCGTAGATCCCTTCCTGC	59.969	171
TRINITY_Contig6671	1	p6	(TTTCTT)3	18	1235	1252	GAGCTCCGCCTCAGTATCAC	59.969	TGCTGGTGCAGTAATGCAGA	59.963	149
TRINITY_Contig6672	1	p2	(GA)16	32	108	139	GCAAAGAGTTCTGGTGTGCG	60.041	TGTCGAGTGGACTTTCAGCC	59.966	223
TRINITY_Contig6674	1	p3	(GCC)5	15	122	136	TGTATGTCGTCATCGGCGAC	60.248	ATCGATTGGTGAGCCACGTT	60.036	207
TRINITY_Contig6674	2	p4	(AATT)5	20	1619	1638	TGTATGTCGTCATCGGCGAC	60.248	ATCGATTGGTGAGCCACGTT	60.036	207
TRINITY_Contig6677	1	p3	(CCG)5	15	1215	1229	GATGCACGAGATAAGCCGGA	59.968	ATGCCTCCTTCAAGAACCCG	60.035	274
TRINITY_Contig6679	1	c*	(GAG)4(<GAG>CGC)(GAGCGC)2	30	384	413	GCGGGGTGGTCTCTTTTGTA	59.964	GCACAGTGACTTTGGCTTGG	59.969	150
TRINITY_Contig6679	2	c	(GGT)4gaccgtgatggcggagaccgcgagggcggcggcctccgtcgcttc(GGT)4	69	1023	1091	GCGGGGTGGTCTCTTTTGTA	59.964	GCACAGTGACTTTGGCTTGG	59.969	150
TRINITY_Contig6679	3	p4	(GCTC)3	12	1372	1383	GCGGGGTGGTCTCTTTTGTA	59.964	GCACAGTGACTTTGGCTTGG	59.969	150
TRINITY_Contig6680	1	p6	(GAACAG)3	18	253	270	GCAAAAGCTAACCAGGGTGC	60.038	TGTGTTTGCCAGTTGCCATG	59.896	123
TRINITY_Contig6689	1	p3	(GCA)6	18	94	111	TGATCCCTGTCGTCACCTCT	59.96	GCACCAGCTTGCCTTTGTAC	60.039	150

TRINITY_Contig6690	1	p3	(CGC)4	12	685	696	GATGTATGCCAAGTGCGCAG	59.971	GTCCCTGGACATGGCATCAA	60.034	259
TRINITY_Contig6691	1	p3	(GGA)7	21	1802	1822	GTTCAACACCACAGCCGAAC	59.971	TAATTGGTGGGCCGTCTGAC	60.036	279
TRINITY_Contig6692	1	c	(TGC)5aagtgaatggcattctgctgcaacagctgggcttgctgttgctgtagaatcgtagaacttgctgcagtggttggac(CTG)4caatacatgg(TGC)12	150	2038	2187	CTTTGCTGCAGTGGTTGGAC	59.969	TGCAGGATGGGATTGGCAAT	60.032	211
TRINITY_Contig6692	2	p4	(GAGG)4	16	4106	4121	CTTTGCTGCAGTGGTTGGAC	59.969	TGCAGGATGGGATTGGCAAT	60.032	211
TRINITY_Contig6692	3	p2	(GC)6	12	4267	4278	CTTTGCTGCAGTGGTTGGAC	59.969	TGCAGGATGGGATTGGCAAT	60.032	211
TRINITY_Contig6694	1	c	(CCA)4gtccccctcgtatctccgcatcgccgcgccgccgcctccctcctctcgatctcgctttcgcttcgctagcgctcccggagg(CCGGA)3	108	235	342	TATTACCCCTCCACCTGCGA	60.032	GTACTCCTCATCCATGGCGG	59.966	185
TRINITY_Contig6698	1	p3	(CTT)4	12	432	443	AAGCTGCTGCACCTGATGAT	60.034	CTGTTGCAACCCCAAACGAG	59.969	123
TRINITY_Contig6700	1	p5	(TCCCC)3	15	87	101	TCCCAATCGATGCTGACGAG	59.897	GCCACCACTACTTCCACCTC	60.036	114
TRINITY_Contig6701	1	p2	(GA)6	12	1125	1136	ACCCTGTACCATTGCTTGCA	59.888	CTGGGGTTGCTCATCCACAT	60.034	239
TRINITY_Contig6704	1	p3	(CCG)5	15	404	418	AACAGGCTTTCGGGATGGAG	60.035	GTCCAGTCGGCGGATCTTAG	59.969	161
TRINITY_Contig6704	2	p3	(CAG)4	12	1106	1117	AACAGGCTTTCGGGATGGAG	60.035	GTCCAGTCGGCGGATCTTAG	59.969	161
TRINITY_Contig6708	1	p3	(CGC)4	12	26	37	GCAAAAGCCATGGACGTTGT	59.968	ACGGACCTCTTGAGCCAATG	60.036	176
TRINITY_Contig6708	2	p3	(GTT)4	12	1170	1181	GCAAAAGCCATGGACGTTGT	59.968	ACGGACCTCTTGAGCCAATG	60.036	176
TRINITY_Contig6709	1	p5	(CGGGG)3	15	1573	1587	GTCAAAGCCCTGCCATTTGG	60.037	CTCCATCGTCCACTCGCTAC	59.97	163
TRINITY_Contig6710	1	p3	(GCC)5	15	896	910	ACGGCCAAGGTGATTAGCAA	59.962	TTACGTGCAGGAGATCGTCG	59.902	169
TRINITY_Contig6711	1	p3	(CGG)4	12	1422	1433	AAGGCTCCAGTGCTCCAATC	60.035	ACAGTGCCGTGCCTCAATAA	59.964	184
TRINITY_Contig6712	1	p5	(AAAGA)3	15	151	165	GCCACTCCAGCAGCTGATAT	59.892	CCTCTGTTTTTGCCCTGTGC	59.967	233
TRINITY_Contig6712	2	p3	(CCG)4	12	1210	1221	GCCACTCCAGCAGCTGATAT	59.892	CCTCTGTTTTTGCCCTGTGC	59.967	233
TRINITY_Contig6714	1	p3	(GCC)4	12	445	456	TCATGTTCCTGGCGGTGATC	60.108	GTCCTCTTTGTGGGAGTGGG	59.963	266
TRINITY_Contig6714	2	p4	(GCCT)3	12	1690	1701	TCATGTTCCTGGCGGTGATC	60.108	GTCCTCTTTGTGGGAGTGGG	59.963	266
TRINITY_Contig6714	3	p4	(ATCT)3	12	1820	1831	TCATGTTCCTGGCGGTGATC	60.108	GTCCTCTTTGTGGGAGTGGG	59.963	266
TRINITY_Contig6715	1	p4	(CTCC)4	16	1166	1181	ATGATCCCCTTGACCTCCGA	60.031	CTGATGATGCGGAGGAGGAC	59.967	178
TRINITY_Contig6719	1	p4	(GCAG)3	12	181	192	ACAAGATGCTCTCTCCCCCT	59.956	AGGTGTTGGGTTGGTTGGAG	60.106	276
TRINITY_Contig6719	2	p6	(CACCGC)5	30	432	461	ACAAGATGCTCTCTCCCCCT	59.956	AGGTGTTGGGTTGGTTGGAG	60.106	276
TRINITY_Contig6721	1	p2	(GA)6	12	4	15	AATGGAACTCCTTCGCTCGG	60.108	CAGTGTGACTGGCAAAGTGC	59.97	229
TRINITY_Contig6724	1	p3	(GCG)4	12	1274	1285	CGCAAACCACGTAAGCACAA	59.972	CCTCGCTACATCCGCATCTT	59.967	206
TRINITY_Contig6725	1	p4	(TGTA)3	12	1932	1943	CAACCTTTTCGCCAGTGTGG	59.969	CACTGAGCAGCACCTTCAGA	59.966	139
TRINITY_Contig6725	2	p5	(GGGGA)4	20	2508	2527	CAACCTTTTCGCCAGTGTGG	59.969	CACTGAGCAGCACCTTCAGA	59.966	139
TRINITY_Contig6726	1	c	(CGAAGG)5caggagggaatcccgaccgaattggggattggggcccccgaatccacctccacg(GCA)4ggggaggaggaggggagggatggaggggggcggcgggaggcctcctggatctggaggggaagggggggagccggaggaggcgtgt(TC)22	223	3443	3665	TGCAGATGTAGGGGACGGTA	60.033	TACAGAGGAGGCATGTCGGA	60.034	277
TRINITY_Contig6727	1	p3	(CAG)4	12	1791	1802	GAACAAGTACCAGGGCGTGA	59.966	CGAAAAGCACACAAAGCCGA	59.971	163
TRINITY_Contig6727	2	p3	(GAA)4	12	2109	2120	GAACAAGTACCAGGGCGTGA	59.966	CGAAAAGCACACAAAGCCGA	59.971	163
TRINITY_Contig6729	1	p6	(CGGCGC)3	18	766	783	GCCAATCCAACTGCGTTGTT	59.968	CCCTGGGTTCTGCCTTTCAT	59.96	157
TRINITY_Contig6729	2	p3	(CTT)7	21	1822	1842	GCCAATCCAACTGCGTTGTT	59.968	CCCTGGGTTCTGCCTTTCAT	59.96	157
TRINITY_Contig6730	1	c	(TCTAG)3ttctagtttctagtatagtattgtatttcccgttgatgaaatttccagtataatatttctcgttgatgaaatttctagttc(TAGTA)4	116	1173	1288	CGATCCCTGGCTACAATCCC	59.965	GGGGACTTTGATGGTGAGCA	59.962	245
TRINITY_Contig6731	1	c	(GCT)4g(CGC)5ggtcggcgagtacacctccgccgccgccatggcctccggcgacgagctgttgcgccacgtggggggcggacggtcggtgaggctcgagcgg(GGA)4gggacggaacggaagagggggaggcgaggccggaggcaaccggagga(GGT)4	190	1206	1395	CAGCGACTAATTGCCCATGC	59.97	GCTTCGGTATTTGGGGACGA	60.108	189
TRINITY_Contig6732	1	p3	(GCG)4	12	212	223	TCATTTCTAAGCGCTGGCCA	60.036	GGACTCCTCGCTCAATAGCC	59.967	123
TRINITY_Contig6733	1	p3	(GCG)5	15	4546	4560	TGCGAACTTTGTGCTCGTTG	59.973	TTGTTCAGGCACCGATCGAA	59.966	181
TRINITY_Contig6736	1	p2	(CT)8	16	206	221	CATTTTCCTGGTGTGCAGCC	60.038	CCAAGCAACTTCTGTGCACC	59.969	243
TRINITY_Contig6737	1	p3	(CCA)4	12	5655	5666	TCGATCGATCACACAGCTCG	59.972	AGTTTGAACGCGAGCACAAC	59.972	244
TRINITY_Contig6737	2	c	(CGC)5acgcgcgcatgg(CGAT)3	39	5893	5931	TCGATCGATCACACAGCTCG	59.972	AGTTTGAACGCGAGCACAAC	59.972	244
TRINITY_Contig6737	3	p3	(GCC)5	15	6079	6093	TCGATCGATCACACAGCTCG	59.972	AGTTTGAACGCGAGCACAAC	59.972	244
TRINITY_Contig6738	1	c	(GCG)6gctgcttcaagtgcggggagcctggccacatggccagggactgctcccagaatggt(GGC)5	89	1549	1637	CTCAAAGTTGTGCTGGTGGC	59.969	CTCGGAGGCAGCAGATAAGG	59.967	253
TRINITY_Contig6739	1	p3	(CGG)7	21	81	101	TGGATCGCATATGCTGGCAT	59.963	TGCTTTTACACTTGTGCGGC	59.97	249
TRINITY_Contig6740	1	p3	(CTG)6	18	368	385	AGAGAGACAGGCAGAGGAGG	60.033	GCTGATCGGTGGAGTAGACG	59.97	115
TRINITY_Contig6740	2	p2	(TA)6	12	754	765	AGAGAGACAGGCAGAGGAGG	60.033	GCTGATCGGTGGAGTAGACG	59.97	115
TRINITY_Contig6742	1	c	(TTTTCC)3t(TTC)5	34	292	325	AAGACGAAGCACGAGAAGCA	59.968	TGTTCTGGAGGAAGTGCGAC	59.966	220
TRINITY_Contig6742	2	p3	(GAA)4	12	668	679	AAGACGAAGCACGAGAAGCA	59.968	TGTTCTGGAGGAAGTGCGAC	59.966	220
TRINITY_Contig6745	1	c	(GCG)4gagg(CGC)6	34	67	100	ATTAGGGATTGGGTTCGCGG	60.179	TCAAACTTGTGAGCCCGTCA	59.821	126
TRINITY_Contig6750	1	p3	(TCC)6	18	177	194	CCAGAACGCAACAAAAGCCA	59.898	GGTCGGGAGTGCAGGTTTAA	59.964	106
TRINITY_Contig6750	2	p3	(GCG)4	12	372	383	CCAGAACGCAACAAAAGCCA	59.898	GGTCGGGAGTGCAGGTTTAA	59.964	106
TRINITY_Contig6752	1	p3	(GGC)5	15	1226	1240	TTTTGGGCAGAAGAAACGCG	59.97	TGGTGACCTCATTGTGGAGC	59.963	160
TRINITY_Contig6754	1	p6	(TCCAGC)3	18	27	44	CTCCTCCTGCTGACCACTTG	60.037	GGTTGATGCTGAGGCTGACT	60.036	268
TRINITY_Contig6754	2	c	(AC)15atatctt(CTC)5	52	724	775	CTCCTCCTGCTGACCACTTG	60.037	GGTTGATGCTGAGGCTGACT	60.036	268
TRINITY_Contig6755	1	c	(TGC)4tgttgt(TGC)7tgttgttgctgttgttgt(TGC)4	69	18	86	CCCTGGCTTTCATGAGCTGA	60.035	TCTTTTCGAGTGGTGGTCGG	59.967	110
TRINITY_Contig6755	2	p4	(CGGA)3	12	542	553	CCCTGGCTTTCATGAGCTGA	60.035	TCTTTTCGAGTGGTGGTCGG	59.967	110
TRINITY_Contig6755	3	p5	(GAGGC)3	15	699	713	CCCTGGCTTTCATGAGCTGA	60.035	TCTTTTCGAGTGGTGGTCGG	59.967	110
TRINITY_Contig6757	1	p3	(CCT)4	12	137	148	CTCGTTTGCTGTGTTCTGGC	60.041	CAACAAGTGGGGCATTGCTC	60.038	120
TRINITY_Contig6757	2	c	(CGG)4gtccgagtcgacggagtcgtccgcg(GGC)4	49	649	697	CTCGTTTGCTGTGTTCTGGC	60.041	CAACAAGTGGGGCATTGCTC	60.038	120
TRINITY_Contig6757	3	p4	(ATGG)3	12	1284	1295	CTCGTTTGCTGTGTTCTGGC	60.041	CAACAAGTGGGGCATTGCTC	60.038	120
TRINITY_Contig6758	1	p6	(CTCGGC)3	18	102	119	GCGTTCACCAGAGAAAGGGA	59.965	CATCACCATGCCCCCTTTCT	60.033	219
TRINITY_Contig6758	2	p3	(GGC)5	15	240	254	GCGTTCACCAGAGAAAGGGA	59.965	CATCACCATGCCCCCTTTCT	60.033	219
TRINITY_Contig6759	1	p3	(CCG)4	12	458	469	CGCTGCTACACCAAGATCCA	60.108	CTTGGGCTCCTCCTTCTTGG	60.035	246
TRINITY_Contig6759	2	p4	(TCGA)3	12	630	641	CGCTGCTACACCAAGATCCA	60.108	CTTGGGCTCCTCCTTCTTGG	60.035	246
TRINITY_Contig6760	1	p6	(CTCCGC)3	18	125	142	CACCGGGCAGACCTATGATC	59.966	GAGCGCTCTGTTTGGCAAAA	59.969	118
TRINITY_Contig6761	1	c	(GCT)5gtcgctggccttgctgctgcgactggggtgcctgctgt(TGC)4	65	686	750	TGGGAGTTCATCTGCTGCTG	60.036	GCACAGTTGACAATGCTGCA	59.969	212
TRINITY_Contig6761	2	p3	(TCT)4	12	1163	1174	TGGGAGTTCATCTGCTGCTG	60.036	GCACAGTTGACAATGCTGCA	59.969	212
TRINITY_Contig6763	1	p4	(GATC)3	12	3075	3086	ACTTGCGGTGGACTGGTTAG	59.965	ACCATGAGGGGGAGACTACC	60.031	121
TRINITY_Contig6764	1	p3	(GCC)4	12	76	87	ACACGGCATGGATTGATGGT	60.034	ATCAGCGAAGTGTGCGTGTA	60.039	118
TRINITY_Contig6765	1	p6	(GGTCGA)3	18	2384	2401	CTATCCGTCGCAGGAAGCAT	59.967	CGACATACTCCAGGCGTACC	59.97	173
TRINITY_Contig6765	2	p6	(GCTGCG)3	18	2732	2749	CTATCCGTCGCAGGAAGCAT	59.967	CGACATACTCCAGGCGTACC	59.97	173
TRINITY_Contig6765	3	p4	(GCGA)4	16	3019	3034	CTATCCGTCGCAGGAAGCAT	59.967	CGACATACTCCAGGCGTACC	59.97	173
TRINITY_Contig6766	1	p3	(CCG)5	15	1144	1158	TAATAAGAATCCCCGCCGGC	59.964	GAAACCCAAACCAAGACGCC	59.968	172
TRINITY_Contig6772	1	c*	(TAT)6(<TAT>ATATATATATATATATATATATATAGACAATAGATTGGAGACCAACTACTAGCTCACAAATGCCCCAGCATGTAGAGGTTTCAATGGACATATACTTTCCTCACCTCGCATAGTACGAACTGCTTGGGTTCATTGGCAGGTTATCGCCTTCTTGTGTAGGATACCCAGAAGTAGCACTTTCTATTTCAAAGCTAGCAGTCGATGCTCCACCGAGAAGGCTTTGAGTCTCCATCTCAGCATCTATTTGTACCCAAGGATGCAGTGAAAGCTTCCTCAACCTACCGAGCTCATCGGCAATTTCCTTCATCGACGGTCTGTTGCTGCCACACATGTCCAGGCATTGCTTGGCTAGCTCCGCGAGGCCTCTGATCAATTCGTTGCTCTCTTGCCCCTTTACATGGCTCGGTAAGACTGCATCAAGATTGTTCTCCTTCATA	46	90	135	AGGGTCTAGGTAGCCACAGG	60.032	CTACACTCGTACGCATCCCC	59.97	174
TRINITY_Contig6773	1	p6	(CCGAGA)3	18	194	211	CTCTGGTTCCTGCGTGACAT	60.037	TTCTGTGCTTCTCTGCCCTG	59.964	176
TRINITY_Contig6779	1	p3	(GGA)5	15	551	565	AGCATCTCCACCACAACCTG	59.963	CTAGATGCCTCAGATGCCGG	60.038	149

TRINITY_Contig6781	1	p3	(TGC)5	15	848	862	CCTGTGCCCACCATCTTCAT	60.034	CTGATCGTGGATCCGACTGG	59.969	137
TRINITY_Contig6781	2	p3	(GGC)4	12	3499	3510	CCTGTGCCCACCATCTTCAT	60.034	CTGATCGTGGATCCGACTGG	59.969	137
TRINITY_Contig6784	1	c	(GGC)4ctcc(GCG)4agtacttgaaccagacgtcgctgctggcctccagctccgccgccgcgcaccactcggccgcc(GCG)4ggcgcggcgggcggcgtggccagctcgctgct(GTA)4	146	350	495	CTCCTTGGCTCGTAGTCGTC	59.901	CACCAGAACGAGGAAGGTCC	60.037	276
TRINITY_Contig6785	1	c	(CGG)10(CGA)4	42	60	101	CCACAAGTTGGCGAACAAGG	59.969	GGCACAACAAGAGGCAAGTG	59.969	222
TRINITY_Contig6791	1	p3	(GAA)7	21	326	346	TAGTGCTACCTGCTGTGTGC	60.037	AGCGTTTGTGCTAATGCGTG	60.11	156
TRINITY_Contig6791	2	p3	(TTC)4	12	908	919	TAGTGCTACCTGCTGTGTGC	60.037	AGCGTTTGTGCTAATGCGTG	60.11	156
TRINITY_Contig6795	1	p2	(TG)6	12	1985	1996	ACCTGAGGCGGACAAAAGAG	59.965	GGATCATGACGACGTTTGCG	59.975	244
TRINITY_Contig6796	1	p2	(AG)7	14	342	355	CAGCGGTCTCATAGGTCGTC	59.97	GGTTGCCTCGTGAAACAACC	59.97	109
TRINITY_Contig6798	1	p3	(CGC)4	12	1903	1914	GTAGTCGGAGAAGAGCGACG	59.973	CATGTACGGCCAGATCCTCC	59.966	172
TRINITY_Contig6802	1	p4	(GGCC)3	12	1276	1287	TCGCTCGAGATGCTCATGTC	59.97	TCCGCCAAGTTCTTCCTGTC	59.965	176
TRINITY_Contig6804	1	c*	(GA)11ccgacgagtgggggcgctggcctgcagccggtgctactaatgctgaagag(GGT)4gaggagctcgtggtgtcgccattgggggcagttcggatgttacggccaggccgcgcggggc(GGCG)3(GGCACG)4*	180	935	1114	TGAGAGACACAGGCAGCTTG	59.966	GGTGGTGAGCGTGACATACA	60.038	107
TRINITY_Contig6804	2	p3	(CCA)5	15	1633	1647	TGAGAGACACAGGCAGCTTG	59.966	GGTGGTGAGCGTGACATACA	60.038	107
TRINITY_Contig6806	1	p5	(TCGTC)3	15	54	68	GAACTGTTCCTCGGGTCTGG	60.037	TGGTGCTCGACAACCTCTTC	59.966	236
TRINITY_Contig6807	1	c	(GGA)4gcacccccccggcggcgc(GGA)4	42	1456	1497	CGGCTCTGCGGATAAGCATA	60.038	GCTTGCGTGCACAATGGTTA	60.04	233
TRINITY_Contig6809	1	p6	(GGCGCT)3	18	414	431	GATGCTCGAGGAGATGGTGG	59.967	ACTGGACGGAGCAGAACAAG	59.966	119
TRINITY_Contig6812	1	p4	(CTCC)4	16	139	154	GAACCCGAAGAGTGCTGTCA	59.966	AGGCAGTGAACGGATGAAGG	60.036	249
TRINITY_Contig6814	1	p4	(ACAA)3	12	175	186	GAGGAGATCGCTAGCTGCAC	60.319	GTCAATGACGCCGACACAAC	60.111	122
TRINITY_Contig6814	2	p3	(GCC)4	12	907	918	GAGGAGATCGCTAGCTGCAC	60.319	GTCAATGACGCCGACACAAC	60.111	122
TRINITY_Contig6817	1	p4	(GTGC)3	12	162	173	GCAGCAGTAGTAGTAGCGGG	59.969	GAGATCATGATGGGGGAGCG	60.037	208
TRINITY_Contig6817	2	c	(TCC)4tcgacttggc(CGA)4	34	561	594	GCAGCAGTAGTAGTAGCGGG	59.969	GAGATCATGATGGGGGAGCG	60.037	208
TRINITY_Contig6819	1	p3	(GAC)4	12	489	500	GACGGCAAGAGTGAACTGGA	59.966	ACCCTGGCCAGTTCAACAAA	60.033	249
TRINITY_Contig6820	1	p6	(GCGCCA)3	18	1322	1339	TGTCGGAGTCGAGTTTGGTG	59.968	AATGGCTGGTGTTCGTCGAT	60.036	198
TRINITY_Contig6822	1	c	(CTC)4tcctccgccggcgcaggcgagtct(GCG)5	51	260	310	CTCATCAAGATCGACCGGCA	59.897	ATCCGTTTCTGGTTCGGGTC	60.037	147
TRINITY_Contig6822	2	p3	(CTC)4	12	496	507	CTCATCAAGATCGACCGGCA	59.897	ATCCGTTTCTGGTTCGGGTC	60.037	147
TRINITY_Contig6825	1	p3	(GCG)4	12	1287	1298	CTCCGAAGCCTCCTCACATC	59.896	TCATGCCATGGGATCGATCG	60.037	232
TRINITY_Contig6828	1	p3	(AGG)4	12	236	247	CTGCCTCGGCCTTGATCTAG	59.967	ATCTGCTCTATGGTCAGCGC	59.967	261
TRINITY_Contig6828	2	c	(CGC)4gggcgtcaagcgccggaaggcggtctccgacctcgaggagcgcgagcg(CGC)4	72	492	563	CTGCCTCGGCCTTGATCTAG	59.967	ATCTGCTCTATGGTCAGCGC	59.967	261
TRINITY_Contig6830	1	c	(CCT)4(CGC)4	24	94	117	GGAAGCCTGCAAGGAGGATT	60.034	CCCTTCCTCTGCCGAACAAT	60.035	256
TRINITY_Contig6832	1	p3	(GCG)4	12	1638	1649	CTCTGCTTGGTGATCCAGCA	60.036	ATCAACACCCATGCTCAGGG	60.034	168
TRINITY_Contig6833	1	p3	(TGC)4	12	620	631	ATAAGGGCTGCATCCGATCG	60.037	GCAGTGGCAGCTATGAATGC	59.97	167
TRINITY_Contig6836	1	p3	(GCG)4	12	252	263	CGGTCGAACGTCAGAAAGGA	60.04	GGAGATCCAGATGCTCCGTG	59.967	108
TRINITY_Contig6836	2	c	(CAC)7ggccacggcctcggcctcccgcagctgcagctgcacgccgaggacgagcaccgccgccaggagcaga(AGC)8gcgcggcctggagcgggcgcgccaagaacccggatctcaaccgggcgcccagccccgagagctccgacgccgagtgaa(GCAG)3	202	392	593	CGGTCGAACGTCAGAAAGGA	60.04	GGAGATCCAGATGCTCCGTG	59.967	108
TRINITY_Contig6836	3	c	(CATC)3g(TC)12	37	979	1015	CGGTCGAACGTCAGAAAGGA	60.04	GGAGATCCAGATGCTCCGTG	59.967	108
TRINITY_Contig6836	4	p4	(CCTC)3	12	1121	1132	CGGTCGAACGTCAGAAAGGA	60.04	GGAGATCCAGATGCTCCGTG	59.967	108
TRINITY_Contig6836	5	p4	(GCCG)5	20	1330	1349	CGGTCGAACGTCAGAAAGGA	60.04	GGAGATCCAGATGCTCCGTG	59.967	108
TRINITY_Contig6837	1	p4	(CCCT)3	12	39	50	GGGAATCTCGTCCTGTGCAA	60.036	TAGATTGGTCGTCGCCATCG	59.97	121
TRINITY_Contig6837	2	c	(AGG)4ccaccgatggcgacgaccaatctagcgccgcgacggcggat(GAC)6	71	652	722	GGGAATCTCGTCCTGTGCAA	60.036	TAGATTGGTCGTCGCCATCG	59.97	121
TRINITY_Contig6837	3	p3	(GCG)4	12	948	959	GGGAATCTCGTCCTGTGCAA	60.036	TAGATTGGTCGTCGCCATCG	59.97	121
TRINITY_Contig6846	1	p4	(TGTT)3	12	1903	1914	TTGTGGTTGGTCCTTGCAGT	60.034	CGACACACCGGAACAGTGTA	59.969	245
TRINITY_Contig6847	1	p4	(TGTT)3	12	1956	1967	TTGTGGTTGGTCCTTGCAGT	60.034	ACAACCCGAAACTCGATGCT	59.965	157
TRINITY_Contig6850	1	p3	(CGC)7	21	70	90	GCAATGGCGTCGAGGTAGTA	59.898	CTTCGACTCCGACATCCTCG	59.972	111
TRINITY_Contig6850	2	c	(AGC)4acggccgcatcagcagcgccatcgcgcaatgccggacggggggatcggat(GGC)4	74	1258	1331	GCAATGGCGTCGAGGTAGTA	59.898	CTTCGACTCCGACATCCTCG	59.972	111
TRINITY_Contig6851	1	p3	(GAG)4	12	356	367	CAACCATGAATGCTACCGCG	59.972	TTCTCGTGCATGAACCTGCT	59.965	105
TRINITY_Contig6854	1	c	(AGCCC)3aggtttcgtaccccttttgaccccaaca(AAAGC)3	58	48	105	GAATTCTCCATTTCGCCGGC	59.971	TGATGACGGCCGATCTCATG	59.967	102
TRINITY_Contig6854	2	c	(CCG)5ggaaacaacaagaccgcggcggggtaccaatcatacgacaacgg(CCA)4	71	222	292	GAATTCTCCATTTCGCCGGC	59.971	TGATGACGGCCGATCTCATG	59.967	102
TRINITY_Contig6854	3	p6	(CATCTC)3	18	1034	1051	GAATTCTCCATTTCGCCGGC	59.971	TGATGACGGCCGATCTCATG	59.967	102
TRINITY_Contig6854	4	p4	(TTAT)3	12	2956	2967	GAATTCTCCATTTCGCCGGC	59.971	TGATGACGGCCGATCTCATG	59.967	102
TRINITY_Contig6857	1	p5	(GTGTC)4	20	1682	1701	CCGTTCTGGCTGAGGTCAAT	60.036	AAGTTCCAGCGCCTAAGCAT	60.035	113
TRINITY_Contig6858	1	p4	(AGCT)3	12	3464	3475	CGCGTAATCTCCACGGATCA	59.97	GCCTCATCTGGTCGCTCTAC	59.969	235
TRINITY_Contig6859	1	p6	(AATCCA)3	18	2925	2942	CTCACTGTCACACCCATCCC	60.036	GAGGAGCAAGAGAGGGAGGA	60.033	223
TRINITY_Contig6859	2	p3	(CTG)5	15	3211	3225	CTCACTGTCACACCCATCCC	60.036	GAGGAGCAAGAGAGGGAGGA	60.033	223
TRINITY_Contig6861	1	p3	(CCT)4	12	971	982	TGGCATGGAGATGGAGGAGA	60.031	GGATCCACGGCATCTAGGTG	59.966	249
TRINITY_Contig6863	1	p4	(GCTC)4	16	16	31	TCGTCTACTTCAACGGGCAC	60.039	TGATGACGTCGTAGCTGAGC	59.901	195
TRINITY_Contig6863	2	p3	(GCG)4	12	393	404	TCGTCTACTTCAACGGGCAC	60.039	TGATGACGTCGTAGCTGAGC	59.901	195
TRINITY_Contig6863	3	p4	(CCGC)3	12	522	533	TCGTCTACTTCAACGGGCAC	60.039	TGATGACGTCGTAGCTGAGC	59.901	195
TRINITY_Contig6864	1	p3	(GCG)4	12	171	182	CAAGCTCTACTACGGCCTCG	59.97	TTGGGGAAGAAGCAGCTCTG	59.963	216
TRINITY_Contig6865	1	c	(AGCC)4gcaccgagatggccgtatccagccgaggcccgcgccggcgagcgcaacctgccgca(CCGCG)3	87	79	165	CTTTTATTCCACCCCGGCCT	60.034	GATCTCGCACCGAACCCTAG	59.969	106
TRINITY_Contig6865	2	p2	(TC)6	12	324	335	CTTTTATTCCACCCCGGCCT	60.034	GATCTCGCACCGAACCCTAG	59.969	106
TRINITY_Contig6865	3	p5	(CTCGC)3	15	602	616	CTTTTATTCCACCCCGGCCT	60.034	GATCTCGCACCGAACCCTAG	59.969	106
TRINITY_Contig6865	4	c	(GGA)4ggtgatggaggtgagggagatggcgatggccgcggccgcc(GCG)4	64	1018	1081	CTTTTATTCCACCCCGGCCT	60.034	GATCTCGCACCGAACCCTAG	59.969	106
TRINITY_Contig6865	5	p6	(GCTGCA)3	18	1607	1624	CTTTTATTCCACCCCGGCCT	60.034	GATCTCGCACCGAACCCTAG	59.969	106
TRINITY_Contig6866	1	p3	(GGA)4	12	725	736	GCGAACCATCCTCTCTAGGC	59.967	AGACCATAACAAGGGCAGCC	60.034	200
TRINITY_Contig6866	2	p3	(GGA)4	12	3861	3872	GCGAACCATCCTCTCTAGGC	59.967	AGACCATAACAAGGGCAGCC	60.034	200
TRINITY_Contig6867	1	p5	(GGCGC)3	15	3844	3858	CCTATCGAGCGCGTAAAGGT	59.97	CTTGGAGGTTTTGGTGCGTG	59.969	171
TRINITY_Contig6868	1	p3	(CGC)4	12	256	267	ACCCCAACGAGTTCCGAATC	60.037	GATCTGGAGCGAGTACACCG	59.97	239
TRINITY_Contig6868	2	p3	(GCC)4	12	1239	1250	ACCCCAACGAGTTCCGAATC	60.037	GATCTGGAGCGAGTACACCG	59.97	239
TRINITY_Contig6869	1	p4	(AATG)4	16	212	227	CCGGTGGTGACGTTGAAGTA	59.968	AGATTTTGCGGCACAACGAC	60.04	220
TRINITY_Contig6870	1	p3	(GCC)4	12	3199	3210	TGATCCTCACATTGGGGTGC	60.034	GGCTGCTTCCCTGTGTACTT	59.963	163
TRINITY_Contig6871	1	p2	(CG)6	12	283	294	GGCTCGATCCTACGACCAAG	59.969	AAATGTGCAGGGGGATGAGG	60.033	101
TRINITY_Contig6873	1	p2	(TG)9	18	153	170	CAAATAAGATGCCCTGGCGC	59.969	GTGCAACCTACACGGGAGAA	59.966	130
TRINITY_Contig6874	1	p4	(CAAC)3	12	8	19	CTGATGGGATCTTCGCCTCC	59.965	GGGAGCGGTAATGGGTGAAA	60.035	212
TRINITY_Contig6875	1	p4	(CGTG)3	12	403	414	AGACAGAACAAAACGTGCGC	59.972	ATTCTTCTTGGTGGGAGCCG	60.035	225
TRINITY_Contig6875	2	p4	(GCGA)3	12	522	533	AGACAGAACAAAACGTGCGC	59.972	ATTCTTCTTGGTGGGAGCCG	60.035	225
TRINITY_Contig6876	1	p3	(GCG)4	12	300	311	GTACTCGCAGTCCTACGTCG	59.974	CCACCAAGAAGCACACAAGC	59.969	200
TRINITY_Contig6876	2	c	(TACA)3tgcatacatatattcattt(ATAC)3	43	2050	2092	GTACTCGCAGTCCTACGTCG	59.974	CCACCAAGAAGCACACAAGC	59.969	200
TRINITY_Contig6876	3	c	(GCA)4tccgtgtcctcctccccagtcaccgcaggtcgcggtctcttgctgtaatcagtgttgccatctgagaatgag(CTC)4	96	3330	3425	GTACTCGCAGTCCTACGTCG	59.974	CCACCAAGAAGCACACAAGC	59.969	200
TRINITY_Contig6876	4	p3	(GGA)4	12	4151	4162	GTACTCGCAGTCCTACGTCG	59.974	CCACCAAGAAGCACACAAGC	59.969	200
TRINITY_Contig6877	1	p2	(AT)6	12	574	585	CGAGGTGAGCAGTTGAGTGT	59.967	TGAAGAAACATACCCCGGGC	60.035	254
TRINITY_Contig6877	2	p5	(CACTG)3	15	2248	2262	CGAGGTGAGCAGTTGAGTGT	59.967	TGAAGAAACATACCCCGGGC	60.035	254
TRINITY_Contig6878	1	p4	(AACG)4	16	84	99	CCTCCAGCTCCAAGTCAAGG	60.036	TTCCCATTCCCATTGCCCTC	60.032	105
TRINITY_Contig6878	2	p3	(CGG)5	15	421	435	CCTCCAGCTCCAAGTCAAGG	60.036	TTCCCATTCCCATTGCCCTC	60.032	105
TRINITY_Contig6878	3	p3	(CGC)4	12	640	651	CCTCCAGCTCCAAGTCAAGG	60.036	TTCCCATTCCCATTGCCCTC	60.032	105
TRINITY_Contig6879	1	p4	(ATGG)3	12	3662	3673	AGGGGAAGGGGATTGATCGA	60.03	AGACGCTGGTGAAGAAGTGG	59.966	143
TRINITY_Contig6879	2	p3	(CAT)4	12	6830	6841	AGGGGAAGGGGATTGATCGA	60.03	AGACGCTGGTGAAGAAGTGG	59.966	143
TRINITY_Contig6879	3	c	(GTC)4gtg(GGC)4	27	7800	7826	AGGGGAAGGGGATTGATCGA	60.03	AGACGCTGGTGAAGAAGTGG	59.966	143
TRINITY_Contig6879	4	p4	(CGCT)4	16	8264	8279	AGGGGAAGGGGATTGATCGA	60.03	AGACGCTGGTGAAGAAGTGG	59.966	143
TRINITY_Contig6880	1	p3	(GCC)5	15	30	44	AAAGCTGATCTTGAGGCCCC	60.034	ATTATATCGCCAGCCCCTGC	60.035	242

TRINITY_Contig6881	1	p3	(AGC)4	12	178	189	GAAGGCGGTGAACCTCTTCA	59.965	CCCTGTTCACATAGTGGCGT	60.036	180
TRINITY_Contig6881	2	p3	(GCG)4	12	3091	3102	GAAGGCGGTGAACCTCTTCA	59.965	CCCTGTTCACATAGTGGCGT	60.036	180
TRINITY_Contig6882	1	p3	(GCG)5	15	4689	4703	CTTGTTGTGGTTGTGGCTCG	59.971	TCATGCGTATAGGCTGCTGG	59.966	172
TRINITY_Contig6883	1	p2	(AG)13	26	686	711	GTCCTGAACGACGACCAGTT	59.968	TACGTGTGCAGCAATCTGGT	59.965	213
TRINITY_Contig6888	1	p3	(CAC)4	12	2643	2654	AAAGACGCGGCATTTGCTTT	59.968	TTGTGGTTGTTGGTCTGGCT	60.034	148
TRINITY_Contig6889	1	p4	(TTGT)5	20	5028	5047	ATGGGTTGGAGGCAGGATTG	60.033	GCCTGATCGATCGTTGGCTA	59.968	214
TRINITY_Contig6891	1	p4	(GCCG)3	12	312	323	GAATGTGTGATGCGGTGCTG	60.179	GTTCAATCCTGTGGACGGGT	59.964	263
TRINITY_Contig6892	1	p3	(CGC)4	12	142	153	GGCTGAGGAGACACACCTTC	60.037	CTAAATCCACAGTGCCGGGT	60.035	176
TRINITY_Contig6894	1	p2	(GA)6	12	489	500	CGAAGAACAAGATGCGCCAG	59.904	CGCGTGTATATTCTCCGCCT	60.039	132
TRINITY_Contig6895	1	c	(GAAGGA)3atcgaggagccgaataggcggagctcaccagcttgagggccttgttgagcgtgaccaccttggggagcggcgc(CTT)4	103	863	965	TCACCTTCCCGAGCAACTTC	59.965	GGGTGGACAAGATGACTGCA	59.963	156
TRINITY_Contig6902	1	p3	(CGC)5	15	674	688	GAGCAAGAAGGTGACGGTGA	59.966	CCGGATTGGAGGTTGGTTGA	59.962	135
TRINITY_Contig6902	2	p3	(GAA)4	12	985	996	GAGCAAGAAGGTGACGGTGA	59.966	CCGGATTGGAGGTTGGTTGA	59.962	135
TRINITY_Contig6903	1	p6	(CTCCGA)3	18	991	1008	ACGCTAAACCTCGGCTGAAA	59.966	AGAGTGAGTCCAGGGATCCC	60.032	133
TRINITY_Contig6903	2	c	(CTT)5(CCT)6	33	1251	1283	ACGCTAAACCTCGGCTGAAA	59.966	AGAGTGAGTCCAGGGATCCC	60.032	133
TRINITY_Contig6904	1	p3	(TGG)4	12	408	419	AATTGCAGACAGGAGAGGGC	60.035	GGCAGCAGCAACAAATGTGA	59.968	130
TRINITY_Contig6908	1	p3	(CGG)4	12	539	550	TGAGGTCGAAGTTGGTGACG	59.968	CTCGAAATACCGAGGGGTCG	59.97	185
TRINITY_Contig6908	2	p3	(CGG)4	12	829	840	TGAGGTCGAAGTTGGTGACG	59.968	CTCGAAATACCGAGGGGTCG	59.97	185
TRINITY_Contig6908	3	c	(GCG)4ctgttgctgtt(GGC)4	35	1461	1495	TGAGGTCGAAGTTGGTGACG	59.968	CTCGAAATACCGAGGGGTCG	59.97	185
TRINITY_Contig6908	4	p2	(GA)6	12	1633	1644	TGAGGTCGAAGTTGGTGACG	59.968	CTCGAAATACCGAGGGGTCG	59.97	185
TRINITY_Contig6909	1	p6	(GGCAAC)3	18	401	418	GCCGCCACTTGTGTTTAGTG	60.04	ATGCTGGGCACTGGAGATTC	60.107	120
TRINITY_Contig6909	2	p3	(CCG)4	12	734	745	GCCGCCACTTGTGTTTAGTG	60.04	ATGCTGGGCACTGGAGATTC	60.107	120
TRINITY_Contig6909	3	c	(CGG)4g(GACGGC)3	31	1645	1675	GCCGCCACTTGTGTTTAGTG	60.04	ATGCTGGGCACTGGAGATTC	60.107	120
TRINITY_Contig6914	1	p5	(TCCTC)3	15	185	199	TATCTGGTTGGGCGGTAGGA	60.032	GCACACCCATTCACACACAC	59.969	217
TRINITY_Contig6914	2	c	(AAT)4ggccgcggcggtggcggtggcttgtgtatattgttgacggcgtgcggtgacagggatgcaagagggtaggaacttggaaggcatggcaagttgt(TTGC)4	122	782	903	TATCTGGTTGGGCGGTAGGA	60.032	GCACACCCATTCACACACAC	59.969	217
TRINITY_Contig6915	1	c	(CCG)5cctggtca(TCG)4	35	1238	1272	CCTCCATGAAGCTCCGGATC	59.965	AGGTTTCGTCCTTGCTCCTG	59.965	183
TRINITY_Contig6918	1	p3	(CCA)4	12	84	95	GCCATTATCCACCACCACCA	60.034	CGCACTCTGCCGATCCATAT	60.038	132
TRINITY_Contig6918	2	p3	(GGA)5	15	501	515	GCCATTATCCACCACCACCA	60.034	CGCACTCTGCCGATCCATAT	60.038	132
TRINITY_Contig6925	1	p6	(GGCGCG)5	30	3	32	AGAAGGCAGAACGTACGCTT	59.683	AACAATTGTGGTCTGCGTGC	59.969	203
TRINITY_Contig6926	1	p2	(AT)6	12	8	19	TCAGGGTCCATCCTTCCCAT	59.954	TCTCGTGGTGTTCAAGGACG	59.968	100
TRINITY_Contig6926	2	p3	(CGC)5	15	488	502	TCAGGGTCCATCCTTCCCAT	59.954	TCTCGTGGTGTTCAAGGACG	59.968	100
TRINITY_Contig6928	1	p3	(CGG)6	18	265	282	ATCCTCGCTCTCCTCACTGT	60.034	AGCTCCTTCCGTCAGTTTCG	60.038	112
TRINITY_Contig6928	2	c	(GCC)4gacggggctgccgtgctgtgcctgttcttg(CCT)4	54	391	444	ATCCTCGCTCTCCTCACTGT	60.034	AGCTCCTTCCGTCAGTTTCG	60.038	112
TRINITY_Contig6929	1	p6	(GGTGAG)3	18	1227	1244	TTCGTGACCTTCGTGACAGG	59.968	CAACTGTGTTGCGCCAATGA	59.969	111
TRINITY_Contig6936	1	p3	(GCC)6	18	323	340	ACGAAGAGATGGAGAACGGC	59.826	GAATCCCCCAAACCCTAGCC	60.106	144
TRINITY_Contig6941	1	p2	(CA)13	26	85	110	AAGGCTCATTGCATGCTCCT	60.033	GAAGCCCATGTTCCCGAGAA	60.035	241
TRINITY_Contig6941	2	c	(GGA)4gggcttgctccgcttgagggagctcttcagcgcgggcttcggcggtggcggcagcgcggga(GGC)4gcgagggaggccgcgccgccggccggggca(GCG)6	133	1376	1508	AAGGCTCATTGCATGCTCCT	60.033	GAAGCCCATGTTCCCGAGAA	60.035	241
TRINITY_Contig6943	1	p6	(GGATGA)3	18	1828	1845	GCACAGATGTCCGAGAGAGG	59.898	ATCTGGTTTCAGCCCTGCTC	60.035	129
TRINITY_Contig6944	1	p3	(CCG)5	15	1929	1943	TGCTCTTCAACTCCAGGCAG	59.964	ATGGTGAGCTTGGTGACTGG	59.963	183
TRINITY_Contig6944	2	p3	(CGC)4	12	2053	2064	TGCTCTTCAACTCCAGGCAG	59.964	ATGGTGAGCTTGGTGACTGG	59.963	183
TRINITY_Contig6945	1	p2	(AT)8	16	59	74	TGTGGTGGTGGTGGGTTAAC	60.107	GCGGAGGTACTTATCCACCG	59.968	166
TRINITY_Contig6945	2	p3	(GTG)4	12	584	595	TGTGGTGGTGGTGGGTTAAC	60.107	GCGGAGGTACTTATCCACCG	59.968	166
TRINITY_Contig6953	1	p3	(CAT)5	15	147	161	CCACTTCTTGTTGCACTGCC	59.969	GATTTGCCATCCCAGGGTCA	60.033	262
TRINITY_Contig6953	2	p3	(CAT)4	12	825	836	CCACTTCTTGTTGCACTGCC	59.969	GATTTGCCATCCCAGGGTCA	60.033	262
TRINITY_Contig6953	3	p3	(ATC)4	12	1936	1947	CCACTTCTTGTTGCACTGCC	59.969	GATTTGCCATCCCAGGGTCA	60.033	262
TRINITY_Contig6954	1	p3	(GCT)5	15	2612	2626	AGGCCTACGAGGAAGGTGAT	60.032	GCCACGGACAAGTTCTCAGA	59.966	191
TRINITY_Contig6956	1	p2	(GT)6	12	1644	1655	CTGACACCTGTAGGGCGTTT	59.965	AAATCTGGCGAAGTCCCCAG	60.035	104
TRINITY_Contig6957	1	p3	(CGC)4	12	858	869	AGAAAGTGCCGGGGATTCAG	60.035	ATCCAGCTACTCGTGCAACC	60.108	137
TRINITY_Contig6959	1	c	(CGC)6gccgaacacgacgagcccgga(GAG)4	51	1253	1303	TCAGCAGTGACGCTAAGCAA	59.967	TACAGCTAGCCTCGATTGCG	59.969	103
TRINITY_Contig6962	1	p4	(AGAC)3	12	268	279	ATCTGCTGCAGACTTGGCAT	60.034	CGACACATCCAAGGTGGTCA	59.965	173
TRINITY_Contig6962	2	p3	(CCG)4	12	1495	1506	ATCTGCTGCAGACTTGGCAT	60.034	CGACACATCCAAGGTGGTCA	59.965	173
TRINITY_Contig6963	1	c	(CGT)4cgcagcagatga(AGC)4	36	528	563	TGCCTGCCTCCTCCTAATCT	60.031	CCTGTACTCGTCAAACCCCC	60.037	120
TRINITY_Contig6963	2	c	(CGC)5acccgcccgggagcaccgc(CGG)5gtggcaccgccgcaggatcaccatcctcttcatcatccacctaatcttcccgccccgatcctcctgcctgcctcctcctaatctt(CTTG)3gctctgaatggtcgagggagggagagattatgaggaggggagtaatggctagctagcgctgcatcttgggggtttgacgagtacaggagga(GGCG)3	249	726	974	TGCCTGCCTCCTCCTAATCT	60.031	CCTGTACTCGTCAAACCCCC	60.037	120
TRINITY_Contig6964	1	p3	(GCT)5	15	534	548	GAAGACGGTCCTGTCGTGTT	59.968	ACAGGAAGCTCGACACCTTG	59.966	185
TRINITY_Contig6965	1	p3	(CAG)4	12	332	343	GCCACTTGTGAGAGGTTCGA	59.966	CCACCGTCCTGTTTCCATGA	59.964	262
TRINITY_Contig6966	1	p6	(TTCACC)3	18	624	641	TCGTTGTGCCATGTGATCGA	60.038	TTCGTCCAGTCCTAGGCTGA	59.961	279
TRINITY_Contig6967	1	p3	(TGG)4	12	227	238	ACCTTGTTAGGCCGTGGATG	60.035	ATCTGTCCAGTGCTGATGCC	60.107	238
TRINITY_Contig6968	1	p3	(GCG)5	15	2375	2389	CAAACATGCACAGCCGGAAA	59.968	GAACGGTCTGTGTCTCTGCA	59.968	268
TRINITY_Contig6968	2	p3	(CCT)4	12	2718	2729	CAAACATGCACAGCCGGAAA	59.968	GAACGGTCTGTGTCTCTGCA	59.968	268
TRINITY_Contig6970	1	p3	(CCA)5	15	171	185	GTCTTCATCAGGGACGGCAA	60.036	TCGCTGTATGGATGTCGTGG	59.898	191
TRINITY_Contig6970	2	p3	(TGG)4	12	465	476	GTCTTCATCAGGGACGGCAA	60.036	TCGCTGTATGGATGTCGTGG	59.898	191
TRINITY_Contig6972	1	p4	(CATC)4	16	65	80	AGAACATGCCGTGGTACTGG	60.036	GTCGTAGCCCTCTTCGAAGG	59.899	239
TRINITY_Contig6972	2	p3	(CCG)4	12	741	752	AGAACATGCCGTGGTACTGG	60.036	GTCGTAGCCCTCTTCGAAGG	59.899	239
TRINITY_Contig6976	1	p4	(TTGA)3	12	30	41	TTCTGAACCACACTCCCTGC	59.891	ATCTTGCTCAGGTCCTTGGC	60.035	102
TRINITY_Contig6976	2	p3	(GAG)4	12	233	244	TTCTGAACCACACTCCCTGC	59.891	ATCTTGCTCAGGTCCTTGGC	60.035	102
TRINITY_Contig6979	1	p3	(CCG)4	12	75	86	GCGACCTCGTGAAGCTTTTC	59.836	TTCGAGCTTCCAACACTCCC	59.965	256
TRINITY_Contig6980	1	p3	(CTT)4	12	1127	1138	GTCATCGTAGCTCTCGTCCG	60.042	CTTGGAGAACGACGTGGTGA	59.968	157
TRINITY_Contig6982	1	p3	(GTG)4	12	945	956	ACGCCGGGGATTCTTTCAAT	60.034	AAGAGGGATTGGGCAAGCTC	60.034	109
TRINITY_Contig6983	1	p3	(CCG)4	12	669	680	CGGCAGAAAACGCAGGAAAA	59.97	TCCTGGGGTCCTACAGCTAC	60.032	279
TRINITY_Contig6983	2	c	(CCCG)3cgcgggcgccggcctccacgaccgccacctcgaaccggt(CCGCGC)3	69	1558	1626	CGGCAGAAAACGCAGGAAAA	59.97	TCCTGGGGTCCTACAGCTAC	60.032	279
TRINITY_Contig6983	3	p5	(CACGA)4	20	1903	1922	CGGCAGAAAACGCAGGAAAA	59.97	TCCTGGGGTCCTACAGCTAC	60.032	279
TRINITY_Contig6984	1	p5	(GAAGC)3	15	275	289	CATTGGGTGCCTGTGGAGAT	60.034	TGTCATTGCGGGCTTTTGTG	59.968	210
TRINITY_Contig6984	2	p3	(TGT)4	12	985	996	CATTGGGTGCCTGTGGAGAT	60.034	TGTCATTGCGGGCTTTTGTG	59.968	210
TRINITY_Contig6985	1	p3	(CAC)4	12	450	461	TACGAGTTGGAGTAGGCGGA	60.035	AACAACAACAGCGACCATGC	59.969	278
TRINITY_Contig6985	2	p2	(GT)10	20	1351	1370	TACGAGTTGGAGTAGGCGGA	60.035	AACAACAACAGCGACCATGC	59.969	278
TRINITY_Contig6987	1	p5	(CCTCT)3	15	218	232	CAACTGCCAAACACCAGAGC	59.969	CGGCGTTGCTTTCACTTTCA	59.971	269
TRINITY_Contig6987	2	p3	(GCC)4	12	448	459	CAACTGCCAAACACCAGAGC	59.969	CGGCGTTGCTTTCACTTTCA	59.971	269
TRINITY_Contig6987	3	p3	(CAG)5	15	2076	2090	CAACTGCCAAACACCAGAGC	59.969	CGGCGTTGCTTTCACTTTCA	59.971	269
TRINITY_Contig6988	1	p3	(CTC)4	12	127	138	GTGTAGCGATGCTCTGCTCT	59.898	GTTGAACCCAATGCCGCAAT	60.037	106
TRINITY_Contig6988	2	p3	(GCG)4	12	408	419	GTGTAGCGATGCTCTGCTCT	59.898	GTTGAACCCAATGCCGCAAT	60.037	106
TRINITY_Contig6990	1	p3	(CCT)5	15	84	98	TTGGAGTCACTGGCGTACAC	59.967	AATTGGTCTCGAGGCAGGTG	60.036	261
TRINITY_Contig6991	1	p3	(AGA)5	15	76	90	TAGATGACGTCGAAGAGCGC	59.972	GGAGAAGAGGAAGCAGCAGG	60.108	228
TRINITY_Contig6994	1	p3	(GCC)4	12	1532	1543	GATTCGTTCGCGCTCTCCTA	59.971	TAGCCTGCACCTTCCTTTGG	59.962	148
TRINITY_Contig6996	1	p2	(TC)17	34	424	457	GCTAGCTAGCTCGAGGTGTG	59.97	TACTCCCGGATCACCACCAT	60.032	119
TRINITY_Contig6996	2	p4	(CTAG)3	12	677	688	GCTAGCTAGCTCGAGGTGTG	59.97	TACTCCCGGATCACCACCAT	60.032	119
TRINITY_Contig6996	3	p5	(GCGCC)3	15	1283	1297	GCTAGCTAGCTCGAGGTGTG	59.97	TACTCCCGGATCACCACCAT	60.032	119
TRINITY_Contig6997	1	p4	(AGCA)3	12	1	12	CAGCATCGATCCATCCAGCT	59.965	GATCGGAGAAGGCCATGAGG	59.965	119
TRINITY_Contig6997	2	c	(ACC)4actaccaggcttcgtcggcggcggggtccggcgctgcc(TCG)4	62	564	625	CAGCATCGATCCATCCAGCT	59.965	GATCGGAGAAGGCCATGAGG	59.965	119
TRINITY_Contig6998	1	p3	(CCG)5	15	55	69	CAAGCCAGAGGTTACAGGGG	60.035	ATACAAGAAGGCACCCCTGC	60.034	198
TRINITY_Contig7000	1	c	(ATCC)3(ATCG)3	24	228	251	GCGGAATTGGAAGCGGAATC	59.971	CGCTCCCTTTTCCTTTCCCT	59.962	108
TRINITY_Contig7000	2	c	(TGC)4ggcgccgcggcgacgcaggaggcgacgagcagaaca(GCC)6	66	1479	1544	GCGGAATTGGAAGCGGAATC	59.971	CGCTCCCTTTTCCTTTCCCT	59.962	108
TRINITY_Contig7000	3	p3	(GCT)4	12	1653	1664	GCGGAATTGGAAGCGGAATC	59.971	CGCTCCCTTTTCCTTTCCCT	59.962	108
TRINITY_Contig7000	4	p4	(CGCA)3	12	1931	1942	GCGGAATTGGAAGCGGAATC	59.971	CGCTCCCTTTTCCTTTCCCT	59.962	108
TRINITY_Contig7001	1	p4	(TAGC)3	12	228	239	TCCGCGCTCCCCATTAATAC	59.966	CTTGGGGTCGAAGTAGCTGG	60.108	261
TRINITY_Contig7001	2	p3	(CGA)4	12	676	687	TCCGCGCTCCCCATTAATAC	59.966	CTTGGGGTCGAAGTAGCTGG	60.108	261
TRINITY_Contig7001	3	p3	(GTC)5	15	923	937	TCCGCGCTCCCCATTAATAC	59.966	CTTGGGGTCGAAGTAGCTGG	60.108	261

TRINITY_Contig7001	4	p3	(CGC)5	15	1102	1116	TCCGCGCTCCCCATTAATAC	59.966	CTTGGGGTCGAAGTAGCTGG	60.108	261
TRINITY_Contig7003	1	p4	(GGCC)3	12	292	303	GTAAATGCCAGCATGCCGAG	59.97	GATGTCGGTAATAGGCGGCA	59.967	126
TRINITY_Contig7003	2	p4	(GGCC)3	12	466	477	GTAAATGCCAGCATGCCGAG	59.97	GATGTCGGTAATAGGCGGCA	59.967	126
TRINITY_Contig7003	3	p5	(GAGCC)3	15	1251	1265	GTAAATGCCAGCATGCCGAG	59.97	GATGTCGGTAATAGGCGGCA	59.967	126
TRINITY_Contig7006	1	p4	(TGCA)3	12	3251	3262	TGAGCCATCATGCACACCAT	60.034	GGCTCGAGACACTTGGTTCA	59.966	193
TRINITY_Contig7008	1	p3	(CTC)4	12	797	808	GAGTGAGCTGCCGAGATCTC	59.969	GCAAAACGGGAGGGTTGTTC	59.968	276
TRINITY_Contig7008	2	p3	(TCG)4	12	935	946	GAGTGAGCTGCCGAGATCTC	59.969	GCAAAACGGGAGGGTTGTTC	59.968	276
TRINITY_Contig7008	3	c*	(CCG)4(<CCG>CTC)(CCGCTC)2	30	1068	1097	GAGTGAGCTGCCGAGATCTC	59.969	GCAAAACGGGAGGGTTGTTC	59.968	276
TRINITY_Contig7009	1	p3	(TCC)5	15	1249	1263	GAGGGGCATATGAGACGGTG	59.966	CCGCCATCAAGGAGAGGATC	59.965	217
TRINITY_Contig7010	1	p3	(GCC)4	12	515	526	CATGCAACATCTCGCGGAAG	59.973	TCGTGGACTGCAATGCTCTT	59.965	268
TRINITY_Contig7011	1	p2	(TC)9	18	18	35	CGTCCTCAAAGGCAAAGCAC	60.04	CACAAGGCGATGCATCCATG	59.97	197
TRINITY_Contig7012	1	p3	(GGC)5	15	1634	1648	CAACGGCCTCGAGATCATGA	59.897	GGCTTCTTCTCCGGCTTCTT	60.036	192
TRINITY_Contig7013	1	p2	(CT)16	32	56	87	CCTGGCATGCTTGATGATGC	59.968	TGATCACCAGTCCAGTGTGC	59.965	107
TRINITY_Contig7013	2	p2	(AG)7	14	3849	3862	CCTGGCATGCTTGATGATGC	59.968	TGATCACCAGTCCAGTGTGC	59.965	107
TRINITY_Contig7014	1	c	(GGC)5gcggcgggcccttcgtcggcgcgcctcttcgcggggtcccggagtagcgtcgcggcgtcgattgggaggaggg(GGA)4	100	59	158	GATCGACCGGAGATTGGACC	59.968	CTCCGACAAAGATCCCTCCG	59.897	105
TRINITY_Contig7015	1	p4	(TCGC)4	16	3683	3698	CCTACAGACTACAGCGCGAG	59.972	TGGTCTCGGCTTGATTGGAC	60.036	145
TRINITY_Contig7016	1	c	(CGAGCT)3cgggcacatagctcacgcgttgctcctgccaccgctgccgcgctcgccggcgaatctcccgccg(CGC)5	97	39	135	GATGCTTCGTCCGCACAATC	59.973	ACTTCTGCCATGCTGGACTC	60.036	185
TRINITY_Contig7016	2	p3	(GCT)6	18	11767	11784	GATGCTTCGTCCGCACAATC	59.973	ACTTCTGCCATGCTGGACTC	60.036	185
TRINITY_Contig7017	1	p4	(TGTT)3	12	569	580	ATTGGAATCGGGTGGTGCAT	60.033	ACAGGTTTGCCTAGTCACCG	59.965	144
TRINITY_Contig7017	2	c	(CCG)4ct(GCA)4	26	2784	2809	ATTGGAATCGGGTGGTGCAT	60.033	ACAGGTTTGCCTAGTCACCG	59.965	144
TRINITY_Contig7018	1	p3	(AGC)7	21	467	487	AACGCTACAACCAGAGGAGC	60.037	GATTCAGATGGCTCGCGAGA	59.969	227
TRINITY_Contig7018	2	p4	(GGCC)3	12	623	634	AACGCTACAACCAGAGGAGC	60.037	GATTCAGATGGCTCGCGAGA	59.969	227
TRINITY_Contig7018	3	p3	(GCC)4	12	3509	3520	AACGCTACAACCAGAGGAGC	60.037	GATTCAGATGGCTCGCGAGA	59.969	227
TRINITY_Contig7019	1	p5	(GCTCC)3	15	189	203	CTCGATTCGGTTTTCGTCGC	59.977	TGTCCTTCTCCATGGTGTGC	59.963	131
TRINITY_Contig7019	2	p2	(TC)6	12	538	549	CTCGATTCGGTTTTCGTCGC	59.977	TGTCCTTCTCCATGGTGTGC	59.963	131
TRINITY_Contig7021	1	p4	(AGAC)3	12	3027	3038	CTTGCCTCCACGTCCAAGAT	60.036	GTACCGTAGCTCGAATGCGA	59.971	274
TRINITY_Contig7022	1	p4	(CCGC)3	12	181	192	TCGTAGACACCACAAAGGCC	59.966	GGTAGAGCAGGTCCTGGAGA	60.033	180
TRINITY_Contig7022	2	c	(GAG)5ggggca(GCG)4	33	447	479	TCGTAGACACCACAAAGGCC	59.966	GGTAGAGCAGGTCCTGGAGA	60.033	180
TRINITY_Contig7023	1	p2	(AC)12	24	107	130	ATTCCAGCAGACCTTCGTGG	60.036	ATCCTTGATGGGGGCAACAG	60.033	274
TRINITY_Contig7023	2	p3	(CAG)5	15	415	429	ATTCCAGCAGACCTTCGTGG	60.036	ATCCTTGATGGGGGCAACAG	60.033	274
TRINITY_Contig7023	3	p6	(CTCCGG)4	24	806	829	ATTCCAGCAGACCTTCGTGG	60.036	ATCCTTGATGGGGGCAACAG	60.033	274
TRINITY_Contig7025	1	p3	(TCT)4	12	462	473	CCCCTTGGCGTTTGTAGTCT	59.964	CATGGAGGAACTTCGCCACT	60.036	242
TRINITY_Contig7026	1	p6	(ATTTGA)3	18	1674	1691	TGATGGGGTGTCGAACTGTG	59.965	GAACATGCATCCACATCCGC	59.97	225
TRINITY_Contig7026	2	p3	(GCG)4	12	2868	2879	TGATGGGGTGTCGAACTGTG	59.965	GAACATGCATCCACATCCGC	59.97	225
TRINITY_Contig7027	1	p3	(GCC)6	18	611	628	TCACAACATGCATCCACGGA	59.964	GAGCGGTGGTCTCTATCTGC	59.969	146
TRINITY_Contig7029	1	p3	(TCA)4	12	286	297	TCACGTGGCAGTTCGATCAA	59.967	TGGTGTCAGCCTTCGAAGAC	59.966	169
TRINITY_Contig7029	2	p3	(GGC)7	21	2087	2107	TCACGTGGCAGTTCGATCAA	59.967	TGGTGTCAGCCTTCGAAGAC	59.966	169
TRINITY_Contig7030	1	p2	(GA)8	16	518	533	GAGAGGGCGCACGATAATCA	59.968	GCTCGGAAGTTGGAGGAACA	59.965	240
TRINITY_Contig7041	1	p4	(GGGA)3	12	2776	2787	GCTCCAGTGTCGGAAGAACA	59.966	AGACCGCACTAGAGGATGGT	60.033	165
TRINITY_Contig7042	1	c	(GGGGT)3agacccgtcccct(TCC)4	40	1095	1134	GCGATGGATGGAGTATGGCA	59.965	AACGGGCCTCAATAATGCCA	60.034	188
TRINITY_Contig7042	2	p2	(CT)7	14	1366	1379	GCGATGGATGGAGTATGGCA	59.965	AACGGGCCTCAATAATGCCA	60.034	188
TRINITY_Contig7042	3	p3	(GTG)4	12	1582	1593	GCGATGGATGGAGTATGGCA	59.965	AACGGGCCTCAATAATGCCA	60.034	188
TRINITY_Contig7043	1	p3	(CGG)5	15	1242	1256	TTCTCATCCTGCGGTGTGTC	60.037	TCGGTGGACAGAGTGCTCTA	59.962	271
TRINITY_Contig7045	1	p3	(TGA)4	12	2151	2162	CTATCACCACGGTAGCGACC	59.97	CTCTTCCTCCTCCTCTGCCT	60.032	108
TRINITY_Contig7045	2	p6	(GGAAGA)4	24	2374	2397	CTATCACCACGGTAGCGACC	59.97	CTCTTCCTCCTCCTCTGCCT	60.032	108
TRINITY_Contig7047	1	p3	(GCG)4	12	358	369	GAAGCAAGATAAAGCGGCGG	59.972	GGTGTGGCACTTCAGCTAGT	59.965	236
TRINITY_Contig7047	2	p4	(TCCA)3	12	3942	3953	GAAGCAAGATAAAGCGGCGG	59.972	GGTGTGGCACTTCAGCTAGT	59.965	236
TRINITY_Contig7049	1	c	(TCC)5tcactagcagcggtggccgcagcaacgccttcctccgtcgcggggaggcaagaaggcacccccacccatcccgg(CCT)4	101	1250	1350	GAGCGCCATCATCATCCTCA	59.966	CAGCTGTCACAGGCACCATA	60.036	247
TRINITY_Contig7050	1	c	(AGG)4acgcggtgacggcgggggcgccggtgggggctgctgccatggcggcgacggcaggaga(GGC)4	82	1645	1726	GACGTCTGGAATAGGCTCGG	59.969	GGCCGCCGATACATCTTACA	59.967	178
TRINITY_Contig7051	1	p4	(AAAC)3	12	952	963	TAATGAGTGGCGTTGTGGCT	59.964	CTGTCTGCAAGGCTCTCACA	59.966	234
TRINITY_Contig7056	1	p3	(CGC)5	15	160	174	TGCCATCGCCTCTTCCATTT	60.034	ACGGAATCTATGCAGCGAGG	59.967	148
TRINITY_Contig7057	1	p3	(CCT)4	12	391	402	AAGAAGACCACTGCCGTAGC	60.037	ACCTCTGGAAGAACTTGCCG	59.965	219
TRINITY_Contig7058	1	p3	(CCA)5	15	1067	1081	GCTGACGAATGACCTCCACA	60.037	CACTGCAGAACACAGTTGCC	59.97	216
TRINITY_Contig7059	1	p3	(GCG)4	12	1477	1488	ATTCGTCATCCTCGTCGTCG	59.972	GTGACAAGTCCTGTGCGAGA	59.968	100
TRINITY_Contig7059	2	c	(TCG)5tactcgtcggcttcgtcatcgag(GTC)6	56	1675	1730	ATTCGTCATCCTCGTCGTCG	59.972	GTGACAAGTCCTGTGCGAGA	59.968	100
TRINITY_Contig7061	1	p3	(CAC)4	12	479	490	CAAGCAACCAACTGCAGCAT	59.967	AAGGATCTGAAGGCCAAGGC	60.034	186
TRINITY_Contig7063	1	p3	(TGC)8	24	1118	1141	AGTCCTTCTTGGCCTGGTTG	59.889	TACGCCTCCAACATCATCCG	59.896	169
TRINITY_Contig7064	1	p3	(CGG)4	12	46	57	TAGCAGCACGTGAGAAGCAA	59.967	TGCATTCGATCTTGGCACCT	60.035	197
TRINITY_Contig7066	1	p4	(GCTC)3	12	149	160	CTAAACCCTGACGCCACCTT	59.964	AAGAAGCGTCGGAATCAGGG	60.108	162
TRINITY_Contig7069	1	p3	(GGC)6	18	72	89	GGCAAAAGCCCACTCCCTAA	60.252	TTAGGAGTGAGCTCGTCGGA	60.036	191
TRINITY_Contig7072	1	p3	(CAG)5	15	621	635	GACAGCCGTGTGAGTTGAGA	59.968	CCCAGTTCCCCACAAGGATC	60.034	180
TRINITY_Contig7072	2	p5	(TGATG)3	15	2068	2082	GACAGCCGTGTGAGTTGAGA	59.968	CCCAGTTCCCCACAAGGATC	60.034	180
TRINITY_Contig7073	1	p5	(GCGGG)3	15	3207	3221	TGTGGACAACGATTCCGGAG	60.038	GCTAGGGCCTCAAGAAAGCA	60.035	139
TRINITY_Contig7075	1	p2	(GC)6	12	4	15	CGCACGGGCACGCTATAATA	60.665	CTGATGCTGAGCTCGCTCTC	60.596	109
TRINITY_Contig7076	1	p5	(TGCTG)3	15	1616	1630	CCCAATCCAAGCCCTGCTAA	60.033	TAAACCAGTGACGTGGCCTC	59.966	279
TRINITY_Contig7077	1	c	(CGC)4tcccggcgcttgttgttctcccgctccctccacgtgggcatccgccccgccccagccacccccagcgcccctcccgcg(CCT)4cccccgccgctcgccatgccgaactcgctgaggaaggggatggggaaggggaagaga(CCGG)3	171	1278	1448	AACTGGAGCACTGATGGAGC	60.036	ATCTTCCCCCTTCCTCCCTC	60.03	157
TRINITY_Contig7077	2	p4	(AGCG)3	12	1718	1729	AACTGGAGCACTGATGGAGC	60.036	ATCTTCCCCCTTCCTCCCTC	60.03	157
TRINITY_Contig7078	1	p3	(GGA)5	15	172	186	ATGCCTGAAGCTCCAGCAAT	60.033	TTGGGCAGCAGGTGATAAGG	60.034	105
TRINITY_Contig7078	2	p3	(AAG)4	12	720	731	ATGCCTGAAGCTCCAGCAAT	60.033	TTGGGCAGCAGGTGATAAGG	60.034	105
TRINITY_Contig7079	1	c*	(GGGGCG)2(GG<GGCG>GCGGCGGCGGCGTGGAGGAGGGAGGCGGCCATCGGGGACGGGTGGCGGTTGGTTGGGCGCCCCGCGGCTGCGACGCGCCGCCTCGGGAAGCGGAGGGAGAGGTGGGAGCGGTGGAATGGAACGGGGGAGGAGAAGAAGGAGGAGAGGAGAAGGGGAAGGGGCCGGGCGGCGGACGGCACGAAGGAGGGTGGCCGAGTGGGGGAAAACGTTGGTCTTCAAGAACACCCTCCTGATCATGACCTCAAACGTTGGGAGCAGTGT)(GGC)4	29	1476	1504	TTCCAAGAGCCCTGAACGTC	59.965	GAATGCAGGCGCTCTCAATG	59.971	228
TRINITY_Contig7081	1	p3	(CCG)5	15	1182	1196	AGAGGTGAGTGTGATCCGGA	59.96	CAGCGCACATGATTCCATCG	60.042	110
TRINITY_Contig7081	2	c	(CCT)4(CGT)4	24	1351	1374	AGAGGTGAGTGTGATCCGGA	59.96	CAGCGCACATGATTCCATCG	60.042	110
TRINITY_Contig7082	1	p3	(CCG)4	12	147	158	CCACGTTTACCAGCTCCCTT	59.964	CTGCACTACACTGCTCACCA	59.966	259
TRINITY_Contig7082	2	p3	(CGC)4	12	2455	2466	CCACGTTTACCAGCTCCCTT	59.964	CTGCACTACACTGCTCACCA	59.966	259
TRINITY_Contig7082	3	p6	(CGGGGA)3	18	2710	2727	CCACGTTTACCAGCTCCCTT	59.964	CTGCACTACACTGCTCACCA	59.966	259
TRINITY_Contig7082	4	p4	(GCCG)3	12	2912	2923	CCACGTTTACCAGCTCCCTT	59.964	CTGCACTACACTGCTCACCA	59.966	259
TRINITY_Contig7082	5	p4	(GAGG)3	12	3070	3081	CCACGTTTACCAGCTCCCTT	59.964	CTGCACTACACTGCTCACCA	59.966	259
TRINITY_Contig7084	1	p4	(TTGC)3	12	2141	2152	TCTTGCACGAGCTACTGCAA	59.967	AATCCAGGCCAGATTTGCGA	60.034	128
TRINITY_Contig7084	2	p4	(GCAA)3	12	2437	2448	TCTTGCACGAGCTACTGCAA	59.967	AATCCAGGCCAGATTTGCGA	60.034	128
TRINITY_Contig7085	1	p5	(GCGCT)3	15	2620	2634	ATCCCTGCACCTGTCCAATG	60.034	TCTGAACCATGTCCTGCACC	59.963	279
TRINITY_Contig7086	1	p4	(ACTC)3	12	124	135	CTCCTCCAGGTACCAGCTCT	60.033	GTTGCCTTGCTCAAGCGAAA	59.969	258
TRINITY_Contig7086	2	c	(ATCA)3gtcattacattcagttgtg(GAAC)5	51	1081	1131	CTCCTCCAGGTACCAGCTCT	60.033	GTTGCCTTGCTCAAGCGAAA	59.969	258
TRINITY_Contig7087	1	p3	(GTC)5	15	73	87	TTAGTTTGGCGTGCGTTTGG	59.97	CACACTCGCACACTACCAGT	59.968	116
TRINITY_Contig7087	2	c	(CCAGCT)3cgtcgagcgacgaggaagaggaggtgatcgacgagaacggcgagattgtcaggag(GAA)4	85	625	709	TTAGTTTGGCGTGCGTTTGG	59.97	CACACTCGCACACTACCAGT	59.968	116
TRINITY_Contig7087	3	c	(CGC)4gcccgccgtccac(GCGCCG)3	43	1157	1199	TTAGTTTGGCGTGCGTTTGG	59.97	CACACTCGCACACTACCAGT	59.968	116
TRINITY_Contig7089	1	p3	(CGG)6	18	185	202	CATGGAGGTGAAGGTCGAGG	59.823	GCATCCATTGATTGGTGGCC	59.895	130

TRINITY_Contig7089	2	p3	(GCC)6	18	422	439	CATGGAGGTGAAGGTCGAGG	59.823	GCATCCATTGATTGGTGGCC	59.895	130
TRINITY_Contig7090	1	p5	(GGCGA)3	15	183	197	CGATTGATGCAGCCAAGCTC	59.971	CCGATCCACCACAAGTCCAA	59.964	109
TRINITY_Contig7091	1	p3	(GTA)4	12	1845	1856	AACAACATTGGCAGCGGAAC	59.968	GCACATGCTCAATGGCTAGC	59.97	262
TRINITY_Contig7091	2	p4	(CTTG)3	12	2048	2059	AACAACATTGGCAGCGGAAC	59.968	GCACATGCTCAATGGCTAGC	59.97	262
TRINITY_Contig7091	3	p4	(CTTG)3	12	2295	2306	AACAACATTGGCAGCGGAAC	59.968	GCACATGCTCAATGGCTAGC	59.97	262
TRINITY_Contig7092	1	c	(GCC)4gaggaggaggg(GGA)5	38	2183	2220	TCGACATTGCAGCAATCCCT	60.035	CGAGGTGCCCAGGTATTTGT	60.035	220
TRINITY_Contig7093	1	c	(GCA)4(GCT)4	24	2129	2152	CGGCGGATTGGTTTTGGTTT	59.967	GAGCTCGTTGCAAAAGCACA	59.971	169
TRINITY_Contig7094	1	p3	(GCG)4	12	202	213	AGAGGGAGCACATGAAAGGC	60.035	GCCATTGCCAAAAGGTCCAG	60.037	146
TRINITY_Contig7096	1	p5	(AAGAA)7	35	145	179	AGGACCAGGGGGAGGTAATC	60.03	AGAATGTTCACCAGGCTCGG	60.036	206
TRINITY_Contig7096	2	p3	(TAG)5	15	2166	2180	AGGACCAGGGGGAGGTAATC	60.03	AGAATGTTCACCAGGCTCGG	60.036	206
TRINITY_Contig7098	1	p5	(GAGGC)3	15	1766	1780	GGCAAACACAACAGGCTGAG	59.969	TTCGATGTCGCGATCTCCTG	59.971	191
TRINITY_Contig7099	1	p3	(CCA)4	12	664	675	CGATCAGAAATGCTCGTCGC	59.772	GTCGACGACTGAGACTGACG	60.179	237
TRINITY_Contig7100	1	p4	(TCCC)3	12	186	197	CCACATGTCGACCTCACCTG	60.39	GGTATGACCCGCATCGAACA	60.179	151
TRINITY_Contig7101	1	p3	(CCG)4	12	10	21	TCAGCTCAGTCACCACGTTC	59.968	CACGACCAACCAAGTACCGA	59.968	136
TRINITY_Contig7102	1	c	(GCC)6ggcgcggtgatggcggcgatggagt ggatcgcatcagcgggctcaacgacgacctgc taatccaggtcctcgtccgcctccactg(CGC) 5	118	49	166	CTCTCGTGGAATTGCTGGGT	60.036	TCTTGCAATTTTCGCCCGTG	60.04	176
TRINITY_Contig7103	1	p3	(CGC)4	12	206	217	CAGCCAACTCGTTCAACTGC	60.041	CCTTCTTCGCCATGACCACA	60.322	105
TRINITY_Contig7105	1	p4	(CAAC)3	12	325	336	AGCAACCACTCGACGACATT	59.966	GTTGGTTGGTTGTTCCGAGC	59.97	193
TRINITY_Contig7111	1	p2	(GA)10	20	393	412	TCACGAGCTTGCTTTCGTCT	59.968	CTGCGCTACCTAGGTGATGG	59.967	149
TRINITY_Contig7111	2	p5	(AGAAA)4	20	887	906	TCACGAGCTTGCTTTCGTCT	59.968	CTGCGCTACCTAGGTGATGG	59.967	149
TRINITY_Contig7112	1	p4	(GCGT)4	16	2632	2647	ACAACCAATGCTGCAGCTTG	59.967	GTGGGAACTCACGCAGTACA	59.967	139
TRINITY_Contig7114	1	p3	(GGC)5	15	1303	1317	ACCAACTCCCAAGCACAACA	60.034	ACTGACGACCCTGAGTACGA	59.964	236
TRINITY_Contig7115	1	p3	(ACG)4	12	2796	2807	TCTTCAGAGTTGCAGCCCAG	59.964	AATGCACGAGCCTTTAGCCT	60.035	159
TRINITY_Contig7116	1	p4	(AAGG)3	12	3143	3154	GCAAAACGAAGCACTGCAGA	59.971	TACTGACGCCGATCATCGTG	59.971	121
TRINITY_Contig7117	1	p3	(GCG)4	12	2799	2810	GCTTCAGAGTCGGAGGATCG	59.97	TCAGCACTGACTGAGGCTTG	59.966	115
TRINITY_Contig7118	1	p3	(GGC)4	12	2673	2684	CCACCTCCCTCCCTTGAAAC	59.962	AACCCCGTTCTGGTGTTTGT	60.034	196
TRINITY_Contig7119	1	p3	(CCG)5	15	156	170	TGCTCTCAGGATAGGTGCCT	60.032	CTCTCGCTCGGTTTCCACTT	60.038	222
TRINITY_Contig7119	2	p3	(AGA)4	12	1993	2004	TGCTCTCAGGATAGGTGCCT	60.032	CTCTCGCTCGGTTTCCACTT	60.038	222
TRINITY_Contig7120	1	p3	(GGA)4	12	1763	1774	TCCTCTGCTCAAAGCCAGTG	59.964	GTCTGCCTGCTGTCAGACAT	60.037	181
TRINITY_Contig7120	2	p5	(GTGCA)4	20	1996	2015	TCCTCTGCTCAAAGCCAGTG	59.964	GTCTGCCTGCTGTCAGACAT	60.037	181
TRINITY_Contig7121	1	p5	(GAGAG)3	15	206	220	CCGAGCAAGTTCGCAAACAA	59.971	GAAGCACTCTGGCCATGAGT	60.036	231
TRINITY_Contig7121	2	p3	(TCC)5	15	364	378	CCGAGCAAGTTCGCAAACAA	59.971	GAAGCACTCTGGCCATGAGT	60.036	231
TRINITY_Contig7124	1	p3	(CCT)5	15	1365	1379	GGCCGGTTCTCTGGATCAAA	60.035	AGATGCATGGCCTTTCAGCT	60.033	208
TRINITY_Contig7125	1	p4	(CTCC)3	12	429	440	GAGCGTCTGGACATCTCTCG	59.971	GCAAGTGAGGCAGTAGCTGA	60.037	239
TRINITY_Contig7125	2	p3	(GGA)4	12	1008	1019	GAGCGTCTGGACATCTCTCG	59.971	GCAAGTGAGGCAGTAGCTGA	60.037	239
TRINITY_Contig7126	1	p3	(GAG)5	15	2595	2609	GGAACTTGTGGCAAGGCAAG	59.967	CTTGTTGGAGATGGCCGTCT	60.036	179
TRINITY_Contig7128	1	p5	(GGCGG)3	15	669	683	AGTCCGAGGAGACTGGAGTC	60.035	CACAGGGGCTTCCATCAACT	59.961	140
TRINITY_Contig7129	1	p5	(GGCGT)3	15	494	508	GTGGTATGGCTACGGGCTAC	59.967	GAGGGAGCCGGGGGATATAT	60.031	118
TRINITY_Contig7131	1	p3	(GCG)5	15	91	105	ACTGCGTAGGTGGTTAAGCC	60.037	GTTTGCGTGGGGGAGTAGAA	59.964	132
TRINITY_Contig7131	2	p3	(CCG)7	21	216	236	ACTGCGTAGGTGGTTAAGCC	60.037	GTTTGCGTGGGGGAGTAGAA	59.964	132
TRINITY_Contig7131	3	c	(CCATCC)3ctcccctcccgcgccgccggccc ccgcttcgcccatggcctct(CCG)4	73	354	426	ACTGCGTAGGTGGTTAAGCC	60.037	GTTTGCGTGGGGGAGTAGAA	59.964	132
TRINITY_Contig7132	1	p3	(CTC)4	12	1229	1240	AACGTTGACGATCCAGCCTT	59.965	TTTTCAGCCGCATGGGATCT	60.034	136
TRINITY_Contig7136	1	c*	(TCCT)2(<TCCT>CCTCCTCCCCTCGA ACCACACCCCCACCCAGGTCATGCT CCCCCCAAGGTCGTCTCGCTTGCCA GTGTGGGCCGCTGTGGCTTGCCCA CCTCCGGATCTACACTCCCTTGGTC GATTTCTCGTTGGTTTCGCCGGCGG CGGTCGTGGTCGTCTTGGTCGCAG CTCCTCCGGATCTAGCGGAGGAAG CAGACGTCCGCTACGCCGCGGGGC CCACGCGTCCGCGCGCCCACGCCAA CCACCCGGCCCGCCGCAGCTTGCTC GCCGCGGCCGCTGCAGCTCCGCCC CCGCCGCCATGGATCCGCCGCCGG AGCTCGAGGGCAGGGGAGGCCGG TGGGCGCACGGGAGGCCGCGCACC GAGGAGGGAGGAGGGGCGCGGC GAGGGAGGAGGGTCGTCCGCCGG CGCTGGAGGCCGTCCGCGAGCTGT	20	101	120	ACCTTCTTCTTCACTGCCGG	59.965	TGAGGGCCAAAATCGGTCAA	59.889	110
TRINITY_Contig7137	1	p3	(GCG)4	12	921	932	GCTGCTTTGCGATGATAGGC	60.041	CCAGAGGCTCAAGGACTTGG	60.036	110
TRINITY_Contig7137	2	p3	(GCT)4	12	1228	1239	GCTGCTTTGCGATGATAGGC	60.041	CCAGAGGCTCAAGGACTTGG	60.036	110
TRINITY_Contig7137	3	p4	(CTAG)3	12	1467	1478	GCTGCTTTGCGATGATAGGC	60.041	CCAGAGGCTCAAGGACTTGG	60.036	110
TRINITY_Contig7141	1	p4	(CCTG)3	12	185	196	TTCTCCAAGTGCAGCCAGAG	59.964	GAAAGCTTTGCTGCTGGTCC	60.039	137
TRINITY_Contig7141	2	p6	(CGGACA)3	18	1193	1210	TTCTCCAAGTGCAGCCAGAG	59.964	GAAAGCTTTGCTGCTGGTCC	60.039	137
TRINITY_Contig7141	3	p4	(GGCA)3	12	1950	1961	TTCTCCAAGTGCAGCCAGAG	59.964	GAAAGCTTTGCTGCTGGTCC	60.039	137
TRINITY_Contig7141	4	p4	(CGGT)3	12	2988	2999	TTCTCCAAGTGCAGCCAGAG	59.964	GAAAGCTTTGCTGCTGGTCC	60.039	137
TRINITY_Contig7143	1	p4	(TATC)3	12	1708	1719	CAGCATCCCTGGCTTGTACA	60.035	TTGCTGCTCCCCGATTTGAT	60.034	152
TRINITY_Contig7143	2	p5	(AGAAC)3	15	1974	1988	CAGCATCCCTGGCTTGTACA	60.035	TTGCTGCTCCCCGATTTGAT	60.034	152
TRINITY_Contig7144	1	p2	(GC)6	12	41	52	TCGCCCTGCTTCAATTCACT	59.963	CAATGCGGTGGTCGTTCTTC	59.834	117
TRINITY_Contig7144	2	p4	(TAGC)3	12	290	301	TCGCCCTGCTTCAATTCACT	59.963	CAATGCGGTGGTCGTTCTTC	59.834	117
TRINITY_Contig7144	3	c	(GCC)5cccgacgcccgtgccgtgc(CCG)4	46	419	464	TCGCCCTGCTTCAATTCACT	59.963	CAATGCGGTGGTCGTTCTTC	59.834	117
TRINITY_Contig7144	4	p4	(GCAT)3	12	794	805	TCGCCCTGCTTCAATTCACT	59.963	CAATGCGGTGGTCGTTCTTC	59.834	117
TRINITY_Contig7145	1	p6	(CTCCGC)3	18	850	867	TCTTCCTTATCCTCCGCCCA	60.032	TTTGAGCGCAGAGGTAGCAA	59.965	203
TRINITY_Contig7145	2	p3	(GGC)4	12	1092	1103	TCTTCCTTATCCTCCGCCCA	60.032	TTTGAGCGCAGAGGTAGCAA	59.965	203
TRINITY_Contig7145	3	p3	(CGC)4	12	2822	2833	TCTTCCTTATCCTCCGCCCA	60.032	TTTGAGCGCAGAGGTAGCAA	59.965	203
TRINITY_Contig7145	4	p3	(GGC)4	12	4022	4033	TCTTCCTTATCCTCCGCCCA	60.032	TTTGAGCGCAGAGGTAGCAA	59.965	203
TRINITY_Contig7145	5	c	(GGC)5ggaggcggaggcgtcgtcgtctatg gttggt(CTG)5	61	4337	4397	TCTTCCTTATCCTCCGCCCA	60.032	TTTGAGCGCAGAGGTAGCAA	59.965	203
TRINITY_Contig7146	1	p6	(CTCCGC)3	18	2202	2219	AGCTGTACAGGAGGAGGAGG	60.033	GACGTCATCTCCCGATTCCC	59.968	246
TRINITY_Contig7146	2	p3	(GGC)4	12	2460	2471	AGCTGTACAGGAGGAGGAGG	60.033	GACGTCATCTCCCGATTCCC	59.968	246
TRINITY_Contig7146	3	p3	(AGG)4	12	4011	4022	AGCTGTACAGGAGGAGGAGG	60.033	GACGTCATCTCCCGATTCCC	59.968	246
TRINITY_Contig7148	1	p4	(CCCT)3	12	98	109	TACGCCATGGACTGCATGTT	60.036	CCAACCTGTGGGTAGCCATT	59.96	227
TRINITY_Contig7149	1	p6	(CAGGCT)3	18	2007	2024	ATGGCAGGCATTCCATGACA	60.033	TCCACTTTCTTCGCCACTCC	59.965	258
TRINITY_Contig7149	2	p3	(CAG)4	12	2137	2148	ATGGCAGGCATTCCATGACA	60.033	TCCACTTTCTTCGCCACTCC	59.965	258
TRINITY_Contig7157	1	p3	(ACG)4	12	31	42	CGTGTACCAGCAGACCAAGT	59.966	CGCGTGTAGTTGTGCAGATG	59.906	108
TRINITY_Contig7157	2	p3	(ACG)4	12	536	547	CGTGTACCAGCAGACCAAGT	59.966	CGCGTGTAGTTGTGCAGATG	59.906	108
TRINITY_Contig7162	1	p4	(CGGT)4	16	42	57	CCATGCCCATCTCCGTGATC	60.605	TCAGGGAACGCTTCTTCCTG	59.678	123
TRINITY_Contig7168	1	p3	(GAC)5	15	364	378	GGGGAATTTCTGGTCGTCGT	60.037	TTGGTGATCTTGTCGGACCG	60.038	137
TRINITY_Contig7168	2	p6	(CTCCAA)3	18	864	881	GGGGAATTTCTGGTCGTCGT	60.037	TTGGTGATCTTGTCGGACCG	60.038	137
TRINITY_Contig7169	1	p3	(GCT)4	12	119	130	CAGCCTCAGGAAAGTCTGGG	60.036	TGCAGTCGCTGTGTGATTCT	59.966	135
TRINITY_Contig7169	2	p5	(GAGGG)3	15	2699	2713	CAGCCTCAGGAAAGTCTGGG	60.036	TGCAGTCGCTGTGTGATTCT	59.966	135
TRINITY_Contig7170	1	p3	(GCT)4	12	340	351	GTGAAGTCACGGAGGAGGTG	60.038	CAGCTGCCTGCCCCATATAA	59.889	274
TRINITY_Contig7170	2	p3	(CGG)4	12	855	866	GTGAAGTCACGGAGGAGGTG	60.038	CAGCTGCCTGCCCCATATAA	59.889	274
TRINITY_Contig7170	3	p6	(CGTCGC)3	18	1053	1070	GTGAAGTCACGGAGGAGGTG	60.038	CAGCTGCCTGCCCCATATAA	59.889	274
TRINITY_Contig7171	1	p4	(GCCT)3	12	1	12	ATCAGACCTCTGGTTGCTGC	60.036	ATACCGGCCGACATGGAATC	59.965	236
TRINITY_Contig7171	2	p3	(GGC)4	12	405	416	ATCAGACCTCTGGTTGCTGC	60.036	ATACCGGCCGACATGGAATC	59.965	236
TRINITY_Contig7171	3	p3	(GCC)4	12	684	695	ATCAGACCTCTGGTTGCTGC	60.036	ATACCGGCCGACATGGAATC	59.965	236
TRINITY_Contig7172	1	p3	(CTC)4	12	69	80	CATAGACCCGAGGTTGCTCC	59.895	AAACCCTGAAGGCCGCATTA	59.961	199
TRINITY_Contig7173	1	p3	(CGG)4	12	249	260	CCGTTCCTTCAGGCTCCATT	60.035	GTTCTTCTTGCCCCTCCTCC	60.035	141
TRINITY_Contig7173	2	p6	(CCGGCG)3	18	731	748	CCGTTCCTTCAGGCTCCATT	60.035	GTTCTTCTTGCCCCTCCTCC	60.035	141
TRINITY_Contig7174	1	p3	(GCG)4	12	1860	1871	AGCAAGGAAACCCCACCAAA	60.032	TTCCCCCTTGATGGAGTTGC	59.96	260
TRINITY_Contig7177	1	p6	(TGCCGC)3	18	376	393	TCTGGTCCTTGTCGAACACG	59.968	GCCTTCACCCTCTTCGACAA	59.965	247
TRINITY_Contig7177	2	p4	(GAGG)3	12	984	995	TCTGGTCCTTGTCGAACACG	59.968	GCCTTCACCCTCTTCGACAA	59.965	247
TRINITY_Contig7178	1	p4	(GCCA)3	12	224	235	GTGTGTGTGTGAGCTTGCTG	59.971	GCCCAGTTGACAAGCACAAG	59.969	156
TRINITY_Contig7178	2	p3	(CCG)4	12	410	421	GTGTGTGTGTGAGCTTGCTG	59.971	GCCCAGTTGACAAGCACAAG	59.969	156
TRINITY_Contig7179	1	p3	(AAG)4	12	845	856	ATCCACTATGGCATCCGCAG	59.964	GTTTCTGAGGCCCATCTGCT	60.035	190
TRINITY_Contig7180	1	p3	(GGA)4	12	2279	2290	CATGGACTGGAGGAGGAGGA	60.032	CACGACCTCTGATAGTCGGC	59.97	145
TRINITY_Contig7185	1	p6	(CCGCCT)3	18	1732	1749	ACAGCAATGCAGACCGTGTA	59.965	TGCTTTTGTAACGGCTGCAC	59.97	186
TRINITY_Contig7186	1	p4	(CTTT)3	12	255	266	GTCACCAAAACTGACGCACC	59.971	AGAAAGCCTTGGAGATGCCC	60.034	164

TRINITY_Contig7186	2	c	(GCCGGC)3gccggacgaggcgggcgcgg ggtaggagtac(CCG)4	61	1688	1748	GTCACCAAAACTGACGCACC	59.971	AGAAAGCCTTGGAGATGCCC	60.034	164
TRINITY_Contig7188	1	p3	(CCG)4	12	148	159	CTGTGTCCAGTGGTCATGCT	59.964	CTGTGTCCAGTGGTCATGCT	59.964	141
TRINITY_Contig7188	2	c	(CGG)8atctggatttgggtggggtgccggat ttgatttttttttcttcttttttttcgcttcacctg(A GA)7	105	3590	3694	CTGTGTCCAGTGGTCATGCT	59.964	CTGTGTCCAGTGGTCATGCT	59.964	141
TRINITY_Contig7189	1	p4	(GAGG)3	12	1279	1290	CGATGGGAAAAATCGGCGAC	59.972	TGAAACGAATTGGCGGAGGA	59.964	173
TRINITY_Contig7191	1	p6	(GGTATT)4	24	1602	1625	CAGGCCATCTTCTTCCAGCA	60.035	CATTCAGCGCGTACAGCTTC	59.974	187
TRINITY_Contig7191	2	c	(TTC)4agctctcgcagctggtaggaccggac cccaacaagccggaagaggagaccagacac aaagattaggcgtagccgcgtaggt(GTCA) 5gtgttaagt(TAAGC)8	162	1781	1942	CAGGCCATCTTCTTCCAGCA	60.035	CATTCAGCGCGTACAGCTTC	59.974	187
TRINITY_Contig7191	3	p4	(GGAT)3	12	2211	2222	CAGGCCATCTTCTTCCAGCA	60.035	CATTCAGCGCGTACAGCTTC	59.974	187
TRINITY_Contig7191	4	p5	(CTGCA)3	15	2492	2506	CAGGCCATCTTCTTCCAGCA	60.035	CATTCAGCGCGTACAGCTTC	59.974	187
TRINITY_Contig7193	1	p4	(TGCG)3	12	967	978	CCAACCTCAACCTCCCCATC	60.034	TCTTGAATCCGTTCCCCTGC	60.035	178
TRINITY_Contig7196	1	p3	(CCG)5	15	1105	1119	TGCTCCCCTGATGATGATGC	59.89	TGGAGCTCTGCGATTTTCGT	60.037	168
TRINITY_Contig7200	1	p3	(TCA)5	15	576	590	CAACGGTTGTCTGTGCAAGG	59.971	GAGCCTTCCAACGACGAAGA	60.039	253
TRINITY_Contig7200	2	c	(GCG)4gaggtcgtagcgtcctcggggagag gaagaggttttgaagggcct(GAG)7	78	1362	1439	CAACGGTTGTCTGTGCAAGG	59.971	GAGCCTTCCAACGACGAAGA	60.039	253
TRINITY_Contig7200	3	p3	(GCG)5	15	1556	1570	CAACGGTTGTCTGTGCAAGG	59.971	GAGCCTTCCAACGACGAAGA	60.039	253
TRINITY_Contig7201	1	p3	(AGC)4	12	212	223	TGGAGTTTTGCTGTGGTGGT	60.034	CTCGAAATGAAGAAGCGGCG	59.974	258
TRINITY_Contig7201	2	p2	(TC)6	12	443	454	TGGAGTTTTGCTGTGGTGGT	60.034	CTCGAAATGAAGAAGCGGCG	59.974	258
TRINITY_Contig7201	3	p3	(CCG)4	12	738	749	TGGAGTTTTGCTGTGGTGGT	60.034	CTCGAAATGAAGAAGCGGCG	59.974	258
TRINITY_Contig7202	1	p2	(GA)8	16	1034	1049	GCTTGCCCGCCTTTCATATG	59.969	GTTCAGCTTCCTGGCTCCAT	60.035	160
TRINITY_Contig7207	1	p4	(CATA)3	12	123	134	CACCAGGCAAGCAATCAACC	60.038	ACAGAGGTGATGCTTCAGGC	60.036	154
TRINITY_Contig7207	2	c	(GGAG)3atgcgggccgggcatggcgccgc cacgcc(GGGA)3	53	792	844	CACCAGGCAAGCAATCAACC	60.038	ACAGAGGTGATGCTTCAGGC	60.036	154
TRINITY_Contig7208	1	p6	(GGTGCG)3	18	150	167	GACGGCTTGGATTGGAAGGA	60.035	GGCCATTCTGGTCCATCACA	60.034	105
TRINITY_Contig7209	1	c	(CGG)4cgc(TGG)4	27	1353	1379	GACTCAGAGAGTGCCCGAAC	60.109	TTGTTGTGGCTGGGTTCCTT	60.033	167
TRINITY_Contig7210	1	p4	(GCGG)3	12	1063	1074	GCCCAGACTCAGCAGAAGTT	59.964	AGGCGCTGCTTGGATACTTT	60.035	111
TRINITY_Contig7211	1	p3	(AAG)8	24	1248	1271	TTGCAGTATGGGCCCTTGAG	60.034	GGAGCAGGAGGAGGGAAGTA	60.032	170
TRINITY_Contig7212	1	p4	(CAGC)3	12	969	980	CGAGACGATCCAGTACTGCC	59.97	AATGTCGACTGACTTGCCGT	59.966	223
TRINITY_Contig7216	1	c	(TGG)4cgtc(GCT)4cctactctgctctactc ggtgtgaggcgagagccatcagcagcaggcg gctgcagagccgg(AGC)4	101	610	710	TAAACAGACCCTGCTGGCTG	59.963	CACCGTCCGGATGAAGTTGA	60.038	261
TRINITY_Contig7217	1	p6	(AGTTTC)3	18	535	552	TATCCACGCATCCATCACCG	59.967	ATGGAGTGCATGGACTGTGG	60.035	117
TRINITY_Contig7219	1	c	(CCG)4ttggcgaaagctgcagcaggta(TC CC)3	46	889	934	GATTCGTCATCGCGGGTTTG	59.974	TTTCGCGGTTGTACCATCCA	59.965	127
TRINITY_Contig7221	1	p2	(AT)6	12	7	18	CCGCCTCCTTCACCAATCTT	60.035	TAGTGAGCAAGGCTTGGAGC	60.036	175
TRINITY_Contig7223	1	p5	(CGGAT)3	15	79	93	CAACGATGAGATTGCTGCGG	59.973	TCCACTTCGGTCGACTTTGG	59.967	241
TRINITY_Contig7224	1	p2	(GC)6	12	3397	3408	TCGCACACCATCTCAGGTTC	60.037	TTTGCGTGTACGAGGAGCAT	60.038	228
TRINITY_Contig7224	2	p3	(CGG)5	15	3547	3561	TCGCACACCATCTCAGGTTC	60.037	TTTGCGTGTACGAGGAGCAT	60.038	228
TRINITY_Contig7225	1	p3	(CTG)6	18	356	373	CCCCCAATCATGCTCTGGTT	60.033	CTCGTTCTCTCATCTCCGCC	59.97	256
TRINITY_Contig7225	2	p3	(CGC)4	12	596	607	CCCCCAATCATGCTCTGGTT	60.033	CTCGTTCTCTCATCTCCGCC	59.97	256
TRINITY_Contig7225	3	c	(TGC)6attccaaaaccaccaccgctgcaaa aagcttgcgccctctccggcctgcagcagccta caca(CCG)4cttcttctcctgcgcagcagctt cttccactccgcggttgctttcgagcaggcggc gggctggcgaaaaggtggagactgga(GAG G)3	186	1084	1269	CCCCCAATCATGCTCTGGTT	60.033	CTCGTTCTCTCATCTCCGCC	59.97	256
TRINITY_Contig7226	1	c	(CTG)6cagctgccccaagtgccgaggaagg caaggttgctgatgatgccgcctctgcctgaaa acaaggacattgtcttgcga(TTCT)3	108	1153	1260	GCGGTGGTGGAAGAAGAGAA	59.965	CACTCCAGATCAGCTTGCCA	60.036	235
TRINITY_Contig7227	1	c	(CTG)6cagctgccccaagtgccgaggaagg caaggttgctgatgatgccgcctctgcctgaaa acaaggacattgtcttgcga(TTCT)3	108	1158	1265	GCGGTGGTGGAAGAAGAGAA	59.965	CACTCCAGATCAGCTTGCCA	60.036	235
TRINITY_Contig7228	1	c	(ACG)7gcaactacatcgc(GGA)5	49	240	288	CCCCGGTAACAACCCAATCA	59.962	AGGTGATGCGTTGCGAACTA	60.038	228
TRINITY_Contig7228	2	p3	(GTC)4	12	523	534	CCCCGGTAACAACCCAATCA	59.962	AGGTGATGCGTTGCGAACTA	60.038	228
TRINITY_Contig7228	3	c	(CGG)4gggcgaatcccccgcgtcgggcc(G CG)5	50	736	785	CCCCGGTAACAACCCAATCA	59.962	AGGTGATGCGTTGCGAACTA	60.038	228
TRINITY_Contig7228	4	p3	(CCG)4	12	1204	1215	CCCCGGTAACAACCCAATCA	59.962	AGGTGATGCGTTGCGAACTA	60.038	228
TRINITY_Contig7228	5	p3	(GCG)4	12	1977	1988	CCCCGGTAACAACCCAATCA	59.962	AGGTGATGCGTTGCGAACTA	60.038	228
TRINITY_Contig7228	6	p5	(AAAAG)3	15	2224	2238	CCCCGGTAACAACCCAATCA	59.962	AGGTGATGCGTTGCGAACTA	60.038	228
TRINITY_Contig7230	1	p3	(GCC)6	18	620	637	TGTCTCTGCCACTCTTGCTG	59.966	TTCACCATTGCTACGGCCAT	60.035	124
TRINITY_Contig7233	1	p4	(CGGG)3	12	1179	1190	TGCCTGTACTGGAATGCCTG	60.035	AATGCCATCATGCCCACTCA	60.033	277
TRINITY_Contig7235	1	p3	(GCC)4	12	1056	1067	AGGAGGAGGAGGATGACCAC	60.032	ATGTCGCAGTCCAGAACCAG	60.037	199
TRINITY_Contig7235	2	c	(GAG)4gatg(ACC)4	28	1203	1230	AGGAGGAGGAGGATGACCAC	60.032	ATGTCGCAGTCCAGAACCAG	60.037	199
TRINITY_Contig7236	1	p4	(AGGG)3	12	2545	2556	GTCCGCAGTCCTTGAAGACA	59.966	ACACCTCTCCGCTCTTCTCT	59.961	221
TRINITY_Contig7237	1	p5	(AGTTC)3	15	201	215	CAAAGAACCGGCATTGGCAA	59.967	CCCACTGGCAACTCATCCTT	59.961	166
TRINITY_Contig7237	2	p3	(GCC)4	12	386	397	CAAAGAACCGGCATTGGCAA	59.967	CCCACTGGCAACTCATCCTT	59.961	166
TRINITY_Contig7237	3	c*	(TCTCC)2(TCTC<C>T)(CT)17	50	1073	1122	CAAAGAACCGGCATTGGCAA	59.967	CCCACTGGCAACTCATCCTT	59.961	166
TRINITY_Contig7238	1	c	(TCT)5ttttcttctcactgcccttctcatcagcc acatcaacctcctccttgacctccacagcctca gctgatcctaactcacccttgtccttcttt(TTC) 6tcactcttctctccctctactgcctcagcagctt catcaaccttgatcttctttgaaggaacagagg catctgcatcagtgtcctcctcatccttctgcc(TCT)5	240	137	376	ACATCGTGCATGGTCACCAT	60.035	GTGCGGACCATCTACGAGAG	59.97	212
TRINITY_Contig7238	2	c	(GCG)4gggtcgtccttcgacttgtg(CTT)4	44	1675	1718	ACATCGTGCATGGTCACCAT	60.035	GTGCGGACCATCTACGAGAG	59.97	212
TRINITY_Contig7239	1	p2	(GA)11	22	2723	2744	CTGGCACCTCGAAAGAAGGT	59.965	TGCTGAGATACTGGCCCTCT	60.032	262
TRINITY_Contig7241	1	p6	(CCTCCG)3	18	2033	2050	GTAATGTGCATCCGCCGTTC	59.972	CAGAAAACGCCCCAACTTGG	59.968	100
TRINITY_Contig7241	2	p4	(TGAA)3	12	4646	4657	GTAATGTGCATCCGCCGTTC	59.972	CAGAAAACGCCCCAACTTGG	59.968	100
TRINITY_Contig7241	3	p3	(GCC)6	18	5027	5044	GTAATGTGCATCCGCCGTTC	59.972	CAGAAAACGCCCCAACTTGG	59.968	100
TRINITY_Contig7241	4	p4	(ATTT)3	12	5323	5334	GTAATGTGCATCCGCCGTTC	59.972	CAGAAAACGCCCCAACTTGG	59.968	100
TRINITY_Contig7242	1	p6	(CCTCCG)4	24	2033	2056	GTAATGTGCATCCGCCGTTC	59.972	CAGAAAACGCCCCAACTTGG	59.968	100
TRINITY_Contig7243	1	p3	(AGG)4	12	1821	1832	GCCAAATGCAAGCACTGGAA	59.966	GCCCATACTTCAGGCTTGGT	60.034	258
TRINITY_Contig7245	1	c	(CCG)5gcccgcttgccgttgccgcccgcggc gcccgagcacggcgcgacgtgcgccatggact t(GCC)4	86	503	588	GAGGGAGGAGGAGGAGGAAG	60.106	TACACGACGCTCTTCCGATC	59.624	113
TRINITY_Contig7245	2	p3	(GCC)5	15	875	889	GAGGGAGGAGGAGGAGGAAG	60.106	TACACGACGCTCTTCCGATC	59.624	113
TRINITY_Contig7245	3	c	(TGG)4ag(GGA)5	29	993	1021	GAGGGAGGAGGAGGAGGAAG	60.106	TACACGACGCTCTTCCGATC	59.624	113
TRINITY_Contig7246	1	p3	(CCG)5	15	670	684	CTAGCTAGCCGAGGTTCGTG	59.97	CCCATTCCCGGTTTCGTGTA	60.036	244
TRINITY_Contig7246	2	p4	(CCGC)3	12	1063	1074	CTAGCTAGCCGAGGTTCGTG	59.97	CCCATTCCCGGTTTCGTGTA	60.036	244
TRINITY_Contig7246	3	p4	(TTCT)4	16	1194	1209	CTAGCTAGCCGAGGTTCGTG	59.97	CCCATTCCCGGTTTCGTGTA	60.036	244
TRINITY_Contig7246	4	p3	(CTA)4	12	1401	1412	CTAGCTAGCCGAGGTTCGTG	59.97	CCCATTCCCGGTTTCGTGTA	60.036	244
TRINITY_Contig7246	5	p5	(GCAGG)3	15	1919	1933	CTAGCTAGCCGAGGTTCGTG	59.97	CCCATTCCCGGTTTCGTGTA	60.036	244
TRINITY_Contig7247	1	p4	(ATTT)3	12	121	132	GCTGGAATCAAGGGGTTCGA	60.035	CCTTCTCGTAATCCTCGGCC	59.968	128
TRINITY_Contig7247	2	p4	(CCTT)3	12	408	419	GCTGGAATCAAGGGGTTCGA	60.035	CCTTCTCGTAATCCTCGGCC	59.968	128
TRINITY_Contig7248	1	c	(CGG)4gacgc(CTT)5	32	39	70	CAAGAAGCTCACCGACACCT	59.966	CTTCCTCCTCCTCCTCTCCC	60.106	229
TRINITY_Contig7248	2	p3	(GAG)4	12	290	301	CAAGAAGCTCACCGACACCT	59.966	CTTCCTCCTCCTCCTCTCCC	60.106	229
TRINITY_Contig7248	3	c*	(GCC)3(<GCC>GCG)(GCCGCG)2	27	527	553	CAAGAAGCTCACCGACACCT	59.966	CTTCCTCCTCCTCCTCTCCC	60.106	229
TRINITY_Contig7252	1	p5	(GGCGA)3	15	237	251	CGTCAATTTCGTCATCCGCC	59.974	AGGGCAGTCTTCTCTGGGAT	59.956	183
TRINITY_Contig7253	1	p2	(AG)15	30	279	308	CGGCAGTCCTTGTAGCTGAT	59.823	GCTTCTTGGAGGAGGGGAAC	60.035	168
TRINITY_Contig7254	1	p3	(GCT)4	12	902	913	CGCGAATTCCCGTGGTTTAC	59.904	AGGCGAGGAACAACGTGAAT	59.965	261
TRINITY_Contig7254	2	c	(CGG)4aggccttattttccggcgaggc(GG CGGG)3	52	1049	1100	CGCGAATTCCCGTGGTTTAC	59.904	AGGCGAGGAACAACGTGAAT	59.965	261
TRINITY_Contig7254	3	p2	(TC)6	12	1410	1421	CGCGAATTCCCGTGGTTTAC	59.904	AGGCGAGGAACAACGTGAAT	59.965	261
TRINITY_Contig7256	1	p3	(GCC)4	12	144	155	GATTGCTGCTAATGCCAGCC	59.968	AGTACAGACGCTGCAGCATT	60.037	144
TRINITY_Contig7259	1	p3	(CGG)4	12	2635	2646	ATGACACTACCCCTCCCCTC	60.031	CCACCCAACGAATGAGGTCA	59.964	162
TRINITY_Contig7261	1	p3	(CAG)4	12	188	199	AGGGCTAACGAGAGCAGAGA	60.034	AGAGCTTCAGGGCCTTCAAC	59.963	151
TRINITY_Contig7261	2	c	(ACG)4(AGG)5	27	554	580	AGGGCTAACGAGAGCAGAGA	60.034	AGAGCTTCAGGGCCTTCAAC	59.963	151
TRINITY_Contig7261	3	p2	(GA)21	42	1027	1068	AGGGCTAACGAGAGCAGAGA	60.034	AGAGCTTCAGGGCCTTCAAC	59.963	151
TRINITY_Contig7262	1	p3	(GCG)4	12	407	418	AGTCTGTGACCTCCTCCTCC	59.96	CGATCGGCGAGTGGGAATTA	59.968	166
TRINITY_Contig7263	1	p5	(CGCGT)3	15	619	633	GCGGCAGCAAGATCAAGATG	59.971	TCTGTCGCTGTTCTTGGACC	59.966	144

TRINITY_Contig7264	1	p4	(AAGC)3	12	64	75	CGGCCAGAGCATCCTACTAG	59.399	TGCTCTGCTCGTTCGCTAAA	60.039	100
TRINITY_Contig7265	1	c	(GGCCGG)3agccggagccacctcgtccgccgcccccgaggaggaggccattgatcgctcgctgcgtggatccgcgccgcgccggatccgat(GAGG)5tgatgggcgtcggcacccgcgcggg(CTGC)4	162	1964	2125	CGCAAACCAACGACACCATT	59.97	TTCCATTTGGCGGTTGCTTG	59.967	126
TRINITY_Contig7265	2	p6	(CGTCCC)3	18	2284	2301	CGCAAACCAACGACACCATT	59.97	TTCCATTTGGCGGTTGCTTG	59.967	126
TRINITY_Contig7267	1	p4	(CATG)3	12	1022	1033	TCAATGACTTGTGGAGGCCC	59.962	GATGAGGAGCATGAGTCGGG	59.967	181
TRINITY_Contig7269	1	p3	(CTC)4	12	197	208	CGCTTCGCTGGAGAAATTCG	59.974	CATGCGCCATTTACAGCTCC	59.97	252
TRINITY_Contig7269	2	p4	(GTTA)3	12	760	771	CGCTTCGCTGGAGAAATTCG	59.974	CATGCGCCATTTACAGCTCC	59.97	252
TRINITY_Contig7274	1	p3	(GCG)5	15	205	219	AGGCGAAGAAGATGCTTGCT	60.036	ACTCGGTCCTATCAAGGCCT	60.032	183
TRINITY_Contig7278	1	p5	(CTCCT)4	20	95	114	ATGTGGTCAGCTCGTGAGTG	60.038	CCTCAGAAGCTCCTTGGTGG	60.036	217
TRINITY_Contig7278	2	p3	(GGA)6	18	222	239	ATGTGGTCAGCTCGTGAGTG	60.038	CCTCAGAAGCTCCTTGGTGG	60.036	217
TRINITY_Contig7281	1	p4	(ACAT)3	12	8	19	AGGTGTCACGCCCTAGAGAT	60.033	CCCTGTTGCCTGGTTCTAGG	60.035	249
TRINITY_Contig7281	2	p3	(GGC)6	18	2167	2184	AGGTGTCACGCCCTAGAGAT	60.033	CCCTGTTGCCTGGTTCTAGG	60.035	249
TRINITY_Contig7283	1	p3	(GGC)7	21	815	835	CGGTGATAGAGATGGCCACC	59.966	TCGAATGCAGCTTCAAGGGT	59.963	231
TRINITY_Contig7283	2	p3	(CGG)4	12	1070	1081	CGGTGATAGAGATGGCCACC	59.966	TCGAATGCAGCTTCAAGGGT	59.963	231
TRINITY_Contig7285	1	p3	(CGC)4	12	561	572	CAACGTCGCCTCTTCTTCCT	60.038	GATGAGGCGAGGTTCCTAGC	59.967	132
TRINITY_Contig7286	1	p3	(ATC)5	15	3305	3319	CTTCAATGGGGTACGGCAGT	60.035	GCCATCTTGGTCCTCAGCTT	60.035	108
TRINITY_Contig7287	1	p6	(CTTCCT)3	18	917	934	TGCTTGAAGATGCAGCCTCA	59.963	GGTGTTGCTTCTGGTGCTTG	59.969	266
TRINITY_Contig7288	1	c	(CGG)4agtccgaagggtggagaaggccgtcgaggaagt(CGG)4	57	869	925	TCTGCCTTGATGACGACGTC	60.109	CAGTCGTTCGGTGCGATCTA	59.902	101
TRINITY_Contig7293	1	p6	(CCCTCT)5	30	83	112	ATTGGCATCTCTCCCCCTCT	60.029	AATTCCACGATCCAACGGCT	60.035	252
TRINITY_Contig7294	1	p6	(GAGGGA)3	18	54	71	GCTGGCTTCTTGGCTAGGAA	60.035	GAGATCTCGCTCCACCCATG	59.967	105
TRINITY_Contig7295	1	p3	(CGG)5	15	1856	1870	AGTGGGCGAGACAGTGAAAG	59.966	GGCCTCGTTGTCGAAAGGTA	60.038	232
TRINITY_Contig7296	1	p3	(GCA)6	18	1157	1174	TCGGTCTCGTACGCAAACAA	59.969	AGCTCTGCAGTGTTCAGGTC	59.965	268
TRINITY_Contig7297	1	c	(CCG)5tgatgcagcatcacctacgccgat(GCG)4	51	213	263	ATCAGGTCCTGGGGCATTTG	60.033	CAGCACTAGTGTGAACCCGT	59.966	104
TRINITY_Contig7298	1	p3	(TTC)6	18	303	320	CATCAGTCATGCTTCAGCGC	59.972	CTGGGAGCCTGTTCGTCTTT	59.965	148
TRINITY_Contig7298	2	p3	(CGC)4	12	4251	4262	CATCAGTCATGCTTCAGCGC	59.972	CTGGGAGCCTGTTCGTCTTT	59.965	148
TRINITY_Contig7299	1	c	(GGA)6agaagcctaccactctatgacagcctggacccacaaaggttg(GTGGA)3	75	378	452	CTTCCTCGTAGCTGAGCTCG	59.971	ATGGAACTCTGCTGGCTCAC	60.036	187
TRINITY_Contig7302	1	p3	(GCG)5	15	116	130	TGGATTCGCTCGTTTCAGCT	60.037	AGCTGAAACTTCGTCGTCGT	59.97	101
TRINITY_Contig7302	2	p3	(TCT)4	12	1032	1043	TGGATTCGCTCGTTTCAGCT	60.037	AGCTGAAACTTCGTCGTCGT	59.97	101
TRINITY_Contig7303	1	p3	(CCA)4	12	201	212	TGGCACCTCCACAGATCAAC	59.963	GACCATTGGCATGTCCTCCA	60.034	109
TRINITY_Contig7304	1	p3	(CCT)4	12	870	881	CCAACATGATCAGGGGCTGT	60.034	GCAGTGCCCGGTTAGTTTTG	60.04	258
TRINITY_Contig7304	2	p3	(GGC)4	12	1207	1218	CCAACATGATCAGGGGCTGT	60.034	GCAGTGCCCGGTTAGTTTTG	60.04	258
TRINITY_Contig7305	1	p3	(CCG)5	15	197	211	TTGCCAACGATACTGCCAGT	59.964	AGATCCCCACTGGCTCATCT	60.03	134
TRINITY_Contig7305	2	p3	(CCG)6	18	2064	2081	TTGCCAACGATACTGCCAGT	59.964	AGATCCCCACTGGCTCATCT	60.03	134
TRINITY_Contig7306	1	p3	(CGG)5	15	828	842	CTTGACGGGCTTGTAGCTGA	60.037	GCAAGGCGAAAAAGGAAGCA	59.968	200
TRINITY_Contig7311	1	p4	(GGGA)3	12	178	189	AGCAACCAATCGAACCGCTA	60.037	AGCAGGAAGAGGAGGAGGAG	60.032	223
TRINITY_Contig7311	2	p3	(CTC)4	12	334	345	AGCAACCAATCGAACCGCTA	60.037	AGCAGGAAGAGGAGGAGGAG	60.032	223
TRINITY_Contig7311	3	c	(CCG)4aagctaccggcggcgaccgtagccgccgcc(GCA)4	54	800	853	AGCAACCAATCGAACCGCTA	60.037	AGCAGGAAGAGGAGGAGGAG	60.032	223
TRINITY_Contig7311	4	p3	(CGT)4	12	1041	1052	AGCAACCAATCGAACCGCTA	60.037	AGCAGGAAGAGGAGGAGGAG	60.032	223
TRINITY_Contig7312	1	c	(CCTC)4cacccacgggccgcagcaggccccat(GGCC)3	55	1724	1778	CGATGCCATTGCCATCCATG	60.039	GAGAGGAGCTTCGAGGATGC	59.968	146
TRINITY_Contig7313	1	p3	(TCC)5	15	237	251	CAGGTGGCTGTGTTGCTAGA	59.965	TAGTGCTACGGGAGTCTGCT	60.034	108
TRINITY_Contig7313	2	p3	(GCC)4	12	1073	1084	CAGGTGGCTGTGTTGCTAGA	59.965	TAGTGCTACGGGAGTCTGCT	60.034	108
TRINITY_Contig7313	3	p3	(CGC)4	12	1275	1286	CAGGTGGCTGTGTTGCTAGA	59.965	TAGTGCTACGGGAGTCTGCT	60.034	108
TRINITY_Contig7314	1	c	(GGC)4gctaacgagcgcttcccggggggcagcgacccgctgctcccgacgaagcgggagggg(GAC)4	81	543	623	TCGTCATGATAGTGCTCGCC	59.969	ATCGGTTCCGGAAAGCACAT	60.035	218
TRINITY_Contig7317	1	c	(TC)18(AC)20	76	368	443	TGCGATACAACGACCGAACA	60.039	ATCGGTGTGTGTGTGTGTGT	60.108	213
TRINITY_Contig7318	1	p3	(CGC)4	12	1141	1152	AGATGCAGAGCTATGGCGAC	59.967	GAACTGGCTTACGCTCCACT	60.037	106
TRINITY_Contig7319	1	c	(CGG)5tgctgcgggacgccgaggcgctgctcaaggggaacctgacgcgggcgcccccggagcaccgtgacgccgcggtgcgcgccctgcgcggctggct(CGGCG)3	124	618	741	TTCCACTACTCGTCGGGGAT	60.034	CGGTAGAACTCGTACTCGGC	59.973	167
TRINITY_Contig7320	1	c	(CCA)4ccgcgt(CCG)4	30	355	384	ACGACGTCCTCGACAACTTC	60.041	GCTGAGTATCCAGGGGATGC	59.964	140
TRINITY_Contig7320	2	p3	(CCG)4	12	630	641	ACGACGTCCTCGACAACTTC	60.041	GCTGAGTATCCAGGGGATGC	59.964	140
TRINITY_Contig7320	3	p3	(GGC)4	12	875	886	ACGACGTCCTCGACAACTTC	60.041	GCTGAGTATCCAGGGGATGC	59.964	140
TRINITY_Contig7320	4	p3	(CCG)5	15	2162	2176	ACGACGTCCTCGACAACTTC	60.041	GCTGAGTATCCAGGGGATGC	59.964	140
TRINITY_Contig7321	1	p4	(CGGC)4	16	1470	1485	CAACGCAACACACATGCTGA	59.971	CCAGGGGGAGATCAGGAAGA	60.031	110
TRINITY_Contig7323	1	c	(GAGC)3atatttttgcctttgccgctgtgctcattccattcggagctcgaaagcttcgcctac(CTTTT)3	84	1396	1479	GGCCTACATCCAGCAGCTAG	59.965	TCTATCTCGAACGCAGCGAC	59.972	165
TRINITY_Contig7323	2	p3	(GAC)4	12	2778	2789	GGCCTACATCCAGCAGCTAG	59.965	TCTATCTCGAACGCAGCGAC	59.972	165
TRINITY_Contig7326	1	p6	(GGGCTC)3	18	2838	2855	GACGTGTACTGCTTGGGACA	59.967	ATCTTGCAGAGGAAAGGGGC	60.034	135
TRINITY_Contig7327	1	p4	(AAAC)3	12	299	310	CAAAACAGACGCCAATGCCA	59.968	CTGACAGGGTTCTGCCTCAG	60.037	266
TRINITY_Contig7330	1	p6	(AGACGA)4	24	20	43	GGTGGGCAGCTATGAGTGTT	60.035	GCACAGCTAATGACCCTGGT	60.035	102
TRINITY_Contig7330	2	p4	(TTTC)3	12	1723	1734	GGTGGGCAGCTATGAGTGTT	60.035	GCACAGCTAATGACCCTGGT	60.035	102
TRINITY_Contig7331	1	p4	(AAGG)3	12	1379	1390	GCGGTTCGGTCTCTACTACG	59.973	ACCTACCTCCTACGGCATGT	60.032	227
TRINITY_Contig7334	1	p3	(CTC)4	12	477	488	CAGTACACGCGCGTTACATG	59.977	CTGTGCAGGGAATACGGGTT	60.035	183
TRINITY_Contig7334	2	p6	(CGTCCT)4	24	642	665	CAGTACACGCGCGTTACATG	59.977	CTGTGCAGGGAATACGGGTT	60.035	183
TRINITY_Contig7334	3	p6	(GTGCGA)3	18	1953	1970	CAGTACACGCGCGTTACATG	59.977	CTGTGCAGGGAATACGGGTT	60.035	183
TRINITY_Contig7334	4	c	(GGC)4gc(AG)8	30	3249	3278	CAGTACACGCGCGTTACATG	59.977	CTGTGCAGGGAATACGGGTT	60.035	183
TRINITY_Contig7335	1	p3	(CGG)4	12	78	89	TGAAGAGGCGAGATGGAGGA	60.033	CAAAGAAATGGGGCGTCAGC	60.109	100
TRINITY_Contig7336	1	p6	(CGCCTC)3	18	187	204	TATGATGATCGCCCCTGTGC	59.965	AGGCATGGCAGAAATGTCGA	60.035	138
TRINITY_Contig7336	2	c	(CGC)4gggggtcgcttcctcgccttccgcaacgagcagtccgcgggcta(CGC)4	69	562	630	TATGATGATCGCCCCTGTGC	59.965	AGGCATGGCAGAAATGTCGA	60.035	138
TRINITY_Contig7338	1	p3	(ACA)5	15	394	408	ATCGTTGCCTTGCCTTCGTA	60.037	CACACGAGGTGTCCATGTCA	59.967	193
TRINITY_Contig7338	2	p6	(GAAGCC)3	18	537	554	ATCGTTGCCTTGCCTTCGTA	60.037	CACACGAGGTGTCCATGTCA	59.967	193
TRINITY_Contig7339	1	p4	(CGGG)3	12	3722	3733	GAAAACAACCACCCACGACG	59.972	ACTGGCTCCGAAGAGAGGAT	60.033	181
TRINITY_Contig7340	1	c	(GTGC)3tctgcttttgctctcag(GCGT)3	41	211	251	GCGCCTTTCTGGAATATGCG	60.041	CTCCTCACGACCACACCTTC	60.038	226
TRINITY_Contig7340	2	p2	(GC)6	12	510	521	GCGCCTTTCTGGAATATGCG	60.041	CTCCTCACGACCACACCTTC	60.038	226
TRINITY_Contig7344	1	c	(TAG)5tcctacaatcaaaaggag(GGAA)6(GCAA)3	69	118	186	AATTGCAGGAGGAGCTGCTT	59.961	ATGACTGCGAACATGCCTCA	60.036	155
TRINITY_Contig7348	1	p3	(GCG)4	12	1120	1131	ACCAATGCCGTCACCTTGAT	59.962	CCAGTTCGCCATCTTGACGA	60.39	192
TRINITY_Contig7357	1	p3	(AAG)4	12	1228	1239	ATCGGCCAACACCTTATGCA	60.035	TGGCAATGTGTTGAAGCGTG	59.969	185
TRINITY_Contig7357	2	p3	(GCG)6	18	1489	1506	ATCGGCCAACACCTTATGCA	60.035	TGGCAATGTGTTGAAGCGTG	59.969	185
TRINITY_Contig7358	1	p3	(GAC)4	12	76	87	CAGAGCAGCAACAAAACGCT	59.97	CAACAACACTGGGCAAGACG	59.971	254
TRINITY_Contig7358	2	p3	(GGA)4	12	1696	1707	CAGAGCAGCAACAAAACGCT	59.97	CAACAACACTGGGCAAGACG	59.971	254
TRINITY_Contig7360	1	p3	(CGT)4	12	997	1008	GAAGTGGGACGTGTTCTCGT	59.968	GAATCGAGGCACTTGTTCGC	59.905	231
TRINITY_Contig7360	2	p3	(CGG)4	12	1979	1990	GAAGTGGGACGTGTTCTCGT	59.968	GAATCGAGGCACTTGTTCGC	59.905	231
TRINITY_Contig7361	1	c	(CT)9tctaggcagcagcggtagcaaggctagctaggctcccgctctcccatccatttcgtccccattgcatgcg(AAGC)3aagaggtcaagaggagc(AGCG)3	129	1226	1354	TCACTTTGCCCCTAGTGCTG	59.963	GTAGATCTACCCGGTTGGCG	59.968	204
TRINITY_Contig7361	2	p3	(GGA)5	15	1479	1493	TCACTTTGCCCCTAGTGCTG	59.963	GTAGATCTACCCGGTTGGCG	59.968	204
TRINITY_Contig7361	3	c	(CCG)4ttcgc(GCA)5ggcggcccaggcgccgtcgcctgcggcgcccatgcccggcggcatgcgcctctcgtttgaccagatggcggggaagacggag(CAC)4gcggcgcccatgctgtacg(CGC)6	163	1799	1961	TCACTTTGCCCCTAGTGCTG	59.963	GTAGATCTACCCGGTTGGCG	59.968	204
TRINITY_Contig7361	4	p3	(CGC)4	12	2231	2242	TCACTTTGCCCCTAGTGCTG	59.963	GTAGATCTACCCGGTTGGCG	59.968	204
TRINITY_Contig7362	1	p2	(GC)7	14	88	101	ATGGGGGACTACTTCGACGA	60.034	CACCAAGGCGCCCAACTATA	60.107	244
TRINITY_Contig7362	2	p6	(CGGGGG)3	18	308	325	ATGGGGGACTACTTCGACGA	60.034	CACCAAGGCGCCCAACTATA	60.107	244
TRINITY_Contig7364	1	p3	(GCG)4	12	3125	3136	TCGAACCTGAACACCAGCTC	59.966	TCACTACCTCCTCCTGCTCC	60.033	211
TRINITY_Contig7365	1	p3	(AGG)4	12	328	339	ACATCCGTCGCATCCTCATC	59.967	CAAGCAGATGCACCAGGAGA	60.036	240
TRINITY_Contig7366	1	p3	(AGG)6	18	141	158	CAATGGAAGCGATGGCATGG	59.968	ATTGCATTCCAGTCGCCAGA	60.035	189
TRINITY_Contig7367	1	p2	(AG)9	18	1702	1719	GAATGTGGACGCATCATGCC	59.97	CAGAAAGGCCCGATAGCGAT	59.966	266
TRINITY_Contig7367	2	p5	(CGCGG)3	15	1852	1866	GAATGTGGACGCATCATGCC	59.97	CAGAAAGGCCCGATAGCGAT	59.966	266
TRINITY_Contig7367	3	p5	(GGACC)3	15	1975	1989	GAATGTGGACGCATCATGCC	59.97	CAGAAAGGCCCGATAGCGAT	59.966	266
TRINITY_Contig7368	1	p3	(GCG)4	12	34	45	GGAAGAAGGAGGGCAATGCT	60.034	CGCTGGGAAGATGGGATTGA	59.819	106

TRINITY_Contig7368	2	p4	(GGGC)3	12	193	204	GGAAGAAGGAGGGCAATGCT	60.034	CGCTGGGAAGATGGGATTGA	59.819	106
TRINITY_Contig7373	1	c	(CTTC)3gggcctgcgcagccgaccgaggccgacgcca(CCG)4	55	1774	1828	CAAGTTTGTGCTCCTTGCCC	59.967	AAGCGTAACCAGAAGCCGAA	59.966	172
TRINITY_Contig7375	1	p4	(ATTC)3	12	53	64	GGGGCGATTCTAGCTGGTAC	59.966	GGAAGGAGGGGAGCTCAGTA	60.032	138
TRINITY_Contig7375	2	c	(ACTG)3atccaggttgttatttcgaccatggtgacgacgctgac(CGAT)3	62	335	396	GGGGCGATTCTAGCTGGTAC	59.966	GGAAGGAGGGGAGCTCAGTA	60.032	138
TRINITY_Contig7376	1	p4	(GGGA)4	16	61	76	TACTCCGCCGCGTTAATCTC	59.97	CCAATGCAGCCTCAAGGAGA	60.035	123
TRINITY_Contig7377	1	p5	(CTCTG)3	15	2963	2977	CCCATAAGTGGCTCCAGCAA	60.034	AAAACTTCCAGTGCCCACCA	60.033	230
TRINITY_Contig7378	1	p3	(GGA)5	15	2107	2121	TCCTGGAAAGCCTTGCTAGC	60.035	GGGATGTTGGTGATCCTGCA	60.034	148
TRINITY_Contig7378	2	p4	(ACGG)3	12	2262	2273	TCCTGGAAAGCCTTGCTAGC	60.035	GGGATGTTGGTGATCCTGCA	60.034	148
TRINITY_Contig7379	1	p5	(CGTTT)5	25	1	25	CGCGTCGAATAAGTCCCGTA	59.972	CACATCGGCTGATCCTCTCC	59.967	144
TRINITY_Contig7379	2	p4	(CCGG)3	12	255	266	CGCGTCGAATAAGTCCCGTA	59.972	CACATCGGCTGATCCTCTCC	59.967	144
TRINITY_Contig7379	3	p4	(TATC)3	12	2647	2658	CGCGTCGAATAAGTCCCGTA	59.972	CACATCGGCTGATCCTCTCC	59.967	144
TRINITY_Contig7381	1	p3	(GCG)4	12	1	12	TTGGAGATGGGGTGGTTTCG	59.962	CGTCTCGTGTTGGGACAGAA	59.968	230
TRINITY_Contig7381	2	p2	(GA)10	20	210	229	TTGGAGATGGGGTGGTTTCG	59.962	CGTCTCGTGTTGGGACAGAA	59.968	230
TRINITY_Contig7385	1	p4	(CTCC)3	12	21	32	TCTCTCTCCCTCCCTCCTCT	59.953	CCTTCGCTACCCCCACAATT	60.034	116
TRINITY_Contig7385	2	p3	(CGG)4	12	607	618	TCTCTCTCCCTCCCTCCTCT	59.953	CCTTCGCTACCCCCACAATT	60.034	116
TRINITY_Contig7385	3	c	(GGGGCG)3gagcagggcccgccgtcggcgctctctcggccatggagctgctgcggagctccggctgctacatgccgttgca(GATGCC)4cggagactacggtgcggcagggttcgcgctcggagagttccgcg(CGC)5	174	775	948	TCTCTCTCCCTCCCTCCTCT	59.953	CCTTCGCTACCCCCACAATT	60.034	116
TRINITY_Contig7385	4	p3	(GCG)4	12	1279	1290	TCTCTCTCCCTCCCTCCTCT	59.953	CCTTCGCTACCCCCACAATT	60.034	116
TRINITY_Contig7389	1	c	(GCCG)4gggcccctctataaatactagcctcgggtaccccgac(CCGG)3	65	187	251	AAGATCGCGGACTTTGGTGT	59.965	TCCCTGCCTTGCACCTAATG	60.034	225
TRINITY_Contig7390	1	p3	(CTC)4	12	1949	1960	CTCAGGAGCCCAGGTTCTTG	60.036	GCTGATGCTCCTATGGGTCC	59.964	165
TRINITY_Contig7390	2	c	(GAG)4gaagagga(GGCGCC)3	38	2187	2224	CTCAGGAGCCCAGGTTCTTG	60.036	GCTGATGCTCCTATGGGTCC	59.964	165
TRINITY_Contig7391	1	c	(TC)6cctctcaccccgcgg(GTC)4	39	911	949	GCTCCCGAGGCAGTAGAATC	59.967	CGTTTTCAGGTGCAAGAGGC	60.04	196
TRINITY_Contig7392	1	p6	(CAGCTC)3	18	498	515	TGGCATAAGCGAGACTAGCG	59.969	AATTCTACGTGCACGACCGT	60.039	206
TRINITY_Contig7393	1	c	(AATCG)3cggg(GAGC)3	31	122	152	AGAGTGCGGCATAAAGGGAC	60.108	TGCAACAGGGAAATCGAGCT	59.963	129
TRINITY_Contig7394	1	p3	(CCG)5	15	695	709	CAAGTGTTAGCCTCCTGGGG	60.035	CGAGACACTATCACGAGGCC	59.97	167
TRINITY_Contig7396	1	p3	(CGC)4	12	463	474	AGGCTCAGCAATCGGTTGAA	59.963	TTTAATGAGCCGAGCAGCCA	60.036	235
TRINITY_Contig7396	2	p3	(CCG)4	12	759	770	AGGCTCAGCAATCGGTTGAA	59.963	TTTAATGAGCCGAGCAGCCA	60.036	235
TRINITY_Contig7396	3	p3	(CCT)5	15	1069	1083	AGGCTCAGCAATCGGTTGAA	59.963	TTTAATGAGCCGAGCAGCCA	60.036	235
TRINITY_Contig7396	4	c	(GAGG)3aggtggaggatggagggaatcggatcgcgaattgaagggctattaatctcggggtcaaccacctcgattcct(TC)6	96	1310	1405	AGGCTCAGCAATCGGTTGAA	59.963	TTTAATGAGCCGAGCAGCCA	60.036	235
TRINITY_Contig7397	1	c	(GCCGCA)4gccgccgcggcgtccatggagagcccgtggtcggggtagtggaacgacatcgccgttgcggggcgggcggggtcggcggcggcgagtcgagggcggggc(GGCGCG)3ggcgcaacgaccccggcggttgggggttttggt(TGG)4	186	1435	1620	GATGAGGATGACGCCGGATT	59.966	GAAACTCCCTGGGGACACAG	59.963	189
TRINITY_Contig7398	1	c	(AGA)4aggaggagaaggagaagaagaaggaggaggaag(AGA)4	57	381	437	TGGAGGCGCTTGGATTTCAT	60.034	CGGTGTGAATTGGTGCGTTT	59.97	151
TRINITY_Contig7400	1	p3	(CGG)4	12	1292	1303	CAGCCCAGTACACGTTCCTT	59.965	ACTGTTCCGAGCACCTGATG	60.037	156
TRINITY_Contig7401	1	p3	(GCT)4	12	630	641	CGTTGCGCCCAAGATTGAAT	59.829	TAGCAAAGCTGAGCCACGAA	59.965	250
TRINITY_Contig7402	1	p4	(TCCC)3	12	91	102	AGCTGTTGTCTGATGTGGGG	59.963	GCCACCGCCAATAGTAAGGT	60.107	135
TRINITY_Contig7402	2	c	(CGA)4ctgcgctgat(GAG)4	34	617	650	AGCTGTTGTCTGATGTGGGG	59.963	GCCACCGCCAATAGTAAGGT	60.107	135
TRINITY_Contig7402	3	p5	(CGCCG)3	15	794	808	AGCTGTTGTCTGATGTGGGG	59.963	GCCACCGCCAATAGTAAGGT	60.107	135
TRINITY_Contig7403	1	p4	(AAAT)3	12	412	423	GTCCGAGCTGTCCAAGAACA	59.966	CGTTGTTTTTCCGGTGGCAT	59.969	197
TRINITY_Contig7403	2	c	(CTGGGA)3ggaggagatgaaggccccatcgggtgatatctgggtcttggtcgtacaaaaccatgtctccacttggtgaacgaatgagaaatctcttg(TTC)4tcgtttgagcggtaaggaagcgagttgcaagtaccgtgggatgtcgccttcgggatgatgcgatggcgacctgacctcacctccgggt(GCC)5	222	1354	1575	GTCCGAGCTGTCCAAGAACA	59.966	CGTTGTTTTTCCGGTGGCAT	59.969	197
TRINITY_Contig7403	3	p2	(CT)6	12	1775	1786	GTCCGAGCTGTCCAAGAACA	59.966	CGTTGTTTTTCCGGTGGCAT	59.969	197
TRINITY_Contig7405	1	p3	(CCA)4	12	417	428	GAGTCGATCGTCTCCGGAAC	59.972	GTCGACACTGACCGTTCCTT	59.968	227
TRINITY_Contig7405	2	p4	(GGCG)3	12	2522	2533	GAGTCGATCGTCTCCGGAAC	59.972	GTCGACACTGACCGTTCCTT	59.968	227
TRINITY_Contig7407	1	p3	(CCG)4	12	75	86	TTCAACGTCGGGGATTTCGT	59.966	TCCATCATGTCGTCGTAGCG	59.971	101
TRINITY_Contig7409	1	p6	(GCGGAC)3	18	366	383	CTTCCTTGCACCTTTTGGGC	59.966	TGACAAACTCATCCGCGTGA	59.967	110
TRINITY_Contig7411	1	p6	(TCCCCC)3	18	59	76	TCCCGTGCCTGTGAAGTTAC	59.966	ATGCCTAGCTGGAGGTGAGA	60.032	136
TRINITY_Contig7411	2	p4	(AAAG)3	12	1577	1588	TCCCGTGCCTGTGAAGTTAC	59.966	ATGCCTAGCTGGAGGTGAGA	60.032	136
TRINITY_Contig7415	1	p3	(CCT)4	12	164	175	CACAGGTTAGCTTGGGCGTA	60.037	CTGTCCCTCCAAGCCTGAAG	60.036	190
TRINITY_Contig7415	2	p2	(CG)8	16	461	476	CACAGGTTAGCTTGGGCGTA	60.037	CTGTCCCTCCAAGCCTGAAG	60.036	190
TRINITY_Contig7415	3	p4	(AAAC)3	12	1715	1726	CACAGGTTAGCTTGGGCGTA	60.037	CTGTCCCTCCAAGCCTGAAG	60.036	190
TRINITY_Contig7416	1	c	(GGTA)3ggtgatgaggaggtttgcaccccattgcagaagaacagccaagaatgatttgcctacaga(CAGC)3	84	331	414	AAGCCTCTCAACCAAGACGG	59.965	TGTCCTGAATCCTTGGACGC	60.036	131
TRINITY_Contig7416	2	p2	(GA)12	24	1707	1730	AAGCCTCTCAACCAAGACGG	59.965	TGTCCTGAATCCTTGGACGC	60.036	131
TRINITY_Contig7419	1	p5	(GCGCG)3	15	1484	1498	AGATGGAGTAGGCGTCCCTT	60.032	TCTTCCGGTGCCTTGTTCTC	59.965	112
TRINITY_Contig7420	1	p6	(CGCCGT)3	18	198	215	TCGGAACAGCAGCATCAGTT	59.965	TTTGTCAAGGGTTGGTGGCT	60.033	171
TRINITY_Contig7420	2	p3	(CGG)4	12	1112	1123	TCGGAACAGCAGCATCAGTT	59.965	TTTGTCAAGGGTTGGTGGCT	60.033	171
TRINITY_Contig7420	3	p5	(CCGCG)3	15	1514	1528	TCGGAACAGCAGCATCAGTT	59.965	TTTGTCAAGGGTTGGTGGCT	60.033	171
TRINITY_Contig7422	1	p3	(GCC)5	15	1723	1737	GCCGATGCCTCGTTAGATGA	59.968	CGGATGACCCATCACGGTAG	59.968	230
TRINITY_Contig7423	1	c	(CA)6(CG)10cacacag(AT)12ag(TA)9	83	305	387	TCTGAAGCACAGGAGGCAAG	59.964	CCCTGGATGTACGCCTGTTT	60.035	160
TRINITY_Contig7423	2	p4	(GACG)3	12	500	511	TCTGAAGCACAGGAGGCAAG	59.964	CCCTGGATGTACGCCTGTTT	60.035	160
TRINITY_Contig7423	3	p3	(GGA)5	15	1465	1479	TCTGAAGCACAGGAGGCAAG	59.964	CCCTGGATGTACGCCTGTTT	60.035	160
TRINITY_Contig7425	1	p3	(GGC)4	12	707	718	CCTCCTCCAGCACCTTCTTG	60.036	GCAGAAGGAAGGAAGCCAT	60.034	130
TRINITY_Contig7426	1	p4	(CCAA)3	12	208	219	AGCGTATATCAGCTGGCACC	59.966	TCATTTTGCATCGCGTCGTC	59.905	129
TRINITY_Contig7426	2	p4	(TCGA)3	12	923	934	AGCGTATATCAGCTGGCACC	59.966	TCATTTTGCATCGCGTCGTC	59.905	129
TRINITY_Contig7426	3	p3	(GCA)4	12	1418	1429	AGCGTATATCAGCTGGCACC	59.966	TCATTTTGCATCGCGTCGTC	59.905	129
TRINITY_Contig7428	1	p3	(AAG)4	12	1329	1340	AGAGCACCACCACAACAACA	60.035	GCCGGGGACTTCATCTTTCA	60.035	263
TRINITY_Contig7428	2	p3	(CTC)5	15	1505	1519	AGAGCACCACCACAACAACA	60.035	GCCGGGGACTTCATCTTTCA	60.035	263
TRINITY_Contig7431	1	p3	(GGA)4	12	2609	2620	CATTGGCCCCCATGACTTCT	60.033	TTTCCGTCAGAAGCAGGCAT	59.963	192
TRINITY_Contig7431	2	p3	(TGG)4	12	2734	2745	CATTGGCCCCCATGACTTCT	60.033	TTTCCGTCAGAAGCAGGCAT	59.963	192
TRINITY_Contig7433	1	p3	(CGC)5	15	331	345	TTCATGTCGCAGTTCTCGCT	60.038	CCACCTCCTCAAGCATGGTT	59.961	104
TRINITY_Contig7434	1	p6	(CGCCGG)3	18	150	167	GTTCGAGAAGGTGTCCCTGG	60.037	GCTGAACCTCCTCCTGAACC	60.036	262
TRINITY_Contig7436	1	p3	(CCA)4	12	904	915	GGAGAAGTACTACGCGCACA	59.83	AGATGTGCCTTCTGGTGGTG	59.963	162
TRINITY_Contig7440	1	c	(GCCA)3caaactctgccacgggaagggcctgacctgacttgtccgtcaccaggccaaacatggcgctcgcgtcca(CCG)5ctgtgctcaagaccccgttcctcggggcccggcgcgcgctcgccaacgccgcctccgtcgccgccaggc(CCGCG)3	180	335	514	CAACTAGCTCACGATTGCGC	59.974	CAGCACCCAAAAGAAGCCAC	59.967	162
TRINITY_Contig7441	1	p3	(TTC)5	15	2598	2612	CACTGGTGCTTTTGGCTGAC	59.969	TTCCGTACTTGTCGTCGCAA	59.969	202
TRINITY_Contig7443	1	p3	(GCC)4	12	180	191	ACTCCTTGGGCAGCTCATTC	60.035	CAAGTGCACTGCTTGACACC	59.97	117
TRINITY_Contig7444	1	p3	(GCG)7	21	209	229	CTTCCCTTGCTTCCCGGAAT	60.034	ACACCTGAATCACCGACGAC	60.039	279
TRINITY_Contig7445	1	p3	(TCA)4	12	502	513	TGCTCGTCGCTTCCTTTTCT	59.966	AGGTGGGCAAATCGAAGAGG	60.035	174
TRINITY_Contig7447	1	p4	(GCGA)3	12	1975	1986	AACAAACAGGCGAGCGTAGA	59.967	GTGGAGAGTGTACGGTTGCA	59.967	227
TRINITY_Contig7447	2	p5	(GGTTT)3	15	2171	2185	AACAAACAGGCGAGCGTAGA	59.967	GTGGAGAGTGTACGGTTGCA	59.967	227
TRINITY_Contig7448	1	c	(GCGGC)3gcggtgctgtgttgtgctgtgtacaagactcctccctctccgatcccatggcctaccgccggaagcaggg(CGC)4	97	176	272	ATAAGGCCCCTCAGTCTCGT	60.032	CCGAATGTATCCGCGACAGA	59.97	256
TRINITY_Contig7449	1	p6	(GAGAGG)3	18	1340	1357	CTTCCCAGGCTTCTTGGAGG	60.035	TTTTGTGAAGAGCAGCGTGC	59.971	154
TRINITY_Contig7451	1	p2	(GA)11	22	1237	1258	CGGAAGCGCGCAGTAAATAC	60.043	CCTCGCTTCTCCTCGTGATC	59.97	100
TRINITY_Contig7452	1	p3	(GGC)4	12	1030	1041	GCCCATCCTCTCCTGAATCG	59.965	CAGTTCAGAAGCAGCCCAGA	59.964	246
TRINITY_Contig7458	1	c	(TTG)4gaagttcaccgttatgttga(GTT)4	44	726	769	TGGCTTCGGGGTACTACGTA	60.035	TGATGTCGCTCGATGGATGG	59.967	246
TRINITY_Contig7459	1	p3	(GGC)7	21	439	459	TGTTCGGCAAAAACGAGCAG	59.971	CAGAACCTCGCCTTGTCCTT	59.965	225
TRINITY_Contig7460	1	c	(CGCC)3ctcctctcgctacaagaat(CCGC)3	43	139	181	TGGGCAGGCCTCTCTCTATT	60.031	GCAGAACTTCACGACCAGGA	59.966	243

TRINITY_Contig7461	1	p3	(GCC)4	12	1726	1737	CCCCCTCCCACATGTTCTTC	60.034	CACGGTGGTGTACAAGTCGA	59.969	161
TRINITY_Contig7462	1	p5	(CGTCT)3	15	85	99	TTACTCGAGCCACCACCAAC	59.966	GTCCGGTGATCAGAACAGCA	60.037	133
TRINITY_Contig7462	2	p3	(CAA)4	12	685	696	TTACTCGAGCCACCACCAAC	59.966	GTCCGGTGATCAGAACAGCA	60.037	133
TRINITY_Contig7462	3	p3	(CAG)5	15	1463	1477	TTACTCGAGCCACCACCAAC	59.966	GTCCGGTGATCAGAACAGCA	60.037	133
TRINITY_Contig7462	4	p3	(CAG)6	18	1661	1678	TTACTCGAGCCACCACCAAC	59.966	GTCCGGTGATCAGAACAGCA	60.037	133
TRINITY_Contig7463	1	p3	(CCG)4	12	255	266	GCCCCAGGACCTACAGAGTA	60.032	TACCGAGCTCCTTTACCCGA	60.034	228
TRINITY_Contig7463	2	p4	(AGTC)5	20	2225	2244	GCCCCAGGACCTACAGAGTA	60.032	TACCGAGCTCCTTTACCCGA	60.034	228
TRINITY_Contig7465	1	p3	(CGC)4	12	512	523	GCGCAGGAGATCTGGTAGTC	59.969	CACCTCCTCCTCCTAAGCCT	60.032	188
TRINITY_Contig7465	2	p3	(AGG)4	12	1561	1572	GCGCAGGAGATCTGGTAGTC	59.969	CACCTCCTCCTCCTAAGCCT	60.032	188
TRINITY_Contig7466	1	p3	(CCG)4	12	1340	1351	GCATACGTCCCTCTGAGAGC	59.969	TGGACGCTCCATATTCGAGC	59.968	153
TRINITY_Contig7469	1	p2	(AG)11	22	939	960	TCGCGTCAACAAAAGGCTTG	59.971	TTGCAAATGAAGTCTGCGGC	60.039	257
TRINITY_Contig7469	2	p2	(TA)9	18	1242	1259	TCGCGTCAACAAAAGGCTTG	59.971	TTGCAAATGAAGTCTGCGGC	60.039	257
TRINITY_Contig7469	3	p3	(AGC)4	12	1414	1425	TCGCGTCAACAAAAGGCTTG	59.971	TTGCAAATGAAGTCTGCGGC	60.039	257
TRINITY_Contig7469	4	p4	(GCTA)3	12	1611	1622	TCGCGTCAACAAAAGGCTTG	59.971	TTGCAAATGAAGTCTGCGGC	60.039	257
TRINITY_Contig7469	5	p4	(CATG)3	12	1954	1965	TCGCGTCAACAAAAGGCTTG	59.971	TTGCAAATGAAGTCTGCGGC	60.039	257
TRINITY_Contig7469	6	p2	(AG)20	40	2988	3027	TCGCGTCAACAAAAGGCTTG	59.971	TTGCAAATGAAGTCTGCGGC	60.039	257
TRINITY_Contig7470	1	p3	(GCG)4	12	1218	1229	CCCGTCCTCAACCAAGACAA	59.892	TCAGGAGCCAAGGATGCAAG	60.035	163
TRINITY_Contig7471	1	p3	(GCG)7	21	54	74	CTATCGCTCCGCTGCCTATT	59.755	CCATTGATCCAGGAGACCCG	59.893	133
TRINITY_Contig7471	2	p3	(CCT)5	15	390	404	CTATCGCTCCGCTGCCTATT	59.755	CCATTGATCCAGGAGACCCG	59.893	133
TRINITY_Contig7472	1	p5	(GCGCG)3	15	283	297	TTAGCGAGCGAGCACTACTG	59.901	GGAAGTCGAAGTAGAGCGGG	59.899	129
TRINITY_Contig7473	1	p3	(GAC)4	12	208	219	GAGCTTTTGGGGTGTTGCAG	59.967	GCTATGTGCCACAGGGTCTT	60.035	254
TRINITY_Contig7474	1	p3	(CGC)5	15	66	80	ACCCTTTCTTCCTCGGCATG	60.035	CCACGTTTGGTTTGACCGTC	59.972	159
TRINITY_Contig7474	2	p3	(CCT)5	15	218	232	ACCCTTTCTTCCTCGGCATG	60.035	CCACGTTTGGTTTGACCGTC	59.972	159
TRINITY_Contig7476	1	p3	(GGC)5	15	2138	2152	TGCCATTTCCATCTTCCCCC	60.032	GAATCTAGTCGAGGCTGGGC	59.967	226
TRINITY_Contig7477	1	p5	(GGGAC)3	15	620	634	GAGGCTTCTTCCACCTCACC	60.036	ATCGCCTCGTTTGGGTTCAT	60.035	129
TRINITY_Contig7477	2	p3	(GGT)4	12	1175	1186	GAGGCTTCTTCCACCTCACC	60.036	ATCGCCTCGTTTGGGTTCAT	60.035	129
TRINITY_Contig7479	1	c	(GAGC)3gaggggtggagtgaaggcgaggagg(AATC)4	53	2208	2260	CGCTCAGCTGGATCAATTGC	59.971	GGCTCCCCTTCAGAGGAGTA	60.032	164
TRINITY_Contig7480	1	p4	(AACC)3	12	13	24	CTTGGGTGGCTCTCCTGAAG	60.036	TGCAAAGAACAAGTGCGACG	59.973	134
TRINITY_Contig7482	1	p3	(ATC)4	12	2164	2175	GACAGCCTCAGAGATCGACG	59.971	AACAGGCACCGCGGTTATTA	60.036	171
TRINITY_Contig7483	1	p3	(TGG)7	21	2169	2189	GCAAAGTTTGGCCACGTCAT	59.968	GCCCTCTTTCAGCATCACCT	60.035	101
TRINITY_Contig7488	1	c	(CGG)5gaggccggcgcggcgtcggggatggggga(CGGG)3	56	91	146	GTTCTGACGCTGACAAGGGA	59.966	GGTTCGCTGGGTCACAAAAC	59.97	267
TRINITY_Contig7490	1	p4	(TCCC)3	12	34	45	GCGATCGATCCGAACTCACT	59.97	CCCCTGGATGTTCTGAACCC	60.034	252
TRINITY_Contig7491	1	p3	(CGC)4	12	3116	3127	AGTTGTCAAACGCACCATGC	59.969	GGCTGACTAGTGAGATCGCC	59.969	119
TRINITY_Contig7491	2	c	(CCG)4tgcaggagcagcgcggcgagcacgaggattg(GGA)5	58	3382	3439	AGTTGTCAAACGCACCATGC	59.969	GGCTGACTAGTGAGATCGCC	59.969	119
TRINITY_Contig7492	1	p3	(CCG)5	15	1576	1590	ACTTGCCAATCGCCTTAGCT	60.035	GGTTTTACCTGGAGGACGCA	59.964	180
TRINITY_Contig7492	2	p6	(CGCCGG)3	18	1739	1756	ACTTGCCAATCGCCTTAGCT	60.035	GGTTTTACCTGGAGGACGCA	59.964	180
TRINITY_Contig7493	1	p4	(TCTA)3	12	8	19	ATTGCCCCAGCTCTTCTGTC	60.035	GAGTGATCATTCCGTTGCGC	59.973	165
TRINITY_Contig7493	2	p3	(CAG)5	15	2576	2590	ATTGCCCCAGCTCTTCTGTC	60.035	GAGTGATCATTCCGTTGCGC	59.973	165
TRINITY_Contig7494	1	p3	(CCG)5	15	916	930	GAGCAGAGGAGGGGTTAGGA	60.032	AGAAATATCTACCCGCGGCG	60.039	150
TRINITY_Contig7497	1	p4	(ACCC)3	12	94	105	GCTCCGTTCCAGAAAAGGGA	59.964	ACATTGCAGGCTCTGTTCGA	59.965	158
TRINITY_Contig7498	1	p4	(GCGA)3	12	1436	1447	TTCTTTGCTGAGGGCGCATA	60.036	GCTGCATTGGACATGTTGCA	60.038	223
TRINITY_Contig7499	1	p3	(CCG)4	12	213	224	TCTCCTCCAGTCATCCCAGG	60.032	CCAATGGAGGAACGGAGCAT	60.107	151
TRINITY_Contig7500	1	p3	(CGG)5	15	349	363	AACAACAACAACGGGAAGCG	59.9	CTGCACGGACTTGGTCTTCT	59.966	222
TRINITY_Contig7500	2	c	(CAG)6caacagcagcagcac(CAA)4cgggaagcggaagcgc(GAG)4	73	490	562	AACAACAACAACGGGAAGCG	59.9	CTGCACGGACTTGGTCTTCT	59.966	222
TRINITY_Contig7502	1	p2	(AC)11	22	601	622	ATGAACCGGTGCTCTGTCTG	60.037	CAAGCAAGCAAGCAGAAGCA	59.968	242
TRINITY_Contig7502	2	p4	(TGCT)3	12	1659	1670	ATGAACCGGTGCTCTGTCTG	60.037	CAAGCAAGCAAGCAGAAGCA	59.968	242
TRINITY_Contig7504	1	p3	(CGC)5	15	1783	1797	GGAACTGGCTCCTTGGAGAC	60.036	ACCGCGAGTATGTGATCGTC	59.971	115
TRINITY_Contig7504	2	p3	(CGG)4	12	2117	2128	GGAACTGGCTCCTTGGAGAC	60.036	ACCGCGAGTATGTGATCGTC	59.971	115
TRINITY_Contig7505	1	c	(CCT)6(ATCC)3	30	538	567	AACTCCATGAACTCCGGCAG	60.036	TGGTGTTGTCCGAGGATGTG	59.965	205
TRINITY_Contig7506	1	p3	(CCT)4	12	104	115	CAGTGCTAGGTGGGTCTTGG	60.036	TTTTCCGCCGCAGAAATTGG	60.039	172
TRINITY_Contig7508	1	p4	(TATC)3	12	140	151	AGTTCTTCGTCCGCTTCTGG	60.038	CCAAAATTCGGTCGATCGCC	59.972	244
TRINITY_Contig7508	2	p3	(CCA)4	12	1152	1163	AGTTCTTCGTCCGCTTCTGG	60.038	CCAAAATTCGGTCGATCGCC	59.972	244
TRINITY_Contig7508	3	p3	(TGC)7	21	1904	1924	AGTTCTTCGTCCGCTTCTGG	60.038	CCAAAATTCGGTCGATCGCC	59.972	244
TRINITY_Contig7509	1	p4	(CGGC)3	12	219	230	AGGTAGGAAAAAGCCCACGG	59.962	GGAGAAGAGGGGGATCTGGT	60.031	122
TRINITY_Contig7509	2	p3	(CGC)6	18	343	360	AGGTAGGAAAAAGCCCACGG	59.962	GGAGAAGAGGGGGATCTGGT	60.031	122
TRINITY_Contig7510	1	p3	(GGA)5	15	134	148	TTGGAGTATTGGGTTCCCGC	60.035	CAACTTGCCACATGAACGCA	59.969	255
TRINITY_Contig7510	2	c	(AGG)6agctgcctcaggggcacccagagacggacacaccg(CTC)5	68	345	412	TTGGAGTATTGGGTTCCCGC	60.035	CAACTTGCCACATGAACGCA	59.969	255
TRINITY_Contig7510	3	p4	(CATA)3	12	7148	7159	TTGGAGTATTGGGTTCCCGC	60.035	CAACTTGCCACATGAACGCA	59.969	255
TRINITY_Contig7512	1	c	(GCG)5gggatagaagcgcgacatccaggtaacctctgatttttgccccgattgggtgcgttggcgtcggcgatggaggagccgaatgtgggcggaggtgcc(GCG)5gatcaggctggtgtcggcgctggagtcgggctggcgcatcgccctggaggaagacca(CGC)4	196	231	426	TCTCTCACTCAGCGCTCTCT	60.036	TGGATGTCGCGCTTCTATCC	59.968	256
TRINITY_Contig7512	2	p3	(CCT)4	12	576	587	TCTCTCACTCAGCGCTCTCT	60.036	TGGATGTCGCGCTTCTATCC	59.968	256
TRINITY_Contig7513	1	p5	(GAGAC)3	15	29	43	GCTCTTGTCGTCCTTGTCCA	59.966	TGTGTCGCTCCTTGCATTCT	59.965	121
TRINITY_Contig7513	2	p3	(CAG)4	12	5738	5749	GCTCTTGTCGTCCTTGTCCA	59.966	TGTGTCGCTCCTTGCATTCT	59.965	121
TRINITY_Contig7513	3	p3	(TGG)4	12	6222	6233	GCTCTTGTCGTCCTTGTCCA	59.966	TGTGTCGCTCCTTGCATTCT	59.965	121
TRINITY_Contig7514	1	c	(GCCC)5gagtccgtccgttcccgtcgcgccacaggacaaggcgacccaaccgccgcccgtctcccaccgtcatccgccgatcgatcgatccattgg(CTC)4	118	455	572	ATCCATTGGCTCCTCCTCCT	60.029	GATCGTCATCAGGTCCGTCC	59.969	218
TRINITY_Contig7516	1	p4	(CCTC)3	12	68	79	CCTTGAACCGAGTCGTGGAA	59.967	CAATGGCATTCCGCATCCTG	59.968	100
TRINITY_Contig7516	2	p3	(GCC)4	12	5291	5302	CCTTGAACCGAGTCGTGGAA	59.967	CAATGGCATTCCGCATCCTG	59.968	100
TRINITY_Contig7516	3	p2	(CT)9	18	5495	5512	CCTTGAACCGAGTCGTGGAA	59.967	CAATGGCATTCCGCATCCTG	59.968	100
TRINITY_Contig7517	1	p2	(GA)7	14	678	691	CACACGGTAAAGCTCCCTGA	59.68	CTTGATTTACCGTGCTCGCG	59.975	108
TRINITY_Contig7518	1	p4	(TCGA)3	12	1264	1275	GACACGGTTTCTTTGGGCAC	59.97	AACTCCAGAGCTCATGTGGC	60.036	101
TRINITY_Contig7519	1	p5	(GCACG)3	15	521	535	CTCCAGATGCAGTGCAGTGA	60.037	CACCAGCCACTATACACGCA	60.108	240
TRINITY_Contig7519	2	c	(CGA)4gggcgccgacttcgcctccgccgccgcgcagttcatccccgccggcgccgcgcccctcccggggttcgcgtcgctcgtcggccatgtgccgccgcg(GCA)6	126	648	773	CTCCAGATGCAGTGCAGTGA	60.037	CACCAGCCACTATACACGCA	60.108	240
TRINITY_Contig7519	3	p4	(TTAA)5	20	1174	1193	CTCCAGATGCAGTGCAGTGA	60.037	CACCAGCCACTATACACGCA	60.108	240
TRINITY_Contig7522	1	p3	(CGG)4	12	1540	1551	GAGAAGGAGAGCACGACGAG	59.902	AGTACCTCGACCCCACCTAC	60.034	175
TRINITY_Contig7523	1	p3	(GCG)4	12	1044	1055	TGCGGAGTTGCTGTAGATGG	60.108	TTCGACAAGGACAACACGCT	60.179	126
TRINITY_Contig7524	1	p3	(GGT)5	15	503	517	AACATGGACGACGGCTTCAT	60.036	CTCTCCTTGGTTTCGCTCGT	60.038	229
TRINITY_Contig7528	1	p3	(CGG)4	12	715	726	GGAAGCCGCTCCTCTACATC	59.967	CAGCGGGTAGATCTCCGAAC	59.969	206
TRINITY_Contig7528	2	p3	(GGT)4	12	2117	2128	GGAAGCCGCTCCTCTACATC	59.967	CAGCGGGTAGATCTCCGAAC	59.969	206
TRINITY_Contig7531	1	p2	(CT)7	14	11	24	TCCGGCAAGCGTACTTATCC	59.896	ACTTCCGGACGTTTCTGACC	59.967	137
TRINITY_Contig7532	1	p4	(TTTC)3	12	217	228	GGGCTACAGCAGTGTTGACT	59.965	CTTCCCTGCAGCTTCCATCA	60.035	109
TRINITY_Contig7534	1	p3	(CGC)5	15	511	525	TACCATTTCGCGACACCCAA	59.965	TCGTGTCGGAACAGACCAAG	59.968	236
TRINITY_Contig7536	1	c	(CACG)3gccggcatggcgccgtcccccccc(CG)7	50	919	968	GCCTGACCAAGTCATCCACA	59.963	ACTTCCTGCTGGCCTTCATC	60.035	239
TRINITY_Contig7536	2	p3	(CGG)4	12	1085	1096	GCCTGACCAAGTCATCCACA	59.963	ACTTCCTGCTGGCCTTCATC	60.035	239
TRINITY_Contig7537	1	p3	(CGG)4	12	80	91	ATACTGGTGTGGAAGCGCAA	59.964	TCTCGCAAGCCACCATCTTT	59.963	107
TRINITY_Contig7541	1	p4	(CATC)3	12	211	222	GCGCATCATCACAGAGCAAG	59.972	CCAGAGCTGACGACGAGATC	59.971	101
TRINITY_Contig7541	2	p3	(GCA)4	12	474	485	GCGCATCATCACAGAGCAAG	59.972	CCAGAGCTGACGACGAGATC	59.971	101
TRINITY_Contig7541	3	p4	(ATTA)3	12	1043	1054	GCGCATCATCACAGAGCAAG	59.972	CCAGAGCTGACGACGAGATC	59.971	101
TRINITY_Contig7542	1	p3	(CGC)4	12	192	203	ACCCTCGATCGGTTGGTTTC	60.037	TTCAGCGTCTCACCACCTTC	59.966	205
TRINITY_Contig7542	2	p4	(GGGC)5	20	515	534	ACCCTCGATCGGTTGGTTTC	60.037	TTCAGCGTCTCACCACCTTC	59.966	205
TRINITY_Contig7544	1	p3	(GCC)4	12	754	765	AAGAGGAGACGTTGCACTGG	59.966	GAGCCAGAGGGAAAACACGA	59.965	171
TRINITY_Contig7544	2	p3	(CGA)4	12	2662	2673	AAGAGGAGACGTTGCACTGG	59.966	GAGCCAGAGGGAAAACACGA	59.965	171
TRINITY_Contig7546	1	p3	(TTC)4	12	1166	1177	ACTGCGAAGGAAATGGAGCA	59.963	CCAAGAAACCTGCACCGTTG	59.969	162
TRINITY_Contig7547	1	p3	(CGC)4	12	94	105	GTCAAGACCAAGGCCATCGA	60.036	CCAAGAAACCTGCACCGTTG	59.969	232
TRINITY_Contig7553	1	p4	(CATG)3	12	322	333	CGGGTTGGGGTCTGCTTTAT	60.034	CAGCCGGGGAGATGTATCAC	59.966	130
TRINITY_Contig7553	2	p3	(CGT)4	12	1215	1226	CGGGTTGGGGTCTGCTTTAT	60.034	CAGCCGGGGAGATGTATCAC	59.966	130
TRINITY_Contig7556	1	c	(AGC)4acccaccggcacgcgcggcgtcggccggccgcccggcgccgccgcagatctggggcgcgggccggggggcaggaagcggcgagcggaaggcagca(GGCG)3	119	1841	1959	GCTCCTCTTCCAATGGGCTT	60.034	ATGGCGATGATGGTGGATCC	59.963	125

TRINITY_Contig7556	2	p5	(GCGAG)4	20	2102	2121	GCTCCTCTTCCAATGGGCTT	60.034	ATGGCGATGATGGTGGATCC	59.963	125
TRINITY_Contig7556	3	p4	(GCGG)3	12	2244	2255	GCTCCTCTTCCAATGGGCTT	60.034	ATGGCGATGATGGTGGATCC	59.963	125
TRINITY_Contig7557	1	p6	(GGGCGC)3	18	353	370	CGTCTTCGTCTCCACCAACA	59.968	ATCGTTGCACTTCGTCAGGT	59.966	261
TRINITY_Contig7559	1	p3	(TGC)5	15	411	425	GGAGTCCACCATTGAAGGGG	60.034	AGCTAAACTGCGGGATGCTT	60.035	113
TRINITY_Contig7559	2	p6	(GGCGGG)3	18	1044	1061	GGAGTCCACCATTGAAGGGG	60.034	AGCTAAACTGCGGGATGCTT	60.035	113
TRINITY_Contig7559	3	p3	(GCG)7	21	1316	1336	GGAGTCCACCATTGAAGGGG	60.034	AGCTAAACTGCGGGATGCTT	60.035	113
TRINITY_Contig7560	1	c	(CGC)4ctcagcgctcgccgccgtcgaggccggcgactacgaggaagttct(CGC)4cccgcacctcctcccggcctccgcctcctccgggccgcccggctccgccgcgcagttctacgccgacctg(GCC)4	151	202	352	TGGTACAGCTGCATGGAAGG	60.035	ATGTGCAGGCAGGCTATGTT	60.034	117
TRINITY_Contig7561	1	p3	(GCT)5	15	3310	3324	AATGTTGTCTCCAGCAGCGA	59.965	TGACGTCTGATTGCACGGAA	59.967	236
TRINITY_Contig7563	1	p2	(TC)6	12	283	294	ATTTGGCTGGCCCAGACTAC	60.034	TTTGCGGCAAACCAAGATGG	59.967	214
TRINITY_Contig7564	1	p3	(CCG)4	12	1279	1290	AATCCTGGCCCGGTTACAAG	60.034	GAGCGATGAGAGCCTCTGTC	59.969	144
TRINITY_Contig7566	1	p3	(GGT)4	12	1477	1488	CTGTGACCAACAACTGCTGC	59.97	GTCGAACAAGGTTGTGGTGC	59.971	246
TRINITY_Contig7569	1	p3	(CGC)4	12	2563	2574	GAGATGATGTGGCCGGTAGG	59.966	GAATCATGCCTCTGCGCAAG	59.971	240
TRINITY_Contig7569	2	p3	(GGC)4	12	3561	3572	GAGATGATGTGGCCGGTAGG	59.966	GAATCATGCCTCTGCGCAAG	59.971	240
TRINITY_Contig7570	1	p3	(TGG)4	12	1794	1805	CTGGTCTCTCGATCTGCGAC	59.971	TCCATTGTTGCCGCTTCTCT	59.963	137
TRINITY_Contig7571	1	p3	(CAT)4	12	53	64	CCGCTGTTGTCGAAGGAGAT	60.109	CGACTACAAGTCCTGGGCAG	60.109	150
TRINITY_Contig7571	2	p3	(CTT)4	12	378	389	CCGCTGTTGTCGAAGGAGAT	60.109	CGACTACAAGTCCTGGGCAG	60.109	150
TRINITY_Contig7572	1	p3	(CGG)5	15	109	123	GACCAGAAGAAGGACGCCAA	59.965	ATCACACCCACCACGATCAC	60.036	193
TRINITY_Contig7575	1	p3	(CGG)4	12	558	569	CTGTAGTTGGGGTTCCGGTC	60.037	ATCTTTCTGGGAACGGGCTG	60.035	128
TRINITY_Contig7576	1	p3	(ACC)4	12	315	326	GGCAGCTACCACGTCTCTTT	60.037	GGTAGACCCGTGCAGATAGC	59.968	184
TRINITY_Contig7578	1	p4	(GCCA)3	12	792	803	ACCCCGGATCTACTCCTAGC	59.887	TGGCATGTCACGGTCATGAA	59.964	174
TRINITY_Contig7579	1	p3	(CGG)4	12	1137	1148	GGTCACCGCAACAAAGACAC	59.971	ACTGGATTCTGCGATTGCCA	60.035	175
TRINITY_Contig7579	2	p3	(AGC)4	12	1310	1321	GGTCACCGCAACAAAGACAC	59.971	ACTGGATTCTGCGATTGCCA	60.035	175
TRINITY_Contig7581	1	c	(TAG)7tgtgctacaagctgcgggcaaagggagagagaaaggaaagcatcgaatcacatcctccattatatttatctgtattccggcatcggtccatcgatcg(CCTT)3	130	473	602	CCTCCTGACCTCTAGTGGCT	60.033	GCGGCGGATTGATCTCCTTA	59.966	272
TRINITY_Contig7581	2	p3	(ACC)4	12	875	886	CCTCCTGACCTCTAGTGGCT	60.033	GCGGCGGATTGATCTCCTTA	59.966	272
TRINITY_Contig7585	1	p3	(GCG)5	15	209	223	GCTTCGAGATCCTGTCCCTG	59.896	GTCCTCGTCGTTGTTGTTGC	60.042	163
TRINITY_Contig7585	2	p3	(AGC)4	12	809	820	GCTTCGAGATCCTGTCCCTG	59.896	GTCCTCGTCGTTGTTGTTGC	60.042	163
TRINITY_Contig7588	1	c	(GCT)4caacctcgtcctgtacgtcttcatgatggggttcgccagctgggcgctcaacagcttcgtcgacg(GCC)4	89	438	526	GAGAGATCTGGAGAGCACGC	59.969	GGCGAACCCCATCATGAAGA	60.107	158
TRINITY_Contig7589	1	p3	(TTG)4	12	353	364	CTACGGCATGCACATCGTTG	59.973	GTCTGGGTGTATGCCTTGCT	60.035	197
TRINITY_Contig7592	1	p3	(GCA)7	21	1745	1765	GAAAGCAGGCCACAGTGTTG	59.969	TTCTGAGCACTCTGCCACTG	59.966	132
TRINITY_Contig7592	2	p3	(GCG)4	12	1910	1921	GAAAGCAGGCCACAGTGTTG	59.969	TTCTGAGCACTCTGCCACTG	59.966	132
TRINITY_Contig7594	1	p3	(CGC)4	12	52	63	CGATCTGATCTAGCGTGCGT	60.041	CCCAAAGCTCCAGATCTGCA	60.035	210
TRINITY_Contig7594	2	p3	(CGC)5	15	198	212	CGATCTGATCTAGCGTGCGT	60.041	CCCAAAGCTCCAGATCTGCA	60.035	210
TRINITY_Contig7594	3	p6	(CGCCGG)3	18	369	386	CGATCTGATCTAGCGTGCGT	60.041	CCCAAAGCTCCAGATCTGCA	60.035	210
TRINITY_Contig7594	4	c	(TTC)4cgggtctgctcattccattcggggtttagtctagggcgcgtccgatctgatcta(GCGT)4	82	604	685	CGATCTGATCTAGCGTGCGT	60.041	CCCAAAGCTCCAGATCTGCA	60.035	210
TRINITY_Contig7596	1	p3	(TGC)4	12	463	474	CTCAGGATCAACACGCCTGT	60.037	GAGTGAGAGCAGCGTTGTCT	60.039	103
TRINITY_Contig7596	2	p3	(GCG)4	12	874	885	CTCAGGATCAACACGCCTGT	60.037	GAGTGAGAGCAGCGTTGTCT	60.039	103
TRINITY_Contig7596	3	p3	(TCA)4	12	1385	1396	CTCAGGATCAACACGCCTGT	60.037	GAGTGAGAGCAGCGTTGTCT	60.039	103
TRINITY_Contig7597	1	p3	(GCG)6	18	478	495	AAGATCGACCTGGAGAGCCT	60.033	TTCAGCTTCCGTTGCAAAGC	59.969	246
TRINITY_Contig7598	1	p3	(AGC)4	12	28	39	CTCATCTGCATGGCTCGGAT	59.965	TTTGCCAGCCCAAAATCAGC	59.965	199
TRINITY_Contig7598	2	p4	(TGCC)4	16	2418	2433	CTCATCTGCATGGCTCGGAT	59.965	TTTGCCAGCCCAAAATCAGC	59.965	199
TRINITY_Contig7601	1	p3	(CTA)4	12	413	424	GCTTTTCGCAGCTTGTCACA	59.971	AGCTCCACTGAAAATCCCGG	60.035	101
TRINITY_Contig7601	2	p3	(GGA)5	15	2769	2783	GCTTTTCGCAGCTTGTCACA	59.971	AGCTCCACTGAAAATCCCGG	60.035	101
TRINITY_Contig7603	1	c*	(AC)5(<AC>A)(ACA)4catgtggcaaacaca(ATC)5ttcatcttcctaataatgaacctacggcaacaacatctttacacgcg(CTTTC)3	117	135	251	ACAGCACACCGAAAAATGCC	59.968	TGAGGCACAGATGGGTGTTC	59.963	128
TRINITY_Contig7603	2	c	(GCT)4ggtggttctcgtcgccctcggtctcggcg(GGC)4	53	1650	1702	ACAGCACACCGAAAAATGCC	59.968	TGAGGCACAGATGGGTGTTC	59.963	128
TRINITY_Contig7603	3	p4	(TTGC)3	12	2286	2297	ACAGCACACCGAAAAATGCC	59.968	TGAGGCACAGATGGGTGTTC	59.963	128
TRINITY_Contig7603	4	p6	(GAGATG)3	18	2405	2422	ACAGCACACCGAAAAATGCC	59.968	TGAGGCACAGATGGGTGTTC	59.963	128
TRINITY_Contig7604	1	c	(CGC)4cccccgagcccgccgagccccagagaaggacagcacgatggttc(AGA)4	68	72	139	CAGCTTGAGGGCAGTGAAGA	59.964	CGCTTGCAACTCCATATGGC	59.97	174
TRINITY_Contig7608	1	p3	(TTA)4	12	2996	3007	GGCCCCTTCAATGTAACCCA	59.96	TCTGCTTCACCAATGGCCAT	59.96	256
TRINITY_Contig7609	1	c	(TTG)10ctgttgccacc(GTT)7	62	271	332	TCAGCGTGTTACCACAGTCC	59.967	TCCATTCGCTGGCAAGAAGT	59.963	137
TRINITY_Contig7610	1	p3	(GTG)8	24	668	691	ATCCAACCAGCTTGACCAGG	59.961	TAGCAATCGGGAGCTTGGTG	60.108	166
TRINITY_Contig7610	2	p6	(GCTGCG)5	30	818	847	ATCCAACCAGCTTGACCAGG	59.961	TAGCAATCGGGAGCTTGGTG	60.108	166
TRINITY_Contig7611	1	p3	(CGG)4	12	2087	2098	TGATTTGCTGCTCCGACTGT	59.965	GTATGCGCCATCTCGGTACA	59.968	196
TRINITY_Contig7611	2	c*	(CGAG)2(CGA<G>GGAG)(GGGAG)4	36	2252	2287	TGATTTGCTGCTCCGACTGT	59.965	GTATGCGCCATCTCGGTACA	59.968	196
TRINITY_Contig7616	1	p3	(CCT)4	12	369	380	TTGTGAGATGGCGGCTGATT	60.035	CCCCAAAAGATGTGGCTTGC	60.037	195
TRINITY_Contig7621	1	p3	(GAC)4	12	842	853	TCGGGGTTCAGCTTCAAGAC	59.965	ATGTGCAGCATCACCATGGA	60.034	276
TRINITY_Contig7622	1	p4	(CGCA)3	12	4	15	CACAGCTCCAATGCCCACTA	60.035	TCCTACCTAGCTCAGCTGCA	60.033	256
TRINITY_Contig7624	1	p3	(GCG)4	12	549	560	TCGCCGTCATCAATCTCCTG	59.897	CTCGCTCGAGCTTTCCTAGG	59.97	232
TRINITY_Contig7625	1	p4	(AGCA)4	16	75	90	TTCCGCCCTCCATACTACCA	60.032	CGAGGGATGAGCGAAGACTC	59.97	239
TRINITY_Contig7630	1	p4	(CCAG)3	12	174	185	AGTATACGCCAAGCCCACAC	60.108	CTACGCTCCTCTTCGACGTC	59.973	159
TRINITY_Contig7630	2	p3	(GGA)4	12	644	655	AGTATACGCCAAGCCCACAC	60.108	CTACGCTCCTCTTCGACGTC	59.973	159
TRINITY_Contig7631	1	c	(CACG)3catccacccgagtccccaaccgcagccgagcacga(CGC)4	59	27	85	TGGAGATCCATCCGAATGCG	59.966	TGAGATCCCAGGAGCCATGA	60.031	233
TRINITY_Contig7633	1	p4	(AAGG)3	12	937	948	GCGCCATCGACAGATTGAAC	59.973	TTGCAAGTGCCACATTGCTC	59.968	217
TRINITY_Contig7635	1	p6	(CTGGGC)4	24	761	784	TGCAACAGGTAGCAGGTAGC	60.036	TACCCGTGCTCGACCTACTT	60.323	118
TRINITY_Contig7637	1	c	(AGC)4ggcctcttccttcacctcagcattcttcttgctcttcttcagtgagatacccttgaatttgaaggagttcttcagggagaa(CTT)6	111	234	344	TCGGCCTTAGGGGTTTCAAC	59.963	CTGCTCCAGCTACCGAAGAG	59.898	118
TRINITY_Contig7641	1	p3	(CGC)6	18	70	87	ACCCGCACTGTAACTGACTG	59.966	CAGAAGCTGGCCTCCTTGAA	59.963	239
TRINITY_Contig7641	2	p3	(GGC)4	12	235	246	ACCCGCACTGTAACTGACTG	59.966	CAGAAGCTGGCCTCCTTGAA	59.963	239
TRINITY_Contig7641	3	p6	(CGGCGC)3	18	874	891	ACCCGCACTGTAACTGACTG	59.966	CAGAAGCTGGCCTCCTTGAA	59.963	239
TRINITY_Contig7645	1	p3	(CCG)4	12	109	120	GCCGAAAGATGAGGCAGAGA	59.823	TCTACACCGCAGCACATCAG	60.109	114
TRINITY_Contig7646	1	p2	(GA)20	40	786	825	TGAGGTGCCTCCATGGGATA	60.03	TCCAGAGGTGGGTCATCGAT	60.032	109
TRINITY_Contig7650	1	p3	(CGC)4	12	959	970	TCACCAAAGCCGTAGAGCAG	60.037	CAGAACTCCTGCGTTCCAGT	59.966	279
TRINITY_Contig7650	2	p3	(GGC)5	15	1100	1114	TCACCAAAGCCGTAGAGCAG	60.037	CAGAACTCCTGCGTTCCAGT	59.966	279
TRINITY_Contig7652	1	p4	(TCCA)3	12	228	239	AGAGGTGGAGCCAGAAAACG	59.965	TATCCGTGTGCTTGTGTGCT	59.965	225
TRINITY_Contig7652	2	p3	(GCC)4	12	401	412	AGAGGTGGAGCCAGAAAACG	59.965	TATCCGTGTGCTTGTGTGCT	59.965	225
TRINITY_Contig7653	1	p4	(CCGT)3	12	10	21	ATGATTGCTGGCTGGAGGTC	60.107	CAAACATCTGCCGCCCAAAA	59.967	169
TRINITY_Contig7653	2	p3	(AAG)4	12	187	198	ATGATTGCTGGCTGGAGGTC	60.107	CAAACATCTGCCGCCCAAAA	59.967	169
TRINITY_Contig7658	1	p3	(GCG)4	12	1064	1075	CCACTCCTTTCGCTTCCCTT	59.963	AACAGAGATGTGGGCGGAAG	60.036	148
TRINITY_Contig7658	2	p3	(GCG)7	21	1211	1231	CCACTCCTTTCGCTTCCCTT	59.963	AACAGAGATGTGGGCGGAAG	60.036	148
TRINITY_Contig7663	1	p4	(CCGC)3	12	514	525	CCACACTCCAACAAGCTTGC	59.969	AGCCGTCCATTACTTGGGTG	60.035	125
TRINITY_Contig7663	2	c	(GGTT)4tggctggctgcacctccaccgcaccggcatgccccac(CG)6gcgtgcagccgtgca(GGT)5	95	1289	1383	CCACACTCCAACAAGCTTGC	59.969	AGCCGTCCATTACTTGGGTG	60.035	125
TRINITY_Contig7668	1	p3	(AGG)4	12	1824	1835	AGAGCCATCATCCATCAGCG	59.965	TCCCAGACTCTTTGCAGCAG	59.964	111
TRINITY_Contig7670	1	c	(GCGCCG)4acagggacgccaccgctggcattgctggttggacggtggcggcgcaaagtcctccgtttcggaatcg(GATGA)3	106	1943	2048	AAATCCACCGCCCGATCTTT	60.034	CAATGGGAGCACTGGGGAAT	60.033	144
TRINITY_Contig7672	1	p5	(CCTCT)3	15	33	47	GAGGAAGGCATGAAGCTGGT	60.035	TCCCTTTCCCTGACATTGCC	59.96	253
TRINITY_Contig7673	1	p5	(TCTTT)3	15	1170	1184	CTTTCACACTTTGGCAGGGC	59.967	GGGTGTGTCGGTGTATGTGT	59.966	118
TRINITY_Contig7674	1	p4	(TCGA)3	12	174	185	CTTCCTGCAGTTGAGCCTGA	59.964	TTCGACAACGCCTACTACGG	59.831	216
TRINITY_Contig7675	1	p3	(CGC)4	12	175	186	TTCCTATGCCGGAGTCTGGA	60.033	CGTGTACAGTGGACGGTTCA	59.969	155
TRINITY_Contig7677	1	p3	(GCT)4	12	665	676	GAGAAAATCAGCAAGCCGGC	60.179	TCCTCCCTCCGTCCTTTTCT	59.883	161
TRINITY_Contig7678	1	p6	(CGGCGA)3	18	244	261	TCCAGTCCTCGGTCTTGGAT	59.959	CAACTGCGAGCATCGAACAG	59.906	257
TRINITY_Contig7679	1	p3	(GTG)4	12	626	637	ACCGGACTTGTTCATCGCTT	59.965	AAGATGGACCACAGCACTGG	59.963	109
TRINITY_Contig7687	1	p4	(TCCA)3	12	271	282	GAAACGAGACACTGCCCTGA	59.966	TGGGGTTCTACATGCTGCTG	60.035	192
TRINITY_Contig7687	2	p3	(CGC)6	18	797	814	GAAACGAGACACTGCCCTGA	59.966	TGGGGTTCTACATGCTGCTG	60.035	192
TRINITY_Contig7687	3	p3	(AGC)4	12	1009	1020	GAAACGAGACACTGCCCTGA	59.966	TGGGGTTCTACATGCTGCTG	60.035	192

TRINITY_Contig7688	1	p3	(TGC)4	12	1204	1215	AGCACCGATCTAGATGCTGC	59.967	CCAAACCAGAGCCAAAACCG	59.968	218
TRINITY_Contig7693	1	p3	(CCA)5	15	274	288	CAGGCAAAAGCTGGGTTTCC	59.966	TGTGGACATGGTGGTGATGG	59.962	194
TRINITY_Contig7697	1	c	(GCGCG)3ccgccggttgcctccgcgc(CCG)4	46	319	364	TCCTTCTTCTTCGCAGCCAG	60.037	TGCACGAGAAGCAGAAACCC	60.885	202
TRINITY_Contig7700	1	p3	(GCC)4	12	301	312	AGCACCTCTCCTTCTTGCTT	58.937	CGGAAGAAAGGTTGCTCGGA	60.32	122
TRINITY_Contig7702	1	p3	(AAG)4	12	292	303	AGTTTATCACGCTGTGGGGG	60.035	ACCTCACTGTACTTGCCACG	59.966	202
TRINITY_Contig7704	1	p3	(CTG)8	24	401	424	TTCCTCGGCCCAGTCATCTA	60.033	GGCCTCCGATCCTGTTACTG	59.895	186
TRINITY_Contig7705	1	p3	(CAC)4	12	42	53	CCTGTATCACCACCACCACC	60.035	GTTCGGGATGATCTCCACCG	60.249	212
TRINITY_Contig7706	1	p3	(CCT)4	12	508	519	GGTTTTGCGCGAAAGGAAGT	59.97	GATCCCGAACGCTGCTGATA	59.968	236
TRINITY_Contig7707	1	p4	(TTTC)3	12	1901	1912	GCAGCTCTCCATGAACAGGT	60.036	CACCTGCGAGGAGAGTTGTT	59.966	234
TRINITY_Contig7713	1	p6	(GGCGGA)3	18	1863	1880	TGAGCGTGGGTTGCAGTTAT	59.964	GCTTACGCCACAGCTAATGC	59.972	115
TRINITY_Contig7713	2	p4	(CTTT)3	12	2068	2079	TGAGCGTGGGTTGCAGTTAT	59.964	GCTTACGCCACAGCTAATGC	59.972	115
TRINITY_Contig7713	3	p4	(CGGC)3	12	2181	2192	TGAGCGTGGGTTGCAGTTAT	59.964	GCTTACGCCACAGCTAATGC	59.972	115
TRINITY_Contig7714	1	p3	(GCC)4	12	198	209	GCAAGCTGGTTTGATGCTCC	60.109	GCCCAGCATCTCCTTCAACT	60.035	257
TRINITY_Contig7715	1	p3	(GCA)5	15	144	158	TGGAGATCTGGTCCAGGGAG	60.032	TCTCTGCCTCCTCTGCATCT	60.032	276
TRINITY_Contig7719	1	p4	(GGCT)3	12	1525	1536	CACCAAGTGATGTGCCGTTG	60.04	TCGAGCTCGCTAAAAGCCAA	60.038	110
TRINITY_Contig7720	1	c	(GGCGGG)3ggccatgtcgtcgaactccggcagcgtgcccagctccgggaaggtctcgtcgtcgatctccgactccggcgtgcgcgtctc(GGAGTG)3cgagagcgacgcctccgactccatgg(CCTTCT)3	161	885	1045	GGAAGGTCTCGTCGTCGATC	59.972	AATGCTGACGCTTTCGATGC	59.902	213
TRINITY_Contig7720	2	p4	(TGGC)4	16	1764	1779	GGAAGGTCTCGTCGTCGATC	59.972	AATGCTGACGCTTTCGATGC	59.902	213
TRINITY_Contig7721	1	p3	(CCT)4	12	1012	1023	AGTGGATGGCGAAGGTGAAG	60.036	CCTGCAGATTCCGCGAATTG	59.971	197
TRINITY_Contig7723	1	p3	(CGC)4	12	1778	1789	TTTGGTTTGCAGCATGACGG	59.968	GCTTGCCGAAGTGCAAAAGA	59.969	244
TRINITY_Contig7723	2	p3	(CCG)4	12	2911	2922	TTTGGTTTGCAGCATGACGG	59.968	GCTTGCCGAAGTGCAAAAGA	59.969	244
TRINITY_Contig7723	3	p3	(GGA)4	12	3061	3072	TTTGGTTTGCAGCATGACGG	59.968	GCTTGCCGAAGTGCAAAAGA	59.969	244
TRINITY_Contig7724	1	p3	(GCG)5	15	1267	1281	CGTGCTGGACATACAGGTGT	60.037	CACACACCACTACTGGCCAT	59.963	239
TRINITY_Contig7724	2	p5	(CGTGG)3	15	1589	1603	CGTGCTGGACATACAGGTGT	60.037	CACACACCACTACTGGCCAT	59.963	239
TRINITY_Contig7727	1	c	(CTGC)3tgcttcttgttgggtctgagctagctcctcttc(GT)11	67	4387	4453	CGATAGTGTCCTCGAAGCCC	59.969	CACACACACGAAGAGGAGCT	59.967	263
TRINITY_Contig7729	1	c	(GTC)4acgtccggcaagggcggcgtcggcaag(ACC)4	51	251	301	CCCGCATTCATCCTCATCGA	59.966	TCGCACTCGGTTCACAATGA	59.967	189
TRINITY_Contig7730	1	c	(TCC)4gctgggtggcagcacgcatcccgccccct(CCG)4	54	295	348	GATGCATTTCGTCGTGACCG	59.975	CTCCCCGTAAACAAGAGCGT	60.038	145
TRINITY_Contig7732	1	p4	(CCTC)4	16	123	138	AATCTGGGCTGTTTACCCCG	60.034	CTTTCCCAAACAACCCAGCG	59.968	129
TRINITY_Contig7732	2	p3	(CGC)5	15	338	352	AATCTGGGCTGTTTACCCCG	60.034	CTTTCCCAAACAACCCAGCG	59.968	129
TRINITY_Contig7733	1	p2	(CT)10	20	329	348	GCACGTATCGATCGGTGTCT	59.971	CAGCCGTTGAGATCGATCGA	59.971	227
TRINITY_Contig7734	1	p2	(TC)8	16	130	145	AAGGAGGAACTTGAGCAGGC	59.963	TCATACTCAGATTCGGCGGC	59.968	104
TRINITY_Contig7738	1	p3	(CTG)4	12	416	427	ACGGAAGCGGCAACTAGAAA	59.966	ATCAGTTCAACCAGCAGCGA	59.965	267
TRINITY_Contig7739	1	p4	(GATG)3	12	1598	1609	GCCCGACGTAGAATCTCAGG	59.969	AATGACAGCTCACGCCTCAA	59.965	139
TRINITY_Contig7740	1	p4	(GCTT)3	12	387	398	TCAGGTGTGGCAGTGTCATC	59.965	GAGTCACAGGTGGAGCTGAC	60.038	163
TRINITY_Contig7742	1	p3	(CCG)6	18	67	84	TGCCCACATGATGCCAGATT	60.033	TCTGGACCCGATACACTCGT	60.035	254
TRINITY_Contig7743	1	p3	(CGC)4	12	401	412	GAAAGCCTCCATCCCCCAAA	59.959	GTAGATCAGCCCGGAGATGC	60.038	221
TRINITY_Contig7744	1	p3	(CGG)4	12	348	359	GACGGGAGCAACTTCCTCAA	59.965	CCTCAATCGGCATGCATGTG	59.97	151
TRINITY_Contig7745	1	p3	(CGC)4	12	105	116	AAAGGTGGACAAGCCTACCG	59.964	CAAGTGTGTGCTGAGGTTGC	59.97	275
TRINITY_Contig7746	1	p3	(CGC)4	12	97	108	GCACACGGGTTTAGGGAGAA	59.964	TGGCAACCATTACGAGCCAT	60.035	263
TRINITY_Contig7748	1	p3	(CCG)4	12	418	429	GTGTTCAGTTGCCTGTGCTG	59.97	CTTCACGTCCAGTCGTCCAA	59.968	278
TRINITY_Contig7750	1	p3	(GCG)7	21	1608	1628	CATCTCTTCCTCGTCCTCGC	59.97	CGACGCCATTGATTGAGCAG	59.973	201
TRINITY_Contig7751	1	p3	(GGC)4	12	1072	1083	AAAACGTGCACCGCTTCTTC	59.971	AGCCTTATCGACGTCGGTTC	59.9	112
TRINITY_Contig7756	1	p3	(CAT)4	12	194	205	GAACCTCTGCCAGACCTTCC	60.036	TTGAGGTCAAGGAGCGAACC	59.965	114
TRINITY_Contig7757	1	p3	(AGC)4	12	52	63	TGGCATGCGATGGAAGAACT	60.035	TCGGTATTGGCAGTGGAACC	60.036	233
TRINITY_Contig7758	1	p3	(GCA)4	12	197	208	GGTCACTACAGCAGCATGGT	60.036	TTTTGGTATTTTGCGGCGCA	59.968	239
TRINITY_Contig7761	1	p3	(CGT)4	12	475	486	AGGCAGGGAAGGAGAGAGAG	60.032	CAGATAAAGCGGCGCTCAAC	59.973	169
TRINITY_Contig7761	2	p3	(GCC)5	15	743	757	AGGCAGGGAAGGAGAGAGAG	60.032	CAGATAAAGCGGCGCTCAAC	59.973	169
TRINITY_Contig7761	3	c	(CCG)5ctggatacgacgaccacggggtcctcgggccggtgcagcgcgctccaagacctcatcttctctct(GCC)8	104	1356	1459	AGGCAGGGAAGGAGAGAGAG	60.032	CAGATAAAGCGGCGCTCAAC	59.973	169
TRINITY_Contig7761	4	p3	(GCG)5	15	1590	1604	AGGCAGGGAAGGAGAGAGAG	60.032	CAGATAAAGCGGCGCTCAAC	59.973	169
TRINITY_Contig7761	5	p3	(CTG)4	12	1841	1852	AGGCAGGGAAGGAGAGAGAG	60.032	CAGATAAAGCGGCGCTCAAC	59.973	169
TRINITY_Contig7761	6	p2	(CT)13	26	2071	2096	AGGCAGGGAAGGAGAGAGAG	60.032	CAGATAAAGCGGCGCTCAAC	59.973	169
TRINITY_Contig7763	1	p3	(CGG)4	12	1180	1191	GCCCACCGATACCTCATCAG	59.966	TGCCATTGTCTCGCCTGAAT	60.035	276
TRINITY_Contig7764	1	p6	(GGCCAG)3	18	1002	1019	CGAAAGTCCAACCTGCTTGC	60.04	ACCACGAGATTTTGGGCCTT	59.888	103
TRINITY_Contig7764	2	p3	(CGG)5	15	1316	1330	CGAAAGTCCAACCTGCTTGC	60.04	ACCACGAGATTTTGGGCCTT	59.888	103
TRINITY_Contig7766	1	p4	(GGGC)3	12	1730	1741	ATCTCCTGCTTCACCTTGCC	60.035	CCCAGACCCAATTCCACCTC	60.034	243
TRINITY_Contig7766	2	p3	(CGC)5	15	1876	1890	ATCTCCTGCTTCACCTTGCC	60.035	CCCAGACCCAATTCCACCTC	60.034	243
TRINITY_Contig7767	1	p4	(TTCT)3	12	2590	2601	ACATGGTTTCCAAAGTGCGC	59.968	AACGTTGCAGACTACAGCGA	59.968	182
TRINITY_Contig7767	2	p3	(CTT)4	12	3236	3247	ACATGGTTTCCAAAGTGCGC	59.968	AACGTTGCAGACTACAGCGA	59.968	182
TRINITY_Contig7768	1	p4	(AGCA)3	12	370	381	TGGCCGAGAACATCTCCCTA	60.033	GTTCTTCCACTCCAGGACCG	60.037	237
TRINITY_Contig7768	2	p2	(AG)26	52	885	936	TGGCCGAGAACATCTCCCTA	60.033	GTTCTTCCACTCCAGGACCG	60.037	237
TRINITY_Contig7772	1	p3	(TGG)4	12	1394	1405	GCCCTCTTCCTTGCCTCATT	60.034	GAAAGTGGAGGGATGGTGGG	60.034	234
TRINITY_Contig7772	2	p3	(GCG)5	15	1511	1525	GCCCTCTTCCTTGCCTCATT	60.034	GAAAGTGGAGGGATGGTGGG	60.034	234
TRINITY_Contig7775	1	p2	(CT)12	24	1269	1292	CCACCTAGAGCTTAGTCGCG	59.97	AGGAGACCGAGAAGTACGCT	60.035	154
TRINITY_Contig7779	1	p3	(CGC)4	12	219	230	AACAGTGGGTAGAAGCACGG	59.965	TTCCTTCAGTTCCTGGCGTC	59.965	131
TRINITY_Contig7779	2	p3	(CAG)7	21	337	357	AACAGTGGGTAGAAGCACGG	59.965	TTCCTTCAGTTCCTGGCGTC	59.965	131
TRINITY_Contig7782	1	c	(GCA)4ctccccagcaccaggagggagacgatcgagagactcggcggcttgggttgggagtcagccggtgtggat(AG)17gctgtgatcacaggagaggagagcagctctctctcag(GACC)3	164	741	904	TCAGCCGGTGTGGATAGAGA	60.034	CTCCTCCTTGAGAGAGGCCT	60.032	192
TRINITY_Contig7783	1	p4	(TGGT)3	12	597	608	TTGGGCTACCTCTCGCTACT	60.033	AGCTGTCAAGACCACACGAG	59.967	146
TRINITY_Contig7784	1	p4	(TCAA)3	12	115	126	TTAGCCAGGAACACTGCAGG	59.963	CCCAGCCCCTCAACTTTGAT	59.96	162
TRINITY_Contig7784	2	p3	(GCG)4	12	2836	2847	TTAGCCAGGAACACTGCAGG	59.963	CCCAGCCCCTCAACTTTGAT	59.96	162
TRINITY_Contig7786	1	p5	(CCTTC)4	20	88	107	GGCGGTTGCTGTTGTTACTG	60.04	CCGTAGATACCGAGCACACC	59.97	194
TRINITY_Contig7786	2	p3	(CGC)4	12	237	248	GGCGGTTGCTGTTGTTACTG	60.04	CCGTAGATACCGAGCACACC	59.97	194
TRINITY_Contig7786	3	p3	(CTG)4	12	536	547	GGCGGTTGCTGTTGTTACTG	60.04	CCGTAGATACCGAGCACACC	59.97	194
TRINITY_Contig7790	1	p5	(CAGTA)3	15	1840	1854	AGGGTCCTCCAGCATCATCT	60.03	GAGGCTGTTGAAGGATGGCT	60.035	133
TRINITY_Contig7790	2	c	(GCGA)3gagatctgggcgcggacgctaccgaggt(AAGG)3gggtaaggaggagcaggcg(AGC)4	83	3225	3307	AGGGTCCTCCAGCATCATCT	60.03	GAGGCTGTTGAAGGATGGCT	60.035	133
TRINITY_Contig7791	1	p2	(CT)6	12	4	15	CCTCAAGGTCTTGCTGGGAG	60.036	ATGAGCAATGCAGCCTTCCT	60.033	242
TRINITY_Contig7791	2	p4	(AGGC)3	12	177	188	CCTCAAGGTCTTGCTGGGAG	60.036	ATGAGCAATGCAGCCTTCCT	60.033	242
TRINITY_Contig7791	3	p3	(CCT)4	12	307	318	CCTCAAGGTCTTGCTGGGAG	60.036	ATGAGCAATGCAGCCTTCCT	60.033	242
TRINITY_Contig7791	4	p3	(GGC)5	15	3259	3273	CCTCAAGGTCTTGCTGGGAG	60.036	ATGAGCAATGCAGCCTTCCT	60.033	242
TRINITY_Contig7791	5	p4	(GTTT)3	12	3587	3598	CCTCAAGGTCTTGCTGGGAG	60.036	ATGAGCAATGCAGCCTTCCT	60.033	242
TRINITY_Contig7792	1	c	(GA)9ggcggaggagagagaaacccctagtctccagc(CGCCT)4	70	40	109	GCTCTTCCACAAAACAGCGG	60.04	GTATCAGCAAAGCGGCTTCG	59.973	215
TRINITY_Contig7792	2	p3	(AGC)4	12	2496	2507	GCTCTTCCACAAAACAGCGG	60.04	GTATCAGCAAAGCGGCTTCG	59.973	215
TRINITY_Contig7795	1	p3	(GGA)5	15	1438	1452	CAAAGAACTCCGGTCGGACA	59.967	GAAGACGTCAAGCCCCATCA	60.036	233
TRINITY_Contig7796	1	p4	(CGGG)3	12	83	94	CAGCCTCAGCATCTACTCCG	59.968	GCACCGAGATAGCTGAGGAC	59.969	147
TRINITY_Contig7796	2	p3	(CGG)5	15	257	271	CAGCCTCAGCATCTACTCCG	59.968	GCACCGAGATAGCTGAGGAC	59.969	147
TRINITY_Contig7799	1	c	(CAG)4gcagagaagag(GGA)4	35	1496	1530	GAGGTACCTGAGGCCTCTGA	60.033	GCGTGACCACATCAAGGGTA	60.037	196
TRINITY_Contig7800	1	p3	(CGG)5	15	392	406	TCAGGTGCTGTGCTTGGTAG	59.965	GCAGTCACGAATTCGCGTAC	59.977	236
TRINITY_Contig7800	2	p3	(GCG)6	18	961	978	TCAGGTGCTGTGCTTGGTAG	59.965	GCAGTCACGAATTCGCGTAC	59.977	236
TRINITY_Contig7801	1	p3	(CCG)4	12	211	222	TCAACTTGAGACGGTTCGGG	59.967	GTGGCTTCCTCTGCATCCTT	60.035	249
TRINITY_Contig7803	1	p3	(CGC)4	12	838	849	GGGTTCGTGATCGAGGTGTT	60.038	CTAGACGGTCGTAGCGCATT	59.971	100
TRINITY_Contig7803	2	p3	(GTG)4	12	2045	2056	GGGTTCGTGATCGAGGTGTT	60.038	CTAGACGGTCGTAGCGCATT	59.971	100
TRINITY_Contig7805	1	p4	(CGGC)3	12	141	152	GCAGCTGTACAACTCCCTGT	59.965	GCGGATCTCCTCCTTGTAGC	59.967	250
TRINITY_Contig7806	1	p3	(GCG)4	12	163	174	CCTAATCGACTCCCACGACG	59.971	GGGAGGATGAGGTGGTGTTG	60.035	239
TRINITY_Contig7809	1	p3	(CAT)5	15	362	376	GAATCTCTCACCACGCCCAA	60.036	CCAGCCACAAGGGTCATCAT	60.034	253
TRINITY_Contig7809	2	p4	(ATTC)3	12	811	822	GAATCTCTCACCACGCCCAA	60.036	CCAGCCACAAGGGTCATCAT	60.034	253
TRINITY_Contig7812	1	c	(GCC)4gctgccggcgggg(CTCC)3ggtcccgtcccctcgcggcaaccctatccctacccgcctt(CGC)9	104	105	208	ATTGACCGTTGAGAGGCAGG	60.036	TTTGCAGACTCTCGTGGACC	59.966	197
TRINITY_Contig7816	1	p4	(CATA)3	12	503	514	AACCCGCCGTTCACTAAGAG	60.038	CCAGAACTGCCAAGGAGAGG	60.036	159
TRINITY_Contig7818	1	p4	(CCCA)3	12	232	243	GTTTGTTGCTCCCAGTGCAG	59.969	TGGGAGGATCTTCCCGCTAA	60.032	181

TRINITY_Contig7819	1	p3	(GAG)4	12	2903	2914	TTATCCTGCTCCTGACCCGA	60.033	TAGGCGTCCCTGGATGTACA	60.033	241
TRINITY_Contig7821	1	p5	(TCAGT)3	15	60	74	GCCCTCTGAGTTTCAGCCAT	60.035	CAAGCTCTTCCAACGCACAC	60.041	230
TRINITY_Contig7821	2	p3	(GAA)4	12	411	422	GCCCTCTGAGTTTCAGCCAT	60.035	CAAGCTCTTCCAACGCACAC	60.041	230
TRINITY_Contig7822	1	p2	(GC)6	12	868	879	GGTGTCTGACCAGTCTGAGC	60.038	AATCCTCGCTCCGATTCGTC	59.969	254
TRINITY_Contig7824	1	c	(CGACCT)4ccgccggcgtg(GCC)5	50	1391	1440	GACATCCCGACGACCTCATC	59.969	TGGATCGGGAGTTCTTTGGC	60.035	140
TRINITY_Contig7824	2	p3	(GCC)5	15	1590	1604	GACATCCCGACGACCTCATC	59.969	TGGATCGGGAGTTCTTTGGC	60.035	140
TRINITY_Contig7825	1	p4	(ACCA)3	12	151	162	CGGCGGATTATTGGATCCCA	59.964	TACTCCTCCGCTCACTCACA	59.962	199
TRINITY_Contig7825	2	p3	(CCT)4	12	905	916	CGGCGGATTATTGGATCCCA	59.964	TACTCCTCCGCTCACTCACA	59.962	199
TRINITY_Contig7825	3	c	(GAC)4ggcgccgcg(TGC)6	39	1162	1200	CGGCGGATTATTGGATCCCA	59.964	TACTCCTCCGCTCACTCACA	59.962	199
TRINITY_Contig7825	4	p3	(GCT)4	12	1403	1414	CGGCGGATTATTGGATCCCA	59.964	TACTCCTCCGCTCACTCACA	59.962	199
TRINITY_Contig7825	5	c	(GCAAG)3ttagcttcgcggcaaaggc(AG)8	50	1736	1785	CGGCGGATTATTGGATCCCA	59.964	TACTCCTCCGCTCACTCACA	59.962	199
TRINITY_Contig7825	6	p4	(GAAA)3	12	2164	2175	CGGCGGATTATTGGATCCCA	59.964	TACTCCTCCGCTCACTCACA	59.962	199
TRINITY_Contig7828	1	p2	(AG)8	16	67	82	GGTGGGTGATTCTTGCCGTA	60.036	GCGCCCACAGATTTCAGTTG	60.11	251
TRINITY_Contig7828	2	p3	(CCA)4	12	582	593	GGTGGGTGATTCTTGCCGTA	60.036	GCGCCCACAGATTTCAGTTG	60.11	251
TRINITY_Contig7828	3	c	(CCT)4cgtcgtcgtcct(CCG)4ccaaccggggcc(GCG)5	63	980	1042	GGTGGGTGATTCTTGCCGTA	60.036	GCGCCCACAGATTTCAGTTG	60.11	251
TRINITY_Contig7829	1	p6	(CCCCGG)3	18	385	402	GCACGAAGATACTACGGCGA	59.971	GATGGGGTGAGTTGAGGTGG	60.035	173
TRINITY_Contig7830	1	p3	(CCG)4	12	133	144	ACAGTGTTCCCTTTAGCCCG	59.964	GCAAGCCTTTTATCTCGCCG	59.972	265
TRINITY_Contig7832	1	c	(TCCA)3tcattccgccacaagccccctcctcgcttcgtcgtccccgccgctcctggaccggcggg(CACC)3	83	178	260	AGCTGACGGGACATCGTTTT	59.965	GGAATCACGGGCACAACAAC	60.04	192
TRINITY_Contig7838	1	c	(CAG)5ccatcatcgtcatgctcaatcctcccaatgctgcga(GCT)4	63	311	373	TTTGACTTGCAAGACGCTGC	59.971	ACATAGGGTTGGTGCCATGG	60.033	171
TRINITY_Contig7838	2	p3	(CGC)4	12	1249	1260	TTTGACTTGCAAGACGCTGC	59.971	ACATAGGGTTGGTGCCATGG	60.033	171
TRINITY_Contig7838	3	c	(AGGC)3(AATC)3	24	2099	2122	TTTGACTTGCAAGACGCTGC	59.971	ACATAGGGTTGGTGCCATGG	60.033	171
TRINITY_Contig7838	4	c	(TCC)6tcgcatcgatcaccacctcgag(CTC)4	52	2231	2282	TTTGACTTGCAAGACGCTGC	59.971	ACATAGGGTTGGTGCCATGG	60.033	171
TRINITY_Contig7841	1	c	(TCG)4tacgggagcacgaggaggtcgtccacgacgaagtggtccccgcaccccgcgggcttcctctcgcgcggggccg(CGC)4cgtagtacccgtactcggg(CGC)4	128	1408	1535	TTGGACATCACGAAGGTGGG	59.964	GTCGAAGCCGTCCAAGAAGA	60.039	264
TRINITY_Contig7841	2	p2	(AG)19	38	1677	1714	TTGGACATCACGAAGGTGGG	59.964	GTCGAAGCCGTCCAAGAAGA	60.039	264
TRINITY_Contig7844	1	p3	(CAG)4	12	1317	1328	CAAGCCTGCACCAGTCTACA	59.965	TTCCTGGTGTCGTTAGTGGC	59.966	271
TRINITY_Contig7845	1	p3	(CCG)4	12	936	947	CACGATTGCCACCAAATGCA	60.038	TTGGCACCAAGAGGAATGCT	59.887	226
TRINITY_Contig7845	2	c	(GGC)4gtgcgggactcgtcggtggagacggcgtcctgatgagaaggcggcgtcgcgcggcgcggaaggggcgagggcgggggcggaggcc(GCG)5	112	1068	1179	CACGATTGCCACCAAATGCA	60.038	TTGGCACCAAGAGGAATGCT	59.887	226
TRINITY_Contig7850	1	p6	(CTCCCG)3	18	2498	2515	TTTTCTGGCACAAGCATGGC	59.966	ACAGCGAAACAGAGGAGGTG	59.966	275
TRINITY_Contig7851	1	p3	(CCG)4	12	1098	1109	AAGAGAAGCCGACGTCGATC	59.901	CCGACGACGTGAGCTACTAC	59.974	216
TRINITY_Contig7851	2	p3	(CGC)4	12	1708	1719	AAGAGAAGCCGACGTCGATC	59.901	CCGACGACGTGAGCTACTAC	59.974	216
TRINITY_Contig7852	1	p4	(CAAG)3	12	272	283	ACAGCAAGCTCTACCGACAC	60.038	TGCAATCGCCTCTGTGAACT	59.965	144
TRINITY_Contig7854	1	p4	(CTCG)3	12	81	92	GAGCTTCAAACCGTGGAGGA	59.965	TGCCGAGCAGCTTGAGTAAA	59.965	242
TRINITY_Contig7854	2	p4	(GTCA)3	12	1163	1174	GAGCTTCAAACCGTGGAGGA	59.965	TGCCGAGCAGCTTGAGTAAA	59.965	242
TRINITY_Contig7854	3	p4	(GATT)4	16	2277	2292	GAGCTTCAAACCGTGGAGGA	59.965	TGCCGAGCAGCTTGAGTAAA	59.965	242
TRINITY_Contig7854	4	p3	(TTG)5	15	2497	2511	GAGCTTCAAACCGTGGAGGA	59.965	TGCCGAGCAGCTTGAGTAAA	59.965	242
TRINITY_Contig7855	1	p3	(GGC)5	15	1779	1793	CACCGACACCTTTGCCTAGT	59.965	CTGATGAAGCGCGACAATGG	59.973	237
TRINITY_Contig7857	1	c	(CCCCG)3gccggccgcccccatcccatcatccatctctgaggaaggatcaccacctcccctccgttcccc(TCGCG)3	93	158	250	CAAAAATGGTGGCCAAGGGG	59.963	TTTTTCCATTGCCAGGCACG	59.967	151
TRINITY_Contig7857	2	c	(CCGC)3tttcccacccctcgaagccgaatccgatggttccacccgttgaccgcctccgccatcggaggccgcgccg(CCT)4cggcccgcgcgccccgccctatagataggcacccgccgcccactccccgatggcggtcgccgg(CGA)4	170	368	537	CAAAAATGGTGGCCAAGGGG	59.963	TTTTTCCATTGCCAGGCACG	59.967	151
TRINITY_Contig7858	1	p3	(GGC)6	18	171	188	ATCTTGCACGCACCCTTGTA	59.964	CCTCGGACATCAAGTGCAGT	60.037	234
TRINITY_Contig7859	1	p4	(CGCA)3	12	138	149	AGGTTTGGTTGCCAAGGACA	60.033	GGGCCTTCGGAGGTAATAGC	59.965	109
TRINITY_Contig7860	1	p2	(TC)15	30	1725	1754	CCCGGTAAACCACATCAGCT	60.035	CTCCGTGCTTGTTGTGGTG	59.971	206
TRINITY_Contig7861	1	p3	(GGC)4	12	130	141	CTCCGTACACTCCGCATCTC	59.97	TGGAGAAGATGACGACGTCG	59.553	190
TRINITY_Contig7864	1	p2	(CG)6	12	261	272	GACCGTGAAGAGCACTGACA	59.968	CTTGACGAGTTGCTCCACCT	59.966	243
TRINITY_Contig7864	2	c	(CAC)4cggatgctgtcggcgtcgggccggctgaacggcatgctctccgacaccctccgcggctacggcggcggcatgggcggcctgggcggcatcggcggg(CAC)7	129	905	1033	GACCGTGAAGAGCACTGACA	59.968	CTTGACGAGTTGCTCCACCT	59.966	243
TRINITY_Contig7864	3	p3	(GGC)5	15	1439	1453	GACCGTGAAGAGCACTGACA	59.968	CTTGACGAGTTGCTCCACCT	59.966	243
TRINITY_Contig7864	4	p3	(AGC)4	12	1854	1865	GACCGTGAAGAGCACTGACA	59.968	CTTGACGAGTTGCTCCACCT	59.966	243
TRINITY_Contig7864	5	p3	(TGG)4	12	2173	2184	GACCGTGAAGAGCACTGACA	59.968	CTTGACGAGTTGCTCCACCT	59.966	243
TRINITY_Contig7865	1	p4	(CGCC)3	12	43	54	TGGTTGTATTGGACACCGGG	59.963	TGCAAGGAAGATGCCGACTT	59.963	150
TRINITY_Contig7866	1	c	(CCG)4tgcgcgcgaggctgctggccgccgccggcatgcgcgcgcgcctcctg(GCC)4gtgctcgcggacctgctggccgccgttggcaagcgcgcggtcctcctga(CCG)4	132	33	164	GGTCTCAACCTCAACCTCCG	60.037	CCTTCTCCTTCTCCACGCAG	60.108	265
TRINITY_Contig7866	2	p3	(TGG)5	15	627	641	GGTCTCAACCTCAACCTCCG	60.037	CCTTCTCCTTCTCCACGCAG	60.108	265
TRINITY_Contig7869	1	c	(GCG)4ggactcgcgcgggccat(GGC)4	41	91	131	CTTTCTCCTCTGCTCTGGCC	60.108	GTGGAGACGTGGAGTGTGAG	60.039	152
TRINITY_Contig7869	2	p3	(CCG)5	15	245	259	CTTTCTCCTCTGCTCTGGCC	60.108	GTGGAGACGTGGAGTGTGAG	60.039	152
TRINITY_Contig7871	1	p4	(AATG)22	88	473	560	GAAAGGGGGAATCAGGCCTC	60.106	AGGACTCAGAGATCCAGCGT	60.034	214
TRINITY_Contig7871	2	p3	(CTC)4	12	2262	2273	GAAAGGGGGAATCAGGCCTC	60.106	AGGACTCAGAGATCCAGCGT	60.034	214
TRINITY_Contig7874	1	p3	(CGA)4	12	134	145	CCTGGTACGGCATCGACTAC	59.97	CGTCGGTCATGTACAGGGAG	59.899	135
TRINITY_Contig7880	1	p3	(GCC)5	15	1784	1798	GTCAAACAGGAGCAGACGGA	59.966	ATGGGAGATGGGAACGAGGA	60.031	177
TRINITY_Contig7882	1	p3	(CTG)4	12	734	745	AGAAGGAGGAGGACGGGATC	60.106	CGATCTCACCCCCACAACAA	59.964	212
TRINITY_Contig7885	1	p5	(GCCCC)3	15	64	78	GATCTCTGGCTAGCTGGCTC	59.682	TTCTGCTCACAAGGTCTGGC	60.25	184
TRINITY_Contig7886	1	p3	(CCG)4	12	310	321	CGAGTGGAAGGAGGAGGAGA	60.034	TTGTCACTGGGGCCTTCATC	59.962	180
TRINITY_Contig7887	1	p3	(GCT)4	12	1517	1528	TAGAGGTTGGCGGCAATGTT	59.962	GATGTCGGCTATCGCTCACA	59.969	128
TRINITY_Contig7888	1	p3	(TCG)4	12	452	463	CCTTCGGACAAGAGCAAGGT	59.965	TTCTGCTTCTTTTGCGTGGC	59.969	216
TRINITY_Contig7888	2	c	(CGC)4(CAC)4	24	1154	1177	CCTTCGGACAAGAGCAAGGT	59.965	TTCTGCTTCTTTTGCGTGGC	59.969	216
TRINITY_Contig7888	3	p3	(CCG)6	18	1336	1353	CCTTCGGACAAGAGCAAGGT	59.965	TTCTGCTTCTTTTGCGTGGC	59.969	216
TRINITY_Contig7890	1	p2	(AG)19	38	1399	1436	GTAGGTCTCAGCTTGCTGCA	60.037	GAGGAGATGGCTAAGGACGC	59.967	155
TRINITY_Contig7890	2	p2	(AG)11	22	1740	1761	GTAGGTCTCAGCTTGCTGCA	60.037	GAGGAGATGGCTAAGGACGC	59.967	155
TRINITY_Contig7891	1	p5	(CGCGC)3	15	211	225	TAAGTCAGGACGGGAGGGAG	60.033	CGCCACAATGTCACAGCAAA	59.969	249
TRINITY_Contig7893	1	p3	(AGC)4	12	367	378	CTACACCGACTATTCGCCCC	59.968	GTCTGGTCATGGAAGCCGAA	60.036	249
TRINITY_Contig7896	1	p4	(CTGC)3	12	1241	1252	ATGCGCTCAAATGGTCCAGA	60.035	CAATCCCGTCTGAGCTTGGT	60.036	247
TRINITY_Contig7897	1	p4	(CTCC)3	12	84	95	TGCTTTCGTGTCGCTTGTTG	59.973	GATGGATATGCCCTCCCAGC	60.035	121
TRINITY_Contig7898	1	p2	(TC)12	24	2326	2349	TACAATTTCAGCCCCGACCC	60.035	TGTCTGGTGGTGTTGCTGTT	60.035	221
TRINITY_Contig7899	1	p3	(GCC)4	12	111	122	TGAGGCTGCTGAAGTTCTCG	60.038	CTTCACCCCTCCACTCCAAC	59.963	235
TRINITY_Contig7899	2	p3	(CCG)4	12	301	312	TGAGGCTGCTGAAGTTCTCG	60.038	CTTCACCCCTCCACTCCAAC	59.963	235
TRINITY_Contig7899	3	c	(GCG)4gaggcgggcgcgggacgcggcgctggcccagcacgacgaggaggagggcggcgaggtgctggccggcgcggtggcggagggggtgaacgacgg(GGA)6	123	444	566	TGAGGCTGCTGAAGTTCTCG	60.038	CTTCACCCCTCCACTCCAAC	59.963	235
TRINITY_Contig7899	4	p3	(CGC)5	15	846	860	TGAGGCTGCTGAAGTTCTCG	60.038	CTTCACCCCTCCACTCCAAC	59.963	235
TRINITY_Contig7899	5	p3	(TTG)4	12	1524	1535	TGAGGCTGCTGAAGTTCTCG	60.038	CTTCACCCCTCCACTCCAAC	59.963	235
TRINITY_Contig7901	1	p4	(CTTT)3	12	1069	1080	GACGGGATCGATGGCAGATT	59.966	TCCCTCATCAGCTCAGTGGA	59.958	180
TRINITY_Contig7902	1	p3	(GCC)5	15	30	44	TTGGGGACGTATTGCTTGCT	59.962	GCGGATCTGTCGAAGGGTAG	59.969	126
TRINITY_Contig7910	1	p3	(CAC)4	12	777	788	CTTGCTGCAGGTCGTGTAGA	60.038	TGATGCTCACAGCTGAAGCA	59.964	121
TRINITY_Contig7911	1	p3	(GCG)4	12	1370	1381	GAGGAGGTAGCAGCGATCAC	59.969	CGGTGTTTTTCTCCCCTCGA	59.966	231
TRINITY_Contig7912	1	p3	(CGG)5	15	227	241	GTCGCTGTCGCAACAAAGTT	59.972	TCTTCTTCAGACGCGCATGT	60.038	259
TRINITY_Contig7913	1	p3	(CGC)4	12	76	87	GCTGTACGGGAGAATCGAGG	59.969	CAACACCTTGCGTTGAGAGC	60.041	239
TRINITY_Contig7913	2	p4	(GACC)3	12	1575	1586	GCTGTACGGGAGAATCGAGG	59.969	CAACACCTTGCGTTGAGAGC	60.041	239
TRINITY_Contig7915	1	p3	(CCG)6	18	1339	1356	GTTGTAGTCATTGCCCCCGA	60.036	TGGAGATGAAGTGGTTCCGC	60.036	122
TRINITY_Contig7921	1	p3	(GGC)4	12	1120	1131	AGTGGAAGAAGCGACGGAAG	60.038	GAGGGGGTGGAATTTGGAGG	60.033	145
TRINITY_Contig7921	2	p3	(CGG)5	15	1410	1424	AGTGGAAGAAGCGACGGAAG	60.038	GAGGGGGTGGAATTTGGAGG	60.033	145
TRINITY_Contig7921	3	c	(CCT)5ccgagtcgt(CGG)5	39	1541	1579	AGTGGAAGAAGCGACGGAAG	60.038	GAGGGGGTGGAATTTGGAGG	60.033	145
TRINITY_Contig7921	4	p3	(CCT)4	12	1806	1817	AGTGGAAGAAGCGACGGAAG	60.038	GAGGGGGTGGAATTTGGAGG	60.033	145
TRINITY_Contig7921	5	p6	(GGAAGG)3	18	2029	2046	AGTGGAAGAAGCGACGGAAG	60.038	GAGGGGGTGGAATTTGGAGG	60.033	145

TRINITY_Contig7924	1	p3	(CTC)4	12	698	709	GGACGTGTTGTGCAAGCATT	59.969	CGCTGAGCTTCGACTCGTAT	59.972	154
TRINITY_Contig7930	1	p3	(CAG)5	15	1531	1545	AGCGGCAGATCATGGAGATG	59.965	TCCTTCTTCACCTGCTGCTG	59.964	163
TRINITY_Contig7932	1	p3	(GCG)4	12	427	438	ACATCATCAGCCGGCCATAG	59.964	TTGGTACTGGGATGCAGCTG	60.035	151
TRINITY_Contig7935	1	p2	(AC)6	12	234	245	GGCAATCTCCTCAGGCTTGT	60.035	CACCTCATGTGCGAGATCGA	59.899	117
TRINITY_Contig7938	1	p4	(AATA)3	12	12	23	ACTCTTGGCAAGGACTTCCG	59.965	ATGTTCCGGTGGACCATTCC	60.034	191
TRINITY_Contig7940	1	p3	(GCG)5	15	417	431	TACACCTGAACGCCACCATC	60.037	CTCCTTTTCCCCGTGCTGAT	60.035	211
TRINITY_Contig7940	2	p4	(AACA)3	12	903	914	TACACCTGAACGCCACCATC	60.037	CTCCTTTTCCCCGTGCTGAT	60.035	211
TRINITY_Contig7942	1	p4	(GCCG)3	12	1793	1804	GCATCAGCAGATCTCTCCCC	59.965	TAGTGGCTGGCTTCCATGTG	60.035	153
TRINITY_Contig7945	1	p3	(CCG)4	12	112	123	TCAGTGTCAACGGTGAGAGC	59.968	GCTGTTGTGGGAGTACACGA	59.967	120
TRINITY_Contig7947	1	p3	(ACC)4	12	745	756	CCTCCTGGCTCCTACTCCTT	60.032	TGATTGAGAGGGAGGGAGGG	60.031	102
TRINITY_Contig7947	2	c	(CTCC)3tggctcctactccttatccaaacaaacacaagcaaat(CAGAGG)3accagctgctgcttcttgcttg(CCCT)3	101	1870	1970	CCTCCTGGCTCCTACTCCTT	60.032	TGATTGAGAGGGAGGGAGGG	60.031	102
TRINITY_Contig7948	1	p3	(GCT)4	12	907	918	GTCTAGCTCGGTTCTGCCTC	59.898	CTCCACGTCCTCCACAAGAC	60.038	125
TRINITY_Contig7948	2	p3	(GGA)5	15	1047	1061	GTCTAGCTCGGTTCTGCCTC	59.898	CTCCACGTCCTCCACAAGAC	60.038	125
TRINITY_Contig7951	1	p3	(CTC)5	15	23	37	TCCAGTCCAGTGGCATTCAC	59.963	CCTGTAGAATGCTGCCCCAA	60.034	234
TRINITY_Contig7951	2	p4	(TTTA)4	16	1376	1391	TCCAGTCCAGTGGCATTCAC	59.963	CCTGTAGAATGCTGCCCCAA	60.034	234
TRINITY_Contig7952	1	p4	(AACA)3	12	154	165	GTTGGGCGTTGGGAGGATTA	60.035	ACTGCCAAGTTCTCTGACCG	59.966	187
TRINITY_Contig7953	1	c	(GATC)3gagcaccgtcgccgcgcgctcgacagcacgaaagcttggtgccgtgccgtgtcggtggcgg(CGGC)3	85	102	186	TGGGATTTGGATTGGGTCGG	60.034	TGCTGGAATGAACCACCTCC	59.962	134
TRINITY_Contig7954	1	p3	(GCT)4	12	384	395	CTAGAAGAGCCCGCCACAAA	60.036	GCAAACTAGCACTGCGATCG	59.974	158
TRINITY_Contig7956	1	c	(TCGCA)3ttgcgacgc(CCG)4	36	391	426	TTGGTTGGTTGCTTCCCTGT	60.033	ATCCAGGTTTACTGCGGCAA	59.962	161
TRINITY_Contig7957	1	p3	(GGC)5	15	2952	2966	CATAAGACCTCGCTCGGACC	59.969	AAGGTTATGAAGGCCCGCAA	59.961	228
TRINITY_Contig7957	2	c	(TCGC)3gtgggcggaggt(AGG)4	36	3127	3162	CATAAGACCTCGCTCGGACC	59.969	AAGGTTATGAAGGCCCGCAA	59.961	228
TRINITY_Contig7960	1	p3	(CCG)6	18	414	431	GCATTTGCATCACTTGGGCA	60.037	AGCATCAGCAGCTCACAGTT	59.964	162
TRINITY_Contig7961	1	p3	(GCA)5	15	165	179	GTCTGATGAGTAGTCGCCGG	59.97	ACAACCGTGGAGCATGTCAT	59.963	168
TRINITY_Contig7961	2	p6	(GCCGCT)3	18	2470	2487	GTCTGATGAGTAGTCGCCGG	59.97	ACAACCGTGGAGCATGTCAT	59.963	168
TRINITY_Contig7963	1	p3	(CAC)6	18	226	243	CCGAGGAGGTTGCTCCAAAT	60.035	CTCCCCTACTCCCATGGTCA	60.031	140
TRINITY_Contig7963	2	p5	(AGAAA)3	15	2590	2604	CCGAGGAGGTTGCTCCAAAT	60.035	CTCCCCTACTCCCATGGTCA	60.031	140
TRINITY_Contig7967	1	p3	(TCC)4	12	46	57	AAAGAGGGGATTGGACAGCG	60.035	TGTGTTCTCCGTTGGCCTAC	59.966	177
TRINITY_Contig7967	2	p3	(CGG)4	12	260	271	AAAGAGGGGATTGGACAGCG	60.035	TGTGTTCTCCGTTGGCCTAC	59.966	177
TRINITY_Contig7968	1	p3	(GCC)5	15	186	200	CCAGACCTCAGCTTCTTGCA	59.964	TGGTACTTCTTCTGCCTGCG	60.037	276
TRINITY_Contig7968	2	p3	(GGC)5	15	465	479	CCAGACCTCAGCTTCTTGCA	59.964	TGGTACTTCTTCTGCCTGCG	60.037	276
TRINITY_Contig7968	3	p3	(GCC)5	15	1503	1517	CCAGACCTCAGCTTCTTGCA	59.964	TGGTACTTCTTCTGCCTGCG	60.037	276
TRINITY_Contig7968	4	p3	(CGG)4	12	1781	1792	CCAGACCTCAGCTTCTTGCA	59.964	TGGTACTTCTTCTGCCTGCG	60.037	276
TRINITY_Contig7970	1	p3	(CGC)4	12	224	235	CGATGTTGATGCTGCATGGG	59.97	CCCAACCTCACCATTCCCTC	60.034	153
TRINITY_Contig7972	1	p4	(CTCC)3	12	176	187	GCTTGGCTGCTTGGTGAAAA	59.895	ACTGAAGGCTCTGGAATGGC	60.035	205
TRINITY_Contig7973	1	p3	(CAC)4	12	1437	1448	GCCGTATTGCTGCTCGAAAG	59.973	TTCTATGGGACCTGCCTCGA	60.033	112
TRINITY_Contig7975	1	p3	(GCG)5	15	431	445	GGCACCAAAACACTTGCACA	60.108	CTTGCACCACGTCTTAGGGT	59.965	121
TRINITY_Contig7978	1	p3	(CGA)4	12	797	808	TGTTGCCACCTGGTGAAGTT	60.034	AGATTAGCGATGGCGAGGTG	59.967	244
TRINITY_Contig7978	2	p3	(CGT)4	12	2015	2026	TGTTGCCACCTGGTGAAGTT	60.034	AGATTAGCGATGGCGAGGTG	59.967	244
TRINITY_Contig7979	1	p5	(TTCAT)3	15	21	35	GGCTTAGGAGACCATCAGGC	59.892	TGACCGGACACTCTGTTTCG	59.968	155
TRINITY_Contig7980	1	p3	(GGC)4	12	496	507	CCGAGGTTCCGATGGACAAA	60.037	GCTTACTTTCCGCTCCCAGT	60.036	203
TRINITY_Contig7980	2	p3	(CCG)5	15	770	784	CCGAGGTTCCGATGGACAAA	60.037	GCTTACTTTCCGCTCCCAGT	60.036	203
TRINITY_Contig7982	1	p3	(GCG)4	12	1376	1387	GCCATCAGGTCCCATTCCAA	60.033	GTTTCTCTCCAAGGTGCCGA	59.965	190
TRINITY_Contig7983	1	p3	(CCG)4	12	56	67	TCTACATCGCCGACATCAGC	59.969	TCTCGATGCTCCTCTGGGAA	60.033	265
TRINITY_Contig7983	2	p3	(GGC)6	18	369	386	TCTACATCGCCGACATCAGC	59.969	TCTCGATGCTCCTCTGGGAA	60.033	265
TRINITY_Contig7988	1	p2	(CT)6	12	1087	1098	TACAGTGTCGCGTATACGGC	59.972	TCAAATGTGAGCCCCTTCCC	59.96	224
TRINITY_Contig7990	1	c	(AC)6cccaagcaaatcgaggaaagtgcaaagcgcagcgtcccaatcagctgacgtggtgattactgttagcagcaaatccatggaggccgtgctgatgcagcg(GGC)5	126	147	272	AGTTCACGTACCAGCGGATG	60.109	GACTGCTGTTCCGTAGTGCT	60.038	257
TRINITY_Contig7992	1	p3	(GCA)4	12	67	78	AACGCCGTGTCCTACAACAT	59.966	GTATCGCCCTTCATCGCAGA	59.968	264
TRINITY_Contig7992	2	p2	(CG)6	12	245	256	AACGCCGTGTCCTACAACAT	59.966	GTATCGCCCTTCATCGCAGA	59.968	264
TRINITY_Contig7992	3	p3	(GCA)5	15	1297	1311	AACGCCGTGTCCTACAACAT	59.966	GTATCGCCCTTCATCGCAGA	59.968	264
TRINITY_Contig7996	1	p3	(GGC)5	15	1191	1205	AAAGCGAGGAATGCCATGGA	60.034	CGCATCATTCAAGCCCCATG	59.968	182
TRINITY_Contig7996	2	c	(GGT)6ggagctggtgctgggctctccggcgagagggatccgtcacgggagggttgctgcgaagggggagaaggaggcgcgacgggatcgaccgccggcggg(GGC)6	132	1359	1490	AAAGCGAGGAATGCCATGGA	60.034	CGCATCATTCAAGCCCCATG	59.968	182
TRINITY_Contig7997	1	p3	(CGG)4	12	69	80	AGCATGGTAAGGCTTGGGAC	60.034	ACATCCCTCTCAAAGCCAGC	60.035	166
TRINITY_Contig7999	1	p3	(TTG)4	12	1477	1488	ACCCAGAGGAGCCTACTCAG	60.033	ATGCCATGACCAGGAAGCAA	59.96	174
TRINITY_Contig8000	1	p4	(CCAT)3	12	2135	2146	TCCATGAAGGTGACGTGGTG	59.965	TGCTCCATCTCCTCCATCCA	60.031	207
TRINITY_Contig8003	1	p3	(CGG)4	12	429	440	GTTCGAGATGGAGGACGACG	60.248	GAACGACAGATTGTTGCCGG	59.834	142
TRINITY_Contig8006	1	c	(GGC)5catggcgggcacgagagagaggagggctcggacgggggcggcggccggcggcgcgtgggtctaggggtttgggattcccccttt(TC)6	112	2055	2166	TTTGCACAATCTTCGCTGGC	60.039	GTGCCAGCAAGTTTCCACTG	59.969	129
TRINITY_Contig8008	1	p3	(CTT)13	39	175	213	GCCCTTGCTTCCGATACTGT	60.108	CATTTGGTGAACGCCGGAAG	60.11	180
TRINITY_Contig8008	2	p4	(CCGG)3	12	438	449	GCCCTTGCTTCCGATACTGT	60.108	CATTTGGTGAACGCCGGAAG	60.11	180
TRINITY_Contig8008	3	p4	(CGCA)3	12	648	659	GCCCTTGCTTCCGATACTGT	60.108	CATTTGGTGAACGCCGGAAG	60.11	180
TRINITY_Contig8009	1	p3	(ACC)4	12	1203	1214	CGAGATGACGAGAGACCACG	59.973	AGAGAGCGGAAGTGGAGGAT	60.033	200
TRINITY_Contig8009	2	p4	(CAGG)4	16	1326	1341	CGAGATGACGAGAGACCACG	59.973	AGAGAGCGGAAGTGGAGGAT	60.033	200
TRINITY_Contig8009	3	c	(CCTC)3ccaacccgggcgcaaacaaaaatccccatccaactagtcccagtagaccatgctagtgctctgtacggcggtcctgttctgttcggcacc(AACA)3	114	1506	1619	CGAGATGACGAGAGACCACG	59.973	AGAGAGCGGAAGTGGAGGAT	60.033	200
TRINITY_Contig8009	4	p4	(AAGG)5	20	1870	1889	CGAGATGACGAGAGACCACG	59.973	AGAGAGCGGAAGTGGAGGAT	60.033	200
TRINITY_Contig8009	5	p3	(GGC)4	12	2199	2210	CGAGATGACGAGAGACCACG	59.973	AGAGAGCGGAAGTGGAGGAT	60.033	200
TRINITY_Contig8011	1	p5	(ATTTA)3	15	27	41	TTACATTGGGGCCAGGAAGG	59.665	CGGTGATGTCACTGCTGCTA	60.109	276
TRINITY_Contig8012	1	p3	(GCG)4	12	28	39	GCTTCGATGCGTTCCACATC	59.973	ATTCTGGCGAGGCTAGCATC	59.965	190
TRINITY_Contig8013	1	p4	(GCTC)3	12	28	39	ACCTTCTTCACGAAGCAGGG	59.965	GAGCAGGATGATGGAGACGG	59.967	108
TRINITY_Contig8013	2	c	(CGT)4cgcattcatagctttttcgatcgcgttggtttaatcgctcgcgccgagtgcacggtgtgtaatagtggtggcgtgcctgcgcc(CGT)4	107	250	356	ACCTTCTTCACGAAGCAGGG	59.965	GAGCAGGATGATGGAGACGG	59.967	108
TRINITY_Contig8013	3	p3	(CGG)4	12	651	662	ACCTTCTTCACGAAGCAGGG	59.965	GAGCAGGATGATGGAGACGG	59.967	108
TRINITY_Contig8013	4	p3	(GAC)4	12	932	943	ACCTTCTTCACGAAGCAGGG	59.965	GAGCAGGATGATGGAGACGG	59.967	108
TRINITY_Contig8014	1	p4	(TTCA)3	12	25	36	ATCACGGTCGAGATGGCATC	59.967	CAGAGACAACACTCGCCACT	59.967	137
TRINITY_Contig8015	1	p6	(GCTCCT)3	18	633	650	ATCCAAGGCGAGTGCTCAAA	59.963	CAATGCAATGCCCAAGCCTT	60.036	192
TRINITY_Contig8015	2	p3	(TGG)4	12	858	869	ATCCAAGGCGAGTGCTCAAA	59.963	CAATGCAATGCCCAAGCCTT	60.036	192
TRINITY_Contig8017	1	c	(CTCCG)4cctcgcctcccacctcaccgcgcccgctccacccagatctcgccgcgatggcccgcgccgccgctgccgccgcccgcgcggc(CCT)4cgccgtcgccggggtcctcatcagtc(CGG)4	152	329	480	TTGTTGATGTTGCCTGCTGC	59.968	GAACTGGCGGAAGAGTGTGA	59.966	131
TRINITY_Contig8017	2	p3	(GAT)4	12	1689	1700	TTGTTGATGTTGCCTGCTGC	59.968	GAACTGGCGGAAGAGTGTGA	59.966	131
TRINITY_Contig8021	1	p5	(CGGCG)3	15	483	497	AACCAATTCAACACGGTGCG	59.97	GCGAGAGATCGACTGGTCTG	59.971	124
TRINITY_Contig8021	2	p3	(GCT)4	12	618	629	AACCAATTCAACACGGTGCG	59.97	GCGAGAGATCGACTGGTCTG	59.971	124
TRINITY_Contig8021	3	p5	(CATGG)3	15	1356	1370	AACCAATTCAACACGGTGCG	59.97	GCGAGAGATCGACTGGTCTG	59.971	124
TRINITY_Contig8024	1	p5	(CGCGC)3	15	547	561	CTTGCCAACACATACACGCC	60.11	TCTCCCCTCCGACATCGAC	60.453	114
TRINITY_Contig8025	1	p3	(TGA)4	12	420	431	CGATCAGCCTCACCAACTGT	60.037	CACACACTGAGCAGACTCGT	59.969	270
TRINITY_Contig8026	1	p4	(CTCG)3	12	305	316	TGCCGGTCGACAAGATTTGA	59.966	GAGATGGGAGCGGATTAGGC	60.037	150
TRINITY_Contig8027	1	p4	(TATT)5	20	547	566	ACCGTGGTGAGAGGAGATCA	59.96	TGCGCTCACCATCATCCTTT	60.035	201
TRINITY_Contig8027	2	p3	(ATG)4	12	1441	1452	ACCGTGGTGAGAGGAGATCA	59.96	TGCGCTCACCATCATCCTTT	60.035	201
TRINITY_Contig8027	3	p2	(CT)8	16	1612	1627	ACCGTGGTGAGAGGAGATCA	59.96	TGCGCTCACCATCATCCTTT	60.035	201
TRINITY_Contig8029	1	c	(AG)6gaagaacgggaaagagaaggagatgtggatgaggctccttctctttcccgttcttc(CT)6	80	176	255	AGGGGCAAAACGGGAAAGAA	60.106	GTCGCATTCATGGTGATGGC	59.97	214
TRINITY_Contig8032	1	p3	(TCA)4	12	311	322	ACTTCACGCTTCTCAGCCTC	60.038	TGAAGTCGACGCTCTCCAAC	60.039	214
TRINITY_Contig8032	2	p3	(GGC)4	12	862	873	ACTTCACGCTTCTCAGCCTC	60.038	TGAAGTCGACGCTCTCCAAC	60.039	214
TRINITY_Contig8033	1	p4	(CCCT)3	12	171	182	CGCGGTGAGTATGCAATGTG	59.973	CCGTCAGGATCTCGTACAGC	59.97	126
TRINITY_Contig8033	2	p3	(CGG)4	12	815	826	CGCGGTGAGTATGCAATGTG	59.973	CCGTCAGGATCTCGTACAGC	59.97	126
TRINITY_Contig8036	1	p3	(GCC)5	15	234	248	GAGCGAGCTGTGCTTATTGC	59.973	GATGCTGTCAACCTGGTGGA	59.963	225
TRINITY_Contig8037	1	p4	(GTAG)4	16	4707	4722	CCTCCACGTCGATAAGGAGC	59.969	TTTAACGTTCTGGGGGAGCC	59.963	167

TRINITY_Contig8038	1	p3	(CGC)4	12	254	265	AACGCTAATATGGCCGCCAT	60.251	TGGGGTCATCGTTACAACCG	60.037	106
TRINITY_Contig8039	1	p4	(AAGG)4	16	224	239	AGCTGCTGCCAGTAGTTCAG	60.037	GCTGCGATTCGTTGATGGTC	59.973	206
TRINITY_Contig8039	2	p5	(CTTTC)4	20	2314	2333	AGCTGCTGCCAGTAGTTCAG	60.037	GCTGCGATTCGTTGATGGTC	59.973	206
TRINITY_Contig8041	1	c	(GCC)4ccagcagct(GCA)4	33	482	514	GTCGCGGCCTTCATAGATGA	59.968	ACCACAGCACAAAAGGGGAA	60.033	116
TRINITY_Contig8042	1	p4	(CCCG)3	12	125	136	GTCGCGGCCTTCATAGATGA	59.968	ACCACAGCACAAAAGGGGAA	60.033	116
TRINITY_Contig8042	2	p3	(TGA)4	12	884	895	GTCGCGGCCTTCATAGATGA	59.968	ACCACAGCACAAAAGGGGAA	60.033	116
TRINITY_Contig8042	3	p3	(TCA)4	12	7250	7261	GTCGCGGCCTTCATAGATGA	59.968	ACCACAGCACAAAAGGGGAA	60.033	116
TRINITY_Contig8042	4	c	(GCC)4ccagcagct(GCA)4	33	8193	8225	GTCGCGGCCTTCATAGATGA	59.968	ACCACAGCACAAAAGGGGAA	60.033	116
TRINITY_Contig8044	1	p2	(TC)6	12	1220	1231	CATAGCGCACAATGTCACCG	59.973	TCAACATGCATCAGTCGGCT	60.036	108
TRINITY_Contig8044	2	p6	(TCATCC)3	18	1380	1397	CATAGCGCACAATGTCACCG	59.973	TCAACATGCATCAGTCGGCT	60.036	108
TRINITY_Contig8045	1	p4	(AATC)3	12	75	86	TACTGTCGGGAGGAGAAGGG	60.033	AGATGCCTTCCTTGCCTGTC	60.035	117
TRINITY_Contig8047	1	p3	(CGG)4	12	1113	1124	CGCGCCTCCTCAATGATACT	59.967	CAAGAGCAGGGAGAAGCACA	59.964	174
TRINITY_Contig8048	1	p3	(GCC)5	15	84	98	CGGACGATGGATGGATGGAG	60.038	TCACAGAGGCATCCTACGGA	60.034	113
TRINITY_Contig8048	2	c	(ACAT)17actagctttacgtgccccagtaat catgacaaggtctcccgttcccggcgtgcaga cacctttgtagtacttgccggccggctagccgg cgccggac(GATG)3	177	1373	1549	CGGACGATGGATGGATGGAG	60.038	TCACAGAGGCATCCTACGGA	60.034	113
TRINITY_Contig8048	3	p5	(AGGTT)3	15	2862	2876	CGGACGATGGATGGATGGAG	60.038	TCACAGAGGCATCCTACGGA	60.034	113
TRINITY_Contig8049	1	p4	(AATA)3	12	1496	1507	TGTGTCACGAGAGCTGCAAT	59.966	TGTTTCACGGGGATGACCAG	59.964	146
TRINITY_Contig8049	2	p3	(GCG)4	12	3506	3517	TGTGTCACGAGAGCTGCAAT	59.966	TGTTTCACGGGGATGACCAG	59.964	146
TRINITY_Contig8049	3	p4	(GCGA)3	12	3696	3707	TGTGTCACGAGAGCTGCAAT	59.966	TGTTTCACGGGGATGACCAG	59.964	146
TRINITY_Contig8050	1	p5	(AGGCG)4	20	12	31	CCATTCCAATTCCACCCCCA	59.958	GGACATACTGATCGAGCGCA	59.969	235
TRINITY_Contig8050	2	p4	(TTCA)3	12	361	372	CCATTCCAATTCCACCCCCA	59.958	GGACATACTGATCGAGCGCA	59.969	235
TRINITY_Contig8055	1	p3	(CGC)6	18	618	635	TGGGTGGATCGATCTGAGGT	60.032	CAAATGTGCAGGCCCAACTC	60.038	111
TRINITY_Contig8057	1	c	(TCCTCG)3tctgcgtcgtcctcgtcgtcctcg ccgcggccgccttcccggtaggaagcggtggc gcccgagagatcgccgacccaggggcgggcg tccgg(GAC)4	122	1122	1243	TGCTCCATCGGTTATCGTCG	59.97	GCTGGTGAAGTTCTCGGTCA	59.966	206
TRINITY_Contig8058	1	p3	(GCG)4	12	213	224	TCGTACTTCGTTCTCGCTCG	59.906	CCGGTAGTCAGTCCACAACC	60.038	125
TRINITY_Contig8058	2	c	(GCG)4gtggcagctcctccactagcagtagc tccggccatggcgatcgtacttcgtt(CTCG)3	76	723	798	TCGTACTTCGTTCTCGCTCG	59.906	CCGGTAGTCAGTCCACAACC	60.038	125
TRINITY_Contig8059	1	c	(CCG)4ccacctacgcgtgcccgtccacccgc gccggcgccggttc(CGA)6gtccaagccctc ctccgcct(CCG)5	105	345	449	CGTGGTGAGAGTCTTGCTGT	59.967	TCCAGATCCCATCTCCGGTT	60.031	127
TRINITY_Contig8061	1	p3	(CGT)4	12	1398	1409	GAGGTAGACGATGGCTGACG	59.97	GACGCTCTTCCGATCTCCAG	59.97	106
TRINITY_Contig8061	2	p5	(GGGAG)3	15	1608	1622	GAGGTAGACGATGGCTGACG	59.97	GACGCTCTTCCGATCTCCAG	59.97	106
TRINITY_Contig8062	1	p3	(CCG)4	12	1416	1427	CAATGCGTGATGCTACGGTG	59.973	CAAGGCCCTGATGAAGCTCA	60.035	116
TRINITY_Contig8064	1	p3	(CCG)7	21	446	466	CGATGTACACCAGGATCGGG	59.968	TGTTCACCGTCATTCCCCAG	59.964	273
TRINITY_Contig8064	2	p3	(CCG)5	15	733	747	CGATGTACACCAGGATCGGG	59.968	TGTTCACCGTCATTCCCCAG	59.964	273
TRINITY_Contig8065	1	p2	(CT)6	12	137	148	ATGATCATCGACGAGGTGC	59.967	GGAGGCAAATGCAGCAAACA	59.966	111
TRINITY_Contig8067	1	p3	(TGA)4	12	2142	2153	TATCAGGGACCGCACTCTGA	60.034	GATGACACAGCGCGAATCAC	59.974	117
TRINITY_Contig8067	2	p3	(CCG)6	18	2985	3002	TATCAGGGACCGCACTCTGA	60.034	GATGACACAGCGCGAATCAC	59.974	117
TRINITY_Contig8068	1	p3	(TAG)4	12	1073	1084	CGCGGGAATCTCTAACGGAA	59.898	GATCCCGTGCTCCAGATCAG	59.967	168
TRINITY_Contig8069	1	p3	(CGA)4	12	742	753	CCGTTTTCACCGCACCAATT	59.969	CCGGCAGTTCTCCATTTCCT	60.035	267
TRINITY_Contig8069	2	c	(CGT)5cctcg(TCGTCC)4	44	1194	1237	CCGTTTTCACCGCACCAATT	59.969	CCGGCAGTTCTCCATTTCCT	60.035	267
TRINITY_Contig8069	3	p6	(TTGCCG)4	24	1493	1516	CCGTTTTCACCGCACCAATT	59.969	CCGGCAGTTCTCCATTTCCT	60.035	267
TRINITY_Contig8069	4	p3	(CCG)4	12	1988	1999	CCGTTTTCACCGCACCAATT	59.969	CCGGCAGTTCTCCATTTCCT	60.035	267
TRINITY_Contig8069	5	p3	(CGG)4	12	2242	2253	CCGTTTTCACCGCACCAATT	59.969	CCGGCAGTTCTCCATTTCCT	60.035	267
TRINITY_Contig8069	6	c	(CCGCTC)3(CCG)4	30	2543	2572	CCGTTTTCACCGCACCAATT	59.969	CCGGCAGTTCTCCATTTCCT	60.035	267
TRINITY_Contig8070	1	p3	(AAT)4	12	1440	1451	GCCATGGCTGACTGAACTCT	60.036	CCGCTCGCTACCTTTCTTCT	59.826	109
TRINITY_Contig8071	1	p3	(CTC)4	12	149	160	GTGGGACGTTGGATGGTGAT	60.035	GTTGCTCCTTTTGGCCCTTG	59.966	131
TRINITY_Contig8071	2	p3	(CCT)4	12	2753	2764	GTGGGACGTTGGATGGTGAT	60.035	GTTGCTCCTTTTGGCCCTTG	59.966	131
TRINITY_Contig8072	1	p3	(GCC)4	12	2684	2695	GTCCGACGTCTACAGCTACG	59.974	CGTACTCCTCGTATGCCCAC	59.97	119
TRINITY_Contig8073	1	c	(CAT)4cacttacac(ACAG)4acaaacagc aacaattattgaagaaccggagtacagattta act(CAG)4	93	167	259	AGAGTTCGGATCTCCCACCA	59.959	CCGATTGAGTGGCTCACCTT	60.036	205
TRINITY_Contig8073	2	p4	(ATCA)3	12	388	399	AGAGTTCGGATCTCCCACCA	59.959	CCGATTGAGTGGCTCACCTT	60.036	205
TRINITY_Contig8073	3	p3	(CGA)4	12	835	846	AGAGTTCGGATCTCCCACCA	59.959	CCGATTGAGTGGCTCACCTT	60.036	205
TRINITY_Contig8073	4	p3	(AGG)4	12	965	976	AGAGTTCGGATCTCCCACCA	59.959	CCGATTGAGTGGCTCACCTT	60.036	205
TRINITY_Contig8074	1	p3	(AAG)4	12	2068	2079	CTCTCGATGCTCGTCCTTCC	59.97	CCGTAGAGCGTCGCATTAGT	59.971	245
TRINITY_Contig8076	1	c	(CGC)4cgatcgcagcaaaagggaggctcca ggcggcgg(GCA)5	60	128	187	GGCTCCAGGCAGTCATTTCT	60.035	TTGACGCAAAAGAACCAGCG	59.971	266
TRINITY_Contig8082	1	c	(GCA)4gccgcggcgctcaggtcgccca(CC G)6	52	249	300	CGTTGCGGCCATAAGTAAGC	59.972	GAGGCAATCTCCGGATGAGG	59.965	219
TRINITY_Contig8082	2	p3	(CCG)4	12	846	857	CGTTGCGGCCATAAGTAAGC	59.972	GAGGCAATCTCCGGATGAGG	59.965	219
TRINITY_Contig8083	1	c	(TGAGC)3(TTAGC)3	30	408	437	GCCACGGTGATGTACAGGAA	60.037	CTAGTGCTCTGCTCGCTCTC	59.971	261
TRINITY_Contig8087	1	p3	(CGG)4	12	915	926	GCGGTACTTGTGCTCCTTCT	60.037	CCATCATCGCGAGGTACCTC	60.039	248
TRINITY_Contig8087	2	p3	(GCC)8	24	1195	1218	GCGGTACTTGTGCTCCTTCT	60.037	CCATCATCGCGAGGTACCTC	60.039	248
TRINITY_Contig8089	1	c	(AC)6gaagaggagcgagctcagacccaaca agaagca(GCAG)3	57	135	191	GTTGGCCACCAGTACGATCA	60.037	ATCTTCAGGTCCACCTTGCG	60.036	178
TRINITY_Contig8090	1	p3	(GCG)4	12	1153	1164	GAATTCGGAAAGGCGGCATC	59.971	TTGTGGACGACGTTAGGTGG	59.968	167
TRINITY_Contig8091	1	p3	(CCG)5	15	3180	3194	CGTCTGCAATGTCATGCTCG	59.974	TATGCCGCAACCTTGGTTCT	59.962	240
TRINITY_Contig8092	1	p3	(CGC)4	12	685	696	GCGGCTCTGATGAGTACCTC	59.969	TCTGGTTGTAGCTGCACTGG	59.965	144
TRINITY_Contig8093	1	p3	(TCC)4	12	135	146	GTTGTGGACTGGGGAGGATG	60.035	CCCTGCTTTGACCACGTTTG	59.969	140
TRINITY_Contig8095	1	p3	(GCG)4	12	916	927	GTCCGCATCCAGATCCTCAG	59.967	TGTGCAGCTTGATGGCAAAC	59.968	262
TRINITY_Contig8096	1	p3	(CGA)4	12	156	167	GGCATTCAACCTGTGGAGGA	59.962	GCCTTTCCTCCTTTCGAGCT	60.036	165
TRINITY_Contig8096	2	p3	(ATG)4	12	485	496	GGCATTCAACCTGTGGAGGA	59.962	GCCTTTCCTCCTTTCGAGCT	60.036	165
TRINITY_Contig8097	1	p5	(TCGAG)3	15	403	417	ATGACTTGCTGCCTGACGAA	59.965	AGTGACGCTCTCCAGAGGAT	60.034	158
TRINITY_Contig8097	2	p3	(CAC)4	12	2182	2193	ATGACTTGCTGCCTGACGAA	59.965	AGTGACGCTCTCCAGAGGAT	60.034	158
TRINITY_Contig8101	1	p4	(TCCA)3	12	22	33	GCAGCAGCATCAGATCAAGC	59.97	GTCCTCCTTCTTGAACCCCG	60.036	229
TRINITY_Contig8105	1	p2	(TA)9	18	509	526	AGGAACTGAGTGTGTGCTCG	59.967	TCCCAGAGTCCCTCCTTCAG	59.958	125
TRINITY_Contig8105	2	c	(TAGA)5(CAGA)3t(AGAC)3	45	650	694	AGGAACTGAGTGTGTGCTCG	59.967	TCCCAGAGTCCCTCCTTCAG	59.958	125
TRINITY_Contig8106	1	p3	(CCA)5	15	898	912	AAGAAGCGTCGGGAACATGT	59.965	GTGGTACGTTGTCTGGAGCA	59.967	160
TRINITY_Contig8106	2	p3	(GGC)5	15	2470	2484	AAGAAGCGTCGGGAACATGT	59.965	GTGGTACGTTGTCTGGAGCA	59.967	160
TRINITY_Contig8108	1	p3	(GCG)4	12	143	154	CAGGGAGGTTCGGTCGAAAA	59.966	TCCACATCCTACAGAGGCGA	60.034	220
TRINITY_Contig8108	2	p3	(CCG)4	12	364	375	CAGGGAGGTTCGGTCGAAAA	59.966	TCCACATCCTACAGAGGCGA	60.034	220
TRINITY_Contig8110	1	p5	(TTCCC)3	15	115	129	GTAACCCACCAGCTGCAGAT	60.035	TGCTCTCGCTGGATGTGTTT	59.965	207
TRINITY_Contig8111	1	p4	(TTTC)3	12	551	562	TGATGTGGTCGTCACCCTG	59.965	TGATCCGGGCTACAGAGACA	60.034	141
TRINITY_Contig8113	1	p3	(CCA)4	12	2703	2714	TGAGGGTCTTGGTAGCGAGA	59.961	TTGTTCAGTGGGTTTGGCCT	60.033	183
TRINITY_Contig8119	1	p4	(GAGG)3	12	399	410	TGGGTGGATTGGGTTCGATG	60.035	GGCTTCCTCCGTTGTTGGTA	59.964	114
TRINITY_Contig8120	1	c	(GCC)8gtggcgtcgccgtcgtctctcccgtca tcggcctcggcactcat(GGC)4	80	2010	2089	AGGATGGAGAGCATGTCCCT	60.03	GCTTGTGCTCCCCAAACATG	60.038	123
TRINITY_Contig8120	2	p3	(GAG)4	12	2208	2219	AGGATGGAGAGCATGTCCCT	60.03	GCTTGTGCTCCCCAAACATG	60.038	123
TRINITY_Contig8122	1	p3	(GCT)4	12	151	162	TGCGTGCTTGTTCTTGTTGG	59.9	TGCCTCACCGACTGAAAGTC	59.966	148
TRINITY_Contig8123	1	p3	(CGG)4	12	421	432	GCAACTTCACCGAGGAGGAA	59.965	CCACTGATCAGAGGCTGGTG	60.108	193
TRINITY_Contig8123	2	p2	(GA)17	34	712	745	GCAACTTCACCGAGGAGGAA	59.965	CCACTGATCAGAGGCTGGTG	60.108	193
TRINITY_Contig8125	1	p6	(GGCCCC)4	24	90	113	AAGAAACCTGTAGGGCGAGC	60.036	GGAGTCCTTTCCAGCAGCTT	59.963	267
TRINITY_Contig8127	1	p3	(GCA)6	18	1897	1914	GCGGTGAATTCACTCCCTGA	60.036	CGTCTCAAAGGACAGCAGGT	59.966	243
TRINITY_Contig8127	2	p3	(TGC)6	18	2503	2520	GCGGTGAATTCACTCCCTGA	60.036	CGTCTCAAAGGACAGCAGGT	59.966	243
TRINITY_Contig8129	1	p3	(CCG)4	12	40	51	TGCGCTGAACATGCTACTGA	60.037	GACCACTGCTGGAACTCCTC	60.037	172
TRINITY_Contig8134	1	p3	(GGC)4	12	1686	1697	CATGCTCTGAAGACAGCGGA	60.108	ATGGCATCTGTGAGGTTGCA	59.961	163
TRINITY_Contig8134	2	p3	(GCG)4	12	1813	1824	CATGCTCTGAAGACAGCGGA	60.108	ATGGCATCTGTGAGGTTGCA	59.961	163
TRINITY_Contig8135	1	p2	(AC)8	16	265	280	CAGACAGCTCGACGGAGATC	59.971	CCTCCATTTCCACGACTGCT	60.036	227
TRINITY_Contig8137	1	c	(CG)6acgatctcaaggctgaggtcctcgtta cccacgtcgtcgtcctcctcctcgtcgccgctgc cgccccggcgcggatgccgggagggcgggga (GCC)4	116	1849	1964	CAGTTTGCTGCCACATGTCC	60.039	ACAGGAAGAAGCACCGGAAG	59.965	271
TRINITY_Contig8138	1	p4	(TATT)3	12	2516	2527	CACCACCATGTGCAATGCAA	59.966	GGCCTCAGTGCAAACTGTTG	59.969	272
TRINITY_Contig8142	1	p5	(CTTTT)3	15	1223	1237	TTGACGTCGGTGGTTAGTGG	59.968	CCATGTCTACTCGACGCCTC	59.97	103
TRINITY_Contig8142	2	p5	(TGGCA)4	20	1361	1380	TTGACGTCGGTGGTTAGTGG	59.968	CCATGTCTACTCGACGCCTC	59.97	103
TRINITY_Contig8142	3	p3	(AAG)4	12	2742	2753	TTGACGTCGGTGGTTAGTGG	59.968	CCATGTCTACTCGACGCCTC	59.97	103
TRINITY_Contig8142	4	c	(CCAT)3tccaatattccattcccatccaatgg tgctatcgctctccctggcgccgccagtagca(GCC)4	82	2859	2940	TTGACGTCGGTGGTTAGTGG	59.968	CCATGTCTACTCGACGCCTC	59.97	103

TRINITY_Contig8145	1	p3	(GCG)5	15	1359	1373	GGTGCTTCAGAAGGAACCGA	59.965	GTGCAGTTTGGAGTCGAGGA	59.966	104
TRINITY_Contig8145	2	c	(GCG)4tcgaggtagacgaacggcgccgggttctgatgcgccatg(GCC)4	63	1683	1745	GGTGCTTCAGAAGGAACCGA	59.965	GTGCAGTTTGGAGTCGAGGA	59.966	104
TRINITY_Contig8147	1	p3	(CGC)5	15	1343	1357	CTGCATCCAACCGTAATGCG	59.972	TGTCGGAGCTCTTCACCAAC	59.966	197
TRINITY_Contig8147	2	p3	(GCG)8	24	1552	1575	CTGCATCCAACCGTAATGCG	59.972	TGTCGGAGCTCTTCACCAAC	59.966	197
TRINITY_Contig8147	3	c	(GTG)4ggtagtggagccgtgga(AGGCG)3tgtaaataaatc(GCG)4	68	1696	1763	CTGCATCCAACCGTAATGCG	59.972	TGTCGGAGCTCTTCACCAAC	59.966	197
TRINITY_Contig8147	4	p4	(GGCA)3	12	1878	1889	CTGCATCCAACCGTAATGCG	59.972	TGTCGGAGCTCTTCACCAAC	59.966	197
TRINITY_Contig8148	1	p3	(CGG)5	15	337	351	CCTCTCCAAGCATGCAGTGA	60.036	GTTGTGCACCTTGAACACCC	59.898	136
TRINITY_Contig8148	2	p3	(CGG)4	12	1045	1056	CCTCTCCAAGCATGCAGTGA	60.036	GTTGTGCACCTTGAACACCC	59.898	136
TRINITY_Contig8148	3	p3	(CGG)4	12	1408	1419	CCTCTCCAAGCATGCAGTGA	60.036	GTTGTGCACCTTGAACACCC	59.898	136
TRINITY_Contig8148	4	p3	(TAC)4	12	1851	1862	CCTCTCCAAGCATGCAGTGA	60.036	GTTGTGCACCTTGAACACCC	59.898	136
TRINITY_Contig8149	1	p3	(CGG)4	12	195	206	GGGTTCACCGGACTCCATTT	59.962	ATACTTGCAAGGGGAGGCAC	60.034	106
TRINITY_Contig8149	2	p3	(CCG)4	12	1228	1239	GGGTTCACCGGACTCCATTT	59.962	ATACTTGCAAGGGGAGGCAC	60.034	106
TRINITY_Contig8151	1	p4	(TCCA)3	12	119	130	ATCCAGCTCCTACAGGCAGA	60.032	GTGTTGTCGACCTGGTAGCA	59.967	123
TRINITY_Contig8151	2	p3	(CCA)5	15	270	284	ATCCAGCTCCTACAGGCAGA	60.032	GTGTTGTCGACCTGGTAGCA	59.967	123
TRINITY_Contig8153	1	p6	(CCACCT)3	18	17	34	ACCTCCATACCCTCCTCCAC	60.031	GGTCTTACGGATCCGACGAC	59.971	113
TRINITY_Contig8153	2	c	(GCC)5tccacctctaccgcccccgccgccgtagcctccaccgccacctccata(TCC)4	75	202	276	ACCTCCATACCCTCCTCCAC	60.031	GGTCTTACGGATCCGACGAC	59.971	113
TRINITY_Contig8153	3	p3	(CCG)4	12	380	391	ACCTCCATACCCTCCTCCAC	60.031	GGTCTTACGGATCCGACGAC	59.971	113
TRINITY_Contig8154	1	p6	(GAGGGG)3	18	1640	1657	AGTAGATGGCAACGCAGACC	60.108	CCTCCATCCTTCCTCCTCCA	60.031	148
TRINITY_Contig8158	1	p4	(GCCG)3	12	1151	1162	GGTGACATCCACGGTCAGTT	59.965	AGCAGGAAGAAGTTGTCCGG	59.965	176
TRINITY_Contig8160	1	c	(GGA)4tgaggcgaccatggccttctcctccgcctgcccgtccttccactccgaggacttcgtgtcccctccgctgaaggcggcgccgggcaccgcgccggc(GCA)5	123	373	495	CCCAACACAAAGCACAGCTC	59.969	ATGTACTCCTCGTCCCCGAA	60.034	225
TRINITY_Contig8160	2	c	(GCG)4agggcgacgtcatgtacatgcgcgcgcggttcgagcgcgtggtgggctcccgcgactcggaggcgttctacatgatgaacccggacagcacca(GCG)7	126	984	1109	CCCAACACAAAGCACAGCTC	59.969	ATGTACTCCTCGTCCCCGAA	60.034	225
TRINITY_Contig8164	1	p6	(GATATA)4	24	81	104	ATGACCTTGGCCTGCTCTTC	60.035	CAGACCTAAGCAACCCACGT	59.965	259
TRINITY_Contig8165	1	c	(GAG)4ctcgcgaaccgaatccctcgccttggcctggcgcttccaac(AG)16	85	1987	2071	TCCGGAACTTCCTTGACAGC	59.965	GCAGTGTATACCGGGGACAG	59.896	108
TRINITY_Contig8166	1	c	(GGA)5caagcgcgggctgtgcaaggagacggggcagagggcagcggtcgcggccgtgcccgcgcacggcggcaagggcggcgcc(TCG)5	109	280	388	TCCGTCATACAATCGGCCAG	59.896	TTGTCCTCCTCCTCCTCCG	60	208
TRINITY_Contig8166	2	p3	(TTC)4	12	672	683	TCCGTCATACAATCGGCCAG	59.896	TTGTCCTCCTCCTCCTCCG	60	208
TRINITY_Contig8168	1	p6	(CGACGC)3	18	2086	2103	TGATCGAATCAGCGAGGAGC	59.969	GATGTCCTCCGTCATCGACC	59.969	200
TRINITY_Contig8169	1	p4	(CCTC)3	12	46	57	CCACCCCTGCTTGGATTGAT	60.033	GACAGAAGACAAGCCCTGCT	59.964	105
TRINITY_Contig8169	2	p2	(CG)7	14	182	195	CCACCCCTGCTTGGATTGAT	60.033	GACAGAAGACAAGCCCTGCT	59.964	105
TRINITY_Contig8170	1	p3	(GGT)5	15	1707	1721	TCCTGACAACGGAGCTTTCC	59.965	CAGAAGGAGGTTGAACGGCT	59.965	244
TRINITY_Contig8171	1	p3	(TAC)4	12	1964	1975	GCGTGGGAGAGAAACAGGAA	59.965	TCAGTTGGTCTGTCAGCGTC	59.968	158
TRINITY_Contig8171	2	p4	(CTGT)3	12	2157	2168	GCGTGGGAGAGAAACAGGAA	59.965	TCAGTTGGTCTGTCAGCGTC	59.968	158
TRINITY_Contig8171	3	p3	(GCG)4	12	2627	2638	GCGTGGGAGAGAAACAGGAA	59.965	TCAGTTGGTCTGTCAGCGTC	59.968	158
TRINITY_Contig8173	1	p3	(CGC)7	21	69	89	GAGCTCTAGCATCTGGGCAG	59.966	ATGTTCACCAGCCCCATCAG	60.034	119
TRINITY_Contig8176	1	p2	(AG)6	12	2001	2012	AACACTGCAGTGGTACGAGG	59.966	TGGGGTACAGGATGAGAGGG	60.031	112
TRINITY_Contig8178	1	p5	(CGATG)3	15	117	131	GCTACTCACCACTGTGCCTT	59.965	TCCGCCCACTGTTCCATTAC	60.036	206
TRINITY_Contig8178	2	p4	(CGCA)3	12	261	272	GCTACTCACCACTGTGCCTT	59.965	TCCGCCCACTGTTCCATTAC	60.036	206
TRINITY_Contig8178	3	p6	(GGCCGA)3	18	719	736	GCTACTCACCACTGTGCCTT	59.965	TCCGCCCACTGTTCCATTAC	60.036	206
TRINITY_Contig8178	4	p5	(GAGAG)3	15	1497	1511	GCTACTCACCACTGTGCCTT	59.965	TCCGCCCACTGTTCCATTAC	60.036	206
TRINITY_Contig8178	5	p5	(CTCTA)3	15	1627	1641	GCTACTCACCACTGTGCCTT	59.965	TCCGCCCACTGTTCCATTAC	60.036	206
TRINITY_Contig8179	1	p3	(CGG)4	12	1406	1417	TCAAACATACAGCCCCCACC	59.961	TTGGATCTGCATGGTGCCAT	60.033	112
TRINITY_Contig8179	2	p3	(GCC)4	12	1818	1829	TCAAACATACAGCCCCCACC	59.961	TTGGATCTGCATGGTGCCAT	60.033	112
TRINITY_Contig8180	1	p3	(TGC)6	18	390	407	TATGTGACGCCGCACTCTTT	60.038	TCACCAGAGCTTCTGTGCTG	59.966	127
TRINITY_Contig8180	2	p4	(GCAA)3	12	1055	1066	TATGTGACGCCGCACTCTTT	60.038	TCACCAGAGCTTCTGTGCTG	59.966	127
TRINITY_Contig8180	3	p6	(GGAGCG)3	18	1387	1404	TATGTGACGCCGCACTCTTT	60.038	TCACCAGAGCTTCTGTGCTG	59.966	127
TRINITY_Contig8180	4	p3	(CGG)4	12	1737	1748	TATGTGACGCCGCACTCTTT	60.038	TCACCAGAGCTTCTGTGCTG	59.966	127
TRINITY_Contig8181	1	p3	(GCT)8	24	308	331	GGTGCTGCGCAAACTACAAA	59.97	TCCGTATCCTGAGCCCTTCA	60.033	277
TRINITY_Contig8181	2	p2	(CG)6	12	3020	3031	GGTGCTGCGCAAACTACAAA	59.97	TCCGTATCCTGAGCCCTTCA	60.033	277
TRINITY_Contig8181	3	p3	(CGG)4	12	3168	3179	GGTGCTGCGCAAACTACAAA	59.97	TCCGTATCCTGAGCCCTTCA	60.033	277
TRINITY_Contig8184	1	p3	(GCC)5	15	79	93	GGACCTCATCAGCATCTCCG	59.967	GACACCGGTGACCTCTGTTT	59.894	177
TRINITY_Contig8186	1	c	(GCCGT)3gccacgccgccgccgt(CGC)4	43	199	241	GATCGTTACTGCCGAGGAGG	59.969	CGAGCAAACAATCTCGAGCG	59.976	262
TRINITY_Contig8187	1	p3	(TCG)4	12	236	247	GCATGAACTGGGAGAGCCTT	60.035	CAGAACTCCGGGTTGTCCTC	60.037	100
TRINITY_Contig8189	1	p3	(CCG)4	12	1441	1452	GAGCATATCTCGCAAGCCCT	59.965	AATCCTGGCGTCATCAGCAA	60.035	169
TRINITY_Contig8190	1	p2	(AG)12	24	152	175	GGGCTACTCCTTCATCAGCC	59.892	TTCATGGTCCTGTTCTCGGC	60.036	225
TRINITY_Contig8192	1	p3	(GCG)4	12	1565	1576	ACCTACTTCCCCTACGGCAT	60.031	TTCATCTGCAGGAACGCCAT	60.035	188
TRINITY_Contig8193	1	c	(CAG)4cacc(GGTA)3	28	231	258	CCCGGGCTTGGAGATATCAC	59.965	AGGGCCACCATCCATCTTTG	60.033	276
TRINITY_Contig8198	1	c	(AGG)4cgcgtgcggggcactcgctacgccgccgccccgggcccgcgccgcgagcaggggagggccgcgccgcccggccgcc(AGG)4	100	1996	2095	CCACCTTCTTCCTCACCACC	59.963	AGGAAAACGGCCAGATGAGG	60.035	127
TRINITY_Contig8199	1	p4	(AGCT)3	12	342	353	ATCGGATTTCGGATCAGGCC	59.965	CGCGAATCGATCCGAATTCG	59.91	104
TRINITY_Contig8200	1	p3	(GAT)6	18	705	722	GACGACGACGACGATGATGA	59.974	CCTCATCAACTTCCCCCACC	60.034	118
TRINITY_Contig8200	2	p3	(ATG)4	12	1192	1203	GACGACGACGACGATGATGA	59.974	CCTCATCAACTTCCCCCACC	60.034	118
TRINITY_Contig8200	3	c	(GAC)4gatgatgatgtggttggctttggtgattctggtggtaggggagtgaaaaagtttgattttggcttgtcagag(GAT)4	96	1308	1403	GACGACGACGACGATGATGA	59.974	CCTCATCAACTTCCCCCACC	60.034	118
TRINITY_Contig8201	1	c	(AAAAG)5acc(AAGA)3	40	218	257	CTCTTCGCTCTTGTCCCAGG	60.108	GACCCGACACTTGAAGCTCA	59.966	207
TRINITY_Contig8201	2	p3	(GCG)4	12	1532	1543	CTCTTCGCTCTTGTCCCAGG	60.108	GACCCGACACTTGAAGCTCA	59.966	207
TRINITY_Contig8201	3	c	(CGCT)3c(GCCTT)3	28	1737	1764	CTCTTCGCTCTTGTCCCAGG	60.108	GACCCGACACTTGAAGCTCA	59.966	207
TRINITY_Contig8203	1	c	(AACGA)3tggctagggaag(GAC)5gaggatgagatggatgaattattgatccatgagatcgcgatctgctagtatttccatattgaaagacgggatttctttggttgcccag(GGAT)3	142	2182	2323	ACAGGAGGAGAAGGATCCCC	60.031	ATCCCCCTCGGAGTAGCTTT	60.031	211
TRINITY_Contig8207	1	p4	(GAGG)3	12	729	740	CTCGGTCGAGACATAACCCG	59.971	GTCCATCGCCAAGTTCAAGC	59.831	229
TRINITY_Contig8208	1	p2	(CT)12	24	22	45	CTGTGCCCTGTTTGCAAGTC	59.969	AGCAGAAGAGTGCACAGGTC	59.965	149
TRINITY_Contig8208	2	p3	(TGA)4	12	1525	1536	CTGTGCCCTGTTTGCAAGTC	59.969	AGCAGAAGAGTGCACAGGTC	59.965	149
TRINITY_Contig8209	1	c	(TTCCT)3aattcctagcggcggcgaccgggcgagtggcgccgcccgccagttcttcgtcgggcgacgagcatcggacagggatgagtgaggaggacaaga(CTG)6	126	40	165	GTTCATTCCTCCGGGCAAGA	60.035	GGTGGTTGCAGGCAAGAATG	60.038	227
TRINITY_Contig8210	1	c	(CCGCC)3ccgatgtacttatccaagccatgccgatcgagcgcttgcctccaatccaagctccgggccgttg(CATC)3	91	160	250	AGGACCAAACATCGCCTCAG	60.036	CCAGCGCCAATTGCATAGAC	59.97	189
TRINITY_Contig8211	1	p4	(CTAG)3	12	1767	1778	GGAGCTCTCATGACCGTAGC	59.969	CAAAGAGGCAGCACAAACCC	59.967	266
TRINITY_Contig8214	1	p4	(AAAG)3	12	1988	1999	AGGGCTGATCCTGCTACTGA	60.032	AATCAGTGCCAGACTCCAGC	60.036	148
TRINITY_Contig8214	2	p4	(GGGA)3	12	2420	2431	AGGGCTGATCCTGCTACTGA	60.032	AATCAGTGCCAGACTCCAGC	60.036	148
TRINITY_Contig8215	1	p3	(TGA)5	15	2044	2058	CAGAAATATTTGGGCCGGCG	59.969	TAATGCGCGAGTTGGAGTGT	60.038	100
TRINITY_Contig8216	1	c	(CTC)4gggctcctcgtcctcgctctccggttcgcggcggcgcttgggccgtccacggcgccggcgggtctgcgtctccgcctcctgctcccggcg(CTC)4ggcccgccgcatcgcctccaggtccttctcgtcg(TCC)4tcgagctccacttcctcctcctcgtcgtcatcatcgtcgtccgcggcggcgcggcggcgctgctgct(GCG)4gcttcctgggattggcgtccgagga(GCC)4ggagggcttggtcttgacgggcacc(TCG)6	319	1921	2239	CAAGATCAGGAAGCTGCCCA	60.035	GGGTAGCACGTGACACAAGA	59.967	279
TRINITY_Contig8218	1	p3	(TAG)4	12	316	327	CGTCGTACACAGTGTCACCA	59.97	GTGTGCAGAGGCTAGCTAGG	59.896	175
TRINITY_Contig8220	1	p3	(CCG)4	12	1201	1212	TCGTCGTCGCCTATCTGTTG	59.902	GATGTTCATCGCAAGCGGAC	59.973	156
TRINITY_Contig8222	1	p3	(CAT)4	12	754	765	GATCCTCCTCTTCTGACGCG	59.97	TTTCCGCTGTTGCTTGCTTC	59.969	254
TRINITY_Contig8222	2	p3	(CTC)4	12	1189	1200	GATCCTCCTCTTCTGACGCG	59.97	TTTCCGCTGTTGCTTGCTTC	59.969	254
TRINITY_Contig8227	1	p4	(CCCG)3	12	735	746	TCAAGCCAACCCCCAATCTC	59.96	TCTCGGTAGGGTCCTTCGTT	59.961	130
TRINITY_Contig8227	2	p3	(GAG)4	12	956	967	TCAAGCCAACCCCCAATCTC	59.96	TCTCGGTAGGGTCCTTCGTT	59.961	130
TRINITY_Contig8232	1	p3	(TCC)5	15	860	874	TCTTTCCGGCGTTAGGCAAT	60.036	ACGAGGAGGGGAAGGTAGAC	60.033	185

TRINITY_Contig8233	1	p4	(CCCG)3	12	46	57	AACCGTGCTGTACTGAGAGC	60.038	CCTGCATCCACCCTTTCCAT	60.033	256
TRINITY_Contig8234	1	p3	(GCG)6	18	765	782	CAGATTCCCTTGCTGACGGT	60.036	ACCTCAGAAATGAGCACGCA	59.965	178
TRINITY_Contig8235	1	c*	(CGCCGG)2(CGCCG<G>CT)(GCT)3(GCG)6	47	1762	1808	TGTCTGGTACACAAGTCGGC	59.967	GCTCGGAGATCGCACATACA	59.969	221
TRINITY_Contig8235	2	c	(CCG)6(GAG)4	30	2087	2116	TGTCTGGTACACAAGTCGGC	59.967	GCTCGGAGATCGCACATACA	59.969	221
TRINITY_Contig8237	1	p2	(AG)21	42	97	138	CCAGAGAGTTCTTGCAGCCA	59.964	TCAGGTCCTGCATCAGCTTG	60.036	150
TRINITY_Contig8237	2	p2	(GT)6	12	604	615	CCAGAGAGTTCTTGCAGCCA	59.964	TCAGGTCCTGCATCAGCTTG	60.036	150
TRINITY_Contig8237	3	p2	(GA)8	16	724	739	CCAGAGAGTTCTTGCAGCCA	59.964	TCAGGTCCTGCATCAGCTTG	60.036	150
TRINITY_Contig8237	4	p2	(TC)8	16	935	950	CCAGAGAGTTCTTGCAGCCA	59.964	TCAGGTCCTGCATCAGCTTG	60.036	150
TRINITY_Contig8238	1	p4	(CCTC)3	12	25	36	TTTCCTCCTGCATCGTGCTT	59.963	ACAAAACCCTTGTCCCCGTT	60.033	215
TRINITY_Contig8238	2	p2	(AG)7	14	1791	1804	TTTCCTCCTGCATCGTGCTT	59.963	ACAAAACCCTTGTCCCCGTT	60.033	215
TRINITY_Contig8239	1	p4	(GGAG)3	12	3041	3052	ACTTCCGATTTAATGCGCGC	59.972	AAACGGCATTGTCGGGTTTG	59.969	266
TRINITY_Contig8245	1	p3	(CGC)7	21	341	361	GCGTTTCATTGAGGAGCGAC	59.905	GGTGCTTGGGTCAATTGCTG	60.038	103
TRINITY_Contig8252	1	p3	(TTC)4	12	1137	1148	CGTCCCCCTTCTTCTTGGAC	60.036	TCCATGAAGATAGACGGCGC	59.968	235
TRINITY_Contig8252	2	c	(CTCCTT)3ttct(TTCTCC)4ttttct(TTCTCC)3ttgtggtccttcttctctgtcttcctcctccttctctgtttatctttt(TCCTTC)7	163	2598	2760	CGTCCCCCTTCTTCTTGGAC	60.036	TCCATGAAGATAGACGGCGC	59.968	235
TRINITY_Contig8252	3	p6	(GCCGGC)3	18	3055	3072	CGTCCCCCTTCTTCTTGGAC	60.036	TCCATGAAGATAGACGGCGC	59.968	235
TRINITY_Contig8252	4	p3	(GGC)4	12	3184	3195	CGTCCCCCTTCTTCTTGGAC	60.036	TCCATGAAGATAGACGGCGC	59.968	235
TRINITY_Contig8257	1	p3	(GAA)16	48	551	598	CAAACACCATTTCAGCGCCA	59.968	AGGCACCTCATCTCCTTCCT	59.956	217
TRINITY_Contig8258	1	p3	(GGC)6	18	123	140	GAGGAGAGGGAGACAGAGCA	60.034	CCTCGATCTGGATGCTCCAC	59.967	210
TRINITY_Contig8265	1	p2	(CT)26	52	1	52	CCGCTGAATCTTCCACCAGT	60.036	GCCTAGAAGCTGCACTCCAA	60.036	173
TRINITY_Contig8265	2	p3	(TGT)4	12	2220	2231	CCGCTGAATCTTCCACCAGT	60.036	GCCTAGAAGCTGCACTCCAA	60.036	173
TRINITY_Contig8266	1	p3	(GAC)4	12	116	127	TGGAGAAAGGCAGTCGACAC	59.966	TGCTTCCGCAGTTGTCATCT	59.965	100
TRINITY_Contig8267	1	p4	(TACA)3	12	2244	2255	TTTCCCAGGAATCGGAGTGC	60.035	TACCGATCTGCCTTCCAGGA	60.033	141
TRINITY_Contig8268	1	p3	(GCG)4	12	156	167	CTGCCATACGCGATTGGTTG	59.972	TTTCTTTGCAAGCTCCGCAC	59.969	113
TRINITY_Contig8269	1	p3	(CGG)4	12	308	319	GCAGGTCCTCCTTCTCGAAC	60.109	TCCATGTCGTCGTTCTCGTC	59.833	175
TRINITY_Contig8269	2	p6	(GCGACC)3	18	427	444	GCAGGTCCTCCTTCTCGAAC	60.109	TCCATGTCGTCGTTCTCGTC	59.833	175
TRINITY_Contig8270	1	p3	(GAG)4	12	2385	2396	TCCAGCTCCAGTTCCGTTTC	59.965	GCTGCTGATGGAGTGGAACT	60.036	119
TRINITY_Contig8271	1	p3	(TGC)4	12	2530	2541	GACGGTAGGTTTGGCTGTGA	59.966	TTAGCTGGCGATGATCCGTC	59.968	209
TRINITY_Contig8273	1	p5	(TGCGT)3	15	27	41	CGGACAAACTCGTCACTGGA	59.968	CTTGCCAATCTCTGCTGGGA	60.035	146
TRINITY_Contig8274	1	p4	(TGTA)3	12	1869	1880	CTCCCCCAGTTCACAGTCAC	59.965	CGTGTGCGAATCGATCAACC	59.975	209
TRINITY_Contig8275	1	p3	(GCT)5	15	1716	1730	CCCTTCACCACCTTCTGACC	59.963	CTTTTTCAGCAGCTGCAGCA	59.968	220
TRINITY_Contig8277	1	p3	(GGC)5	15	2509	2523	TTCACTCTTGTCCGCACTCC	59.966	GGAGATGAGGCCACGTTTGA	60.036	259
TRINITY_Contig8280	1	p6	(GGCAGC)3	18	142	159	TGGAGACATTCGAGATGCCG	59.897	TCGATGTCGTGGAACGTGTT	59.969	163
TRINITY_Contig8280	2	p2	(CT)22	44	270	313	TGGAGACATTCGAGATGCCG	59.897	TCGATGTCGTGGAACGTGTT	59.969	163
TRINITY_Contig8280	3	c	(GC)6acccgccattgaagctagcgcgagagcggcggagaggcgaggagtc(GGCG)3	70	452	521	TGGAGACATTCGAGATGCCG	59.897	TCGATGTCGTGGAACGTGTT	59.969	163
TRINITY_Contig8280	4	p5	(ATTTT)3	15	2333	2347	TGGAGACATTCGAGATGCCG	59.897	TCGATGTCGTGGAACGTGTT	59.969	163
TRINITY_Contig8281	1	p3	(GCT)4	12	1146	1157	GATCGCCGTCTTCGACATCT	59.97	AACACGTAGGATGTGGCGTT	59.966	174
TRINITY_Contig8282	1	p3	(CCG)4	12	260	271	ACCACACCTCAACAGCACAA	60.035	TGGCGACGGTATGATGATGG	59.967	190
TRINITY_Contig8282	2	p5	(TGGAG)3	15	1158	1172	ACCACACCTCAACAGCACAA	60.035	TGGCGACGGTATGATGATGG	59.967	190
TRINITY_Contig8283	1	p3	(CCG)4	12	491	502	TTTTGGTGTGTGGAGGGCTT	60.033	CCAGTCCTCACTCGTGTTCC	60.038	108
TRINITY_Contig8285	1	p3	(CCG)4	12	694	705	TTTTCTGCAGCAAAGACGCC	59.969	TTAGACCTTCTCCTCCGGGG	60.032	119
TRINITY_Contig8285	2	p4	(GCCG)3	12	933	944	TTTTCTGCAGCAAAGACGCC	59.969	TTAGACCTTCTCCTCCGGGG	60.032	119
TRINITY_Contig8289	1	c	(GCC)5accgccgaagccgccggcgcgttccccgcctccaccgcctcgtcggactccgactcgaaga(CCT)4	89	74	162	GTTCTTGGAGCTGGCCTGAT	60.035	GATGGCTCGTATGGTGAGGG	59.966	232
TRINITY_Contig8289	2	p3	(GGC)4	12	595	606	GTTCTTGGAGCTGGCCTGAT	60.035	GATGGCTCGTATGGTGAGGG	59.966	232
TRINITY_Contig8293	1	p3	(CCG)6	18	60	77	GATGGAGTAGAGGACGCAGC	59.969	GAGCTCGGCTAATCGCTTCT	59.969	162
TRINITY_Contig8296	1	p3	(CTG)4	12	554	565	CGCAGCCGAGAACGTATAGT	59.971	CAGTGTGCAAGGCTGTTGTC	59.97	254
TRINITY_Contig8296	2	p5	(CTGGA)3	15	1198	1212	CGCAGCCGAGAACGTATAGT	59.971	CAGTGTGCAAGGCTGTTGTC	59.97	254
TRINITY_Contig8296	3	c	(GA)6(GGGA)4	28	1316	1343	CGCAGCCGAGAACGTATAGT	59.971	CAGTGTGCAAGGCTGTTGTC	59.97	254
TRINITY_Contig8297	1	p3	(CCG)4	12	73	84	GCATCAACTAAGAAGCGCGG	59.973	AGGGCCACTTTTCCACCAAA	60.032	128
TRINITY_Contig8298	1	p4	(GGGT)3	12	1991	2002	GATCCCGGATGCTGAAGAGG	59.965	CCTTTGTGGTTTGAAGGCGG	59.968	267
TRINITY_Contig8301	1	p3	(GCC)4	12	75	86	CTTTGCAACACCACACCTCG	59.971	GAAGGAGCGGTGGTTTCTCA	59.965	164
TRINITY_Contig8302	1	p3	(GGC)4	12	243	254	CCGCCTCCAAGAAGTTCCAT	60.035	GCAGTTCTTGTAGTCGCCCT	60.037	101
TRINITY_Contig8302	2	p3	(CCT)5	15	593	607	CCGCCTCCAAGAAGTTCCAT	60.035	GCAGTTCTTGTAGTCGCCCT	60.037	101
TRINITY_Contig8303	1	p3	(CGG)4	12	1386	1397	CTGCTCAGACCGTCAGAAGG	60.109	CTCCCTCGCCTCCACAAATT	60.035	125
TRINITY_Contig8304	1	p3	(GCG)6	18	3869	3886	CTCAGGCCACCCGAATTCTT	60.035	TGCAGCATCTCATAGCCGAG	59.967	251
TRINITY_Contig8306	1	p3	(CCT)4	12	2494	2505	AGAGATCCGACCCCATTCCA	60.031	CCTCGGTGGATCTTCGATGG	59.967	194
TRINITY_Contig8308	1	p5	(GCACT)3	15	189	203	CTTCTTCCTCGCGCAATTCG	59.974	AGCCCAGAAAGAACAGCTCC	59.963	193
TRINITY_Contig8308	2	p3	(CGA)4	12	397	408	CTTCTTCCTCGCGCAATTCG	59.974	AGCCCAGAAAGAACAGCTCC	59.963	193
TRINITY_Contig8309	1	p3	(CGC)4	12	485	496	TGTATCCATGGGTTGCGGAG	59.82	CTAGGAGCGCCGAAGAAGAG	59.97	173
TRINITY_Contig8310	1	c	(ATGGA)3gatggcaatgctcaactcaaatgctgcactctgc(ATCT)3	61	141	201	AGCACAACCTGAGTGACCTG	59.893	GGAACGGATCAACGACCTGT	60.038	249
TRINITY_Contig8310	2	p3	(TGG)4	12	648	659	AGCACAACCTGAGTGACCTG	59.893	GGAACGGATCAACGACCTGT	60.038	249
TRINITY_Contig8311	1	p6	(GAAGAT)4	24	1815	1838	TCCTGCGAAGTTGCTCTCAG	60.038	CTAGTCCATCGTCGGAGCAC	59.97	239
TRINITY_Contig8312	1	p2	(AG)7	14	110	123	CCGTCATGGCCAAAGCAAAA	59.967	ACATTGGGTGAGGTAGCAGC	60.035	187
TRINITY_Contig8312	2	c	(AG)10ggacagatagagagccggggcgaagagccaccgatgtgttcttgaggagcctgtgcaggggaggtccaatccagaggccgcca(TTCC)3	115	277	391	CCGTCATGGCCAAAGCAAAA	59.967	ACATTGGGTGAGGTAGCAGC	60.035	187
TRINITY_Contig8316	1	p3	(CCT)5	15	1876	1890	TTGATGCAACGACGCCTACT	60.038	TGCACACACAGCAATGCTTC	59.969	185
TRINITY_Contig8317	1	c	(GGC)5gacctcggccggtgt(GGCGC)3	45	82	126	ACTTCCAAGTGCGAGCTACC	60.037	TCTGGCTAGGGGCATACACT	60.031	213
TRINITY_Contig8319	1	c	(TCC)4tcggcgatgtccacctcgaggggcctgttggcgcgcgccgacacgagcttcactgacctggaccggcgcgaattcatcaacgccgcggatca(GCCGC)3	120	1229	1348	TACAACATTCTCCCCGCCAC	60.036	ATCCACCCCTCCCACTACTC	60.031	213
TRINITY_Contig8320	1	p3	(GAC)4	12	982	993	AGTCCGTGAAGTTGAGGCTG	59.966	CTCTTCGACGTCAAGGGCAT	60.109	208
TRINITY_Contig8320	2	p3	(AGC)5	15	1214	1228	AGTCCGTGAAGTTGAGGCTG	59.966	CTCTTCGACGTCAAGGGCAT	60.109	208
TRINITY_Contig8321	1	c	(CCG)4cccgcggcgcccgtccatgcgctcctcccctcagaattg(ACCCC)3acccgatccgcgcgcaggcccctccaccatggatcgcaccatcagcg(GCA)4gcggaggcagcgccaccacggcggccagccagaacccgcacctcggcgtcaacaagctcggccgcagcatccgcaaggcca(CGC)5cctc(GCA)6ggccg(CCGC)3cgcagccccagccgcaggtctacaacatcagcaagaaccagttccgcgacatcgtgcagcagctcaccgccggcaacccgtcc(CCG)4	355	58	412	CTCAGAATTGACCCCACCCC	60.034	ATGTCGCGGAACTGGTTCTT	59.965	271
TRINITY_Contig8321	2	p3	(CCG)4	12	611	622	CTCAGAATTGACCCCACCCC	60.034	ATGTCGCGGAACTGGTTCTT	59.965	271
TRINITY_Contig8322	1	c	(TGC)4(TGG)4tgcggggccgcgtcccccatgatcgatccgaggaactgctggtcgtcaaaaccccctccgcgtggcgcggcggcgggcg(CTC)5ctgcttgggcgccgccgcggccgggagctgcacgtgcaggtgcggcgcgggggcgcccagcctcgcgccgcggccggcgcg(GGC)5	216	5602	5817	ATCAACACCAACCTTTGCGC	59.968	GGGATTGTGGGTGGCACATA	60.034	203
TRINITY_Contig8322	2	p4	(GAGG)3	12	6012	6023	ATCAACACCAACCTTTGCGC	59.968	GGGATTGTGGGTGGCACATA	60.034	203
TRINITY_Contig8322	3	c	(GCGGC)3gcggtgcgt(GCCA)3cccg(GCGAC)4aggagcaagagagaa(GCAG)3	87	6204	6290	ATCAACACCAACCTTTGCGC	59.968	GGGATTGTGGGTGGCACATA	60.034	203
TRINITY_Contig8326	1	p3	(CGC)4	12	409	420	CGTCAAGCCACCCCAAAAAG	59.968	TCCTCCGAGTTTGATGGCAC	60.036	209
TRINITY_Contig8326	2	p3	(GCC)6	18	572	589	CGTCAAGCCACCCCAAAAAG	59.968	TCCTCCGAGTTTGATGGCAC	60.036	209
TRINITY_Contig8326	3	p3	(CGG)4	12	911	922	CGTCAAGCCACCCCAAAAAG	59.968	TCCTCCGAGTTTGATGGCAC	60.036	209
TRINITY_Contig8327	1	p4	(GACG)3	12	394	405	TGGAGATCCAAAGGCCCAAC	59.96	TTTGTTCGGCAGATGCTTGC	60.039	168
TRINITY_Contig8329	1	p3	(TCG)5	15	1338	1352	GGTCCTGGTAGATCATGGCG	59.966	CGACCGGTTCTATCTCCGTG	59.971	197
TRINITY_Contig8330	1	p6	(CGGCGC)4	24	2265	2288	CCTCTGGTGATTCTGCGGTT	60.036	CACATCGGCGCGTTTCTAAG	59.975	202
TRINITY_Contig8331	1	c	(CCTCCA)3(CCT)4	30	115	144	ATTGCAACAAACAGGGACGC	59.968	CTCCGTTTTGGCCAAACAGG	59.968	232
TRINITY_Contig8332	1	p2	(CG)6	12	566	577	CACCAACGGGCAAACACAAT	59.896	GGAGAAGGTGTACGAGCTCG	59.901	131
TRINITY_Contig8333	1	p3	(GAA)6	18	1282	1299	AACAGTCCGCCCCTCTCTAT	60.032	CTAGAAGCCCGCAACTGGAA	60.036	171
TRINITY_Contig8335	1	p5	(CCGCC)3	15	1877	1891	TGGAAGTTCTTCACCGAGGC	59.965	CTCGTATCTCGGGCATTGCT	59.967	169
TRINITY_Contig8337	1	p3	(CCG)4	12	1821	1832	GTGACAGTAGAGATCGGCGG	59.97	TCAACTTTGGAGGCGGAGTC	59.965	280

TRINITY_Contig8337	2	p4	(CGGG)3	12	2116	2127	GTGACAGTAGAGATCGGCGG	59.97	TCAACTTTGGAGGCGGAGTC	59.965	280
TRINITY_Contig8339	1	p3	(GCA)5	15	316	330	TGCGTCTTCAGCAAAACTGC	59.971	CTTTCCTCCCAATGACCGCT	60.035	219
TRINITY_Contig8340	1	p3	(GAG)5	15	123	137	AAGCTCTTGAGCGTGCTGAT	60.037	AGCTGGCTCCCTATCCAAGA	60.031	270
TRINITY_Contig8341	1	p5	(CTACA)3	15	65	79	GTTGTTCAGCCGCTGTGAAG	60.041	TTCGACGCCATGGAAAGGAA	59.964	202
TRINITY_Contig8341	2	p6	(GGGCGC)4	24	714	737	GTTGTTCAGCCGCTGTGAAG	60.041	TTCGACGCCATGGAAAGGAA	59.964	202
TRINITY_Contig8341	3	p4	(GGAA)3	12	1088	1099	GTTGTTCAGCCGCTGTGAAG	60.041	TTCGACGCCATGGAAAGGAA	59.964	202
TRINITY_Contig8342	1	p5	(GCGGT)3	15	771	785	GCCCGAAATTCCTCCCTGAA	60.034	CTGTTCTTCGTGGTCGACCA	59.968	235
TRINITY_Contig8343	1	p3	(GGC)5	15	1181	1195	ACCTGCTGTGATCCCATTGG	60.034	GGATTAGAGTACGCGACGCA	59.971	134
TRINITY_Contig8343	2	p3	(GAG)5	15	1386	1400	ACCTGCTGTGATCCCATTGG	60.034	GGATTAGAGTACGCGACGCA	59.971	134
TRINITY_Contig8343	3	p4	(CTCG)3	12	1551	1562	ACCTGCTGTGATCCCATTGG	60.034	GGATTAGAGTACGCGACGCA	59.971	134
TRINITY_Contig8343	4	p3	(CCG)4	12	1796	1807	ACCTGCTGTGATCCCATTGG	60.034	GGATTAGAGTACGCGACGCA	59.971	134
TRINITY_Contig8343	5	p4	(ACAT)4	16	1958	1973	ACCTGCTGTGATCCCATTGG	60.034	GGATTAGAGTACGCGACGCA	59.971	134
TRINITY_Contig8343	6	c	(GGCC)3ggcggtgacgtggagagagagagt(GA)7	50	2753	2802	ACCTGCTGTGATCCCATTGG	60.034	GGATTAGAGTACGCGACGCA	59.971	134
TRINITY_Contig8343	7	p4	(GGCC)4	16	2942	2957	ACCTGCTGTGATCCCATTGG	60.034	GGATTAGAGTACGCGACGCA	59.971	134
TRINITY_Contig8343	8	c	(GCCG)3gcgaccgcgatggcgacagcgcgatcgctcgccgcgctcccctacgccagagccacgcgccccgggcctcgccgctccgcctggccgccgcgcgtcgt(CGC)5	125	3441	3565	ACCTGCTGTGATCCCATTGG	60.034	GGATTAGAGTACGCGACGCA	59.971	134
TRINITY_Contig8344	1	p6	(GCCATC)3	18	2045	2062	CAAACTTCCCACCACGCTTG	59.969	GCGCCTTTGGTGTGCTAAAA	59.969	240
TRINITY_Contig8345	1	p3	(CGG)4	12	345	356	GCCACACCGGTAAACAGAGA	59.966	GCTGTTGCCTGTTGGTTCTG	59.969	131
TRINITY_Contig8346	1	p4	(TTTA)3	12	282	293	GGATGCGACGAAGCTATGGA	59.968	GGTCTGGTCGTTGAAGGGAG	60.037	168
TRINITY_Contig8346	2	p4	(AAGA)3	12	941	952	GGATGCGACGAAGCTATGGA	59.968	GGTCTGGTCGTTGAAGGGAG	60.037	168
TRINITY_Contig8348	1	c	(CGG)4caacaacaaga(AGC)5	38	474	511	AGCAGTTCCGGAAGCTCTTC	60.037	CTTGCACATGTTGACGCACA	59.971	209
TRINITY_Contig8349	1	p3	(TGA)4	12	956	967	ACAGAAGGGCCCATCCAATG	60.033	TCTGCTCATGGGCTCTTGTG	60.036	134
TRINITY_Contig8350	1	p5	(TTTGT)3	15	1096	1110	CCGACGACATGGCTAGTCTC	59.97	GAGAACCAACGACAGGAGCA	59.966	115
TRINITY_Contig8351	1	p3	(AAG)5	15	12	26	GGAAGCTTCCCTTGTTCGGA	59.964	ACGGTTGTTGTCGAATCCGA	59.968	116
TRINITY_Contig8352	1	p3	(TCC)6	18	667	684	CTTGGTTGGTCTGCTCGGAT	60.036	ATCTCCCCTTCCCAATCCGA	60.03	113
TRINITY_Contig8353	1	p4	(TCTT)3	12	10	21	AAGTCTGAACTCGCAAGCGA	59.968	CATGGTGAACGCCTTCTCCT	60.036	108
TRINITY_Contig8353	2	p3	(CTC)4	12	588	599	AAGTCTGAACTCGCAAGCGA	59.968	CATGGTGAACGCCTTCTCCT	60.036	108
TRINITY_Contig8353	3	p3	(GAG)12	36	781	816	AAGTCTGAACTCGCAAGCGA	59.968	CATGGTGAACGCCTTCTCCT	60.036	108
TRINITY_Contig8356	1	c	(GA)9g(GA)6	31	60	90	GGTCGCCAGGTGTAAAAGGA	59.964	TCCACCCCAGGATTACCCAT	59.953	141
TRINITY_Contig8356	2	p3	(GCT)4	12	893	904	GGTCGCCAGGTGTAAAAGGA	59.964	TCCACCCCAGGATTACCCAT	59.953	141
TRINITY_Contig8359	1	p3	(CGC)4	12	193	204	AAGAACCTCAGAGTGTGCGG	59.966	GGAGCAGCGACTCCTGTATC	59.969	270
TRINITY_Contig8361	1	c	(CCT)4cgtcgcccgaggccaccgcctcctcctcggcctccccaacctcctcgtcgaaggcctctcccgcca(CCT)5	93	1003	1095	CCGGCTTGCAAATGGTTTCA	59.967	AACAGAGGAGCAATGGGACG	60.036	251
TRINITY_Contig8362	1	p3	(GAC)4	12	53	64	AGGGTGAATCTGATCGGGGA	60.031	CAGACGGGGTACTTGTGGTC	60.038	146
TRINITY_Contig8362	2	p4	(TCCT)3	12	1825	1836	AGGGTGAATCTGATCGGGGA	60.031	CAGACGGGGTACTTGTGGTC	60.038	146
TRINITY_Contig8364	1	p3	(AGC)4	12	2157	2168	AAAGGGAGGCTGCAGCTAAG	60.035	GGATGCTCGTGATGCCCATA	59.965	108
TRINITY_Contig8364	2	p6	(CCTCCG)3	18	2488	2505	AAAGGGAGGCTGCAGCTAAG	60.035	GGATGCTCGTGATGCCCATA	59.965	108
TRINITY_Contig8367	1	c	(TCT)6tc(AG)9	38	616	653	GTGACTTGGACCGGGTACTG	60.038	TTAACATTCTGACCCGGCCC	60.035	127
TRINITY_Contig8367	2	p4	(GGGA)3	12	2080	2091	GTGACTTGGACCGGGTACTG	60.038	TTAACATTCTGACCCGGCCC	60.035	127
TRINITY_Contig8372	1	p3	(AGC)4	12	953	964	CAGCAAGAGCAAGCAAGCAA	59.968	GCAAGCGCCCTTATTCATGG	59.969	240
TRINITY_Contig8372	2	p4	(AGCA)3	12	1647	1658	CAGCAAGAGCAAGCAAGCAA	59.968	GCAAGCGCCCTTATTCATGG	59.969	240
TRINITY_Contig8372	3	p3	(GCA)4	12	2047	2058	CAGCAAGAGCAAGCAAGCAA	59.968	GCAAGCGCCCTTATTCATGG	59.969	240
TRINITY_Contig8379	1	c	(CCCCT)3cctctcgcc(CGCT)4	40	115	154	TTAGCTGTTCGTCCCGTGTC	60.039	TGTACTGGGAACTGGACCCT	59.807	172
TRINITY_Contig8380	1	p3	(GCA)5	15	321	335	GAGTTGCTCGAGGGGGAAAA	59.964	GTTTCCCTCTCTCGAGCTCG	59.9	181
TRINITY_Contig8382	1	p3	(CGA)5	15	1009	1023	CCGAGGCTACACGTCAGATC	59.97	CCCGTGTGCTACTGTGCTAA	60.038	166
TRINITY_Contig8384	1	c	(CG)6(CTCC)3	24	187	210	AGATGGTGTCCAAGGTGCTG	59.963	ATAGCCCACTGTTGCCATCC	60.107	194
TRINITY_Contig8384	2	p3	(CGG)4	12	654	665	AGATGGTGTCCAAGGTGCTG	59.963	ATAGCCCACTGTTGCCATCC	60.107	194
TRINITY_Contig8385	1	p3	(GGC)7	21	1818	1838	GCCATAGCGATACCCTTGCT	59.965	AAGGGATGCCAATGAAGGGG	60.032	194
TRINITY_Contig8387	1	p5	(TGATT)3	15	1831	1845	ATATCTCTGTGCTGCCTCGC	59.967	TATCGCCATCATCACCACCG	59.967	211
TRINITY_Contig8388	1	p2	(AG)15	30	134	163	GAAGGCATTGCACCACAAGG	60.038	TCCTGGCTAGCCTTCAGACT	59.958	122
TRINITY_Contig8392	1	p3	(AGC)4	12	587	598	GAAGAGCAGACCCCCAAGAC	60.036	ATCAACGGCGTGTTCTTCCT	59.965	206
TRINITY_Contig8393	1	p4	(CGGG)3	12	1415	1426	TGCAATCCATCAGCAGCTCA	60.035	AGCGGATTGAGGATATGCCG	60.037	109
TRINITY_Contig8393	2	c	(GTTG)5gctggctccgctacgctacgcttcgtctttctggttggc(CGCCG)3	74	1533	1606	TGCAATCCATCAGCAGCTCA	60.035	AGCGGATTGAGGATATGCCG	60.037	109
TRINITY_Contig8394	1	p4	(CTTT)3	12	1263	1274	GCTGTTCACGTGTCCTCTCA	59.968	GGGTCTGGAGAAGCTGCATT	60.035	130
TRINITY_Contig8395	1	p6	(CCTCTC)3	18	68	85	ATGGGCAGCACGGATTCC	60.124	AGCCCTCAGAGCTAAGGTCA	59.958	249
TRINITY_Contig8395	2	p3	(GGC)4	12	264	275	ATGGGCAGCACGGATTCC	60.124	AGCCCTCAGAGCTAAGGTCA	59.958	249
TRINITY_Contig8395	3	p3	(CGG)5	15	454	468	ATGGGCAGCACGGATTCC	60.124	AGCCCTCAGAGCTAAGGTCA	59.958	249
TRINITY_Contig8396	1	p3	(CGG)4	12	908	919	ACGAACCAAAACAGCAAGCC	59.898	ATCAATTTGGCTTGGCTGCG	60.109	174
TRINITY_Contig8396	2	p3	(GGA)6	18	1197	1214	ACGAACCAAAACAGCAAGCC	59.898	ATCAATTTGGCTTGGCTGCG	60.109	174
TRINITY_Contig8397	1	p4	(CCTG)4	16	1375	1390	TATCATTCGGGTCTGCTCGC	59.968	TCCCCACTGGCAAAACTTGT	60.033	224
TRINITY_Contig8398	1	c	(GCG)4agg(GGA)5	30	312	341	GTGTCACTGCAGAGACGACA	59.969	AGAACAGGGCTTGCTTCGAA	59.891	124
TRINITY_Contig8404	1	p3	(CGC)5	15	213	227	CCGTCCTATCCGTTCTTGGG	59.896	GCCGAGAAACCTGACCAGAA	59.965	274
TRINITY_Contig8405	1	p3	(CAG)5	15	839	853	GCTGTTGCCCCAAGGACTAT	60.034	TCATCAGCACCACCCATGTC	60.035	203
TRINITY_Contig8407	1	p3	(GCG)5	15	821	835	ATCCGCTGGAGGTAGACGTA	59.818	CACCTACTACTACGGCTGCG	59.971	114
TRINITY_Contig8407	2	p3	(CCA)4	12	987	998	ATCCGCTGGAGGTAGACGTA	59.818	CACCTACTACTACGGCTGCG	59.971	114
TRINITY_Contig8409	1	c	(CGG)4cctcggcggcgccccgtcctcccgcgcctgcacgaccatcccgctcgggcgcacctcccagccggcccggtcctccgcggccgccgcga(CCG)4	113	899	1011	TTGGAGACCTTGAGCACGTC	59.966	CAGATCACGACGCTGATCGA	59.972	149
TRINITY_Contig8409	2	c	(GGAA)4gcctctgcgtgcg(GCGA)4gggagggggcaattgatgcggggtgcgcgcggttggtgcagtgccgtggagtgtga(GTG)4	113	1130	1242	TTGGAGACCTTGAGCACGTC	59.966	CAGATCACGACGCTGATCGA	59.972	149
TRINITY_Contig8410	1	c	(GAGG)3gataggctggggagccttacttccagacgtcgtgctcgcgct(CGG)6	72	1154	1225	CCAATCTCGCAATTCCGCTG	59.971	GCCAAACTTCGCTCCGTTTT	59.97	272
TRINITY_Contig8410	2	c	(GCC)4gctcgcttcggtttcctttccccgctggccctgggctgcctgctggctt(TGC)4	73	1581	1653	CCAATCTCGCAATTCCGCTG	59.971	GCCAAACTTCGCTCCGTTTT	59.97	272
TRINITY_Contig8411	1	c	(TAG)4tacatgcagtacatgtcatcagccaatgagctgacatcgtcagccgcatgggtgaagctcaagtcct(GGA)4	91	220	310	AGATCTTGTCCCTGCCGTTG	60.036	ACCGGCTGATTTTTCCGCTA	60.036	254
TRINITY_Contig8413	1	p6	(CAACGC)3	18	235	252	TTCGACGATGCCACTGACAA	59.967	TTGCGCTGTCCCTTGATGAT	60.035	158
TRINITY_Contig8414	1	p3	(GAG)4	12	319	330	CAGCATGGAAGGAGATGGCA	60.107	GGGCAGCACATCTCACTCTT	60.036	176
TRINITY_Contig8415	1	p3	(ACG)4	12	259	270	CGCCAGCGATACTTGGATCT	59.967	AGGTCGTTGTGGTACGTCAC	59.969	188
TRINITY_Contig8421	1	p3	(GCC)4	12	804	815	AGTTGGAGTGGGCGTACTTG	59.965	AGAAGGTGAAGCCGCTGTAC	60.037	225
TRINITY_Contig8423	1	p3	(TCC)4	12	242	253	CCCAACCGCAATCCTCATCT	60.107	TGAGGAGGAGGAGGACGAAG	60.034	247
TRINITY_Contig8423	2	p3	(GCG)4	12	627	638	CCCAACCGCAATCCTCATCT	60.107	TGAGGAGGAGGAGGACGAAG	60.034	247
TRINITY_Contig8424	1	p6	(GAGGCG)4	24	692	715	GATGCGTTCGGGGATTAGGT	59.894	CATTTTACACCCCCTCCCCC	60.033	204
TRINITY_Contig8424	2	p5	(AGGGA)3	15	1193	1207	GATGCGTTCGGGGATTAGGT	59.894	CATTTTACACCCCCTCCCCC	60.033	204
TRINITY_Contig8425	1	p3	(CGC)4	12	292	303	TCGCAATTTTGTGGCTGGTG	59.968	TCCCCTTCAAGCATGCCATT	59.958	250
TRINITY_Contig8429	1	p3	(GCG)5	15	796	810	GAGACGACGACGAGGATGTC	59.973	TCCTTCTCCTCCGTCTCCAG	60.034	209
TRINITY_Contig8429	2	p3	(GAG)4	12	1011	1022	GAGACGACGACGAGGATGTC	59.973	TCCTTCTCCTCCGTCTCCAG	60.034	209
TRINITY_Contig8430	1	c	(AAG)4aaaaaaaagctcacgcgccgaagaaacacgcaaccctctcctgcccccggaaaaacc(ATCG)4	85	375	459	GTAGTAGTACCAGGCGCACC	59.898	AGGCCACAGTAAGGAATGGC	60.034	241
TRINITY_Contig8431	1	p4	(GAGC)3	12	61	72	GGCCTGCTCCTTCTCTCTCT	60.687	TTTGGTTCCACACTGGCACT	60.034	118
TRINITY_Contig8431	2	p2	(TC)16	32	805	836	GGCCTGCTCCTTCTCTCTCT	60.687	TTTGGTTCCACACTGGCACT	60.034	118
TRINITY_Contig8432	1	p3	(GGA)5	15	330	344	GCAGGTCATCAGTGTGAGCT	60.037	AGCTGTGCAATCCGACAAGA	59.965	224
TRINITY_Contig8432	2	p3	(GGA)4	12	446	457	GCAGGTCATCAGTGTGAGCT	60.037	AGCTGTGCAATCCGACAAGA	59.965	224
TRINITY_Contig8435	1	p3	(CGG)7	21	67	87	ATCGTCATCGTCGGAAGGTG	59.899	GCAAATCGGGAGACAGGGAA	60.035	205
TRINITY_Contig8437	1	p6	(CGCTGT)3	18	624	641	ACAAGTCTACCGGTGCATGG	60.036	TGGAGGAAGCAAGCTCCAAG	59.963	276
TRINITY_Contig8437	2	p3	(GGA)4	12	1337	1348	ACAAGTCTACCGGTGCATGG	60.036	TGGAGGAAGCAAGCTCCAAG	59.963	276
TRINITY_Contig8437	3	c	(GCC)4gtcgtggaggagcggtgcgtcgcggacgggaacgagcgcctgcgcttccactgctccaccatgctctgctccctg(GGC)5	102	1866	1967	ACAAGTCTACCGGTGCATGG	60.036	TGGAGGAAGCAAGCTCCAAG	59.963	276
TRINITY_Contig8441	1	p3	(GCT)5	15	294	308	GGCAGCTTCTCCCTTTCCTT	59.961	TTCCTGCAACCGTACTGACC	59.966	113
TRINITY_Contig8441	2	p6	(CGCCGG)3	18	444	461	GGCAGCTTCTCCCTTTCCTT	59.961	TTCCTGCAACCGTACTGACC	59.966	113
TRINITY_Contig8444	1	p3	(CGG)4	12	465	476	AGGTCGTCTCCAAAGCCAAG	59.965	GGTCCTCGCGATCAGAAACA	60.109	244
TRINITY_Contig8444	2	p2	(CG)7	14	652	665	AGGTCGTCTCCAAAGCCAAG	59.965	GGTCCTCGCGATCAGAAACA	60.109	244
TRINITY_Contig8448	1	p6	(CCCCCG)3	18	1347	1364	ACGTTTCGCTTTGTCACTGC	59.972	ACCGCTCTTTGAGGCTTACC	60.036	149

TRINITY_Contig8448	2	c	(CTT)4cgaggacggctcggccgcatcagcgtctgggctggggtgcttgcgagggggcgtcgcgggcgg(AGC)4	87	1507	1593	ACGTTTCGCTTTGTCACTGC	59.972	ACCGCTCTTTGAGGCTTACC	60.036	149
TRINITY_Contig8448	3	p3	(CGG)5	15	1720	1734	ACGTTTCGCTTTGTCACTGC	59.972	ACCGCTCTTTGAGGCTTACC	60.036	149
TRINITY_Contig8449	1	p4	(TCCC)3	12	16	27	GGCGAGTTCGAATCGCTAGA	59.971	AAAAGCTCTTGCAGTGCACG	59.97	254
TRINITY_Contig8453	1	p3	(CGG)5	15	928	942	ACAGCTTCTCGAACACCTGG	59.966	TACATCCCTCCCCAATCCGT	60.031	156
TRINITY_Contig8454	1	c	(GGAG)4agcggcgagcgctgaaa(CTGC)3	45	69	113	TGAGAACATCGGTGGCCTTC	60.036	TCGTGATGCTTCCCTTTCCC	60.035	225
TRINITY_Contig8457	1	p3	(ATG)4	12	1806	1817	CAACACCTGTACCGACTGCT	59.966	CCAGGTGGTACTGACTGCTG	60.037	100
TRINITY_Contig8458	1	c	(CCG)5atccccgtccccggcacgccgagagatccccgccgcaccgagcgtggatgccgcgccacggccaccacgagccggccccgcccgc(CTG)4	112	166	277	CTCGAGCTCTGCTGAGTCTG	59.901	ATCAGCAGTCCTCCTCGACT	60.034	112
TRINITY_Contig8465	1	p3	(GGA)4	12	460	471	GAACCAACAGCAGTGCAAGG	59.969	CTGGCCCTGAGCTATCCATG	59.964	171
TRINITY_Contig8466	1	p3	(GCG)4	12	1089	1100	GATGGCGGACGTAGGAAGAG	59.969	CGCGGGAGGGTTTCATATCA	59.894	152
TRINITY_Contig8466	2	p3	(CGG)5	15	1285	1299	GATGGCGGACGTAGGAAGAG	59.969	CGCGGGAGGGTTTCATATCA	59.894	152
TRINITY_Contig8470	1	c	(CGC)5gccacctgccgcagttaaccgctccattaatctagacctctcctccttcctcctcctcggctcctcgcttctcc(TG)6	101	606	706	CTCGCTTCTCCTGTGTGTGT	59.967	GATCATCACAGGCTCCCTCG	59.967	146
TRINITY_Contig8471	1	p4	(CCCG)3	12	1554	1565	GAGGAGCTGACTTTGACCCC	60.036	ATCTGGCATTTGGCTGTGGA	59.96	254
TRINITY_Contig8474	1	p3	(GCC)4	12	257	268	AGATGAAGGTCTACCGCCCT	60.032	ACCAGCTGCTGCAACCATAT	60.034	218
TRINITY_Contig8475	1	p3	(CGC)4	12	843	854	CAGTTCAAGGGCGTGGTACT	59.965	AGGCACTGCAGCAGATTGAT	60.034	196
TRINITY_Contig8475	2	p2	(GC)7	14	1147	1160	CAGTTCAAGGGCGTGGTACT	59.965	AGGCACTGCAGCAGATTGAT	60.034	196
TRINITY_Contig8475	3	p3	(CGC)4	12	1668	1679	CAGTTCAAGGGCGTGGTACT	59.965	AGGCACTGCAGCAGATTGAT	60.034	196
TRINITY_Contig8475	4	p3	(CTC)5	15	1803	1817	CAGTTCAAGGGCGTGGTACT	59.965	AGGCACTGCAGCAGATTGAT	60.034	196
TRINITY_Contig8477	1	p3	(CCG)5	15	73	87	TGTGGAGAAGGGGGTTCAGA	60.105	GCACGGCATTTCATTCAGCA	60.109	114
TRINITY_Contig8478	1	c	(CGCC)3cccatgaagctgttcgccttcgtgcggcgcgcgcgccagt(CGC)4cgacgtcgaccgcgccggccgcggcgcccgaggacgccaccacggccgccgcgtccgcggagaagaa(GCG)4	143	333	475	AGTCTCTTGGATTGCTGCCC	60.035	GACAACAGCGAGAGGAACCA	59.966	194
TRINITY_Contig8479	1	p3	(CCG)4	12	3691	3702	CCAAAGCCACAGTTGCACTC	59.969	GCGGGAGTGTGAAGAGTTGA	59.966	170
TRINITY_Contig8482	1	p6	(CCAGGA)3	18	2129	2146	GCACTACAAGTCGGTGGTCA	59.967	GTAGGTAGACATGCCCTCGC	59.968	229
TRINITY_Contig8483	1	p3	(GGC)5	15	167	181	CGGAAGATCCTGGACATCCG	59.967	CTTCACGTCCAGGAACAGCT	59.966	210
TRINITY_Contig8484	1	c	(CTAG)3ctcgatccgcacgtcaaagaccttcgccgctagctttctcgtcaagcct(GCCGC)3agcgtca(TCG)5	98	125	222	CATTTCTACTGCGCGTGTCG	59.976	ACACGCTCCTCTCTTCTCCT	59.961	163
TRINITY_Contig8486	1	p3	(GAC)4	12	1178	1189	GATGGCGCAGATCGAGAAGA	59.969	TAGACGAAGGTGGCGAGGTA	60.035	174
TRINITY_Contig8488	1	c	(ACGC)3gggcggcaccaccggactgaaactgaaacgcactccagcgatccagtccagccgtccagccagcggcagcagt(GCGA)5	105	179	283	GACCATTATCCGGTCGGGTC	59.967	GTGGAGGATCGGTCGATGTC	59.969	194
TRINITY_Contig8490	1	c	(GCT)4gggagcaggacgacgagggggaggagcgatcggtgggagagacaggagcgctgc(TGCT)3ggtcgcgacgggggcggggctgccggcccgctgctctc(GCT)6	134	2243	2376	GTTCTGCTTGGTTGCCTTGG	59.967	GGAAAGAACACCAACGCCAC	59.97	114
TRINITY_Contig8490	2	p5	(GTGGC)3	15	2550	2564	GTTCTGCTTGGTTGCCTTGG	59.967	GGAAAGAACACCAACGCCAC	59.97	114
TRINITY_Contig8492	1	p4	(AACC)3	12	32	43	TTTGCCTGTTTTGCTCGCTC	59.969	CACATCCCAGTTCCCAGCTT	59.961	249
TRINITY_Contig8492	2	p6	(CCGACC)3	18	271	288	TTTGCCTGTTTTGCTCGCTC	59.969	CACATCCCAGTTCCCAGCTT	59.961	249
TRINITY_Contig8494	1	p3	(CGC)4	12	331	342	GAGAGTCGTGCGTACCGTAG	59.974	GAGGAAGACGAGATCTGCGG	59.97	122
TRINITY_Contig8494	2	p5	(GGCTT)4	20	2163	2182	GAGAGTCGTGCGTACCGTAG	59.974	GAGGAAGACGAGATCTGCGG	59.97	122
TRINITY_Contig8494	3	p3	(GCG)6	18	2320	2337	GAGAGTCGTGCGTACCGTAG	59.974	GAGGAAGACGAGATCTGCGG	59.97	122
TRINITY_Contig8494	4	p2	(AT)12	24	3319	3342	GAGAGTCGTGCGTACCGTAG	59.974	GAGGAAGACGAGATCTGCGG	59.97	122
TRINITY_Contig8495	1	p4	(GGCA)3	12	1288	1299	TGTGCTGAGAGCTTTCCCTG	59.964	TTTGCAAGCTGGACATTGGC	59.966	264
TRINITY_Contig8498	1	p3	(TCG)11	33	385	417	AAGGACCAGTGCAACAACCA	60.034	AGTCCACGTCACACTCGTTC	59.97	136
TRINITY_Contig8499	1	c	(TCGCA)3gcagctcgggcaccggtgatattatttcggcgagcgctgctgaaaaaatgcctcatgatatctctggtccgggcgcacgttctatctgtctgca(CTGCC)4	129	5541	5669	AACAGCAGATTCAGGGTCGG	60.036	GTGATTCCGCGTACGCTTTC	59.975	248
TRINITY_Contig8499	2	p3	(CGC)7	21	6458	6478	AACAGCAGATTCAGGGTCGG	60.036	GTGATTCCGCGTACGCTTTC	59.975	248
TRINITY_Contig8501	1	p3	(GCC)4	12	908	919	GATCCTGGAACGGATCGGAC	59.968	ACGACATCAAGGTCTCCTGC	59.752	153
TRINITY_Contig8502	1	p4	(CCCT)3	12	84	95	CACCAAACTGTGCTTCCAGC	59.969	TTGCAAAGCAACACGACTCG	59.973	278
TRINITY_Contig8502	2	p4	(TTAT)3	12	1860	1871	CACCAAACTGTGCTTCCAGC	59.969	TTGCAAAGCAACACGACTCG	59.973	278
TRINITY_Contig8503	1	p3	(GCA)4	12	63	74	TCGCACTGTCCTTCCACTTC	59.966	ATGGACATGCTGGGACCAAG	60.034	244
TRINITY_Contig8503	2	c	(GCT)8ccgccgccgccttgtcgccggcgaggccggggaggaggggctccgtggaca(TGGC)4cgcgcgacgtcgctggtgtggcttggggcgcctgtggcggcgcggtgg(GCGT)3	151	2146	2296	TCGCACTGTCCTTCCACTTC	59.966	ATGGACATGCTGGGACCAAG	60.034	244
TRINITY_Contig8506	1	p3	(CGG)8	24	66	89	GACTTCGCCTCTTTCGACCA	60.039	TCTCGCAATGACAGTGCCAT	60.036	180
TRINITY_Contig8506	2	c	(GGA)6attcgaggaggaggatgaggaggaactca(AGG)4	59	255	313	GACTTCGCCTCTTTCGACCA	60.039	TCTCGCAATGACAGTGCCAT	60.036	180
TRINITY_Contig8509	1	p3	(CTC)4	12	3272	3283	CACATCAAACACAGCGTGCA	59.971	GACTGGCAAGCAACCAAGTG	59.969	254
TRINITY_Contig8510	1	p3	(GCC)5	15	421	435	TGCCTTGCCAGAACGAGATT	59.963	CAGCGTGTTGTAGCTAGCGA	60.456	126
TRINITY_Contig8510	2	p3	(CGG)5	15	942	956	TGCCTTGCCAGAACGAGATT	59.963	CAGCGTGTTGTAGCTAGCGA	60.456	126
TRINITY_Contig8510	3	p3	(CGG)4	12	1444	1455	TGCCTTGCCAGAACGAGATT	59.963	CAGCGTGTTGTAGCTAGCGA	60.456	126
TRINITY_Contig8512	1	p3	(CCT)4	12	96	107	GTGTGTGTGTGTGTGTACGC	59.974	ACGGCAGCAGTTGAGTAGAC	60.038	153
TRINITY_Contig8512	2	p2	(TG)28	56	945	1000	GTGTGTGTGTGTGTGTACGC	59.974	ACGGCAGCAGTTGAGTAGAC	60.038	153
TRINITY_Contig8512	3	p3	(CCA)4	12	1425	1436	GTGTGTGTGTGTGTGTACGC	59.974	ACGGCAGCAGTTGAGTAGAC	60.038	153
TRINITY_Contig8513	1	p4	(GCTT)3	12	1027	1038	TCTTTGCGCACACCACAATG	59.969	TCTGGATTGCTCCTCTCGGA	60.033	162
TRINITY_Contig8513	2	p3	(CCA)4	12	1998	2009	TCTTTGCGCACACCACAATG	59.969	TCTGGATTGCTCCTCTCGGA	60.033	162
TRINITY_Contig8513	3	p6	(TTGGGA)3	18	2487	2504	TCTTTGCGCACACCACAATG	59.969	TCTGGATTGCTCCTCTCGGA	60.033	162
TRINITY_Contig8513	4	p3	(GGT)4	12	2630	2641	TCTTTGCGCACACCACAATG	59.969	TCTGGATTGCTCCTCTCGGA	60.033	162
TRINITY_Contig8516	1	p3	(CTT)4	12	944	955	CGGTGACCTTACCTCAACCC	60.037	GGGGCATCATTTGGGTCAGA	60.033	273
TRINITY_Contig8516	2	p3	(GCG)6	18	1272	1289	CGGTGACCTTACCTCAACCC	60.037	GGGGCATCATTTGGGTCAGA	60.033	273
TRINITY_Contig8517	1	p3	(CCG)7	21	2366	2386	TCCTTCAAGCGGGCTACAAG	60.036	TGAGAGAGGTTGCAAGGCTG	59.964	225
TRINITY_Contig8519	1	p2	(CT)16	32	1	32	CCGTAGCATCGAGGACTGTC	59.97	CTCTACCGCGTCTGTAGCAG	59.972	193
TRINITY_Contig8519	2	p3	(GCC)5	15	166	180	CCGTAGCATCGAGGACTGTC	59.97	CTCTACCGCGTCTGTAGCAG	59.972	193
TRINITY_Contig8519	3	p4	(CTCC)3	12	3475	3486	CCGTAGCATCGAGGACTGTC	59.97	CTCTACCGCGTCTGTAGCAG	59.972	193
TRINITY_Contig8520	1	p3	(CTT)4	12	467	478	CCGGGGCTTCTTCTTCTTGT	59.963	TCTTCTCCGCCTCTACTCCC	60.107	192
TRINITY_Contig8521	1	p6	(CGCGTG)3	18	74	91	TTTGGCGTGCTGTTCTTTCG	59.971	AGCAGGATCGTTGGAATGCA	60.035	212
TRINITY_Contig8521	2	c	(CGG)4gtgctgttgct(GCG)4(GAG)4	47	514	560	TTTGGCGTGCTGTTCTTTCG	59.971	AGCAGGATCGTTGGAATGCA	60.035	212
TRINITY_Contig8522	1	p3	(CGG)4	12	353	364	CAACCAGCTGACCAGTAGCA	59.965	CGAGGTCAGGTTCTGCAGTT	59.966	202
TRINITY_Contig8523	1	p3	(GCC)4	12	78	89	GAGAAACCCTTGCCCTCTCC	60.035	TTTGGCCCCAATGTGGTCTT	60.105	220
TRINITY_Contig8523	2	p2	(GT)6	12	1740	1751	GAGAAACCCTTGCCCTCTCC	60.035	TTTGGCCCCAATGTGGTCTT	60.105	220
TRINITY_Contig8525	1	p3	(GCA)4	12	1105	1116	CAGGAGGCTTTGGAGATGCA	60.035	CAGTCTTGTTGCCAACAGCC	59.969	273
TRINITY_Contig8526	1	p3	(CCG)4	12	502	513	AGCCATAACAAGCTCCACCC	60.034	GCTGGTCAGGAAGTTCGACA	59.966	109
TRINITY_Contig8526	2	p3	(GGC)6	18	711	728	AGCCATAACAAGCTCCACCC	60.034	GCTGGTCAGGAAGTTCGACA	59.966	109
TRINITY_Contig8527	1	p2	(CT)6	12	453	464	ACTCCCGAACTTGATGCTGG	60.036	GCATGGTTTGCCCCCAATTT	59.962	121
TRINITY_Contig8530	1	p3	(GCC)4	12	166	177	TACGTCCATGCCAAGCACAT	60.036	CAACTGCGTACACAGGGACT	59.966	118
TRINITY_Contig8533	1	p3	(TGG)4	12	14508	14519	GTGTTCACCGACGAGGACTT	59.968	TTTGCTGCCGACACATTTGG	59.968	200
TRINITY_Contig8534	1	p4	(ATTT)3	12	50	61	TAGGTGCCTCTACATGGGCT	60.031	CTGAATTTAGTGAGCGGCGC	59.973	120
TRINITY_Contig8534	2	p3	(GAA)4	12	918	929	TAGGTGCCTCTACATGGGCT	60.031	CTGAATTTAGTGAGCGGCGC	59.973	120
TRINITY_Contig8534	3	p3	(CGT)4	12	1199	1210	TAGGTGCCTCTACATGGGCT	60.031	CTGAATTTAGTGAGCGGCGC	59.973	120
TRINITY_Contig8534	4	p4	(GCTG)3	12	2081	2092	TAGGTGCCTCTACATGGGCT	60.031	CTGAATTTAGTGAGCGGCGC	59.973	120
TRINITY_Contig8534	5	p4	(GCTA)3	12	6245	6256	TAGGTGCCTCTACATGGGCT	60.031	CTGAATTTAGTGAGCGGCGC	59.973	120
TRINITY_Contig8535	1	p4	(CATC)3	12	5485	5496	ATGGATCGATCACCGTCTGC	59.967	TGGCTCCTGCGATTTTGCTA	60.036	213
TRINITY_Contig8535	2	p3	(GCT)6	18	7516	7533	ATGGATCGATCACCGTCTGC	59.967	TGGCTCCTGCGATTTTGCTA	60.036	213
TRINITY_Contig8535	3	c	(GAGAA)3gata(AGCTC)3	34	8414	8447	ATGGATCGATCACCGTCTGC	59.967	TGGCTCCTGCGATTTTGCTA	60.036	213
TRINITY_Contig8535	4	p3	(TAC)4	12	9685	9696	ATGGATCGATCACCGTCTGC	59.967	TGGCTCCTGCGATTTTGCTA	60.036	213
TRINITY_Contig8535	5	p4	(GAAG)4	16	9828	9843	ATGGATCGATCACCGTCTGC	59.967	TGGCTCCTGCGATTTTGCTA	60.036	213
TRINITY_Contig8537	1	p5	(GCCCG)3	15	3246	3260	GGTACCACCCATCCTACCCT	60.03	GGGTCTACTGGAGCTACCCA	60.032	201
TRINITY_Contig8538	1	p5	(GAGAA)3	15	7023	7037	TTCACAGGAATCGGGACACG	60.038	TTTGTCGGCTGTTTTCGCTG	59.971	106
TRINITY_Contig8539	1	p3	(CGC)4	12	108	119	GGTTGCTGCAACTGGTGAAG	59.969	TCCACCATTTTTGCAGCAGC	59.966	276
TRINITY_Contig8540	1	p3	(AGA)4	12	664	675	CAAGTCGCATGCTCATCTGC	59.972	AGGGACCGCACACAGTTATG	60.036	136
TRINITY_Contig8542	1	p3	(AAG)8	24	6254	6277	CTCTCGAAATCCTCCTCGGC	59.969	CGACCGCAAGCAGTTCAAAA	59.971	204

TRINITY_Contig8543	1	p4	(CAAT)5	20	99	118	GCATTCGAAGCATCCGTGTC	59.973	GCGGCTCCTGAAGATCTACC	59.967	107
TRINITY_Contig8544	1	p4	(GTAG)3	12	1213	1224	TCATTTGGGTCCCCATCACG	60.035	TACCCAAGGATGAGCGGAGA	60.033	202
TRINITY_Contig8544	2	p4	(CCTC)4	16	6193	6208	TCATTTGGGTCCCCATCACG	60.035	TACCCAAGGATGAGCGGAGA	60.033	202
TRINITY_Contig8545	1	p3	(CCG)7	21	4842	4862	TGGGCATGCTTAGGGAACTG	60.034	GAGAACACGAGGCAGTCCAA	59.966	220
TRINITY_Contig8546	1	p3	(TCA)4	12	1270	1281	GCCCGATTCATGGACAATGC	59.969	AACCAGGCTACCTCAAAGGC	59.962	212
TRINITY_Contig8547	1	p3	(GCC)4	12	30	41	AGTTCCAGGATCTGCCAAGC	60.035	CCGACAACAAACAAGCCCAG	59.969	280
TRINITY_Contig8547	2	p5	(TGGAT)3	15	4787	4801	AGTTCCAGGATCTGCCAAGC	60.035	CCGACAACAAACAAGCCCAG	59.969	280
TRINITY_Contig8548	1	p3	(CGC)6	18	692	709	CTAGCTTACGCTCTGACCCG	59.97	TATGAGTCGCCGGCTCATTC	59.968	244
TRINITY_Contig8548	2	c	(TCCGTG)4tccgcgtccgggacggggtcgtcgggcctcaccgtgaggaactttgacctcaacctggcgcctctgccggagaacgccgggatgaagaggtggggt(GAG)4	132	855	986	CTAGCTTACGCTCTGACCCG	59.97	TATGAGTCGCCGGCTCATTC	59.968	244
TRINITY_Contig8549	1	p4	(GTCA)3	12	353	364	GAACTCCCTCTCAAGTGCCC	60.036	AAGGCCTAGGTGATGGGGAT	60.029	263
TRINITY_Contig8551	1	p3	(GCT)4	12	624	635	CTGAGGACGTTGCTGTCAGT	59.967	ATCAGGTCCCTTCCTCCTCC	60.031	245
TRINITY_Contig8551	2	p3	(GAG)5	15	890	904	CTGAGGACGTTGCTGTCAGT	59.967	ATCAGGTCCCTTCCTCCTCC	60.031	245
TRINITY_Contig8552	1	c	(CCGG)4cctgtctttctgaagctgaagaggagtgctgttcttgccggacgtctggctgtccgttgct(CCGG)3	89	172	260	TAGGTCTGGCCGGTGATGTA	60.033	AGCCACCACAACAAAAGGGA	60.033	166
TRINITY_Contig8554	1	p4	(GCAG)3	12	1281	1292	CAGTCCCAGCAGACTTCAGG	60.037	CAACCTCAACAGTGTGCGTG	59.972	257
TRINITY_Contig8556	1	p4	(CCCT)4	16	118	133	CAACACTCTGGACGACGTCA	59.97	GGCGTGGACACTTTGTTTCC	59.97	111
TRINITY_Contig8556	2	p6	(TCTTCC)3	18	1281	1298	CAACACTCTGGACGACGTCA	59.97	GGCGTGGACACTTTGTTTCC	59.97	111
TRINITY_Contig8556	3	p6	(GCGCCC)3	18	2061	2078	CAACACTCTGGACGACGTCA	59.97	GGCGTGGACACTTTGTTTCC	59.97	111
TRINITY_Contig8556	4	p4	(TGTT)3	12	3168	3179	CAACACTCTGGACGACGTCA	59.97	GGCGTGGACACTTTGTTTCC	59.97	111
TRINITY_Contig8557	1	c	(CTCGCC)5gccctcagggcggaggccgttgccggcgctctccgtcgcgctgcccacgcggccggcggtcgctct(GCA)6	114	195	308	CCTGCTGACCTGGCTACAAA	59.963	CCTTCGCTTCGCACGTTATG	59.975	182
TRINITY_Contig8557	2	p3	(GGA)4	12	840	851	CCTGCTGACCTGGCTACAAA	59.963	CCTTCGCTTCGCACGTTATG	59.975	182
TRINITY_Contig8557	3	p4	(GCTA)3	12	1455	1466	CCTGCTGACCTGGCTACAAA	59.963	CCTTCGCTTCGCACGTTATG	59.975	182
TRINITY_Contig8557	4	p3	(ATT)4	12	1591	1602	CCTGCTGACCTGGCTACAAA	59.963	CCTTCGCTTCGCACGTTATG	59.975	182
TRINITY_Contig8558	1	p3	(CTT)4	12	5207	5218	GGCGTCGAACCAAGAAGAGA	60.039	CACTGTCTTCGACGTGGACA	59.97	139
TRINITY_Contig8559	1	p4	(TCCA)3	12	4167	4178	GACAACAATGGTGGGTGTGC	59.968	GGACAACGCTTCTGATCCCA	60.036	209
TRINITY_Contig8560	1	p3	(GGT)4	12	2535	2546	TTGTCCACCTGGTCAGCATC	59.963	TCTTCGCCGGGCTAACAATT	60.036	120
TRINITY_Contig8561	1	p5	(GTCGC)3	15	43	57	TTTCACAAACGCAGCCCATG	59.968	GATGCATGGTGGACTGTGGA	60.035	156
TRINITY_Contig8561	2	p3	(GCA)5	15	880	894	TTTCACAAACGCAGCCCATG	59.968	GATGCATGGTGGACTGTGGA	60.035	156
TRINITY_Contig8561	3	c	(CAG)6caaaat(CAG)4	36	1499	1534	TTTCACAAACGCAGCCCATG	59.968	GATGCATGGTGGACTGTGGA	60.035	156
TRINITY_Contig8562	1	c	(GCC)7(GCA)4	33	575	607	TCTGCTGTACGGTCGCTTTT	59.967	GCTTCTGGTCTTCACACCGA	59.966	117
TRINITY_Contig8563	1	c	(CCTCC)3tcctcccggagccc(CCG)4	41	116	156	GGATGGTGTGGAAGATGCCA	60.034	AACAACACCTTGCTCCACCA	60.034	220
TRINITY_Contig8564	1	p2	(AT)11	22	1512	1533	GCCATGACCTAGCTGCCTAG	59.965	TGGTTGTAAGTCACCGAGGC	59.966	181
TRINITY_Contig8564	2	p3	(TCC)4	12	4255	4266	GCCATGACCTAGCTGCCTAG	59.965	TGGTTGTAAGTCACCGAGGC	59.966	181
TRINITY_Contig8564	3	p3	(CGC)4	12	4464	4475	GCCATGACCTAGCTGCCTAG	59.965	TGGTTGTAAGTCACCGAGGC	59.966	181
TRINITY_Contig8564	4	p3	(CAC)4	12	4797	4808	GCCATGACCTAGCTGCCTAG	59.965	TGGTTGTAAGTCACCGAGGC	59.966	181
TRINITY_Contig8565	1	p4	(ATAA)3	12	109	120	GAAGCAACTCCTCCCGTTCA	59.965	CCACAAGGGCATGCTAAGGA	60.034	205
TRINITY_Contig8565	2	c	(GGC)4cgctcccctcttcccggccgctccggctccgacggcggttcctccgccc(CCG)5tagaggtcggggtccccgagccccacctcattccgcacctcgaacctgaccagcggcatgtcg(CCT)5gctg(CTC)5	173	4336	4508	GAAGCAACTCCTCCCGTTCA	59.965	CCACAAGGGCATGCTAAGGA	60.034	205
TRINITY_Contig8567	1	p4	(CGCC)3	12	4301	4312	ATTTCTGCAGCCAGTCGTCA	59.965	GATTGTTCACATCGAGCGGC	59.973	218
TRINITY_Contig8568	1	p4	(CCGG)3	12	112	123	CTCGATTCGACGCCATCTGA	59.971	CAGTACTCGTACCCGATGCC	59.97	169
TRINITY_Contig8569	1	p3	(TCC)4	12	1207	1218	ATCCTGCTCCTGGATCACCT	60.03	TGGGCGGTTACTATCCTGGA	60.032	120
TRINITY_Contig8569	2	p3	(TGG)4	12	4569	4580	ATCCTGCTCCTGGATCACCT	60.03	TGGGCGGTTACTATCCTGGA	60.032	120
TRINITY_Contig8570	1	p6	(AAAAGA)3	18	403	420	GACGGCAGGGTCTATAGCAC	59.968	GCGCAGTTGAACTCTTTGCA	59.971	103
TRINITY_Contig8570	2	c	(GAGCTC)3cttgtgaatctcctctgcgtctgt(GA)6	54	804	857	GACGGCAGGGTCTATAGCAC	59.968	GCGCAGTTGAACTCTTTGCA	59.971	103
TRINITY_Contig8570	3	p3	(CCA)4	12	4200	4211	GACGGCAGGGTCTATAGCAC	59.968	GCGCAGTTGAACTCTTTGCA	59.971	103
TRINITY_Contig8571	1	p4	(CCTT)4	16	87	102	AGTGCGCACTTTATGGTCCA	59.964	CATGTGGCAAAAGCAACGGA	59.968	276
TRINITY_Contig8571	2	p3	(ATG)4	12	606	617	AGTGCGCACTTTATGGTCCA	59.964	CATGTGGCAAAAGCAACGGA	59.968	276
TRINITY_Contig8573	1	p4	(CATT)3	12	12	23	GCCACGAATCGGAGAGGTAG	59.969	AGTTATGGCCTCCTATGCGC	59.965	115
TRINITY_Contig8573	2	p2	(TC)7	14	4562	4575	GCCACGAATCGGAGAGGTAG	59.969	AGTTATGGCCTCCTATGCGC	59.965	115
TRINITY_Contig8574	1	p3	(CGC)4	12	4216	4227	AGGCAGTTTCCATGAGCTCC	60.035	AGATAGGTGGGCCAGGGAAT	60.029	237
TRINITY_Contig8575	1	p4	(TCCC)4	16	245	260	CCACGTTTGCTTCACATGCA	59.969	GATTTTATCCCGCACGGTGC	59.971	155
TRINITY_Contig8575	2	p3	(GCG)4	12	556	567	CCACGTTTGCTTCACATGCA	59.969	GATTTTATCCCGCACGGTGC	59.971	155
TRINITY_Contig8575	3	p3	(TGC)4	12	679	690	CCACGTTTGCTTCACATGCA	59.969	GATTTTATCCCGCACGGTGC	59.971	155
TRINITY_Contig8576	1	c	(GTC)4gatcggtaaacgcgtgagatagtggtagcagcgagcccccacattcgacggaagcggccgatcgagttgagagatgggaaaaggacgctatcc(AGCT)3	117	4298	4414	CATCTTGTCGATCTCCCCGG	59.967	GTCTGATACCGACGACGACC	59.973	252
TRINITY_Contig8577	1	c	(AC)12atcaagctagtatacgtgaccacaacatggggaaat(GAATG)3	75	112	186	GCAGAGGTGGATGTCAACCA	59.963	GGTGGACTACGGCAAGTTCA	59.966	181
TRINITY_Contig8577	2	p6	(TACAGG)3	18	2728	2745	GCAGAGGTGGATGTCAACCA	59.963	GGTGGACTACGGCAAGTTCA	59.966	181
TRINITY_Contig8578	1	p3	(GGC)4	12	346	357	GCGTGCTTTCAGTTCTTGCA	59.971	CAGGATCATTCCCTCGAGGC	59.965	119
TRINITY_Contig8579	1	p3	(CAC)5	15	35	49	GCAATGATGGCAGAGAAGCG	59.971	TCGTTGGTTTCAGCTCTCCC	59.965	128
TRINITY_Contig8580	1	p3	(GAG)4	12	590	601	CTTACCGGAGATCCACGTCG	59.971	CTCGTCCGCTCTGATGTCTC	59.971	261
TRINITY_Contig8582	1	p4	(GGAG)3	12	3100	3111	CAGTTCTCGATGCGCCATTG	59.973	GCAATGTCACTGCCCCTACT	60.035	255
TRINITY_Contig8582	2	p2	(GA)6	12	3300	3311	CAGTTCTCGATGCGCCATTG	59.973	GCAATGTCACTGCCCCTACT	60.035	255
TRINITY_Contig8582	3	p3	(CTG)6	18	5176	5193	CAGTTCTCGATGCGCCATTG	59.973	GCAATGTCACTGCCCCTACT	60.035	255
TRINITY_Contig8583	1	p3	(CGG)4	12	766	777	TTCTCACCTCTGCATGGCTG	60.036	ATGGGAGCCAATGGATGCAA	60.032	222
TRINITY_Contig8583	2	p6	(TGGCAC)4	24	1039	1062	TTCTCACCTCTGCATGGCTG	60.036	ATGGGAGCCAATGGATGCAA	60.032	222
TRINITY_Contig8583	3	p3	(CCA)4	12	3689	3700	TTCTCACCTCTGCATGGCTG	60.036	ATGGGAGCCAATGGATGCAA	60.032	222
TRINITY_Contig8587	1	p3	(TGC)6	18	3918	3935	CTGAGAAGTGCCGTCCAACT	59.966	GGAACGATGCTCGGTAGAGG	59.969	165
TRINITY_Contig8588	1	p3	(AGG)4	12	2595	2606	CCGCAAATCCGAGCTAGCTA	59.968	CAGATGGCGATTTGAGCACG	59.973	189
TRINITY_Contig8591	1	p3	(CGG)4	12	305	316	CCACATCACTTTGCAAGGCC	60.038	CTTTGTGGCTGCAGACAACC	59.969	201
TRINITY_Contig8591	2	p3	(GAA)4	12	1361	1372	CCACATCACTTTGCAAGGCC	60.038	CTTTGTGGCTGCAGACAACC	59.969	201
TRINITY_Contig8593	1	p4	(CCGC)4	16	37	52	CCAAGCACACCGATGCAAAA	59.968	TGATCTCTGCATCGGCGTAC	59.969	144
TRINITY_Contig8594	1	p3	(GCC)7	21	234	254	TTCTGGGGGATGTTGATGCC	60.033	TCTGGTTTAGCCGTGCGAAT	60.037	273
TRINITY_Contig8594	2	p4	(TCAG)3	12	3789	3800	TTCTGGGGGATGTTGATGCC	60.033	TCTGGTTTAGCCGTGCGAAT	60.037	273
TRINITY_Contig8595	1	p3	(TCA)6	18	2073	2090	TCGCGTCGAGGGAAAGAAAA	59.968	CGAGTCGAACCATCTACCCG	59.971	114
TRINITY_Contig8596	1	p3	(CCT)6	18	173	190	GGCACGTGTGGAAGAGAGAA	59.966	ATGAGGACGACATCGGATGC	59.967	111
TRINITY_Contig8597	1	p3	(TCC)4	12	1810	1821	TGTGCAACTGCTCTGCGATA	60.037	GCCTTCCCAACACGTCAAAC	59.97	136
TRINITY_Contig8599	1	c	(CCA)4ctaccaccagtaggc(CGGA)3cggggcccgcccaacaaccctcctccgcccgccgtccttt(CCG)5	93	252	344	GTTGCAGCTGTGTGCAATGA	59.969	CTTGACGAGTAGCGCATTGC	59.974	279
TRINITY_Contig8599	2	p3	(GAA)4	12	640	651	GTTGCAGCTGTGTGCAATGA	59.969	CTTGACGAGTAGCGCATTGC	59.974	279
TRINITY_Contig8599	3	p3	(CGC)4	12	910	921	GTTGCAGCTGTGTGCAATGA	59.969	CTTGACGAGTAGCGCATTGC	59.974	279
TRINITY_Contig8600	1	p3	(GCC)4	12	351	362	TGGTCGTTTGAGACTTCGGG	59.967	CCATGTGTAGCTGCCTGGAA	60.035	243
TRINITY_Contig8602	1	p3	(CTA)4	12	1990	2001	ACAATGAGCATGTGGGGAGG	60.034	GGGAATTGGCACCTCCATCA	60.033	173
TRINITY_Contig8602	2	p2	(GA)8	16	4163	4178	ACAATGAGCATGTGGGGAGG	60.034	GGGAATTGGCACCTCCATCA	60.033	173
TRINITY_Contig8604	1	p4	(TAGT)4	16	1272	1287	TCCTCGGCAAGTGACTTGTC	59.966	TTGCCAAGGCTAGAGCAGAC	60.036	201
TRINITY_Contig8605	1	c	(TGC)5tggaactgcgaaccgaacga(CTG)5cggcctgctcgccgagccgaatccaaacgggttgggctt(CTG)6	107	1651	1757	GCAGAAGCCCAACCCTTTTG	59.966	AGACGCAGAGTTACGTGTGG	60.039	173
TRINITY_Contig8605	2	c	(CAG)6aagcccaacccgtttggattcggctcggcgagcaggcc(GCA)5gtcgttcggttcgcagttc(CAG)5	105	2396	2500	GCAGAAGCCCAACCCTTTTG	59.966	AGACGCAGAGTTACGTGTGG	60.039	173
TRINITY_Contig8606	1	p3	(ACT)6	18	2647	2664	GAAAGCTGCCAAAGTGCGAA	59.969	AGGTTTGGTCGCTGTCAGAG	59.966	156
TRINITY_Contig8606	2	c	(GTCG)3gc(GCT)5	29	3914	3942	GAAAGCTGCCAAAGTGCGAA	59.969	AGGTTTGGTCGCTGTCAGAG	59.966	156
TRINITY_Contig8609	1	c	(GACC)3gacatgtttccaagtaaaccaattattacccagaaaagggtggaaaaggaagaagaaaatgaa(AAT)4aaatatcggactctccctataacggacgctagacctacaaacttattgaacgcacctctcgaccaaaacggt(ATGA)3	171	192	362	CTGGCCCTTTGCAGTTCAAC	59.967	AACTCAGGTACCAACGCCAG	59.965	167
TRINITY_Contig8609	2	p3	(CTG)6	18	2612	2629	CTGGCCCTTTGCAGTTCAAC	59.967	AACTCAGGTACCAACGCCAG	59.965	167
TRINITY_Contig8609	3	c	(GAGG)3gggacggggtgggagagggggggcg(AGT)4	49	4062	4110	CTGGCCCTTTGCAGTTCAAC	59.967	AACTCAGGTACCAACGCCAG	59.965	167

TRINITY_Contig8610	1	p3	(CCA)4	12	149	160	TGAAGCAGAGCAGCGAAGAA	59.966	CGGAGAAGATGACGAGCCTC	59.97	118
TRINITY_Contig8610	2	p4	(ACCG)3	12	3847	3858	TGAAGCAGAGCAGCGAAGAA	59.966	CGGAGAAGATGACGAGCCTC	59.97	118
TRINITY_Contig8610	3	p5	(ACGAC)3	15	3970	3984	TGAAGCAGAGCAGCGAAGAA	59.966	CGGAGAAGATGACGAGCCTC	59.97	118
TRINITY_Contig8611	1	p4	(CCTC)3	12	3837	3848	TCATGGCGCAGATCCTTTGT	60.035	TTTGGACAGGACGCATCTCC	60.036	155
TRINITY_Contig8611	2	p3	(GGA)4	12	4013	4024	TCATGGCGCAGATCCTTTGT	60.035	TTTGGACAGGACGCATCTCC	60.036	155
TRINITY_Contig8613	1	p3	(GGC)4	12	3941	3952	AGCCCGTACCTGGTATCCTT	60.031	GAGTGGCCATGGGTCATGAA	60.034	254
TRINITY_Contig8616	1	p3	(CGG)4	12	587	598	ACGCTCTGTACACTGGGTTG	59.966	GCACGGCTTTGGGAACATTT	59.967	226
TRINITY_Contig8616	2	p3	(CGC)4	12	737	748	ACGCTCTGTACACTGGGTTG	59.966	GCACGGCTTTGGGAACATTT	59.967	226
TRINITY_Contig8616	3	p3	(TGA)4	12	987	998	ACGCTCTGTACACTGGGTTG	59.966	GCACGGCTTTGGGAACATTT	59.967	226
TRINITY_Contig8617	1	p3	(GGC)4	12	73	84	CACAGCGAACTTTTTGCGGA	59.971	AGCTTCTGGCATGCTCTGTT	59.962	143
TRINITY_Contig8617	2	c	(CCG)7cctc(AGCG)4	41	230	270	CACAGCGAACTTTTTGCGGA	59.971	AGCTTCTGGCATGCTCTGTT	59.962	143
TRINITY_Contig8617	3	c	(CCG)4ggcgacgacccggggggcggtgacc(CCT)4cgccgcgcccgatctgcacgtggccgccgtccgacac(GGACGA)3	104	660	763	CACAGCGAACTTTTTGCGGA	59.971	AGCTTCTGGCATGCTCTGTT	59.962	143
TRINITY_Contig8618	1	p4	(GAAA)3	12	2857	2868	GGCAAGTCCAACATCCTCGA	60.036	CCTGGCCCTGCTTATAGACG	59.966	106
TRINITY_Contig8620	1	c	(CCG)4aagccgaagccaggcgcaatcttctccttctatctatctcgcccccggagccgagcgccggcgcccgagcagcgtc(CCG)4gctcgatccgagcggccgccgcggcgtcagatcccgcgggctagcgccgcgctccccgcgccgctctccggcagatccgc(CCG)4gggagcatgctcgctcgccgcgccgctcctcctcccttttt(CCTC)3	244	3492	3735	GCAGCCCGAAGAATTTAGCG	59.972	AAGAGGGTGCTTGGATCACG	60.036	214
TRINITY_Contig8620	2	p3	(GCT)5	15	3895	3909	GCAGCCCGAAGAATTTAGCG	59.972	AAGAGGGTGCTTGGATCACG	60.036	214
TRINITY_Contig8621	1	p3	(GCA)5	15	246	260	CGACAGCTCTCCCCATGTTT	60.036	ATCGCGGTTTGGTTTGTTGG	59.969	265
TRINITY_Contig8621	2	p3	(GCG)4	12	367	378	CGACAGCTCTCCCCATGTTT	60.036	ATCGCGGTTTGGTTTGTTGG	59.969	265
TRINITY_Contig8621	3	p5	(ATACC)4	20	1245	1264	CGACAGCTCTCCCCATGTTT	60.036	ATCGCGGTTTGGTTTGTTGG	59.969	265
TRINITY_Contig8621	4	p2	(GA)17	34	3964	3997	CGACAGCTCTCCCCATGTTT	60.036	ATCGCGGTTTGGTTTGTTGG	59.969	265
TRINITY_Contig8622	1	p3	(GAT)4	12	2403	2414	TTCGACCTGCAGCATCAGTT	59.965	TCACCATGTCGAACGGGAAG	60.038	191
TRINITY_Contig8622	2	p5	(GTTTG)3	15	3741	3755	TTCGACCTGCAGCATCAGTT	59.965	TCACCATGTCGAACGGGAAG	60.038	191
TRINITY_Contig8624	1	p5	(TTTAT)3	15	37	51	GTTGGGGATCTTGGCCATGA	60.033	GATCATGCGCAAGTTCGACC	59.973	211
TRINITY_Contig8624	2	p3	(TCG)4	12	897	908	GTTGGGGATCTTGGCCATGA	60.033	GATCATGCGCAAGTTCGACC	59.973	211
TRINITY_Contig8624	3	p2	(GA)7	14	1081	1094	GTTGGGGATCTTGGCCATGA	60.033	GATCATGCGCAAGTTCGACC	59.973	211
TRINITY_Contig8624	4	p3	(CTA)5	15	1430	1444	GTTGGGGATCTTGGCCATGA	60.033	GATCATGCGCAAGTTCGACC	59.973	211
TRINITY_Contig8624	5	p4	(TGTA)3	12	2325	2336	GTTGGGGATCTTGGCCATGA	60.033	GATCATGCGCAAGTTCGACC	59.973	211
TRINITY_Contig8624	6	p3	(CCG)5	15	3805	3819	GTTGGGGATCTTGGCCATGA	60.033	GATCATGCGCAAGTTCGACC	59.973	211
TRINITY_Contig8625	1	p6	(CGGCGC)3	18	301	318	CGCAGAATTGGTATGCAGCC	59.97	CGATGCCTTCCCAACTGAGT	60.036	237
TRINITY_Contig8626	1	p3	(CGG)4	12	398	409	GGGCTGGTGCTGTTTTGAAG	59.967	CGTAGCTCCGGTGGAAGATC	59.969	100
TRINITY_Contig8626	2	p3	(CCG)4	12	3839	3850	GGGCTGGTGCTGTTTTGAAG	59.967	CGTAGCTCCGGTGGAAGATC	59.969	100
TRINITY_Contig8627	1	p2	(TG)34	68	3829	3896	ACAATGTTCGACGAGTGGCT	59.966	TGGAGCAGATAGGCCTTCCT	60.031	245
TRINITY_Contig8628	1	p3	(CGC)4	12	3362	3373	TGGTGTTGGGGAAAAGCCTT	60.032	AAAAGCCTAGGATGTGGCCC	60.033	237
TRINITY_Contig8629	1	p3	(AAG)4	12	3596	3607	CTCCATTTCGCATGCTGCTC	59.971	GGGTGCCTGAAGAGAGCAAT	60.035	217
TRINITY_Contig8629	2	p4	(GGGA)3	12	3842	3853	CTCCATTTCGCATGCTGCTC	59.971	GGGTGCCTGAAGAGAGCAAT	60.035	217
TRINITY_Contig8630	1	p3	(GAG)4	12	3361	3372	CCTCGGAGATGTGATGGACG	59.969	CTGCAGCTCTGGTCTGTTCA	59.966	254
TRINITY_Contig8630	2	c	(CGG)4ttaaatccgttgctcgggccgtgcgctccagctagctgccgacgggaccggatcggatt(GGGC)3	83	3513	3595	CCTCGGAGATGTGATGGACG	59.969	CTGCAGCTCTGGTCTGTTCA	59.966	254
TRINITY_Contig8631	1	p3	(CGG)5	15	3827	3841	CTTTTGCGATGTGCCCAACA	59.968	GGCTGTTGGTGCTGACATTG	60.039	134
TRINITY_Contig8633	1	p4	(GGGA)5	20	12	31	GGGGGAAGGAACGAGAAGTG	60.036	CGCGCAAAACTGACAAGTCA	59.973	194
TRINITY_Contig8634	1	p4	(CGCT)3	12	1078	1089	AGCCAATCAGGAAGCAGGTC	60.035	CCTCTCCAATCGTCGTCTCG	59.972	180
TRINITY_Contig8634	2	p5	(TCTTT)3	15	1449	1463	AGCCAATCAGGAAGCAGGTC	60.035	CCTCTCCAATCGTCGTCTCG	59.972	180
TRINITY_Contig8634	3	p2	(GA)13	26	1874	1899	AGCCAATCAGGAAGCAGGTC	60.035	CCTCTCCAATCGTCGTCTCG	59.972	180
TRINITY_Contig8634	4	p3	(GGC)4	12	3181	3192	AGCCAATCAGGAAGCAGGTC	60.035	CCTCTCCAATCGTCGTCTCG	59.972	180
TRINITY_Contig8634	5	p3	(CCG)4	12	3320	3331	AGCCAATCAGGAAGCAGGTC	60.035	CCTCTCCAATCGTCGTCTCG	59.972	180
TRINITY_Contig8634	6	p4	(AGCC)3	12	3640	3651	AGCCAATCAGGAAGCAGGTC	60.035	CCTCTCCAATCGTCGTCTCG	59.972	180
TRINITY_Contig8635	1	p4	(CTTG)3	12	882	893	GCGACCCTTTTTCAAACCCC	59.967	CGCAGACTGAGACAACTGGT	59.967	120
TRINITY_Contig8637	1	c	(GGC)5gacctcggccggtgt(GGCGC)3	45	82	126	ACTTCCAAGTGCGAGCTACC	60.037	TCTGGCTAGGGGCATACACT	60.031	213
TRINITY_Contig8638	1	p3	(CCG)4	12	125	136	CCTTTGGCAGGGCATTCCTA	60.033	CCAGCGGGTTTTTGACATGG	60.038	121
TRINITY_Contig8638	2	c	(CT)11catcgatcgtctccggatagggctcggcgcc(CGG)4	65	3643	3707	CCTTTGGCAGGGCATTCCTA	60.033	CCAGCGGGTTTTTGACATGG	60.038	121
TRINITY_Contig8640	1	p3	(GGA)5	15	3419	3433	TGCTTGAGAGACCCGTTGAC	59.966	AGGTACTTATGGGGCGGTCT	60.031	252
TRINITY_Contig8641	1	p2	(GA)7	14	373	386	TGTGCCATGCAGAGGTACAG	60.036	CTTCTCTTCACCGCCCAGTT	59.965	203
TRINITY_Contig8641	2	p4	(AAAG)3	12	2606	2617	TGTGCCATGCAGAGGTACAG	60.036	CTTCTCTTCACCGCCCAGTT	59.965	203
TRINITY_Contig8643	1	c	(GCG)5gctgccccggccaggtacaggggcaggcacacccgcaccgcgctctgct(CGG)5	79	1054	1132	CGTCTCGATCATCGTGTCGT	59.974	AGTGTAGCCTCCAGTCCGAT	60.033	102
TRINITY_Contig8643	2	p3	(GCC)5	15	2677	2691	CGTCTCGATCATCGTGTCGT	59.974	AGTGTAGCCTCCAGTCCGAT	60.033	102
TRINITY_Contig8644	1	p2	(TC)6	12	1708	1719	CGCGCATGGTCTTCATCAAG	59.973	TAAACCAGACTGTCCGGCAC	59.966	104
TRINITY_Contig8647	1	p3	(TGG)4	12	3379	3390	GTACATCGAGACCGTGCGAT	59.971	TCCAGGATCCGGCTGAACTA	60.033	263
TRINITY_Contig8650	1	p4	(ACCT)3	12	159	170	AAAAAGCCACGCTCAGCAAG	59.969	ACGGATGGGTTAAGCAAGGG	60.034	139
TRINITY_Contig8650	2	p6	(TCTCTT)9	54	2785	2838	AAAAAGCCACGCTCAGCAAG	59.969	ACGGATGGGTTAAGCAAGGG	60.034	139
TRINITY_Contig8650	3	p5	(GCGGC)3	15	3714	3728	AAAAAGCCACGCTCAGCAAG	59.969	ACGGATGGGTTAAGCAAGGG	60.034	139
TRINITY_Contig8652	1	p4	(AGCA)3	12	1141	1152	CATCTCCGTCACCATCTCGG	59.969	GTAGATGGACGCTCTGCTCC	59.969	220
TRINITY_Contig8652	2	p3	(CGG)5	15	3186	3200	CATCTCCGTCACCATCTCGG	59.969	GTAGATGGACGCTCTGCTCC	59.969	220
TRINITY_Contig8653	1	p6	(CCGGAG)3	18	108	125	TGGGTTGGGGTTTCAAGCTT	60.032	TTTTTCGCATGCACCTGTGG	59.968	130
TRINITY_Contig8653	2	p3	(CGG)4	12	462	473	TGGGTTGGGGTTTCAAGCTT	60.032	TTTTTCGCATGCACCTGTGG	59.968	130
TRINITY_Contig8653	3	p3	(GCT)5	15	611	625	TGGGTTGGGGTTTCAAGCTT	60.032	TTTTTCGCATGCACCTGTGG	59.968	130
TRINITY_Contig8653	4	p3	(CGA)5	15	2531	2545	TGGGTTGGGGTTTCAAGCTT	60.032	TTTTTCGCATGCACCTGTGG	59.968	130
TRINITY_Contig8653	5	p3	(CGT)4	12	2794	2805	TGGGTTGGGGTTTCAAGCTT	60.032	TTTTTCGCATGCACCTGTGG	59.968	130
TRINITY_Contig8653	6	p2	(TC)18	36	3572	3607	TGGGTTGGGGTTTCAAGCTT	60.032	TTTTTCGCATGCACCTGTGG	59.968	130
TRINITY_Contig8654	1	c	(GGC)4ggtga(GGCGG)3gagaaccggcaggcgaggagggaaaaggaagt(TGG)4	76	3539	3614	ATAGACCCTCCATTGGCCCT	60.029	ACCGCCTGAAATTGGGAGAG	60.035	231
TRINITY_Contig8655	1	p6	(GGGGAC)3	18	3331	3348	TCAGCGCATTCTTCAGAGCA	60.037	TTGCTGATGGATGCTGAGCA	60.035	256
TRINITY_Contig8656	1	p3	(GCC)4	12	654	665	TTCTCACCAGCAGCCTTCTG	59.964	ATCCAGATTCCCAGGAGGCT	60.029	148
TRINITY_Contig8657	1	p3	(GGC)5	15	3293	3307	CACCTACGGGGAAGCCAATT	60.034	CACCTGAGGATATCGGTGCC	59.966	259
TRINITY_Contig8658	1	p3	(GCG)6	18	3576	3593	TGCGGCCTGATTGTCAAGAT	60.035	GAGCTCGGTCCTGTATCTGC	59.969	279
TRINITY_Contig8659	1	p4	(AGGC)3	12	3583	3594	GACAACCGGAACACAAAGGC	59.97	GCTCGAGTCCTGGATACACG	59.97	104
TRINITY_Contig8662	1	p3	(CAT)4	12	1436	1447	GTTGCAGCAGTTCCAGTTGG	59.969	ATCAAAGGCCTCGTCCTTGG	60.035	276
TRINITY_Contig8664	1	p3	(GGC)4	12	1216	1227	ATGGGTTGGTTGATGCGGAT	60.033	GGCGCGCCACTAGTAAAATG	59.972	160
TRINITY_Contig8664	2	p3	(CGC)4	12	3578	3589	ATGGGTTGGTTGATGCGGAT	60.033	GGCGCGCCACTAGTAAAATG	59.972	160
TRINITY_Contig8667	1	p2	(TA)7	14	3373	3386	GCATCGCGGTGTACAAATCC	59.972	TTGTTCCCGGCTGAGTACAC	59.966	199
TRINITY_Contig8669	1	p3	(GGC)4	12	1675	1686	CGGAGAAGAGCGACGTCTAC	59.973	CGATCTCCTTGACGACCTCG	59.972	276
TRINITY_Contig8670	1	p3	(AGC)4	12	212	223	TGTGCAACCGCCAAATCAAG	59.968	CGGTGTAGCCTTCTGTGTGT	59.966	119
TRINITY_Contig8670	2	p6	(TCGCCG)3	18	374	391	TGTGCAACCGCCAAATCAAG	59.968	CGGTGTAGCCTTCTGTGTGT	59.966	119
TRINITY_Contig8670	3	p4	(ATTC)6	24	3195	3218	TGTGCAACCGCCAAATCAAG	59.968	CGGTGTAGCCTTCTGTGTGT	59.966	119
TRINITY_Contig8671	1	p3	(AAT)5	15	963	977	AAACGCGTGTACAAAGTGGC	59.972	GCCGCTGCTTATTCTTTCCG	59.972	132
TRINITY_Contig8671	2	p3	(GCT)5	15	2979	2993	AAACGCGTGTACAAAGTGGC	59.972	GCCGCTGCTTATTCTTTCCG	59.972	132
TRINITY_Contig8671	3	p5	(TCCAA)3	15	3334	3348	AAACGCGTGTACAAAGTGGC	59.972	GCCGCTGCTTATTCTTTCCG	59.972	132
TRINITY_Contig8672	1	p4	(ACAG)3	12	1511	1522	GCAAAAGCAACGGGTAGCAA	59.969	GACAATGGTGGTCCTGCTGA	59.963	151
TRINITY_Contig8673	1	p3	(GGA)4	12	3009	3020	CGTGAGAAAGACAGCTCGGT	60.039	GACTGCTTGCGCTTTTTCCA	59.969	254
TRINITY_Contig8674	1	c	(GGA)4gggccaggaggaggaacccgcggcgggacggg(GGGA)3ggcatttggagccggggcggcggggttcttggcccgcgggggcgaggtgga(GGT)4	119	3156	3274	GGAGCCAACAACTGCCAAAG	59.967	CAAACAACCAGCTCAGTGGC	59.969	191
TRINITY_Contig8674	2	p2	(AG)13	26	3447	3472	GGAGCCAACAACTGCCAAAG	59.967	CAAACAACCAGCTCAGTGGC	59.969	191
TRINITY_Contig8675	1	p2	(GC)6	12	3376	3387	GGGGTAAAGAGGTGAACGGG	60.036	CTCGGCCCATATTTTTGCGG	59.969	157
TRINITY_Contig8676	1	p3	(GCC)7	21	151	171	AGGAGGATGGATTGGAGCCT	60.029	TCTGAAAGCGTCCAGTGCAT	59.965	203
TRINITY_Contig8678	1	p4	(TCCG)3	12	819	830	GCTCGGGGCACAAAACAAAT	59.967	CGCTTGTTTATCCTCACGCG	59.975	110
TRINITY_Contig8678	2	p5	(CGCGC)3	15	1066	1080	GCTCGGGGCACAAAACAAAT	59.967	CGCTTGTTTATCCTCACGCG	59.975	110
TRINITY_Contig8678	3	p4	(CCAT)5	20	2390	2409	GCTCGGGGCACAAAACAAAT	59.967	CGCTTGTTTATCCTCACGCG	59.975	110
TRINITY_Contig8678	4	p5	(CAGCA)3	15	3402	3416	GCTCGGGGCACAAAACAAAT	59.967	CGCTTGTTTATCCTCACGCG	59.975	110
TRINITY_Contig8679	1	p6	(CTCTTC)3	18	1118	1135	AGTAGCTTCCCTTCAACGCC	60.036	AGTTCGTCGTGGCACATCTT	59.966	174
TRINITY_Contig8679	2	p4	(GCGA)3	12	3338	3349	AGTAGCTTCCCTTCAACGCC	60.036	AGTTCGTCGTGGCACATCTT	59.966	174
TRINITY_Contig8681	1	p3	(GCC)5	15	369	383	TCAACTTGACACCCGAGCTC	59.966	GCAGGAATTTCGACATCCGC	59.972	228

TRINITY_Contig8681	2	p3	(TGC)4	12	1565	1576	TCAACTTGACACCCGAGCTC	59.966	GCAGGAATTTCGACATCCGC	59.972	228
TRINITY_Contig8682	1	p3	(GCA)4	12	780	791	TGCAATCAGGCTCGGTTCAT	60.035	CAGGCTGTACCGCTACGTAG	59.971	180
TRINITY_Contig8683	1	p3	(GCC)4	12	1205	1216	TTGTCCAGCTTCTCGTCCAC	59.966	TGCACTTCACTGAGTCGGAC	59.968	184
TRINITY_Contig8683	2	p3	(CCG)4	12	3147	3158	TTGTCCAGCTTCTCGTCCAC	59.966	TGCACTTCACTGAGTCGGAC	59.968	184
TRINITY_Contig8685	1	p3	(GGA)4	12	345	356	GCCTTCGTCTCGGAGAACAA	60.039	GCGGAAACAATCCTGCGATC	59.972	248
TRINITY_Contig8686	1	p2	(GA)7	14	34	47	TCAAGAATTAATGCGCGCGG	59.973	ACACGAGATTGCTGAGCACA	59.966	221
TRINITY_Contig8686	2	p3	(CGA)4	12	339	350	TCAAGAATTAATGCGCGCGG	59.973	ACACGAGATTGCTGAGCACA	59.966	221
TRINITY_Contig8686	3	p3	(AGC)4	12	627	638	TCAAGAATTAATGCGCGCGG	59.973	ACACGAGATTGCTGAGCACA	59.966	221
TRINITY_Contig8686	4	p2	(AT)6	12	1608	1619	TCAAGAATTAATGCGCGCGG	59.973	ACACGAGATTGCTGAGCACA	59.966	221
TRINITY_Contig8686	5	c	(GCCT)3cagctggtcaaacagagggccggc cagagcacccgaatacagcagtccaatataat agcttgcatcagtcaaagtggtttcagctac(A AG)4	111	2325	2435	TCAAGAATTAATGCGCGCGG	59.973	ACACGAGATTGCTGAGCACA	59.966	221
TRINITY_Contig8688	1	p3	(CGG)4	12	26	37	GGATCCCCTTGGTGGTGAAG	60.034	CGTCGCCGAGATATGGAACA	59.97	155
TRINITY_Contig8688	2	p5	(TTTAT)3	15	2425	2439	GGATCCCCTTGGTGGTGAAG	60.034	CGTCGCCGAGATATGGAACA	59.97	155
TRINITY_Contig8689	1	p3	(CTG)4	12	1475	1486	CATCTTCTGGCGAACGCATG	59.973	ATCACCAACTCTGCTGTGGG	59.963	232
TRINITY_Contig8689	2	p3	(TGG)4	12	3488	3499	CATCTTCTGGCGAACGCATG	59.973	ATCACCAACTCTGCTGTGGG	59.963	232
TRINITY_Contig8691	1	p3	(GCA)4	12	3364	3375	ACCAGCGCTCCAATTCTTCA	59.963	AGCCTGGCAGAGGTTGATTC	60.035	118
TRINITY_Contig8692	1	p2	(AG)7	14	49	62	CTCATGGGATGCAATTGCCG	59.968	AACCAAGAGCCTCTCACTGC	59.964	272
TRINITY_Contig8692	2	p3	(TGA)8	24	2800	2823	CTCATGGGATGCAATTGCCG	59.968	AACCAAGAGCCTCTCACTGC	59.964	272
TRINITY_Contig8694	1	p3	(GGC)4	12	3394	3405	CTGCAGCAAAAACATGGGCT	59.966	AGGCTAGGTATGGAGGCACA	60.031	180
TRINITY_Contig8695	1	p3	(CAT)4	12	25	36	GCCTGTGGTGTAGAATGGCT	60.035	GTCTGCGGTTGAGGTTCTCA	59.966	145
TRINITY_Contig8701	1	p6	(GCGGCC)3	18	2513	2530	AACGCAAAACTTCTGACGGC	59.971	TTTGGGCAGGAACAGTTGGT	60.033	153
TRINITY_Contig8701	2	c	(GCC)4ccccgcagcggcagcgatcccatcg cgcggcaggaacagcagcgactcgatgtcctc cgccgtgaaccgatt(GGC)4	96	2710	2805	AACGCAAAACTTCTGACGGC	59.971	TTTGGGCAGGAACAGTTGGT	60.033	153
TRINITY_Contig8702	1	p3	(CTC)4	12	3062	3073	CATTGCCACGGTTTTCCCAG	60.038	AACTGGGGGACTGCTAATGC	60.034	126
TRINITY_Contig8705	1	p4	(ATGG)3	12	971	982	TGCCCACAAGATAGCCCTTG	60.034	CATCGGTGGAGACTCGTACG	59.972	116
TRINITY_Contig8705	2	c*	(TTCT)2(TTCTTTTTCTTTTTCTTTTTC TGTAGACAAATACACTGGATTGAC CACAGCTGACGGTCGTGTGGGGAC AACTAGCATCAGCTAAACAAGTAC GCTGTATCTTTTTTTTTTTTTGGTCA ACAAGAAGTACACTGGATCGGCCA CAGCTGACGGTCGTATGGGGACAA CTAGCATCAGCTACTTCGGACGGCT CCACGAATGCGTGAATCCCCACAGC TGCACAAAAGAAATGTCAGTGAAG GGCATGCGAAATCAAGAACAGAAA CGAAAATATCTGACAGCGGCTGCA GAATGGAAATCAAGCATTGGAATG TTCCATCTGAATAGTGGTTTTCTAA TAGTTCGCTAATTCAAACTACATGA TGTAAGTAAGAGAAATGCGAAGAT GGTGCTGTTTCTTTTTTCTCCCCACA CCTGAAATGTCGTGCAATGGTCGTT	25	1246	1270	TGCCCACAAGATAGCCCTTG	60.034	CATCGGTGGAGACTCGTACG	59.972	116
TRINITY_Contig8705	3	p5	(CACGG)3	15	3390	3404	TGCCCACAAGATAGCCCTTG	60.034	CATCGGTGGAGACTCGTACG	59.972	116
TRINITY_Contig8706	1	p5	(CCTCT)3	15	3064	3078	ATATGCAGTCGGAGCAGTCG	59.969	AGGCCGGAGAAGATACTGGT	60.032	153
TRINITY_Contig8707	1	p3	(CCT)4	12	1068	1079	ATTCTTTCTGCGGTCCTGGG	60.035	CCTCGTCAATGATGCTTGCG	59.973	138
TRINITY_Contig8707	2	p2	(AG)6	12	1193	1204	ATTCTTTCTGCGGTCCTGGG	60.035	CCTCGTCAATGATGCTTGCG	59.973	138
TRINITY_Contig8707	3	p4	(TCTT)3	12	1623	1634	ATTCTTTCTGCGGTCCTGGG	60.035	CCTCGTCAATGATGCTTGCG	59.973	138
TRINITY_Contig8708	1	p4	(TCGA)4	16	3240	3255	CCCTGCAATCAGCTCTGTGA	60.036	TCGCAGTGAACGAGAACGAA	59.97	128
TRINITY_Contig8709	1	c	(CCG)4cgaccgcgcgaccgcgccgacgaag gcctcgagcgaaga(GGCGG)3	66	3190	3255	GGAGCTATGACCGGTACACG	59.97	AGCATCTGGGAAGGCAAGAC	60.035	198
TRINITY_Contig8711	1	p6	(GGGGGC)3	18	185	202	TGTCCCTATTCCCGGCTACA	60.032	TGGAGATTCGTCCCCTGGAT	60.031	181
TRINITY_Contig8711	2	p3	(AGA)4	12	1798	1809	TGTCCCTATTCCCGGCTACA	60.032	TGGAGATTCGTCCCCTGGAT	60.031	181
TRINITY_Contig8712	1	p4	(GCAG)3	12	194	205	CACGCACAAAAAGGTGAGGG	59.969	CTCCGGCACCAGAAGAAACT	59.965	260
TRINITY_Contig8712	2	p3	(TGC)4	12	2158	2169	CACGCACAAAAAGGTGAGGG	59.969	CTCCGGCACCAGAAGAAACT	59.965	260
TRINITY_Contig8712	3	p3	(TTG)4	12	2701	2712	CACGCACAAAAAGGTGAGGG	59.969	CTCCGGCACCAGAAGAAACT	59.965	260
TRINITY_Contig8715	1	p4	(CCAA)3	12	160	171	TCGGAGCTCCTGACTGATGA	60.034	CACTCCACCAAGGCTTAGG	60.035	116
TRINITY_Contig8717	1	c	(TCG)4atgaaggagaacagcagcgcatttc caggccccatcctgaacgggatagagatcatg aagatgcacttcagctcaggttc(CGT)4	104	1655	1758	CGATCCATCCGGCAAGATCA	59.966	TTCGATCTGGCCGATGATGG	59.966	162
TRINITY_Contig8719	1	p4	(CCTC)3	12	442	453	AACGACGGGATGCAGGAATT	60.035	TCCTCATACTCGCTTCCCCA	60.033	162
TRINITY_Contig8719	2	c	(GGT)4tcttcgcggcagagcaggagcagag gagagggca(AGG)5	61	944	1004	AACGACGGGATGCAGGAATT	60.035	TCCTCATACTCGCTTCCCCA	60.033	162
TRINITY_Contig8719	3	p3	(CTC)4	12	1613	1624	AACGACGGGATGCAGGAATT	60.035	TCCTCATACTCGCTTCCCCA	60.033	162
TRINITY_Contig8719	4	p4	(TTGC)3	12	3227	3238	AACGACGGGATGCAGGAATT	60.035	TCCTCATACTCGCTTCCCCA	60.033	162
TRINITY_Contig8722	1	c	(GCC)5tccatcgcccgcggaaaccctaggcc cccgcgcactctcccgccctccaacgacgagtt (GGGCGC)3	92	3092	3183	ATACTAGTAACCTGCGCGGC	59.968	TCCCCTTCTCTTACCCTCCG	60.032	256
TRINITY_Contig8723	1	p5	(GGGGA)3	15	3259	3273	CTCGCGACAATGCCAATCAG	59.973	CCCTCACTTGTTCCGATGCT	60.036	201
TRINITY_Contig8725	1	p5	(AGGAA)3	15	22	36	TGACCCCAATGTGGACGATG	60.036	ATTGCACCCCTGAATCTGGG	60.033	108
TRINITY_Contig8725	2	p3	(CGG)4	12	161	172	TGACCCCAATGTGGACGATG	60.036	ATTGCACCCCTGAATCTGGG	60.033	108
TRINITY_Contig8726	1	c	(TC)6acaccccagcagcttcttcgtctcct(C TCC)3	50	96	145	CCGGATGTCTCCTCGATGTG	59.969	ATGAGCTTAGAGGGGAGCCA	60.031	185
TRINITY_Contig8726	2	p3	(CAG)4	12	1973	1984	CCGGATGTCTCCTCGATGTG	59.969	ATGAGCTTAGAGGGGAGCCA	60.031	185
TRINITY_Contig8726	3	p3	(CAG)8	24	2180	2203	CCGGATGTCTCCTCGATGTG	59.969	ATGAGCTTAGAGGGGAGCCA	60.031	185
TRINITY_Contig8728	1	p3	(TCA)4	12	2280	2291	TAGGAGAGTGGGTAGTGGGC	60.032	ATCTCGAGTGCAGGCATAGC	59.967	186
TRINITY_Contig8728	2	p6	(GGCAAT)3	18	3212	3229	TAGGAGAGTGGGTAGTGGGC	60.032	ATCTCGAGTGCAGGCATAGC	59.967	186
TRINITY_Contig8729	1	p3	(GCC)4	12	100	111	ACCCCTCCTTGATCTCCCTC	60.031	ACCCTTTCATGGCAGATGGG	60.033	173
TRINITY_Contig8730	1	p3	(ACC)6	18	145	162	GCAAGGGGTATCTCCTGACG	59.895	CTCGTCGGTGAAAGTGGTGA	59.968	127
TRINITY_Contig8730	2	p3	(CGC)4	12	3198	3209	GCAAGGGGTATCTCCTGACG	59.895	CTCGTCGGTGAAAGTGGTGA	59.968	127
TRINITY_Contig8732	1	p3	(TCC)4	12	1025	1036	GGTGTGCGTGTTACCCAAAC	59.97	GTCAAACACAACGTGTCGGG	59.973	161
TRINITY_Contig8732	2	c*	(GGT)4(<GGT>GGA)(GGTGGA)2	30	1166	1195	GGTGTGCGTGTTACCCAAAC	59.97	GTCAAACACAACGTGTCGGG	59.973	161
TRINITY_Contig8732	3	p3	(TTC)4	12	2056	2067	GGTGTGCGTGTTACCCAAAC	59.97	GTCAAACACAACGTGTCGGG	59.973	161
TRINITY_Contig8733	1	p4	(GGAG)3	12	21	32	GCAACCAGCTTTGACACCTG	59.969	CGGCCCTTTGAGTCAGTCAT	60.036	237
TRINITY_Contig8733	2	c	(CCTG)3ccttcctacctgcgcgcggcggcgg gctcgaggtcccggcggtggtgctggagcga gccaca(GGC)4	87	176	262	GCAACCAGCTTTGACACCTG	59.969	CGGCCCTTTGAGTCAGTCAT	60.036	237
TRINITY_Contig8734	1	c	(GCG)6gccccggtaaggtacaggggcaggc acacccgcagcgcgctccgct(CGG)4ccttc ctctc(CCGG)3	98	578	675	CCCCAACCTTAATCCGCTGT	60.034	AGCAGAACGAACTCGCTTCA	59.968	169
TRINITY_Contig8734	2	p3	(GCC)4	12	2213	2224	CCCCAACCTTAATCCGCTGT	60.034	AGCAGAACGAACTCGCTTCA	59.968	169
TRINITY_Contig8735	1	p4	(CGCC)3	12	463	474	TGGGTTTTGCTCCCCCTTTT	60.031	GAGCCATCCACAAGGCTTCT	60.035	163
TRINITY_Contig8736	1	p2	(TG)6	12	3075	3086	GCCGTCGGGATAGTACCAAG	59.968	TAGGGCAATGGAAGCATGCA	60.033	210
TRINITY_Contig8737	1	p3	(GCC)6	18	2897	2914	GTACACGTGCAGGTCACTCA	59.968	CTGGAGGTGTATTGCAGGCA	60.035	222
TRINITY_Contig8738	1	p3	(CTG)4	12	177	188	AGCGTCTGGAGAAGGTTGTG	59.966	CCCGTTGCTCATGTACTCGT	60.109	155
TRINITY_Contig8738	2	c	(CCG)4gcaacgtcgt(GATC)3	34	2475	2508	AGCGTCTGGAGAAGGTTGTG	59.966	CCCGTTGCTCATGTACTCGT	60.109	155
TRINITY_Contig8738	3	p3	(GGC)5	15	2826	2840	AGCGTCTGGAGAAGGTTGTG	59.966	CCCGTTGCTCATGTACTCGT	60.109	155
TRINITY_Contig8738	4	c	(GCT)5atgatcttcttcttgttgttcgttgcgt gtggttgatcgaggaggta(TGG)4	74	3064	3137	AGCGTCTGGAGAAGGTTGTG	59.966	CCCGTTGCTCATGTACTCGT	60.109	155
TRINITY_Contig8739	1	p3	(TTC)4	12	217	228	CAGGACGTCAAGACTGCCTT	59.966	AGTGGCATGGGGAATTCCTG	60.033	124
TRINITY_Contig8740	1	p3	(GCG)5	15	3080	3094	CATCCGAGTAGCTGCACGAT	59.969	TTCAGCTGCTCAGTGGACTG	59.966	250
TRINITY_Contig8741	1	c	(CAG)4gcagcagagagcgc(AGCA)3	38	88	125	ACAACTCCGACAACTCCGTC	59.968	CGAAGATGTGCTTGCCGATG	59.973	225
TRINITY_Contig8741	2	p3	(CGG)4	12	906	917	ACAACTCCGACAACTCCGTC	59.968	CGAAGATGTGCTTGCCGATG	59.973	225
TRINITY_Contig8742	1	p3	(GGC)4	12	3188	3199	TATGAAGGGGCGCAGGTTTT	59.961	GGGCTATCATGACGCATGGA	59.965	154
TRINITY_Contig8743	1	p3	(CGC)5	15	118	132	GCTGTCGCACAACAACATGT	59.971	TTGTGGCTCACGTCGATGAA	59.967	222
TRINITY_Contig8743	2	p3	(GCC)4	12	319	330	GCTGTCGCACAACAACATGT	59.971	TTGTGGCTCACGTCGATGAA	59.967	222
TRINITY_Contig8743	3	p3	(CCG)4	12	1868	1879	GCTGTCGCACAACAACATGT	59.971	TTGTGGCTCACGTCGATGAA	59.967	222
TRINITY_Contig8744	1	p6	(GCCAGG)3	18	2186	2203	GGTGTTGTCCATCTGCCGTA	60.037	TGACAGGGCCATGGTACCTA	59.957	102
TRINITY_Contig8745	1	p5	(TTTTG)3	15	3042	3056	GGGTCGCTAGATTCGAAGGG	59.968	CAACTGCACCATTTCCGCAA	59.968	132
TRINITY_Contig8746	1	p3	(GGA)5	15	3172	3186	TGGTGTTTTCCGATCTGCGA	59.966	CCTTTGGCTTTGTCCGGTTG	59.968	113
TRINITY_Contig8750	1	p3	(CGC)4	12	2836	2847	ACGCGAGCCATGAGGAATAG	59.967	TCCTGGAGCACATTCAGCAG	60.036	148

TRINITY_Contig8750	2	p3	(GGC)4	12	2975	2986	ACGCGAGCCATGAGGAATAG	59.967	TCCTGGAGCACATTCAGCAG	60.036	148
TRINITY_Contig8750	3	c	(GGAA)5ggcagtgtggcctggcactggcagtgcatggggatgc(GA)15	87	3090	3176	ACGCGAGCCATGAGGAATAG	59.967	TCCTGGAGCACATTCAGCAG	60.036	148
TRINITY_Contig8751	1	p4	(GCCT)3	12	1197	1208	GGAATTGGGAAGGGGAAGGG	60.032	AGACAGACGCGGCTATGATG	59.969	199
TRINITY_Contig8752	1	p3	(ATC)4	12	2450	2461	CATACCATCGTAGCAGCGGT	59.968	TTTGGCCACTTCCCCAAACT	60.032	132
TRINITY_Contig8753	1	p4	(GGTA)3	12	379	390	TCTCTCGTGGGTTGTGAAGC	59.966	CAACCACCACTTCGCACTTG	59.971	115
TRINITY_Contig8753	2	p3	(GAG)4	12	2611	2622	TCTCTCGTGGGTTGTGAAGC	59.966	CAACCACCACTTCGCACTTG	59.971	115
TRINITY_Contig8753	3	p3	(CGC)4	12	2751	2762	TCTCTCGTGGGTTGTGAAGC	59.966	CAACCACCACTTCGCACTTG	59.971	115
TRINITY_Contig8753	4	p3	(GAA)4	12	2949	2960	TCTCTCGTGGGTTGTGAAGC	59.966	CAACCACCACTTCGCACTTG	59.971	115
TRINITY_Contig8755	1	p4	(CGCC)3	12	463	474	CCGATTACCTCCTCAGCGTC	59.969	AATTGCGCTCTTGGCCTACT	60.035	180
TRINITY_Contig8757	1	p3	(CGC)5	15	37	51	AACATTCTCTGCCCTGCTCC	60.035	CCCACCATCTTGGCCAGAAT	60.033	205
TRINITY_Contig8757	2	p3	(CGG)5	15	953	967	AACATTCTCTGCCCTGCTCC	60.035	CCCACCATCTTGGCCAGAAT	60.033	205
TRINITY_Contig8758	1	p5	(AGACG)3	15	698	712	TCACCCGATCTGGATGGAGT	60.032	TAGCATCGTCTGGGGTGAGA	60.034	266
TRINITY_Contig8758	2	p3	(CCG)4	12	1805	1816	TCACCCGATCTGGATGGAGT	60.032	TAGCATCGTCTGGGGTGAGA	60.034	266
TRINITY_Contig8760	1	c	(CATT)3(CATC)3	24	16	39	ATCTTCACAAGCGTCACGGT	59.966	ATCCTCATCCAGGTCCCGAA	60.031	228
TRINITY_Contig8762	1	p3	(CAG)4	12	371	382	CCCGGTTTGTCTCTTCTCCC	60.036	ACCTCAACATGACCACCGTC	59.965	143
TRINITY_Contig8762	2	p3	(GCA)4	12	774	785	CCCGGTTTGTCTCTTCTCCC	60.036	ACCTCAACATGACCACCGTC	59.965	143
TRINITY_Contig8762	3	p3	(GCT)4	12	2375	2386	CCCGGTTTGTCTCTTCTCCC	60.036	ACCTCAACATGACCACCGTC	59.965	143
TRINITY_Contig8762	4	p3	(CCG)4	12	2989	3000	CCCGGTTTGTCTCTTCTCCC	60.036	ACCTCAACATGACCACCGTC	59.965	143
TRINITY_Contig8763	1	p3	(CGC)4	12	249	260	GCCCCTGATCCATATCCAGC	60.035	TTTCGCTCTTCAAGTCGCCT	59.966	167
TRINITY_Contig8763	2	p4	(CTCC)4	16	2190	2205	GCCCCTGATCCATATCCAGC	60.035	TTTCGCTCTTCAAGTCGCCT	59.966	167
TRINITY_Contig8764	1	p5	(TCCCC)3	15	2	16	GGAAGCCGAGAAGGAGATCG	59.969	GTGAGGTCGTAGCCGAGATG	59.97	248
TRINITY_Contig8764	2	p3	(CGG)4	12	880	891	GGAAGCCGAGAAGGAGATCG	59.969	GTGAGGTCGTAGCCGAGATG	59.97	248
TRINITY_Contig8766	1	c	(AGGC)3ggcaaggggcgcacaagttggcgcagcgagccgagccctttattccccggatccccgcaaccaaattgagtcgggggaacca(GCG)6	112	53	164	ATGCTGATCTTCCGCCGTAG	59.967	CTATGGGTTCGTGTGGCCTT	60.035	209
TRINITY_Contig8766	2	p3	(GGC)4	12	428	439	ATGCTGATCTTCCGCCGTAG	59.967	CTATGGGTTCGTGTGGCCTT	60.035	209
TRINITY_Contig8766	3	p3	(CGC)4	12	2991	3002	ATGCTGATCTTCCGCCGTAG	59.967	CTATGGGTTCGTGTGGCCTT	60.035	209
TRINITY_Contig8767	1	p3	(GCG)4	12	3010	3021	TCGATCGGGGTTCCTTGTTG	60.037	GAAGCGTCCGAGATCTCCAG	59.97	269
TRINITY_Contig8768	1	p4	(CACG)3	12	717	728	TCAACCTTCGCATCCCCTTC	60.035	GACATCAGCTCGACGAGGAG	59.971	154
TRINITY_Contig8769	1	p3	(GAG)5	15	186	200	GTCAACCGAAGAGGATGGCA	60.036	CCACCCTCAGAGATCCCTCA	60.032	225
TRINITY_Contig8770	1	p3	(AGG)5	15	1271	1285	TGCCGGGCACATTATCAGTT	60.035	GCTGCAAATGGATGCTACCG	59.97	107
TRINITY_Contig8771	1	p4	(CACG)3	12	187	198	GCCCTACCCCCACTTTGTAC	60.035	GCCACGTCGACTGATACGAT	59.971	183
TRINITY_Contig8772	1	c	(AGA)4a(CAAG)3	25	2621	2645	AGAACAAGCAAGCAAGCACG	59.97	AGAGATCCAGAGCCCCCAAT	60.029	251
TRINITY_Contig8777	1	p3	(CCG)4	12	2359	2370	TTGGATCTTCAGAACGGGGC	60.035	AAAACAAGTCTTCGCGTGCC	59.971	159
TRINITY_Contig8778	1	p3	(GCA)4	12	141	152	TCAGTGGTTGGCTTGATCCC	59.962	CCACAGTTGCAGTCCAAACG	59.971	259
TRINITY_Contig8778	2	p3	(CCG)4	12	2261	2272	TCAGTGGTTGGCTTGATCCC	59.962	CCACAGTTGCAGTCCAAACG	59.971	259
TRINITY_Contig8778	3	p3	(CGC)5	15	2692	2706	TCAGTGGTTGGCTTGATCCC	59.962	CCACAGTTGCAGTCCAAACG	59.971	259
TRINITY_Contig8778	4	p4	(GGAG)3	12	3089	3100	TCAGTGGTTGGCTTGATCCC	59.962	CCACAGTTGCAGTCCAAACG	59.971	259
TRINITY_Contig8779	1	p5	(ATTTC)3	15	107	121	ATCGTGGACTCTGTGCCAAG	60.037	ACCCACTGATGCATTGGAGG	60.034	133
TRINITY_Contig8779	2	p3	(CGC)4	12	2985	2996	ATCGTGGACTCTGTGCCAAG	60.037	ACCCACTGATGCATTGGAGG	60.034	133
TRINITY_Contig8780	1	p3	(GCG)8	24	2943	2966	CGGCTGCTCTCATGCATTTC	59.971	GCATCTTGCCTCTGGTCTGT	60.036	272
TRINITY_Contig8782	1	c	(GTT)4gtcgtcgctgccgttcccgtggccgttgttggcgctggtgtggttgccgccatcgtcgggggtggcc(CTG)4	90	2878	2967	TCCAGCATCCTTTCGCTGTT	59.963	CAGTGAAAGTGGTGTTGGCG	59.971	205
TRINITY_Contig8783	1	p3	(CGA)5	15	1062	1076	ATCGTCCTGCCCTCCTACTT	60.032	GCATCTACATCGCTTGCAGC	60.041	154
TRINITY_Contig8784	1	p3	(CGG)4	12	2551	2562	GTACGTTTGCATCGATGGCC	59.972	ACCACTTGAGCTCGTTCTCG	60.039	225
TRINITY_Contig8788	1	p3	(ATG)4	12	2637	2648	GAAGCTCCTTAAGGCAGCCA	60.035	GGGTCTGCACACTTCCATGA	59.963	158
TRINITY_Contig8790	1	p3	(GAG)4	12	2582	2593	CACTATGATTGCCGCCTTGC	59.97	CTCCTCCTCCTCGTCTTGGA	60.034	280
TRINITY_Contig8791	1	p2	(TC)8	16	89	104	TCAACGTCAACCTCATGGGG	59.964	CTACTGTGATCCGCCGAGTC	59.97	251
TRINITY_Contig8791	2	c	(GGC)4agcggaggggggaggaggtgaaggagaaggga(GGC)5	58	379	436	TCAACGTCAACCTCATGGGG	59.964	CTACTGTGATCCGCCGAGTC	59.97	251
TRINITY_Contig8791	3	p4	(TTTG)3	12	1222	1233	TCAACGTCAACCTCATGGGG	59.964	CTACTGTGATCCGCCGAGTC	59.97	251
TRINITY_Contig8792	1	p3	(GCG)4	12	21	32	CAGAGCGTCCAGATCCATCC	59.967	TAGCATAGTACTGTGGCGCG	59.97	157
TRINITY_Contig8793	1	p5	(CAACA)3	15	76	90	GCAGTCGTCCAACACAACAC	59.972	TCTACATCAGATGGGCCCCA	60.03	279
TRINITY_Contig8793	2	p3	(GCG)4	12	2862	2873	GCAGTCGTCCAACACAACAC	59.972	TCTACATCAGATGGGCCCCA	60.03	279
TRINITY_Contig8795	1	p4	(CCCG)3	12	376	387	GAATCGCACTGAATCCGTGC	59.973	GCAGCACCCTCCTTTCTTCT	59.963	199
TRINITY_Contig8795	2	p6	(GGCGCG)4	24	1373	1396	GAATCGCACTGAATCCGTGC	59.973	GCAGCACCCTCCTTTCTTCT	59.963	199
TRINITY_Contig8795	3	p3	(GCC)4	12	1736	1747	GAATCGCACTGAATCCGTGC	59.973	GCAGCACCCTCCTTTCTTCT	59.963	199
TRINITY_Contig8795	4	p3	(GCG)4	12	1915	1926	GAATCGCACTGAATCCGTGC	59.973	GCAGCACCCTCCTTTCTTCT	59.963	199
TRINITY_Contig8795	5	p4	(TCAG)3	12	2393	2404	GAATCGCACTGAATCCGTGC	59.973	GCAGCACCCTCCTTTCTTCT	59.963	199
TRINITY_Contig8797	1	p3	(CGG)4	12	2519	2530	CAACAAAAGCAGTGGGGACG	59.969	ACATGGTGCAGGGCTTCTAC	60.035	222
TRINITY_Contig8797	2	p3	(GGC)4	12	2655	2666	CAACAAAAGCAGTGGGGACG	59.969	ACATGGTGCAGGGCTTCTAC	60.035	222
TRINITY_Contig8797	3	c	(CGCCGG)4aggtgtcgccggcgtcc(ATCGG)3	56	2774	2829	CAACAAAAGCAGTGGGGACG	59.969	ACATGGTGCAGGGCTTCTAC	60.035	222
TRINITY_Contig8798	1	c	(CAG)4ctagtgtcgatccctctcagagtattgcaactgcaccttgggccacccacaatgcagcaccg(CCA)4	86	2095	2180	CCACCAATGTACCCTCCACC	60.034	GAGGGATCGACACTAGCTGC	59.969	101
TRINITY_Contig8798	2	p5	(TAGAT)4	20	2861	2880	CCACCAATGTACCCTCCACC	60.034	GAGGGATCGACACTAGCTGC	59.969	101
TRINITY_Contig8799	1	p3	(GAT)5	15	260	274	CGGACATTTCGTTCATCGGC	59.974	GTCCATGGTCTGCTTCAGCT	60.036	251
TRINITY_Contig8799	2	p3	(GCG)6	18	1997	2014	CGGACATTTCGTTCATCGGC	59.974	GTCCATGGTCTGCTTCAGCT	60.036	251
TRINITY_Contig8799	3	p2	(GA)15	30	2735	2764	CGGACATTTCGTTCATCGGC	59.974	GTCCATGGTCTGCTTCAGCT	60.036	251
TRINITY_Contig8800	1	p3	(CGC)5	15	70	84	TCGCATCTTCAGATCCTCGC	59.969	TCCGGGAAAGCAATACCCAC	60.035	141
TRINITY_Contig8800	2	p3	(CGC)5	15	193	207	TCGCATCTTCAGATCCTCGC	59.969	TCCGGGAAAGCAATACCCAC	60.035	141
TRINITY_Contig8801	1	c	(CGACGG)3cgagggtttgatttactactacag(CGA)4	54	1309	1362	GCACTACAGGTGGGGGAAAA	59.888	TCGCAGTAGAAGTGGGAGGA	59.961	210
TRINITY_Contig8801	2	p3	(CGG)4	12	1596	1607	GCACTACAGGTGGGGGAAAA	59.888	TCGCAGTAGAAGTGGGAGGA	59.961	210
TRINITY_Contig8801	3	p3	(GTC)4	12	2303	2314	GCACTACAGGTGGGGGAAAA	59.888	TCGCAGTAGAAGTGGGAGGA	59.961	210
TRINITY_Contig8802	1	p4	(CATA)3	12	1573	1584	CCGCACATTCATTAGCTGGC	59.97	CGAGAACGAGAAGGGCAGTT	60.038	243
TRINITY_Contig8802	2	p4	(GCCG)4	16	2474	2489	CCGCACATTCATTAGCTGGC	59.97	CGAGAACGAGAAGGGCAGTT	60.038	243
TRINITY_Contig8803	1	p4	(GATA)3	12	152	163	GCCTGCAACCTTGGAAACTG	59.967	CGGTCCTCTATGCTCGGTTC	59.969	116
TRINITY_Contig8803	2	p3	(CTC)5	15	880	894	GCCTGCAACCTTGGAAACTG	59.967	CGGTCCTCTATGCTCGGTTC	59.969	116
TRINITY_Contig8804	1	p2	(CG)6	12	414	425	ACCTTGGCCCTTGAACCAAA	60.032	CTTTCAGATAACTGCGCGCC	59.973	215
TRINITY_Contig8805	1	p3	(GCC)4	12	2703	2714	CCACGAGAGGGCTTCAACTT	59.965	TACCACTGGATGCGCGATAC	59.968	254
TRINITY_Contig8806	1	p4	(GTTG)3	12	2690	2701	TGCAGATTCACGAAGGCCAT	60.035	CCATCCGTTTCAGCTCCCTT	60.035	270
TRINITY_Contig8807	1	c	(CGG)5ggaacccgacgtggcgcctcttctggaggctctcctcgcgcttggccttgcggatgtccaccatctggttctc(GCG)4	100	2691	2790	GCATACACGTTCACATCGCC	59.974	TCAAGAAAGAGGCTGCCTGG	59.963	220
TRINITY_Contig8807	2	c	(GGC)4ggggtggggggaagtggagcggggggagttgcgcaggcgagccgctcg(CGCA)3	72	2892	2963	GCATACACGTTCACATCGCC	59.974	TCAAGAAAGAGGCTGCCTGG	59.963	220
TRINITY_Contig8808	1	p3	(CCG)4	12	2164	2175	GCATCGCAAATTTCGCAAGC	59.976	CCTAGATCGCTGCAGTGAGG	59.968	188
TRINITY_Contig8808	2	p3	(GAG)5	15	2400	2414	GCATCGCAAATTTCGCAAGC	59.976	CCTAGATCGCTGCAGTGAGG	59.968	188
TRINITY_Contig8808	3	p2	(TC)8	16	2699	2714	GCATCGCAAATTTCGCAAGC	59.976	CCTAGATCGCTGCAGTGAGG	59.968	188
TRINITY_Contig8809	1	p3	(TCC)4	12	152	163	GTGTCACTGTACTCCGGCAA	59.967	AGTTCTCCATGCACAGCGAA	59.965	100
TRINITY_Contig8809	2	p3	(CGG)5	15	1008	1022	GTGTCACTGTACTCCGGCAA	59.967	AGTTCTCCATGCACAGCGAA	59.965	100
TRINITY_Contig8809	3	p3	(GCG)5	15	2116	2130	GTGTCACTGTACTCCGGCAA	59.967	AGTTCTCCATGCACAGCGAA	59.965	100
TRINITY_Contig8809	4	p3	(CGG)4	12	2418	2429	GTGTCACTGTACTCCGGCAA	59.967	AGTTCTCCATGCACAGCGAA	59.965	100
TRINITY_Contig8811	1	p3	(GAC)4	12	564	575	CTGATGAACACCGAGGCAGT	60.037	TCATCAATTCCTGGTGCCCC	60.033	200
TRINITY_Contig8811	2	p3	(CGC)6	18	766	783	CTGATGAACACCGAGGCAGT	60.037	TCATCAATTCCTGGTGCCCC	60.033	200
TRINITY_Contig8812	1	p3	(AGC)4	12	69	80	TGATGTGCAACAGCGTTGTG	59.971	GAAGCAAGGGCAAAATGGCA	59.965	247
TRINITY_Contig8812	2	p5	(GAGAA)3	15	2621	2635	TGATGTGCAACAGCGTTGTG	59.971	GAAGCAAGGGCAAAATGGCA	59.965	247
TRINITY_Contig8813	1	p6	(CTGCTC)3	18	122	139	TGGCGAGGGTGGTGATACTA	60.033	CCAATCCGACCAGAAGCCTT	60.035	246
TRINITY_Contig8814	1	p3	(CCG)4	12	120	131	CAACGTGCTGTGTGTGACTG	59.973	ACAGCCATTTCCTCCACCAG	59.961	127
TRINITY_Contig8815	1	p3	(GCT)5	15	731	745	TACCCATGGCCTTGAGCTTG	60.034	AGGAGGAGGAGCTCGACATT	60.033	189
TRINITY_Contig8815	2	p3	(CGG)4	12	2749	2760	TACCCATGGCCTTGAGCTTG	60.034	AGGAGGAGGAGCTCGACATT	60.033	189
TRINITY_Contig8815	3	p3	(ATA)5	15	3067	3081	TACCCATGGCCTTGAGCTTG	60.034	AGGAGGAGGAGCTCGACATT	60.033	189
TRINITY_Contig8816	1	p3	(CGC)5	15	63	77	AGCTGTGGACGAACTTGGAG	59.966	GAGCCTCAAAGTCGGTCACA	59.966	120
TRINITY_Contig8817	1	c	(AGA)4ggaatgtgaatgataacgagggcttgagtggcgatggcgattcggtagttgagaatgagc(AGG)4agaagg(AGA)4	102	409	510	AGCAGGAGGAGGAGGAGAAG	60.032	CGACTTCCTCATCGCTCTCC	59.97	108
TRINITY_Contig8819	1	p3	(TCG)4	12	389	400	TCAAACACGGAGGAGAAGGC	59.965	CGCACAAGAGGCGATTCATG	59.973	133
TRINITY_Contig8819	2	p4	(CTAG)3	12	684	695	TCAAACACGGAGGAGAAGGC	59.965	CGCACAAGAGGCGATTCATG	59.973	133
TRINITY_Contig8819	3	p2	(AG)15	30	1635	1664	TCAAACACGGAGGAGAAGGC	59.965	CGCACAAGAGGCGATTCATG	59.973	133

TRINITY_Contig8819	4	p5	(GGATC)3	15	2346	2360	TCAAACACGGAGGAGAAGGC	59.965	CGCACAAGAGGCGATTCATG	59.973	133
TRINITY_Contig8819	5	p3	(GCG)4	12	2510	2521	TCAAACACGGAGGAGAAGGC	59.965	CGCACAAGAGGCGATTCATG	59.973	133
TRINITY_Contig8819	6	p6	(CGCCGG)3	18	2638	2655	TCAAACACGGAGGAGAAGGC	59.965	CGCACAAGAGGCGATTCATG	59.973	133
TRINITY_Contig8819	7	p4	(AGCT)4	16	2908	2923	TCAAACACGGAGGAGAAGGC	59.965	CGCACAAGAGGCGATTCATG	59.973	133
TRINITY_Contig8821	1	p4	(ACAG)3	12	200	211	CTACGCGTGCCACGTAAATG	59.976	TTGCAAAGCCAGTTGATGCC	59.966	101
TRINITY_Contig8821	2	p3	(CGG)4	12	2759	2770	CTACGCGTGCCACGTAAATG	59.976	TTGCAAAGCCAGTTGATGCC	59.966	101
TRINITY_Contig8821	3	p3	(CCT)5	15	2872	2886	CTACGCGTGCCACGTAAATG	59.976	TTGCAAAGCCAGTTGATGCC	59.966	101
TRINITY_Contig8822	1	p4	(CAAC)3	12	300	311	GGCCAATCGGAAAGGAGGAA	60.034	TCGTCTCCGCTTCGAAACAA	59.969	189
TRINITY_Contig8822	2	c	(CGC)4caccctcgtcctcagcggaaggagcaggagcgggcgccatagaaaccccaccgggattcccccaatcttccttcagagaaccctgt(GGAC)3	110	2288	2397	GGCCAATCGGAAAGGAGGAA	60.034	TCGTCTCCGCTTCGAAACAA	59.969	189
TRINITY_Contig8823	1	p2	(TC)28	56	187	242	ACTGACAGCAGCAGTGGATC	60.037	CTGAGGATGGTGTTGCTGGT	59.963	102
TRINITY_Contig8823	2	c	(CT)8ccccat(GCC)6	40	526	565	ACTGACAGCAGCAGTGGATC	60.037	CTGAGGATGGTGTTGCTGGT	59.963	102
TRINITY_Contig8823	3	c	(AGG)4cgc(AGG)4agcgcagcaggaagcagatgagcagg(AGC)4acaaccaccggctgctccagctccaacaacaagtgcaggaccaggcagacccgccgccgccccccgtcttccagctccagcacctgca(GGC)4cgtgaggcagcggggcctgcccgccgagtatgcccttctcgcgcccatgggcgacgccggccagtc(CCA)6	249	742	990	ACTGACAGCAGCAGTGGATC	60.037	CTGAGGATGGTGTTGCTGGT	59.963	102
TRINITY_Contig8823	4	p3	(GCG)6	18	1164	1181	ACTGACAGCAGCAGTGGATC	60.037	CTGAGGATGGTGTTGCTGGT	59.963	102
TRINITY_Contig8823	5	p3	(CGG)4	12	1879	1890	ACTGACAGCAGCAGTGGATC	60.037	CTGAGGATGGTGTTGCTGGT	59.963	102
TRINITY_Contig8823	6	p3	(GCA)4	12	2128	2139	ACTGACAGCAGCAGTGGATC	60.037	CTGAGGATGGTGTTGCTGGT	59.963	102
TRINITY_Contig8823	7	p3	(GCC)4	12	2257	2268	ACTGACAGCAGCAGTGGATC	60.037	CTGAGGATGGTGTTGCTGGT	59.963	102
TRINITY_Contig8823	8	p4	(GGTC)3	12	2510	2521	ACTGACAGCAGCAGTGGATC	60.037	CTGAGGATGGTGTTGCTGGT	59.963	102
TRINITY_Contig8824	1	p3	(GCG)5	15	1134	1148	GGCACGACCATCTATCTGGG	59.966	TCCCGCTTCTTTACCAACCC	59.963	245
TRINITY_Contig8824	2	p3	(CCG)6	18	1534	1551	GGCACGACCATCTATCTGGG	59.966	TCCCGCTTCTTTACCAACCC	59.963	245
TRINITY_Contig8824	3	p4	(TTCC)3	12	2537	2548	GGCACGACCATCTATCTGGG	59.966	TCCCGCTTCTTTACCAACCC	59.963	245
TRINITY_Contig8825	1	p3	(GCC)4	12	149	160	CCCAACGAATCCTGGTTCGA	60.037	GCCCATCCCATGAGTTTCCA	60.033	167
TRINITY_Contig8826	1	p3	(TGC)4	12	688	699	TTGGAACGAGGCCACTCTTC	59.965	ACGACGATGATATGTGCGCT	59.969	196
TRINITY_Contig8827	1	p3	(GCC)4	12	2745	2756	TCCTCTCCATCACCCTCCTG	60.032	CATGCTGCATTGGATCAGGC	59.968	277
TRINITY_Contig8827	2	p4	(GCTC)3	12	2893	2904	TCCTCTCCATCACCCTCCTG	60.032	CATGCTGCATTGGATCAGGC	59.968	277
TRINITY_Contig8828	1	p4	(ACTG)3	12	2162	2173	TAGGCTCCTCCTTGGTACCC	60.031	ATTGGAGTTGAGCTGGAGGC	60.035	227
TRINITY_Contig8829	1	p3	(ATG)4	12	933	944	ACCCAGGAGTCGGATGATGA	60.032	CAGCACCCAACAAAGCTTCC	59.967	235
TRINITY_Contig8829	2	p3	(AGG)4	12	1089	1100	ACCCAGGAGTCGGATGATGA	60.032	CAGCACCCAACAAAGCTTCC	59.967	235
TRINITY_Contig8830	1	p4	(GGCG)3	12	98	109	ATGGCCATCACTTTGCCTCA	59.96	AAGGCCCACAGACTGCATAC	60.035	255
TRINITY_Contig8832	1	p4	(AGCT)3	12	621	632	TCGGCTTCATGACACGAACA	59.967	GCCCCTTGTTGTGCTAGCTA	60.035	133
TRINITY_Contig8832	2	p5	(TCGTC)3	15	809	823	TCGGCTTCATGACACGAACA	59.967	GCCCCTTGTTGTGCTAGCTA	60.035	133
TRINITY_Contig8832	3	p5	(TCCAG)3	15	1334	1348	TCGGCTTCATGACACGAACA	59.967	GCCCCTTGTTGTGCTAGCTA	60.035	133
TRINITY_Contig8832	4	p4	(TTAT)3	12	1652	1663	TCGGCTTCATGACACGAACA	59.967	GCCCCTTGTTGTGCTAGCTA	60.035	133
TRINITY_Contig8832	5	p3	(GCA)4	12	1921	1932	TCGGCTTCATGACACGAACA	59.967	GCCCCTTGTTGTGCTAGCTA	60.035	133
TRINITY_Contig8832	6	p3	(GCG)5	15	2723	2737	TCGGCTTCATGACACGAACA	59.967	GCCCCTTGTTGTGCTAGCTA	60.035	133
TRINITY_Contig8833	1	p4	(GCGG)3	12	2686	2697	CGCAGTGTCTGGGTTACTGT	59.966	CTGCGAAGGATGCAATGCTC	59.971	187
TRINITY_Contig8834	1	c	(CGACGG)3cgagggtttgatttactactacag(CGA)4	54	1206	1259	GCACTACAGGTGGGGGAAAA	59.888	TCGCAGTAGAAGTGGGAGGA	59.961	210
TRINITY_Contig8834	2	p3	(CGG)4	12	1493	1504	GCACTACAGGTGGGGGAAAA	59.888	TCGCAGTAGAAGTGGGAGGA	59.961	210
TRINITY_Contig8834	3	p3	(GTC)4	12	2200	2211	GCACTACAGGTGGGGGAAAA	59.888	TCGCAGTAGAAGTGGGAGGA	59.961	210
TRINITY_Contig8836	1	p3	(CCT)4	12	246	257	ACCTGCAGTGTTCCAAACCA	60.034	GCCCTCACCTGATCGATGAG	59.967	144
TRINITY_Contig8836	2	p3	(GAC)4	12	405	416	ACCTGCAGTGTTCCAAACCA	60.034	GCCCTCACCTGATCGATGAG	59.967	144
TRINITY_Contig8837	1	p3	(TGA)4	12	1924	1935	GCAGGCGTTGATATTGCTGG	59.97	GCGACTCTTCTTCAGCCTGT	60.038	224
TRINITY_Contig8837	2	p2	(AT)6	12	2676	2687	GCAGGCGTTGATATTGCTGG	59.97	GCGACTCTTCTTCAGCCTGT	60.038	224
TRINITY_Contig8839	1	p3	(GCC)4	12	172	183	GGCTGTGCAACCTTTTGGAG	59.967	CTTGAGCGCCAAGTTTTGCT	59.969	192
TRINITY_Contig8839	2	p6	(GCAGTA)3	18	2383	2400	GGCTGTGCAACCTTTTGGAG	59.967	CTTGAGCGCCAAGTTTTGCT	59.969	192
TRINITY_Contig8841	1	p5	(CGTCT)3	15	85	99	GCTACCCTTGCATTGCATCG	59.97	GCACGTCGCCCTGGAATATA	59.967	146
TRINITY_Contig8841	2	p3	(GGC)5	15	1241	1255	GCTACCCTTGCATTGCATCG	59.97	GCACGTCGCCCTGGAATATA	59.967	146
TRINITY_Contig8841	3	p3	(TTC)4	12	2151	2162	GCTACCCTTGCATTGCATCG	59.97	GCACGTCGCCCTGGAATATA	59.967	146
TRINITY_Contig8842	1	p5	(CCGGC)3	15	2868	2882	GTGCATACACTCAGCTGGGT	60.036	TTTTCAAGGCTGTTGCTCGC	59.969	173
TRINITY_Contig8843	1	p6	(GCATAT)3	18	399	416	ACATTTGCTGATCCGCTCCA	60.035	TGAGTTTGAGGCCTCAGCTG	59.964	106
TRINITY_Contig8843	2	p3	(TCA)4	12	625	636	ACATTTGCTGATCCGCTCCA	60.035	TGAGTTTGAGGCCTCAGCTG	59.964	106
TRINITY_Contig8843	3	p2	(CG)6	12	2410	2421	ACATTTGCTGATCCGCTCCA	60.035	TGAGTTTGAGGCCTCAGCTG	59.964	106
TRINITY_Contig8843	4	p3	(CGC)5	15	2759	2773	ACATTTGCTGATCCGCTCCA	60.035	TGAGTTTGAGGCCTCAGCTG	59.964	106
TRINITY_Contig8844	1	p4	(CCGC)3	12	1804	1815	TGGCTGCAGACAACTCAGAG	59.966	GCACACCATGGAGAAGGACA	59.963	262
TRINITY_Contig8844	2	c	(CTC)7gccggcggcctctttataaacacctcctccccccgcccctctcctccccacacagctcacgcgcagcagtcaccaccacccacagagcacgggg(GA)7	129	2441	2569	TGGCTGCAGACAACTCAGAG	59.966	GCACACCATGGAGAAGGACA	59.963	262
TRINITY_Contig8844	3	c	(GCC)4gaccccgccgacctccgcgccgcgcacctcgcccgggcgtgagcgccgttccgcccgtccgatacagcataccaacgaa(GGGAAG)3	109	2858	2966	TGGCTGCAGACAACTCAGAG	59.966	GCACACCATGGAGAAGGACA	59.963	262
TRINITY_Contig8844	4	c	(GTGC)3gtggtgtgggctggacgccctgttctagctggtgctctcctaccggattcagatg(TAC)4	79	3097	3175	TGGCTGCAGACAACTCAGAG	59.966	GCACACCATGGAGAAGGACA	59.963	262
TRINITY_Contig8844	5	p4	(TTTA)3	12	3863	3874	TGGCTGCAGACAACTCAGAG	59.966	GCACACCATGGAGAAGGACA	59.963	262
TRINITY_Contig8845	1	p4	(CGCC)3	12	49	60	GGTATCGCTGGCCAGTCTAC	59.968	GTTTGTCCGCTTGTTCCCAC	59.97	138
TRINITY_Contig8846	1	p3	(CCA)4	12	2673	2684	TCCTCCACCGATGACCAGAT	60.032	CGGGCTGCACTTGATATTGC	59.97	239
TRINITY_Contig8848	1	p6	(AAGGCC)3	18	285	302	CTTAGCCCTGGAAGCTGCTT	60.035	AGGGCAATTCATCGGCTTCA	60.034	231
TRINITY_Contig8849	1	p3	(GGC)4	12	2509	2520	CGCTCCTTCTCCATCTCGAC	59.97	GTGCTCTCCGACAACCTTGA	59.966	157
TRINITY_Contig8851	1	c	(CCG)5ctgctctccttgcgctggtttttaatcgctccgcctccgcgcgcaggcctggcttgagggattcgtcaccgacggcggctacgcctgcgcctgccccg(CCT)4	125	538	662	GGTTTTGCGCGAAAGGAAGT	59.97	GATCCCGAACGCTGCTGATA	59.968	236
TRINITY_Contig8852	1	p3	(CTC)4	12	524	535	GTGGCTATCCGCAGACTCTC	59.969	GCCATTGTTCCAGCACGTTT	59.968	189
TRINITY_Contig8853	1	p3	(CGT)4	12	313	324	GTCGAATTTGCGAGGTCTGC	59.905	TGGGATCTAAGCCTGACGGA	60.033	239
TRINITY_Contig8853	2	p3	(GCC)4	12	648	659	GTCGAATTTGCGAGGTCTGC	59.905	TGGGATCTAAGCCTGACGGA	60.033	239
TRINITY_Contig8853	3	p5	(TGGCA)3	15	1084	1098	GTCGAATTTGCGAGGTCTGC	59.905	TGGGATCTAAGCCTGACGGA	60.033	239
TRINITY_Contig8853	4	c	(CGT)4catcatggagcagccgccggtggtgaactgccttggccacggcgctcgcgaagccggcggccgacacgcttg(CGC)4cccgggcgacgagggcgagcgagttggagacgtgctcgtaggcgtcgtcgagggacgcgaggaggccggccacccccgggccg(GAC)4	191	2037	2227	GTCGAATTTGCGAGGTCTGC	59.905	TGGGATCTAAGCCTGACGGA	60.033	239
TRINITY_Contig8853	5	c	(TGG)4cgcttgtgcccgtggcattgggttgtgccggcggtggtcaggaggaggagcgc(GAG)4	77	2714	2790	GTCGAATTTGCGAGGTCTGC	59.905	TGGGATCTAAGCCTGACGGA	60.033	239
TRINITY_Contig8854	1	p3	(AAG)6	18	1992	2009	TCAGACTTCAGCCGCCAATT	59.963	CAAGGAAACAGTGGCTGTGC	59.969	170
TRINITY_Contig8856	1	p3	(CTC)5	15	3820	3834	GACTCCTCCTCCTCCTCCTG	60.106	AGAGCGCCTAGTGGAGAAGA	60.034	174
TRINITY_Contig8857	1	p3	(CCG)4	12	298	309	GCCTCTGTTTATTGCTCGCG	59.973	CGAGCTACCGTTAGTCCGAC	59.973	227
TRINITY_Contig8857	2	c	(TCC)4at(CGC)4	26	450	475	GCCTCTGTTTATTGCTCGCG	59.973	CGAGCTACCGTTAGTCCGAC	59.973	227
TRINITY_Contig8857	3	p3	(CGC)5	15	1453	1467	GCCTCTGTTTATTGCTCGCG	59.973	CGAGCTACCGTTAGTCCGAC	59.973	227
TRINITY_Contig8859	1	p4	(TGGA)3	12	646	657	GCAGTTGCTTGGTCATGCAA	59.968	AAGCATCGTCAAGCTGTCCA	59.965	132
TRINITY_Contig8859	2	p4	(TTGC)3	12	2692	2703	GCAGTTGCTTGGTCATGCAA	59.968	AAGCATCGTCAAGCTGTCCA	59.965	132
TRINITY_Contig8860	1	p3	(CGC)4	12	99	110	GGTCGTAAAAAGGCCTCGGA	60.037	GTCCACCAGTTTTCGCAACC	59.97	160
TRINITY_Contig8861	1	p4	(TATC)15	60	284	343	CTGCACACCGAACGTGAAAG	60.042	CATGTGGAGGTACCCCACAC	60.035	204
TRINITY_Contig8861	2	p3	(GCG)4	12	2775	2786	CTGCACACCGAACGTGAAAG	60.042	CATGTGGAGGTACCCCACAC	60.035	204
TRINITY_Contig8862	1	c	(GCGG)3gcgagcagcggccgaggcgagagcggcggcggctgcagagagggatgaggtggtgggccgtggaaccggggcgcccagccgcgtgtgg(AGCGC)3	115	2699	2813	GGCCACGAGTTCTTCTGTCA	59.966	ATCGAATCTGTGGAGGGGGA	60.031	240
TRINITY_Contig8863	1	p3	(GCC)4	12	896	907	TTGCAACTTGCCAATGTCCG	59.968	CAACTGCTCCAAGCTCCTGA	59.964	226
TRINITY_Contig8863	2	p2	(GA)7	14	4426	4439	TTGCAACTTGCCAATGTCCG	59.968	CAACTGCTCCAAGCTCCTGA	59.964	226
TRINITY_Contig8864	1	p3	(TCT)4	12	969	980	CCAAAACGGGAAACAGCAGG	59.968	AGTGGCTTGATTCCATGGGG	60.033	156
TRINITY_Contig8864	2	p3	(CGC)6	18	1804	1821	CCAAAACGGGAAACAGCAGG	59.968	AGTGGCTTGATTCCATGGGG	60.033	156

TRINITY_Contig8864	3	p4	(GAAA)5	20	2353	2372	CCAAAACGGGAAACAGCAGG	59.968	AGTGGCTTGATTCCATGGGG	60.033	156
TRINITY_Contig8864	4	c	(CGA)4atgatggaaacgatct(GAG)4gattactggtagtattacggaggaacaggaaggggttttagcgaatggaggtggagtggagccagcggaagagactag(CCTC)3	129	2689	2817	CCAAAACGGGAAACAGCAGG	59.968	AGTGGCTTGATTCCATGGGG	60.033	156
TRINITY_Contig8865	1	p3	(GGC)7	21	1962	1982	CGAAGTCGACCTCCACGTAG	59.903	GCTCCCTTTCCCTTCCCTTC	60.034	267
TRINITY_Contig8865	2	c	(TGT)4tgctggtcg(CCT)5	36	2155	2190	CGAAGTCGACCTCCACGTAG	59.903	GCTCCCTTTCCCTTCCCTTC	60.034	267
TRINITY_Contig8865	3	c	(CTG)4gtggtccaccacgga(AGC)6	45	2343	2387	CGAAGTCGACCTCCACGTAG	59.903	GCTCCCTTTCCCTTCCCTTC	60.034	267
TRINITY_Contig8865	4	p4	(GGTG)3	12	2830	2841	CGAAGTCGACCTCCACGTAG	59.903	GCTCCCTTTCCCTTCCCTTC	60.034	267
TRINITY_Contig8865	5	p5	(GGAAG)3	15	2964	2978	CGAAGTCGACCTCCACGTAG	59.903	GCTCCCTTTCCCTTCCCTTC	60.034	267
TRINITY_Contig8865	6	p2	(TA)6	12	3117	3128	CGAAGTCGACCTCCACGTAG	59.903	GCTCCCTTTCCCTTCCCTTC	60.034	267
TRINITY_Contig8867	1	p4	(TCCC)3	12	896	907	GTGTTTTCACGCTGGCACAT	59.969	CCCCTTCGTCTTCTTGCCAT	60.035	149
TRINITY_Contig8867	2	p3	(TTG)12	36	2009	2044	GTGTTTTCACGCTGGCACAT	59.969	CCCCTTCGTCTTCTTGCCAT	60.035	149
TRINITY_Contig8868	1	p3	(AGG)4	12	229	240	AGGTGGATGGCTCCTGAGAT	60.03	AGGGTTTTGAGAGCCGATGG	60.035	203
TRINITY_Contig8869	1	p6	(CCTCCG)3	18	3	20	AATCGCCCATTCAGTGTGGT	59.962	GTGGCATCCAGAAGGGTTGA	59.962	226
TRINITY_Contig8869	2	c	(GCA)4gggcgacggcgggaccggcgctccgaccgcccctgcaggccccgtcgtcccgtcagacgtcagcgtc(GCT)5	94	411	504	AATCGCCCATTCAGTGTGGT	59.962	GTGGCATCCAGAAGGGTTGA	59.962	226
TRINITY_Contig8869	3	c	(GCG)5gccacgaccatctcggcgccggggctccggacgccccgtggtgttcgccggcggcgtcgtcggcctcgtcgtca(TCC)4	101	806	906	AATCGCCCATTCAGTGTGGT	59.962	GTGGCATCCAGAAGGGTTGA	59.962	226
TRINITY_Contig8872	1	p3	(CGC)4	12	140	151	TTAGGGAGTGGCACATGCAG	60.035	GAGGGGACGAGACCTTCTCT	60.034	132
TRINITY_Contig8872	2	c	(GAGGAT)5gatgggcatatcaggtcggatagtgatgatgacgatgaagacgacagc(GAT)4(GAC)4	102	644	745	TTAGGGAGTGGCACATGCAG	60.035	GAGGGGACGAGACCTTCTCT	60.034	132
TRINITY_Contig8876	1	p3	(CCG)4	12	520	531	TGCTGGCCGAAGTTGAAGAT	59.963	CTTGTAACCATGCCGGGACT	60.035	233
TRINITY_Contig8876	2	p3	(GCG)10	30	790	819	TGCTGGCCGAAGTTGAAGAT	59.963	CTTGTAACCATGCCGGGACT	60.035	233
TRINITY_Contig8876	3	p3	(GAT)4	12	2629	2640	TGCTGGCCGAAGTTGAAGAT	59.963	CTTGTAACCATGCCGGGACT	60.035	233
TRINITY_Contig8877	1	c	(CCG)5caccgatgggacgcaaagactcctcctcgtcgtcct(CCG)4	63	137	199	GGAGGTTAGGAGGAGCCAGA	60.032	GAAGTGAACACAACACGGGC	59.971	240
TRINITY_Contig8878	1	p3	(GGC)4	12	178	189	GGGATCAAACTGAGGTCGCA	60.036	CACCTGCATGTTCTTCACGC	60.11	266
TRINITY_Contig8880	1	p3	(GCG)6	18	2639	2656	TCCAGGGCATCCTTATCCCA	60.029	TCAGTACTTTGACGCACGCT	59.968	141
TRINITY_Contig8881	1	p4	(CCCT)3	12	592	603	GAGGAAGGCGATAGGTTCCG	59.968	CTCGCACTATGCAAATGGCC	59.97	228
TRINITY_Contig8882	1	c	(CCT)6cttcctcctcgtcttcaccgcct(CCG)4	53	2508	2560	AAGACTGCAGCATGGCTTCT	59.962	TAGCCCCTGAAGTGGATCGA	60.033	263
TRINITY_Contig8887	1	p5	(CCCCT)5	25	106	130	AGAGTCCCTGCTCCATGGAT	60.03	TCGTAGGTGTAGAGCCAGCT	60.034	138
TRINITY_Contig8887	2	p3	(TCA)4	12	454	465	AGAGTCCCTGCTCCATGGAT	60.03	TCGTAGGTGTAGAGCCAGCT	60.034	138
TRINITY_Contig8888	1	p3	(GCG)6	18	449	466	CGGAAGGCAAAGGGAGGAAT	60.034	TAGGGGAGGAGTTCGTGGAG	60.033	116
TRINITY_Contig8888	2	p5	(GCCGC)3	15	617	631	CGGAAGGCAAAGGGAGGAAT	60.034	TAGGGGAGGAGTTCGTGGAG	60.033	116
TRINITY_Contig8889	1	p5	(GCGGC)3	15	2679	2693	CCATGCTAGCGCCACATAGA	59.966	TATTCGCTCCTTTGCAGGCA	60.036	196
TRINITY_Contig8891	1	p3	(AGT)4	12	2230	2241	TCAGCCAATTTGCTCTCCGT	59.963	GTTGGAGTTGCTGCCTTTGG	59.967	107
TRINITY_Contig8891	2	c	(GAG)5caggggtattggaaggccagagg(CGGCGA)3	56	2543	2598	TCAGCCAATTTGCTCTCCGT	59.963	GTTGGAGTTGCTGCCTTTGG	59.967	107
TRINITY_Contig8894	1	p5	(GTAAA)3	15	2625	2639	CGGAGCAGTAGCATCTCCTG	59.968	CCAAGGATGCCTTCATGGGT	60.033	208
TRINITY_Contig8895	1	c	(GCC)4caataacgcgagcgccgaccccagcgagcttctcgccgcgatcccctcgatcagtgaccgcgcgccgagggtcccggcgtcctgtccc(GCG)4	112	381	492	GATCACCGTCGATGACCTCC	59.969	CCCCCTAAGAAGCCCAGTTG	60.034	262
TRINITY_Contig8895	2	p6	(GTGCCG)3	18	816	833	GATCACCGTCGATGACCTCC	59.969	CCCCCTAAGAAGCCCAGTTG	60.034	262
TRINITY_Contig8895	3	p6	(ATTCGC)3	18	2086	2103	GATCACCGTCGATGACCTCC	59.969	CCCCCTAAGAAGCCCAGTTG	60.034	262
TRINITY_Contig8896	1	p4	(CTCC)3	12	325	336	CTGCTGGCCTCTATCCATGG	59.964	GCAAGCAAGAACAAGCTGCT	59.968	120
TRINITY_Contig8896	2	p3	(GGC)5	15	696	710	CTGCTGGCCTCTATCCATGG	59.964	GCAAGCAAGAACAAGCTGCT	59.968	120
TRINITY_Contig8897	1	c	(CGC)5cgtccgccatccccatcacggcgcacgcc(GCG)4	56	847	902	TGGCGGCAATAGATCGAGAC	59.968	GTTTACCAACAACACCCGCC	59.969	120
TRINITY_Contig8900	1	p3	(CCG)5	15	1299	1313	TAATAAGAATCCCCGCCGGC	59.964	GAAACCCAAACCAAGACGCC	59.968	172
TRINITY_Contig8901	1	p6	(TCCTCA)3	18	1899	1916	ACAATGAGCTGAACCCGAGG	60.036	GGTGCCTTTGTTTCCGTTCC	59.968	126
TRINITY_Contig8904	1	p4	(CAAC)3	12	1650	1661	TGCACCTGAAACCCCTTGTT	60.033	GCTGAAGTCGGTCCACAAGA	59.966	242
TRINITY_Contig8907	1	p3	(CCG)4	12	119	130	CCCAATCCAGGAAGCACCAT	60.033	AACGCAAAAACATGGGACCG	59.969	221
TRINITY_Contig8907	2	p3	(GAG)4	12	511	522	CCCAATCCAGGAAGCACCAT	60.033	AACGCAAAAACATGGGACCG	59.969	221
TRINITY_Contig8910	1	p6	(CCGCCT)3	18	261	278	TTCGACATTGGCAGCAGGAT	60.035	CGGTGGGTATGACCTGATCG	59.968	226
TRINITY_Contig8912	1	p4	(ATGG)3	12	263	274	CCTCCAAGCTGCCAGATCAA	60.035	TTGTTGTTCAGCGCGACTTG	59.973	218
TRINITY_Contig8912	2	p3	(GCG)5	15	2223	2237	CCTCCAAGCTGCCAGATCAA	60.035	TTGTTGTTCAGCGCGACTTG	59.973	218
TRINITY_Contig8912	3	p2	(GA)7	14	2689	2702	CCTCCAAGCTGCCAGATCAA	60.035	TTGTTGTTCAGCGCGACTTG	59.973	218
TRINITY_Contig8913	1	p3	(CCG)4	12	445	456	TCAAGGACCACACGCTTCTC	59.966	GTCGTGATTGCGATGAAGGC	59.973	158
TRINITY_Contig8914	1	c	(GAGAC)4gagccagacgagaggacctgatttgttgttgccttagcgccgtgcgcccgtgccgcgccgcggggcggttgc(TGG)5	108	2470	2577	GTGCTCTTCACGTCCTCCAA	59.966	GCCTCCCTCAGCAACATTCT	60.035	116
TRINITY_Contig8915	1	p4	(GGCA)3	12	2309	2320	GTCCTGGCACAATCCCATGA	60.034	TCCAGGGCTACGTCAACAAC	59.966	137
TRINITY_Contig8916	1	p3	(GCT)4	12	1470	1481	TATGCGCCATCCAAAATGCG	59.969	AGCACCTGTCAACAGAGCTC	59.965	217
TRINITY_Contig8916	2	p3	(GTG)4	12	2001	2012	TATGCGCCATCCAAAATGCG	59.969	AGCACCTGTCAACAGAGCTC	59.965	217
TRINITY_Contig8917	1	p5	(TTGCA)4	20	213	232	TGTACGTAGCGGCAACTGTT	59.968	TCGCTCTCTTTGGTTGCCAT	59.963	202
TRINITY_Contig8917	2	p4	(GCAG)4	16	566	581	TGTACGTAGCGGCAACTGTT	59.968	TCGCTCTCTTTGGTTGCCAT	59.963	202
TRINITY_Contig8917	3	c	(CTTCG)4ccataatgttacctcagcgtgcatgcggtgccaaccaaccaccaccatgtacagcttaca(GGAG)3	92	795	886	TGTACGTAGCGGCAACTGTT	59.968	TCGCTCTCTTTGGTTGCCAT	59.963	202
TRINITY_Contig8917	4	p3	(GCG)6	18	1005	1022	TGTACGTAGCGGCAACTGTT	59.968	TCGCTCTCTTTGGTTGCCAT	59.963	202
TRINITY_Contig8917	5	p3	(GCC)5	15	1356	1370	TGTACGTAGCGGCAACTGTT	59.968	TCGCTCTCTTTGGTTGCCAT	59.963	202
TRINITY_Contig8917	6	c	(CCG)4ccatcagcgccgcgccggacccgcggctgcccaggcagtagaaccggatctgcagcgcgcgcgacgtcagtcgg(TCGA)3tcgccatgaagaagcaagcggcgcagaggactggag(GCC)4	146	1633	1778	TGTACGTAGCGGCAACTGTT	59.968	TCGCTCTCTTTGGTTGCCAT	59.963	202
TRINITY_Contig8917	7	p3	(GCC)4	12	2200	2211	TGTACGTAGCGGCAACTGTT	59.968	TCGCTCTCTTTGGTTGCCAT	59.963	202
TRINITY_Contig8917	8	p4	(GGAG)3	12	2393	2404	TGTACGTAGCGGCAACTGTT	59.968	TCGCTCTCTTTGGTTGCCAT	59.963	202
TRINITY_Contig8917	9	p2	(GA)8	16	2514	2529	TGTACGTAGCGGCAACTGTT	59.968	TCGCTCTCTTTGGTTGCCAT	59.963	202
TRINITY_Contig8918	1	p3	(GGT)4	12	97	108	AAACGTAGACAGCAGCCCTC	60.037	TGGATGCTGGTAATGCTGCA	60.034	253
TRINITY_Contig8918	2	c	(GCG)5gggaccgtgggagcacggcgagctcgccgatgctgaatgcgctccccaccgggaacatctgcccgtccgggcgcatcc(CGG)4	105	585	689	AAACGTAGACAGCAGCCCTC	60.037	TGGATGCTGGTAATGCTGCA	60.034	253
TRINITY_Contig8920	1	p6	(CGGGAA)3	18	1858	1875	AATCGGGAAATTCGGCTGGT	60.034	GGTTTTTGTCCGTTCCTGGC	59.968	266
TRINITY_Contig8920	2	p3	(TCC)4	12	2069	2080	AATCGGGAAATTCGGCTGGT	60.034	GGTTTTTGTCCGTTCCTGGC	59.968	266
TRINITY_Contig8922	1	c	(CTGC)3cgctcgccccccgaattttggagccccgcacgg(CGTC)3	58	629	686	TGACACCGTACCCCGTATCT	60.034	GTCCGCGGTTTTGAGCTTTT	59.97	179
TRINITY_Contig8922	2	p4	(AAGC)3	12	856	867	TGACACCGTACCCCGTATCT	60.034	GTCCGCGGTTTTGAGCTTTT	59.97	179
TRINITY_Contig8922	3	p3	(AGC)4	12	969	980	TGACACCGTACCCCGTATCT	60.034	GTCCGCGGTTTTGAGCTTTT	59.97	179
TRINITY_Contig8922	4	p3	(GAG)4	12	1394	1405	TGACACCGTACCCCGTATCT	60.034	GTCCGCGGTTTTGAGCTTTT	59.97	179
TRINITY_Contig8923	1	c	(CGC)4(AGG)6	30	153	182	GTGGCGAAGATCATGAACGC	59.973	GAATCCGTGTGGGTGAACCT	59.964	275
TRINITY_Contig8923	2	p4	(CCGC)3	12	285	296	GTGGCGAAGATCATGAACGC	59.973	GAATCCGTGTGGGTGAACCT	59.964	275
TRINITY_Contig8923	3	c*	(CTG)4cacctcctcctcatcgtcgc(CCT)4(GCG)4caggcgcaggcgccgaccaccacct(CCA)5(CCACAG)3*	111	450	560	GTGGCGAAGATCATGAACGC	59.973	GAATCCGTGTGGGTGAACCT	59.964	275
TRINITY_Contig8924	1	c	(GCA)5aagcaagcaggccagcagccgcccagagcggagccag(CTCGC)3ctcctcagtcgcctcgcctccacttctatatcacttccccctctgcccccgccccgtgcgccatcgcgcacatcca(CG)6cccacacgtgcggctccccggattcgcgccgccgcggcag(AGCC)3	207	20	226	GGAGCGGTTCAAGTCCTTCA	59.965	TATGCGGGGTAGGAGGAACA	60.032	263
TRINITY_Contig8924	2	p3	(CCG)5	15	536	550	GGAGCGGTTCAAGTCCTTCA	59.965	TATGCGGGGTAGGAGGAACA	60.032	263
TRINITY_Contig8924	3	p3	(CCG)4	12	1115	1126	GGAGCGGTTCAAGTCCTTCA	59.965	TATGCGGGGTAGGAGGAACA	60.032	263
TRINITY_Contig8928	1	c	(GGCG)3gtggaggagtcgatgctgtagtccgaggagcagtcgtcgtagtcgtcgccgctgtcgaaggcgagctgccggaaccgggacatgttctgggagcctcct(CCG)4	123	553	675	ATGGTAGGGGAGTAGCGGTT	60.031	CGGACTACAGCATCGACTCC	59.97	233
TRINITY_Contig8928	2	p3	(CTT)4	12	2000	2011	ATGGTAGGGGAGTAGCGGTT	60.031	CGGACTACAGCATCGACTCC	59.97	233
TRINITY_Contig8929	1	p3	(CGG)7	21	2624	2644	ACACTGATCTCCACGTGCTG	60.038	GACAAGTTTGGACGCACGTC	60.042	204

TRINITY_Contig8930	1	c	(CGC)4ggggggcgggggcagcggcgggga cgcgggcatcgagtgctcccctccgattccccc cgcccct(CCG)5	91	2184	2274	GAAGAGATAGCGTCGCCGAA	59.971	AACATGGTGTTCCGCATCCT	59.962	149
TRINITY_Contig8931	1	p2	(AT)8	16	2026	2041	ATATGACCTGGGACCCGGAA	60.031	CATATGCTACCGCCCCTCTG	60.037	271
TRINITY_Contig8931	2	p4	(GGCG)3	12	2628	2639	ATATGACCTGGGACCCGGAA	60.031	CATATGCTACCGCCCCTCTG	60.037	271
TRINITY_Contig8932	1	c	(CCT)5ccgcgcgcaaccgcccacgcagaca cgcgcacgcgctagaatggagtcgagaagag agc(GGT)4	86	21	106	CAAAAGGCTCCTGTTGGCAC	59.967	CAATGGCATACTGAACGCGG	59.972	193
TRINITY_Contig8932	2	c	(CGCC)4ggagcgaagggaagagggggaat gcaggcggcgcgccacggcccggcggcg(G GC)4	79	237	315	CAAAAGGCTCCTGTTGGCAC	59.967	CAATGGCATACTGAACGCGG	59.972	193
TRINITY_Contig8932	3	p3	(GCT)5	15	4205	4219	CAAAAGGCTCCTGTTGGCAC	59.967	CAATGGCATACTGAACGCGG	59.972	193
TRINITY_Contig8932	4	p3	(GCG)6	18	4557	4574	CAAAAGGCTCCTGTTGGCAC	59.967	CAATGGCATACTGAACGCGG	59.972	193
TRINITY_Contig8933	1	p3	(CCA)4	12	1031	1042	CGATGGAGTAGGAGCCGAAC	59.969	TGCCCTCCTACCATCATCCA	60.03	179
TRINITY_Contig8933	2	c	(AG)7aagatgtgccaagcagaggtgtgctg atgatgtgctactg(GGA)4	66	2595	2660	CGATGGAGTAGGAGCCGAAC	59.969	TGCCCTCCTACCATCATCCA	60.03	179
TRINITY_Contig8934	1	p3	(GCT)7	21	204	224	GACCGTTGCGATTCATGCTC	59.973	TGCTCGACAAAACACAAGCG	59.973	194
TRINITY_Contig8934	2	p3	(CCG)5	15	577	591	GACCGTTGCGATTCATGCTC	59.973	TGCTCGACAAAACACAAGCG	59.973	194
TRINITY_Contig8934	3	p3	(CGA)4	12	2252	2263	GACCGTTGCGATTCATGCTC	59.973	TGCTCGACAAAACACAAGCG	59.973	194
TRINITY_Contig8934	4	p5	(GAGCA)3	15	2522	2536	GACCGTTGCGATTCATGCTC	59.973	TGCTCGACAAAACACAAGCG	59.973	194
TRINITY_Contig8935	1	p5	(CCTCC)3	15	15	29	CCTTTTTGCCGGAGTTGTGG	59.968	CACCACAAGCTTGTCCTTGC	59.969	112
TRINITY_Contig8936	1	p2	(AT)9	18	1139	1156	CCTAGCACCACTGTGTCAGG	60.037	TCCCGCTGTTTCCATACCAC	60.036	106
TRINITY_Contig8936	2	p3	(CCG)4	12	2098	2109	CCTAGCACCACTGTGTCAGG	60.037	TCCCGCTGTTTCCATACCAC	60.036	106
TRINITY_Contig8936	3	p4	(GGCG)3	12	2244	2255	CCTAGCACCACTGTGTCAGG	60.037	TCCCGCTGTTTCCATACCAC	60.036	106
TRINITY_Contig8940	1	p3	(CGA)4	12	164	175	CCTGACGTGTGGGACTGTAC	60.039	TAGAGACGCGCCCTGAATTC	59.898	267
TRINITY_Contig8940	2	c	(GCT)5tcg(TTC)4	30	628	657	CCTGACGTGTGGGACTGTAC	60.039	TAGAGACGCGCCCTGAATTC	59.898	267
TRINITY_Contig8940	3	p4	(CTGC)3	12	805	816	CCTGACGTGTGGGACTGTAC	60.039	TAGAGACGCGCCCTGAATTC	59.898	267
TRINITY_Contig8940	4	p6	(CACCAG)3	18	1722	1739	CCTGACGTGTGGGACTGTAC	60.039	TAGAGACGCGCCCTGAATTC	59.898	267
TRINITY_Contig8941	1	c	(CGCT)3cggcgtggatccccgccgctagagt agaggtgggagtcggtccctcccggcggcgag gcgagcgggtcggccgcc(GTGGG)3	102	2461	2562	GGAGGGACAGGAGAGCAGTA	60.033	ACCGACTCCCACCTCTACTC	60.034	235
TRINITY_Contig8942	1	p3	(AAG)5	15	240	254	CCAACCAAGAGACCCCTTCC	59.962	GCCCAACAAATCACAGCGTT	59.968	218
TRINITY_Contig8942	2	p3	(CCG)4	12	1302	1313	CCAACCAAGAGACCCCTTCC	59.962	GCCCAACAAATCACAGCGTT	59.968	218
TRINITY_Contig8944	1	p2	(CT)9	18	319	336	CAGCAGGTCCAGCTTCTTGA	59.964	TTCGCCAAGCAGAAGTACGT	59.967	177
TRINITY_Contig8944	2	p3	(CGC)4	12	588	599	CAGCAGGTCCAGCTTCTTGA	59.964	TTCGCCAAGCAGAAGTACGT	59.967	177
TRINITY_Contig8944	3	p4	(GAAG)4	16	2126	2141	CAGCAGGTCCAGCTTCTTGA	59.964	TTCGCCAAGCAGAAGTACGT	59.967	177
TRINITY_Contig8945	1	c	(CTT)4cgccgtcgtcataagaagaccaccgc ttattttgctgcgactgaacaca(ATGG)3	73	819	891	GGCGTGAGTATGAGAGAGCC	59.969	GTCCGTTCACAACTCCTCGT	59.968	191
TRINITY_Contig8945	2	p3	(GAA)4	12	1023	1034	GGCGTGAGTATGAGAGAGCC	59.969	GTCCGTTCACAACTCCTCGT	59.968	191
TRINITY_Contig8945	3	p4	(GGCT)4	16	1163	1178	GGCGTGAGTATGAGAGAGCC	59.969	GTCCGTTCACAACTCCTCGT	59.968	191
TRINITY_Contig8945	4	p3	(CCG)4	12	2105	2116	GGCGTGAGTATGAGAGAGCC	59.969	GTCCGTTCACAACTCCTCGT	59.968	191
TRINITY_Contig8945	5	p3	(CAG)5	15	2260	2274	GGCGTGAGTATGAGAGAGCC	59.969	GTCCGTTCACAACTCCTCGT	59.968	191
TRINITY_Contig8945	6	p2	(GA)6	12	2552	2563	GGCGTGAGTATGAGAGAGCC	59.969	GTCCGTTCACAACTCCTCGT	59.968	191
TRINITY_Contig8947	1	p3	(CGG)4	12	990	1001	CCAACGAGACAGACGGTTCA	59.968	ATGTGCATGGTGGAGACCTG	60.035	171
TRINITY_Contig8947	2	p3	(GCG)4	12	1448	1459	CCAACGAGACAGACGGTTCA	59.968	ATGTGCATGGTGGAGACCTG	60.035	171
TRINITY_Contig8947	3	p4	(CCCG)3	12	1762	1773	CCAACGAGACAGACGGTTCA	59.968	ATGTGCATGGTGGAGACCTG	60.035	171
TRINITY_Contig8947	4	c	(TGCC)3tgtgggtgtgtcggt(GCG)4	39	1965	2003	CCAACGAGACAGACGGTTCA	59.968	ATGTGCATGGTGGAGACCTG	60.035	171
TRINITY_Contig8948	1	p3	(GCT)5	15	469	483	TCCTTCGCTGACACCTTCAC	59.966	TCAGGGTGAGAGGGAGATGG	60.032	147
TRINITY_Contig8948	2	c	(CGG)5cgtgagggggagggggaggaggat gcgaggtccgaaaaaccctagtg(GGC)4	74	2493	2566	TCCTTCGCTGACACCTTCAC	59.966	TCAGGGTGAGAGGGAGATGG	60.032	147
TRINITY_Contig8949	1	p3	(GGT)4	12	1654	1665	TTCTCGCCTTTCCTTCGCTT	59.965	TGATGAGGTCCTCACGCCTA	60.034	163
TRINITY_Contig8949	2	c	(CGG)4gggctggtagtacatctgcggcggg ggcggggcctgctgcgggggcccggggccga aattgaactgcggctggt(GCG)4ggccggg tggtggcggcggcggggcacccgcgtggtaa ggcggcgccggcatccggccgtacggcgcct(GCG)6cgaacgggttcatctgcggcgggaag ccaccctgcggtggcgggggcggcgggtgctg ctgcggcggcgggtacggccggtacgacgcc atccc(GCG)4	291	2000	2290	TTCTCGCCTTTCCTTCGCTT	59.965	TGATGAGGTCCTCACGCCTA	60.034	163
TRINITY_Contig8951	1	p3	(GCA)4	12	267	278	GCGTGTCCCTTCAAACCAAC	59.97	AACATGAACCGGTGCTGCTA	59.964	123
TRINITY_Contig8951	2	p3	(TCA)6	18	2041	2058	GCGTGTCCCTTCAAACCAAC	59.97	AACATGAACCGGTGCTGCTA	59.964	123
TRINITY_Contig8952	1	p4	(TTCT)3	12	2322	2333	ATTGGTTCATGGACGTCGCT	60.036	GCCGTGGGATTTGAACGATG	59.901	137
TRINITY_Contig8953	1	p3	(GCA)4	12	325	336	TCGTTACATGCACACACGGA	59.968	GTCGGAGATGCTGCTCTACC	59.969	242
TRINITY_Contig8955	1	c	(GTGCG)3c(CGAGG)3	31	302	332	GAACGAATCGGATGTGCTGC	59.973	CCACAGGAGAGTCCAACACC	59.965	123
TRINITY_Contig8955	2	p3	(GGC)4	12	477	488	GAACGAATCGGATGTGCTGC	59.973	CCACAGGAGAGTCCAACACC	59.965	123
TRINITY_Contig8955	3	p3	(GCA)4	12	683	694	GAACGAATCGGATGTGCTGC	59.973	CCACAGGAGAGTCCAACACC	59.965	123
TRINITY_Contig8956	1	p5	(TCAGA)6	30	306	335	AGATGCACATCGGTCAGCAA	60.036	TCCTCTCCGTGTTTCGTGTG	59.968	227
TRINITY_Contig8956	2	p3	(CGC)4	12	991	1002	AGATGCACATCGGTCAGCAA	60.036	TCCTCTCCGTGTTTCGTGTG	59.968	227
TRINITY_Contig8956	3	p3	(GCG)4	12	1746	1757	AGATGCACATCGGTCAGCAA	60.036	TCCTCTCCGTGTTTCGTGTG	59.968	227
TRINITY_Contig8956	4	c	(CGG)5catggcgcggcgcgcggcgggagg ggtggggaaaaaatcggaaatttgggggcgtt ggcgcgtggagtgcgggacggcgctgaatca gcgcgccaacta(CT)6	126	2222	2347	AGATGCACATCGGTCAGCAA	60.036	TCCTCTCCGTGTTTCGTGTG	59.968	227
TRINITY_Contig8957	1	c	(GCT)4actcctgctcttctcggtgcgagggca gaccgccgtggctccgcatggtgtgcgtgctcg acgtcgtcatcgcgtt(CGACGC)3	106	394	499	ATGGATTTGCCCTCTTGCGA	60.034	GTGCATGCATCATCGGACAC	59.971	131
TRINITY_Contig8958	1	p3	(CTC)5	15	64	78	TGGGGTTTTGGAGGCTTTGT	60.032	CAATAATGCAGGCCTTCCGC	59.969	236
TRINITY_Contig8958	2	p3	(CTG)4	12	2504	2515	TGGGGTTTTGGAGGCTTTGT	60.032	CAATAATGCAGGCCTTCCGC	59.969	236
TRINITY_Contig8960	1	p3	(CGG)4	12	2154	2165	GCGATGGAGACGAAGACGAT	59.97	ACTGCAAGTTGGCATGCTTG	59.967	205
TRINITY_Contig8963	1	p3	(CGG)4	12	1993	2004	AGTGAAGCATCATGGCAGCT	60.034	TGAAATGCCCGTTGCCTTTG	59.967	273
TRINITY_Contig8963	2	p3	(CGC)4	12	2571	2582	AGTGAAGCATCATGGCAGCT	60.034	TGAAATGCCCGTTGCCTTTG	59.967	273
TRINITY_Contig8964	1	p2	(CG)7	14	1484	1497	GCCAAGCCAAAGGAATGCAA	59.965	CAACTACTTCAGGGCGCTCA	60.037	239
TRINITY_Contig8964	2	p3	(CCT)5	15	1839	1853	GCCAAGCCAAAGGAATGCAA	59.965	CAACTACTTCAGGGCGCTCA	60.037	239
TRINITY_Contig8964	3	p3	(CCG)4	12	2310	2321	GCCAAGCCAAAGGAATGCAA	59.965	CAACTACTTCAGGGCGCTCA	60.037	239
TRINITY_Contig8969	1	p3	(TTC)4	12	276	287	GCTTGTTTCGCGAACCCTTT	59.97	CAGCAAAAGGTTCGCAACGA	59.971	207
TRINITY_Contig8970	1	p3	(CGC)4	12	117	128	CCAGGGCAGCAAACTTCAAC	59.967	CGCCTTGACCATAACCACCT	60.035	181
TRINITY_Contig8970	2	p6	(GCGGTG)3	18	351	368	CCAGGGCAGCAAACTTCAAC	59.967	CGCCTTGACCATAACCACCT	60.035	181
TRINITY_Contig8972	1	p5	(CGCCG)3	15	15	29	GCTTCAGACACGTTCCTCCA	59.966	CAGGTTTCAACAAGGTGCGG	59.969	193
TRINITY_Contig8973	1	p3	(GCA)4	12	427	438	CTTTCCGTCCTCCACTGTCC	60.037	GATCATACCGACCATGCCGT	59.966	101
TRINITY_Contig8973	2	p3	(AAT)4	12	2085	2096	CTTTCCGTCCTCCACTGTCC	60.037	GATCATACCGACCATGCCGT	59.966	101
TRINITY_Contig8974	1	p3	(AGA)4	12	425	436	GACTGGACACCGCAAGAGAA	59.966	CCAAGGGAAGCAAGGAGGAG	60.035	144
TRINITY_Contig8974	2	p3	(CGG)4	12	618	629	GACTGGACACCGCAAGAGAA	59.966	CCAAGGGAAGCAAGGAGGAG	60.035	144
TRINITY_Contig8975	1	p3	(GAG)4	12	2195	2206	AGGTCTTGCAGCTAAGGCAG	60.036	CAACAATGAGGGGCAGGCTA	60.034	140
TRINITY_Contig8978	1	p3	(AGG)4	12	2305	2316	AGCCCTCCAGGATAGCTTGA	60.031	ACCTGTTTGAACACTGGCCA	60.034	100
TRINITY_Contig8979	1	p4	(AACG)3	12	338	349	CACCCCTCCTGGATGAACAC	60.035	CCAAGGTCAAGGTCACCTCC	59.963	154
TRINITY_Contig8979	2	p3	(CGC)4	12	2282	2293	CACCCCTCCTGGATGAACAC	60.035	CCAAGGTCAAGGTCACCTCC	59.963	154
TRINITY_Contig8979	3	p4	(GGCG)3	12	2479	2490	CACCCCTCCTGGATGAACAC	60.035	CCAAGGTCAAGGTCACCTCC	59.963	154
TRINITY_Contig8981	1	p3	(GAG)4	12	1509	1520	CGTCCTACTCCATCACGGTG	59.899	ATGTTCTGGTGCGTGTAGGG	60.036	223
TRINITY_Contig8981	2	p4	(CATA)3	12	1701	1712	CGTCCTACTCCATCACGGTG	59.899	ATGTTCTGGTGCGTGTAGGG	60.036	223
TRINITY_Contig8981	3	p4	(CAAT)3	12	1867	1878	CGTCCTACTCCATCACGGTG	59.899	ATGTTCTGGTGCGTGTAGGG	60.036	223
TRINITY_Contig8982	1	p3	(CTC)4	12	51	62	ATCTGCAAGGTGTTCGAGGG	60.036	GGAAGATCGTCTCCGTCGTC	59.972	230
TRINITY_Contig8982	2	p3	(GCT)4	12	1553	1564	ATCTGCAAGGTGTTCGAGGG	60.036	GGAAGATCGTCTCCGTCGTC	59.972	230
TRINITY_Contig8983	1	p3	(CGG)4	12	2241	2252	GAACCAGGTCATCGCCTTGA	60.036	GAAGGCCATGGACAAGCTCT	60.035	277
TRINITY_Contig8985	1	c	(CGGAA)3cgagacgggacgagaaggaag cagcgcaaggccggacggacctccacggcag cccagctcagttcaactgctcggggcggtggg gggcaacgagaggt(GGA)6	131	2399	2529	TGACAGTCGTGAAGGTGACG	59.97	ACAGCCACCAGTTGAACACA	60.035	222
TRINITY_Contig8986	1	p6	(CCGGCG)3	18	2372	2389	GCGTAGTCCATGAGCTCCTC	59.969	CCGGCCACTACGTCATCTAC	59.97	214
TRINITY_Contig8986	2	p4	(GGAG)3	12	2517	2528	GCGTAGTCCATGAGCTCCTC	59.969	CCGGCCACTACGTCATCTAC	59.97	214
TRINITY_Contig8987	1	p3	(CGC)4	12	263	274	TCCGGCGTCGAAAAACAAAC	59.972	GGACATCGACGGTGCTAGAG	59.97	244
TRINITY_Contig8988	1	p4	(GCAG)3	12	2402	2413	GATGCGACTAGTGATCGGCA	59.969	TGCTCCGTTGAGTGAAGGAC	59.966	146

TRINITY_Contig8989	1	p3	(GCC)5	15	1877	1891	TGTTCCTGTCGACTGAACGG	59.968	CTCTCGTGGAGGAACCCCTA	60.033	139
TRINITY_Contig8989	2	p5	(ACCCT)3	15	2198	2212	TGTTCCTGTCGACTGAACGG	59.968	CTCTCGTGGAGGAACCCCTA	60.033	139
TRINITY_Contig8991	1	c	(ACGGG)4acgaccctttcggcggcccgtccccaatcccccatccgtggcacgaggccacggca(GCGCCT)3gcgcgcacgccaacccgacgcgaccggcggtgggcagcggcgcaccaacgcaaagccaacccaccccgccccgaccacacccactcacgagg(CCGC)3	198	69	266	TACCGTACAGCAGGAGACCA	59.961	GTGACATGGCTCCCAGACAA	59.963	183
TRINITY_Contig8992	1	p3	(TGG)4	12	1664	1675	CTAGGGCAGGCGAGAGATTG	59.967	GCACAGGAGGAGAGAGAGGA	60.034	107
TRINITY_Contig8992	2	p3	(CCG)4	12	2217	2228	CTAGGGCAGGCGAGAGATTG	59.967	GCACAGGAGGAGAGAGAGGA	60.034	107
TRINITY_Contig8992	3	c	(TC)8ccccctccctgctccctcctctctcctcctgtgccgctctgcctcctctctcctcctgtgccgctctg(TC)8	104	2424	2527	CTAGGGCAGGCGAGAGATTG	59.967	GCACAGGAGGAGAGAGAGGA	60.034	107
TRINITY_Contig8993	1	c	(CCG)4cagtgagcagcgc(CAG)4	37	2393	2429	CAATTGCTGTAGCCATCGCC	59.97	CGGCAATATCGAGGGAAGCT	59.966	251
TRINITY_Contig8997	1	p3	(TCC)4	12	133	144	ATCTCCTGCACCAAGTCTGC	60.036	CTTTTGCGTGACCAAGGTGG	59.969	152
TRINITY_Contig8998	1	p4	(ACAA)3	12	814	825	GTACCTGGGCAGAAACAGCT	59.963	ACATCAATGGCTGCTCGACA	60.036	115
TRINITY_Contig8998	2	p3	(GAA)4	12	1251	1262	GTACCTGGGCAGAAACAGCT	59.963	ACATCAATGGCTGCTCGACA	60.036	115
TRINITY_Contig9000	1	p3	(CGT)6	18	1912	1929	CTTGGCGATCTTGCGGAATG	59.971	TGGGAGCCTGAAAGATGCAG	60.035	109
TRINITY_Contig9002	1	p3	(GCG)4	12	2333	2344	GGAGCAGCGACTCCTGTATC	59.969	AAGAACCTCAGAGTGTGCGG	59.966	270
TRINITY_Contig9004	1	p3	(TTC)4	12	1767	1778	GTCCTTCCTGCACCATGACA	59.963	GGAGAGAGTGGGGGTGGTAT	60.031	100
TRINITY_Contig9004	2	p6	(ACCGCC)4	24	2177	2200	GTCCTTCCTGCACCATGACA	59.963	GGAGAGAGTGGGGGTGGTAT	60.031	100
TRINITY_Contig9005	1	p3	(AAG)4	12	1492	1503	ACGTGAACTGGAGAGCTTGG	59.966	TCTTCCTCGCGCTGATCATC	59.969	195
TRINITY_Contig9005	2	p3	(GAA)4	12	2178	2189	ACGTGAACTGGAGAGCTTGG	59.966	TCTTCCTCGCGCTGATCATC	59.969	195
TRINITY_Contig9007	1	p4	(CAGG)3	12	2387	2398	CGCGGTGTAGTAGACGTACC	59.973	GCCTCCCTCCCCTGTAAAAC	60.034	226
TRINITY_Contig9008	1	p2	(GA)14	28	2	29	TTTGGATGTCGTCGTGCTCA	59.967	AAGTGAGTCCATGGGGCATG	60.034	119
TRINITY_Contig9008	2	p3	(CGC)4	12	518	529	TTTGGATGTCGTCGTGCTCA	59.967	AAGTGAGTCCATGGGGCATG	60.034	119
TRINITY_Contig9009	1	p3	(CGC)5	15	63	77	GGCCAAGAAGATTCCGGTCA	60.035	ACACTCAAACATTGCGGCAC	59.969	206
TRINITY_Contig9009	2	p3	(GTC)4	12	458	469	GGCCAAGAAGATTCCGGTCA	60.035	ACACTCAAACATTGCGGCAC	59.969	206
TRINITY_Contig9011	1	p3	(CGC)4	12	2328	2339	CGTAAGGCGATCGATTCCCA	59.968	AGCAAAGTGGCATAGGTCCC	60.034	250
TRINITY_Contig9012	1	p4	(TCTG)4	16	188	203	ATGGGTTCGTTGCTGGAGAG	60.036	GCACAGAGAGAACACCGTGA	59.968	174
TRINITY_Contig9012	2	p3	(CTG)4	12	1855	1866	ATGGGTTCGTTGCTGGAGAG	60.036	GCACAGAGAGAACACCGTGA	59.968	174
TRINITY_Contig9012	3	p4	(GATG)5	20	3448	3467	ATGGGTTCGTTGCTGGAGAG	60.036	GCACAGAGAGAACACCGTGA	59.968	174
TRINITY_Contig9017	1	p6	(ATAAAG)4	24	208	231	TGCTTCCTGGTGGTGTGATC	59.963	AATCGCCCATAACGAGCGAT	59.966	168
TRINITY_Contig9017	2	p4	(CTTG)3	12	2109	2120	TGCTTCCTGGTGGTGTGATC	59.963	AATCGCCCATAACGAGCGAT	59.966	168
TRINITY_Contig9021	1	p3	(CAG)5	15	1127	1141	TGTCGAAAGGACCACAGACG	59.968	TCAGGGATCTGATCTGGCA	60.031	207
TRINITY_Contig9022	1	p4	(GCCG)4	16	1153	1168	GTCGCCCAAGTTCAAGGAGA	59.965	GTCAACCTCTGCGAAGGTGA	59.966	259
TRINITY_Contig9022	2	p6	(CTCCCA)3	18	1714	1731	GTCGCCCAAGTTCAAGGAGA	59.965	GTCAACCTCTGCGAAGGTGA	59.966	259
TRINITY_Contig9022	3	p3	(CAG)4	12	2001	2012	GTCGCCCAAGTTCAAGGAGA	59.965	GTCAACCTCTGCGAAGGTGA	59.966	259
TRINITY_Contig9023	1	p4	(CCAT)6	24	3213	3236	AGGATGAGACCCTGCCTGAT	60.03	CGCCCAATCATAAGCTGCAC	59.97	209
TRINITY_Contig9024	1	c	(TTG)4(CTG)6	30	2230	2259	ACACGAGAAGCAGGGAATGG	60.036	TGCAATATGATGCTGTGCGC	59.966	224
TRINITY_Contig9025	1	p5	(TTTCT)4	20	2	21	AGATGAGGCTGCTGAGGAGA	60.032	AAGAATGTGGTTCGACGCCT	59.965	190
TRINITY_Contig9025	2	c	(ACC)6(GCT)5	33	1282	1314	AGATGAGGCTGCTGAGGAGA	60.032	AAGAATGTGGTTCGACGCCT	59.965	190
TRINITY_Contig9025	3	p3	(GGT)8	24	2383	2406	AGATGAGGCTGCTGAGGAGA	60.032	AAGAATGTGGTTCGACGCCT	59.965	190
TRINITY_Contig9026	1	p3	(TCC)4	12	192	203	GCCGAACATGAGGTCCTTGA	60.036	GACCAAGATCTCGCTCGAGG	59.97	145
TRINITY_Contig9026	2	c	(CTC)4cgcgccgccgaacatgaggtccttgacgctccgctgctccgccaccgcgccgccgcgccgggcg(GCC)5	91	1169	1259	GCCGAACATGAGGTCCTTGA	60.036	GACCAAGATCTCGCTCGAGG	59.97	145
TRINITY_Contig9026	3	c	(AGGCG)3cgcgtgc(GCGG)4	38	1875	1912	GCCGAACATGAGGTCCTTGA	60.036	GACCAAGATCTCGCTCGAGG	59.97	145
TRINITY_Contig9026	4	p3	(GAA)4	12	2113	2124	GCCGAACATGAGGTCCTTGA	60.036	GACCAAGATCTCGCTCGAGG	59.97	145
TRINITY_Contig9027	1	p3	(CGT)4	12	84	95	TGGCATGGCTACCGATTTGT	60.035	AGGAGCCCTATGTAAGGCCA	60.03	114
TRINITY_Contig9028	1	p5	(GAGCC)3	15	2261	2275	GGTGCACCCTTTCAGCAAAG	59.967	TGTAACTCAAGGCTGCCCTG	59.963	193
TRINITY_Contig9030	1	p4	(CGGC)3	12	163	174	TGCGGAAGGCTGACAAGAAT	59.963	TCGGCAAAAGTCTGGGAGTC	59.965	271
TRINITY_Contig9032	1	c	(GCG)4agggggccttcacggagcccccat(TCC)4	48	2542	2589	AAAGTTGCTTCTGCTGCTGC	59.968	TCGCGTTTGTTTGCAAGAGG	59.971	105
TRINITY_Contig9032	2	p3	(CTG)4	12	2966	2977	AAAGTTGCTTCTGCTGCTGC	59.968	TCGCGTTTGTTTGCAAGAGG	59.971	105
TRINITY_Contig9032	3	p4	(CGGC)3	12	3319	3330	AAAGTTGCTTCTGCTGCTGC	59.968	TCGCGTTTGTTTGCAAGAGG	59.971	105
TRINITY_Contig9033	1	p3	(CCG)4	12	116	127	GTTGGTGCCAGCTGAACTTG	59.969	AAGGCAGTTCTGGATCAGGC	60.035	152
TRINITY_Contig9033	2	p3	(CCG)4	12	274	285	GTTGGTGCCAGCTGAACTTG	59.969	AAGGCAGTTCTGGATCAGGC	60.035	152
TRINITY_Contig9035	1	p3	(GCC)4	12	181	192	GGAGACCATGCATCACACCA	60.035	GGCTGAGGCAAATCTCCACT	60.035	131
TRINITY_Contig9038	1	p3	(TCC)4	12	324	335	TCCTCAAGCACTCGGTGTTC	59.966	AACGGCAGGGTAGGCTACTA	60.033	205
TRINITY_Contig9039	1	p3	(GCA)5	15	564	578	GGCGACTACTAGCAGAGCAG	59.97	TCCTTTACGCTGGTCTGCAG	60.037	246
TRINITY_Contig9039	2	p3	(CAG)4	12	1881	1892	GGCGACTACTAGCAGAGCAG	59.97	TCCTTTACGCTGGTCTGCAG	60.037	246
TRINITY_Contig9039	3	c	(CAG)5agcgaggcgatgtactacgccggcccatcggccaaggacgagccgcgagacgacatgtcgtttttcgagcagccattgctgttctgatgaccggacg(AACC)3	124	2070	2193	GGCGACTACTAGCAGAGCAG	59.97	TCCTTTACGCTGGTCTGCAG	60.037	246
TRINITY_Contig9040	1	p4	(CATG)3	12	2304	2315	ACGCTGATCCATGCACTTGA	60.036	AGCGTGGACTCTACAACAGC	60.038	176
TRINITY_Contig9041	1	p3	(GTC)5	15	273	287	TTCCAGACCACACGAGATGC	60.037	ATGTGGGCAACGTTCGTTTG	59.97	200
TRINITY_Contig9041	2	p3	(GCA)4	12	1092	1103	TTCCAGACCACACGAGATGC	60.037	ATGTGGGCAACGTTCGTTTG	59.97	200
TRINITY_Contig9041	3	p3	(CGC)4	12	1746	1757	TTCCAGACCACACGAGATGC	60.037	ATGTGGGCAACGTTCGTTTG	59.97	200
TRINITY_Contig9041	4	p4	(CGGC)3	12	1985	1996	TTCCAGACCACACGAGATGC	60.037	ATGTGGGCAACGTTCGTTTG	59.97	200
TRINITY_Contig9042	1	p3	(GCC)4	12	399	410	CGATCGAGCTACCAGTGACC	59.97	ACTCGTCTCCCTTCTCCCTC	60.034	103
TRINITY_Contig9043	1	p2	(GA)11	22	2390	2411	AAAGGCCAACACACAGACCA	60.034	AGAGAAACAAGGCCGCATCA	59.963	146
TRINITY_Contig9044	1	c	(CCG)5(CAG)4	27	439	465	CTCTGGGACGCCTTCTTCAG	60.108	AGTTGCCGCCGTAAAAGTA	59.965	122
TRINITY_Contig9046	1	p4	(CCCA)3	12	135	146	GCGAGCAAAAAGACACTGCA	59.971	GAGCGCGATTCCATTTCCAC	59.972	183
TRINITY_Contig9049	1	p4	(GCCG)3	12	1418	1429	CTGTTGTCGGTGTTGCTGTG	59.972	AGAGCACGCTACGATCGAAG	59.972	130
TRINITY_Contig9051	1	p3	(GCC)4	12	162	173	TTTCCGAGCCGCAAACAAAG	59.97	ATCGTCATGGTATCGCCGAC	60.039	212
TRINITY_Contig9051	2	p3	(GCC)4	12	431	442	TTTCCGAGCCGCAAACAAAG	59.97	ATCGTCATGGTATCGCCGAC	60.039	212
TRINITY_Contig9051	3	p3	(CGC)4	12	766	777	TTTCCGAGCCGCAAACAAAG	59.97	ATCGTCATGGTATCGCCGAC	60.039	212
TRINITY_Contig9053	1	p3	(GCG)6	18	255	272	TCGAGGAGCAGTGGGTGATA	60.034	CATGGCAATGACTGGATGCG	59.97	214
TRINITY_Contig9053	2	p3	(TCT)4	12	2203	2214	TCGAGGAGCAGTGGGTGATA	60.034	CATGGCAATGACTGGATGCG	59.97	214
TRINITY_Contig9054	1	p6	(GGGGCA)3	18	1810	1827	CACCCTGACTGCCTGAATGT	59.963	CTGGGGGATTGGGAAACTCC	60.033	122
TRINITY_Contig9055	1	p5	(CCAGA)3	15	1777	1791	TGGAAGCAGCCGTTTAGGAG	60.036	ATCAGGTCATGCCCCTCTCT	60.03	234
TRINITY_Contig9056	1	p3	(CCA)4	12	36	47	CCCTAAGCCCCCAAAGAAGG	60.033	TTCGCCTTCACTTGCTTTGC	59.969	186
TRINITY_Contig9056	2	p3	(TGA)7	21	1715	1735	CCCTAAGCCCCCAAAGAAGG	60.033	TTCGCCTTCACTTGCTTTGC	59.969	186
TRINITY_Contig9059	1	p3	(GGA)5	15	2363	2377	GAGCACACGAAGACACTGGA	59.968	TGTTTCTCTGTCCGGCTTCC	59.965	238
TRINITY_Contig9060	1	p3	(CGC)5	15	209	223	ACCAACGGACAGCTACCAAG	59.965	ATCACGCGGAGAAGTTGGTT	59.965	123
TRINITY_Contig9064	1	p3	(CGT)4	12	1147	1158	CACTGTTCCCGGCCATTACT	60.035	GAGCTCAGACTTCAGAGGCC	59.824	102
TRINITY_Contig9067	1	p6	(GGGCTC)4	24	2133	2156	CTCCTGCGCATTTCAGCATC	59.971	CCGCTGCTGTTACTGTGAGA	60.038	279
TRINITY_Contig9068	1	p3	(CGC)5	15	206	220	CTGCGAACGCTTGTTATCGG	59.975	ACTCCATCCTGCAGCTTGTC	60.036	191
TRINITY_Contig9069	1	p4	(CAGA)3	12	108	119	CAGATCTGCGCGTGGATTTG	59.973	TCCTTGGATGACCCACTTGC	59.962	189
TRINITY_Contig9069	2	p6	(TCTGGC)3	18	1654	1671	CAGATCTGCGCGTGGATTTG	59.973	TCCTTGGATGACCCACTTGC	59.962	189
TRINITY_Contig9069	3	p3	(GCT)4	12	2222	2233	CAGATCTGCGCGTGGATTTG	59.973	TCCTTGGATGACCCACTTGC	59.962	189
TRINITY_Contig9070	1	p3	(GGA)5	15	570	584	ATTTGACCAGGGCATCACGT	59.962	CAAGCGTTTGAGTTCACCGG	60.041	111
TRINITY_Contig9070	2	p3	(GGC)6	18	1028	1045	ATTTGACCAGGGCATCACGT	59.962	CAAGCGTTTGAGTTCACCGG	60.041	111
TRINITY_Contig9070	3	c	(GCG)5gaaggcgggctgacgag(GCT)4	44	1455	1498	ATTTGACCAGGGCATCACGT	59.962	CAAGCGTTTGAGTTCACCGG	60.041	111
TRINITY_Contig9070	4	p6	(TTTTCC)3	18	1730	1747	ATTTGACCAGGGCATCACGT	59.962	CAAGCGTTTGAGTTCACCGG	60.041	111
TRINITY_Contig9072	1	p4	(AGGA)5	20	1072	1091	TCCACTCGGAGTCGTCTCTT	59.964	CGATCGACGATGGAGAAGCA	59.971	263
TRINITY_Contig9072	2	c	(CTCC)4ttccttcccttggaagacgcgcctccttcc(CCTT)4	62	1263	1324	TCCACTCGGAGTCGTCTCTT	59.964	CGATCGACGATGGAGAAGCA	59.971	263
TRINITY_Contig9072	3	p3	(ACC)13	39	1818	1856	TCCACTCGGAGTCGTCTCTT	59.964	CGATCGACGATGGAGAAGCA	59.971	263
TRINITY_Contig9075	1	p5	(CTGGT)3	15	2414	2428	CTAAAGAGCAATGCGTCGGC	59.973	TTGAATGAGGCTCCCAGCTG	60.035	239
TRINITY_Contig9077	1	c	(CTCTT)3ctccttcccctcctcctccctttgcgtccccttttactccccctccccctctctccagtc(CT)6	86	20	105	GGGCAGGCAGAGAAGGAATT	60.034	TGGGGTCTATGGCCTCATGA	60.03	256
TRINITY_Contig9077	2	p3	(GGT)4	12	2045	2056	GGGCAGGCAGAGAAGGAATT	60.034	TGGGGTCTATGGCCTCATGA	60.03	256
TRINITY_Contig9079	1	c	(CAG)4gcagaccggggcaggcgcgcacgcgctacagaagggcctgccacccctgtggctttgaccacgacgcgca(GCC)6	100	244	343	CCCTCTTGCGGTTTGGTTTG	59.968	CCAAAAAGCGCAAGGAAGCT	59.967	131
TRINITY_Contig9080	1	p3	(GAG)5	15	944	958	TGCTCACAACATGCACTTGC	59.969	CCTCCTACGATTCGGGCTTC	59.968	108
TRINITY_Contig9083	1	p3	(CGG)5	15	2006	2020	CAGCCAGTGACAGTTGCAAC	59.97	GCGCAAACAAGGCACCTAAA	59.969	145
TRINITY_Contig9084	1	p2	(AT)6	12	1588	1599	AATGTTGGTGTTTGCCGGTG	59.896	AACTCCCTGAGACATGCACG	60.037	199
TRINITY_Contig9086	1	c	(CGCCT)3(CCCCT)3	30	41	70	TGACACATGCTCTTGCTCGT	59.966	GTAACCCTCCTTCCAAGGCC	60.034	150
TRINITY_Contig9087	1	p4	(GGGA)3	12	2291	2302	AAAGAAGGCGTACGAGGCAA	59.966	ATCGGGTTCCTCTGCTCTCT	60.033	130
TRINITY_Contig9089	1	p3	(CGC)5	15	363	377	GCTGTGAAGGGAAAGAGCCT	59.963	ACGAAGTCACCACAGACGTC	59.97	165

TRINITY_Contig9089	2	p3	(GCG)4	12	696	707	GCTGTGAAGGGAAAGAGCCT	59.963	ACGAAGTCACCACAGACGTC	59.97	165
TRINITY_Contig9089	3	p6	(TCGTCC)3	18	1243	1260	GCTGTGAAGGGAAAGAGCCT	59.963	ACGAAGTCACCACAGACGTC	59.97	165
TRINITY_Contig9090	1	p3	(AGG)4	12	1562	1573	GTGGCCAGGACGACAATGTA	60.037	GAGGCCCGACAAGAAGAACA	59.965	162
TRINITY_Contig9090	2	p3	(TGA)4	12	2245	2256	GTGGCCAGGACGACAATGTA	60.037	GAGGCCCGACAAGAAGAACA	59.965	162
TRINITY_Contig9091	1	c	(CCTC)5cacccaccccaccctcacccactcgtccccctccccttccccctccgcatcccatgtcccgcctccatctcctcccgctcctcctcttcgct(CTC)5cgccccatcccgctcgctcccagccggccgccccctccccgacgccgcaatgcgcgctcaatttcaccgcgctgcgcccccttcctcg(CGC)4	227	90	316	GAGTGGAAAGAGGGCGTTCA	59.965	AGAGTGCTCCAACAAGGACG	59.966	170
TRINITY_Contig9092	1	c	(GAAA)5aaaaagttttccc(TTCT)6	57	8	64	GTTGGAGTTCTGGTCGCAGA	59.966	TTTGGTTTTGTAGCGCGTGG	59.97	136
TRINITY_Contig9095	1	p4	(GGCG)3	12	2022	2033	TGTGATGCCCCGATGTTGAA	59.963	CTGACCTACGCTACGCTCTG	59.972	143
TRINITY_Contig9095	2	p3	(CCG)4	12	2188	2199	TGTGATGCCCCGATGTTGAA	59.963	CTGACCTACGCTACGCTCTG	59.972	143
TRINITY_Contig9096	1	p5	(CAGAG)5	25	48	72	GGATGAGGTACGAGGCCATG	59.966	GCGCTGCAATCATCTCAGTG	59.972	153
TRINITY_Contig9096	2	p3	(CTC)5	15	434	448	GGATGAGGTACGAGGCCATG	59.966	GCGCTGCAATCATCTCAGTG	59.972	153
TRINITY_Contig9097	1	p4	(CCGC)3	12	135	146	GCTTCTAGATCCCACGCTCC	59.967	AGTGAGGTTAGCGAGCGATG	59.899	209
TRINITY_Contig9097	2	c	(CCG)7cgcgtattaactctcgcccactgactccccggctc(GGCT)4	73	324	396	GCTTCTAGATCCCACGCTCC	59.967	AGTGAGGTTAGCGAGCGATG	59.899	209
TRINITY_Contig9097	3	p3	(ATT)6	18	1536	1553	GCTTCTAGATCCCACGCTCC	59.967	AGTGAGGTTAGCGAGCGATG	59.899	209
TRINITY_Contig9097	4	p3	(AGC)4	12	2208	2219	GCTTCTAGATCCCACGCTCC	59.967	AGTGAGGTTAGCGAGCGATG	59.899	209
TRINITY_Contig9098	1	p3	(GCA)6	18	2105	2122	TCACACAGGATGGCTGTGTC	59.965	TGGCAAGGCTCCAGCAATTA	59.961	120
TRINITY_Contig9098	2	p2	(AG)6	12	2262	2273	TCACACAGGATGGCTGTGTC	59.965	TGGCAAGGCTCCAGCAATTA	59.961	120
TRINITY_Contig9099	1	c	(AG)6tagttggcgcgctgattcagcgccgtcccgcactccacgcgccaacgcccccaaatttccgattttttccccaccccctcccgccgcgcgccgcgccat(GCC)5	125	277	401	CCTCCAAGGTCCACAAGTCC	59.963	GCCTTGTCTCGTATGGCTT	60.108	137
TRINITY_Contig9099	2	p3	(CCG)4	12	866	877	CCTCCAAGGTCCACAAGTCC	59.963	GCCTTGTCTCGTATGGCTT	60.108	137
TRINITY_Contig9099	3	p4	(GCGT)3	12	2185	2196	CCTCCAAGGTCCACAAGTCC	59.963	GCCTTGTCTCGTATGGCTT	60.108	137
TRINITY_Contig9100	1	p3	(GCT)7	21	204	224	TCGTTGCAGCAACCCAAATG	59.968	TGGTGTCGGAGCTAAGCTTG	60.037	254
TRINITY_Contig9100	2	p3	(CCG)5	15	488	502	TCGTTGCAGCAACCCAAATG	59.968	TGGTGTCGGAGCTAAGCTTG	60.037	254
TRINITY_Contig9100	3	p3	(CGA)4	12	2160	2171	TCGTTGCAGCAACCCAAATG	59.968	TGGTGTCGGAGCTAAGCTTG	60.037	254
TRINITY_Contig9103	1	p4	(GCCC)3	12	238	249	ATCTTCCCCTGTCCCTCTCC	60.031	GATCAGAGGAAGTCGCCGAG	59.97	148
TRINITY_Contig9104	1	p3	(GCC)4	12	171	182	GAATGCCAATGCCGGACATC	59.969	ACACCTCCGAGACAGCATTG	60.037	105
TRINITY_Contig9105	1	p3	(TGA)4	12	2149	2160	CTGTCGCGGCTATTTGTTCG	59.975	TCTACCTCTCCGGCCTTTGA	59.959	108
TRINITY_Contig9106	1	p3	(CCG)4	12	2077	2088	TCCTCACGCTGGCTTTTCAT	59.963	ACCGACTGCTGGGATTCAAG	60.036	228
TRINITY_Contig9107	1	c	(CGT)6cctcagaagcacatgtcgaggac(GCA)4gatggcggcgcagcatcctttccagaagccgtcgccgccgcggctggtggtctccg(GCT)4	121	3369	3489	TGCCAATCGGAGCAATCCTT	60.034	AACGCCTTGATTCCCTCCTG	60.035	214
TRINITY_Contig9108	1	p5	(TCCAA)3	15	469	483	GAGACGCAACGAATCATGGC	59.973	ATAGCTGCGATTCGTCAGGG	59.967	219
TRINITY_Contig9109	1	p3	(CGG)5	15	1887	1901	CTTGGCGTCGCTGTTTTTCA	59.971	CATATAAACGCGCGGCTAGC	59.907	219
TRINITY_Contig9109	2	p3	(GGC)5	15	2077	2091	CTTGGCGTCGCTGTTTTTCA	59.971	CATATAAACGCGCGGCTAGC	59.907	219
TRINITY_Contig9109	3	p3	(CAG)5	15	2368	2382	CTTGGCGTCGCTGTTTTTCA	59.971	CATATAAACGCGCGGCTAGC	59.907	219
TRINITY_Contig9110	1	p3	(AGG)5	15	1539	1553	TCTCTCAACAAGTCGACGGC	60.039	CGCCGCAGAAATGTAAGCTC	59.973	155
TRINITY_Contig9110	2	p3	(TGA)4	12	2225	2236	TCTCTCAACAAGTCGACGGC	60.039	CGCCGCAGAAATGTAAGCTC	59.973	155
TRINITY_Contig9113	1	p6	(CCTCTG)3	18	159	176	CCACCTACGACATCGAGCTC	59.97	GTCTTGGAGCTCTTGGGGTC	60.036	148
TRINITY_Contig9113	2	p3	(GGC)4	12	1668	1679	CCACCTACGACATCGAGCTC	59.97	GTCTTGGAGCTCTTGGGGTC	60.036	148
TRINITY_Contig9114	1	p3	(CCG)4	12	304	315	ATGGGAGACAATGGTGGCAG	60.034	GAGCTCGCGATGAATCTCCA	59.969	203
TRINITY_Contig9115	1	p6	(CCGCGG)3	18	2080	2097	GCCATTGCCTTCTTCCTCCT	60.034	TCTGGAACCAGACAGCGAAC	59.966	178
TRINITY_Contig9117	1	p2	(AG)6	12	1877	1888	GCATAGCAATGTGGAGCAGC	59.97	ATGCCCATCAAGGTCACCAG	60.034	264
TRINITY_Contig9117	2	p3	(GCG)4	12	2128	2139	GCATAGCAATGTGGAGCAGC	59.97	ATGCCCATCAAGGTCACCAG	60.034	264
TRINITY_Contig9117	3	p3	(AGA)4	12	2334	2345	GCATAGCAATGTGGAGCAGC	59.97	ATGCCCATCAAGGTCACCAG	60.034	264
TRINITY_Contig9118	1	p4	(AAGC)3	12	671	682	GACGTTGTGGGACCATCTGT	59.965	TGCTCTGCAGAAGTGACCAG	59.966	145
TRINITY_Contig9118	2	p3	(TTG)4	12	1302	1313	GACGTTGTGGGACCATCTGT	59.965	TGCTCTGCAGAAGTGACCAG	59.966	145
TRINITY_Contig9123	1	p3	(GCT)4	12	278	289	ACCTCCGGTTCAACGACTTC	59.967	GAGGAGGATCTCGTTGAGCG	59.97	218
TRINITY_Contig9123	2	p3	(GCC)5	15	1424	1438	ACCTCCGGTTCAACGACTTC	59.967	GAGGAGGATCTCGTTGAGCG	59.97	218
TRINITY_Contig9124	1	p3	(AGG)5	15	2288	2302	TCACAGCAAAAATGGCCAGC	59.966	GCTGCTTCCTTGACTAGGCA	60.036	258
TRINITY_Contig9125	1	p4	(AGAA)7	28	1022	1049	AGTTATTGGGAGCCACCGTG	60.035	AAATCAATCCAGCCAGGGGG	60.032	242
TRINITY_Contig9126	1	p3	(GGC)4	12	417	428	GCAAGGGTGGTGAGCTTACT	59.963	CGAGTATACAGGGACACGGC	59.97	191
TRINITY_Contig9126	2	p3	(GCT)5	15	1783	1797	GCAAGGGTGGTGAGCTTACT	59.963	CGAGTATACAGGGACACGGC	59.97	191
TRINITY_Contig9129	1	c	(CCG)4cacatggcggcggccccgcctcccccgggcacgatgggagccggggcgggccagccccgccc(CCG)4	87	128	214	AGCCGCTGTACCGTGATATG	59.968	AGAATGCTGGCAAATCCGGA	60.034	213
TRINITY_Contig9131	1	p3	(CCT)5	15	151	165	GCAGAATGAATCCTGCGCAG	59.971	TGAACCTTGGTAACGGTCGG	59.966	276
TRINITY_Contig9131	2	p3	(GCT)4	12	2174	2185	GCAGAATGAATCCTGCGCAG	59.971	TGAACCTTGGTAACGGTCGG	59.966	276
TRINITY_Contig9132	1	p3	(CGG)4	12	1567	1578	AAACAGCTCAGAGTCGCCTC	60.038	AATCTTAACCTGGGGCGTGG	60.034	177
TRINITY_Contig9134	1	p3	(GCG)4	12	560	571	ACGTACCATCAAGCCGGTTT	59.964	CTAATGCGAACGTGTGCGAG	59.976	236
TRINITY_Contig9134	2	p3	(CCG)4	12	1194	1205	ACGTACCATCAAGCCGGTTT	59.964	CTAATGCGAACGTGTGCGAG	59.976	236
TRINITY_Contig9134	3	p5	(CGGGC)3	15	1402	1416	ACGTACCATCAAGCCGGTTT	59.964	CTAATGCGAACGTGTGCGAG	59.976	236
TRINITY_Contig9135	1	p3	(GCG)4	12	1299	1310	CACCCTGACTGCCTGAATGT	59.963	CTGGGGGATTGGGAAACTCC	60.033	122
TRINITY_Contig9137	1	p4	(GTTT)3	12	2322	2333	CGCCGTGAGCTTTAGCAATC	59.973	CGCCCTCGAGGTTTTCTCTT	60.037	101
TRINITY_Contig9138	1	p4	(GTTC)3	12	150	161	CTTGAGAGCCTTGGTGCTGA	59.964	GGGGTTGTTCTTTGTTCGCC	59.968	249
TRINITY_Contig9139	1	p3	(CGG)5	15	1894	1908	CCGGAGGCACCTTCGTATAC	59.968	GGACTGCTAGGCTGTTGTGT	59.965	180
TRINITY_Contig9140	1	p5	(TCCCC)3	15	52	66	ATGAGTCCTGGTAGAGCGGT	60.033	GGCCGCACTGTTTTTGGAAT	59.967	116
TRINITY_Contig9140	2	p3	(CGG)4	12	684	695	ATGAGTCCTGGTAGAGCGGT	60.033	GGCCGCACTGTTTTTGGAAT	59.967	116
TRINITY_Contig9140	3	p3	(CGC)4	12	1978	1989	ATGAGTCCTGGTAGAGCGGT	60.033	GGCCGCACTGTTTTTGGAAT	59.967	116
TRINITY_Contig9141	1	p3	(TCG)4	12	643	654	AGACCTTCTTGGAATGGCCG	60.035	ACCATTCCAAGAAGCCCGAG	60.035	114
TRINITY_Contig9141	2	p3	(CCG)4	12	2057	2068	AGACCTTCTTGGAATGGCCG	60.035	ACCATTCCAAGAAGCCCGAG	60.035	114
TRINITY_Contig9143	1	p3	(CGA)4	12	667	678	CTGAGATCCGAGCCCATCAC	59.967	ATGGTGAACATGTGGGCCAT	59.958	134
TRINITY_Contig9143	2	p5	(CGGCC)3	15	915	929	CTGAGATCCGAGCCCATCAC	59.967	ATGGTGAACATGTGGGCCAT	59.958	134
TRINITY_Contig9143	3	p3	(GGC)4	12	1126	1137	CTGAGATCCGAGCCCATCAC	59.967	ATGGTGAACATGTGGGCCAT	59.958	134
TRINITY_Contig9143	4	p3	(CGC)4	12	1762	1773	CTGAGATCCGAGCCCATCAC	59.967	ATGGTGAACATGTGGGCCAT	59.958	134
TRINITY_Contig9147	1	p3	(GGC)4	12	344	355	GTACAAGTACATGCAGCGCG	59.975	TGTACCAGTGCACCTCGAAC	59.967	164
TRINITY_Contig9147	2	p4	(GTGC)3	12	777	788	GTACAAGTACATGCAGCGCG	59.975	TGTACCAGTGCACCTCGAAC	59.967	164
TRINITY_Contig9149	1	p4	(TTGG)3	12	729	740	GACCTCAGCTGTGCCTTTCT	59.964	TGCGTGCTCTCATAAGTGCA	60.037	191
TRINITY_Contig9151	1	p4	(TTGC)4	16	226	241	TTCGGCTCCTCTGATCCAGA	60.033	AGGATCAGAGTGCCTCGACT	60.034	105
TRINITY_Contig9152	1	p3	(CTC)4	12	881	892	TACATCACAGCACAACGGCT	59.965	ACTGCGCGGATTAGATCAGG	59.967	196
TRINITY_Contig9152	2	p3	(CGG)4	12	1387	1398	TACATCACAGCACAACGGCT	59.965	ACTGCGCGGATTAGATCAGG	59.967	196
TRINITY_Contig9152	3	c	(CGG)7cgcctcctggtcgcgccgcatcccccgcccgagctgcagcagctcgtccatggcggcggagacggggctgcccg(GGA)5	110	1541	1650	TACATCACAGCACAACGGCT	59.965	ACTGCGCGGATTAGATCAGG	59.967	196
TRINITY_Contig9152	4	p4	(GGAG)3	12	1908	1919	TACATCACAGCACAACGGCT	59.965	ACTGCGCGGATTAGATCAGG	59.967	196
TRINITY_Contig9152	5	p4	(CGAA)4	16	2143	2158	TACATCACAGCACAACGGCT	59.965	ACTGCGCGGATTAGATCAGG	59.967	196
TRINITY_Contig9154	1	p3	(CCT)4	12	741	752	AAGCAAAGGGAGGCGAGTAC	60.036	CCAACTCCAAGACCCCTTCC	59.962	136
TRINITY_Contig9154	2	p3	(CTG)4	12	1159	1170	AAGCAAAGGGAGGCGAGTAC	60.036	CCAACTCCAAGACCCCTTCC	59.962	136
TRINITY_Contig9154	3	c	(AG)8ggagtcagggaga(GAG)6	47	2228	2274	AAGCAAAGGGAGGCGAGTAC	60.036	CCAACTCCAAGACCCCTTCC	59.962	136
TRINITY_Contig9159	1	p6	(GCGCTG)3	18	2243	2260	CATGCTACGTCCTGGGCTAG	59.967	CATCGATCGGTCACGCACTA	59.971	106
TRINITY_Contig9162	1	p3	(GAG)4	12	1278	1289	TGACCTACCACAACGACACG	59.969	GAATGACCAGGACAGCAGCT	60.036	176
TRINITY_Contig9162	2	p3	(CGG)5	15	2182	2196	TGACCTACCACAACGACACG	59.969	GAATGACCAGGACAGCAGCT	60.036	176
TRINITY_Contig9164	1	p4	(AAAG)3	12	357	368	TGCCAAAAATGACCGCAGTG	59.968	GTTCAGTCTCGGCTGTTCA	59.966	260
TRINITY_Contig9164	2	c*	(GCGCCC)2(GCGC<CC>G)(CCG)4	31	1028	1058	TGCCAAAAATGACCGCAGTG	59.968	GTTCAGTCTCGGCTGTTCA	59.966	260
TRINITY_Contig9164	3	p3	(CGC)5	15	2307	2321	TGCCAAAAATGACCGCAGTG	59.968	GTTCAGTCTCGGCTGTTCA	59.966	260
TRINITY_Contig9165	1	p4	(AAGC)3	12	2124	2135	GGGATTCCGCTGAGATAGGC	60.037	AAAGATTGCAACGGTGTGGC	59.968	211
TRINITY_Contig9166	1	p3	(CAG)4	12	1000	1011	CTTGAGGGAGGAGTGCAAGG	60.036	TGGCATAGTAGCATGCTCCG	59.966	199
TRINITY_Contig9167	1	p3	(CGG)4	12	132	143	GCTGCCCCTTTGGATACCTT	60.033	CCTGTGCACGAGGTTACAGT	59.966	114
TRINITY_Contig9168	1	c	(TCCGA)3tccatcaggcgacgaccgccccgatccggcattgatgtcggccgcctttgtga(CGG)6	87	210	296	TCGATGCTCAGCACCACATT	60.036	TACACCCTCATCCCCACGAT	60.032	251
TRINITY_Contig9168	2	p3	(CGC)4	12	624	635	TCGATGCTCAGCACCACATT	60.036	TACACCCTCATCCCCACGAT	60.032	251
TRINITY_Contig9168	3	p3	(CGG)5	15	972	986	TCGATGCTCAGCACCACATT	60.036	TACACCCTCATCCCCACGAT	60.032	251
TRINITY_Contig9168	4	p3	(CGC)5	15	2071	2085	TCGATGCTCAGCACCACATT	60.036	TACACCCTCATCCCCACGAT	60.032	251

TRINITY_Contig9168	5	c	(GGGAGA)3agcttgtcacgagctagcctagctacacgacacaaccagcggcgcgccagcagagagaggcatgccggaggcgccgggcgccatgcgagaga(CGG)5	125	2255	2379	TCGATGCTCAGCACCACATT	60.036	TACACCCTCATCCCCACGAT	60.032	251
TRINITY_Contig9170	1	p3	(CCG)4	12	47	58	GAGTAGTGAGCCGAATCCCG	59.969	CACGTGGCGGTTTCCAAATT	59.969	244
TRINITY_Contig9170	2	p5	(CCACG)3	15	675	689	GAGTAGTGAGCCGAATCCCG	59.969	CACGTGGCGGTTTCCAAATT	59.969	244
TRINITY_Contig9170	3	c	(CGG)5cgctcacccgcccccgccgatgtcgtcccaccgccgccgcgccaagaaccccgacctcaaccgggagccgaccccggacac(CGA)4gtgaagcgcgctgattttgtctcctct(TCC)5atcatctactagtctctagtagtccgttaggcaacaactactcacccgaggag(TAGC)5	223	1212	1434	GAGTAGTGAGCCGAATCCCG	59.969	CACGTGGCGGTTTCCAAATT	59.969	244
TRINITY_Contig9170	4	p3	(GGC)4	12	1668	1679	GAGTAGTGAGCCGAATCCCG	59.969	CACGTGGCGGTTTCCAAATT	59.969	244
TRINITY_Contig9171	1	p2	(TC)8	16	396	411	AATTTTGCGAGCCCCGAAAC	60.039	GTGGAGCAGAGGGAGAGAGA	60.034	235
TRINITY_Contig9171	2	p3	(CAC)6	18	769	786	AATTTTGCGAGCCCCGAAAC	60.039	GTGGAGCAGAGGGAGAGAGA	60.034	235
TRINITY_Contig9171	3	p3	(CCG)4	12	1223	1234	AATTTTGCGAGCCCCGAAAC	60.039	GTGGAGCAGAGGGAGAGAGA	60.034	235
TRINITY_Contig9172	1	p3	(GCG)4	12	2077	2088	TTTGCATGCGACTCAACAGC	60.04	CTGCGAACATGGATTCAGCG	59.973	212
TRINITY_Contig9175	1	p3	(AGC)5	15	108	122	ACCTCCTTCCGGCTATCCTT	60.031	AGGAGGAGCGAGAAGGTGAT	60.033	179
TRINITY_Contig9176	1	p4	(CGAG)5	20	1774	1793	GTCCATATCGCACTCGCAGA	59.969	GGTCGTCTCGGTTGTTTCCT	59.967	272
TRINITY_Contig9177	1	c	(CCTCT)3cttgtggattccaccaacccgccccccttttcttggctgcgtgcgtatcgccgtcctaggcattcgcccccctggcg(GCCT)3	104	155	258	CAGGCACAAAGCTTGGTGTC	59.969	CCATGGAGGCCAGCAAGTAA	60.034	187
TRINITY_Contig9177	2	p4	(AGGC)3	12	1506	1517	CAGGCACAAAGCTTGGTGTC	59.969	CCATGGAGGCCAGCAAGTAA	60.034	187
TRINITY_Contig9178	1	p4	(TAGC)3	12	297	308	AGTCCATGTTCTGTGCTCGG	60.037	AGTCCATGTTCTGTGCTCGG	60.037	267
TRINITY_Contig9178	2	p3	(CGC)4	12	740	751	AGTCCATGTTCTGTGCTCGG	60.037	AGTCCATGTTCTGTGCTCGG	60.037	267
TRINITY_Contig9178	3	p3	(CGG)4	12	1535	1546	AGTCCATGTTCTGTGCTCGG	60.037	AGTCCATGTTCTGTGCTCGG	60.037	267
TRINITY_Contig9178	4	p4	(AGCT)3	12	1979	1990	AGTCCATGTTCTGTGCTCGG	60.037	AGTCCATGTTCTGTGCTCGG	60.037	267
TRINITY_Contig9179	1	p3	(GCC)4	12	154	165	GCTCTGTTGCTGTGTGGTTG	59.97	ATCCAGGTCCGGATTGGGTA	60.031	279
TRINITY_Contig9180	1	p3	(CGC)4	12	1179	1190	GTACGATCAGAGCGACCCAG	59.97	AGCAGGACATCAGGCTCAAC	60.036	210
TRINITY_Contig9181	1	c	(AGG)5aaaagctcaagatctgggaggagggggccaggaggagcggaa(GGC)5	73	542	614	GCCTTCATCTACCCAACGCT	60.108	GTGATCAGTATGGGGGAGCG	59.966	152
TRINITY_Contig9182	1	p3	(CCT)7	21	259	279	GGTGAACGACTACCAACCGT	59.967	AGAACACAGACAGCCGATCG	60.109	273
TRINITY_Contig9182	2	p4	(TGCT)4	16	540	555	GGTGAACGACTACCAACCGT	59.967	AGAACACAGACAGCCGATCG	60.109	273
TRINITY_Contig9182	3	p3	(GCC)5	15	959	973	GGTGAACGACTACCAACCGT	59.967	AGAACACAGACAGCCGATCG	60.109	273
TRINITY_Contig9182	4	p2	(AG)6	12	1267	1278	GGTGAACGACTACCAACCGT	59.967	AGAACACAGACAGCCGATCG	60.109	273
TRINITY_Contig9185	1	p3	(CGC)5	15	969	983	GACAGGTCCGTCTTGGTGAG	60.038	CGAGGACGGTGGTGAAGAAA	59.967	151
TRINITY_Contig9185	2	p5	(CGCCG)3	15	1495	1509	GACAGGTCCGTCTTGGTGAG	60.038	CGAGGACGGTGGTGAAGAAA	59.967	151
TRINITY_Contig9186	1	p4	(CACG)3	12	62	73	TCGGTTTTCCTTCCTCCGTG	59.966	ATAGAGGCACACGAAGCACC	60.108	121
TRINITY_Contig9186	2	p3	(GCG)4	12	418	429	TCGGTTTTCCTTCCTCCGTG	59.966	ATAGAGGCACACGAAGCACC	60.108	121
TRINITY_Contig9186	3	c*	(AG)6(<AG>GG)(AGGG)3	28	2232	2259	TCGGTTTTCCTTCCTCCGTG	59.966	ATAGAGGCACACGAAGCACC	60.108	121
TRINITY_Contig9187	1	p4	(CTCG)3	12	163	174	AATCTCTGGGCGGCAATCAA	60.034	CTTGCAGTTATCGCAGCGTC	59.974	104
TRINITY_Contig9188	1	p3	(GCG)4	12	2146	2157	AGGATTACGAGCAGCGCTAC	59.969	TAGGGAAAGAACAGCAGGCG	60.036	115
TRINITY_Contig9189	1	p5	(CGAAG)3	15	26	40	TTGGATGCGTTATGGTGCCT	60.035	GCCAAACTGGACGCAACAAT	59.968	222
TRINITY_Contig9189	2	p4	(AATT)3	12	876	887	TTGGATGCGTTATGGTGCCT	60.035	GCCAAACTGGACGCAACAAT	59.968	222
TRINITY_Contig9190	1	p3	(CGC)4	12	196	207	CGACGCTATCACCAATCGA	59.97	GAATGACGGGTAGAGCTCCG	59.969	139
TRINITY_Contig9191	1	c	(CGA)4ggacagcgagcgccgtgctggcgcgtcgcccgccgtggacgggct(CGA)6cggcc(AGG)9	107	694	800	GCTTTGAGAAACCCAGCTGC	60.039	CCAATGCTCTTCATCGCAGC	59.971	258
TRINITY_Contig9192	1	p3	(GGC)4	12	3351	3362	GATGACCGATGTCTCCGGAC	59.969	CATGGGTCCTACTGGGGGTA	60.031	265
TRINITY_Contig9193	1	p5	(GACCT)4	20	89	108	TTGTTTTTCTCCGCGTGCTG	59.971	CGACCGATGCTCATGGATCA	59.967	182
TRINITY_Contig9193	2	p4	(CCCT)3	12	335	346	TTGTTTTTCTCCGCGTGCTG	59.971	CGACCGATGCTCATGGATCA	59.967	182
TRINITY_Contig9193	3	p5	(CTCCC)4	20	460	479	TTGTTTTTCTCCGCGTGCTG	59.971	CGACCGATGCTCATGGATCA	59.967	182
TRINITY_Contig9193	4	p6	(CGCTCT)3	18	644	661	TTGTTTTTCTCCGCGTGCTG	59.971	CGACCGATGCTCATGGATCA	59.967	182
TRINITY_Contig9193	5	p3	(AGG)4	12	871	882	TTGTTTTTCTCCGCGTGCTG	59.971	CGACCGATGCTCATGGATCA	59.967	182
TRINITY_Contig9194	1	p3	(CGC)4	12	454	465	TGAAACACAGTTGGCTCGGT	60.107	ACTAAACTTCAGGCCCCGTC	59.676	103
TRINITY_Contig9195	1	p3	(TCC)4	12	171	182	CTTCGTATCGCTGCCGTACT	59.971	GCACCAAAAGTCACGACCAC	59.971	185
TRINITY_Contig9195	2	c	(TGCCG)3cgctgcgctgctggcacggctcggtgtggtccccccacgtccggccagcccccagctgcgagcctgcgagcgagagacgcgcgcgccgcgaaccctcc(GCTG)3	126	788	913	CTTCGTATCGCTGCCGTACT	59.971	GCACCAAAAGTCACGACCAC	59.971	185
TRINITY_Contig9195	3	p3	(CGC)4	12	1630	1641	CTTCGTATCGCTGCCGTACT	59.971	GCACCAAAAGTCACGACCAC	59.971	185
TRINITY_Contig9197	1	p4	(CTTT)3	12	1184	1195	GCATGGTCCACGGGAGTTTA	60.036	CCGTAGTGGTTGCCTGGATT	60.035	201
TRINITY_Contig9197	2	p3	(CGC)6	18	2026	2043	GCATGGTCCACGGGAGTTTA	60.036	CCGTAGTGGTTGCCTGGATT	60.035	201
TRINITY_Contig9198	1	p2	(CT)8	16	53	68	AGCGTTCGTCTCTTCGTGTT	59.97	CACAACCGTGGAAAACCGTC	59.972	199
TRINITY_Contig9198	2	p3	(GCG)6	18	176	193	AGCGTTCGTCTCTTCGTGTT	59.97	CACAACCGTGGAAAACCGTC	59.972	199
TRINITY_Contig9198	3	p3	(GGC)4	12	415	426	AGCGTTCGTCTCTTCGTGTT	59.97	CACAACCGTGGAAAACCGTC	59.972	199
TRINITY_Contig9198	4	p6	(CACACG)3	18	672	689	AGCGTTCGTCTCTTCGTGTT	59.97	CACAACCGTGGAAAACCGTC	59.972	199
TRINITY_Contig9198	5	p3	(ACG)4	12	872	883	AGCGTTCGTCTCTTCGTGTT	59.97	CACAACCGTGGAAAACCGTC	59.972	199
TRINITY_Contig9198	6	p4	(CCTC)3	12	1964	1975	AGCGTTCGTCTCTTCGTGTT	59.97	CACAACCGTGGAAAACCGTC	59.972	199
TRINITY_Contig9198	7	p3	(CTG)4	12	2128	2139	AGCGTTCGTCTCTTCGTGTT	59.97	CACAACCGTGGAAAACCGTC	59.972	199
TRINITY_Contig9199	1	p4	(GTGA)4	16	175	190	ACCTGATGCCCATGTCCAAG	60.034	GGACTCCGACGTTTTGTCCT	59.967	227
TRINITY_Contig9199	2	p3	(GAC)4	12	1241	1252	ACCTGATGCCCATGTCCAAG	60.034	GGACTCCGACGTTTTGTCCT	59.967	227
TRINITY_Contig9199	3	p3	(AAG)4	12	1595	1606	ACCTGATGCCCATGTCCAAG	60.034	GGACTCCGACGTTTTGTCCT	59.967	227
TRINITY_Contig9203	1	p3	(GCG)4	12	720	731	CCTGGAATGGATGTTGGGCT	60.033	CCTGGAATGGATGTTGGGCT	60.033	167
TRINITY_Contig9205	1	p3	(CCG)4	12	101	112	TCACATGCTTCCCACTTCCC	59.962	CGAGATCAAATGGCGGCTTG	59.971	225
TRINITY_Contig9206	1	p3	(CGC)4	12	196	207	CGACGCTATCACCAATCGA	59.97	GAATGACGGGTAGAGCTCCG	59.969	139
TRINITY_Contig9207	1	p5	(TTATT)3	15	62	76	GATCATCCTGGAGCCTGTCG	59.967	CACGAATTCGGCGACATCAC	59.975	163
TRINITY_Contig9208	1	p3	(TCC)5	15	2010	2024	CCACCGTTATGTCAAGCCCT	60.035	GTGCCTCCAAATGCTACCCT	60.034	271
TRINITY_Contig9209	1	p4	(TTCC)3	12	1965	1976	TCGATAGGCGACATCGTGTG	59.971	CAGGAAGGAAGGAACACGCT	59.965	102
TRINITY_Contig9210	1	p3	(TGC)7	21	384	404	CAGTGGACCTTTGCAGCCTA	59.963	TTGTCTCGGGCATTTTGGGA	59.889	120
TRINITY_Contig9214	1	p3	(ATC)4	12	77	88	AGTAGCCGTGTTCTCTTGCC	60.037	GGTTTGGCCTCACAAGCAAG	59.967	138
TRINITY_Contig9215	1	p3	(CGG)4	12	586	597	GGAGTGGAAGCTGTCGTTGA	59.966	GAGGATGTTCAAGACGGCCA	60.036	199
TRINITY_Contig9215	2	c	(CGG)4cgtcttcgcgggcgccgccgtcgctgct(GTC)4	52	975	1026	GGAGTGGAAGCTGTCGTTGA	59.966	GAGGATGTTCAAGACGGCCA	60.036	199
TRINITY_Contig9215	3	p3	(CTG)4	12	1276	1287	GGAGTGGAAGCTGTCGTTGA	59.966	GAGGATGTTCAAGACGGCCA	60.036	199
TRINITY_Contig9215	4	c	(TGG)4tcgtagtcgtagggcgccgcgggtggca(GCG)4	52	1484	1535	GGAGTGGAAGCTGTCGTTGA	59.966	GAGGATGTTCAAGACGGCCA	60.036	199
TRINITY_Contig9215	5	c	(GAG)4gacgaggacgggaagctaatgccggcgtggcgcgggtcgaggaggccgccgtcgacgccgatcggatggctgggcacgccgatgggcggggaaact(CCG)4	120	1652	1771	GGAGTGGAAGCTGTCGTTGA	59.966	GAGGATGTTCAAGACGGCCA	60.036	199
TRINITY_Contig9215	6	c	(GCT)4atccatgg(CGAC)3	32	1996	2027	GGAGTGGAAGCTGTCGTTGA	59.966	GAGGATGTTCAAGACGGCCA	60.036	199
TRINITY_Contig9215	7	p3	(AGA)5	15	2199	2213	GGAGTGGAAGCTGTCGTTGA	59.966	GAGGATGTTCAAGACGGCCA	60.036	199
TRINITY_Contig9220	1	p3	(CGG)7	21	1981	2001	GCCGCGGGAATAATCAAACC	59.97	AAGCCCCCGAAAATCTCCTG	60.034	246
TRINITY_Contig9222	1	p3	(CAG)4	12	1267	1278	CTTTCTGGAGATGGCAGGCA	60.035	ATCCACCATTCCCAAGCCTG	60.033	145
TRINITY_Contig9223	1	p3	(AGC)4	12	206	217	CGATACAAGCATGCCACACG	59.973	TCCAAGAAACATGCTCCGCT	59.963	252
TRINITY_Contig9223	2	p3	(GCC)6	18	853	870	CGATACAAGCATGCCACACG	59.973	TCCAAGAAACATGCTCCGCT	59.963	252
TRINITY_Contig9223	3	p6	(CGTCGC)3	18	1149	1166	CGATACAAGCATGCCACACG	59.973	TCCAAGAAACATGCTCCGCT	59.963	252
TRINITY_Contig9223	4	p6	(CGCCCC)3	18	1432	1449	CGATACAAGCATGCCACACG	59.973	TCCAAGAAACATGCTCCGCT	59.963	252
TRINITY_Contig9225	1	p3	(TGC)5	15	1756	1770	AATGGACATTCCCGGTCACC	60.034	CAGCAGCAGCAAGTACCTCT	60.037	259
TRINITY_Contig9225	2	p3	(CAG)5	15	2837	2851	AATGGACATTCCCGGTCACC	60.034	CAGCAGCAGCAAGTACCTCT	60.037	259
TRINITY_Contig9225	3	p3	(CCG)4	12	2961	2972	AATGGACATTCCCGGTCACC	60.034	CAGCAGCAGCAAGTACCTCT	60.037	259
TRINITY_Contig9226	1	p3	(GGA)6	18	1051	1068	GACGATCGCATCAAAGCCAC	59.973	AGCTCTGCCATGGTCCATTC	60.107	177
TRINITY_Contig9227	1	p4	(CCCT)4	16	6	21	TCCGATGAAGATGCTCTCGC	59.969	CGAAGTTGGCACCAAAACGT	59.9	193
TRINITY_Contig9227	2	p3	(GCC)6	18	302	319	TCCGATGAAGATGCTCTCGC	59.969	CGAAGTTGGCACCAAAACGT	59.9	193
TRINITY_Contig9228	1	p4	(ATGC)4	16	330	345	CAGGACCATCTCCATCTCGC	59.967	CACTCCACTCCCACTTCGAC	60.038	115
TRINITY_Contig9228	2	p3	(GCG)4	12	669	680	CAGGACCATCTCCATCTCGC	59.967	CACTCCACTCCCACTTCGAC	60.038	115
TRINITY_Contig9228	3	p3	(CGA)4	12	1162	1173	CAGGACCATCTCCATCTCGC	59.967	CACTCCACTCCCACTTCGAC	60.038	115
TRINITY_Contig9228	4	p3	(CCG)4	12	1686	1697	CAGGACCATCTCCATCTCGC	59.967	CACTCCACTCCCACTTCGAC	60.038	115
TRINITY_Contig9229	1	p2	(AT)7	14	2147	2160	GCTCTTGCCTTGTACCGTCT	60.037	CCGTTTGTTTTCCACGGGAC	59.97	141
TRINITY_Contig9231	1	p2	(CG)6	12	1893	1904	TGCAGCAGTGAACATTGCAC	59.969	ACAGAGCACACTTCCTCAGC	59.965	241
TRINITY_Contig9231	2	p3	(CGC)5	15	2028	2042	TGCAGCAGTGAACATTGCAC	59.969	ACAGAGCACACTTCCTCAGC	59.965	241
TRINITY_Contig9232	1	p3	(AGC)4	12	282	293	CTACTGACGAGTGGGATCGC	59.97	CCAGTCTGTGTGGCTACTGG	60.037	143
TRINITY_Contig9233	1	p3	(ACC)4	12	4	15	GGTGTGGGTGGAGATGAAGG	60.035	CATCTCCCCAAACACCCTCC	60.034	118

TRINITY_Contig9235	1	p3	(GCC)5	15	474	488	GTTTTGGGAGCTGCCACAG	59.967	CCCTTGTCGTTGACGTACCA	59.968	278
TRINITY_Contig9236	1	p4	(CGTC)3	12	1	12	CGCGTCCTACATCTTCCTCC	59.969	TTGTCCAGAGTAGCTGCTGC	60.037	235
TRINITY_Contig9236	2	p3	(CTG)4	12	3144	3155	CGCGTCCTACATCTTCCTCC	59.969	TTGTCCAGAGTAGCTGCTGC	60.037	235
TRINITY_Contig9238	1	p3	(CGC)4	12	399	410	CTCCCTCCCTGATGGTCTGA	60.032	ATGATGTCCTTGTTGCCGGT	59.962	255
TRINITY_Contig9239	1	p2	(CT)9	18	198	215	AGACATCCCAAAGCTGGAGC	60.035	AGGAGCATAGAAGCTGGGGA	60.031	103
TRINITY_Contig9241	1	p5	(TACAA)3	15	81	95	CCCCAAGACCCAAGATCACC	60.034	TCTTTGTTTGCGCGTTCTGG	59.971	100
TRINITY_Contig9243	1	p3	(CGC)4	12	582	593	GCGAAGTGCGGGATAGATGA	59.968	TAACCGATCACCCATACGCG	59.969	124
TRINITY_Contig9243	2	p3	(CGA)4	12	943	954	GCGAAGTGCGGGATAGATGA	59.968	TAACCGATCACCCATACGCG	59.969	124
TRINITY_Contig9243	3	p3	(CGG)4	12	2126	2137	GCGAAGTGCGGGATAGATGA	59.968	TAACCGATCACCCATACGCG	59.969	124
TRINITY_Contig9245	1	p3	(GCG)4	12	2048	2059	TATCCCCCTCTCTCCCCTCT	60.026	TCAAGCCAAACATTGCCAGC	59.966	177
TRINITY_Contig9246	1	c	(GGC)4gggagacgagagcaggtggagcggcatggctgcggcgctgtctgctgggcggcg(GGC)4	78	2041	2118	ACACCGTTACAGCCTGAGTG	59.966	TACGGGGTGTCTCTCGTCTT	59.963	199
TRINITY_Contig9247	1	p2	(AG)7	14	1845	1858	CTGGATTAGACCGTCGGACG	59.971	CTACCCAGGGCAGCAAAAGA	59.962	121
TRINITY_Contig9248	1	p3	(TGA)4	12	1030	1041	TCCACAGCGGTAGTTGGAAC	59.966	ATGGCTTCACCCAATCCAGG	60.033	184
TRINITY_Contig9250	1	c	(CGGAGG)5(CGGAAG)3	48	2963	3010	GCACCCTCCGTTCTTCTCAA	59.965	CCTCTTCTCTCTCTGCCCCT	60.032	189
TRINITY_Contig9253	1	p4	(TCTT)3	12	512	523	CAGCTTGAGCAGGATGTCCA	60.036	GTCCACAGGGTACATGGTGG	60.035	205
TRINITY_Contig9253	2	c	(GGC)4catggggcggtgggaggtggggaattcgggcgcggtggagccgtgcaat(CCTGAG)3	79	2087	2165	CAGCTTGAGCAGGATGTCCA	60.036	GTCCACAGGGTACATGGTGG	60.035	205
TRINITY_Contig9254	1	p3	(TCG)4	12	739	750	CCATGGCAATTAAGACGGCG	59.971	CTATATCTCGCGCCCGGATC	59.97	233
TRINITY_Contig9254	2	p3	(GAC)5	15	1549	1563	CCATGGCAATTAAGACGGCG	59.971	CTATATCTCGCGCCCGGATC	59.97	233
TRINITY_Contig9254	3	p4	(GGTA)3	12	1919	1930	CCATGGCAATTAAGACGGCG	59.971	CTATATCTCGCGCCCGGATC	59.97	233
TRINITY_Contig9254	4	p3	(GAT)4	12	2076	2087	CCATGGCAATTAAGACGGCG	59.971	CTATATCTCGCGCCCGGATC	59.97	233
TRINITY_Contig9256	1	p3	(GCT)4	12	1193	1204	CCTACACTATCACTGCGCGT	59.899	AAGGGGAAGACAACGACGAC	59.967	277
TRINITY_Contig9256	2	p2	(GA)15	30	2104	2133	CCTACACTATCACTGCGCGT	59.899	AAGGGGAAGACAACGACGAC	59.967	277
TRINITY_Contig9257	1	p3	(CCT)5	15	1395	1409	TTTTGGCGCACTCGTCTTTG	59.971	GATCAACGTGTGGAGCTCA	60.037	200
TRINITY_Contig9259	1	p3	(CAA)4	12	34	45	GCACAGGGACATACGAGGAG	59.897	CCTCCAAGCACGTCTTCCAT	60.036	150
TRINITY_Contig9259	2	p3	(GCG)4	12	1721	1732	GCACAGGGACATACGAGGAG	59.897	CCTCCAAGCACGTCTTCCAT	60.036	150
TRINITY_Contig9259	3	c	(GCG)4(AGG)4	24	1973	1996	GCACAGGGACATACGAGGAG	59.897	CCTCCAAGCACGTCTTCCAT	60.036	150
TRINITY_Contig9260	1	p6	(GGCGCC)3	18	1558	1575	TAGCGAACCTGCTTGACCTG	60.037	GAGGTACTACGTGCCCATGG	59.896	242
TRINITY_Contig9261	1	p3	(CCG)4	12	143	154	CATCGCTTCGCTTCTTGCTC	59.974	TCGCGGATCTGCTCATCTTC	59.969	156
TRINITY_Contig9261	2	p3	(CAG)4	12	1692	1703	CATCGCTTCGCTTCTTGCTC	59.974	TCGCGGATCTGCTCATCTTC	59.969	156
TRINITY_Contig9264	1	p3	(CGC)7	21	1905	1925	AAGGGGGCAGACTACAATGC	60.034	CACAGACTGGAGAAAGGCGT	59.966	158
TRINITY_Contig9264	2	p4	(GGAG)3	12	2128	2139	AAGGGGGCAGACTACAATGC	60.034	CACAGACTGGAGAAAGGCGT	59.966	158
TRINITY_Contig9267	1	p2	(CT)7	14	172	185	ATGCATGGCGGGATAGATGG	60.035	ATGTCCTGCAATGCATCCCA	60.033	167
TRINITY_Contig9268	1	p3	(CCA)4	12	34	45	CAGGGTTCAAGTGCAACAGC	59.969	ACACGACGACTAGTACGGGA	60.037	197
TRINITY_Contig9268	2	p3	(CCG)4	12	250	261	CAGGGTTCAAGTGCAACAGC	59.969	ACACGACGACTAGTACGGGA	60.037	197
TRINITY_Contig9268	3	c*	(TGG)3(T<GG>CC)(GGCC)2	22	1766	1787	CAGGGTTCAAGTGCAACAGC	59.969	ACACGACGACTAGTACGGGA	60.037	197
TRINITY_Contig9269	1	p3	(GGA)4	12	342	353	ATGTTCGGCTTCGTCACCTT	59.965	TCTGCAGCTTCCTGAACCTG	59.964	148
TRINITY_Contig9269	2	p3	(GCC)4	12	642	653	ATGTTCGGCTTCGTCACCTT	59.965	TCTGCAGCTTCCTGAACCTG	59.964	148
TRINITY_Contig9269	3	p3	(GGC)6	18	758	775	ATGTTCGGCTTCGTCACCTT	59.965	TCTGCAGCTTCCTGAACCTG	59.964	148
TRINITY_Contig9271	1	p3	(CCT)4	12	123	134	GACGTGGAAGCAACCTCTGA	59.966	TGCAAGGTCGAGCACAGATT	59.965	264
TRINITY_Contig9271	2	p4	(TTTC)3	12	1404	1415	GACGTGGAAGCAACCTCTGA	59.966	TGCAAGGTCGAGCACAGATT	59.965	264
TRINITY_Contig9271	3	p3	(GGA)4	12	3977	3988	GACGTGGAAGCAACCTCTGA	59.966	TGCAAGGTCGAGCACAGATT	59.965	264
TRINITY_Contig9272	1	p4	(CGGC)3	12	156	167	GGCCTGCAAAGTTGTGAGTG	59.969	TACTTGTTCATTCGCGGCCT	60.037	100
TRINITY_Contig9273	1	p3	(CGC)4	12	179	190	CCGGTTGCAAGTTGTCAAGG	59.969	TCTTCTGGATGGCCTGCTTG	60.035	169
TRINITY_Contig9274	1	c	(GCC)4gcgccgccgccaccgccgtcgtggtcgacgtcggagaccgcgc(CGT)5	70	608	677	GTGTCCGAGCTCAACAAGGA	59.966	CAGGATATGAACTTGCGCGC	60.042	196
TRINITY_Contig9274	2	c	(CGT)4(CCT)4	24	1287	1310	GTGTCCGAGCTCAACAAGGA	59.966	CAGGATATGAACTTGCGCGC	60.042	196
TRINITY_Contig9274	3	p3	(CCT)4	12	1551	1562	GTGTCCGAGCTCAACAAGGA	59.966	CAGGATATGAACTTGCGCGC	60.042	196
TRINITY_Contig9276	1	p3	(CGC)4	12	799	810	TGCAGCAGCTTTGAGATGGA	59.963	TGCAGCAGCTTTGAGATGGA	59.963	107
TRINITY_Contig9276	2	p2	(CG)6	12	1238	1249	TGCAGCAGCTTTGAGATGGA	59.963	TGCAGCAGCTTTGAGATGGA	59.963	107
TRINITY_Contig9281	1	p4	(CCGG)3	12	1219	1230	CCGCAGCACAAGCAAAAGAT	60.039	GCTTGCGATCGTCCATCAAC	59.973	120
TRINITY_Contig9282	1	p5	(AGGAG)4	20	2119	2138	AGGCTGCCAAGATTACACCC	60.034	ATCTCAGCCTCCCAATCCCT	60.029	247
TRINITY_Contig9284	1	p3	(CGG)4	12	2055	2066	AGTCAGCCACGTCAGTTGAG	59.967	CGTGCTGGAGTGGAGGAATT	60.036	107
TRINITY_Contig9285	1	c	(CGGCGT)3cgcgcttggacggggagctggaggcgcgggt(CGC)4	61	1996	2056	CCATCTCCTCCTTGCTTGCA	60.035	GACAGCAACAATGGCCAAGG	60.038	258
TRINITY_Contig9286	1	p3	(CGG)4	12	2046	2057	GCTCGTTCTGGAGGGAAACA	59.965	GCTTTTGAGGTTGCTGCGAA	59.969	175
TRINITY_Contig9287	1	p5	(CTCGG)3	15	106	120	ACCAGAGGATCAAAGCCTGC	60.035	ATCATCCGTGCGCTCTAGTG	59.969	105
TRINITY_Contig9288	1	p3	(GGC)4	12	2080	2091	AACAGATGCTGCCTCCTCAC	60.036	ACATCAACTCGGCGCACTCA	59.966	146
TRINITY_Contig9289	1	p4	(CCGC)3	12	216	227	TGGTTGCAGAGGAAGACGTC	59.966	CAGCGTCTCTCATCCTCGAC	59.971	172
TRINITY_Contig9290	1	c	(CGC)4agcgttccccgaaccctaacccccccccctctcca(TCCCC)3g(CGC)5	79	1	79	ACTACGAGGGAGGGAGCAAT	60.032	CTTGAGCCCGGTGTGGTAAT	60.035	104
TRINITY_Contig9291	1	p4	(TTGA)3	12	173	184	GGTCGTACTAAACCCCGCAA	60.037	GATGGGAATGGCGGATTTGC	59.967	251
TRINITY_Contig9291	2	p4	(AATA)3	12	476	487	GGTCGTACTAAACCCCGCAA	60.037	GATGGGAATGGCGGATTTGC	59.967	251
TRINITY_Contig9291	3	p4	(GCCG)3	12	649	660	GGTCGTACTAAACCCCGCAA	60.037	GATGGGAATGGCGGATTTGC	59.967	251
TRINITY_Contig9292	1	c	(GC)7gagagcacactcacttgtacggccggagagaaggaaatcaatcttcttta(TGGC)4	80	1993	2072	CACGTACTTGGGTGACAGCT	59.966	CTGCTATCCTTCTCGAGCGG	60.04	123
TRINITY_Contig9294	1	p5	(CGGCA)3	15	20	34	GTTGGTGTTGGTGTTCGAGC	59.971	CGGCGTGCTACAACATCATG	59.973	118
TRINITY_Contig9294	2	p3	(GCT)4	12	188	199	GTTGGTGTTGGTGTTCGAGC	59.971	CGGCGTGCTACAACATCATG	59.973	118
TRINITY_Contig9295	1	p3	(CAC)4	12	159	170	TCGTCCAGGTGTACAGCAAC	59.967	CAAAGCCATCGTACTTGCCG	59.903	138
TRINITY_Contig9295	2	p3	(CCT)5	15	319	333	TCGTCCAGGTGTACAGCAAC	59.967	CAAAGCCATCGTACTTGCCG	59.903	138
TRINITY_Contig9295	3	c	(CTC)5tgcctctccctgccccgcctccccttcgcgctg(CCGCTC)3	66	576	641	TCGTCCAGGTGTACAGCAAC	59.967	CAAAGCCATCGTACTTGCCG	59.903	138
TRINITY_Contig9295	4	p3	(CTA)4	12	797	808	TCGTCCAGGTGTACAGCAAC	59.967	CAAAGCCATCGTACTTGCCG	59.903	138
TRINITY_Contig9296	1	p3	(CGC)4	12	648	659	CAAATCATGTCGCGTGTCCC	59.903	AGTACGTGTTCAGCTCGTGG	60.039	196
TRINITY_Contig9296	2	p3	(GCC)4	12	1527	1538	CAAATCATGTCGCGTGTCCC	59.903	AGTACGTGTTCAGCTCGTGG	60.039	196
TRINITY_Contig9296	3	p3	(CGC)6	18	1898	1915	CAAATCATGTCGCGTGTCCC	59.903	AGTACGTGTTCAGCTCGTGG	60.039	196
TRINITY_Contig9298	1	p3	(CGA)4	12	1083	1094	GAACAGCCTCGGGTCAAAGA	59.965	TGTCTCTGGCAGCTAAACCG	60.037	198
TRINITY_Contig9299	1	p5	(CCGCG)3	15	254	268	ACTTGCCTTTGCCTACCTCC	59.962	GACTGGTGCGTGGTTAATGC	59.832	209
TRINITY_Contig9299	2	p6	(GGCACC)3	18	447	464	ACTTGCCTTTGCCTACCTCC	59.962	GACTGGTGCGTGGTTAATGC	59.832	209
TRINITY_Contig9299	3	p3	(CGG)4	12	721	732	ACTTGCCTTTGCCTACCTCC	59.962	GACTGGTGCGTGGTTAATGC	59.832	209
TRINITY_Contig9300	1	c	(GCG)4acctgggaatggaggagc(GGA)4gagcacgagctcgcgaacgccacatcgtcgccggcgtcct(CGG)4	94	1963	2056	AGGAGCTGGGCCAAGATCTA	60.031	CAAGTCCTTTGCCACGGTTG	59.969	223
TRINITY_Contig9302	1	p6	(AGGAAG)3	18	204	221	TGTGCGGGTAGGAGTCCTTA	59.96	TCTTCAACCTCGACGGCTTC	60.039	231
TRINITY_Contig9302	2	p4	(GCTA)3	12	1838	1849	TGTGCGGGTAGGAGTCCTTA	59.96	TCTTCAACCTCGACGGCTTC	60.039	231
TRINITY_Contig9302	3	p4	(ATTC)4	16	2018	2033	TGTGCGGGTAGGAGTCCTTA	59.96	TCTTCAACCTCGACGGCTTC	60.039	231
TRINITY_Contig9303	1	c	(CTT)5ccctggtta(TCAGT)3	39	92	130	GGTGTACCTGCAGAACGACA	59.967	ATGAACTCGCCGTTGGTCTT	59.965	105
TRINITY_Contig9304	1	p5	(CATTG)3	15	657	671	GCATGTCGATCTTGCCGAAC	59.973	AGCCGAGAAGGGAGGAAGAT	60.032	229
TRINITY_Contig9304	2	c	(CAGG)3cagccgcggggggacggacacggccagcaggtggaaaccaacgggcgagtgccggctcggcctc(GGCT)3	88	1890	1977	GCATGTCGATCTTGCCGAAC	59.973	AGCCGAGAAGGGAGGAAGAT	60.032	229
TRINITY_Contig9305	1	p3	(GTA)5	15	1885	1899	GGAAGGACGCCTAGTACGTG	59.9	ATCCCGGGCGTCAAAAGAAT	60.034	175
TRINITY_Contig9305	2	p4	(CAGG)3	12	2094	2105	GGAAGGACGCCTAGTACGTG	59.9	ATCCCGGGCGTCAAAAGAAT	60.034	175
TRINITY_Contig9306	1	c	(CCCGTC)3ccg(GCC)4	33	646	678	GTCGGGATCGTCCAGTCATC	59.969	AGAAGGTGCTCATTGTCGGG	60.036	235
TRINITY_Contig9307	1	p3	(GGC)6	18	1452	1469	ACCTTCCTAAGAACTGCCGC	60.036	CATCTCCTTCGTCCTCGTCG	59.972	249
TRINITY_Contig9307	2	p3	(GGC)5	15	1746	1760	ACCTTCCTAAGAACTGCCGC	60.036	CATCTCCTTCGTCCTCGTCG	59.972	249
TRINITY_Contig9308	1	p2	(AT)10	20	625	644	TCGCTCACCTGACTTTGACC	59.966	TGAGTGTTTCCCCTACGCAC	59.966	258
TRINITY_Contig9309	1	p3	(GGC)4	12	1652	1663	GCGTCTCTCCTACGTACAGC	59.972	CGATCTGCCGAGAGAGCAAT	59.968	128
TRINITY_Contig9312	1	p6	(GCCGGA)3	18	1821	1838	AGGTGGGGTGCAAAGAAACA	60.033	GGATGGCTGCTCTTCAGGTT	60.035	267
TRINITY_Contig9312	2	p3	(GGC)7	21	1965	1985	AGGTGGGGTGCAAAGAAACA	60.033	GGATGGCTGCTCTTCAGGTT	60.035	267
TRINITY_Contig9314	1	p3	(CGC)4	12	1958	1969	GCCACAAGTACGAACTGGGA	59.966	AGGAAAAACAGGAAACGCGC	59.97	195
TRINITY_Contig9314	2	p3	(CCT)4	12	2556	2567	GCCACAAGTACGAACTGGGA	59.966	AGGAAAAACAGGAAACGCGC	59.97	195
TRINITY_Contig9317	1	p3	(AGG)4	12	2048	2059	GGCTGCATCTGTCAGAGGTT	60.036	GAAACTGCCCTCATGGAGCT	60.035	240
TRINITY_Contig9318	1	p3	(CGG)5	15	926	940	TCGCTCCTCCTGATCTCACA	60.034	CAGCTGCTGACACCTCTTCA	59.966	118
TRINITY_Contig9319	1	c	(GCCC)3ccctacccggccgcacccccagcgcgc(CCAG)3	52	32	83	AGAAGTGCTCAGTTCACCCG	59.966	GCAGTGGTCTTGCAACACTG	59.97	193
TRINITY_Contig9322	1	p5	(AACGG)3	15	153	167	AATCACTCGCTTCCCCGTTT	59.964	GTTTGCCGGTTTGGTTCTCC	59.968	183
TRINITY_Contig9322	2	p6	(CCGCCC)3	18	379	396	AATCACTCGCTTCCCCGTTT	59.964	GTTTGCCGGTTTGGTTCTCC	59.968	183
TRINITY_Contig9323	1	p3	(GGC)4	12	1127	1138	AAGCTGGCGTTCTGGATCAA	59.963	TGTTCCTCCGACGTTCACTG	59.968	129
TRINITY_Contig9323	2	p3	(CGC)4	12	1721	1732	AAGCTGGCGTTCTGGATCAA	59.963	TGTTCCTCCGACGTTCACTG	59.968	129

TRINITY_Contig9325	1	c	(GCG)4cggtcgccgccaccctgccctcgcctctggcaccggcggcggcaaccaccgcccac(CTG)4	80	464	543	GTCAGAGGTTGAGTCCGTGG	60.038	CTTCCCACCATGAACGACGA	60.038	137
TRINITY_Contig9325	2	p3	(GCC)6	18	831	848	GTCAGAGGTTGAGTCCGTGG	60.038	CTTCCCACCATGAACGACGA	60.038	137
TRINITY_Contig9326	1	p3	(CAG)5	15	189	203	CGGGATCCAGTGATGAGCTC	59.967	CGTCCAAGTCCAACTCGTCA	59.968	240
TRINITY_Contig9326	2	p3	(CGC)5	15	1516	1530	CGGGATCCAGTGATGAGCTC	59.967	CGTCCAAGTCCAACTCGTCA	59.968	240
TRINITY_Contig9327	1	c	(CGC)4cggggccgcga(CGC)4	35	705	739	CTGTACGTGTTCACCACCGA	59.969	ACTTGCTTCATCCTGGTGGG	59.961	242
TRINITY_Contig9327	2	p5	(CGGCG)3	15	1786	1800	CTGTACGTGTTCACCACCGA	59.969	ACTTGCTTCATCCTGGTGGG	59.961	242
TRINITY_Contig9328	1	p4	(GCCG)3	12	1939	1950	CGAACCAAGCTAGGCAAGGA	60.036	CCTTCTCCTCATCGTCGTCG	59.972	279
TRINITY_Contig9329	1	p3	(GCT)5	15	2565	2579	GCAGGCACTTCATATGCAGC	59.97	GGGGAGGTAGTGATCAGGGT	60.031	226
TRINITY_Contig9330	1	p3	(GCG)4	12	1040	1051	AGACTGCTAGCCTGCTAGGT	60.033	TCGTGGGGTTTTTAGTGCGA	59.894	190
TRINITY_Contig9336	1	p2	(GC)6	12	715	726	GCCGTCTCTGCCTTGTTTTG	60.04	AAAGGAGAGCCGAAGCCATC	60.107	261
TRINITY_Contig9336	2	p4	(CCGT)3	12	959	970	GCCGTCTCTGCCTTGTTTTG	60.04	AAAGGAGAGCCGAAGCCATC	60.107	261
TRINITY_Contig9338	1	c	(GCG)6gccccccgg(AGC)4(ACC)4	51	1268	1318	GAGATCTACGAGCTCACGCC	60.041	GATCACAGCTCAGAGGCTCC	59.895	180
TRINITY_Contig9341	1	p3	(GAC)4	12	92	103	TATCCGGTGTCCATGAGGGT	60.032	TCACTAACACTGGCCGAACC	59.966	207
TRINITY_Contig9341	2	p3	(GCC)6	18	361	378	TATCCGGTGTCCATGAGGGT	60.032	TCACTAACACTGGCCGAACC	59.966	207
TRINITY_Contig9341	3	p3	(GGA)4	12	484	495	TATCCGGTGTCCATGAGGGT	60.032	TCACTAACACTGGCCGAACC	59.966	207
TRINITY_Contig9341	4	p4	(CCGG)4	16	1052	1067	TATCCGGTGTCCATGAGGGT	60.032	TCACTAACACTGGCCGAACC	59.966	207
TRINITY_Contig9343	1	p3	(GAG)4	12	1524	1535	ACGTTTGGTCGCCTAAGGTT	59.893	ACAGGCTGCTGTACAAGGTC	59.965	278
TRINITY_Contig9343	2	p4	(TGCG)3	12	1992	2003	ACGTTTGGTCGCCTAAGGTT	59.893	ACAGGCTGCTGTACAAGGTC	59.965	278
TRINITY_Contig9344	1	p4	(AATA)3	12	1946	1957	GCAATGTGGCACTTTCAGCA	59.968	AGTCATGGTGCTCGCTTTCA	59.965	277
TRINITY_Contig9345	1	p3	(GAA)4	12	607	618	AGGAGGTTGAAGACGAAGCG	60.038	TGGCTCAGTACTCCGTAGCT	60.034	194
TRINITY_Contig9346	1	p3	(GCT)5	15	511	525	TTTCTCGGTGTACGTCGCAA	59.969	GTGGCACATTCCTCCCGTTA	60.036	113
TRINITY_Contig9346	2	p3	(GGC)4	12	1942	1953	TTTCTCGGTGTACGTCGCAA	59.969	GTGGCACATTCCTCCCGTTA	60.036	113
TRINITY_Contig9349	1	c	(CGT)5tgccggtgctgccgt(CGCCGG)3aggtggtgagc(GTG)4	71	757	827	GACGAGGGGTGGTTTTGCTA	59.964	TCCTAGCTCAGCTTCCCCAT	60.031	169
TRINITY_Contig9349	2	p3	(GCC)4	12	1017	1028	GACGAGGGGTGGTTTTGCTA	59.964	TCCTAGCTCAGCTTCCCCAT	60.031	169
TRINITY_Contig9349	3	p5	(CTGAG)3	15	1622	1636	GACGAGGGGTGGTTTTGCTA	59.964	TCCTAGCTCAGCTTCCCCAT	60.031	169
TRINITY_Contig9349	4	c	(GATC)3gacaagcc(CACT)3	32	1993	2024	GACGAGGGGTGGTTTTGCTA	59.964	TCCTAGCTCAGCTTCCCCAT	60.031	169
TRINITY_Contig9350	1	p4	(CGTG)3	12	113	124	TCAGCAGAGGAGGAGCTCAT	60.032	GGCAGGGGGATCAAATGGAA	60.032	214
TRINITY_Contig9351	1	p3	(CGC)4	12	148	159	AAGACGCGCAGAAGACAGAA	59.968	CATACTCTCGGACGCCATCC	60.039	226
TRINITY_Contig9352	1	p3	(GGC)6	18	1868	1885	TATTCCGCCCTCCTACCACA	60.032	AGATTGGGGGTACTGCGTTG	60.035	135
TRINITY_Contig9353	1	c	(AGCCT)3a(GCC)4	28	1887	1914	CTGCGCATCTCTTCCTCGAT	59.968	GCGATAAACGACGAGGGGAT	59.968	227
TRINITY_Contig9354	1	p3	(CGG)4	12	1952	1963	ACATTCAGCAGCTTCACGGA	59.965	AACCGTACTTTTGCACGTGC	59.972	246
TRINITY_Contig9356	1	p4	(GGAA)3	12	53	64	CCTGGGCCCAAACTCATCAT	60.033	GAGGGAAGGGAACTCAACGG	60.036	116
TRINITY_Contig9356	2	p6	(TAAAAA)3	18	234	251	CCTGGGCCCAAACTCATCAT	60.033	GAGGGAAGGGAACTCAACGG	60.036	116
TRINITY_Contig9357	1	p3	(CGC)4	12	1811	1822	CCGACACCTTGGCATTCTCT	60.036	GGGATCCACGGCTATCACTG	59.966	163
TRINITY_Contig9357	2	p3	(CGC)4	12	1937	1948	CCGACACCTTGGCATTCTCT	60.036	GGGATCCACGGCTATCACTG	59.966	163
TRINITY_Contig9358	1	p3	(AGC)4	12	1834	1845	GCAATACAACGCGTACGCAT	59.974	CCTGGTCATCAGCAAAGGGT	59.961	213
TRINITY_Contig9360	1	p3	(CGC)4	12	685	696	TCGCTCTTGAGGTGGTTCAC	59.966	CTGAGCCTCCCGATCATCAC	59.967	197
TRINITY_Contig9363	1	p3	(CCT)4	12	456	467	ACACCAACGTCCGGAAGATC	60.038	AACCGTCCATTCCCCGATTC	60.107	263
TRINITY_Contig9365	1	p2	(AC)12	24	143	166	TTGCCCAGGAACTCGAAGTC	59.965	GTCGGCTTTCAGACCAAGGA	59.965	161
TRINITY_Contig9365	2	p3	(GCG)4	12	875	886	TTGCCCAGGAACTCGAAGTC	59.965	GTCGGCTTTCAGACCAAGGA	59.965	161
TRINITY_Contig9365	3	p6	(GGCCAG)4	24	1188	1211	TTGCCCAGGAACTCGAAGTC	59.965	GTCGGCTTTCAGACCAAGGA	59.965	161
TRINITY_Contig9365	4	c	(ACG)4ccggcgccgcggacgcgccgcggaaagagcagga(CAG)5	61	1764	1824	TTGCCCAGGAACTCGAAGTC	59.965	GTCGGCTTTCAGACCAAGGA	59.965	161
TRINITY_Contig9370	1	p3	(CGC)4	12	1042	1053	CCCGCACCTCAAAACTCTCT	59.965	TTTGTACGCCTTTTGCCGTG	59.97	107
TRINITY_Contig9370	2	c	(GGCG)3cggtggggaaggaaggggaagagggaggagtggccgcagtaaagacgggctctttaaaaagaggagcagaaatggtgcagg(AGC)4	105	1517	1621	CCCGCACCTCAAAACTCTCT	59.965	TTTGTACGCCTTTTGCCGTG	59.97	107
TRINITY_Contig9370	3	p3	(GCG)4	12	1962	1973	CCCGCACCTCAAAACTCTCT	59.965	TTTGTACGCCTTTTGCCGTG	59.97	107
TRINITY_Contig9373	1	p3	(GAG)4	12	1929	1940	ATCAACCGGGAACATGCCAT	60.033	GACGCAGACTTCTTGGTGGA	59.966	152
TRINITY_Contig9374	1	p5	(AGAGG)3	15	127	141	CTCACCGAGCTGAAACAGGT	59.966	GGAAAGGGAGGAGGGGATCT	60.03	207
TRINITY_Contig9375	1	p4	(TCCT)3	12	2136	2147	CCTGTTTGCCCTTCCCCTAG	60.034	CCGAGCAAACACCCTTGTTG	59.969	169
TRINITY_Contig9376	1	p3	(GGC)5	15	870	884	GTATCCCTCGGCTGCTCTTC	59.967	CAGATGGAAATGCGCTTCGG	59.971	156
TRINITY_Contig9376	2	c	(TC)6cctctcaccccgcgg(GTC)4	39	1944	1982	GTATCCCTCGGCTGCTCTTC	59.967	CAGATGGAAATGCGCTTCGG	59.971	156
TRINITY_Contig9377	1	c	(CGC)4agg(CTC)4	27	564	590	GCGAATTGTTTGGCCTGTGT	59.968	CTGACGCCGATTCTCCTACC	59.969	195
TRINITY_Contig9377	2	p3	(GCG)6	18	731	748	GCGAATTGTTTGGCCTGTGT	59.968	CTGACGCCGATTCTCCTACC	59.969	195
TRINITY_Contig9377	3	p3	(GAC)4	12	896	907	GCGAATTGTTTGGCCTGTGT	59.968	CTGACGCCGATTCTCCTACC	59.969	195
TRINITY_Contig9377	4	p3	(GCC)4	12	1259	1270	GCGAATTGTTTGGCCTGTGT	59.968	CTGACGCCGATTCTCCTACC	59.969	195
TRINITY_Contig9377	5	p3	(GGA)4	12	1421	1432	GCGAATTGTTTGGCCTGTGT	59.968	CTGACGCCGATTCTCCTACC	59.969	195
TRINITY_Contig9377	6	p6	(CGGCGC)3	18	1675	1692	GCGAATTGTTTGGCCTGTGT	59.968	CTGACGCCGATTCTCCTACC	59.969	195
TRINITY_Contig9378	1	p6	(CATTTG)3	18	143	160	GCCATCGACATTCCATGCAC	59.97	GTTAGATCACGAGCCCGGAG	59.969	183
TRINITY_Contig9379	1	c	(CGAG)3tcaagagtc(AG)12	45	1996	2040	GCGTTGTGATGACCTCTGGA	60.037	CCGGGCAAATGCATCTGATG	59.968	146
TRINITY_Contig9381	1	p3	(TCC)4	12	1845	1856	GGTCCTCCAGGCTTGTTCTC	60.036	TATCCCTGTCGACTCCGGTT	60.034	116
TRINITY_Contig9383	1	p2	(AT)9	18	1723	1740	ATCATGGCGTTCACCACCAT	60.034	GACACGCTCATCATCGGCTA	59.969	107
TRINITY_Contig9385	1	p4	(TAGC)3	12	50	61	GCGTACCTGCATCTCATCGA	59.969	CCTTCTTGACGTTCCCCCTC	60.036	112
TRINITY_Contig9385	2	c	(CGG)4acggggccagggcgcacatcgccaacctcgacaaggtgctcgggaagccgccgaggcccgcgagccacgccgccag(GCAGGA)3	106	345	450	GCGTACCTGCATCTCATCGA	59.969	CCTTCTTGACGTTCCCCCTC	60.036	112
TRINITY_Contig9386	1	c	(CGC)4gcacccgaagaagagcgccgcgtcgtgt(CCG)5	55	528	582	CATGCTCACGTGGTCCTTCT	60.037	CTCTACTTCGAGCAGCTCCG	59.971	273
TRINITY_Contig9386	2	p2	(TC)9	18	2114	2131	CATGCTCACGTGGTCCTTCT	60.037	CTCTACTTCGAGCAGCTCCG	59.971	273
TRINITY_Contig9388	1	p3	(AAG)4	12	142	153	GACATCCTCCTCGGCAACAA	60.036	GACATCTCGACCGATCACCC	59.969	216
TRINITY_Contig9389	1	c	(CCG)4cgcgtcctgccgccgccgctgatcctgatccgttggccacggccgccttggcttgcttgggc(GCA)4	86	1330	1415	CAGCCTTTGCCCTTGAAACC	59.966	ACTGCATCCCAAGTGTCTGG	59.963	103
TRINITY_Contig9389	2	c	(CCCA)3(CCTA)3	24	1872	1895	CAGCCTTTGCCCTTGAAACC	59.966	ACTGCATCCCAAGTGTCTGG	59.963	103
TRINITY_Contig9389	3	c	(AGGC)3aaggcaa(CCAC)4tgatggtgccg(GGCT)3ggcagcgc(GGAG)3ggtggttgcct(TGCC)3	101	2002	2102	CAGCCTTTGCCCTTGAAACC	59.966	ACTGCATCCCAAGTGTCTGG	59.963	103
TRINITY_Contig9390	1	p3	(AGC)5	15	205	219	CGAGCTAGCTCCATCAAGCA	59.896	AAAGGCAAGGCAAACCGAAC	59.896	165
TRINITY_Contig9390	2	c	(TGCTGG)3tggttctccc(GCT)4	40	1396	1435	CGAGCTAGCTCCATCAAGCA	59.896	AAAGGCAAGGCAAACCGAAC	59.896	165
TRINITY_Contig9390	3	p3	(GCG)4	12	1549	1560	CGAGCTAGCTCCATCAAGCA	59.896	AAAGGCAAGGCAAACCGAAC	59.896	165
TRINITY_Contig9390	4	p3	(GGA)4	12	1790	1801	CGAGCTAGCTCCATCAAGCA	59.896	AAAGGCAAGGCAAACCGAAC	59.896	165
TRINITY_Contig9392	1	p3	(GCC)4	12	1481	1492	GCCTGTCTGAGAACACACGA	59.968	GAGCCACAAAACGGGAACAC	59.97	245
TRINITY_Contig9394	1	c	(CCTCCG)3ccttcgcggcggcgccgggggacgtggccgcgatgctgccggactcgccgc(CGCGG)3	84	696	779	AGACAATGGAGCAGCAGGTC	60.036	GGAGTGGATTTGGAGCTGCT	60.035	221
TRINITY_Contig9394	2	p4	(AAAG)3	12	1235	1246	AGACAATGGAGCAGCAGGTC	60.036	GGAGTGGATTTGGAGCTGCT	60.035	221
TRINITY_Contig9394	3	c	(CAG)5acgcattt(GCA)4	35	1613	1647	AGACAATGGAGCAGCAGGTC	60.036	GGAGTGGATTTGGAGCTGCT	60.035	221
TRINITY_Contig9395	1	p2	(TA)6	12	1055	1066	TAAAAGTGCCCTTGCGTTGC	59.969	CGACTGAGCCTACAAGCCAT	59.823	176
TRINITY_Contig9395	2	p4	(GTTG)3	12	1707	1718	TAAAAGTGCCCTTGCGTTGC	59.969	CGACTGAGCCTACAAGCCAT	59.823	176
TRINITY_Contig9396	1	p3	(TTC)4	12	1757	1768	TGAACTTCTTCCCGGACGTG	59.967	AAGAGGTGAAGAGCGTCAGC	60.038	105
TRINITY_Contig9397	1	p4	(GCCG)3	12	1604	1615	GGTGGAGTGTGAAAGCGAGA	59.966	GGGGAGCAGTCAAAGTACCC	60.035	123
TRINITY_Contig9397	2	p2	(AG)6	12	1799	1810	GGTGGAGTGTGAAAGCGAGA	59.966	GGGGAGCAGTCAAAGTACCC	60.035	123
TRINITY_Contig9399	1	p3	(GCG)7	21	1837	1857	TTGAATGTGCTGCCAACGTG	59.969	CAACATCATGAACCCGGGGA	60.035	236
TRINITY_Contig9400	1	p3	(CGG)5	15	365	379	TCGAGGAGGAAGAGGTCGTT	59.962	CTGCTTCGTGGTGGAGTTCT	59.966	108
TRINITY_Contig9400	2	p3	(CCT)6	18	1634	1651	TCGAGGAGGAAGAGGTCGTT	59.962	CTGCTTCGTGGTGGAGTTCT	59.966	108
TRINITY_Contig9403	1	c	(TCC)5t(CTC)4	28	552	579	ATGGAAGCATGGGTCTGTGG	60.034	CGTCATCAGCTCGAGAGGAC	59.971	103
TRINITY_Contig9403	2	p3	(GCG)5	15	1005	1019	ATGGAAGCATGGGTCTGTGG	60.034	CGTCATCAGCTCGAGAGGAC	59.971	103
TRINITY_Contig9404	1	p3	(GCG)4	12	491	502	TTCTGGCACGTTGTCATCGA	59.967	CCATGCCATGTCATGCCATG	59.967	147
TRINITY_Contig9404	2	c	(CCG)4ctgctgctaaccatgcaggatcctgattagttacatcag(GCTCA)3gctagctgctccctgattggctcggaactgaccagcgtgtggaatctgacaccgcgcc(GCCG)3cccggtcggggccgcacgattttt(CTC)4	171	1245	1415	TTCTGGCACGTTGTCATCGA	59.967	CCATGCCATGTCATGCCATG	59.967	147
TRINITY_Contig9404	3	p4	(GATG)3	12	1700	1711	TTCTGGCACGTTGTCATCGA	59.967	CCATGCCATGTCATGCCATG	59.967	147
TRINITY_Contig9404	4	c	(GTTG)3gttagatgttacaagaacagtgatcagtactactgaatactgatcgatcgccgcccagcccat(CATG)3	87	1847	1933	TTCTGGCACGTTGTCATCGA	59.967	CCATGCCATGTCATGCCATG	59.967	147
TRINITY_Contig9405	1	p5	(CCGCC)3	15	41	55	GGACATTGCAGGAAGGGTCA	59.962	AGTGCGTCAATGGCTGATGA	60.036	117

TRINITY_Contig9406	1	c	(CCT)5gaaacagtcaccggcgcctcctccaatcccgtcctcctccatgct(GCC)5	75	150	224	GTGCTTGCAGGAAGAGGACT	59.964	TCGTTGCCTTCATCGTTCGA	60.039	189
TRINITY_Contig9406	2	p4	(CTCC)3	12	504	515	GTGCTTGCAGGAAGAGGACT	59.964	TCGTTGCCTTCATCGTTCGA	60.039	189
TRINITY_Contig9407	1	p3	(AGG)5	15	54	68	GGGTTTCTTGGATGCCTGGA	59.96	CGACGCTTGCAATCTACACG	59.976	196
TRINITY_Contig9407	2	p5	(AAGAA)3	15	1260	1274	GGGTTTCTTGGATGCCTGGA	59.96	CGACGCTTGCAATCTACACG	59.976	196
TRINITY_Contig9407	3	p3	(TGA)4	12	1388	1399	GGGTTTCTTGGATGCCTGGA	59.96	CGACGCTTGCAATCTACACG	59.976	196
TRINITY_Contig9408	1	p4	(CAAC)4	16	1486	1501	CGTACGGAGGAGGAGAATGC	59.969	CTCGCTCGCTTAGGAGACTG	59.971	227
TRINITY_Contig9409	1	c	(GCT)5ggggatgcggcgggcgctgctggggcggggcgccggtggggttggggttgccgccggaggccat(GGC)5	94	1844	1937	CAGCATCCTCAACCCATGGT	60.034	CTGGGTTTCAGTCAGTCCCC	59.963	151
TRINITY_Contig9410	1	p4	(TGGT)3	12	2003	2014	AGGGGTCATGCCATCAAAGG	60.033	CACCACATTGGGGAGAGGTC	60.035	156
TRINITY_Contig9411	1	p3	(CGG)4	12	1350	1361	GCGTTGCGAAGACCAAACTT	59.971	TGCAGCCATCTGATCGATCC	59.966	273
TRINITY_Contig9411	2	p4	(ATGT)3	12	2403	2414	GCGTTGCGAAGACCAAACTT	59.971	TGCAGCCATCTGATCGATCC	59.966	273
TRINITY_Contig9412	1	p3	(AGC)5	15	1393	1407	AGGGAGCTCCTCAAGACGAT	60.033	AGGAACACCGTCATCTGCTG	60.037	269
TRINITY_Contig9415	1	p3	(CAC)6	18	391	408	AAGTGAGACTTGACGCAGGG	59.966	GGCCAACCGAACCAATCAAC	60.039	200
TRINITY_Contig9415	2	p6	(CCGCAG)3	18	854	871	AAGTGAGACTTGACGCAGGG	59.966	GGCCAACCGAACCAATCAAC	60.039	200
TRINITY_Contig9415	3	p3	(CGC)4	12	1648	1659	AAGTGAGACTTGACGCAGGG	59.966	GGCCAACCGAACCAATCAAC	60.039	200
TRINITY_Contig9416	1	p3	(GGC)4	12	695	706	GTCACCGTGTTCACTTGTGC	59.972	TGGACATGCGAAGGGAACTC	60.036	100
TRINITY_Contig9416	2	c	(GCC)4tcctcctggatgggtggggctcggagtaggcggccggcagtgccgtggcggtgcgtca(TG)6	82	1891	1972	GTCACCGTGTTCACTTGTGC	59.972	TGGACATGCGAAGGGAACTC	60.036	100
TRINITY_Contig9418	1	p3	(CGG)7	21	1885	1905	ACCATTCTTAAGGCGCGACA	60.037	CTTCTTGCCGAGGTGGTTCT	59.965	215
TRINITY_Contig9419	1	p6	(AGGTGG)3	18	147	164	GAGTTTTATGCTCAGCGCCG	59.973	TCCTTCGTGTCTGTGAGCAC	59.968	150
TRINITY_Contig9419	2	c	(GGC)5ggtggtgatgagaaaccatgtttacttccact(TGG)8cggtgaagcaggtttcctaccacttt(GTG)7	118	514	631	GAGTTTTATGCTCAGCGCCG	59.973	TCCTTCGTGTCTGTGAGCAC	59.968	150
TRINITY_Contig9420	1	p3	(CGC)4	12	299	310	AGGCCATGCTGCAAACTTTG	59.966	CGCCCCGTAGTACCTGAATC	59.968	143
TRINITY_Contig9420	2	p3	(TGG)4	12	1015	1026	AGGCCATGCTGCAAACTTTG	59.966	CGCCCCGTAGTACCTGAATC	59.968	143
TRINITY_Contig9420	3	p4	(TGGA)3	12	1375	1386	AGGCCATGCTGCAAACTTTG	59.966	CGCCCCGTAGTACCTGAATC	59.968	143
TRINITY_Contig9422	1	p4	(TGAT)3	12	2003	2014	GTACAACTCGAAGCAGGCCT	60.037	TTCATCGCCTCGATCTGCTC	59.969	123
TRINITY_Contig9423	1	p3	(TGC)5	15	294	308	TTGGACACGGAGAAGCTGTC	59.966	GCTACTCCATGGGCTCGTAC	59.968	169
TRINITY_Contig9423	2	p6	(TGCTGG)4	24	610	633	TTGGACACGGAGAAGCTGTC	59.966	GCTACTCCATGGGCTCGTAC	59.968	169
TRINITY_Contig9423	3	c	(CTG)5(CTC)4	27	737	763	TTGGACACGGAGAAGCTGTC	59.966	GCTACTCCATGGGCTCGTAC	59.968	169
TRINITY_Contig9423	4	p3	(CCG)5	15	1080	1094	TTGGACACGGAGAAGCTGTC	59.966	GCTACTCCATGGGCTCGTAC	59.968	169
TRINITY_Contig9423	5	p3	(CCG)4	12	1282	1293	TTGGACACGGAGAAGCTGTC	59.966	GCTACTCCATGGGCTCGTAC	59.968	169
TRINITY_Contig9423	6	p4	(CGCC)3	12	1465	1476	TTGGACACGGAGAAGCTGTC	59.966	GCTACTCCATGGGCTCGTAC	59.968	169
TRINITY_Contig9423	7	p4	(GCGA)3	12	1715	1726	TTGGACACGGAGAAGCTGTC	59.966	GCTACTCCATGGGCTCGTAC	59.968	169
TRINITY_Contig9424	1	p3	(CCG)8	24	117	140	CCAGCAAGTTCAACCCAAGC	59.967	GAAGTGGTTCCTGCCTCTCC	60.036	109
TRINITY_Contig9425	1	p4	(ATCC)3	12	24	35	CAACGGTGGCAGGAAACAAG	59.969	ACCGCCTCTTGAACTGTCTG	59.966	279
TRINITY_Contig9425	2	p3	(TCC)4	12	209	220	CAACGGTGGCAGGAAACAAG	59.969	ACCGCCTCTTGAACTGTCTG	59.966	279
TRINITY_Contig9426	1	p3	(CGC)4	12	1797	1808	AATCTCGATCTGCGTCGTCC	59.97	TCACCATTGGACTTCTGCCC	59.962	156
TRINITY_Contig9426	2	p4	(CAGG)4	16	1971	1986	AATCTCGATCTGCGTCGTCC	59.97	TCACCATTGGACTTCTGCCC	59.962	156
TRINITY_Contig9428	1	c	(GCG)6(GCA)6	36	82	117	GTATCCGCCGCGTAACAAAC	59.975	GAATAGGTCGTAGGCGTCGG	60.041	252
TRINITY_Contig9428	2	p6	(CGAGGA)3	18	943	960	GTATCCGCCGCGTAACAAAC	59.975	GAATAGGTCGTAGGCGTCGG	60.041	252
TRINITY_Contig9430	1	p2	(TC)7	14	64	77	GATAGCACGGTGAGCGAAGA	59.899	AATGTCACAGCGCCCATGTA	60.036	157
TRINITY_Contig9430	2	c	(CGG)4tggcggggcggccgagagcgacggggagagcaaagggcagcagcaacagcacccgcggccgcg(GCA)5	90	416	505	GATAGCACGGTGAGCGAAGA	59.899	AATGTCACAGCGCCCATGTA	60.036	157
TRINITY_Contig9430	3	p6	(GGCCGC)3	18	803	820	GATAGCACGGTGAGCGAAGA	59.899	AATGTCACAGCGCCCATGTA	60.036	157
TRINITY_Contig9430	4	p4	(CTGC)3	12	1055	1066	GATAGCACGGTGAGCGAAGA	59.899	AATGTCACAGCGCCCATGTA	60.036	157
TRINITY_Contig9430	5	p4	(GTGC)3	12	1332	1343	GATAGCACGGTGAGCGAAGA	59.899	AATGTCACAGCGCCCATGTA	60.036	157
TRINITY_Contig9430	6	p4	(TTTG)3	12	1967	1978	GATAGCACGGTGAGCGAAGA	59.899	AATGTCACAGCGCCCATGTA	60.036	157
TRINITY_Contig9432	1	p4	(TCCA)3	12	731	742	TGAGATTGGTTTGCTTGCGC	60.039	TCGGCCCAGAGTATCCTGAA	60.033	236
TRINITY_Contig9432	2	p3	(AGT)4	12	1301	1312	TGAGATTGGTTTGCTTGCGC	60.039	TCGGCCCAGAGTATCCTGAA	60.033	236
TRINITY_Contig9433	1	c	(CCG)8(CCACTG)3ccgacgccatg(GGA)4	65	55	119	TCTCGCAGTCCCAAACTTCC	59.965	CTGGACTCAAGCACAGCAGA	59.966	157
TRINITY_Contig9433	2	p3	(TGA)4	12	878	889	TCTCGCAGTCCCAAACTTCC	59.965	CTGGACTCAAGCACAGCAGA	59.966	157
TRINITY_Contig9433	3	p3	(TCA)4	12	2969	2980	TCTCGCAGTCCCAAACTTCC	59.965	CTGGACTCAAGCACAGCAGA	59.966	157
TRINITY_Contig9434	1	p3	(CCG)4	12	835	846	CCATATGCGGTATGCCTCGT	60.037	TCTCTGATCTCCAGCGTCCA	60.034	182
TRINITY_Contig9435	1	p6	(CCGCCT)3	18	261	278	TTCGACATTGGCAGCAGGAT	60.035	CGGTGGGTATGACCTGATCG	59.968	226
TRINITY_Contig9436	1	p3	(CGG)4	12	244	255	GAAGGGAGGCTCAGGAACAC	60.036	TCAACTTGATGAGCGCTGGT	59.965	113
TRINITY_Contig9438	1	p3	(CCG)4	12	1074	1085	CTAAAGAACGCGGCTGCATC	59.973	CAGTCCTCACTGCGTTCCAT	60.037	203
TRINITY_Contig9440	1	p3	(CCG)4	12	454	465	TGGATTTGGTGACCTCGGTG	59.964	ACGCAGGGCTGATTTCAGAA	59.963	217
TRINITY_Contig9440	2	c	(GT)14(GA)8	44	825	868	TGGATTTGGTGACCTCGGTG	59.964	ACGCAGGGCTGATTTCAGAA	59.963	217
TRINITY_Contig9440	3	p3	(CGC)6	18	1618	1635	TGGATTTGGTGACCTCGGTG	59.964	ACGCAGGGCTGATTTCAGAA	59.963	217
TRINITY_Contig9442	1	p3	(CCG)5	15	194	208	CATCAAGTCTCGGAGCTGCA	60.108	TGCGGAGTTCGATGACACAA	59.967	158
TRINITY_Contig9442	2	c	(CGA)4cgggcgtcttggagtgcgcctcgcagaccttgtgccgccggtggtagtcgcggcacttgctcaggtccgccgtgcacccctccaccgcgcacgacgggca(CTG)6	130	1014	1143	CATCAAGTCTCGGAGCTGCA	60.108	TGCGGAGTTCGATGACACAA	59.967	158
TRINITY_Contig9442	3	c	(CCG)4cgccgcccgcgtggtcgtgctccagctccgcgaggtcccacgcgctgcccggcgccttgagatcccagtccatgccgacgccggc(CTCAG)4	117	1422	1538	CATCAAGTCTCGGAGCTGCA	60.108	TGCGGAGTTCGATGACACAA	59.967	158
TRINITY_Contig9442	4	c	(GAG)4gaatcagaaatggaaggtgcagtgtatagata(GCG)5	59	1802	1860	CATCAAGTCTCGGAGCTGCA	60.108	TGCGGAGTTCGATGACACAA	59.967	158
TRINITY_Contig9442	5	p2	(GA)6	12	1995	2006	CATCAAGTCTCGGAGCTGCA	60.108	TGCGGAGTTCGATGACACAA	59.967	158
TRINITY_Contig9443	1	p3	(GCG)4	12	1816	1827	TTCAAGGATGAGTTGCGCCT	59.963	GTCAGATGCGCTCATTGCTG	59.972	128
TRINITY_Contig9445	1	p5	(TCATG)5	25	1287	1311	TGGGCCCCCAATGTTATTCC	60.032	CATTCAGCGGGCAGATTTCG	59.971	269
TRINITY_Contig9447	1	p3	(GCG)5	15	705	719	CGTCCAGTCTGACACGAACA	59.97	AACCTCTGCTTCTTCGCCAA	59.891	276
TRINITY_Contig9447	2	p4	(GCGT)3	12	1920	1931	CGTCCAGTCTGACACGAACA	59.97	AACCTCTGCTTCTTCGCCAA	59.891	276
TRINITY_Contig9449	1	p3	(TGC)7	21	1947	1967	GAACCTTCTTCGTCCAGCCA	59.965	CCGTTCCAATCCATTCCCCA	60.034	225
TRINITY_Contig9450	1	p3	(CCT)4	12	306	317	CCGTGTTCTTGGGCTTGTTG	59.969	CGAACATCCCAGTGTGGTCA	59.965	272
TRINITY_Contig9454	1	p3	(CGA)4	12	255	266	ATCGCGGTGTCCAACTTCTT	59.965	GGAATGCCGGCGATGTAGTA	59.967	122
TRINITY_Contig9455	1	p3	(GCC)6	18	1814	1831	AGCTGATCAACCGTCCCAAG	60.036	ACAAACTCCTCATGGCTGGG	59.961	112
TRINITY_Contig9458	1	p4	(GAGG)3	12	167	178	GACGAACATCACACAGGGGT	59.965	CTGGTGATGTGGGTGGAGAC	60.036	226
TRINITY_Contig9460	1	p3	(CGC)4	12	682	693	ATTGTTCATGGTGGCGGAGT	59.962	CAGCCTTTCACTCCCTCGTT	59.965	227
TRINITY_Contig9461	1	p3	(GCC)4	12	634	645	TCATCTACTACTGCTGCGCG	59.971	CCCGATAGCAGAACCCGATC	60.038	153
TRINITY_Contig9463	1	c	(GGC)4ggtggcg(GCA)6	37	378	414	ATTGGAGCATCGGTGGTTGT	59.962	AGCCACTTGATCGCCATTGA	60.035	159
TRINITY_Contig9464	1	p3	(GGA)4	12	366	377	TATGCTTGCCACCTCACTGG	60.035	TCAGTTCCTCGGAAGGCAAC	59.965	150
TRINITY_Contig9464	2	p3	(TGG)4	12	858	869	TATGCTTGCCACCTCACTGG	60.035	TCAGTTCCTCGGAAGGCAAC	59.965	150
TRINITY_Contig9465	1	p3	(CCA)4	12	107	118	GGAAGGTGTTCGACGAGGAG	60.109	AGGACTCGAACTGGATGGA	59.959	190
TRINITY_Contig9465	2	p3	(CCG)6	18	1656	1673	GGAAGGTGTTCGACGAGGAG	60.109	AGGACTCGAACTGGATGGA	59.959	190
TRINITY_Contig9466	1	p5	(ATCTC)3	15	76	90	GCAGCATCTCCGAGTACCTC	59.969	AGGTTCGACAGGAATGGCAG	60.036	204
TRINITY_Contig9466	2	p3	(CCG)4	12	880	891	GCAGCATCTCCGAGTACCTC	59.969	AGGTTCGACAGGAATGGCAG	60.036	204
TRINITY_Contig9466	3	p3	(CCG)6	18	1040	1057	GCAGCATCTCCGAGTACCTC	59.969	AGGTTCGACAGGAATGGCAG	60.036	204
TRINITY_Contig9467	1	p4	(GGTA)3	12	371	382	AGCTTCAGAGGCCATGCTTT	59.961	AATAAAGGGACGCCATGCCA	60.034	129
TRINITY_Contig9469	1	c	(GGA)5agagaggaggcggag(GGC)5	45	1814	1858	CAGGCTGCTTGGTCAAACAC	59.969	TGTGGCTGTGTGGACAAGTT	60.035	149
TRINITY_Contig9470	1	p3	(CCG)4	12	254	265	TGTCACCTTCGAGAACGTGG	59.968	CCGATGTCAAGGGCCTTCTT	60.035	264
TRINITY_Contig9471	1	p3	(CTC)4	12	181	192	TTGGAGGTTGGAAGCGAGAC	59.965	GACCATAGCGTCCATCCCAG	59.966	193
TRINITY_Contig9472	1	p3	(CCG)4	12	68	79	GTTTGGACTTTGGAACGGGC	59.968	GGTGATTTCGGGTTCCAGGT	59.962	274
TRINITY_Contig9472	2	p3	(GAG)4	12	694	705	GTTTGGACTTTGGAACGGGC	59.968	GGTGATTTCGGGTTCCAGGT	59.962	274
TRINITY_Contig9478	1	c	(TCC)4tggccctcgcgtacttcaactggctcccggcgggcgcggccccgca(GGC)4	71	169	239	TATGGTTGGTGGTGGTCAGC	59.962	GGTGGTCCTTCAGGCTTTCA	59.89	233
TRINITY_Contig9480	1	p4	(GCTA)5	20	1912	1931	GCTCGTAGTACCACGAGCTC	59.972	ATACCACCGCTCCAACCTTG	60.035	215
TRINITY_Contig9483	1	p3	(CGC)6	18	119	136	ATTCTCACGTGACGGAACCC	60.038	ACAGATGCATCTCCTCCCCT	60.03	258
TRINITY_Contig9484	1	p2	(TC)6	12	298	309	GAGGAGGAATGCAACCAGCT	60.035	GGGCGTGGCTACTACATCTC	59.968	250
TRINITY_Contig9485	1	p4	(AAGC)3	12	421	432	GCCACTATGATGCACAAGCG	59.971	TCTTGCGCGCAGTACTACAA	60.039	113
TRINITY_Contig9485	2	p3	(GGC)4	12	991	1002	GCCACTATGATGCACAAGCG	59.971	TCTTGCGCGCAGTACTACAA	60.039	113
TRINITY_Contig9485	3	p3	(TCG)4	12	1280	1291	GCCACTATGATGCACAAGCG	59.971	TCTTGCGCGCAGTACTACAA	60.039	113
TRINITY_Contig9486	1	p3	(GGC)5	15	422	436	GAACGATTCGTCAAGCTCGC	59.976	ATACAAAGACGCCGGTGGTT	59.964	161
TRINITY_Contig9486	2	p3	(CTC)4	12	1313	1324	GAACGATTCGTCAAGCTCGC	59.976	ATACAAAGACGCCGGTGGTT	59.964	161
TRINITY_Contig9487	1	p2	(AT)6	12	196	207	CCTGCCTACTTGAACCACGT	59.965	TGTTGGAGGACCCTGAGGAA	60.105	114
TRINITY_Contig9487	2	p3	(CGG)4	12	491	502	CCTGCCTACTTGAACCACGT	59.965	TGTTGGAGGACCCTGAGGAA	60.105	114

TRINITY_Contig9488	1	c	(TCCA)3taccgccgccattcttctcgtctctctcat(CGG)4	54	4	57	AGGAGGATGCCTTGAGAGGT	59.956	GTCTTGATGGGTTCCTGGGG	60.034	130
TRINITY_Contig9491	1	p6	(GGCGAC)5	30	157	186	TCGCGGTCCAACCTCTACTA	60.035	CTCCATGGCGAGTCTCATCC	59.967	237
TRINITY_Contig9491	2	p3	(CGG)4	12	946	957	TCGCGGTCCAACCTCTACTA	60.035	CTCCATGGCGAGTCTCATCC	59.967	237
TRINITY_Contig9493	1	p4	(CGCC)3	12	1182	1193	TGCAACGACGAACAGAGGAA	59.897	GGAGCTACACGTACGACGAG	59.974	202
TRINITY_Contig9493	2	p4	(TGCG)3	12	1512	1523	TGCAACGACGAACAGAGGAA	59.897	GGAGCTACACGTACGACGAG	59.974	202
TRINITY_Contig9495	1	p3	(CGC)5	15	384	398	GACCCGGAAATACCCAGCAA	60.035	TCACGACGAGGTGGAAGTTG	59.968	250
TRINITY_Contig9495	2	p4	(AAGC)3	12	1442	1453	GACCCGGAAATACCCAGCAA	60.035	TCACGACGAGGTGGAAGTTG	59.968	250
TRINITY_Contig9496	1	p3	(TCC)5	15	569	583	ATGATGCCACCCTGACCAAG	60.034	CAGCATTGGGTTCAACAGGC	60.038	155
TRINITY_Contig9498	1	c	(GCC)4ggacctctgcggcaaggcgtctcctt gcgggtcggtggcggggaccgcga(GGC)4	74	273	346	TCAAGAACCACTGCGTCCTC	59.966	TACTCCAGTTGGTGCAGCTG	59.965	154
TRINITY_Contig9498	2	p3	(CGC)4	12	461	472	TCAAGAACCACTGCGTCCTC	59.966	TACTCCAGTTGGTGCAGCTG	59.965	154
TRINITY_Contig9498	3	p3	(CCT)4	12	674	685	TCAAGAACCACTGCGTCCTC	59.966	TACTCCAGTTGGTGCAGCTG	59.965	154
TRINITY_Contig9499	1	p5	(GAATG)3	15	236	250	ACCTAGAGACGGGGGACTTC	60.033	TGCATCGACGAACACCTTCA	59.967	243
TRINITY_Contig9499	2	p3	(GCG)9	27	451	477	ACCTAGAGACGGGGGACTTC	60.033	TGCATCGACGAACACCTTCA	59.967	243
TRINITY_Contig9501	1	p5	(TCTTT)3	15	1741	1755	GGACGGCTGATTTCATTGGC	59.899	CCCTGTGGATCAGTTGGCAT	60.034	181
TRINITY_Contig9502	1	p3	(CGC)4	12	1762	1773	CCTGAGCGGGGGTTTTACAT	60.034	GCGTAGAGATCTGCAGGTCC	59.969	180
TRINITY_Contig9503	1	p5	(AGAAG)3	15	178	192	CCAGGATCCACCATTGCCTT	60.033	ATGAGCTGGGTCAGAGGGAT	60.03	212
TRINITY_Contig9503	2	p3	(CCG)5	15	1093	1107	CCAGGATCCACCATTGCCTT	60.033	ATGAGCTGGGTCAGAGGGAT	60.03	212
TRINITY_Contig9503	3	p6	(GCGGAC)3	18	1479	1496	CCAGGATCCACCATTGCCTT	60.033	ATGAGCTGGGTCAGAGGGAT	60.03	212
TRINITY_Contig9504	1	p3	(GGC)5	15	429	443	CCCAGCTCTCGATGATGACC	59.967	AGTTTCGAACGGAAGGGGTC	59.966	109
TRINITY_Contig9505	1	p4	(TTTC)5	20	1758	1777	AAGACCACAAGCAACCCCAA	60.033	TGTACAAGCTGCTCCCATGG	60.035	114
TRINITY_Contig9507	1	p2	(CG)6	12	127	138	CTGTCCTGGGATCTGCGTTT	60.036	AGCACCGATGTACCAGCAAA	59.964	106
TRINITY_Contig9509	1	p3	(CGC)5	15	104	118	ATTCCTTGTCTCCTGTGGCG	60.036	TCTGGCCCTGATATCCCACA	60.03	182
TRINITY_Contig9509	2	p3	(CCG)4	12	673	684	ATTCCTTGTCTCCTGTGGCG	60.036	TCTGGCCCTGATATCCCACA	60.03	182
TRINITY_Contig9510	1	p4	(CTAG)3	12	459	470	CTCGATCGAGTACAGTGGCC	59.97	ACGACGCAAAGAATTCCCCT	59.964	159
TRINITY_Contig9511	1	p3	(GAG)5	15	225	239	GGCCGAAAGTTCTGTCTGGA	59.965	CATTGAGGGGGCACTGACAT	60.034	224
TRINITY_Contig9512	1	c	(CGGCGC)3gggggcgtagtaggacgcgcttatgctcggccgcgcctggttgtagctcatcaccaca(TGC)5	91	712	802	AGATAGCTCGTGCATCTGCC	59.967	ATTAGCACTGCTCGGGCTTT	60.035	204
TRINITY_Contig9512	2	p3	(GCG)4	12	933	944	AGATAGCTCGTGCATCTGCC	59.967	ATTAGCACTGCTCGGGCTTT	60.035	204
TRINITY_Contig9514	1	p4	(AATT)3	12	1117	1128	ACCATGGCGTGATCCAACAT	60.034	ATTGTTGCGTCCAGTTTGGC	59.968	140
TRINITY_Contig9515	1	p4	(GACT)3	12	1870	1881	CGGTGAGGTCGATGTCGTAG	59.972	CAAGATGCCGGTGTTCCTCT	60.036	122
TRINITY_Contig9516	1	c	(GGC)5agcgtctggatccgcttccgagtccggcggcggctccccggcggctccggccgg(GGC)4	81	160	240	CGGATGATGCTACTCGTGCT	59.969	TGCAACAGGACGCATTTGTG	59.969	140
TRINITY_Contig9517	1	p4	(TTTC)3	12	1858	1869	ATCCGTGTCGACCAAACCTC	60.038	ACCTGTTCATGGAGAGCTGC	60.036	260
TRINITY_Contig9518	1	c	(TGCA)3ttcatgtggctccgaccccgaggaagtcaatgaacgccggtgacggcgccgcc(GCG)4	77	454	530	GAACGGGAAGCTGCAGTAGT	60.037	AACCACTGGCACGTCATCAT	59.963	258
TRINITY_Contig9518	2	c	(GCC)6gctggcggcctccaacgacggccaggggaggcgcctgtcggcgtgtgccgccgcgctgccgggcccggggaacaggcagaaggga(GGC)5	118	698	815	GAACGGGAAGCTGCAGTAGT	60.037	AACCACTGGCACGTCATCAT	59.963	258
TRINITY_Contig9518	3	p3	(TGG)4	12	945	956	GAACGGGAAGCTGCAGTAGT	60.037	AACCACTGGCACGTCATCAT	59.963	258
TRINITY_Contig9518	4	p3	(CTC)4	12	1190	1201	GAACGGGAAGCTGCAGTAGT	60.037	AACCACTGGCACGTCATCAT	59.963	258
TRINITY_Contig9518	5	p3	(GCT)4	12	1403	1414	GAACGGGAAGCTGCAGTAGT	60.037	AACCACTGGCACGTCATCAT	59.963	258
TRINITY_Contig9518	6	p4	(CGGT)3	12	1619	1630	GAACGGGAAGCTGCAGTAGT	60.037	AACCACTGGCACGTCATCAT	59.963	258
TRINITY_Contig9519	1	p3	(CGG)4	12	971	982	GACTCACTATCCTCGCCGTG	59.97	GTCAGTCTTTGCCGTCTCCA	59.966	180
TRINITY_Contig9519	2	p3	(CGC)5	15	1493	1507	GACTCACTATCCTCGCCGTG	59.97	GTCAGTCTTTGCCGTCTCCA	59.966	180
TRINITY_Contig9519	3	p3	(CGG)4	12	4926	4937	GACTCACTATCCTCGCCGTG	59.97	GTCAGTCTTTGCCGTCTCCA	59.966	180
TRINITY_Contig9520	1	p4	(ATAC)4	16	244	259	TGCTGTTGCCCTCAGAAGAG	59.964	GAGATCCAGCAGACATCGGG	59.967	187
TRINITY_Contig9520	2	p2	(TA)18	36	439	474	TGCTGTTGCCCTCAGAAGAG	59.964	GAGATCCAGCAGACATCGGG	59.967	187
TRINITY_Contig9520	3	p3	(CAC)4	12	1188	1199	TGCTGTTGCCCTCAGAAGAG	59.964	GAGATCCAGCAGACATCGGG	59.967	187
TRINITY_Contig9520	4	p3	(TCC)4	12	1751	1762	TGCTGTTGCCCTCAGAAGAG	59.964	GAGATCCAGCAGACATCGGG	59.967	187
TRINITY_Contig9522	1	p5	(GTTTT)3	15	93	107	GCCGAAGAGGAACTTGCTCT	60.037	CGAGAGGGAGCTATCGGGTA	59.964	130
TRINITY_Contig9522	2	p3	(GGC)4	12	790	801	GCCGAAGAGGAACTTGCTCT	60.037	CGAGAGGGAGCTATCGGGTA	59.964	130
TRINITY_Contig9524	1	p5	(GTGGT)3	15	693	707	CGTCAACATCAGCATGCTCG	59.974	GCTATCTCCGCCAGGTACAC	59.968	205
TRINITY_Contig9525	1	p3	(GCG)5	15	490	504	AAGCTCGCTCTCCACTTCAC	60.038	GGAATGAACTGGTGAGGGGG	60.034	182
TRINITY_Contig9525	2	p3	(GCC)4	12	678	689	AAGCTCGCTCTCCACTTCAC	60.038	GGAATGAACTGGTGAGGGGG	60.034	182
TRINITY_Contig9525	3	p3	(CTC)4	12	1179	1190	AAGCTCGCTCTCCACTTCAC	60.038	GGAATGAACTGGTGAGGGGG	60.034	182
TRINITY_Contig9525	4	p3	(GCT)4	12	1392	1403	AAGCTCGCTCTCCACTTCAC	60.038	GGAATGAACTGGTGAGGGGG	60.034	182
TRINITY_Contig9525	5	p4	(CGGT)3	12	1608	1619	AAGCTCGCTCTCCACTTCAC	60.038	GGAATGAACTGGTGAGGGGG	60.034	182
TRINITY_Contig9526	1	p3	(CGG)4	12	363	374	ACTGAGCACACAGAACTCCG	59.967	AAGCAAAGACGAAGCCGAGA	59.966	111
TRINITY_Contig9528	1	p4	(TTGA)3	12	173	184	GGTCGTACTAAACCCCGCAA	60.037	GATGGGAATGGCGGATTTGC	59.967	251
TRINITY_Contig9528	2	p4	(AATA)3	12	476	487	GGTCGTACTAAACCCCGCAA	60.037	GATGGGAATGGCGGATTTGC	59.967	251
TRINITY_Contig9528	3	p4	(GCCG)3	12	649	660	GGTCGTACTAAACCCCGCAA	60.037	GATGGGAATGGCGGATTTGC	59.967	251
TRINITY_Contig9533	1	p3	(CCT)5	15	1677	1691	AGGCACCTAAATGCTGCCAT	60.033	ATCGTTGCGCCATTTCATCG	59.972	164
TRINITY_Contig9534	1	c	(GGC)4gagcgggccatcgag(GGATC)3cggggcagcgtgctagggttggtttagatctgatttga(TTGCT)3	96	1823	1918	CGTCAAAGGGCACAGATCCT	60.036	GTCACGGAGAACACCAGGAG	60.038	169
TRINITY_Contig9535	1	p3	(CGG)4	12	291	302	GCAGCGGAAATGCGAACTAG	59.973	TCTGAAGGTTCCCCGCAATC	60.035	101
TRINITY_Contig9535	2	p3	(GCC)5	15	1445	1459	GCAGCGGAAATGCGAACTAG	59.973	TCTGAAGGTTCCCCGCAATC	60.035	101
TRINITY_Contig9535	3	p4	(CTTT)3	12	2927	2938	GCAGCGGAAATGCGAACTAG	59.973	TCTGAAGGTTCCCCGCAATC	60.035	101
TRINITY_Contig9536	1	p3	(AGG)4	12	514	525	GACTTGGACGAGGACACCAG	60.038	CGGGTTCGAGAATGGAGGAG	59.897	187
TRINITY_Contig9536	2	p3	(ACG)4	12	775	786	GACTTGGACGAGGACACCAG	60.038	CGGGTTCGAGAATGGAGGAG	59.897	187
TRINITY_Contig9536	3	p3	(CGG)5	15	1217	1231	GACTTGGACGAGGACACCAG	60.038	CGGGTTCGAGAATGGAGGAG	59.897	187
TRINITY_Contig9536	4	p3	(CCG)5	15	1381	1395	GACTTGGACGAGGACACCAG	60.038	CGGGTTCGAGAATGGAGGAG	59.897	187
TRINITY_Contig9536	5	p3	(CTC)5	15	1600	1614	GACTTGGACGAGGACACCAG	60.038	CGGGTTCGAGAATGGAGGAG	59.897	187
TRINITY_Contig9536	6	p6	(GCGCCT)3	18	1790	1807	GACTTGGACGAGGACACCAG	60.038	CGGGTTCGAGAATGGAGGAG	59.897	187
TRINITY_Contig9537	1	p3	(GGC)6	18	404	421	CGATAGGCGCCGAGGATATC	59.97	GTAAAGGTGCTGCTCCCAGT	59.963	216
TRINITY_Contig9537	2	c	(CCAT)3gatccgttcgcctcctttcactaaaaaaaaatcaattcgcttccttcccctacccctccgcctca(GCC)4	90	618	707	CGATAGGCGCCGAGGATATC	59.97	GTAAAGGTGCTGCTCCCAGT	59.963	216
TRINITY_Contig9539	1	c	(GGC)4tgcagcagcggcgccgagagcgtctggttgtggttcgggtgacccgccggggg(CGGGGA)3(CGG)4gggaggcgggcgaggcggaggggccgggggaggcgacgtcgacggcggaggcgtcccggcgaggccggcgccgggagaggccatgggcgcgggcg(GGAA)3	202	1688	1889	AAACAGCTATGTCACCGCCA	59.964	CCGCATGTTCCTATTGCTGC	59.97	119
TRINITY_Contig9542	1	p3	(TCA)4	12	577	588	CCCTCAACAAACTGCATGGC	60.038	GCATCGAAATGGTGGTACGC	59.972	204
TRINITY_Contig9542	2	p5	(GGAGC)3	15	4069	4083	CCCTCAACAAACTGCATGGC	60.038	GCATCGAAATGGTGGTACGC	59.972	204
TRINITY_Contig9544	1	c	(AGCT)4ttggtggaatcggaatcggacggagcgcgaggaagaatggcgatgcagagagccgccgcggccgc(CCT)4ccccctcctc(GCG)4	115	25	139	CAACACGGTGCAGCATGAAA	59.969	CCCCTGCCACAGAGACAAAT	59.961	278
TRINITY_Contig9544	2	p4	(GGTT)3	12	1824	1835	CAACACGGTGCAGCATGAAA	59.969	CCCCTGCCACAGAGACAAAT	59.961	278
TRINITY_Contig9546	1	p4	(GATC)3	12	1628	1639	CGTGCTCTTTAATTCGCCGG	59.974	GCCACACTTGCTGTCTTGTG	59.97	186
TRINITY_Contig9547	1	p2	(TC)6	12	282	293	TAGAATCCCCAGCTCTGCCT	60.031	CACGTACAACCCTCCGTTCA	59.968	144
TRINITY_Contig9547	2	p3	(AGG)4	12	3062	3073	TAGAATCCCCAGCTCTGCCT	60.031	CACGTACAACCCTCCGTTCA	59.968	144
TRINITY_Contig9548	1	p3	(CGC)4	12	755	766	GACGAGGATGAAGCACGTCT	59.829	TGTACAGTCCGCTGGTGTTC	59.967	104
TRINITY_Contig9548	2	c	(CGGCG)3ccacctgctgccggcgaggggcggaggagcggcgcctccgtggcatcgg(CGC)4	76	1642	1717	GACGAGGATGAAGCACGTCT	59.829	TGTACAGTCCGCTGGTGTTC	59.967	104
TRINITY_Contig9549	1	p3	(CAC)4	12	195	206	GGCGCCAACAATCATACGAC	59.972	GCTACGATCTCGAGCACCTC	60.041	218
TRINITY_Contig9550	1	c	(GCG)4gagtcggaccccgccgatgccgaggcggcgaaggagcggcggaggagcgcgccgccgatccggcggaggagggcgcg(GGA)4	101	1664	1764	ACACAGTACTCTGCAAGCCC	59.965	GTCGGTGCGTTCATAATGCC	59.972	188
TRINITY_Contig9550	2	p3	(GCC)4	12	1871	1882	ACACAGTACTCTGCAAGCCC	59.965	GTCGGTGCGTTCATAATGCC	59.972	188
TRINITY_Contig9551	1	p3	(GCT)4	12	1018	1029	ATAGATGCCAGTGGTAGCGC	59.966	TGGGTTTTCCGGTGTTTGGA	60.033	119
TRINITY_Contig9551	2	p3	(CCG)4	12	1620	1631	ATAGATGCCAGTGGTAGCGC	59.966	TGGGTTTTCCGGTGTTTGGA	60.033	119
TRINITY_Contig9552	1	p3	(ACG)4	12	166	177	GGCAAATTACGACGACGACG	59.977	TTTGAGAAGGATCCCACCGC	60.035	258

TRINITY_Contig9554	1	p3	(AAG)4	12	1798	1809	AAGCGCCTGAAGAACATCGA	60.037	CGACACACTCACGACACTCA	59.971	133
TRINITY_Contig9555	1	c	(GGC)4ggaggcgggtggtggacctggtggtttaatagagggaggggtcaggaggaagggtcccaaagggtggatgggatctgatgggaagt(TGG)4	110	2387	2496	CCAGTGAGAAACCGCAGTCT	59.966	ATCTTGCACTGTTGAGCCGA	59.965	176
TRINITY_Contig9557	1	p3	(AGC)4	12	1593	1604	CGAGGAGAACGAGCTGATCC	59.97	AATCTCTTGGAGGGTGCTGC	60.035	241
TRINITY_Contig9558	1	p2	(TA)7	14	257	270	CAGCTGATGAATTGACGGCG	59.973	TCATACAACGGGTCACTGGC	60.037	152
TRINITY_Contig9558	2	p3	(CGC)4	12	683	694	CAGCTGATGAATTGACGGCG	59.973	TCATACAACGGGTCACTGGC	60.037	152
TRINITY_Contig9558	3	p3	(TCG)4	12	820	831	CAGCTGATGAATTGACGGCG	59.973	TCATACAACGGGTCACTGGC	60.037	152
TRINITY_Contig9558	4	p3	(CCT)5	15	2902	2916	CAGCTGATGAATTGACGGCG	59.973	TCATACAACGGGTCACTGGC	60.037	152
TRINITY_Contig9558	5	p2	(AG)26	52	3079	3130	CAGCTGATGAATTGACGGCG	59.973	TCATACAACGGGTCACTGGC	60.037	152
TRINITY_Contig9558	6	p4	(AGGA)3	12	3253	3264	CAGCTGATGAATTGACGGCG	59.973	TCATACAACGGGTCACTGGC	60.037	152
TRINITY_Contig9559	1	p3	(CCG)4	12	1669	1680	CGAAGATGACGAGGAGGAGC	59.97	TCCCTCGAGTTCTACAGCGA	60.036	276
TRINITY_Contig9559	2	p3	(CGC)4	12	1888	1899	CGAAGATGACGAGGAGGAGC	59.97	TCCCTCGAGTTCTACAGCGA	60.036	276
TRINITY_Contig9560	1	p4	(CTTG)3	12	98	109	AGAGGAAGCTCTCTCCCAGG	60.032	GCGGTTGTGGTACTCTCCAA	59.966	259
TRINITY_Contig9561	1	p3	(GGC)4	12	989	1000	CAGTTCAGCTACGACCTGCA	60.038	AATCATCTGCAGCACGGTGA	60.036	216
TRINITY_Contig9562	1	p3	(CGC)4	12	458	469	CGAGGACCACGAGGAACAAA	59.967	ATCCACGACTCACGATGTCG	59.901	215
TRINITY_Contig9563	1	p6	(TGGAGC)3	18	201	218	TTTTGCCGACTCCGAATGGA	59.964	GGATTACATGCGTTCCGTGC	59.972	129
TRINITY_Contig9563	2	p3	(GCG)6	18	608	625	TTTTGCCGACTCCGAATGGA	59.964	GGATTACATGCGTTCCGTGC	59.972	129
TRINITY_Contig9565	1	c	(CGC)5cgtggaagttgagggctttcttgcgccacgcggcctcctt(GCC)4	67	1454	1520	TCGGCATCAAGATACGCTCC	59.968	TTGTCCCACTGATGCAGACC	59.963	213
TRINITY_Contig9566	1	c*	(AGCC)3(<AGCC>T)(AGCCT)2gcctt(CCAAG)3	47	46	92	AGAGGCTTCCTATGACCGGT	60.032	TACCAGCCTTCTGCACAGTG	59.965	180
TRINITY_Contig9568	1	p3	(GCT)5	15	1051	1065	TGGAGTTTCCATCAGGTGCC	59.962	AACAGCAGCAGCAGCATTTC	60.039	245
TRINITY_Contig9568	2	p3	(TGC)4	12	1326	1337	TGGAGTTTCCATCAGGTGCC	59.962	AACAGCAGCAGCAGCATTTC	60.039	245
TRINITY_Contig9571	1	p3	(TCA)4	12	111	122	CTACCGCGGCATAAACAACG	59.974	TATTTCCGGTATGCGGAGGC	59.966	197
TRINITY_Contig9571	2	p4	(GCCT)3	12	544	555	CTACCGCGGCATAAACAACG	59.974	TATTTCCGGTATGCGGAGGC	59.966	197
TRINITY_Contig9571	3	p3	(GGC)4	12	888	899	CTACCGCGGCATAAACAACG	59.974	TATTTCCGGTATGCGGAGGC	59.966	197
TRINITY_Contig9571	4	p3	(CGC)5	15	1353	1367	CTACCGCGGCATAAACAACG	59.974	TATTTCCGGTATGCGGAGGC	59.966	197
TRINITY_Contig9571	5	p4	(AATC)4	16	2395	2410	CTACCGCGGCATAAACAACG	59.974	TATTTCCGGTATGCGGAGGC	59.966	197
TRINITY_Contig9573	1	p4	(TGCA)3	12	231	242	GGAACAACTTACGGGAGCGA	60.038	GGGGCGAGTTTGTGTACGTA	60.038	233
TRINITY_Contig9573	2	p3	(CGC)4	12	906	917	GGAACAACTTACGGGAGCGA	60.038	GGGGCGAGTTTGTGTACGTA	60.038	233
TRINITY_Contig9573	3	p3	(CGC)4	12	1292	1303	GGAACAACTTACGGGAGCGA	60.038	GGGGCGAGTTTGTGTACGTA	60.038	233
TRINITY_Contig9573	4	c	(AGG)4gaggttgaggaggcgccgcca(CCG)6	51	1553	1603	GGAACAACTTACGGGAGCGA	60.038	GGGGCGAGTTTGTGTACGTA	60.038	233
TRINITY_Contig9575	1	p4	(TTCT)3	12	1363	1374	ACCAGCCACCATCACATCAG	60.035	CCAAACCATGCTGCTGTGAC	60.039	102
TRINITY_Contig9575	2	p3	(GAG)4	12	1740	1751	ACCAGCCACCATCACATCAG	60.035	CCAAACCATGCTGCTGTGAC	60.039	102
TRINITY_Contig9576	1	c	(CGG)4caagagcagcacagtgaagcagctcaacgtggggggcaagctcttctcgctggaggcaag(CTCCCT)3	90	92	181	TAACTCTGAAGCCCACGCAG	60.037	CCACATTCCCAGACCGAACA	59.964	180
TRINITY_Contig9577	1	p4	(TTCT)3	12	1789	1800	TCCCAGTGTGTCCCCCTTTA	60.104	GCACATCGCTTGTCCATTCC	59.901	234
TRINITY_Contig9578	1	p3	(CAG)4	12	935	946	GGGAAGCCGAAAGTGACTGA	59.965	CCAGGCCCTTGAAGTCGATT	60.035	271
TRINITY_Contig9581	1	p3	(GGA)6	18	1678	1695	TGATCACCGAGGCAAGTGAC	60.037	TCAGCTACCCTGAGATGGCT	60.032	108
TRINITY_Contig9581	2	p5	(TCGCC)4	20	1841	1860	TGATCACCGAGGCAAGTGAC	60.037	TCAGCTACCCTGAGATGGCT	60.032	108
TRINITY_Contig9582	1	p6	(GGCGGA)3	18	1706	1723	CTTGGGTGCCTCTCCTGAAG	60.036	CATTCTCATTTGGCTCGGCG	59.971	155
TRINITY_Contig9582	2	p3	(GCG)6	18	1833	1850	CTTGGGTGCCTCTCCTGAAG	60.036	CATTCTCATTTGGCTCGGCG	59.971	155
TRINITY_Contig9583	1	p3	(GCG)4	12	587	598	ACCGCAGCAAACCATGTTTC	59.968	TACGGAGCGGCTGATTCATC	59.968	151
TRINITY_Contig9584	1	p3	(TCC)4	12	997	1008	GGAGCTCGCTTACCATCCTC	59.967	GAACTTCATGGTGGCGGAGA	60.036	227
TRINITY_Contig9585	1	p3	(GCC)4	12	710	721	GGCTGAAGTCCTGGATTGCT	60.035	GGTCGCCAAGTTCCAGAAGA	59.965	100
TRINITY_Contig9586	1	p3	(CGG)4	12	1777	1788	TCACTCCCCATCCGAAAAGC	60.035	AGCTGGGTGCCAATGCTATT	60.033	247
TRINITY_Contig9589	1	c	(CGC)4ccccgcggccgtcgacgatgcca(GTGCCG)4	59	366	424	TATTCAGGGGGCGCAATGTT	60.034	TGGCACAAAAACACCCCTCT	60.033	217
TRINITY_Contig9591	1	p4	(GCCG)3	12	404	415	TTCCAGCCAGACAACTCGTC	59.966	CAGGAGGACCATGTTGCCAT	60.034	159
TRINITY_Contig9591	2	p3	(GCG)5	15	1527	1541	TTCCAGCCAGACAACTCGTC	59.966	CAGGAGGACCATGTTGCCAT	60.034	159
TRINITY_Contig9592	1	p3	(CGG)4	12	260	271	CGTCTCCCGGTATGAACTCG	59.971	GACCTCCTGCGGCAGTATAC	59.968	178
TRINITY_Contig9592	2	p3	(GCG)4	12	606	617	CGTCTCCCGGTATGAACTCG	59.971	GACCTCCTGCGGCAGTATAC	59.968	178
TRINITY_Contig9592	3	p6	(ATGCAG)3	18	1033	1050	CGTCTCCCGGTATGAACTCG	59.971	GACCTCCTGCGGCAGTATAC	59.968	178
TRINITY_Contig9593	1	p3	(CCG)4	12	1587	1598	AAAAACCCGACTCAAAGCGC	59.97	GGGGCAGGCTGCTTTAGTTA	60.034	253
TRINITY_Contig9594	1	c	(CCA)4cccccgcctccgcccccacctagatccccctcccctgcctgccccccatcgttaaaattc(CCT)5cccccaatcgcgtcgcggcccgcggcgtagattcctcctcccacgctcggctccctccgcccatt(CCCTC)3	167	78	244	TGGTCACGATTCGTCAACGT	59.969	CAAGCGCATCACATCCATGG	59.97	268
TRINITY_Contig9595	1	p3	(GCC)4	12	1656	1667	CCAGCTCCATTTCTCAGCCA	60.035	ATGCTTCCCTCCCGTTTCAG	60.035	258
TRINITY_Contig9595	2	p4	(CGTG)4	16	1825	1840	CCAGCTCCATTTCTCAGCCA	60.035	ATGCTTCCCTCCCGTTTCAG	60.035	258
TRINITY_Contig9597	1	p3	(CAC)6	18	58	75	GAGGCGATGATCCGTGGATT	59.966	TGACTTTGGGCCAAACCAGT	60.033	159
TRINITY_Contig9598	1	p3	(CCG)4	12	14	25	GAGACCCGTCCTTCAACCTG	60.037	GAGGACGAGAGGAGGAGGTT	60.034	106
TRINITY_Contig9598	2	p6	(GCCTCC)4	24	188	211	GAGACCCGTCCTTCAACCTG	60.037	GAGGACGAGAGGAGGAGGTT	60.034	106
TRINITY_Contig9599	1	p3	(GAA)6	18	118	135	CAACGACTGTACGGGTCCAA	59.968	ACTGCTGGAACTATTGCCCC	60.034	157
TRINITY_Contig9599	2	p3	(CGA)4	12	1814	1825	CAACGACTGTACGGGTCCAA	59.968	ACTGCTGGAACTATTGCCCC	60.034	157
TRINITY_Contig9600	1	p3	(GCC)5	15	84	98	GATCATGGTGAGGTTGGCCA	60.034	GATGACCATCTGGCACCACA	60.035	126
TRINITY_Contig9600	2	p3	(CGC)4	12	422	433	GATCATGGTGAGGTTGGCCA	60.034	GATGACCATCTGGCACCACA	60.035	126
TRINITY_Contig9603	1	p4	(CCGC)4	16	1820	1835	TCCTGCTAAGATTCCCCGGA	60.032	GGGACCACCAGACTGCATAC	60.108	202
TRINITY_Contig9604	1	p5	(GGTTT)3	15	1686	1700	AACGGCTTTGCAAACTCGTC	59.971	CAAACCCTAGCTTCCCCGTT	59.962	110
TRINITY_Contig9605	1	c	(CAG)6gggcaggaccagcaccagaagcagaggcaggccggcgccgacgacc(ACG)4	76	1016	1091	CTGTGATTGCGTACATGCGG	59.973	AGTACAGGACACAAGGTGCG	59.966	222
TRINITY_Contig9605	2	p3	(CCT)4	12	1227	1238	CTGTGATTGCGTACATGCGG	59.973	AGTACAGGACACAAGGTGCG	59.966	222
TRINITY_Contig9605	3	p4	(TCGA)3	12	1647	1658	CTGTGATTGCGTACATGCGG	59.973	AGTACAGGACACAAGGTGCG	59.966	222
TRINITY_Contig9606	1	p3	(TCT)6	18	1517	1534	TCTTCGTCATAGCCATCGCC	59.968	GGGTTCGTCCAGCACAAGTA	59.966	144
TRINITY_Contig9606	2	c	(GGC)4gacgggcgatcgcggcgggcggtggaagcccgggtggtggg(CGAT)4ggaacgaagg(GGGAA)3	94	1717	1810	TCTTCGTCATAGCCATCGCC	59.968	GGGTTCGTCCAGCACAAGTA	59.966	144
TRINITY_Contig9609	1	p3	(ACC)4	12	1470	1481	GAAAACCAGGGCACAAGCAG	59.967	GGGTGCTTACCAGGCAAGAT	60.034	126
TRINITY_Contig9611	1	p4	(CGGG)3	12	1757	1768	TCATCACAGCCCGAAAGACC	60.036	AGGGATGGAGCTGAAAAGGC	60.034	181
TRINITY_Contig9612	1	p3	(GGC)4	12	478	489	GTGGGATTCCTGAAGCACCA	59.962	TGTTTTGCTGGACACAGGGT	60.034	257
TRINITY_Contig9612	2	p3	(GAT)4	12	1289	1300	GTGGGATTCCTGAAGCACCA	59.962	TGTTTTGCTGGACACAGGGT	60.034	257
TRINITY_Contig9613	1	p3	(GCC)5	15	729	743	TAGCCAGCCAGCTCCTAGAA	60.032	CCACACCCATTCTGCCTGAT	60.034	115
TRINITY_Contig9613	2	p2	(AG)6	12	1553	1564	TAGCCAGCCAGCTCCTAGAA	60.032	CCACACCCATTCTGCCTGAT	60.034	115
TRINITY_Contig9613	3	p4	(AATT)3	12	1679	1690	TAGCCAGCCAGCTCCTAGAA	60.032	CCACACCCATTCTGCCTGAT	60.034	115
TRINITY_Contig9616	1	p3	(CTG)6	18	551	568	AAGGATGGCCACCTTGTAGC	60.034	TGCTCTCCTGTCAGATCCGA	60.034	249
TRINITY_Contig9619	1	p3	(CCT)4	12	179	190	GAAGCTGATAGGGACGGAGC	59.967	ATGGCCTTGATTTCGTCGGT	60.035	267
TRINITY_Contig9619	2	p5	(TGTTG)6	30	1744	1773	GAAGCTGATAGGGACGGAGC	59.967	ATGGCCTTGATTTCGTCGGT	60.035	267
TRINITY_Contig9620	1	p3	(CCT)4	12	41	52	GCAAAGATCCAGGGTCACGA	60.036	GCTGATGTGGGACTGTTGGA	59.963	128
TRINITY_Contig9621	1	p4	(CCTC)3	12	206	217	AGACATGGAAATGCTGCGGA	60.035	CTTGGCACTTGCTCCACAAC	59.969	248
TRINITY_Contig9621	2	p3	(GCG)4	12	896	907	AGACATGGAAATGCTGCGGA	60.035	CTTGGCACTTGCTCCACAAC	59.969	248
TRINITY_Contig9622	1	p3	(GCA)4	12	388	399	CCTGCCTCCCCAATGTCATT	60.033	TGGACCAGCTGCTGAATCAG	60.036	233
TRINITY_Contig9623	1	c	(CCG)4gaccgcggccggaggctgctgctggccggcttcttcaccggagaaggcgcgggcgacggcaag(GCC)4	88	1432	1519	CGCCTGATTTGATCTTGCGG	59.971	CATCAGCTCCCCGAGATTCC	59.965	158
TRINITY_Contig9626	1	p2	(TC)6	12	18	29	TCAACCGAAACAGCTACCCC	59.964	CATCAGGTCCAGCGAGATCG	60.319	231
TRINITY_Contig9626	2	p3	(AGC)5	15	263	277	TCAACCGAAACAGCTACCCC	59.964	CATCAGGTCCAGCGAGATCG	60.319	231
TRINITY_Contig9626	3	p4	(CCGC)3	12	431	442	TCAACCGAAACAGCTACCCC	59.964	CATCAGGTCCAGCGAGATCG	60.319	231
TRINITY_Contig9626	4	p6	(GGCGCG)3	18	766	783	TCAACCGAAACAGCTACCCC	59.964	CATCAGGTCCAGCGAGATCG	60.319	231
TRINITY_Contig9626	5	p3	(GCT)5	15	1392	1406	TCAACCGAAACAGCTACCCC	59.964	CATCAGGTCCAGCGAGATCG	60.319	231
TRINITY_Contig9627	1	p2	(TC)6	12	18	29	TGTGCCGATCAACAAGACGA	59.967	CGCTGACGCCAATGGTATTG	59.972	245
TRINITY_Contig9627	2	p4	(ACGG)3	12	1539	1550	TGTGCCGATCAACAAGACGA	59.967	CGCTGACGCCAATGGTATTG	59.972	245
TRINITY_Contig9627	3	p3	(CGC)4	12	1726	1737	TGTGCCGATCAACAAGACGA	59.967	CGCTGACGCCAATGGTATTG	59.972	245
TRINITY_Contig9628	1	p6	(CGCCCG)4	24	1394	1417	TGTTGCAACTTACAGCACGC	59.971	GGAGAGCTTGGCTGCCTTTA	60.035	135
TRINITY_Contig9629	1	p3	(CGC)5	15	584	598	CCAAGGAGCTCATGAACGGT	60.036	CCCCACGAGGACCACTAAAC	60.037	224
TRINITY_Contig9630	1	p3	(ATT)4	12	231	242	TATTCAAACGGGTGCTGGCT	59.962	CCCTATATGTGGCGTCGACC	60.039	196
TRINITY_Contig9630	2	p4	(CAAG)3	12	489	500	TATTCAAACGGGTGCTGGCT	59.962	CCCTATATGTGGCGTCGACC	60.039	196
TRINITY_Contig9631	1	p5	(CCTCG)5	25	1434	1458	AGCAGCATCAAGGAAGTGCT	59.962	GCTGTGACTGGGATGAAGCT	60.036	197
TRINITY_Contig9631	2	p6	(GGCCGA)4	24	1787	1810	AGCAGCATCAAGGAAGTGCT	59.962	GCTGTGACTGGGATGAAGCT	60.036	197
TRINITY_Contig9632	1	p6	(AGCGAG)4	24	228	251	CTTCTCTCGATACCACGGGC	59.969	GGGAGCTCTGATTGGATCGG	59.965	190
TRINITY_Contig9635	1	p3	(CCA)5	15	177	191	TTGCTTGGCACCTTCGATCT	59.963	AGTTCCTTGACACGCTCTGG	59.966	179

TRINITY_Contig9636	1	c	(AGAC)3acatagatgaattatgttcagtaat aatgtcagtagtcgtatactactagttagttatt ctcgatctgttaat(TAC)4	97	725	821	CACGCGTTCTTTCGTTTGGT	59.973	TGAGGCTGTAGGGGCTAACT	59.957	118
TRINITY_Contig9636	2	p4	(CTGC)3	12	1343	1354	CACGCGTTCTTTCGTTTGGT	59.973	TGAGGCTGTAGGGGCTAACT	59.957	118
TRINITY_Contig9636	3	c	(AAAG)3aaaaggcaataaaaaaagaaaat gcagatgaaa(AAAG)3gcaataaaaaaga gaatatgcagatgaaa(AAAG)3	98	1528	1625	CACGCGTTCTTTCGTTTGGT	59.973	TGAGGCTGTAGGGGCTAACT	59.957	118
TRINITY_Contig9638	1	p3	(CGG)4	12	963	974	ACTTCTGGAGCTTCAGCCAC	59.964	GATGTTCGTGTTCGGCATCG	59.975	140
TRINITY_Contig9640	1	p3	(CCG)4	12	454	465	TGGATTTGGTGACCTCGGTG	59.964	GCAGCTCACCAAAGAAGTGC	60.04	177
TRINITY_Contig9640	2	c	(GT)13(GA)8	42	825	866	TGGATTTGGTGACCTCGGTG	59.964	GCAGCTCACCAAAGAAGTGC	60.04	177
TRINITY_Contig9640	3	p3	(CGC)6	18	1453	1470	TGGATTTGGTGACCTCGGTG	59.964	GCAGCTCACCAAAGAAGTGC	60.04	177
TRINITY_Contig9645	1	p3	(CCG)6	18	600	617	GCACATAGCTTGCATGAGGC	59.97	CAATCAACAAAGGCGTGGCA	59.968	275
TRINITY_Contig9645	2	p3	(CCG)5	15	826	840	GCACATAGCTTGCATGAGGC	59.97	CAATCAACAAAGGCGTGGCA	59.968	275
TRINITY_Contig9647	1	p4	(CCGG)3	12	1424	1435	CTTTGCTTTCCTGGCCACAC	59.967	CTCTTCAGCACTTCCCCCTG	60.036	159
TRINITY_Contig9648	1	p2	(AG)16	32	111	142	ATCAAGGCTGGGTAGTTGGC	60.034	TCAAGAACCCCAGGTCTCCA	60.105	141
TRINITY_Contig9648	2	p3	(TGA)4	12	1099	1110	ATCAAGGCTGGGTAGTTGGC	60.034	TCAAGAACCCCAGGTCTCCA	60.105	141
TRINITY_Contig9649	1	c	(CGC)5(GGC)4	27	1450	1476	GGGTCAAGCAACTACAGGCT	59.963	TGACCAGCTGGGATGCAAAT	59.96	253
TRINITY_Contig9650	1	c	(GCGA)3gtgagctcgcgatgcggaggcga gcgagtggaggctggagaggcgaggtgggcg gcggcaggtggcgctcgtcgcgttgcgt(CGG G)3	106	1510	1615	ATCCAACAGCCCATCCAAGG	60.033	AACTCTTCCACTGGCGTCTG	59.966	101
TRINITY_Contig9650	2	p5	(AGGCG)3	15	1876	1890	ATCCAACAGCCCATCCAAGG	60.033	AACTCTTCCACTGGCGTCTG	59.966	101
TRINITY_Contig9654	1	p3	(GAC)5	15	371	385	TTCTTCCTCAACGCGCAGAT	60.037	TAGCCGCCATCGACATTCTC	59.968	124
TRINITY_Contig9654	2	p3	(CGG)5	15	1114	1128	TTCTTCCTCAACGCGCAGAT	60.037	TAGCCGCCATCGACATTCTC	59.968	124
TRINITY_Contig9654	3	p3	(GAG)5	15	1565	1579	TTCTTCCTCAACGCGCAGAT	60.037	TAGCCGCCATCGACATTCTC	59.968	124
TRINITY_Contig9655	1	p3	(AGC)4	12	2380	2391	TGAGCTTGCCCTTGAGGAAG	59.963	TGGCTCGCCCAAATTGTTTG	59.967	261
TRINITY_Contig9655	2	p3	(ACT)4	12	3132	3143	TGAGCTTGCCCTTGAGGAAG	59.963	TGGCTCGCCCAAATTGTTTG	59.967	261
TRINITY_Contig9656	1	p6	(TGTTGC)3	18	536	553	AGTAATCAGTGCACTGCCCC	60.035	CCAGTTGTTGCAGTTCTGGC	59.969	125
TRINITY_Contig9657	1	c	(ACGC)3gacggcaacggcaacaggagctc gcctaccagtacgccagggaaggcgcgtacct cgcgctggtggcccggaggaagcaggcgctc aagggcgt(CGC)4	118	513	630	CATGCCATGATCCGCCCTAT	60.035	TTTATTAGGGGCAGCCGCAA	60.034	116
TRINITY_Contig9659	1	c*	(GGGACG)2(GGGA<CG>G)(CGG)3 gggaggcgggcgaggcggaggggccggggg aggcgacgtcgacggcggaggcgtcccggcg aggccggcgccgggagaggccatgggcgcgg gcg(GGAA)3	135	1713	1847	TCCATCCAACAGCCTTGTCC	59.962	CGTCGTGACCTACAACTGCT	60.039	176
TRINITY_Contig9663	1	p3	(CAG)4	12	142	153	GAGGCGAGATCAGAGGCTTC	59.968	TCGCAAGTTCTTCTCGCCTT	59.966	131
TRINITY_Contig9663	2	p3	(GAG)4	12	1682	1693	GAGGCGAGATCAGAGGCTTC	59.968	TCGCAAGTTCTTCTCGCCTT	59.966	131
TRINITY_Contig9665	1	p3	(CGT)4	12	270	281	CGTCGTACATCCGGATCCTC	59.762	ACGACCTTTCAGCAGCAAGA	59.893	268
TRINITY_Contig9665	2	p3	(CGC)4	12	616	627	CGTCGTACATCCGGATCCTC	59.762	ACGACCTTTCAGCAGCAAGA	59.893	268
TRINITY_Contig9665	3	p3	(CCG)4	12	1092	1103	CGTCGTACATCCGGATCCTC	59.762	ACGACCTTTCAGCAGCAAGA	59.893	268
TRINITY_Contig9665	4	p3	(GTC)4	12	1475	1486	CGTCGTACATCCGGATCCTC	59.762	ACGACCTTTCAGCAGCAAGA	59.893	268
TRINITY_Contig9667	1	p5	(CAAGC)3	15	263	277	CAAACAGCATCAACACGGCA	59.969	TGGTGCTAAGTGGGACGAAC	59.966	243
TRINITY_Contig9668	1	p4	(AGGG)3	12	218	229	AAAGCTAGCCCTTCACCGTC	60.036	AGGGTAGTTTTGGCCGAGTG	59.964	174
TRINITY_Contig9668	2	p3	(GAC)4	12	1583	1594	AAAGCTAGCCCTTCACCGTC	60.036	AGGGTAGTTTTGGCCGAGTG	59.964	174
TRINITY_Contig9670	1	p4	(TTCA)3	12	167	178	CCGGTGAAGAGGACCTTGTC	60.037	CATCGGGAGGTACATGGACG	59.968	105
TRINITY_Contig9670	2	p3	(GCC)4	12	1084	1095	CCGGTGAAGAGGACCTTGTC	60.037	CATCGGGAGGTACATGGACG	59.968	105
TRINITY_Contig9671	1	c	(GGC)5gggaccagcgccggcagggaggag gacaagaagcgggtggggaaagctgcga(C GG)7ctactggagcttgtgatttgcagtgcgg gatgttgaatagcatggattaaagctctagtcc cgcccggcgacgcctcgccatgaatgacggc(GCG)4gaggagggaaggcacccgcggcaat ggcggcggcgcatccaccagatgccggcggg cgtacgaggtcgagg(GC)6cccaggcggcg gcccccgacctggagtcgagggcgggcggag agggctacccagatgaccaggaagcgacggc gtagcccgcagttgaccgtcgggg(GGC)4	384	1980	2363	TAGCCCCTTCGGTTAGCTCT	60.032	TGGTAGAGCAGGGCTGTTTG	59.963	180
TRINITY_Contig9672	1	p4	(CTGC)3	12	60	71	GTTGTTGTCCTCCACCTCGT	59.894	AAGAGCGTCAAGGTGTGGAG	59.966	186
TRINITY_Contig9672	2	p3	(TCG)6	18	421	438	GTTGTTGTCCTCCACCTCGT	59.894	AAGAGCGTCAAGGTGTGGAG	59.966	186
TRINITY_Contig9674	1	p3	(CCA)4	12	3	14	CCCATTGCTGGTACATGGGT	60.033	TGCGTGGCAAGAGGAAAAGA	60.179	260
TRINITY_Contig9674	2	c*	(CAGCAC)2(CAG<CAC>)(CAC)3	27	527	553	CCCATTGCTGGTACATGGGT	60.033	TGCGTGGCAAGAGGAAAAGA	60.179	260
TRINITY_Contig9674	3	p3	(GCG)5	15	834	848	CCCATTGCTGGTACATGGGT	60.033	TGCGTGGCAAGAGGAAAAGA	60.179	260
TRINITY_Contig9675	1	p3	(CGC)5	15	281	295	CAGGGGTCAGGCAGAGAAAG	60.036	TTCCGAGGAGTATGCGCATC	59.968	221
TRINITY_Contig9675	2	p5	(GCGGC)3	15	669	683	CAGGGGTCAGGCAGAGAAAG	60.036	TTCCGAGGAGTATGCGCATC	59.968	221
TRINITY_Contig9675	3	p3	(GCC)6	18	1439	1456	CAGGGGTCAGGCAGAGAAAG	60.036	TTCCGAGGAGTATGCGCATC	59.968	221
TRINITY_Contig9676	1	p3	(GCT)4	12	632	643	CACTACCGTGCCTTGTCTGT	59.966	GCCTTGAGCTGAGACCACAT	60.036	112
TRINITY_Contig9676	2	p3	(GCT)4	12	1388	1399	CACTACCGTGCCTTGTCTGT	59.966	GCCTTGAGCTGAGACCACAT	60.036	112
TRINITY_Contig9676	3	c	(CT)6agctctctctcccctgtatgtggtctcag ctcaaggctagctgctgttgcttctgtgtccaag tgtgattatatttgagagaggagaagga(GA TC)3	115	1606	1720	CACTACCGTGCCTTGTCTGT	59.966	GCCTTGAGCTGAGACCACAT	60.036	112
TRINITY_Contig9679	1	p4	(CGAG)4	16	478	493	GCTTTGGACTGGCGTCTACT	60.037	TGTGAACTGTAACCTCGCCC	59.966	103
TRINITY_Contig9679	2	c	(CGC)4agcct(GCG)5	32	742	773	GCTTTGGACTGGCGTCTACT	60.037	TGTGAACTGTAACCTCGCCC	59.966	103
TRINITY_Contig9680	1	p3	(CGG)4	12	1179	1190	TCGAGAGGAAGGAGGTGGAG	60.034	ATGTAGGTGCGGTCCGTTT	59.964	259
TRINITY_Contig9680	2	c	(ACGT)3gacgcgtgcgactggcccgatcat acttttgtac(TATG)6	70	1575	1644	TCGAGAGGAAGGAGGTGGAG	60.034	ATGTAGGTGCGGTCCGTTT	59.964	259
TRINITY_Contig9681	1	p3	(GAC)4	12	469	480	ACGACGACGACGATAATGGG	59.971	GGTGTAGGCCCATGAAGCTT	60.034	139
TRINITY_Contig9683	1	p5	(CCCTC)4	20	1304	1323	ATCATAGACATGGCAGCCGG	59.964	GGACGGTACTCTTGTTGCCA	59.966	237
TRINITY_Contig9683	2	c	(GCG)10agggcgcgcgaggagggcgagct ctcctccggcgc(CGA)4	77	1500	1576	ATCATAGACATGGCAGCCGG	59.964	GGACGGTACTCTTGTTGCCA	59.966	237
TRINITY_Contig9685	1	p5	(GCGCC)3	15	285	299	CTTTCCTTCGCCAGAGGTGT	59.965	GGCATGCATAGGAAACGCTG	59.97	135
TRINITY_Contig9686	1	c	(GTGA)3gtatgtaagtg(TGCAC)3	38	1723	1760	TCTGGGCTCTTGCTAGGGAT	60.031	GTCACCCCAAACGATTCCCT	59.962	280
TRINITY_Contig9689	1	p3	(CCG)4	12	633	644	AAGTACTGGGACGATCGGGA	60.034	TTCCATCAGGCAGCTTCCAG	60.035	199
TRINITY_Contig9691	1	p3	(GGC)5	15	405	419	CAGATCTGGGAGGGAAGGGA	60.031	TGCAGGAACCTGATGACGAC	60.037	180
TRINITY_Contig9691	2	p4	(GGAG)3	12	756	767	CAGATCTGGGAGGGAAGGGA	60.031	TGCAGGAACCTGATGACGAC	60.037	180
TRINITY_Contig9691	3	p3	(CGC)4	12	1551	1562	CAGATCTGGGAGGGAAGGGA	60.031	TGCAGGAACCTGATGACGAC	60.037	180
TRINITY_Contig9693	1	p4	(GATC)3	12	394	405	TCGACTCATGCCGTACATCG	59.971	TAGCTCCTCCTTGCACTCCT	59.958	234
TRINITY_Contig9693	2	p4	(CTAG)3	12	1095	1106	TCGACTCATGCCGTACATCG	59.971	TAGCTCCTCCTTGCACTCCT	59.958	234
TRINITY_Contig9693	3	c	(GGC)4(GGT)5	27	1479	1505	TCGACTCATGCCGTACATCG	59.971	TAGCTCCTCCTTGCACTCCT	59.958	234
TRINITY_Contig9693	4	c	(CCG)5acct(GCAA)4ccagtcgcgtcagc tcctcgatccctcgccggccgggtcagcggaa ag(CGAT)3	95	2051	2145	TCGACTCATGCCGTACATCG	59.971	TAGCTCCTCCTTGCACTCCT	59.958	234
TRINITY_Contig9693	5	p4	(GAAT)3	12	2866	2877	TCGACTCATGCCGTACATCG	59.971	TAGCTCCTCCTTGCACTCCT	59.958	234
TRINITY_Contig9696	1	p3	(CGG)4	12	1031	1042	CAAAAAGCGGTCAATGGCCA	59.967	GAGGTCAAGTGCGGTTCTCA	59.966	211
TRINITY_Contig9697	1	c	(CAT)4agaaatggattttgattataattacac gcacagagaccctctctactgtagtctgtagct gtgcaagtggtggtgtgcacgcctgata(AAC AG)3	115	34	148	GCGCAGAGACGATTCGTTTC	59.976	TGGGGAGGAGGAGGAAGAAG	59.957	200
TRINITY_Contig9697	2	p3	(TCC)4	12	333	344	GCGCAGAGACGATTCGTTTC	59.976	TGGGGAGGAGGAGGAAGAAG	59.957	200
TRINITY_Contig9697	3	p4	(GTGC)3	12	952	963	GCGCAGAGACGATTCGTTTC	59.976	TGGGGAGGAGGAGGAAGAAG	59.957	200
TRINITY_Contig9697	4	p5	(CGCCA)3	15	1226	1240	GCGCAGAGACGATTCGTTTC	59.976	TGGGGAGGAGGAGGAAGAAG	59.957	200
TRINITY_Contig9698	1	p3	(GCG)6	18	1698	1715	GTGGCGGACGGATAATGGAT	59.965	TCCTGACCTGCAATGTGGAC	59.963	219
TRINITY_Contig9699	1	p4	(AGCT)3	12	258	269	ACTGACTGGCTGACTGGTTG	59.893	ATTAATGGCTCCCGGCTAGC	59.963	234
TRINITY_Contig9699	2	p3	(CGG)4	12	391	402	ACTGACTGGCTGACTGGTTG	59.893	ATTAATGGCTCCCGGCTAGC	59.963	234
TRINITY_Contig9699	3	c	(TGC)4tggtggtggctgtagtactc(CTG)1 2	68	881	948	ACTGACTGGCTGACTGGTTG	59.893	ATTAATGGCTCCCGGCTAGC	59.963	234
TRINITY_Contig9699	4	p3	(CCA)6	18	1780	1797	ACTGACTGGCTGACTGGTTG	59.893	ATTAATGGCTCCCGGCTAGC	59.963	234
TRINITY_Contig9703	1	p3	(GGC)4	12	263	274	CTTCTTCTTGGGCACGAGGT	59.965	TGGCGTCTTCTTCTGCTACG	60.109	256
TRINITY_Contig9703	2	p6	(CGGCGC)3	18	1306	1323	CTTCTTCTTGGGCACGAGGT	59.965	TGGCGTCTTCTTCTGCTACG	60.109	256
TRINITY_Contig9703	3	p3	(GCG)4	12	1452	1463	CTTCTTCTTGGGCACGAGGT	59.965	TGGCGTCTTCTTCTGCTACG	60.109	256
TRINITY_Contig9704	1	p4	(AGGA)3	12	1579	1590	AGCGAGCTGTGAACCTAACC	60.037	CCTCTGAAGTCGTCGCCTTT	60.038	250
TRINITY_Contig9707	1	p5	(AATAC)3	15	223	237	GACCAACCAACAGGCTCGTA	59.966	TTGAGGCTCTGAGGATCCGA	60.033	241

TRINITY_Contig9707	2	p3	(CCG)4	12	1209	1220	GACCAACCAACAGGCTCGTA	59.966	TTGAGGCTCTGAGGATCCGA	60.033	241
TRINITY_Contig9707	3	c	(GCT)4gcgggtcctccgctgcctcgtccc(CCG)4	48	1324	1371	GACCAACCAACAGGCTCGTA	59.966	TTGAGGCTCTGAGGATCCGA	60.033	241
TRINITY_Contig9707	4	p4	(GCTC)3	12	1509	1520	GACCAACCAACAGGCTCGTA	59.966	TTGAGGCTCTGAGGATCCGA	60.033	241
TRINITY_Contig9708	1	p3	(ATG)4	12	879	890	CGCTGCCATATGAGGAGAGG	60.038	GGAATTAGCAAAGCGAGGCG	59.972	182
TRINITY_Contig9709	1	p3	(CGA)4	12	1467	1478	CCTGGCCAGGTGGAAGAATT	59.96	TCCTGCAGGATGCTGACAAG	60.036	180
TRINITY_Contig9710	1	p5	(CCCCG)3	15	125	139	TGTTACGGGTGCTGTTTGGT	60.107	AGGCTGCTGCAATGTAAGGT	59.961	243
TRINITY_Contig9710	2	p3	(CTG)8	24	1617	1640	TGTTACGGGTGCTGTTTGGT	60.107	AGGCTGCTGCAATGTAAGGT	59.961	243
TRINITY_Contig9711	1	p5	(AATAC)3	15	223	237	CGGTCACACCTCATCCACAA	59.965	CGCCATGAAGGTAATGCAGC	59.97	212
TRINITY_Contig9711	2	p3	(CCG)4	12	1207	1218	CGGTCACACCTCATCCACAA	59.965	CGCCATGAAGGTAATGCAGC	59.97	212
TRINITY_Contig9711	3	c	(GCT)4gcgggtcctccgctgcctcgtccc(CCG)4	48	1322	1369	CGGTCACACCTCATCCACAA	59.965	CGCCATGAAGGTAATGCAGC	59.97	212
TRINITY_Contig9711	4	p4	(GCTC)3	12	1507	1518	CGGTCACACCTCATCCACAA	59.965	CGCCATGAAGGTAATGCAGC	59.97	212
TRINITY_Contig9712	1	p3	(GGC)5	15	1779	1793	ACCTTGGCCTGCATCTTTGT	60.179	CAGCTATCCCTGCTACACGG	59.967	122
TRINITY_Contig9714	1	p3	(GAG)4	12	14	25	GCAAGAGGAGGAGGAGAGGA	60.033	GCTCCAGCGCAATTAGCTTC	59.971	234
TRINITY_Contig9714	2	c	(GCTGCC)3gccgccgct(GCC)4	39	147	185	GCAAGAGGAGGAGGAGAGGA	60.033	GCTCCAGCGCAATTAGCTTC	59.971	234
TRINITY_Contig9715	1	p3	(CGC)4	12	207	218	AAGGGACCTTAATTGGGGCG	60.034	ACCGCCATAAGCCTCATAGC	59.965	164
TRINITY_Contig9716	1	p3	(GCC)4	12	105	116	AGAAGGCCGTGGAGGTAGAT	60.032	GCCATAGCGACCATCTTCGA	59.968	257
TRINITY_Contig9716	2	p3	(GAC)4	12	408	419	AGAAGGCCGTGGAGGTAGAT	60.032	GCCATAGCGACCATCTTCGA	59.968	257
TRINITY_Contig9717	1	c	(GCT)4gaagtcgatggcacgtttcgaccc(CGA)4	48	909	956	CCTCCCTTTCCTCTCCTCCA	59.957	GATGGTGTTCAGGGACCTGG	60.035	131
TRINITY_Contig9717	2	p3	(GCT)4	12	1301	1312	CCTCCCTTTCCTCTCCTCCA	59.957	GATGGTGTTCAGGGACCTGG	60.035	131
TRINITY_Contig9718	1	p3	(CCG)4	12	418	429	GTGTTCAGTTGCCTGTGCTG	59.97	CTTCACGTCCAGTCGTCCAA	59.968	273
TRINITY_Contig9721	1	c	(GCT)4gtggtaggtggcgcgtcgctcttccttaactgctcgccggcg(CCTC)3	66	48	113	ATCCGAAGCTACAGCATCCG	59.967	TCCGCGAGTACCTCTTCTGA	60.036	240
TRINITY_Contig9721	2	p3	(GCC)4	12	865	876	ATCCGAAGCTACAGCATCCG	59.967	TCCGCGAGTACCTCTTCTGA	60.036	240
TRINITY_Contig9721	3	p6	(GCTCCC)4	24	1025	1048	ATCCGAAGCTACAGCATCCG	59.967	TCCGCGAGTACCTCTTCTGA	60.036	240
TRINITY_Contig9721	4	p4	(GCAC)3	12	1625	1636	ATCCGAAGCTACAGCATCCG	59.967	TCCGCGAGTACCTCTTCTGA	60.036	240
TRINITY_Contig9721	5	p3	(GCC)4	12	2102	2113	ATCCGAAGCTACAGCATCCG	59.967	TCCGCGAGTACCTCTTCTGA	60.036	240
TRINITY_Contig9722	1	p3	(CGC)5	15	1094	1108	CACTACACGCAGCCCACTAA	60.038	GTGTTTCTCGCGATGATGCC	59.973	279
TRINITY_Contig9722	2	p3	(GAG)4	12	1417	1428	CACTACACGCAGCCCACTAA	60.038	GTGTTTCTCGCGATGATGCC	59.973	279
TRINITY_Contig9723	1	c	(CGA)4ggccaccgggaaggtgcgcgtccgcgtacgccgcaagcgatcccgcgcctccgtctccgcttccgc(CCG)4	90	142	231	GGGAGTCCTTGAAGCCCTTC	60.035	CCACAGGCACATGAAGAGGT	59.963	265
TRINITY_Contig9725	1	p3	(CCG)4	12	1569	1580	TACACGAGCGCCTTCTTGTT	59.967	CGGTGTACAAGGGGGTTCTC	60.037	178
TRINITY_Contig9726	1	p3	(GGC)4	12	699	710	CCCTGAACTCCTTGAGCTGG	60.036	GACCATCAGCTCCCTCATCG	59.967	152
TRINITY_Contig9728	1	p3	(GCG)4	12	1051	1062	CTGCACGGGTTCAGCTTCTA	60.037	ATGAACACGGTGTCCATCCC	60.035	223
TRINITY_Contig9732	1	p3	(CCG)4	12	1611	1622	GTTCTGTCTCTGACGGTGCA	59.968	AGGTTCGTTCTTCCGGGAAC	59.966	116
TRINITY_Contig9734	1	p6	(CCGTAC)4	24	984	1007	GCCACTGCTCATATCGACGA	59.969	CATTGTGTGCTGCCGTTTCA	59.969	184
TRINITY_Contig9734	2	p5	(CAGAA)3	15	1555	1569	GCCACTGCTCATATCGACGA	59.969	CATTGTGTGCTGCCGTTTCA	59.969	184
TRINITY_Contig9734	3	p2	(GT)10	20	1730	1749	GCCACTGCTCATATCGACGA	59.969	CATTGTGTGCTGCCGTTTCA	59.969	184
TRINITY_Contig9735	1	p3	(GCA)4	12	397	408	CCGAACCCGATTCCCATCAA	60.107	TCCGCCGACTAGAGAGGAAA	60.035	130
TRINITY_Contig9737	1	c	(GCG)4gcctcgtcgtgcc(GCT)4	37	744	780	CACAGTTCGGACCACGGTAA	59.968	CGTCACGGTCTCTCTTGCTT	60.039	150
TRINITY_Contig9737	2	p2	(GA)6	12	1447	1458	CACAGTTCGGACCACGGTAA	59.968	CGTCACGGTCTCTCTTGCTT	60.039	150
TRINITY_Contig9737	3	p4	(GGAT)5	20	1593	1612	CACAGTTCGGACCACGGTAA	59.968	CGTCACGGTCTCTCTTGCTT	60.039	150
TRINITY_Contig9737	4	p5	(CTGCG)3	15	1715	1729	CACAGTTCGGACCACGGTAA	59.968	CGTCACGGTCTCTCTTGCTT	60.039	150
TRINITY_Contig9739	1	p3	(CGC)4	12	246	257	CTCGAGCTCGTCTGCTACAG	59.973	AGGTGTGACCCATCCTCGTA	59.959	206
TRINITY_Contig9740	1	p3	(GCC)4	12	856	867	GTGTGGCATCTGCGTTGTTT	59.969	GTGTGGCATCTGCGTTGTTT	59.969	216
TRINITY_Contig9741	1	p4	(CAAT)3	12	194	205	AGGTCAGCTCATTGTCCAGC	60.036	AGCCGTATCAGAATCGCCAG	59.967	147
TRINITY_Contig9741	2	p3	(AGT)4	12	620	631	AGGTCAGCTCATTGTCCAGC	60.036	AGCCGTATCAGAATCGCCAG	59.967	147
TRINITY_Contig9741	3	p4	(AACA)3	12	772	783	AGGTCAGCTCATTGTCCAGC	60.036	AGCCGTATCAGAATCGCCAG	59.967	147
TRINITY_Contig9741	4	c	(GAG)4cgagcgggccgccacctgcgccgcgcgtgcgctg(CCGC)3atgtgagcgcggaggcgtggcggctcatgtgctgtccgcccgccccgcgtctcgtgcgctgcatgtggggatgtggaaggt(TTGG)3aagcgccggcgggcgaggaggacggtgacgcgccgccctccgcgtgcgtggcggcggcagatcgatgcgcgccgccaccccgccggccagc(CAGG)5	263	1137	1399	AGGTCAGCTCATTGTCCAGC	60.036	AGCCGTATCAGAATCGCCAG	59.967	147
TRINITY_Contig9742	1	p3	(TAC)4	12	560	571	TGTCACAAGATCGCAGCCAT	60.036	GCTTTCAGGGGTTGTGCAAG	59.967	179
TRINITY_Contig9742	2	p4	(GAAG)3	12	786	797	TGTCACAAGATCGCAGCCAT	60.036	GCTTTCAGGGGTTGTGCAAG	59.967	179
TRINITY_Contig9742	3	p3	(GCG)4	12	3877	3888	TGTCACAAGATCGCAGCCAT	60.036	GCTTTCAGGGGTTGTGCAAG	59.967	179
TRINITY_Contig9742	4	p5	(GGGAC)3	15	4545	4559	TGTCACAAGATCGCAGCCAT	60.036	GCTTTCAGGGGTTGTGCAAG	59.967	179
TRINITY_Contig9744	1	p6	(AGAAGC)5	30	61	90	TGGGGAGGGATGGTTAGAGG	60.03	TCCAACTGCATCACCACTCC	59.963	101
TRINITY_Contig9745	1	p3	(CTG)4	12	552	563	GGGCTTCCTCATCAACTGCT	60.035	GCAGTTGCTCTCTCTGGTGT	59.965	214
TRINITY_Contig9746	1	p3	(GCG)4	12	1510	1521	CGTCAGAGCAGCACCAACTA	60.038	ATCACGGCGTCTGTTGTTCT	59.966	124
TRINITY_Contig9747	1	p3	(TCG)4	12	711	722	ATCGGTGTCGTTTTTGTGCG	60.042	GACCCAGTTTCCCCTTAGGC	60.034	208
TRINITY_Contig9748	1	p3	(CGG)5	15	638	652	ATCTTGGATGCTGGACGGTG	60.108	CATGGATGCGATGCACACAG	59.971	237
TRINITY_Contig9748	2	p3	(CGC)4	12	958	969	ATCTTGGATGCTGGACGGTG	60.108	CATGGATGCGATGCACACAG	59.971	237
TRINITY_Contig9748	3	p3	(GGC)4	12	1171	1182	ATCTTGGATGCTGGACGGTG	60.108	CATGGATGCGATGCACACAG	59.971	237
TRINITY_Contig9749	1	p4	(CTAG)3	12	48	59	CAGAACCTGGTCATCGCCTT	60.036	CCCTCGAGGTCCTTCCAGTA	60.033	134
TRINITY_Contig9749	2	p3	(TCG)4	12	1082	1093	CAGAACCTGGTCATCGCCTT	60.036	CCCTCGAGGTCCTTCCAGTA	60.033	134
TRINITY_Contig9749	3	p3	(CGG)4	12	1361	1372	CAGAACCTGGTCATCGCCTT	60.036	CCCTCGAGGTCCTTCCAGTA	60.033	134
TRINITY_Contig9750	1	p3	(CGG)4	12	1177	1188	GTCGATCTTGCTCCTCGAGG	59.97	CCCTCGTGCTCATCGTCTAC	59.97	278
TRINITY_Contig9751	1	p3	(CTT)4	12	894	905	CCGTGACATGGTGGGAGAAA	59.964	GTGTTGCCCTGGCTTCTTTG	59.967	102
TRINITY_Contig9752	1	c	(GCA)4gcggcg(GCA)4ggcc(CCG)4	46	492	537	CAGTGGACTACAGCTGCACA	59.966	GCTAGAGATGGAGCGGTGC	59.969	163
TRINITY_Contig9752	2	p3	(CTC)4	12	720	731	CAGTGGACTACAGCTGCACA	59.966	GCTAGAGATGGAGCGGTGC	59.969	163
TRINITY_Contig9752	3	p3	(TCT)4	12	992	1003	CAGTGGACTACAGCTGCACA	59.966	GCTAGAGATGGAGCGGTGC	59.969	163
TRINITY_Contig9752	4	p4	(GATC)4	16	1323	1338	CAGTGGACTACAGCTGCACA	59.966	GCTAGAGATGGAGCGGTGC	59.969	163
TRINITY_Contig9752	5	p3	(CGC)4	12	2037	2048	CAGTGGACTACAGCTGCACA	59.966	GCTAGAGATGGAGCGGTGC	59.969	163
TRINITY_Contig9752	6	c	(AGA)4tgccgccggtggtggt(GCC)4	40	2186	2225	CAGTGGACTACAGCTGCACA	59.966	GCTAGAGATGGAGCGGTGC	59.969	163
TRINITY_Contig9752	7	p4	(ACTG)3	12	2384	2395	CAGTGGACTACAGCTGCACA	59.966	GCTAGAGATGGAGCGGTGC	59.969	163
TRINITY_Contig9753	1	p3	(CCG)4	12	2089	2100	TGGATTTCTGAAGCGCCCAT	60.034	TCTGACTGGAGCACAAGCAG	59.966	112
TRINITY_Contig9754	1	p6	(CGCGGC)3	18	408	425	GGTCCATCACCACCACTACG	60.108	CTCCTCGTCGAGCTCATCAC	59.971	225
TRINITY_Contig9756	1	p3	(GCG)4	12	1450	1461	GCGAGGAGAACACAGGACAA	59.966	GTGGCTATCTATGCCGGCAT	60.036	269
TRINITY_Contig9757	1	p2	(AG)7	14	4	17	TGGAGACAGCGGAGGATACA	60.034	CCTCCATGCTTGCTTCATGC	59.898	203
TRINITY_Contig9757	2	c	(CG)8tgcttgctcgcgatctgctagggttccctccggcggggcccaccacccatgccgt(CGG)4cctccgcggcggcg(CTC)4	109	256	364	TGGAGACAGCGGAGGATACA	60.034	CCTCCATGCTTGCTTCATGC	59.898	203
TRINITY_Contig9758	1	p6	(GTGCCT)6	36	1619	1654	ATGGTACGAAGGCCAACTGG	60.035	AGTGTGGTGTGAGGGCAAAA	60.034	219
TRINITY_Contig9758	2	c	(CGG)4ggaaccctagcgcgggctccgggcggggcatgctcgggcgcgcggtcgcattccccgaggaaggagacgacgccagtgggggc(AGTGG)3	111	2133	2243	ATGGTACGAAGGCCAACTGG	60.035	AGTGTGGTGTGAGGGCAAAA	60.034	219
TRINITY_Contig9759	1	p3	(CAT)4	12	283	294	TCTGCTAGACTCACCGTCCA	59.962	GGTCAGTTCGTGGATGCTGA	60.037	212
TRINITY_Contig9760	1	p3	(CGC)4	12	178	189	TCCGAGGTGCAGCTACATTC	59.824	GCTCACTCAACCAGCAAAGC	60.04	108
TRINITY_Contig9760	2	c	(CCG)4cagc(AGG)4	28	807	834	TCCGAGGTGCAGCTACATTC	59.824	GCTCACTCAACCAGCAAAGC	60.04	108
TRINITY_Contig9762	1	c	(CTG)7aa(TCC)4	35	14	48	GTCGAGGAAGAGATCGCCAG	59.97	GATCGAATGCCTGCTGCTTG	59.971	240
TRINITY_Contig9762	2	c	(AAT)6aaaga(AGC)4	35	517	551	GTCGAGGAAGAGATCGCCAG	59.97	GATCGAATGCCTGCTGCTTG	59.971	240
TRINITY_Contig9762	3	c	(GGATGT)3catccccctggactacgaattccagtacctcctgcccatgtgatcttttatttctagttttttttgtt(TC)7tgtgtgtgtctgtctgtgatcatcatgcatgtactatatgtatgtatacttcca(GTAT)3	167	1596	1762	GTCGAGGAAGAGATCGCCAG	59.97	GATCGAATGCCTGCTGCTTG	59.971	240
TRINITY_Contig9763	1	p3	(CGC)4	12	66	77	TCCAGCGATCTCTCTGTGGA	60.034	CTCGAACTTTGCCGCTATGC	59.973	177
TRINITY_Contig9766	1	p3	(CGC)4	12	574	585	ACCTTCTCAAGCTCCTTCGC	60.037	GCGCTGCCTCTCTCTATCTG	60.039	267
TRINITY_Contig9767	1	p3	(CCG)4	12	865	876	CCCGTTTCCAGAAAGTCCCA	59.89	CTTCCGTCTGCCTTGTTCT	59.965	144
TRINITY_Contig9768	1	p3	(GCC)4	12	149	160	GACGGTGGGATAGCATGGAG	59.966	ATGATTCCACTGCCGCAAGA	60.035	153
TRINITY_Contig9768	2	p3	(CAT)4	12	312	323	GACGGTGGGATAGCATGGAG	59.966	ATGATTCCACTGCCGCAAGA	60.035	153
TRINITY_Contig9768	3	p3	(CTC)4	12	446	457	GACGGTGGGATAGCATGGAG	59.966	ATGATTCCACTGCCGCAAGA	60.035	153
TRINITY_Contig9770	1	p4	(CCGG)3	12	67	78	ATCCTCCGGCTGATTTGACC	59.819	CCCTCACCATCTCGTCATCG	59.969	151
TRINITY_Contig9770	2	p3	(GCC)4	12	267	278	ATCCTCCGGCTGATTTGACC	59.819	CCCTCACCATCTCGTCATCG	59.969	151
TRINITY_Contig9770	3	p3	(CCA)5	15	1069	1083	ATCCTCCGGCTGATTTGACC	59.819	CCCTCACCATCTCGTCATCG	59.969	151
TRINITY_Contig9771	1	p5	(CTGAA)3	15	337	351	GCCGGCTTTATTCGCTGAAG	59.972	TCCATGAGGTGACACCAACG	59.965	254

TRINITY_Contig9772	1	c*	(AAAG)3aatcgcaagaccatgtccttgttctcaggcagaggcggcatcatcagcaaccttgccttcctcggcacttgg(GGCAGC)3(GCA)6*	115	210	324	CACTCCAGATCAGCTTGCCA	60.036	GCGGTGGTGGAAGAAGAGAA	59.965	241
TRINITY_Contig9774	1	p4	(GTCT)3	12	1709	1720	CTCAATGCCGCAACCAAACA	59.968	AGGCCGAGCATCAATGTTGA	60.035	162
TRINITY_Contig9776	1	p4	(CTGG)3	12	164	175	CTTGCAAGAACAGGCGACAC	60.041	GTTCAAGCGCATCTACAGCG	59.974	171
TRINITY_Contig9776	2	p3	(CGG)4	12	1563	1574	CTTGCAAGAACAGGCGACAC	60.041	GTTCAAGCGCATCTACAGCG	59.974	171
TRINITY_Contig9781	1	p6	(CAGCCG)3	18	334	351	GGAACTGACACCACCGACAT	59.965	CGGTGGGAGCCTTCATTCTT	60.035	112
TRINITY_Contig9781	2	p3	(CCG)4	12	604	615	GGAACTGACACCACCGACAT	59.965	CGGTGGGAGCCTTCATTCTT	60.035	112
TRINITY_Contig9782	1	p6	(GGAGCA)3	18	1491	1508	AGTGGGATGAACAAGGCCAG	59.961	CGCTCTGAGATTCTCCGGTC	59.97	228
TRINITY_Contig9783	1	p3	(AAG)4	12	867	878	GTGTTGCCCTGGCTTCTTTG	59.967	CCGTGACATGGTGGGAGAAA	59.964	102
TRINITY_Contig9784	1	p3	(GGT)4	12	1721	1732	TGCATCCTTGAAGTCGCTGT	59.965	CCGCGACATCCGTTGATTTC	59.974	278
TRINITY_Contig9787	1	p3	(CGC)6	18	1326	1343	TCCATCCTCTGCACTCTCCA	59.958	AATGGCAAATGCAGTGCTGG	60.037	138
TRINITY_Contig9787	2	p5	(CGGCG)3	15	1652	1666	TCCATCCTCTGCACTCTCCA	59.958	AATGGCAAATGCAGTGCTGG	60.037	138
TRINITY_Contig9788	1	p4	(GATC)3	12	32	43	TCGCACATGATGACGGTGAA	60.038	TACGCGCCATTGCTGTAGAT	59.896	224
TRINITY_Contig9788	2	p3	(GAA)4	12	155	166	TCGCACATGATGACGGTGAA	60.038	TACGCGCCATTGCTGTAGAT	59.896	224
TRINITY_Contig9788	3	c	(AATT)3gctagcttattaattgttgcttgcttactgttacttatatatctagtaatagtcatatatacatatatataggcttaattac(TA)7tcccaaataaaattagtagctagttc(ATCG)3	146	1306	1451	TCGCACATGATGACGGTGAA	60.038	TACGCGCCATTGCTGTAGAT	59.896	224
TRINITY_Contig9788	4	p3	(ATT)4	12	1568	1579	TCGCACATGATGACGGTGAA	60.038	TACGCGCCATTGCTGTAGAT	59.896	224
TRINITY_Contig9790	1	p3	(GGC)4	12	1564	1575	TGGGAATCACGATCTACGCG	59.97	TGCTGGACGTTCAGGAACTC	59.966	271
TRINITY_Contig9791	1	p2	(GC)6	12	2185	2196	TCGTGTTGGAGCCTTGGTAC	59.966	GCGTCGAACGGAATTGTAGC	59.975	238
TRINITY_Contig9793	1	p3	(CGG)4	12	1297	1308	ACAGTGCACTCAGCTACCAC	59.966	CTGAAGTTGCCACTGGTTGC	59.969	145
TRINITY_Contig9797	1	p3	(GCT)4	12	732	743	GCACCGCAAGATATTGCTGG	59.97	TGCGTTGCATGGGTAGACAT	60.036	146
TRINITY_Contig9798	1	p3	(CCG)5	15	1020	1034	GAGGTCCTCCAATGGCTGAC	60.107	GGGTGGTGATCCAGAACCAG	60.035	191
TRINITY_Contig9799	1	p3	(TCA)4	12	111	122	CTACCGCGGCATAAACAACG	59.974	TATTTCCGGTATGCGGAGGC	59.966	197
TRINITY_Contig9799	2	p4	(GCCT)3	12	544	555	CTACCGCGGCATAAACAACG	59.974	TATTTCCGGTATGCGGAGGC	59.966	197
TRINITY_Contig9799	3	p3	(GGC)4	12	888	899	CTACCGCGGCATAAACAACG	59.974	TATTTCCGGTATGCGGAGGC	59.966	197
TRINITY_Contig9800	1	p6	(CCGCCT)4	24	106	129	TTCATCGGGTGGGTAGGACT	59.958	GTCTTCAACCTCGTCGTCGT	60.041	266
TRINITY_Contig9800	2	p3	(GAC)4	12	1476	1487	TTCATCGGGTGGGTAGGACT	59.958	GTCTTCAACCTCGTCGTCGT	60.041	266
TRINITY_Contig9803	1	c	(TGA)4tgctgtactctcaagtctcaactagcagacgagtcaggtaacaccacccctactcttcttcgcaaacgcacat(CAC)7	106	113	218	CGCATGAAAGCACGTATGGG	59.972	GGGTGGTGTTACCTGACTCG	60.038	168
TRINITY_Contig9803	2	p4	(GGGC)3	12	1655	1666	CGCATGAAAGCACGTATGGG	59.972	GGGTGGTGTTACCTGACTCG	60.038	168
TRINITY_Contig9804	1	p3	(CGG)5	15	1676	1690	TGCCCATACGTTCACGAACA	59.966	AAGTCCGGGATCAGCTACCT	60.032	156
TRINITY_Contig9805	1	p3	(GCC)5	15	42	56	ACGTTGTTTCTTGGAACGCG	59.973	ATAACACTCACCAGGGCAGC	60.035	223
TRINITY_Contig9806	1	c	(CGC)5cttcttgctcttgcgcctgcggtggcgtgagtagggcggccgagatggcgagctggcgcgctgcgcggtcgcgtc(CGG)4	102	838	939	CCATATGGTCGACGACGTGG	60.594	GAGAGGAGGTATCACCCCGT	60.106	112
TRINITY_Contig9807	1	p3	(GCC)6	18	18	35	ATCGCTCAGGGCATTGTCAA	60.035	AAGTGGCCAGTACTCGTGTG	59.966	249
TRINITY_Contig9808	1	p6	(CCCATG)3	18	1196	1213	AGCTCAGTTACTGCCGGAAC	60.037	GGGAAGAGGTGGGAGAGGAT	60.031	120
TRINITY_Contig9809	1	p5	(AACGG)3	15	361	375	CTCCACGACCATGTTCACCA	59.965	AAGACACCAGCATTCTCCCG	60.036	164
TRINITY_Contig9809	2	p6	(GGTGGA)3	18	978	995	CTCCACGACCATGTTCACCA	59.965	AAGACACCAGCATTCTCCCG	60.036	164
TRINITY_Contig9809	3	p3	(CGG)5	15	1106	1120	CTCCACGACCATGTTCACCA	59.965	AAGACACCAGCATTCTCCCG	60.036	164
TRINITY_Contig9809	4	p3	(GAC)4	12	1380	1391	CTCCACGACCATGTTCACCA	59.965	AAGACACCAGCATTCTCCCG	60.036	164
TRINITY_Contig9810	1	p3	(GAC)5	15	642	656	CGACGGTGATGGTACCTGAG	59.899	TCTCAAGCACCGAGTTCCAC	59.966	156
TRINITY_Contig9812	1	p3	(TCC)4	12	626	637	TCGATACAGGCCTGGACTGA	60.034	GGGGAGCAGAGAGAGGAGAA	60.033	113
TRINITY_Contig9812	2	p3	(CGG)4	12	775	786	TCGATACAGGCCTGGACTGA	60.034	GGGGAGCAGAGAGAGGAGAA	60.033	113
TRINITY_Contig9812	3	p3	(GGC)4	12	1479	1490	TCGATACAGGCCTGGACTGA	60.034	GGGGAGCAGAGAGAGGAGAA	60.033	113
TRINITY_Contig9813	1	p4	(CTAG)3	12	254	265	AGCCAGAGACAAGCACAGTC	59.965	CTGCAACAAACCTGTGACCG	59.971	232
TRINITY_Contig9813	2	p3	(GCG)4	12	420	431	AGCCAGAGACAAGCACAGTC	59.965	CTGCAACAAACCTGTGACCG	59.971	232
TRINITY_Contig9813	3	p3	(CAG)4	12	846	857	AGCCAGAGACAAGCACAGTC	59.965	CTGCAACAAACCTGTGACCG	59.971	232
TRINITY_Contig9816	1	p2	(TC)6	12	41	52	TTGCGGATCACAGGTACGAC	60.109	TCGACGTTTCAAGAGTGCGA	59.97	257
TRINITY_Contig9817	1	p4	(GAAT)4	16	46	61	CGTAGACGATCCCCATGACG	60.04	CAGCTCCTCTACCTGTCCCT	60.033	226
TRINITY_Contig9817	2	p3	(GGC)5	15	888	902	CGTAGACGATCCCCATGACG	60.04	CAGCTCCTCTACCTGTCCCT	60.033	226
TRINITY_Contig9817	3	c	(CGG)5cgtgcccgcagcacccccgcggtgccgctcctcccgcgcgc(TCG)4tggtcaccgtctccgtcgccatctctg(CTC)4ccccccgcgcgcgcgagccgtgaccctg(GCGT)3	148	1145	1292	CGTAGACGATCCCCATGACG	60.04	CAGCTCCTCTACCTGTCCCT	60.033	226
TRINITY_Contig9817	4	p4	(GCAC)3	12	1471	1482	CGTAGACGATCCCCATGACG	60.04	CAGCTCCTCTACCTGTCCCT	60.033	226
TRINITY_Contig9818	1	p3	(GAG)4	12	1690	1701	AAGGCCCCCAAGTTTGTCAA	60.032	GGCGTCAGGAGATCAAGGAG	59.896	191
TRINITY_Contig9819	1	p4	(CCGT)3	12	1367	1378	CGAGATCAAGGAGACGTCGG	59.972	CAATGATCTTGATGGCGCGG	60.041	251
TRINITY_Contig9820	1	p3	(GGC)4	12	539	550	ACTAGACCCATCCAGGCCAT	60.03	TCTCCTTGAACTTGGTCCGC	59.965	274
TRINITY_Contig9821	1	p4	(TTTC)3	12	107	118	CCCTGGGTCAGATGGAGAGA	60.032	CACGAACAAAATGGGCAGCA	59.968	258
TRINITY_Contig9823	1	c	(TTGCA)3tgcgctagtcttctcatcatctctgctagctgcttcctctctccccccacctcgatcgatcggccgtcgatcgtctgccctccggactcgctccca(ATCG)3	123	89	211	GGGGGAGGGGAGGATAAAGT	60.029	TATAGGCATCAGCTGGTGCG	59.966	135
TRINITY_Contig9823	2	p3	(CGA)4	12	764	775	GGGGGAGGGGAGGATAAAGT	60.029	TATAGGCATCAGCTGGTGCG	59.966	135
TRINITY_Contig9823	3	c	(AGC)4gccagcaggaggctgcaggccgggccggctgccatcatcgtccgggtcctcgccatgggaccggacgggag(GGT)4ggcaagcttgggaagggggcgccaccaccgcgcgccggaggtgtttgtggggccgggggaggggaggataaagttggggcaggcgcgggacgga(CG)6gagggcctgccggcgcgcctggagaggcagcgagctcgc(GACG)3acgacgcctacgcgcaccagctgatgcctatacttagacgacggccggcgcgcacatgatcgatgcgctcgcttcgct(AACC)3aatcatccctcccaatcatttccacatgcctgcct(CTGC)3	389	1246	1634	GGGGGAGGGGAGGATAAAGT	60.029	TATAGGCATCAGCTGGTGCG	59.966	135
TRINITY_Contig9827	1	p4	(CCAT)3	12	96	107	CCCAGCCCTCTAGAAGACCT	60.032	TTCCCGAGGATCCTTTTGCC	60.034	169
TRINITY_Contig9827	2	p3	(GCA)4	12	410	421	CCCAGCCCTCTAGAAGACCT	60.032	TTCCCGAGGATCCTTTTGCC	60.034	169
TRINITY_Contig9827	3	p3	(GGA)4	12	759	770	CCCAGCCCTCTAGAAGACCT	60.032	TTCCCGAGGATCCTTTTGCC	60.034	169
TRINITY_Contig9827	4	p3	(GCC)5	15	1089	1103	CCCAGCCCTCTAGAAGACCT	60.032	TTCCCGAGGATCCTTTTGCC	60.034	169
TRINITY_Contig9827	5	c	(AAG)11aaaagtgcggatttgtgccctgaatcttccctgctctg(TC)9	89	1519	1607	CCCAGCCCTCTAGAAGACCT	60.032	TTCCCGAGGATCCTTTTGCC	60.034	169
TRINITY_Contig9828	1	p3	(TCC)6	18	1317	1334	GGGTCCAGGCCAGAGGTATA	60.105	ATGGTGTTGTAGGCTTCGCA	59.964	270
TRINITY_Contig9828	2	p3	(GGC)4	12	1607	1618	GGGTCCAGGCCAGAGGTATA	60.105	ATGGTGTTGTAGGCTTCGCA	59.964	270
TRINITY_Contig9829	1	c	(GCG)6gccgcggcgtcgctgcccggcctcgacgccgacgccatccgccggctgcccgtcacgctgta(CCGC)3	92	482	573	GAGAAGGTGAAGGTGCTCCC	60.036	CTGATTCAAAGGCCGGGTCT	60.035	240
TRINITY_Contig9830	1	p3	(CGC)5	15	1460	1474	CGGTGAACAAGGAGCTGCTA	60.037	GGGTAGTCCAGGAGGTAGCA	60.032	136
TRINITY_Contig9830	2	p3	(GCG)6	18	1786	1803	CGGTGAACAAGGAGCTGCTA	60.037	GGGTAGTCCAGGAGGTAGCA	60.032	136
TRINITY_Contig9832	1	p6	(AAAAAT)3	18	22	39	GCCAAGAAGCTGGCTAGGAA	60.035	GGACCCGGGCAGTCAAATTA	60.035	142
TRINITY_Contig9833	1	p4	(TTTG)3	12	583	594	CTTCGCCTTCCTCGTTGACT	60.038	GTGTGGAAGATCCCATGGCA	60.034	178
TRINITY_Contig9833	2	p4	(TGTT)3	12	973	984	CTTCGCCTTCCTCGTTGACT	60.038	GTGTGGAAGATCCCATGGCA	60.034	178
TRINITY_Contig9834	1	p3	(CTC)4	12	364	375	GCTCTTTCTCCCTCGATCCG	59.969	CGACCCCTCCATCATCTTCG	59.967	112
TRINITY_Contig9834	2	c	(CAG)4cttct(GCA)4	29	1531	1559	GCTCTTTCTCCCTCGATCCG	59.969	CGACCCCTCCATCATCTTCG	59.967	112
TRINITY_Contig9834	3	c	(GCA)5gatgttgcatcatcagcaacagcaacttcagcagcagcacctc(CAG)5caactacaacagcagcagcaacaacaacagcttaat(CAG)4caattacaacaacagcagcagcaccttaat(CAG)4caattacaacaacagcagcagcaattacaacaacggcagcaacaccttaat(CAG)4caattacaacaacagcagcaactacaacaacagcagcaccttagtcagcagcaacagg(AGC)4	296	1716	2011	GCTCTTTCTCCCTCGATCCG	59.969	CGACCCCTCCATCATCTTCG	59.967	112
TRINITY_Contig9834	4	p3	(GCA)4	12	3443	3454	GCTCTTTCTCCCTCGATCCG	59.969	CGACCCCTCCATCATCTTCG	59.967	112
TRINITY_Contig9838	1	p2	(CT)8	16	136	151	CGCAAGGTCCTTGGTTTTGG	59.968	GCCGGACTACTACTCCATGC	59.968	253
TRINITY_Contig9838	2	p3	(GCT)5	15	1571	1585	CGCAAGGTCCTTGGTTTTGG	59.968	GCCGGACTACTACTCCATGC	59.968	253
TRINITY_Contig9840	1	p3	(GCC)4	12	223	234	TCAGGCTGGTACACTCCGTA	59.961	ACCCCTGACAAGGCATTGAG	59.961	218
TRINITY_Contig9840	2	p3	(CGA)4	12	610	621	TCAGGCTGGTACACTCCGTA	59.961	ACCCCTGACAAGGCATTGAG	59.961	218
TRINITY_Contig9843	1	p6	(CGCGCC)3	18	464	481	TTACCACTGCCAGTCGACAC	59.967	GTGTCTTGCCACCAACGTTC	59.971	274

TRINITY_Contig9844	1	p3	(GGA)4	12	1606	1617	CAAGCACTACAGAGCACGGA	60.038	TGTTCCTTAAGGCGTCCGTC	60.038	267
TRINITY_Contig9846	1	p3	(TCC)4	12	210	221	GGATCGTCAGCCAGTCAGAG	59.898	CCACCTTGCGTTCAAGCTTC	60.04	273
TRINITY_Contig9848	1	p3	(GCT)4	12	624	635	GATTCAATGCCGCCGAGAAC	59.972	CGATGTCGGAGCTCTGAGAC	59.971	246
TRINITY_Contig9850	1	p2	(CG)6	12	131	142	CGAGAGGAGCAGAAACAGCA	60.038	GCTCTTCTTCTTGGCGTCCT	60.037	104
TRINITY_Contig9850	2	p3	(CAG)4	12	680	691	CGAGAGGAGCAGAAACAGCA	60.038	GCTCTTCTTCTTGGCGTCCT	60.037	104
TRINITY_Contig9852	1	p3	(GCC)4	12	139	150	CTCAGGTCTTGGGCAGGAAG	60.036	AGCTCACCGTCCTCCAGTAT	60.033	184
TRINITY_Contig9852	2	p3	(GCG)5	15	503	517	CTCAGGTCTTGGGCAGGAAG	60.036	AGCTCACCGTCCTCCAGTAT	60.033	184
TRINITY_Contig9853	1	c	(AG)6g(GGA)5	28	41	68	TCCTCGTTCGGGTCAGTTTG	59.967	CAGCCTGACAGAGAAAGCCA	59.964	197
TRINITY_Contig9853	2	p3	(CGG)4	12	413	424	TCCTCGTTCGGGTCAGTTTG	59.967	CAGCCTGACAGAGAAAGCCA	59.964	197
TRINITY_Contig9853	3	p4	(GGGC)3	12	1129	1140	TCCTCGTTCGGGTCAGTTTG	59.967	CAGCCTGACAGAGAAAGCCA	59.964	197
TRINITY_Contig9853	4	p4	(GGCC)3	12	1570	1581	TCCTCGTTCGGGTCAGTTTG	59.967	CAGCCTGACAGAGAAAGCCA	59.964	197
TRINITY_Contig9853	5	p4	(GCGA)3	12	1869	1880	TCCTCGTTCGGGTCAGTTTG	59.967	CAGCCTGACAGAGAAAGCCA	59.964	197
TRINITY_Contig9854	1	p3	(GCC)4	12	1289	1300	CCGTAATCGCCTAAGCCGAT	60.039	TGACCAGGTACACTAGGGGG	59.958	115
TRINITY_Contig9854	2	p3	(CGT)5	15	1417	1431	CCGTAATCGCCTAAGCCGAT	60.039	TGACCAGGTACACTAGGGGG	59.958	115
TRINITY_Contig9855	1	c	(CG)6ccggaccggcagcgcca(TCCC)3	42	1221	1262	CCTCCCTCCTCCTCTCGAAA	60.033	CAGGGCACTGTTAAGCTCGA	60.037	142
TRINITY_Contig9856	1	p3	(GCG)4	12	1081	1092	CTCAACGACACCGGAATCCA	60.038	TGGATGATGTTGGCCTGCAT	60.033	133
TRINITY_Contig9857	1	p4	(CTCG)3	12	1511	1522	GATCCTCTCAGAGACGCGTG	59.971	AAGGATGGGAGGCTCTGGAT	60.029	129
TRINITY_Contig9858	1	p3	(CGG)4	12	158	169	TGTGCAGTGGTGAAAGCAGA	60.107	CACCCTTGGGCTCCATCAAT	60.033	141
TRINITY_Contig9858	2	p3	(GGC)6	18	1355	1372	TGTGCAGTGGTGAAAGCAGA	60.107	CACCCTTGGGCTCCATCAAT	60.033	141
TRINITY_Contig9859	1	p3	(GCC)6	18	517	534	CCCAGCTGTCGTAAGTGTT	59.965	AAGCCGTCTTGAGGTGTCTG	59.966	108
TRINITY_Contig9860	1	p3	(CCG)7	21	1161	1181	GAGAACGACCTTCCAGCCAA	59.965	GCCAAGACTGCAGCAGAGTA	60.037	233
TRINITY_Contig9862	1	c	(ACT)4atctaactgtcgtgt(TACA)3	39	834	872	CCTCTTTCCTGCTGCCATCA	60.035	ACCTCCATCGTTGCGTTTCT	59.965	213
TRINITY_Contig9862	2	p3	(CTC)4	12	2303	2314	CCTCTTTCCTGCTGCCATCA	60.035	ACCTCCATCGTTGCGTTTCT	59.965	213
TRINITY_Contig9866	1	p3	(GTG)4	12	763	774	TTGCATGGACTGTGGCCTAG	60.035	TGAGCCTACCTCATGCTCCT	60.032	155
TRINITY_Contig9868	1	p6	(CCAGCT)3	18	426	443	TTAGTTTGGCGTGCGTTTGG	59.97	GAGCGAACACAAAGCAGCAA	59.971	132
TRINITY_Contig9868	2	p3	(GAA)4	12	584	595	TTAGTTTGGCGTGCGTTTGG	59.97	GAGCGAACACAAAGCAGCAA	59.971	132
TRINITY_Contig9868	3	p6	(GCGCCG)3	18	1068	1085	TTAGTTTGGCGTGCGTTTGG	59.97	GAGCGAACACAAAGCAGCAA	59.971	132
TRINITY_Contig9870	1	p5	(GCTTG)3	15	3510	3524	CAGGCGAAACCAAAACCAGG	59.968	CTCGCTAAGCCGGTGATCAT	59.967	115
TRINITY_Contig9871	1	p3	(CGG)4	12	492	503	GGTAGTGTGGAGGGGGAGAT	60.031	CACTCTCCCCTGCTCTACCT	60.033	251
TRINITY_Contig9872	1	p3	(CTC)5	15	105	119	CTCCTCCTCCTCTTCCTGCT	60.032	ACTCAACCCTGCTAACACCG	59.965	228
TRINITY_Contig9872	2	p4	(TTTG)3	12	1613	1624	CTCCTCCTCCTCTTCCTGCT	60.032	ACTCAACCCTGCTAACACCG	59.965	228
TRINITY_Contig9873	1	p3	(GCG)4	12	1087	1098	GGTAGTGGTAAGTGCTGCGT	60.038	TCCAAGCACAGCCGATCAAT	60.035	106
TRINITY_Contig9877	1	p3	(GCC)6	18	230	247	GAGCCTCTGGTTTCTGTGCT	59.964	GGCTAGCCAAACTACCTGCA	60.035	263
TRINITY_Contig9877	2	p3	(CTG)5	15	407	421	GAGCCTCTGGTTTCTGTGCT	59.964	GGCTAGCCAAACTACCTGCA	60.035	263
TRINITY_Contig9877	3	p3	(GCG)4	12	2116	2127	GAGCCTCTGGTTTCTGTGCT	59.964	GGCTAGCCAAACTACCTGCA	60.035	263
TRINITY_Contig9877	4	c	(CTG)4cgcgggtggttgcttgctagcttgtgttcttcctctccctcacctcgcccccggcgttgatgaggagcagcat(GGA)5att(GAGG)3	115	2232	2346	GAGCCTCTGGTTTCTGTGCT	59.964	GGCTAGCCAAACTACCTGCA	60.035	263
TRINITY_Contig9877	5	p2	(AG)6	12	2560	2571	GAGCCTCTGGTTTCTGTGCT	59.964	GGCTAGCCAAACTACCTGCA	60.035	263
TRINITY_Contig9879	1	p3	(GGC)6	18	306	323	AGGGAGATGATGACCGGGAA	60.031	AGGACGAGCCACCTTCTTTG	59.965	100
TRINITY_Contig9879	2	p3	(GCC)4	12	807	818	AGGGAGATGATGACCGGGAA	60.031	AGGACGAGCCACCTTCTTTG	59.965	100
TRINITY_Contig9880	1	p6	(CGGCTG)3	18	252	269	GAAGGTTGAGGTTGAGCGGA	59.965	GAGGTAGTTGGTATCGGGCG	59.968	266
TRINITY_Contig9883	1	p3	(GCG)4	12	1319	1330	CAAGGACAACTGCGGCTTTC	60.04	ATGCTGCTCCAAGAAGGCAT	60.033	273
TRINITY_Contig9883	2	p3	(GCG)6	18	1470	1487	CAAGGACAACTGCGGCTTTC	60.04	ATGCTGCTCCAAGAAGGCAT	60.033	273
TRINITY_Contig9884	1	p3	(CTG)5	15	301	315	CAACGGCCAACAAGAACCAG	59.969	ATGTAACTCTGGTGCAGGCC	60.035	142
TRINITY_Contig9885	1	p5	(GGGAG)3	15	1536	1550	CTGCTGCAGGACTTTCTGGA	59.964	GATGGTGCTGCTGGAGAACT	60.036	124
TRINITY_Contig9886	1	p3	(CCG)4	12	181	192	CTTCAGTTCCCCCAGCTCAG	60.036	GGCGTTCCCGATGATGAGAT	59.966	207
TRINITY_Contig9887	1	c	(TC)8tt(TC)7(GA)8tttt(GTTA)4	68	800	867	TGCCTTTGGTTGTCACCTGT	60.034	GTTTGTTGGCAGGTCAGCTG	59.969	249
TRINITY_Contig9889	1	p3	(CGG)5	15	1587	1601	GGCTCTCGCATCGATGAAGA	59.969	TTGTGCTGCAGTAGTCTCCG	60.038	153
TRINITY_Contig9890	1	p3	(GCG)5	15	1563	1577	GCAACAGCGCGCAATGTATA	59.973	GGATGTCACACAACGGCAAC	60.04	252
TRINITY_Contig9891	1	p5	(TCCCC)3	15	60	74	GTGGTTCAGGCATGGAGGAA	59.962	ATGCCTTCACCCTGCTCTTC	60.035	100
TRINITY_Contig9893	1	c	(AGCT)3tgcttagcagcggcggggga(AGG)4	44	1721	1764	TGCGGTGTTGATTGTTGCTG	59.969	ACGGCCAGGGAATGTACAAG	60.035	154
TRINITY_Contig9898	1	p3	(CGT)4	12	1316	1327	AGGCCCTAAGTACTGGGGAG	60.031	ACCAACAGCAGCAGAGGATC	60.036	148
TRINITY_Contig9902	1	p3	(GCC)4	12	1605	1616	CGCAAACACAACCACCGAAT	59.97	ATAGGACACTGCCTTGGTGC	60.035	216
TRINITY_Contig9903	1	p3	(GCG)4	12	1217	1228	CTTCGACGTCTTCAGCCACT	60.039	ATGAGATCGATCGTGTGCCC	59.967	116
TRINITY_Contig9903	2	c	(GA)6aagagatggaggaagtggtcaagttttatttggaggctt(GA)13	77	1590	1666	CTTCGACGTCTTCAGCCACT	60.039	ATGAGATCGATCGTGTGCCC	59.967	116
TRINITY_Contig9904	1	p4	(AGCT)3	12	1534	1545	TTTATGTCCGACGTGGCGAA	60.038	CGAGTACTGGATCGACGACG	60.043	276
TRINITY_Contig9906	1	p3	(CGG)4	12	1438	1449	GCCGTTTCCACCTTGTTCAC	59.97	GGACACACAGGATTGTCCGT	59.965	193
TRINITY_Contig9907	1	p3	(CCA)4	12	1481	1492	CCTCCCATCTCCAGACCAGA	60.032	AGGAATTTGCTTGCGGGAGA	59.962	100
TRINITY_Contig9908	1	p3	(CGT)4	12	721	732	GCTCGAGGAAGGGGATCATG	59.965	CACTCCCAGTCCCACTGTTC	59.965	180
TRINITY_Contig9909	1	p2	(CT)12	24	34	57	GCAGATTCTCTCCCTCGACG	59.97	CAAGGTCACCATCCTGGACC	60.035	170
TRINITY_Contig9909	2	p4	(TTCT)3	12	1514	1525	GCAGATTCTCTCCCTCGACG	59.97	CAAGGTCACCATCCTGGACC	60.035	170
TRINITY_Contig9915	1	p5	(GGAAG)3	15	2754	2768	TGCTTGTGCTGAGGTCTCTG	59.966	CATTCTCCGGAATCCTCGGG	59.966	235
TRINITY_Contig9917	1	p3	(AGC)5	15	777	791	TACTTCATGTCCGGCTACGC	59.898	CGAAACCAAGCGAACTCGTC	59.839	279
TRINITY_Contig9921	1	p3	(GGC)5	15	1977	1991	TCTTCACTACCGCTCGAGGA	60.036	GCTGTCCTGCTGAACTCCAT	60.036	214
TRINITY_Contig9922	1	c	(CTCC)3ctctccctcgtgctctgttcgatgtgaccatgggcaaactaccatcctctccaaac(GAGG)3	80	213	292	CATTGATCAGCTTGAGCCGC	59.971	CTCTTCCACAACGGCCAGAT	60.036	208
TRINITY_Contig9922	2	p3	(GCT)4	12	885	896	CATTGATCAGCTTGAGCCGC	59.971	CTCTTCCACAACGGCCAGAT	60.036	208
TRINITY_Contig9922	3	c	(CCG)4tcc(TCG)4	27	1216	1242	CATTGATCAGCTTGAGCCGC	59.971	CTCTTCCACAACGGCCAGAT	60.036	208
TRINITY_Contig9922	4	p3	(CCG)5	15	1465	1479	CATTGATCAGCTTGAGCCGC	59.971	CTCTTCCACAACGGCCAGAT	60.036	208
TRINITY_Contig9923	1	p2	(CA)6	12	228	239	CTGAATCCGGTCGAGACGAG	59.972	GCCGGTCGCTTTCTGTTTTT	59.97	212
TRINITY_Contig9923	2	p3	(CGC)4	12	1273	1284	CTGAATCCGGTCGAGACGAG	59.972	GCCGGTCGCTTTCTGTTTTT	59.97	212
TRINITY_Contig9923	3	p3	(GCT)7	21	1452	1472	CTGAATCCGGTCGAGACGAG	59.972	GCCGGTCGCTTTCTGTTTTT	59.97	212
TRINITY_Contig9925	1	p5	(CGAAT)3	15	1549	1563	TCTTGGCACGCTCATCCTTT	59.963	GGCGAACCAACTGAAGAGGA	59.965	250
TRINITY_Contig9927	1	p6	(CATTGA)3	18	915	932	GCCGTCATCATCAGTTTGCC	59.901	TCGTTATCAGCTGATCCCGC	59.968	207
TRINITY_Contig9927	2	p6	(TCAATG)3	18	1988	2005	GCCGTCATCATCAGTTTGCC	59.901	TCGTTATCAGCTGATCCCGC	59.968	207
TRINITY_Contig9928	1	c	(GA)10gcagggaagattcagggcacaaatccgcactt(TTC)6	70	121	190	TTCCCGAGGATCCTTTTGCC	60.034	CCCAGCCCTCTAGAAGACCT	60.032	169
TRINITY_Contig9928	2	p3	(CGC)4	12	514	525	TTCCCGAGGATCCTTTTGCC	60.034	CCCAGCCCTCTAGAAGACCT	60.032	169
TRINITY_Contig9928	3	p3	(TCC)4	12	899	910	TTCCCGAGGATCCTTTTGCC	60.034	CCCAGCCCTCTAGAAGACCT	60.032	169
TRINITY_Contig9928	4	p3	(TGC)4	12	1248	1259	TTCCCGAGGATCCTTTTGCC	60.034	CCCAGCCCTCTAGAAGACCT	60.032	169
TRINITY_Contig9928	5	p4	(ATGG)3	12	1562	1573	TTCCCGAGGATCCTTTTGCC	60.034	CCCAGCCCTCTAGAAGACCT	60.032	169
TRINITY_Contig9931	1	p3	(GAG)4	12	1549	1560	CGAGGAGATGAGGTCCATGC	59.967	CCGAGGTGTCCATCGAGATG	59.969	224
TRINITY_Contig9932	1	p3	(GAA)5	15	944	958	AGGTGTCACATCCAGCTTGG	59.963	ATGCCGTGATGTCAACGCTA	60.108	125
TRINITY_Contig9934	1	p3	(GCG)4	12	1611	1622	AATTGGGCACGCCACTGATA	60.035	CTTCATATTTGGGGCTGCGC	59.969	194
TRINITY_Contig9935	1	p3	(CCG)4	12	1525	1536	CGATCGAAGAGGCAGAGGAC	59.97	CCTTACCACGCTCGATCTCC	59.969	174
TRINITY_Contig9936	1	p4	(CTGC)3	12	302	313	GGCGGACACAAAACCAAGTC	59.97	ACTCCATGCTCCTCCATCCT	60.03	117
TRINITY_Contig9938	1	p3	(GGC)4	12	1545	1556	CGGCGATCTATCAGCAGGTT	59.967	CTCCCCGGACTTCAAGAACC	60.036	132
TRINITY_Contig9940	1	p6	(GCTCCG)3	18	105	122	CGTTCTGGTTGCACAACAGG	59.971	GCGAACGTGTGGCTTCTTTT	59.971	221
TRINITY_Contig9940	2	p4	(ATCT)3	12	1500	1511	CGTTCTGGTTGCACAACAGG	59.971	GCGAACGTGTGGCTTCTTTT	59.971	221
TRINITY_Contig9941	1	p3	(TCC)6	18	213	230	GAGAGGTTGACTTTGGCGGA	59.965	CGTCAACAGCCTCAACTCCT	59.966	213
TRINITY_Contig9941	2	p3	(GCG)4	12	1266	1277	GAGAGGTTGACTTTGGCGGA	59.965	CGTCAACAGCCTCAACTCCT	59.966	213
TRINITY_Contig9941	3	p3	(AGC)4	12	1752	1763	GAGAGGTTGACTTTGGCGGA	59.965	CGTCAACAGCCTCAACTCCT	59.966	213
TRINITY_Contig9943	1	p3	(GCG)4	12	1469	1480	TCCTCGTCCTCATCACCCAT	60.032	TGCAGGGTCAAATGGAGCAT	59.96	136
TRINITY_Contig9944	1	p6	(CGCCGG)3	18	195	212	TCCTTCATGGCGTTCCTTCC	60.035	CCTCCCTAGCTGCTTTGCTT	60.035	165
TRINITY_Contig9944	2	c	(CGC)7cctcttcaccacgtcatccgcg(GCC)4	55	528	582	TCCTTCATGGCGTTCCTTCC	60.035	CCTCCCTAGCTGCTTTGCTT	60.035	165
TRINITY_Contig9944	3	c	(CT)7tctttttctgttttctctctcccctctctctatctgtctcttttctttttttctgatgtcgtctctctc(CT)6	94	1347	1440	TCCTTCATGGCGTTCCTTCC	60.035	CCTCCCTAGCTGCTTTGCTT	60.035	165
TRINITY_Contig9945	1	p4	(TTTG)3	12	990	1001	ACCAAACACACTGGTCAGCA	60.035	TCTCTGCGTGTTTGTTTGCG	59.973	143
TRINITY_Contig9945	2	p3	(GGC)4	12	1630	1641	ACCAAACACACTGGTCAGCA	60.035	TCTCTGCGTGTTTGTTTGCG	59.973	143
TRINITY_Contig9946	1	c	(CTC)4tctctattgctcggtcgcggggcgatggtggcagtctgtcttctt(CTC)4tctct(CGG)4	86	1098	1183	TCTCTGCTCCCTTCCTCTCC	60.033	AAGACAGACTGCCACCATCG	60.037	169
TRINITY_Contig9949	1	p4	(CGCA)3	12	89	100	AAACTCCTGGAGCAGTCTGC	59.964	TGTGGATGCACAGCGATCAT	60.108	105
TRINITY_Contig9950	1	p4	(TTGT)3	12	708	719	TGATTGCTGGGCCATCCATT	60.032	AGCAAATGACGGCTTCTCCA	59.963	107
TRINITY_Contig9951	1	p3	(AGG)5	15	887	901	CCTCGCCCAAGAAGTGTTCT	59.965	CTGCCTGCTCCTGAATTGGA	60.035	166
TRINITY_Contig9951	2	p3	(GCC)4	12	1570	1581	CCTCGCCCAAGAAGTGTTCT	59.965	CTGCCTGCTCCTGAATTGGA	60.035	166
TRINITY_Contig9953	1	p3	(AGG)4	12	1001	1012	TGGAAAAGACTACGGCGGAC	60.038	CAATCTCGGACTCGGACTCG	59.972	156
TRINITY_Contig9954	1	p3	(GCT)4	12	1414	1425	TCGATCTCCTCCTCGGTGTT	60.034	TCGAGGTGTGGAACAAGTCG	59.968	118

TRINITY_Contig9954	2	p3	(AGC)5	15	1556	1570	TCGATCTCCTCCTCGGTGTT	60.034	TCGAGGTGTGGAACAAGTCG	59.968	118
TRINITY_Contig9955	1	p3	(GGC)5	15	1543	1557	CAAAGGGCGAAACCAACAGG	59.968	CCCTGGGCTGAAGACATTGT	59.961	200
TRINITY_Contig9957	1	p4	(TAAC)3	12	824	835	GTTTGTTGGCAGGTCAGCTG	59.969	TGCCTTTGGTTGTCACCTGT	60.034	249
TRINITY_Contig9960	1	c	(CGC)4cctcgtcgtcgtcgccgccgcaatgcgcggcttcttgctcttgctggcgagcaccgccgactcaatgccccggg(CGC)6	105	560	664	CTGCATGAACTGGGTCGAGT	60.037	TGCCGGCCAGTTAGAATTGT	59.962	240
TRINITY_Contig9960	2	p6	(GCGGCC)3	18	949	966	CTGCATGAACTGGGTCGAGT	60.037	TGCCGGCCAGTTAGAATTGT	59.962	240
TRINITY_Contig9960	3	c	(GGC)4acacctgaccgaggagtgctgcggcggcgaccaggcagcggcgttcg(CGC)5	74	1071	1144	CTGCATGAACTGGGTCGAGT	60.037	TGCCGGCCAGTTAGAATTGT	59.962	240
TRINITY_Contig9961	1	p3	(GCG)4	12	620	631	CTTGAAGTGCGTCTCCCAGT	59.966	CACGCTTTGTGGGGAAACAG	59.969	244
TRINITY_Contig9962	1	p3	(GAA)4	12	1590	1601	TCAGCTCCTGATCTGACGGA	60.034	ACAGGGGGTACTCAGGATCC	60.031	186
TRINITY_Contig9963	1	p4	(GCGT)4	16	1490	1505	ACCAACTTGTCCATGGCGAT	59.962	ACGCTGTGAGGAGGTTGATG	60.037	130
TRINITY_Contig9966	1	c	(CGC)4gcgccggctcgccctcccctccgcatcctcccccgcccccgcggtagctccccgccgtttctcccgccgacgcggggccggctccgccgacgcagacgctc(CCG)4	124	249	372	ACTTGTTCGGATGAGGCTGG	60.036	ACGCTGCTTCAAGCTTGTTG	59.97	109
TRINITY_Contig9967	1	p3	(CGG)6	18	372	389	ATGGGGTGATTGAGGCTTGG	60.033	CCTGACCACAGCACCATCAT	60.035	109
TRINITY_Contig9967	2	p6	(CCGCGC)3	18	533	550	ATGGGGTGATTGAGGCTTGG	60.033	CCTGACCACAGCACCATCAT	60.035	109
TRINITY_Contig9968	1	p3	(AGC)4	12	14	25	GTGGACGGCGGGATATACTG	60.039	TCAACCTAGCACGTGTTGGG	60.25	146
TRINITY_Contig9968	2	p3	(CCT)5	15	191	205	GTGGACGGCGGGATATACTG	60.039	TCAACCTAGCACGTGTTGGG	60.25	146
TRINITY_Contig9969	1	p5	(CGCCC)3	15	1512	1526	CCATGCCACGGTATATGCCT	59.963	ATACCGCCGGTGGATCATTC	59.965	269
TRINITY_Contig9970	1	p3	(CCG)4	12	331	342	CTAACCGTACGGCCTGGTAC	59.899	ACCTCTCCCCCTCTCATCAC	60.032	169
TRINITY_Contig9970	2	p3	(GTG)5	15	1310	1324	CTAACCGTACGGCCTGGTAC	59.899	ACCTCTCCCCCTCTCATCAC	60.032	169
TRINITY_Contig9971	1	p2	(GC)6	12	252	263	CATCAAGATCGCCAAGCACG	59.973	ACGACGTCCTTCCCAAACTC	59.967	231
TRINITY_Contig9972	1	p3	(GCA)5	15	524	538	CTGCCTTTGCCAATGTGACC	60.038	CTGCTCCTACCCTTCCTCCT	60.032	190
TRINITY_Contig9973	1	p3	(GCG)4	12	1234	1245	TCTTACTGGTGGAAACGGCC	59.964	CTCTCCAGTGCTTCAGCCAA	59.964	142
TRINITY_Contig9974	1	p5	(GCCGC)3	15	1375	1389	TTTACACCGGACACGACCTG	59.968	TCGGAAAAGCAGCAGTAGCA	59.965	125
TRINITY_Contig9974	2	p5	(TTTTC)3	15	1615	1629	TTTACACCGGACACGACCTG	59.968	TCGGAAAAGCAGCAGTAGCA	59.965	125
TRINITY_Contig9975	1	c	(ATCG)3agtacaggcttagcgatgcgcggctggcgcgcgg(CGC)4	58	98	155	GCCAACCATTTTCGACCACC	60.039	CTCCTCGTCATGTCCATCGG	59.969	208
TRINITY_Contig9975	2	p4	(CGCC)3	12	530	541	GCCAACCATTTTCGACCACC	60.039	CTCCTCGTCATGTCCATCGG	59.969	208
TRINITY_Contig9975	3	p3	(CGC)4	12	894	905	GCCAACCATTTTCGACCACC	60.039	CTCCTCGTCATGTCCATCGG	59.969	208
TRINITY_Contig9975	4	p3	(CGG)4	12	1020	1031	GCCAACCATTTTCGACCACC	60.039	CTCCTCGTCATGTCCATCGG	59.969	208
TRINITY_Contig9975	5	p3	(CGG)4	12	1491	1502	GCCAACCATTTTCGACCACC	60.039	CTCCTCGTCATGTCCATCGG	59.969	208
TRINITY_Contig9975	6	p5	(GGCTC)3	15	1633	1647	GCCAACCATTTTCGACCACC	60.039	CTCCTCGTCATGTCCATCGG	59.969	208
TRINITY_Contig9976	1	c*	(AGC)3(<AGC>A)(AGCA)2agtgtgcgagcagcgtacgtacgtgacacgagacacggctagctagagccatagcacgcacacacgcgcac(AG)7	102	28	129	ATGTGGGCCTAGGATCCCTT	60.029	ATGAAACACACGCACACACG	59.973	228
TRINITY_Contig9976	2	c	(GCG)5gcattgcgccggccgagggccgcgcggccgccgccgtggaggagcgcgagctgcccctgatcgacgtcgagtgcctgctggcgacgacgggg(GGC)5	122	262	383	ATGTGGGCCTAGGATCCCTT	60.029	ATGAAACACACGCACACACG	59.973	228
TRINITY_Contig9976	3	p3	(GCT)4	12	1365	1376	ATGTGGGCCTAGGATCCCTT	60.029	ATGAAACACACGCACACACG	59.973	228
TRINITY_Contig9976	4	c*	(TGCG)4(T<GCG>CGCGCGCGCGTGTGTGCGTGTGTTTCATATTTTTTTCTTGCCTATTTCAAGGCGCATAATCGAACTGTGGTACCACATACCAGTGAATAAAATCGGCAAAGCG)(GC)5	29	1524	1552	ATGTGGGCCTAGGATCCCTT	60.029	ATGAAACACACGCACACACG	59.973	228
TRINITY_Contig9977	1	p5	(CATCC)4	20	64	83	TGTCTGCCAAGTTGCATTGC	59.968	AAGCAGGACTTGTGGTAGGC	59.963	182
TRINITY_Contig9977	2	p4	(GGTG)3	12	509	520	TGTCTGCCAAGTTGCATTGC	59.968	AAGCAGGACTTGTGGTAGGC	59.963	182
TRINITY_Contig9979	1	p3	(CGA)4	12	1108	1119	CGGCAGGAAAGGAAACTCCA	60.251	CGAGGGAGAAAGAGACGAGC	59.9	256
TRINITY_Contig9979	2	p4	(ATAC)3	12	1880	1891	CGGCAGGAAAGGAAACTCCA	60.251	CGAGGGAGAAAGAGACGAGC	59.9	256
TRINITY_Contig9980	1	p4	(AGGC)3	12	232	243	GGGAACTTCAGCATCCCCAA	59.96	GCATATCCCGTTGCCGAAAC	59.971	117
TRINITY_Contig9981	1	p3	(CCG)4	12	1501	1512	GAGCCGGGTAGCTGATGTAC	59.968	TGGTCCGCCTACATCTACCA	60.033	274
TRINITY_Contig9982	1	p6	(CAGCAA)3	18	218	235	GGGTGTTGGCGAGTCTACAA	59.966	AATGCATGTCACCTGGAGGG	60.034	258
TRINITY_Contig9983	1	c	(CCG)5gggcctccctggcgaccgctgccgta(GCC)4a(CCG)6	72	54	125	CTGAGGGCTCCAAGATCGTC	59.896	TTCTTCTTCAGTGACGGGGC	59.965	190
TRINITY_Contig9984	1	p3	(CGC)5	15	1263	1277	CAACAGACCTCCATGCCCAT	60.034	GTTTTTGCATGGGAGCTGCA	59.966	109
TRINITY_Contig9985	1	p4	(GGCG)6	24	274	297	CCATGGTTGCTGCATTGCTT	60.037	ATCCCACCAAGATGGCCAAG	60.033	277
TRINITY_Contig9986	1	c	(GCT)7gacgacga(CGG)4	41	268	308	GATCATGTGCAACGACGACG	59.976	CATGAGCCGGTTCACCTTCT	60.036	100
TRINITY_Contig9987	1	p3	(CGC)4	12	1576	1587	GCATTGCTCTTCTTTGGGGC	60.108	AGCTACATCGGCACAACACA	59.965	150
TRINITY_Contig9989	1	p3	(CCG)4	12	536	547	CACCATTGGCACTCTAGGCA	60.035	GTCAGAGTCGGATTCGGCAT	59.897	100
TRINITY_Contig9989	2	p3	(GGC)4	12	968	979	CACCATTGGCACTCTAGGCA	60.035	GTCAGAGTCGGATTCGGCAT	59.897	100
TRINITY_Contig9989	3	p2	(GA)8	16	1389	1404	CACCATTGGCACTCTAGGCA	60.035	GTCAGAGTCGGATTCGGCAT	59.897	100
TRINITY_Contig9991	1	p6	(GCGCCG)4	24	425	448	GATGTGGGCTCCGTATCCTG	59.966	GAGTGGAGGGGATGGAGACT	60.032	200
TRINITY_Contig9991	2	p3	(ACC)5	15	3465	3479	GATGTGGGCTCCGTATCCTG	59.966	GAGTGGAGGGGATGGAGACT	60.032	200
TRINITY_Contig9992	1	p3	(CGC)4	12	1340	1351	TAATGGGACCGTTGCAGACC	60.036	GACAACCCAAAGTCGGCAAC	59.97	204
TRINITY_Contig9992	2	p3	(GCG)5	15	3345	3359	TAATGGGACCGTTGCAGACC	60.036	GACAACCCAAAGTCGGCAAC	59.97	204
TRINITY_Contig9993	1	c	(CGG)4agggggaggagagcgcggcggcgaggcggagcgcgaagcgggtctggtgcgcgatggagaggcggatgtcggcggtggccat(GGTC)4	110	1424	1533	AAACAGCACCACACCAGTCA	60.035	CCATTCAGCCCAGAAGCAGA	60.035	237
TRINITY_Contig9994	1	p4	(TCCC)3	12	37	48	AGCGAGGCCAAATCTTTCGA	60.036	CACTGCCTGTCCCCATGATT	60.034	148
TRINITY_Contig9994	2	p3	(GCG)4	12	167	178	AGCGAGGCCAAATCTTTCGA	60.036	CACTGCCTGTCCCCATGATT	60.034	148
TRINITY_Contig9995	1	p3	(GGC)4	12	32	43	ACTGTTCTGGGGTATGCAGC	60.035	GTGGAGTCGTCTTCTTGGCA	59.966	227
TRINITY_Contig9995	2	p3	(GCC)5	15	180	194	ACTGTTCTGGGGTATGCAGC	60.035	GTGGAGTCGTCTTCTTGGCA	59.966	227
TRINITY_Contig9995	3	p3	(GGC)4	12	298	309	ACTGTTCTGGGGTATGCAGC	60.035	GTGGAGTCGTCTTCTTGGCA	59.966	227
TRINITY_Contig9995	4	p2	(GC)6	12	487	498	ACTGTTCTGGGGTATGCAGC	60.035	GTGGAGTCGTCTTCTTGGCA	59.966	227
TRINITY_Contig9996	1	p3	(CGC)5	15	49	63	AGCTCTCCCCTTCCCACTAG	60.032	TCAATTGGAGCAAGGGACCC	59.96	136
TRINITY_Contig9998	1	p4	(AACA)3	12	105	116	GTGTGAGGGGACAGAAGAGC	60.037	TGAGCAAGATCCAGTGCCTG	60.036	199
TRINITY_Contig9999	1	p3	(CCT)4	12	3148	3159	TCTTATCGTACGTCACCGCG	59.973	TCACACACTCGCACACTCTC	59.969	168
TRINITY_Contig10000	1	c	(AAC)4tagtgcactcttattactagcaatgcttattgcactcattcacatgatctat(AAG)4	76	246	321	GCAAGGGGAAGTGCCAAAAG	59.966	GCACCTCATTTGGCGTGTTT	59.968	154
TRINITY_Contig10001	1	p5	(CGAAT)3	15	83	97	TTGATGGGAAGCCACCATCC	60.033	CACTCTTCAGGCGTCACAGT	59.967	232
TRINITY_Contig10001	2	p3	(CGG)4	12	246	257	TTGATGGGAAGCCACCATCC	60.033	CACTCTTCAGGCGTCACAGT	59.967	232
TRINITY_Contig10003	1	p4	(GATA)3	12	91	102	TGATATCGGAGACAGCACGC	59.969	TGGATTGAGTTGGAGACGCC	60.036	111
TRINITY_Contig10003	2	p5	(CACAG)3	15	210	224	TGATATCGGAGACAGCACGC	59.969	TGGATTGAGTTGGAGACGCC	60.036	111
TRINITY_Contig10003	3	p4	(TCTG)4	16	1467	1482	TGATATCGGAGACAGCACGC	59.969	TGGATTGAGTTGGAGACGCC	60.036	111
TRINITY_Contig10008	1	p3	(GCC)11	33	14	46	ACTGAGAGCGCGTTCAAGAA	59.968	CAGGTCAGCGTTGGTCATCT	60.037	111
TRINITY_Contig10009	1	c	(GCC)4gacgagatggaaaacgcgtggacgcattgagagacggcggcggccgcgagcagcacggactggaagagcttcatatt(GA)8	105	1476	1580	TACTCGACAAAGACACGGCC	60.039	AAGGTGCTGAGCGACAATCA	59.965	107
TRINITY_Contig10010	1	p4	(GCTC)3	12	43	54	AATGGGACTCTGCGGGAAAG	60.035	CAGCATTCTCATGTGCGTCG	59.974	115
TRINITY_Contig10012	1	c	(TCTCCT)3tttccttctccttcctcttccgcctctccttctccttcctcctccgcctcctctcctcccccctccctctccttcctcctct(TCCGCC)3	117	1008	1124	TTTACGAAGGCTGCTCGTGT	59.967	TGGTCTACCCTTGCATGCTG	60.035	168
TRINITY_Contig10012	2	p3	(CCG)6	18	1234	1251	TTTACGAAGGCTGCTCGTGT	59.967	TGGTCTACCCTTGCATGCTG	60.035	168
TRINITY_Contig10012	3	p3	(CCT)4	12	1425	1436	TTTACGAAGGCTGCTCGTGT	59.967	TGGTCTACCCTTGCATGCTG	60.035	168
TRINITY_Contig10013	1	p3	(GCG)4	12	136	147	GATAGGTGGGCTTGGCACTT	60.034	GCACAAGAGCAAATAGGCCG	59.901	158
TRINITY_Contig10015	1	p3	(GCG)6	18	390	407	GCAGTCGAACATGAGGTCCA	60.037	CACGTGGACCTCTACTACGC	59.902	120
TRINITY_Contig10016	1	p2	(CA)6	12	12	23	TCACACACACACAACCGGAA	60.036	GCGAAATGGACTGCATCGAC	59.973	203
TRINITY_Contig10016	2	p3	(GCG)8	24	282	305	TCACACACACACAACCGGAA	60.036	GCGAAATGGACTGCATCGAC	59.973	203
TRINITY_Contig10017	1	p3	(CGC)4	12	259	270	GATCCTCAGATCCTGACGCG	60.04	CGCTTGCTGCTATGCTCTTG	59.972	116
TRINITY_Contig10017	2	p2	(GA)7	14	377	390	GATCCTCAGATCCTGACGCG	60.04	CGCTTGCTGCTATGCTCTTG	59.972	116
TRINITY_Contig10018	1	p3	(CGC)4	12	197	208	GACCTGGAGGCGGTTTACAA	59.964	GCACGCGCACTAAGAATCTG	59.974	224
TRINITY_Contig10018	2	p3	(CGC)4	12	610	621	GACCTGGAGGCGGTTTACAA	59.964	GCACGCGCACTAAGAATCTG	59.974	224
TRINITY_Contig10018	3	p6	(CGGCAT)3	18	755	772	GACCTGGAGGCGGTTTACAA	59.964	GCACGCGCACTAAGAATCTG	59.974	224
TRINITY_Contig10018	4	p6	(TCCGCC)3	18	954	971	GACCTGGAGGCGGTTTACAA	59.964	GCACGCGCACTAAGAATCTG	59.974	224
TRINITY_Contig10018	5	p3	(GCA)4	12	1112	1123	GACCTGGAGGCGGTTTACAA	59.964	GCACGCGCACTAAGAATCTG	59.974	224
TRINITY_Contig10019	1	c	(CCT)4ctgcctcctcctcccgtgcctctcccaaccactcccggctccttccccctcgccgacgg(CGGCGC)3	89	110	198	GCGACATGGAGAGTCTCGAG	59.971	AGCGGGTGATGAAGAATGCA	60.035	280

TRINITY_Contig10020	1	p3	(TGG)4	12	1478	1489	CTTGACCTTGGGGCAGTCAT	59.961	CGCCAACGTTTCGGAAAGTT	59.972	200
TRINITY_Contig10024	1	p3	(GCG)6	18	1	18	TAGCTGGCTGTTTGTCTCGG	60.037	TGAGGATTATCGTCGCTGGC	59.968	217
TRINITY_Contig10025	1	p3	(CGA)4	12	1200	1211	TCCTTCTCGCCCTTCCTGTA	59.959	CACACCGAGACCAGCTTCTT	59.966	191
TRINITY_Contig10026	1	p3	(CCG)5	15	64	78	CGTAGTTGATTTGCCTGGCG	59.903	GCTAACCCTTCTGCCTTGGT	59.962	141
TRINITY_Contig10026	2	p3	(CCG)4	12	214	225	CGTAGTTGATTTGCCTGGCG	59.903	GCTAACCCTTCTGCCTTGGT	59.962	141
TRINITY_Contig10027	1	p3	(GCC)4	12	479	490	GTCACGTACTCCTGCTCGTC	60.179	GGATGCTCGACAAGTTCCCA	60.036	214
TRINITY_Contig10027	2	p3	(GGC)4	12	1285	1296	GTCACGTACTCCTGCTCGTC	60.179	GGATGCTCGACAAGTTCCCA	60.036	214
TRINITY_Contig10029	1	p3	(CGG)4	12	1100	1111	GCGACTTGCAAGCAATCACA	60.04	GCTGGAGGCTTTATGCTTGC	59.898	207
TRINITY_Contig10031	1	p4	(AATT)3	12	294	305	TGGACTCTGAGGCAGCAATG	60.036	TTGGGGTGCTGTCTGAATCC	59.962	207
TRINITY_Contig10031	2	p3	(GCC)5	15	1327	1341	TGGACTCTGAGGCAGCAATG	60.036	TTGGGGTGCTGTCTGAATCC	59.962	207
TRINITY_Contig10032	1	p3	(GCC)4	12	1557	1568	ACAGCTCCAAGAAGCTCACC	59.964	GGCCGGACAATTGTGCTTTT	59.967	183
TRINITY_Contig10033	1	p3	(CGC)4	12	300	311	GTGACAACAGACAGGGGAGG	59.965	AGGAGGTGAAAAGGCATCCG	60.035	117
TRINITY_Contig10033	2	p3	(GAC)4	12	541	552	GTGACAACAGACAGGGGAGG	59.965	AGGAGGTGAAAAGGCATCCG	60.035	117
TRINITY_Contig10034	1	p3	(CGG)5	15	1510	1524	GGCGCGTGTAGTTATGCTTG	59.974	CACAAGGGAAGCAGCAGAGA	59.964	108
TRINITY_Contig10035	1	p4	(AGCA)3	12	264	275	TGTAGGCTCTCTGGATGGCT	60.032	ATTATCTGGTCCAGGGCCCT	60.029	107
TRINITY_Contig10035	2	p3	(ATA)4	12	3105	3116	TGTAGGCTCTCTGGATGGCT	60.032	ATTATCTGGTCCAGGGCCCT	60.029	107
TRINITY_Contig10035	3	p2	(TG)7	14	3960	3973	TGTAGGCTCTCTGGATGGCT	60.032	ATTATCTGGTCCAGGGCCCT	60.029	107
TRINITY_Contig10036	1	c	(AGCT)3tgctcgccaatcgccatggcggccaccaccatccct(CTC)5	63	58	120	AGAACGTCAACTCCGTCGTC	60.041	CTAGCTACCCGTGACGAACC	59.9	220
TRINITY_Contig10039	1	p4	(GATA)4	16	251	266	GCGAGTATAAAAGCGCAGGC	60.042	TCCTGCTGCACGATGATCTC	59.896	251
TRINITY_Contig10039	2	p4	(GATT)3	12	1319	1330	GCGAGTATAAAAGCGCAGGC	60.042	TCCTGCTGCACGATGATCTC	59.896	251
TRINITY_Contig10042	1	c	(GGC)4gcccaccc(CCA)4	32	107	138	GTCGAAGGTGGCTTTGAGGA	59.965	TCTCCTCTCCATCCACCAGG	60.032	148
TRINITY_Contig10042	2	p6	(GGGCCA)3	18	266	283	GTCGAAGGTGGCTTTGAGGA	59.965	TCTCCTCTCCATCCACCAGG	60.032	148
TRINITY_Contig10043	1	c	(GGC)6aggggcaaacccgcggcgcagtcccgtcggcgtcgcggcggcacctgtcgtcg(TCC)4	84	645	728	CAACCCGTACCTGGACTTCC	60.037	CACACCTCTGCTCTGCTCAA	59.966	229
TRINITY_Contig10043	2	p3	(CGC)4	12	1151	1162	CAACCCGTACCTGGACTTCC	60.037	CACACCTCTGCTCTGCTCAA	59.966	229
TRINITY_Contig10044	1	p3	(GCG)10	30	434	463	GGCCTGGAAGAGTGAGCAAT	60.035	CGTCAAGTACAGCTCCTGCA	60.038	108
TRINITY_Contig10044	2	p3	(CGC)6	18	2580	2597	GGCCTGGAAGAGTGAGCAAT	60.035	CGTCAAGTACAGCTCCTGCA	60.038	108
TRINITY_Contig10044	3	p3	(CCG)5	15	2768	2782	GGCCTGGAAGAGTGAGCAAT	60.035	CGTCAAGTACAGCTCCTGCA	60.038	108
TRINITY_Contig10046	1	p3	(CGG)4	12	988	999	GCTGTCAACCGTGTGTCAAC	59.972	CACAGGTACACGGAGCTTGT	59.966	156
TRINITY_Contig10046	2	p4	(ACCA)3	12	1431	1442	GCTGTCAACCGTGTGTCAAC	59.972	CACAGGTACACGGAGCTTGT	59.966	156
TRINITY_Contig10048	1	p5	(GCGTT)3	15	242	256	GGCCAAGGCTTGATCTGTCT	60.035	CTGTGTCAGTGCTGGTCCAT	59.964	134
TRINITY_Contig10049	1	c	(CTCC)3ctcgtccgcgtgctcctccccttccccctccgccggcgcgggcggctcgcggccgccgcctcggtccccggcggctgcggaaggg(GCT)7	119	988	1106	GGCCTCCATCTCCATAGCAC	59.964	TGCAGAGGTGTGAGAACGTC	59.968	233
TRINITY_Contig10049	2	p3	(GGA)7	21	1350	1370	GGCCTCCATCTCCATAGCAC	59.964	TGCAGAGGTGTGAGAACGTC	59.968	233
TRINITY_Contig10049	3	p5	(CGTCT)3	15	1499	1513	GGCCTCCATCTCCATAGCAC	59.964	TGCAGAGGTGTGAGAACGTC	59.968	233
TRINITY_Contig10050	1	p3	(GGT)4	12	1323	1334	TCCCAGCCTCAAGATACCGA	60.033	CACTATCCGGACGAAGAGGC	59.969	226
TRINITY_Contig10050	2	p3	(AGC)5	15	1438	1452	TCCCAGCCTCAAGATACCGA	60.033	CACTATCCGGACGAAGAGGC	59.969	226
TRINITY_Contig10051	1	p3	(GCG)4	12	268	279	GTGGCGCAAATGGTTCGATT	60.109	ACCCATGGCCAAAGAAGAGG	59.96	276
TRINITY_Contig10053	1	p3	(CGG)6	18	1486	1503	ATCGAGCCATGCTTCTCTCG	59.968	CAAACTGACTTGGCACACCG	59.971	238
TRINITY_Contig10054	1	p3	(CGC)4	12	851	862	GGGGTCAAGGTCAACTACGG	60.037	GACATGTGGGGTAGTTGCCA	59.962	137
TRINITY_Contig10054	2	p3	(GCG)4	12	1062	1073	GGGGTCAAGGTCAACTACGG	60.037	GACATGTGGGGTAGTTGCCA	59.962	137
TRINITY_Contig10055	1	p2	(TC)7	14	1531	1544	CTAGGATGCTTCGGCTGGAG	59.967	GCTGCTGCCCCTACAACTAA	60.035	145
TRINITY_Contig10057	1	p3	(GGC)5	15	3353	3367	GCGACGGAAACATGCTGATC	59.973	GCAGCCTTCTTCCTTCCCTT	59.961	235
TRINITY_Contig10063	1	p3	(GCC)4	12	187	198	ATCTCGCCGGTGATGTTGTT	60.036	ATCGTGCTCAAGTCCAACCC	60.322	182
TRINITY_Contig10065	1	p3	(CTC)4	12	1050	1061	TCTCCACGCATAGTCCTCCA	60.034	TGGACAACATCATCTCGCCC	60.108	170
TRINITY_Contig10065	2	p3	(GGC)4	12	1461	1472	TCTCCACGCATAGTCCTCCA	60.034	TGGACAACATCATCTCGCCC	60.108	170
TRINITY_Contig10069	1	p6	(CTCGGC)3	18	52	69	AGGTGAGGAGCTTGAGGGAT	59.956	TGTATCAGCAGCAACCCCTG	60.035	220
TRINITY_Contig10069	2	p2	(TA)8	16	1124	1139	AGGTGAGGAGCTTGAGGGAT	59.956	TGTATCAGCAGCAACCCCTG	60.035	220
TRINITY_Contig10072	1	p3	(GTC)4	12	1968	1979	CGGCGAGACAATAAGCAAGC	59.973	GCTCTTCGATCCTCTGGACG	59.97	102
TRINITY_Contig10072	2	p4	(GATG)4	16	2439	2454	CGGCGAGACAATAAGCAAGC	59.973	GCTCTTCGATCCTCTGGACG	59.97	102
TRINITY_Contig10073	1	p3	(CCG)5	15	565	579	CGACGAGGAGATGGATCAGC	60.04	CTTCTGCTGCTGCTCTCTGT	60.037	262
TRINITY_Contig10075	1	p3	(GCT)4	12	788	799	CATGGCTGAGATGATCCGCT	59.965	AGGTTCGTCACGTGTTCCTC	59.968	253
TRINITY_Contig10076	1	c	(AAAT)3ccaagagccccgtgtctcaa(CT)10tttatt(TC)6	70	104	173	CTCGAGCATCACGGACTACC	59.97	CTGCTCCTCTTCCTCCTCCT	60.032	277
TRINITY_Contig10076	2	c	(GCTGGA)3ggccatggtgggggcctctacggggatc(CTGCCG)3ctgatcacttcgccgccttcgaccacgacgacttcttcttccagagatcgccgtgcgccggggaa(GGC)4	141	281	421	CTCGAGCATCACGGACTACC	59.97	CTGCTCCTCTTCCTCCTCCT	60.032	277
TRINITY_Contig10076	3	p6	(CGGTGC)4	24	543	566	CTCGAGCATCACGGACTACC	59.97	CTGCTCCTCTTCCTCCTCCT	60.032	277
TRINITY_Contig10076	4	p3	(GGA)4	12	697	708	CTCGAGCATCACGGACTACC	59.97	CTGCTCCTCTTCCTCCTCCT	60.032	277
TRINITY_Contig10076	5	p3	(GGC)5	15	1052	1066	CTCGAGCATCACGGACTACC	59.97	CTGCTCCTCTTCCTCCTCCT	60.032	277
TRINITY_Contig10077	1	p6	(CCCGCG)3	18	186	203	AGAAGTACACCCTTGCCAGC	59.963	GCCAGTACTGCTCGAACCTT	60.037	120
TRINITY_Contig10080	1	p6	(CCAGCG)3	18	1083	1100	GCCTCTCCTCGATCAGCTTC	59.968	ATTGACGTTCGAGCTGGTGT	59.966	263
TRINITY_Contig10081	1	p6	(CCGCTC)3	18	1321	1338	TGCCCTGTTCCTCATCAACC	59.962	GGCAACTTCAGGCTGAGGAT	60.035	192
TRINITY_Contig10082	1	p3	(CGG)4	12	226	237	TCCAAGATCAACCTCGACGC	60.109	GTTCACCGTCGTGTTGTTGG	59.973	186
TRINITY_Contig10083	1	p3	(CTC)5	15	697	711	AGCTCCTTCATGACGTGCTC	60.108	AAGAGCTGGAGGAGGAGGAG	60.032	184
TRINITY_Contig10083	2	p3	(GCC)4	12	1216	1227	AGCTCCTTCATGACGTGCTC	60.108	AAGAGCTGGAGGAGGAGGAG	60.032	184
TRINITY_Contig10083	3	p2	(GA)25	50	1408	1457	AGCTCCTTCATGACGTGCTC	60.108	AAGAGCTGGAGGAGGAGGAG	60.032	184
TRINITY_Contig10084	1	p5	(AAAAG)3	15	160	174	CGATGTCAAGCCTTGATGCG	59.973	AGCCGACCTACAGCTAAGGA	60.033	130
TRINITY_Contig10084	2	p3	(CAA)4	12	878	889	CGATGTCAAGCCTTGATGCG	59.973	AGCCGACCTACAGCTAAGGA	60.033	130
TRINITY_Contig10085	1	p4	(CAAT)3	12	455	466	CGATGGCGCTCTTGGATTTG	59.971	TCCCGACCTTCTCCATCCAT	60.031	220
TRINITY_Contig10085	2	p3	(GGC)6	18	1830	1847	CGATGGCGCTCTTGGATTTG	59.971	TCCCGACCTTCTCCATCCAT	60.031	220
TRINITY_Contig10087	1	p4	(TCGA)3	12	1197	1208	TCTCCCAGACGCTTGCATTT	59.963	CAGAGTCGGCAAGGGGATTT	60.035	142
TRINITY_Contig10089	1	p2	(AC)6	12	231	242	ACTAGTCGAACCTGCCATGC	60.108	TGATGAAGAACTCGTGGGGC	60.036	245
TRINITY_Contig10089	2	p3	(GCG)4	12	768	779	ACTAGTCGAACCTGCCATGC	60.108	TGATGAAGAACTCGTGGGGC	60.036	245
TRINITY_Contig10089	3	p3	(GCG)4	12	1233	1244	ACTAGTCGAACCTGCCATGC	60.108	TGATGAAGAACTCGTGGGGC	60.036	245
TRINITY_Contig10091	1	p6	(ACCTGA)3	18	158	175	GGAGGCGAGGAATACGAAGG	59.968	CTCGCCATCATGTCCTCCTC	59.967	150
TRINITY_Contig10091	2	p3	(CGC)6	18	675	692	GGAGGCGAGGAATACGAAGG	59.968	CTCGCCATCATGTCCTCCTC	59.967	150
TRINITY_Contig10091	3	p3	(GGA)4	12	1369	1380	GGAGGCGAGGAATACGAAGG	59.968	CTCGCCATCATGTCCTCCTC	59.967	150
TRINITY_Contig10093	1	p3	(GGT)4	12	342	353	CTACAGCTACGGCGTGCATA	59.97	ACAACAGCTCCAGTACAGGC	59.965	263
TRINITY_Contig10093	2	p3	(GGC)4	12	1445	1456	CTACAGCTACGGCGTGCATA	59.97	ACAACAGCTCCAGTACAGGC	59.965	263
TRINITY_Contig10094	1	p4	(CCGC)3	12	157	168	GGCATTGTGAAGGAGGAGCT	60.035	CAGGTCGAACGTCTTGGTCA	59.968	141
TRINITY_Contig10094	2	c	(CGG)5ccgcgggcattgtgaaggaggagctcctggagcagcagccgctgcaggacggcgtgc(GCG)5	87	471	557	GGCATTGTGAAGGAGGAGCT	60.035	CAGGTCGAACGTCTTGGTCA	59.968	141
TRINITY_Contig10096	1	p3	(GGC)4	12	1508	1519	GCGTTCTTCAGGAGGCTGAT	60.108	CTGCTGTCTTCCGCTTCTGA	60.038	218
TRINITY_Contig10097	1	p3	(CCG)4	12	224	235	ATTGGTGGCCTCACAGATGG	60.034	GGCAACCAGTACTGTGACCA	59.892	232
TRINITY_Contig10098	1	p3	(CGC)4	12	519	530	CGAATGAGATCTTCCGGCGA	59.969	TCAGGACAACGAAGCTGACC	59.966	124
TRINITY_Contig10098	2	p3	(CAG)4	12	1246	1257	CGAATGAGATCTTCCGGCGA	59.969	TCAGGACAACGAAGCTGACC	59.966	124
TRINITY_Contig10098	3	p3	(CGC)4	12	1605	1616	CGAATGAGATCTTCCGGCGA	59.969	TCAGGACAACGAAGCTGACC	59.966	124
TRINITY_Contig10099	1	p4	(GCTT)4	16	1108	1123	ACATTGTCTTTCGGCCCCAT	59.96	GCTTTGCCTGCTTCCAAACA	59.895	119
TRINITY_Contig10101	1	p3	(CCG)6	18	1554	1571	GTGAAGCCATGCCTTCTCCT	60.035	GCCAGCGACGATAATCCTCA	59.968	150
TRINITY_Contig10102	1	p3	(CCG)5	15	144	158	CACCATCACCTAGCAGCCAA	60.035	GAGAGAGACGCCTGCATCTC	59.969	118
TRINITY_Contig10102	2	p3	(GCC)7	21	705	725	CACCATCACCTAGCAGCCAA	60.035	GAGAGAGACGCCTGCATCTC	59.969	118
TRINITY_Contig10102	3	p3	(CCA)5	15	916	930	CACCATCACCTAGCAGCCAA	60.035	GAGAGAGACGCCTGCATCTC	59.969	118
TRINITY_Contig10105	1	p3	(CCA)6	18	132	149	ACGATGCCGACAGGATCATC	59.967	TGATGCACCCGTCTCTGTTC	60.037	189
TRINITY_Contig10108	1	c	(TCGCGG)3ctcctcctctccttgtccttatccctctcccgctcttttctccttgtcatcacggtcccggtcccgccgctcccgactgctgcggtgccgg(TCCCGC)3	126	1119	1244	CAAGCCTCCACCTTCATCGT	60.036	CACGCCTTGAAGACGCAAAA	59.971	155
TRINITY_Contig10110	1	p3	(CGG)4	12	1344	1355	ATTTCCCATGGGGTTGCCAT	59.955	TGGTCACGGGTTCAAGATGG	59.964	145
TRINITY_Contig10113	1	p3	(TCC)4	12	122	133	TGGGCATGTACCGCAAGATT	60.035	GTATTGGGCTCCGGATCCAG	59.965	106
TRINITY_Contig10113	2	p6	(GCGCAC)3	18	530	547	TGGGCATGTACCGCAAGATT	60.035	GTATTGGGCTCCGGATCCAG	59.965	106
TRINITY_Contig10113	3	p2	(AG)8	16	1469	1484	TGGGCATGTACCGCAAGATT	60.035	GTATTGGGCTCCGGATCCAG	59.965	106
TRINITY_Contig10115	1	c	(TCCC)3cagtcccccggcattttcttcgcctcctcg(CGC)4	54	58	111	TGCACCGGTACTGCATTGAT	60.036	GTACCTCCACAGCAGGAACC	60.036	274
TRINITY_Contig10116	1	c	(GCG)4gggggccacaacggccggcgcaggagcaacggcgcggggacgcggcagcacctccgtttcgccgctagagttctccgt(GGC)6	108	1411	1518	TTCTTCTGTCCGTGGCTGTC	59.966	AGCTGCCTCAGGGGAAATTC	60.034	253
TRINITY_Contig10117	1	p3	(CGC)4	12	108	119	TTGGCTCCAAAGTGGGATCC	59.96	TCCCGGTCCATGGAAGATCT	60.031	160
TRINITY_Contig10119	1	p3	(GGC)5	15	237	251	GCGATTCCGAACGTCATTGG	59.974	GCCAAGGGTTACAGACAGCT	59.963	195

TRINITY_Contig10120	1	p4	(ATCC)3	12	452	463	TTTCACCTGGTGCTACCGTC	59.966	CTGTCCACCCACTGAAGTCC	59.965	272
TRINITY_Contig10123	1	p3	(TGA)4	12	40	51	TGCGTGCATCCAACAAACAG	59.969	AGGAGAAGGAGGTTGTTGCG	59.965	192
TRINITY_Contig10123	2	p4	(CACC)3	12	199	210	TGCGTGCATCCAACAAACAG	59.969	AGGAGAAGGAGGTTGTTGCG	59.965	192
TRINITY_Contig10123	3	p3	(GCG)5	15	1062	1076	TGCGTGCATCCAACAAACAG	59.969	AGGAGAAGGAGGTTGTTGCG	59.965	192
TRINITY_Contig10124	1	p3	(GCG)6	18	1102	1119	GCCAGGTTCCTGTTCTCCTC	60.036	TCTTCCACCACGACTGCTTC	59.966	133
TRINITY_Contig10126	1	p5	(TCCTC)4	20	70	89	AGTGAGGAGTGGCTGAAACG	59.966	GCTGCTTCTCAGGGCTTGTA	60.036	246
TRINITY_Contig10127	1	c	(CT)14cg(CT)10	50	21	70	TAGTTCATCTCGCTCGCCAC	59.899	ATGAGTCCAGCTTCCCTTGC	60.035	276
TRINITY_Contig10127	2	p3	(GCG)4	12	317	328	TAGTTCATCTCGCTCGCCAC	59.899	ATGAGTCCAGCTTCCCTTGC	60.035	276
TRINITY_Contig10128	1	c	(GCT)7tct(GCG)4	36	1345	1380	TGATCCTCTTCTCCCCTGGG	60.031	TATGCCTGAACCCGTTTGCT	59.962	159
TRINITY_Contig10128	2	p3	(GAG)4	12	1532	1543	TGATCCTCTTCTCCCCTGGG	60.031	TATGCCTGAACCCGTTTGCT	59.962	159
TRINITY_Contig10130	1	p3	(GTT)7	21	1423	1443	CTCGGACACCTCGCATACTC	59.97	GGTGGACTTGTCGAAGAGCA	59.966	270
TRINITY_Contig10132	1	p3	(CGC)6	18	1223	1240	CACTGTTGACGCCAAAGGTG	59.971	TGCGCTCAAGCCTAAGAACA	59.965	118
TRINITY_Contig10134	1	p3	(GCG)4	12	1077	1088	ATGCCGCAATACAAATCGCC	59.969	TCTTGCAATGAGCTCTCGCA	60.037	254
TRINITY_Contig10134	2	c*	(CGGC)2(<CGGC>GGCGGCGGATGAGCATTAAAAAAGGAGGAGAGGCCAGGCTTGGGACGATCAGTTGCGTACGGACCACGGGGGGATACAGCATCCAGGCTGAATGATTGAACGAATGGGTGGTGCGTGCCATGTCATGAAGACATGAACTCTTTTTTTGCGAGGATGAAGACATGAACTCGGCGTGCCAGCTGCCGCATTGCGGCGGGGAACAGAACACAAGAGGAGGTCGCGCGGCGAGAGGATGCAGCATGGCCGCCGCGCGACCTCCTCTTG)(CGG)3	20	1279	1298	ATGCCGCAATACAAATCGCC	59.969	TCTTGCAATGAGCTCTCGCA	60.037	254
TRINITY_Contig10135	1	p6	(GCGGAG)3	18	512	529	CCAGCTATCCCGTGTCGTAC	59.97	TCAGGCTCATCATCCCCTGA	60.031	209
TRINITY_Contig10135	2	p4	(GCCG)3	12	729	740	CCAGCTATCCCGTGTCGTAC	59.97	TCAGGCTCATCATCCCCTGA	60.031	209
TRINITY_Contig10137	1	p2	(TC)6	12	43	54	GGGCTACTGCTTCGAGATCC	59.967	AGCACTACCCATCATCCCCT	60.03	109
TRINITY_Contig10137	2	p4	(GCCA)3	12	794	805	GGGCTACTGCTTCGAGATCC	59.967	AGCACTACCCATCATCCCCT	60.03	109
TRINITY_Contig10138	1	p4	(ATCC)3	12	527	538	CTCTCACTGTTCGTGGCACT	59.967	GAAGCAGTCGTGGAAGTGGA	59.966	183
TRINITY_Contig10139	1	c	(CGAGC)4cgaaccgagct(GAGCC)3gatcaaatagatctagcagggcagcaaaacagggagacggcaacacgcgacgcggacgatggcgacggcgattccaacggccacgacaaggaaatca(CGG)4caggacggcggcaaacgacggcagcggcggccggtggatggatgaaggccggcgaagcgct(CGG)6	234	2123	2356	AAAACAGGGAGACGGCAACA	60.107	TCGTTCTTGGACAGTGGAGC	59.966	237
TRINITY_Contig10140	1	p2	(CT)15	30	28	57	CTGGTACCTCCGGATCAACG	59.898	CCAGCGGTCGTAGTACCATC	59.97	255
TRINITY_Contig10140	2	p3	(CAG)6	18	1201	1218	CTGGTACCTCCGGATCAACG	59.898	CCAGCGGTCGTAGTACCATC	59.97	255
TRINITY_Contig10141	1	c	(GCC)4gctctgaacaaacg(TTCT)3	38	367	404	GATCGGAGTGCAGGTTTCGA	60.109	GCACCCCAACGAAAAATGCT	59.967	245
TRINITY_Contig10141	2	p4	(GATC)3	12	1095	1106	GATCGGAGTGCAGGTTTCGA	60.109	GCACCCCAACGAAAAATGCT	59.967	245
TRINITY_Contig10144	1	p3	(GCC)6	18	1835	1852	CAGATCCTGATGCGAGGACC	59.967	TCCTGGACCAGAGCTACCTC	60.033	203
TRINITY_Contig10146	1	p3	(GCG)5	15	294	308	GCCTCTGCTAGCGATGGAAT	59.965	CATGCGCTGTCTTGCAAAGT	60.04	223
TRINITY_Contig10146	2	p3	(GGC)6	18	413	430	GCCTCTGCTAGCGATGGAAT	59.965	CATGCGCTGTCTTGCAAAGT	60.04	223
TRINITY_Contig10146	3	c	(GCC)4atggtggcggtggcggtgatggtggttcttggcttggccttcgcccctggagcagcaggaggttgcctgactttg(GGC)4	99	758	856	GCCTCTGCTAGCGATGGAAT	59.965	CATGCGCTGTCTTGCAAAGT	60.04	223
TRINITY_Contig10146	4	p5	(ATCAG)3	15	1435	1449	GCCTCTGCTAGCGATGGAAT	59.965	CATGCGCTGTCTTGCAAAGT	60.04	223
TRINITY_Contig10147	1	p2	(CG)6	12	144	155	GGGACTGCCGTATACAGCTC	59.968	GCTTTGTGTGCTGTCAACGT	59.901	175
TRINITY_Contig10149	1	p4	(CTAG)3	12	222	233	GAGGCCCATTCATCCAACCA	60.033	GTCGTAGAACTGCGGGAACA	60.039	257
TRINITY_Contig10150	1	p3	(CTG)5	15	309	323	AGCTGAATGGGCATGAGCTT	60.033	CCCGGACCTCGAAAACTTGA	59.966	241
TRINITY_Contig10150	2	p3	(TGC)4	12	1269	1280	AGCTGAATGGGCATGAGCTT	60.033	CCCGGACCTCGAAAACTTGA	59.966	241
TRINITY_Contig10152	1	p3	(CGC)4	12	99	110	CTCGATTCCATCATGCGGGA	59.966	AGGCAGAAGTGGTGGAATCG	60.036	144
TRINITY_Contig10152	2	p4	(AGGG)3	12	232	243	CTCGATTCCATCATGCGGGA	59.966	AGGCAGAAGTGGTGGAATCG	60.036	144
TRINITY_Contig10152	3	p3	(AGC)4	12	1532	1543	CTCGATTCCATCATGCGGGA	59.966	AGGCAGAAGTGGTGGAATCG	60.036	144
TRINITY_Contig10153	1	c	(CGC)4agccgtcgcccgccaagt(CTC)4	42	271	312	CGATCAGACCAGATCACCCG	59.969	TCGGAGGAGGAGGAGACTTG	60.034	209
TRINITY_Contig10153	2	p3	(CGC)4	12	505	516	CGATCAGACCAGATCACCCG	59.969	TCGGAGGAGGAGGAGACTTG	60.034	209
TRINITY_Contig10154	1	p3	(CTG)6	18	281	298	ATTGAAGGCCTCGGGTCAAG	60.035	GGTAGTAGGGGTAGTGGGCA	60.032	248
TRINITY_Contig10155	1	p5	(GAAAC)3	15	71	85	GATGGTGTAGAGCGAGGTGG	59.897	ACCGTCGTCTTCAGCAATGT	59.966	181
TRINITY_Contig10155	2	p3	(CAG)4	12	1382	1393	GATGGTGTAGAGCGAGGTGG	59.897	ACCGTCGTCTTCAGCAATGT	59.966	181
TRINITY_Contig10156	1	p3	(TGA)4	12	43	54	GACCAGCAACCCCGTTGATA	60.036	TGAGGAGCCATTGCCAGAAG	60.035	134
TRINITY_Contig10156	2	p4	(CAAG)3	12	1001	1012	GACCAGCAACCCCGTTGATA	60.036	TGAGGAGCCATTGCCAGAAG	60.035	134
TRINITY_Contig10158	1	p3	(CGC)4	12	620	631	CTCTCCAGCCGCACGATATT	59.967	CGGGGTTGAAAGGGCAAAAG	59.967	143
TRINITY_Contig10158	2	p3	(CGC)4	12	1790	1801	CTCTCCAGCCGCACGATATT	59.967	CGGGGTTGAAAGGGCAAAAG	59.967	143
TRINITY_Contig10160	1	p3	(GGC)4	12	1275	1286	GTTGTCCTCCTTGAAGGCGA	59.965	AGTCATCGCACCGCATCTAG	59.969	156
TRINITY_Contig10162	1	p3	(GCG)4	12	1071	1082	TGCTTCTTCTGCTCGCTCAA	59.966	TGAGGTTGACGACGAGTTGG	59.968	154
TRINITY_Contig10165	1	c	(CCCAC)3cagcccccgcaattcgcgcagattgcagaagcgcccctcgc(CGG)4	68	153	220	TTGCATTTTGCCGCACATGA	59.967	CTGCATGCAATCTACGTGGC	59.971	264
TRINITY_Contig10166	1	p3	(CTT)6	18	313	330	GAGGTCGGTGATGTTGACGT	60.039	ACGAGAAGAAGCTGCACTCC	60.038	110
TRINITY_Contig10167	1	c	(CGG)5cgctggtctccggaggccatggagcccgccgccgcctcctcgccgtcgcggacggcggcggcaggcaccaccgacgcctaggggtgggcgagc(GGCG)3	120	330	449	ACTCGATCGCTCGCGTATTT	59.971	TTTTTGCCTTCCATGCACCG	59.967	132
TRINITY_Contig10167	2	p3	(GCG)4	12	722	733	ACTCGATCGCTCGCGTATTT	59.971	TTTTTGCCTTCCATGCACCG	59.967	132
TRINITY_Contig10167	3	p3	(CGG)4	12	1246	1257	ACTCGATCGCTCGCGTATTT	59.971	TTTTTGCCTTCCATGCACCG	59.967	132
TRINITY_Contig10169	1	p6	(AAGGCC)3	18	491	508	AAGCGGAAAAGGTCCTCAGG	59.963	AATCACAAATCATGGCCGCG	59.899	210
TRINITY_Contig10169	2	p3	(GTC)6	18	1081	1098	AAGCGGAAAAGGTCCTCAGG	59.963	AATCACAAATCATGGCCGCG	59.899	210
TRINITY_Contig10171	1	c	(GTT)8(GCT)4	36	1070	1105	CTGCTGGTAAGTGAGTGGGG	60.036	TGAGACAAGCCAAGGAGCAG	59.964	236
TRINITY_Contig10171	2	p3	(GCT)5	15	1241	1255	CTGCTGGTAAGTGAGTGGGG	60.036	TGAGACAAGCCAAGGAGCAG	59.964	236
TRINITY_Contig10172	1	p5	(GCGCC)3	15	1475	1489	TTGAGACTTGCGGACCTCAC	59.966	GCAAGGAGGGCCTGAATCTT	60.034	139
TRINITY_Contig10173	1	p5	(CGCCG)3	15	66	80	CTTGTCCACCACCACCAGTT	60.107	AGACTCAGCTGCGGTTATCG	59.899	227
TRINITY_Contig10173	2	p3	(CCG)5	15	193	207	CTTGTCCACCACCACCAGTT	60.107	AGACTCAGCTGCGGTTATCG	59.899	227
TRINITY_Contig10173	3	p3	(CGC)6	18	338	355	CTTGTCCACCACCACCAGTT	60.107	AGACTCAGCTGCGGTTATCG	59.899	227
TRINITY_Contig10173	4	p3	(CCA)4	12	688	699	CTTGTCCACCACCACCAGTT	60.107	AGACTCAGCTGCGGTTATCG	59.899	227
TRINITY_Contig10175	1	p4	(GGGT)3	12	83	94	GTGGTTTTCAAAGACCGGGC	59.968	ATCCAAACACGGCAACAAGC	59.968	264
TRINITY_Contig10176	1	p3	(GAG)5	15	571	585	CCACATCCCTCACCAGCATT	60.034	TCCTTCAGTGTGCCGTTCTC	59.966	155
TRINITY_Contig10176	2	p3	(GGC)4	12	788	799	CCACATCCCTCACCAGCATT	60.034	TCCTTCAGTGTGCCGTTCTC	59.966	155
TRINITY_Contig10176	3	p3	(CCG)5	15	1279	1293	CCACATCCCTCACCAGCATT	60.034	TCCTTCAGTGTGCCGTTCTC	59.966	155
TRINITY_Contig10180	1	c	(CCCG)3ccgccgccattgaccgaggtaccgccgccg(CCT)5cgcgccacaaggggggttccgatctgccgccattcccgtgccctgctcgtgccgc(CCCT)3	124	276	399	CGGCCTGTTCCACCATAAGT	60.035	GGGCCTACATGGAGCACTTT	60.034	231
TRINITY_Contig10181	1	p3	(GCT)4	12	597	608	ACCTGACCAAGAGATGCAGC	60.036	CTGGAGGAGGAGGAGATGCT	60.106	193
TRINITY_Contig10181	2	p6	(GGCGCC)3	18	936	953	ACCTGACCAAGAGATGCAGC	60.036	CTGGAGGAGGAGGAGATGCT	60.106	193
TRINITY_Contig10181	3	p3	(CGC)4	12	1120	1131	ACCTGACCAAGAGATGCAGC	60.036	CTGGAGGAGGAGGAGATGCT	60.106	193
TRINITY_Contig10181	4	c	(CTC)4cagctgccgctgctcgttgcccagtctgccccgggcaacgcgctgctcatctcgtgcactaaaccccgacgcccttt(TGAGC)3	103	1314	1416	ACCTGACCAAGAGATGCAGC	60.036	CTGGAGGAGGAGGAGATGCT	60.106	193
TRINITY_Contig10182	1	p3	(GGT)5	15	2354	2368	CAGCGCATGGAGGTAATTGC	59.97	CCCCAAAATCTTCCGACGGA	60.036	203
TRINITY_Contig10184	1	p3	(TGC)4	12	497	508	GAGAAGAAGGTCGTGCCGAA	60.039	TTCGTTCGCCTTTGCCTTTG	59.97	278
TRINITY_Contig10184	2	p3	(CGT)4	12	1679	1690	GAGAAGAAGGTCGTGCCGAA	60.039	TTCGTTCGCCTTTGCCTTTG	59.97	278

TRINITY_Contig10185	1	c*	(GCC)4(GCCCCGCCCCGCCGCGTAGTCATCTGCGGCGGGGGCGTGGTGGGCGCGTGCACGGCCTACTTCCTCTCCACCCACGCGGCGTCGCCCACCGTCCCGACCCTCGTCGAGAGGTGCGCCCCGGCGTGCGCCGCCTCGGGCAAAGCCGGCGGCTTCCTGGCCCTCGACTGGTGCGACTCCACCCCGGGGCTCTCCGCGCTCGCGCGGGCCTCCTACGCGCTCCACCGCCGCCTCGCCGCGGACCTCGGCGGCGCCGGCGCCTACGGCTACCGCCCCGTCCACACCCTCTCCGTCTGCGTCCCCTCCGCTGCTCCCAAGCCCGCCTCGCCGCACCCGACGCTCCCGGCCTGGGTCGACCCGTCGGCGTCCGCGGCCCCGCCGCGGGAGCTCGGCACCCCGGCCACCACCGCGCAGGTCCACCCGGGCCTCTTCACCAAGG	25	139	163	TGTCCAGCCAGCTGAAGAAG	59.964	GAGAAACCTTGCCGGACTGA	59.965	251
TRINITY_Contig10187	1	c	(CCG)5ccccccttcgacgactccgccctcgcccgcgactcccccacctcccacctgtccttccactgcgcc(GAG)4	92	521	612	ACGCTACAACTACACGTCGG	60.11	TCGGGTTTCTGACGATGTCG	60.109	235
TRINITY_Contig10190	1	p4	(AAAC)3	12	5	16	AAGCTGTGTCTCCAATGGGG	59.961	TCCATTCCTGGAGAGTCGGT	59.959	172
TRINITY_Contig10190	2	p3	(CGG)4	12	1505	1516	AAGCTGTGTCTCCAATGGGG	59.961	TCCATTCCTGGAGAGTCGGT	59.959	172
TRINITY_Contig10194	1	p3	(CCG)4	12	431	442	TCTCTTTGTTGATCCCGGGC	60.035	CATCACCACTCACCCTCCAC	60.036	121
TRINITY_Contig10194	2	p6	(CGGGAA)3	18	883	900	TCTCTTTGTTGATCCCGGGC	60.035	CATCACCACTCACCCTCCAC	60.036	121
TRINITY_Contig10196	1	p4	(CAAC)3	12	192	203	CCCCCTCCGCTTAGCTAAAC	60.179	TGAGCTGGTGGTGGTGTTTT	60.034	160
TRINITY_Contig10196	2	p3	(CGG)4	12	1304	1315	CCCCCTCCGCTTAGCTAAAC	60.179	TGAGCTGGTGGTGGTGTTTT	60.034	160
TRINITY_Contig10202	1	p3	(CCG)5	15	1404	1418	GGTTCTGTACAGAGGCCACC	60.036	CAGCAGGAAAGAGCACAGGA	59.964	120
TRINITY_Contig10203	1	p3	(CGC)4	12	157	168	TCTCCGCGCGAGACTAAATC	59.971	TCAATGCCTCCGGTATACGC	59.967	256
TRINITY_Contig10203	2	c	(GCG)5cggcgc(GGAG)3	33	300	332	TCTCCGCGCGAGACTAAATC	59.971	TCAATGCCTCCGGTATACGC	59.967	256
TRINITY_Contig10203	3	p3	(GCC)4	12	1203	1214	TCTCCGCGCGAGACTAAATC	59.971	TCAATGCCTCCGGTATACGC	59.967	256
TRINITY_Contig10203	4	p4	(CATT)4	16	2088	2103	TCTCCGCGCGAGACTAAATC	59.971	TCAATGCCTCCGGTATACGC	59.967	256
TRINITY_Contig10204	1	c	(AAAT)3aaaaacagcagcaacaaatggcacagatcgacaagta(GC)6	61	95	155	GCAGCACACAAAACACACCA	60.109	ACGTTCGTCCTCGTGTACAC	60.041	154
TRINITY_Contig10204	2	p3	(TTA)4	12	668	679	GCAGCACACAAAACACACCA	60.109	ACGTTCGTCCTCGTGTACAC	60.041	154
TRINITY_Contig10207	1	p3	(AAC)4	12	607	618	TCGACAAGTTCCCAGAAGCC	59.965	AACTTCGTGACGACGCTCTT	59.97	139
TRINITY_Contig10212	1	c	(ATCG)3attattgatcccttttaccgtgcatttgtgctgc(TGCG)5	66	1176	1241	TCAAGGACGACCAGTTCAGC	59.966	CGAGCATGGAGAGGCTGTAG	59.968	231
TRINITY_Contig10214	1	p3	(GCC)4	12	1448	1459	ACACATTTCTAGGGGCTGGC	60.034	GGTCTCGAAAGGTTGCAGGA	59.965	139
TRINITY_Contig10216	1	p4	(GCTA)3	12	3144	3155	TCCTGCGTTTGGTAGTCCAC	59.966	GGTACGCACAAGGTGGAGGA	59.966	224
TRINITY_Contig10216	2	p3	(GGT)4	12	3372	3383	TCCTGCGTTTGGTAGTCCAC	59.966	GGTACGCACAAGGTGGAGGA	59.966	224
TRINITY_Contig10217	1	p2	(AG)7	14	200	213	CGGTGATAACGATGGGAGGG	59.966	TAGGACGTCCCGGCTAAAGA	60.034	228
TRINITY_Contig10217	2	p3	(CGC)4	12	567	578	CGGTGATAACGATGGGAGGG	59.966	TAGGACGTCCCGGCTAAAGA	60.034	228
TRINITY_Contig10217	3	p3	(CCG)4	12	974	985	CGGTGATAACGATGGGAGGG	59.966	TAGGACGTCCCGGCTAAAGA	60.034	228
TRINITY_Contig10217	4	p3	(TGG)5	15	1240	1254	CGGTGATAACGATGGGAGGG	59.966	TAGGACGTCCCGGCTAAAGA	60.034	228
TRINITY_Contig10219	1	p3	(AGA)4	12	1865	1876	TTGGGCAGCATCACCATCAT	60.033	TTCGTCCTCGTTCTCCTTGC	60.039	239
TRINITY_Contig10222	1	p3	(GCG)6	18	915	932	TCTCCGACGAGGTCATCAGT	60.035	ATTGGTGCTGCGAGATGTGA	60.036	276
TRINITY_Contig10224	1	p3	(CCT)4	12	137	148	GAAGGCGAGGAGGAAGAAGG	59.823	AGTCCAGCCCGTTCTTCTTG	59.965	203
TRINITY_Contig10224	2	c	(CGG)4gtccgagtcgacggagtcgtccgcg(GGC)4	49	649	697	GAAGGCGAGGAGGAAGAAGG	59.823	AGTCCAGCCCGTTCTTCTTG	59.965	203
TRINITY_Contig10224	3	p4	(ATGG)3	12	1297	1308	GAAGGCGAGGAGGAAGAAGG	59.823	AGTCCAGCCCGTTCTTCTTG	59.965	203
TRINITY_Contig10225	1	c	(GCG)6gca(TCC)5	36	98	133	ACAAGTCACAGAGCTCACGG	59.967	CATGCACAACAGCGATAGGC	59.971	224
TRINITY_Contig10225	2	p5	(GAAAT)3	15	3229	3243	ACAAGTCACAGAGCTCACGG	59.967	CATGCACAACAGCGATAGGC	59.971	224
TRINITY_Contig10226	1	p3	(CGA)4	12	523	534	GGAGGAGGAGAAGGCCAGTA	60.032	ATCCCACCTACCACCTCTCC	60.031	249
TRINITY_Contig10226	2	p3	(GAC)5	15	779	793	GGAGGAGGAGAAGGCCAGTA	60.032	ATCCCACCTACCACCTCTCC	60.031	249
TRINITY_Contig10226	3	p3	(GAG)5	15	1304	1318	GGAGGAGGAGAAGGCCAGTA	60.032	ATCCCACCTACCACCTCTCC	60.031	249
TRINITY_Contig10227	1	p4	(GAGG)4	16	1379	1394	AGTTGTTTCGCGTCTGGGAT	59.965	TAGCTCTCTCGCCCTTGAGT	60.034	121
TRINITY_Contig10230	1	p3	(GCC)4	12	219	230	TCAGATCCCCGTCAATCCCT	60.031	TCGGAGAAGGGAAGACCGAT	60.033	187
TRINITY_Contig10231	1	p4	(CGGC)3	12	80	91	TCTTCCGACAGCAGTGGAAC	59.966	GCTTCTGGGGTTCATCTCCC	60.107	258
TRINITY_Contig10231	2	c	(CGTCC)4ggcccgtaccgttacaccacgagacgacgttgacgttacagcgtgggatcaaagaggcagcagccgcatgcgcctacacagcg(CCCGGC)3	121	270	390	TCTTCCGACAGCAGTGGAAC	59.966	GCTTCTGGGGTTCATCTCCC	60.107	258
TRINITY_Contig10231	3	p6	(TCGGGG)3	18	693	710	TCTTCCGACAGCAGTGGAAC	59.966	GCTTCTGGGGTTCATCTCCC	60.107	258
TRINITY_Contig10232	1	p3	(CGC)5	15	312	326	TCATATGACGCTCGGAAGGC	59.968	TAGCTGGTTGGATGCACCTG	60.035	198
TRINITY_Contig10233	1	p4	(CTGC)3	12	229	240	AACACCTATGGCTTCCGCAA	59.962	GGGGTCGTCGTTCTTCTTGT	59.967	156
TRINITY_Contig10233	2	p3	(CGC)4	12	863	874	AACACCTATGGCTTCCGCAA	59.962	GGGGTCGTCGTTCTTCTTGT	59.967	156
TRINITY_Contig10233	3	p3	(GCG)4	12	982	993	AACACCTATGGCTTCCGCAA	59.962	GGGGTCGTCGTTCTTCTTGT	59.967	156
TRINITY_Contig10234	1	p2	(AC)11	22	106	127	GGTTCGCGACGACATCTTTG	59.907	GGACTCCTTCAGCAACACGA	59.966	118
TRINITY_Contig10234	2	p3	(TCG)4	12	701	712	GGTTCGCGACGACATCTTTG	59.907	GGACTCCTTCAGCAACACGA	59.966	118
TRINITY_Contig10234	3	p3	(GTA)4	12	1408	1419	GGTTCGCGACGACATCTTTG	59.907	GGACTCCTTCAGCAACACGA	59.966	118
TRINITY_Contig10235	1	p4	(GCCT)3	12	399	410	CATCACATCGCTCGCACTTG	59.974	CGCCCCTAGCAGAAAGGAAA	60.035	211
TRINITY_Contig10235	2	p3	(CGG)5	15	1231	1245	CATCACATCGCTCGCACTTG	59.974	CGCCCCTAGCAGAAAGGAAA	60.035	211
TRINITY_Contig10237	1	c	(GA)10aaagaaaaatcccaatccccgccgccgcgagatcgccgggcgccgccatgtccactccagccgccgcctactt(CGC)4gcgcaagcagtacttctgctaccaatgcaaccgcaccgtcctcatcgcggcct(CCG)5	173	63	235	TGTTGGAATTGCTGCGGTTG	59.968	CCCATTGTTCCCATCCTGCT	60.033	166
TRINITY_Contig10245	1	p3	(GCC)4	12	396	407	ATCTCCTGGGTCCCTTCCTC	60.031	CCGGATTCACCTCTTCCTCG	59.897	196
TRINITY_Contig10246	1	p5	(CCCTT)3	15	1330	1344	AAGTACAGCCACCACGAGTG	59.966	GTCTTGTCAAGGAGCACGGA	59.966	125
TRINITY_Contig10247	1	p3	(AGC)4	12	172	183	AACTCAAGCTGGGTCTCTGC	59.964	TGCAAACTCTCCCGGAACTC	59.965	220
TRINITY_Contig10247	2	p3	(TGA)4	12	801	812	AACTCAAGCTGGGTCTCTGC	59.964	TGCAAACTCTCCCGGAACTC	59.965	220
TRINITY_Contig10247	3	p3	(AGC)6	18	1018	1035	AACTCAAGCTGGGTCTCTGC	59.964	TGCAAACTCTCCCGGAACTC	59.965	220
TRINITY_Contig10247	4	p4	(TTGG)3	12	1318	1329	AACTCAAGCTGGGTCTCTGC	59.964	TGCAAACTCTCCCGGAACTC	59.965	220
TRINITY_Contig10248	1	p3	(GAC)5	15	261	275	ATTGTCGCCGATGAAGGTGT	60.036	CATTGGCATCAGGCTGCATC	59.968	253
TRINITY_Contig10249	1	p4	(CGAG)4	16	1396	1411	GCCTACCCATTGCTCACCTT	60.034	TGGTGCTCTGATCACTGCTG	60.037	274
TRINITY_Contig10250	1	p6	(GCCTCC)3	18	112	129	CGGATGGTCGAGATGTCCTG	59.969	CAGCTCGGTGGAGTTCTTGT	59.966	222
TRINITY_Contig10250	2	p3	(GAA)5	15	1319	1333	CGGATGGTCGAGATGTCCTG	59.969	CAGCTCGGTGGAGTTCTTGT	59.966	222
TRINITY_Contig10250	3	p3	(TTC)5	15	1574	1588	CGGATGGTCGAGATGTCCTG	59.969	CAGCTCGGTGGAGTTCTTGT	59.966	222
TRINITY_Contig10250	4	p4	(AGCA)3	12	1699	1710	CGGATGGTCGAGATGTCCTG	59.969	CAGCTCGGTGGAGTTCTTGT	59.966	222
TRINITY_Contig10250	5	p6	(GGAGGC)3	18	2778	2795	CGGATGGTCGAGATGTCCTG	59.969	CAGCTCGGTGGAGTTCTTGT	59.966	222
TRINITY_Contig10251	1	p2	(CT)8	16	242	257	GCTTCTTCTCCCTCGATCCG	59.969	ACTTCGGCTGATCGATCGAC	59.97	105
TRINITY_Contig10251	2	p3	(CTA)4	12	412	423	GCTTCTTCTCCCTCGATCCG	59.969	ACTTCGGCTGATCGATCGAC	59.97	105
TRINITY_Contig10251	3	c	(TTG)4ttaccttgttgaggtggtggatggcggatcggtcgatcgatcagccgaagttgtagccattgctgttgctcgtcgtcgagga(CGGG)3	106	632	737	GCTTCTTCTCCCTCGATCCG	59.969	ACTTCGGCTGATCGATCGAC	59.97	105
TRINITY_Contig10251	4	p3	(CTG)4	12	933	944	GCTTCTTCTCCCTCGATCCG	59.969	ACTTCGGCTGATCGATCGAC	59.97	105
TRINITY_Contig10251	5	c	(CCG)4cggcggcagtcaccgcggtcgactccgtccccgccggcgcgccgatc(CCG)5	74	1289	1362	GCTTCTTCTCCCTCGATCCG	59.969	ACTTCGGCTGATCGATCGAC	59.97	105
TRINITY_Contig10251	6	p3	(TGG)4	12	1484	1495	GCTTCTTCTCCCTCGATCCG	59.969	ACTTCGGCTGATCGATCGAC	59.97	105
TRINITY_Contig10252	1	p4	(CCTC)3	12	356	367	CGCTTTGCTGAGGCTTTTGT	59.969	CACTCCTTCCTTCAGGGCAG	60.036	111
TRINITY_Contig10252	2	p2	(GT)6	12	1373	1384	CGCTTTGCTGAGGCTTTTGT	59.969	CACTCCTTCCTTCAGGGCAG	60.036	111
TRINITY_Contig10254	1	p3	(GCT)4	12	863	874	TGGCGATGAACACGTTCTGA	59.967	TCAGAACAATGCGTCGACGA	60.04	118
TRINITY_Contig10258	1	p3	(CGG)6	18	1346	1363	AGTGACGCGTCAACACTGAT	59.968	TGCTGTGAAGACTCGCATGT	59.966	252
TRINITY_Contig10259	1	p3	(GGC)4	12	157	168	CCACTACTTCCTGTGGCTGG	60.036	ATGAGGTGAGGCTCGACTCT	60.034	181
TRINITY_Contig10260	1	p5	(CGGAT)3	15	1164	1178	CAAAAGACGGGCATGCCAAA	59.967	GCGAGGAAACCAGGAACGTA	60.038	233
TRINITY_Contig10260	2	p6	(GAGAAT)3	18	1849	1866	CAAAAGACGGGCATGCCAAA	59.967	GCGAGGAAACCAGGAACGTA	60.038	233
TRINITY_Contig10262	1	p3	(GGC)5	15	174	188	CTTCCCCTACCTCTCGTCCA	60.033	GAGGGAATGTTGGGACCTGG	60.034	234
TRINITY_Contig10262	2	c	(GCT)4(GCGGGG)4acaggggctgggggcggggccgcggccgggggt(GGAGCC)3ggagcgcggacgggagcaggagccggcacctgcggcggcacttccgccgggcgctggcgcc(GCG)4	160	3416	3575	CTTCCCCTACCTCTCGTCCA	60.033	GAGGGAATGTTGGGACCTGG	60.034	234
TRINITY_Contig10263	1	p3	(CGC)5	15	102	116	CGTCCTCCAAGCTGGTCTTT	59.965	ACACTCTCATCCTGCTTGCC	60.036	199

TRINITY_Contig10264	1	p3	(CGC)5	15	1306	1320	TAGGCAACAGCAGCAATGGA	59.962	TTGATGCTGCAGTTGTTGGC	59.968	187
TRINITY_Contig10265	1	p4	(CCCT)3	12	97	108	GACGGCGGATCAAGATCAT	59.966	GAGACGACGAAGGCCATCAT	59.897	134
TRINITY_Contig10265	2	p3	(ACC)4	12	948	959	GACGGCGGATCAAGATCAT	59.966	GAGACGACGAAGGCCATCAT	59.897	134
TRINITY_Contig10267	1	p3	(AAG)4	12	75	86	ATCGAACCCGTGGGAGTAGA	60.034	TGCAACGTGAAATGGCTTGG	59.968	210
TRINITY_Contig10267	2	p4	(AAAC)3	12	200	211	ATCGAACCCGTGGGAGTAGA	60.034	TGCAACGTGAAATGGCTTGG	59.968	210
TRINITY_Contig10268	1	p3	(GCT)4	12	1332	1343	TCTCTAGTCGCTGTAGCCGT	60.108	TGGTAGCTACCCGTCGTGTA	60.036	156
TRINITY_Contig10270	1	c	(CT)25gaatcttggatttctgatgcggtgcagtgcgcgcatggatcggcggccggagcgcaaggcgaagcgcgcgag(GGA)5	137	540	676	ACACCCTCTCTCCATCTCCC	60.032	ATGTCGGTCACGTACGATCG	59.973	227
TRINITY_Contig10270	2	p4	(GGAC)3	12	1077	1088	ACACCCTCTCTCCATCTCCC	60.032	ATGTCGGTCACGTACGATCG	59.973	227
TRINITY_Contig10273	1	p4	(GGAG)3	12	1278	1289	TCAGGACCCTCATCGTCACT	59.96	GCCTCACAAGCTACCACTGT	59.965	131
TRINITY_Contig10274	1	c	(TGC)6tcatcctcctcctcttggtcgttgtcgtcg(TCA)4	60	2285	2344	TCTTATCAAATTGCGCCGCG	59.973	CTGCTCCTCGGTTGATTGGT	60.036	116
TRINITY_Contig10274	2	p3	(CGC)6	18	2746	2763	TCTTATCAAATTGCGCCGCG	59.973	CTGCTCCTCGGTTGATTGGT	60.036	116
TRINITY_Contig10274	3	c	(AGTGG)3tttctcctttttaccggtagcgaagggcgggggcggggcg(GGACA)3	70	3510	3579	TCTTATCAAATTGCGCCGCG	59.973	CTGCTCCTCGGTTGATTGGT	60.036	116
TRINITY_Contig10275	1	p3	(CCG)7	21	1533	1553	ACTTCTCGGTCTCCTCGACA	59.964	AACCGCTGGTACGTGCTTAA	59.966	220
TRINITY_Contig10275	2	p3	(TGG)4	12	1923	1934	ACTTCTCGGTCTCCTCGACA	59.964	AACCGCTGGTACGTGCTTAA	59.966	220
TRINITY_Contig10280	1	p3	(CGC)4	12	641	652	AGTCTGGGCGGATCAACAAG	60.036	TGCATTCGCACTTGATCCCT	60.035	125
TRINITY_Contig10281	1	p3	(GCT)5	15	1106	1120	GCTCCTCTGCTCTGCTCTTC	60.179	TTCGCGTTCGATCAGAGCTT	60.109	124
TRINITY_Contig10281	2	p4	(CGCC)3	12	1305	1316	GCTCCTCTGCTCTGCTCTTC	60.179	TTCGCGTTCGATCAGAGCTT	60.109	124
TRINITY_Contig10282	1	c	(ACC)4g(CGC)4	25	416	440	CTCCTCGACGTATTCTGGGC	59.969	TCCTGCTCCGGGGTGTAATA	60.032	110
TRINITY_Contig10284	1	p3	(GCT)4	12	2177	2188	GCAGAAGCAGGTGAGTCCTT	59.964	CATGGTGATGTGGGACGTGA	60.036	140
TRINITY_Contig10285	1	p4	(CTAG)3	12	1253	1264	TCAGCCTCACCAAGAACGTC	59.966	GCCGAAGAGCTTGGAGAACT	60.037	107
TRINITY_Contig10286	1	p3	(GCC)5	15	936	950	CCAACCAAACAGAGCACAGC	59.969	GTACTTGTCCTTCCACCGCA	59.966	141
TRINITY_Contig10288	1	p3	(GCA)5	15	995	1009	CAAGAAACACCTGCACCTGC	59.969	TCCGGTCGGAGGTAGTTCAT	60.034	278
TRINITY_Contig10289	1	p3	(GCG)4	12	813	824	ATTGCTTCCCCTTTCCTCCG	60.034	TGCTCATGGTGGAGAACGAC	60.037	198
TRINITY_Contig10291	1	p5	(ATGCC)3	15	401	415	GTGGTGGCGGTTTCCTTTTC	59.968	TCATCGTAGAGGTGCGCATC	59.969	177
TRINITY_Contig10291	2	p5	(CTGGG)3	15	5112	5126	GTGGTGGCGGTTTCCTTTTC	59.968	TCATCGTAGAGGTGCGCATC	59.969	177
TRINITY_Contig10291	3	c	(AGTA)3ctagtagtgattactgtatgtatg(ATTA)3	48	5705	5752	GTGGTGGCGGTTTCCTTTTC	59.968	TCATCGTAGAGGTGCGCATC	59.969	177
TRINITY_Contig10291	4	p6	(CGAAGC)3	18	6027	6044	GTGGTGGCGGTTTCCTTTTC	59.968	TCATCGTAGAGGTGCGCATC	59.969	177
TRINITY_Contig10291	5	p5	(GGATC)3	15	6237	6251	GTGGTGGCGGTTTCCTTTTC	59.968	TCATCGTAGAGGTGCGCATC	59.969	177
TRINITY_Contig10292	1	p2	(CT)11	22	46	67	CTAACCCTGCACCTCCGAAG	60.108	TTCAACCGCTCCAGCATCAT	60.035	269
TRINITY_Contig10292	2	p4	(ACAT)3	12	910	921	CTAACCCTGCACCTCCGAAG	60.108	TTCAACCGCTCCAGCATCAT	60.035	269
TRINITY_Contig10293	1	p3	(GGA)6	18	1166	1183	ACTGCCCAGCATTCCAATGA	59.96	GTCATTGGGAGATGGGCCAA	60.033	152
TRINITY_Contig10295	1	p3	(TCA)4	12	562	573	TAGCGTCTGGTTTTGCGAGT	59.967	GGGGTCATGTTGATCTGCCA	60.034	139
TRINITY_Contig10297	1	p4	(TAGA)3	12	1526	1537	CAACCTGCAAAGCCAACTCC	59.967	TGGAAAAGTGGCGCCCATAT	60.034	102
TRINITY_Contig10297	2	p3	(TTC)4	12	1959	1970	CAACCTGCAAAGCCAACTCC	59.967	TGGAAAAGTGGCGCCCATAT	60.034	102
TRINITY_Contig10298	1	p2	(AT)8	16	3	18	CTCAGGATCATCCGTGACCG	59.969	GAGGATTCATCGCCTCCTGG	59.965	236
TRINITY_Contig10300	1	p3	(CCG)4	12	381	392	ATGCGTGTACTGCGAACGTA	60.11	TACGTGCAGAAAGGCTCCTC	59.753	100
TRINITY_Contig10300	2	p3	(GCC)4	12	506	517	ATGCGTGTACTGCGAACGTA	60.11	TACGTGCAGAAAGGCTCCTC	59.753	100
TRINITY_Contig10301	1	p3	(CGC)5	15	361	375	AAAGGATGGCCGGAGTTCAG	60.035	CTAGTCGTCGTACTCACCGC	59.974	130
TRINITY_Contig10301	2	p3	(GGA)4	12	589	600	AAAGGATGGCCGGAGTTCAG	60.035	CTAGTCGTCGTACTCACCGC	59.974	130
TRINITY_Contig10301	3	p2	(TA)10	20	1400	1419	AAAGGATGGCCGGAGTTCAG	60.035	CTAGTCGTCGTACTCACCGC	59.974	130
TRINITY_Contig10301	4	p4	(TCGA)3	12	1530	1541	AAAGGATGGCCGGAGTTCAG	60.035	CTAGTCGTCGTACTCACCGC	59.974	130
TRINITY_Contig10303	1	p4	(GCTA)3	12	1111	1122	GTCGATTTAGGAGGAGCGGG	59.968	TACACACCGGCTTGCTCAAT	59.964	118
TRINITY_Contig10304	1	p3	(GGT)4	12	2662	2673	CCAGCAAGTCCTCAGAGCAA	59.964	CGAGTCCGATGAAGAGTCCG	59.972	236
TRINITY_Contig10304	2	p3	(CGC)4	12	2998	3009	CCAGCAAGTCCTCAGAGCAA	59.964	CGAGTCCGATGAAGAGTCCG	59.972	236
TRINITY_Contig10304	3	c	(GCT)5gcgcggtcga(GGT)6	43	3112	3154	CCAGCAAGTCCTCAGAGCAA	59.964	CGAGTCCGATGAAGAGTCCG	59.972	236
TRINITY_Contig10304	4	c	(GAGG)3cgagggggtggaaggg(GA)8	44	3415	3458	CCAGCAAGTCCTCAGAGCAA	59.964	CGAGTCCGATGAAGAGTCCG	59.972	236
TRINITY_Contig10305	1	c	(CGCC)4cccatgaagctgttcgcctttgtgcggcgcgcgcgcggt(CGC)4	68	61	128	GATGACGCCTACCACTACCG	59.97	AAGCGAGTGCAAAGCTGTTG	59.97	220
TRINITY_Contig10305	2	p5	(GCTGT)3	15	1105	1119	GATGACGCCTACCACTACCG	59.97	AAGCGAGTGCAAAGCTGTTG	59.97	220
TRINITY_Contig10306	1	p3	(CGA)4	12	131	142	CTTCCTCCGCAAACAACAGC	60.04	AGATGTGCATGAGTTGGCGA	60.036	203
TRINITY_Contig10309	1	p4	(GGAA)4	16	76	91	CATTCTCGCCATGCATTGGG	59.968	AGCAATGCACCATCTCTGCT	60.034	211
TRINITY_Contig10310	1	p3	(TCT)6	18	594	611	GACCGTACCAGCACCAAGAA	59.966	TTGCTGTGGAAGTCCGGATC	60.036	168
TRINITY_Contig10311	1	p4	(CCGG)3	12	215	226	AGCACAAGCAAACGAAGCAG	59.97	CAGGGATCGAGGAGTTGGTG	59.823	233
TRINITY_Contig10311	2	p3	(CTC)6	18	663	680	AGCACAAGCAAACGAAGCAG	59.97	CAGGGATCGAGGAGTTGGTG	59.823	233
TRINITY_Contig10311	3	c	(AGC)5agggcaggtggcgagcctttactccagcggaaagggcgcgttaggtccgcacgcgcgcgcagcagctcggcgctgg(GGCC)3	103	1143	1245	AGCACAAGCAAACGAAGCAG	59.97	CAGGGATCGAGGAGTTGGTG	59.823	233
TRINITY_Contig10312	1	p3	(GGC)4	12	1150	1161	CATCATGCCACGCAATCAGG	59.97	GTTGTCGCATCGCATGTCTC	59.974	169
TRINITY_Contig10313	1	p3	(CGC)7	21	876	896	CCCGTAGAGCTCATGCAGAC	60.249	CGAGCAGATCCCGTTCTACC	59.969	114
TRINITY_Contig10313	2	p3	(GTG)4	12	1306	1317	CCCGTAGAGCTCATGCAGAC	60.249	CGAGCAGATCCCGTTCTACC	59.969	114
TRINITY_Contig10318	1	p3	(CAC)5	15	1	15	CCTTGTTTGTTTGCCCTCGG	59.968	TGGGCAAGAAGATCAGGCTG	60.035	151
TRINITY_Contig10318	2	p3	(CCG)5	15	307	321	CCTTGTTTGTTTGCCCTCGG	59.968	TGGGCAAGAAGATCAGGCTG	60.035	151
TRINITY_Contig10318	3	p2	(CA)6	12	1010	1021	CCTTGTTTGTTTGCCCTCGG	59.968	TGGGCAAGAAGATCAGGCTG	60.035	151
TRINITY_Contig10319	1	c	(GCG)7gctacggcaacagcagcgcccccta(CGG)6	64	643	706	TATCCCTGCATGTTCGCCTC	59.893	TGCCACCATGATGAACAGCT	59.961	184
TRINITY_Contig10321	1	p2	(TC)6	12	98	109	CCAATTGGGATTAAGCGGCG	59.969	AGAACATCCCATGCACCCAG	60.034	135
TRINITY_Contig10323	1	c	(ATTA)3ggcggcattaac(CATG)3tggacatcaggagcgccggcggcggcgat(CAC)4	77	318	394	AGAGCCATGGCACGGTAAAA	59.962	CCAGCCATGGTGCATTGAAC	60.109	259
TRINITY_Contig10324	1	p3	(ATC)4	12	201	212	AAGGCACACTGCTGGAGTAC	59.965	CAGTGAGTCAGTGCTGAGCA	59.967	100
TRINITY_Contig10324	2	p3	(GCC)7	21	543	563	AAGGCACACTGCTGGAGTAC	59.965	CAGTGAGTCAGTGCTGAGCA	59.967	100
TRINITY_Contig10324	3	p3	(TCC)4	12	808	819	AAGGCACACTGCTGGAGTAC	59.965	CAGTGAGTCAGTGCTGAGCA	59.967	100
TRINITY_Contig10324	4	p6	(GGCGCC)3	18	1157	1174	AAGGCACACTGCTGGAGTAC	59.965	CAGTGAGTCAGTGCTGAGCA	59.967	100
TRINITY_Contig10326	1	p3	(TGC)5	15	1130	1144	TGGAAGAAGAGCTGCTTGGG	59.963	ACACGTACAGAGCTGACACG	60.04	121
TRINITY_Contig10329	1	c	(CCAC)3cag(TC)7	29	23	51	AGGCCTCTCTCAAGGAGGAG	60.032	GTGCTTCCGTCAACCACAAC	59.971	222
TRINITY_Contig10329	2	c	(GA)22ggctgtgatcgatcacaggaggaggagcagctctctcagtctcag(GACC)3	104	163	266	AGGCCTCTCTCAAGGAGGAG	60.032	GTGCTTCCGTCAACCACAAC	59.971	222
TRINITY_Contig10329	3	p4	(CTTC)3	12	1578	1589	AGGCCTCTCTCAAGGAGGAG	60.032	GTGCTTCCGTCAACCACAAC	59.971	222
TRINITY_Contig10329	4	p3	(GGC)5	15	2059	2073	AGGCCTCTCTCAAGGAGGAG	60.032	GTGCTTCCGTCAACCACAAC	59.971	222
TRINITY_Contig10329	5	c	(CTC)4tgtcctcactcctcagtccttactcagtgatgagagagaa(AG)11	74	2381	2454	AGGCCTCTCTCAAGGAGGAG	60.032	GTGCTTCCGTCAACCACAAC	59.971	222
TRINITY_Contig10331	1	p4	(TAAT)3	12	45	56	CGCTGCGCTTCAAGATCTTC	59.974	TCATCGACCTGTTCTGCACC	60.037	276
TRINITY_Contig10333	1	c	(TCT)6(TCC)6tcttccctctctccctcctcctcccctcgccctc(CTCCGG)3	89	995	1083	CTTCCCTCTCTCCCTCCTCC	60.106	ACAAGAAGGCCTTCATCCGG	60.035	174
TRINITY_Contig10333	2	p4	(TGGC)3	12	1355	1366	CTTCCCTCTCTCCCTCCTCC	60.106	ACAAGAAGGCCTTCATCCGG	60.035	174
TRINITY_Contig10334	1	p4	(ATCG)3	12	255	266	GACCCAGGACTTCAAGACGG	60.037	AACGCGTTGTAGAAGAGCCA	59.967	129
TRINITY_Contig10334	2	p4	(GTTG)4	16	518	533	GACCCAGGACTTCAAGACGG	60.037	AACGCGTTGTAGAAGAGCCA	59.967	129
TRINITY_Contig10336	1	p3	(CCT)4	12	157	168	AACAATGCCGCCCCATAAGA	60.034	GATGATAACCCGGCCATCGT	59.965	253
TRINITY_Contig10339	1	p4	(TAAT)3	12	45	56	AGGACTCGAACGTGTTGACC	59.968	GCAAACGGGGTTGAAGGAAC	59.968	185
TRINITY_Contig10339	2	p5	(CGCCG)3	15	746	760	AGGACTCGAACGTGTTGACC	59.968	GCAAACGGGGTTGAAGGAAC	59.968	185
TRINITY_Contig10340	1	p3	(CGG)4	12	597	608	CTCCGTTTGCAGGTCTGTCT	59.966	CCCACCTGAAGGAGAAGCTG	60.036	153
TRINITY_Contig10340	2	p3	(TGC)4	12	993	1004	CTCCGTTTGCAGGTCTGTCT	59.966	CCCACCTGAAGGAGAAGCTG	60.036	153
TRINITY_Contig10340	3	p6	(TATTTG)3	18	1132	1149	CTCCGTTTGCAGGTCTGTCT	59.966	CCCACCTGAAGGAGAAGCTG	60.036	153
TRINITY_Contig10341	1	p3	(CGG)5	15	491	505	TGAGAAGAGGGCCGAGATGA	60.033	TATGAGCAGGGAAGCCTCCT	60.031	256
TRINITY_Contig10341	2	p3	(GAG)4	12	1050	1061	TGAGAAGAGGGCCGAGATGA	60.033	TATGAGCAGGGAAGCCTCCT	60.031	256
TRINITY_Contig10342	1	p4	(GCTG)3	12	71	82	AACAACCCACCAAGTGCTGA	60.034	GCAGCATTCCCTGTCTCCTT	60.035	272
TRINITY_Contig10342	2	p3	(GCG)6	18	417	434	AACAACCCACCAAGTGCTGA	60.034	GCAGCATTCCCTGTCTCCTT	60.035	272
TRINITY_Contig10342	3	p3	(CCG)5	15	2576	2590	AACAACCCACCAAGTGCTGA	60.034	GCAGCATTCCCTGTCTCCTT	60.035	272
TRINITY_Contig10344	1	p3	(GGC)5	15	303	317	CATCGAGTAACCGGGGATGG	59.966	ACAGAGCACGGACCGAATTT	59.965	209
TRINITY_Contig10345	1	p3	(CGG)5	15	907	921	GCTCGCAGTTTGCACAAGAA	59.971	TGTTGGTTGCATTGCAGACG	59.969	157
TRINITY_Contig10345	2	p5	(CGGTT)3	15	1314	1328	GCTCGCAGTTTGCACAAGAA	59.971	TGTTGGTTGCATTGCAGACG	59.969	157
TRINITY_Contig10346	1	p3	(GCC)7	21	966	986	GATCTCGTCCTGGAGCTTCG	59.97	CTCCTGATCCATCCGTGCTC	59.967	145
TRINITY_Contig10346	2	c	(AGG)4(CCG)7	33	2138	2170	GATCTCGTCCTGGAGCTTCG	59.97	CTCCTGATCCATCCGTGCTC	59.967	145
TRINITY_Contig10347	1	c	(CGC)4cgtcgccgaccttgcgccccttcatcaggagaggcgacctcatggtcctgggcgacccgacgacatccat(GCC)4	94	1223	1316	CTGCGTTGGTGGTGATGTTG	60.04	AGCGCCTTCTCAATCCACAA	59.963	175
TRINITY_Contig10350	1	p5	(TTTTC)3	15	1400	1414	CATCTTCCCAGAGGCAACGT	60.036	GTGGTCCACAGATGCAGGAA	59.963	249
TRINITY_Contig10353	1	p4	(ATCG)3	12	424	435	GATGGCGATCGTTGAGGGAT	59.966	CGGGAGGCATTCAAGGTCTT	60.035	270
TRINITY_Contig10353	2	p2	(TC)7	14	1186	1199	GATGGCGATCGTTGAGGGAT	59.966	CGGGAGGCATTCAAGGTCTT	60.035	270
TRINITY_Contig10355	1	p2	(CT)9	18	1340	1357	GACTTGCCCCTGAGCAATCT	60.035	AGGTCAGTGTGACTTGCCTG	59.893	245

TRINITY_Contig10356	1	p3	(GGC)4	12	253	264	GACCCCGCAAAAATTTCCCC	60.037	GCCGCCCTACTCCTTAATCC	59.965	188
TRINITY_Contig10356	2	p4	(GAGG)3	12	1267	1278	GACCCCGCAAAAATTTCCCC	60.037	GCCGCCCTACTCCTTAATCC	59.965	188
TRINITY_Contig10358	1	p3	(TCA)4	12	44	55	GGCTTGGCATCCTTCTCCTT	60.034	CTCCACGATCGTCCTCAAGG	59.899	240
TRINITY_Contig10358	2	c	(CTG)4cccgtagtacggctgccggg(CGC)5	47	342	388	GGCTTGGCATCCTTCTCCTT	60.034	CTCCACGATCGTCCTCAAGG	59.899	240
TRINITY_Contig10358	3	p3	(CCG)6	18	508	525	GGCTTGGCATCCTTCTCCTT	60.034	CTCCACGATCGTCCTCAAGG	59.899	240
TRINITY_Contig10358	4	c	(AG)7gagggagtgtgcggggaagga(GAAG)3	47	1317	1363	GGCTTGGCATCCTTCTCCTT	60.034	CTCCACGATCGTCCTCAAGG	59.899	240
TRINITY_Contig10359	1	p3	(CCG)4	12	24	35	GCATGGAGAGGTCGTACTCG	59.97	CGCTGCCTAATCCTGTGGAT	59.893	197
TRINITY_Contig10359	2	p3	(CCG)4	12	188	199	GCATGGAGAGGTCGTACTCG	59.97	CGCTGCCTAATCCTGTGGAT	59.893	197
TRINITY_Contig10359	3	p3	(GCG)4	12	806	817	GCATGGAGAGGTCGTACTCG	59.97	CGCTGCCTAATCCTGTGGAT	59.893	197
TRINITY_Contig10360	1	p6	(CGAGCA)3	18	1308	1325	CAGGGCCGTGATGTAGGTTT	60.035	CAAGCTGTCAGGCAGGAAGA	59.964	163
TRINITY_Contig10362	1	p3	(CGG)5	15	53	67	AAGTCTCAGCTTGGCTCACC	59.964	CCGTTGCAGTAGGTGGTCTT	59.965	202
TRINITY_Contig10363	1	p4	(GATA)3	12	144	155	GATAGATACGGAGCCGGTGC	60.109	CAGGTACCAGCTCATCGACC	59.897	222
TRINITY_Contig10363	2	p3	(GAG)4	12	614	625	GATAGATACGGAGCCGGTGC	60.109	CAGGTACCAGCTCATCGACC	59.897	222
TRINITY_Contig10363	3	p3	(GCC)5	15	910	924	GATAGATACGGAGCCGGTGC	60.109	CAGGTACCAGCTCATCGACC	59.897	222
TRINITY_Contig10365	1	p4	(CATG)3	12	858	869	CTGCCAAGAAGATGGGCAGA	60.035	CATGATGGTCAGCAGCAAGC	59.899	101
TRINITY_Contig10367	1	p3	(GGT)4	12	1794	1805	GCAAAGGCTGGAACATGCAA	59.966	TGCACAGAAAGCAAATCGCC	60.039	223
TRINITY_Contig10368	1	p3	(CGA)4	12	192	203	CTGGTACGGCATCGACTACC	59.97	TGCTCGTCGAAGGAGAGGTA	60.036	231
TRINITY_Contig10368	2	p6	(CGGCGC)3	18	1239	1256	CTGGTACGGCATCGACTACC	59.97	TGCTCGTCGAAGGAGAGGTA	60.036	231
TRINITY_Contig10369	1	p2	(GT)9	18	1353	1370	CTGCCTCATGTTGGGCCTTA	60.034	TTGATCGTTGTCGCCAAGGA	59.966	146
TRINITY_Contig10372	1	p4	(TGGT)4	16	3508	3523	TTGGGATCCTCGCTTGGAAC	60.035	GAGAGCACCAGGTATCGACG	59.97	238
TRINITY_Contig10374	1	p3	(CTC)4	12	313	324	ATTCTCCTTGGCAGGCCTTC	60.034	ATTTCGTGGGCTCCTCAAGG	60.035	227
TRINITY_Contig10374	2	p3	(AAG)4	12	820	831	ATTCTCCTTGGCAGGCCTTC	60.034	ATTTCGTGGGCTCCTCAAGG	60.035	227
TRINITY_Contig10376	1	p6	(CGCGGC)3	18	1229	1246	TTACTCGCAGTTGCTCCGTT	59.967	GCTCCATGTGTACACCGGAA	60.037	185
TRINITY_Contig10377	1	p4	(TTAT)3	12	119	130	AACTTCACGATGGGCGACAT	60.036	ACAACGTGCAGGCCTACTAC	60.038	244
TRINITY_Contig10379	1	p2	(TA)8	16	125	140	CGTCGTAGGCTATCTTGCGT	59.971	TGACCTTCGCCACCAACTAC	59.966	192
TRINITY_Contig10379	2	c	(GGT)4gatggac(GGGA)4	35	1291	1325	CGTCGTAGGCTATCTTGCGT	59.971	TGACCTTCGCCACCAACTAC	59.966	192
TRINITY_Contig10380	1	p3	(GCG)4	12	818	829	TGGTCCCTCCTCATCTCCAG	60.032	CAAACTTTGCCCTGAACCGG	59.968	171
TRINITY_Contig10380	2	p3	(CTA)4	12	2128	2139	TGGTCCCTCCTCATCTCCAG	60.032	CAAACTTTGCCCTGAACCGG	59.968	171
TRINITY_Contig10380	3	c	(TC)13gct(CTCG)3	41	2475	2515	TGGTCCCTCCTCATCTCCAG	60.032	CAAACTTTGCCCTGAACCGG	59.968	171
TRINITY_Contig10381	1	p2	(AC)7	14	655	668	TGCTAGTTGTCAAGCAGGGG	59.963	GAAAGCTGCCCCAGGAGATT	60.034	170
TRINITY_Contig10382	1	p3	(GCC)4	12	190	201	TCCATGTGGGCTTAGGCTTG	60.034	TGGCAGTTTCTGGGTGTTGT	60.034	115
TRINITY_Contig10383	1	p3	(GGC)5	15	1260	1274	AGCGAAGGGAAAAAGGGAGG	59.962	AATTCCCTCCTCCTCCCTCC	60.03	208
TRINITY_Contig10383	2	p2	(GA)8	16	1378	1393	AGCGAAGGGAAAAAGGGAGG	59.962	AATTCCCTCCTCCTCCCTCC	60.03	208
TRINITY_Contig10384	1	p3	(CGA)6	18	435	452	CTGATGCAGCGGATCAGGAT	59.965	GACGTCGCCTAGTGTGGTAG	59.902	228
TRINITY_Contig10384	2	p3	(CAC)4	12	927	938	CTGATGCAGCGGATCAGGAT	59.965	GACGTCGCCTAGTGTGGTAG	59.902	228
TRINITY_Contig10385	1	p4	(ATCG)3	12	21	32	AGCAAGTATGGGCTGTGACC	60.035	GGGGAACTAGCGAGATCAGC	59.967	224
TRINITY_Contig10385	2	p3	(GCG)4	12	726	737	AGCAAGTATGGGCTGTGACC	60.035	GGGGAACTAGCGAGATCAGC	59.967	224
TRINITY_Contig10386	1	p3	(ACC)4	12	299	310	CACCGGGTCATGTTCATGGA	60.036	ACCTTCACCTTCTTGCTCGG	59.965	200
TRINITY_Contig10387	1	p3	(CGA)8	24	570	593	CAGTCCTCCTCCTCGTACCA	60.034	CCAGGAGTTGGGCATCATGT	60.034	110
TRINITY_Contig10387	2	c	(GAC)4ggccatggcggatgaggtcgtcacccgtcgtgctccggctgcatgctcgatcagtatgcgtccgcggattaactagcattagctcttgactcgttg(TTAA)3	120	1231	1350	CAGTCCTCCTCCTCGTACCA	60.034	CCAGGAGTTGGGCATCATGT	60.034	110
TRINITY_Contig10390	1	p4	(TGCC)3	12	79	90	TTGCTCTAGCGCAACTGACA	59.967	TGAAAAGCACGCCATAGGGT	59.962	103
TRINITY_Contig10390	2	p4	(GGCT)3	12	210	221	TTGCTCTAGCGCAACTGACA	59.967	TGAAAAGCACGCCATAGGGT	59.962	103
TRINITY_Contig10390	3	p3	(GGT)5	15	477	491	TTGCTCTAGCGCAACTGACA	59.967	TGAAAAGCACGCCATAGGGT	59.962	103
TRINITY_Contig10391	1	p6	(CGGGGG)4	24	514	537	AGGAAAAGGAGGAGGCAACG	59.963	TCCACCACCAGCACCAATAC	59.962	273
TRINITY_Contig10391	2	p6	(CCCCCG)4	24	863	886	AGGAAAAGGAGGAGGCAACG	59.963	TCCACCACCAGCACCAATAC	59.962	273
TRINITY_Contig10391	3	p3	(CCA)4	12	1882	1893	AGGAAAAGGAGGAGGCAACG	59.963	TCCACCACCAGCACCAATAC	59.962	273
TRINITY_Contig10392	1	p3	(CGG)4	12	624	635	GGACGGGTCATCTCATCGTC	59.969	AGTACTCTCCCGTGTCCCTC	60.034	143
TRINITY_Contig10392	2	p4	(CGCA)3	12	859	870	GGACGGGTCATCTCATCGTC	59.969	AGTACTCTCCCGTGTCCCTC	60.034	143
TRINITY_Contig10397	1	p4	(GGAT)4	16	597	612	ACTAACTCGCGCTTTCCCAA	59.966	CCGTTTGTTCGCCACCATTT	59.969	134
TRINITY_Contig10397	2	c	(CATC)3ccgctcccagccgctgcgatggggaggaggagcctgccgtcgccgagccgcgccgcggttttgctccggcctgccgagtgcggggtgccc(GCCG)3	114	777	890	ACTAACTCGCGCTTTCCCAA	59.966	CCGTTTGTTCGCCACCATTT	59.969	134
TRINITY_Contig10397	3	p6	(GCGCAG)3	18	1224	1241	ACTAACTCGCGCTTTCCCAA	59.966	CCGTTTGTTCGCCACCATTT	59.969	134
TRINITY_Contig10397	4	p4	(ACGC)3	12	1405	1416	ACTAACTCGCGCTTTCCCAA	59.966	CCGTTTGTTCGCCACCATTT	59.969	134
TRINITY_Contig10397	5	p3	(GCT)4	12	1969	1980	ACTAACTCGCGCTTTCCCAA	59.966	CCGTTTGTTCGCCACCATTT	59.969	134
TRINITY_Contig10397	6	p5	(GCCTC)3	15	2583	2597	ACTAACTCGCGCTTTCCCAA	59.966	CCGTTTGTTCGCCACCATTT	59.969	134
TRINITY_Contig10397	7	p5	(GAGGA)3	15	3066	3080	ACTAACTCGCGCTTTCCCAA	59.966	CCGTTTGTTCGCCACCATTT	59.969	134
TRINITY_Contig10398	1	p3	(CCG)6	18	490	507	CGATCAAGGGCATGGCATTG	59.968	TCCCTCGGTCCAGCTTTTTC	59.964	257
TRINITY_Contig10398	2	p4	(AGCT)3	12	1017	1028	CGATCAAGGGCATGGCATTG	59.968	TCCCTCGGTCCAGCTTTTTC	59.964	257
TRINITY_Contig10399	1	p3	(CGA)4	12	1299	1310	CTTCTCGTACGTCGACTCGG	59.975	GGCGATCCAGAACTTCCACA	60.036	220
TRINITY_Contig10401	1	c	(GCG)5gtcactgcgagggtgctgc(GGT)5gcggcctggcgaggaaggccgggcccgccgc(GGA)4tgagcaggccgaa(GGC)5ggagcggcgtgtagcggcgccattctccgcccctgtctcttcctctcggc(AGCG)4	186	805	990	GGTGCAAAAGAGACCGAGGA	59.965	CGCCTGTCACCTGTCAGAAT	60.037	116
TRINITY_Contig10402	1	p4	(CGGC)4	16	1298	1313	GCTACCCCCAACAAGCAGAT	60.034	ATATCCACGCCACTGCTACG	59.968	197
TRINITY_Contig10406	1	p4	(CACC)3	12	114	125	TCAGACTGCTGCAAGTCTGG	59.966	CCGGGATGTCTCTGATGCTC	59.967	236
TRINITY_Contig10410	1	p3	(AGA)4	12	1170	1181	TTTCTCAGAAGGGTCCACGC	59.965	AAGCTTCCCACGATCACCTG	60.036	280
TRINITY_Contig10411	1	p4	(CAGA)3	12	100	111	ACCGCCCTGTCCTTGTATTG	60.035	TCCTCTCCCTCTACCTGCAC	60.033	126
TRINITY_Contig10413	1	p4	(CGCC)3	12	318	329	TTTGGGTCAGTGTACGAGGC	59.966	AAGCTGCCAACGATCCTTGA	59.963	236
TRINITY_Contig10415	1	c	(GCT)4ggggattctccgcatcg(CCCGTC)3	47	1245	1291	CACAGGAGTGCCAAAACTGC	59.969	ATGCTCTGACCACTCCTTGC	60.036	172
TRINITY_Contig10416	1	p3	(AAG)5	15	1194	1208	TCAAGTCGGACAAGACGGTG	59.968	GTCCCGAATATTTTGGCGCC	59.97	255
TRINITY_Contig10417	1	p3	(CGC)4	12	110	121	CTCGGTCAGATCGCGAATCA	59.971	ATAGGCACGAGGGGAGTCTT	60.032	176
TRINITY_Contig10417	2	p4	(AGGA)3	12	308	319	CTCGGTCAGATCGCGAATCA	59.971	ATAGGCACGAGGGGAGTCTT	60.032	176
TRINITY_Contig10418	1	p3	(GCC)6	18	516	533	GAGCCACTCCCAAAATCCGA	60.035	CTGCTTCGCGCACTATTTCC	59.973	208
TRINITY_Contig10418	2	p4	(CAAA)3	12	2246	2257	GAGCCACTCCCAAAATCCGA	60.035	CTGCTTCGCGCACTATTTCC	59.973	208
TRINITY_Contig10419	1	p3	(CTG)4	12	1323	1334	GGTTACGACTCGGACCGATC	59.971	CTTCATCTCCAGGGCGGTTT	60.035	117
TRINITY_Contig10423	1	p3	(CGG)5	15	61	75	TAGACGATGACGACGGCATG	59.971	GGTTCGCTGAAGCTGCAAAA	59.969	236
TRINITY_Contig10423	2	p3	(TCA)4	12	807	818	TAGACGATGACGACGGCATG	59.971	GGTTCGCTGAAGCTGCAAAA	59.969	236
TRINITY_Contig10425	1	p3	(CCG)4	12	974	985	TGGAGGCATGTCCATTTGCT	59.96	AGAGGGCAAGAGTGTCGTTG	59.966	122
TRINITY_Contig10425	2	p6	(GCGGGG)3	18	1118	1135	TGGAGGCATGTCCATTTGCT	59.96	AGAGGGCAAGAGTGTCGTTG	59.966	122
TRINITY_Contig10425	3	p3	(GCG)5	15	1247	1261	TGGAGGCATGTCCATTTGCT	59.96	AGAGGGCAAGAGTGTCGTTG	59.966	122
TRINITY_Contig10426	1	p3	(TCT)5	15	1073	1087	TCCTCTGGGTGATCGCTACA	60.034	AACCGCTTCACCTACACCTG	59.965	251
TRINITY_Contig10426	2	p4	(GATT)4	16	1235	1250	TCCTCTGGGTGATCGCTACA	60.034	AACCGCTTCACCTACACCTG	59.965	251
TRINITY_Contig10426	3	p3	(GGC)4	12	2504	2515	TCCTCTGGGTGATCGCTACA	60.034	AACCGCTTCACCTACACCTG	59.965	251
TRINITY_Contig10426	4	p3	(GAG)4	12	2891	2902	TCCTCTGGGTGATCGCTACA	60.034	AACCGCTTCACCTACACCTG	59.965	251
TRINITY_Contig10429	1	c	(AGC)4aggtggatactgatcctccatgggaaacac(CCTCGG)3cgttggccttggcctcggccttt(GCGCCG)3	101	142	242	TCCGATTGATCTACTGCGGC	59.968	CAGTGTTCACGAGCTCACCT	59.967	155
TRINITY_Contig10429	2	p3	(AAG)4	12	881	892	TCCGATTGATCTACTGCGGC	59.968	CAGTGTTCACGAGCTCACCT	59.967	155
TRINITY_Contig10431	1	p3	(GCG)4	12	200	211	CAATTCGGTCGAGCAGCATG	59.973	CGACGAAGAGCAGGAGGATC	59.97	171
TRINITY_Contig10431	2	p3	(AGG)4	12	518	529	CAATTCGGTCGAGCAGCATG	59.973	CGACGAAGAGCAGGAGGATC	59.97	171
TRINITY_Contig10432	1	p5	(GCGGC)3	15	274	288	CGCAGGAAGAAGAAGGAGCA	60.037	ACCCTACTCGTCACTCGTCA	59.964	138
TRINITY_Contig10433	1	p5	(GCGGC)3	15	274	288	GAAGAAGAAGCAGCAGCAGC	59.831	ACCCTACTCGTCACTCGTCA	59.964	137
TRINITY_Contig10433	2	p3	(CCA)4	12	438	449	GAAGAAGAAGCAGCAGCAGC	59.831	ACCCTACTCGTCACTCGTCA	59.964	137
TRINITY_Contig10433	3	p3	(AGC)4	12	1038	1049	GAAGAAGAAGCAGCAGCAGC	59.831	ACCCTACTCGTCACTCGTCA	59.964	137
TRINITY_Contig10434	1	p3	(CGC)4	12	4075	4086	AAGGGAGGCGGAGTTCCTAT	60.031	TCCAACATCGAGGAGCCCTA	60.033	138
TRINITY_Contig10435	1	c	(CAC)4catcaccaaagcctcttcctcccg(TCC)5	51	337	387	TCCTCAGCCCGTATCTCCAA	60.033	GTGGTGATGGTCAGGTGAGG	60.036	168
TRINITY_Contig10435	2	p3	(GCG)4	12	988	999	TCCTCAGCCCGTATCTCCAA	60.033	GTGGTGATGGTCAGGTGAGG	60.036	168
TRINITY_Contig10436	1	c	(CGC)4ggcgtt(GCC)4	30	948	977	GAAAACGCAACGAACTGCCT	59.971	TCGACCTGTGACTGCTTGAC	59.968	191
TRINITY_Contig10437	1	p3	(GCG)7	21	1161	1181	GGCGCCAGCTAGAGTAGAAG	59.969	TGCAGTGTTCAAAGCCATGC	59.968	205
TRINITY_Contig10437	2	c	(CGG)4ggacgggcgtcgggtcggcgggggcctggccggcggcggactggtggggaggagtgcggtggcgacggggaagggag(CCGAT)3	104	1340	1443	GGCGCCAGCTAGAGTAGAAG	59.969	TGCAGTGTTCAAAGCCATGC	59.968	205
TRINITY_Contig10439	1	p4	(AGGA)3	12	1367	1378	TTCTGTTTCTTCCGGTCGGG	59.966	TTCTTCCCGTCCCGTCCTAT	60.033	170

TRINITY_Contig10440	1	c	(TTCT)3cgttacctcgcttttctatgagttttcttttgcatgtcgggtggtgctgatgccgatgtgcgt(CTTTG)4cttcagatagtcgtcactcggtagacataagagcgtagccactctcactcccacctaggccgcggcatggagaaggagaa(GAG)4	187	764	950	CCAAATCCCTCTCGATCCGG	59.966	CAGGATCGCTGGTGCTCTAG	59.968	150
TRINITY_Contig10440	2	p6	(GCCGGC)3	18	1077	1094	CCAAATCCCTCTCGATCCGG	59.966	CAGGATCGCTGGTGCTCTAG	59.968	150
TRINITY_Contig10441	1	c	(GCGG)3cgaggcggagctgcgatgagggcgaggcggggagggagcggggcggggagaagagggcgcgcgcgtcgagggccacgcggggaggggacaa(GGC)4	115	1214	1328	AAACGCTTGGGCATTGCATT	59.965	CGGTGTCGTGTCAGAACTCA	59.97	179
TRINITY_Contig10444	1	p6	(TATGGA)3	18	568	585	AGTATAGGTGGCACGGAGGT	60.032	TGTCCGGGAACTCTAGGAGG	60.033	145
TRINITY_Contig10445	1	c	(GCG)5tgggcgctagaggacgacgacccatcggttccggcggctgc(GGCGGG)3	74	582	655	TGGATCGTCGACACCTCTCT	60.035	CACCAAATCCGGAACCCTGA	59.962	196
TRINITY_Contig10446	1	p3	(CGG)4	12	488	499	AAGGGAAAGATCAGGGCGTG	60.035	TGGTGTTCAGGATTCCACCG	59.964	177
TRINITY_Contig10447	1	c	(AGC)5aaggttagctgctgcaggtgatgatgggacgattgctgctgcgaggaggcggcggccatgatt(GATC)4	94	1224	1317	AGGTAGCTTCGTCCTCTCGT	60.035	GAGAGGTTCGACGCCTACAG	59.901	140
TRINITY_Contig10448	1	p3	(CGC)4	12	710	721	CGGCTTCGCTCTCGACTTAT	59.97	AGGTTGCATCATGGGGAAGG	60.033	118
TRINITY_Contig10448	2	p3	(GTC)4	12	834	845	CGGCTTCGCTCTCGACTTAT	59.97	AGGTTGCATCATGGGGAAGG	60.033	118
TRINITY_Contig10449	1	p3	(TGG)4	12	148	159	GTGCGAGGACTACTACGACG	59.974	GAAGCAGTCGTGGAAGTGGA	59.966	130
TRINITY_Contig10450	1	p4	(TACC)3	12	776	787	GCGTCTGGACAACTGATGGA	60.037	TTGGACTAGCCTCTGCCTCT	59.958	100
TRINITY_Contig10452	1	p3	(CTC)9	27	10	36	GGCGATCCAGTGTATCCTGG	59.966	GCGACGTGCCTTCGTAAATC	59.975	263
TRINITY_Contig10454	1	p3	(CGC)4	12	385	396	GGATGCTGGACCCAAATCCA	60.033	AGCCCAGCCTTGAATTCCTC	60.034	280
TRINITY_Contig10454	2	p3	(CGC)4	12	944	955	GGATGCTGGACCCAAATCCA	60.033	AGCCCAGCCTTGAATTCCTC	60.034	280
TRINITY_Contig10455	1	c	(TCC)4gttaccaaattgatgagaggagatcgaaaatc(CGG)4	56	6	61	GGGCGGAATTTCAGATGTGC	59.899	GTTGCCTTACAGGTGCCTCT	59.963	240
TRINITY_Contig10458	1	c	(GA)9ggcagccttgatgaacccggg(CCATC)3	54	53	106	CCGACGACCAGGTCTTCAAA	59.967	TACCCAGAGCAGACCGATGA	60.034	182
TRINITY_Contig10458	2	p4	(CGGC)3	12	214	225	CCGACGACCAGGTCTTCAAA	59.967	TACCCAGAGCAGACCGATGA	60.034	182
TRINITY_Contig10458	3	p3	(TAG)4	12	867	878	CCGACGACCAGGTCTTCAAA	59.967	TACCCAGAGCAGACCGATGA	60.034	182
TRINITY_Contig10460	1	p3	(CGC)6	18	191	208	CTAGCGAGAAAGGGAGAGCG	59.97	AGGCAAAAGTGTGACCACCA	60.034	153
TRINITY_Contig10460	2	p5	(GTGAT)3	15	1042	1056	CTAGCGAGAAAGGGAGAGCG	59.97	AGGCAAAAGTGTGACCACCA	60.034	153
TRINITY_Contig10461	1	p5	(ATCCA)4	20	290	309	CTGCTGCTGCTTGGAAACAG	60.039	ACCTCTCCATCCACCCTCTC	60.032	157
TRINITY_Contig10461	2	c	(GAT)4gagaaactctcctcgagatcttactctacacacacctcgcccggctt(CGGC)3	71	830	900	CTGCTGCTGCTTGGAAACAG	60.039	ACCTCTCCATCCACCCTCTC	60.032	157
TRINITY_Contig10461	3	c	(CAG)4(GAG)4	24	1080	1103	CTGCTGCTGCTTGGAAACAG	60.039	ACCTCTCCATCCACCCTCTC	60.032	157
TRINITY_Contig10463	1	p2	(AT)7	14	195	208	GGCTGCTGGAACTTTCTTGC	60.039	CCTTTGTTATCCCCGGGCTT	60.034	127
TRINITY_Contig10464	1	p3	(AAT)4	12	371	382	TGCTGTGTGCTGTCTCTCTG	59.967	CAACAGGTGCTGCAAGTTCC	59.969	254
TRINITY_Contig10465	1	p4	(TCTT)3	12	19	30	GCTCTGCAACAGCCTTTGAC	60.04	CGTCAGGCTCCTCAAGTGTT	59.966	243
TRINITY_Contig10465	2	p4	(GAAA)3	12	1332	1343	GCTCTGCAACAGCCTTTGAC	60.04	CGTCAGGCTCCTCAAGTGTT	59.966	243
TRINITY_Contig10466	1	p3	(GGC)5	15	215	229	CAGTGATCGGATCGGACTCG	60.041	ACTGGTGCATGCAAAGCAAG	59.967	105
TRINITY_Contig10466	2	c	(CCGG)3ccccgcggtgtcggtgtcggtgacggccacgag(GCA)4	57	490	546	CAGTGATCGGATCGGACTCG	60.041	ACTGGTGCATGCAAAGCAAG	59.967	105
TRINITY_Contig10467	1	p3	(ACG)4	12	935	946	AGCAAAAGCAGTCCTGTGGT	60.107	GATGGGAATGGCGGATTTGC	59.967	238
TRINITY_Contig10469	1	p3	(GCC)4	12	1137	1148	TTGGCTCAGTTCCTCTGCAG	59.964	CGTCTACTCGCAGTCCATCC	59.97	231
TRINITY_Contig10470	1	p3	(CGC)4	12	323	334	TGCTGGTCTGTTGTAGCAGG	59.965	TGATCGCACAGGTTTCCTCC	60.036	176
TRINITY_Contig10471	1	p3	(CGA)4	12	321	332	CTGATTGCTTCCATGACGCG	59.973	CCCTGCAATGTCATCCCAGT	60.034	176
TRINITY_Contig10473	1	p4	(TGCT)3	12	1258	1269	ATGAACCGGTGCTCTGTCTG	60.037	CAAGCAAGCAAGCAGAAGCA	59.968	242
TRINITY_Contig10476	1	p2	(CG)6	12	597	608	GAGGTGGTGGAGGAGGAGAT	60.032	TTCCGAGTCCGATCCTTCCT	60.033	231
TRINITY_Contig10476	2	p3	(CGT)4	12	748	759	GAGGTGGTGGAGGAGGAGAT	60.032	TTCCGAGTCCGATCCTTCCT	60.033	231
TRINITY_Contig10476	3	p4	(GATC)3	12	1095	1106	GAGGTGGTGGAGGAGGAGAT	60.032	TTCCGAGTCCGATCCTTCCT	60.033	231
TRINITY_Contig10477	1	p3	(TCA)4	12	878	889	CCAAGTGCCAGTGACAAACG	59.971	AGGGGTGGCTGAATCACATG	60.034	137
TRINITY_Contig10478	1	p4	(TAGC)4	16	80	95	AGCTACTGGATCCACCGTCT	60.033	ACAATGACTGGGCCAGACAG	59.963	161
TRINITY_Contig10478	2	p2	(TG)7	14	1875	1888	AGCTACTGGATCCACCGTCT	60.033	ACAATGACTGGGCCAGACAG	59.963	161
TRINITY_Contig10479	1	p3	(GCC)5	15	168	182	CAAGCACACGAGCTCAGAGA	60.039	ACAATGACCTGGTGCTCCTG	59.963	219
TRINITY_Contig10479	2	p3	(GAA)4	12	438	449	CAAGCACACGAGCTCAGAGA	60.039	ACAATGACCTGGTGCTCCTG	59.963	219
TRINITY_Contig10480	1	p3	(CGC)4	12	650	661	GTAGCTCAGGACGGACATGG	59.897	GCCTCTTCATCTACTCCGGC	59.967	137
TRINITY_Contig10480	2	p3	(GAG)4	12	836	847	GTAGCTCAGGACGGACATGG	59.897	GCCTCTTCATCTACTCCGGC	59.967	137
TRINITY_Contig10483	1	c	(TCC)4(TCT)5tcctccgaggcgccagcagacctgcgggacctcgtcgctagctgcaattaacggcg(GCA)5	98	76	173	TCAAGCTGTTCGGGAAGACC	59.965	ACCTTGTCGGGCTTCTTGAG	59.965	256
TRINITY_Contig10483	2	p3	(AGC)4	12	339	350	TCAAGCTGTTCGGGAAGACC	59.965	ACCTTGTCGGGCTTCTTGAG	59.965	256
TRINITY_Contig10483	3	p3	(CCG)5	15	736	750	TCAAGCTGTTCGGGAAGACC	59.965	ACCTTGTCGGGCTTCTTGAG	59.965	256
TRINITY_Contig10484	1	p3	(GCC)4	12	273	284	TCTCAAATTCATGCGGCCCT	60.034	TAGGTGGAGCGATCCTGACA	60.034	275
TRINITY_Contig10484	2	p3	(CGG)5	15	1080	1094	TCTCAAATTCATGCGGCCCT	60.034	TAGGTGGAGCGATCCTGACA	60.034	275
TRINITY_Contig10485	1	p3	(CCT)4	12	594	605	GGCCTCATCGTCCTCAACAA	60.036	AGGTAAGGCGAGACTGGAGT	59.96	194
TRINITY_Contig10491	1	p6	(CAGCAC)3	18	116	133	AATGTCAACCCAGATGCCGT	59.962	ATGTCGGGGGCAATGGAAAT	60.032	200
TRINITY_Contig10492	1	c	(CGG)4ccagcagctgaaaccggcgcgccctgcccatcgagaatctcagggtcctcgggatctctcggcccctctctctgctctccaccgctctgccttgccgc(GGT)6gtggagtgtccgtggagtgtactggctctagctctactggcgacgcctcgctcgcggctcgcctgccgcctcggtgggttgc(CTCCG)3	226	1086	1311	GCCCATCGAGAATCTCAGGG	59.965	AAACGACTGCGCAAGAAACC	59.971	280
TRINITY_Contig10493	1	p3	(GCC)4	12	973	984	GGAGACATGGAGGAGGAGGT	60.032	ACACGAGGCTAGGAATCCCT	60.032	106
TRINITY_Contig10495	1	p2	(TC)15	30	1070	1099	GCAAGCCAACACCCCTATCT	60.034	CATCTGATCCAGGTCGCCTC	59.967	216
TRINITY_Contig10499	1	p3	(CGC)4	12	363	374	TCCCCTGCAACCATCACTTC	59.962	CAGCCTCTGAGGTGTGGAAG	60.037	149
TRINITY_Contig10499	2	p4	(AAGC)3	12	1242	1253	TCCCCTGCAACCATCACTTC	59.962	CAGCCTCTGAGGTGTGGAAG	60.037	149
TRINITY_Contig10500	1	p3	(CCG)5	15	866	880	GCTACGGAGGTACATCTGCC	59.968	TGAGTTCGTCAAGTCGGTGG	59.968	220
TRINITY_Contig10500	2	p3	(CTC)4	12	1142	1153	GCTACGGAGGTACATCTGCC	59.968	TGAGTTCGTCAAGTCGGTGG	59.968	220
TRINITY_Contig10501	1	c	(CCG)4gat(CCG)4cgtggtccaaccccggcagatccgccccggcgtcctccgcggccccgtcctacgacgacctcttcggcaccg(CCGCCC)4	123	272	394	GCCGGGTTCGATGATCTGAT	59.966	AAACTATCCGCAGCAGCCTT	60.035	257
TRINITY_Contig10501	2	c	(CGC)4cagcgt(ACG)4	30	635	664	GCCGGGTTCGATGATCTGAT	59.966	AAACTATCCGCAGCAGCCTT	60.035	257
TRINITY_Contig10502	1	p4	(CTCC)3	12	38	49	CGTCTTCAACCGGGTGTACA	59.968	CTCCGGAGCGTGAACAATCT	60.109	202
TRINITY_Contig10504	1	p6	(CGAAGC)4	24	1012	1035	GGTCAGGCACTTAAGCACCT	59.963	CTTGGATGTGGACATGGCCT	60.034	102
TRINITY_Contig10505	1	p4	(GATG)7	28	831	858	GTGGTGTGCATCAGAAGGGA	59.963	CAGGCAAACACAGCAGCAAT	59.967	255
TRINITY_Contig10505	2	p3	(GGC)4	12	1158	1169	GTGGTGTGCATCAGAAGGGA	59.963	CAGGCAAACACAGCAGCAAT	59.967	255
TRINITY_Contig10506	1	p3	(CAT)4	12	659	670	TTCACCTTCGGCAGTGACTC	59.966	ACTCAAGATGGCGGTCGTTT	59.965	232
TRINITY_Contig10506	2	p3	(CGG)8	24	1093	1116	TTCACCTTCGGCAGTGACTC	59.966	ACTCAAGATGGCGGTCGTTT	59.965	232
TRINITY_Contig10507	1	p3	(GCG)5	15	1246	1260	CTCTGTGCAGCCTCGTCTAG	59.9	CAAGCTTGAAGGGAGAGGCA	59.963	103
TRINITY_Contig10508	1	p3	(CTC)4	12	843	854	CTCCAAGCGTTCATCATGCG	59.973	ACCTGTGGCCATGTCTTCTG	59.963	153
TRINITY_Contig10509	1	p6	(GCCGTC)4	24	220	243	ACGGGAGGTAGAGGAAGGAC	60.033	CCTCCCGCAAGTTCATGTCT	60.036	204
TRINITY_Contig10509	2	c	(TCG)4tccgcgccggcgccaccgtggtggccgagcgct(CCG)4	57	580	636	ACGGGAGGTAGAGGAAGGAC	60.033	CCTCCCGCAAGTTCATGTCT	60.036	204
TRINITY_Contig10509	3	c	(CGC)6cccgccgcgcggctgctgcaggtactgcgcgcccgcgttcgccctggacgcccacgccgccgacgccacggacaccgacttccgcctc(GCG)4	118	867	984	ACGGGAGGTAGAGGAAGGAC	60.033	CCTCCCGCAAGTTCATGTCT	60.036	204
TRINITY_Contig10510	1	p4	(GCTT)3	12	964	975	CATCCAAATCGCTGCAGCTC	59.971	CGCCAGCAGAGAGAGATCAG	59.969	234
TRINITY_Contig10510	2	p3	(GAT)4	12	2316	2327	CATCCAAATCGCTGCAGCTC	59.971	CGCCAGCAGAGAGAGATCAG	59.969	234
TRINITY_Contig10510	3	c	(TG)6(TA)14	40	2991	3030	CATCCAAATCGCTGCAGCTC	59.971	CGCCAGCAGAGAGAGATCAG	59.969	234
TRINITY_Contig10511	1	p3	(CGC)4	12	1274	1285	CACCACCCTGAAGCATCCAT	60.034	GGTGCTCTGAGTTCTGCCTT	59.964	208
TRINITY_Contig10512	1	p6	(CCGCTC)3	18	998	1015	GAGAGATTCCTACGCAGCCC	59.967	GGCGCGTGTCTTGAACTTTT	59.971	190
TRINITY_Contig10514	1	c	(CT)6gcg(CGC)7	36	102	137	TCGTCCGGTCCGTTTTGAAT	59.966	TCCTCTGGCTGCCTCTGTAT	60.032	254
TRINITY_Contig10514	2	p3	(CCT)4	12	458	469	TCGTCCGGTCCGTTTTGAAT	59.966	TCCTCTGGCTGCCTCTGTAT	60.032	254
TRINITY_Contig10514	3	p3	(AAT)4	12	4099	4110	TCGTCCGGTCCGTTTTGAAT	59.966	TCCTCTGGCTGCCTCTGTAT	60.032	254
TRINITY_Contig10515	1	p3	(CGG)4	12	294	305	TCTGTAGCACGTCCGTCTA	60.036	TCCTCGTAGAAAAACCGGGC	60.037	146
TRINITY_Contig10516	1	p5	(GAGGG)3	15	1201	1215	GATGATGCTGAGTCGGAGGG	59.967	CTGCTATGCCCCCAGTTTGA	60.034	159
TRINITY_Contig10518	1	p3	(GAG)4	12	137	148	CGAGGATGACGATGAAGGGG	59.967	CAGGGCTGCCAAAATCAACC	60.037	200
TRINITY_Contig10518	2	p3	(TCC)4	12	308	319	CGAGGATGACGATGAAGGGG	59.967	CAGGGCTGCCAAAATCAACC	60.037	200
TRINITY_Contig10521	1	p4	(TTTC)3	12	880	891	GCTTGCCCACTAGTCCCAAT	60.034	TGCAGTTTTCCTCGCAAAGC	59.969	210
TRINITY_Contig10521	2	p3	(CCG)4	12	1028	1039	GCTTGCCCACTAGTCCCAAT	60.034	TGCAGTTTTCCTCGCAAAGC	59.969	210

TRINITY_Contig10523	1	p4	(CGCC)3	12	1163	1174	ATGTCTTGTAGCAGCCGCAT	60.108	ACTCCAGGCAAAGCGTAGAC	60.037	177
TRINITY_Contig10525	1	p3	(CCG)4	12	271	282	GGCAATGCACTTCCCCTACT	60.034	ACACACCCAAGACATCCGTC	59.965	169
TRINITY_Contig10527	1	p3	(TCC)5	15	9	23	GGGAGATCGAGCTGATGTGG	59.967	AAACAGGCGTACATCCAGGG	60.035	259
TRINITY_Contig10527	2	p4	(TCCG)3	12	973	984	GGGAGATCGAGCTGATGTGG	59.967	AAACAGGCGTACATCCAGGG	60.035	259
TRINITY_Contig10527	3	p2	(TA)6	12	1100	1111	GGGAGATCGAGCTGATGTGG	59.967	AAACAGGCGTACATCCAGGG	60.035	259
TRINITY_Contig10530	1	p3	(GCC)4	12	3474	3485	TGCACAGGTTTTTGGTCCCT	60.033	AGTGCTTGTAGAATGCCCCC	60.034	163
TRINITY_Contig10533	1	p6	(GTCGGA)3	18	913	930	GCCTTAGCAGGGCGATATGT	59.965	AGGATGATGATGCCGCTAGC	60.037	162
TRINITY_Contig10535	1	p3	(GGC)7	21	78	98	CCGCCTCCTCAACGATATCC	60.038	GTCCACGAAAGCTAGCTGGT	60.037	253
TRINITY_Contig10536	1	p3	(CAG)4	12	2159	2170	CCACCGGGGATTATGTCGAG	59.966	GAGCAGTACGACGATCCCAG	59.97	176
TRINITY_Contig10538	1	p3	(CGG)4	12	200	211	CGATGACAGGGACGATGAGG	59.969	CTTAACGGTGGCTGACGTCT	60.039	202
TRINITY_Contig10539	1	p3	(GGC)4	12	155	166	TGGTGCGGCAAAGTAGGATT	59.962	GGGTGTCCATGGATCAGCAA	60.034	160
TRINITY_Contig10540	1	c	(TCC)4cgccgccgcaccagcagcgcc(GCG)4gcatcgtcgtgacgcggatgatgctgtcctcccaccgcgaca(GCG)4	99	92	190	ATCGACTTGGCGTTGCTGTA	60.038	ATTCGAAGCAGCTCTGGTCC	60.108	280
TRINITY_Contig10541	1	p6	(TCGGCC)3	18	184	201	TTGCTTGATGCCAGGGTCAT	59.96	GCAGAGGTCTCCCTTCCAAC	60.036	139
TRINITY_Contig10543	1	p3	(CGC)4	12	155	166	ACTCCTCATCTCCTTCCCCC	60.031	CTGGTCGCGGTAGAAGCTAG	59.97	213
TRINITY_Contig10543	2	p6	(AAACAG)3	18	3583	3600	ACTCCTCATCTCCTTCCCCC	60.031	CTGGTCGCGGTAGAAGCTAG	59.97	213
TRINITY_Contig10544	1	p3	(CGG)5	15	669	683	TCACGATAGCCAGCCAACTC	59.824	AAGAGACCGCAACCGCTAAA	59.966	249
TRINITY_Contig10545	1	p3	(CAA)6	18	212	229	AACTGTCTGGACGAAGCTGG	59.966	TACGAGGCCCTCTTCTGTCA	59.961	114
TRINITY_Contig10545	2	p4	(GTGC)3	12	1148	1159	AACTGTCTGGACGAAGCTGG	59.966	TACGAGGCCCTCTTCTGTCA	59.961	114
TRINITY_Contig10547	1	p3	(GCC)5	15	943	957	AGGTCACCGGTCTCGATGTA	60.035	GGTGTGGGTGCAGTTCTACA	59.892	177
TRINITY_Contig10547	2	p3	(GTA)4	12	1310	1321	AGGTCACCGGTCTCGATGTA	60.035	GGTGTGGGTGCAGTTCTACA	59.892	177
TRINITY_Contig10550	1	p3	(CCA)4	12	625	636	GCAACCACTACGACTGACCA	59.967	CCGCTAGCCACAACAGGTTA	60.037	199
TRINITY_Contig10550	2	p2	(TA)8	16	1231	1246	GCAACCACTACGACTGACCA	59.967	CCGCTAGCCACAACAGGTTA	60.037	199
TRINITY_Contig10553	1	p3	(GGC)4	12	897	908	TCATGAGCTCTCCCATGGGA	60.031	GGGTGTGCAAGGAGATTGGA	59.962	101
TRINITY_Contig10553	2	p2	(TC)14	28	1085	1112	TCATGAGCTCTCCCATGGGA	60.031	GGGTGTGCAAGGAGATTGGA	59.962	101
TRINITY_Contig10555	1	p3	(TGG)5	15	1106	1120	GAGGGAGATGAGGATGCTGC	59.965	ACCACCAGCAGCTACAATCC	60.035	245
TRINITY_Contig10556	1	p3	(CGC)6	18	1247	1264	AGGCGGAGTCCTTGAACTTG	59.965	CCCTGCTATTCCTCGCGATT	59.966	183
TRINITY_Contig10557	1	p3	(GCG)7	21	880	900	CCTCCCCTCTGTTTTTCCCC	59.96	TGGGCTCCCGAGAATATCCA	60.105	164
TRINITY_Contig10558	1	p3	(GGC)4	12	466	477	GTTTTTGCTGGGGCTGTAGC	60.038	AAGCGACCGAATGTGGACAT	60.036	228
TRINITY_Contig10558	2	p3	(CGG)4	12	1217	1228	GTTTTTGCTGGGGCTGTAGC	60.038	AAGCGACCGAATGTGGACAT	60.036	228
TRINITY_Contig10559	1	p2	(AT)6	12	100	111	GGTCATCGATGTTTGTGGCG	59.903	CTTCCGGTTGTTCTGGCTCT	59.965	264
TRINITY_Contig10559	2	p3	(CGG)5	15	1126	1140	GGTCATCGATGTTTGTGGCG	59.903	CTTCCGGTTGTTCTGGCTCT	59.965	264
TRINITY_Contig10559	3	p3	(GGC)5	15	1367	1381	GGTCATCGATGTTTGTGGCG	59.903	CTTCCGGTTGTTCTGGCTCT	59.965	264
TRINITY_Contig10559	4	p3	(CGC)5	15	2318	2332	GGTCATCGATGTTTGTGGCG	59.903	CTTCCGGTTGTTCTGGCTCT	59.965	264
TRINITY_Contig10560	1	p4	(GGGC)3	12	865	876	TTTAGCTCCGCCTCCCAAAG	60.035	GAAGCAAGCGGGCGAATAAG	59.972	208
TRINITY_Contig10560	2	c	(GA)15ggctggtgccatggaccgtcgggactcggaggcgagcgcac(CGGCCG)3cggcggggcgcgccgcgagcggcgacgggacc(GCG)4	133	1110	1242	TTTAGCTCCGCCTCCCAAAG	60.035	GAAGCAAGCGGGCGAATAAG	59.972	208
TRINITY_Contig10560	3	p3	(GAG)4	12	1525	1536	TTTAGCTCCGCCTCCCAAAG	60.035	GAAGCAAGCGGGCGAATAAG	59.972	208
TRINITY_Contig10563	1	c	(GAG)4gaagctcgaattgaagtgttgagcagttgatggatgcaccgaagtggctcggcagtggcgccata(TATGC)3	92	384	475	CAGAGGAGGAGGAGGAAGCT	60.032	AAGAGGTAGGGGGCATAGCA	60.03	106
TRINITY_Contig10563	2	p3	(TGG)4	12	788	799	CAGAGGAGGAGGAGGAAGCT	60.032	AAGAGGTAGGGGGCATAGCA	60.03	106
TRINITY_Contig10563	3	p3	(CAA)4	12	912	923	CAGAGGAGGAGGAGGAAGCT	60.032	AAGAGGTAGGGGGCATAGCA	60.03	106
TRINITY_Contig10563	4	c	(TTC)8cttcatgtttgaatcttcattcgtatgttaatcagcagttgtattctctctgcagtagtactaccgtacctcctctcctgtatcagctgtaagttatat(TAGC)3	135	1062	1196	CAGAGGAGGAGGAGGAAGCT	60.032	AAGAGGTAGGGGGCATAGCA	60.03	106
TRINITY_Contig10564	1	p2	(GA)8	16	1301	1316	AGGTGCTTCGACGACTTGAG	60.039	AGTCCACAACAATGCAACGC	59.969	193
TRINITY_Contig10565	1	p6	(CGGAGC)3	18	831	848	GAGGGAGGAGGTGGAGATGT	60.032	GAGGCAGAATCCAAACACGC	59.831	178
TRINITY_Contig10565	2	p6	(GGAGGT)3	18	1253	1270	GAGGGAGGAGGTGGAGATGT	60.032	GAGGCAGAATCCAAACACGC	59.831	178
TRINITY_Contig10567	1	p3	(CGG)5	15	820	834	ATCTGGCCGTTGTGGAAGAG	60.036	ACGAGTATGTCTGCGAGCTG	59.9	115
TRINITY_Contig10568	1	p4	(CTAG)3	12	35	46	TGCTATGTGCTATCCAGGCG	59.966	GGAAACCGAAGATGCCAGGA	60.035	147
TRINITY_Contig10568	2	p6	(GGCGCG)3	18	1710	1727	TGCTATGTGCTATCCAGGCG	59.966	GGAAACCGAAGATGCCAGGA	60.035	147
TRINITY_Contig10569	1	p3	(GCG)6	18	73	90	GGCAGGAGAATGGACAAGCT	60.035	GGATCAGCGCCATGATCTCA	59.966	200
TRINITY_Contig10569	2	p3	(AGC)6	18	287	304	GGCAGGAGAATGGACAAGCT	60.035	GGATCAGCGCCATGATCTCA	59.966	200
TRINITY_Contig10571	1	p3	(GCA)4	12	324	335	TCTCAGTGGTCGAGTTGCAC	59.968	AATGGCTTCCCGTACCCTTG	60.034	179
TRINITY_Contig10574	1	p3	(ACC)4	12	301	312	GTTGCAGCTATCAGAACGCG	59.974	TGCACGTCACCATCGTACAA	59.968	140
TRINITY_Contig10575	1	p3	(GGC)4	12	261	272	GGCACATGGTTCCAGAAGGA	59.962	GCTACATTTCACACCGGGGA	60.036	267
TRINITY_Contig10576	1	p3	(CCG)4	12	283	294	GAGGGAATGTTGGGACCTGG	60.034	CTTCCCCTACCTCTCGTCCA	60.033	234
TRINITY_Contig10578	1	p3	(GCG)4	12	450	461	ACGGAAGCGTAGTTGATGCA	60.038	CTCGATGGATCTGGGCAGAC	59.967	108
TRINITY_Contig10578	2	p3	(GAG)4	12	1068	1079	ACGGAAGCGTAGTTGATGCA	60.038	CTCGATGGATCTGGGCAGAC	59.967	108
TRINITY_Contig10579	1	p3	(GGC)4	12	151	162	GTGTACAAGGTGATCGCCGA	60.109	GGGCAGAAGGTCGATTCGAT	59.895	100
TRINITY_Contig10579	2	c	(GCA)6gaacgcca(CCG)4	38	549	586	GTGTACAAGGTGATCGCCGA	60.109	GGGCAGAAGGTCGATTCGAT	59.895	100
TRINITY_Contig10579	3	p2	(GC)6	12	755	766	GTGTACAAGGTGATCGCCGA	60.109	GGGCAGAAGGTCGATTCGAT	59.895	100
TRINITY_Contig10583	1	c	(GCCAGC)3cccggtccccgcgcccatgccgccggtgccggcgccggtgcctgctcctgtgcccgcgccgccgccggtgccggcgcctgtcccgccggtgcccat(GCC)4	126	82	207	GCATCAGAGCTCTGCAGACA	60.108	ATTTGTTGGAGCCGGGGATT	59.959	123
TRINITY_Contig10583	2	p2	(AG)19	38	503	540	GCATCAGAGCTCTGCAGACA	60.108	ATTTGTTGGAGCCGGGGATT	59.959	123
TRINITY_Contig10583	3	c	(CAG)4gagaagcaggggagccgccatggctgcacgagctcgagctccctggactggaccggaccgcgagcgaactcta(GAGC)5	105	929	1033	GCATCAGAGCTCTGCAGACA	60.108	ATTTGTTGGAGCCGGGGATT	59.959	123
TRINITY_Contig10584	1	p6	(GGCTGC)3	18	1152	1169	GAGGACGGAGATCCTCTCGT	60.179	CTCGGGAAGCTCCTACTCCT	60.106	218
TRINITY_Contig10585	1	p3	(GCC)4	12	898	909	GAGTGAAGAGACAGTGGGCC	60.037	AACACCAAATTGCATCCGCC	60.037	194
TRINITY_Contig10587	1	p3	(GTC)4	12	1137	1148	CACTCCCATTTCTCCCCACC	60.034	AAGAGGCTCACTTTCCGCTC	60.037	136
TRINITY_Contig10588	1	p4	(CAAG)3	12	326	337	CACACTAGGTGCTGGCCTAC	60.108	AATTGTTCCGGTTTGCCACG	59.969	254
TRINITY_Contig10590	1	p5	(GAGCA)3	15	95	109	TCTCGAGGGATCGATGACCA	59.817	GGACAGAAGAGTTCACGGGG	60.037	165
TRINITY_Contig10590	2	p3	(CCT)4	12	465	476	TCTCGAGGGATCGATGACCA	59.817	GGACAGAAGAGTTCACGGGG	60.037	165
TRINITY_Contig10590	3	p3	(CGG)4	12	877	888	TCTCGAGGGATCGATGACCA	59.817	GGACAGAAGAGTTCACGGGG	60.037	165
TRINITY_Contig10591	1	p3	(TGG)4	12	2505	2516	TCCTCATCCTCTTCCCCCTG	60.031	CAAACGCTGCGGAGATAACG	59.975	254
TRINITY_Contig10591	2	p4	(TGAA)3	12	3812	3823	TCCTCATCCTCTTCCCCCTG	60.031	CAAACGCTGCGGAGATAACG	59.975	254
TRINITY_Contig10592	1	p3	(GCC)4	12	623	634	GCCCCGATAATCATCAGCGA	60.037	GATCTCCCATGCAGAGGTCG	59.967	205
TRINITY_Contig10592	2	p4	(AGCT)3	12	900	911	GCCCCGATAATCATCAGCGA	60.037	GATCTCCCATGCAGAGGTCG	59.967	205
TRINITY_Contig10594	1	p3	(CCG)5	15	155	169	ATCTCCCGGTCGTTCGAGTA	60.108	GCGTACTGGTACTGGCAGTT	60.038	275
TRINITY_Contig10594	2	p3	(CGG)5	15	912	926	ATCTCCCGGTCGTTCGAGTA	60.108	GCGTACTGGTACTGGCAGTT	60.038	275
TRINITY_Contig10597	1	p3	(GCG)6	18	7	24	GAGGACACCACCACCAAGAG	59.965	TTTCTTCCACGTCCACCTCG	59.967	196
TRINITY_Contig10597	2	p3	(CGA)4	12	268	279	GAGGACACCACCACCAAGAG	59.965	TTTCTTCCACGTCCACCTCG	59.967	196
TRINITY_Contig10598	1	p3	(GCG)4	12	703	714	TGTTGGACGTGAACGAGGAG	59.968	TCGTGTGGGTGGTTGATGAG	59.965	169
TRINITY_Contig10599	1	p3	(CCG)4	12	667	678	AATCGAAGCACACTGGGGAG	60.036	AGCAGCAGCCCATTTCTTCT	59.961	129
TRINITY_Contig10601	1	p2	(TG)6	12	1150	1161	TTCCGGAACAGGAAGTACGC	60.038	CGAGGCGAAACTCATGCATG	59.973	202
TRINITY_Contig10605	1	p3	(GAG)4	12	502	513	AGATCACCAAGGACGGCAAG	60.036	AAGCCCAAGTCCGTGATGAG	60.036	109
TRINITY_Contig10607	1	p3	(GCG)4	12	906	917	GCACCACGTGACAAAGTTCC	59.971	CACCACCAAGTGCCTCTCAT	59.963	225
TRINITY_Contig10608	1	c	(ACCA)3gccacagccaacgcccgccccgagcaagagtaacgttgggggaacggggggcggtggcatggcaccgcgccaccgctcgcc(GCTG)3	105	35	139	CACGGCAATGCAGGAAAACA	59.968	ATCGGTATCACGAACTCGGC	59.97	251
TRINITY_Contig10608	2	p3	(GAG)4	12	909	920	CACGGCAATGCAGGAAAACA	59.968	ATCGGTATCACGAACTCGGC	59.97	251
TRINITY_Contig10608	3	p3	(GGA)4	12	1079	1090	CACGGCAATGCAGGAAAACA	59.968	ATCGGTATCACGAACTCGGC	59.97	251
TRINITY_Contig10608	4	p3	(TCC)4	12	2930	2941	CACGGCAATGCAGGAAAACA	59.968	ATCGGTATCACGAACTCGGC	59.97	251
TRINITY_Contig10608	5	p4	(GAGC)3	12	3686	3697	CACGGCAATGCAGGAAAACA	59.968	ATCGGTATCACGAACTCGGC	59.97	251
TRINITY_Contig10608	6	c	(AGCC)3agcggcgagcggtggcgcggtgccatgccaccgcccccgttcccccaacgttactcttgctcggggcgggcgttggctgtggc(TGGT)3	108	3868	3975	CACGGCAATGCAGGAAAACA	59.968	ATCGGTATCACGAACTCGGC	59.97	251
TRINITY_Contig10610	1	p3	(CCG)4	12	70	81	ATAGCTCGATTGCAGGTCGG	59.967	AGAACGGACAAGGGAAGCTG	59.965	216
TRINITY_Contig10612	1	p3	(GCA)4	12	431	442	CTGCGCAGATGAAGGCATTC	59.971	TTCCATGGTGCACTAGCTGG	60.035	159
TRINITY_Contig10613	1	p3	(CAG)7	21	288	308	AATGGGGAGAGGTCGTCAGA	59.959	GGAGATTAAGACGCCCTGGG	59.893	196
TRINITY_Contig10613	2	p4	(TTGA)3	12	1164	1175	AATGGGGAGAGGTCGTCAGA	59.959	GGAGATTAAGACGCCCTGGG	59.893	196
TRINITY_Contig10614	1	p3	(TCC)4	12	66	77	CTATAACCGATGGCGCTCGT	60.039	AGAGCTCGGTCAGAGGCTAA	60.034	149
TRINITY_Contig10621	1	p3	(GTA)4	12	1272	1283	GCAGCCAGAAGAAGCTACCA	60.036	CCCTGTTTGGTGTGACGGTA	59.893	148
TRINITY_Contig10623	1	p6	(TACGAG)3	18	205	222	TTCATCTCCAAGGTGCGCTT	59.963	CCTCTGGATGTCGTCGATGG	59.969	210
TRINITY_Contig10623	2	p3	(GTG)4	12	1632	1643	TTCATCTCCAAGGTGCGCTT	59.963	CCTCTGGATGTCGTCGATGG	59.969	210

TRINITY_Contig10626	1	p5	(CAGTA)3	15	131	145	GGAAGCCATAGGTGTCGGAG	59.895	GGGGAATCGTCAGAGCGTAG	59.969	150
TRINITY_Contig10626	2	p2	(GC)7	14	1211	1224	GGAAGCCATAGGTGTCGGAG	59.895	GGGGAATCGTCAGAGCGTAG	59.969	150
TRINITY_Contig10627	1	p3	(GGA)4	12	161	172	TCTTCTCCTCCACACACCCA	60.105	TGACGACTCCTCCGATTCCT	60.034	177
TRINITY_Contig10627	2	p6	(CACGGG)3	18	319	336	TCTTCTCCTCCACACACCCA	60.105	TGACGACTCCTCCGATTCCT	60.034	177
TRINITY_Contig10627	3	p3	(GCG)5	15	460	474	TCTTCTCCTCCACACACCCA	60.105	TGACGACTCCTCCGATTCCT	60.034	177
TRINITY_Contig10627	4	p3	(GCG)5	15	881	895	TCTTCTCCTCCACACACCCA	60.105	TGACGACTCCTCCGATTCCT	60.034	177
TRINITY_Contig10628	1	p4	(GATC)3	12	1	12	GATCTTTGATGTCGCACGCC	59.973	CAGTGTATGCTGGGCCAGAA	60.035	135
TRINITY_Contig10628	2	p4	(CCGC)3	12	171	182	GATCTTTGATGTCGCACGCC	59.973	CAGTGTATGCTGGGCCAGAA	60.035	135
TRINITY_Contig10628	3	p3	(CGA)4	12	764	775	GATCTTTGATGTCGCACGCC	59.973	CAGTGTATGCTGGGCCAGAA	60.035	135
TRINITY_Contig10628	4	p3	(GGA)4	12	4279	4290	GATCTTTGATGTCGCACGCC	59.973	CAGTGTATGCTGGGCCAGAA	60.035	135
TRINITY_Contig10629	1	p3	(CTG)5	15	884	898	GCGCCTTTTCTTTGACTGCA	59.969	CGTCACACTGTCTGGCTTCT	59.967	148
TRINITY_Contig10630	1	p6	(TCCGCG)3	18	247	264	GCTGGAGTGCTATGTGGGTT	60.035	TACTGGCTCTGTTACCCGGA	59.96	203
TRINITY_Contig10631	1	p4	(AACC)3	12	1293	1304	GAAGCTTCAGCCCTCCCAAT	60.034	GATGCAATCTGACCCCCACA	60.034	270
TRINITY_Contig10633	1	c	(CCG)4cctcgcgtccccgtgccgccgcagcg gcga(CGCCTC)3	60	422	481	GTCATCAGGACGGGGAGTTG	60.108	GTGTGAGCTGTGACGATCGA	60.11	117
TRINITY_Contig10633	2	p3	(CGG)4	12	664	675	GTCATCAGGACGGGGAGTTG	60.108	GTGTGAGCTGTGACGATCGA	60.11	117
TRINITY_Contig10633	3	p4	(GCGT)3	12	1156	1167	GTCATCAGGACGGGGAGTTG	60.108	GTGTGAGCTGTGACGATCGA	60.11	117
TRINITY_Contig10635	1	p4	(CAAA)3	12	2935	2946	CATAGCCATTTCGTGCCTGC	59.97	CTCGGCCAGGAATAGCTCAG	59.967	182
TRINITY_Contig10635	2	p5	(AAAAG)5	25	3178	3202	CATAGCCATTTCGTGCCTGC	59.97	CTCGGCCAGGAATAGCTCAG	59.967	182
TRINITY_Contig10636	1	p3	(CCG)4	12	304	315	GAGGAAGAAGACCTTGGGCC	60.035	GGATTTGTGTGGTTGGGTGC	59.967	193
TRINITY_Contig10636	2	p3	(CGA)4	12	643	654	GAGGAAGAAGACCTTGGGCC	60.035	GGATTTGTGTGGTTGGGTGC	59.967	193
TRINITY_Contig10638	1	p4	(AGCT)3	12	28	39	CTAGACGACGACGAGGAAGC	59.973	GACCCGTTCAAATGATGGCG	59.901	112
TRINITY_Contig10638	2	p3	(CTC)7	21	609	629	CTAGACGACGACGAGGAAGC	59.973	GACCCGTTCAAATGATGGCG	59.901	112
TRINITY_Contig10643	1	p3	(ATC)4	12	721	732	GGCTCGTCCTATGGCAGTAC	59.968	TGAATCTCGCAGCCCTGATC	59.894	118
TRINITY_Contig10644	1	c	(TTCT)3tcaaacagcttttcttggcacaaatt taatttactcaaaggatttcacttctttctctacc aattg(CCTA)3	90	82	171	GGGTCGAGGGTCTTGTTGAG	60.037	CTTCGTCTCCATGCTCCTCC	59.896	201
TRINITY_Contig10644	2	c	(GAG)4catggagacgaagggggagcgcgag gaggagcgggcgcgcgggcagggccgccgc gcgcgggggcttggggtccgcgggcat(GGC)4gcggcgcggtggtggttagggacagggcg gcggccgcatg(GCGGT)3	160	1077	1236	GGGTCGAGGGTCTTGTTGAG	60.037	CTTCGTCTCCATGCTCCTCC	59.896	201
TRINITY_Contig10645	1	p3	(GGC)4	12	1170	1181	CTTCGAGTACCACCACCACC	60.038	CAGGACATATGCGCCAGCTA	59.966	177
TRINITY_Contig10645	2	p3	(ACC)4	12	1930	1941	CTTCGAGTACCACCACCACC	60.038	CAGGACATATGCGCCAGCTA	59.966	177
TRINITY_Contig10646	1	p3	(CGC)4	12	998	1009	CGGGAAGACCTCGATGATGG	59.967	ATCGAGCGAGATGAGCAAGG	59.968	108
TRINITY_Contig10647	1	p5	(CAGCT)3	15	147	161	GCTCGGACCAAAGGAATCCA	60.035	CTCAACCAGTCGTCCACTCC	60.038	158
TRINITY_Contig10647	2	p5	(TTCCG)3	15	350	364	GCTCGGACCAAAGGAATCCA	60.035	CTCAACCAGTCGTCCACTCC	60.038	158
TRINITY_Contig10647	3	p3	(CGG)6	18	616	633	GCTCGGACCAAAGGAATCCA	60.035	CTCAACCAGTCGTCCACTCC	60.038	158
TRINITY_Contig10647	4	p6	(CCGCGC)3	18	859	876	GCTCGGACCAAAGGAATCCA	60.035	CTCAACCAGTCGTCCACTCC	60.038	158
TRINITY_Contig10647	5	p4	(GGTC)3	12	1102	1113	GCTCGGACCAAAGGAATCCA	60.035	CTCAACCAGTCGTCCACTCC	60.038	158
TRINITY_Contig10648	1	c	(AGG)4acggggaggagcttgcggctgcctc gctggctggagttcgttgttggtcaacgggagg agaggagagtggagaggagagc(GAGG)3	104	1050	1153	TCAACGGGAGGAGAGGAGAG	60.034	AGATCACAGCTCGCACCAAA	59.965	108
TRINITY_Contig10649	1	p3	(GCG)4	12	316	327	GACTAGCTAGCCGTTCCGTC	59.971	GCCCTACAATGACCTGCCAT	60.107	220
TRINITY_Contig10649	2	p3	(GCA)6	18	714	731	GACTAGCTAGCCGTTCCGTC	59.971	GCCCTACAATGACCTGCCAT	60.107	220
TRINITY_Contig10650	1	p4	(TCCT)3	12	1796	1807	AGCAAGACTGTCGTGGTCAG	59.967	AATTGGAGGTGGCTACTGGC	60.034	219
TRINITY_Contig10651	1	p6	(GTGTAT)4	24	1043	1066	CATGGAGACCATCGACTCGG	59.969	CTCGCTGCTCATCTCCTCAG	59.969	131
TRINITY_Contig10652	1	p5	(CGCGC)3	15	14	28	GGCAGTGAATGTTGCACGTT	59.969	GCTCAACCATGGCCTTCTCT	60.035	257
TRINITY_Contig10656	1	p3	(CCT)4	12	983	994	CCTCGCCAAACTTGAAGTGC	60.04	AGAATCTCGGGCGCCTAATG	59.966	256
TRINITY_Contig10656	2	p5	(GCGGG)3	15	1135	1149	CCTCGCCAAACTTGAAGTGC	60.04	AGAATCTCGGGCGCCTAATG	59.966	256
TRINITY_Contig10657	1	p3	(TGT)4	12	667	678	TTGTTGTCGTCGGAGGTCAG	59.968	CCAGCCATCGTCATCCTCTC	59.967	101
TRINITY_Contig10658	1	p3	(GCA)7	21	388	408	GGACAAAGAGCGCTTGCAAA	59.969	TGGCTGCAGATAACATGGCA	60.034	234
TRINITY_Contig10658	2	p4	(TGTT)3	12	2757	2768	GGACAAAGAGCGCTTGCAAA	59.969	TGGCTGCAGATAACATGGCA	60.034	234
TRINITY_Contig10659	1	p6	(GAACTC)3	18	1042	1059	TAGGCGTTCACACTTGGTCC	59.966	CAGCACCCGTTCTGACTTCT	59.966	277
TRINITY_Contig10660	1	p3	(CCT)6	18	286	303	ACCTGCCTCTCCATGCTAGA	60.032	CTAGAGCGGCTAGGACAACG	59.97	270
TRINITY_Contig10661	1	p3	(GCT)4	12	377	388	ATGGAAGGTGTGCTCCATGG	60.034	ATCTCCCAGCAGCTGACCTA	60.032	177
TRINITY_Contig10662	1	p4	(CCGA)3	12	149	160	GACGTAGGAGAAGGCGATGG	59.969	TCACGAAGGTTGGGACATGG	59.964	131
TRINITY_Contig10665	1	c	(CGAT)4ggtttttccgggggcaggagaggg ttgcgtgtttcttcggcgcgtgagcttt(TTC)5	83	804	886	TTGTTGCGAGAGACACCCAA	59.821	CATGCGTTTTGTGCTTCCCA	59.968	162
TRINITY_Contig10667	1	c	(GCCG)3ccgccgcgcgccggttg(TGC)5	44	515	558	AGAGATCGATCGAGCGGGTA	59.965	CCGTGGTGAAGCTTTTGGTG	59.969	182
TRINITY_Contig10668	1	p3	(AGC)5	15	4307	4321	TCCGGATGAATCCTCCCCTT	60.03	GCTTTGCAGTTGGAAGCGAA	59.969	126
TRINITY_Contig10669	1	p4	(CAGC)3	12	1	12	ATGGTGGTGGCATCGATTGT	60.034	CGATGACGCACTGATCGGTA	59.971	270
TRINITY_Contig10669	2	p3	(GCG)4	12	161	172	ATGGTGGTGGCATCGATTGT	60.034	CGATGACGCACTGATCGGTA	59.971	270
TRINITY_Contig10670	1	p6	(GTGTAT)3	18	1051	1068	CATGGAGACCATCGACTCGG	59.969	CTCGCTGCTCATCTCCTCAG	59.969	148
TRINITY_Contig10671	1	c	(GCG)6atggggagcgacacggagacag(A GA)4	52	1200	1251	GAGCCGATCTGGAAGTCGAG	59.97	TGGGAAATTCTCCTCCGTGC	60.035	156
TRINITY_Contig10673	1	p3	(ATG)4	12	1043	1054	GAACCAAGGAGAAGACGGCA	59.965	GAGACTTGTTCAGCACGGGA	59.966	236
TRINITY_Contig10680	1	c	(AGGG)3gttgggggacgtcagggcttggct gccttctctgccttgccttctccctccaatccag gcgctaagaaatactata(CGG)4	100	151	250	GCCCAAGAAGGACGATGGAA	60.035	CTTGACGGTGGCCTTCATCT	60.036	203
TRINITY_Contig10680	2	p4	(GAAT)3	12	1214	1225	GCCCAAGAAGGACGATGGAA	60.035	CTTGACGGTGGCCTTCATCT	60.036	203
TRINITY_Contig10681	1	p4	(AGGC)3	12	30	41	GTCGGCTTCTTCGAGAGGAG	59.9	GGTCTTGTTCGCCATCCAGA	60.036	210
TRINITY_Contig10684	1	c	(TC)14ttcctctcgctctcgtcacaatgctctc gcgcctgctgcgccccgccggccgctcgctcgc ggt(CGCGC)3	107	43	149	CTCGAAGGGTCTCTGGGAGA	60.034	TGATATCACCGGCCGACATG	59.967	209
TRINITY_Contig10685	1	p3	(AGG)4	12	534	545	CTGCGGAGGAAGAGGCTATG	59.967	CGACCCCAGCCTTTTGATCT	60.035	274
TRINITY_Contig10688	1	p3	(CGC)4	12	175	186	GCCTGACGGTTGGGAAACTA	59.964	ACGCCTCCGACTGAAAATGT	59.965	163
TRINITY_Contig10688	2	p3	(CGG)5	15	625	639	GCCTGACGGTTGGGAAACTA	59.964	ACGCCTCCGACTGAAAATGT	59.965	163
TRINITY_Contig10689	1	p3	(CGG)6	18	2263	2280	GAGATGCAGCACCTCAACCT	60.036	GCCGAAGTCGTCAGGGATAG	59.969	213
TRINITY_Contig10691	1	p4	(CCCG)3	12	49	60	GGAGAGCTCGTTGGTGAACA	59.966	CGTCGTACCCGATGTAGCTC	60.041	209
TRINITY_Contig10691	2	p3	(CGT)4	12	1049	1060	GGAGAGCTCGTTGGTGAACA	59.966	CGTCGTACCCGATGTAGCTC	60.041	209
TRINITY_Contig10692	1	p2	(GC)6	12	189	200	ATCTGGATCAGGCAGCATCG	59.965	TGACACCTCTTCTGGTTCGC	59.966	274
TRINITY_Contig10693	1	p3	(GGC)5	15	324	338	CTATTTGAAGCGGGGCATGC	59.969	TGCTCCAGAGACCCCTAGAC	60.033	156
TRINITY_Contig10694	1	p3	(CCG)4	12	713	724	GCTTCATGCTGGCGATGATG	60.04	CGATCGTCTTCCTTCTCCCG	59.97	180
TRINITY_Contig10694	2	p6	(GACGCG)3	18	877	894	GCTTCATGCTGGCGATGATG	60.04	CGATCGTCTTCCTTCTCCCG	59.97	180
TRINITY_Contig10695	1	p3	(GAG)4	12	1	12	CGACGTATCCTGTGGTCTGG	59.899	GGCACAAAGCTTTCTCTGCC	60.039	228
TRINITY_Contig10697	1	p3	(TGG)4	12	140	151	TTCCCAGGCAGTGATCCAAC	59.962	TCGACTGCATATACCGTGGC	59.968	115
TRINITY_Contig10697	2	p3	(GGC)4	12	997	1008	TTCCCAGGCAGTGATCCAAC	59.962	TCGACTGCATATACCGTGGC	59.968	115
TRINITY_Contig10698	1	c	(CCG)5ttcaccgggagcaccactcccccgcc gtgcatctctgcagctctcctcctctacgagctc tggtgatcgacgga(GATC)3	101	1375	1475	AGGCCTCCAGGAAAGAATGC	60.034	CGATCTCCGTCGATCACCAG	60.041	240
TRINITY_Contig10699	1	c	(CACG)4agcgcgccagcaccaccacgccac cactga(GCTCC)3	61	105	165	CAAAGCTGCTCAACCACCAC	59.969	TCCTTTTCCTTTCCGGAGCC	59.962	160
TRINITY_Contig10699	2	p3	(GCG)5	15	368	382	CAAAGCTGCTCAACCACCAC	59.969	TCCTTTTCCTTTCCGGAGCC	59.962	160
TRINITY_Contig10699	3	p3	(CCG)5	15	693	707	CAAAGCTGCTCAACCACCAC	59.969	TCCTTTTCCTTTCCGGAGCC	59.962	160
TRINITY_Contig10700	1	p3	(GGC)4	12	1112	1123	TTGGGAGCCACAACAAGGTT	60.033	CCCCATTGTTGACTGAGGCT	59.961	119
TRINITY_Contig10703	1	p3	(CGG)4	12	1016	1027	AGTCAGGCAAGCCAAGGATC	60.035	TTGTGTGAGCTAGGTGGCTG	59.965	238
TRINITY_Contig10703	2	p6	(CGTCGC)3	18	1214	1231	AGTCAGGCAAGCCAAGGATC	60.035	TTGTGTGAGCTAGGTGGCTG	59.965	238
TRINITY_Contig10706	1	p3	(CGC)4	12	1483	1494	AGTCTCAGCATCGCATACGG	59.969	GTGCGGTGCATGATGTTGTT	60.039	168
TRINITY_Contig10707	1	p3	(GGA)4	12	1031	1042	TGGTGTCGGTGAAGTACTGC	59.967	ACTTCGGCTTTGATCCCTGG	60.035	198
TRINITY_Contig10709	1	p3	(CCG)6	18	1072	1089	GGCTTGAACTAGGACACCCC	60.035	TACGAGTTCAAACCCGCTCC	60.038	276
TRINITY_Contig10709	2	p3	(CGG)6	18	1199	1216	GGCTTGAACTAGGACACCCC	60.035	TACGAGTTCAAACCCGCTCC	60.038	276
TRINITY_Contig10711	1	c	(GAGG)4gagacgtcgcctgcgatgacacc ggcctcacccgccgccgacaccgttatcatcgc gagcgagcag(AGC)6	100	48	147	ACGAGGGCTGTTCCATTAGC	60.108	CAAGCTTTCACCCACGAAGC	60.04	238
TRINITY_Contig10711	2	c	(CG)6gccaaggaa(GGC)5	36	467	502	ACGAGGGCTGTTCCATTAGC	60.108	CAAGCTTTCACCCACGAAGC	60.04	238
TRINITY_Contig10713	1	c	(GTG)4cccggtcggacccggcgggcggcgc (TCGCG)3	52	399	450	GCTTCAGCCGCTTTGAACAA	59.969	CAAGCTTGCTACCAGACCGA	60.037	100
TRINITY_Contig10714	1	p2	(AT)11	22	37	58	CCTACTTCGACGAGTACCGC	59.973	CGTAGCTGGGGATGTCGTAC	59.97	168
TRINITY_Contig10719	1	p2	(GA)8	16	252	267	CTCCAACACACACGCACATG	60.041	TCCCCTTCCAGATAGCTGCT	60.031	257
TRINITY_Contig10719	2	c	(GGC)5gggac(AGG)4	32	416	447	CTCCAACACACACGCACATG	60.041	TCCCCTTCCAGATAGCTGCT	60.031	257

TRINITY_Contig10726	1	c	(GCC)4ggcggcgaccccggagctcctccgcgccgaggacgaggatagcggcttcgccggcggcgcggtggtggggact(GCC)4	97	290	386	ACTTGATCACGCCGGAGAAG	60.109	AATCAAGAGCGGCTACCACC	60.108	161
TRINITY_Contig10726	2	p6	(GCAGTG)4	24	1194	1217	ACTTGATCACGCCGGAGAAG	60.109	AATCAAGAGCGGCTACCACC	60.108	161
TRINITY_Contig10727	1	p3	(GCG)4	12	352	363	GCATGGAGAACAAGGAGGCT	60.035	AACCTCCCGGTCTCTACCTC	60.033	261
TRINITY_Contig10727	2	p3	(ACC)4	12	1119	1130	GCATGGAGAACAAGGAGGCT	60.035	AACCTCCCGGTCTCTACCTC	60.033	261
TRINITY_Contig10730	1	p4	(AGCG)3	12	138	149	TACCCGGCCTACTTCGAGAT	59.816	ATCTACGACGGCAGCGTATG	60.04	171
TRINITY_Contig10730	2	p2	(TC)11	22	1001	1022	TACCCGGCCTACTTCGAGAT	59.816	ATCTACGACGGCAGCGTATG	60.04	171
TRINITY_Contig10733	1	p3	(GGC)4	12	1081	1092	GGGTGGAGACGTCGATCATC	59.969	CAACCCTTGCAGCCTTGAAC	59.967	153
TRINITY_Contig10734	1	p6	(GGCGAG)3	18	1005	1022	CTACCAGTCTCGATGGCGTC	59.97	CGTGATCAAGCTGATGCACG	59.974	186
TRINITY_Contig10735	1	p3	(TCG)4	12	486	497	TGCCCTCGCAATCAGGAATT	60.034	TTCCGTCCAGAACCGTGAAG	59.967	205
TRINITY_Contig10735	2	p4	(CCAG)3	12	1211	1222	TGCCCTCGCAATCAGGAATT	60.034	TTCCGTCCAGAACCGTGAAG	59.967	205
TRINITY_Contig10736	1	p4	(GGCA)3	12	1165	1176	TTCGGCTTCCAGTTGGAGAC	59.965	TGAGAGATGTGCCGTTTGCT	59.965	129
TRINITY_Contig10737	1	p3	(CGC)4	12	201	212	GGTCCCGATCCGCATAAAGT	59.894	GCAACTGTTGGATCGGATGC	59.901	124
TRINITY_Contig10738	1	p3	(GTA)4	12	82	93	GCATTCGCCATCATCATCGG	59.833	GGTCCCGAACTTCTGATGCA	60.036	103
TRINITY_Contig10738	2	c	(CGG)5ccggttgaagaggtaccccttgggctgctcctcgtgccccggggtcgtccaccgcccggccccct(CGC)4	92	509	600	GCATTCGCCATCATCATCGG	59.833	GGTCCCGAACTTCTGATGCA	60.036	103
TRINITY_Contig10740	1	p3	(CCT)4	12	281	292	GGTTTCGCTTGGGGATCAGA	60.035	CGGTATGCATTTGGCTCGTG	59.972	209
TRINITY_Contig10740	2	p3	(GAC)4	12	519	530	GGTTTCGCTTGGGGATCAGA	60.035	CGGTATGCATTTGGCTCGTG	59.972	209
TRINITY_Contig10742	1	p3	(GCC)5	15	406	420	TCATTCACCTTGGGTCCTGC	59.962	AGCTAGGCCGCATTTTCACT	60.035	121
TRINITY_Contig10742	2	c*	(GCG)3(GCGCCGGCGCCGGCGCGACCTGCTGCTGCGGCTGCGCCTGTGCCAGCGGCTGCACATGGTGCTGCTGCGCGTCGCCGCCCGGCGCCGAGGACGGCCCGGCTGGCCCCGGGGGATCCATGCAGCTCGCTAGTCTCTAGCTAGCTCACCCGGCCGGCCGGGGCCGCGGCCGGCTCCCAGTGGAGACGCGCGGCACCAGGCAGGCACCGTCCGGGGCGCGGGTGGGAGAGCGACGGGGCGGGGTGGAGTGGAGGAAAGGGAGGGAGGTGAAC<CGGCG>C)(CGGCGC)2	25	944	968	TCATTCACCTTGGGTCCTGC	59.962	AGCTAGGCCGCATTTTCACT	60.035	121
TRINITY_Contig10742	3	p4	(CCGG)3	12	1099	1110	TCATTCACCTTGGGTCCTGC	59.962	AGCTAGGCCGCATTTTCACT	60.035	121
TRINITY_Contig10743	1	p5	(GCGGC)3	15	1192	1206	AAGTGACCTCAGCTTGTCCG	59.966	TGAGGATCAGGCGTACAGGA	60.034	112
TRINITY_Contig10748	1	p3	(GCG)4	12	305	316	TCTCGTGCGGAATGAAACCA	59.966	TGTACCCAGCAATCCGCAAT	60.035	273
TRINITY_Contig10748	2	p4	(ACAT)3	12	967	978	TCTCGTGCGGAATGAAACCA	59.966	TGTACCCAGCAATCCGCAAT	60.035	273
TRINITY_Contig10750	1	p3	(GCC)5	15	246	260	CTCCGTGACGAACCTCACTC	60.11	AACTTGGTAGGCTGCTCGTC	60.037	254
TRINITY_Contig10750	2	p3	(CCA)4	12	693	704	CTCCGTGACGAACCTCACTC	60.11	AACTTGGTAGGCTGCTCGTC	60.037	254
TRINITY_Contig10752	1	p3	(GAC)4	12	4610	4621	CAGCCGCGATTGACTTCATG	59.973	CTTGCATCAGTCATCGCTGC	59.972	181
TRINITY_Contig10755	1	p2	(AG)6	12	977	988	CTTCTCCCTCCTCTCCTCCC	60.106	AGGTGGAGCAGAGGAGAGAG	60.033	152
TRINITY_Contig10757	1	c	(GCC)4ggaagcgggggaacc(GGA)4	39	901	939	ATTCTGATCTGAGCTCGCCG	59.968	AGGTCACCATGATGGCCAAG	60.034	128
TRINITY_Contig10758	1	p4	(TTGT)3	12	547	558	AGCAGGGAATGGCACGTTAA	59.962	GCTTGTCCATGGAAGGGGAA	59.96	154
TRINITY_Contig10760	1	p3	(CCT)4	12	548	559	TCACGTGCGAGTCACATTCA	59.969	TCCAGTAGGTGCTCCGATCA	60.034	133
TRINITY_Contig10760	2	c	(CCG)6ct(CGC)5	35	976	1010	TCACGTGCGAGTCACATTCA	59.969	TCCAGTAGGTGCTCCGATCA	60.034	133
TRINITY_Contig10762	1	p4	(CTAG)3	12	158	169	GCGCCGGCTACACATATACT	60.039	GCTACACCAACTGCTGAGGT	59.965	242
TRINITY_Contig10763	1	c	(GGC)4aggctccgcg(TCC)4gccgccgccacgtggtggtcc(GCG)4	67	689	755	GATTGGCGAATTCCCTTGCC	59.897	GGAGATCGAGGAGGACGTCT	60.179	249
TRINITY_Contig10763	2	p6	(GGCCGC)5	30	962	991	GATTGGCGAATTCCCTTGCC	59.897	GGAGATCGAGGAGGACGTCT	60.179	249
TRINITY_Contig10764	1	p3	(CGT)4	12	264	275	TCTTCCGCAACTCCATCACC	60.036	ATCAGGCTGTCCGTCTTGTG	60.037	133
TRINITY_Contig10766	1	p5	(CGCCA)3	15	304	318	GCGGACGCATTCTTGTTCTC	59.905	CAGCTCCTTGATGTGGCTCA	60.036	130
TRINITY_Contig10768	1	p3	(GGC)4	12	924	935	AGGACCGGAGATTACGTGGA	60.034	TGTAGCCTGCTTGATGGAGC	60.108	100
TRINITY_Contig10770	1	p3	(CGT)4	12	497	508	CGAGTACCCAACTGCCCAAT	60.035	TTCGCCTCTTCGATCACTCG	59.901	256
TRINITY_Contig10770	2	p4	(CGCC)3	12	835	846	CGAGTACCCAACTGCCCAAT	60.035	TTCGCCTCTTCGATCACTCG	59.901	256
TRINITY_Contig10770	3	c	(GCGACG)3gcggcgcgtcggcgtcagattggttggcggc(GAGGA)5aagccgagcgcgcacgagacctgacctccaggtccaggcagaacgaga(GAGC)4	138	1067	1204	CGAGTACCCAACTGCCCAAT	60.035	TTCGCCTCTTCGATCACTCG	59.901	256
TRINITY_Contig10771	1	p3	(CGC)6	18	324	341	CTCAAGAACAGCGCAACGAC	60.111	CACCTCACCGTACCTCATCG	59.899	108
TRINITY_Contig10771	2	p4	(GACG)3	12	936	947	CTCAAGAACAGCGCAACGAC	60.111	CACCTCACCGTACCTCATCG	59.899	108
TRINITY_Contig10772	1	p6	(CGCCAA)4	24	32	55	CTTTATCACGGCGCTTGAGC	59.973	CGCTTGAACTTGTCGGCAAA	59.971	201
TRINITY_Contig10773	1	c	(CGG)4aggagcccgcttgagcggcggccatggcggagcgggcgagatgagccgcggcaaaaccctaggccggcgaggtgagcggagcggagccgagcgagagc(GGT)4	123	1047	1169	ACGACATGCTGTGCTAACCA	59.965	TGCAGTGAATTTGCACACCG	59.969	200
TRINITY_Contig10774	1	p3	(CGA)4	12	801	812	GCAACATCCAGGCAAAGCAA	59.966	TATGCTTGAGGCCCCTGTTG	60.034	146
TRINITY_Contig10775	1	p4	(CTTG)4	16	794	809	CTCTTGCTTGCTTGCTTGCA	59.968	ACCTCAAAGATGCCGTGGAG	60.036	256
TRINITY_Contig10776	1	p3	(CGC)5	15	220	234	CGCCACCACCAGCCATATAT	59.963	TGTCGTCTTCCTCCTCCTGT	59.887	162
TRINITY_Contig10776	2	p3	(GCG)4	12	464	475	CGCCACCACCAGCCATATAT	59.963	TGTCGTCTTCCTCCTCCTGT	59.887	162
TRINITY_Contig10778	1	c	(CTCC)3gcccccgccccgcccctcccatccctcgcatgtgcgcgctttgca(GCG)4	69	290	358	TGGCAGCAACTACCTGGATG	60.035	CACATGCTCTGCTCCTCACA	60.037	280
TRINITY_Contig10778	2	p3	(TAA)5	15	1148	1162	TGGCAGCAACTACCTGGATG	60.035	CACATGCTCTGCTCCTCACA	60.037	280
TRINITY_Contig10781	1	p4	(CTCG)3	12	589	600	TTCCCTTCGCTGTTGGAGAC	59.965	CGCTTCTCTGGTCCTAGCTG	59.898	178
TRINITY_Contig10786	1	c	(TCA)5taatgctgcttctcctgaagaagctcaagaagtc(TCT)4	61	481	541	GAACGATCTCGAGGGCTACG	60.041	GCACAGACAGCTTCCTCCTT	59.964	215
TRINITY_Contig10786	2	c	(GCG)4aaacgatctggttggacaccgtga(GCC)5	51	766	816	GAACGATCTCGAGGGCTACG	60.041	GCACAGACAGCTTCCTCCTT	59.964	215
TRINITY_Contig10788	1	p3	(CCG)5	15	777	791	CGCGAAACTTTTTGGCAGGA	59.97	CCTCTCCCTTGCTCCTCTCT	60.032	107
TRINITY_Contig10789	1	p4	(CAAG)3	12	562	573	CAATGTGCGTTCTGTGGCAA	59.969	TGCCTTGCTCTCTTTCTCGG	60.037	117
TRINITY_Contig10789	2	c	(ACGC)3ctagctgagcttcgagctgtgctctggagtagagtgagaagctggag(GA)6tgtgagtttgctgatgatgagatggaggagggttgtggagctccacgtccagggggaaggatttaagggcggcggggatgggcgacgggcgag(GA)13	190	684	873	CAATGTGCGTTCTGTGGCAA	59.969	TGCCTTGCTCTCTTTCTCGG	60.037	117
TRINITY_Contig10790	1	p3	(GCG)4	12	2148	2159	TCATCGTATACCTTGCGGGC	59.967	CAGCGGATCATACATGGGCT	59.964	275
TRINITY_Contig10791	1	p3	(CGC)4	12	357	368	GCGAGATCAGGTTCACCCTC	60.179	GCCAGTCAGACACCATCACA	59.965	130
TRINITY_Contig10791	2	p6	(CGGCGC)3	18	519	536	GCGAGATCAGGTTCACCCTC	60.179	GCCAGTCAGACACCATCACA	59.965	130
TRINITY_Contig10792	1	c	(CGA)4ctgcgctgat(GAG)4	34	500	533	AGCTGTTGTCTGATGTGGGG	59.963	GCCACCGCCAATAGTAAGGT	60.107	135
TRINITY_Contig10792	2	p5	(CGCCG)3	15	677	691	AGCTGTTGTCTGATGTGGGG	59.963	GCCACCGCCAATAGTAAGGT	60.107	135
TRINITY_Contig10792	3	p3	(GGC)4	12	898	909	AGCTGTTGTCTGATGTGGGG	59.963	GCCACCGCCAATAGTAAGGT	60.107	135
TRINITY_Contig10793	1	p4	(CTAG)3	12	1142	1153	ATAGTCCTCCAGCTCCAGGG	60.105	GGTGTGGGACTGGGATCTTG	60.035	110
TRINITY_Contig10795	1	p5	(CGCCT)4	20	214	233	TCACCTCCATCCACCAGCTA	59.957	CGCCGCAGCCATCAAAATAA	59.9	128
TRINITY_Contig10795	2	p3	(GCG)4	12	479	490	TCACCTCCATCCACCAGCTA	59.957	CGCCGCAGCCATCAAAATAA	59.9	128
TRINITY_Contig10795	3	c	(CGG)4cgagctgctcctgcggcctgcggaagctctgcgcctc(CTG)4	61	848	908	TCACCTCCATCCACCAGCTA	59.957	CGCCGCAGCCATCAAAATAA	59.9	128
TRINITY_Contig10795	4	p3	(TTG)4	12	1109	1120	TCACCTCCATCCACCAGCTA	59.957	CGCCGCAGCCATCAAAATAA	59.9	128
TRINITY_Contig10797	1	p3	(AAG)4	12	189	200	ATCGGCTACAGGATGGAGGT	60.105	ACAGGAACTTCTTGGCCTCG	59.965	109
TRINITY_Contig10797	2	p3	(ACG)4	12	573	584	ATCGGCTACAGGATGGAGGT	60.105	ACAGGAACTTCTTGGCCTCG	59.965	109
TRINITY_Contig10798	1	p4	(GGAT)4	16	227	242	CACCTCACCCTCACTGACAC	59.966	AACCGAAGCCCGATTGAGAG	60.108	216
TRINITY_Contig10798	2	p4	(AGCT)3	12	471	482	CACCTCACCCTCACTGACAC	59.966	AACCGAAGCCCGATTGAGAG	60.108	216
TRINITY_Contig10798	3	p3	(GAG)5	15	598	612	CACCTCACCCTCACTGACAC	59.966	AACCGAAGCCCGATTGAGAG	60.108	216
TRINITY_Contig10798	4	p4	(GCCT)3	12	1102	1113	CACCTCACCCTCACTGACAC	59.966	AACCGAAGCCCGATTGAGAG	60.108	216
TRINITY_Contig10799	1	p3	(CTC)4	12	487	498	CGACGCTCATCTCTCTGGTC	59.971	TCCTGAACACCTTGTCGCTC	59.966	144
TRINITY_Contig10801	1	p3	(GCA)6	18	150	167	TGCACAGGAATCGAGGTGTC	60.037	CACCCTCCTCGCAACTTCTT	59.965	148
TRINITY_Contig10802	1	p3	(GCA)4	12	1007	1018	CCGCGCCTGCTATTTAGAGA	59.968	CCGACGCCATCTCTCTTCTC	59.97	151
TRINITY_Contig10804	1	p3	(CGG)7	21	1122	1142	AAACAGGGTAGACGCCTTGG	59.964	TCTGCAAGTACCTCAGCAGC	60.037	215
TRINITY_Contig10806	1	p5	(TCTGA)3	15	1123	1137	CGAAGTTGATGACGAACGGC	59.907	AGCAAGTACCCGAGGCTCTA	60.033	184
TRINITY_Contig10808	1	p4	(GATC)3	12	388	399	ACTACAAGCAGAGCGTGACC	60.038	CCCATGCTGTGATCGATCGA	59.967	122
TRINITY_Contig10808	2	p3	(GGC)5	15	612	626	ACTACAAGCAGAGCGTGACC	60.038	CCCATGCTGTGATCGATCGA	59.967	122
TRINITY_Contig10808	3	c	(CCA)4ccggcgcggcggttct(GCC)5	43	994	1036	ACTACAAGCAGAGCGTGACC	60.038	CCCATGCTGTGATCGATCGA	59.967	122
TRINITY_Contig10810	1	p3	(CGC)5	15	100	114	CTCCCTCTCAGGTCTCGTCA	60.035	GTTTAGTCGCCGCGCTTAAG	59.975	198
TRINITY_Contig10810	2	c	(CCAA)4cc(CA)7	32	304	335	CTCCCTCTCAGGTCTCGTCA	60.035	GTTTAGTCGCCGCGCTTAAG	59.975	198

TRINITY_Contig10810	3	c	(CCG)4gttttcttcttcctcgggacgacggcgccgccgccggtgctgga(GCC)6	74	553	626	CTCCCTCTCAGGTCTCGTCA	60.035	GTTTAGTCGCCGCGCTTAAG	59.975	198
TRINITY_Contig10813	1	p4	(TTAA)8	32	46	77	CAGGACCTGTCTTGGGTGAC	59.965	CTTCACCTTTTCCCCTCCCC	59.96	164
TRINITY_Contig10813	2	p3	(GGC)4	12	398	409	CAGGACCTGTCTTGGGTGAC	59.965	CTTCACCTTTTCCCCTCCCC	59.96	164
TRINITY_Contig10813	3	p5	(GAGGG)3	15	1095	1109	CAGGACCTGTCTTGGGTGAC	59.965	CTTCACCTTTTCCCCTCCCC	59.96	164
TRINITY_Contig10815	1	p3	(GGA)5	15	586	600	CGTGTTAGCAATCATGGCGG	59.972	GAGAAAGGATCGCGTCTCGT	59.901	275
TRINITY_Contig10819	1	p3	(GAG)5	15	1023	1037	ATCTCACTCGCCAACACAGG	60.037	TTCATGGGGTGCTACAGCTG	60.035	236
TRINITY_Contig10822	1	p3	(TCG)4	12	660	671	TCAGAGTCCGAGGCAGAAGA	59.962	CCCATAGATACAACCGGGCC	59.964	171
TRINITY_Contig10824	1	p3	(ACA)4	12	1113	1124	CCTTTGCACCTCGAGGGATT	60.035	ATCTCTCATGCCACCCTCCT	60.03	115
TRINITY_Contig10827	1	p3	(AGG)4	12	610	621	TCATCAGTGCGCTACGGTTT	60.038	TTTAGCCCACGTCACTCCAC	59.966	114
TRINITY_Contig10828	1	p3	(CCA)4	12	990	1001	TTCCGCTGCTTCTTTCGTCT	59.966	AGGTCTGCGCTTACGACAAA	59.967	209
TRINITY_Contig10830	1	p3	(CGC)5	15	91	105	AAGGCCGGAAAGCAACCATA	59.961	TCACCCATTTGCCTCGAGAC	60.036	207
TRINITY_Contig10832	1	c	(AGA)4(AGG)4(AGA)4(AGG)4(AGA)4	60	595	654	GGAGGTCAAGGACAAGACGG	60.037	GCATGGCGAAATCCATCGTC	60.041	280
TRINITY_Contig10832	2	c	(CT)20(AT)20	80	971	1050	GGAGGTCAAGGACAAGACGG	60.037	GCATGGCGAAATCCATCGTC	60.041	280
TRINITY_Contig10833	1	p3	(TTC)5	15	132	146	GGGAGGAGAGGAGAGGGATG	60.179	TCCCCTCCCCATTCTCTCTC	59.734	241
TRINITY_Contig10833	2	p3	(CGC)4	12	328	339	GGGAGGAGAGGAGAGGGATG	60.179	TCCCCTCCCCATTCTCTCTC	59.734	241
TRINITY_Contig10833	3	c*	(GGGGA)3(G<GGGA>)(GGGA)2	28	897	924	GGGAGGAGAGGAGAGGGATG	60.179	TCCCCTCCCCATTCTCTCTC	59.734	241
TRINITY_Contig10834	1	p4	(CAAG)3	12	96	107	CCTGACGAGACCAACGAGAC	60.11	AGGAGCTTAGCGAGGAGGAA	60.033	263
TRINITY_Contig10838	1	p3	(CGC)4	12	1467	1478	GTCTTCTGCCAGCCCATCTT	60.035	AAGAAAGGGAGGAGCATGGC	60.034	179
TRINITY_Contig10839	1	p3	(TGC)4	12	1069	1080	CGTGCATCATGAGCGAGTTG	59.974	TCTTCAACCGCTGGACCTTC	59.965	186
TRINITY_Contig10840	1	c	(GCA)4ggctccaccggcaccgtactaccagcagcagccgcagtactaccagccggggccgcccccgcagccgtggggc(CAG)4	97	143	239	TTGGATCGGGGATCTCCAGT	60.031	CCTTCAGACTGCCCACTCTG	60.037	126
TRINITY_Contig10841	1	c	(ACC)4atag(CCA)4aa(ACC)5	45	712	756	AGCGCAAACATGCAACAGAG	60.04	GGCAATCTGTAGTGGCTGGT	60.035	118
TRINITY_Contig10843	1	p6	(CGGCAA)4	24	889	912	GCTTCCGTTGAATGGAGGA	60.035	GAAAGCGAATCGTCCCATGC	59.972	119
TRINITY_Contig10844	1	p3	(GGA)5	15	405	419	TACACGTCATGTCATGCCCC	60.108	ATCTGAGGGGAGGAAGGACC	60.031	232
TRINITY_Contig10846	1	p4	(CAAG)3	12	332	343	TGTCCGTGCATTCAGGTCTC	60.037	CACGACCACGACCCTAACAA	59.968	141
TRINITY_Contig10847	1	p4	(ATGA)3	12	113	124	TCTTCATTATCGCCTCGGCC	59.966	CCCAGGACAAGGCAAGCTAA	59.962	121
TRINITY_Contig10847	2	p3	(CGG)4	12	1017	1028	TCTTCATTATCGCCTCGGCC	59.966	CCCAGGACAAGGCAAGCTAA	59.962	121
TRINITY_Contig10849	1	p3	(GGA)4	12	897	908	GGAGCGTTGGAAGAGTGTGA	59.966	TATCTCGGACGGGTCTCCTC	59.892	145
TRINITY_Contig10849	2	p3	(CTC)4	12	1151	1162	GGAGCGTTGGAAGAGTGTGA	59.966	TATCTCGGACGGGTCTCCTC	59.892	145
TRINITY_Contig10850	1	p3	(CGC)4	12	472	483	GCAGGCATGATCTCCTCCTC	59.965	ATGCCAGTGCTAGTGCCAAT	60.034	127
TRINITY_Contig10850	2	c	(CGC)5ggcgcccgctgcgcgccttcatcggcgacggcgacgacgtgagcgacggcggcgaggacggcgcggagtacatcggcacagtggcaggcatgat(CTC)4	121	678	798	GCAGGCATGATCTCCTCCTC	59.965	ATGCCAGTGCTAGTGCCAAT	60.034	127
TRINITY_Contig10851	1	p3	(GGA)6	18	811	828	GTCACGTGGGTAGGGTTCTG	60.038	TACTCGGGCCTTCTTCTCCA	59.959	136
TRINITY_Contig10854	1	p4	(CCCG)3	12	774	785	CATTGCGTGCGTACCTGATG	59.973	CCTTGCAGAGGTGGACAGAG	60.037	210
TRINITY_Contig10854	2	p3	(CGC)4	12	2281	2292	CATTGCGTGCGTACCTGATG	59.973	CCTTGCAGAGGTGGACAGAG	60.037	210
TRINITY_Contig10855	1	p3	(CAT)4	12	681	692	TGTGCTTGAAGTTTCTGCGC	59.971	GTCTCGTGCCAAGGGAAAGA	59.965	193
TRINITY_Contig10855	2	p3	(GCC)4	12	938	949	TGTGCTTGAAGTTTCTGCGC	59.971	GTCTCGTGCCAAGGGAAAGA	59.965	193
TRINITY_Contig10856	1	p4	(CAGA)3	12	14	25	ACTGGAGCTGCTCACAAGTC	59.965	CACGCCTAGTCACTTGTGGT	59.966	117
TRINITY_Contig10856	2	c	(CGCGCC)3cacgcgcgg(CACC)3	39	149	187	ACTGGAGCTGCTCACAAGTC	59.965	CACGCCTAGTCACTTGTGGT	59.966	117
TRINITY_Contig10857	1	p3	(GAC)5	15	179	193	CTCACCTCTTCCACCAGCAG	60.037	TCCATGCTCTAGGAGGTGCT	60.032	159
TRINITY_Contig10857	2	p3	(GAG)4	12	632	643	CTCACCTCTTCCACCAGCAG	60.037	TCCATGCTCTAGGAGGTGCT	60.032	159
TRINITY_Contig10860	1	p6	(TCCGAC)3	18	246	263	ACAAGAACTGGTTTGCCGGA	60.107	TATCACCCAATGCCCACACC	60.034	130
TRINITY_Contig10861	1	p3	(TCG)4	12	236	247	CAGGTGCCCTTCCTGTGAAT	59.961	CTGATCGCGGTCCTCGATAC	60.11	104
TRINITY_Contig10861	2	p3	(GCG)4	12	491	502	CAGGTGCCCTTCCTGTGAAT	59.961	CTGATCGCGGTCCTCGATAC	60.11	104
TRINITY_Contig10861	3	p4	(TTGC)3	12	1111	1122	CAGGTGCCCTTCCTGTGAAT	59.961	CTGATCGCGGTCCTCGATAC	60.11	104
TRINITY_Contig10864	1	c	(TTTTC)3tccccaaatctgatgctcctccttgccggcgggagcaggctccg(GGC)4	71	7	77	GATGAGGACGGAGATGGTGC	60.25	CTCAGAAGCTCGTTCCCTCC	59.825	279
TRINITY_Contig10865	1	p3	(CCA)4	12	612	623	ACCCAGACCCATAGCCTGAT	60.03	TGCCATCCTCGTCCTCCTTA	60.033	149
TRINITY_Contig10865	2	p6	(CACCGC)4	24	826	849	ACCCAGACCCATAGCCTGAT	60.03	TGCCATCCTCGTCCTCCTTA	60.033	149
TRINITY_Contig10866	1	p3	(CGG)4	12	959	970	TCGGATCCATCCCCCTTCTT	60.03	ATCCGAAACCCCAGGTTGAC	59.962	190
TRINITY_Contig10868	1	p3	(GCG)4	12	578	589	CATTGAGTACACGCCCCAGT	60.036	CACCTCTCCATCGTTGGCTT	60.036	237
TRINITY_Contig10868	2	p3	(CCG)5	15	1216	1230	CATTGAGTACACGCCCCAGT	60.036	CACCTCTCCATCGTTGGCTT	60.036	237
TRINITY_Contig10869	1	c	(CAG)5(CCG)5	30	112	141	CCTTCCTGGTGCAGTCCAAT	59.961	GCTGCCCCCTTGTTATTCCT	60.033	205
TRINITY_Contig10869	2	p3	(CTC)5	15	1257	1271	CCTTCCTGGTGCAGTCCAAT	59.961	GCTGCCCCCTTGTTATTCCT	60.033	205
TRINITY_Contig10870	1	p2	(CT)6	12	37	48	GCAAAAGCAGAAAGGGAGGC	60.038	CAGTAGCTCTCTCTGCTCGC	59.971	118
TRINITY_Contig10870	2	p3	(ACC)4	12	1450	1461	GCAAAAGCAGAAAGGGAGGC	60.038	CAGTAGCTCTCTCTGCTCGC	59.971	118
TRINITY_Contig10870	3	p3	(GCA)5	15	1661	1675	GCAAAAGCAGAAAGGGAGGC	60.038	CAGTAGCTCTCTCTGCTCGC	59.971	118
TRINITY_Contig10872	1	p4	(TGGC)3	12	444	455	AAAAACTGCAGCACTCGCAG	59.97	TTCTCCGCGTTTAACCGTGA	59.968	181
TRINITY_Contig10872	2	p3	(GAC)4	12	874	885	AAAAACTGCAGCACTCGCAG	59.97	TTCTCCGCGTTTAACCGTGA	59.968	181
TRINITY_Contig10879	1	p3	(CGG)4	12	374	385	TTTACCCATACGGACCCCAC	59.089	GGAGCAGAGGAGGAAGGAGA	60.033	108
TRINITY_Contig10879	2	p4	(AGGA)3	12	1004	1015	TTTACCCATACGGACCCCAC	59.089	GGAGCAGAGGAGGAAGGAGA	60.033	108
TRINITY_Contig10881	1	p5	(AGTGA)3	15	164	178	AGCAGTGTGTACTCAACCCG	59.966	CAACGCTGACACCAACTTGG	59.971	223
TRINITY_Contig10882	1	p3	(GCC)5	15	148	162	AGGAGCAGCTGATGACCCTA	60.032	AGCTTCTCTCTCTCCGAGCA	60.035	142
TRINITY_Contig10882	2	p3	(CTG)6	18	268	285	AGGAGCAGCTGATGACCCTA	60.032	AGCTTCTCTCTCTCCGAGCA	60.035	142
TRINITY_Contig10882	3	p5	(AGAGA)3	15	1164	1178	AGGAGCAGCTGATGACCCTA	60.032	AGCTTCTCTCTCTCCGAGCA	60.035	142
TRINITY_Contig10883	1	p3	(CGC)4	12	687	698	CCAATGACGAAATCGCGGTC	59.974	ATGGCTTATCCTGTCCCCCT	60.029	197
TRINITY_Contig10886	1	p4	(GCCG)4	16	755	770	CTGAGGTAGTGGATCGCGTC	59.97	AATTTGGAGTCCCTGGCGAG	60.035	109
TRINITY_Contig10887	1	p3	(GCG)5	15	134	148	GGAAGATCGGCGAGGAGAAG	59.969	CCCCCATAATCGTGAGGTCG	59.966	258
TRINITY_Contig10891	1	p3	(GCG)5	15	1591	1605	TCCTCTTCTTCTCCGGAGCA	59.96	TCGCATCCTCGTCTCGATTG	59.971	218
TRINITY_Contig10893	1	c	(CT)9a(TC)6	31	26	56	ACTCCGCTCCATCCTCTCTT	60.033	CTGCTCGACCATGGACTTGT	60.037	115
TRINITY_Contig10893	2	p6	(CTCCCG)3	18	199	216	ACTCCGCTCCATCCTCTCTT	60.033	CTGCTCGACCATGGACTTGT	60.037	115
TRINITY_Contig10894	1	p6	(GGCGCC)3	18	296	313	TCGTACAGCTATTCGTCGGC	59.971	CCATTGTCTGCTCCATGGGT	60.034	263
TRINITY_Contig10895	1	p3	(ATG)4	12	355	366	ATAGAGCGCTTCCCATCTGC	59.965	AGAGTGAAGCCAAGGAACGG	59.965	181
TRINITY_Contig10895	2	p3	(CTC)5	15	2539	2553	ATAGAGCGCTTCCCATCTGC	59.965	AGAGTGAAGCCAAGGAACGG	59.965	181
TRINITY_Contig10898	1	p3	(GGC)4	12	1081	1092	TCATCTGCAGCCTCCTCTCT	60.032	CCTTGTGCACGAAATTGCCA	59.968	101
TRINITY_Contig10901	1	p4	(GATC)3	12	41	52	ATCCACCGCCATGCCTTTAA	60.034	ATTCGCGGATGAGGATCACC	59.966	145
TRINITY_Contig10901	2	p4	(CCAG)3	12	235	246	ATCCACCGCCATGCCTTTAA	60.034	ATTCGCGGATGAGGATCACC	59.966	145
TRINITY_Contig10902	1	p5	(CCCCG)3	15	451	465	ACGCACAAGAAATCCCCCAT	59.96	CAAGCCTGACTTCCTGCTGA	59.964	203
TRINITY_Contig10903	1	p3	(CGC)4	12	444	455	GCGTTACAAAGGCAGCACAA	59.97	GAATTCTGTGCTGGTTGCGG	60.11	103
TRINITY_Contig10903	2	p3	(CCT)4	12	885	896	GCGTTACAAAGGCAGCACAA	59.97	GAATTCTGTGCTGGTTGCGG	60.11	103
TRINITY_Contig10904	1	p3	(GAG)4	12	605	616	GTACGGCCGAAGAAAATCGC	59.974	CATCGGTCGAGAAGGACGAG	59.972	270
TRINITY_Contig10907	1	p3	(GGC)5	15	309	323	ACCTCAGTGTGCTAGTGTGC	59.966	CATCATCAGGAGGACGCGAA	59.897	179
TRINITY_Contig10907	2	p4	(CCGG)3	12	1081	1092	ACCTCAGTGTGCTAGTGTGC	59.966	CATCATCAGGAGGACGCGAA	59.897	179
TRINITY_Contig10909	1	p3	(CCG)4	12	106	117	GAAGGTCTCGCTCTCCATCG	59.97	CGAAGGCGGTGAACAGGTAT	60.108	146
TRINITY_Contig10911	1	p4	(AAGG)3	12	1795	1806	CCTTCCAGAAAGAGCCTGCA	59.963	CCTTCCTTCCTTGTCGCCTT	59.963	163
TRINITY_Contig10912	1	c*	(GCCGC)2(GCCGCCGCCGCGCGCGCCTCAAAGCCTTCAGGGACGACGCCGCTGTTCCCGGCGCTCACGACCCAGACGAACGGCACGGCGCTGCGCTCCAGGGTCTCGGCCAGTGCTGCCGCCACTGCTGGTGTCAGCACTGCCTGGCTCCCGAAGCACACGTACACGACAGAGCCTTCCGGGAAGGGGTCGAGCCACGCGCTGAGATTGGCGGCCCCGACGGCCGCCTCCCCGCCGCGCGGGCCGGCGGGGTCCGTTTCCGGCGCCACAGGGCCGACCGCGAAGATGCGCTTGAACCCCATGTCTTCTAGAGGCGCGTCCAGGTACCTCCCCTCGAGCGCCCGGAGCGTGTTCGACACGAACACCCAACTCTCCTGCAGGTTCCAGAGGAAGTTCTGCCTCACCGACTCGCGGACCTTCTCTTCGGATCCTCCTTCCAAG	21	574	594	CCTGCAGGTTCCAGAGGAAG	60.036	GGGGATCGTGTTCACACCTT	59.964	242
TRINITY_Contig10916	1	c	(GCG)4accgcggctacggcggagga(GGC)4	44	382	425	GGGGAAAAATGGTGTGGTGC	59.965	TTGGTGACCCCTTGTTGCTT	60.033	102
TRINITY_Contig10916	2	c	(CGG)5ctacggtggcggcgggggcggaggcggccgtggctgcttcaagtgtggcgaggagggccacatggccagggactgctcccagaatggt(GGC)5tacggcggaggtggcggcggctacgga(GGC)4	157	569	725	GGGGAAAAATGGTGTGGTGC	59.965	TTGGTGACCCCTTGTTGCTT	60.033	102

TRINITY_Contig10916	3	p4	(ATCT)3	12	867	878	GGGGAAAAATGGTGTGGTGC	59.965	TTGGTGACCCCTTGTTGCTT	60.033	102
TRINITY_Contig10916	4	p4	(ATCT)3	12	1034	1045	GGGGAAAAATGGTGTGGTGC	59.965	TTGGTGACCCCTTGTTGCTT	60.033	102
TRINITY_Contig10919	1	p3	(GGC)4	12	1074	1085	GCGATGTGTCCGAGCAAATC	59.973	GGTTAGCTTCTGGGGCACAT	60.034	171
TRINITY_Contig10922	1	p3	(CTC)4	12	89	100	GACAACACCACAAGCGTGAC	59.972	ACATGATGTCAGCCCAGGTG	60.035	127
TRINITY_Contig10924	1	p3	(TGC)6	18	110	127	GGATCATCAGCTCCTCAGGC	59.965	GACTTGGACCCAGGACACTG	59.965	126
TRINITY_Contig10924	2	p3	(CAG)4	12	315	326	GGATCATCAGCTCCTCAGGC	59.965	GACTTGGACCCAGGACACTG	59.965	126
TRINITY_Contig10926	1	p6	(GCCGCA)4	24	664	687	GCTGAGACTGCTATCCCGTC	59.969	TGGTCTTAACTGCGTGGACC	59.966	280
TRINITY_Contig10926	2	p6	(GCGACG)3	18	908	925	GCTGAGACTGCTATCCCGTC	59.969	TGGTCTTAACTGCGTGGACC	59.966	280
TRINITY_Contig10927	1	p3	(GAC)5	15	162	176	TCAAACTCCTTGAGGTCCGC	59.965	AGGTCCACGTCGTGTACAAC	59.969	154
TRINITY_Contig10932	1	p3	(GCG)4	12	740	751	AGCAAGGACAGACAGACGTG	59.967	CGTCCGTTTTGAACTCGTCG	59.842	147
TRINITY_Contig10932	2	p4	(GTCC)4	16	928	943	AGCAAGGACAGACAGACGTG	59.967	CGTCCGTTTTGAACTCGTCG	59.842	147
TRINITY_Contig10934	1	p4	(AAAG)7	28	97	124	AGCCCCGAATCCCTCCTAAT	60.104	AGAGCTCGCTCCATGTTGTC	60.108	187
TRINITY_Contig10937	1	p3	(GAC)4	12	350	361	TAGTAAGCACAAGCCGCGAT	59.825	GGGAGAAAGCAAACTCGGGA	59.964	104
TRINITY_Contig10938	1	c	(CGC)5gcgcgccctgctgctggtgctacccggcgccgcggaggtcaccatctccaggcgcggggtcgcctcggccgcctgaaccatgac(CAG)4	111	815	925	CCGGATGTTGTTGGTCTCCA	59.964	TGTTCGTTCCAAACACACGC	59.903	280
TRINITY_Contig10940	1	p3	(CCG)4	12	544	555	TCCTCCTCCTGCCTCATCAA	59.957	TCTACTTCCTGGGAGTCCGG	60.033	104
TRINITY_Contig10942	1	c*	(GCCT)2(GC<CT>)(CT)18cctgtcacctcctccgtcctccccccttcctcttgttgttcttcttcctccttggccttggtcctttagggtgcatgccatgcata(GATG)3	147	17	163	GCCTGCCTCTCTCTCTCTCT	60.107	GTGGGGAATGTCTCCTTGGG	60.034	206
TRINITY_Contig10942	2	p4	(ATCC)3	12	317	328	GCCTGCCTCTCTCTCTCTCT	60.107	GTGGGGAATGTCTCCTTGGG	60.034	206
TRINITY_Contig10942	3	c	(GGA)4a(GAG)5gaagaagaagacgaggaggaagaggaggaagat(GAG)4	73	564	636	GCCTGCCTCTCTCTCTCTCT	60.107	GTGGGGAATGTCTCCTTGGG	60.034	206
TRINITY_Contig10942	4	p3	(CAC)4	12	1291	1302	GCCTGCCTCTCTCTCTCTCT	60.107	GTGGGGAATGTCTCCTTGGG	60.034	206
TRINITY_Contig10942	5	p3	(GCT)4	12	1503	1514	GCCTGCCTCTCTCTCTCTCT	60.107	GTGGGGAATGTCTCCTTGGG	60.034	206
TRINITY_Contig10942	6	p3	(CGC)4	12	1937	1948	GCCTGCCTCTCTCTCTCTCT	60.107	GTGGGGAATGTCTCCTTGGG	60.034	206
TRINITY_Contig10943	1	p4	(CTGG)3	12	97	108	TCCTCGATCTTCCTGGCAGA	60.033	GAGAGCCATGAGTCGAGAGC	59.969	151
TRINITY_Contig10943	2	p3	(CGA)4	12	407	418	TCCTCGATCTTCCTGGCAGA	60.033	GAGAGCCATGAGTCGAGAGC	59.969	151
TRINITY_Contig10943	3	p3	(AGC)4	12	790	801	TCCTCGATCTTCCTGGCAGA	60.033	GAGAGCCATGAGTCGAGAGC	59.969	151
TRINITY_Contig10943	4	c	(GCG)6gctggtggtggggggacgaacg(TGC)4	52	1506	1557	TCCTCGATCTTCCTGGCAGA	60.033	GAGAGCCATGAGTCGAGAGC	59.969	151
TRINITY_Contig10943	5	p2	(TA)8	16	1823	1838	TCCTCGATCTTCCTGGCAGA	60.033	GAGAGCCATGAGTCGAGAGC	59.969	151
TRINITY_Contig10944	1	p4	(CCCG)3	12	521	532	CTAGGCCGTCCAAACCAACT	59.964	GCCGAATAAGAAGGCGCTTG	59.972	186
TRINITY_Contig10944	2	p3	(CCG)4	12	802	813	CTAGGCCGTCCAAACCAACT	59.964	GCCGAATAAGAAGGCGCTTG	59.972	186
TRINITY_Contig10946	1	p6	(GCCACG)3	18	425	442	GGGGCTACTCTGCGAAAAGT	60.036	CCTGATTTGTGTCCGGGGAA	59.962	125
TRINITY_Contig10946	2	p6	(ACGAGG)3	18	683	700	GGGGCTACTCTGCGAAAAGT	60.036	CCTGATTTGTGTCCGGGGAA	59.962	125
TRINITY_Contig10947	1	p3	(AAT)5	15	102	116	AGTTGCGACGTCCACTACAG	60.039	GAGGCTGCTGTTCTTCGTCT	60.038	162
TRINITY_Contig10948	1	p3	(GCA)4	12	71	82	CCACAGCGGAATCTCACTT	60.036	CAGCCCCCTCCTTATTGCAA	60.033	109
TRINITY_Contig10948	2	c	(GAT)4(GAG)10	42	718	759	CCACAGCGGAATCTCACTT	60.036	CAGCCCCCTCCTTATTGCAA	60.033	109
TRINITY_Contig10949	1	p3	(AGC)4	12	837	848	GTATGAGGGTCGCCTCCATG	59.966	GTTTGTCAGCAGCTTCACCG	60.041	161
TRINITY_Contig10951	1	p3	(CTC)4	12	494	505	GAGGTTGGCGAAGTGGATCA	60.036	CGAACCTGAAACCCCTCTCC	60.036	142
TRINITY_Contig10951	2	p3	(GGC)4	12	646	657	GAGGTTGGCGAAGTGGATCA	60.036	CGAACCTGAAACCCCTCTCC	60.036	142
TRINITY_Contig10952	1	c	(GCC)4gcgcccgtaccgcttccccgcgctcccggaggcgaaggaggagc(AGG)4	68	426	493	GACAGAGGCTCTCATCGCTC	59.969	CACGAAACACATGGCACGTT	59.971	194
TRINITY_Contig10952	2	c	(CG)6ggtgcgtatccgccgcctcttcct(TGG)4	48	2892	2939	GACAGAGGCTCTCATCGCTC	59.969	CACGAAACACATGGCACGTT	59.971	194
TRINITY_Contig10953	1	p3	(TTC)4	12	294	305	GCTCTTCTTCTTGGAGCCGT	60.037	CCGCCGAGGAAGACATAGAC	59.969	173
TRINITY_Contig10953	2	p3	(CGG)5	15	1046	1060	GCTCTTCTTCTTGGAGCCGT	60.037	CCGCCGAGGAAGACATAGAC	59.969	173
TRINITY_Contig10954	1	p3	(CGG)5	15	436	450	CGTGGACACCTTAATGCCCT	60.035	GCATCCTCTGATCTGCTCCC	59.965	213
TRINITY_Contig10954	2	p3	(CGC)4	12	787	798	CGTGGACACCTTAATGCCCT	60.035	GCATCCTCTGATCTGCTCCC	59.965	213
TRINITY_Contig10954	3	p3	(CGG)4	12	1012	1023	CGTGGACACCTTAATGCCCT	60.035	GCATCCTCTGATCTGCTCCC	59.965	213
TRINITY_Contig10955	1	p6	(GCGGAG)3	18	171	188	GCCGACGTCTCCATCAAGAA	60.109	TTGATGGCCGTGTAGGTGTC	60.037	245
TRINITY_Contig10956	1	p3	(GCC)4	12	353	364	AAGACGGCCATCTGGTTCTG	60.036	GATGATGTTGGTGATGGCGC	59.97	141
TRINITY_Contig10956	2	c	(TG)19g(GT)13c(TG)17tc(TG)11	124	974	1097	AAGACGGCCATCTGGTTCTG	60.036	GATGATGTTGGTGATGGCGC	59.97	141
TRINITY_Contig10958	1	p6	(GCGCCG)3	18	125	142	GGAGGTGTCGCTATCCTTCG	59.969	CGCTCACCTGACAGGATGTT	60.037	225
TRINITY_Contig10959	1	c	(GCA)4ggaggaggcgctgctagcctcctcctactagtgctctcgaccctcctggcaacggcagtttcctccgtctccatcatcc(CCA)4cttgcagcagcagttgctttctt(CCTC)3ggaaattcactcatcactcgcccatctccatcacggaggctcctccccaccaccataaccacagcagagaagatcgaaattctctccaccctc(TGCCG)4ccgatgccgacgcctccacttctctgctctcacaaccaaaattaacaactcaaatcacaatggggatgggccggatggccgcctccttcctccc(CCT)4	357	155	511	ACTCGAGACTCTGCACCTCT	59.962	GAGATGTCGAGGCAGACGAG	59.971	165
TRINITY_Contig10961	1	p3	(GCT)4	12	613	624	TCATCAAGACCGGCGACAAA	59.966	GCCACACAGAAGGGGCTAAT	60.034	269
TRINITY_Contig10964	1	p3	(CCT)4	12	1745	1756	CACTGTGTTGGATCCTCCCC	60.035	GTAGCCCTGGCACTCCAAAT	60.034	118
TRINITY_Contig10966	1	p3	(GCC)5	15	362	376	ATGGGCGTGGGTCTCTAGAT	60.105	CAACACCAAGCATCACCCAC	59.685	108
TRINITY_Contig10967	1	p6	(CGTCCT)3	18	171	188	CCCGCTTTTGGTCCCCTTAT	60.034	GTTCTGCGTGTCCGGTCTTA	60.039	144
TRINITY_Contig10969	1	p4	(TGAT)3	12	1060	1071	TCTTGACGAGATCGCACGAG	59.903	TCCAAGTCGGACAAGATCGC	60.109	256
TRINITY_Contig10970	1	p4	(CAGC)3	12	500	511	CGCCGAGTAGGAGCATTGAT	59.967	GGTACAACTGCTGCCTCCAT	60.035	198
TRINITY_Contig10970	2	p6	(GCGCGG)3	18	613	630	CGCCGAGTAGGAGCATTGAT	59.967	GGTACAACTGCTGCCTCCAT	60.035	198
TRINITY_Contig10972	1	p3	(TCG)4	12	375	386	TGGTGGAATGGTGATGGTGG	59.961	TCCGTTCCTGCTGTCCTTTC	59.965	101
TRINITY_Contig10972	2	p3	(CCG)6	18	684	701	TGGTGGAATGGTGATGGTGG	59.961	TCCGTTCCTGCTGTCCTTTC	59.965	101
TRINITY_Contig10974	1	p3	(ACG)4	12	897	908	GAAGGCGGAGAAGACACGAA	60.039	CGGAAAGACTGAGCGTGACT	60.039	106
TRINITY_Contig10975	1	p3	(GGA)4	12	512	523	AGTAAGTCTCCCGCCTCCAT	60.032	AAGGCTTGCTTCTTCCTCCC	59.961	138
TRINITY_Contig10978	1	p3	(CGG)5	15	160	174	GAAGGTCGAGGTGAAGGTGG	60.037	TGAACACTCGACTGGGTTCG	59.968	212
TRINITY_Contig10979	1	p3	(CTC)4	12	749	760	TACTGCACCTTGGGCTTCTG	59.963	TTCCTCCTCCCTTCTCCCTG	59.957	120
TRINITY_Contig10982	1	p3	(CCG)5	15	105	119	GCCATCAAGCAGGTGGTAGT	60.035	AGGGCTGTTGCATGACTGAA	59.889	186
TRINITY_Contig10984	1	p3	(GCC)5	15	675	689	CTGAAGGCCATCGTTCCCTT	60.035	ATTCCCCTCCTCCTCCAGTC	60.031	181
TRINITY_Contig10986	1	c	(CGC)5ctccaccgcgccggccgacaaccgcgcaccccgcc(GCCTCG)3	68	209	276	TCATCTCCGTCTCCCTCTCC	59.816	CGGCTCTCGCTTGATCTTCT	59.898	105
TRINITY_Contig10990	1	p3	(GTG)4	12	611	622	CTTGACGACCTCACCGTTCA	59.968	TTGTGCGCATCACCAACAAG	59.969	239
TRINITY_Contig10991	1	c	(AG)7gaaactgctaac(CTGA)3	38	245	282	CGTCACTTCTTCCTGGGCTT	59.965	AAGGGCACAAGGAGGAATCG	60.035	181
TRINITY_Contig10992	1	p3	(CCG)4	12	952	963	ATAGCACTGTCACCACCACG	60.037	CCAGATCTTCGCCTACGGAC	59.969	102
TRINITY_Contig10993	1	p3	(GGC)4	12	507	518	CAGAGGGACCATCATACGGC	59.966	ACGTCATCGATCGAGCTCAC	59.972	157
TRINITY_Contig10996	1	p3	(CGG)4	12	244	255	CGAGTTCAGCGCGATAGGAT	60.04	CTCTGTGCAGTCATCTGGCA	60.037	255
TRINITY_Contig10997	1	p3	(CCT)4	12	293	304	GCAGCCATAACCCCCTTCTT	60.033	TAATACACAGGGGCGGAGGA	60.032	182
TRINITY_Contig10998	1	p3	(CCG)4	12	380	391	AGACGACCCCTGATCATCCA	60.032	ATTGTTGTTCCCGCTTGCAC	59.968	119
TRINITY_Contig10999	1	c	(GCC)8cggaggctcctctcccgccgcg(CCT)7	67	49	115	TGGTGTTCTCTTCGTGCTCC	59.966	GTGGCACTGGTTCTACCCTC	60.036	131
TRINITY_Contig11000	1	p6	(CCGGCG)3	18	205	222	CCACCTTCTCCAACCTCACC	59.963	CATGTCGTCGGTGAGGTTGA	60.039	161
TRINITY_Contig11002	1	c	(AAAC)3gttggatcttttgagagagataaa(AG)8taaccttaccagttagttgaaacgagagagtgtctgtttgtctagatcac(TCT)8	126	7	132	ACGCTACTGGATGCAAGCTT	60.036	GATAGTCTCATCGGCGGCAA	59.968	110
TRINITY_Contig11002	2	p3	(CCA)4	12	765	776	ACGCTACTGGATGCAAGCTT	60.036	GATAGTCTCATCGGCGGCAA	59.968	110
TRINITY_Contig11003	1	p3	(GGA)4	12	611	622	TACAAGCGCGGCTCCTAAAT	59.823	CCAGTCACCTCCTTCCACAC	59.965	203
TRINITY_Contig11004	1	p3	(GCG)4	12	96	107	CCACATCCACCCATGCTCTT	60.034	TAAGTACGTACAGGGCGGGA	60.035	231
TRINITY_Contig11004	2	c	(AAG)5agaagaa(CGC)5	37	726	762	CCACATCCACCCATGCTCTT	60.034	TAAGTACGTACAGGGCGGGA	60.035	231
TRINITY_Contig11005	1	p3	(GAG)4	12	1487	1498	GATGGCTTAGGACGCCTCTC	59.967	ATGGCCATGCTTCATCACCA	60.033	185
TRINITY_Contig11005	2	p2	(GA)12	24	1668	1691	GATGGCTTAGGACGCCTCTC	59.967	ATGGCCATGCTTCATCACCA	60.033	185
TRINITY_Contig11009	1	p5	(TGCGT)3	15	26	40	CATCCAGAGAGCAGCGTAGG	59.968	CAGCGTGGATGAATCAAGCG	59.973	157
TRINITY_Contig11009	2	p2	(AG)9	18	1730	1747	CATCCAGAGAGCAGCGTAGG	59.968	CAGCGTGGATGAATCAAGCG	59.973	157
TRINITY_Contig11009	3	p5	(ACGCA)3	15	1946	1960	CATCCAGAGAGCAGCGTAGG	59.968	CAGCGTGGATGAATCAAGCG	59.973	157
TRINITY_Contig11010	1	p3	(CCT)4	12	203	214	ATGACATCAGTGGAAGCGCA	60.036	TTTTTGTACCGTTGCTGCCG	59.97	239
TRINITY_Contig11011	1	c	(GAT)5ttgggagatggtgacgacaat(GAG)4gaa(GAG)4	63	705	767	CCACTTTGCTCCAAAACCGG	59.968	GCCTGTGTAAGTTCCCCCTC	60.035	171
TRINITY_Contig11012	1	p4	(GCCC)3	12	214	225	AGTCGGTTTTGTGTTTGGCG	59.9	AGCAAGGCAGCTTCAGAGTT	59.89	119
TRINITY_Contig11013	1	p3	(CCG)4	12	380	391	CCACACGTCTAAGCACCCTT	59.965	TTCCTAGTGGACGACGGGAT	60.034	252

TRINITY_Contig11014	1	c*	(GGC)3(GGCAGCGGCAGCGGCAGAGATGGACTGGAGTTCTGTGACGGCGGAAGACCTGATGGACGCGCTGCGGGAGGTGGACTGGTCGACACCGCCGCGCCCCGTCTCCGAGTTCTTCTCCCGCTTCACCGCCCCCCGATCCTACTCCAAGTGGACCAGCCGCCTCAAGTGCAACCTCTACTATTACAGGACAAACTACTTCATCTTGGTAATGTTCATTCTTGGAATGGGCTTTCTTAGGAAGCCACTTGCCATCCTTGCTGCTTTAGCAACTGGTCTCAGCATTGCGTTTCTCAATGACAGGTGACTTTAACTTGCGCTGAAACGCGCATATTATACTTTGACTATGGTTCGTGGAGTGTTCTTAATTCTATACTCTGTTCTCCCCCATGATCTGGAAGTCTGGAAAAATGCATATTAATGTTCAACAAATCATTTTCAACTG	25	149	173	CGTTCCCAGTTCCCATCGAA	60.037	CCGTCACAGAACTCCAGTCC	60.038	148
TRINITY_Contig11015	1	p3	(CCT)5	15	157	171	TCAAGACCGGCACTGTTCTC	59.966	AGTGCCGCACCATTGTGATA	60.036	260
TRINITY_Contig11016	1	c	(AG)6g(GGA)5	28	41	68	CCATCCGTCATGTCCGACAA	60.108	GGAACAGAGGACGAGTGCAA	59.966	230
TRINITY_Contig11016	2	p3	(CGG)4	12	428	439	CCATCCGTCATGTCCGACAA	60.108	GGAACAGAGGACGAGTGCAA	59.966	230
TRINITY_Contig11016	3	p4	(GGGC)3	12	1175	1186	CCATCCGTCATGTCCGACAA	60.108	GGAACAGAGGACGAGTGCAA	59.966	230
TRINITY_Contig11016	4	p4	(GGCC)3	12	1616	1627	CCATCCGTCATGTCCGACAA	60.108	GGAACAGAGGACGAGTGCAA	59.966	230
TRINITY_Contig11016	5	p4	(GCGA)3	12	1915	1926	CCATCCGTCATGTCCGACAA	60.108	GGAACAGAGGACGAGTGCAA	59.966	230
TRINITY_Contig11017	1	p2	(AC)8	16	4	19	CCTGAAGCAGGTCAAGGAGG	60.036	CGTCTTCTTCTTGGCCGAGT	60.038	217
TRINITY_Contig11018	1	p3	(GCC)4	12	154	165	CTTCGCGCAGGATTTGAGTG	59.904	TCAACAAGCATCCCGAGACC	60.036	180
TRINITY_Contig11019	1	p4	(ACCA)4	16	157	172	TCTTCTTCCACGGTGCTTCC	59.965	TGTCTCGTCGTCGCTCAAAA	59.97	151
TRINITY_Contig11019	2	p4	(CTCG)3	12	889	900	TCTTCTTCCACGGTGCTTCC	59.965	TGTCTCGTCGTCGCTCAAAA	59.97	151
TRINITY_Contig11019	3	c*	(TTCT)2(<TTCT>TCTTCTTCTTTTTTTTATTAGAAAAAAGTGTATGATCTTTTGAGCGACGACGAGACAACGGGGATCAAATTATGAATAGCTTTAAAAAGTTTGCATATATAAACAACCAAACGTGTAATAGGTGTGGCTGAACT)(TTC)3	20	1363	1382	TCTTCTTCCACGGTGCTTCC	59.965	TGTCTCGTCGTCGCTCAAAA	59.97	151
TRINITY_Contig11021	1	c	(GCC)5acacgcccacaacacaccgccttccttcctc(CCG)4	58	100	157	CTCCAACATCGTCCCCAACA	59.964	CCGCCTTTGTTGAGCATCAC	60.11	222
TRINITY_Contig11021	2	p3	(CGC)4	12	345	356	CTCCAACATCGTCCCCAACA	59.964	CCGCCTTTGTTGAGCATCAC	60.11	222
TRINITY_Contig11022	1	p3	(AGC)4	12	208	219	ATGTTCTGCGACCGTGTTCT	59.966	GCTTCCTCCTGTCTCCACAC	60.037	134
TRINITY_Contig11022	2	p3	(CGG)5	15	642	656	ATGTTCTGCGACCGTGTTCT	59.966	GCTTCCTCCTGTCTCCACAC	60.037	134
TRINITY_Contig11022	3	p3	(GGT)4	12	769	780	ATGTTCTGCGACCGTGTTCT	59.966	GCTTCCTCCTGTCTCCACAC	60.037	134
TRINITY_Contig11023	1	p2	(AG)10	20	1031	1050	CACGCCCTTCCATTCTTCCT	60.035	TCATCAAGTACACGGACGCC	60.109	248
TRINITY_Contig11024	1	p3	(GGC)5	15	815	829	CATTCTCAGCGTTGGCATCG	59.973	CTTTGACGATCCCCAGCACT	60.036	137
TRINITY_Contig11024	2	p4	(GCCT)3	12	1045	1056	CATTCTCAGCGTTGGCATCG	59.973	CTTTGACGATCCCCAGCACT	60.036	137
TRINITY_Contig11027	1	p3	(GCC)5	15	77	91	TCTCTTCTCCCCTCGACCTG	60.034	GATGAGGTGCGAGATGGAGG	59.967	148
TRINITY_Contig11029	1	c	(CGG)4gcgcgcgagacggaacccccttcgcggt(GGGC)3	51	799	849	AGCACGCCACATCTCATTGA	60.036	CTGCCGGTTGTTGAACTTGG	59.969	236
TRINITY_Contig11029	2	p2	(GA)11	22	1007	1028	AGCACGCCACATCTCATTGA	60.036	CTGCCGGTTGTTGAACTTGG	59.969	236
TRINITY_Contig11030	1	p2	(TC)6	12	1005	1016	GCAGCCATGGCCCATATTTG	59.966	GGCGGTGTTCAGAGTTCAGA	59.966	194
TRINITY_Contig11032	1	p3	(CCA)4	12	44	55	GGTGGTGGTGGCTGTAATCA	59.962	AGTAGAACGCACGGCTTTGA	59.967	146
TRINITY_Contig11032	2	p3	(CCT)4	12	197	208	GGTGGTGGTGGCTGTAATCA	59.962	AGTAGAACGCACGGCTTTGA	59.967	146
TRINITY_Contig11032	3	c	(GGA)4gcccggaggacggggacatgttgctggtgctgggcgcttgctaggtagctcgccgcggcggcaatggaggggaggaggcgtggcgg(GGC)4	110	320	429	GGTGGTGGTGGCTGTAATCA	59.962	AGTAGAACGCACGGCTTTGA	59.967	146
TRINITY_Contig11032	4	p3	(CGG)4	12	661	672	GGTGGTGGTGGCTGTAATCA	59.962	AGTAGAACGCACGGCTTTGA	59.967	146
TRINITY_Contig11033	1	p4	(GCCG)5	20	383	402	GAGCAAGCAAGCCGATCATG	59.971	AGCCGCACGAACTAGTTGAA	59.967	148
TRINITY_Contig11033	2	p4	(TTCA)3	12	2251	2262	GAGCAAGCAAGCCGATCATG	59.971	AGCCGCACGAACTAGTTGAA	59.967	148
TRINITY_Contig11034	1	p6	(CGCCAC)3	18	1082	1099	CGCCACCAACTGGAAGATCT	60.036	TCCTGGTGTAGTGGTGTTGC	59.892	251
TRINITY_Contig11036	1	c	(CTC)4tctttctct(CTCG)4	37	54	90	AACAGCATGAGGAACCCCAG	59.961	ACGGATGGCTTCCACTTCTG	60.036	185
TRINITY_Contig11040	1	p6	(GGAGCC)3	18	628	645	GACGTCAACGAGGAAGTGGT	59.968	AACTGTTCGTGTCCGAGACC	59.968	216
TRINITY_Contig11040	2	p3	(GCG)4	12	1807	1818	GACGTCAACGAGGAAGTGGT	59.968	AACTGTTCGTGTCCGAGACC	59.968	216
TRINITY_Contig11041	1	p3	(GCA)4	12	1737	1748	AACACGAGTCCACGAGTCAC	59.97	GTACTCCTCCACGTCCTCCT	60.034	223
TRINITY_Contig11043	1	p3	(GAC)5	15	929	943	ATGGTGGGAGAGAGGTCCTC	60.032	CTGAGTTCCGACCGACTTCC	60.109	273
TRINITY_Contig11044	1	p3	(CCA)4	12	776	787	CCATCCCTTTGGCCTTAGCA	60.033	TGGATGGTTGGGTCGATGTG	60.036	129
TRINITY_Contig11044	2	p3	(GAG)4	12	1748	1759	CCATCCCTTTGGCCTTAGCA	60.033	TGGATGGTTGGGTCGATGTG	60.036	129
TRINITY_Contig11046	1	p5	(CGCGC)3	15	576	590	AGCCAGCAACTCTAGCACTG	60.037	CATCACCGTCAGGAGGATCG	59.969	248
TRINITY_Contig11048	1	c	(CCGG)3cgagctccgcctcggcgtcctcgatgagcagagcagagcgcgtccaggtcgtccaagccgtccagcatggccagaaactccaccttgctaacgctg(TCC)4acactatctgggccctcgtcgtcggtggtcaccaggttgtcggacggcgggcgccgcctggagatggtacccttctcgatggtgatgaacccggaagac(TCG)5tactcttctggtggcccgcctgcaggggcgggggccggggacacccgcaggcagctcagcagcgccgccgccgtgcagcccccagcacatgcacc(GCG)4	341	198	538	AGAGTCAGAAAGAGCACGGC	60.038	CTCTAGATCAGCTGCGGTCG	60.041	135
TRINITY_Contig11048	2	p4	(ATGG)3	12	1031	1042	AGAGTCAGAAAGAGCACGGC	60.038	CTCTAGATCAGCTGCGGTCG	60.041	135
TRINITY_Contig11049	1	c	(GCT)5ggtcttgccgcgcttgctgctgccgcaattttgttgcgcaagag(GAA)4	71	875	945	CAAAAACATTTCCCGGCCGT	59.967	CCTCGCACTCGTACTCGTAC	59.974	125
TRINITY_Contig11049	2	c*	(TGCCGC)2(T<GCCGC>CGCCGCCGCCGCCGCCGCTGCTGCTGCATAGAGGTGTGCTTGTTGGGTGAGGCATGCCAGATTACCTGCAGTCCCTTTGGCTTTCAGGGCTCGTAGTTCCTGGTGATGTAAATATTTGTTTTGTTGGCACCGTGAGAACAGAGGGAATGTACATATATGTAAGAGTGGTGAAATAGCACTTATATATGAGTACTGAACATATGTCATAGTATGTGACTAGGCTGACCCCTTTTTATTTCTTTTCTTTCTTTTCTTGTTCTTTTTCTTTGTGAAAAAAAGGGACTGGCATGACCTATTGATAACTGTGGTATGCTTAATCTGCCGTTGCACAAGTCAGATTGCCATGATATGTACTACATTACTGAATAATCCATACAAGAAGTAGATTGGCGCACAGGGTAAGCTCACATGGATCCACCATACACGATGC	34	2097	2130	CAAAAACATTTCCCGGCCGT	59.967	CCTCGCACTCGTACTCGTAC	59.974	125
TRINITY_Contig11050	1	c	(CAG)6ccgcgtcggcgccgcctttccccccgcacgggcccgcctccgccaccggatcgagcgtgcgcctcgccggcgggcgtaggct(GCG)4	112	226	337	TCACCGTAGTTGCTGGTGTC	59.967	GTTGACAGCTGCACACAGTG	59.971	251
TRINITY_Contig11052	1	c	(CTCTG)3ctccgccccctcctgctcctgtataaatagctgctgctgcaggg(AGGC)3(AGCA)4	87	103	189	TACTGGGAGGTAGCAGCCAT	60.031	GTCTGTGATTGCGAGACCCA	60.037	153
TRINITY_Contig11053	1	p3	(GCC)4	12	555	566	TAGAAGCCAGGTTGCCACAG	59.963	TTTCTCCTGCAGCTGGACAG	59.964	144
TRINITY_Contig11053	2	p3	(GCG)4	12	876	887	TAGAAGCCAGGTTGCCACAG	59.963	TTTCTCCTGCAGCTGGACAG	59.964	144
TRINITY_Contig11054	1	p3	(CTC)4	12	152	163	GAGAGAGCAATGGGGAGCAG	60.179	GACTTCAGACGGACCTTGGG	60.037	248
TRINITY_Contig11055	1	c	(GTC)5ccctt(CCTC)3	32	131	162	CAACCGCTGTGGAAAACCAG	59.969	GGAACCAAACCGCCCTCTTA	59.963	270
TRINITY_Contig11060	1	p3	(ATG)5	15	615	629	ACAGCTACTACCAGCGCAAG	60.109	TCATCATCATGCCCTCGACG	59.967	122
TRINITY_Contig11061	1	p4	(GGAC)3	12	89	100	CGGGTGGAGTGGCTATCCTA	60.469	CCGGAACAGAAACGATCCGA	60.109	174
TRINITY_Contig11061	2	p2	(GC)9	18	490	507	CGGGTGGAGTGGCTATCCTA	60.469	CCGGAACAGAAACGATCCGA	60.109	174
TRINITY_Contig11061	3	c	(GTG)5gcggcgcggcgctgtccgatccgattcccgcgcccacccccttgc(CG)6	71	776	846	CGGGTGGAGTGGCTATCCTA	60.469	CCGGAACAGAAACGATCCGA	60.109	174
TRINITY_Contig11061	4	p3	(CGC)5	15	973	987	CGGGTGGAGTGGCTATCCTA	60.469	CCGGAACAGAAACGATCCGA	60.109	174

TRINITY_Contig11063	1	p3	(TCC)4	12	1142	1153	AGGAGATGCAATCCCCTCCT	60.029	CATACATCGTCGGCGGGTAA	59.969	157
TRINITY_Contig11064	1	p6	(CGGCGC)3	18	158	175	CGATGAAACGCCTGCAGAAG	59.904	AGGTCAGCAAGCTGCCTTAG	60.036	244
TRINITY_Contig11064	2	p3	(CGA)4	12	455	466	CGATGAAACGCCTGCAGAAG	59.904	AGGTCAGCAAGCTGCCTTAG	60.036	244
TRINITY_Contig11064	3	p4	(CTTT)3	12	2088	2099	CGATGAAACGCCTGCAGAAG	59.904	AGGTCAGCAAGCTGCCTTAG	60.036	244
TRINITY_Contig11067	1	p3	(GCT)4	12	756	767	AGAAGAGGGAAAAGGCCGTG	59.963	TACATGGCGAATCGAGCTCC	59.968	254
TRINITY_Contig11068	1	c	(CCG)4cggcatccggctccgtgtcggggggct gtcaacaggcatcgctccttctcgtccacctcct gctcccctctccgcctccctctcctccg(CGC)4	112	130	241	GCAGGAGGCTTTGAATCCCT	60.034	TGAATCCAGGTGACATGGCC	60.034	260
TRINITY_Contig11070	1	p6	(GGCCGC)4	24	710	733	TTGCAGAGCTCGTCGTTCAT	60.038	CGGTCATCGACGAATTTGGC	59.974	257
TRINITY_Contig11070	2	p3	(CGC)6	18	913	930	TTGCAGAGCTCGTCGTTCAT	60.038	CGGTCATCGACGAATTTGGC	59.974	257
TRINITY_Contig11072	1	p3	(CGG)4	12	978	989	GTGGACGTCGAACTCTTGGT	59.968	CAATCCGTGCTGCAATGGAG	59.9	233
TRINITY_Contig11074	1	c	(CATG)3cacacggaaatcgatcgaaaccta cataaaatatgctcagtccggcataccaaatg gg(GC)7	84	196	279	GTCGCGAGGAAGACGAAGAA	60.11	CCCATTTGGTATGCCGGACT	60.107	121
TRINITY_Contig11074	2	p3	(GCC)5	15	807	821	GTCGCGAGGAAGACGAAGAA	60.11	CCCATTTGGTATGCCGGACT	60.107	121
TRINITY_Contig11075	1	p6	(CCCGAG)3	18	490	507	CACCGAACCCACGAGAAAGA	59.967	GGGCGTCTAACTCTCATCCG	59.969	191
TRINITY_Contig11077	1	p2	(CT)12	24	33	56	GAGAGCGAGAGAGAAGCACG	60.248	CTCCTCTCCATCACTCCGGA	60.106	104
TRINITY_Contig11077	2	p2	(GA)15	30	159	188	GAGAGCGAGAGAGAAGCACG	60.248	CTCCTCTCCATCACTCCGGA	60.106	104
TRINITY_Contig11077	3	p5	(CGGCG)3	15	304	318	GAGAGCGAGAGAGAAGCACG	60.248	CTCCTCTCCATCACTCCGGA	60.106	104
TRINITY_Contig11078	1	p3	(CCT)5	15	365	379	CTCTTGAGGAGGCACTTGGG	60.036	CCGGAGCGACTTCCAATTCT	60.108	217
TRINITY_Contig11078	2	p3	(CCG)4	12	622	633	CTCTTGAGGAGGCACTTGGG	60.036	CCGGAGCGACTTCCAATTCT	60.108	217
TRINITY_Contig11078	3	p4	(GAGG)3	12	949	960	CTCTTGAGGAGGCACTTGGG	60.036	CCGGAGCGACTTCCAATTCT	60.108	217
TRINITY_Contig11079	1	p3	(CCG)4	12	3	14	ACATCAAGAAGGTGCTCGGG	60.036	CTGCACGACCATGTAGCTGA	60.109	215
TRINITY_Contig11081	1	p3	(GGT)4	12	878	889	GCCACCAGTTGAATGCCAAG	60.038	TCGTCCTCCATGTCCGATCT	60.107	205
TRINITY_Contig11082	1	c	(CGCC)3tccgcctcccgctcctcctcgggcg gcgcccaactccacccgttctcctctccctgtcc accataccctagcaggtacaggcggcggcga gtactccac(CGG)4	123	20	142	CAACTCCACCCGTTCTCCTC	60.037	CCGGATCCATTTGCCACCTA	59.817	103
TRINITY_Contig11082	2	p3	(CGG)5	15	248	262	CAACTCCACCCGTTCTCCTC	60.037	CCGGATCCATTTGCCACCTA	59.817	103
TRINITY_Contig11083	1	p3	(CTC)7	21	389	409	GTTCCTGCAACAAGAAGCCG	60.04	CCCACTTGCATACCTGGTGT	59.962	184
TRINITY_Contig11090	1	p3	(CGG)4	12	991	1002	TGGCTGAACATCACCTGCAT	59.961	TCTGTTGGCTGCGTCATTCT	59.965	175
TRINITY_Contig11092	1	p4	(GCCG)3	12	320	331	TTCCCTCCCTCTCCTAACCG	60.032	CCGGTGGTGTACTTCAACCA	59.893	223
TRINITY_Contig11092	2	p3	(CGC)4	12	798	809	TTCCCTCCCTCTCCTAACCG	60.032	CCGGTGGTGTACTTCAACCA	59.893	223
TRINITY_Contig11096	1	c	(GCG)4cggagttcggcgacgccggtacc(GCGCG)3	51	677	727	AGTGGTAGGCTGCTAGGACA	59.959	ATGAAGCAAAAGCACTCGCG	60.11	241
TRINITY_Contig11097	1	p6	(ATTTGA)3	18	397	414	TGATGGGGTGTCGAACTGTG	59.965	GAACATGCATCCACATCCGC	59.97	225
TRINITY_Contig11098	1	c	(CGC)4cggcctcgcccgcctgcttgatgcca(CCG)4	50	652	701	GGCGGCGATAGATAGGTGAG	59.829	TGGTGTCGGTGATGTTCTCG	60.039	171
TRINITY_Contig11098	2	p3	(GCT)7	21	834	854	GGCGGCGATAGATAGGTGAG	59.829	TGGTGTCGGTGATGTTCTCG	60.039	171
TRINITY_Contig11100	1	p3	(CGG)4	12	1760	1771	GTAGCAACGACGAATGACGC	59.977	CCAAAGATGCCTAGCTGCCT	60.107	105
TRINITY_Contig11101	1	p3	(CGC)6	18	78	95	CTGATGATCCTCACCTCGGC	59.967	TGGAAGACGACCTCGAGCTA	60.036	235
TRINITY_Contig11103	1	p4	(CCAG)3	12	20	31	AGGGCAGATAGGGAGCTCAA	60.031	TCAGGGTGTTCACTTCTCGC	59.966	172
TRINITY_Contig11103	2	p3	(GAT)6	18	3201	3218	AGGGCAGATAGGGAGCTCAA	60.031	TCAGGGTGTTCACTTCTCGC	59.966	172
TRINITY_Contig11104	1	p3	(TCG)4	12	618	629	AGCTGATCATCGTCGTCGTC	59.972	TCCCGGCGTATCTCATTTCG	59.968	240
TRINITY_Contig11105	1	p3	(CGG)4	12	178	189	TTGCAGCTGATGAGGGTGAG	60.036	CCTGTGGAGCTTGTACTGCA	59.965	142
TRINITY_Contig11106	1	c	(CGG)4tggctccgaatccgctggccctggtg ctcacttaattttctcgcgtccctcctctgaccc aattccatcggatgctgcgac(GGCGGT)3	112	232	343	TCCCCTCCTCCGATCCAATT	60.03	AGAGGCGAGGTAGGTGATGT	60.033	201
TRINITY_Contig11107	1	p3	(CCG)4	12	889	900	GACCAGGGCCAAGAATGTCA	59.962	AGTGCCGATTGTCTCACCAG	60.037	209
TRINITY_Contig11107	2	p3	(TCG)4	12	1480	1491	GACCAGGGCCAAGAATGTCA	59.962	AGTGCCGATTGTCTCACCAG	60.037	209
TRINITY_Contig11108	1	c	(GCCTG)3ctggctgcacgttgatctttgcg(TGCC)3	50	603	652	GCTCGTAGGAGTCGTACAGC	59.972	CGCGATCGAGTTCCTGATCA	59.971	118
TRINITY_Contig11112	1	p4	(AGCA)3	12	725	736	GGAACGAGCAAGCAAGCAAA	59.969	TTTCACTTGCAATGCTGGCC	59.966	255
TRINITY_Contig11113	1	p4	(CACG)3	12	378	389	CACCCGTCACTGATCTCTCG	59.9	TGCAGATCTGGAGCTTTGGG	60.035	124
TRINITY_Contig11113	2	p6	(CCGGCG)3	18	676	693	CACCCGTCACTGATCTCTCG	59.9	TGCAGATCTGGAGCTTTGGG	60.035	124
TRINITY_Contig11113	3	p3	(GGC)5	15	849	863	CACCCGTCACTGATCTCTCG	59.9	TGCAGATCTGGAGCTTTGGG	60.035	124
TRINITY_Contig11113	4	p3	(GGC)4	12	1000	1011	CACCCGTCACTGATCTCTCG	59.9	TGCAGATCTGGAGCTTTGGG	60.035	124
TRINITY_Contig11114	1	p5	(ATCCA)3	15	115	129	CCGGGTGAGATGGAAAAGCT	60.035	CGCCATGACCTCGTCGAATA	59.97	112
TRINITY_Contig11114	2	p3	(GCG)4	12	413	424	CCGGGTGAGATGGAAAAGCT	60.035	CGCCATGACCTCGTCGAATA	59.97	112
TRINITY_Contig11114	3	p3	(CGC)4	12	650	661	CCGGGTGAGATGGAAAAGCT	60.035	CGCCATGACCTCGTCGAATA	59.97	112
TRINITY_Contig11115	1	p3	(CGC)4	12	368	379	AACAATTGGAACGCCACAGC	59.968	GCGTTAATGGCGGCGATTAG	60.111	104
TRINITY_Contig11115	2	c	(AGC)5acaaggggggcggcgagcgggagc ggctgagcgcgtctcaggacacggggctcacc tccgacgcgcaccccgagat(CTC)5	106	622	727	AACAATTGGAACGCCACAGC	59.968	GCGTTAATGGCGGCGATTAG	60.111	104
TRINITY_Contig11115	3	p4	(GATC)3	12	832	843	AACAATTGGAACGCCACAGC	59.968	GCGTTAATGGCGGCGATTAG	60.111	104
TRINITY_Contig11115	4	p4	(CGAT)3	12	1141	1152	AACAATTGGAACGCCACAGC	59.968	GCGTTAATGGCGGCGATTAG	60.111	104
TRINITY_Contig11115	5	p3	(TGC)5	15	1363	1377	AACAATTGGAACGCCACAGC	59.968	GCGTTAATGGCGGCGATTAG	60.111	104
TRINITY_Contig11115	6	p3	(CGG)4	12	1625	1636	AACAATTGGAACGCCACAGC	59.968	GCGTTAATGGCGGCGATTAG	60.111	104
TRINITY_Contig11116	1	p4	(GATC)3	12	407	418	TGGCACAGAGAGCTGAGTTG	59.966	GAAGGACCCGGGCATTGTTA	60.035	140
TRINITY_Contig11117	1	p3	(CGC)4	12	372	383	GTCGATTAGCTCCGACAGGG	59.969	CTGCTGTTCCCGTTCTACGT	60.039	231
TRINITY_Contig11117	2	p3	(GGC)5	15	1338	1352	GTCGATTAGCTCCGACAGGG	59.969	CTGCTGTTCCCGTTCTACGT	60.039	231
TRINITY_Contig11121	1	p6	(CCACGG)3	18	955	972	CTCTTGCTTGCCTGAAGGGA	59.963	GGCATGGCTGGTATCGATGA	59.965	244
TRINITY_Contig11123	1	p3	(GCG)4	12	1135	1146	TTGAAGACGAGGATGTGGGC	60.036	TGAACCGTGGTCAGTACGTG	59.969	265
TRINITY_Contig11125	1	p3	(CGG)6	18	80	97	CAAATTCTTGGGGGACGGGA	59.961	CCACAAAGCCGAGTTCCTCT	59.965	138
TRINITY_Contig11126	1	p3	(CGA)5	15	195	209	TGCACGGAGAACATGAACGA	59.967	TAGTAGGTGTCGTCGTCGGT	60.037	113
TRINITY_Contig11129	1	p4	(TTTC)3	12	4	15	GGTGCAATACCCCATGTGGA	60.034	GTAACGCTATCTGGCCTGCT	59.895	159
TRINITY_Contig11130	1	p2	(GA)6	12	973	984	CCAGCGAAGAGGAAGAAGCA	60.037	ATCTCCTGCTTGCGCTTCTT	60.036	131
TRINITY_Contig11131	1	c	(GAAAT)3ctccaccccactgatcgatctttc tggccgttcctcttcctcgggagag(GA)25g gaaggggggaaaagaggccgtcgaattcttgc atcgtcgtcttctcgctttgcttggcgtccgcaa gtcaagtccacctcgtcaaagcaagccaac(C T)7	224	253	476	TCCACCCCACTGATCGATCT	60.032	GATGCAAGAATTCGACGGCC	59.972	133
TRINITY_Contig11132	1	p3	(CGG)4	12	326	337	CAGTGCCAAATGTCGTTGCA	59.969	GACGCCCTTCTTGAACTCCA	59.965	137
TRINITY_Contig11133	1	p4	(TCTA)3	12	417	428	CTGAATGGATGTGGCCCTGT	60.034	GTGTGAGAAAGGGAGGGAGC	60.036	169
TRINITY_Contig11133	2	p2	(AG)12	24	968	991	CTGAATGGATGTGGCCCTGT	60.034	GTGTGAGAAAGGGAGGGAGC	60.036	169
TRINITY_Contig11133	3	p2	(TA)9	18	1277	1294	CTGAATGGATGTGGCCCTGT	60.034	GTGTGAGAAAGGGAGGGAGC	60.036	169
TRINITY_Contig11133	4	p3	(CAC)4	12	1605	1616	CTGAATGGATGTGGCCCTGT	60.034	GTGTGAGAAAGGGAGGGAGC	60.036	169
TRINITY_Contig11138	1	p4	(AAGG)3	12	800	811	AGTGACCCTGCCCATCATTG	60.034	CACAAGTCGCCTTCCTTCCT	59.965	279
TRINITY_Contig11140	1	p3	(TCC)4	12	529	540	GCAAAGGACAGGAGGAGGTC	60.036	CCAAAGGGCGAAGAAGAGGT	59.963	117
TRINITY_Contig11140	2	p3	(GCC)4	12	865	876	GCAAAGGACAGGAGGAGGTC	60.036	CCAAAGGGCGAAGAAGAGGT	59.963	117
TRINITY_Contig11140	3	p2	(AG)8	16	997	1012	GCAAAGGACAGGAGGAGGTC	60.036	CCAAAGGGCGAAGAAGAGGT	59.963	117
TRINITY_Contig11141	1	p4	(GCAT)3	12	221	232	TTACTGTGGTCACATGGCCC	59.962	CGTCGTGTACCAGGTCAACA	59.969	265
TRINITY_Contig11141	2	c	(CTT)6gctctc(CGCCGG)3	42	391	432	TTACTGTGGTCACATGGCCC	59.962	CGTCGTGTACCAGGTCAACA	59.969	265
TRINITY_Contig11143	1	c	(CGCCG)3cgcaagtaccggcctcggccgtc gccggcggagggagtgaggaggtcac(GAG G)3	76	916	991	ATCCGCGTGTCAGTGAAAGT	59.966	TGGGACTCAACTACCACCCA	60.105	136
TRINITY_Contig11144	1	c	(GAC)4gagtggaaggggctaatcggggag gaggccttcgggagaggcgcgtggcggatgg gccccacccg(GCT)4	89	725	813	CAGCATGCGCATCCAGTTAC	59.971	AACACTGTCGACGTTCTCCC	59.968	216
TRINITY_Contig11148	1	p2	(GA)7	14	1	14	AAGAACAAGCTCCTCGCCTC	60.037	CCAAAGGCCCAAATTCACCG	60.037	260
TRINITY_Contig11149	1	p3	(CCG)4	12	157	168	ATCAGCACAGGGGGTTGATG	60.034	CCCAGCCAATTACCCACCAT	60.032	244
TRINITY_Contig11150	1	p3	(GCC)7	21	144	164	CTTGCACCCCAAAAGGATGC	60.037	AGCTGCGATGACTATGTCCG	59.969	209
TRINITY_Contig11154	1	p3	(CTG)4	12	672	683	TGTCATACTCCTCCGGCAGA	60.034	GAGAACGCCTTCCTCCTCAC	60.109	100
TRINITY_Contig11154	2	p6	(GGCGAC)3	18	909	926	TGTCATACTCCTCCGGCAGA	60.034	GAGAACGCCTTCCTCCTCAC	60.109	100
TRINITY_Contig11159	1	p3	(CGC)4	12	751	762	GGAAGTAGACGACGAGCGAG	59.973	TCTTCTTCGTGGTGGCCTTC	59.965	279
TRINITY_Contig11160	1	p2	(TC)18	36	7	42	GCGCCACCACAATCAATCTC	59.901	GGGTCGATCTCGTTCTCGTC	59.972	240
TRINITY_Contig11160	2	p3	(AGC)7	21	225	245	GCGCCACCACAATCAATCTC	59.901	GGGTCGATCTCGTTCTCGTC	59.972	240
TRINITY_Contig11161	1	p4	(CCTC)3	12	50	61	TTCCAGCGTTCTCCGAAACA	59.895	TTCTCCAACTTCTCGCAGCG	60.667	157
TRINITY_Contig11161	2	p3	(CCG)6	18	195	212	TTCCAGCGTTCTCCGAAACA	59.895	TTCTCCAACTTCTCGCAGCG	60.667	157
TRINITY_Contig11161	3	p4	(CCGC)4	16	367	382	TTCCAGCGTTCTCCGAAACA	59.895	TTCTCCAACTTCTCGCAGCG	60.667	157
TRINITY_Contig11163	1	p3	(GCG)4	12	770	781	TCTTCTCTCTCTGCTCCGCT	60.035	TGGGGAGGGAAAGAGAGAGG	59.957	138
TRINITY_Contig11167	1	p3	(GCG)4	12	295	306	AAGTTGTAGAGCGCGGTGAA	59.967	CAGAGGAAGTCGTTGCCAGT	59.966	248

TRINITY_Contig11168	1	p2	(CT)7	14	869	882	TGAACTCTTCCTGGTTCGCC	59.965	CATCTGCCTCGACATCCTCC	59.967	152
TRINITY_Contig11169	1	p3	(CTG)4	12	314	325	ATATCTCCGCAATGCTCGCA	59.966	CGTCTGCACTAGCTACCGAG	59.972	162
TRINITY_Contig11171	1	p3	(GCG)4	12	535	546	TGGAATGGAGCAGAGCACAG	60.036	GACATGACCCTAGCCGTAGC	59.968	167
TRINITY_Contig11175	1	p4	(CGAT)3	12	103	114	CGACGACGAGGAGAAGAAGG	59.903	TCCTTGCCCTTCTTCCACAC	59.89	264
TRINITY_Contig11175	2	p3	(GGC)4	12	246	257	CGACGACGAGGAGAAGAAGG	59.903	TCCTTGCCCTTCTTCCACAC	59.89	264
TRINITY_Contig11176	1	p3	(GCG)6	18	471	488	GAAGCTGGTGGCCATGTACT	60.035	CACTACGACAGCTCACTCCG	60.179	111
TRINITY_Contig11178	1	p6	(CGGCGA)3	18	872	889	GGGGGTGGTACCATTCACTG	60.034	AACTGCCAATCGGAGTCCTG	60.036	258
TRINITY_Contig11179	1	p4	(TGGT)3	12	910	921	CGACGAAGAGGATCTCGGTG	59.972	ACTTCTTCTTCGGCGGTCTG	60.038	201
TRINITY_Contig11181	1	p4	(TCCA)3	12	125	136	ACATGCAATAGCCGTGGTGA	60.036	CGAATCCGGTCAATTCGCAC	59.974	161
TRINITY_Contig11182	1	p2	(TA)10	20	857	876	CTGACTTCCTCAGCTGCACA	59.966	GAGCTCCTTGATCTCCTGCC	59.893	261
TRINITY_Contig11184	1	p2	(CT)12	24	210	233	GAGGTGGTTCAACCTGGAGG	59.963	TCTTCATCGGCACCAGGTTC	60.036	239
TRINITY_Contig11184	2	p3	(GCC)4	12	891	902	GAGGTGGTTCAACCTGGAGG	59.963	TCTTCATCGGCACCAGGTTC	60.036	239
TRINITY_Contig11184	3	p3	(GAT)4	12	1080	1091	GAGGTGGTTCAACCTGGAGG	59.963	TCTTCATCGGCACCAGGTTC	60.036	239
TRINITY_Contig11184	4	p3	(GCG)4	12	2054	2065	GAGGTGGTTCAACCTGGAGG	59.963	TCTTCATCGGCACCAGGTTC	60.036	239
TRINITY_Contig11185	1	p3	(CCG)4	12	239	250	TGATGAACTCGCTCGGGATG	59.897	GATACAGTCTGGCAGCAGCA	60.108	172
TRINITY_Contig11185	2	p3	(CAC)4	12	735	746	TGATGAACTCGCTCGGGATG	59.897	GATACAGTCTGGCAGCAGCA	60.108	172
TRINITY_Contig11187	1	p3	(CGG)4	12	265	276	AAGGCAAGCACTCGACTTCA	59.893	TCACCCTCTGCGACATGTTC	60.037	235
TRINITY_Contig11187	2	p4	(CGGG)3	12	819	830	AAGGCAAGCACTCGACTTCA	59.893	TCACCCTCTGCGACATGTTC	60.037	235
TRINITY_Contig11188	1	c	(CTC)4gtcgtcgcaggcgtaggcgtcgtcgtcgcgaccccactcgagagccggggcgagcacgagctgctccctgtccgcccgcgcgccct(CGCCGG)3	116	433	548	CCCCCAACAGAGACAACTCC	59.963	GGTTCTACGCGGAGATGAGG	59.969	240
TRINITY_Contig11188	2	p6	(GGAGCC)3	18	889	906	CCCCCAACAGAGACAACTCC	59.963	GGTTCTACGCGGAGATGAGG	59.969	240
TRINITY_Contig11189	1	p3	(TCA)4	12	393	404	ATGTCTGAAGCCCGTCTGTG	60.037	GCCGGAAATTTCCCCAAACC	60.037	210
TRINITY_Contig11191	1	p3	(CGA)5	15	274	288	GTTCCCAAGATCAAGGCCGA	60.035	ACAGAAAGGTATCAGGGCC	60.034	255
TRINITY_Contig11197	1	p4	(CACG)4	16	148	163	TGACGAACCCCATGAACCAG	59.964	TCCTCTACCTCTCCACTGCC	60.033	231
TRINITY_Contig11197	2	p3	(CGC)4	12	438	449	TGACGAACCCCATGAACCAG	59.964	TCCTCTACCTCTCCACTGCC	60.033	231
TRINITY_Contig11198	1	p3	(CTC)4	12	59	70	TGCTACAGGGGATGCAACAG	60.035	GATGAGGCAGTGAGGGCTTT	60.035	276
TRINITY_Contig11201	1	p3	(GCC)4	12	55	66	TGACGTGCGTGTGGTATGAA	59.968	GGGAGCGTCCACTGGATATG	59.966	117
TRINITY_Contig11201	2	p3	(GGC)5	15	378	392	TGACGTGCGTGTGGTATGAA	59.968	GGGAGCGTCCACTGGATATG	59.966	117
TRINITY_Contig11201	3	c	(GC)6tgaggagcgcgaggagcgccggcaccaactcgttgccgcgctgctcgccaagcttggcggcggctcctcgt(GATC)3	95	609	703	TGACGTGCGTGTGGTATGAA	59.968	GGGAGCGTCCACTGGATATG	59.966	117
TRINITY_Contig11201	4	p5	(TGCTT)3	15	1501	1515	TGACGTGCGTGTGGTATGAA	59.968	GGGAGCGTCCACTGGATATG	59.966	117
TRINITY_Contig11202	1	p3	(GCC)4	12	139	150	TCCGAGTTGCGCTGAGATTT	60.037	TGCCTGGTCTAGTTGCACTG	59.965	199
TRINITY_Contig11206	1	c	(CGC)5gccacctgccgcagttaaccgctccattaatctagacctctcctcttcctcctcctcggctcctcgcttctcc(TG)6	101	627	727	CTCGCTTCTCCTGTGTGTGT	59.967	GATCATCACAGGCTCCCTCG	59.967	146
TRINITY_Contig11207	1	p3	(TGA)5	15	425	439	TGGCTCCGAGTGATAGCAAC	59.824	CCGCGGAAAGACAGATTTGC	60.179	215
TRINITY_Contig11208	1	p3	(CAG)4	12	449	460	GTGCGAAACTGAGGAGGACA	59.966	TCAGCTTCTCGGTGGACAAC	59.966	131
TRINITY_Contig11208	2	p3	(GAC)4	12	1892	1903	GTGCGAAACTGAGGAGGACA	59.966	TCAGCTTCTCGGTGGACAAC	59.966	131
TRINITY_Contig11209	1	p6	(AGCTCT)3	18	886	903	GGTAGCTTGCGCCCTAGTAG	59.968	GCAGCAACTCCAACTTGGTG	59.969	199
TRINITY_Contig11209	2	p3	(CGA)4	12	1377	1388	GGTAGCTTGCGCCCTAGTAG	59.968	GCAGCAACTCCAACTTGGTG	59.969	199
TRINITY_Contig11209	3	p3	(CAG)5	15	1499	1513	GGTAGCTTGCGCCCTAGTAG	59.968	GCAGCAACTCCAACTTGGTG	59.969	199
TRINITY_Contig11209	4	p4	(ATGC)3	12	1771	1782	GGTAGCTTGCGCCCTAGTAG	59.968	GCAGCAACTCCAACTTGGTG	59.969	199
TRINITY_Contig11214	1	p3	(GGA)5	15	875	889	GCAGGACCTCTCGAAGTAGC	59.898	CCACAGATGGAGGTGTTCC	60.035	175
TRINITY_Contig11216	1	p3	(GCT)4	12	696	707	GGACAAGAAGAGGCTTCCCC	60.035	AACCAGCCGCCCTTATTGAA	59.961	144
TRINITY_Contig11216	2	p4	(GGGT)3	12	1630	1641	GGACAAGAAGAGGCTTCCCC	60.035	AACCAGCCGCCCTTATTGAA	59.961	144
TRINITY_Contig11217	1	p3	(GAG)6	18	851	868	GGCGTCTTGAAGAGCAGAGT	60.038	ATTCCAAAACCAACCACGCG	59.969	241
TRINITY_Contig11221	1	p3	(GGC)4	12	829	840	ATCTCGCCTCGACGTCATTC	59.97	TTGCAACAACTTCAGCGACG	59.973	248
TRINITY_Contig11223	1	p5	(GCTGC)3	15	7	21	CAGTACCACGCTCGACAACT	60.039	ACGATTCTGCTTCGACGGTT	60.039	191
TRINITY_Contig11227	1	p3	(CCA)4	12	3418	3429	CACTGTTGCCGGCGAATATG	59.972	GCATGTGCAGGAAACGGTTT	59.968	150
TRINITY_Contig11229	1	p3	(CCG)4	12	1289	1300	GATCAGATCTGCCCGGTGAG	59.967	CGACTTCACCTACCTCTCGC	59.901	156
TRINITY_Contig11229	2	p3	(GCG)5	15	1453	1467	GATCAGATCTGCCCGGTGAG	59.967	CGACTTCACCTACCTCTCGC	59.901	156
TRINITY_Contig11231	1	p3	(ACC)5	15	109	123	CCCGATAAGTACACGGGCTC	59.968	CCAAACGCCTCTTCAACAGC	60.04	176
TRINITY_Contig11232	1	p3	(TCC)5	15	229	243	TGGAGCATTTGGTTGGTGGT	60.106	ATCCGTTTGCAGGTCCGAAT	60.035	182
TRINITY_Contig11237	1	p3	(CTT)5	15	435	449	AACGGCCGTGTGATACAACT	59.966	AGAGTACCCTGCTGACCCAA	60.179	256
TRINITY_Contig11237	2	p3	(CGG)4	12	848	859	AACGGCCGTGTGATACAACT	59.966	AGAGTACCCTGCTGACCCAA	60.179	256
TRINITY_Contig11240	1	p3	(TCC)4	12	717	728	TCAGGCTCTGAACACTGCAG	59.966	GCTAGGAGAGGGACGATTGC	59.967	277
TRINITY_Contig11241	1	p3	(ACG)4	12	583	594	TCAGTTTCTCCAATCCCGGC	60.035	ATCTCGATGTCCGTGATGCC	59.967	187
TRINITY_Contig11242	1	p4	(CTGG)4	16	81	96	CGCATCCGCCTCATTCTAGT	59.967	CGCACTTTCAAGAGGCCAAC	60.04	183
TRINITY_Contig11243	1	p2	(GC)6	12	117	128	AACCAAGTGACACTGCAGCT	60.107	CCATCAATTGGTCCAGGCCA	60.325	164
TRINITY_Contig11243	2	p3	(CCG)5	15	364	378	AACCAAGTGACACTGCAGCT	60.107	CCATCAATTGGTCCAGGCCA	60.325	164
TRINITY_Contig11244	1	p3	(GCG)4	12	435	446	TGTTTTGCCGAGCACCAATG	59.968	GGTGTGGCAGATCATGACCA	60.035	113
TRINITY_Contig11246	1	p4	(TTTC)3	12	825	836	CTTCCGCGTCTTCACACTCT	60.039	CTGCCGCAGATGATGTTGTG	59.902	108
TRINITY_Contig11247	1	p3	(TCC)4	12	172	183	CATCTCGATCAGCTCCACGG	60.319	GAACCTGGTGTCGTGCTCC	60.668	277
TRINITY_Contig11247	2	p3	(GGC)4	12	677	688	CATCTCGATCAGCTCCACGG	60.319	GAACCTGGTGTCGTGCTCC	60.668	277
TRINITY_Contig11250	1	p2	(AC)7	14	101	114	CGAGCTCCTCTTCGTCATCC	59.97	GTGCCGACGTTGTACTGGTA	60.039	144
TRINITY_Contig11251	1	p3	(CGC)4	12	689	700	GCAGGACCAATTTACCGGGA	60.035	CTTTGTTCCCCATTGCCTGC	60.037	138
TRINITY_Contig11251	2	p3	(GGC)4	12	868	879	GCAGGACCAATTTACCGGGA	60.035	CTTTGTTCCCCATTGCCTGC	60.037	138
TRINITY_Contig11252	1	p3	(AAG)4	12	671	682	TATGAAGGTGCTGGTGCCAG	60.035	TCGCTCGCGATCTTCTTCTC	59.972	165
TRINITY_Contig11254	1	c	(GCA)4gccggagcagagcgaggagaggcgcgccgcgcaggacgcgcggtcggcgcgcgccaagctgcggtggaaggccgtggcgcacgagatcatggccaaga(GCG)6cgcggcggaggag(GCA)5	156	277	432	GCGAGGAGTGGAATAGCTCC	59.967	GGCGCAGATGCTTTGTTTCA	60.039	119
TRINITY_Contig11254	2	p4	(GCGG)3	12	581	592	GCGAGGAGTGGAATAGCTCC	59.967	GGCGCAGATGCTTTGTTTCA	60.039	119
TRINITY_Contig11256	1	p2	(CT)14	28	7	34	AAAGAATGGCTCGTGGCTCA	59.963	AGTTTTGTGCTTGCCTTGGC	60.179	139
TRINITY_Contig11257	1	p3	(CGG)6	18	340	357	TTCGCCACCACCTTCTTCTC	59.965	CAAGAAGGAGGCTCCCAAGG	60.035	171
TRINITY_Contig11257	2	p6	(GGCTTC)3	18	836	853	TTCGCCACCACCTTCTTCTC	59.965	CAAGAAGGAGGCTCCCAAGG	60.035	171
TRINITY_Contig11260	1	p3	(GCG)5	15	504	518	AGTAAAGCAGGCCAGCTGAG	60.036	CTCCTTAGCGGCATCCTCAG	59.967	205
TRINITY_Contig11262	1	p3	(GGC)4	12	689	700	ATGCCCAGACAAGCCACAAT	60.252	ACCTCGAGCTACAGTGGGAT	60.033	112
TRINITY_Contig11264	1	p2	(GA)7	14	2065	2078	GCAATGAAAGGGTGCTGCAA	59.966	GACATCAATCACGCGCTCAC	59.974	280
TRINITY_Contig11265	1	p6	(CTGATT)3	18	133	150	GCTCTCGTAGTGCTCCATCC	59.969	GGGATCAAGGAGAACGCCAA	60.035	228
TRINITY_Contig11267	1	p3	(GCG)5	15	13	27	GAGGACTTGGGTTGAGAGGC	60.036	GGGCGACAACACAACAGAAC	59.971	191
TRINITY_Contig11267	2	p2	(GA)8	16	171	186	GAGGACTTGGGTTGAGAGGC	60.036	GGGCGACAACACAACAGAAC	59.971	191
TRINITY_Contig11267	3	p3	(GCA)4	12	360	371	GAGGACTTGGGTTGAGAGGC	60.036	GGGCGACAACACAACAGAAC	59.971	191
TRINITY_Contig11267	4	p3	(GGC)4	12	477	488	GAGGACTTGGGTTGAGAGGC	60.036	GGGCGACAACACAACAGAAC	59.971	191
TRINITY_Contig11267	5	p6	(GAGGAC)3	18	747	764	GAGGACTTGGGTTGAGAGGC	60.036	GGGCGACAACACAACAGAAC	59.971	191
TRINITY_Contig11269	1	p3	(CAC)4	12	129	140	AGATGTGCAGCTCCAGGTTC	60.036	GAAGAGGAAGCACTTCGCCT	60.037	215
TRINITY_Contig11270	1	p5	(GCCCA)3	15	265	279	GAGGCATGGATCGATCTGGG	60.037	ACTACTTCGTCTGCACCGTG	60.039	142
TRINITY_Contig11271	1	p3	(TGC)8	24	559	582	CGAACTCTTCTCCCCCAACC	60.036	TGCAATGCAAGCGATGATCG	59.972	113
TRINITY_Contig11271	2	p3	(GGC)5	15	988	1002	CGAACTCTTCTCCCCCAACC	60.036	TGCAATGCAAGCGATGATCG	59.972	113
TRINITY_Contig11271	3	c	(TGC)4gcttgcggcggctgcatgacctc(CTG)4gtggtggtggttctcctggagcgcggcgaggtcctggacctggtgctgcatcatctgcggcggcggggcgagaagagcagcgggtggtggtgcgccggc(GCG)4	159	1126	1284	CGAACTCTTCTCCCCCAACC	60.036	TGCAATGCAAGCGATGATCG	59.972	113
TRINITY_Contig11271	4	p3	(TGC)4	12	1874	1885	CGAACTCTTCTCCCCCAACC	60.036	TGCAATGCAAGCGATGATCG	59.972	113
TRINITY_Contig11271	5	c	(TTCT)3ctcctcgcgatcactcctatctctctctcctgctggcttcttg(CT)6ccactcctctgtgctgctgtctgtgcagtcgatcaggggcagcagcagatggccacagctg(TCTA)3tagagctggcagcagccgtgcagggaggaagggagagggggaggaggacgaggcagct(AGC)4	208	2288	2495	CGAACTCTTCTCCCCCAACC	60.036	TGCAATGCAAGCGATGATCG	59.972	113
TRINITY_Contig11272	1	p3	(GGC)4	12	926	937	TGCACCTCGCTGAAGCTAAA	59.965	GTTCGGTGTCAAGTTGGTGC	59.971	215
TRINITY_Contig11273	1	c	(GAG)5gccgagttggcttcgtgcggtgcgggcacgtcgttgctgact(TGC)6	75	455	529	CTACCCCATCGCCAGAATCC	59.965	ATGGGCTGCCAAAGGATCTC	60.106	153
TRINITY_Contig11275	1	p3	(ACG)4	12	1359	1370	GTCCGAGTCCGACCTCTACT	60.108	CGCCTTGTTGCTGTTGTACC	60.04	199
TRINITY_Contig11278	1	c	(CGC)5ggccccggcccaccgaggcct(CTC)6(CGC)4	66	325	390	GCCGCTTAGCTTGAACCATG	59.901	ATCATGCCCCTCCCTCTCTT	60.029	256
TRINITY_Contig11279	1	p3	(ACC)4	12	168	179	TCAAGCCCCTTCCACCAATC	59.96	CGCGCCACAAAAGATACCAG	59.903	251
TRINITY_Contig11280	1	p3	(TCG)5	15	280	294	TCCCTATAAATCGCACGGCC	59.966	GCCGAATGAATCTTGGTGCC	59.899	141
TRINITY_Contig11280	2	c	(GCCACG)3ccg(CGT)4	33	401	433	TCCCTATAAATCGCACGGCC	59.966	GCCGAATGAATCTTGGTGCC	59.899	141

TRINITY_Contig11281	1	p5	(CAGCG)3	15	239	253	CACGCGCAACAAAAACCAAC	59.976	ACGCTCTGGTATTGGTGGTG	60.036	159
TRINITY_Contig11281	2	c	(GAC)8ggctccgccggcggcggcgaggag ggcggccccgcccgctgcgccg(CCGC)3	82	545	626	CACGCGCAACAAAAACCAAC	59.976	ACGCTCTGGTATTGGTGGTG	60.036	159
TRINITY_Contig11284	1	p3	(GCC)4	12	282	293	GGTGATGAAGGCCATGAGCT	60.107	ATCTTGCCGACCTTGGACAG	60.036	129
TRINITY_Contig11285	1	p4	(TTGT)3	12	1476	1487	CAAAGGAACAAGGCAGCACC	59.967	GACGTAGGAGAAGCCGATGG	59.969	154
TRINITY_Contig11285	2	p4	(TAGC)3	12	1936	1947	CAAAGGAACAAGGCAGCACC	59.967	GACGTAGGAGAAGCCGATGG	59.969	154
TRINITY_Contig11286	1	p3	(GGC)4	12	186	197	CTCCACCATCTTCCTCGACG	59.899	CGATAACCATGCAAGCCAGC	59.97	191
TRINITY_Contig11288	1	p3	(CCG)4	12	261	272	TCCATGAACTGGCCACCATC	60.034	AGGGTCCCATGCCTTTGATG	60.033	266
TRINITY_Contig11288	2	p3	(CCG)4	12	880	891	TCCATGAACTGGCCACCATC	60.034	AGGGTCCCATGCCTTTGATG	60.033	266
TRINITY_Contig11288	3	p3	(CAG)4	12	2400	2411	TCCATGAACTGGCCACCATC	60.034	AGGGTCCCATGCCTTTGATG	60.033	266
TRINITY_Contig11288	4	p3	(CTC)4	12	3157	3168	TCCATGAACTGGCCACCATC	60.034	AGGGTCCCATGCCTTTGATG	60.033	266
TRINITY_Contig11288	5	p3	(CCT)5	15	3283	3297	TCCATGAACTGGCCACCATC	60.034	AGGGTCCCATGCCTTTGATG	60.033	266
TRINITY_Contig11291	1	c	(CTG)8gttgtgattgtggtggttgctgctgttgc tggtggtgattgtggttgtggttctgatgg(TG C)5	97	2225	2321	TCCATGGTAGCGAGGACTCA	60.034	TCCTAGATTCCGCCTCCCAA	60.032	110
TRINITY_Contig11293	1	p2	(CT)6	12	208	219	AGCTAGCTAGGGAGTGTGCT	60.033	CGATGGCTGCCCAAATCTTG	59.898	134
TRINITY_Contig11293	2	c	(GCC)4caaccacgacgatggcaagcccccca ccacccccgccac(CCT)9	77	786	862	AGCTAGCTAGGGAGTGTGCT	60.033	CGATGGCTGCCCAAATCTTG	59.898	134
TRINITY_Contig11294	1	p3	(GCC)4	12	160	171	TGCATTGGAAGGTGGTCTCC	59.962	CACCACCACGCTCTTCTTCT	59.966	113
TRINITY_Contig11294	2	c	(CAG)5agggagctggagagcagcaa(CAG)4	47	346	392	TGCATTGGAAGGTGGTCTCC	59.962	CACCACCACGCTCTTCTTCT	59.966	113
TRINITY_Contig11294	3	p3	(AAG)4	12	595	606	TGCATTGGAAGGTGGTCTCC	59.962	CACCACCACGCTCTTCTTCT	59.966	113
TRINITY_Contig11295	1	p3	(GGC)4	12	686	697	ACGAGAAGAGGGGATCGACA	60.034	TCACCATCATGCCCAAGGAC	60.034	116
TRINITY_Contig11298	1	p5	(TCTGC)3	15	256	270	ACCGTCACCTTCGACCTCTA	59.963	TCTGCAGTCCCCATCCCATA	60.03	137
TRINITY_Contig11302	1	p4	(CTGC)3	12	1133	1144	TTTCCTGCCGCAAAGAAAGC	59.968	GGAAGTGCTGCTGAGTTCCT	59.964	173
TRINITY_Contig11303	1	p4	(GAGC)3	12	17	28	TTCTCGCCTTTCGTTCTCGT	59.687	CACAGCTGCGAAGAGTGAGA	60.039	128
TRINITY_Contig11303	2	p4	(AGCT)3	12	656	667	TTCTCGCCTTTCGTTCTCGT	59.687	CACAGCTGCGAAGAGTGAGA	60.039	128
TRINITY_Contig11303	3	p2	(CT)6	12	931	942	TTCTCGCCTTTCGTTCTCGT	59.687	CACAGCTGCGAAGAGTGAGA	60.039	128
TRINITY_Contig11311	1	c	(CCT)6cgtggccgcgctcctcctccccgcccc ggcggcctccgccgcggacgctcccgcctcg(GCGTCC)3	94	57	150	GCCCCTCAGTCAATGTCCAA	59.962	CAGCGATTGGAATGCCATGG	59.968	103
TRINITY_Contig11311	2	p3	(CAG)5	15	680	694	GCCCCTCAGTCAATGTCCAA	59.962	CAGCGATTGGAATGCCATGG	59.968	103
TRINITY_Contig11312	1	p5	(AGGAG)3	15	228	242	GCATGCCGCTCCTGAGATAT	60.037	CGAAAAGCTTGTGGGCGAAA	59.97	116
TRINITY_Contig11315	1	p6	(CGCCCC)3	18	249	266	AAACCGTGAGCTCTCTCGTG	60.039	TCGTCAGGCGCTTGATTTCT	60.037	100
TRINITY_Contig11315	2	p3	(CCG)4	12	497	508	AAACCGTGAGCTCTCTCGTG	60.039	TCGTCAGGCGCTTGATTTCT	60.037	100
TRINITY_Contig11317	1	p3	(GCC)4	12	137	148	AGCTACATGGAGGCCACCTA	60.031	ATGGAGCTTCCCGAGTAGGT	60.032	244
TRINITY_Contig11317	2	p3	(TCC)5	15	291	305	AGCTACATGGAGGCCACCTA	60.031	ATGGAGCTTCCCGAGTAGGT	60.032	244
TRINITY_Contig11318	1	p3	(GCG)4	12	512	523	GGTGATGGAGATGTGTCCCG	60.179	CTGCTCGCCTCCTACTTCTG	59.898	123
TRINITY_Contig11318	2	p3	(GCG)4	12	818	829	GGTGATGGAGATGTGTCCCG	60.179	CTGCTCGCCTCCTACTTCTG	59.898	123
TRINITY_Contig11319	1	p3	(GCG)4	12	860	871	TTTGCACGCCATTAGCATCG	59.901	GAGACGATGGCCATGAGGAG	59.967	262
TRINITY_Contig11319	2	p4	(TGGC)3	12	1147	1158	TTTGCACGCCATTAGCATCG	59.901	GAGACGATGGCCATGAGGAG	59.967	262
TRINITY_Contig11319	3	p5	(TGCAA)3	15	1855	1869	TTTGCACGCCATTAGCATCG	59.901	GAGACGATGGCCATGAGGAG	59.967	262
TRINITY_Contig11321	1	p3	(AGC)4	12	160	171	TCGGCTTCTACCTCCTCTCC	60.107	CATGGACAGCAGCAGGAAGA	60.036	251
TRINITY_Contig11322	1	p3	(GCA)4	12	321	332	TGCCCTACTCGCAGCATATG	59.966	CTGCATCTGTCGCTTCATGC	59.972	223
TRINITY_Contig11322	2	p4	(CAAC)3	12	920	931	TGCCCTACTCGCAGCATATG	59.966	CTGCATCTGTCGCTTCATGC	59.972	223
TRINITY_Contig11323	1	c	(CT)8g(AAGC)4	33	83	115	ACGGCGCTGCAAGAAAATTT	59.968	ATTGGCGTCTCGTTGTCCAT	60.036	181
TRINITY_Contig11326	1	p5	(CATGG)4	20	1263	1282	GTACGCGAAGTAGGGGTAGC	59.97	GTGTTCAAGGGCAACGACAC	59.971	261
TRINITY_Contig11327	1	p3	(CGC)4	12	1091	1102	TAACACAGCTTGCTCCCTGG	59.963	TCGACTTTGGGGGCTAACAC	59.964	135
TRINITY_Contig11327	2	c	(GCC)5ggcacagcagaggacgacgcgcgg(TTC)4	51	1228	1278	TAACACAGCTTGCTCCCTGG	59.963	TCGACTTTGGGGGCTAACAC	59.964	135
TRINITY_Contig11327	3	p3	(GCT)4	12	1457	1468	TAACACAGCTTGCTCCCTGG	59.963	TCGACTTTGGGGGCTAACAC	59.964	135
TRINITY_Contig11328	1	p4	(GCCA)3	12	229	240	GTAACGGCAACATGATCGGC	59.972	TGCCGCCCTGATTTTTGTTG	59.967	153
TRINITY_Contig11328	2	c	(CCG)4cgccgccactctgcgacgtctgcaag gtgccgtgagcctgtgcctgacccctcgtcggt gtgcttcggccggagagccggatcggagctgg (TGC)4	115	415	529	GTAACGGCAACATGATCGGC	59.972	TGCCGCCCTGATTTTTGTTG	59.967	153
TRINITY_Contig11329	1	p3	(GCC)6	18	752	769	ATGGTTGTACTGAGCGTGCA	59.965	CACATTTTGAGCCGCGAGAG	59.904	255
TRINITY_Contig11330	1	p6	(GCCGGC)3	18	401	418	ATTCGTAAACTCGAGCGGCA	60.109	ACTTGCAGTAGCTGGAGTCG	59.755	258
TRINITY_Contig11332	1	p3	(GGC)6	18	343	360	TGAGAGCTGGACATGCATGG	60.107	ATGGCATGAGAAAGACGGCA	60.035	187
TRINITY_Contig11333	1	p5	(GATCG)3	15	259	273	ACGCTTCCTTTTCTAGCCCC	60.035	AGAGGGTTATGGGAGGGCTT	59.954	128
TRINITY_Contig11334	1	p3	(GCG)4	12	329	340	AGCTTGAGATCTCGCGGATG	59.968	AACATGCTTATCCGCCCGAA	60.107	166
TRINITY_Contig11335	1	p3	(GCG)7	21	853	873	CAGCGCTACTAGCACATCGA	59.971	ATGGACTGCCTCAAGCTCAC	60.036	147
TRINITY_Contig11337	1	c	(GAGG)3gagagg(CGC)4	30	470	499	TGGCATCCAACAATACCGCT	60.035	TCTCCTTCTCCCTCCGAGTG	60.034	257
TRINITY_Contig11338	1	p3	(CGT)4	12	570	581	ATCTCGCCTCGACGTCATTC	59.97	TTGCAACAACTTCAGCGACG	59.973	248
TRINITY_Contig11338	2	p3	(GGC)4	12	778	789	ATCTCGCCTCGACGTCATTC	59.97	TTGCAACAACTTCAGCGACG	59.973	248
TRINITY_Contig11339	1	p3	(GAA)4	12	823	834	ACAACACCTCATCATCGCGT	60.037	TTGCTTGCGTTGGGATTTCG	60.04	132
TRINITY_Contig11340	1	p4	(GCCG)3	12	480	491	GAGGTAGGGATTCCATGGCG	59.965	CACCTGCACTCACCCAGAAT	59.963	117
TRINITY_Contig11345	1	p3	(AGG)4	12	807	818	TCCGGAACTTGGACTTGAGC	59.965	CGGGATGATGCACTTCCAGT	60.108	197
TRINITY_Contig11350	1	p3	(GGA)5	15	1	15	CCTCCGTTCACGTAAAGCGA	60.388	AAACGACAAGGAACGCAAGC	59.971	133
TRINITY_Contig11350	2	p4	(TCGC)3	12	730	741	CCTCCGTTCACGTAAAGCGA	60.388	AAACGACAAGGAACGCAAGC	59.971	133
TRINITY_Contig11351	1	p4	(AGCC)4	16	114	129	TCCATTCCCATTCCTCCCCT	59.952	TCGAATGCCAAGTCTGTCCC	60.036	113
TRINITY_Contig11351	2	p3	(GCC)4	12	323	334	TCCATTCCCATTCCTCCCCT	59.952	TCGAATGCCAAGTCTGTCCC	60.036	113
TRINITY_Contig11351	3	p4	(TTGA)3	12	1493	1504	TCCATTCCCATTCCTCCCCT	59.952	TCGAATGCCAAGTCTGTCCC	60.036	113
TRINITY_Contig11352	1	p3	(GCC)6	18	120	137	ACTGCATGCTCTTGGAACGA	59.965	AAGCAGTAGCAACCTGTGGG	60.251	187
TRINITY_Contig11352	2	p5	(CGCGG)3	15	322	336	ACTGCATGCTCTTGGAACGA	59.965	AAGCAGTAGCAACCTGTGGG	60.251	187
TRINITY_Contig11355	1	p3	(CGC)4	12	248	259	TGAACAAACGTGCATGCGAG	60.041	ATCTTCCATGGCACGTCGTT	60.036	260
TRINITY_Contig11358	1	p4	(TCCC)3	12	228	239	CAAACAAACCACGGCCAGAG	59.969	GGAGGGAAAGCTTCTGTGCT	59.963	151
TRINITY_Contig11360	1	p3	(CGC)4	12	271	282	GAGAATCTTTGACGGGGGCA	60.035	GCGAAACAAGATCGTCTGGC	59.905	183
TRINITY_Contig11360	2	p3	(GTA)4	12	1940	1951	GAGAATCTTTGACGGGGGCA	60.035	GCGAAACAAGATCGTCTGGC	59.905	183
TRINITY_Contig11360	3	p3	(GCA)7	21	2528	2548	GAGAATCTTTGACGGGGGCA	60.035	GCGAAACAAGATCGTCTGGC	59.905	183
TRINITY_Contig11361	1	p3	(CCA)4	12	102	113	ATCCTCTCCCCTCCACACTC	60.032	TTTATCGCCGCTGGTTCTGT	60.037	241
TRINITY_Contig11362	1	p3	(GGC)4	12	49	60	GCTGCCTTGACGCCTTATTG	59.901	ACCTTTGGCATGGACGCATA	60.035	234
TRINITY_Contig11363	1	p6	(TCCCCG)3	18	725	742	GCAGGGTACACCGTACAACA	59.966	GAGGGGTGTGAGGGTGATTG	60.035	116
TRINITY_Contig11365	1	p4	(GGAG)3	12	779	790	GCTTCTCCAAGGTCCATGCT	60.035	GGAAAAAGGACAAGCGCCTG	60.039	228
TRINITY_Contig11365	2	p6	(GAGGGG)3	18	910	927	GCTTCTCCAAGGTCCATGCT	60.035	GGAAAAAGGACAAGCGCCTG	60.039	228
TRINITY_Contig11366	1	p3	(TCG)5	15	106	120	TTCGCACAACCAGCTCATCT	59.965	CGACGCTGCACATGATTGAG	59.974	215
TRINITY_Contig11367	1	p3	(CCT)4	12	189	200	GCACCTCCTCCTCCTCTGTA	60.033	GAGGCGCGATCTCTTACGAA	59.971	121
TRINITY_Contig11368	1	p3	(GCG)4	12	1938	1949	CCTTCCCTTCAGGTGGCAAT	59.96	TATCCTCCACCAAGAGCCGA	60.033	185
TRINITY_Contig11368	2	p5	(GTGGA)3	15	2156	2170	CCTTCCCTTCAGGTGGCAAT	59.96	TATCCTCCACCAAGAGCCGA	60.033	185
TRINITY_Contig11368	3	p2	(TG)9	18	2664	2681	CCTTCCCTTCAGGTGGCAAT	59.96	TATCCTCCACCAAGAGCCGA	60.033	185
TRINITY_Contig11369	1	p3	(ACA)4	12	130	141	CGCAGCTACAAGGACTCCAA	60.037	CGAATTGCTTCTACGGCGTG	59.975	209
TRINITY_Contig11369	2	c	(CTT)4ctcctccggtggaggcgatggttgt(GGCAAA)3	55	1667	1721	CGCAGCTACAAGGACTCCAA	60.037	CGAATTGCTTCTACGGCGTG	59.975	209
TRINITY_Contig11369	3	p3	(CCG)7	21	2716	2736	CGCAGCTACAAGGACTCCAA	60.037	CGAATTGCTTCTACGGCGTG	59.975	209
TRINITY_Contig11369	4	p3	(CAG)4	12	3463	3474	CGCAGCTACAAGGACTCCAA	60.037	CGAATTGCTTCTACGGCGTG	59.975	209
TRINITY_Contig11370	1	p3	(TCG)4	12	488	499	ACCATGATCCGATGACGACG	59.969	CGATCGACGGGTAGTTGAGG	59.971	258
TRINITY_Contig11372	1	p3	(CGC)4	12	788	799	GAGAGTGTCGCCGACTTTGA	60.039	GCTTGTTGCTTCCTCTGTGC	60.04	223
TRINITY_Contig11375	1	p4	(CTAG)4	16	85	100	CGGAGGCGTACAGGAACTAC	59.9	CTCTTGTACAGCGTCCCGTT	60.039	187
TRINITY_Contig11375	2	p6	(CGCCGT)3	18	462	479	CGGAGGCGTACAGGAACTAC	59.9	CTCTTGTACAGCGTCCCGTT	60.039	187
TRINITY_Contig11376	1	c	(GAGGAA)3gagagcagaagaaaatgaga tggagagagtc(AG)7	63	141	203	TCTTCTCCTGCTGCTTGCTC	60.037	TGTGATGGCAGGTCTGTGTC	59.965	180
TRINITY_Contig11376	2	p4	(TCGA)3	12	811	822	TCTTCTCCTGCTGCTTGCTC	60.037	TGTGATGGCAGGTCTGTGTC	59.965	180
TRINITY_Contig11377	1	p3	(GCG)4	12	506	517	AGCTTCACCTTGTAGACCGC	60.037	TCCGGTTCTCTGCAACTTCC	59.965	156
TRINITY_Contig11377	2	p5	(TGGGA)3	15	697	711	AGCTTCACCTTGTAGACCGC	60.037	TCCGGTTCTCTGCAACTTCC	59.965	156
TRINITY_Contig11378	1	p3	(AGC)5	15	387	401	GCCAAGAAGGTGGTGCTACT	59.963	TCTATGGTCCCACTCCCAGG	60.031	270
TRINITY_Contig11383	1	p3	(GGC)4	12	131	142	TCCCCTACCAAAACCTGCAC	59.888	GCGTGAACGTCGTTATCAGC	59.977	187
TRINITY_Contig11384	1	c	(TTCTT)3tc(CTTTT)4	37	607	643	AGACAAGGGCAGTTGGCTAC	59.963	GATCATGGCGCTTTCCGTTC	59.972	107
TRINITY_Contig11389	1	c	(CGC)4agctctgggacccgccgccgctgccg tactccccctcagctcccgcagcggatgagtac tcctcgtcgtcgagcttccggcgtcggagcg(C GC)4	114	638	751	GCAGCGGATGAGTACTCCTC	59.969	GGAGCGGTACTGGTCAACAA	59.966	255
TRINITY_Contig11393	1	p3	(CGG)5	15	417	431	CTAGCTAGACACGGGAAGCG	59.97	CGCTTGTAGTGGTCCTTGGT	59.965	265
TRINITY_Contig11399	1	c	(ACCTCC)3a(CCACCG)4	43	607	649	GGGCAAGCTTGGTTCCTTTG	59.966	AGAGGAAGGAGGGCTCTCTG	60.032	258
TRINITY_Contig11400	1	p3	(CGG)5	15	267	281	GAGCTCGCTGGACTATCACC	59.969	GAAGGTCTTGTCGACGCAGA	60.039	274
TRINITY_Contig11401	1	p4	(CATG)3	12	408	419	GCTCAACTGGTACGAGCACT	60.038	GAGGGCAAGGTTGGAATCCA	59.96	213

TRINITY_Contig11401	2	p2	(GC)7	14	710	723	GCTCAACTGGTACGAGCACT	60.038	GAGGGCAAGGTTGGAATCCA	59.96	213
TRINITY_Contig11403	1	c	(CTCG)3ctaccggctgcatctacaatggcgcgcttccagctcgcggccctggccctggccatgctctt(CGC)4	86	230	315	TCGGCATTCGATTCGGTAGG	59.968	ATGGGGGATCACAACACCAC	59.96	100
TRINITY_Contig11403	2	c	(GCC)5gtg(GCC)5	33	623	655	TCGGCATTCGATTCGGTAGG	59.968	ATGGGGGATCACAACACCAC	59.96	100
TRINITY_Contig11403	3	p4	(TTGG)3	12	826	837	TCGGCATTCGATTCGGTAGG	59.968	ATGGGGGATCACAACACCAC	59.96	100
TRINITY_Contig11404	1	c	(ATCG)3atcagtgccgtcatcgttcgccgccgagcacagccggcagccgagaggggtcgagagggccggggcagatgggaagcgt(CGG)4ctggcc(GGA)5	124	168	291	GTGATCCCCTGAAGCCACAA	59.962	GCCCCTCGAAGGAAATGGAA	60.034	159
TRINITY_Contig11404	2	p3	(CGC)4	12	511	522	GTGATCCCCTGAAGCCACAA	59.962	GCCCCTCGAAGGAAATGGAA	60.034	159
TRINITY_Contig11404	3	p5	(CTCCG)3	15	626	640	GTGATCCCCTGAAGCCACAA	59.962	GCCCCTCGAAGGAAATGGAA	60.034	159
TRINITY_Contig11405	1	p3	(GCC)5	15	103	117	TTCCTGCCCTACTTCCCCTT	60.179	ATCGGTCAACCAGTTCACCC	59.964	220
TRINITY_Contig11405	2	c	(CCG)4cactactccggcgcctgggccgtgaccgccgcctcgtcgccggcagcgacgctgctagcagtcatgctctccgggt(TGC)5agcagtggtagatctgctgcagcagtagtgaactgttccttcacttacactagagg(GGGT)3	171	559	729	TTCCTGCCCTACTTCCCCTT	60.179	ATCGGTCAACCAGTTCACCC	59.964	220
TRINITY_Contig11408	1	c	(CT)10ccccctcactcttcaccggttggac(GAA)5	60	1	60	CACCGGTTGGACGAAGAAGA	59.967	ACTACCTGGAGGATGTCGCT	60.033	270
TRINITY_Contig11409	1	p3	(CGC)5	15	385	399	CGATGAGCTCGGTGGAGTAC	59.97	ACATGCCCGGTGTCTAGTTG	60.036	148
TRINITY_Contig11409	2	p3	(CCG)4	12	591	602	CGATGAGCTCGGTGGAGTAC	59.97	ACATGCCCGGTGTCTAGTTG	60.036	148
TRINITY_Contig11409	3	c	(CTT)4ccggcgttctcgggcgtc(TGAT)3	42	811	852	CGATGAGCTCGGTGGAGTAC	59.97	ACATGCCCGGTGTCTAGTTG	60.036	148
TRINITY_Contig11409	4	p4	(TGTC)3	12	1989	2000	CGATGAGCTCGGTGGAGTAC	59.97	ACATGCCCGGTGTCTAGTTG	60.036	148
TRINITY_Contig11413	1	p2	(CT)12	24	648	671	TTCACGCTCATGTCCTGCAT	60.036	TCCATCCAGACACACAGCAC	59.965	272
TRINITY_Contig11414	1	c	(CCCAAC)3cccggcggcgggggcccgtggtt(CGG)5	56	375	430	GCCCTGTGTGTCAGTAGCTT	59.965	ACATACCACGAGCTGAGCAC	60.109	118
TRINITY_Contig11414	2	p3	(CGG)4	12	629	640	GCCCTGTGTGTCAGTAGCTT	59.965	ACATACCACGAGCTGAGCAC	60.109	118
TRINITY_Contig11414	3	c	(TAGC)3tattagtactagtgcactactaccagtggtttgcttgtgatggagctgtgggagcttgattagcgtggtgctcagctcgtggtatgtgc(GT)6	113	755	867	GCCCTGTGTGTCAGTAGCTT	59.965	ACATACCACGAGCTGAGCAC	60.109	118
TRINITY_Contig11416	1	p3	(TGT)5	15	1696	1710	CTTCTTCCTCGCGCAATTCG	59.974	AGCCCCAGAAAGAACAGCTC	59.963	193
TRINITY_Contig11417	1	p4	(CTGC)3	12	498	509	GCATTGCTGATTGCTCCCAG	59.898	CAGGTGTGGGCTCCAGTATC	59.821	103
TRINITY_Contig11420	1	p3	(CGC)4	12	440	451	TTCCTTTCTCGTTCCACCCG	59.966	CGTGCTTCTTGATCTTGCCG	59.904	237
TRINITY_Contig11422	1	p5	(GGGAG)3	15	1294	1308	TGCTCTGAAATGGTCCACCC	59.962	CAGATGTCCCATCCCCGAAG	59.893	237
TRINITY_Contig11423	1	c	(CCA)4attcaaccg(CGACC)3caaccccccgcacgcattcccacct(CCTC)3cccccagatctctctccccccct(GC)6	106	209	314	GTCGCAACAAATCCTACGCC	59.904	GCAGTCTGAGCCACAGTTCT	59.965	169
TRINITY_Contig11423	2	p3	(ATG)4	12	1531	1542	GTCGCAACAAATCCTACGCC	59.904	GCAGTCTGAGCCACAGTTCT	59.965	169
TRINITY_Contig11424	1	p3	(GGC)4	12	822	833	TCCATCTAACCCGCTCCAGA	60.033	CTACAAGCGCACCAAGAAGC	59.833	175
TRINITY_Contig11426	1	p3	(CAC)4	12	305	316	AGCTAAGCCATGCTCGCTAG	59.967	GGAGCTGGAGTGGGGAGATA	60.105	244
TRINITY_Contig11426	2	p6	(CTCCAG)3	18	437	454	AGCTAAGCCATGCTCGCTAG	59.967	GGAGCTGGAGTGGGGAGATA	60.105	244
TRINITY_Contig11429	1	p3	(ATC)4	12	842	853	GGATTTGCCGCCAACACTTT	59.967	ATGATGATTTTTCGCCGCCG	59.971	157
TRINITY_Contig11432	1	p3	(GCG)4	12	996	1007	ACAGTGGCCCTGGTTTTTCA	60.033	TCTCACGTTTCTCACCCGTG	59.968	205
TRINITY_Contig11433	1	p2	(TC)18	36	290	325	GGACGGCAAGAAGAAGGACA	59.965	TGAACTCGCTCTTGGTCCAC	59.966	119
TRINITY_Contig11433	2	p3	(CGC)4	12	761	772	GGACGGCAAGAAGAAGGACA	59.965	TGAACTCGCTCTTGGTCCAC	59.966	119
TRINITY_Contig11433	3	c	(GAA)5gcaggagcacgac(GCG)4	40	1085	1124	GGACGGCAAGAAGAAGGACA	59.965	TGAACTCGCTCTTGGTCCAC	59.966	119
TRINITY_Contig11437	1	p4	(AGCG)3	12	2186	2197	GGTACGTTCAACTGGGTCGT	59.967	CAATTAATCGCTGCACCCG	59.971	144
TRINITY_Contig11440	1	c	(CG)7tcatcagatgctcttcctgccgccgtcgaggtcgagccgtacgcccgcgcgcc(GCG)6	85	285	369	GTTTCACATGTGTGCCCCAC	59.968	AAGAGCGCGCTAGGAGATTC	59.969	113
TRINITY_Contig11440	2	p3	(CGC)4	12	633	644	GTTTCACATGTGTGCCCCAC	59.968	AAGAGCGCGCTAGGAGATTC	59.969	113
TRINITY_Contig11442	1	p4	(GAAA)3	12	1046	1057	CTGCCAACAACAAAGTCCCG	59.969	AGATGGGCATTGCTTCAGCT	60.033	241
TRINITY_Contig11443	1	p4	(CTGC)3	12	26	37	CCTCCCCGTCTTGCTGAAAT	60.035	GCAGCAGCTCCTCTTTCTCA	60.037	191
TRINITY_Contig11443	2	p6	(CCATCC)3	18	153	170	CCTCCCCGTCTTGCTGAAAT	60.035	GCAGCAGCTCCTCTTTCTCA	60.037	191
TRINITY_Contig11443	3	c	(CGC)5agcagcagacgccccagtccgccgccgcccaggagctgggcgggcccttcaggtaccagccgctccaccaccacgccctgccgccgcag(CAC)6	122	505	626	CCTCCCCGTCTTGCTGAAAT	60.035	GCAGCAGCTCCTCTTTCTCA	60.037	191
TRINITY_Contig11443	4	p3	(CAC)5	15	753	767	CCTCCCCGTCTTGCTGAAAT	60.035	GCAGCAGCTCCTCTTTCTCA	60.037	191
TRINITY_Contig11443	5	p3	(GGC)5	15	1089	1103	CCTCCCCGTCTTGCTGAAAT	60.035	GCAGCAGCTCCTCTTTCTCA	60.037	191
TRINITY_Contig11443	6	p3	(GCT)5	15	1373	1387	CCTCCCCGTCTTGCTGAAAT	60.035	GCAGCAGCTCCTCTTTCTCA	60.037	191
TRINITY_Contig11443	7	p3	(GCG)4	12	1799	1810	CCTCCCCGTCTTGCTGAAAT	60.035	GCAGCAGCTCCTCTTTCTCA	60.037	191
TRINITY_Contig11443	8	p2	(GC)6	12	2292	2303	CCTCCCCGTCTTGCTGAAAT	60.035	GCAGCAGCTCCTCTTTCTCA	60.037	191
TRINITY_Contig11446	1	c	(ACC)4ccaattggtcaacatggcgatggctgccgcagcctccgc(CCT)7gctcggcc(CCG)4	92	93	184	CCCAATTGGTCAACATGGCG	60.109	GTCGAGGAAGAGCTCGAAGG	59.9	224
TRINITY_Contig11446	2	p3	(GGC)6	18	567	584	CCCAATTGGTCAACATGGCG	60.109	GTCGAGGAAGAGCTCGAAGG	59.9	224
TRINITY_Contig11447	1	c	(AGG)5aagaggagagcggcggcgcggcggctatcgggtgccggccgccgggcgcggccccggctcatggccagagcgcgcgcgccgcc(CCCT)3	110	1092	1201	TTCGCCTTGTACTGCTCCAG	60.037	GAGTTCAGCTGGGACGTCAA	59.966	116
TRINITY_Contig11447	2	p5	(TTGCG)3	15	1334	1348	TTCGCCTTGTACTGCTCCAG	60.037	GAGTTCAGCTGGGACGTCAA	59.966	116
TRINITY_Contig11448	1	p3	(GCC)5	15	374	388	CGGAGCTCGAGGGTTTTTCT	60.037	CGTGAGTAGCACTGCTAGGG	59.899	114
TRINITY_Contig11448	2	p3	(CCG)5	15	540	554	CGGAGCTCGAGGGTTTTTCT	60.037	CGTGAGTAGCACTGCTAGGG	59.899	114
TRINITY_Contig11450	1	c	(CCG)5aagcaggagcccgcc(CCG)4aaggtggtggagatcccctacccgtgg(CCGTAC)3ccgttcccggcgtggccgtccgagtgctgctgccaccacgggcacggcgggtgccactgctgctcctgcggcaaggcccccgaggccccccc(GCCGCA)3gtacatcccgatgccgcagtacgtgccgcagccgtaccccctgcaacccct(GCG)4	259	122	380	CCAAGAAGGAGGAGCCCAAG	60.035	GTAGGGGATCTCCACCACCT	60.031	170
TRINITY_Contig11453	1	p3	(CAC)5	15	220	234	TTTGCACATCAGGATCCCCC	60.033	TTCACGCCTCTCCACTTGTC	59.966	231
TRINITY_Contig11453	2	p2	(GC)6	12	506	517	TTTGCACATCAGGATCCCCC	60.033	TTCACGCCTCTCCACTTGTC	59.966	231
TRINITY_Contig11456	1	p3	(CCG)4	12	711	722	ATGCCAGCTTCATCACCGAA	60.035	GTGTACGGGGACATTAGCGT	59.826	108
TRINITY_Contig11459	1	c	(TG)6tacgctggcacggtgatttcggctttgccgtcgtcgtgttgcgagttgcgatagatcgtgatgagctgagatgtgc(CGAT)3	100	245	344	CGTGCAGTGCTTTCTTGGTC	60.041	CTATCGCAACTCGCAACACG	59.976	159
TRINITY_Contig11459	2	c	(CGT)4ccatcccctcgtagtcgtggcagcacgcggtgccgccgcaggaggagtaggaggaggacgacgaggcggatgaggagtcctctgccgccgcg(GCGCGT)3cgcgggtccgggtgcgggcacgggcacgactccgcgacgctctctgtgtcgctgtcgtactccatctcgatgccggcgc(GGGA)3gcagcgcgaagggcggcggcgggtgcagggtggccatggcgga(GGGAG)5	281	626	906	CGTGCAGTGCTTTCTTGGTC	60.041	CTATCGCAACTCGCAACACG	59.976	159
TRINITY_Contig11460	1	p3	(GCG)5	15	3920	3934	TCCCTCCATCGAGCTTCTCA	60.033	CGCTGATGCTACCAGAGGAG	59.968	143
TRINITY_Contig11463	1	p3	(CGG)4	12	229	240	GCACGCATAATGCTCAGTCG	60.043	ACATGGAGTACAGGCGGTTG	60.036	187
TRINITY_Contig11465	1	p4	(ACCA)3	12	308	319	AATTTGGTAGGGAGGCGGTG	60.034	TAACGTAACAGGCCACCACC	59.965	233
TRINITY_Contig11465	2	c	(CAC)5cgccagcca(AGCC)3	36	1044	1079	AATTTGGTAGGGAGGCGGTG	60.034	TAACGTAACAGGCCACCACC	59.965	233
TRINITY_Contig11468	1	p4	(ATTT)3	12	843	854	AGCCTACCCGCGAAAATCAA	60.036	GCAGCAGACAAGACCAAAGC	60.04	229
TRINITY_Contig11469	1	p3	(CGC)7	21	64	84	TGCTGCGGAATGGTGTAGTT	59.964	CACGGGAGGTGGAACAATCA	59.964	210
TRINITY_Contig11472	1	p3	(GCG)4	12	1075	1086	GGGGGCCATCACAAAACAAC	59.965	CGTTTGGTCCGGTTTGGTTC	59.97	100
TRINITY_Contig11472	2	p3	(CGG)4	12	2093	2104	GGGGGCCATCACAAAACAAC	59.965	CGTTTGGTCCGGTTTGGTTC	59.97	100
TRINITY_Contig11472	3	p3	(GCT)6	18	2368	2385	GGGGGCCATCACAAAACAAC	59.965	CGTTTGGTCCGGTTTGGTTC	59.97	100
TRINITY_Contig11476	1	p6	(GGGAGC)3	18	332	349	GAGTGGGATGAAGCCACGAA	60.036	AATCCTAGGGGCAAGCACAC	60.034	156
TRINITY_Contig11479	1	p3	(CGA)4	12	291	302	ATTTCAGAGTCACAGCGCCA	59.965	GCCATCCTCTGCCACTTCTT	60.035	153
TRINITY_Contig11480	1	p3	(CCG)4	12	535	546	TGCTTGCACGTATGGTCTGT	59.965	CAAATTTCCTACGGGCCCCT	60.034	113
TRINITY_Contig11480	2	p4	(GTTT)3	12	692	703	TGCTTGCACGTATGGTCTGT	59.965	CAAATTTCCTACGGGCCCCT	60.034	113
TRINITY_Contig11482	1	p4	(ACGT)3	12	1011	1022	ACACAAGCCCACAACAGACA	60.035	TTTTTCAAACGTCGGGCGAC	59.972	245
TRINITY_Contig11484	1	p5	(CCTCC)3	15	61	75	CTGCCACCAAACGGAAACTG	59.969	CTGGATCTTGGCCTTGACGT	60.036	149

TRINITY_Contig11486	1	c	(GAA)5aatcgatcctgccagcaa(CATT)3	45	240	284	AAGACGCACTCACATCACGT	59.968	AAGATCACCGTCGAGGAGGA	60.034	246
TRINITY_Contig11486	2	p3	(CGC)4	12	473	484	AAGACGCACTCACATCACGT	59.968	AAGATCACCGTCGAGGAGGA	60.034	246
TRINITY_Contig11486	3	p4	(AGCT)3	12	772	783	AAGACGCACTCACATCACGT	59.968	AAGATCACCGTCGAGGAGGA	60.034	246
TRINITY_Contig11487	1	p3	(AAG)6	18	65	82	GGGGAGAGGTGGTAGGTGAT	60.031	ATGGTCACCTGCTACAACGG	60.036	145
TRINITY_Contig11487	2	c	(CAGC)3tagcttggagctcggagccttgtaggattaagtcgatagtagcatgggggagaggtggtaggtgatcaccggc(GCCG)3	97	220	316	GGGGAGAGGTGGTAGGTGAT	60.031	ATGGTCACCTGCTACAACGG	60.036	145
TRINITY_Contig11487	3	p3	(GGA)6	18	837	854	GGGGAGAGGTGGTAGGTGAT	60.031	ATGGTCACCTGCTACAACGG	60.036	145
TRINITY_Contig11488	1	c	(GCC)5tcgccaccgatcccctctctctgctctggtg(GCT)5cctcgagctcgattggattcgtgcgtgttttcctg(TC)6ccccc(CCT)4	125	1458	1582	GCCGCTTGTTTTGTGCAAAC	59.974	TGTTAGGCCGCTTGAGGAAG	60.036	254
TRINITY_Contig11491	1	p5	(CCCGG)3	15	181	195	GTACCTAACTGAGAGCGGGC	59.897	GACCTGTACGGGTGCAAGAG	60.39	177
TRINITY_Contig11491	2	p3	(GAG)6	18	747	764	GTACCTAACTGAGAGCGGGC	59.897	GACCTGTACGGGTGCAAGAG	60.39	177
TRINITY_Contig11493	1	p3	(TCT)4	12	2590	2601	CGCTTTCTCCCACTTCCACT	59.965	TTGTTGGTGCTCCTGTGGTT	60.034	211
TRINITY_Contig11494	1	p3	(GCG)6	18	526	543	AAGCCAGCCAATTGTGCTTG	59.966	GCCCGATGTTGAGAAGTGGA	60.036	103
TRINITY_Contig11494	2	p3	(TTG)4	12	1037	1048	AAGCCAGCCAATTGTGCTTG	59.966	GCCCGATGTTGAGAAGTGGA	60.036	103
TRINITY_Contig11498	1	p6	(GCCGCG)3	18	695	712	TCCGGGATCACCTCAGGAAT	60.031	CCTACAGGTCACCCTCCTCA	59.959	101
TRINITY_Contig11498	2	p3	(TGG)4	12	849	860	TCCGGGATCACCTCAGGAAT	60.031	CCTACAGGTCACCCTCCTCA	59.959	101
TRINITY_Contig11499	1	p3	(TCT)4	12	783	794	ACCGCCTCTGCTTCCTTAAC	60.036	CATGAGGAAGCTGGCCAAGA	60.035	120
TRINITY_Contig11503	1	p4	(ATGG)3	12	304	315	CCTCGATTCGACGGACAGAG	59.972	GTGTTCGAGTGTGCTTGCTG	60.042	203
TRINITY_Contig11503	2	p3	(CCG)4	12	512	523	CCTCGATTCGACGGACAGAG	59.972	GTGTTCGAGTGTGCTTGCTG	60.042	203
TRINITY_Contig11503	3	p4	(GAGC)3	12	694	705	CCTCGATTCGACGGACAGAG	59.972	GTGTTCGAGTGTGCTTGCTG	60.042	203
TRINITY_Contig11505	1	p3	(CGG)6	18	701	718	ACATCTCACGCCCCCATTTT	59.96	CGCTTCCATCTCCTCGATCC	60.039	149
TRINITY_Contig11506	1	p5	(GCCGC)3	15	283	297	CCATGGCTAGGGTTTGGGTT	59.959	CCAAATTCCCCCGCCATTTC	59.822	105
TRINITY_Contig11506	2	p3	(CGG)5	15	720	734	CCATGGCTAGGGTTTGGGTT	59.959	CCAAATTCCCCCGCCATTTC	59.822	105
TRINITY_Contig11511	1	p3	(GCG)4	12	951	962	GTTTGGGGAAGGTGGAGGAG	59.962	GTCAGTCAGCTTGAGCAGGT	59.965	114
TRINITY_Contig11512	1	c	(CCT)4cccagccccg(CGC)5catggccaaggaggagggcaggaaggagaag(AAGAGC)3aaggcggcggccaaggaggca(GCC)5	122	88	209	GTGAAGCTGGAGGACGTCAA	59.966	CGATCACTCGTCGTCCGAAT	59.972	234
TRINITY_Contig11512	2	p3	(AGA)4	12	577	588	GTGAAGCTGGAGGACGTCAA	59.966	CGATCACTCGTCGTCCGAAT	59.972	234
TRINITY_Contig11512	3	p3	(AAG)4	12	870	881	GTGAAGCTGGAGGACGTCAA	59.966	CGATCACTCGTCGTCCGAAT	59.972	234
TRINITY_Contig11515	1	p3	(GCG)4	12	344	355	CCGTGTCAACAGTCTCTGCT	59.967	CCAGCGTAGACCGTCTTCAG	60.179	226
TRINITY_Contig11516	1	c	(GGC)4gcagggtacacggacccccccgcct(CCACCG)4ctaccaccgccgcctcccgcgggaggctggtagtagggaatgtaggcccc(CGG)4gtagctccacgacggcgacgacggggccggcggcgggtacccgtacgaggacggggagga(CGG)4	194	294	487	CGGCGGATTCCTGTAGTACC	59.968	TTCCCTACTACCAGCCTCCC	60.031	146
TRINITY_Contig11516	2	p3	(GAG)5	15	590	604	CGGCGGATTCCTGTAGTACC	59.968	TTCCCTACTACCAGCCTCCC	60.031	146
TRINITY_Contig11516	3	p2	(CG)6	12	770	781	CGGCGGATTCCTGTAGTACC	59.968	TTCCCTACTACCAGCCTCCC	60.031	146
TRINITY_Contig11518	1	p3	(CGC)4	12	230	241	TTGCAGAGCATGACGAGGAG	60.108	CGTGAACCTCGACCTCAACA	59.968	168
TRINITY_Contig11520	1	p4	(CGGC)3	12	366	377	ATCCATTCGTTCCGCACTGT	60.036	AGGGGTCGATGAGGGAATCA	60.031	222
TRINITY_Contig11522	1	p4	(TTGT)3	12	367	378	AACGGGAGACATGAGAGGGA	59.959	GAGATCCCGCAGATTCGAGG	60.039	137
TRINITY_Contig11522	2	c	(TC)6agctgcttctctgattccgactcttctcggttctcaaaactccaagatc(CATG)3	73	539	611	AACGGGAGACATGAGAGGGA	59.959	GAGATCCCGCAGATTCGAGG	60.039	137
TRINITY_Contig11526	1	p3	(GCG)4	12	139	150	CTTCAAGTCCAACGTCGGGA	59.967	ACGCTCTCAGTCTCACTTGC	60.039	172
TRINITY_Contig11526	2	p3	(GAG)4	12	619	630	CTTCAAGTCCAACGTCGGGA	59.967	ACGCTCTCAGTCTCACTTGC	60.039	172
TRINITY_Contig11527	1	p4	(AGGA)3	12	89	100	TTCTCCCGGACCTCCATCTT	59.957	CGAGCGAGAGAAGGTAGCTG	59.971	241
TRINITY_Contig11527	2	c	(CGA)4cgggtgcttcacccgcagcaccatctgcatcggctgctgctccacgccgccgccctgcatatcgtatcg(TCC)5tggcgcaccaagacgcagctaccttct(CTCG)3	135	674	808	TTCTCCCGGACCTCCATCTT	59.957	CGAGCGAGAGAAGGTAGCTG	59.971	241
TRINITY_Contig11529	1	p3	(CGC)4	12	168	179	TGCGTGCTGGATTTGTACCT	59.964	CCCGACGCTAATCAGTCCTC	59.969	158
TRINITY_Contig11531	1	p3	(TCC)5	15	120	134	GAAGTGTCCAGGACCATGGG	60.035	CAGCCGATCGCACAAAGATG	59.973	213
TRINITY_Contig11531	2	p3	(CGC)4	12	576	587	GAAGTGTCCAGGACCATGGG	60.035	CAGCCGATCGCACAAAGATG	59.973	213
TRINITY_Contig11531	3	p4	(GGAG)3	12	1090	1101	GAAGTGTCCAGGACCATGGG	60.035	CAGCCGATCGCACAAAGATG	59.973	213
TRINITY_Contig11532	1	p3	(CTC)4	12	409	420	CGCTTTCTCCGATCTCCTCC	59.969	GCACAGCAAGGAAGCGAAAA	59.969	238
TRINITY_Contig11532	2	p4	(TTTG)3	12	1761	1772	CGCTTTCTCCGATCTCCTCC	59.969	GCACAGCAAGGAAGCGAAAA	59.969	238
TRINITY_Contig11534	1	p4	(ATAG)3	12	80	91	GCTCGACCAAGTTGCCTACT	60.037	CAGCGGATCGACTCTACCAC	59.97	104
TRINITY_Contig11534	2	p5	(CTCTG)4	20	1530	1549	GCTCGACCAAGTTGCCTACT	60.037	CAGCGGATCGACTCTACCAC	59.97	104
TRINITY_Contig11538	1	p3	(CCG)4	12	801	812	CTTGGACAAGGTTTGCCAGC	59.967	CCAGCAAACACAACCGAGTG	59.971	156
TRINITY_Contig11538	2	p2	(AG)7	14	3534	3547	CTTGGACAAGGTTTGCCAGC	59.967	CCAGCAAACACAACCGAGTG	59.971	156
TRINITY_Contig11539	1	c	(GGA)4ggcgacgaaaccctagccgccgtgcgcgtagggttaatggcattggaggcggtg(GACGGC)3	84	683	766	AGTCTGGCTTGTGAACCTGG	59.891	TCTCCAGGTCCTCCAGCTC	59.999	194
TRINITY_Contig11543	1	p2	(GA)17	34	57	90	TCAAGAGGTTGCTCCGGAAC	59.965	AGTTGGTGTCGTCCTTGGTC	59.894	278
TRINITY_Contig11544	1	p3	(GCG)4	12	880	891	ACACTAACGCCAGGTCCTTG	59.965	CACGCTCATGCTCGCTTTAC	59.974	254
TRINITY_Contig11546	1	c	(CGG)4cgacaccaagccgctcatcctgctcgtcggctggctccccgccgccgtctccgtcgccttcctcggcaccatccggatcatacgcgcgccg(CGCC)3	115	707	821	CGCGCTGTTCAAGTTCAACA	59.973	CGGAGATGATGGCCAGGATC	60.037	277
TRINITY_Contig11547	1	c	(ACG)5cgccgccctt(GCC)6cgccgccctctccttgcacaggccgcgcttg(TCC)7	95	512	606	AATTCACAAGTTGGCAGCGC	60.319	GACACATCCTGAGGACGCAA	60.037	104
TRINITY_Contig11550	1	p6	(CGGCAG)3	18	146	163	TTCTACATGGTGTCCGCCAC	60.037	GCACTGACGCAAACACACAT	59.971	243
TRINITY_Contig11550	2	p3	(CGC)4	12	418	429	TTCTACATGGTGTCCGCCAC	60.037	GCACTGACGCAAACACACAT	59.971	243
TRINITY_Contig11551	1	p3	(CGC)4	12	255	266	AGCACCAAAGCATCTCCCTC	60.035	CATTGGTGGTGTGTGCAGTG	59.969	163
TRINITY_Contig11556	1	c	(CAG)4cc(GCA)5	29	10	38	AAGAAGCCACTGTACACGCA	59.894	TACCTGATGTGGAGGTCGGT	59.959	158
TRINITY_Contig11557	1	p2	(GA)9	18	1537	1554	ACGTCCCTCTTAGGCGTAGT	60.035	ATCGTCAAGGGCTCGTTTGT	59.965	210
TRINITY_Contig11559	1	p3	(GGA)4	12	399	410	GGAGGACAAGCTGGAGGTTC	60.036	ATTTTGGCTGTTCCCGTTGC	59.967	156
TRINITY_Contig11561	1	c	(CGG)4caggccatcagacgacggtcgtcgagaggccgcggccgccggaggactgc(CGG)4	74	89	162	GCGCATGTTCGAGAAGGTTG	60.179	TCCACCCTTTGGTCATCACG	59.964	224
TRINITY_Contig11561	2	c	(CAG)4gaggaggc(CTT)4cccacaggcggcggcattgcaggtgcagcagcgct(TGG)4cgccgccgcgcgtcgtgatctccatga(AAG)6	124	552	675	GCGCATGTTCGAGAAGGTTG	60.179	TCCACCCTTTGGTCATCACG	59.964	224
TRINITY_Contig11563	1	p4	(ATCA)3	12	274	285	CGGTCAACTGGCCCATAACT	60.035	GTTTCCTTCGGCACTGAGGA	59.965	132
TRINITY_Contig11564	1	p4	(AACG)3	12	24	35	TGGGGAAGAGGGTCAGAGAG	59.958	AACAGAGGCACACCACCAAA	60.034	129
TRINITY_Contig11564	2	p3	(TCG)4	12	390	401	TGGGGAAGAGGGTCAGAGAG	59.958	AACAGAGGCACACCACCAAA	60.034	129
TRINITY_Contig11567	1	p5	(TTTGG)3	15	612	626	TCCGCGTTGGTCACATGTAA	59.966	CCTTGCTCTTCCTTCCCTGG	60.035	273
TRINITY_Contig11568	1	p3	(TCG)4	12	518	529	TAGTGCAGGGACCAGCAATG	60.035	GGCAGCAGAGGGATGAAAGT	60.035	125
TRINITY_Contig11571	1	p6	(GCACGG)4	24	371	394	TCTACCGGAACAACAAGGGC	59.964	GCCAGAGATCTTCTCCAGCC	59.893	206
TRINITY_Contig11571	2	c	(GCC)4gacgccgaggtggaggacgccaagaaggtggccgccgcggtggagctggccgacgccacgaccaacaaggtcgccagccaggtgtaaggctgctgggc(GGT)4	122	759	880	TCTACCGGAACAACAAGGGC	59.964	GCCAGAGATCTTCTCCAGCC	59.893	206
TRINITY_Contig11573	1	p3	(CGG)4	12	805	816	TGGAAAGGAAATCGCACGGA	59.964	GCGTGTAAATAGCAGGCAGC	59.972	104
TRINITY_Contig11576	1	p3	(GTC)4	12	506	517	CCTCGACGCTAAGTTCACGA	59.833	TCCTCGAGGAGAAGGTCGTT	59.962	240
TRINITY_Contig11578	1	p3	(CAC)5	15	1476	1490	AGGAGGGACATGGAGATGCT	60.03	ACGCAATGAGAAGGGCAAGA	59.963	131
TRINITY_Contig11579	1	p6	(GTGGGA)3	18	235	252	GCCGTGAGCCCTAACAAGAA	60.321	CCGCCGTGACTAATCCCTAG	59.686	156
TRINITY_Contig11579	2	p6	(CGTCGC)4	24	777	800	GCCGTGAGCCCTAACAAGAA	60.321	CCGCCGTGACTAATCCCTAG	59.686	156
TRINITY_Contig11580	1	p2	(CT)7	14	34	47	TTGGCCGTGATCTCCTTGAC	60.036	CAGCAGCGACATTTACGACG	59.976	168
TRINITY_Contig11581	1	p3	(GCC)4	12	86	97	TGGTTCAATCGGTGAGAGGC	60.036	GTGGAACAACACGTGTGCTC	59.972	154
TRINITY_Contig11587	1	p3	(AGC)4	12	742	753	GTTCTCCCTGACCACCTTGG	59.963	GTGGTGGACGACGACTTCTT	59.968	276
TRINITY_Contig11588	1	p4	(CATG)3	12	191	202	CTGGACTCCGCGATTCTCTC	59.97	CTCTTCCTCAACCTGCCCAG	60.036	185
TRINITY_Contig11589	1	p3	(CGC)4	12	167	178	CCCTGAGAAATCTGCCGGAG	60.179	GCGCCCAGGTCTGAAATTTC	59.829	250
TRINITY_Contig11590	1	p3	(GGC)4	12	567	578	CAAAGGGATCAGCACGGACT	60.036	GCTTCATGACTGCCCTCTGT	60.036	164
TRINITY_Contig11593	1	p3	(CCG)4	12	62	73	TTTCCCTCCTTATCGCTCGC	59.896	GCACGACAAAGCAAAGCAGA	59.971	224
TRINITY_Contig11593	2	p3	(CGC)4	12	492	503	TTTCCCTCCTTATCGCTCGC	59.896	GCACGACAAAGCAAAGCAGA	59.971	224
TRINITY_Contig11595	1	p3	(CCG)4	12	243	254	GTAATGGGTGGCAGTGTGGA	59.962	AGTCTACTACTCCCTCCGGC	59.817	107
TRINITY_Contig11595	2	p3	(GCG)7	21	413	433	GTAATGGGTGGCAGTGTGGA	59.962	AGTCTACTACTCCCTCCGGC	59.817	107
TRINITY_Contig11596	1	p3	(CCG)4	12	66	77	CTCCACCACCAACCAGAGTC	59.965	CGTGTGGAAGGAGAGCTCTG	60.109	127
TRINITY_Contig11601	1	p3	(GGA)4	12	445	456	GAACCTCAGACGGCTGTTCA	59.966	CACTTCCAGAAGCTCGAGCA	60.038	194

TRINITY_Contig11603	1	p4	(GGCC)3	12	587	598	AGGGATAGTGAGGGAACGCT	60.032	CCTTCCCCTCTTTCGTTCCC	60.035	116
TRINITY_Contig11605	1	p3	(TTA)4	12	22	33	AGGCACACAGACACAACACA	60.036	AAGATCAAGTCCAGGTGCGG	60.036	148
TRINITY_Contig11605	2	c	(GCGTCG)4cccgt(GAC)4	41	790	830	AGGCACACAGACACAACACA	60.036	AAGATCAAGTCCAGGTGCGG	60.036	148
TRINITY_Contig11606	1	p6	(CAGGAG)3	18	275	292	GACAACGGCATCAAGATCGC	59.973	GCATCGAGCAGGTACTCTCC	59.969	218
TRINITY_Contig11607	1	p4	(CACC)3	12	45	56	CGATCGAAGAGGAACGGGAG	59.97	CGAATTCCTTGAACAGCGGC	60.179	100
TRINITY_Contig11608	1	c*	(GGCG)2(GGC<G>GCACG)(GGCACG)2	29	280	308	CAAAGGCCTGAGAGGGATCG	60.179	CTCTTCCCCATGGTGGCATT	60.033	163
TRINITY_Contig11608	2	p5	(GCGCG)3	15	502	516	CAAAGGCCTGAGAGGGATCG	60.179	CTCTTCCCCATGGTGGCATT	60.033	163
TRINITY_Contig11608	3	p6	(GCGCGG)5	30	761	790	CAAAGGCCTGAGAGGGATCG	60.179	CTCTTCCCCATGGTGGCATT	60.033	163
TRINITY_Contig11609	1	p4	(GAGG)3	12	1920	1931	GAATTTGCCCTGCTCCCTCT	60.034	TATTCGCTCTCCACTGACGC	59.899	193
TRINITY_Contig11611	1	p4	(CTTC)3	12	719	730	ACTTGCCCGTCTTGAGGAAG	59.965	AGGAAGCTAGGGACGAGAGG	60.106	141
TRINITY_Contig11612	1	c*	(CGTCGA)3cgagcccctgcttc(CCG)5(GAGCGG)3*	64	1625	1688	TCCACCAGATATGCCCCTGA	60.03	CGAGAGAAGGCGGCCAATAT	59.966	177
TRINITY_Contig11613	1	c	(GAGG)3cggagcggatgt(CGG)4	36	49	84	GGAAGATGATCCAGCGAGGG	59.965	TTTGACGTCGGTAGGTTCGG	60.039	265
TRINITY_Contig11614	1	p3	(CCG)4	12	64	75	GACTTCCCCTACTCCTCCGT	60.033	GAGAAGAAGACCCTCGCTCG	59.9	223
TRINITY_Contig11617	1	p4	(AGCT)3	12	17	28	TCAAGATCGATCGAGCTGCC	59.969	AAGCTCGTCACTCTCATGGC	60.108	162
TRINITY_Contig11617	2	p3	(CGC)4	12	301	312	TCAAGATCGATCGAGCTGCC	59.969	AAGCTCGTCACTCTCATGGC	60.108	162
TRINITY_Contig11617	3	p5	(GATCA)3	15	582	596	TCAAGATCGATCGAGCTGCC	59.969	AAGCTCGTCACTCTCATGGC	60.108	162
TRINITY_Contig11620	1	p4	(CAAC)3	12	137	148	AAGCAACCAACCAACCCAGA	60.033	CCTCTGAGCCGGAGTTGTTT	59.965	118
TRINITY_Contig11620	2	c	(GCGCCG)3gcgagccccagctgccgctcgctcacgacgacgctgccctccgacttgctgcgcacgtgccgggtcggcgt(GCC)5	104	372	475	AAGCAACCAACCAACCCAGA	60.033	CCTCTGAGCCGGAGTTGTTT	59.965	118
TRINITY_Contig11621	1	c	(GGC)5gcgggcgggggagcgttgttgacggagcgcggggaggtggaggacgacgcggcggaggcggccgaggccgggagcagcagcaggcgcggggtcgg(GGC)4	122	404	525	TAATCCCCAAGCTCCGCATC	59.891	CGCCAATAAGGAGCTAGGGG	59.965	171
TRINITY_Contig11621	2	p4	(GGGC)3	12	725	736	TAATCCCCAAGCTCCGCATC	59.891	CGCCAATAAGGAGCTAGGGG	59.965	171
TRINITY_Contig11624	1	p3	(GCC)4	12	599	610	GACCCTCCACCTGCTTTCTC	60.036	GGATGACATCTCCCAGCTCG	59.967	159
TRINITY_Contig11624	2	c	(CGG)4ctcggccggcggccaggacgcgcgccttgtcgtccaagaccaattcaacggca(GCG)4	77	1678	1754	GACCCTCCACCTGCTTTCTC	60.036	GGATGACATCTCCCAGCTCG	59.967	159
TRINITY_Contig11624	3	p3	(GGC)5	15	2051	2065	GACCCTCCACCTGCTTTCTC	60.036	GGATGACATCTCCCAGCTCG	59.967	159
TRINITY_Contig11625	1	p3	(GCG)6	18	796	813	TCTGGAAGTAGGCGACGAGA	60.036	CCGGTTCAAGGACATCGACA	60.038	110
TRINITY_Contig11627	1	p3	(CGC)5	15	592	606	ACAACGACCTCGCTCCATTT	59.965	GTGGAGACGGGTTCCCTTTT	59.89	142
TRINITY_Contig11630	1	p2	(TC)6	12	73	84	TCGCACGACATCATCTACCG	59.971	GACCCGTGAGCATCTCGTAG	59.97	247
TRINITY_Contig11630	2	p3	(CGC)5	15	319	333	TCGCACGACATCATCTACCG	59.971	GACCCGTGAGCATCTCGTAG	59.97	247
TRINITY_Contig11631	1	c	(CGCT)3tcctcccccgcccgccgcgagggtttgggtgg(GGA)4gacgagatcgaggacggcagggggt(GGAG)3	93	2571	2663	TTCCACGCGCTAGAGCTATG	59.969	TGTATGTATGCGGCGATGCT	59.967	193
TRINITY_Contig11633	1	p5	(GTGCC)3	15	228	242	TGCAAGCCCCTACTTTCCTG	59.962	ATGGCTACCGCACAGACAAA	59.964	116
TRINITY_Contig11636	1	p3	(TCG)4	12	496	507	TACGGAGAGGTTTCCCGTCT	59.961	CCACTCGGAACCTCATGCTT	60.036	159
TRINITY_Contig11637	1	p2	(GA)6	12	69	80	TGTATCACTGGGGCGGACTA	60.033	GTTGGCAAGCAAGGTGTCTG	59.969	101
TRINITY_Contig11637	2	p6	(GTCAGA)3	18	541	558	TGTATCACTGGGGCGGACTA	60.033	GTTGGCAAGCAAGGTGTCTG	59.969	101
TRINITY_Contig11637	3	p3	(CTA)4	12	666	677	TGTATCACTGGGGCGGACTA	60.033	GTTGGCAAGCAAGGTGTCTG	59.969	101
TRINITY_Contig11642	1	c	(AGCC)4agcgagagcgagctaccggtggacgcagacgacac(GCTA)3	63	123	185	CGATCTCACTAGCCAGCCAG	59.968	CGTTGATCTCGTCCTCGGAG	59.972	231
TRINITY_Contig11643	1	p2	(CT)12	24	94	117	AGTTTGGAGTTCCTCTGGCG	59.965	TCGTAGAGCTTGTCATAGGCG	59.969	112
TRINITY_Contig11645	1	p3	(CGC)5	15	11	25	ACCCTTTGCTGGCTCATCTC	60.035	GCAGGGAAACTCCAGAGACC	60.036	275
TRINITY_Contig11651	1	p4	(CCTC)3	12	735	746	TGCGAACCTGCGACATGTAT	60.108	ATCGGAGCTTTTACGCTGCT	60.108	125
TRINITY_Contig11652	1	p3	(CGT)5	15	251	265	CTGGCTCCATCCTCTCTCCT	60.106	GCCACGGTCATAGTCATCGT	59.898	137
TRINITY_Contig11652	2	p4	(GCTT)3	12	727	738	CTGGCTCCATCCTCTCTCCT	60.106	GCCACGGTCATAGTCATCGT	59.898	137
TRINITY_Contig11654	1	c	(AGC)4catggggatagccgtggtggctcccgtcgcggcgg(CCT)4	59	84	142	GCTCCTGGAGTTAGCAGCAA	60.036	GGAGCGCTCATGATAAGCCT	59.965	156
TRINITY_Contig11660	1	p3	(CTC)4	12	558	569	CCTCGTTGAAGTCCTCCCAC	60.037	TTCCATCAGCGTTCCCTCAC	60.036	135
TRINITY_Contig11664	1	p3	(AGC)4	12	3896	3907	GCGCAGATGCAGTTAGAAGC	59.973	CGAGTCTCGATCAGGTCAGC	59.971	188
TRINITY_Contig11665	1	p3	(GCC)4	12	621	632	AAGCGAACGGAGTACTGGTG	60.039	CGGTACCAAGATCTTCGCGA	59.9	182
TRINITY_Contig11669	1	p3	(CGG)4	12	266	277	AAACCTACGCATGGGGGAAG	60.034	GACGGACAGCACCTTGAAGA	59.966	269
TRINITY_Contig11670	1	p3	(AGA)4	12	12	23	TTTTGCGTGGACATTGCTGG	59.968	CCCATGCGCTATGTTTGCTC	59.97	272
TRINITY_Contig11670	2	p3	(TCT)4	12	791	802	TTTTGCGTGGACATTGCTGG	59.968	CCCATGCGCTATGTTTGCTC	59.97	272
TRINITY_Contig11671	1	p3	(TGC)5	15	411	425	GAAGCAACCTGTCTACCCCC	60.035	CTTGGGGTTCTTGCTAGGGG	60.034	113
TRINITY_Contig11673	1	p3	(CGG)4	12	458	469	TGGTGTTCTGGTCGCTGTAC	59.967	TACCTCTCCCTCACCCTCAC	59.665	279
TRINITY_Contig11675	1	p2	(GA)26	52	20	71	TTTTCCCAGATCCGCATCCC	60.107	GAGTAATCCCCGCTCTGCTC	59.967	224
TRINITY_Contig11675	2	p5	(CAGAG)3	15	494	508	TTTTCCCAGATCCGCATCCC	60.107	GAGTAATCCCCGCTCTGCTC	59.967	224
TRINITY_Contig11675	3	p3	(TCC)4	12	686	697	TTTTCCCAGATCCGCATCCC	60.107	GAGTAATCCCCGCTCTGCTC	59.967	224
TRINITY_Contig11675	4	p3	(GCC)5	15	1868	1882	TTTTCCCAGATCCGCATCCC	60.107	GAGTAATCCCCGCTCTGCTC	59.967	224
TRINITY_Contig11676	1	p4	(TTTA)3	12	765	776	AGAAGATGCCCCAGATGCTG	59.817	TAGGAAGAGCAGCACCATGC	60.108	118
TRINITY_Contig11678	1	p2	(GC)7	14	240	253	ATCACGATTTCTCCGTCCCG	59.898	GACCTACTGACTGGTGGTGC	60.037	219
TRINITY_Contig11680	1	c	(CT)6cg(AT)6	26	2395	2420	GGCAGGACCTCAATCGTGAA	60.036	AGAGAGAGAGCCAGCATCCA	60.032	168
TRINITY_Contig11683	1	p5	(CCTCC)3	15	720	734	GCAGCAAGTTCAAGCAACGA	59.971	AGGGGAGGAGAGCAGAAGAG	60.032	161
TRINITY_Contig11683	2	p3	(GAA)4	12	1049	1060	GCAGCAAGTTCAAGCAACGA	59.971	AGGGGAGGAGAGCAGAAGAG	60.032	161
TRINITY_Contig11683	3	p3	(GCG)5	15	1318	1332	GCAGCAAGTTCAAGCAACGA	59.971	AGGGGAGGAGAGCAGAAGAG	60.032	161
TRINITY_Contig11686	1	p3	(GCC)4	12	1433	1444	CAAACGTTGCACACACGCTA	59.973	CTGTGGATAAGGCAGGGCAA	60.034	110
TRINITY_Contig11687	1	p6	(GGATTT)3	18	682	699	GCAGTGAGAGCGGACTTCTT	60.038	CTCCTTTCCCTCCGTCACAC	60.037	125
TRINITY_Contig11690	1	p3	(GCC)4	12	324	335	CGCAGAGCTGTATGGTTTGC	59.902	GGAGAAACCTTCCTGGCCTC	60.035	141
TRINITY_Contig11690	2	p3	(GCC)5	15	504	518	CGCAGAGCTGTATGGTTTGC	59.902	GGAGAAACCTTCCTGGCCTC	60.035	141
TRINITY_Contig11691	1	p5	(TCTCC)4	20	1330	1349	CCGTAGCAGATGAGCACGAT	59.969	AAGGTGAAGACGACGAACCC	59.967	157
TRINITY_Contig11692	1	p3	(CTA)5	15	76	90	TGTCCTTAAAGACGTGCGCT	59.967	TCGTGTTATGCTGAACGGCT	60.038	108
TRINITY_Contig11692	2	p4	(AGGA)3	12	787	798	TGTCCTTAAAGACGTGCGCT	59.967	TCGTGTTATGCTGAACGGCT	60.038	108
TRINITY_Contig11693	1	p3	(CGC)5	15	486	500	ACTTGGAGATGGTGGGCATG	60.034	ACGCGCTGGTTGAAATGTTC	60.04	104
TRINITY_Contig11695	1	p3	(CCT)7	21	241	261	AGCTTGTGTGCGAACGAGTA	59.968	CACGACCGCGACATCAATTC	59.975	134
TRINITY_Contig11695	2	p3	(CCA)4	12	1918	1929	AGCTTGTGTGCGAACGAGTA	59.968	CACGACCGCGACATCAATTC	59.975	134
TRINITY_Contig11698	1	p3	(CGT)4	12	373	384	CGGATAGGAGCAGAGCACAG	59.968	TACGGCAATGAGGAGGAGGA	60.033	171
TRINITY_Contig11701	1	p4	(CTCC)3	12	43	54	GGGCTTCGTCCTCTACATCG	59.969	GGAGTGGAACGATGGTGTGT	59.965	136
TRINITY_Contig11702	1	p3	(AGA)5	15	694	708	CCCAACGCAAACCCAAAGAG	59.968	TCTCTCTCCCTGACGTCCTG	60.035	275
TRINITY_Contig11702	2	p2	(GA)8	16	837	852	CCCAACGCAAACCCAAAGAG	59.968	TCTCTCTCCCTGACGTCCTG	60.035	275
TRINITY_Contig11707	1	p3	(GCA)5	15	247	261	CCAGGCTGTCCGTCAAGAAT	60.036	GGCCATGGAGGGTAAACGAA	60.035	210
TRINITY_Contig11707	2	c	(TCCC)3cggtgtctctcttcctctcttcctccactacctcctctgttcctctgtcttcccttcttctccacttcgccggcacaagcagcagcacaggtgca(CGG)4accttcgcaacaggggcgga(CGG)4	151	1495	1645	CCAGGCTGTCCGTCAAGAAT	60.036	GGCCATGGAGGGTAAACGAA	60.035	210
TRINITY_Contig11707	3	p3	(CGC)4	12	3705	3716	CCAGGCTGTCCGTCAAGAAT	60.036	GGCCATGGAGGGTAAACGAA	60.035	210
TRINITY_Contig11709	1	p3	(CAT)4	12	540	551	AAAAGCGCGAAAACAGACCC	59.97	GGGGGAGTCAGGCTTGAATC	60.107	160
TRINITY_Contig11713	1	c	(GCG)5(GAATC)3gcccggcgagcgagcccccttgagtaggagtagatctcggggaggcgggcggggcgctcagta(CGG)4	105	254	358	TCCCAGGATCCCTTTCCCTT	59.877	TCACCAGCAACATCCACCTC	59.963	256
TRINITY_Contig11713	2	p5	(GGTCG)3	15	685	699	TCCCAGGATCCCTTTCCCTT	59.877	TCACCAGCAACATCCACCTC	59.963	256
TRINITY_Contig11716	1	p3	(CTG)4	12	2662	2673	TCAACACCACCAAACAGCCT	60.034	CGACGAGGAGGTGATGATGG	59.969	253
TRINITY_Contig11716	2	p3	(TCT)6	18	3558	3575	TCAACACCACCAAACAGCCT	60.034	CGACGAGGAGGTGATGATGG	59.969	253
TRINITY_Contig11720	1	p6	(GCACGA)3	18	297	314	CAGCGACGAGGGTGTTAGTT	60.039	GGGGAAAGACGTACGTGTGT	59.967	171
TRINITY_Contig11720	2	p4	(GCCG)3	12	436	447	CAGCGACGAGGGTGTTAGTT	60.039	GGGGAAAGACGTACGTGTGT	59.967	171
TRINITY_Contig11721	1	p3	(CCG)5	15	317	331	TGGAATTGGACTTTGGCGGA	59.889	CACGGGACAGCACAAAACAG	59.971	207
TRINITY_Contig11721	2	p4	(GGTG)3	12	517	528	TGGAATTGGACTTTGGCGGA	59.889	CACGGGACAGCACAAAACAG	59.971	207
TRINITY_Contig11722	1	c	(GAG)4aagaagaaaccaagaaaggagcgaggatgtacaacg(CGC)4	60	249	308	AGATCGCCTCCGTCTCTCTT	60.107	GCAGTGGGTTCAGAATCGGA	60.036	197
TRINITY_Contig11722	2	p3	(CTG)4	12	436	447	AGATCGCCTCCGTCTCTCTT	60.107	GCAGTGGGTTCAGAATCGGA	60.036	197
TRINITY_Contig11727	1	p6	(CCTTCC)4	24	1419	1442	TCGTGGTCCTCGTAGCAGTA	60.036	TGGAGGAGGTGGAGATGGAG	60.032	118
TRINITY_Contig11728	1	p5	(GGGAG)3	15	755	769	CGTCTGGGAGCCTCAAACTT	59.965	GCGGTCGAGTACTAGCAGTC	59.972	122
TRINITY_Contig11733	1	p3	(GAG)6	18	73	90	CACTTCACTGACAGCGAGGT	59.967	TCGACCAGCTGATCGTTAGC	59.899	201
TRINITY_Contig11734	1	p3	(CTG)5	15	455	469	CACAAGACACACAACAGCGG	59.972	ACTCCTGTGGCAAGAAGCTC	59.964	166
TRINITY_Contig11736	1	p3	(CTT)4	12	61	72	CGAGCACCTCAAGAACACCT	59.966	TCTGAGCACATCGTCGATCG	59.972	152
TRINITY_Contig11737	1	c	(TCGG)3tggctcatcggcagcctgaactcctcgagatcgtcctcgtcatccagatccgcttggtgccgc(CCG)7	96	1509	1604	TCGTCACTGTCATTGCCGAA	59.967	CAAAAAGCATTGCAGGCCCT	59.964	154

TRINITY_Contig11737	2	c*	(CGGC)2(<CGGC>GGCGGCGGAGCGCCGACGAAGAGGTAGCGACGAGAGGGGGTGGGTAGGCCGGTAGGGGACGGGGAGGAGATTGCGGCGGCTCCAGGGT)(CGG)3	20	1775	1794	TCGTCACTGTCATTGCCGAA	59.967	CAAAAAGCATTGCAGGCCCT	59.964	154
TRINITY_Contig11738	1	p3	(GCT)4	12	199	210	GCAAGGTTGCGGAAAGACAT	59.402	CATCAGCAGGATGCCATTGC	59.968	100
TRINITY_Contig11738	2	p3	(GTC)4	12	422	433	GCAAGGTTGCGGAAAGACAT	59.402	CATCAGCAGGATGCCATTGC	59.968	100
TRINITY_Contig11738	3	p4	(ACGC)4	16	686	701	GCAAGGTTGCGGAAAGACAT	59.402	CATCAGCAGGATGCCATTGC	59.968	100
TRINITY_Contig11740	1	p3	(AGC)4	12	112	123	CGATCCATCACTCACTCCCG	59.969	TCAGAGACCAACACGCTGAC	59.968	212
TRINITY_Contig11741	1	p4	(GGCA)3	12	1527	1538	TCTTGGCATTGTCGCATCCT	60.035	CACCCTTTGCCATGTGGTTG	59.966	127
TRINITY_Contig11741	2	p4	(CTCC)3	12	1736	1747	TCTTGGCATTGTCGCATCCT	60.035	CACCCTTTGCCATGTGGTTG	59.966	127
TRINITY_Contig11741	3	p3	(GAG)4	12	2026	2037	TCTTGGCATTGTCGCATCCT	60.035	CACCCTTTGCCATGTGGTTG	59.966	127
TRINITY_Contig11742	1	p3	(CGC)4	12	343	354	CCTCACATTTCCACCGTCCA	59.964	CCATGAGCCACACCAAAACG	60.039	271
TRINITY_Contig11744	1	p3	(GAA)5	15	23	37	GGACACCCCGTCTCATCTTC	59.824	CGACAACCTGAACCGAGGAA	59.967	113
TRINITY_Contig11745	1	p4	(CGCC)3	12	571	582	CGAACCCATCAGCCCTTCAT	60.107	CCTACTACAACTGCCAGCCC	60.108	147
TRINITY_Contig11745	2	p3	(TGC)6	18	722	739	CGAACCCATCAGCCCTTCAT	60.107	CCTACTACAACTGCCAGCCC	60.108	147
TRINITY_Contig11746	1	p3	(CTC)5	15	146	160	CAGAGCAGGTCCAAGCAAGA	59.964	AACCCCTCCAGCATCTCGTA	60.325	165
TRINITY_Contig11747	1	p3	(CGC)4	12	1206	1217	CTCTCAGCAGGCTCAACACA	59.966	CTCCGCTTTCAGGGACAACT	59.965	263
TRINITY_Contig11749	1	c	(CGC)5cgtggtcaaggacggcaaccccaggaagaaggacgcgaacgctaaaaagcagccgccggcgaaggccggggccagggccaagtcgaaggccatac(GCGA)3cggaaacggaagagggcagcgtggacttcgaccggccgtccaagttcctggtggtctgcctcaacgccatccgcgacgcggtggcgcccgaggacggcg(GCA)4	233	347	579	TGTGGGGCTGATGAATGTCC	60.034	GTTTCACGAGAGGGGCATCA	60.036	200
TRINITY_Contig11751	1	p3	(CGG)4	12	2281	2292	CAACCAACACAGAGCAAGCC	59.969	GTGGTCACGTACTCGGTGTT	59.969	145
TRINITY_Contig11756	1	p2	(TC)9	18	84	101	ACATCCCTCATCGTCTCCCA	60.032	CCCTCGAGCCTTCCTTTGTT	59.963	223
TRINITY_Contig11756	2	p3	(CGG)4	12	229	240	ACATCCCTCATCGTCTCCCA	60.032	CCCTCGAGCCTTCCTTTGTT	59.963	223
TRINITY_Contig11756	3	p3	(CCG)4	12	766	777	ACATCCCTCATCGTCTCCCA	60.032	CCCTCGAGCCTTCCTTTGTT	59.963	223
TRINITY_Contig11756	4	c	(TGA)4acttgagcttgagatgaggtgggccgggtggtgtttgctttttctgttggtgttagttaatttccttgtacaaaactgcacctaaccgt(GAA)4	113	1109	1221	ACATCCCTCATCGTCTCCCA	60.032	CCCTCGAGCCTTCCTTTGTT	59.963	223
TRINITY_Contig11759	1	p5	(AGATG)3	15	106	120	GCAGGTAGACGTCGATCTCG	60.042	CTCTCCATGAACGTCGTGCT	60.109	199
TRINITY_Contig11762	1	p6	(GAAGAG)4	24	3164	3187	TGGCCTGCCTCTGTTATTGG	60.034	CGCTTTGCAAGTCGATCTCG	59.976	101
TRINITY_Contig11764	1	p3	(CGG)4	12	659	670	ACTGGAGGGAGTAGTGTGCT	59.885	CGATGGAGGATGACAGGGTG	59.894	162
TRINITY_Contig11766	1	p3	(CCT)4	12	388	399	GTCTCCATCCTGTAGCTCGC	59.969	GGATGAGATCGACAGGGCTG	59.967	157
TRINITY_Contig11768	1	p3	(GGC)5	15	746	760	CCACATGCGCATATCTCCCT	59.964	TGGCGGCACCTACAATGATT	60.035	155
TRINITY_Contig11769	1	p2	(TC)17	34	63	96	GTACCACCACCACCAGTACG	60.038	ATCATGAGGTCCTGCTCCCT	60.03	213
TRINITY_Contig11769	2	p3	(GCC)4	12	248	259	GTACCACCACCACCAGTACG	60.038	ATCATGAGGTCCTGCTCCCT	60.03	213
TRINITY_Contig11769	3	p3	(ACC)4	12	636	647	GTACCACCACCACCAGTACG	60.038	ATCATGAGGTCCTGCTCCCT	60.03	213
TRINITY_Contig11769	4	p3	(CCA)4	12	769	780	GTACCACCACCACCAGTACG	60.038	ATCATGAGGTCCTGCTCCCT	60.03	213
TRINITY_Contig11769	5	p3	(GGC)4	12	1109	1120	GTACCACCACCACCAGTACG	60.038	ATCATGAGGTCCTGCTCCCT	60.03	213
TRINITY_Contig11771	1	p4	(GGAA)3	12	267	278	ACGTCCTCCTTTGATGCGAA	59.68	AAAGGCCCCCATTACACGAG	60.034	145
TRINITY_Contig11774	1	p3	(GGC)4	12	558	569	AGAGGACCATTTGAGCCAGC	60.035	TGGTTCACCACGAGGACATG	59.965	199
TRINITY_Contig11775	1	p6	(GCGACG)3	18	1432	1449	GTTCATGTTGTGCCGTTGCT	59.969	TGCAGTGGATGGACTATCGC	59.895	235
TRINITY_Contig11775	2	p3	(CGC)5	15	1885	1899	GTTCATGTTGTGCCGTTGCT	59.969	TGCAGTGGATGGACTATCGC	59.895	235
TRINITY_Contig11779	1	p3	(GAG)4	12	539	550	GGAGGAGGAGGGGAAGATGT	60.031	CAACCCCAACCACTACCCTC	59.962	179
TRINITY_Contig11780	1	p3	(CAG)4	12	1372	1383	GATACTAGGTGTGCCTCGCC	59.968	CCGGAAAAGAGCCCGTAGTT	60.037	150
TRINITY_Contig11782	1	p4	(GGAC)3	12	817	828	GTTGCGCACTACCTTTGCAA	59.97	GCGGCCTCTTTACTGGTTCT	60.036	175
TRINITY_Contig11782	2	c	(CTAT)3agctgcctat(GCAC)3	34	1014	1047	GTTGCGCACTACCTTTGCAA	59.97	GCGGCCTCTTTACTGGTTCT	60.036	175
TRINITY_Contig11783	1	p5	(AAGAA)3	15	1610	1624	CATGGTTGGGAAAACGCAGG	60.038	GCCATGCAGAATGAGATGCG	60.04	118
TRINITY_Contig11786	1	p3	(GGT)5	15	2812	2826	ACTGCTCATGTGGTGCCTAC	60.036	CACCACAGAACAAGGCAAGC	59.969	217
TRINITY_Contig11787	1	p4	(CCGG)3	12	88	99	GGACTTTAAAAAGGGCGCCC	59.754	TCGACATCAACCAGCAGAGC	60.39	100
TRINITY_Contig11787	2	p4	(GCGG)3	12	1060	1071	GGACTTTAAAAAGGGCGCCC	59.754	TCGACATCAACCAGCAGAGC	60.39	100
TRINITY_Contig11787	3	p3	(CGT)4	12	1368	1379	GGACTTTAAAAAGGGCGCCC	59.754	TCGACATCAACCAGCAGAGC	60.39	100
TRINITY_Contig11789	1	p3	(GCG)4	12	530	541	CTGATCCATGTCGCCGATCA	59.967	GAGCAAGGGTTCAAGGAGCT	59.963	139
TRINITY_Contig11792	1	p3	(GGC)6	18	679	696	ACCTGGAGTAAGTTGCCGTG	59.965	CATGGTGAGGATCTCCGCTC	59.967	144
TRINITY_Contig11795	1	p2	(GT)7	14	262	275	GGAGCTTCGAGATGAGGAGC	59.968	CGTACGGTCAGATCTTCCGG	59.971	228
TRINITY_Contig11796	1	p3	(GCG)5	15	103	117	AGGCTGAAGAGGAGGAGGAG	60.032	CGAGATGAACTCCTCCGCTC	59.97	177
TRINITY_Contig11796	2	c	(TGC)4ggcgtcgcggccgggagacggcgcgaagagggtaaagaggctgaa(GAG)4	69	330	398	AGGCTGAAGAGGAGGAGGAG	60.032	CGAGATGAACTCCTCCGCTC	59.97	177
TRINITY_Contig11797	1	p3	(GCC)4	12	620	631	TCTCTTGTGACCGAAGCACC	59.966	GATCTCGAGGGTTCCGGTTG	60.179	180
TRINITY_Contig11801	1	p4	(CAAT)3	12	273	284	TCGTGGCCCATTAACTGACC	60.036	TGCCGGTAGCTGGAAAGAAG	60.036	194
TRINITY_Contig11801	2	p3	(GGC)5	15	1555	1569	TCGTGGCCCATTAACTGACC	60.036	TGCCGGTAGCTGGAAAGAAG	60.036	194
TRINITY_Contig11803	1	p4	(CCCT)3	12	611	622	CGCTCTCGGCTTGATTTTCG	59.974	TGCCAAGGCTCACTGAGAAG	59.964	163
TRINITY_Contig11804	1	p3	(TCC)4	12	625	636	AGAAAGAGACGCCTTGCCAA	59.891	GTCTCGTTCGTGAAAGCAGC	59.838	123
TRINITY_Contig11805	1	c	(GGCC)3tgggcccattg(GGCC)3	35	472	506	TAACGACGTCCAAAGGGGTG	59.966	ATAGCCCTAGCCCTGGGAAA	60.029	200
TRINITY_Contig11810	1	p3	(TCA)4	12	95	106	GCCATCTCCTTCTTCCTCGG	59.894	GCAAGTCTCAGCAACACAGC	60.041	115
TRINITY_Contig11810	2	p3	(CCG)4	12	294	305	GCCATCTCCTTCTTCCTCGG	59.894	GCAAGTCTCAGCAACACAGC	60.041	115
TRINITY_Contig11810	3	p3	(CGC)4	12	460	471	GCCATCTCCTTCTTCCTCGG	59.894	GCAAGTCTCAGCAACACAGC	60.041	115
TRINITY_Contig11811	1	p4	(CGCA)3	12	71	82	GTCCGCCAGACAGTAGCAAA	60.32	GGCGGTGTTTCATTTCCTCG	59.832	170
TRINITY_Contig11812	1	c	(CCTCT)3cttgtggattccaccaacccgcccccctttttcttggctgcgtgcgtatcgccgtcctaggcattcgcccccctggcg(GCCT)3	104	155	258	CGCCTGATTATCGTGGGGAA	59.894	ATCCTCAAGGCCTCGACTCT	60.033	141
TRINITY_Contig11817	1	p3	(GAC)4	12	662	673	AGGTCATGGTTGGGGTTTGG	60.179	GTTGGTTGTGCTGCATCTGG	60.039	135
TRINITY_Contig11818	1	p2	(CG)6	12	610	621	GAGGTGGTGGAGGAGGAGAT	60.032	TTCCGAGTCCGATCCTTCCT	60.033	231
TRINITY_Contig11818	2	p3	(CGT)4	12	762	773	GAGGTGGTGGAGGAGGAGAT	60.032	TTCCGAGTCCGATCCTTCCT	60.033	231
TRINITY_Contig11818	3	p4	(GATC)3	12	1109	1120	GAGGTGGTGGAGGAGGAGAT	60.032	TTCCGAGTCCGATCCTTCCT	60.033	231
TRINITY_Contig11823	1	p4	(GTCC)3	12	124	135	TCTTCACCTTCTTCGTCGGC	60.039	CCCAAGTCCCCTGTTCGATC	60.108	270
TRINITY_Contig11825	1	p3	(TCC)4	12	28	39	GGATTTCCAGAAGCCCCGAA	60.034	CAGGGTAGAAGGACGGAGGA	60.033	131
TRINITY_Contig11825	2	p3	(CGC)4	12	576	587	GGATTTCCAGAAGCCCCGAA	60.034	CAGGGTAGAAGGACGGAGGA	60.033	131
TRINITY_Contig11826	1	p3	(CGC)4	12	1019	1030	GTTCTTGGTGCTGTTGCTGG	59.969	CGCCATTGTTGAGCGATGAG	59.973	183
TRINITY_Contig11828	1	p4	(GATG)3	12	1355	1366	TTCGAATCCAGTTGGCGTGA	59.966	TCACTGACGCAGACACTGAC	59.969	110
TRINITY_Contig11828	2	p2	(TA)7	14	1746	1759	TTCGAATCCAGTTGGCGTGA	59.966	TCACTGACGCAGACACTGAC	59.969	110
TRINITY_Contig11828	3	p2	(GT)11	22	2232	2253	TTCGAATCCAGTTGGCGTGA	59.966	TCACTGACGCAGACACTGAC	59.969	110
TRINITY_Contig11829	1	p3	(TGC)7	21	2133	2153	TACAGGAGTATGGCTGGCCT	60.031	CCTACTTGCACCTGGAAGCA	59.963	126
TRINITY_Contig11829	2	p6	(ACCTCT)3	18	2306	2323	TACAGGAGTATGGCTGGCCT	60.031	CCTACTTGCACCTGGAAGCA	59.963	126
TRINITY_Contig11830	1	p3	(ACG)4	12	173	184	TGCTCTTCTGGTACTTGCGG	60.037	CCCCCTCAAATCCTCCAACC	60.033	239
TRINITY_Contig11831	1	p3	(CGC)4	12	214	225	AGCAGAAATCGTGTGGTCGA	59.683	CAGACACCACACGTACGTCA	59.97	179
TRINITY_Contig11833	1	c	(CGG)4ccacttctccgccgggttcttggtgggcgggctcgcgccggccctcgtccacgcgcctggtggtggtgctggccccgccgcggctcttcca(CCG)4	115	249	363	GACCAATGACCACTGCTCGA	60.037	AACCACTCGGCTCCCAAAAA	60.106	121
TRINITY_Contig11835	1	p3	(GGA)4	12	544	555	TCATCATCGTCGTCGTCGTC	59.974	GGTGGTTTTGTCGTTCGTGG	59.972	189
TRINITY_Contig11835	2	c	(TCG)5ccatct(TCC)5tcatcccacttgaagagatccacctcctggtcaccagcttccccacctccgtctgtccacagcctcaggaacctctccccgtcctcacctcccg(CGC)4	142	1547	1688	TCATCATCGTCGTCGTCGTC	59.974	GGTGGTTTTGTCGTTCGTGG	59.972	189
TRINITY_Contig11835	3	p5	(GAGGA)3	15	1978	1992	TCATCATCGTCGTCGTCGTC	59.974	GGTGGTTTTGTCGTTCGTGG	59.972	189
TRINITY_Contig11836	1	c	(CGC)5ccgccgacgaggacgcccccccgctgctcgatccaccggctccgccgccgccatca(CCG)4	83	449	531	GCAGGATCTCTAGCTGGAGC	59.682	GCCTATGAGACGACGAGCAA	59.899	107
TRINITY_Contig11836	2	p6	(AGCTGA)3	18	647	664	GCAGGATCTCTAGCTGGAGC	59.682	GCCTATGAGACGACGAGCAA	59.899	107
TRINITY_Contig11842	1	p2	(CT)26	52	686	737	CCTGTTGGCAGAGAATGGGT	59.961	CGTGTGTGATGCTAGCGTTG	59.906	173
TRINITY_Contig11844	1	p3	(CGG)4	12	51	62	GTCGCCTGTGGTGATCTTCA	60.037	GTGTGCAGGCTCTCACAGAT	60.037	174
TRINITY_Contig11846	1	p5	(GAGCG)3	15	318	332	CAAAACCTACACGTGGCAGC	60.04	CTAGTAGCAAGCTCCGCTCC	59.969	119
TRINITY_Contig11846	2	p3	(CCA)4	12	489	500	CAAAACCTACACGTGGCAGC	60.04	CTAGTAGCAAGCTCCGCTCC	59.969	119
TRINITY_Contig11847	1	c	(TC)8ttcatc(CT)11	44	239	282	ATGCTGCGAATTTGGGAGGA	60.034	GGATGCTTCCGGTGAGTTGA	60.036	162
TRINITY_Contig11847	2	p4	(CGAG)3	12	541	552	ATGCTGCGAATTTGGGAGGA	60.034	GGATGCTTCCGGTGAGTTGA	60.036	162
TRINITY_Contig11847	3	p6	(CCACCT)4	24	1841	1864	ATGCTGCGAATTTGGGAGGA	60.034	GGATGCTTCCGGTGAGTTGA	60.036	162
TRINITY_Contig11848	1	p4	(CCGC)3	12	176	187	TTTCTGCCAGTATTGCGGGT	59.962	ACCTAGGGGACGAGCATTCT	60.032	203
TRINITY_Contig11848	2	p3	(AGA)4	12	5019	5030	TTTCTGCCAGTATTGCGGGT	59.962	ACCTAGGGGACGAGCATTCT	60.032	203
TRINITY_Contig11849	1	p5	(TCTCA)3	15	613	627	CTCTCCATGAACGTCGTGCT	60.109	ATCAGACGCGCAGGTAGATG	59.969	208

TRINITY_Contig11850	1	p6	(GGCCGG)3	18	371	388	TAGCCCTGTTCATCGGAAGC	59.822	GACTGTAACTGGACACGGGT	59.322	121
TRINITY_Contig11851	1	p3	(CGG)4	12	1041	1052	GTCGAGCACTTTCTGGTCCA	59.966	GTACCTGGCGTGGAGTATCG	59.97	270
TRINITY_Contig11851	2	p3	(CGG)4	12	1341	1352	GTCGAGCACTTTCTGGTCCA	59.966	GTACCTGGCGTGGAGTATCG	59.97	270
TRINITY_Contig11851	3	p3	(TTC)4	12	2312	2323	GTCGAGCACTTTCTGGTCCA	59.966	GTACCTGGCGTGGAGTATCG	59.97	270
TRINITY_Contig11852	1	p3	(GCG)4	12	668	679	CTTTGCCGCAGCTGAAACAT	60.039	CGTGCTCTCTTCACCACAGT	59.967	240
TRINITY_Contig11853	1	p4	(GATG)3	12	1904	1915	GTGACGCCAAAGGAACAACC	59.97	GATTTCTCGCGCCACACATC	59.973	184
TRINITY_Contig11858	1	p3	(CCG)6	18	815	832	GTACTTGCGGACCATTGGGA	60.036	CCCCCACTACATAGAACCGC	59.894	144
TRINITY_Contig11863	1	p4	(GTTC)3	12	73	84	AGAAGAATTCCCCACGCGTT	59.964	TATCCAGTAGGCCAGCCACT	60.031	212
TRINITY_Contig11867	1	c	(TA)10(CATA)3	32	565	596	CTACAGCTAGCTGACGCCTC	59.97	TCCTCCGTCCTTCTACCCTG	60.033	268
TRINITY_Contig11868	1	p2	(GT)12	24	724	747	TTCCCTAGTGTTGTGGGTGC	59.89	GGTGGAGTGGTGGATGTCAG	60.036	280
TRINITY_Contig11872	1	p3	(ATG)4	12	1579	1590	TGGTGTCAGCCTTCGAAGAC	59.966	TCACGTGGCAGTTCGATCAA	59.967	169
TRINITY_Contig11874	1	p2	(TC)6	12	59	70	GGATGAGACCGTAGAGCAGC	59.969	CAGACCGACGTCATGATCGT	59.901	241
TRINITY_Contig11874	2	p4	(GCGA)3	12	684	695	GGATGAGACCGTAGAGCAGC	59.969	CAGACCGACGTCATGATCGT	59.901	241
TRINITY_Contig11880	1	p3	(GCG)4	12	115	126	TCAAGGTCGAACGGCAAGAA	59.895	CGAGCGATGCACAATCACTG	59.974	176
TRINITY_Contig11882	1	p5	(AAGAA)3	15	1555	1569	CATGGTTGGGAAAACGCAGG	60.038	GCCATGCAGAATGAGATGCG	60.04	118
TRINITY_Contig11884	1	p3	(CGG)5	15	550	564	CCAGCTCTCGTCGAACATGT	60.109	GTGCAGTTCCAAACGAGCAG	60.041	105
TRINITY_Contig11895	1	p5	(GGGCC)3	15	25	39	GTCGCGATTTGATTTCCCCG	59.972	CTTGAGCAGTTCGCCACCTA	60.037	135
TRINITY_Contig11895	2	p3	(GGA)5	15	1929	1943	GTCGCGATTTGATTTCCCCG	59.972	CTTGAGCAGTTCGCCACCTA	60.037	135
TRINITY_Contig11896	1	p4	(GCTA)3	12	149	160	CACGAGTTGAAGAACCCGGA	59.967	CCTCTTCGACGAGATGGACG	59.972	186
TRINITY_Contig11896	2	p3	(CGC)4	12	1336	1347	CACGAGTTGAAGAACCCGGA	59.967	CCTCTTCGACGAGATGGACG	59.972	186
TRINITY_Contig11898	1	c	(CTC)4ctttcctctccgtcgtctccgtctccatc tccatctcctcctccgcttccctcc(CCACG) 3	82	112	193	CGTCCATTGGCTATGTTGCG	59.972	TCCGAGTTGCAAAGCCTGAT	59.963	213
TRINITY_Contig11898	2	p3	(CCT)6	18	444	461	CGTCCATTGGCTATGTTGCG	59.972	TCCGAGTTGCAAAGCCTGAT	59.963	213
TRINITY_Contig11898	3	p2	(TC)16	32	2126	2157	CGTCCATTGGCTATGTTGCG	59.972	TCCGAGTTGCAAAGCCTGAT	59.963	213
TRINITY_Contig11899	1	p4	(CTAG)3	12	1759	1770	ACGGACTTGTGGAGGTAGGA	59.886	TCGGTCAAGCTTCCAAGGTC	59.965	141
TRINITY_Contig11900	1	c	(GGC)4ggtgtgcccgcgcgga(GGCG)3	40	2351	2390	AGCACCACGGATTCTGAAGG	60.036	CTTTTGACACACGGTGCCTG	59.971	274
TRINITY_Contig11900	2	c	(GCGA)3gccgagagattgaggtggaggag gtggtggtgtgggctggctcctggctgcgtt(G CGGC)3(GCGGT)3	96	2504	2599	AGCACCACGGATTCTGAAGG	60.036	CTTTTGACACACGGTGCCTG	59.971	274
TRINITY_Contig11901	1	p3	(TGC)4	12	842	853	CCTGTCAGGACGGACCAAAA	59.892	AGATACGCCATGACGACGTC	59.971	190
TRINITY_Contig11906	1	p3	(CAG)6	18	211	228	TCCATGTTGGCAGAGACCAC	59.963	AGTAGGATTAGCCCGGTCGT	60.106	103
TRINITY_Contig11906	2	p3	(CGG)4	12	372	383	TCCATGTTGGCAGAGACCAC	59.963	AGTAGGATTAGCCCGGTCGT	60.106	103
TRINITY_Contig11909	1	c	(CCTT)3gcttgttccaccaccatccctaaccc agttcataaataagcaagctgatagcaaatta aggc(GAA)5	89	264	352	CAGATCAGATCGGAGGCCAC	59.967	CATTGAATGCGAGCCATGCA	59.898	125
TRINITY_Contig11910	1	p3	(GCG)5	15	76	90	ATCGTAGAGAAGAAGCGGCG	59.97	ATCACGACCTTGGAACCGTC	60.038	221
TRINITY_Contig11911	1	p3	(CTA)4	12	453	464	CTGCTGTAGTAGTAGGCGCC	59.969	TCCGCTCGTGTATAAGTCGC	59.971	136
TRINITY_Contig11912	1	p4	(AGCT)3	12	45	56	CCGAGCTGCTTCCTTCTCAA	60.037	GAGGCAGTTGAGGTTGTCGA	59.966	264
TRINITY_Contig11913	1	p6	(CCGCCT)4	24	214	237	ATGGCCAACTCAACCCGAAT	59.96	CTGCAGTAGGTGGGGGAAAG	60.035	145
TRINITY_Contig11913	2	p5	(CTTTC)3	15	670	684	ATGGCCAACTCAACCCGAAT	59.96	CTGCAGTAGGTGGGGGAAAG	60.035	145
TRINITY_Contig11914	1	p3	(CGC)6	18	1323	1340	AGGCCGCGTTCCTGTATATG	59.966	TTCATGGAGCACACCACCTC	59.963	280
TRINITY_Contig11915	1	c	(CT)6ccag(TC)6	28	44	71	CGCGAAGATCCAGTGTACGA	59.902	GTCATCTTGCCTCGGAGGAG	59.896	117
TRINITY_Contig11916	1	p3	(CAC)4	12	213	224	CGGTTCACAGCACACTACCT	59.966	TATCTGCAAGGAGGAGGGCT	60.031	112
TRINITY_Contig11916	2	p4	(CCTC)3	12	1680	1691	CGGTTCACAGCACACTACCT	59.966	TATCTGCAAGGAGGAGGGCT	60.031	112
TRINITY_Contig11917	1	p4	(GCGA)3	12	464	475	CGAAGTGCTTTTGTCACGCA	59.973	AGCCCAACACTGCTGCATAT	60.034	242
TRINITY_Contig11920	1	p3	(GCC)4	12	675	686	GCCCATCACCACACTCATCA	60.035	CAGTGTGTATGTGTTGCCGC	60.11	145
TRINITY_Contig11921	1	p3	(CCG)7	21	137	157	CGGATGAAGCTTTCGCCTTG	59.902	CACAAGTCGCAGCAACAGAC	60.042	139
TRINITY_Contig11922	1	p3	(GCA)4	12	74	85	TCAAGGCGTCCATGGAGAAC	60.036	ACGGACGCTAGCTTTTTGGA	59.966	231
TRINITY_Contig11923	1	p3	(GCG)4	12	197	208	TTTAGAGCCTGCACGGAAGG	60.036	CCGTGCACCTGTTTCTCTCT	59.966	181
TRINITY_Contig11930	1	p3	(CGA)4	12	1056	1067	ACTGGCTGTTGACTACTGGC	59.965	CTCTTGATCTGCCGCCTGAT	59.894	147
TRINITY_Contig11931	1	p3	(TCC)5	15	153	167	TTCATCACGATCAGGCCCAC	60.108	GTTGTTCATCTCGTTGGCCG	59.834	217
TRINITY_Contig11932	1	p4	(GGCA)4	16	167	182	GAGGAAGATGGCGATGAGGG	59.965	GGTGCTCTTCATCCTGCTGT	60.036	145
TRINITY_Contig11932	2	c	(CTCTC)3tactcttctctctcttgtccctgaaa caacaa(CCT)4	59	431	489	GAGGAAGATGGCGATGAGGG	59.965	GGTGCTCTTCATCCTGCTGT	60.036	145
TRINITY_Contig11933	1	c	(GA)9gtggagtgcaggcaggaggaaggga aggagggcgtgcaggtgcagtacagtggcag gccgcttct(CTCTC)3ctccccttgcttggctt gct(GCAC)3gctcgctgctgcggctgctgct ccacgtctctgtctccgctttaaggctgtcacct ttccccatcccccctcctg(CTCC)3	216	15	230	TTTGTGCAAAGGCGTCCATG	59.968	TCGTTCTCCACTGTGTGAGC	59.968	214
TRINITY_Contig11935	1	p6	(TCCAGC)3	18	205	222	CGCTGGACTTGCCTCTTGTA	60.037	GAAGTGCTGGAAGATCCGCT	60.108	211
TRINITY_Contig11936	1	p3	(CCG)6	18	189	206	TTTGGTCGCTGGATGTGTCA	59.892	ATCCCTGACCTAGGCCAGTT	59.955	116
TRINITY_Contig11937	1	p4	(GGCC)3	12	506	517	CTCCTGCGTCATCTGCTGAT	59.896	CTCTGCAGCCTGAAATCCCA	60.035	275
TRINITY_Contig11937	2	p4	(GCCT)4	16	971	986	CTCCTGCGTCATCTGCTGAT	59.896	CTCTGCAGCCTGAAATCCCA	60.035	275
TRINITY_Contig11937	3	p3	(ATG)5	15	1100	1114	CTCCTGCGTCATCTGCTGAT	59.896	CTCTGCAGCCTGAAATCCCA	60.035	275
TRINITY_Contig11942	1	p3	(CGC)4	12	3417	3428	CCACGATCGAAATTTGGGGC	59.899	CCGTGGGGAGAATATGGAGC	59.965	153
TRINITY_Contig11944	1	p3	(CGC)5	15	432	446	AAGACGACTTTGACCCCGAC	59.967	GCGTGGTGGAAGTGGTAGAA	59.966	211
TRINITY_Contig11944	2	p3	(CGC)8	24	1892	1915	AAGACGACTTTGACCCCGAC	59.967	GCGTGGTGGAAGTGGTAGAA	59.966	211
TRINITY_Contig11946	1	p4	(CTAG)3	12	210	221	GAGAGGAGTTGTTGGCGTCA	59.966	ATCGGTGTAAGCAGGTGGTG	60.036	193
TRINITY_Contig11946	2	p3	(GCA)4	12	434	445	GAGAGGAGTTGTTGGCGTCA	59.966	ATCGGTGTAAGCAGGTGGTG	60.036	193
TRINITY_Contig11946	3	p3	(GCG)8	24	1138	1161	GAGAGGAGTTGTTGGCGTCA	59.966	ATCGGTGTAAGCAGGTGGTG	60.036	193
TRINITY_Contig11949	1	p6	(TCATCC)3	18	245	262	TGCCAAGAGTGCATCTCCAG	60.036	TGGCTGATGGAGATGGAGGA	60.031	177
TRINITY_Contig11949	2	p4	(GCCA)3	12	2085	2096	TGCCAAGAGTGCATCTCCAG	60.036	TGGCTGATGGAGATGGAGGA	60.031	177
TRINITY_Contig11949	3	p3	(CGG)4	12	2381	2392	TGCCAAGAGTGCATCTCCAG	60.036	TGGCTGATGGAGATGGAGGA	60.031	177
TRINITY_Contig11950	1	p3	(AGA)4	12	4	15	CGGAGCATACACTCTGCCAT	59.894	AACACACACGGACACGACTT	60.109	105
TRINITY_Contig11951	1	p3	(CGC)5	15	1054	1068	CTGATGATCGGGGAGCTGAC	59.967	GCAATCGCAATCCAATCCCC	59.967	155
TRINITY_Contig11951	2	p3	(CGG)4	12	2137	2148	CTGATGATCGGGGAGCTGAC	59.967	GCAATCGCAATCCAATCCCC	59.967	155
TRINITY_Contig11952	1	p3	(CCG)4	12	25	36	GAGGGTCAGCATCGTCAGAG	59.898	CGACACTGATGGTCTACCGG	59.899	248
TRINITY_Contig11954	1	p3	(CCA)4	12	478	489	TCGGTGAAGTTCATGGTGGG	59.964	TCAAGGCCATCGTCGAGAAG	59.827	193
TRINITY_Contig11955	1	p3	(CGC)5	15	443	457	CGACGAGGGGGAGAAGAAAG	59.826	TACAGGAAGCCGAAGCCTTG	60.036	168
TRINITY_Contig11959	1	p4	(AGCG)3	12	178	189	GTTCGGTGAAGCCCAGTACA	59.966	CCGAGGAGGGTCTTCTGAGA	60.034	132
TRINITY_Contig11959	2	p3	(CGG)4	12	395	406	GTTCGGTGAAGCCCAGTACA	59.966	CCGAGGAGGGTCTTCTGAGA	60.034	132
TRINITY_Contig11961	1	p3	(GTC)4	12	417	428	CTATCCATCTCGGTCGTCGC	60.11	GCCAGAGAGATCGTCCATGG	59.967	274
TRINITY_Contig11963	1	p4	(TCCA)3	12	229	240	CTCAATCCAACCCAGCGTCT	60.036	TAGGGAAGTCAGGTACCGGG	60.033	234
TRINITY_Contig11963	2	p3	(GCC)4	12	491	502	CTCAATCCAACCCAGCGTCT	60.036	TAGGGAAGTCAGGTACCGGG	60.033	234
TRINITY_Contig11964	1	p4	(GGAG)3	12	281	292	CAGGGACATGCAGGAGAAGG	60.107	GTGGGGTTCACGTGTCAGAT	59.965	187
TRINITY_Contig11968	1	c	(GATA)7gatgatcaacacccccaaatcaac (ACA)4	64	101	164	CAGTAGTGGACCATCCGACG	59.899	TACGTCAACTGCGTCGACAA	59.971	260
TRINITY_Contig11969	1	p3	(CGG)4	12	614	625	ACTTGTCGCTCGCTCTTTCA	59.968	GACTCACGCCAAGAGACACA	59.968	100
TRINITY_Contig11969	2	p3	(CCT)4	12	769	780	ACTTGTCGCTCGCTCTTTCA	59.968	GACTCACGCCAAGAGACACA	59.968	100
TRINITY_Contig11969	3	c	(CGAAGA)3gctaggaaggagaagaagag acggtggttggcgtcggggacggaggttagca gctgtggcatttagc(GGT)5	100	919	1018	ACTTGTCGCTCGCTCTTTCA	59.968	GACTCACGCCAAGAGACACA	59.968	100
TRINITY_Contig11972	1	p3	(CCG)4	12	1	12	TGGCACCGAAGAAGGACAAG	60.25	CTCCGTGCCAAGGATCCATT	60.107	235
TRINITY_Contig11972	2	p3	(AGA)4	12	135	146	TGGCACCGAAGAAGGACAAG	60.25	CTCCGTGCCAAGGATCCATT	60.107	235
TRINITY_Contig11973	1	p3	(GCT)4	12	169	180	CGCTTGACTTGAGCTGAGGA	60.038	AGCAAGGGACACTTTTGCCT	60.106	104
TRINITY_Contig11973	2	p3	(CGG)5	15	373	387	CGCTTGACTTGAGCTGAGGA	60.038	AGCAAGGGACACTTTTGCCT	60.106	104
TRINITY_Contig11975	1	p3	(GGC)5	15	320	334	AGGAGGACCTCGACCTTCTC	60.034	CAGCGCGGTTCAAACTTCAA	59.971	176
TRINITY_Contig11975	2	p3	(CTC)5	15	562	576	AGGAGGACCTCGACCTTCTC	60.034	CAGCGCGGTTCAAACTTCAA	59.971	176
TRINITY_Contig11976	1	p4	(CCGC)3	12	52	63	CTCCTCCATCCACTCCTCCA	60.032	ACCATGCAGCACATCCTCAA	59.961	255
TRINITY_Contig11976	2	p3	(GAG)5	15	639	653	CTCCTCCATCCACTCCTCCA	60.032	ACCATGCAGCACATCCTCAA	59.961	255
TRINITY_Contig11978	1	p4	(TCCA)4	16	16	31	TCTTCGACTTCGTGTGGGTG	59.968	ACTTGGACCAGAGCATGACG	60.037	246
TRINITY_Contig11979	1	p3	(GGC)4	12	1090	1101	CAGTTTCGGCTCAACGTGTG	60.042	CATACATCTCCTGCCCGGTC	59.966	275
TRINITY_Contig11984	1	c*	(CCG)4ctgcttatacgtcgcgtccgggaacg gagggagggacccccccccccccccccccc cccccccccccccccccccccgctcccac(C T)6(TCT)4*	122	6	127	GATTGGTACGAGAGGGCGAG	59.969	AAGAGCTTCGCCGTGATGAA	60.037	221
TRINITY_Contig11984	2	p3	(CGG)5	15	231	245	GATTGGTACGAGAGGGCGAG	59.969	AAGAGCTTCGCCGTGATGAA	60.037	221
TRINITY_Contig11985	1	p3	(CCG)4	12	149	160	TCGCGATCGGATCTGGAAAG	59.969	TGCGCAACAAAACTACAGGC	59.97	208
TRINITY_Contig11986	1	c	(CGCCGG)3cgccgtgggaggcgggcaag gtcgtccgcggcttcccgcagcacgccaggag cccgagcgcgagctggattccgagcatcgggg aggaggac(GAG)4	122	898	1019	GTCTCATCTCGGCGTCAGAG	59.971	AGCATCTTGAAGCCCCCTTC	60.034	192
TRINITY_Contig11988	1	p4	(CCGC)3	12	193	204	AAGCAAGTCGAACAGAGCGA	59.968	ACAGGCCTTTCCCCACAAAA	60.032	233

TRINITY_Contig11989	1	p3	(AGA)4	12	19	30	TGCCATAGTCGGGGTGTCTA	60.033	AGCTAGAGCACTCAGACCGA	60.035	105
TRINITY_Contig11989	2	c	(AGC)6aaccacagcagtcgtctccaactaagaaccagcaagggaccagtaaccaacagctgcttctttcc(CAG)4gaccagaatttgcaactgcagcagcaacagcagcttttacttcaacaactgcagcg(GCA)4	163	1879	2041	TGCCATAGTCGGGGTGTCTA	60.033	AGCTAGAGCACTCAGACCGA	60.035	105
TRINITY_Contig11990	1	c	(GTA)4cccggtcgccggcgggtagtacgcggggagcgc(CGG)4	57	335	391	GCGCGACGACTAGTACATCA	59.973	CTACTACTACCCGCCGATGC	59.76	204
TRINITY_Contig11990	2	p3	(CGC)4	12	639	650	GCGCGACGACTAGTACATCA	59.973	CTACTACTACCCGCCGATGC	59.76	204
TRINITY_Contig11992	1	p3	(GAG)5	15	2479	2493	CAAGCGTGACAATTGCACCA	59.969	TGATGCCCTCGCTCATTGTT	60.035	153
TRINITY_Contig11992	2	p3	(TGG)4	12	2632	2643	CAAGCGTGACAATTGCACCA	59.969	TGATGCCCTCGCTCATTGTT	60.035	153
TRINITY_Contig11992	3	p3	(GCG)5	15	2805	2819	CAAGCGTGACAATTGCACCA	59.969	TGATGCCCTCGCTCATTGTT	60.035	153
TRINITY_Contig11993	1	p4	(CCGG)3	12	111	122	CGAGATCTGCAACAAGGGGT	60.036	TTCTTCTCGCCGTGCTTTCT	59.966	219
TRINITY_Contig11993	2	p3	(AGC)4	12	1022	1033	CGAGATCTGCAACAAGGGGT	60.036	TTCTTCTCGCCGTGCTTTCT	59.966	219
TRINITY_Contig11994	1	p6	(GTCGCC)3	18	483	500	TGTCTCCTGCCTCAAGCTTG	59.964	CACACCCTCCGGATCCAATC	60.179	244
TRINITY_Contig12000	1	p3	(AGC)4	12	508	519	GCCAGTATCGCACCCACATA	59.894	CCACTGCTCTCGGACATGTT	60.037	184
TRINITY_Contig12002	1	c	(CT)6tttggc(CT)11cgtggtcccccgcgtcctcgtttttttaccccctcgcctgccggtcgccttgcaccccatccaccacgaaacggccgcctg(CAC)4	133	26	158	CGCTTTGCCATTTTTGCTGC	60.111	TCGCGTTCTCTATCCGCTTC	59.971	128
TRINITY_Contig12002	2	p4	(GCGA)3	12	292	303	CGCTTTGCCATTTTTGCTGC	60.111	TCGCGTTCTCTATCCGCTTC	59.971	128
TRINITY_Contig12002	3	p4	(GATG)3	12	1214	1225	CGCTTTGCCATTTTTGCTGC	60.111	TCGCGTTCTCTATCCGCTTC	59.971	128
TRINITY_Contig12005	1	p5	(CGGAG)3	15	332	346	CCTAGGGAGATCCAACACGC	59.895	CTTCCTCCACCACATCCACC	60.035	209
TRINITY_Contig12009	1	p3	(GAT)7	21	639	659	GCCGCCGATATCCAGATTCA	60.037	GACGGATCGATCTTGGGGTC	59.968	107
TRINITY_Contig12010	1	c	(GCA)4gct(GCC)5a(CAG)4	43	1044	1086	ACCGGCACCAGAAGAAGATG	60.036	GCGGCGGTGTTATTGTTGTT	60.04	141
TRINITY_Contig12012	1	p6	(GCTGCC)3	18	209	226	TTATCGCATGCTTTGTCGCG	59.974	GAGTGTCCTCGGAACACAGG	60.038	262
TRINITY_Contig12014	1	p3	(CGC)5	15	378	392	GAGATCCTCGGCTCGAACTG	59.97	GATTGGAGCGGTGAGGACAA	60.036	218
TRINITY_Contig12014	2	p4	(GGTG)3	12	618	629	GAGATCCTCGGCTCGAACTG	59.97	GATTGGAGCGGTGAGGACAA	60.036	218
TRINITY_Contig12015	1	p3	(CGC)5	15	92	106	CCTCCGCAGCAGAGGTTTTA	60.036	AGCATTTCCCTCAGCTTGCT	59.961	190
TRINITY_Contig12018	1	p2	(TA)6	12	301	312	TATATAGTTGCCGCCGCTGG	60.038	GAGCCTCTTCTCCGAGCTTG	60.179	264
TRINITY_Contig12019	1	p6	(CGGCGC)4	24	419	442	TGACATCAGCCTTCCAGCAG	60.036	TTGCGCTTCTGTGATGGGAT	60.035	248
TRINITY_Contig12019	2	p3	(GCT)4	12	1209	1220	TGACATCAGCCTTCCAGCAG	60.036	TTGCGCTTCTGTGATGGGAT	60.035	248
TRINITY_Contig12020	1	p5	(GGCCC)3	15	238	252	GTTGCTTCCTCCAGCTAGCA	60.036	TCCATCCACCAACCACAACC	60.179	132
TRINITY_Contig12021	1	c	(CT)16cgcctcgccgccggcga(CGAGCT)3	67	6	72	GCGCTATGCAAAACTCGAGG	59.973	GGGCGAGAGTTGAAAGTGGA	59.965	150
TRINITY_Contig12022	1	p2	(CT)12	24	248	271	AAGAGCGAGAAAGCAGGCTT	59.964	GCTTGTTCTCCTGAGCTGGT	59.964	154
TRINITY_Contig12029	1	p3	(CGG)4	12	109	120	AGCAGCAAGGAAGCTACGAG	60.108	CACGTCTTGCACTCGAACAC	59.77	152
TRINITY_Contig12030	1	p3	(CGC)4	12	375	386	AGAGGATCCGCTTGCAACC	60.078	CCTCCTCTTCTCTCTCCCCC	60.106	128
TRINITY_Contig12030	2	p3	(GCC)4	12	583	594	AGAGGATCCGCTTGCAACC	60.078	CCTCCTCTTCTCTCTCCCCC	60.106	128
TRINITY_Contig12031	1	p6	(GTAGGG)3	18	131	148	CAGGGCTCCTTCTTCTTGGG	60.035	AGATCCTGGCTGCTGTTTCC	60.035	184
TRINITY_Contig12032	1	p3	(GCG)4	12	239	250	GGGTTTTGGTGATGCTGCTG	60.038	TCCGGTCCTTCTCATACCGT	60.034	107
TRINITY_Contig12034	1	p3	(GCG)4	12	3651	3662	CCGGTCGTATCCAAACCCAA	60.036	GATAGGATCGCAAGGGACCG	60.038	103
TRINITY_Contig12035	1	p3	(CGC)5	15	185	199	TCTTCCGAGCACTGGTCAAC	59.966	CGCCCAAATGCTGTGACTTT	59.685	236
TRINITY_Contig12036	1	p3	(GGC)4	12	421	432	GCAACAGCCATGCAATCCAT	59.823	TGACGTACGTGTGGTGTCTG	59.97	113
TRINITY_Contig12037	1	p4	(ATTA)3	12	484	495	CCATGCTCTGGGCGTAGTAG	59.967	CCCGAGAAGAAGGAGGAGGA	60.033	259
TRINITY_Contig12037	2	p3	(AGC)6	18	1498	1515	CCATGCTCTGGGCGTAGTAG	59.967	CCCGAGAAGAAGGAGGAGGA	60.033	259
TRINITY_Contig12042	1	p5	(AAAAG)3	15	155	169	CGTACAACCACCCAATCCCA	59.963	CAACATCGAGACCAGGCACT	60.037	136
TRINITY_Contig12046	1	p3	(CCG)4	12	254	265	GCTCCCTCATCCAGAAGCTC	59.893	GCGTTGCAGCAAAGCTTAGT	60.04	249
TRINITY_Contig12049	1	p3	(CCG)4	12	228	239	GCGCTCTAGGTAGGAACGAC	59.971	GCGATTGGGCGTTAATCACC	59.971	274
TRINITY_Contig12049	2	p3	(CGC)6	18	355	372	GCGCTCTAGGTAGGAACGAC	59.971	GCGATTGGGCGTTAATCACC	59.971	274
TRINITY_Contig12049	3	c	(CTC)6gcgctctaggtaggaacgactcctcggggtcggccggagtgcctcga(CCGC)4	81	506	586	GCGCTCTAGGTAGGAACGAC	59.971	GCGATTGGGCGTTAATCACC	59.971	274
TRINITY_Contig12049	4	p2	(AG)7	14	751	764	GCGCTCTAGGTAGGAACGAC	59.971	GCGATTGGGCGTTAATCACC	59.971	274
TRINITY_Contig12053	1	p2	(CA)8	16	36	51	CTCCCAGTCTCCACACACAC	59.966	GGCCAGCAAAAATGAGCCAA	59.965	274
TRINITY_Contig12053	2	p2	(CG)6	12	261	272	CTCCCAGTCTCCACACACAC	59.966	GGCCAGCAAAAATGAGCCAA	59.965	274
TRINITY_Contig12054	1	p2	(CT)19	38	853	890	GTGGTGGTGGTAAGGATGGG	60.034	AGTGTACTGGCAAGCATCCC	60.035	173
TRINITY_Contig12058	1	p6	(GAGGGA)3	18	621	638	AGCAAGCGTGACTGAAGGAA	59.893	CCGTGGATGGGCTCAGATTT	60.107	115
TRINITY_Contig12063	1	p3	(CCG)5	15	1815	1829	CGGTCTCCGGATGGATGAAG	59.967	TGCATGTCCGATCCTCGATG	59.967	154
TRINITY_Contig12064	1	c	(CTG)5gctcatcttctggtacagcgccaccatcttgctgtacgcctccatgccgcccggt(TGC)6	88	396	483	ATCCGCACTCATTTGCCAGA	60.035	TTGCTTGATCGAAACCCCGA	59.964	231
TRINITY_Contig12064	2	p3	(GGC)4	12	761	772	ATCCGCACTCATTTGCCAGA	60.035	TTGCTTGATCGAAACCCCGA	59.964	231
TRINITY_Contig12065	1	p4	(GAAA)4	16	81	96	CAAGGAGATGCGTGGCAATG	59.9	GCATCAGAACGAGGAGGAGG	59.896	245
TRINITY_Contig12065	2	c	(AGCT)3atagctactcatcatcatcgtggccgccatggctctccacctcgtcg(TCCTGA)3	77	213	289	CAAGGAGATGCGTGGCAATG	59.9	GCATCAGAACGAGGAGGAGG	59.896	245
TRINITY_Contig12065	3	p2	(GC)6	12	1020	1031	CAAGGAGATGCGTGGCAATG	59.9	GCATCAGAACGAGGAGGAGG	59.896	245
TRINITY_Contig12065	4	p3	(GCC)6	18	1508	1525	CAAGGAGATGCGTGGCAATG	59.9	GCATCAGAACGAGGAGGAGG	59.896	245
TRINITY_Contig12065	5	p3	(CGC)4	12	1731	1742	CAAGGAGATGCGTGGCAATG	59.9	GCATCAGAACGAGGAGGAGG	59.896	245
TRINITY_Contig12068	1	p4	(CTCC)3	12	1	12	TGGATGAATGATGCCGGACC	60.179	CACTGCGTGCATGCATGTTA	59.831	148
TRINITY_Contig12068	2	p4	(CCGT)3	12	174	185	TGGATGAATGATGCCGGACC	60.179	CACTGCGTGCATGCATGTTA	59.831	148
TRINITY_Contig12069	1	p3	(GGC)4	12	126	137	TGCTATCACCTGCCAGTGTG	60.036	TACGTTCTCCTCGGGGACTT	59.961	182
TRINITY_Contig12073	1	p6	(CAGCCG)3	18	370	387	GGCCAACACGATGGAGAAGA	60.036	CGCGTACATGTAGGAGTCCG	60.317	178
TRINITY_Contig12078	1	p6	(CGCCGG)3	18	142	159	GGCAACACCAAGTACGAGGA	59.966	CCCTACCACCAGAGTCCTCA	59.959	264
TRINITY_Contig12078	2	p3	(GGC)5	15	298	312	GGCAACACCAAGTACGAGGA	59.966	CCCTACCACCAGAGTCCTCA	59.959	264
TRINITY_Contig12080	1	p3	(GCC)4	12	174	185	GTTGAGGAGACTGCGGAGAC	60.109	CTCCCTCTCCTACCTCCTCG	59.89	138
TRINITY_Contig12081	1	p3	(CTG)4	12	398	409	GTTGTCAGCGTAGGCGTAGT	60.109	AATGCCACCTGGAGAACGAG	60.036	263
TRINITY_Contig12081	2	c*	(TCGCCG)2(T<CGCCG>CCGCCGCCGCACCTCGCGGCCCGCCGCCTCCTGCGCCGCGCTCTCGGCCTCTCCCCTTGCGCCGCGCCGTTGGGCACC)(CGC)4	28	837	864	GTTGTCAGCGTAGGCGTAGT	60.109	AATGCCACCTGGAGAACGAG	60.036	263
TRINITY_Contig12082	1	p3	(CCG)5	15	423	437	GCCATCCCTGTAGCGTAGTC	59.968	AACTCGTGTTCGACCCTGTC	59.968	261
TRINITY_Contig12085	1	p3	(GGC)4	12	352	363	GTCGTCGTCTCCTCATCGAC	59.973	GGGCCTTCTCTTCTGCACTT	59.963	120
TRINITY_Contig12086	1	p3	(CAC)4	12	418	429	AGATCAGTTGTGTCTCGCGG	60.109	GATACTGCTGTGGCGCAAAG	59.902	269
TRINITY_Contig12090	1	c	(GCCG)3ccgcgaccgaaaaaaccccacgcattctcgaacctccccacctttc(CCGG)4	74	79	152	TAGCCGACAAGGTTGCTCTG	60.037	TACAGCAGCCTGGATCTCCT	60.032	263
TRINITY_Contig12090	2	p2	(GA)7	14	397	410	TAGCCGACAAGGTTGCTCTG	60.037	TACAGCAGCCTGGATCTCCT	60.032	263
TRINITY_Contig12092	1	c	(CCCCG)5ctgccatcctgacgcccgcaccggtactcaccgaggtacggtcccctcct(TCCC)3	87	40	126	CTCCCCGCTTGCCTGTATAG	59.966	TGCAACAATGGTCAGGCTCA	60.179	214
TRINITY_Contig12099	1	p3	(GCC)5	15	549	563	AGCGACGTGTTCTCGTACTG	60.11	TGACTCACAAACCCTCGTCG	59.968	148
TRINITY_Contig12100	1	c	(GCG)4agcggctccagcagctccgtctcgtactcgtcc(CCG)5	60	2812	2871	CCAAGGGCAAGAACAAGCAC	59.967	GATCGATCTCTGCCTGGGTG	59.967	269
TRINITY_Contig12101	1	p3	(CGC)7	21	1061	1081	TCGATCAGAGCGTATCGTGC	60.041	ACAGTGACATCGAGGGCAAG	60.037	128
TRINITY_Contig12101	2	p3	(AGG)5	15	1660	1674	TCGATCAGAGCGTATCGTGC	60.041	ACAGTGACATCGAGGGCAAG	60.037	128
TRINITY_Contig12102	1	p4	(CGCT)3	12	358	369	TGCAAGACCACACCTTCGAC	60.532	GACGCCCTACCCACTCATTC	60.179	118
TRINITY_Contig12104	1	p4	(AGCG)3	12	393	404	GTTGCAAGGCGTCCAAGAAG	60.04	AGTCAGTCGCGTGAGAAAGG	60.039	153
TRINITY_Contig12105	1	p4	(GGGC)3	12	731	742	GCCACAAGCCAGTTATCCCT	60.034	CTTCGGTCCTCTGTATCCGC	59.969	275
TRINITY_Contig12107	1	p3	(CCG)4	12	310	321	GCCACCTTCTACTACGGCAA	59.752	TGGGGCTAAACAGCTGATGG	60.034	181
TRINITY_Contig12108	1	p6	(CCGGCT)3	18	455	472	AACGCCATTAAGCAGACCGA	60.037	CTTGTACGGGGTGTCGAACA	59.968	234
TRINITY_Contig12113	1	p3	(GAG)4	12	373	384	ACCATTTTACCCTTCGCGGT	59.963	CAGCTTCAACATGCCCAACC	60.038	125
TRINITY_Contig12113	2	c	(GGA)5gaagaaggggaacgagccatacgccgtgccgacgagcggtgccttctacatgcacgatgaccgcttccaggag(GGCCGT)3	106	546	651	ACCATTTTACCCTTCGCGGT	59.963	CAGCTTCAACATGCCCAACC	60.038	125
TRINITY_Contig12118	1	p3	(CTT)4	12	594	605	CCGTCGCTTGAGAGTTGAGA	59.759	CTAGTGGCAGGAGCATCCAG	59.894	109
TRINITY_Contig12120	1	p3	(GCG)6	18	194	211	GTAGCTCTCCATCGCCGAAG	60.318	CTCCTCCTCTCCTCTCCTCC	59.521	111
TRINITY_Contig12120	2	p5	(GAGGA)3	15	570	584	GTAGCTCTCCATCGCCGAAG	60.318	CTCCTCCTCTCCTCTCCTCC	59.521	111
TRINITY_Contig12121	1	p3	(CGC)4	12	365	376	TCTTCCGTCTCGTCTCGTCT	60.038	ACAGCTTGTTCTCCTCCAGC	59.964	216
TRINITY_Contig12121	2	c	(CGCC)3tgcccagctgcgaagtag(GCCGGC)3ggcagcacgtccatccacgactccgccgtcatcagccgcccacgactccgatcggccgcgccgggccgaccaagatcgcgcctttccctgtcgcggg(GAGAC)3	160	595	754	TCTTCCGTCTCGTCTCGTCT	60.038	ACAGCTTGTTCTCCTCCAGC	59.964	216

TRINITY_Contig12121	3	c	(CGTCT)3ccccgcgacagggaaaggcgcgatcttggtcggcccggcgcggccgatcggagtcgtgggcggctgatgacggcggagtcgtggatggacgtgctgc(CGCCGG)3cctacttcgcggggca(GGCG)3	158	1207	1364	TCTTCCGTCTCGTCTCGTCT	60.038	ACAGCTTGTTCTCCTCCAGC	59.964	216
TRINITY_Contig12122	1	p3	(GCG)4	12	895	906	TGCTGGATCAAACGTTCCGA	59.966	AAATGTCCAACGCTCCGAGT	59.965	150
TRINITY_Contig12125	1	p4	(GGCG)3	12	411	422	GGCCCCCACAAAGATTCTGA	59.96	TGGCAGAGGAAAGTACAGCG	60.037	140
TRINITY_Contig12126	1	p3	(GCT)5	15	136	150	GCAGCAGTGTGTTCTTGCTC	60.041	CATGATCTGACGCTACCCGG	60.319	153
TRINITY_Contig12126	2	p3	(GCG)4	12	255	266	GCAGCAGTGTGTTCTTGCTC	60.041	CATGATCTGACGCTACCCGG	60.319	153
TRINITY_Contig12128	1	p3	(AGA)4	12	1909	1920	TCTTGGCGTCCACCTTCTTC	59.965	GACGGGGCAGAAGAACAAGA	59.965	137
TRINITY_Contig12130	1	p3	(CAG)4	12	659	670	AAACGTTGCTGACACGAACG	59.974	CGATGGCAAGAGACGATCGA	59.971	239
TRINITY_Contig12131	1	p3	(GCG)4	12	183	194	TTGGTCGACAAGATGGGACG	60.038	TTCGGTATGCTCCACCACAG	59.751	150
TRINITY_Contig12133	1	c	(GGCG)3cgtggggttcccattcgtgccggtctcttccttcgacccccgtttgcgagggtagcgtcgcggtga(GGC)5	93	315	407	CTGCGTGCCATGTTCCTCTA	60.108	GCGAGCAATTTGAAGCGGAA	60.11	105
TRINITY_Contig12134	1	p4	(GAGG)3	12	563	574	GGCCCCCACAAAGATTCTGA	59.96	TGGGGTGAGCAGGATGACTA	59.957	178
TRINITY_Contig12138	1	p5	(AAAAG)3	15	44	58	TGTTGGAACATGGACGAGGG	59.964	CAGGCAGTACACCTCGGTTT	59.965	122
TRINITY_Contig12139	1	p3	(GGC)4	12	459	470	AATGGACCCATGCGTTGCTA	60.035	TCCATGTACGGCAAGCTTGT	59.964	171
TRINITY_Contig12141	1	p3	(TCC)4	12	360	371	CAGATGGATCCCAGGAAGGC	59.89	CTTCGTCTGTCTCCATGGCC	60.461	280
TRINITY_Contig12142	1	p3	(TCG)4	12	278	289	GGCCCTCATCTTGTTCGACA	60.036	ATGACCGTGATGAGTCGCAG	60.179	270
TRINITY_Contig12143	1	p3	(GCG)6	18	170	187	GACGAGAGCTCCTTCTACGC	59.972	GAGCACTAAACCCTACCGGC	60.462	108
TRINITY_Contig12145	1	p3	(GCT)4	12	72	83	GTGGACGAAGAAGCAGGACA	59.966	TCTTCAGGTGCTTCAGCCTG	59.964	254
TRINITY_Contig12145	2	p3	(CGG)4	12	361	372	GTGGACGAAGAAGCAGGACA	59.966	TCTTCAGGTGCTTCAGCCTG	59.964	254
TRINITY_Contig12145	3	p4	(ATGT)4	16	564	579	GTGGACGAAGAAGCAGGACA	59.966	TCTTCAGGTGCTTCAGCCTG	59.964	254
TRINITY_Contig12146	1	p4	(GCCG)4	16	825	840	CTTCGTCTGCCTCGTCAACT	60.039	GTAGAACTCGATCGTCGCGT	59.974	220
TRINITY_Contig12146	2	p3	(GCG)4	12	1063	1074	CTTCGTCTGCCTCGTCAACT	60.039	GTAGAACTCGATCGTCGCGT	59.974	220
TRINITY_Contig12148	1	p3	(CGG)5	15	1544	1558	CTGAATCAGGTCGGGTTGCT	60.036	TGAGGTCAAGGTCGTGCATC	60.037	256
TRINITY_Contig12149	1	p3	(CCG)4	12	640	651	TAGTGGCAGCTGGGTTCTTG	59.963	GTCCACCTCACCGCTTGTTA	59.966	211
TRINITY_Contig12149	2	p3	(GTG)4	12	821	832	TAGTGGCAGCTGGGTTCTTG	59.963	GTCCACCTCACCGCTTGTTA	59.966	211
TRINITY_Contig12152	1	p3	(GGC)4	12	377	388	GGTTGCAGGCGCTGTAAAAA	59.969	TCTCCTCATCCTCACCCCTG	60.032	114
TRINITY_Contig12153	1	p3	(CAT)4	12	135	146	ACACAACTTGCTACTGGCCA	59.817	CCATGCTGGGATTGGTCTGT	60.034	123
TRINITY_Contig12154	1	p4	(CTGT)3	12	59	70	TGAGGCAGCTGTTCCAGAAG	59.964	GTGGATCAACTACCTCCGCC	60.179	170
TRINITY_Contig12154	2	c	(CGT)4ccgccgcctgctcc(CCG)4tagcctccgctg(TGC)5tccagctccgtgtacggctccggcttggcgg(CGC)5	111	377	487	TGAGGCAGCTGTTCCAGAAG	59.964	GTGGATCAACTACCTCCGCC	60.179	170
TRINITY_Contig12154	3	p3	(CGC)4	12	1229	1240	TGAGGCAGCTGTTCCAGAAG	59.964	GTGGATCAACTACCTCCGCC	60.179	170
TRINITY_Contig12157	1	p3	(TGG)4	12	556	567	ACCGTCGTGCTTGATGTTCT	59.966	GGACTGGAAGGGTCTCAACG	60.037	154
TRINITY_Contig12161	1	p4	(TGGA)3	12	1554	1565	GTAGCCCATGTCACAAGGCT	60.035	CGTGTTCTTGGCACCTTTGG	59.969	173
TRINITY_Contig12164	1	p3	(CCG)4	12	58	69	CAACAGCTCCTTCTGCCTGA	59.964	ATGAGAAGGTGCGCAACTGA	59.965	102
TRINITY_Contig12168	1	p3	(GGC)4	12	506	517	GCCTCCTCGCCATAATTCCA	59.891	GAAGCGAAAATCTTCCCGGC	59.901	112
TRINITY_Contig12170	1	c	(CTG)4ctactagtgctacctgcctgagtgccggcctggtcaaacttgcccagtccgtcgcatcccattcg(AGCC)4tcgccttcgccgcagcgacgtgcgcgtgcgtcggtcgcctccctctctagctgtgtgcctgcgc(GCG)6ggccggcggt(GATGGA)3	203	247	449	TGGGGAAGAACACGTACAGC	59.966	GGAAGACACAGAAGGGGAGC	60.036	174
TRINITY_Contig12172	1	p4	(TGTA)13	52	477	528	CTGATTCCCTCGTCGACAGG	59.899	GTGGAACTAGCGGAAGGGTC	60.108	120
TRINITY_Contig12174	1	p3	(CGC)5	15	1035	1049	ACACGTCACAAAATGGCACG	59.971	CTCCACAGCTGGTTATGCGA	60.108	119
TRINITY_Contig12179	1	p3	(GGA)4	12	127	138	TATGCGCCACCAGGTTGTAG	60.108	CCAGCAATCAGAGCAGGCT	60.078	114
TRINITY_Contig12186	1	p4	(CTCG)3	12	15	26	AAGCTCCGCGAATCAAGGAA	60.036	AGTCGAACCAGCACCTTCTG	59.966	135
TRINITY_Contig12188	1	c	(CCG)4cacggaagaag(GCCGC)3	38	124	161	TGAATCCTGACGCGTGACAA	59.967	AGCTAGTTGACACGCTGACC	60.038	143
TRINITY_Contig12190	1	p5	(TCTTC)3	15	328	342	GTAAACGTCTGTGGAGCCCA	59.966	CAGGAGGTGAGGGATGAGGA	60.032	119
TRINITY_Contig12191	1	p3	(GAA)4	12	1677	1688	TATCAGTTACCGACGCGAGC	59.971	TCACGGGGAGGGCAATAAAC	60.035	223
TRINITY_Contig12193	1	p3	(ACC)6	18	384	401	CCACCCAATTAGCCTCGGTT	60.034	TGCTCACCTTGACCACCATC	59.963	122
TRINITY_Contig12194	1	c	(AGGAC)3(AGGTC)4aggatcatcacctcacctcttcg(CCGCA)4	78	12	89	AAGATCTCCATCGCGTCGTC	59.97	TACACCTTCTGCACCGTGAC	59.967	170
TRINITY_Contig12194	2	c*	(GCG)3(<GCG>AGC)(GCGAGC)2	27	650	676	AAGATCTCCATCGCGTCGTC	59.97	TACACCTTCTGCACCGTGAC	59.967	170
TRINITY_Contig12200	1	p4	(GAGG)3	12	66	77	TTCGGAGCGATTTCGTGGAA	60.038	CCCTCCAGCAAAGCATCTGA	60.035	186
TRINITY_Contig12205	1	p3	(CGC)4	12	116	127	GATGAGCGAGTATGTCCCCG	60.039	GTGAAGGCAAGGAACGGAGA	59.965	118
TRINITY_Contig12208	1	p5	(GCTGA)3	15	289	303	GCGGCTATTTAAGAAGGCGC	60.041	CTCAGCCGATCAGTCAGGTC	59.898	110
TRINITY_Contig12212	1	p3	(CGG)4	12	1157	1168	GTGTTGCAGTGAAGCAGGTG	59.97	ACCACTCCGTATCAGCTCCT	60.033	100
TRINITY_Contig12214	1	p5	(GCGAG)3	15	4219	4233	GAATCAAGGATGTGCGCGAC	59.973	ATACCCGTGGTACTGGCTCT	60.032	262
TRINITY_Contig12216	1	p5	(GGTCA)3	15	2937	2951	GGGTGTGGGTTGATCGGATT	60.034	TGCGTGGTAGCACATTGTCT	59.965	240
TRINITY_Contig12216	2	p3	(CTT)5	15	3065	3079	GGGTGTGGGTTGATCGGATT	60.034	TGCGTGGTAGCACATTGTCT	59.965	240
TRINITY_Contig12220	1	p2	(AT)9	18	42	59	CGATGAGGACGGTGGTGTAG	59.899	CTTACACCGGCGACTCCAAT	60.108	251
TRINITY_Contig12220	2	p3	(AGG)7	21	969	989	CGATGAGGACGGTGGTGTAG	59.899	CTTACACCGGCGACTCCAAT	60.108	251
TRINITY_Contig12221	1	p3	(CCG)4	12	1395	1406	ATCAGCTCAACCTCACGCAA	59.965	TGGCGAGAATTGCAACTTGC	60.039	179
TRINITY_Contig12221	2	p4	(GGTG)3	12	1592	1603	ATCAGCTCAACCTCACGCAA	59.965	TGGCGAGAATTGCAACTTGC	60.039	179
TRINITY_Contig12222	1	c*	(AG)10atggcgcccccgccccaccagcgggcgctgctcctgttcccgctcgtct(TCC)6(CCCCG)3*	100	58	157	TCGGGGCCATCAACAACTAC	60.036	CCTTCGTCATCCTCGTAGCC	59.969	145
TRINITY_Contig12222	2	p3	(GGC)4	12	326	337	TCGGGGCCATCAACAACTAC	60.036	CCTTCGTCATCCTCGTAGCC	59.969	145
TRINITY_Contig12223	1	c	(GGC)4gcgcatggcaagtcagtctcctccattcccctgcggctgtcaccgccgaccgaa(TCCC)3actcccat(GCC)6gcgttgagaggaaggggaaggcgaagccggaggatggggaaggtgccgcaagccatgcg(CGC)4	175	7	181	GGCGCTTAAATTGCTCGAGG	59.972	CGATCGACACAACACCCTGA	60.039	196
TRINITY_Contig12223	2	p6	(AGGGGG)5	30	346	375	GGCGCTTAAATTGCTCGAGG	59.972	CGATCGACACAACACCCTGA	60.039	196
TRINITY_Contig12228	1	p4	(CCTG)3	12	41	52	AGCAGAAACGCTTGCACTTG	59.97	CAGATGGACAGGTCGGATCG	59.969	144
TRINITY_Contig12231	1	p2	(CT)6	12	2698	2709	ACAAAGGGTTCCATAGGCCG	60.034	TAGAAAGGGAGGGGATGGGG	60.029	241
TRINITY_Contig12240	1	c	(GCCG)5cgacccgaatcggagggcgggttccacgatcagcggatcccatcgccc(GCG)4gcattgatagacgatccc(GCCG)3	110	639	748	GTGCACCTCCTTCGTCAAGA	59.966	CGAGGTGTCAGCATTGGTCT	60.037	250
TRINITY_Contig12240	2	p3	(CGA)4	12	1166	1177	GTGCACCTCCTTCGTCAAGA	59.966	CGAGGTGTCAGCATTGGTCT	60.037	250
TRINITY_Contig12240	3	p3	(GGC)4	12	2327	2338	GTGCACCTCCTTCGTCAAGA	59.966	CGAGGTGTCAGCATTGGTCT	60.037	250
TRINITY_Contig12240	4	p3	(AGG)4	12	2651	2662	GTGCACCTCCTTCGTCAAGA	59.966	CGAGGTGTCAGCATTGGTCT	60.037	250
TRINITY_Contig12242	1	p3	(CAG)4	12	1316	1327	CACTGTGACTGTCCCATCCC	60.036	TTCTTGCAGGCCCTCATTGT	59.887	108
TRINITY_Contig12245	1	c	(CCAC)3g(ACCC)3	25	7	31	CCATTTCAGAGCTCCGGGTT	60.035	CTCCGAATCTCTCCACGAGC	59.97	241
TRINITY_Contig12245	2	p3	(GCC)5	15	849	863	CCATTTCAGAGCTCCGGGTT	60.035	CTCCGAATCTCTCCACGAGC	59.97	241
TRINITY_Contig12246	1	p3	(CGC)6	18	91	108	GTGTTCTTCCTGTACGCCCT	59.679	TCTTCCATGGAGATCGACGC	59.614	266
TRINITY_Contig12246	2	p2	(AG)15	30	411	440	GTGTTCTTCCTGTACGCCCT	59.679	TCTTCCATGGAGATCGACGC	59.614	266
TRINITY_Contig12247	1	p3	(CGC)4	12	2623	2634	TCGGTGGTGGTAGTTTCAGC	59.966	ATGATGCATGCCACTGTCCA	60.034	167
TRINITY_Contig12249	1	p3	(TGA)4	12	505	516	ATCTCTCGGCTCCTGAGGTT	60.033	TTCTCACGCCTGTTCAGGTC	59.966	178
TRINITY_Contig12251	1	p3	(GCG)4	12	1067	1078	GCTGCAGGTGTGTTTTCTGG	59.969	CAGGGAAGGAAGAGCAAGGG	60.035	230
TRINITY_Contig12253	1	p3	(CGT)4	12	165	176	AATTGAAGAGAGTGGCGGGG	60.035	CGCGTCTCGTCTCATCATCA	59.972	125
TRINITY_Contig12257	1	p3	(CGC)5	15	35	49	ACCCTAGTGGCTCCATCCAT	60.03	GTCGGAGTTGTAGTCACGGG	60.109	237
TRINITY_Contig12257	2	c	(GAAGAG)3gacgagcaggagcgcccgccgagccca(CGC)4cgtgacgacgacggcgactgcgg(CGA)6	98	312	409	ACCCTAGTGGCTCCATCCAT	60.03	GTCGGAGTTGTAGTCACGGG	60.109	237
TRINITY_Contig12258	1	p3	(GCC)4	12	165	176	AGGAGGACTTCGAGGACCTC	60.034	GCTTGCTTTGCTTGCACTCT	59.968	215
TRINITY_Contig12259	1	p3	(GGC)4	12	1275	1286	TCAGAGGACACCATGGCAAC	59.963	CTGAGGTTGGTTGCTTTGCC	59.967	199
TRINITY_Contig12259	2	p4	(CTCC)3	12	1526	1537	TCAGAGGACACCATGGCAAC	59.963	CTGAGGTTGGTTGCTTTGCC	59.967	199
TRINITY_Contig12261	1	c	(GTG)4gtcgccgccggcgcctccgccgccgcccgcccccgcgacatgcagcgacgacgacggcgggaccaccgcgtcgacgtcgtcggggc(GCT)5	113	171	283	CCTGGTTCTGCAGGAAGGAG	60.036	TGTTCGGGGCCAGTAATGTC	60.036	153
TRINITY_Contig12262	1	c	(GA)7ggaga(GTG)5	34	36	69	TGTTAACCACGGGTGTCGAG	59.968	CCTCATCTGCGGATTTTGCG	59.971	266
TRINITY_Contig12262	2	p4	(AGGC)5	20	190	209	TGTTAACCACGGGTGTCGAG	59.968	CCTCATCTGCGGATTTTGCG	59.971	266
TRINITY_Contig12262	3	p4	(AGGC)3	12	2514	2525	TGTTAACCACGGGTGTCGAG	59.968	CCTCATCTGCGGATTTTGCG	59.971	266
TRINITY_Contig12263	1	p3	(CGC)5	15	359	373	GATAAAATGCTAGCTCGCCGC	60.136	AGCTCCGACTGAAGAGGCTA	60.034	100
TRINITY_Contig12264	1	c	(AAT)10gg(CTT)4	44	71	114	TGTAGGGTTGACAACAGGTGT	59.159	CCAAGCACAACTCCACCAGA	60.179	129
TRINITY_Contig12269	1	p3	(CCG)4	12	534	545	GAAAGAGTTGGCCGAGGTGA	59.965	CCGGAGAAGTAGGACCTGGA	60.033	213
TRINITY_Contig12270	1	p3	(CTG)4	12	673	684	CTGTCAGACACCTTCCGACC	60.038	GGGGTGCCCTCTAGCAATTT	60.033	200
TRINITY_Contig12272	1	p3	(TCC)4	12	54	65	GGCCAGGTAGGAAGCTTTGT	59.962	CAACGTGAGCCGATAAAGCG	59.975	238
TRINITY_Contig12272	2	p3	(GCC)4	12	408	419	GGCCAGGTAGGAAGCTTTGT	59.962	CAACGTGAGCCGATAAAGCG	59.975	238

TRINITY_Contig12272	3	p4	(CGGG)3	12	601	612	GGCCAGGTAGGAAGCTTTGT	59.962	CAACGTGAGCCGATAAAGCG	59.975	238
TRINITY_Contig12277	1	p3	(GGT)5	15	47	61	TTCTCGACGGCGGAATATGG	59.968	TCTGGCGTTGGAGCTTTCTT	59.891	211
TRINITY_Contig12279	1	c	(ACTG)3atcactgatcgatcggcggcgggagacggaagaatctgtcgttccaaccgctccccagatccggagtcaaagggcgccttgagccaaaggcaggggcgag(CAC)5	127	140	266	AGAATGGATCCCCTGGAGCT	60.029	CGATCCCGAGACCGAAGAAG	59.97	132
TRINITY_Contig12279	2	p3	(TCG)4	12	465	476	AGAATGGATCCCCTGGAGCT	60.029	CGATCCCGAGACCGAAGAAG	59.97	132
TRINITY_Contig12280	1	p4	(AATA)3	12	1820	1831	TGGAGACCGAGTTCCAGGAT	59.959	TTGGGTTCACGGTGATGAGG	59.964	100
TRINITY_Contig12285	1	p2	(CG)6	12	227	238	CAGCTGCTCCTGGATCATCC	60.25	CGTACGCTCGTATTCCCTCG	60.384	211
TRINITY_Contig12286	1	p4	(GGCC)3	12	17	28	GTCAGGGGATCCAGTTGTGG	60.035	CTCCGGGATCAATCAGGACG	59.967	244
TRINITY_Contig12286	2	p3	(CGG)6	18	242	259	GTCAGGGGATCCAGTTGTGG	60.035	CTCCGGGATCAATCAGGACG	59.967	244
TRINITY_Contig12286	3	p5	(GCCCT)3	15	948	962	GTCAGGGGATCCAGTTGTGG	60.035	CTCCGGGATCAATCAGGACG	59.967	244
TRINITY_Contig12286	4	p5	(TTTTC)4	20	1102	1121	GTCAGGGGATCCAGTTGTGG	60.035	CTCCGGGATCAATCAGGACG	59.967	244
TRINITY_Contig12287	1	p3	(GCG)4	12	220	231	TGTTGCCAGTGTCCAGTGTT	60.035	CCATGTTGTGCTTGCGTCAA	59.969	185
TRINITY_Contig12288	1	p4	(CCGG)4	16	457	472	GTCATCCTCTCCGTTCCACG	60.179	GTTGCTTTTGTTCCGGGCAT	59.967	106
TRINITY_Contig12291	1	p3	(CGT)4	12	120	131	TACAAGTGGTGGAACGAGGC	59.966	GATGTTCACGTTCGGGGACT	60.038	234
TRINITY_Contig12295	1	p5	(AGACG)3	15	2073	2087	GAGGATTATCCGAGCTCGCC	60.109	GTCTCGTCTGCTTGGACACA	59.968	173
TRINITY_Contig12296	1	p6	(GGCGGA)3	18	29	46	GCCATCTTGTGTGGGATGA	60.034	TCCTGACCGATTGAACCAGC	60.036	269
TRINITY_Contig12300	1	p5	(GGATC)3	15	60	74	CAGCGAAACGTCTGCACAAA	59.973	CGAGGATGGAGATCACGGTG	59.969	253
TRINITY_Contig12303	1	p3	(CGT)4	12	447	458	TACTGCTCTCCTCATGGGCT	60.032	GCAGAAGCTGGAGGTGGAAT	60.035	235
TRINITY_Contig12306	1	c	(CGGCA)3cgccgcccgcgtcgcgtgaccgcgcgcagcgctccgctctccttcctcctggttctctccatctccatctctcccaccccctccg(CGCC)3	113	21	133	TCGCCGTGATGGATCATCTG	59.967	AGAGCAGTCGCAAGCTTCTT	59.965	100
TRINITY_Contig12308	1	p6	(AAGGCC)4	24	384	407	TCCTGAGGAACATGCGCTTT	59.963	GGCTTAACCTCCTTGGGCTT	59.96	133
TRINITY_Contig12309	1	p3	(CGC)4	12	1008	1019	TCGTTCTGGTGGTCGTTGAG	59.968	TGGGGAAGAAATGGTCGTCG	60.037	247
TRINITY_Contig12310	1	p3	(ACC)4	12	214	225	TCTCAAATCCAACGCACCGA	59.966	TGGAGCGCGAGAAACTAAGG	60.109	220
TRINITY_Contig12310	2	p3	(CCG)4	12	377	388	TCTCAAATCCAACGCACCGA	59.966	TGGAGCGCGAGAAACTAAGG	60.109	220
TRINITY_Contig12311	1	p3	(GGA)4	12	357	368	AGAGCAGCGAGGGCATTATC	59.965	TTACCTAGGGCCGATGTGGA	60.032	126
TRINITY_Contig12312	1	p3	(CGC)6	18	53	70	CCGCAAGAAGCTACCACAGA	60.037	AGGCCATTGCAGGAATCCAA	59.958	186
TRINITY_Contig12313	1	p3	(CGC)5	15	223	237	TCGCCTTCACCACCTTCTTC	59.965	GTCGCGGTTGATGAAGAAGC	59.905	212
TRINITY_Contig12313	2	c*	(GGC)3(GGCGACGGCGACGGCGAGTGCGCGCCAGTCGTCGGGCGGAGCGTCCAGTTCCGCCTCGCGTTCACCGGCCTGACCTACAGCGTCCGCGCCAAGCAGCGTGGCGCGCGCCCCGGCCTGCTGCCCCTGAAGCGCCGCTCCGAGCGCGTCACCGCGGCGGCGCCCGACGCGAACGCGCCGCGCACGAGGGCGCTGCTGGACGGCATCTCCGGGGAGGCGAGGGAGGGCGAGATCCTGGCCGTCATGGGCGCCAGCGGCTCCGGCAAGTCTACGCTCATCGACGCGCTCGCCAACCGCATCTCCCGCGACGCGCTCAAGGGCGCCGTCACGCTCAACGGCGAGCCCCTCACGGGGAACGTCCTCAAGTCCATCTCCGCGTACGTCATGCAGGACGACCTGCTGTTCCCCATGCTCACCGTCACCGAGACGCTCTCC	26	414	439	TCGCCTTCACCACCTTCTTC	59.965	GTCGCGGTTGATGAAGAAGC	59.905	212
TRINITY_Contig12314	1	p5	(CCCCT)3	15	86	100	TCTCGTGGTTCCCCAACATC	59.676	TGCCAACCACCATTCTCCAA	59.813	235
TRINITY_Contig12314	2	p3	(CAT)4	12	637	648	TCTCGTGGTTCCCCAACATC	59.676	TGCCAACCACCATTCTCCAA	59.813	235
TRINITY_Contig12315	1	p3	(ACT)4	12	17	28	CCTCCATCACCGAGCTCATC	59.967	GTAGTTCACGGCGAGGTAGG	59.9	179
TRINITY_Contig12315	2	p3	(GCC)4	12	156	167	CCTCCATCACCGAGCTCATC	59.967	GTAGTTCACGGCGAGGTAGG	59.9	179
TRINITY_Contig12315	3	p3	(CGA)7	21	296	316	CCTCCATCACCGAGCTCATC	59.967	GTAGTTCACGGCGAGGTAGG	59.9	179
TRINITY_Contig12318	1	p6	(AGCGGC)3	18	2	19	GTGACCACGAGATGCTGACA	60.038	GCCCCAGCTTCCTGTAGTTC	60.393	221
TRINITY_Contig12321	1	p4	(GAGG)3	12	978	989	CACTGTAGCCTGTCCCCTTG	60.036	CTGTGCGGATGAGATAGCGT	59.969	119
TRINITY_Contig12322	1	p3	(TCG)5	15	790	804	TAATCGTCGTCGTCGTCGTC	59.976	TAGTCACCAGCCAGCCATTG	60.035	166
TRINITY_Contig12322	2	p3	(GAA)4	12	1173	1184	TAATCGTCGTCGTCGTCGTC	59.976	TAGTCACCAGCCAGCCATTG	60.035	166
TRINITY_Contig12322	3	p3	(CAC)4	12	1442	1453	TAATCGTCGTCGTCGTCGTC	59.976	TAGTCACCAGCCAGCCATTG	60.035	166
TRINITY_Contig12322	4	p3	(CCG)4	12	1660	1671	TAATCGTCGTCGTCGTCGTC	59.976	TAGTCACCAGCCAGCCATTG	60.035	166
TRINITY_Contig12323	1	p3	(CGG)4	12	994	1005	ACTCACTCTCTTGCAGGCAC	59.965	AGGTTGTGGTCTTGGTTGCA	60.034	128
TRINITY_Contig12331	1	c	(GAT)5(GAG)4	27	1484	1510	GCCTACACTGTGTCACCGAA	59.967	ACACTGGATCTTGCGGGAAG	60.036	117
TRINITY_Contig12332	1	c	(CGG)4caagcccggcgtgtacgggtacgacaccaacttcgccgtggacggcgacgccggcgcgatgcggaaggtggccgcggtccgg(GGC)4	106	1060	1165	GTGAAGGAGTACGTCGGTGG	60.109	TTCCAGTAGGCGTGGTTCAC	59.966	203
TRINITY_Contig12335	1	p4	(GCCA)3	12	109	120	GCTGCCTCCAACTAGTCCTG	60.108	GAATCTCTGGTTGGGTCGGG	60.108	112
TRINITY_Contig12335	2	p6	(GCTCCC)3	18	357	374	GCTGCCTCCAACTAGTCCTG	60.108	GAATCTCTGGTTGGGTCGGG	60.108	112
TRINITY_Contig12336	1	p4	(CTAG)3	12	1306	1317	GTGGAAGAACATGCGGAGGA	60.036	CCCTCTCTGCTCCCTCTCTT	60.032	280
TRINITY_Contig12337	1	p2	(TC)17	34	12	45	TGCGTGCATTGGTGGAAAAG	59.968	CAACAAGCAACCCTTTCCGG	59.968	149
TRINITY_Contig12337	2	c	(GCA)5cgatcgagcggagagctg(CGGC)3	45	198	242	TGCGTGCATTGGTGGAAAAG	59.968	CAACAAGCAACCCTTTCCGG	59.968	149
TRINITY_Contig12338	1	p6	(CCGTCG)3	18	37	54	CCTCCCATCCAGAATCCACG	59.893	TCTCGATTTGGTGGTGTGCA	59.892	141
TRINITY_Contig12343	1	p3	(AGC)5	15	401	415	CCGCCGATACACCACCTTTA	59.824	CCAGACAACATGGTGGACGA	59.965	125
TRINITY_Contig12345	1	p3	(CAG)4	12	345	356	CAGCAGCAGGAGAAGGAAGG	60.393	GGCCATTACACCACACCAGA	59.962	120
TRINITY_Contig12347	1	p3	(CCG)4	12	337	348	GGGGAAAGTCTCTCACCGTG	60.037	CCATCTCCCACCTCTCAGGA	60.032	211
TRINITY_Contig12349	1	p3	(GCC)4	12	95	106	TTGGGCCTTTGAACCAGGTT	60.032	GGCTTCTGATCAAGGGGCTT	60.034	193
TRINITY_Contig12349	2	p3	(AAG)4	12	1364	1375	TTGGGCCTTTGAACCAGGTT	60.032	GGCTTCTGATCAAGGGGCTT	60.034	193
TRINITY_Contig12349	3	p3	(CTG)5	15	2734	2748	TTGGGCCTTTGAACCAGGTT	60.032	GGCTTCTGATCAAGGGGCTT	60.034	193
TRINITY_Contig12351	1	c	(TCC)5ctccctacttaaatgaagcgtccgcaccaccgtttccaccaaactcccctgccc(CCT)4cgcaccaaccaacccaggccacccagccacccсctcgc(GCGA)4	135	304	438	GCAAATCCGTGGCTTGGTTT	59.967	GAAAACATTGCAGCCAGCCA	59.966	213
TRINITY_Contig12351	2	p6	(TGCGCG)3	18	637	654	GCAAATCCGTGGCTTGGTTT	59.967	GAAAACATTGCAGCCAGCCA	59.966	213
TRINITY_Contig12351	3	p4	(GGCT)5	20	1367	1386	GCAAATCCGTGGCTTGGTTT	59.967	GAAAACATTGCAGCCAGCCA	59.966	213
TRINITY_Contig12354	1	p6	(GGCGTC)3	18	164	181	CCTCTCCATGGACGCTTTGT	60.036	GACGCGATGAACTCAATGGC	59.973	254
TRINITY_Contig12358	1	p2	(GA)7	14	6636	6649	CAGGAAGAGTGCTCCTTGGG	60.036	CCTGCTTCTGCCATGGTAA	60.034	212
TRINITY_Contig12362	1	p3	(CGG)5	15	741	755	CTTTCCCTTTCGCTTTCGGC	60.11	TGCGTGAGAGGGAGAAGAGA	59.962	101
TRINITY_Contig12364	1	p4	(GCGA)3	12	407	418	AAGCTTATCCCATCTGCCGC	60.536	ACCCACACCACTGTTCGTTT	60.035	124
TRINITY_Contig12369	1	p4	(GTAT)3	12	244	255	TTTCGGTGTCCATTTCGGCT	60.251	GAGTAGTCTCGCTCATGGGC	59.969	174
TRINITY_Contig12371	1	c	(GTG)5gccgtggtggaggtggaggtggtggacgaggaggacgtggccgcggtggcgccgccg(CGC)4	85	76	160	ACCATGATGATCGGTCGAGC	59.967	CAGCTAAGGAAGGCCACACA	59.963	185
TRINITY_Contig12371	2	c	(GGT)4cgctc(CCG)6aggagacactggagtggtg(GCT)4	66	536	601	ACCATGATGATCGGTCGAGC	59.967	CAGCTAAGGAAGGCCACACA	59.963	185
TRINITY_Contig12371	3	c	(GAGG)3gaagggctcaatcagtgtgtgcaggaggaggagacatggacggcgcccaccagcgaaagcgaggagggctccagcacgc(AATGG)3	106	1066	1171	ACCATGATGATCGGTCGAGC	59.967	CAGCTAAGGAAGGCCACACA	59.963	185
TRINITY_Contig12373	1	p5	(CCGCG)3	15	121	135	AGGGAGCATTCCGGTTTCTG	60.035	TAGGTTTGTTCCACCTCGCC	59.964	129
TRINITY_Contig12373	2	p3	(CCG)7	21	382	402	AGGGAGCATTCCGGTTTCTG	60.035	TAGGTTTGTTCCACCTCGCC	59.964	129
TRINITY_Contig12378	1	c	(GCGT)3atgaacgggcagtactggtggggcttgtggcggcgcgtgcggtgcggccatccatcacctacggcctcgtcgccgt(CCGG)3	100	256	355	CATCCATCACCTACGGCCTC	59.966	GCAGCGTCCTGTCCAAAAAG	60.04	138
TRINITY_Contig12384	1	p3	(GGC)4	12	267	278	GCGGGCAAGAAAGCAAAGAA	59.968	GAATGGTGAGGAGGTGGGTG	59.969	106
TRINITY_Contig12387	1	p4	(AAGG)3	12	1731	1742	GCGGTTCGGTCTCTACTACG	59.973	ACCTACCTCCTACGGCATGT	60.032	227
TRINITY_Contig12388	1	p3	(TGT)4	12	333	344	GGGAGATTCCACTGGCACAA	59.962	GCCATACAAAGGCTCGTTGC	60.179	274
TRINITY_Contig12390	1	p6	(AGGCAG)4	24	43	66	AATATCACGGTCCATGGGCC	59.89	CATCCCTACCCGTCACGATG	59.968	198
TRINITY_Contig12391	1	p3	(CTC)6	18	1448	1465	ATCATATCGCGAGCAGGCAA	59.966	ACGTCCAGTGTGAATCGCAT	60.037	115
TRINITY_Contig12398	1	p3	(GAC)6	18	46	63	CCTAGTCTTTGGTCTGCCCG	60.108	CCTCTGCTGGCCCATTGTTA	60.034	107
TRINITY_Contig12399	1	p3	(CGG)4	12	788	799	TCCATACTGCTGGTCGAGGA	60.034	TGTGGATGTGCCTGTCCATC	60.035	259
TRINITY_Contig12399	2	p6	(TGGGCG)3	18	1820	1837	TCCATACTGCTGGTCGAGGA	60.034	TGTGGATGTGCCTGTCCATC	60.035	259
TRINITY_Contig12400	1	p5	(CCTTG)4	20	11	30	CGATTCATCTTCGGCGAGGA	59.969	GCATTAAGGCGGCATCCAAG	59.969	262
TRINITY_Contig12400	2	p3	(GCA)4	12	900	911	CGATTCATCTTCGGCGAGGA	59.969	GCATTAAGGCGGCATCCAAG	59.969	262
TRINITY_Contig12400	3	p4	(GTAG)3	12	5412	5423	CGATTCATCTTCGGCGAGGA	59.969	GCATTAAGGCGGCATCCAAG	59.969	262

TRINITY_Contig12400	4	p3	(GTC)4	12	5567	5578	CGATTCATCTTCGGCGAGGA	59.969	GCATTAAGGCGGCATCCAAG	59.969	262
TRINITY_Contig12400	5	c	(GCT)4gcggagagaaccgcagcgacgggg acacggacaccaagggggaccccagcagcag cattctcctttctcaatcgca(AGGGGG)3	106	5893	5998	CGATTCATCTTCGGCGAGGA	59.969	GCATTAAGGCGGCATCCAAG	59.969	262
TRINITY_Contig12401	1	p3	(CGG)5	15	383	397	AGAAGACCAAGTCCGTGCAG	59.966	ACACAACACAGAGCACCCAA	60.035	138
TRINITY_Contig12401	2	c	(GGTT)3gggcgcaggtgacgctggaggag gaccgggtgctggtgatgaagggcggcag(C AC)4(CAA)4cgggaagcggaagcgc(GA G)4	116	566	681	AGAAGACCAAGTCCGTGCAG	59.966	ACACAACACAGAGCACCCAA	60.035	138
TRINITY_Contig12404	1	p3	(GCT)8	24	101	124	CCATCTTCAGCCGGATCCTC	59.965	TTCTTGACAGCTACAGGGCG	60.037	266
TRINITY_Contig12408	1	p3	(GCG)4	12	2197	2208	TGTTTCCTTCGACTGGTCCG	59.967	GACAGCCGACCTCAACAGAA	59.966	220
TRINITY_Contig12409	1	p3	(CGC)6	18	37	54	GTTCAACGGTGTCTTGGCAC	59.971	GGGGATCCCCGGGTATTTTC	59.888	104
TRINITY_Contig12409	2	p3	(GAC)5	15	366	380	GTTCAACGGTGTCTTGGCAC	59.971	GGGGATCCCCGGGTATTTTC	59.888	104
TRINITY_Contig12411	1	p5	(CCACC)3	15	6	20	ACCTTTGTGAGCTCGAGGTG	59.966	AAGTCGCCGGTATGCATAGG	59.966	119
TRINITY_Contig12411	2	p4	(CCGC)3	12	201	212	ACCTTTGTGAGCTCGAGGTG	59.966	AAGTCGCCGGTATGCATAGG	59.966	119
TRINITY_Contig12412	1	p2	(AC)6	12	398	409	ACTCCTCCATCCCATCAGCT	60.03	TTCATGTACAGGGGCGACAC	60.037	280
TRINITY_Contig12415	1	c	(TGCGGC)3tgctgccgcggccgcgcggga gggggagcgtgggtctgcggcgcctgcggcg gcgggggctgcggccgctgctgcttgggcttg gggcggtta(TGC)4	123	92	214	GTGATGCTGCGATGTGTTGG	60.179	GCCATTCTCCATGTCAGGCT	60.107	196
TRINITY_Contig12425	1	p3	(GCC)5	15	497	511	TCATCGTCTGCATGCTCGAC	60.526	TGGGTCGTTGGACATGAAGG	59.964	167
TRINITY_Contig12426	1	p3	(GCC)4	12	2879	2890	AGCCTTGGGATCTCCTGCTA	60.031	TGTCCACGGTGTCCTTGATG	59.965	207
TRINITY_Contig12426	2	p3	(CGG)5	15	3294	3308	AGCCTTGGGATCTCCTGCTA	60.031	TGTCCACGGTGTCCTTGATG	59.965	207
TRINITY_Contig12428	1	c	(CGG)4tgcctcctccaattccgtcctcctccct gcta(CCG)5	59	132	190	TGCAATCTGGACCTTTCCCC	59.96	CCTCCATGACGTTAGAGGCC	59.895	175
TRINITY_Contig12429	1	p3	(CGC)4	12	106	117	ATCTCTGCGTCGTGGTGTTT	59.966	GATGCGCGTTCACATCTTCC	59.973	133
TRINITY_Contig12429	2	p4	(ATTG)3	12	3251	3262	ATCTCTGCGTCGTGGTGTTT	59.966	GATGCGCGTTCACATCTTCC	59.973	133
TRINITY_Contig12429	3	p4	(TGGC)4	16	3957	3972	ATCTCTGCGTCGTGGTGTTT	59.966	GATGCGCGTTCACATCTTCC	59.973	133
TRINITY_Contig12431	1	p3	(GGC)4	12	6	17	CGAGAAGTGGTGGTGAGCTT	59.966	GACCTCAGCTCGCTCTACAC	59.9	268
TRINITY_Contig12431	2	p3	(CCG)5	15	328	342	CGAGAAGTGGTGGTGAGCTT	59.966	GACCTCAGCTCGCTCTACAC	59.9	268
TRINITY_Contig12431	3	p3	(TGC)5	15	532	546	CGAGAAGTGGTGGTGAGCTT	59.966	GACCTCAGCTCGCTCTACAC	59.9	268
TRINITY_Contig12436	1	p3	(GGT)4	12	1488	1499	CCTCCACGCTCATCTCCATC	59.967	TCTCCAGCGGGAAGATCTCA	60.033	255
TRINITY_Contig12444	1	p3	(CAG)5	15	901	915	CGCCACTGTTGTTCTCCTCT	59.966	CGATGCCACAGTATACGGCT	59.968	102
TRINITY_Contig12446	1	c	(GGC)4catggcgcccagtcaccgaagacac cgacggcggccgcgacgccgagacacgggag cacgcctgaaccaccggaggagaaggccgat (AGG)4	111	214	324	CCAACCCAGGATACCCAACC	60.034	GTGCTGGGCCAACAATTCAG	60.038	191
TRINITY_Contig12446	2	p3	(GCC)5	15	532	546	CCAACCCAGGATACCCAACC	60.034	GTGCTGGGCCAACAATTCAG	60.038	191
TRINITY_Contig12446	3	c	(GGC)4catggcgcccagtcaccgaagacac cgacggcggccgcgacgccgagacacgggag cacgcctgaaccaccggaggagaaggccgat (AGG)4	111	740	850	CCAACCCAGGATACCCAACC	60.034	GTGCTGGGCCAACAATTCAG	60.038	191
TRINITY_Contig12447	1	p3	(CTC)4	12	122	133	TGGGGAGGAGGCATAATGGA	60.029	AAAAGACCAGCATCCCTCGG	60.035	270
TRINITY_Contig12447	2	c	(CCT)4cgcgcgcgccgccggcctcctcgccg cctcgttcccggcctccgacctccagcagcacg cgctcgccgtcgcgcg(CCT)4(CGC)4	111	491	601	TGGGGAGGAGGCATAATGGA	60.029	AAAAGACCAGCATCCCTCGG	60.035	270
TRINITY_Contig12448	1	p3	(AGG)6	18	1302	1319	CGCCTGCTTCAGTGTTAGGA	60.037	AGTGGCATCCTGAGAAAGCC	60.035	244
TRINITY_Contig12450	1	p3	(GCG)5	15	1806	1820	GGTTTCTCAAGCTCCAGCCT	59.963	GGTGGGCGATTTATAGGCGA	59.966	154
TRINITY_Contig12451	1	p3	(TGC)4	12	575	586	GCCACAGTGCAAGTGTCAAG	59.97	TCGAGTCAAGCAAAGGGTCC	59.965	208
TRINITY_Contig12452	1	c	(GCA)4ggcctaccgcgagcctgcgaccgcg acggcgccggcggcggccacagccgccagcg agggcg(CGT)6	92	291	382	TGGAGGACCAGGATCCAGAG	60.032	CTCCACGTACCACCAGTCAC	60.039	189
TRINITY_Contig12453	1	p4	(TCCG)3	12	94	105	TCATGGACTTTGCCGTGGAA	59.89	GTACCGGTAGAAGCGGATGG	59.968	206
TRINITY_Contig12454	1	p3	(CGC)5	15	625	639	AAGCCATCTCACTGACAGGC	60.036	GATGGTGACGTCCTTGGAGG	60.108	195
TRINITY_Contig12455	1	p3	(GCT)4	12	684	695	AGAACCAGAGCGACGAACTG	60.039	CTCCACTTGCCATCGGACTT	60.036	253
TRINITY_Contig12457	1	c	(GCG)6tccccgcccggcgtcgggacgttgg agcagctccccgtcgacgcctccacctgctcgt gcacgt(GCG)5	97	67	163	TCAGCTAGCCGATGTTGACC	59.824	CGTCATCCAGGACTCGATGG	59.969	206
TRINITY_Contig12457	2	p3	(CCG)4	12	397	408	TCAGCTAGCCGATGTTGACC	59.824	CGTCATCCAGGACTCGATGG	59.969	206
TRINITY_Contig12460	1	p6	(GGCAGC)3	18	152	169	TACACCTCCACGTCCGATCT	60.035	CAAAGCGATACGGCTTGTCG	59.975	253
TRINITY_Contig12460	2	p4	(AAGA)3	12	316	327	TACACCTCCACGTCCGATCT	60.035	CAAAGCGATACGGCTTGTCG	59.975	253
TRINITY_Contig12462	1	p3	(GGT)4	12	82	93	AGTTCGGGGGAGTACACGTA	59.962	TGGGCTACACTTCGGTTGAC	59.966	205
TRINITY_Contig12467	1	p4	(GCTC)3	12	31	42	CAGGTTGCCGTTGCTCATG	59.789	GGAGGCAGGCATTTTGTTGG	60.037	256
TRINITY_Contig12470	1	p3	(GCC)4	12	116	127	GTCCAAGATCGAGAGGAGCG	59.97	TGCTCTTTAGTCGGCCGTTT	59.966	190
TRINITY_Contig12470	2	p3	(GTC)4	12	229	240	GTCCAAGATCGAGAGGAGCG	59.97	TGCTCTTTAGTCGGCCGTTT	59.966	190
TRINITY_Contig12471	1	p4	(GCCG)3	12	104	115	TAGCAAAGGGTCGAGGCCTA	60.325	CGCAGATGATCGATCGAGCT	60.11	111
TRINITY_Contig12472	1	p6	(CCGCGG)3	18	348	365	TGTCCTCTCTCTCCCTGAGC	60.034	AAAACGCTGTTCCGAAACCG	59.972	130
TRINITY_Contig12472	2	p3	(GGC)4	12	511	522	TGTCCTCTCTCTCCCTGAGC	60.034	AAAACGCTGTTCCGAAACCG	59.972	130
TRINITY_Contig12477	1	p3	(GGC)4	12	3033	3044	TCCTAGAGAATGGCTGGCCT	60.031	GCGCTGCTTCTATCTCGACT	59.97	244
TRINITY_Contig12479	1	p3	(GCC)5	15	1541	1555	CGAAGGTGCACAGCATGTTC	60.11	AGATGCTCCTTTGCCAGGTC	60.035	243
TRINITY_Contig12481	1	p4	(ATCG)4	16	11	26	AACTAAGACGGCCACCATGG	60.035	CTGCCCAGGAAGGTCTTCTG	60.036	108
TRINITY_Contig12482	1	p3	(GGC)6	18	782	799	TCTCACTGAGGACGACGAGT	59.965	CTAGCCTCTTCGCCTCCATG	59.967	158
TRINITY_Contig12484	1	p3	(CCG)5	15	296	310	GTCGTGATGATGAAGTGCGC	59.974	CGATACGCTGAGCCAATCCT	59.967	126
TRINITY_Contig12484	2	p3	(CCG)4	12	485	496	GTCGTGATGATGAAGTGCGC	59.974	CGATACGCTGAGCCAATCCT	59.967	126
TRINITY_Contig12486	1	p3	(GCC)4	12	671	682	GCCATCGGCTACCTCTTCTC	59.967	CAAAAATCGTGGTGCCAGCA	59.968	103
TRINITY_Contig12487	1	p4	(GCGA)3	12	114	125	GAAAGTCTCCCAGCCCAGAC	60.036	CCATTTGGTAGGCCGCTCG	61.18	105
TRINITY_Contig12489	1	p3	(GCG)5	15	386	400	TCTTACGGTCCGCAAGAACC	60.038	TTGTACTTGTACGGCCGGTC	60.038	218
TRINITY_Contig12495	1	p4	(ACGC)3	12	89	100	GGAGCCATGGAGAATCGGTT	59.819	TCCACCTTCCTAGCTCCCTC	60.032	180
TRINITY_Contig12504	1	p3	(GCG)5	15	139	153	CGGAACAAATGGCAACCTGG	60.038	GCTGCAGGTAGTCCTTCTGG	60.108	160
TRINITY_Contig12504	2	p3	(CGC)5	15	285	299	CGGAACAAATGGCAACCTGG	60.038	GCTGCAGGTAGTCCTTCTGG	60.108	160
TRINITY_Contig12505	1	p3	(CGC)4	12	119	130	CAGGGCCCTTCTCTTCCAAG	60.035	CACTGCGTTGATGCGAGATG	59.974	260
TRINITY_Contig12511	1	c	(GGCCA)3tcgatgctgagctcggcgtcctc ggcgggggcgggga(TGGGGG)3	70	244	313	ACAAGCTACCCACTGTTCGG	59.965	ATGGAGATGGAGAGCTGGGT	60.03	242
TRINITY_Contig12511	2	p3	(CCG)5	15	464	478	ACAAGCTACCCACTGTTCGG	59.965	ATGGAGATGGAGAGCTGGGT	60.03	242
TRINITY_Contig12513	1	p2	(GA)10	20	401	420	TCTCGACCTTGGGAAAGTGC	59.965	CGTGTCGCTACAGGATCTCC	59.97	224
TRINITY_Contig12515	1	p3	(CCG)4	12	227	238	CGGAGAAGAGCGTCACGAG	60.226	CTCCACTCCACGCTCTTCTC	59.828	127
TRINITY_Contig12517	1	p3	(GGC)4	12	383	394	TTCCTCCAGGAGTTCGGGAA	60.179	ATGGAATTTCTGCCCCCGTT	59.959	247
TRINITY_Contig12517	2	p3	(CGG)4	12	852	863	TTCCTCCAGGAGTTCGGGAA	60.179	ATGGAATTTCTGCCCCCGTT	59.959	247
TRINITY_Contig12518	1	p3	(GCG)4	12	1443	1454	CCAGCTTCCGTTTGCTTCAC	60.04	GCGAGCTCCTCATCCTTCTC	59.968	209
TRINITY_Contig12518	2	p3	(CCA)5	15	1626	1640	CCAGCTTCCGTTTGCTTCAC	60.04	GCGAGCTCCTCATCCTTCTC	59.968	209
TRINITY_Contig12520	1	p3	(TCC)4	12	374	385	ATTGAGCAGGCGTAGGTGTG	60.392	TGGAGGAGGAGGATGGTGAG	60.032	237
TRINITY_Contig12522	1	p3	(GCG)4	12	339	350	GCGCGCTTACTTCTTCTTCG	59.976	GCGCTCAAGTTCTGCAACAA	59.971	105
TRINITY_Contig12526	1	p3	(GAG)4	12	400	411	GTCGGCAGTGTACCCTTGAA	59.966	TGATCTCACGGTAGGTTGCG	59.827	194
TRINITY_Contig12526	2	p3	(ACG)4	12	914	925	GTCGGCAGTGTACCCTTGAA	59.966	TGATCTCACGGTAGGTTGCG	59.827	194
TRINITY_Contig12528	1	p2	(GA)6	12	7	18	TCAAGTGCACGTTCGATGGA	59.967	AGAGCCATACTGGTGTTGCC	60.035	144
TRINITY_Contig12532	1	p5	(AGAGG)3	15	217	231	TGTGACGCCGCCTATGATAC	59.968	TGACCAGTGAGCTTGTCGTC	59.968	152
TRINITY_Contig12532	2	c	(CCCG)3gccgcc(AG)6	30	427	456	TGTGACGCCGCCTATGATAC	59.968	TGACCAGTGAGCTTGTCGTC	59.968	152
TRINITY_Contig12532	3	c	(CGG)4tggcta(CGG)7gtacgggcgccgc gagggcggcggtggcta(CGG)6	87	1054	1140	TGTGACGCCGCCTATGATAC	59.968	TGACCAGTGAGCTTGTCGTC	59.968	152
TRINITY_Contig12532	4	p6	(GGATTT)3	18	1812	1829	TGTGACGCCGCCTATGATAC	59.968	TGACCAGTGAGCTTGTCGTC	59.968	152
TRINITY_Contig12536	1	p2	(TC)16	32	67	98	AGTGCAGGCTGCTTTCTGAT	59.962	CCTTCAGGCGTGGAATGTCT	60.036	187
TRINITY_Contig12536	2	p4	(GCCG)4	16	1145	1160	AGTGCAGGCTGCTTTCTGAT	59.962	CCTTCAGGCGTGGAATGTCT	60.036	187
TRINITY_Contig12541	1	p4	(CCGC)3	12	47	58	TCATGGACTCAGGGAGGGAG	60.032	TTCTTGGTGGAAGGAGCGTC	59.965	258
TRINITY_Contig12550	1	p3	(GCC)4	12	2949	2960	ACAAAGGTTCGGATCGGCTT	59.964	TGTTGCCATGGTGTGCAATG	59.966	183
TRINITY_Contig12550	2	p3	(CCT)4	12	3070	3081	ACAAAGGTTCGGATCGGCTT	59.964	TGTTGCCATGGTGTGCAATG	59.966	183
TRINITY_Contig12550	3	p4	(CCCT)3	12	3455	3466	ACAAAGGTTCGGATCGGCTT	59.964	TGTTGCCATGGTGTGCAATG	59.966	183
TRINITY_Contig12553	1	p3	(GGT)4	12	305	316	ATCCGGATGCTTCATGACGG	60.25	CTTATCCGATGGCGTCCCTC	60.038	278
TRINITY_Contig12554	1	p2	(CT)7	14	54	67	TTGCTCGATTGAAGGGCCAT	60.034	TGGTCGATCGCACACTCAAA	59.967	175
TRINITY_Contig12558	1	p3	(GCT)4	12	625	636	GATAAGGCACGGGGTCTGAG	59.895	CCCTTCTTTGCTCCGTCCAT	60.035	131
TRINITY_Contig12559	1	p3	(GGA)4	12	345	356	AATGCCTTCCCTGCTGGTAC	60.034	TTCAAGCAATGCAGCCACAC	59.968	280
TRINITY_Contig12561	1	p3	(CTG)4	12	171	182	AGCTGCGTCCATCGACATAG	59.969	ATGTTGTTGTGGAAACGCCG	59.97	138
TRINITY_Contig12561	2	p6	(CCTCCA)3	18	339	356	AGCTGCGTCCATCGACATAG	59.969	ATGTTGTTGTGGAAACGCCG	59.97	138
TRINITY_Contig12561	3	p3	(CCT)4	12	728	739	AGCTGCGTCCATCGACATAG	59.969	ATGTTGTTGTGGAAACGCCG	59.97	138

TRINITY_Contig12561	4	p3	(CCG)5	15	1102	1116	AGCTGCGTCCATCGACATAG	59.969	ATGTTGTTGTGGAAACGCCG	59.97	138
TRINITY_Contig12565	1	p5	(CCGGC)3	15	1633	1647	GGCATGCACGATGGGATTTC	59.969	TGCGCATTTTTCTGCTGCTT	59.968	115
TRINITY_Contig12566	1	c	(TCCTCG)3atggccgagtcatcgatgaagttgtcctcgcgcgaccgcttccgccca(CCG)4cccctt(GCC)4gcgggaggccttcccgcgcctggcctcctcctcgtagtcgtcctcgtca(TCC)4tcgtccaggtcgtaggcctcctcctcg(TCC)6acc(TCG)6	222	41	262	GTCATCCTCCTCCTCCTCGT	60.106	AGTTTCCCCTTTCCTCTGCG	59.963	174
TRINITY_Contig12568	1	p3	(CCG)4	12	79	90	CCAACTCGTGGCTCTTCTCC	60.39	GCAGATGTACCAGATGGCGA	59.895	125
TRINITY_Contig12568	2	p3	(GCC)4	12	430	441	CCAACTCGTGGCTCTTCTCC	60.39	GCAGATGTACCAGATGGCGA	59.895	125
TRINITY_Contig12568	3	p3	(GCG)6	18	549	566	CCAACTCGTGGCTCTTCTCC	60.39	GCAGATGTACCAGATGGCGA	59.895	125
TRINITY_Contig12568	4	c	(GCC)6acgaagcagcag(GCC)4	42	720	761	CCAACTCGTGGCTCTTCTCC	60.39	GCAGATGTACCAGATGGCGA	59.895	125
TRINITY_Contig12568	5	c	(AGC)7tagccgtagtgacagcagcaatacactgatactagctagtgtggtaac(CTA)4ggatc(AT)7(AG)8	116	871	986	CCAACTCGTGGCTCTTCTCC	60.39	GCAGATGTACCAGATGGCGA	59.895	125
TRINITY_Contig12570	1	p4	(CACG)3	12	633	644	TCGAAGGGGGTTCTTTGCAA	59.816	CGGAATCGACAGCACTACGA	59.902	237
TRINITY_Contig12571	1	p3	(GCG)6	18	166	183	AGCAGCCAAGGCAAGAAGAT	59.961	TCGGGAGTGGTCATCAGAGT	59.96	191
TRINITY_Contig12580	1	p3	(CGC)4	12	334	345	TTCATCAGTTCGCGCTCCTT	60.037	TGCATCGGATCGTCTCTTCG	59.971	195
TRINITY_Contig12582	1	p3	(CCG)5	15	74	88	TAGTCAGGACCAGGTGGAGG	59.959	CCTTCCTCTGCCTCACCAAG	60.036	172
TRINITY_Contig12583	1	p5	(CGCTA)3	15	57	71	CGCTACGCTACGCTACAGAA	59.973	AAGAACGGATCTGGAGGGA	59.957	155
TRINITY_Contig12584	1	p3	(GGA)5	15	167	181	CTTGGCACGTTTCCAACAGG	59.969	GCAGTATTGGTGGGCAGAGT	60.035	142
TRINITY_Contig12587	1	c	(CGG)4gcgcgcgagacggaaccccttcgcggcga(GCGG)3	53	1579	1631	TATGTTGGACAAGCGCCCAT	60.035	ACATCCTCCATCTCTGGCCT	60.03	123
TRINITY_Contig12587	2	p2	(GA)10	20	1763	1782	TATGTTGGACAAGCGCCCAT	60.035	ACATCCTCCATCTCTGGCCT	60.03	123
TRINITY_Contig12593	1	p3	(CAC)4	12	502	513	ATCCCAGAAAGAGCAGTGGC	60.035	TCCTCACAGGAGCTTGCTTG	59.964	112
TRINITY_Contig12595	1	p3	(GGC)7	21	123	143	GATTCGATTCACTTGGCGGC	59.972	GGTCCTGCCTCTTCTGGTTC	60.036	205
TRINITY_Contig12597	1	p3	(GTC)5	15	108	122	CTTCTTGCCGTCCTTCTGGT	59.965	ATGCCCCACGACAAGAAGAG	60.036	123
TRINITY_Contig12600	1	p3	(TGG)4	12	210	221	GAGGAGACGAGAGATGCTGC	59.969	AAGAAGCTCATGCATGGCCT	60.033	151
TRINITY_Contig12600	2	p3	(CGG)4	12	327	338	GAGGAGACGAGAGATGCTGC	59.969	AAGAAGCTCATGCATGGCCT	60.033	151
TRINITY_Contig12600	3	c	(TGC)5tggtgacgcggtgacgaacaaga(GCT)4	50	454	503	GAGGAGACGAGAGATGCTGC	59.969	AAGAAGCTCATGCATGGCCT	60.033	151
TRINITY_Contig12609	1	p3	(GGC)4	12	324	335	TTACCGGCTCTAGCTCTGGT	60.033	AATGTGGAGTTGAGGGCGAG	60.036	146
TRINITY_Contig12610	1	p3	(GCC)4	12	91	102	CCTCCACCCATTCCCTCCTA	60.03	CCGAGAACGAGAAGGAGCAA	59.756	172
TRINITY_Contig12618	1	p4	(CACC)3	12	187	198	ATTGCAGCCAATCCGTCTCA	60.035	TTTCTTTGCAGCGCTGTGAC	59.971	188
TRINITY_Contig12618	2	p3	(GCG)4	12	504	515	ATTGCAGCCAATCCGTCTCA	60.035	TTTCTTTGCAGCGCTGTGAC	59.971	188
TRINITY_Contig12620	1	c	(CT)6gcttctctcgtcgccg(ACGC)3agccgtcgcctccgccgccccgcgcccccgcctccgcgggccgagtcggctgccatggcggccacggcagatccgcgtgcgaagcccctggcgg(CGC)4	146	16	161	CTCCAAGGAAGGAAGCTGGG	60.035	GAGACAGAACTTGGGAGGGC	60.036	163
TRINITY_Contig12620	2	c	(CCG)5aagaccgccgcgagcccgccgccgagacggact(CCGAGC)3tccgtgcg(CCT)5	90	449	538	CTCCAAGGAAGGAAGCTGGG	60.035	GAGACAGAACTTGGGAGGGC	60.036	163
TRINITY_Contig12620	3	p3	(TCG)4	12	665	676	CTCCAAGGAAGGAAGCTGGG	60.035	GAGACAGAACTTGGGAGGGC	60.036	163
TRINITY_Contig12620	4	c	(CCCT)3cgtccctcacctcgccatctggtgttgtacgtgtgctccattcccgcgcctgcctgtacatacagttgtgtgctttgtggtta(CTG)4	107	1125	1231	CTCCAAGGAAGGAAGCTGGG	60.035	GAGACAGAACTTGGGAGGGC	60.036	163
TRINITY_Contig12621	1	p3	(GAT)4	12	473	484	TGGAAGAAATGGGCGGAGAC	60.035	AGCAAGAACCAGCATCCCTC	60.035	218
TRINITY_Contig12622	1	p4	(ACCC)3	12	130	141	TTCTTCAGCCCGCTCTTCTC	59.752	AGAAGGTTCTTGCGTGCGTA	59.967	150
TRINITY_Contig12622	2	p3	(CCG)6	18	456	473	TTCTTCAGCCCGCTCTTCTC	59.752	AGAAGGTTCTTGCGTGCGTA	59.967	150
TRINITY_Contig12624	1	p3	(ACC)4	12	24	35	AAGGTTGATGCCACTCGGAG	60.036	AGCGGATCCAACGACACAAT	60.036	140
TRINITY_Contig12624	2	p3	(GCT)6	18	967	984	AAGGTTGATGCCACTCGGAG	60.036	AGCGGATCCAACGACACAAT	60.036	140
TRINITY_Contig12637	1	p3	(GGC)4	12	293	304	TCACGGAAAAGGGAGATGCC	60.035	CTCCGTTCGTATTCCCCTGG	59.896	158
TRINITY_Contig12638	1	p3	(GCT)4	12	112	123	TGCTCAACGTCGTCGACTAG	59.835	AAAAATCCGGCATGTGTGGC	60.037	142
TRINITY_Contig12638	2	p4	(GCCG)4	16	263	278	TGCTCAACGTCGTCGACTAG	59.835	AAAAATCCGGCATGTGTGGC	60.037	142
TRINITY_Contig12639	1	c	(AGAT)3tattcgtcgcaggc(GACGG)3	41	121	161	TACGAAACGATAGACCGCGG	59.972	GCGGGATAGGCTAACCCATC	60.036	272
TRINITY_Contig12639	2	p5	(CCTCT)3	15	294	308	TACGAAACGATAGACCGCGG	59.972	GCGGGATAGGCTAACCCATC	60.036	272
TRINITY_Contig12645	1	p3	(GGC)5	15	289	303	TAGTTACCCGAGAGACCCGG	60.107	CCTCTCCTCATCCTCCACGA	60.106	150
TRINITY_Contig12652	1	p6	(CCCCGA)3	18	13	30	TCCATTCTTCCCTGCTGCAG	60.035	CGGCGGGTAATGTAGCTCAT	59.966	125
TRINITY_Contig12652	2	p4	(TGCG)3	12	384	395	TCCATTCTTCCCTGCTGCAG	60.035	CGGCGGGTAATGTAGCTCAT	59.966	125
TRINITY_Contig12653	1	p3	(GTC)4	12	396	407	CTGCTCCAAGTCCAACGTCT	59.966	AGGAGAAGGAGTAGCCCAGG	60.032	239
TRINITY_Contig12659	1	p3	(CCG)4	12	551	562	CTCGTCGGTGGTACTACTGC	59.902	TTGTCCGCCTCCTTCTTGTC	59.965	257
TRINITY_Contig12662	1	p3	(CCG)4	12	27	38	GAGGGAGGGGACAGGGAG	60.041	CCTGGCAAAGGCGAATGATG	59.898	137
TRINITY_Contig12669	1	c	(GCG)4cctggaggaggcggaggacgacgccgaggtg(GGC)4atcgggagacgagcccggggctccggtgggctagggtacggggggaa(CGCGG)3	117	401	517	AATCTCACTGTTCGCCGAGG	60.109	CATCGAGATCCTCATGGGGC	60.037	239
TRINITY_Contig12669	2	p3	(GGA)4	12	657	668	AATCTCACTGTTCGCCGAGG	60.109	CATCGAGATCCTCATGGGGC	60.037	239
TRINITY_Contig12676	1	p2	(CT)6	12	211	222	TGAGCCATCTCCAACGAACC	60.036	TGTCTACGCCAGAGGAGGAA	59.961	133
TRINITY_Contig12676	2	p6	(AACCCT)3	18	362	379	TGAGCCATCTCCAACGAACC	60.036	TGTCTACGCCAGAGGAGGAA	59.961	133
TRINITY_Contig12677	1	p3	(CCT)4	12	403	414	ATCTTCGACGTCGTACTCGC	59.974	CCGAAGTCAGCCTTCCACTT	59.965	165
TRINITY_Contig12678	1	c	(CCTCC)5tctccagcgccaaaacctcccccggccggcgaattccccacgcgccgcctccagcccgatcggatcctcgggggcacccc(CG)6	118	144	261	GATGGAGAGGTACGAGCTGC	59.969	TGAGCACGACCTCCTTGAAC	59.966	206
TRINITY_Contig12695	1	p4	(GACA)3	12	3351	3362	GCCTTCTTCATCCCCGAACA	60.035	GAGTCGGTGCTGTACAACCA	59.967	198
TRINITY_Contig12696	1	p3	(CTT)4	12	689	700	AAGGTTCTGTCGGCATCCAG	60.036	AGCACCCTCAGGCTCAAATC	60.035	181
TRINITY_Contig12697	1	p4	(TCTT)3	12	875	886	GATCCGATCCACTCTGCCAG	59.967	CGAGGAGATCTGGCAGAACC	59.896	131
TRINITY_Contig12698	1	c	(GAA)8gacgaagatcgccggtggccgtctctcgtcggggtgtggtgacgaggacgacgggaccggacgggagaggagagtcagtagc(GA)11	128	3336	3463	AATGCAAAGCCTTGGCCATG	60.036	ACTGACTCTCCTCTCCCGTC	60.035	241
TRINITY_Contig12699	1	p3	(CCG)5	15	423	437	TGAATGAGATGGAGGCGGTG	59.821	GGGACGAAGCCGAAAAATCG	59.904	139
TRINITY_Contig12699	2	c	(GGGAG)3tgagcttcgtggaagcttcgtggggcgccgatttttcggcttcgtcccgtcccctccggttggttgttacta(CT)11	109	984	1092	TGAATGAGATGGAGGCGGTG	59.821	GGGACGAAGCCGAAAAATCG	59.904	139
TRINITY_Contig12700	1	p3	(CGG)6	18	1871	1888	ACCATTCTTAAGGCGCGACA	60.037	CTTCTTGCCGAGGTGGTTCT	59.965	215
TRINITY_Contig12701	1	p2	(TC)7	14	95	108	CGTGAGCGCCAGATTATCCT	59.967	CCCTCGATTCGCTCTTCTCC	59.969	122
TRINITY_Contig12701	2	p3	(GGC)4	12	1165	1176	CGTGAGCGCCAGATTATCCT	59.967	CCCTCGATTCGCTCTTCTCC	59.969	122
TRINITY_Contig12701	3	p3	(GCC)4	12	1625	1636	CGTGAGCGCCAGATTATCCT	59.967	CCCTCGATTCGCTCTTCTCC	59.969	122
TRINITY_Contig12702	1	p4	(CACG)3	12	2093	2104	GGCGGGTTTTTCGTACAACC	60.04	TCATGGCTTTCCTTCCGGTC	60.035	157
TRINITY_Contig12705	1	p3	(GAA)4	12	1755	1766	CTCCTTCAGGGCCACAAGAG	60.036	AGCTCAACTGCTGGAGGAAC	59.964	197
TRINITY_Contig12706	1	p4	(GCGG)4	16	228	243	ACCAAAGCTCATTCCTCGGG	60.035	GCCACTGAGAGGAGTCCAAC	60.037	227
TRINITY_Contig12708	1	p6	(CGCAAA)3	18	13	30	TGCCCCAATGTCATCGTTGA	59.963	GCGACCATTGCCATAGCTTG	59.97	184
TRINITY_Contig12709	1	p3	(CGC)5	15	1627	1641	GCAGCTGAAAATCTCGAGCG	59.974	CTGCTGTCCTATTGGCACCA	60.035	101
TRINITY_Contig12710	1	p3	(CGC)6	18	1586	1603	AAGGATCTCCAGCAACTGCC	60.035	CAAAGTATGCGGCGTCTTCG	59.975	186
TRINITY_Contig12711	1	p3	(CGC)5	15	1603	1617	GCAGCTGAAAATCTCGAGCG	59.974	CTGCTGTCCTATTGGCACCA	60.035	101
TRINITY_Contig12712	1	p3	(TAT)4	12	2959	2970	CAACAGGTGCTGCAAGTTCC	59.969	TGCTGTGTGCTGTCTCTCTG	59.967	254
TRINITY_Contig12713	1	p3	(CTA)4	12	340	351	CGCTAGTAGCTCCAGAGTGC	59.97	ATCGTACTACGCGAGCTTGG	59.971	123
TRINITY_Contig12713	2	p3	(CGG)5	15	1083	1097	CGCTAGTAGCTCCAGAGTGC	59.97	ATCGTACTACGCGAGCTTGG	59.971	123
TRINITY_Contig12714	1	p3	(CCA)5	15	1147	1161	TCCTGGGTATCAAGGCGGTA	60.032	AGTTCGAGTTGCCTCCTTGG	59.965	236
TRINITY_Contig12716	1	p4	(TTCC)3	12	14	25	TTCAGCTCCGGACAAGGTTC	59.965	GCGTTTGCTTAGCATCGGAG	59.973	113
TRINITY_Contig12716	2	p4	(CCCG)3	12	155	166	TTCAGCTCCGGACAAGGTTC	59.965	GCGTTTGCTTAGCATCGGAG	59.973	113
TRINITY_Contig12716	3	p4	(TAGC)3	12	1774	1785	TTCAGCTCCGGACAAGGTTC	59.965	GCGTTTGCTTAGCATCGGAG	59.973	113
TRINITY_Contig12717	1	p5	(GAGGC)3	15	5124	5138	CCATAGTCAGACGCCCCATC	59.966	TCATATGCACCCTGGGAGGA	60.03	101
TRINITY_Contig12721	1	p3	(GAG)4	12	375	386	ACTGCTCCAAACTCGCATCA	59.965	CAGGTGCCCCATCAATTCCT	60.033	188
TRINITY_Contig12721	2	p3	(CGC)4	12	1163	1174	ACTGCTCCAAACTCGCATCA	59.965	CAGGTGCCCCATCAATTCCT	60.033	188
TRINITY_Contig12723	1	p3	(CGA)4	12	470	481	GCCACCTAATCACGCCATCT	60.179	GGATGTTGGAAGGGTCCCTG	60.034	126
TRINITY_Contig12724	1	p3	(AGG)5	15	2448	2462	TTAGTAGCAGGTGCAGCCAC	60.036	ACTCCTTCCTCTCGGGTCTC	60.034	194
TRINITY_Contig12725	1	p3	(CCT)4	12	163	174	ACTCCTTCCTCTCGGGTCTC	60.034	TTAGTAGCAGGTGCAGCCAC	60.036	194
TRINITY_Contig12727	1	p2	(GA)7	14	6537	6550	CAGGAAGAGTGCTCCTTGGG	60.036	CCTGCTTCTGCCATGGGTAA	60.034	212
TRINITY_Contig12728	1	p2	(TC)7	14	87	100	CCTGCTTCTGCCATGGGTAA	60.034	CAGGAAGAGTGCTCCTTGGG	60.036	212
TRINITY_Contig12729	1	p3	(GGA)4	12	1932	1943	GCTTGGTCTCCCAGATGCTT	60.035	TGACGAGGCTGTGCTTGTAG	60.038	232
TRINITY_Contig12730	1	p3	(GCC)5	15	104	118	GCTCGTACTACCTGCTCGTC	59.972	GGAACAGGTCCGTGTGGTAG	60.038	141
TRINITY_Contig12731	1	p3	(CCG)4	12	238	249	TTTGCCTGACGATCACTGCT	59.965	AACTTCATCCTGCCCATGGG	60.033	171

TRINITY_Contig12731	2	p3	(CTC)6	18	500	517	TTTGCCTGACGATCACTGCT	59.965	AACTTCATCCTGCCCATGGG	60.033	171
TRINITY_Contig12732	1	c	(CAG)5cgagaaagccaaatctctcgtccagcgaggagcgatcgagaagaagaggaggtgagcagcagcgatggcgaggtctggcaagatgat(GGC)4	114	113	226	GTTGACAAGGCAAACCGGAC	59.97	GGATGGGTCGTCAACTCGTT	60.038	108
TRINITY_Contig12732	2	p3	(CGG)5	15	350	364	GTTGACAAGGCAAACCGGAC	59.97	GGATGGGTCGTCAACTCGTT	60.038	108
TRINITY_Contig12733	1	p4	(ACTC)3	12	156	167	TTGCCGTAGTCCTCGAACAC	60.039	TGCTCGTCAAGGACAAGGAC	59.966	169
TRINITY_Contig12733	2	p3	(CGC)5	15	864	878	TTGCCGTAGTCCTCGAACAC	60.039	TGCTCGTCAAGGACAAGGAC	59.966	169
TRINITY_Contig12734	1	p3	(CGC)4	12	1	12	AACTGATGCCGCTTTCAGGA	59.963	TGGCCCATCTAGAGGAAGCT	60.031	127
TRINITY_Contig12735	1	p3	(CCG)4	12	1698	1709	CCTTACAGTGGTGTGGGTCC	59.964	GCAGAAGGTGACGGGTTACA	59.966	224
TRINITY_Contig12735	2	c*	(GGCG)2(<GGCG>GCGGCGGCGCGGGCTCCTAGGGTTTGGTGCTCTGGGTCCGGAGGGGGGGGGGGGAGAGGAATGGGGAACGGATCCAGGCGGTTATGTAGCGGAGGAGGGCTGGAATGGCGGAGGCGGGGGAGGCCGCCAAAACGGGATTTCGAAATGGAGCGGAGGGAGGGAGGGAGGGAGCGGGGCGGCGGTTCGCTGGGCGCTCCGGCGGCGGGAGCGGCGCACTTCCCGATCTGAAACGCGGAGGCGGGCGGTGGTGGGCGGGGAGCTTGCCTTTT)(GGC)3	20	1815	1834	CCTTACAGTGGTGTGGGTCC	59.964	GCAGAAGGTGACGGGTTACA	59.966	224
TRINITY_Contig12735	3	p4	(GGAG)5	20	1979	1998	CCTTACAGTGGTGTGGGTCC	59.964	GCAGAAGGTGACGGGTTACA	59.966	224
TRINITY_Contig12737	1	p3	(GCG)4	12	746	757	CTGCTACTGCGACTACTCCG	59.972	CGTTGCGCTTGATGTACTCG	59.976	107
TRINITY_Contig12737	2	p3	(CGG)5	15	1145	1159	CTGCTACTGCGACTACTCCG	59.972	CGTTGCGCTTGATGTACTCG	59.976	107
TRINITY_Contig12739	1	p3	(CGC)4	12	604	615	TCTCCTCCAACGCCTTCAAC	59.965	GGAGAGGTTGTTGAAGCGGA	59.965	167
TRINITY_Contig12739	2	p3	(CCG)4	12	816	827	TCTCCTCCAACGCCTTCAAC	59.965	GGAGAGGTTGTTGAAGCGGA	59.965	167
TRINITY_Contig12739	3	p3	(CGC)6	18	1282	1299	TCTCCTCCAACGCCTTCAAC	59.965	GGAGAGGTTGTTGAAGCGGA	59.965	167
TRINITY_Contig12739	4	p3	(GTC)4	12	1511	1522	TCTCCTCCAACGCCTTCAAC	59.965	GGAGAGGTTGTTGAAGCGGA	59.965	167
TRINITY_Contig12739	5	p3	(AGG)4	12	2792	2803	TCTCCTCCAACGCCTTCAAC	59.965	GGAGAGGTTGTTGAAGCGGA	59.965	167
TRINITY_Contig12739	6	p3	(GAC)4	12	4074	4085	TCTCCTCCAACGCCTTCAAC	59.965	GGAGAGGTTGTTGAAGCGGA	59.965	167
TRINITY_Contig12739	7	p3	(CGG)4	12	4769	4780	TCTCCTCCAACGCCTTCAAC	59.965	GGAGAGGTTGTTGAAGCGGA	59.965	167
TRINITY_Contig12742	1	p4	(GCTT)3	12	207	218	TGTCAGGTCGTGCTTGAGTC	59.968	TGTCGTGTGTGCTTCTACCC	59.967	107
TRINITY_Contig12742	2	p4	(TGCT)4	16	388	403	TGTCAGGTCGTGCTTGAGTC	59.968	TGTCGTGTGTGCTTCTACCC	59.967	107
TRINITY_Contig12742	3	p3	(CGC)4	12	720	731	TGTCAGGTCGTGCTTGAGTC	59.968	TGTCGTGTGTGCTTCTACCC	59.967	107
TRINITY_Contig12743	1	p3	(GGT)4	12	327	338	ATGTGCATAAGGGGGCAGCTT	60.033	ATGTGCAATCGGAAGCTCCA	60.035	206
TRINITY_Contig12744	1	p3	(GGT)4	12	321	332	CAGCTTAGCCGGCCTAGTAC	59.968	AGAAGAAATGGGCTGGACGG	60.035	206
TRINITY_Contig12744	2	p4	(AAAT)4	16	1230	1245	CAGCTTAGCCGGCCTAGTAC	59.968	AGAAGAAATGGGCTGGACGG	60.035	206
TRINITY_Contig12744	3	p4	(CGGC)3	12	1457	1468	CAGCTTAGCCGGCCTAGTAC	59.968	AGAAGAAATGGGCTGGACGG	60.035	206
TRINITY_Contig12744	4	p3	(TTC)5	15	1883	1897	CAGCTTAGCCGGCCTAGTAC	59.968	AGAAGAAATGGGCTGGACGG	60.035	206
TRINITY_Contig12744	5	p4	(ATCA)3	12	2546	2557	CAGCTTAGCCGGCCTAGTAC	59.968	AGAAGAAATGGGCTGGACGG	60.035	206
TRINITY_Contig12745	1	p3	(GGT)4	12	321	332	TCGCATATCGAGGACGCAAA	59.898	CCCGCTTCATCATCTCCCTC	59.965	138
TRINITY_Contig12746	1	c	(GCC)5ggtttagtggcgacggcggtcgccgaggccgccgcccgcggggcga(GGGAGC)3	79	1318	1396	TGACCATCAGTTCCGTGCTC	60.037	GTGGATCTCGTGAGTAGCGG	59.97	199
TRINITY_Contig12747	1	c	(CGCC)3gcccatgccggctccgccgccgacgttgttgtccaggccgagggagagccccggcgaggacgagaacccggcggagaacgggaactgcatcccg(CCGCCT)3	124	10	133	GAATCGGGTCTGTGGAAGCA	60.036	CCGTTTGTGTTTGGTCGGAC	59.972	109
TRINITY_Contig12749	1	p3	(CGG)4	12	2531	2542	ACTGGAGCCTCTGCAACTTC	59.964	CGTCCCCAAGCGAGAGATAC	59.969	123
TRINITY_Contig12750	1	p3	(AGC)5	15	569	583	GAGGACGACAGCAACTGACA	59.968	AGCTCATAACCGGTTGCCAA	59.962	156
TRINITY_Contig12750	2	p3	(GGA)4	12	849	860	GAGGACGACAGCAACTGACA	59.968	AGCTCATAACCGGTTGCCAA	59.962	156
TRINITY_Contig12751	1	p3	(CCT)4	12	140	151	CGACAACAACCTCGAGACCA	59.968	ATGACGCCGAGGATGATCAC	59.967	114
TRINITY_Contig12751	2	p3	(GCT)4	12	296	307	CGACAACAACCTCGAGACCA	59.968	ATGACGCCGAGGATGATCAC	59.967	114
TRINITY_Contig12751	3	p3	(GTC)4	12	2339	2350	CGACAACAACCTCGAGACCA	59.968	ATGACGCCGAGGATGATCAC	59.967	114
TRINITY_Contig12752	1	c	(CCT)4ccatgccactagcactagatctaccct(GCCACC)3atatctatcccttcccggaaacattctgcacgcggcagctagcagctgcagaaatggctacttcccctcctgccctcctaaca(CTG)6	158	140	297	CGACAACAACCTCGAGACCA	59.968	ATGACGCCGAGGATGATCAC	59.967	114
TRINITY_Contig12752	2	p3	(GTC)4	12	2343	2354	CGACAACAACCTCGAGACCA	59.968	ATGACGCCGAGGATGATCAC	59.967	114
TRINITY_Contig12752	3	p6	(ACCCGT)3	18	3288	3305	CGACAACAACCTCGAGACCA	59.968	ATGACGCCGAGGATGATCAC	59.967	114
TRINITY_Contig12753	1	p3	(GGC)7	21	982	1002	GTAGGAATCTGCGGCCTCTG	60.25	AGAGCACTTGCCGCTATGTT	60.036	136
TRINITY_Contig12755	1	p3	(GGC)4	12	2204	2215	CAGGACTTGGATCCTTCCGG	59.821	CAAACAAGGAGCCGAACTGC	60.04	103
TRINITY_Contig12756	1	p3	(CAG)5	15	263	277	GCAGAGTGCCAGAAACTTGC	60.04	CCTTCCCGAGCTGTCACATT	60.036	101
TRINITY_Contig12759	1	p3	(GCG)5	15	1246	1260	TGGTTGGCCGTATCTCCCTA	60.032	AGGCTGGGGTTATTGGCTTC	60.033	173
TRINITY_Contig12760	1	p4	(CCGC)3	12	1517	1528	CGGAGACCACGTTCAGAACA	59.968	AAAGTACCAGGGCTACACGC	60.037	224
TRINITY_Contig12763	1	c	(CTC)4ccctgctgctgctactggtgttcttctccatgtggaggtgtgcttgctttggtggtggtctctggaacagaagcc(AGCT)3	99	1997	2095	GAGGTGTGCTTGCTTTGGTG	59.969	TCCATCTCAGCTACCTGCCT	60.032	126
TRINITY_Contig12764	1	c	(CCCGCA)3cg(TCC)4	32	746	777	AATCCCCCAAACCGAAGACC	59.961	CCACCCGCTTACAGTTGCTA	60.037	262
TRINITY_Contig12764	2	p3	(CGG)4	12	964	975	AATCCCCCAAACCGAAGACC	59.961	CCACCCGCTTACAGTTGCTA	60.037	262
TRINITY_Contig12765	1	p3	(GCG)4	12	105	116	CATCTCCAGCGCTCTCTCTG	59.969	GCTGTAGATGGCCTCGTACC	59.968	131
TRINITY_Contig12765	2	p5	(AGCAG)3	15	997	1011	CATCTCCAGCGCTCTCTCTG	59.969	GCTGTAGATGGCCTCGTACC	59.968	131
TRINITY_Contig12765	3	c	(GGAT)3catggacggggaatgggaatcttgggagctcccatggcagctacagcgac(CCGG)3	74	1285	1358	CATCTCCAGCGCTCTCTCTG	59.969	GCTGTAGATGGCCTCGTACC	59.968	131
TRINITY_Contig12767	1	p4	(GCGT)3	12	185	196	CTTTGACTGCCGACAACAGC	60.041	AATTGTCCACCATGCGTTGC	60.038	243
TRINITY_Contig12768	1	p6	(GGGCGG)3	18	129	146	CTCGCTGATCGACCAGTACC	59.97	TTGCTCATGGCCTCCTTCAG	60.035	162
TRINITY_Contig12768	2	p4	(CCAC)3	12	253	264	CTCGCTGATCGACCAGTACC	59.97	TTGCTCATGGCCTCCTTCAG	60.035	162
TRINITY_Contig12768	3	p3	(GCC)4	12	2630	2641	CTCGCTGATCGACCAGTACC	59.97	TTGCTCATGGCCTCCTTCAG	60.035	162
TRINITY_Contig12768	4	p5	(CGCAC)4	20	3119	3138	CTCGCTGATCGACCAGTACC	59.97	TTGCTCATGGCCTCCTTCAG	60.035	162
TRINITY_Contig12769	1	p4	(CATC)4	16	153	168	CTCGCAGCGCTTGGTATTTC	59.973	GATGTTGGACACGAACCCCT	59.964	117
TRINITY_Contig12769	2	p6	(CACGGC)3	18	322	339	CTCGCAGCGCTTGGTATTTC	59.973	GATGTTGGACACGAACCCCT	59.964	117
TRINITY_Contig12769	3	p3	(CGG)5	15	495	509	CTCGCAGCGCTTGGTATTTC	59.973	GATGTTGGACACGAACCCCT	59.964	117
TRINITY_Contig12769	4	c	(GCG)4aagagcatggccagggccagggccgcgagctggaagcgcgccattgtagatgcagccggt(AGCG)3	84	818	901	CTCGCAGCGCTTGGTATTTC	59.973	GATGTTGGACACGAACCCCT	59.964	117
TRINITY_Contig12771	1	p5	(TCCTC)3	15	321	335	TCCGTCCCTTTCCATCCTCT	59.957	AGGCTCTCCTTCCCAATCCT	59.955	139
TRINITY_Contig12772	1	p4	(CCTC)3	12	120	131	GCAAGCACATATTGAGCGGG	59.97	TTGTCCGCCGTATACCGATG	59.969	246
TRINITY_Contig12773	1	p3	(GCG)4	12	270	281	CTCCCCCTGATCCTCCTCAA	60.031	CCAATTGCGTGCTCAACCAA	59.968	134
TRINITY_Contig12773	2	p3	(CGC)4	12	957	968	CTCCCCCTGATCCTCCTCAA	60.031	CCAATTGCGTGCTCAACCAA	59.968	134
TRINITY_Contig12773	3	p3	(CCA)4	12	1428	1439	CTCCCCCTGATCCTCCTCAA	60.031	CCAATTGCGTGCTCAACCAA	59.968	134
TRINITY_Contig12773	4	p3	(CCG)5	15	2037	2051	CTCCCCCTGATCCTCCTCAA	60.031	CCAATTGCGTGCTCAACCAA	59.968	134
TRINITY_Contig12777	1	p3	(TCG)4	12	95	106	CAGAACAAGCCAAGCCAACC	59.967	ATAGGACTTGGACTCGCCCT	60.032	125
TRINITY_Contig12780	1	p4	(AGCG)3	12	34	45	AAGGCTCTTCACGTCCCAAG	59.965	TGATGGGACGATCCTCCCTT	60.031	235
TRINITY_Contig12781	1	p3	(CGC)4	12	228	239	CTGCTCCTCGATGTCGTACC	59.97	CGCTCTACTACCTGCTCGTG	59.972	104
TRINITY_Contig12781	2	p3	(CGC)4	12	689	700	CTGCTCCTCGATGTCGTACC	59.97	CGCTCTACTACCTGCTCGTG	59.972	104
TRINITY_Contig12781	3	p3	(GCC)4	12	1107	1118	CTGCTCCTCGATGTCGTACC	59.97	CGCTCTACTACCTGCTCGTG	59.972	104
TRINITY_Contig12782	1	p3	(CGC)5	15	2935	2949	GGAGTCAGGAAGTTGACCGG	60.037	CAACGCTGGTTCATTTGGCA	59.968	146
TRINITY_Contig12782	2	p5	(GCGCC)4	20	3145	3164	GGAGTCAGGAAGTTGACCGG	60.037	CAACGCTGGTTCATTTGGCA	59.968	146
TRINITY_Contig12786	1	p3	(GGC)4	12	1215	1226	GGGCCTACATGGAGCACTTT	60.034	CGGCCTGTTCCACCATAAGT	60.035	230
TRINITY_Contig12786	2	p3	(AGG)4	12	1730	1741	GGGCCTACATGGAGCACTTT	60.034	CGGCCTGTTCCACCATAAGT	60.035	230
TRINITY_Contig12787	1	p4	(GGCG)3	12	494	505	TACACGTTCAAGCAGCACCT	59.894	CCCTTGTTGTAGCGGAGGTT	59.964	173
TRINITY_Contig12788	1	c	(GGT)5tacaaat(GC)7agccgcgcaaggaaaaagatgcaggggtggca(CGGCG)4	88	1407	1494	CGGTCTTGAGTCCGATCCTG	59.899	AACCTCCGCTACAACAAGGG	59.964	160
TRINITY_Contig12788	2	p3	(CCA)4	12	1639	1650	CGGTCTTGAGTCCGATCCTG	59.899	AACCTCCGCTACAACAAGGG	59.964	160
TRINITY_Contig12789	1	p5	(CAACA)3	15	103	117	GCACATGCATGCACGAAGAT	59.9	ACGCCCCGTTCTGATTAAGG	60.108	135
TRINITY_Contig12791	1	p3	(GAG)4	12	126	137	TTGCAGCATCAGAGGTCTGG	60.036	AAGCTTCGTCCAAGGTAGGC	60.036	178
TRINITY_Contig12792	1	p3	(CCG)4	12	223	234	AAAGTCAGGGATGCGCATGA	60.035	CAACTTGAGCAGGGCAGAGA	59.964	254
TRINITY_Contig12792	2	p6	(AGGTCC)4	24	346	369	AAAGTCAGGGATGCGCATGA	60.035	CAACTTGAGCAGGGCAGAGA	59.964	254
TRINITY_Contig12796	1	c	(CGC)4tt(TCC)4	26	103	128	GGCGTCATGATTGCTTGGAC	59.901	GCGGTCTTCAATGATCGTGC	59.973	167
TRINITY_Contig12797	1	c	(GCCGCG)3ccagccgctgcagc(CG)6	44	1938	1981	GATCCCCCTGTTCCTCCTCT	60.031	AGCAAGGGCCCTCTATCAGA	60.031	206

TRINITY_Contig12798	1	p3	(GCC)7	21	57	77	GACGGGTGCTCTGAAGAACA	59.966	AAGCGCTCCTTCCATGTCAA	59.963	176
TRINITY_Contig12799	1	p3	(TGC)4	12	390	401	CGCTGCACTCTTTTGGCTTT	59.969	GACTTTGCCTCCAAGGTCGA	59.965	229
TRINITY_Contig12799	2	p4	(GCAA)3	12	1049	1060	CGCTGCACTCTTTTGGCTTT	59.969	GACTTTGCCTCCAAGGTCGA	59.965	229
TRINITY_Contig12799	3	p6	(GGAGCG)3	18	1381	1398	CGCTGCACTCTTTTGGCTTT	59.969	GACTTTGCCTCCAAGGTCGA	59.965	229
TRINITY_Contig12799	4	p3	(CGG)4	12	1731	1742	CGCTGCACTCTTTTGGCTTT	59.969	GACTTTGCCTCCAAGGTCGA	59.965	229
TRINITY_Contig12800	1	p3	(CGG)4	12	322	333	GCAAGCGCTCAAAAGGGAAA	59.968	GGCCAAAACCACGGTTGATC	60.039	177
TRINITY_Contig12801	1	p4	(AAAT)3	12	1086	1097	ACGGTTGAAGTGCCAATTGC	59.968	GAGTGGATGCAGCTGGTCAT	60.107	270
TRINITY_Contig12801	2	p6	(CGCCGT)3	18	2005	2022	ACGGTTGAAGTGCCAATTGC	59.968	GAGTGGATGCAGCTGGTCAT	60.107	270
TRINITY_Contig12803	1	p6	(CCGCCC)4	24	2175	2198	AGGATGGCGGGAACTTCAAG	60.035	GATGACACAGCGCGAATCAC	59.974	238
TRINITY_Contig12803	2	c	(CCG)4tctcctgctgcgcgctcaagaagccgcaggaaggtct(CGC)4	61	4728	4788	AGGATGGCGGGAACTTCAAG	60.035	GATGACACAGCGCGAATCAC	59.974	238
TRINITY_Contig12804	1	p4	(ATGT)3	12	928	939	TGCTCTTCCAAGAAGCTCGG	60.037	TGCGGGGTGTACTTGAAGAC	59.966	271
TRINITY_Contig12806	1	p5	(AATAC)3	15	227	241	GACCAACCAACAGGCTCGTA	59.966	CGACAACGTCTGAGTGAGCT	60.04	265
TRINITY_Contig12806	2	p3	(CCG)4	12	956	967	GACCAACCAACAGGCTCGTA	59.966	CGACAACGTCTGAGTGAGCT	60.04	265
TRINITY_Contig12806	3	c	(GCT)4gcgggtcctccgctgcctcgtccc(CCG)4	48	1071	1118	GACCAACCAACAGGCTCGTA	59.966	CGACAACGTCTGAGTGAGCT	60.04	265
TRINITY_Contig12806	4	p4	(GCTC)3	12	1256	1267	GACCAACCAACAGGCTCGTA	59.966	CGACAACGTCTGAGTGAGCT	60.04	265
TRINITY_Contig12808	1	p3	(CCG)4	12	126	137	CCAGTGCCATGAGTGACCTT	59.963	TCCAGCATCCCCCTGTATCA	60.03	203
TRINITY_Contig12808	2	p3	(CCG)4	12	530	541	CCAGTGCCATGAGTGACCTT	59.963	TCCAGCATCCCCCTGTATCA	60.03	203
TRINITY_Contig12809	1	p3	(GCG)5	15	631	645	TGCAGTGATGGGAGAACACC	59.963	TCTTCGAGGACCCCTTCGAT	60.033	198
TRINITY_Contig12809	2	p3	(CGC)5	15	819	833	TGCAGTGATGGGAGAACACC	59.963	TCTTCGAGGACCCCTTCGAT	60.033	198
TRINITY_Contig12810	1	p6	(CGGCGA)3	18	437	454	ATCGAAGGGGTCCTCGAAGA	60.033	TTCGCGTTCATGGACATGGA	60.037	135
TRINITY_Contig12810	2	p3	(GCG)4	12	624	635	ATCGAAGGGGTCCTCGAAGA	60.033	TTCGCGTTCATGGACATGGA	60.037	135
TRINITY_Contig12810	3	p3	(CGC)5	15	788	802	ATCGAAGGGGTCCTCGAAGA	60.033	TTCGCGTTCATGGACATGGA	60.037	135
TRINITY_Contig12813	1	p3	(GGA)4	12	931	942	TTCAGAGAGCTTGCCGGAAG	60.037	TTCTGCGAAGGGATTGGCAT	60.034	226
TRINITY_Contig12813	2	p3	(GCC)5	15	1146	1160	TTCAGAGAGCTTGCCGGAAG	60.037	TTCTGCGAAGGGATTGGCAT	60.034	226
TRINITY_Contig12813	3	p3	(GGA)4	12	1305	1316	TTCAGAGAGCTTGCCGGAAG	60.037	TTCTGCGAAGGGATTGGCAT	60.034	226
TRINITY_Contig12813	4	c	(GGGCGT)3gggcggtgtgggcgccgccggggcggacggcggcggcgcggacggggt(CGG)4	78	1449	1526	TTCAGAGAGCTTGCCGGAAG	60.037	TTCTGCGAAGGGATTGGCAT	60.034	226
TRINITY_Contig12813	5	c	(GA)7atcacaggaaagctagctacaatagtaatcaatcaagctagcggcagcagcggggagaggagggggaggcggcggcagcagcctggccaggcgagtagga(GCAG)3	126	1729	1854	TTCAGAGAGCTTGCCGGAAG	60.037	TTCTGCGAAGGGATTGGCAT	60.034	226
TRINITY_Contig12814	1	c	(GGA)4ggtgcattttgctgctgccaatttgggtatgttccaccgtatggatcctccttgta(CGG)4	80	931	1010	CAGATGAGCAAGGCCAAGGA	60.035	AGGAAAACCTAGCGCGACAA	59.966	153
TRINITY_Contig12814	2	p3	(GGA)4	12	1134	1145	CAGATGAGCAAGGCCAAGGA	60.035	AGGAAAACCTAGCGCGACAA	59.966	153
TRINITY_Contig12814	3	p6	(GGGCGT)3	18	1278	1295	CAGATGAGCAAGGCCAAGGA	60.035	AGGAAAACCTAGCGCGACAA	59.966	153
TRINITY_Contig12814	4	p3	(GGA)4	12	1678	1689	CAGATGAGCAAGGCCAAGGA	60.035	AGGAAAACCTAGCGCGACAA	59.966	153
TRINITY_Contig12815	1	p3	(CCG)7	21	128	148	AACCTGTGCAGCATGTACGA	59.965	CAGGAGGTGTCGAAGTCGTC	60.11	107
TRINITY_Contig12815	2	p3	(CCG)4	12	377	388	AACCTGTGCAGCATGTACGA	59.965	CAGGAGGTGTCGAAGTCGTC	60.11	107
TRINITY_Contig12818	1	p3	(CGG)6	18	904	921	CCACAGGGAGCATCATTGGT	60.034	CGAAAGAGATCGCGTCCGTA	59.973	184
TRINITY_Contig12819	1	p3	(CCG)6	18	287	304	CTGGGGAAGTGGAAGCTCTG	60.036	TCTCTCTCCACTCCACCTCG	60.035	157
TRINITY_Contig12819	2	p3	(CCT)5	15	594	608	CTGGGGAAGTGGAAGCTCTG	60.036	TCTCTCTCCACTCCACCTCG	60.035	157
TRINITY_Contig12819	3	p3	(GCG)4	12	731	742	CTGGGGAAGTGGAAGCTCTG	60.036	TCTCTCTCCACTCCACCTCG	60.035	157
TRINITY_Contig12819	4	c	(GGC)5atcgcgccgtcacctcccgtgccgccgtgccaagcggcgg(CAC)5	70	1043	1112	CTGGGGAAGTGGAAGCTCTG	60.036	TCTCTCTCCACTCCACCTCG	60.035	157
TRINITY_Contig12820	1	c	(CAG)4caccagcagcagcggcggcgtggccgtgcc(CCG)4cgtcgccgcctccatctagcccgcgcacggagcgatcgccggcgatcggaccgcacggaagaacagagaacgct(CGCC)3	140	2787	2926	AGTCCAGGTACGTCCCTCTC	60.034	ACGAACAAGGGGAGCAGATG	60.036	179
TRINITY_Contig12820	2	p4	(GGGA)3	12	3201	3212	AGTCCAGGTACGTCCCTCTC	60.034	ACGAACAAGGGGAGCAGATG	60.036	179
TRINITY_Contig12821	1	c	(TCCC)3ggtcccgggccgatcgagccgatggggtggccgtgg(CGCTGC)3	67	764	830	ATCTACTTCCCCCTCCGCT	60.031	GCGTGACTGACTGTCTGTGA	59.969	193
TRINITY_Contig12821	2	p4	(GAAA)3	12	1447	1458	ATCTACTTCCCCCTCCGCT	60.031	GCGTGACTGACTGTCTGTGA	59.969	193
TRINITY_Contig12822	1	p3	(CGA)6	18	193	210	GTGGATTTGGAGGGGAAGGG	60.033	CAGGAACTTCCACGCGTCA	59.968	220
TRINITY_Contig12822	2	p4	(CTCA)3	12	2162	2173	GTGGATTTGGAGGGGAAGGG	60.033	CAGGAACTTCCACGCGTCA	59.968	220
TRINITY_Contig12823	1	p3	(GTG)4	12	3398	3409	CCACTTGCTTGTCACAAGGC	59.969	CCGACGAGTACCAGATGAGC	59.97	226
TRINITY_Contig12824	1	c*	(TCCG)2(T<CCG>)(CCG)3	21	31	51	GAGATCGCCGAGCAGATTCA	59.969	CCAGCACCCCATCATCAACT	60.034	217
TRINITY_Contig12824	2	p3	(CGG)4	12	161	172	GAGATCGCCGAGCAGATTCA	59.969	CCAGCACCCCATCATCAACT	60.034	217
TRINITY_Contig12824	3	p3	(ACC)4	12	4064	4075	GAGATCGCCGAGCAGATTCA	59.969	CCAGCACCCCATCATCAACT	60.034	217
TRINITY_Contig12825	1	p3	(CGC)4	12	428	439	GTACGACCCATCCAAGCCAA	60.036	AGTGGTGACAGGGTTGGTTG	60.107	112
TRINITY_Contig12825	2	c	(CCG)4gggcgtccga(GCC)4	34	1621	1654	GTACGACCCATCCAAGCCAA	60.036	AGTGGTGACAGGGTTGGTTG	60.107	112
TRINITY_Contig12825	3	p5	(GGGGC)4	20	1865	1884	GTACGACCCATCCAAGCCAA	60.036	AGTGGTGACAGGGTTGGTTG	60.107	112
TRINITY_Contig12825	4	p4	(GGCG)3	12	2029	2040	GTACGACCCATCCAAGCCAA	60.036	AGTGGTGACAGGGTTGGTTG	60.107	112
TRINITY_Contig12826	1	p3	(GGC)4	12	2237	2248	CAGATGGGGCAGATCTCGAC	59.967	TTCCTCTACCTCGAGCAGCT	60.034	245
TRINITY_Contig12827	1	p2	(CG)7	14	108	121	GGTCATTTCATGTTCGGCGG	59.901	GAGGTTGGGGCTGAATTGGA	59.96	202
TRINITY_Contig12827	2	p4	(ATCC)3	12	736	747	GGTCATTTCATGTTCGGCGG	59.901	GAGGTTGGGGCTGAATTGGA	59.96	202
TRINITY_Contig12828	1	p4	(GATG)3	12	143	154	GATCGGACGCTGGAGAAGAG	59.97	CAGGGGTAGCAAACTCAGCA	59.963	198
TRINITY_Contig12828	2	c*	(GGCG)2(G<GCG>CGCGCGCGCGGGGAAGGCGGAGGTGGGGAAGCGACCGAGCGGAGCGAGAGCTGCGGTGAGGGCGGCGAGTGGGAGATCGGACGCTGGAGAAGAGAAGGCGGTCCGGGTGGGTGCGCCCAAGTATTGATTCTGATGAGTTGTAGCTATAAAAAGAGGTAGGATTCCGCACACTACATTTGGTGTCAGGTTGGAATGACCTGTGCTACCTTTTAGACACACTGATGGCTATGCAGCTGCTTAGAAGCCTCAGTCTGCTGAGTTTGCTACCCCTGAATGTACGAAGAAGTAGATCTGCAAGATGACCATATCCAAGTGTATGAGCACTGGCGTGTTCATGTAACGATGCAAGATGCCTTGCTCCCTGATCAGACCTCTGATGGAGGTGGATTTGTGTCCTCAATAGCTCGGTGGTGCTGCATAC	21	899	919	GATCGGACGCTGGAGAAGAG	59.97	CAGGGGTAGCAAACTCAGCA	59.963	198
TRINITY_Contig12831	1	p4	(GCAC)3	12	700	711	CATCCTGTACGCCGTCTACC	59.97	AAGAACTTCTGGAGGACGCG	60.038	243
TRINITY_Contig12831	2	p3	(AAG)5	15	1351	1365	CATCCTGTACGCCGTCTACC	59.97	AAGAACTTCTGGAGGACGCG	60.038	243
TRINITY_Contig12831	3	p2	(AC)6	12	1573	1584	CATCCTGTACGCCGTCTACC	59.97	AAGAACTTCTGGAGGACGCG	60.038	243
TRINITY_Contig12832	1	p3	(CGG)6	18	132	149	TTGGGAGGTCGAAACTCGTG	59.967	TTCCAGACGTGTCCAGCTTC	59.966	180
TRINITY_Contig12833	1	c	(ACCA)3tggagttgttgccacggcctctcctcctcatccttctggccgtgtgggctct(CGC)4	76	117	192	GCGTCGCGTTCAAGATCTTC	59.976	CCTGCCGAACTCCTTCTTGT	59.965	188
TRINITY_Contig12834	1	p6	(GGGTTT)3	18	2783	2800	TCGGTGCACTTTACAGGTC	59.966	CAGGGTGAGCTGGAACATGT	59.963	158
TRINITY_Contig12834	2	c	(CAG)4ctgctgcacaggcacaggcttgggcttcggcttcggctgcttcc(GCT)5	71	2996	3066	TCGGTGCACTTTACAGGTC	59.966	CAGGGTGAGCTGGAACATGT	59.963	158
TRINITY_Contig12836	1	p3	(CCG)4	12	1188	1199	AGCAAGATCGAGCAACCACA	59.965	GTCCATGGTCAAGATGGGCA	60.034	162
TRINITY_Contig12837	1	p3	(GCC)4	12	2902	2913	CAGCAGTTTGATCGATGCCG	59.973	TCCCCCAACATCTCTCGGAT	60.031	210
TRINITY_Contig12838	1	c	(GCC)7ttccctcgccttccttttcgtctctgactttttctccaagctcatctccctcca(TCCC)3	87	63	149	CTTGCAGCACTTGGGAACAC	59.969	GCCAAACCCAAGTTTCGACC	59.968	251
TRINITY_Contig12839	1	p3	(CCG)5	15	1812	1826	GGATTTTTGGCTAGCTCGCG	59.972	CAGGGAAACACTGTTGCAGC	59.969	149
TRINITY_Contig12842	1	p3	(CGC)4	12	225	236	CGCCTTTGATGCTCTGGAGA	60.108	GCAGACAAACAAGCAAGCGA	59.971	208
TRINITY_Contig12842	2	p6	(GCTCCG)3	18	547	564	CGCCTTTGATGCTCTGGAGA	60.108	GCAGACAAACAAGCAAGCGA	59.971	208
TRINITY_Contig12845	1	p3	(GCG)4	12	1087	1098	CAAGGTCCGGAGCCTTGATT	60.035	GGAAAGGAGAAGTGCGTGGA	59.965	138
TRINITY_Contig12845	2	p5	(GCGTC)4	20	1225	1244	CAAGGTCCGGAGCCTTGATT	60.035	GGAAAGGAGAAGTGCGTGGA	59.965	138
TRINITY_Contig12846	1	p3	(CGG)4	12	1302	1313	CATCCGTCAAGCATTGCTCG	59.973	AGTGACTCCAATGGGCCATG	60.034	118
TRINITY_Contig12847	1	c*	(TTC)4catccttatttttgtccattaattcttgacatgatcaacaaacaatccgtacatag(ATACAT)4(AT)6*(AC)6	114	181	294	TGCAAACACGACCGGGAATA	59.965	CCTCACCTCGCCTACCAATC	59.895	208

TRINITY_Contig12848	1	c	(GCCG)3ccatgctggaaactgaaacatgtccggccgctgtgctggaaactgaaacaggtcagtttccggcgtgcctgc(CGG)4	96	49	144	ACAGCCCTTCACTTGAGCTC	59.964	GAGTGCCGAACCACCTTGTA	59.966	175
TRINITY_Contig12849	1	p3	(GCG)4	12	1418	1429	CAACCGTAGCAGCAACCCTA	60.037	GTGGCATGTCGCTGTTTGTT	59.969	151
TRINITY_Contig12849	2	p5	(TCGCG)3	15	1734	1748	CAACCGTAGCAGCAACCCTA	60.037	GTGGCATGTCGCTGTTTGTT	59.969	151
TRINITY_Contig12851	1	p5	(TTGGG)3	15	1219	1233	AGGATGCAGCTGAGAACACC	60.036	TTCCTCGCTCAGGGTATCCA	60.033	212
TRINITY_Contig12856	1	c	(AACC)3aacaaccaaccacacaccca(GAT)5	47	156	202	TGTTGATCCCCTTCTTCGCC	60.035	CAAGACCTTCACCGTCAGCT	59.966	113
TRINITY_Contig12856	2	p3	(CCG)4	12	1589	1600	TGTTGATCCCCTTCTTCGCC	60.035	CAAGACCTTCACCGTCAGCT	59.966	113
TRINITY_Contig12856	3	p3	(GCG)4	12	1926	1937	TGTTGATCCCCTTCTTCGCC	60.035	CAAGACCTTCACCGTCAGCT	59.966	113
TRINITY_Contig12857	1	p3	(CGG)5	15	679	693	GCTTGGGCAGATCAACTCCT	60.035	GCAAGCAATGTGCCACTTGA	59.968	178
TRINITY_Contig12858	1	p3	(GCG)5	15	444	458	AGCCAGGTACGAACGAACAG	60.039	GCCTCTTCCCCCTTCTTGTC	60.035	269
TRINITY_Contig12858	2	p3	(CCA)4	12	610	621	AGCCAGGTACGAACGAACAG	60.039	GCCTCTTCCCCCTTCTTGTC	60.035	269
TRINITY_Contig12859	1	p2	(TA)10	20	431	450	GAGGCTTCGATGAGGTTGCT	60.108	TCTATGTGTTGCGTGCCTGT	59.965	125
TRINITY_Contig12860	1	c	(TCT)4(TCC)4cttctccacattctcttccacctccgttcccctttcttctcccccttcttct(TCC)4	87	743	829	TCCTCTTCCATGGCCGTCTA	60.033	CCAAGCAACAACACGACCAG	59.971	263
TRINITY_Contig12860	2	p3	(GCA)4	12	1360	1371	TCCTCTTCCATGGCCGTCTA	60.033	CCAAGCAACAACACGACCAG	59.971	263
TRINITY_Contig12860	3	p3	(CGG)4	12	2254	2265	TCCTCTTCCATGGCCGTCTA	60.033	CCAAGCAACAACACGACCAG	59.971	263
TRINITY_Contig12860	4	p3	(GGC)5	15	3068	3082	TCCTCTTCCATGGCCGTCTA	60.033	CCAAGCAACAACACGACCAG	59.971	263
TRINITY_Contig12860	5	p2	(CG)6	12	3436	3447	TCCTCTTCCATGGCCGTCTA	60.033	CCAAGCAACAACACGACCAG	59.971	263
TRINITY_Contig12861	1	c	(TCT)4(TCC)4cttctccacattctcttccacctccgttcccctttcttctcccccttcttct(TCC)4	87	743	829	TCCTCTTCCATGGCCGTCTA	60.033	CCAAGCAACAACACGACCAG	59.971	263
TRINITY_Contig12861	2	p3	(GCA)4	12	1360	1371	TCCTCTTCCATGGCCGTCTA	60.033	CCAAGCAACAACACGACCAG	59.971	263
TRINITY_Contig12861	3	p3	(CGG)4	12	2254	2265	TCCTCTTCCATGGCCGTCTA	60.033	CCAAGCAACAACACGACCAG	59.971	263
TRINITY_Contig12861	4	p3	(CCA)5	15	2662	2676	TCCTCTTCCATGGCCGTCTA	60.033	CCAAGCAACAACACGACCAG	59.971	263
TRINITY_Contig12861	5	p3	(GGC)5	15	3159	3173	TCCTCTTCCATGGCCGTCTA	60.033	CCAAGCAACAACACGACCAG	59.971	263
TRINITY_Contig12861	6	p6	(GAGCCG)3	18	3883	3900	TCCTCTTCCATGGCCGTCTA	60.033	CCAAGCAACAACACGACCAG	59.971	263
TRINITY_Contig12861	7	p4	(AAAT)3	12	4041	4052	TCCTCTTCCATGGCCGTCTA	60.033	CCAAGCAACAACACGACCAG	59.971	263
TRINITY_Contig12862	1	p3	(GGC)4	12	288	299	CATGCCAGGCTCACTCTTCA	60.036	GTACTTGAGCCCGAACAGCT	60.037	106
TRINITY_Contig12862	2	p3	(CGG)5	15	1005	1019	CATGCCAGGCTCACTCTTCA	60.036	GTACTTGAGCCCGAACAGCT	60.037	106
TRINITY_Contig12863	1	c	(CGAA)3gatgtgctcgcaagatccgtcgactccggcagatact(CGACGG)3	67	709	775	ACAGAAGGAGCATGACTGGC	60.036	GTTGCCTTCATCTCCGTCCA	60.036	247
TRINITY_Contig12864	1	c	(CT)11gctcctctgctctttatactcaccttctctctctgccctcacctga(CTCC)3	80	15	94	GCTCCTTCCCCCTCTCGATA	60.179	AAGAATCCTCCTTTCGCCGG	60.108	160
TRINITY_Contig12864	2	p3	(GGC)4	12	350	361	GCTCCTTCCCCCTCTCGATA	60.179	AAGAATCCTCCTTTCGCCGG	60.108	160
TRINITY_Contig12867	1	p3	(GGC)5	15	179	193	GTCAAGGACTGGCTGCTGAT	60.036	GTGGTTGTAGTAGGCCACCC	60.036	114
TRINITY_Contig12868	1	p4	(GAAG)3	12	2169	2180	CACAACCGCACTGTTCCAAG	59.971	GACCCTCCATCTCGATGCTG	59.967	155
TRINITY_Contig12870	1	c*	(CCG)4ttcgtgtttgatgatgttggcgatgacctca(CGC)4tgccgaaa(CCG)5(GCCGAT)3*	92	719	810	TTCACTTCACCTCGACCACG	59.968	GTCTGGCTGTCTGTTCACGA	59.968	102
TRINITY_Contig12870	2	p3	(CGG)4	12	1382	1393	TTCACTTCACCTCGACCACG	59.968	GTCTGGCTGTCTGTTCACGA	59.968	102
TRINITY_Contig12874	1	c	(TTGG)3gttactcaatgtgttgtgagcagaggagt(AGAA)3	53	1051	1103	ATGATCGAAGCTCATCGCGT	59.969	CAGGTTGTTTGTGTCGCAGG	59.971	280
TRINITY_Contig12875	1	p3	(GCC)4	12	1259	1270	ACACGTCGTACTGGTGGAAC	59.969	GAGCCACCTGCAGAACATCT	60.036	218
TRINITY_Contig12875	2	p4	(GCCG)3	12	2066	2077	ACACGTCGTACTGGTGGAAC	59.969	GAGCCACCTGCAGAACATCT	60.036	218
TRINITY_Contig12875	3	c	(CCT)4gccccccttcactctctcctccgctcgggtttgaggccggagagtgtggggttctctcctctgggtcggcgctgcgctttgggtggggcggatcggtgc(GGTGG)4gtcggcgagcgctacggtttcgtgcccgcttttccccaccttttctccccgtccgtgccggtgc(GCT)6	212	2208	2419	ACACGTCGTACTGGTGGAAC	59.969	GAGCCACCTGCAGAACATCT	60.036	218
TRINITY_Contig12879	1	p4	(CCGG)3	12	220	231	TCAGATCCGCTTCCCAACAC	60.036	CGGCGGCATTTTCTCAGATG	59.971	266
TRINITY_Contig12879	2	p4	(AAAG)3	12	695	706	TCAGATCCGCTTCCCAACAC	60.036	CGGCGGCATTTTCTCAGATG	59.971	266
TRINITY_Contig12879	3	p5	(GCGCC)3	15	1228	1242	TCAGATCCGCTTCCCAACAC	60.036	CGGCGGCATTTTCTCAGATG	59.971	266
TRINITY_Contig12879	4	p3	(TGC)4	12	1510	1521	TCAGATCCGCTTCCCAACAC	60.036	CGGCGGCATTTTCTCAGATG	59.971	266
TRINITY_Contig12880	1	p4	(GCCC)3	12	15	26	GCCCTACTTTCCTGCACCTT	59.962	AAAGAAGTAGCTGCACCCCC	59.962	195
TRINITY_Contig12880	2	p3	(GCG)4	12	210	221	GCCCTACTTTCCTGCACCTT	59.962	AAAGAAGTAGCTGCACCCCC	59.962	195
TRINITY_Contig12881	1	p3	(CCT)5	15	4233	4247	GGCATCGCATTCTTCGTCAC	59.973	GACTTTCTGGTGGCTCGTCA	59.966	128
TRINITY_Contig12881	2	p4	(GAGC)3	12	4355	4366	GGCATCGCATTCTTCGTCAC	59.973	GACTTTCTGGTGGCTCGTCA	59.966	128
TRINITY_Contig12881	3	p3	(AGC)4	12	4574	4585	GGCATCGCATTCTTCGTCAC	59.973	GACTTTCTGGTGGCTCGTCA	59.966	128
TRINITY_Contig12882	1	p3	(CGG)5	15	397	411	AGGGCCCTCCAGATACACAT	60.03	CCGTAGAGCAGGTCATCGAC	59.97	158
TRINITY_Contig12882	2	p4	(GAAA)3	12	2081	2092	AGGGCCCTCCAGATACACAT	60.03	CCGTAGAGCAGGTCATCGAC	59.97	158
TRINITY_Contig12883	1	p3	(CGC)4	12	601	612	AAGCCAACCTGAACCCACAA	60.033	ATGAGCTCCGGACTTGTGTG	60.037	254
TRINITY_Contig12884	1	c	(GGA)7gggggttggtgaggcggcggtgaggaggagcggg(GAA)4gacgacgaggaggaggcgctgtcgcagagctcgtcgatcggggcggcgtcgtcggacagctcgtccatcggggagaactcggcgtcggaca(AGG)4	170	88	257	CGACGACGAGAAGAAGAGGG	59.903	ATCGTCTTCCTCCTGCTCCT	60.033	115
TRINITY_Contig12884	2	p3	(AAG)4	12	428	439	CGACGACGAGAAGAAGAGGG	59.903	ATCGTCTTCCTCCTGCTCCT	60.033	115
TRINITY_Contig12884	3	p3	(CGC)5	15	669	683	CGACGACGAGAAGAAGAGGG	59.903	ATCGTCTTCCTCCTGCTCCT	60.033	115
TRINITY_Contig12884	4	c	(GCC)4gcggcaccgacggcggcggcccgacgccgccgccacgtccgcgggctcg(GAA)4	73	798	870	CGACGACGAGAAGAAGAGGG	59.903	ATCGTCTTCCTCCTGCTCCT	60.033	115
TRINITY_Contig12884	5	c	(GGATGG)3gccgcggccgtgcggctgaca(CGGCG)3	54	1087	1140	CGACGACGAGAAGAAGAGGG	59.903	ATCGTCTTCCTCCTGCTCCT	60.033	115
TRINITY_Contig12885	1	c	(CGCGC)3cgtgtcagccgcacggccgcgg(CCCATC)3	55	229	283	CCTGCGGGTTCAGATTCAGT	60.036	AGGTTGTTCCATCCGTGGTC	59.964	219
TRINITY_Contig12885	2	c	(TTC)4cgagcccgcggacgtggcggcggcgtcgggccgccgccgtcggtgcc(GCG)4	71	501	571	CCTGCGGGTTCAGATTCAGT	60.036	AGGTTGTTCCATCCGTGGTC	59.964	219
TRINITY_Contig12885	3	p3	(GCG)5	15	685	699	CCTGCGGGTTCAGATTCAGT	60.036	AGGTTGTTCCATCCGTGGTC	59.964	219
TRINITY_Contig12885	4	p3	(TCT)4	12	916	927	CCTGCGGGTTCAGATTCAGT	60.036	AGGTTGTTCCATCCGTGGTC	59.964	219
TRINITY_Contig12885	5	c	(TCC)4ttgtccgacgccgagttctccccgatggacgagctgtccgacgacgccgccccgatcgacgagctctgcgacagcgcctcctcctcgtcg(TCT)4tccccgctcctcctcaccgccgcctcaccaaccc(CCT)7	169	1544	1712	CCTGCGGGTTCAGATTCAGT	60.036	AGGTTGTTCCATCCGTGGTC	59.964	219
TRINITY_Contig12890	1	p3	(CCG)4	12	121	132	GGCGATTCTAGAGTTCGCGA	59.971	GCCGAACATAAACCCCAGGA	60.035	245
TRINITY_Contig12892	1	p3	(CGA)4	12	390	401	GGCCATCCAGGAGTCTATGC	59.964	TGTGAGTACGACCCCGATCT	60.035	139
TRINITY_Contig12892	2	p3	(GGC)4	12	572	583	GGCCATCCAGGAGTCTATGC	59.964	TGTGAGTACGACCCCGATCT	60.035	139
TRINITY_Contig12892	3	p3	(GCG)4	12	764	775	GGCCATCCAGGAGTCTATGC	59.964	TGTGAGTACGACCCCGATCT	60.035	139
TRINITY_Contig12892	4	p4	(GGGT)3	12	1699	1710	GGCCATCCAGGAGTCTATGC	59.964	TGTGAGTACGACCCCGATCT	60.035	139
TRINITY_Contig12892	5	c	(CTC)5ggcttctcaccctcc(CT)6	42	1947	1988	GGCCATCCAGGAGTCTATGC	59.964	TGTGAGTACGACCCCGATCT	60.035	139
TRINITY_Contig12892	6	p4	(TCCG)3	12	2142	2153	GGCCATCCAGGAGTCTATGC	59.964	TGTGAGTACGACCCCGATCT	60.035	139
TRINITY_Contig12895	1	p3	(CGC)4	12	644	655	CGATGAGGGTCCTGACGATG	59.969	TTCCCTCGTTCACCTTGCTC	59.965	240
TRINITY_Contig12896	1	p3	(CGG)4	12	478	489	ATGCAGGAGCCCTTCTTCAC	60.035	CGCTCTCCATGTCCTCGATC	60.04	229
TRINITY_Contig12898	1	p4	(GCGG)3	12	1838	1849	AGCAACATTCCTACCTGCCC	60.034	AGCTTTCGGGTTCCTCCATG	60.035	106
TRINITY_Contig12900	1	p3	(GCC)5	15	399	413	CAGGAAAACGAACCACAGCG	60.041	TCCGTGCCTTTCCTGAGTTC	59.965	183
TRINITY_Contig12900	2	p4	(AAGA)4	16	1132	1147	CAGGAAAACGAACCACAGCG	60.041	TCCGTGCCTTTCCTGAGTTC	59.965	183
TRINITY_Contig12901	1	c	(CGCAGC)3agggcgggagcgccgtggagaagggggagagcgagaagacgacgacaaaggaa(GGC)4	83	347	429	GACTCGGACTCGTCGAATCC	59.972	ATGGGGAGTTTCGAGCTTGG	60.035	128
TRINITY_Contig12902	1	p6	(CCCCTC)3	18	3232	3249	AAGGTGTTCCGGTAAGGCAG	59.964	CGACGAGGCTACTGATCACC	59.97	178
TRINITY_Contig12903	1	p3	(GTC)4	12	1	12	CCTAGGCTGGGAGACTCACT	60.033	CAGCATACCCTGTAGCTCCG	59.967	191
TRINITY_Contig12903	2	p3	(CGG)4	12	190	201	CCTAGGCTGGGAGACTCACT	60.033	CAGCATACCCTGTAGCTCCG	59.967	191
TRINITY_Contig12903	3	p3	(CGC)4	12	384	395	CCTAGGCTGGGAGACTCACT	60.033	CAGCATACCCTGTAGCTCCG	59.967	191
TRINITY_Contig12903	4	p3	(GCC)4	12	551	562	CCTAGGCTGGGAGACTCACT	60.033	CAGCATACCCTGTAGCTCCG	59.967	191
TRINITY_Contig12903	5	p3	(TGC)4	12	2531	2542	CCTAGGCTGGGAGACTCACT	60.033	CAGCATACCCTGTAGCTCCG	59.967	191
TRINITY_Contig12908	1	p3	(TAG)4	12	704	715	GGGGCAACGAACATACTCCA	60.036	GAGAAGCCCGTCATCTACCG	59.969	175
TRINITY_Contig12908	2	p4	(CAAC)3	12	1678	1689	GGGGCAACGAACATACTCCA	60.036	GAGAAGCCCGTCATCTACCG	59.969	175
TRINITY_Contig12908	3	p4	(GTGC)3	12	2036	2047	GGGGCAACGAACATACTCCA	60.036	GAGAAGCCCGTCATCTACCG	59.969	175
TRINITY_Contig12911	1	p4	(CCGC)3	12	170	181	TGGTCCTCCATGGTGCTCTA	59.957	TTCCAGACGAATGGCACCTC	60.036	149
TRINITY_Contig12911	2	p3	(CGC)4	12	517	528	TGGTCCTCCATGGTGCTCTA	59.957	TTCCAGACGAATGGCACCTC	60.036	149
TRINITY_Contig12911	3	p3	(CGA)4	12	754	765	TGGTCCTCCATGGTGCTCTA	59.957	TTCCAGACGAATGGCACCTC	60.036	149

TRINITY_Contig12913	1	p4	(GCCT)4	16	18	33	TTGCCGAATGCCTTAGGGTT	59.961	GCCACCAACCACAGCTAGAT	60.035	226
TRINITY_Contig12915	1	p4	(TCCT)4	16	1554	1569	GGGCAACAAGGCGAAAGAAG	60.039	GCGCTTGCAACTGGTAACAA	59.97	146
TRINITY_Contig12918	1	p5	(GAGGC)4	20	293	312	CATTGCGCTGCCATCTCTTC	59.971	TATTCACAGTCGCAGCTGCA	60.037	137
TRINITY_Contig12921	1	p3	(TTC)4	12	3281	3292	CACATTTTGAGGGCACGCAA	59.968	GGGGATCCTGTCAGAGGTCT	60.032	130
TRINITY_Contig12924	1	p3	(AGG)4	12	115	126	AGATGCGGTGTCAGGGAAAG	60.036	TTGCAAGCGACTTCAAGCAC	59.971	100
TRINITY_Contig12924	2	p3	(CGT)4	12	365	376	AGATGCGGTGTCAGGGAAAG	60.036	TTGCAAGCGACTTCAAGCAC	59.971	100
TRINITY_Contig12925	1	p3	(CGG)4	12	1117	1128	CCAACTCTGACTGTCCCCAC	59.965	TGGCGAACTTTCTTTTGCGG	59.97	126
TRINITY_Contig12925	2	p3	(CGC)4	12	1489	1500	CCAACTCTGACTGTCCCCAC	59.965	TGGCGAACTTTCTTTTGCGG	59.97	126
TRINITY_Contig12925	3	p3	(GCT)4	12	1933	1944	CCAACTCTGACTGTCCCCAC	59.965	TGGCGAACTTTCTTTTGCGG	59.97	126
TRINITY_Contig12926	1	p3	(TCG)4	12	940	951	TCTGTATCCAATCTCGGCGC	59.968	CGAAGACAAACACGGTGCTG	60.042	223
TRINITY_Contig12927	1	p5	(GTGTA)3	15	1279	1293	CATCCTCCATTCGCCATCGA	59.966	CAGGCACGGAACCAAACTTG	59.969	129
TRINITY_Contig12928	1	p3	(GCG)4	12	84	95	CGTCATCTTCGGCCACCATA	59.896	CGTGTAGAAGCGGTAGGTCC	59.9	148
TRINITY_Contig12928	2	p3	(CGC)5	15	947	961	CGTCATCTTCGGCCACCATA	59.896	CGTGTAGAAGCGGTAGGTCC	59.9	148
TRINITY_Contig12928	3	p3	(AGG)4	12	2405	2416	CGTCATCTTCGGCCACCATA	59.896	CGTGTAGAAGCGGTAGGTCC	59.9	148
TRINITY_Contig12929	1	p5	(CCGCG)3	15	407	421	CGGTGATTACGGAGTCGAGG	59.971	GGACCAATGTGCTCACCAGA	59.963	271
TRINITY_Contig12931	1	p3	(GGA)4	12	3134	3145	CATCGCCGCATTTTCTCTGG	59.971	TTCCTTCTCCTCCTCCTCCG	60.033	194
TRINITY_Contig12933	1	p5	(CGCGC)3	15	4004	4018	TGGCGGAGTCCAGTAGATGA	60.034	TGGCGGAGTCCAGTAGATGA	60.034	210
TRINITY_Contig12934	1	p3	(GGC)4	12	2165	2176	TTATTCCCAGCCACCGCAAT	60.034	ATGCTGCTGTGATGGCTCTT	60.034	159
TRINITY_Contig12934	2	p3	(GTG)4	12	2307	2318	TTATTCCCAGCCACCGCAAT	60.034	ATGCTGCTGTGATGGCTCTT	60.034	159
TRINITY_Contig12935	1	c	(AAGG)3aagcaaggtgtggagtaggtg(GAT)4	45	2076	2120	CAGCAAGCACAACACCAGTC	59.97	CCCTTGCTGGTCAGGCTTTA	59.962	117
TRINITY_Contig12936	1	c	(CGG)4gcagcccgaccccgggtagtatgccgccggcggcggggggct(GTA)4(CGG)4	78	453	530	ATCTGCTCCCGTTTTCTGCA	59.963	ATCTGCTCCCGTTTTCTGCA	59.963	201
TRINITY_Contig12936	2	c	(CCG)4(TAC)4	24	1427	1450	ATCTGCTCCCGTTTTCTGCA	59.963	ATCTGCTCCCGTTTTCTGCA	59.963	201
TRINITY_Contig12938	1	p6	(CCGCGG)3	18	374	391	TCCACTTCTCTCTCCTCCCG	60.034	ACGCTTGAAAACGCTGTTCC	59.971	190
TRINITY_Contig12942	1	p3	(TCC)5	15	171	185	TCGGTTTCGTCAGGTCACTG	59.968	GGAGAGGGGATGAGAAGGGT	60.031	119
TRINITY_Contig12944	1	p3	(CCG)5	15	907	921	GCGTCAGCACACACATCATC	59.904	TGGACGGCGGGTATAAATCG	59.968	205
TRINITY_Contig12945	1	p3	(ATG)4	12	1716	1727	TGTTGATAGGCAGGGCTGTG	60.035	TACGCTTCCTCCCGCATTTT	60.036	187
TRINITY_Contig12947	1	p2	(GC)8	16	230	245	AGCAGGAATCGGAGGAGTCT	60.033	AAGAAGAGGAAGACGCTGCC	60.037	253
TRINITY_Contig12947	2	p3	(CTG)5	15	616	630	AGCAGGAATCGGAGGAGTCT	60.033	AAGAAGAGGAAGACGCTGCC	60.037	253
TRINITY_Contig12947	3	p2	(TG)6	12	808	819	AGCAGGAATCGGAGGAGTCT	60.033	AAGAAGAGGAAGACGCTGCC	60.037	253
TRINITY_Contig12948	1	c	(CGC)4ctcccgctcgccgcccagcgttctcgcactc(CCA)4	55	819	873	GCTTCTCAAGGTCACGGTGA	59.966	TTCTGCTGCTCCTTGCCTAC	60.036	224
TRINITY_Contig12949	1	c	(AAAACT)4aaaatgcaggcggaataaatcaaa(CTAG)3	60	175	234	ACAGGAGCAGCATTCTCCAC	60.036	ACGCTGACCTTGTCAAGAGG	59.966	274
TRINITY_Contig12951	1	p3	(CTT)4	12	2663	2674	GCCGGACACCAGAAACACTA	59.966	CTCTCGGATGCTGCTACCTG	59.968	174
TRINITY_Contig12951	2	p3	(CCT)5	15	2967	2981	GCCGGACACCAGAAACACTA	59.966	CTCTCGGATGCTGCTACCTG	59.968	174
TRINITY_Contig12951	3	c	(CGAA)3gaactcggcgccaaact(CCG)4tcgcagcaacggcggcgatggcgaccccggccctacgctagatacgaaaccctcac(GCCG)3	109	3392	3500	GCCGGACACCAGAAACACTA	59.966	CTCTCGGATGCTGCTACCTG	59.968	174
TRINITY_Contig12952	1	c	(CGGC)3gtgagggtttcgtatctagcgtagggccggggtcgccatcgccgccgttgctgcga(CGG)4agtttggcgccgagttc(TTCG)3	109	161	269	GGAGTCAAGAACGCACCTCA	59.966	CAGGTAGCAGCATCCGAGAG	59.968	202
TRINITY_Contig12952	2	p3	(GGA)5	15	678	692	GGAGTCAAGAACGCACCTCA	59.966	CAGGTAGCAGCATCCGAGAG	59.968	202
TRINITY_Contig12952	3	p3	(AAG)4	12	987	998	GGAGTCAAGAACGCACCTCA	59.966	CAGGTAGCAGCATCCGAGAG	59.968	202
TRINITY_Contig12952	4	p4	(AAAG)3	12	4629	4640	GGAGTCAAGAACGCACCTCA	59.966	CAGGTAGCAGCATCCGAGAG	59.968	202
TRINITY_Contig12953	1	c	(CGGC)3gtgagggtttcgtatctagcgtagggccggggtcgccatcgccgccgttgctgcga(CGG)4agtttggcgccgagttc(TTCG)3	109	161	269	CTCTCGGATGCTGCTACCTG	59.968	GCCGGACACCAGAAACACTA	59.966	183
TRINITY_Contig12953	2	p3	(GGA)5	15	678	692	CTCTCGGATGCTGCTACCTG	59.968	GCCGGACACCAGAAACACTA	59.966	183
TRINITY_Contig12953	3	p3	(AAG)4	12	987	998	CTCTCGGATGCTGCTACCTG	59.968	GCCGGACACCAGAAACACTA	59.966	183
TRINITY_Contig12953	4	p4	(AAAG)3	12	4664	4675	CTCTCGGATGCTGCTACCTG	59.968	GCCGGACACCAGAAACACTA	59.966	183
TRINITY_Contig12953	5	p3	(CTG)4	12	6003	6014	CTCTCGGATGCTGCTACCTG	59.968	GCCGGACACCAGAAACACTA	59.966	183
TRINITY_Contig12954	1	p5	(CCGCG)3	15	492	506	AGCAAGCTGGATCCTGCAAT	60.033	GTTTCAGTGTGCCAGCTTGG	59.969	235
TRINITY_Contig12955	1	p4	(TGGC)3	12	285	296	CGCACTGTGAGTCCATGACT	60.038	TTCCGTCCGTTGCAGTCTAC	60.039	189
TRINITY_Contig12955	2	p3	(TGG)5	15	683	697	CGCACTGTGAGTCCATGACT	60.038	TTCCGTCCGTTGCAGTCTAC	60.039	189
TRINITY_Contig12955	3	p3	(CTC)5	15	1114	1128	CGCACTGTGAGTCCATGACT	60.038	TTCCGTCCGTTGCAGTCTAC	60.039	189
TRINITY_Contig12955	4	p2	(CG)7	14	1382	1395	CGCACTGTGAGTCCATGACT	60.038	TTCCGTCCGTTGCAGTCTAC	60.039	189
TRINITY_Contig12957	1	p4	(CCTG)3	12	164	175	CACCTGCACTGCACTCACTA	59.966	AAGGCCAAGATGTCAGGCTC	60.035	188
TRINITY_Contig12957	2	p4	(ACAG)4	16	334	349	CACCTGCACTGCACTCACTA	59.966	AAGGCCAAGATGTCAGGCTC	60.035	188
TRINITY_Contig12957	3	p4	(GCCT)3	12	931	942	CACCTGCACTGCACTCACTA	59.966	AAGGCCAAGATGTCAGGCTC	60.035	188
TRINITY_Contig12957	4	c	(GCC)5gcgcaggcgcagggaggtctccatcaacgggtttgcttgactcttccttggcgaggg(GGA)4gagatcccagcagcagaggctgggggggggatct(CTC)4cccctcgccaaggaagagtcaagcaaacccgttgatggagacctccctgcgcctgc(GCG)5	201	1871	2071	CACCTGCACTGCACTCACTA	59.966	AAGGCCAAGATGTCAGGCTC	60.035	188
TRINITY_Contig12958	1	p4	(GGCG)4	16	2547	2562	TCATGGATGGCCTCCTCTCA	60.031	GTCCAGTGGGTCCAGTTCTG	59.965	160
TRINITY_Contig12959	1	p3	(CCT)4	12	530	541	CGATCAAGTCAGCATGCAGC	59.972	CAACAGCAGCAACAGGAACC	59.969	161
TRINITY_Contig12962	1	c	(CGC)4gtcaccgtctcgaacgccaccacgcgcctgccggccagcagcaccacctccacctccgcgccctggaacggtagcagccgca(CGT)4gcggcatccgaaacctgctcgccgtgcgatcctgttctgcccgattctccgccg(CGT)5	175	329	503	AGGGCTCCGGAGAAATCTCT	60.032	TGCTCTCCTCCTCCACCTAC	60.033	140
TRINITY_Contig12962	2	c	(CGGCG)3gcgtgcgcgacggcgaagagcggggacgcgagcgcccgcgcccaggacgggtgcacgaggcggaggcgc(GCG)5	99	1238	1336	AGGGCTCCGGAGAAATCTCT	60.032	TGCTCTCCTCCTCCACCTAC	60.033	140
TRINITY_Contig12962	3	p3	(GAT)4	12	1519	1530	AGGGCTCCGGAGAAATCTCT	60.032	TGCTCTCCTCCTCCACCTAC	60.033	140
TRINITY_Contig12964	1	c	(CAC)4ccccagccacgcccaagcatcccttccaagccctctcattcctctcacc(CACT)3	73	2315	2387	GTGTGGTGCTTTTGGCTGAG	59.969	ACGGATGATGGATCGTGCTC	59.967	146
TRINITY_Contig12965	1	c	(CAC)4ccccagccacgcccaagcatcccttccaagccctctcattcctctcacc(CACT)3	73	2330	2402	GTGTGGTGCTTTTGGCTGAG	59.969	ACGGATGATGGATCGTGCTC	59.967	146
TRINITY_Contig12967	1	c	(GAG)5atctcttcctcagcc(GAG)4gaccagtccgacgccgccgggtccggatctggcgaggagggggacgaggacgaggaggacgccgcggcacctccggcggg(GGA)4ggctgagggagaggggcagcagggggatgaggaggaggtcgatgaggaggagatcgaggccgtcactaccggcgccggcgcc(GAG)4	228	163	390	CCGCTACAAGAACCCATGGT	60.035	ACAAGGGAGACATGCGGAAG	60.036	161
TRINITY_Contig12967	2	p3	(AGA)4	12	530	541	CCGCTACAAGAACCCATGGT	60.035	ACAAGGGAGACATGCGGAAG	60.036	161
TRINITY_Contig12968	1	p3	(GCT)5	15	1954	1968	AGCAGAAGAGAGAGGGGGAG	60.032	GGGAGGAAAGGAAACCGAGG	60.035	139
TRINITY_Contig12968	2	p3	(CGG)5	15	2297	2311	AGCAGAAGAGAGAGGGGGAG	60.032	GGGAGGAAAGGAAACCGAGG	60.035	139
TRINITY_Contig12969	1	p5	(AGGGA)3	15	2082	2096	GAAGGGAAGAAGACCGGTGG	60.036	CTGTTGCCTGTTGGATCCCT	59.961	130
TRINITY_Contig12974	1	p3	(CCG)4	12	75	86	ATTCAAGAGGACCAAGCCGG	60.035	CTACGACATCAGAGGGCAC	59.897	272
TRINITY_Contig12974	2	p3	(GAC)4	12	973	984	ATTCAAGAGGACCAAGCCGG	60.035	CTACGACATCAGAGGGCAC	59.897	272
TRINITY_Contig12975	1	p3	(CCG)4	12	75	86	CTCTCGTAGTCGTCCTTCGC	59.973	GAAGAAGAAGCGCTCCCTGT	60.037	274
TRINITY_Contig12975	2	p3	(GAC)4	12	887	898	CTCTCGTAGTCGTCCTTCGC	59.973	GAAGAAGAAGCGCTCCCTGT	60.037	274
TRINITY_Contig12976	1	p3	(CCG)4	12	75	86	CTCTCGTAGTCGTCCTTCGC	59.973	GAAGAAGAAGCGCTCCCTGT	60.037	274
TRINITY_Contig12976	2	p3	(GAC)4	12	982	993	CTCTCGTAGTCGTCCTTCGC	59.973	GAAGAAGAAGCGCTCCCTGT	60.037	274
TRINITY_Contig12978	1	p6	(GCGCCG)3	18	114	131	CCCGTCATGATGTACTCCGG	59.968	AGGCCTCCAACATCTTGCTC	60.035	151
TRINITY_Contig12978	2	p3	(CCG)5	15	1314	1328	CCCGTCATGATGTACTCCGG	59.968	AGGCCTCCAACATCTTGCTC	60.035	151
TRINITY_Contig12978	3	p3	(GTG)4	12	1500	1511	CCCGTCATGATGTACTCCGG	59.968	AGGCCTCCAACATCTTGCTC	60.035	151
TRINITY_Contig12979	1	c	(AG)7ttcgctctggtgggatccaggcgagccctaaaaaaccactaaaaattgatgctcgtcctttgatgatggatt(AAAG)4	102	94	195	TTCGCCGCCAAAAACAAGAG	59.97	GTCAGATCGAACGGCATTGC	59.973	200
TRINITY_Contig12981	1	p3	(TCG)4	12	1166	1177	CTGCAGGAGGAAGTGGAGTG	60.037	GGTGAGCAGCTGAAGACAGT	59.965	208
TRINITY_Contig12982	1	p3	(ATT)4	12	593	604	TGGATGGAGTGGGGGATCAT	60.029	TGGGACAAGTTCTTCGACGG	59.967	156
TRINITY_Contig12982	2	p3	(TCG)4	12	1673	1684	TGGATGGAGTGGGGGATCAT	60.029	TGGGACAAGTTCTTCGACGG	59.967	156
TRINITY_Contig12983	1	p3	(CCG)6	18	1263	1280	AGAACCTGGTGAGCGAGTTG	59.966	CGAGGTTCACCAGGATGGAG	59.823	141
TRINITY_Contig12983	2	p4	(AGGA)3	12	1524	1535	AGAACCTGGTGAGCGAGTTG	59.966	CGAGGTTCACCAGGATGGAG	59.823	141
TRINITY_Contig12984	1	p3	(TCG)6	18	1166	1183	CGTGCTGCTGCTTAAGGTTC	59.833	CCGCCGCGTGCAAATAAATA	59.972	190

TRINITY_Contig12984	2	p3	(CGG)4	12	1371	1382	CGTGCTGCTGCTTAAGGTTC	59.833	CCGCCGCGTGCAAATAAATA	59.972	190
TRINITY_Contig12984	3	p4	(GGGC)3	12	1705	1716	CGTGCTGCTGCTTAAGGTTC	59.833	CCGCCGCGTGCAAATAAATA	59.972	190
TRINITY_Contig12985	1	p3	(AGA)4	12	2557	2568	TCAGCAACCGCACTTCTGAT	59.965	GTACACGGAGGTTCTTGGCA	59.966	158
TRINITY_Contig12986	1	p3	(TCT)4	12	226	237	TGTGCAAAGACCACGCTTTG	59.9	GCTGCTTTGCAACAAGCTCT	59.968	106
TRINITY_Contig12986	2	p3	(CTC)6	18	528	545	TGTGCAAAGACCACGCTTTG	59.9	GCTGCTTTGCAACAAGCTCT	59.968	106
TRINITY_Contig12986	3	c	(CT)8gccccccctccctcgagttttttgttctcttctcccagctcaccccacc(CT)8	78	1198	1275	TGTGCAAAGACCACGCTTTG	59.9	GCTGCTTTGCAACAAGCTCT	59.968	106
TRINITY_Contig12988	1	p3	(CGC)4	12	896	907	GGAAGGACTCAGTGACGTGG	60.038	CCATCTCCATCCGCATCCTC	60.037	116
TRINITY_Contig12988	2	c	(GCC)4cctgcccgcttctcgacgcccatcgatcttctcgacggcccttccccgcccttttttctccaaatcgca(GTCCCG)3	97	1283	1379	GGAAGGACTCAGTGACGTGG	60.038	CCATCTCCATCCGCATCCTC	60.037	116
TRINITY_Contig12989	1	p4	(GGCG)3	12	1768	1779	GACGCCCTTGGTGATGTGTA	60.037	TTCTTTTGCCACACCGCATG	59.968	136
TRINITY_Contig12989	2	p3	(CCG)6	18	1918	1935	GACGCCCTTGGTGATGTGTA	60.037	TTCTTTTGCCACACCGCATG	59.968	136
TRINITY_Contig12989	3	p3	(CCG)4	12	3410	3421	GACGCCCTTGGTGATGTGTA	60.037	TTCTTTTGCCACACCGCATG	59.968	136
TRINITY_Contig12990	1	p3	(TCG)5	15	145	159	CATTGCGCCCTTGAAGTAGC	59.901	GACTTGGTGCACCATGGAGA	59.963	256
TRINITY_Contig12991	1	p4	(TTTA)3	12	137	148	GCTTTGCAATGGATGTGGCA	60.037	AGTTGCAGTACGGGTGACAG	59.966	186
TRINITY_Contig12991	2	p4	(CGGC)3	12	1198	1209	GCTTTGCAATGGATGTGGCA	60.037	AGTTGCAGTACGGGTGACAG	59.966	186
TRINITY_Contig12992	1	c	(CCG)6(GCG)4	30	79	108	TGCGAATAGGATCCCGGTTG	59.894	TAAGATCGCGCGAACACCAT	60.179	133
TRINITY_Contig12992	2	c	(GCC)5tccgatcgcgaggcgagggagaagcgggggggtttggcgggcgcggggcagcaactaaccgtgtggggatgggttgacgtggatggatac(GTGCG)3	119	225	343	TGCGAATAGGATCCCGGTTG	59.894	TAAGATCGCGCGAACACCAT	60.179	133
TRINITY_Contig12993	1	p4	(GGCT)3	12	3626	3637	ATTGGCCCCTTTACCCATG	60.031	CCTACGTGTGTGGCTTCACT	59.966	270
TRINITY_Contig12994	1	p6	(CTGCCG)3	18	79	96	TTCGATCCAGCTGTATCGCC	59.968	GCATGCCATTGACAAAGGCA	60.037	220
TRINITY_Contig12996	1	p3	(CCG)5	15	199	213	AGACCACTCCCCAGATCTCC	60.032	ATGACTCCTGGTGGCAATGG	60.034	228
TRINITY_Contig12996	2	c	(GA)8aaaaaaaaaggaaaggcgagcaagaaagagaagcaagcaaaccaaaccccaaggaggccaagacgcggacggca(GCAG)3ccatgccgcggtcgccgtctccatctccgtcgccgtcgccggagaggcaccaccaccaacccgcgcgccggagggtcggcgccgcctcgtcccagatgct(CCA)4	215	448	662	AGACCACTCCCCAGATCTCC	60.032	ATGACTCCTGGTGGCAATGG	60.034	228
TRINITY_Contig12996	3	p3	(CGG)4	12	849	860	AGACCACTCCCCAGATCTCC	60.032	ATGACTCCTGGTGGCAATGG	60.034	228
TRINITY_Contig12997	1	c	(AGT)6aacaattaattaaaatctcttgtctcttcttgtctcgaatttgtttgctgatcgtgg(CGA)4	87	104	190	TAGCTGGAACCCTATCCCCC	60.105	GGAGTACCCCGTGATGTTGG	60.108	229
TRINITY_Contig12997	2	c	(GGT)4gaccatagcggagttggccgacggcaagcggccaagggaccggacgagctcgcgcggcgcttgctcgcggcc(GCG)4	97	665	761	TAGCTGGAACCCTATCCCCC	60.105	GGAGTACCCCGTGATGTTGG	60.108	229
TRINITY_Contig12997	3	p3	(GCC)5	15	1092	1106	TAGCTGGAACCCTATCCCCC	60.105	GGAGTACCCCGTGATGTTGG	60.108	229
TRINITY_Contig12997	4	p3	(CGC)4	12	1541	1552	TAGCTGGAACCCTATCCCCC	60.105	GGAGTACCCCGTGATGTTGG	60.108	229
TRINITY_Contig12998	1	p4	(AGCA)3	12	896	907	GGTACTGCTCCTTCACCCAC	60.036	CCTCCATGGGTTCCAAGGAC	60.034	231
TRINITY_Contig13000	1	p3	(CGG)4	12	250	261	TTCATGGACACGCATGGTCA	59.964	AGAGTTCTCCAGGCTCCACA	60.179	141
TRINITY_Contig13001	1	p5	(CACTC)5	25	55	79	TCTCCCCGATTCACTCCACT	59.959	GAACTGGCCGTTGATCAGGA	60.036	278
TRINITY_Contig13001	2	p3	(CGC)4	12	752	763	TCTCCCCGATTCACTCCACT	59.959	GAACTGGCCGTTGATCAGGA	60.036	278
TRINITY_Contig13002	1	p3	(CGG)4	12	386	397	TTGCAGTGTGGTCCTCGTAC	59.967	ACTACATCATCTACGCCGCG	60.04	132
TRINITY_Contig13002	2	p3	(CCG)4	12	1032	1043	TTGCAGTGTGGTCCTCGTAC	59.967	ACTACATCATCTACGCCGCG	60.04	132
TRINITY_Contig13002	3	p3	(GTA)5	15	1291	1305	TTGCAGTGTGGTCCTCGTAC	59.967	ACTACATCATCTACGCCGCG	60.04	132
TRINITY_Contig13003	1	p5	(CCTCG)4	20	86	105	GATGGAACGGTGCTCCTGAA	60.036	ACACGCTTCTCTTCAGGAGC	60.038	274
TRINITY_Contig13003	2	p3	(CCG)4	12	240	251	GATGGAACGGTGCTCCTGAA	60.036	ACACGCTTCTCTTCAGGAGC	60.038	274
TRINITY_Contig13005	1	p3	(CGC)6	18	3048	3065	TCCTGCTAGCCTTGCTGAAC	60.036	TTGCAGGGGAGGTTGTTTGT	60.033	271
TRINITY_Contig13005	2	p3	(CCG)5	15	3199	3213	TCCTGCTAGCCTTGCTGAAC	60.036	TTGCAGGGGAGGTTGTTTGT	60.033	271
TRINITY_Contig13005	3	p4	(TAGC)3	12	3465	3476	TCCTGCTAGCCTTGCTGAAC	60.036	TTGCAGGGGAGGTTGTTTGT	60.033	271
TRINITY_Contig13006	1	p2	(AG)6	12	148	159	CGTCGCTCGGAGACATCTAC	60.042	TGTATTCATTGCTGTGCGCG	59.903	138
TRINITY_Contig13006	2	p3	(CGC)5	15	287	301	CGTCGCTCGGAGACATCTAC	60.042	TGTATTCATTGCTGTGCGCG	59.903	138
TRINITY_Contig13006	3	p2	(GT)6	12	787	798	CGTCGCTCGGAGACATCTAC	60.042	TGTATTCATTGCTGTGCGCG	59.903	138
TRINITY_Contig13007	1	p3	(CGG)5	15	13	27	GGAGGAGGAGGAGGAGAAGG	60.106	GGTCGTAGTAGTCGCCGAAG	59.973	173
TRINITY_Contig13007	2	c	(GCG)4acagcagcgccgccggtac(AGG)5	46	153	198	GGAGGAGGAGGAGGAGAAGG	60.106	GGTCGTAGTAGTCGCCGAAG	59.973	173
TRINITY_Contig13008	1	p3	(GCG)6	18	735	752	TATCACTTGGAACGGGGCAC	60.036	AAAGCCCACCAAGGATACGG	60.034	268
TRINITY_Contig13009	1	p3	(GCG)6	18	745	762	CCAGCGTTTCCTCCTCCTTT	59.963	AAAGCCCACCAAGGATACGG	60.034	183
TRINITY_Contig13010	1	p3	(AGG)4	12	14	25	AGGAGGAGGAAGGAGCAGGAG	60.032	TGTACCCTTCTTGCGGAAC	59.964	100
TRINITY_Contig13012	1	p3	(GCC)4	12	47	58	TGGGTTGAATCGTTCGCTGA	59.966	TCAGCCTCTCTTTGCCCATG	60.035	194
TRINITY_Contig13013	1	p3	(CGG)4	12	1954	1965	GGTCGCCCGATCATCTGAAT	59.966	GCTCGAACCAGGAGAAACGA	60.039	175
TRINITY_Contig13014	1	c	(CAGAGG)4ccaaaggggaaggggaaatccgcctcgatttcctcgacgccaagcaacccccctcgtcgcctcgcccgcccgtctcct(TCC)4	114	24	137	TGTTTCGCAAAGACGTGCAG	59.973	CACTAGCAGTTGTCCTGGCA	59.965	218
TRINITY_Contig13014	2	p3	(GCA)4	12	651	662	TGTTTCGCAAAGACGTGCAG	59.973	CACTAGCAGTTGTCCTGGCA	59.965	218
TRINITY_Contig13014	3	p4	(TATC)3	12	4497	4508	TGTTTCGCAAAGACGTGCAG	59.973	CACTAGCAGTTGTCCTGGCA	59.965	218
TRINITY_Contig13016	1	p3	(TGC)4	12	147	158	GGGACTGCAAAACACCGAAC	59.97	CAGGTTCGGGGTTTAGCAGT	59.964	230
TRINITY_Contig13016	2	p3	(CTG)4	12	353	364	GGGACTGCAAAACACCGAAC	59.97	CAGGTTCGGGGTTTAGCAGT	59.964	230
TRINITY_Contig13018	1	p5	(TCTTT)3	15	70	84	GTGCTTTCACTGCCTGTGTG	59.97	GCTGTTTACCGTTACCCCCA	59.963	237
TRINITY_Contig13020	1	p5	(GATCG)3	15	223	237	GGGTCCAGGGACTCTGAGAT	60.032	CCAGGTCATCGCTCGAGTAC	59.97	223
TRINITY_Contig13021	1	p5	(GCCAT)3	15	403	417	CGTGAGAAAGCAACAACGCA	59.973	ACGAAGTCGCTGTGGTTGAT	59.966	274
TRINITY_Contig13021	2	p3	(GGA)4	12	523	534	CGTGAGAAAGCAACAACGCA	59.973	ACGAAGTCGCTGTGGTTGAT	59.966	274
TRINITY_Contig13021	3	p3	(CGG)4	12	2684	2695	CGTGAGAAAGCAACAACGCA	59.973	ACGAAGTCGCTGTGGTTGAT	59.966	274
TRINITY_Contig13023	1	p3	(GAG)4	12	1379	1390	CTGCAGGCTCCGTATGGTAG	59.967	TCAACCTCTCCGCAAACCTC	59.965	221
TRINITY_Contig13023	2	p3	(AGG)5	15	1950	1964	CTGCAGGCTCCGTATGGTAG	59.967	TCAACCTCTCCGCAAACCTC	59.965	221
TRINITY_Contig13025	1	p5	(GCCGC)3	15	62	76	GGGAGCCCTAAAAGTGGGAC	60.034	CCCTTCGGAGTCAGTGGAAC	60.037	134
TRINITY_Contig13025	2	p3	(CCG)5	15	178	192	GGGAGCCCTAAAAGTGGGAC	60.034	CCCTTCGGAGTCAGTGGAAC	60.037	134
TRINITY_Contig13026	1	p4	(CGCC)3	12	94	105	CAGCTGCTCGATCGATCCAT	60.038	TGCCTGCCTCCTCCTAATCT	60.031	168
TRINITY_Contig13026	2	p3	(CCG)5	15	300	314	CAGCTGCTCGATCGATCCAT	60.038	TGCCTGCCTCCTCCTAATCT	60.031	168
TRINITY_Contig13026	3	p4	(AGCT)3	12	560	571	CAGCTGCTCGATCGATCCAT	60.038	TGCCTGCCTCCTCCTAATCT	60.031	168
TRINITY_Contig13027	1	p2	(GA)7	14	1049	1062	AACTTCGAGCTCCAGAAGGC	60.037	CATCGAGCGCGTTCTTGAAG	59.976	112
TRINITY_Contig13029	1	p4	(TGAT)3	12	8	19	CTTGGTGATTGCCGCAAACA	59.968	CACTACGAGGAAGCCGATCC	59.969	261
TRINITY_Contig13030	1	p3	(CCG)4	12	176	187	AAAGCCTCCATCCCTTCAGC	60.034	GCATCCGTAGTGGTTGTGGA	60.037	125
TRINITY_Contig13032	1	p3	(TGC)4	12	393	404	GACCAGGTGGAGAGGGATCT	60.032	CGGCCTCACTCACTGTCTTT	59.966	121
TRINITY_Contig13032	2	p3	(GCG)4	12	1974	1985	GACCAGGTGGAGAGGGATCT	60.032	CGGCCTCACTCACTGTCTTT	59.966	121
TRINITY_Contig13034	1	p4	(TGAG)3	12	1877	1888	AGGGATACGGAAGAGCCCTT	60.031	ATGCCAACCCGTGCATAGAA	60.035	244
TRINITY_Contig13035	1	p3	(CCG)5	15	1967	1981	TCAGCGAAACGACCATTGGA	59.966	TGAGATCCTCCCGGTTAGCA	60.033	232
TRINITY_Contig13035	2	c	(GCT)4g(CGC)4	25	2144	2168	TCAGCGAAACGACCATTGGA	59.966	TGAGATCCTCCCGGTTAGCA	60.033	232
TRINITY_Contig13036	1	p4	(CCCG)3	12	756	767	GCGTCCTGAAAAAGCGATCC	59.903	CCGATCCCTTTCTCTCGAGC	59.969	149
TRINITY_Contig13036	2	p3	(CGC)4	12	2260	2271	GCGTCCTGAAAAAGCGATCC	59.903	CCGATCCCTTTCTCTCGAGC	59.969	149
TRINITY_Contig13038	1	c	(GCAGC)3catgggaaagaagcacgccgcaggtagcggcggcggctggttcgccgccgtcaggaaggtgttccggccgtcgtcatcatcgtcctccgccaccacctca(AAGGAC)3	133	71	203	TGGCGTTGACAAAGTCCAGT	60.107	TGGCCAAGTATACGTGCGTT	60.037	147
TRINITY_Contig13038	2	c	(GGT)4tgcccgcgggaccacgaggaggccgacgat(GAC)5	57	408	464	TGGCGTTGACAAAGTCCAGT	60.107	TGGCCAAGTATACGTGCGTT	60.037	147
TRINITY_Contig13038	3	c	(CTC)4ccgcgccgcgacccctccggccacccgcccctccttttcccttcccggagcaggcggacg(CCA)4gccgcgcctcgacctggcgttggtgggcgaccatcaggacgccagcgacgacggcgaggtggcggaggac(CTG)4	165	777	941	TGGCGTTGACAAAGTCCAGT	60.107	TGGCCAAGTATACGTGCGTT	60.037	147
TRINITY_Contig13041	1	p3	(TCC)5	15	389	403	TTCGGTTGGATTCCTCCTGC	60.035	TGGGATTGGGGAGAATGTGC	60.033	260
TRINITY_Contig13041	2	p6	(GGACGG)3	18	608	625	TTCGGTTGGATTCCTCCTGC	60.035	TGGGATTGGGGAGAATGTGC	60.033	260
TRINITY_Contig13044	1	p3	(GGC)4	12	941	952	AGCACCACCTACCTAGAGCA	59.959	CTTCAGCGTCGCATAAGCAC	59.974	239
TRINITY_Contig13044	2	p3	(GGC)4	12	1094	1105	AGCACCACCTACCTAGAGCA	59.959	CTTCAGCGTCGCATAAGCAC	59.974	239
TRINITY_Contig13044	3	p4	(CTCC)3	12	1456	1467	AGCACCACCTACCTAGAGCA	59.959	CTTCAGCGTCGCATAAGCAC	59.974	239
TRINITY_Contig13045	1	p3	(GCG)4	12	449	460	AGGTCCACCTGTCCCTATCC	60.031	CAAGAAGGAGGAGCTGGTGG	60.036	172
TRINITY_Contig13045	2	p3	(CTT)4	12	708	719	AGGTCCACCTGTCCCTATCC	60.031	CAAGAAGGAGGAGCTGGTGG	60.036	172
TRINITY_Contig13046	1	p3	(CGC)5	15	304	318	GGCTTTCATTGCTCGCGATT	59.901	GACGACGGAGGAAACAAGCT	60.32	189
TRINITY_Contig13050	1	c	(CCG)4gcggcaagaggaaggcggccgacgtcgacgagctcgaggccgtcac(CGA)4	70	126	195	TACGATACGACATGTGCGGG	59.97	GCAGGAGAAGTGTCAGCCTT	59.964	280

TRINITY_Contig13050	2	p3	(CCG)4	12	541	552	TACGATACGACATGTGCGGG	59.97	GCAGGAGAAGTGTCAGCCTT	59.964	280
TRINITY_Contig13051	1	p3	(CAC)4	12	207	218	GCAGATACGGAAGGCGAGAA	59.898	CATCGACGACATCCAGAGGG	59.969	261
TRINITY_Contig13051	2	p6	(ACTCGT)3	18	1388	1405	GCAGATACGGAAGGCGAGAA	59.898	CATCGACGACATCCAGAGGG	59.969	261
TRINITY_Contig13054	1	p3	(CTG)4	12	117	128	AACCCACCGAATCCTTTGCT	59.888	CCCCGGCTAGTTGATCGATC	60.038	173
TRINITY_Contig13056	1	p3	(CAG)5	15	616	630	TCCATCCCACCGTCAATGTG	60.036	ATCTGTATGGCAGTAGCGGC	59.966	129
TRINITY_Contig13057	1	p3	(CGG)6	18	263	280	AGACGGTCACACAGCTCTTG	59.967	AACACATGGCCCCTGAAGAG	59.961	191
TRINITY_Contig13061	1	c	(CGC)4cggcctcgcccgcctgcttgatgcca(CCG)4	50	1917	1966	AGTAGAACGCCACCAGGTTG	59.965	CTGAAGCTGTCCGTGTTCCT	59.966	235
TRINITY_Contig13061	2	p3	(GCT)7	21	2099	2119	AGTAGAACGCCACCAGGTTG	59.965	CTGAAGCTGTCCGTGTTCCT	59.966	235
TRINITY_Contig13064	1	c	(GCG)4agcatccccatggctcctctt(CTG)4	45	2125	2169	CAGGAACACCAGTTTGCACG	59.971	CAAGCTTCACTCCTGTCCGT	59.966	160
TRINITY_Contig13064	2	p3	(GGC)4	12	2443	2454	CAGGAACACCAGTTTGCACG	59.971	CAAGCTTCACTCCTGTCCGT	59.966	160
TRINITY_Contig13065	1	p4	(TAGC)3	12	95	106	CGACGTGACAGTGACAGGAA	59.97	TGAACTATGGTGCCGTGGAC	60.037	220
TRINITY_Contig13065	2	p3	(GCA)5	15	619	633	CGACGTGACAGTGACAGGAA	59.97	TGAACTATGGTGCCGTGGAC	60.037	220
TRINITY_Contig13065	3	p3	(CGC)4	12	1136	1147	CGACGTGACAGTGACAGGAA	59.97	TGAACTATGGTGCCGTGGAC	60.037	220
TRINITY_Contig13066	1	p4	(ATAC)4	16	217	232	TTGACAAGCATGGGGAGGTC	59.962	GGATCTCCCATGTAGCTGCC	59.964	265
TRINITY_Contig13066	2	p3	(GAC)4	12	380	391	TTGACAAGCATGGGGAGGTC	59.962	GGATCTCCCATGTAGCTGCC	59.964	265
TRINITY_Contig13066	3	p3	(GGC)4	12	512	523	TTGACAAGCATGGGGAGGTC	59.962	GGATCTCCCATGTAGCTGCC	59.964	265
TRINITY_Contig13069	1	c	(CCG)4ctgcgggatgctccttggccgcgggctcgacag(CCT)4	57	1762	1818	ACGAATCCGGTTGTCCTGAC	60.038	CCTGCACGACCTGAAGTTCT	59.966	186
TRINITY_Contig13073	1	p3	(GTT)4	12	611	622	GCTGCGATTGCCTGAATCTG	59.971	GTGCAGCTACCATGACCACT	60.036	178
TRINITY_Contig13074	1	c*	(AGTTGT)2(A<GTTGT>TGTTGTTGCTGCAAGTAGAGAGCAGGGTTCGCCACAGCCAGTTGGTTGAACGGAAGCAGTTGTTGTTGCTGCCAGTATGCTGCGGGGTTCACAATGGCCAGCTGGTTGAATAGGTGCGAGAACACCTGTTGTTGCTGCTGCACTGCTGCGATTGCCACGGCCAAGGGAGAGATCGCTGTCGCGAAAGCCTGCTGTTGCCTGTAGGATTGCACGAATGAGTGTGCAGATCCCACGGCTGTCAACGGTGAGAAGTATTGTGGAACAGTGTCCATGCTAGCAGCACTCATCAGCGGCGAAGAGCATTGAGGAATAAAGACCGCAGTAGCAGCACTAGACGAAAGAGCTATGAGAGCAAGAAGAACAAATATCTTGGCTGCCATTGTTGCTAAGAGTTTGATATTGTGCTTACTAAGATTCTA)(G	25	598	622	TATGCTGCGGGGTTCACAAT	60.035	GACAGCGATCTCTCCCTTGG	59.896	105
TRINITY_Contig13075	1	p3	(CAG)4	12	157	168	ATCGAGGACGGGATGAGGAA	60.106	TGTCCGTCCACTTCAGATGC	60.037	100
TRINITY_Contig13075	2	p4	(GGAG)3	12	320	331	ATCGAGGACGGGATGAGGAA	60.106	TGTCCGTCCACTTCAGATGC	60.037	100
TRINITY_Contig13075	3	p4	(AACC)4	16	993	1008	ATCGAGGACGGGATGAGGAA	60.106	TGTCCGTCCACTTCAGATGC	60.037	100
TRINITY_Contig13075	4	p4	(TCGT)3	12	1182	1193	ATCGAGGACGGGATGAGGAA	60.106	TGTCCGTCCACTTCAGATGC	60.037	100
TRINITY_Contig13075	5	p4	(GCCC)3	12	1785	1796	ATCGAGGACGGGATGAGGAA	60.106	TGTCCGTCCACTTCAGATGC	60.037	100
TRINITY_Contig13076	1	p3	(CAG)4	12	157	168	GCATCTGAAGTGGACGGACA	60.037	AGAACGATGAAAGCAGGCGA	60.037	169
TRINITY_Contig13076	2	p4	(GGAG)3	12	320	331	GCATCTGAAGTGGACGGACA	60.037	AGAACGATGAAAGCAGGCGA	60.037	169
TRINITY_Contig13076	3	p4	(AACC)4	16	993	1008	GCATCTGAAGTGGACGGACA	60.037	AGAACGATGAAAGCAGGCGA	60.037	169
TRINITY_Contig13076	4	p4	(GCCC)3	12	1687	1698	GCATCTGAAGTGGACGGACA	60.037	AGAACGATGAAAGCAGGCGA	60.037	169
TRINITY_Contig13077	1	p6	(CCTGCG)3	18	654	671	ATGCAAGGTCAGCTCAGCTT	59.962	GCTGAGTAATTGGCCAAGCG	59.901	183
TRINITY_Contig13082	1	c	(CAC)4(CAG)5	27	2	28	AGCTCTCTACAGATGGGGCA	60.032	TGCTTCCACATACAGCACGT	59.965	127
TRINITY_Contig13082	2	p3	(CTG)4	12	667	678	AGCTCTCTACAGATGGGGCA	60.032	TGCTTCCACATACAGCACGT	59.965	127
TRINITY_Contig13084	1	p3	(CTG)6	18	842	859	ATATCGAGGCTGGTCGCAAG	59.967	CTGCTGGGTGGATCCTGTTT	59.961	258
TRINITY_Contig13085	1	p4	(CAGC)4	16	522	537	AAAACAATTCCAACCCGCGG	59.967	CGGCCAATTAGATTTGCGGG	59.969	249
TRINITY_Contig13088	1	p3	(GCC)4	12	26	37	CATCGAGCCGACGAAGGTTA	59.9	CTCAGATGTGGTGAAGGCGT	60.037	183
TRINITY_Contig13089	1	p3	(CAA)4	12	1259	1270	AATCACAAGCAGCCCATCGA	60.035	TGCTGCGTTCAAAGTGTTCG	59.973	253
TRINITY_Contig13090	1	p3	(CGA)4	12	3	14	GCACAAAACCCAAATCCCCC	59.964	TAGAATCACGCGGAGATGGC	59.968	128
TRINITY_Contig13090	2	p2	(CG)6	12	240	251	GCACAAAACCCAAATCCCCC	59.964	TAGAATCACGCGGAGATGGC	59.968	128
TRINITY_Contig13092	1	p3	(GCG)5	15	250	264	CAGGAAGTCCTTGAGCCTGG	60.036	CTTCTACGTCTACGCCGTCC	59.973	103
TRINITY_Contig13092	2	p6	(ACTTGG)4	24	926	949	CAGGAAGTCCTTGAGCCTGG	60.036	CTTCTACGTCTACGCCGTCC	59.973	103
TRINITY_Contig13093	1	p3	(CGG)4	12	104	115	ATCTCCTTGCATGCTGGCTT	60.033	TCCTAACAACATCAGCGCGT	60.038	251
TRINITY_Contig13093	2	p3	(GGC)4	12	240	251	ATCTCCTTGCATGCTGGCTT	60.033	TCCTAACAACATCAGCGCGT	60.038	251
TRINITY_Contig13096	1	p3	(CAA)4	12	1291	1302	GTGGCTGCAGAGTGGAGATT	60.036	GGAGGCCAAGATGGGTTTGA	59.96	101
TRINITY_Contig13100	1	p6	(AGCGGC)3	18	1162	1179	GGAGTCCAGGTCCAGGATCT	60.032	GTCGGGGCTCAAGAACTTCA	59.965	112
TRINITY_Contig13102	1	p4	(GGTC)3	12	1299	1310	CCGATCGTCCTCATCATGGG	60.038	TCGCCCACCTTACCAAATCC	60.035	237
TRINITY_Contig13102	2	p5	(GGAGC)3	15	1432	1446	CCGATCGTCCTCATCATGGG	60.038	TCGCCCACCTTACCAAATCC	60.035	237
TRINITY_Contig13103	1	p3	(CGG)4	12	489	500	ATCCTCTTCTCCGACTGCCT	60.033	CCTCATCAGAGCTAGCAGGC	59.966	155
TRINITY_Contig13103	2	p4	(ACCT)4	16	1587	1602	ATCCTCTTCTCCGACTGCCT	60.033	CCTCATCAGAGCTAGCAGGC	59.966	155
TRINITY_Contig13103	3	p4	(ATAC)3	12	1783	1794	ATCCTCTTCTCCGACTGCCT	60.033	CCTCATCAGAGCTAGCAGGC	59.966	155
TRINITY_Contig13105	1	c	(GCCT)3accaccaccacagtcaacaccggcg(GAGC)3	49	1688	1736	CGATCCTCATGTCAGGACGG	59.969	AATAGCCGATGCCGTGGTAG	59.966	236
TRINITY_Contig13106	1	p3	(CCG)4	12	287	298	CACCTCATCATCCTCTCGCC	59.967	GTTCCTGATGGTGTGGTCGT	59.965	252
TRINITY_Contig13107	1	p4	(CGAG)3	12	14	25	TGTTTGTGTGTCGACCACCA	60.036	TGACACCTACGAGGAGGTCC	60.323	248
TRINITY_Contig13109	1	p4	(ATGG)3	12	3927	3938	TCAGTCTGCGAAGGTGAACC	59.966	TCAGCCGAACGTCGTGTTAA	59.969	266
TRINITY_Contig13115	1	c	(TCC)5cctcgtcgcctcctccccgaccct(CTC)5	54	99	152	AAATCCTCCTCCTCCTCCCC	60.03	TGAGGAGGTGGAGGAGGAAG	59.958	181
TRINITY_Contig13115	2	p3	(CGC)4	12	1073	1084	AAATCCTCCTCCTCCTCCCC	60.03	TGAGGAGGTGGAGGAGGAAG	59.958	181
TRINITY_Contig13116	1	p4	(AAAC)3	12	1921	1932	CAAAGAAAGGGCACACTGGC	59.967	GTGGCCATCTCTATCACCGG	59.966	265
TRINITY_Contig13116	2	p3	(GCG)4	12	3143	3154	CAAAGAAAGGGCACACTGGC	59.967	GTGGCCATCTCTATCACCGG	59.966	265
TRINITY_Contig13117	1	p3	(GCG)6	18	817	834	AAAAGAAGTGCTTCGCTGCG	60.041	CCACATCAGACAGTGCACCT	59.964	229
TRINITY_Contig13119	1	p2	(GC)6	12	1647	1658	GCTACCAGCTTGTCCAGGTT	59.963	GGAAGCTGGTACAAGCTGGT	59.963	131
TRINITY_Contig13121	1	p6	(TCTTCC)3	18	425	442	TGCAAGAGCTTCACGTCTGT	59.895	AGAAGGCGCTGTTAGTGTCC	60.037	161
TRINITY_Contig13122	1	p2	(CT)6	12	34	45	TGTCCGTCGCCTACAAGAAC	60.039	CCTCGATCTTGGAGCGGTAC	59.969	138
TRINITY_Contig13122	2	c	(AGC)6cgtctcgacgcgccact(AGCG)3	47	256	302	TGTCCGTCGCCTACAAGAAC	60.039	CCTCGATCTTGGAGCGGTAC	59.969	138
TRINITY_Contig13124	1	p3	(GCC)7	21	288	308	TCATATCTTCCGGCTCGCAC	59.968	CCGCATCATTGCCGATCATC	59.833	164
TRINITY_Contig13124	2	p3	(CAG)4	12	588	599	TCATATCTTCCGGCTCGCAC	59.968	CCGCATCATTGCCGATCATC	59.833	164
TRINITY_Contig13125	1	p4	(TCCT)4	16	242	257	AAGTTCATCCAGAGGCAGCC	60.035	CGCTTATCTTCGGCAAAGCC	59.972	164
TRINITY_Contig13125	2	p3	(CGC)6	18	504	521	AAGTTCATCCAGAGGCAGCC	60.035	CGCTTATCTTCGGCAAAGCC	59.972	164
TRINITY_Contig13126	1	p3	(GCG)4	12	381	392	GGTGGATCTTGAACGGAGGG	60.108	CCACCCTCCGCTCAAGATTT	60.035	159
TRINITY_Contig13126	2	p3	(CCG)5	15	750	764	GGTGGATCTTGAACGGAGGG	60.108	CCACCCTCCGCTCAAGATTT	60.035	159
TRINITY_Contig13126	3	p3	(GGA)4	12	904	915	GGTGGATCTTGAACGGAGGG	60.108	CCACCCTCCGCTCAAGATTT	60.035	159
TRINITY_Contig13127	1	c	(ACG)5(AGG)6	33	1404	1436	TTCCAATCCCACTGACCACG	59.964	TGTGGCACTGCAGAGGTTAG	59.965	100
TRINITY_Contig13130	1	p4	(TCCA)3	12	218	229	GAGATGCTGTAGTACCCGCC	59.968	CAGGACAACACCACCTCCTC	59.965	101
TRINITY_Contig13130	2	p3	(AGC)4	12	1462	1473	GAGATGCTGTAGTACCCGCC	59.968	CAGGACAACACCACCTCCTC	59.965	101
TRINITY_Contig13131	1	p3	(CCG)4	12	226	237	CTTCTGAGCTCCCCAACCAG	60.036	CCCCCAAGTAGGTACCTCCA	59.956	180
TRINITY_Contig13131	2	p3	(GAG)4	12	451	462	CTTCTGAGCTCCCCAACCAG	60.036	CCCCCAAGTAGGTACCTCCA	59.956	180
TRINITY_Contig13133	1	p3	(GGC)5	15	173	187	CTCAGCTCAGCTCAGATCCG	59.969	CTATAAACAGCCGCCCCCTC	60.25	213
TRINITY_Contig13133	2	p5	(CAGCT)3	15	512	526	CTCAGCTCAGCTCAGATCCG	59.969	CTATAAACAGCCGCCCCCTC	60.25	213
TRINITY_Contig13133	3	c*	(GTGCC)3gcggtggggagggggcggctgtttatagccagccagtcagggacgccggagctgagctgagcc(GTG)5(GCGTC)3*	107	685	791	CTCAGCTCAGCTCAGATCCG	59.969	CTATAAACAGCCGCCCCCTC	60.25	213
TRINITY_Contig13136	1	p2	(CT)8	16	18	33	CCAACCAGCTAGCTTTCGGA	60.036	TAGAGGTCGATCTCGGCGAT	59.965	160
TRINITY_Contig13136	2	p3	(GCG)5	15	833	847	CCAACCAGCTAGCTTTCGGA	60.036	TAGAGGTCGATCTCGGCGAT	59.965	160
TRINITY_Contig13136	3	p6	(GTGGCG)3	18	1448	1465	CCAACCAGCTAGCTTTCGGA	60.036	TAGAGGTCGATCTCGGCGAT	59.965	160
TRINITY_Contig13136	4	p3	(GGC)5	15	1792	1806	CCAACCAGCTAGCTTTCGGA	60.036	TAGAGGTCGATCTCGGCGAT	59.965	160
TRINITY_Contig13136	5	p4	(CCTT)4	16	1995	2010	CCAACCAGCTAGCTTTCGGA	60.036	TAGAGGTCGATCTCGGCGAT	59.965	160
TRINITY_Contig13137	1	p5	(GTCTC)4	20	2890	2909	GCTCGAGGTCGGTCTTATGG	59.969	GAAGGCCGAGAAGGAGATCG	59.969	215
TRINITY_Contig13139	1	p3	(GAG)4	12	2012	2023	TATTGCACCTAAGCGGCCAA	60.035	ACAGTATCACCTTGCCCTGC	60.035	235
TRINITY_Contig13140	1	c	(GCG)4gaggccgggcggggccgcggcgggggcggaggagggggacggcggaggggacgacgtcggggccgcgccgagcccgcacgcggc(GAGGCG)3	114	1647	1760	GTACGACGACGAGGATTCCC	59.971	TCGAGGAGGAGGAAGTGGAG	60.034	104
TRINITY_Contig13141	1	p3	(ACC)4	12	1573	1584	CGACAGTCCCAGAGCAAAGT	59.966	GTGGGAGGATGCACCAGAAA	59.962	189
TRINITY_Contig13143	1	p3	(CGC)4	12	81	92	TAAGCCGGGCGTTACAGTTT	59.965	CATCCCACCCGCATAATCGA	59.965	137
TRINITY_Contig13148	1	p3	(CTC)5	15	228	242	TCGATGGTTTCTTCGTCGCA	60.039	AGTAAACCTCCGCAATCGCA	60.037	276

TRINITY_Contig13149	1	p3	(GCC)5	15	2574	2588	CTCGATCGGCTAGTTTCGCT	59.97	CGTGCTGTGATTCGTTAGCG	59.976	147
TRINITY_Contig13150	1	p3	(CGG)5	15	139	153	ACCAGATCTGCAAAGCCGAA	59.963	GTTGCGAAGTTGGCCACAAT	59.968	159
TRINITY_Contig13150	2	p6	(CGACGC)3	18	323	340	ACCAGATCTGCAAAGCCGAA	59.963	GTTGCGAAGTTGGCCACAAT	59.968	159
TRINITY_Contig13151	1	p3	(GCA)4	12	1895	1906	GCTTCCCGATCGAATCTGGT	59.895	TCAACTACGACAAGGCCACC	59.966	124
TRINITY_Contig13153	1	p3	(CCG)4	12	96	107	AACGCCGTCTACGTCACTTT	59.969	GTGACATCCTGGGTGGTCTG	60.036	183
TRINITY_Contig13155	1	p4	(ATTT)3	12	114	125	TCGATGGTGTCCCTGAGAGT	59.96	TGAGGAACATGGTGGACGTG	59.965	277
TRINITY_Contig13155	2	p4	(GCCG)3	12	489	500	TCGATGGTGTCCCTGAGAGT	59.96	TGAGGAACATGGTGGACGTG	59.965	277
TRINITY_Contig13156	1	p3	(CCT)4	12	684	695	TACGGCAGTACAGCACACAG	60.039	GCTGCACTTGTTCGTCGTTT	59.972	162
TRINITY_Contig13157	1	p3	(CGG)6	18	259	276	GGATGAGGAAGACGAGCTCG	59.97	CGACGGAACCCTAGGATTCG	59.97	183
TRINITY_Contig13157	2	c	(GGC)5gtgatgcagttcctccgaagcgc(CGG)4	50	530	579	GGATGAGGAAGACGAGCTCG	59.97	CGACGGAACCCTAGGATTCG	59.97	183
TRINITY_Contig13158	1	p3	(GAC)6	18	21	38	GGATCGAGGAAGACGACGAC	59.972	GTAGACGTATCCCTTGGGCG	59.968	158
TRINITY_Contig13159	1	p3	(AGC)5	15	87	101	ACATTGGTCTTCTCGCAGCA	59.965	TCCGAAAACGCATACCCACA	59.965	162
TRINITY_Contig13159	2	c	(CGG)4gcggatctgccggagagcggcgcggggagcgcggcgctagcccgcgggatctgacgccgcggcggccgctcggatcgagc(CGG)4ggggcggagggacgctgctcggggcgccggcgctcggctccggggacgagatagatagaaggaaaagattgcgcctggcttcaactt(CGG)4	203	317	519	ACATTGGTCTTCTCGCAGCA	59.965	TCCGAAAACGCATACCCACA	59.965	162
TRINITY_Contig13159	3	p3	(AGC)5	15	2062	2076	ACATTGGTCTTCTCGCAGCA	59.965	TCCGAAAACGCATACCCACA	59.965	162
TRINITY_Contig13160	1	p3	(CTG)4	12	862	873	TTGCACAAATCTGCCAAGGC	59.966	CGACAGTGAACCTGGAAGCT	59.966	220
TRINITY_Contig13160	2	c	(CAG)6aagcccaacccgtttggattcggctcggcgagcaggcc(GCA)5gtcgttcggttcgcagttc(CAG)6	108	5655	5762	TTGCACAAATCTGCCAAGGC	59.966	CGACAGTGAACCTGGAAGCT	59.966	220
TRINITY_Contig13160	3	c	(TGC)6tggaactgcgaaccgaacga(CTG)5cggcctgctcgccgagccgaatccaaacgggttgggctt(CTG)6	110	6023	6132	TTGCACAAATCTGCCAAGGC	59.966	CGACAGTGAACCTGGAAGCT	59.966	220
TRINITY_Contig13160	4	p3	(GCA)4	12	10907	10918	TTGCACAAATCTGCCAAGGC	59.966	CGACAGTGAACCTGGAAGCT	59.966	220
TRINITY_Contig13162	1	p6	(TCTCCC)3	18	2017	2034	CCGACTGCGAGTACGACTAC	59.974	GGTCGAGGACTTTGTCGTGT	59.968	141
TRINITY_Contig13162	2	p3	(TCT)5	15	2138	2152	CCGACTGCGAGTACGACTAC	59.974	GGTCGAGGACTTTGTCGTGT	59.968	141
TRINITY_Contig13163	1	p4	(GACC)3	12	366	377	GCCGATGCCAGAGATCTACC	60.038	GCAACGTTCAGCTTCAGCAA	59.971	106
TRINITY_Contig13164	1	p3	(CTG)4	12	1033	1044	CAAGGGGCAGTAGGACAACA	59.6	CGCATGCGTGTGTTTCGTAT	59.905	113
TRINITY_Contig13165	1	p3	(CCG)4	12	87	98	CGGACGAATCCAGAGAGAGC	59.97	GATCCATTGTTGCCGCATCC	59.969	211
TRINITY_Contig13165	2	p3	(GCT)6	18	403	420	CGGACGAATCCAGAGAGAGC	59.97	GATCCATTGTTGCCGCATCC	59.969	211
TRINITY_Contig13166	1	p3	(CGC)4	12	228	239	ACAACGTCTACTGCACCCTG	59.966	GTCCGTGATCACCACCTTGT	59.965	152
TRINITY_Contig13166	2	p3	(ACG)4	12	1683	1694	ACAACGTCTACTGCACCCTG	59.966	GTCCGTGATCACCACCTTGT	59.965	152
TRINITY_Contig13168	1	p3	(CCA)4	12	236	247	ATGTAGGGGCATCAGGGACT	60.03	AACAACGTGGGTCCTTGTGT	60.035	109
TRINITY_Contig13168	2	p3	(CCG)4	12	384	395	ATGTAGGGGCATCAGGGACT	60.03	AACAACGTGGGTCCTTGTGT	60.035	109
TRINITY_Contig13168	3	p4	(CGGC)3	12	2054	2065	ATGTAGGGGCATCAGGGACT	60.03	AACAACGTGGGTCCTTGTGT	60.035	109
TRINITY_Contig13170	1	c	(CTG)6cagtgcctgctgcatcaggagcgc(CTG)6	60	1849	1908	CAACAGCAGGCTGGTATGGA	60.035	GGGCAATCGAGGAAATTCGC	59.971	213
TRINITY_Contig13171	1	p3	(GAG)4	12	102	113	GGTCACCGAGTTCGTTTCCT	59.967	AGACGGGATCAGGAAAGGGA	59.957	109
TRINITY_Contig13171	2	p4	(GAGG)3	12	504	515	GGTCACCGAGTTCGTTTCCT	59.967	AGACGGGATCAGGAAAGGGA	59.957	109
TRINITY_Contig13173	1	p3	(GCT)4	12	707	718	GGCCTGGGAATCCGTGTTTA	60.035	GGAGACGGCGAGGATAAAGG	59.968	121
TRINITY_Contig13176	1	p3	(ATA)4	12	75	86	AAGGAGGGGAGGATAGACCG	59.811	CAGTTCAGGCCAGGCTGTTA	59.963	170
TRINITY_Contig13176	2	p3	(GCC)4	12	334	345	AAGGAGGGGAGGATAGACCG	59.811	CAGTTCAGGCCAGGCTGTTA	59.963	170
TRINITY_Contig13176	3	p3	(CGG)4	12	504	515	AAGGAGGGGAGGATAGACCG	59.811	CAGTTCAGGCCAGGCTGTTA	59.963	170
TRINITY_Contig13179	1	p3	(CCG)5	15	1315	1329	ATGAGTCACTGCTCTGGTGC	60.037	TGGCGTTGGCTTCACAAAAG	59.898	249
TRINITY_Contig13180	1	p4	(TTCC)3	12	10	21	ACGCTCATGCTGTAACCGAA	60.038	GTGAAGCCCCACAGATTCCA	59.962	226
TRINITY_Contig13180	2	p3	(GCG)4	12	369	380	ACGCTCATGCTGTAACCGAA	60.038	GTGAAGCCCCACAGATTCCA	59.962	226
TRINITY_Contig13181	1	p3	(TCA)6	18	225	242	ACGCTAACTCGAGCAACCAA	59.967	CAGGTGGTTCGTTCTCCTCC	60.037	161
TRINITY_Contig13181	2	p3	(CGC)5	15	603	617	ACGCTAACTCGAGCAACCAA	59.967	CAGGTGGTTCGTTCTCCTCC	60.037	161
TRINITY_Contig13182	1	c	(TGA)4cgacagcgaccatgaaggcag(TGA)6	51	1070	1120	GGTCCAATCCCAAGCCAGAA	59.96	GCTTCATGCCACGCCTAATG	59.97	121
TRINITY_Contig13184	1	p4	(TCCC)3	12	139	150	TCATCTCGTGTCATCTCGCG	59.972	TACTCCCAGCAGGCATCTCT	60.032	213
TRINITY_Contig13184	2	p4	(TGTA)3	12	2340	2351	TCATCTCGTGTCATCTCGCG	59.972	TACTCCCAGCAGGCATCTCT	60.032	213
TRINITY_Contig13185	1	p4	(AACC)3	12	167	178	GTGGTAGAGGAGTGGGCAAC	60.036	TTCCCCGTAGTGCAGTTCAC	59.966	222
TRINITY_Contig13185	2	p6	(GCAACG)3	18	281	298	GTGGTAGAGGAGTGGGCAAC	60.036	TTCCCCGTAGTGCAGTTCAC	59.966	222
TRINITY_Contig13186	1	p2	(GT)8	16	1067	1082	CCAGTAAGCCAGTCTGTGCA	59.965	CCCCCGCTTTCATCTTCCTT	60.034	203
TRINITY_Contig13186	2	c	(GGGA)3gga(AG)6	27	1716	1742	CCAGTAAGCCAGTCTGTGCA	59.965	CCCCCGCTTTCATCTTCCTT	60.034	203
TRINITY_Contig13186	3	p3	(GCA)4	12	2003	2014	CCAGTAAGCCAGTCTGTGCA	59.965	CCCCCGCTTTCATCTTCCTT	60.034	203
TRINITY_Contig13188	1	c	(CCT)5gctcctgcacctcct(CTC)4	42	237	278	AGCGCAGTCTACCAAATGCT	60.036	CCTGTTTGGTAGGGGCATGT	59.96	149
TRINITY_Contig13189	1	p3	(GCG)4	12	145	156	CAGATCTGGGAGGGAAGGGA	60.031	ATCAAGCTCATCGGTGCCAA	60.035	122
TRINITY_Contig13189	2	c	(CCTC)3cgtgcgtccacgacaacaaccac(GGAG)3	47	719	765	CAGATCTGGGAGGGAAGGGA	60.031	ATCAAGCTCATCGGTGCCAA	60.035	122
TRINITY_Contig13189	3	p3	(CGC)6	18	1554	1571	CAGATCTGGGAGGGAAGGGA	60.031	ATCAAGCTCATCGGTGCCAA	60.035	122
TRINITY_Contig13189	4	p5	(CTCGG)4	20	1993	2012	CAGATCTGGGAGGGAAGGGA	60.031	ATCAAGCTCATCGGTGCCAA	60.035	122
TRINITY_Contig13191	1	p5	(GGAGA)3	15	4525	4539	CTCCTCCGGTCGAATCGAAG	59.97	AGCAGCTCCAGTTCCCTTTC	59.963	108
TRINITY_Contig13192	1	p4	(CCAC)3	12	1871	1882	CTCCCCCTCTCTCCACAAGA	59.958	CTTCTTGCTCGTGGAGTCGT	60.039	137
TRINITY_Contig13193	1	p3	(TGA)4	12	1142	1153	GCTGAACCAAGCCACAACTG	59.969	ACCAATGTTGCTGCTTGCTG	59.967	268
TRINITY_Contig13196	1	p3	(GCG)4	12	1005	1016	GCGCGGCAAGATAAAGAAGG	59.972	GTTCCCACCACATCCTCGTT	59.964	225
TRINITY_Contig13196	2	c	(GGA)5gcggcggaggcagggaggggct(GCG)4	49	1549	1597	GCGCGGCAAGATAAAGAAGG	59.972	GTTCCCACCACATCCTCGTT	59.964	225
TRINITY_Contig13197	1	p5	(AAAAG)3	15	90	104	TCAAACTCGTGGCACTTGGA	59.821	GATGCCGTGGTTCCCATTTC	59.544	108
TRINITY_Contig13197	2	p3	(GCC)5	15	500	514	TCAAACTCGTGGCACTTGGA	59.821	GATGCCGTGGTTCCCATTTC	59.544	108
TRINITY_Contig13197	3	c*	(TCC)3(TC<C>T)(CT)5	23	885	907	TCAAACTCGTGGCACTTGGA	59.821	GATGCCGTGGTTCCCATTTC	59.544	108
TRINITY_Contig13198	1	p3	(CGG)4	12	664	675	GTTGACTCAGAAGCCCACGA	59.966	TCCTCGGTTCTTGAACTGGC	59.965	207
TRINITY_Contig13198	2	p3	(GCA)4	12	1235	1246	GTTGACTCAGAAGCCCACGA	59.966	TCCTCGGTTCTTGAACTGGC	59.965	207
TRINITY_Contig13203	1	p3	(CGC)4	12	1537	1548	GGCCTCCGGTAAAGTGGAAA	59.963	AGCTGCTTCCTCAACAGTCC	59.964	122
TRINITY_Contig13204	1	p3	(AGC)4	12	395	406	CACAGCAGTGCCCATAGGAA	60.035	CGCCACCTTCCTCTTTCTGT	59.965	244
TRINITY_Contig13208	1	p3	(GCC)4	12	935	946	CCACGGCTTTCCTGATCAGT	60.036	CCCTTTTGTGCTTGTGGACG	59.969	227
TRINITY_Contig13210	1	p3	(GCC)5	15	334	348	GAGCACTGATCAACCCACCA	59.963	GTCTCCAGCATCGGATCTGG	59.967	232
TRINITY_Contig13210	2	p3	(GCG)4	12	559	570	GAGCACTGATCAACCCACCA	59.963	GTCTCCAGCATCGGATCTGG	59.967	232
TRINITY_Contig13215	1	c	(GCC)5gtgggcgtctgagagggccgggctctggcagagcgcgagcg(CGA)4	68	1249	1316	CTCAACCGACCTGCTTCACT	59.966	ACAACGAGCTCTGCCGTAAA	59.967	230
TRINITY_Contig13216	1	p3	(CCA)5	15	79	93	CTCTTCCAGGCACAAGAGGG	60.036	GGAAACTCCTGGACCCTGTG	59.963	207
TRINITY_Contig13216	2	p3	(GGT)5	15	3726	3740	CTCTTCCAGGCACAAGAGGG	60.036	GGAAACTCCTGGACCCTGTG	59.963	207
TRINITY_Contig13217	1	p3	(GGC)4	12	401	412	CTTGCCCTAGAGCAGTACCG	59.897	TCGACTGCATATACCGTGGC	59.968	140
TRINITY_Contig13217	2	p5	(GAGGG)4	20	601	620	CTTGCCCTAGAGCAGTACCG	59.897	TCGACTGCATATACCGTGGC	59.968	140
TRINITY_Contig13218	1	p3	(CGG)9	27	1405	1431	CCGGTCACCAAACTTGCTTG	59.969	GAGTGCCTGAAGGAAGTCCC	60.036	179
TRINITY_Contig13219	1	p4	(GGTC)3	12	183	194	ATCCCTGGCTGAACGTGAAG	60.036	CAACGTCACCGAGGTCTTCA	59.968	188
TRINITY_Contig13219	2	p3	(CGG)4	12	1007	1018	ATCCCTGGCTGAACGTGAAG	60.036	CAACGTCACCGAGGTCTTCA	59.968	188
TRINITY_Contig13220	1	c	(TGG)5tgcccctcaggctcagcccagctcgtgctgcgctgctggaagctggctggctctcgaga(GT)6	86	1339	1424	TTGTTGCTGGAGAGGTCGAC	59.966	CTCATGTCGCTGTCCCTGTT	60.037	176
TRINITY_Contig13222	1	p6	(GGGGCC)3	18	162	179	GGACCATTTCCTCAAGCGGT	60.323	CAGTCCTTCCAGCAGACGAG	60.109	216
TRINITY_Contig13222	2	c	(TCCC)3ctcgtcct(CCCG)3	32	445	476	GGACCATTTCCTCAAGCGGT	60.323	CAGTCCTTCCAGCAGACGAG	60.109	216
TRINITY_Contig13223	1	p6	(CTCGGA)3	18	1150	1167	AACTGCTGATGCCATCCGAA	60.035	AAGAATAGCAGGCGCCACTT	60.035	133
TRINITY_Contig13224	1	p4	(CTTG)3	12	458	469	TGCCATGACCCGCAGTTATT	60.035	TGTTAGTGGGGGCGAGGATA	60.032	124
TRINITY_Contig13225	1	p3	(GCC)6	18	761	778	AACTTTTATGGCCGGAGCGA	60.036	GATCGCGCCTCATCAGGTAA	59.968	228
TRINITY_Contig13226	1	p3	(CGC)4	12	43	54	TTGAAAGACGACCACTCGGG	59.967	CGTGAACCTGGGCCCTTAAT	60.034	105
TRINITY_Contig13227	1	p5	(GCTCT)3	15	104	118	TACTCCTGATCAGCGACCCA	60.034	TTGCAACCTTCGGGCAAATG	59.967	107
TRINITY_Contig13230	1	p3	(CGC)4	12	3272	3283	TGGTGTTGGGGAAAAGCCTT	60.032	AAAAGCCTAGGATGTGGCCC	60.033	237
TRINITY_Contig13231	1	p3	(TCG)4	12	203	214	GGCTGAGATATCCCGGTTGG	59.965	CCTGTCCCCACGCATACATT	60.107	212
TRINITY_Contig13232	1	p4	(CCTT)3	12	247	258	ACATTGCTGCGAGGAAGACA	59.965	GAGTCGAACAGTGCAGTCCA	59.968	122
TRINITY_Contig13233	1	p3	(CGC)5	15	439	453	CCCGTCCTACATCGTCTTCG	59.971	CTGCCCCTTGAGCTTGATGA	60.035	103
TRINITY_Contig13234	1	p3	(GGC)4	12	353	364	AAGCACACACCACGGAAAGA	60.107	AGCTTGTGGATGCTGTACCC	60.035	162
TRINITY_Contig13234	2	p4	(GAGG)3	12	847	858	AAGCACACACCACGGAAAGA	60.107	AGCTTGTGGATGCTGTACCC	60.035	162
TRINITY_Contig13235	1	p3	(TCC)4	12	1061	1072	CGATGCCTACTGCTACCAGG	59.967	TACAAGATCGAGGCGATGCC	59.968	201
TRINITY_Contig13235	2	p4	(TGTA)3	12	2656	2667	CGATGCCTACTGCTACCAGG	59.967	TACAAGATCGAGGCGATGCC	59.968	201
TRINITY_Contig13236	1	p3	(CGC)4	12	150	161	CTGAATAAGCTGCGGCGTTC	59.973	AGACTACTCCATGGAGCCGT	60.033	136
TRINITY_Contig13236	2	p2	(TC)8	16	918	933	CTGAATAAGCTGCGGCGTTC	59.973	AGACTACTCCATGGAGCCGT	60.033	136
TRINITY_Contig13238	1	p3	(CCG)4	12	1696	1707	AGACTGTCGATCCCAGCTCT	60.034	TTGCAGCGTGTTACAGCAAC	59.971	205

TRINITY_Contig13239	1	c	(GGC)4ggtggcggcggcgcctgctacaactgcggccagaccggccacatggcgagggactgctcctct(GGC)6cggttcgga(GGC)4	114	1826	1939	GTATCCACTTCTTGGCCCCC	60.107	TGGGGAGGGACTATGAGGTG	60.031	183
TRINITY_Contig13242	1	p3	(CGC)4	12	837	848	GGGTTCGTGATCGAGGTGTT	60.038	CTAGACGGTCGTAGCGCATT	59.971	100
TRINITY_Contig13242	2	p3	(GTG)4	12	2044	2055	GGGTTCGTGATCGAGGTGTT	60.038	CTAGACGGTCGTAGCGCATT	59.971	100
TRINITY_Contig13242	3	p3	(GCT)4	12	2395	2406	GGGTTCGTGATCGAGGTGTT	60.038	CTAGACGGTCGTAGCGCATT	59.971	100
TRINITY_Contig13246	1	p4	(AACC)3	12	58	69	GAGATTGAACATCGCGGCAC	59.973	TCGTACTCCCATTCTCCGGT	60.034	179
TRINITY_Contig13246	2	p3	(CGG)4	12	771	782	GAGATTGAACATCGCGGCAC	59.973	TCGTACTCCCATTCTCCGGT	60.034	179
TRINITY_Contig13247	1	p6	(GAGGCG)3	18	1844	1861	CACGGAGATACTCGGGCTTC	59.969	GACTTCGAGAGTGTGGCCAA	59.966	127
TRINITY_Contig13249	1	p5	(AAATA)3	15	37	51	CCGTGTGCCTGTGAACAAAG	59.971	GGTACCCAAGTTTACGGGCA	59.963	224
TRINITY_Contig13249	2	c	(TG)6ttcttcttttgtattaacattaatgttaatacaaagaaga(AC)6	63	2488	2550	CCGTGTGCCTGTGAACAAAG	59.971	GGTACCCAAGTTTACGGGCA	59.963	224
TRINITY_Contig13251	1	c	(CGC)5cgtggtcaaggacggcaaccccaggaagaaggacgcgaacgctaaaaagcagccgccggcgaaggccggggccagggccaagtcgaaggccatac(GCGA)3cggaaacggaagagggcagcgtggacttcgaccggccgtccaagttcctggtggtcgcctcaacgccatccgcgacgcggtggcgcccgaggacggcg(GCA)4	233	347	579	AGGAAGGAGAGGCTTGGGAT	59.955	ACCGACAGAGCGAACAACAT	59.966	131
TRINITY_Contig13252	1	p3	(GTC)4	12	259	270	GGCGCTCCAACCTCTAGATC	59.967	GAGGTTGGACGTTGCTGGTA	59.966	100
TRINITY_Contig13252	2	p2	(TG)8	16	890	905	GGCGCTCCAACCTCTAGATC	59.967	GAGGTTGGACGTTGCTGGTA	59.966	100
TRINITY_Contig13253	1	p4	(GAGG)3	12	30	41	CCACGTACCTGTTCCTCACC	60.038	CTCTCGTCGGAACCAACCAA	59.967	259
TRINITY_Contig13254	1	c	(GGC)4gagagcgacgagcagggccgtcggccttgcgaacgtggccatggcagctgacgactgcagcta(CTAG)3	88	613	700	GAGAATGCCGATGAGGAGGG	59.965	CCCTCTCTGCTCCCTCTCTT	60.032	133
TRINITY_Contig13255	1	p3	(TCT)4	12	361	372	TACGTGCCCAGGTACAGGTA	59.961	TCCTCAACTTCCCGCTTTCC	59.964	214
TRINITY_Contig13255	2	p3	(CGG)4	12	522	533	TACGTGCCCAGGTACAGGTA	59.961	TCCTCAACTTCCCGCTTTCC	59.964	214
TRINITY_Contig13255	3	p3	(GCC)4	12	670	681	TACGTGCCCAGGTACAGGTA	59.961	TCCTCAACTTCCCGCTTTCC	59.964	214
TRINITY_Contig13255	4	p3	(CGC)4	12	1907	1918	TACGTGCCCAGGTACAGGTA	59.961	TCCTCAACTTCCCGCTTTCC	59.964	214
TRINITY_Contig13256	1	p3	(CGG)5	15	1028	1042	TAAAGCAAAAAGGCGGCACC	59.967	ACCCATCCTCCTCTTCCCTC	60.031	276
TRINITY_Contig13258	1	p3	(GCA)4	12	428	439	AACACCTCGTACCCGTCAAC	59.967	ATTCGCCCTCGATTCCATCC	59.965	112
TRINITY_Contig13258	2	p4	(AAGC)3	12	1414	1425	AACACCTCGTACCCGTCAAC	59.967	ATTCGCCCTCGATTCCATCC	59.965	112
TRINITY_Contig13259	1	p3	(CGG)6	18	1284	1301	GTGCTCCCACCAATCAGACA	59.963	GAGGCGATAAGGGAGAGTGC	59.967	151
TRINITY_Contig13261	1	p6	(CCGTGC)3	18	218	235	TGCCCATGCCTAGCAATGAA	60.033	GCTCCAAACGATGTCCCTGA	60.036	141
TRINITY_Contig13263	1	p3	(CGA)4	12	20	31	GCGGGTTGACGACAATATGC	59.972	ACAGCATGGTGGAGGACATG	60.035	213
TRINITY_Contig13263	2	p6	(GCTTCT)3	18	307	324	GCGGGTTGACGACAATATGC	59.972	ACAGCATGGTGGAGGACATG	60.035	213
TRINITY_Contig13263	3	c	(GCAC)3gcagcgaaacgaaacggaattcgagagaccagatccctcacccgtgaccggagca(GGAT)3(AGG)6	97	1694	1790	GCGGGTTGACGACAATATGC	59.972	ACAGCATGGTGGAGGACATG	60.035	213
TRINITY_Contig13264	1	p3	(CAG)4	12	168	179	AAGAGGCAGTCTTGGTGAGC	59.964	CGAGGCTTGCAATCGTGATG	59.973	126
TRINITY_Contig13264	2	p4	(CCAC)3	12	4279	4290	AAGAGGCAGTCTTGGTGAGC	59.964	CGAGGCTTGCAATCGTGATG	59.973	126
TRINITY_Contig13264	3	p2	(CG)6	12	4719	4730	AAGAGGCAGTCTTGGTGAGC	59.964	CGAGGCTTGCAATCGTGATG	59.973	126
TRINITY_Contig13265	1	p3	(GGC)5	15	1114	1128	CTACGAGCTGGCCTACTTCG	59.97	AAAGACGGAATGCTCGCTGA	60.037	259
TRINITY_Contig13266	1	p6	(GCCGGC)3	18	687	704	TTTCCTCCAGGCAGAGTTGG	59.598	GTGATCTTTGCAGACGCGAC	59.906	252
TRINITY_Contig13266	2	c	(GCTA)3gagctctgca(GATG)3	34	886	919	TTTCCTCCAGGCAGAGTTGG	59.598	GTGATCTTTGCAGACGCGAC	59.906	252
TRINITY_Contig13269	1	c	(TC)10ttgtttttctctcacttcatgcctttttttgct(CTCTCA)3	70	1	70	GAGCAAGTGGGTGGATCGAA	60.036	TGTTGTCTCGGTTGAGGACG	59.968	248
TRINITY_Contig13269	2	c	(GCC)8ggtgttggtgttctggtcggagacctcgccgttggccgacggcagcgcgctggagcacaccgacgagcacgacaggatgttgtt(GCC)5	123	209	331	GAGCAAGTGGGTGGATCGAA	60.036	TGTTGTCTCGGTTGAGGACG	59.968	248
TRINITY_Contig13269	3	p2	(TC)6	12	628	639	GAGCAAGTGGGTGGATCGAA	60.036	TGTTGTCTCGGTTGAGGACG	59.968	248
TRINITY_Contig13271	1	p3	(CTG)4	12	132	143	GGCCGGCAGATCTACTTCTC	59.967	CTCAGCGAACGAAATCTGCG	59.976	172
TRINITY_Contig13271	2	p3	(CGC)4	12	822	833	GGCCGGCAGATCTACTTCTC	59.967	CTCAGCGAACGAAATCTGCG	59.976	172
TRINITY_Contig13274	1	p3	(GCC)5	15	1660	1674	AGCTTATACAGGAGGGGCCA	60.03	GCAGGACATTGGTGGGATGA	60.034	230
TRINITY_Contig13274	2	p3	(GGA)4	12	1909	1920	AGCTTATACAGGAGGGGCCA	60.03	GCAGGACATTGGTGGGATGA	60.034	230
TRINITY_Contig13275	1	p3	(CTT)4	12	1	12	CCTCGACGACAAGATCCTCG	59.972	CGCAACAGCCGGTATTTAGC	59.972	102
TRINITY_Contig13275	2	p3	(CCG)4	12	1065	1076	CCTCGACGACAAGATCCTCG	59.972	CGCAACAGCCGGTATTTAGC	59.972	102
TRINITY_Contig13276	1	c	(CCGGCG)3ccggcccaggtggcggccgaagcgccgaggccgaggcacatctcgccctcgagcgcgctgcgggcggcgccgccggcgtcgatgggcgcga(CGC)4	118	260	377	GGACTCGCGGTTCTTGATCA	60.109	CGGTGGCTTCCGCAATAATG	59.971	232
TRINITY_Contig13276	2	p3	(GTC)4	12	643	654	GGACTCGCGGTTCTTGATCA	60.109	CGGTGGCTTCCGCAATAATG	59.971	232
TRINITY_Contig13277	1	c	(GATC)3taccggatcgcaacacgccag(CCAC)3	45	634	678	GACAAGAGCAGGTCAGCCTT	59.964	ATTTTGCTCGAATCGCGACG	59.975	148
TRINITY_Contig13277	2	c	(CGT)5cct(CGG)4	30	1368	1397	GACAAGAGCAGGTCAGCCTT	59.964	ATTTTGCTCGAATCGCGACG	59.975	148
TRINITY_Contig13279	1	p3	(TCC)4	12	816	827	GCATCTCCGCTTTTCCTCCT	60.107	CTCTAGGACGATGCAGAGCG	60.041	208
TRINITY_Contig13279	2	p3	(GGC)4	12	1079	1090	GCATCTCCGCTTTTCCTCCT	60.107	CTCTAGGACGATGCAGAGCG	60.041	208
TRINITY_Contig13282	1	p4	(AACC)3	12	125	136	GAAGAAAGGGAATGCGGTGC	59.829	GGAACACGCAGTCCAAAACC	59.97	169
[truncated: 6,881,789 more chars]
